# Mechanistic and genetic basis of single-strand templated repair at Cas12a-induced DNA breaks in *Chlamydomonas reinhardtii*

**DOI:** 10.1038/s41467-021-27004-1

**Published:** 2021-11-19

**Authors:** Aron Ferenczi, Yen Peng Chew, Erika Kroll, Charlotte von Koppenfels, Andrew Hudson, Attila Molnar

**Affiliations:** 1 Institute of Molecular Plant Sciences, University of Edinburgh, Edinburgh, EH9 3BF UK; 2 Present Address: Department of Biointeractions and Crop Protection, Rothamsted Research, Harpenden, AL5 2JQ UK

**Keywords:** Double-strand DNA breaks, Plant biotechnology, CRISPR-Cas9 genome editing

## Abstract

Single-stranded oligodeoxynucleotides (ssODNs) are widely used as DNA repair templates in CRISPR/Cas precision genome editing. However, the underlying mechanisms of single-strand templated DNA repair (SSTR) are inadequately understood, constraining rational improvements to precision editing. Here we study SSTR at CRISPR/Cas12a-induced DNA double-strand breaks (DSBs) in the eukaryotic model green microalga *Chlamydomonas reinhardtii*. We demonstrate that ssODNs physically incorporate into the genome during SSTR at Cas12a-induced DSBs. This process is genetically independent of the Rad51-dependent homologous recombination and Fanconi anemia pathways, is strongly antagonized by non-homologous end-joining, and is mediated almost entirely by the alternative end-joining enzyme polymerase θ. These findings suggest differences in SSTR between *C. reinhardtii* and animals. Our work illustrates the promising potentially of *C. reinhardtii* as a model organism for studying nuclear DNA repair.

## Abstract (web summary)

Single-stranded oligodeoxynucleotides are often used as templates for DNA repair during genome editing. Here the authors show that, unlike in animals, single-strand templated DNA repair in *Chlamydomonas reinhardtii* relies on the alternative end-joining enzyme polymerase θ.

## Introduction

Short single-stranded oligodeoxynucleotide (ssODN or ssDNA) repair templates used in conjunction with targeted nuclease-induced DNA breaks remain one of the most accessible, versatile, and efficient means of introducing directed gene edits in vivo (i.e., precision editing)^1–4^. Short ssODNs with homology arms on the order of tens of nucleotides enable precision editing where homologous recombination (HR) occurs at prohibitively low levels^3^. The process governing this form of editing is called single-strand templated repair (SSTR), is poorly understood, and has only been studied using Cas9 in human cells^5–10^ and yeast^11^. A lack of understanding of SSTR hampers rational improvements to precision editing and highlights an area of DNA repair metabolism requiring further investigation.

Two mechanistic forms of SSTR exist: single-strand DNA incorporation (ssDI) and (Rad51-independent) synthesis-dependent strand annealing (SDSA)^5–7,12^. During ssDI (also called ‘bridge model’^12^), ssODNs physically incorporate into the genome, while during SDSA, ssODNs serve as templates for genomic DNA synthesis without their physical genomic incorporation^5–8^. In human cells, both forms are present at DNA single-strand nicks depending on the orientation of the ssODN relative to the nick^5,6^, while SDSA prevails at double-strand breaks (DSBs)^6,7^.

Genetically, SSTR is independent of HR at Cas9-induced DSBs in both human cells^5,6,9,10^ and fungi^11^. Recent work in human cells has implicated the Fanconi anemia (FA) pathway in SSTR at Cas9-induced DSBs^10^, which is known for resolving DNA crosslinks through mobilizing repair mechanisms such as HR and translesion synthesis^13,14^. However, the downstream repair enzymes recruited by FA in SSTR are still unknown. In addition, our knowledge is limited about the molecular mechanisms that govern SSTR in other eukaryotes and using other nucleases, including whether the role of FA is conserved or specific to Cas9.

The eukaryotic green microalga *Chlamydomonas reinhardtii* has promising potential as a model organism for studying nuclear DNA repair. Induction of targeted DNA breaks—one of the cornerstones of studying DNA repair—has recently been made possible through a plethora of gene editing techniques^3,15–21^. The same techniques enable targeted gene knockouts with routine efficiency for reverse genetic studies. Its relatively small (~110 Mb) haploid genome further facilitates genetic analysis^22^. Since green algae are basally divergent within the plant kingdom (*Viridiplantae*), *C. reinhardtii* is uniquely positioned to provide insights into the conservation of DNA repair pathways. Yet relatively little is known about DNA repair genes in *C. reinhardtii*^23–26^.

Here we elucidate the DNA repair events underpinning SSTR at CRISPR/Cas12a-induced DSBs in *C. reinhardtii*. Using both previously used and novel techniques, we find that ssODNs physically incorporate into the genome during Cas12a-induced DSB repair via the ssDI form of SSTR. Genetic analysis reveals that this process is independent of HR and FA, is in strong competition with non-homologous end-joining (NHEJ), and is mediated entirely and specifically by the alt-EJ enzyme polymerase θ (encoded by *POLQ*). To enable our investigation, we functionally validate all major DSB repair pathways: HR, NHEJ and alt-EJ, as well as the DNA crosslink-resolving FA pathway, and verify the involvement of known canonical enzymes in each pathway. We observe that NHEJ mediates the majority of DSB repair even in the presence of ssODNs, which strongly suggests that *C. reinhardtii* is acutely dependent on NHEJ for maintaining genome stability.

## Results

### Experimental set-up: the fkb12 assay

**Fig. 1:**
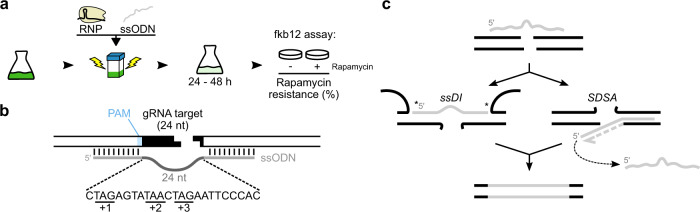
Schematic of experimental set-up and single-strand templated repair (SSTR) pathways. **a** Transfection involves culturing cells, electroporating (i.e., transfecting) with ssODNs and ribonucleoproteins (RNPs) consisting of Cas12a and guide RNA (gRNA), culture recovery, and in the case of the fkb12 assay plating onto media with and without rapamycin to calculate rapamycin resistance (i.e., editing efficiency). **b** Schematic of *FKB12* locus and ssODN design including homology arms (light grey) flanking a central non-homologous region (dark grey) containing stop codons in all three reading frames (underlined). PAM: protospacer-adjacent motif. **c** Illustration of two known forms of SSTR: single-strand DNA incorporation (ssDI) and synthesis-dependent strand annealing (SDSA). Asterisks (*) illustrate ligation or gap-filling DNA synthesis necessary during ssDI.

To survey SSTR, we employed our previously developed experimental system whereby DNA DSBs are induced in vivo by delivering CRISPR/Cas12a (formerly Cpf1) ribonucleoproteins (RNPs) targeted at the *FK506-binding protein 12* (*FKB12*) locus^3,27^. *FKB12* loss-of-function results in high tolerance (i.e., resistance) to rapamycin, which we quantified by plating known volumes of cells onto media with and without rapamycin (Fig. 1a, Supplementary Fig. 1). Gene editing efficiency was calculated as the percentage of rapamycin resistance colonies, which we refer to as ‘fkb12 assay’. Repeated plating of a mixture of wild type (wt) and *fkb12* mutant cells confirmed robustness of the fkb12 assay (Supplementary Fig. 1).

To use the fkb12 assay for surveying SSTR, ssODNs were co-transfected into cells alongside Cas12a RNPs. The ssODNs harboured a 24 nucleotide (nt) long non-homologous central region to replace the guide RNA (gRNA)-targeted genomic DNA sequence while also introducing stop codons in all 3 reading frames of *FKB12*, which was flanked by short (<75 nt) homologous sequences (referred to as homology arms, Fig. 1b). Although co-delivery of ssODNs and RNPs primarily induces SSTR editing events, allowing SSTR to be quantified by rapamycin resistance, it occasionally generates insertion-deletions (indels)^3^. We therefore tested rapamycin-resistant colonies for indels by colony PCR and subsequent Sanger sequencing, where the determination of population-level SSTR was needed.

### Terminally modified ssODNs suggest SSTR mechanism

**Fig. 2:**
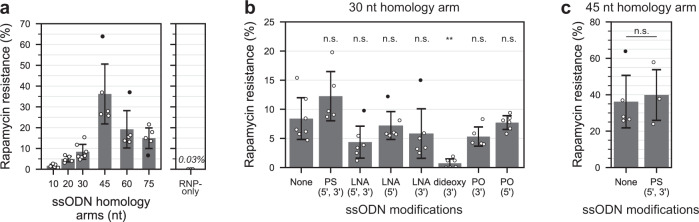
Gene editing using terminally modified ssODNs. Rapamycin resistance quantifies single-strand templated repair (SSTR). **a** Left panel: editing using varying nucleotide (nt) length ssODN homology arms (5≤n≤7, Supplementary Data 1). Right panel: editing without ssODNs (RNP-only, *n* = 3). **b** Editing using ssODNs with 30 nt homology arm that were either unmodified (‘None’, *n* = 7), bi-terminally modified with phosphothioate bonds (PS, *n* = 5, *p* = 0.211) or locked nucleic acid (LNA, *n* = 6, *p* = 0.140), or modified on one terminus with either LNA (5′, *n* = 6, *p* = 0.980; 3′, *n* = 6, *p* = 0.569), a dideoxy nucleotide (3′, *n* = 6, *p* = 5.86 × 10^−4^), or phosphate groups (3′, *n* = 5, *p* = 0.430; or 5′, *n* = 6, *p* = 0.999). All p values come from a post-hoc Dunnett’s test comparing each modified ssODN to the unmodified (‘None’) control (full Dunnett statistics in Supplementary Data 15). Analysis of variance (ANOVA) test (*F*(7,39) = 6.245) *p* = 6.09 × 10^−5^, Levene’s test *p* = 0.097. **c** Editing with 45 nt homology unmodified (*n* = 5, ‘None’) and bi-terminally PS-modified ssODNs (*n* = 3, one-sided Student’s *t* test *t*(6) = −0.301, *p* = 0.387, ${H}_{a}{:{{{{{\rm{PS}}}}}}} \; > \; {{{{{{\rm{None}}}}}}}$, Levene’s test *p* = 0.923). Black dots represent biological outliers which are included in our analyses (incl. mean averaging and statistical testing). Modifications and ssODNs are illustrated in Supplementary Fig. 2. Data in Supplementary Data 1. Bars are mean averages. Error bars are standard deviations. Repeats are biological (separately grown cultures). ***p < 0.001, n.s.: not significant, nt: nucleotide. ${H}_{a}$: alternative hypothesis.

We first established the optimal ssODN to survey SSTR by examining the effect ssODN homology arm lengths on DNA editing (Fig. 2a, left panel, fkb12 assay data in Supplementary Data 1). SSTR increased quasi-exponentially as the homology arms approached 45 nucleotides (nt). However, SSTR reduced with >45 nt homology arms, presumably due to stronger ssODN secondary structures. RNPs alone, without ssODNs, induced indels at 0.03% of all viable cells (Fig. 2a, right panel)^3^. We proceeded using ssODNs with 30 nt homology arms to enable detection of both increases and decreases in SSTR.

We sought to initially distinguish between two existing mechanistic models (or forms) of SSTR: single-strand DNA incorporation (ssDI) and synthesis-dependent strand annealing (SDSA, Fig. 1c)^5–7,12^. During ssDI, ssODNs are physically incorporated into the genome by annealing across the DSB presumably followed by ligation (also aptly called the ‘bridge model’^12^). During SDSA, ssODNs serve as templates for genomic DNA synthesis at the site of DSB without their physical genomic incorporation^5–8^.

First, we investigated terminal ssODN modifications shown to increase editing in human cells^2^. We tested phosphothioate (PS) linkages, which increase resistance to exonucleases^28^, or locked nucleic acids (LNAs), which in addition also stabilize nucleic acid duplexes^29^, and statistically compared them (and subsequent ssODN modifications) against the unmodified ssODN control (post-hoc Dunnett’s test, modifications illustrated in Supplementary Fig. 2). PS modifications provided a modest non-significant ~1.5-fold increase in editing (wt = 8.4%, PS = 12.3%, *p* = 0.211, Fig. 2b) and a lesser effect on ssODNs with 45 nt homology arms (wt = 36.2%, PS = 39.8%, one-sided Student’s *t* test *p *= 0.387, Fig. 2c). Unexpectedly, LNA modifications halved editing (wt = 8.4%, LNA = 4.4%, *p* = 0.140, Fig. 2b) and appeared to exert greater effect when located at the 3′ of the ssODN (wt = 8.4%; 5′LNA = 7.2%, *p* = 0.980; 3′LNA = 5.8%, *p* = 0.569; Fig. 2b). Mechanistically, terminal LNAs appear unlikely to interfere with the SDSA form of SSTR since genomic DNA synthesis (which is inherent to SDSA) could terminate prior to reaching the ssODN’s terminal LNA nucleotide. Instead, terminal LNAs are more likely to impair editing if SSTR proceeded via the ssDI form, possibly by interfering with ligation or by triggering a DNA damage response pathway such as mismatch repair since LNAs are a non-natural nucleotide. To better exploit ligation as a differentiator of ssDI and SDSA, we designed terminally modified ssODNs to inhibit its ligation.

A necessary step of ssDI, but not SDSA, is either ssODN ligation or the ssODN priming short-range gap-filling synthesis to achieve its direct incorporation into the genome (see asterisks in Fig. 1c). Both processes require a 3′ ssODN hydroxy group, so we designed two ssODNs lacking this feature to test inhibition of SSTR (i.e., ssDI). One carried a 3′ dideoxy nucleotide while another carried a 3′ phosphate (PO, modifications illustrated in Supplementary Fig. 2). Surprisingly, the 3′ dideoxy modification almost entirely abolished editing and SSTR, suggesting ssDI as being the major form of SSTR at DSBs (wt = 8.4%, dideoxy = 0.7%, *p* = 5.86 × 10^−4^, Fig. 2b). The 3′ PO modification showed a more modest decrease in editing (wt = 8.4%, 3′PO = 5.3%, *p* = 0.430, Fig. 2b), presumably due to cellular phosphatases removing the 3′ PO and allowing ligation to occur. We next envisioned SSTR could be enhanced by facilitating ligation of the ssODN through a 5′ PO modification, but this had no effect (wt = 8.4%, 5′PO = 7.7%, *p* = 0.999, Fig. 2b; modification illustrated in Supplementary Fig. 2). We concluded that 5′ ssODN phosphorylation is not a limiting step of ssDI. Taken together, terminally modified ssODNs provided indication of ssDI being the prevailing form of SSTR at Cas12a-induced DSBs in *C. reinhardtii*.

### Editing symmetry around DSBs distinguishes ssDI and SDSA

**Fig. 3:**
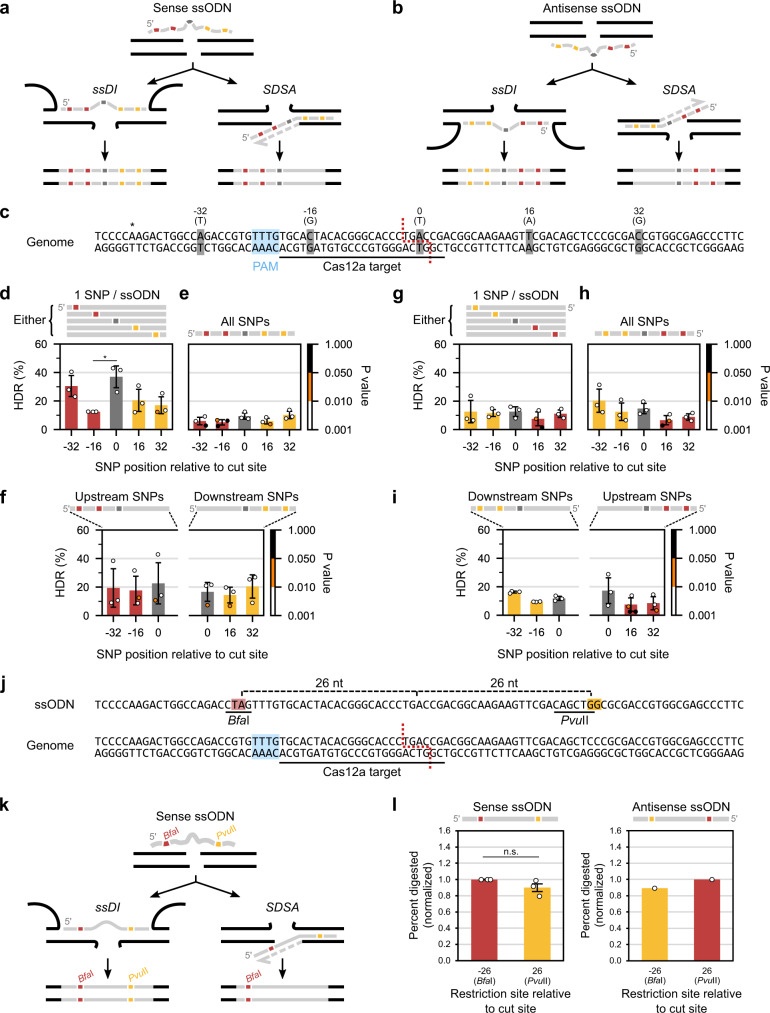
Symmetrical editing up- and downstream of the DNA double-stranded break (DSB). **a**, **b** Fate of SNPs carried on sense (**a**) and antisense (**b**) ssODNs through single-strand DNA incorporation (ssDI) and synthesis-dependent strand annealing (SDSA). **c** Illustration of SNPs introduced into *FKB12* using ssODNs (grey), annotated with the position (top) relative to the centre of the Cas12a-induced staggered DSB (red dotted line) and the SNP base being introduced (top, brackets). Asterisk (*) marks the base used for normalization during EditR. **d**–**i** Homology-directed repair (HDR, i.e., ssODN-mediated editing or SSTR) obtained using either five ssODNs carrying one SNP each (**d**, **g**, *n* = 3), one ssODN carrying all five SNPs (**e**, **h**, *n* = 3), or two ssODNs carrying either all up- or downstream SNPs (**f**, **i**, *n* = 3) using sense (**d**–**f**) or antisense (**g**–**i**) ssODNs. Colour-coded *p* values relate to the significance of SNP detection from the chromatogram background noise by EditR (i.e., SNPs above α = 0.05 are indistinguishable from background noise, *p* values inversely correlate with editing levels and sequencing quality). HDR values and SNP detection *p* values are in Supplementary Data 2 and 12, respectively. Of all analysis of variance (ANOVA) tests applied to each panel (**d**–**i**) only (**d**) was significant at (*F*(4,10) = 4.844), *p* = 0.020; post-hoc Tukey test reveals one significant comparison between SNPs −16 and 0, *p *= 0.023. Full ANOVA and post-hoc results in Supplementary Data 16 and 17, respectively. **j** Illustration of the restriction sites (*Bfa*I, *Pvu*II) carried on a single ssODN, with the distance shown (top) relative to the Cas12a-induced staggered DSB (red dotted line). **k** Fate of restriction sites through ssDI and SDSA; only sense ssODN illustrated. **l** Restriction digestion of DNA from cells transfected with sense (*n* = 3) or antisense (*n* = 1) ssODNs. Values normalized to the site on the ssODN 5′ (sense: *Bfa*I, antisense: *Pvu*II). Sense ssODN one-sided one-sample Student’s *t* test *t*(2) = −1.742, *p* = 0.112, ${H}_{0}{:}{\mu }_{{PvuII}}=1$, Shapiro–Wilk for *Pvu*II is *p* = 0.712. Gel images, band quantification and non-normalized digestion efficiencies in Supplementary Fig. 6. Bars are mean averages. Error bars are standard deviations. Repeats are biological (separately grown cultures). ${H}_{0}$: null hypothesis. PAM: protospacer-adjacent motif.

To further substantiate ssDI as being the prevailing form of SSTR at DSBs, we exploited another key difference between SDSA and ssDI: editing symmetry^6,7^. Since DNA synthesis is unidirectional, ssODNs carrying single nucleotide polymorphisms (SNPs) up- and downstream of the DSB produce different outcomes through SDSA and ssDI. Through SDSA, only SNPs in the 5′ ssODN homology arm generate genomic edits, while ssDI allows SNPs in both homology arms to generate genomic edits (Fig. 3a, b). To exploit this principle, we designed ssODNs containing SNPs at positions −32, −16, 0, 16 and 32 relative to the centre of the Cas12a-induced staggered DSB (Fig. 3c). Such distances sufficiently distinguished SSTR symmetry around the DSB in previous studies^6,7^. Three ways of introducing these SNPs were tested to gauge SSTR symmetry, either: (1) five ssODNs each carried one SNP, (2) two ssODNs each carried SNPs in either the up- or downstream homology arm, (3) a single ssODN carried all five SNPs; these were tested as both sense and antisense ssODNs (see line illustrations atop Fig. 3d–f for sense and Fig. 3g–i for antisense ssODNs). Analysis of variance (ANOVA) was applied to each of the six sets of ssODN experiments (Fig. 3d–i, six ANOVAs) to detect SNP asymmetry. All ssODNs were transfected separately into cells (not as mixtures). To detect SSTR (i.e., homology-directed repair, HDR, in the form of SNPs), transfected cell populations were recovered for 2 days, the corresponding *FKB12* locus was amplified by PCR and Sanger sequenced. SNPs were detected for significance above background levels in the sequencing chromatograms using EditR^30^. All EditR quality metrics were generally in line with recommended guidelines for robust SNP detection (Supplementary Figs. 3, 4)^30^. SNPs were then quantified using chromatogram peak heights, with normalization implemented using positive and negative controls to correct the local sequence-dependent variability in base peak heights (see “Methods”, all EditR data in Supplementary Data 2)^31^. No SNPs were detected in wt sequences (Supplementary Fig. 5, Supplementary Data 2). To see evidence for SDSA, we expected lower levels of editing from SNPs contained on the 3′ homology arm of the ssODNs (yellow SNPs in Fig. 3a, b). We found no significant or discernible asymmetry in the SNPs induced around the DSB, not when SNPs were contained on five separate ssODNs (Fig. 3d, g), nor when two ssODNs contained SNPs in either the up- or downstream homology arm, (Fig. 3f, i) nor when a single ssODN carried all five SNPs (Fig. 3e, h) in either ssODN orientation (sense ssODNs: Fig. 3d–f, antisense ssODNs: Fig. 3g–i). Slight variation between SNPs was observed when each SNP was introduced using separate sense ssODNs (post-hoc test of SNPs ‘−16’ and ‘0’ is *p* = 0.023, Fig. 3d), possibly through quality differences across the oligonucleotides, though it remained clear that SNPs to both sides of the DSB are symmetrically incorporated, consistent with ssDI and not SDSA.

We further tested editing symmetry by using ssODNs to introduce restriction sites around the DSB to ensure the results obtained using SNPs were not confounded by potential base-specific biases in Sanger sequencing. Two different restriction sites (*Bfa*I, *Pvu*II) were introduced via dinucleotide changes at positions −26 and +26 on an ssODN (Fig. 3j). This ssODN was tested in both sense and antisense orientations. After transfection, DNA was extracted and then digested with either enzyme. As before, SDSA would result in less editing encoded in the 3′ arm of the ssODN (yellow in Fig. 3k), so we normalized results to editing achieved by the 5′ arm of the ssODN. Sense ssODNs demonstrated no significant difference in the editing encoded by the ssODN 3′ arm (*Pvu*II, 0.90 normalized digestion efficiency) compared to the 5′ arm (*Bfa*I, 1.00 normalized digestion efficiency), though we did observe a ~10% reduction in the *Pvu*II digestion (one-sided one-sample Student’s *t* test *p* = 0.112, Fig. 3l left panel). SSTR using antisense ssODNs was barely detectable (Supplementary Fig. 6), presumably due to locus-specific ssODN strand bias^4^, but one quantifiable biological replicate provided the same ~10% reduction when the restriction site was encoded by the ssODN 3′ arm (*Bfa*I, 0.89 normalized digestion efficiency) as compared to the 5′ arm (*Pvu*II, 1.00 normalized digestion efficiency, Fig. 3l right panel). Identical restriction enzyme activities were observed in control digestion, thereby eliminating restriction enzyme activity as a possible confounding variable (Supplementary Fig. 6). Marginal SSTR asymmetry may suggest a small proportion of SSTR proceeding via SDSA, but we cannot rule out this being an assay artefact. Taken together, SNP and restriction site results demonstrate that both ssODN homology arms introduce gene edits with similar efficacy symmetrically around the DSB. This outcome is expected of ssDI, not SDSA, which corroborates ssDI as the prevailing form of SSTR at Cas12a-induced DSBs in *C. reinhardtii*.

### Biotin-labelling of the genome using ssODNs proves ssDI

**Fig. 4:**
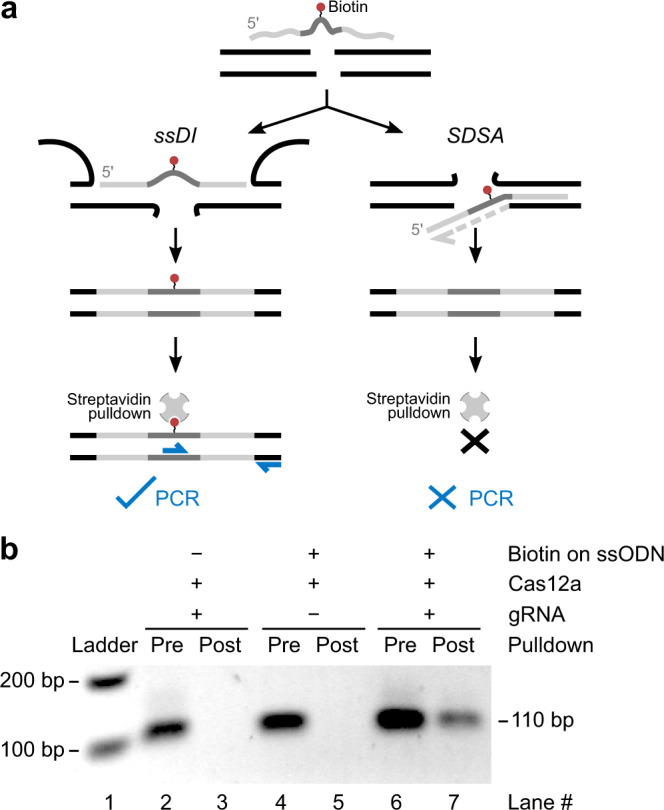
Biotinylated ssODNs tag the genome with biotin, enabling streptavidin-mediated pulldown of genomic DNA. **a** Schematic showing that biotin (red dot) carried on an ssODN only integrates into the genome via single-strand DNA incorporation (ssDI), and not via synthesis-dependent strand annealing (SDSA). The biotinylated genome can then be pulled down using streptavidin-coated beads and detected using PCR using the beads as templates after pulldown, with one primer specific to the ssODN-induced mutation and another specific to a region outside the ssODN sequence. **b** Biotin assay performed by extracting DNA from cells transfected with either biotinylated or non-biotinylated ssODNs, performing streptavidin-bead pulldown and PCR of the pre-pulldown DNA and the post-pulldown streptavidin beads. Raw gel images are in Supplementary Fig. 14. gRNA: guide RNA.

To test ssODN incorporation into the genome using direct, physical (non-quantitative) means, we transfected cells using biotinylated ssODNs (and RNPs) and tested whether we can subsequently pull down biotinylated genomic DNA with streptavidin-coated beads^7,32^ (Fig. 4a). After recovering transfected cells for 24 h, DNA was extracted and co-digested with restriction enzymes (to facilitate genomic DNA pulldown) and exonuclease I (to remove residual ssODN contamination). Genomic DNA was then subjected to streptavidin pulldown using magnetic beads; beads were then added directly to PCR. One PCR primer overlapped the ssODN-induced mutation and the other annealed outside the ssODN region to prevent amplification of residual ssODN (Fig. 4a).

SSTR using biotinylated ssODNs allowed successful pulldown of genomic DNA (Fig. 4b lanes 6, 7). In contrast, transfections using non-biotinylated ssODN (Fig. 4b lanes 2, 3) or with biotinylated ssODN but without functional RNPs (gRNA omitted, Fig. 4b lanes 4, 5) yielded negative results. Whilst this does not rule out SDSA, it provided unambiguous, direct physical evidence of ssDI.

### Genetic analysis of DNA repair pathways involved in SSTR

We next examined the involvement of DNA repair pathways in SSTR at Cas12a-induce DSBs to address an incomplete understanding of its enzymatic basis and to investigate whether ssDI might have different genetic requirements compared to DSB repair via SDSA in human cells^6,7,10^. We examined all known major DSB repair pathways; homologous recombination (HR), non-homologous end-joining (NHEJ), alternative end-joining (alt-EJ), and the DNA crosslink resolving Fanconi anemia (FA) pathway. To our knowledge, canonical enzymes in HR and FA have not yet been confirmed functionally in *C. reinhardtii*. Encouragingly, analysis of published RNA-seq data^33^ confirmed expression of homologues of the key enzymes from each pathway, though only around half of all known core FA enzymes were found, as reported previously for the model flowering plant *Arabidopsis thaliana*^34^ (Supplementary Fig. 7). To enable robust conclusions to be drawn about the involvement DNA repair pathways, we knocked out two separate canonical enzymes from each of the pathways except alt-EJ (where we knocked out only one) and confirmed their DNA repair-deficient phenotypes before examining SSTR. Each mutant genotype was statistically compared to wt (post-hoc Games–Howell test, Tukey adjusted *p* value).

Mutations were generated by antibiotic resistance marker-mediated gene disruption. We used *aphVIII*, encoding paromomycin resistance, which we co-transfected into cells alongside Cas12a RNPs targeting the DNA repair genes of interest. Confounding effects of off-target *aphVIII* integrations were mitigated by generating three independent mutants per target, except for *FANCM* where we only generated one mutant line due to the low rate of mutant recovery (for gene targeting efficiencies, see Supplementary Data 3). Mutants were identified by colony PCR of paromomycin-resistant cells and sequenced across both *aphVIII*-genome junctions to ensure they were independent insertion events (Supplementary Figs. 8–11). We derived population-level precise (i.e., scarless) SSTR using the fkb12 assay followed by PCR and sequencing of the corresponding *FKB12* locus among rapamycin-resistant colonies (see Methods, colony PCR data in Supplementary Data 4, population-level SSTR data in Supplementary Data 5); this was used for statistical testing for differences in SSTR.

**Fig. 5:**
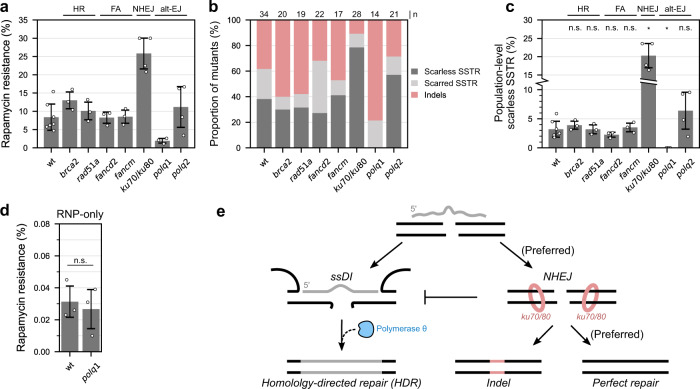
DNA repair pathways mutants tested for single-strand templated repair (SSTR). **a** Fkb12 assay results (3≤n≤7, see Supplementary Data 1) obtained using mutants in the indicated DNA repair pathways (top). Within genotypes, each replicate represents an independently created mutant line, except for *fancm* where 3 separately grown cultures of the same *fancm* line served as biological replicates. Data in Supplementary Data 1. **b** Colony PCR of rapamycin-resistant cells with samples sizes (*n*) indicated (top). Scarred SSTR was defined as any editing event that included at least part of the central non-homologous ssODN sequence, but that was not a perfect homology-directed event and contained unintended SNPs, insertions, deletions, and local sequence duplications. Data in Supplementary Data 4. **c** Population-level scarless SSTR obtained by multiplying rapamycin resistance (**a**) with scarless SSTR detected by colony PCR (**b**). Analysis of variance (ANOVA) test (*F*(7,22) = 32.487, *p* = 3.54 × 10^−10^), Levene’s test *p* = 3.98 × 10^−6^, post-hoc Games–Howell test used to compare each genotype to wt (Tukey corrected *p* values). Significant comparisons: wt-*ku70/80* *p* = 0.013, wt-*polq1* *p* = 0.013 (full Games–Howell statistics in Supplementary Data 18). Data in Supplementary Data 5. **d** RNP-only editing in wt (*n* = 7) and *polq1* (*n* = 3, one-sided Student’s *t* test *t*(4) = −0.422, *p* = 0.347, ${H}_{a}{:polq}1 \; < \; {{{{{{\rm{None}}}}}}}$, Levene’s test *p* = 670); this same wt data are also in Fig. 2a, right panel. Repeats are biological (separately grown cultures). Data in Supplementary Data 1. **e** Model of DSB repair in *C. reinhardtii*, including SSTR, based on this work. More detailed models presented in Supplementary Fig. 12. Bars are mean averages. Error bars are standard deviations. *p < 0.05, n.s.: not significant, ${H}_{a}$: alternative hypothesis, *n*: sample size.

HR is the best characterized homology-dependent DNA repair pathway and SSTR has been shown to be independent of HR in fungi and humans^5,6,9–11^. To test whether this was also the case in *C. reinhardtii*, we generated HR mutants by knocking out the genes encoding RAD51 and its metazoan co-factor, BRCA2. These mediate the indispensable steps of DNA homology search and strand exchange^35^. Previous studies using dsDNA repair templates containing long (1–2 kb) homology arms have shown HR to be extremely infrequent in *C. reinhardtii*^36–40^. We therefore decided to assess the DNA repair deficiency of our mutants indirectly by plating cells on both zeocin^25,41^, which induces both single- and double-stranded DNA breaks^42^, and the DNA crosslinking agent mitomycin C (MMC)^43^. Both DSBs and DNA crosslinks are conventionally subject to repair by HR^13,14^. HR mutants proved more sensitive compared to wt (i.e., hypersensitive) for both DNA-damaging agents (Supplementary Fig. 8), demonstrating that this pathway operates in *C. reinhardtii* and requires both genes. We then tested SSTR in *brca2* and *rad51* mutants. Neither mutation led to any differences in the levels of rapamycin resistance, nor indel frequencies, nor any significant differences in population-level SSTR levels (wt = 3.2%; *brca2* = 3.9%, *p* = 0.973; *rad51* = 3.2%, *p* = 1.000; Fig. 5a–c). This mirrors findings in human cells^5,6,9,10^ and yeast^11^ and suggests the independence of SSTR from HR is broadly conserved across eukaryotes.

A recent genetic screen has suggested that FA is involved in SSTR at Cas9-induced DSBs in human cells (along with several other factors)^10^. FA resolves DNA interstrand crosslinks that are recognised by a multimeric ‘core complex’, which signals via the central FANCD2-I complex to downstream repair factors such as HR and translesion synthesis to resolve the damage^13,14^. To test for FA involvement in SSTR at Cas12a-induced DSBs in *C. reinhardtii*, we generated FA-deficient cells by knocking out *FANCD2* and *FANCM*, which are members of the FANCD2-I complex and core complex, respectively. Both mutations reduced SSTR by 85–95% in human cells^10^. We initially assessed the FA-deficient phenotypes of *fancd2* and *fancm* cells by plating cells on media containing MMC. All mutant lines proved hypersensitive to MMC (Supplementary Fig. 9)^43^. One *fancd2* line (#3), which was strikingly more hypersensitive to MMC than the others, appeared to carry a large (≥10 kb) deletion at the 5′ *aphVIII* junction (Supplementary Fig. 9), suggesting that a larger genomic deletion may exacerbate the MMC-hypersensitive phenotype in this line. MMC hypersensitivity thus demonstrated functionality of the FA pathway in *C. reinhardtii*. However, no significant effects were observed in rapamycin resistance or population-level SSTR (wt = 3.2%; *fancd2* = 2.3%, *p* = 0.784; *fancm* = 3.5%, *p* = 1.000; Fig. 5a–c). Although we detected a slight reduction in population-level scarless SSTR in *fancd2*, we could not rule the possibility that this was due to sampling bias in colony PCR (Fig. 5b). Intriguingly, the lack of clear involvement of the FA pathway in SSTR at Cas12a-induced DSBs in *C. reinhardtii* is in stark contrasts with its evident role at Cas9-induced DSBs in human cells^10^.

We next investigated the end-joining pathway NHEJ, which mechanistically competes with SSTR for DSB repair^10^. To gauge the magnitude of this competition, we generated NHEJ mutants by knocking out the genes encoding KU70 and KU80, components of the conserved KU70/80 complex which bind terminal DNA ends to mediate their re-joining^44^. All *ku70* and *ku80* lines displayed hypersensitivity to zeocin, confirming their requirement for NHEJ in *C. reinhardtii*^25^ (Supplementary Fig. 10). Population-level scarless SSTR was significantly 6-fold higher in *ku70/80* lines (wt = 3.2%, *ku70/80* = 20.3%, *p* = 0.013, Fig. 5c) with near-complete abrogation of indels among rapamycin-resistant cells (Fig. 5b). This demonstrates marked competition between SSTR and NHEJ for DSB repair in *C. reinhardtii*.

Finally, we investigated alt-EJ, a relatively uncharacterized pathway^45^. An emerging understanding of alt-EJ implicates the involvement of polymerase θ (encoded by *POLQ*) and the presence of microhomologies at the sites of DSB repair^45–47^, which are presumably interlinked observations since polymerase θ extends DNA primed by microhomologies as short as 2 bp in vitro^46,47^. We hypothesized that the annealing of the ssODN to the DSB during ssDI may present microhomologies that serve as substrates for extension by polymerase θ, which thus may be involved in SSTR. We generated *polq1* knockouts, which demonstrated marked hypersensitivity to zeocin, more so than NHEJ-deficient *ku70/80* lines (Supplementary Fig. 11)^26^. We hypothesize this reflects a role of polymerase θ in both single- and double-strand break repair^48,49^, which are both induced by zeocin^42^. To our surprise, *polq1* lines completely abolished population-level SSTR (wt = 3.2%, *polq1* = 0.0%, *p* = 0.013, Fig. 5c); rapamycin-resistant mutations in *polq1* were almost exclusively indels (Fig. 5b).

A paralogue of *POLQ1*, annotated as *POLQ2*, lacks a polymerase domain but has a helicase domain and is most likely an orthologue of a human POLQ-like helicase HELQ (Supplementary Data 6). We generated *polq2* lines, which showed very subtle hypersensitivity to zeocin (Supplementary Fig. 11). However, *polq2* lines appeared to display elevated SSTR (wt = 3.2%, *polq2* = 6.4%, *p* = 0.723, Fig. 5c), suggesting that POLQ2 may potentially antagonize SSTR, possibly through its role in DSB repair.

To investigate whether reduced SSTR was due to specific involvement of polymerase θ, and not due to general decreased viability upon DSB-induction, we performed RNP-only fkb12 assays. Indels occurred with similar frequencies among wt and *polq1* mutants, indicating that scarless SSTR abrogation in *polq1* lines was due to specific involvement of polymerase θ in SSTR (wt = 0.031%, *polq1* = 0.027%, one-sided Student’s *t* test *p* = 0.347, Fig. 5d). Taken together, our results suggest polymerase θ specifically mediates almost all SSTR at Cas12a-induced DSBs in *C. reinhardtii*.

## Discussion

Here, we take two approaches to elucidate the DNA repair events underpinning SSTR at Cas12a-induced DSBs in *C. reinhardtii*. First, we demonstrate that ssODNs physically incorporate into the genome during DSB repair via ssDI (or ‘bridge model’^12^). This is illustrated by ligation-inhibiting dideoxy-modified ssODNs almost entirely abrogating SSTR (Fig. 2b), an ability to introduce SNPs and restriction sites symmetrically around the DSB (Fig. 3) and an ability to pull down genomic DNA once it has been tagged using a biotinylated ssODN (Fig. 4). Second, genetic analysis reveals that SSTR is independent of HR and FA, is in strong competition with NHEJ, and is mediated almost exclusively and specifically by the alt-EJ enzyme polymerase θ (POLQ, encoded by *POLQ1* in *C. reinhardtii*, Fig. 5c–e). The latter observation was also very recently made using Cas9 in *C. reinhardtii*^26^.

Polymerase θ may play a number of different roles in ssDI (Supplementary Fig. 12). Upon ssODNs annealing, polymerase θ may repair the second genomic DNA strand and heteroduplex DNA may then be resolved by mismatch repair (MMR) or mitotic segregation; MMR appears more likely since segregation would cause central SNPs to lead to higher SSTR levels (Fig. 3). Alternatively, polymerase θ may convert ssODNs into dsDNA^50^, leveraging its minimal priming requirements (≥2 bp^46,47^), and dsDNA may then mediate repair. However, this is unlikely, since in this case dsDNA should be better at editing than ssODNs, but we find the opposite (ssODN=36.2%, dsDNA =17.0%, one-sided Student’s *t* test *p* = 0.048, Supplementary Fig. 12). It would also be unclear what the downstream repair pathway would be, if not HR (Fig. 5). Finally, polymerase θ may perform short-range gap-filling synthesis on either side of the ssODN to secure it into the genome (replacing or preceding ligation), with the second genomic strand presumably ligated (or also repaired by polymerase θ). A combination of these scenarios may also exist with further work needed to address these possibilities.

Our findings strikingly contrast SSTR at Cas9-induced DSBs in human cells in several respects. Mechanistically, SDSA is the prevailing form of SSTR at Cas9-induced DSBs in human cells^6–8^, whereas we find ssDI to be the main form of SSTR at Cas12a-induced DSBs in *C. reinhardtii* with little to no evidence for SDSA. Furthermore, a genetic screen found several components of FA being involved in SSTR at Cas9-induced DSBs in human cells^10^, yet we find no such clear FA involvement at Cas12a-induced DSBs in *C. reinhardtii*. Our supplementary analysis of this screen reveals no detectable involvement of KU70/80 or polymerase θ in SSTR at Cas9-induced DSBs in human cells (Supplementary Fig. 13), which contrasts our finding that NHEJ strongly antagonizes SSTR while polymerase θ specifically mediates it at Cas12a-induced and Cas9-induced^26^ DSBs in *C. reinhardtii* (Fig. 5c–e).

Taking the combination of differences found with respect to human cells, it is possible that (in human cells) FA is involved in the SDSA form of SSTR, while (in *C. reinhardtii*) polymerase θ mediates the ssDI form. This would mean that ssDI and SDSA have different genetic requirements and may vary in prevalence across organisms. Cas9 nickases would be ideal to test this hypothesis in human cells where nicks can separately evoke ssDI and SDSA depending on the orientation of the ssODN relative to the nick^5–7^. However, it is also possible that our use of Cas12a contributes to the differences we observe compared to human cells, where (to our knowledge) all studies have been performed using Cas9, since these nucleases have different dissociation kinetics from cleaved DNA^51–53^. Such kinetic differences may affect access to DNA by repair enzymes to varying degrees^54^, however the POLQ-dependence of SSTR at Cas9-induced DSBs in *C. reinhardtii*^26^ suggest these findings to be nuclease-agnostic. Furthermore, polymerase θ also has broader relevance in ssDNA-mediated events in land plants as suggested by its recently discovered and substantial role in *Agrobacterium tumefaciens*-mediated single-stranded transfer-DNA (T-DNA) transfer and integration in *Arabidopsis thaliana*^55,56^. Further work is needed, in both plants and animals, to address whether differences across gene editing enzymes can bias SSTR (or any DNA repair) mechanisms or pathways.

Our findings raise further questions regarding SSTR in *C. reinhardtii*, such as whether SSTR is genetically identical to polymerase θ-mediated end-joining (TMEJ)^11^. It is also important to understand whether SSTR produces homo- or heteroduplex DNA edits (see models in Supplementary Fig. 12), since mitotic segregation of heteroduplex edits would limit ssODN-mediated precision editing to 50% in *C. reinhardtii* (assuming random segregation). These questions can now be addressed using the experimental system we present.

The six-fold increase in SSTR in NHEJ-deficient *C. reinhardtii ku70/80* lines demonstrates strong competition between SSTR and NHEJ for DSB repair (Fig. 5c). This indicates that DSBs are primarily repaired by NHEJ even when SSTR is an available repair option. We propose that such prevalence of NHEJ indicates an acute dependence on NHEJ for genome stability maintenance in *C. reinhardtii*. This mechanism would reconcile with limited opportunities for homology-dependent repair throughout its cell cycle owing to its haploid genome and the majority of its multiple fission cycle spent in G1 phase^57^. It would further reconcile with observations that plasmids harbouring long homology arms integrate largely at random into the genome and at low frequency^36–40,58,59^, despite the fact that recombination between co-transformed plasmids occurs readily^60,61^. We also note that while editing as high as 30% of all cells is possible using ssODNs (Fig. 2a)—implying ≥30% of cells experience DSBs—RNPs alone induce indels at only 0.03% (Fig. 5c). This confirms NHEJ as being a faithful repair mechanism but implies bleak prospects for Cas12a RNP-only editing in *C. reinhardtii*.

Our newfound mechanistic knowledge of SSTR suggests that certain strategies to increase precision editing, such as covalently attaching the ssODN to Cas9^62–65^ or fusion of the gRNA and ssODN into a single molecule^66^, would be futile attempts in *C. reinhardtii* due to the direct incorporation of ssODNs into the genome. Instead, we see promise in using nickases in this organism. The role of polymerase θ at single- as well as double-strand breaks^48,49^, suggested by heightened zeocin hypersensitivity compared to NHEJ-deficient *ku70/80* lines (Supplementary Figs. 10, 11), could be exploited by using nickases to preferentially engage polymerase θ and thereby predispose repair to SSTR^5,6,9^. Nickases would also bypass the SSTR-suppressing role of NHEJ (Fig. 5c) which only mediates DSB repair, and consequently may reduce unwanted NHEJ-mediated indels. In addition, timed ssODN delivery to exploit circadian *POLQ1* regulation may be another strategy to improve SSTR (Supplementary Fig. 7).

Finally, this work highlights the untapped potential of *C. reinhardtii* as a model organism for studying DNA repair. *C. reinhardtii* is a haploid, fast-growing, tractable organism with a well-annotated, relatively small, haploid genome^22,67^. Our work demonstrates the state of readiness of genetic manipulation in this organism to enable routine reverse genetics^3,15–21^. Notably, antibiotic resistance transgene-mediated gene disruption (see also^15,18–21^) seems to offer more consistent target gene knock-out capabilities than transgene-free ssODN-mediated editing^3^. The fkb12 assay is a readily accessible technique for isolating and profiling DNA repair events at an endogenous nuclear locus. Finally, we demonstrate the existence of all major DSB repair pathways (HR, NHEJ, alt-EJ) and their canonical enzymes (BRCA2, RAD51; KU70, KU80; POLQ1, POLQ2), as well as a functional FA pathway through analysis of FANCD2 and FANCM and establish simple assays to confirm their presence, which opens avenues to investigate these further. Expanding the set of model organisms used to study DNA repair across the tree of life provides unique evolutionary insights into the conservation of DNA repair pathways.

## Methods

### *C. reinhardtii* strain and growth conditions

*Chlamydomonas reinha*rdtii strain cc-1883 (*cw15*) was provided by Sinead Collins (University of Edinburgh, UK). Cells were cultured using Tris-acetate-phosphate (TAP) media^68^, supplemented with 1.5% agar for solid media. Unless stated otherwise, cells were maintained under constant, cool, fluorescent, white light (50*–*100 μmol m^−2^ s^−1^) at 28 °C. Liquid cultures were shaken at 110 rpm (Stuart SSL1 Orbital Shaker, SciTech LabApp SLA-OS-200).

### Purification of Cas12a

An expression plasmid for *E. coli* codon-optimized *Lachnospiraceae bacterium ND2006* Cas12a (formerly Cpf1) bearing an N-terminal MBP-6xHis-NLS tag (190 kDa) was a gift from Prof Jin-Soo Kim (Seoul National University, Republic of Korea, Addgene plasmid 79008). This was transformed into Rosetta 2(DE3)pLysS cells (Novagen, Merck) and selected on 50 μg/mL chloramphenicol and 50 μg/mL carbenicillin. To express Cas12a, overnight starter cultures (20 mL) grown in LB medium with antibiotics were used to inoculate 2 L expression cultures (at 1% v/v), then incubated by shaking at 110 rpm, 37 °C (New Brunswick Innova 44R). Upon reaching OD600 0.6*–*0.9, cultures were cooled in an ice-water bath for 5 min, supplemented with isopropyl-β-D-thiogalactoside (IPTG, 0.5 mM) and incubated overnight (16*–*20 h) at 16 °C, shaken at 110 rpm. Cells were harvested (6000 × *g*, 15 min, 4 °C), flash-frozen in liquid nitrogen and stored at *−*80 °C until purification. To following steps were all performed at 4 °C. To begin purification, the thawed cell pellet (3–5 g) was resuspended in 40 mL extraction buffer (50 mM HEPES [pH 7.5], 1 M NaCl, 5 mM MgCl_2_, 10 mM imidazole, 10% glycerol, 250 mM γ-aminobutyric acid [GABA]) supplemented with EDTA-free Halt protease inhibitor (1×, Thermo Fisher Scientific) and 1 mg/mL lysozyme, and incubated on ice for 30 min for lysis. The cell lysate was sonicated on ice to shear DNA and reduce viscosity (5 cycles of: 10 s on, 30 s off, amplitude 3.0, Soniprep 150), clarified by centrifugation (25,000 × *g*, 15 min, 4 °C) and passed through syringe filters (0.22 μm). Affinity purification was performed using cobalt resin (5 mL, HisPur, Thermo Fisher Scientific) as per the manufacturer’s batch protocol using extraction buffer for resin equilibration and washing. The elution buffer was the same as the extraction buffer except with 250 mM imidazole. Four elutions (5;mL each) were pooled (20 mL), concentrated (Vivaspin 20, 50k MWCO, GE Healthcare) and buffer exchanged (Zeba 40k MWCO spin desalting columns, Thermo Fisher Scientific) into storage buffer (20 mM HEPES [pH 7.5], 500 mM NaCl, 5 mM MgCl2, 1% glycerol, 1 mM DTT, 150 mM GABA) as per manufacturers’ instructions. Protein concentration was measured by Bradford (Sigma). Final protein concentrations were 18–20 mg/mL. Yields were 13–18 mg per L of culture. Single-use aliquots were snap-frozen in liquid nitrogen and stored at −80 °C.

### guide RNAs and ssODNs

Guide RNAs (gRNAs) were purchased from Sigma and Synthego and were resuspended to 200*–*700 µM (Supplementary Data 7). Single-stranded oligodeoxynucleotides (ssODNs) were purchased from Sigma, IDT and QIAGEN as dried oligonucleotides and were resuspended to 1 mM (Supplementary Data 8).

### *C. reinhardtii* transfection (with ssODNs, dsDNA or RNP-only)

*C. reinhardtii* cells were transfected as previously described^27^. All transfections were biological replicates, defined as transfections performed either on separately grown liquid cultures of the same line (wt, *fancm*) or of separate lines (all other DNA repair mutants); most transfections were performed on different dates. Cultures were grown to ~1*–*3 × 10^6^ cells/mL and quantified by staining with 10% Lugol’s iodine (5% w/w iodine, 10% potassium iodide) and counted using a haemocytometer. Dense cultures were diluted with TAP while dilute cultures were concentrated by centrifugation (1000*–*3000 *g*, 10*–*30 min) followed by partial supernatant removal to 2 × 10^6^ cells/mL. To form ribonucleoproteins (RNPs), Cas12a (0.263 nmol, 50 µg) was incubated with gRNA (0.789 nmol) at 37 °C for 10*–*15 min. Cells (125 µL, 2.5 × 10^5^ cells) were then mixed with RNPs, ssODN (2.63 nmol), supplemented with sucrose (40 mM, 2 M stock solution) in electroporation cuvettes (4; mm) and electroporated (600 V, 50 μF, 200 Ω, 4 mm; Gene Pulser Xcell with CE and PC modules, Bio-Rad). For RNP-only transfections, ssODNs were omitted. For dsDNA transfections, instead of ssODNs, dsDNA was added (2.63 nmol, 5.26 µL), which was made by mixing two complementary ssODNs (2.63 nmol, 2.63 µL, 1 mM, oligos ‘ssODN_45’ and ‘ssODN_45_revcomp’ in Supplementary Data 8) and denaturing then annealing them using, 95 °C, 5 min > −0.3 °C/s to 20 °C (Supplementary Fig. 12b, left panel). Next, the cells were transferred to 5 mL liquid TAP pre-supplemented with sucrose (40 mM) and recovered for 24*–*48 h on a shaker. After recovery, for experiments where SNP and restriction site were introduced using ssODNs (Fig. 3), 1 mL recovered cultures were harvested by centrifugation (17,000 × *g*, 1 min), supernatant was removed and pellets were stored at *−*20 °C until analysis (for further analysis, see ‘EditR analysis of SNP experiments’). For fkb12 assay experiments, cells were plated using freshly prepared starch solution (30%). To prepare this, starch was sterilized by washing a pre-weighed amount twice in absolute ethanol, thrice in sterile, distilled water, once in TAP and finally resuspending to 30% in TAP, all under sterile conditions. Washes involved vortexing to resuspend the starch, centrifugation (3000 × *g*, 10 s) and decanting. To plate cells transfected with RNPs and ssODNs, 30*–*250 µL recovered cells was added to 1000 µL starch solution, mixed well by pipetting and spread equally (usually as 2 × 450 µL) onto one TAP plate and one TAP plate supplemented with rapamycin (10 μM). To plate cells transfected using RNPs only, 50 µL recovered cells were added to 500 µL starch solution, mixed by pipetting then spread onto a TAP, and separately 4 mL of the same culture was pelleted by centrifugation (3000 × *g*, 30 min), supernatant was removed by leaving behind approximately 200 µL (to mitigate the loss of any of the pelleted cells), 300 µL starch solution was added, the pellet was resuspended in the total volume of approximately 500 µL and then spread onto a TAP plate supplemented with rapamycin (10 μM). Rapamycin plates were always freshly prepared by adding rapamycin (10 mM stock) into autoclaved flasks and adding molten TAP in increments with swirling in between; this prevented rapamycin precipitation. Plated cells were grown under low light (5*–*10 μmol m^−2^; s^−1^) to limit rapamycin photodegradation. After 7 days, plates were imaged (Canon PowerShot G16) and quantified using OpenCFU (v 3.9.0) by drawing a 3-point circle mask around the area covered by starch to exclude the plate label and using threshold settings: regular, 2, and radius settings: 5 min, auto-max^69^.

### Measuring SSTR in DNA repair mutants

SSTR was surveyed using the fkb12 assay followed by colony PCR of *FKB12* among rapamycin-resistant colonies (Supplementary Data 4). We derived population-level precise (i.e., scarless) SSTR by multiplying rapamycin resistance (%) by the proportion of scarless SSTR events (also scarred SSTR events and indels) identified by colony PCR (Supplementary Data 5). Scarred SSTR was defined as any editing event that included at least part of the central non-homologous ssODN sequence, but that was not a perfect homology-directed event and contained unintended SNPs, insertions, deletions, and local sequence duplications. Population-level scarless SSTR was used for statistical testing of population-level SSTR. A post-hoc Games–Howell test (Tukey corrected *p* values), was used to compare each mutant genotype to the wt since this test is compatible with the highly significant inequality of variances (Levene’s test, *p* = 3.98 × 10^−6^).

### *C. reinhardtii* transfection using aphVIII (paro^R^)

DNA repair mutants in line CC-1883 were generated by antibiotic-mediated gene disruption using *aphVIII*, which encodes paromomycin resistance (paro^R^), using guide RNAs targeted at DNA repair genes (Supplementary Data 7). The *aphVIII* gene, driven by the *HSP70A-RBCS2* promoter and terminated by the *RBCS2* terminator, was amplified from plasmid pSI103-1 (PCR conditions in Supplementary Data 9). Conditions for generating various mutants varied slightly in terms of whether the cultures were grown under synchronized conditions for transfection (14l:10d) and whether target locus-specific homology arms (50 bp) were added to *aphVIII* by PCR (for details, see Supplementary Data 3). Transfection was performed identically to ssODN-mediated editing, but with the following changes: cultures were grown under synchronized conditions (14l:10d) for certain targets (Supplementary Data 3), all electroporation component volumes were halved including cells (62.5 µL, 1.25 × 10^5^ cells, 2 × 10^6^ cells/mL) and RNPs (Cas12a: 0.131 nmol/25 µg, gRNA: 0.393 nmol), and *aphVIII* (1 µg, 0.9 pmol) was used instead of ssODNs. For recovery, electroporated cells were supplemented with 250 µL TAP with sucrose (40 mM), divided equally as 3 × ~100 µL into three sterile Eppendorf tubes and recovered for 24 h on an orbital shaker in either upright or horizontal position. For plating, 400 µL starch solution (30%) was added to each tube and spread onto TAP plates containing paromomycin (15–22.5 µg/mL). Identifying one mutant from each plate would ensure each mutant was an independent insertion events. Plated cells were grown under low light (5–10 μmol m^−2^ s^−1^) to limit paromomycin photodegradation. After 7 days, mutants were identified by colony PCR. An initial PCR screen involved amplifying the whole target locus allowing for 2.5–3 kb amplicons to look for *aphVIII* insertions. Unsuccessful PCR amplification were re-screened with internal *aphVIII* primers in combination with locus-specific forward/reverse primers to amplify the *aphVIII* integration junctions. Locus-*aphVIII* junctions were Sanger sequenced. Primers and PCR conditions in Supplementary Data 9. All DNA repair mutant lines are available from the Chlamydomonas Research Center, University of Minnesota (Supplementary Data 10).

### *C. reinhardtii* colony PCR

Colony PCR of *C. reinhardtii* cells was performed one of two ways. Cells were either picked into the Phire Plant Direct PCR Kit (Thermo Fisher Scientific) dilution buffer and used as per manufacturer’s instructions or picked into 10 µL TBE and Triton X-100 (0.2%), lysed at 95 °C for 5 min, of which then 0.5 µL or 1.0 µL was added to 5 µL or 10 µL GoTaq Long PCR Master Mix (Promega) reactions, respectively. Primers and PCR conditions in Supplementary Data 9.

### Generating positive and negative controls for EditR analysis

Positive control SNP-containing *FKB12* sequences were generated by overlap PCR^70^. For each desired SNP-containing sequence, two complementary primers were designed containing the desired SNP. These were used in combination with the *FKB12* forward and reverse primers to generate two PCR products (primers and PCR conditions in Supplementary Data 11). These were purified using the QIAquick Gel Extraction Kit (Qiagen), yielding 15*–*100 nM. Of each pair of corresponding PCR products (called ‘upstream arm’ and ‘downstream arm’ in Supplementary Data 11), 50 fmol was used to create an equimolar mixture (1*–*5 µL final volume), which was denatured at 95 °C for 5 min, then annealed by cooling at *−*0.3 °C/s to 20 °C. Of the resulting annealed templates, 0.5 µL was added to 50 µL Phusion PCR reactions (Thermo Fisher Scientific) as per manufacturer’s instruction using the *FKB12* forward and reverse primers (Supplementary Data 9), using the following PCR cycle with an initial extension step: 72 °C, 30 s > (98 °C, 10 s > 69 °C, 10 s > 72 °C, 30 s) × 35 > 72 °C, 5 min. Resultant full-length SNP-containing *FKB12* sequences were gel purified using the QIAquick Gel Extraction Kit (Qiagen). To finally re-amplify the full-length SNP-containing sequences, 1 µL of the purified PCR products was used as a template for 50 µL Phire Direct Plant Direct PCR Master mix (Thermo Fisher Scientific) reactions as per manufacturer’s instruction using the *FKB12* forward and reverse primers (primers and PCR conditions in Supplementary Data 9). Resulting PCR products were Sanger sequenced. A negative control was simply a sequenced PCR product of wild-type *FKB12*.

### EditR analysis of SNP experiments

Frozen cell samples (harvested post-24 h recovery after transfection with SNP-containing ssODNs) were suspended in 10 µL Phire Plant Direct PCR Kit (Thermo Fisher Scientific) dilution buffer, then *FKB12* was PCR amplified using the Phire Plant Direct PCR Master Mix as per manufacturer’s instructions and Sanger sequenced (primers and PCR conditions Supplementary Data 9). Wild-type (wt) cells were also analysed (Supplementary Fig. 5). Positive control and negative controls were generated as described under ‘Generating positive and negative controls for EditR analysis’. Sequencing Phred scores were used for plotting and chromatograms were analysed using EditR (v 1.0.10)^30^. The sequence entered for analysis by EditR spanned from 10 nucleotides upstream of the most upstream SNP (*−*32 position)—included for normalization purposes (as described later)—to the most downstream SNP (32 position), defining a 75-nucleotide sequence (AAGACTGGCCAGACCGTGTTTGTGCACTACACGGGCACCCTGACCGACGGCAAGAAGTTCGACAGCTCCCGCGAC). The 5′ start and 3′ end for analysis were set to 200 and 400, respectively, to calibrate the probability distribution models of the background base calls closely around the region of analysis for accuracy. The region of analysis was approximately between positions 285 and 360 nucleotides. SNP detection p values (Supplementary Data 12) and EditR quality metrics including average percent signal, model mu, Filliben’s correlation (Supplementary Data 13) and were compiled into spreadsheets for plotting. To determine editing levels, the chromatogram peak heights outputted by EditR were normalized using positive and negative control chromatograms as described^31^ to mitigate the local sequence-dependent variance in peak heights. The following passage makes reference to table column headings in Supplementary Data 2, which contain the sense and antisense ssODN-mediated SNP HDR data, as well as the SNP HDR data from *n* = 3 wt sequences (samples ‘None (wt sequence)’, presented in Supplementary Fig. 5). First, the nucleotide located 10 bases upstream (*−*42 position) of the most upstream SNP in the analysis (*−*32 position) was chosen for normalization (corresponding to an ‘A’ base, marked with asterisk in Fig. 3c). Next, all four base peaks (A, T, G, C) at each position within a chromatogram were normalized by dividing their peak heights by the peak height of the ‘A’ base at position *−*42 within the same chromatogram (columns ‘Relative peak heights normalized to *−*42 (A)’). Chromatogram peak heights were hereby converted from absolute values to relative ones (i.e., relative peak heights), allowing for normalization across chromatograms and thus enabling the use of positive and negative controls chromatograms. For the SNP in each chromatogram to be analysed, the relative peak height for the wt base (column ‘WT_base’) was divided by the relative peak height of wt base at the same position in the negative control chromatogram (column ‘WT base (norm_neg)’), and relative peak height for the SNP base (column, ‘SNP_base’) was divided by the relative peak height of SNP base at the same position in the positive control chromatogram created for that SNP (column ‘SNP base (norm_pos)’). Hereby, the relative peak heights were further normalized for their local sequence-dependence—an artefact of Sanger sequencing^31^. Finally, for each SNP position, editing percentage was calculated by dividing the normalized SNP peak height by the sum of the SNP and wt normalized peak heights at that position (column ‘Editing (norm)’, plotted as homology-directed repair (HDR) in Fig. 3d–i). Non-normalized values outputted directly by EditR are provided under column ‘Editing (EditR). All SNP quantification data (incl. HDR) is in Supplementary Data 2.

### Sanger sequencing

PCR reactions (5 µL) were cleaned using exonuclease I (0.2 U/μL; NEB) and shrimp alkaline phosphatase (0.07 U/μL; NEB) in 10 µL reactions by incubation at 37 °C for 30 min followed by enzyme denaturation at 80 °C for 10 min. Reactions were sequenced either using BigDye Terminator version 3.1 (Applied Biosystems) as per manufacturer’s instructions followed by capillary analysis at Edinburgh Genomics or by sending samples to Genewiz for sequencing and capillary analysis. Sequences were aligned using Jalview^71^ using the MAFFT algorithm^72^ with minor manual adjustment if needed. For SNP experiments, sequencing chromatograms were used for EditR analyses.

### Restriction enzyme control digestion

To gauge the efficiency of the restriction enzymes (REs), 20 fmol (580 ng) of two complementary ssODNs designed to contain restriction sites for both enzymes (‘ssODN_REs_ctrl’ and ‘ssODN_REs_ctrl _rc’ in Supplementary Data 8) were annealed in NEB2 buffer (1×) in a final volume of 12 µL by denaturing at 98 °C for 5 min, then cooled at *−*0.1 °C/s to 20 °C (‘ssODN_REs_ctrl’ and ‘ssODN_REs_ctrl_rc’ in Supplementary Data 8). Then, 200 ng (2 µL) of annealed ssODNs was digested with either *Bfa*I or *Pvu*II-HF (1 U, New England Biolabs) in CutSmart buffer (1×) in a total volume of 10 µL and incubated at 37 °C for 1 h. Immediately after digestion, reactions were separated on an agarose gel (1.5%) stained with SYBR Safe (1×), imaged (UVP BioDoc-It) and analysed semi-quantitatively by gel densitometry using ImageJ (Supplementary Data 14).

### Restriction enzyme site ssODN analysis

Cells were transfected with ssODNs designed to contain restriction enzyme (RE) sites (‘ssODN_REs’ and ‘ssODN_REs_rc’ in Supplementary Data 8). Cells were recovered for 2 days on an orbital shaker to yield enough cells for harvest. After recovery, cells were harvested by centrifugation (17,000 × *g*, 1 min), supernatant was removed, and DNA was extracted using CTAB buffer as described^73^. Briefly, cells were suspended in 0.7 mL CTAB buffer pre-heated to 65 °C (100 Tris, pH 8.0, 20 mM EDTA, 1.4 M NaCl, 2% w/v polyvinyl pyrrolidone, 2% w/v CTAB), incubated at 65 °C for 1 h, then DNA was extracted using standard phenol:chloroform extraction (using 0.7 mL solvent), and resuspended in 15 µL DEPC-treated H_2_O. Recovered DNA was 1–40 µg per sample (80–2800 ng/µL). *FKB12* was amplified by adding 1 µL DNA into 200 µL GoTaq Long PCR Master Mix (Promega) reactions as per manufacturer’s instructions. PCR reactions were purified using the MinElute PCR Purification Kit (QIAGEN) and measured using a Nanodrop (Thermo Fisher Scientific). DNA (1500 ng) was digested using either *Bfa*I or *Pvu*II-HF (20 U, New England Biolabs) in CutSmart buffer (1×) in 20 µL reactions by incubating at 37 °C for 1 h. Immediately thereafter, reactions were resolved on an agarose gel (1.5%) stained with SYBR Safe (1×), imaged (UVP BioDoc-It), and quantified semi-quantitatively using ImageJ (v1.51j8, raw ImageJ values in Supplementary Data 14).

### Biotinylated ssODN transfection and pulldown

An ssODN (100 nt) was designed with a biotinylated thymidine at the centre of the Cas12a-induced staggered cut, surrounded by 12 nt of non-homology to the *FKB12* target which carried stop codons on each reading frame (‘ssODN_bio’ in Supplementary Data 8). A non-biotinylated variant was also designed (‘ssODN_nobio’ in Supplementary Data 8). Cells were transfected with either the biotinylated or the non-biotinylated ssODN as described under ‘C. reinhardtii transfection using ssODNs’, except with double volumes for all components, including Cas12a (0.524 nmol/100 µg), gRNA (1.578 nmol), cells (250 µL, 5 × 10^5^ cells, 2 × 10^6^ cells/mL), and ssODN (5.26 nmol). For a control transfection with the biotinylated ssODN but no gRNA, Cas12a was still incubated at 37 °C for 10 min. After transfection, 250 µL TAP with sucrose (40 mM) was added and cells were transferred to Eppendorf tubes for overnight (24 h) recovery on an orbital shaker. Afterwards, cells were harvested by centrifugation (17,000 × *g*, 2 min), supernatant was removed and cells were stored at −80 °C until use. DNA was extracted from thawed cells using the GenElute Plant Genomic DNA Miniprep Kit (Sigma) as per manufacturer’s instructions, which is optimized for recovery of long genomic DNA, with use of RNAse A and elution in 50 µL volume. DNA was measured using Qubit DNA dsDNA HS Assay Kit (Invitrogen) as per manufacturer’s instructions. Recovered DNA was 60–80 ng corresponding to ~5 × 10^5^ genomes (assuming 660 Da/bp, 120 Mbp genome^22^). All further steps were performed using filter pipettes to minimize aerosol contamination. DNA was co-digested using *Nco*I, *Pvu*II-HF (1 U each, New England Biolabs) and exonuclease I (2 U, New England Biolabs) in CutSmart buffer (1×) at 37 °C for 1 h followed by 80 °C for 10 min for enzyme denaturation in a final volume of 60 µL. Streptavidin-coated magnetic beads (10 µL, Sera-Mag, medium binding capacity 3500–4500 pmol/mg, GE Healthcare) were transferred to Eppendorf tubes and washed twice using wash buffer (5 mM Tris [pH 8], 1 M NaCl, 0.5 mM EDTA, 0.1% SDS, 1 mg/mL BSA). Washing consisted of mixing then placing beads on a magnetic stand (Promega) and removing supernatant. Of the treated DNA, 50 µL was added to the beads (10 µL left behind for subsequent PCR of pre-pulldown DNA) and mixed by vortexing for 15 min (TopMix FB15024, Thermo Fisher Scientific) on a low speed (the lowest possible speed on the vortex). Beads were separated on a magnetic stand, supernatant was discarded. Beads were washed five times using wash buffer, once with DEPC-treated water, and resuspended in DEPC-treated water (10 µL). Washes consisted of vortexing on low speed (as before) for 5 min, separating beads on a magnetic stand and discarding the supernatant. To perform PCR, digested pre-pulldown DNA (0.5 µL) or post-pulldown beads (0.5 µL) were added to 5 µL GoTaq Long PCR Master Mix (Promega) as per manufacturer’s instructions (primers and PCR conditions Supplementary Data 9). PCR products were resolved on an agarose gel (2%) stained with SYBR Safe (1×) and imaged (UVP BioDoc-It). Global image adjustments to the gel image were performed using GNU Image Manipulating Program (GIMP).

### RNA-seq re-analysis

Zones et al.^33^ generated hourly gene expression (RPKM) using cells grown under synchronized conditions. These were plotted for selected genes using authors’ Supplementary Data Set 1^33^. Lack of raw data for the mitotic index necessitated tracing the authors’ Fig. 1c using vector graphics (Inkscape). Average gene expression was calculated by first averaging the expression level of each of two replicates across the entire 24 h sampling period, followed by averaging of the two replicates.

### MMC and zeocin assays

Mitomycin C (MMC, Stratech) was dissolved in DMSO to 10 mg/mL to create a stock solution. Zeocin (100 mg/mL, Invitrogen) was diluted to 10 mg/mL using distilled water. MMC and zeocin were added to lukewarm, liquid TAP agar at concentrations indicated in the figures. For each experiment, liquid *C. reinhardtii* cultures were normalized by OD_750_ and used to create a (5- or 10-fold) dilution series in starch solution (30%), which were then dotted (5–10 µL) onto plates. All plates were grown under low light (5–10 μmol m^−2^ s^−1^) to limit zeocin/MMC photodegradation and imaged after 7 d of growth (Canon PowerShot G16), except MMC plates containing the Fanconi anemia (FA) mutants which, after 7 d under low light, were kept in the dark for 10 to exacerbate the MMC-induced hypersensitive phenotypes. Images were globally colour-adjusted using GIMP to facilitate visual comparison across experiments.

### Statistical analyses

All statistics were performed in JASP (v0.14.1). All transfections were independent biological replicates. Transfections performed with the same strain were considered biological replicates if they were performed on separately grown liquid cultures; most biological replicates were also performed on different dates. Averages are means. Error bars are standard deviations. All data is treated as parametric and assumed ANOVA being robust to slight normality violations. Since repeated plating of a mixture of wild type (wt) and *fkb12* cells using the fkb12 assay showed normal data distribution (Shapiro–Wilk *n* = 10, skewness = 0.896, *p* = 0.197, Supplementary Fig. 1b, c), but some biologically replicated fkb12 assays were non-normal (see Shapiro–Wilk *p* values in Supplementary Data 1), biological outliers in fkb12 assay results (black circles in Fig. 2, Fig. 5a,d) were identified using the Tukey’s fences method (6/72 datapoints, Supplementary Data 1), where outliers are datapoints residing below $Q1-1.5\times {{{{{{\rm{IQR}}}}}}}$ or above $Q3+1.5\times {{{{{{\rm{IQR}}}}}}}$ ($Q1$: first quartile, $Q3$, third quartile, ${{{{{{\rm{IQR}}}}}}}$: interquartile range, quartiles were determined using Excel’s inclusive method). Biological outliers were only detected for experiments with ≥5 biological replicates as this is the minimum amount we deem meaningful for quartile determination. Outliers were identified, but retained in all analyses, including mean averaging and statistical testing.

### Reporting summary

Further information on research design is available in the Nature Research Reporting Summary linked to this article.

## Supplementary information

Supplementary Information

Description of Additional Supplementary Files

Supplementary Data 1

Supplementary Data 2

Supplementary Data 3

Supplementary Data 4

Supplementary Data 5

Supplementary Data 6

Supplementary Data 7

Supplementary Data 8

Supplementary Data 9

Supplementary Data 10

Supplementary Data 11

Supplementary Data 12

Supplementary Data 13

Supplementary Data 14

Supplementary Data 15

Supplementary Data 16

Supplementary Data 17

Supplementary Data 18

Reporting Summary

## Data Availability

Colony counts underlying fkb12 assay results (Fig. 2 and Fig. 5a, d) are in Supplementary Data 1, plate images supporting these colony counts are in Source Data. EditR SNP quantification (HDR) results for sense ssODNs (Fig. 3d–f), antisense ssODNs (Fig. 3g–i) and wt sequences (Supplementary Fig. 5b) are in Supplementary Data 2; *p* values of SNP detection (Fig. 3d–I, Supplementary Fig. 5b, Supplementary Data 2) are in Supplementary Data 12; EditR quality control metrics including average noise, model µ and Filliben’s coefficient (Supplementary Data 2) are in Supplementary Data 13; EditR raw output files from which all these metrics were derived and the sequencing chromatograms used both as an input for EditR and to derive Phred scores are in the Source Data. Restriction digestion values (Fig. 3l) and the gel images which these are based on are in Supplementary Fig. 6a and raw gel densitometry ImageJ values used to quantify restriction digestion are in Supplementary Data 14 including the control digestion in Supplementary Fig. 6c. Colony PCR data (Fig. 5b, Supplementary Fig. 12b) are in Supplementary Data 4, raw sequences and corresponding alignment categories (SSTR—scarless, SSTR—scarred, Indel) are in the Source Data. Population-level scarless SSTR data (Fig. 5c) is in Supplementary Data 5. DNA repair mutant cell lines are available from the Chlamydomonas Research Center, University of Minnesota (Supplementary Data 10). All unprocessed gel images (relating to Fig. 4b and Supplementary Fig. 6a, c) are in Supplementary Fig. 14. Source data are provided with this paper.

## Source data

Source Data
