# Supplementary material for: Mechanistic and genetic basis of single-strand templated repair at Cas12a-induced DNA breaks in Chlamydomonas reinhardtii
Source: Nat Commun. 2021 Nov 19;12:6751. doi: 10.1038/s41467-021-27004-1 (PMC8604939; doi:10.1038/s41467-021-27004-1)
Supplement: Supplementary file 1 — Supplementary Information [file 41467_2021_27004_MOESM1_ESM.pdf]

Supplementary Information

Mechanistic and genetic basis of single-strand templated repair at Cas12a-induced DNA breaks in *Chlamydomonas reinhardtii*

Aron Ferenczi[1], Yen Peng Chew[1], Erika Kroll[1,2], Charlotte von Koppenfels[1], Andrew Hudson[1], Attila Molnar[1]*

[1]Institute of Molecular Plant Sciences, University of Edinburgh, Edinburgh, EH9 3BF, United Kingdom

[2]current address: Department of Biointeractions and Crop Protection, Rothamsted Research, Harpenden, AL5 2JQ, United Kingdom.

*corresponding author, attila.molnar@ed.ac.uk

p.2 | Supplementary Fig. 1. Demonstration and characterization of the fkb12 assay.

p.3 | Supplementary Fig. 2. illustrations of nucleotide modifications and modified ssODNs.

p.4 | Supplementary Fig. 3. Quality control metrics for SNP results using sense ssODNs

p.6 | Supplementary Fig. 4. Quality control metrics for SNP results using antisense ssODNs

p.8 | Supplementary Fig. 5. EditR analysis of wt sequences (n=3).

p.9 | Supplementary Fig. 6. Supporting information for restriction site introduction into *FKB12*

p.10 | Supplementary Fig. 7. RNA sequencing analysis re-plotted from Zones *et al.* (2015).

p.11 | Supplementary Fig. 8. Homologous recombination (HR) mutant characterization.

p.13 | Supplementary Fig. 9. Fanconi anemia (FA) mutant characterization.

p.14 | Supplementary Fig. 10. Non-homologous end-joining (NHEJ) mutant characterization.

p.15 | Supplementary Fig. 11. Alternative end-joining (alt-EJ) mutant characterization.

p.16 | Supplementary Fig. 12. Speculated roles of polymerase θ in ssDI.

p.18 | Supplementary Fig. 13. Recreation and analysis of Richardson *et al.* (2018) Fig. 1b.

p.19 | Supplementary Fig. 14. Raw, uncropped gel images.

Supplementary Fig. 1

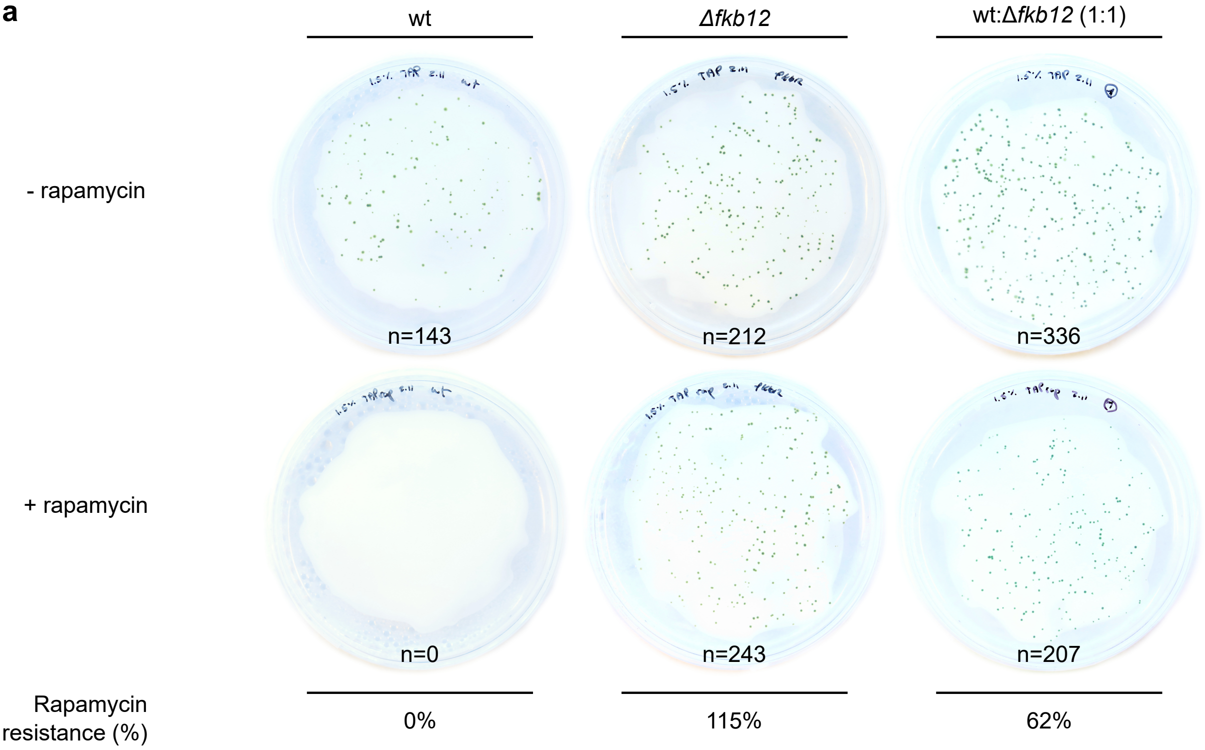

b

	Colony count		
**Sample**	**- rapamycin**	**+ rapamycin**	**Rapamycin resistance (%)**
wt	143	0	0%
Δ fkb12	212	243	115%
mix 1	336	207	62%
mix 2	358	222	62%
mix 3	344	205	60%
mix 4	339	250	74%
mix 5	350	205	59%
mix 6	360	241	67%
mix 7	341	256	75%
mix 8	332	272	82%
mix 9	416	234	56%
mix 10	381	238	62%

c

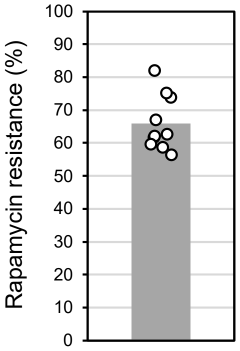

Demonstration and characterization of the fkb12 assay. **a**, Wild-type (wt), *fkb12*, and a 1:1 mix of wt:*fkb12* cells plated on media without and with rapamycin for total cell count (n) and rapamycin-resistant (*fkb12*) cell count, respectively. Deviation of the plated *fkb12* cells from the expected 100% rapamycin resistance reflects assay variance. **b**, A single mix of wt:*fkb12* cells plated ten times using the fkb12 assay and used to test data (i.e., rapamycin resistance) distribution normality and variation (Shapiro-Wilk normality test: n=10, skewness=0.896, p=0.197; coefficient of variation 13%). High-quality plate images are in Source Data. **c**, Rapamycin resistance values plotted from panel **b**.

Supplementary Fig. 2

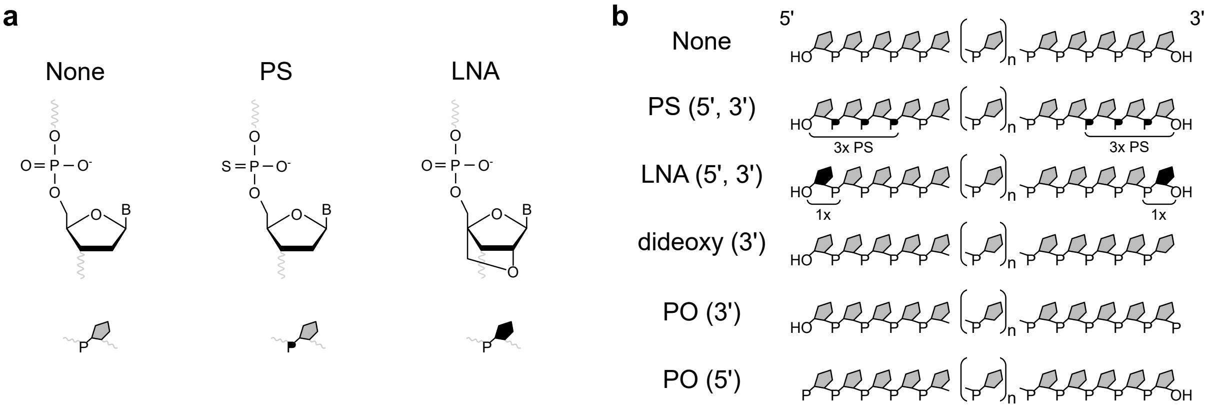

Illustrations of nucleotide modifications (**a**) and modified ssODNs (**b**). ssODN termini are referred to as 5' and 3'. White circles: phosphates, grey pentagons: deoxyribose sugars, PS: phosphothioate, LNA: locked nucleic acid, PO: phosphate.

Supplementary Fig. 3

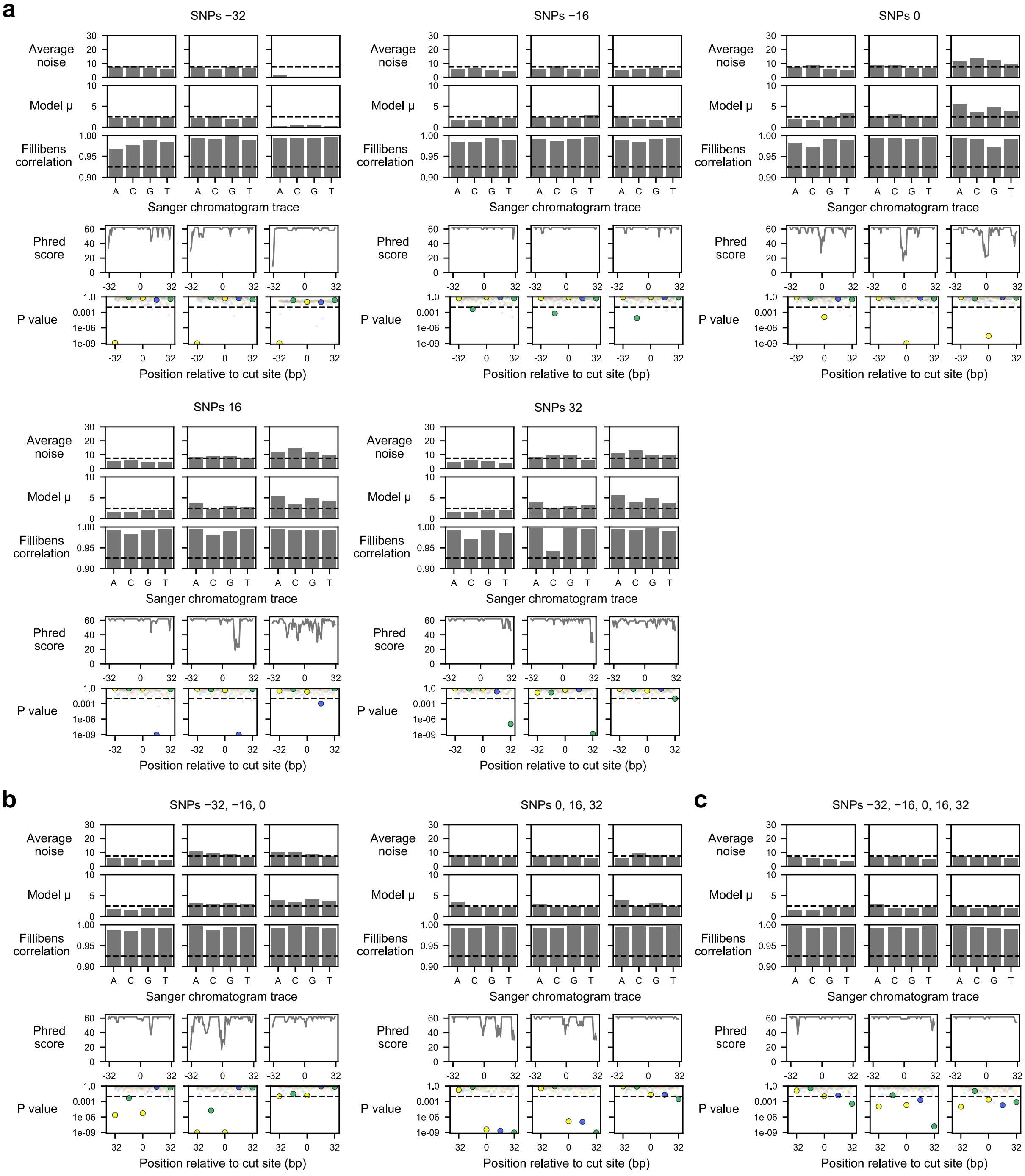

Quality control metrics for SNP results obtained using sense ssODNs (relating to Fig. 3d–f); either five ssODNs carrying one SNP each (**a**), two ssODNs carrying either all up- or downstream SNPs (**b**) or one ssODN carrying all five SNPs (**c**). Three columns of graphs represent biological repeats 1–3 from left-to-right. EditR metrics include average sequencing noise (≤7.5%), model μ (≤2.5%) and Filliben's correlation coefficient (≥92.5%) – EditR authors' recommended guidelines are in brackets and are plotted as dotted lines[30]. Additional plotted quality indicators are sequencing Phred scores and EditR detection p values for each nucleotide in each of the 65 positions contained within the region of analysis

(the intended SNP bases are larger opaque circles). HDR data are in Supplementary Data 2 (column, ‘Editing (norm)’), p values are in Supplementary Data 12, quality control metrics are in Supplementary Data 13, EditR raw output files and input sequencing chromatograms (including Phred scores) are in the Source Data. Blue: adenosine (A), red: cytidine (C), green: guanosine (G), yellow: thymidine (T).

Supplementary Fig. 4

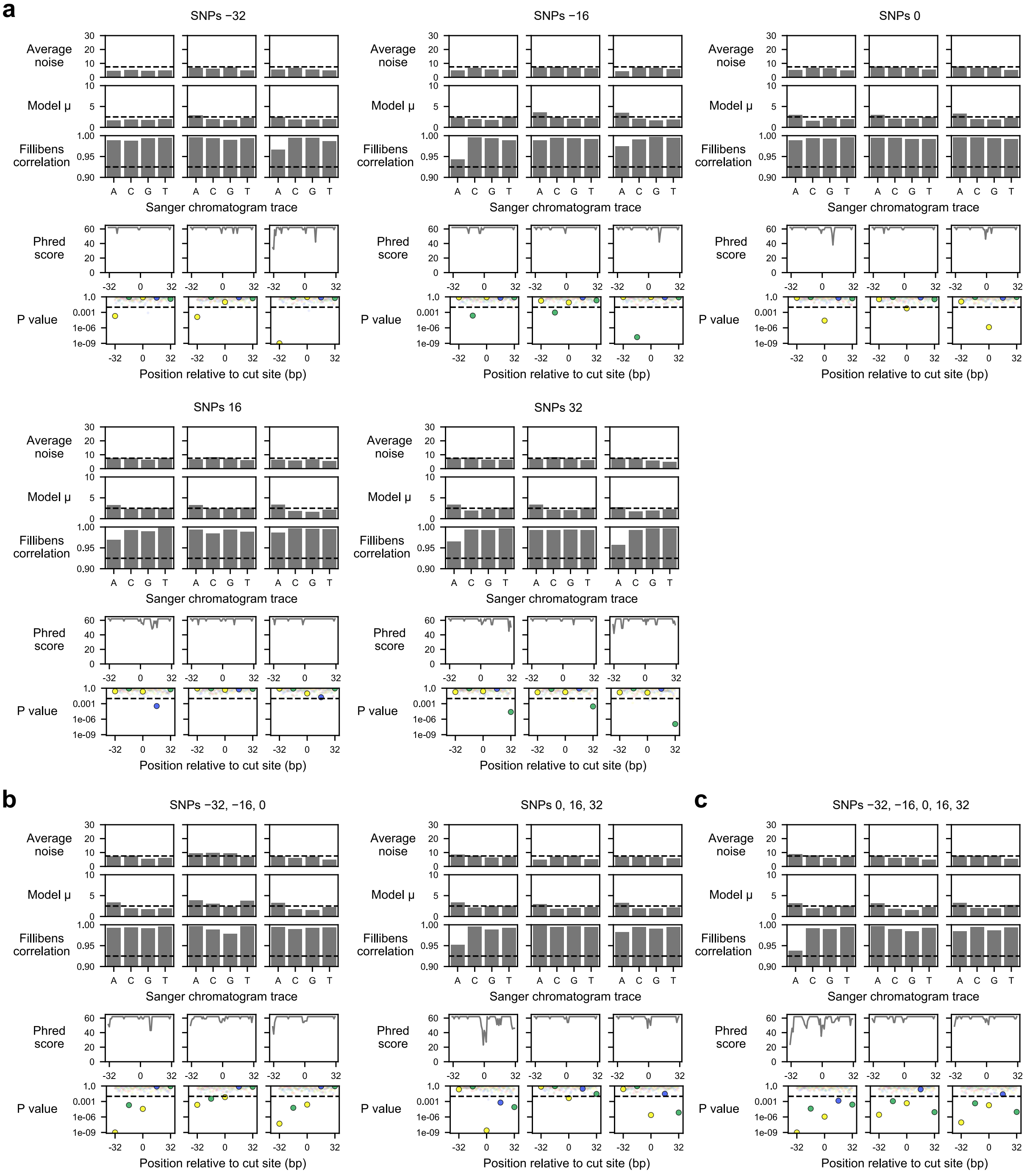

Quality control metrics for results obtained using antisense ssODNs (relating to Fig. 3g–i); either five ssODNs carrying one SNP each (**a**), two ssODNs carrying either all up- or downstream SNPs (**b**) or one ssODN carrying all five SNPs (**c**). Three columns of graphs represent biological repeats 1–3 from left-to-right. EditR metrics include average sequencing noise (≤7.5%), model μ (≤2.5%) and Filliben's correlation coefficient (≥92.5%) – EditR authors' recommended guidelines are in brackets and are plotted as dotted lines[30]. Additional plotted quality indicators are sequencing Phred scores and EditR detection p values for each nucleotide in each of 65 positions contained within the region of analysis (the

intended SNP bases are larger opaque circles). HDR data are in Supplementary Data 2 (column, ‘Editing (norm)’), p values are in Supplementary Data 12, quality control metrics are in Supplementary Data 13, EditR raw output files and input sequencing chromatograms (including Phred scores) are in the Source Data. Blue: adenosine (A), red: cytidine (C), green: guanosine (G), yellow: thymidine (T).

Supplementary Fig. 5

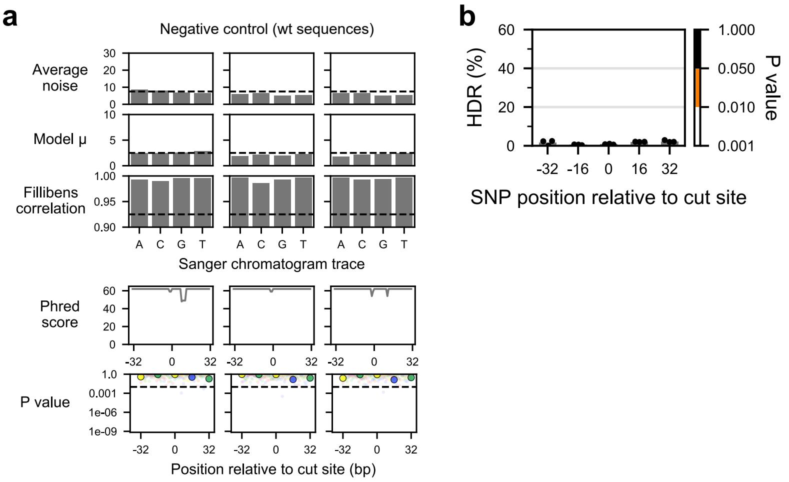

EditR analysis of wt sequences (n=3 biological repeats, separately grown cultures). **a,** Quality control metrics. Three columns of graphs represent repeats 1–3 from left-to-right. EditR metrics include average sequencing noise (≤7.5%), model μ (≤2.5%) and Filliben's correlation coefficient (≥92.5%) – EditR authors' recommended guidelines are in brackets and are plotted as dotted lines[30]. Additional plotted quality indicators are sequencing Phred scores and EditR detection p values for each nucleotide in each of 65 positions contained within the region of analysis (the intended SNP bases are larger opaque circles). **b,** Homology-directed repair (HDR, i.e., SSTR) quantified from wt sequences (which do not contain SNPs). P values relate to the significance of SNP detection from the chromatogram background noise by EditR (i.e., SNPs above α=0.05 are indistinguishable from background noise, inversely correlates with level of editing and sequencing quality). Blue: adenosine (A), red: cytidine (C), green: guanosine (G), yellow: thymidine (T). HDR data are in Supplementary Data 2 (column, 'Editing (norm)'), p values are in Supplementary Data 12, quality control metrics are in Supplementary Data 13, EditR raw output files and input sequencing chromatograms (including Phred scores) are in the Source Data.

Supplementary Fig. 6

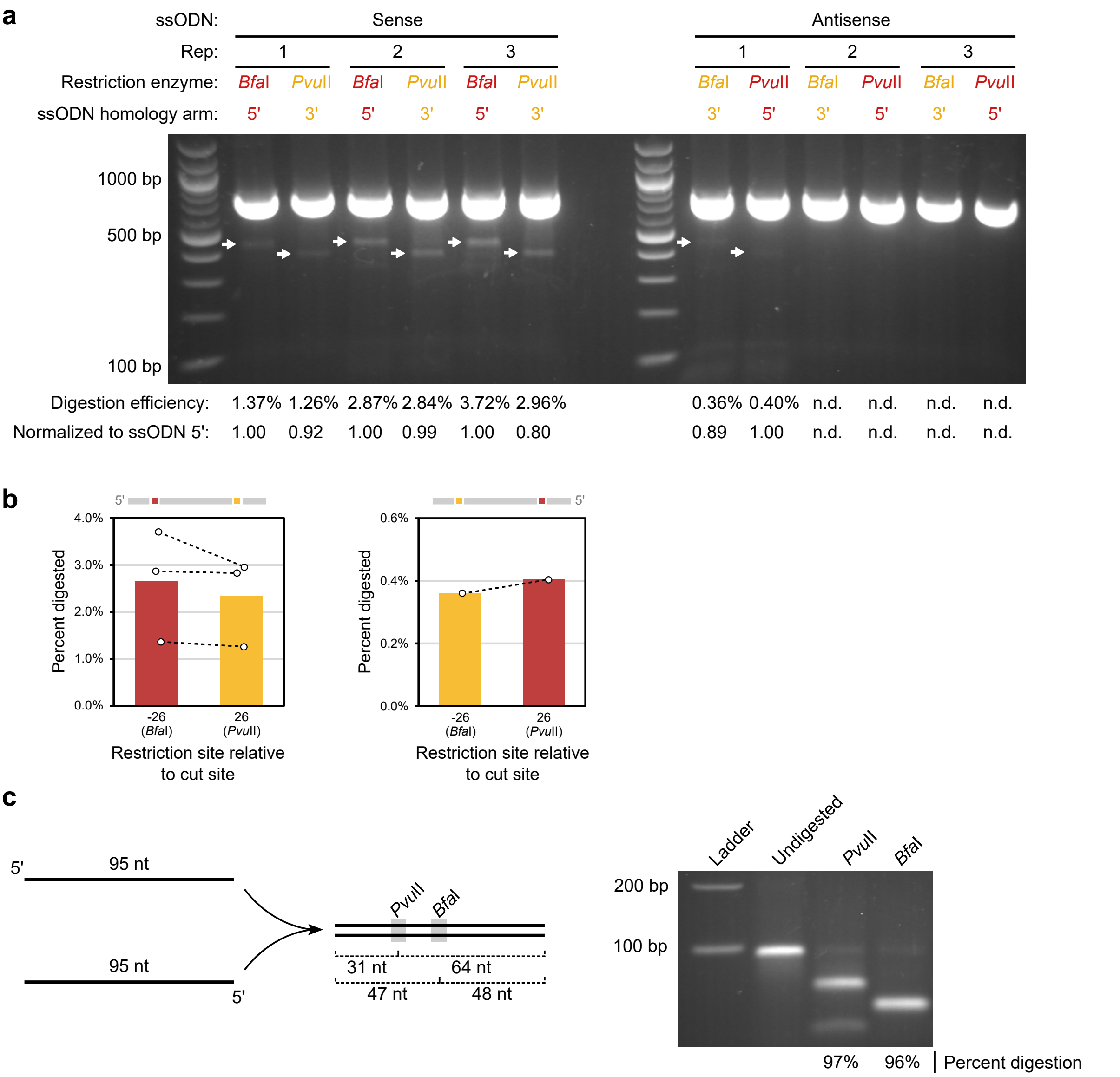

Supporting information for ssODN-mediated restriction site introduction into *FKB12* (Fig. 3j–l). **a**, Gel images used to analyse digestion efficiency by gel densitometry semi-quantitatively using ImageJ and then normalized to the restriction site that is present on the ssODN 5' (*Bfa*I for sense ssODNs, *Pvu*II for antisense ssODNs). White arrows indicate digestion products. Normalized digestion efficiencies are presented in Fig. 3l. Gel densitometry band (pixel) intensities underlying the quantification are in Supplementary Data 14. **b**, Non-normalized digestion efficiencies plotted from panel **a**. Dotted lines connect paired values from the same experiment. **c**, Control digestion using either *Bfa*I or *Pvu*II-HF by annealing together two complementary ssODNs carrying restriction sites for both enzymes ('ssODN_REs_ctrl' and 'ssODN_REs_ctrl_rc' in Supplementary Data 8). Percent digestion semi-quantified using ImageJ. ImageJ data in Supplementary Data 14. Raw gel images are in Supplementary Figure 14.

Supplementary Fig.7

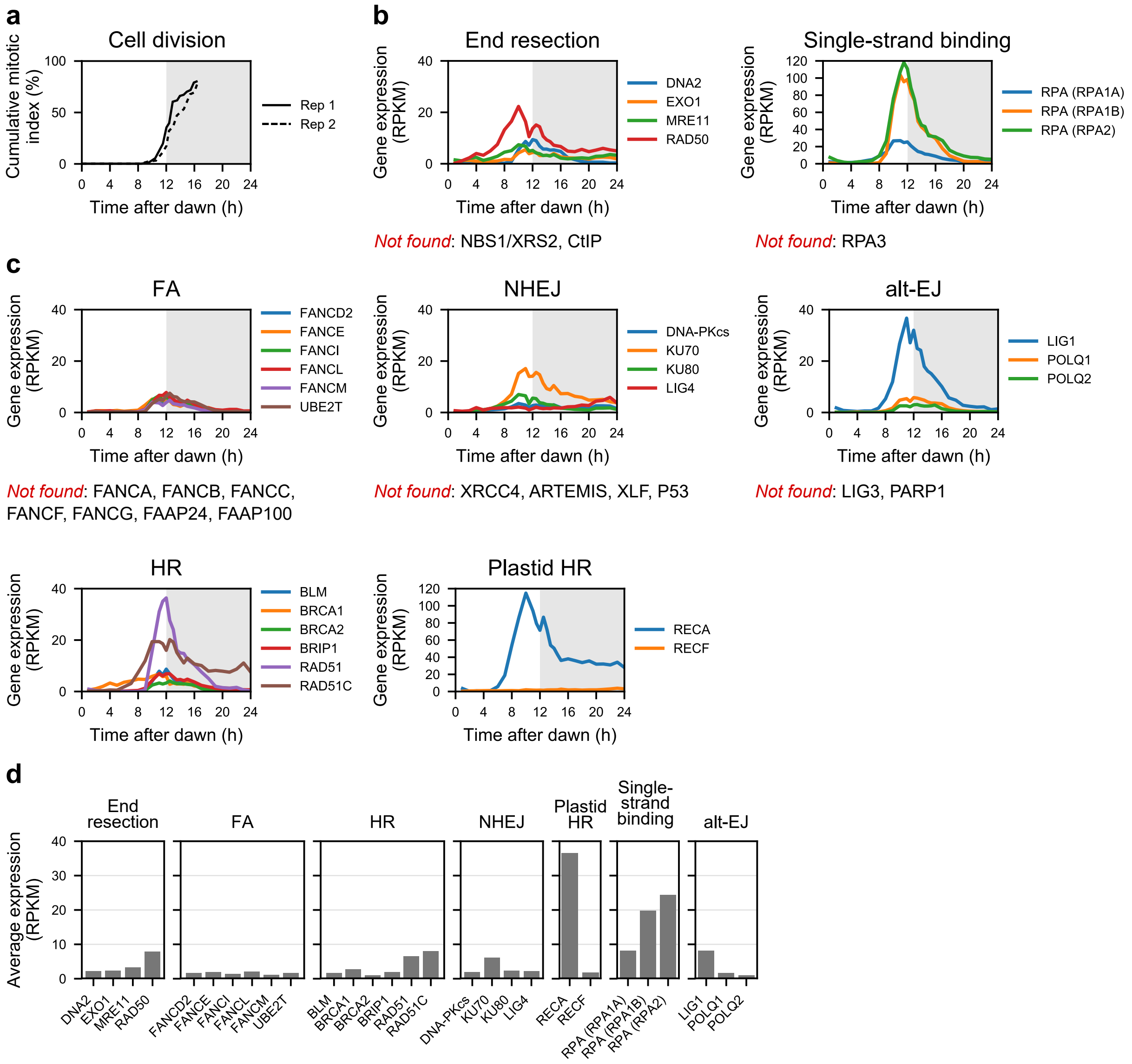

RNA sequencing analysis re-plotted from Zones *et al.* (2015) Supplementary Data Set 1[33]. Gene expression in reads per kilobase per million mapped (RPKM). Genes not found in the genome (v5.5) by keyword searching on Phytozome (v12) are shown as ‘Not found’. Shaded region represents the dark phase of cell growth. **a**, Cumulative mitotic index i.e., fraction of cells passed division. **b**, Expression of DNA end-resection and single-strand DNA binding proteins. **c**, Expression of selected Fanconi anemia (FA), non-homologous end-joining (NHEJ), alternative end-joining (alt-EJ), homologous recombination (HR) and plastid HR (i.e., chloroplast HR) genes. **d**, Average expression for genes in panels **b**, **c**.

Supplementary Fig. 8

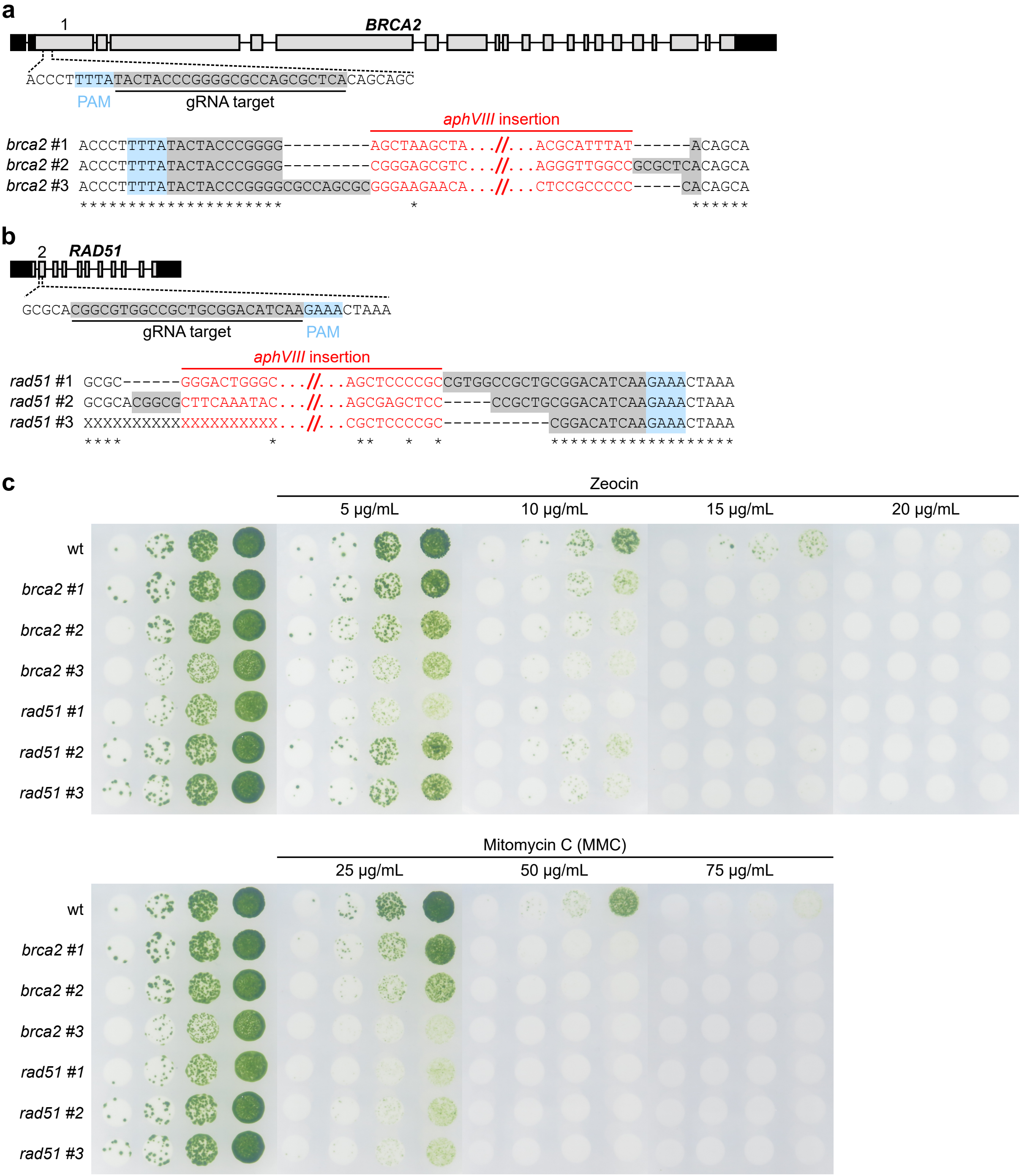

Homologous recombination (HR) mutant characterization. **a,b**, Sequencing of the genome-*aphVIII* junctions for *brca2* (**a**) and *rad51* (**b**) lines. The wt sequences annotated with the PAM (blue) and gRNA target (grey) are shown in the schematics of the corresponding loci, which are drawn to scale (grey boxes: exons, lines: introns, black boxes: 5' and 3' untranslated regions). The exon number targeted by the gRNA is shown above the exon. Series of 'X's represent DNA we failed to amplify in *rad51* #3. Stars (*) denote nucleotides where all sequences match (excluding 'X's). The *aphVIII* insertion is in red. Series of dots

(…) with a central double-lined break (//) represent a variable-length sequence break. **c**, Dot assays using wt, *brca2* and *rad51* lines on zeocin and mitomycin C (MMC) at the indicated concentrations. The non-selective plate image (left) in the top (zeocin) and bottom (MMC) panels is the same image since all plates were plated on the same day using the same prepared cell dilutions, thus it serves a non-selective control for both series of plates.

Supplementary Fig. 9

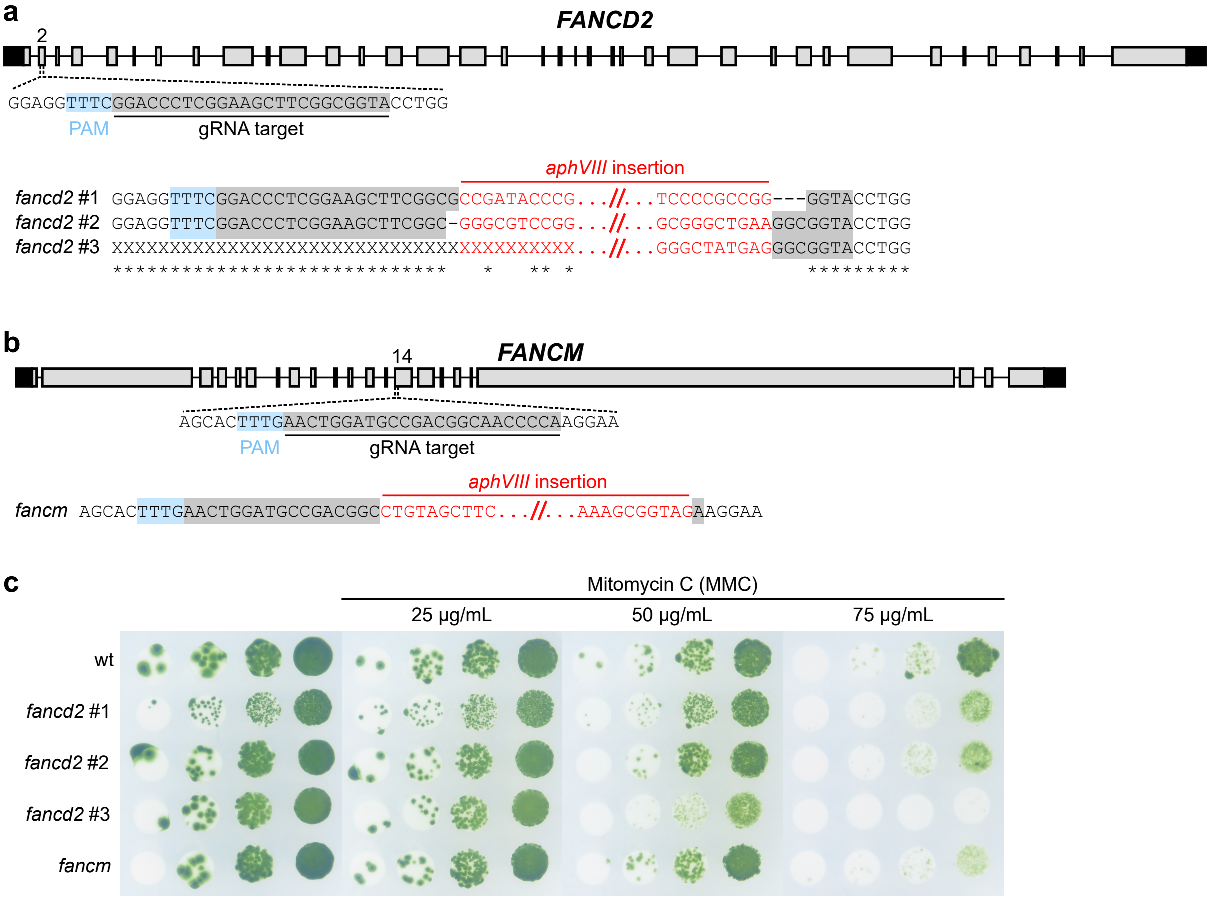

Fanconi anemia (FA) mutant characterization. **a**,**b**, Sequencing of the genome-*aphVIII* junctions for *fancd2* (**a**) and *fancm* (**b**) lines. The wt sequences annotated with the PAM (blue) and gRNA target (grey) are shown in the schematics of the corresponding loci, which are drawn to scale (grey boxes: exons, lines: introns, black boxes: 5' and 3' untranslated regions). The exon number targeted by the gRNA is shown above the exon. Series of 'X's represent DNA we failed to amplify in *fancd2* #3 using primers as far away as 10 kbp from the expected *aphVIII* insertion site. Stars (*) denote nucleotides where all sequences match (excluding 'X's). The *aphVIII* insertion is in red. Series of dots (…) with a central double-lined break (//) represent a variable-length sequence break. **c**, Dot assays using wt, *fancd2* and *fancm* lines on mitomycin C (MMC) at the indicated concentrations. All plates were plated on the same day using the same prepared cell dilutions.

Supplementary Fig. 10

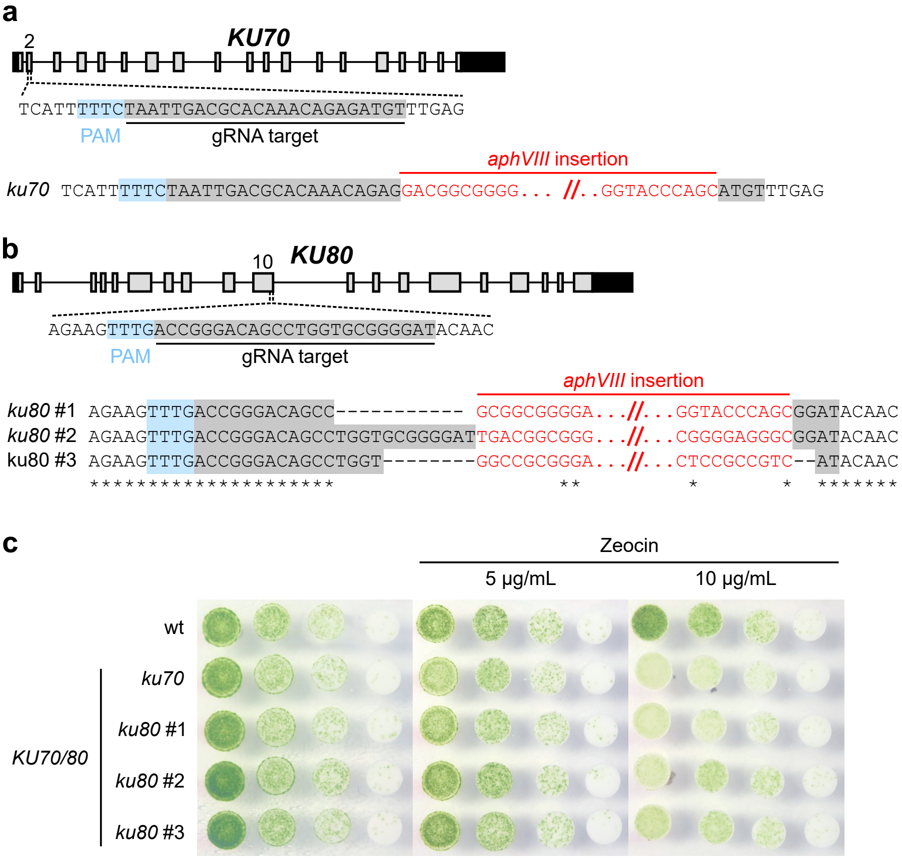

Non-homologous end-joining (NHEJ) mutant characterization. **a**,**b**, Sequencing of the genome-*aphVIII* junctions for *ku70* (**a**) and *ku80* (**b**) lines. The wt sequences annotated with the PAM (blue) and gRNA target (grey) are shown in the schematics of the corresponding loci, which are drawn to scale (grey boxes: exons, lines: introns, black boxes: 5’ and 3’ untranslated regions). The exon number targeted by the gRNA is shown above the exon. Stars (*) denote nucleotides where all sequences match. The *aphVIII* insertion is in red. Series of dots (…) with a central double-lined break (//) represent a variable-length sequence break. **c**, Dot assays using wt, *ku70* and *ku80* lines on zeocin at the indicated concentrations. All plates were plated on the same day using the same prepared cell dilutions.

Supplementary Fig. 11

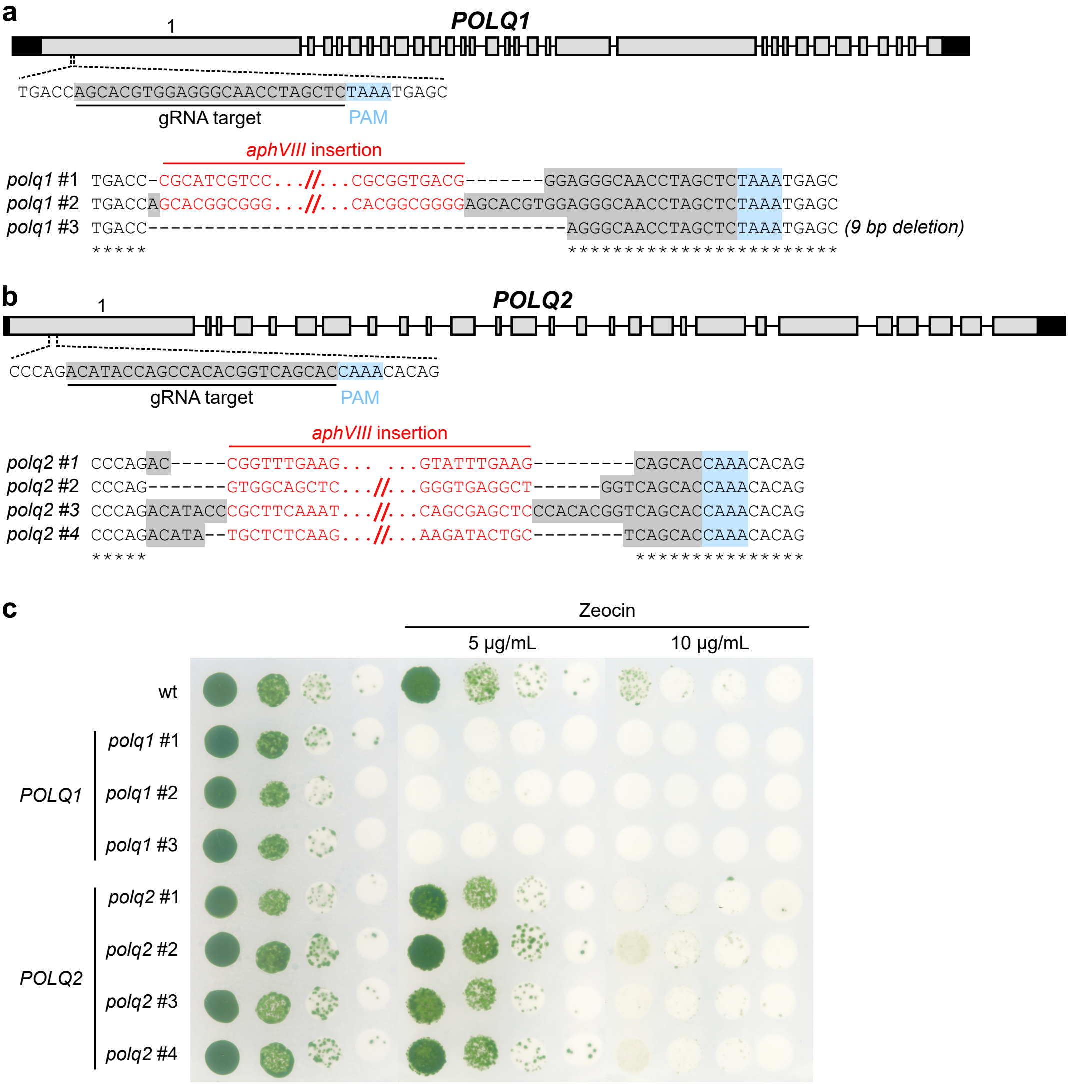

Alternative end-joining (alt-EJ) mutant characterization. **a,b**, Sequencing of the genome-*aphVIII* junctions for *polq1* (**a**) and *polq2* (**b**) lines. The wt sequences annotated with the PAM (blue) and gRNA target (grey) are shown in the schematics of the corresponding loci, which are drawn to scale (grey boxes: exons, lines: introns, black boxes: 5' and 3' untranslated regions). The exon number targeted by the gRNA is shown above the exon. Stars (*) denote nucleotides where all sequences match. The *aphVIII* insertion is in red. Series of dots (…) with a central double-lined break (//) represent a variable-length sequence break. **c**, Dot assays using wt, *polq1* and *polq2* lines on zeocin at the indicated concentrations. All plates were plated on the same day using the same prepared cell dilutions.

Supplementary Fig. 12

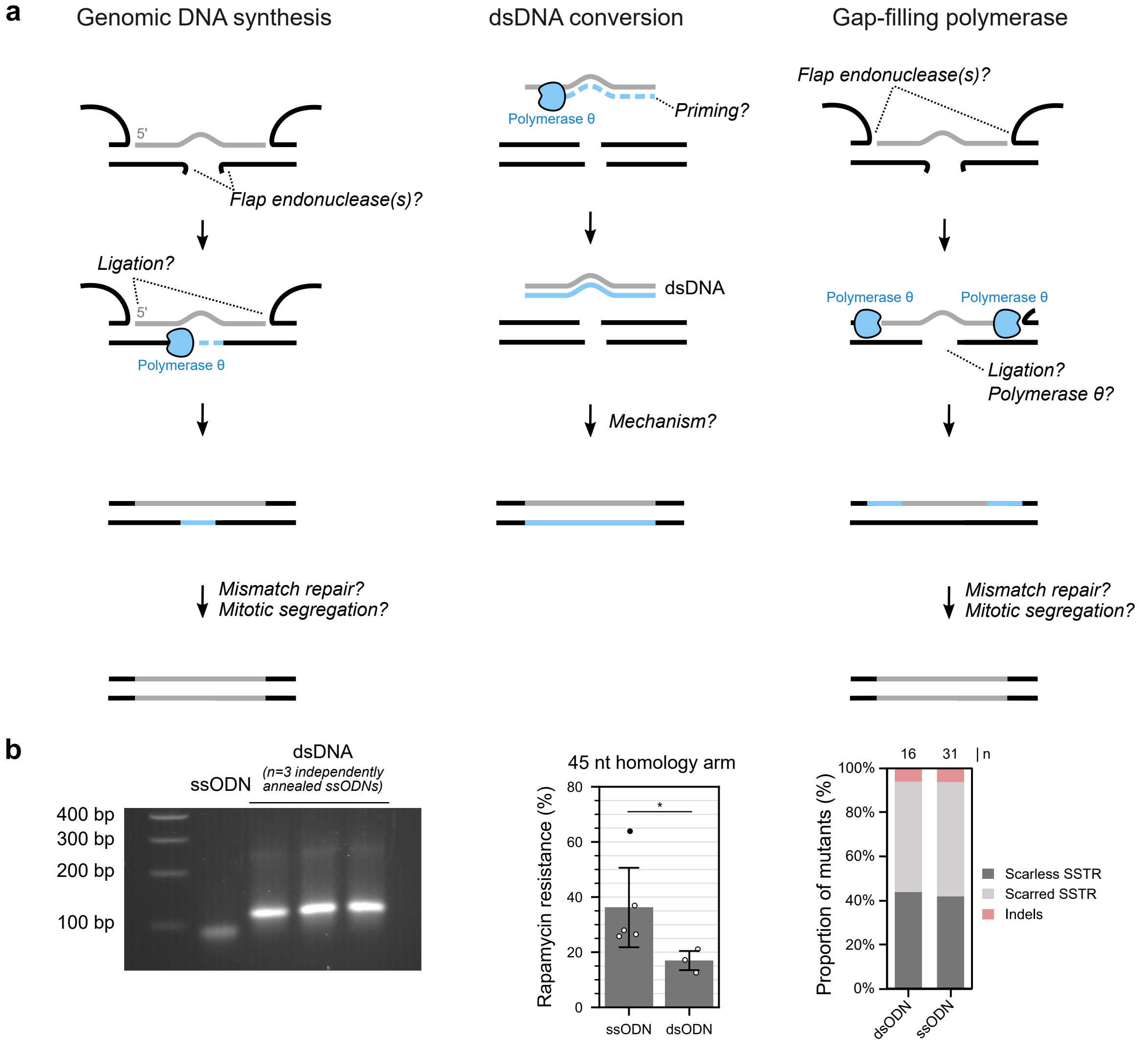

Speculative models of polymerase θ-mediated ssDI. Assumed (unknown) steps are stated with question marks. **a,** Genomic DNA synthesis (left): the ssODN ligates into the genome and polymerase θ may repair the second genomic DNA strand. Heteroduplex DNA may be resolved by mismatch repair (MMR) or mitotic segregation. dsDNA conversion (middle): polymerase θ may convert ssODNs into dsDNA through an unknown (perhaps random) priming mechanism[50] and subsequently achieve editing via an unknown DNA repair pathway mechanism (not HR, Fig. 5). Gap-filling polymerase (right): polymerase θ may synthesize DNA on either side of the ssODN after it anneals (replacing or preceding ligation). The second strand may be ligated or repaired by polymerase θ (by the same mechanism), with heteroduplex DNA subsequently resolved by MMR or mitotic segregation. **b**, dsDNA-mediated editing. Left: complementary ssODNs were annealed (n=3) to make dsDNA. Middle: dsDNA editing (17.0%, n=3) was lower than ssODN-mediated (36.2%, n=5) editing using ssODNs with 45 nt homology arms (one-sided Student's t-test t(6)=−1.921, p=0.048, $H_a: dsDNA > ssODN$, Levene's test p=0.209). Repeats are biological (separately grown

cultures). Bars are mean averages. Error bars are standard deviations. Data in Supplementary Data 1. Right: Colony PCR of rapamycin-resistant cells with samples sizes (n) indicated (top); ssODN results are replotted from our prior work[3]. Scarred SSTR was defined as any editing event that included at least part of the central non-homologous ssODN sequence, but that was not a perfect homology-directed event and contained unintended SNPs, insertions, deletions, and local sequence duplications. Data in Supplementary Data 4* $p<0.05$, H_a: alternative hypothesis, n: sample size.

Supplementary Fig. 13

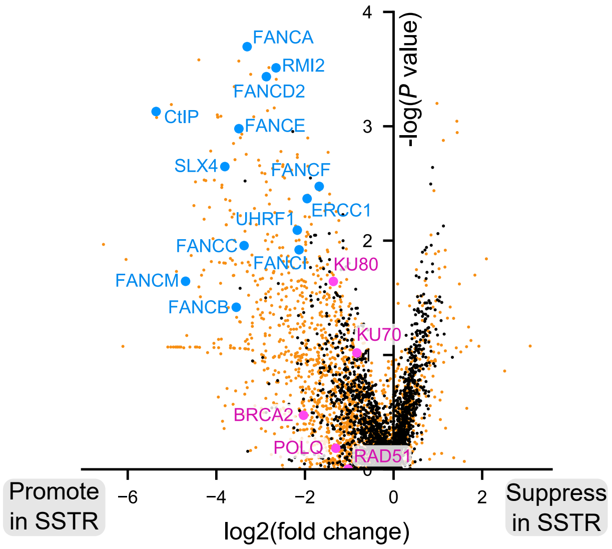

Recreation and supplementary analysis of Richardson *et al.* (2018) Fig. 1b[10]. Figure plotting using authors' Supplementary Data 1 worksheet 'SSTR'. Genes analysed in our study were identified in the authors' dataset and plotted in pink (note: though we also analyzed FANCD2 and FANCM, these are blue to recreate the authors' Fig. 1b[10]). Blue: FA genes as highlighted in authors' Fig. 1b[10], pink: additional genes we analyzed, orange: screen-results, black: negative control results (assay background). All data from Richardson *et al.* (2018)[10].

Supplementary Fig. 14

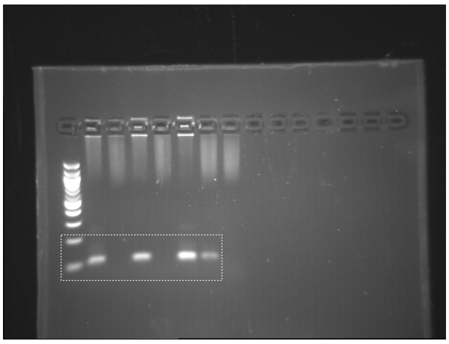

Fig. 4

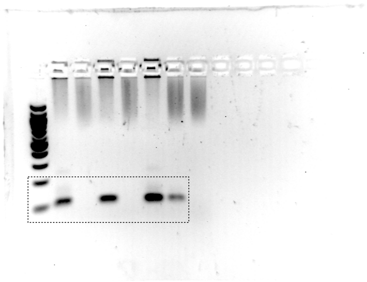

Fig. 4 (adjusted)

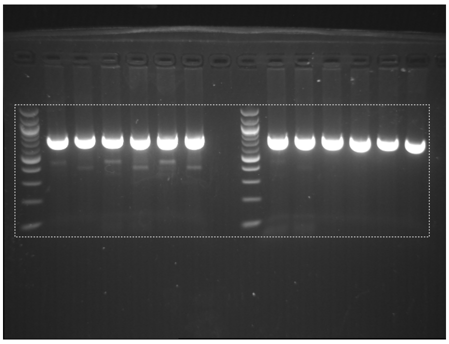

Supplementary Fig. 6a

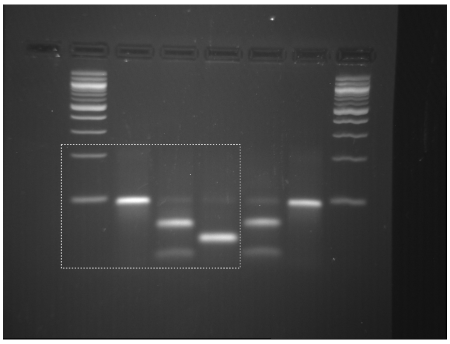

Supplementary Fig. 6c

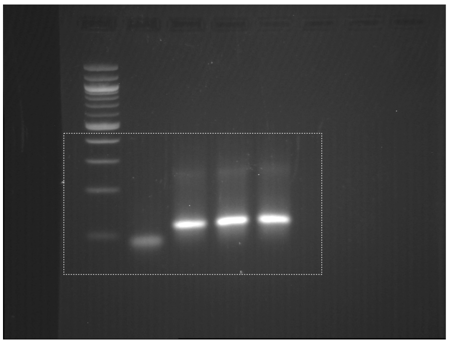

Supplementary Fig. 12b

Raw, uncropped gel images. Dotted lines indicate the cropping that was used.
