# Supplementary material for: Mechanistic and genetic basis of single-strand templated repair at Cas12a-induced DNA breaks in Chlamydomonas reinhardtii
Source: Nat Commun. 2021 Nov 19;12:6751. doi: 10.1038/s41467-021-27004-1 (PMC8604939; doi:10.1038/s41467-021-27004-1)
Supplement: Supplementary file 2 — Description of Additional Supplementary Files [file 41467_2021_27004_MOESM2_ESM.pdf]

Description of Additional Supplementary Files

File: Supplementary Data 1

Description: Description: Colony counts for all fkb12 assays (Fig. 2, Fig. 5a,d) including Shapiro-Wilk normality-test p values and the identified biological outliers with their corresponding outlier detection ranges. Plate images are in Source Data. Rapamycin^R: rapamycin-resistant.

File: Supplementary Data 2

Description: SNP values for ssODN experiments (Fig. 3d–i). The manually normalized SNP levels plotted as homology-directed repair (HDR) in Fig. 3d–i are under 'Editing (norm)'. The non-normalized SNP levels outputted by EditR, under column 'Editing (EditR)', are not used in our analyses. Column headers are explained in detail under Methods, 'EditR analysis of SNP experiments'. Source data (chromatograms used for analysis, EditR raw outputs) are in the Source Data.

File: Supplementary Data 3

Description: List of transfection conditions used to generate DNA repair mutants and screening results. N/A: not applicable, paro^R: paromomycin resistant.

File: Supplementary Data 4

Description: Proportion on rapamycin-resistant colonies detected by colony PCR (Fig. 5b, Supplementary Fig. 12b). Categories correspond to Fig. 5b and Supplementary Fig. 12b. SSTR : single-strand templated-repair, n: number of colonies analysed.

File: Supplementary Data 5

Description: Population-level scarless SSTR (Fig. 5c) obtained by multiplying rapamycin resistance (%) for each repeat in Fig. 5a (Supplementary Data 1) by the corresponding genotype's proportion of scarless SSTR level detected by colony PCR in Fig. 5b (Supplementary Data 4). Each genotype's repeat number corresponds to the repeats in Supplementary Data 1. SSTR : single-strand templated-repair.

File: Supplementary Data 6

Description: Protein-protein BLAST (blastp) using *C. reinhardtii* protein sequence *POLQ2* (Cre08.g384390.t1.1) against the NCBI *Homo sapiens* (taxid: 9606) NCBI Protein Reference Sequences (refseq_protein) database. Ascending by E value. Output taken from NCBI. Column 'Gene' and 'Synonym' were added manually using annotation from the NCBI Protein database.

File: Supplementary Data 7

Description: List of gRNAs used in this study. The (Lb)Cas12a repeat is highlighted in all gRNA sequences.

File: Supplementary Data 8

Description: List of ssODNs used in this study.

File: Supplementary Data 9

Description: PCR primers and cycling conditions used in this study. Under column 'Amplicon', tilda (~) indicates approximate amplicon sizes judged from running PCR products on agarose gels for the *aphVIII*-insertional mutants. Among the DNA repair mutants, the 5' and 3' of the *aphVIII* inserts were often amplified separately (see column 'Notes'). Mutant line numbers (#) correspond to Supplementary Figs. 8–11.

File: Supplementary Data 10

Description: List of CC numbers under which our DNA repair mutant strains (in background CC-1883) are available from the Chlamydomonas Resource Center, University of Minnesota. For genotyping PCR primers and expected amplicon sizes, see Supplementary Data 9.

File: Supplementary Data 11

Description: Primers and PCR conditions used to generate positive control *FKB12* sequences containing SNPs at positions -32, -16, 0, 16, 32 relative to the middle of the Cpf1 cut-site for EditR analysis. SNPs are highlighted.

File: Supplementary Data 12

Description: EditR p values of SNP detection. 'Guide position' refers to the sequence supplied to EditR for analysis, but with the first 10 bases omitted as this was only included for normalization purposes (AGACCGTGTTTGTGCACTACACGGGCACCCTGACCGACG-GCAAGAAGTTCGACAGCTCCCGCGAC). 'Focal base' refers to each of the four being bases called at each position. Data plotted in Fig. 3d–i and Supplementary Figure 3, 4. Source data (EditR raw outputs) are in the Source Data.

File: Supplementary Data 13

Description: EditR quality metrics including average sequencing noise, model μ and Filliben's correlation coefficient (plotted in Supplementary Figures 3, 4). Names of ssODNs correspond to Supplementary Data 8. Source data (EditR raw outputs) are in the Source Data.

File: Supplementary Data 14

Description: Gel densitometry values (column 'Band pixel density') obtained using ImageJ for the gel images in Supplementary Figure 6, which contains the digested DNA from transfected cells plotted in Fig. 3l and Supplementary Figure 6b, and the control digestion in Supplementary Figure 6c. Names of ssODNs correspond to Supplementary Data 8.

File: Supplementary Data 15

Description: Post-hoc Dunnett's test statistics from Fig. 2b. n_1 and n_2: sample sizes for each group in the comparison, SE: standard error of the mean difference, t: t-statistic, p: Dunnett's p value, *** $p<0.001$.

File: Supplementary Data 16

Description: Analysis of variance (ANOVA) and Levene's test results of data in Fig. 3d–l (both tests applied to each panel separately). Tests applied to SNP levels (i.e., levels of homology directed repair, HDR) in column 'Editing (norm)' of Supplementary Data 2. df: degrees of freedom, F: F-statistic, p: p value, * $p<0.05$

File: Supplementary Data 17

Description: Post-hoc Tukey’s honest significance difference (HSD) test results of data in Fig. 3d–I (both tests applied to each panel separately). Tests applied to SNP levels (i.e., levels of homology directed repair, HDR) in column ‘Editing (norm)’ of Supplementary Data 2. ANOVA p value added for reference from Supplementary Data 16 (see this table for full ANOVA results). Group 1 and 2: comparison groups (i.e., SNP positions), SE: standard error of the mean difference, t: t-statistic, p: Tukey’s corrected p value, * $p<0.05$.

File: Supplementary Data 18

Description: Post-hoc Games-Howell test of population-level SSTR in wild-type (wt) and DNA repair mutant lines plotted in Fig. 5c. This test was compatible with unequal sample sizes and variances across the genotypes (Levene’s test, $p=3.98\times10^{-6}$). All pairwise comparisons are performed as part of the Games-Howell test, but our analysis only pertains to comparisons against the wt control as part of our experimental design. SE: standard error of the mean difference, df: degrees of freedom, p_{Tukey}: Tukey's corrected p value for multiple comparisons; * $p < .05$.
