# Supplementary material for: Mechanistic and genetic basis of single-strand templated repair at Cas12a-induced DNA breaks in Chlamydomonas reinhardtii
Source: Nat Commun. 2021 Nov 19;12:6751. doi: 10.1038/s41467-021-27004-1 (PMC8604939; doi:10.1038/s41467-021-27004-1)
Supplement: Supplementary file 21 — Reporting Summary [file 41467_2021_27004_MOESM21_ESM.pdf]

nature research

Corresponding author(s): Attila Molnar

Last updated by author(s): Oct 13, 2021

Reporting Summary

Nature Research wishes to improve the reproducibility of the work that we publish. This form provides structure for consistency and transparency in reporting. For further information on Nature Research policies, see our Editorial Policies and the Editorial Policy Checklist.

Statistics

For all statistical analyses, confirm that the following items are present in the figure legend, table legend, main text, or Methods section.

n/a	Confirmed	
☐	☒	The exact sample size (*n*) for each experimental group/condition, given as a discrete number and unit of measurement
☐	☒	A statement on whether measurements were taken from distinct samples or whether the same sample was measured repeatedly
☐	☒	The statistical test(s) used AND whether they are one- or two-sided<br>*Only common tests should be described solely by name; describe more complex techniques in the Methods section.*
☒	☐	A description of all covariates tested
☐	☒	A description of any assumptions or corrections, such as tests of normality and adjustment for multiple comparisons
☐	☒	A full description of the statistical parameters including central tendency (e.g. means) or other basic estimates (e.g. regression coefficient) AND variation (e.g. standard deviation) or associated estimates of uncertainty (e.g. confidence intervals)
☐	☒	For null hypothesis testing, the test statistic (e.g. F, t, r) with confidence intervals, effect sizes, degrees of freedom and P value noted<br>*Give P values as exact values whenever suitable.*
☒	☐	For Bayesian analysis, information on the choice of priors and Markov chain Monte Carlo settings
☒	☐	For hierarchical and complex designs, identification of the appropriate level for tests and full reporting of outcomes
☒	☐	Estimates of effect sizes (e.g. Cohen's *d*, Pearson's *r*), indicating how they were calculated

Our web collection on statistics for biologists contains articles on many of the points above.

Software and code

Policy information about availability of computer code

Data collection	Desktop software OpenCFU (v 3.9.0) was used to count colonies. Web tool EditR (no version number) was used to detect and quantify SNPs. Desktop software ImageJ (v1.51j8) was used to semi-quantify restriction digestion reactions using gel images.
Data analysis	OpenCFU (v 3.9.0) was used to count colonies on plates. ImageJ (v1.51j8) was used to semi-quantify gels in Supplementary Fig. 6a,c. All statistics were performed in JASP (v0.14.1). EditR (v 1.0.10) was used to quantify SNPs from Sanger sequencing chromatograms, followed by manual correction using positive and negative SNP sequencing controls as described under Methods, 'EditR analysis of SNP experiments'.

For manuscripts utilizing custom algorithms or software that are central to the research but not yet described in published literature, software must be made available to editors and reviewers. We strongly encourage code deposition in a community repository (e.g. GitHub). See the Nature Research guidelines for submitting code & software for further information.

Data

Policy information about availability of data

All manuscripts must include a data availability statement. This statement should provide the following information, where applicable:

- Accession codes, unique identifiers, or web links for publicly available datasets
- A list of figures that have associated raw data
- A description of any restrictions on data availability

Colony counts underlying fkb12 assay results (Fig. 2 and Fig. 5a,d) are in Supplementary Data 1, plate images supporting these colony counts are in Source Data. EditR SNP quantification (HDR) results for sense ssODNs (Fig. 3d–f), antisense ssODNs (Fig. 3g–i) and wt sequences (Supplementary Figure 5b) are in Supplementary Data 2; p values of SNP detection (Fig. 3d–I, Supplementary Figure 5b, Supplementary Data 2) are in Supplementary Data 11; EditR quality control metrics including average noise, model μ and Filliben's coefficient (Supplementary Data 2) are in Supplementary Data 12; EditR raw output files from which all these metrics were derived and the sequencing chromatograms used both as an input for EditR and to derive Phred scores are in the Source Data. Restriction digestion values (Fig. 3l)

and the gel images which these are based on are in Supplementary Figure 6a and raw gel densitometry ImageJ values used to quantify restriction digestion are in Supplementary Data 13 including the control digestion in Supplementary Figure 6c. Colony PCR data (Fig. 5b) are in Supplementary Data 4, raw sequences and corresponding alignment categories (SSTR – scarless, SSTR – scarred, Indel) are in the Source Data. Population-level scarless SSTR data (Fig. 5c) is in Supplementary Data 5. DNA repair mutant cell lines are available from the Chlamydomonas Research Center, University of Minnesota (Supplementary Data 18). All unprocessed gel images (relating to Fig. 4b and Supplementary Figure 6a,c) are in Supplementary Figure 14.

Field-specific reporting

Please select the one below that is the best fit for your research. If you are not sure, read the appropriate sections before making your selection.

☒ Life sciences ☐ Behavioural & social sciences ☐ Ecological, evolutionary & environmental sciences

For a reference copy of the document with all sections, see nature.com/documents/nr-reporting-summary-flat.pdf

Life sciences study design

All studies must disclose on these points even when the disclosure is negative.

Sample size	No sample size calculations were performed due to our study being exploratory rather than confirming. Samples sizes are 3≤n≤6, with 3 being the minimum we deem necessary for statistics and 6 being the upper range we typically use for heightened statistical power to detect biological effects which have effect sizes we think are worth finding. These numbers are routinely used across all our studies.
Data exclusions	No data was excluded from the analysis. Biological outliers were identified, but retained in all analyses.
Replication	To confirm replication, we performed biological experimental repeats, which were mostly performed on separate dates to ensure as much independence from each other as possible. All attempts at data replication were successful.
Randomization	Blinding and randomization were not applicable to this study.
Blinding	Blinding and randomization were not applicable to this study.

Reporting for specific materials, systems and methods

We require information from authors about some types of materials, experimental systems and methods used in many studies. Here, indicate whether each material, system or method listed is relevant to your study. If you are not sure if a list item applies to your research, read the appropriate section before selecting a response.

Materials & experimental systems

n/a	Involved in the study	
☒	☐	Antibodies
☐	☒	Eukaryotic cell lines
☒	☐	Palaeontology and archaeology
☒	☐	Animals and other organisms
☒	☐	Human research participants
☒	☐	Clinical data
☒	☐	Dual use research of concern

Methods

n/a	Involved in the study	
☒	☐	ChIP-seq
☒	☐	Flow cytometry
☒	☐	MRI-based neuroimaging

Eukaryotic cell lines

Policy information about cell lines

Cell line source(s)	Chlamydomonas reinhardtii strain cc-1883 was provided by Sinead Collins (University of Edinburgh, UK) and can be purchased from the Chlamydomonas Resource Center (University of Minnesota). DNA repair mutants in this background were generated in this study.
Authentication	Authentication on cc-1883 was based on phenotypic observations when plated and in liquid culture; no further genetic authentication was performed. DNA repair mutant lines we generated were identified by colony PCR using diagnostic primers and confirmed by Sanger sequencing.
Mycoplasma contamination	N/A
Commonly misidentified lines (See ICLAC register)	N/A
