# Supplementary material for: Mechanistic and genetic basis of single-strand templated repair at Cas12a-induced DNA breaks in Chlamydomonas reinhardtii
Source: Nat Commun. 2021 Nov 19;12:6751. doi: 10.1038/s41467-021-27004-1 (PMC8604939; doi:10.1038/s41467-021-27004-1)
Supplement: Supplementary file 22 — Source Data [file 41467_2021_27004_MOESM22_ESM.zip › Source Data/EditR analysis/EditR outputs/Antisense/rep1_ssODN_antisense_0.html]

EditR v1.0.8 report


# EditR v1.0.8 report

- Data QA
  - Filtering data
  - Percent noise peak area
  - Base information
- Predicted editing
  - Editing bar plot
  - Editing table plot
  - Table of editing results
- For use in R

## Data QA

### Filtering data

What the data looked like prefiltering:

and the post filtering signal / noise plot:

### Percent noise peak area

### Base information

Here’s information about the signal of each base, the critical percent value where any higher value would be called as significant, and Filliben’s correlation for how well the noise was modelled by the zero adjusted gamma distribution.

| Base | Average percent signal | Average peak area | Critical percent value | model mu | Fillibens correlation |
| --- | --- | --- | --- | --- | --- |
| A | 94.80579 | 601.0556 | 9.691470 | 2.970871 | 0.9895007 |
| C | 93.27840 | 589.7846 | 3.795522 | 1.564479 | 0.9946026 |
| G | 93.54258 | 623.6491 | 7.888231 | 2.182578 | 0.9932952 |
| T | 94.95200 | 677.2326 | 6.306552 | 1.955345 | 0.9961924 |

## Predicted editing

### Editing bar plot

### Editing table plot

### Table of editing results


Here’s the entire guide region

| Sanger position | Guide position | Guide sequence | Sanger base call | Focal base | Focal base peak area | p value |  |
| --- | --- | --- | --- | --- | --- | --- | --- |
| 272 | 1 | A | A | A | 97.21 | 0.000000e+00 | \* |
| 272 | 1 | A | A | C | 2.05 | 1.986542e-01 |  |
| 272 | 1 | A | A | G | 0.74 | 7.651925e-01 |  |
| 272 | 1 | A | A | T | 0.00 | 9.361702e-01 |  |
| 273 | 2 | A | A | A | 96.32 | 0.000000e+00 | \* |
| 273 | 2 | A | A | C | 1.53 | 3.887508e-01 |  |
| 273 | 2 | A | A | G | 1.84 | 4.512665e-01 |  |
| 273 | 2 | A | A | T | 0.31 | 9.022967e-01 |  |
| 274 | 3 | G | G | A | 2.73 | 3.872835e-01 |  |
| 274 | 3 | G | G | C | 0.91 | 6.883559e-01 |  |
| 274 | 3 | G | G | G | 94.90 | 0.000000e+00 | \* |
| 274 | 3 | G | G | T | 1.46 | 5.299127e-01 |  |
| 275 | 4 | A | A | A | 97.34 | 0.000000e+00 | \* |
| 275 | 4 | A | A | C | 0.92 | 6.849481e-01 |  |
| 275 | 4 | A | A | G | 1.01 | 6.824592e-01 |  |
| 275 | 4 | A | A | T | 0.73 | 7.802462e-01 |  |
| 276 | 5 | C | C | A | 3.46 | 2.791873e-01 |  |
| 276 | 5 | C | C | C | 92.30 | 0.000000e+00 | \* |
| 276 | 5 | C | C | G | 2.20 | 3.696863e-01 |  |
| 276 | 5 | C | C | T | 2.04 | 3.588588e-01 |  |
| 277 | 6 | T | T | A | 3.71 | 2.482705e-01 |  |
| 277 | 6 | T | T | C | 1.15 | 5.726429e-01 |  |
| 277 | 6 | T | T | G | 2.81 | 2.601200e-01 |  |
| 277 | 6 | T | T | T | 92.33 | 0.000000e+00 | \* |
| 278 | 7 | G | G | A | 3.00 | 3.445267e-01 |  |
| 278 | 7 | G | G | C | 1.54 | 3.850380e-01 |  |
| 278 | 7 | G | G | G | 94.41 | 0.000000e+00 | \* |
| 278 | 7 | G | G | T | 1.05 | 6.687654e-01 |  |
| 279 | 8 | G | G | A | 4.30 | 1.864945e-01 |  |
| 279 | 8 | G | G | C | 1.01 | 6.413214e-01 |  |
| 279 | 8 | G | G | G | 93.55 | 0.000000e+00 | \* |
| 279 | 8 | G | G | T | 1.14 | 6.389712e-01 |  |
| 280 | 9 | C | C | A | 4.44 | 1.739056e-01 |  |
| 280 | 9 | C | C | C | 91.12 | 0.000000e+00 | \* |
| 280 | 9 | C | C | G | 2.57 | 2.997260e-01 |  |
| 280 | 9 | C | C | T | 1.87 | 4.051317e-01 |  |
| 281 | 10 | C | C | A | 2.74 | 3.854026e-01 |  |
| 281 | 10 | C | C | C | 92.59 | 0.000000e+00 | \* |
| 281 | 10 | C | C | G | 3.57 | 1.654553e-01 |  |
| 281 | 10 | C | C | T | 1.10 | 6.532251e-01 |  |
| 282 | 11 | A | A | A | 93.97 | 0.000000e+00 | \* |
| 282 | 11 | A | A | C | 2.01 | 2.095077e-01 |  |
| 282 | 11 | A | A | G | 2.93 | 2.435283e-01 |  |
| 282 | 11 | A | A | T | 1.10 | 6.534023e-01 |  |
| 283 | 12 | G | G | A | 3.61 | 2.603326e-01 |  |
| 283 | 12 | G | G | C | 1.55 | 3.820671e-01 |  |
| 283 | 12 | G | G | G | 93.30 | 0.000000e+00 | \* |
| 283 | 12 | G | G | T | 1.55 | 5.011424e-01 |  |
| 284 | 13 | A | A | A | 97.46 | 0.000000e+00 | \* |
| 284 | 13 | A | A | C | 0.82 | 7.289964e-01 |  |
| 284 | 13 | A | A | G | 0.82 | 7.427492e-01 |  |
| 284 | 13 | A | A | T | 0.91 | 7.204640e-01 |  |
| 285 | 14 | C | C | A | 3.89 | 2.273718e-01 |  |
| 285 | 14 | C | C | C | 92.81 | 0.000000e+00 | \* |
| 285 | 14 | C | C | G | 1.80 | 4.613429e-01 |  |
| 285 | 14 | C | C | T | 1.50 | 5.169627e-01 |  |
| 286 | 15 | C | C | A | 5.16 | 1.207893e-01 |  |
| 286 | 15 | C | C | C | 90.18 | 0.000000e+00 | \* |
| 286 | 15 | C | C | G | 2.50 | 3.128211e-01 |  |
| 286 | 15 | C | C | T | 2.16 | 3.297245e-01 |  |
| 287 | 16 | G | G | A | 5.47 | 1.025645e-01 |  |
| 287 | 16 | G | G | C | 1.04 | 6.296552e-01 |  |
| 287 | 16 | G | G | G | 91.86 | 0.000000e+00 | \* |
| 287 | 16 | G | G | T | 1.63 | 4.758653e-01 |  |
| 288 | 17 | T | T | A | 0.00 | 8.611111e-01 |  |
| 288 | 17 | T | T | C | 1.62 | 3.518035e-01 |  |
| 288 | 17 | T | T | G | 2.83 | 2.578384e-01 |  |
| 288 | 17 | T | T | T | 95.56 | 0.000000e+00 | \* |
| 289 | 18 | G | G | A | 3.94 | 2.219433e-01 |  |
| 289 | 18 | G | G | C | 0.00 | 8.604651e-01 |  |
| 289 | 18 | G | G | G | 95.48 | 0.000000e+00 | \* |
| 289 | 18 | G | G | T | 0.58 | 8.312403e-01 |  |
| 290 | 19 | T | T | A | 1.63 | 5.998866e-01 |  |
| 290 | 19 | T | T | C | 0.81 | 7.299663e-01 |  |
| 290 | 19 | T | T | G | 2.28 | 3.536724e-01 |  |
| 290 | 19 | T | T | T | 95.28 | 0.000000e+00 | \* |
| 291 | 20 | T | T | A | 1.73 | 5.782336e-01 |  |
| 291 | 20 | T | T | C | 1.59 | 3.641524e-01 |  |
| 291 | 20 | T | T | G | 2.89 | 2.492147e-01 |  |
| 291 | 20 | T | T | T | 93.80 | 0.000000e+00 | \* |
| 292 | 21 | T | T | A | 0.14 | 8.571430e-01 |  |
| 292 | 21 | T | T | C | 1.54 | 3.836724e-01 |  |
| 292 | 21 | T | T | G | 3.23 | 2.034923e-01 |  |
| 292 | 21 | T | T | T | 95.09 | 0.000000e+00 | \* |
| 293 | 22 | G | G | A | 2.55 | 4.195467e-01 |  |
| 293 | 22 | G | G | C | 0.00 | 8.604651e-01 |  |
| 293 | 22 | G | G | G | 96.50 | 0.000000e+00 | \* |
| 293 | 22 | G | G | T | 0.95 | 7.038809e-01 |  |
| 294 | 23 | T | T | A | 0.00 | 8.611111e-01 |  |
| 294 | 23 | T | T | C | 1.93 | 2.334895e-01 |  |
| 294 | 23 | T | T | G | 1.76 | 4.710038e-01 |  |
| 294 | 23 | T | T | T | 96.31 | 0.000000e+00 | \* |
| 295 | 24 | G | G | A | 2.85 | 3.685468e-01 |  |
| 295 | 24 | G | G | C | 1.16 | 5.659489e-01 |  |
| 295 | 24 | G | G | G | 94.83 | 0.000000e+00 | \* |
| 295 | 24 | G | G | T | 1.16 | 6.296593e-01 |  |
| 296 | 25 | C | C | A | 1.74 | 5.766583e-01 |  |
| 296 | 25 | C | C | C | 94.43 | 0.000000e+00 | \* |
| 296 | 25 | C | C | G | 2.09 | 3.939468e-01 |  |
| 296 | 25 | C | C | T | 1.74 | 4.422010e-01 |  |
| 297 | 26 | A | A | A | 93.05 | 0.000000e+00 | \* |
| 297 | 26 | A | A | C | 3.68 | 1.243885e-02 |  |
| 297 | 26 | A | A | G | 2.04 | 4.031711e-01 |  |
| 297 | 26 | A | A | T | 1.23 | 6.077651e-01 |  |
| 298 | 27 | C | C | A | 1.46 | 6.347869e-01 |  |
| 298 | 27 | C | C | C | 96.05 | 0.000000e+00 | \* |
| 298 | 27 | C | C | G | 0.29 | 8.961100e-01 |  |
| 298 | 27 | C | C | T | 2.20 | 3.219537e-01 |  |
| 299 | 28 | T | T | A | 1.99 | 5.257043e-01 |  |
| 299 | 28 | T | T | C | 1.99 | 2.167654e-01 |  |
| 299 | 28 | T | T | G | 0.26 | 9.028210e-01 |  |
| 299 | 28 | T | T | T | 95.76 | 0.000000e+00 | \* |
| 300 | 29 | A | A | A | 95.42 | 0.000000e+00 | \* |
| 300 | 29 | A | A | C | 1.74 | 3.025729e-01 |  |
| 300 | 29 | A | A | G | 0.95 | 7.017666e-01 |  |
| 300 | 29 | A | A | T | 1.90 | 3.978330e-01 |  |
| 301 | 30 | C | C | A | 1.99 | 5.254999e-01 |  |
| 301 | 30 | C | C | C | 93.88 | 0.000000e+00 | \* |
| 301 | 30 | C | C | G | 2.14 | 3.823890e-01 |  |
| 301 | 30 | C | C | T | 1.99 | 3.732940e-01 |  |
| 302 | 31 | A | A | A | 92.80 | 0.000000e+00 | \* |
| 302 | 31 | A | A | C | 2.67 | 7.514085e-02 |  |
| 302 | 31 | A | A | G | 4.53 | 9.090392e-02 |  |
| 302 | 31 | A | A | T | 0.00 | 9.361702e-01 |  |
| 303 | 32 | C | C | A | 1.37 | 6.553151e-01 |  |
| 303 | 32 | C | C | C | 93.50 | 0.000000e+00 | \* |
| 303 | 32 | C | C | G | 2.56 | 3.007647e-01 |  |
| 303 | 32 | C | C | T | 2.56 | 2.452100e-01 |  |
| 304 | 33 | G | G | A | 3.51 | 2.723259e-01 |  |
| 304 | 33 | G | G | C | 1.65 | 3.364466e-01 |  |
| 304 | 33 | G | G | G | 93.60 | 0.000000e+00 | \* |
| 304 | 33 | G | G | T | 1.24 | 6.033673e-01 |  |
| 305 | 34 | G | G | A | 2.13 | 4.970434e-01 |  |
| 305 | 34 | G | G | C | 0.85 | 7.140784e-01 |  |
| 305 | 34 | G | G | G | 96.88 | 0.000000e+00 | \* |
| 305 | 34 | G | G | T | 0.14 | 9.284835e-01 |  |
| 306 | 35 | G | G | A | 3.32 | 2.983377e-01 |  |
| 306 | 35 | G | G | C | 1.22 | 5.372798e-01 |  |
| 306 | 35 | G | G | G | 94.42 | 0.000000e+00 | \* |
| 306 | 35 | G | G | T | 1.05 | 6.710206e-01 |  |
| 307 | 36 | C | C | A | 2.94 | 3.534049e-01 |  |
| 307 | 36 | C | C | C | 94.54 | 0.000000e+00 | \* |
| 307 | 36 | C | C | G | 1.47 | 5.466600e-01 |  |
| 307 | 36 | C | C | T | 1.05 | 6.698513e-01 |  |
| 308 | 37 | A | A | A | 95.46 | 0.000000e+00 | \* |
| 308 | 37 | A | A | C | 2.65 | 7.875726e-02 |  |
| 308 | 37 | A | A | G | 0.57 | 8.198367e-01 |  |
| 308 | 37 | A | A | T | 1.32 | 5.746572e-01 |  |
| 309 | 38 | C | C | A | 1.50 | 6.265815e-01 |  |
| 309 | 38 | C | C | C | 95.63 | 0.000000e+00 | \* |
| 309 | 38 | C | C | G | 1.23 | 6.158905e-01 |  |
| 309 | 38 | C | C | T | 1.64 | 4.721417e-01 |  |
| 310 | 39 | C | C | A | 4.22 | 1.941374e-01 |  |
| 310 | 39 | C | C | C | 92.92 | 0.000000e+00 | \* |
| 310 | 39 | C | C | G | 0.45 | 8.536643e-01 |  |
| 310 | 39 | C | C | T | 2.41 | 2.753434e-01 |  |
| 311 | 40 | C | C | A | 3.28 | 3.038361e-01 |  |
| 311 | 40 | C | C | C | 92.82 | 0.000000e+00 | \* |
| 311 | 40 | C | C | G | 1.56 | 5.222349e-01 |  |
| 311 | 40 | C | C | T | 2.34 | 2.898826e-01 |  |
| 312 | 41 | T | T | A | 0.00 | 8.611111e-01 |  |
| 312 | 41 | T | T | C | 1.29 | 5.026566e-01 |  |
| 312 | 41 | T | T | G | 2.87 | 2.516626e-01 |  |
| 312 | 41 | T | T | T | 95.84 | 0.000000e+00 | \* |
| 313 | 42 | G | G | A | 4.78 | 1.467061e-01 |  |
| 313 | 42 | G | G | C | 1.00 | 6.467795e-01 |  |
| 313 | 42 | G | G | G | 93.00 | 0.000000e+00 | \* |
| 313 | 42 | G | G | T | 1.22 | 6.094233e-01 |  |
| 314 | 43 | A | A | A | 83.20 | 0.000000e+00 | \* |
| 314 | 43 | A | A | C | 1.32 | 4.871343e-01 |  |
| 314 | 43 | A | A | G | 2.91 | 2.456981e-01 |  |
| 314 | 43 | A | A | T | 12.57 | 2.505715e-05 | \* |
| 315 | 44 | C | C | A | 2.71 | 3.908000e-01 |  |
| 315 | 44 | C | C | C | 92.82 | 0.000000e+00 | \* |
| 315 | 44 | C | C | G | 3.35 | 1.888657e-01 |  |
| 315 | 44 | C | C | T | 1.12 | 6.465038e-01 |  |
| 316 | 45 | C | C | A | 6.80 | 5.055303e-02 |  |
| 316 | 45 | C | C | C | 87.73 | 0.000000e+00 | \* |
| 316 | 45 | C | C | G | 4.15 | 1.155573e-01 |  |
| 316 | 45 | C | C | T | 1.33 | 5.734845e-01 |  |
| 317 | 46 | G | G | A | 5.84 | 8.468109e-02 |  |
| 317 | 46 | G | G | C | 1.69 | 3.193877e-01 |  |
| 317 | 46 | G | G | G | 91.71 | 0.000000e+00 | \* |
| 317 | 46 | G | G | T | 0.75 | 7.739302e-01 |  |
| 318 | 47 | A | A | A | 97.41 | 0.000000e+00 | \* |
| 318 | 47 | A | A | C | 0.97 | 6.606055e-01 |  |
| 318 | 47 | A | A | G | 0.86 | 7.282604e-01 |  |
| 318 | 47 | A | A | T | 0.76 | 7.733095e-01 |  |
| 319 | 48 | C | C | A | 3.64 | 2.565168e-01 |  |
| 319 | 48 | C | C | C | 92.20 | 0.000000e+00 | \* |
| 319 | 48 | C | C | G | 2.25 | 3.591079e-01 |  |
| 319 | 48 | C | C | T | 1.91 | 3.949272e-01 |  |
| 320 | 49 | G | G | A | 5.90 | 8.208778e-02 |  |
| 320 | 49 | G | G | C | 1.97 | 2.232611e-01 |  |
| 320 | 49 | G | G | G | 91.40 | 0.000000e+00 | \* |
| 320 | 49 | G | G | T | 0.74 | 7.794096e-01 |  |
| 321 | 50 | G | G | A | 3.01 | 3.429589e-01 |  |
| 321 | 50 | G | G | C | 1.15 | 5.750207e-01 |  |
| 321 | 50 | G | G | G | 95.42 | 0.000000e+00 | \* |
| 321 | 50 | G | G | T | 0.43 | 8.732565e-01 |  |
| 322 | 51 | C | C | A | 3.40 | 2.865486e-01 |  |
| 322 | 51 | C | C | C | 92.93 | 0.000000e+00 | \* |
| 322 | 51 | C | C | G | 2.36 | 3.388144e-01 |  |
| 322 | 51 | C | C | T | 1.31 | 5.795481e-01 |  |
| 323 | 52 | A | A | A | 97.49 | 0.000000e+00 | \* |
| 323 | 52 | A | A | C | 1.25 | 5.211820e-01 |  |
| 323 | 52 | A | A | G | 1.25 | 6.086722e-01 |  |
| 323 | 52 | A | A | T | 0.00 | 9.361702e-01 |  |
| 324 | 53 | A | A | A | 96.01 | 0.000000e+00 | \* |
| 324 | 53 | A | A | C | 0.87 | 7.072749e-01 |  |
| 324 | 53 | A | A | G | 2.95 | 2.397575e-01 |  |
| 324 | 53 | A | A | T | 0.17 | 9.247578e-01 |  |
| 325 | 54 | G | G | A | 2.94 | 3.543721e-01 |  |
| 325 | 54 | G | G | C | 0.00 | 8.604651e-01 |  |
| 325 | 54 | G | G | G | 96.44 | 0.000000e+00 | \* |
| 325 | 54 | G | G | T | 0.63 | 8.149619e-01 |  |
| 326 | 55 | A | A | A | 97.80 | 0.000000e+00 | \* |
| 326 | 55 | A | A | C | 0.00 | 8.604651e-01 |  |
| 326 | 55 | A | A | G | 1.73 | 4.787060e-01 |  |
| 326 | 55 | A | A | T | 0.47 | 8.622012e-01 |  |
| 327 | 56 | A | A | A | 95.46 | 0.000000e+00 | \* |
| 327 | 56 | A | A | C | 0.73 | 7.640645e-01 |  |
| 327 | 56 | A | A | G | 2.00 | 4.140722e-01 |  |
| 327 | 56 | A | A | T | 1.81 | 4.203326e-01 |  |
| 328 | 57 | G | G | A | 1.75 | 5.752981e-01 |  |
| 328 | 57 | G | G | C | 0.25 | 8.574633e-01 |  |
| 328 | 57 | G | G | G | 94.26 | 0.000000e+00 | \* |
| 328 | 57 | G | G | T | 3.74 | 9.575102e-02 |  |
| 329 | 58 | T | T | A | 0.38 | 8.363757e-01 |  |
| 329 | 58 | T | T | C | 2.26 | 1.463917e-01 |  |
| 329 | 58 | T | T | G | 0.75 | 7.630089e-01 |  |
| 329 | 58 | T | T | T | 96.62 | 0.000000e+00 | \* |
| 330 | 59 | T | T | A | 1.40 | 6.492339e-01 |  |
| 330 | 59 | T | T | C | 2.44 | 1.094314e-01 |  |
| 330 | 59 | T | T | G | 1.05 | 6.710454e-01 |  |
| 330 | 59 | T | T | T | 95.11 | 0.000000e+00 | \* |
| 331 | 60 | C | C | A | 3.54 | 2.688484e-01 |  |
| 331 | 60 | C | C | C | 90.80 | 0.000000e+00 | \* |
| 331 | 60 | C | C | G | 4.42 | 9.696404e-02 |  |
| 331 | 60 | C | C | T | 1.24 | 6.036207e-01 |  |
| 332 | 61 | G | G | A | 7.73 | 3.038142e-02 |  |
| 332 | 61 | G | G | C | 1.72 | 3.107488e-01 |  |
| 332 | 61 | G | G | G | 89.91 | 0.000000e+00 | \* |
| 332 | 61 | G | G | T | 0.64 | 8.102070e-01 |  |
| 333 | 62 | A | A | A | 96.73 | 0.000000e+00 | \* |
| 333 | 62 | A | A | C | 1.31 | 4.947872e-01 |  |
| 333 | 62 | A | A | G | 1.96 | 4.221975e-01 |  |
| 333 | 62 | A | A | T | 0.00 | 9.361702e-01 |  |
| 334 | 63 | C | C | A | 3.06 | 3.344746e-01 |  |
| 334 | 63 | C | C | C | 94.19 | 0.000000e+00 | \* |
| 334 | 63 | C | C | G | 1.13 | 6.460295e-01 |  |
| 334 | 63 | C | C | T | 1.61 | 4.802849e-01 |  |
| 335 | 64 | A | A | A | 91.73 | 0.000000e+00 | \* |
| 335 | 64 | A | A | C | 2.19 | 1.616181e-01 |  |
| 335 | 64 | A | A | G | 6.08 | 3.325937e-02 |  |
| 335 | 64 | A | A | T | 0.00 | 9.361702e-01 |  |
| 336 | 65 | G | G | A | 1.73 | 5.777099e-01 |  |
| 336 | 65 | G | G | C | 2.46 | 1.071345e-01 |  |
| 336 | 65 | G | G | G | 94.36 | 0.000000e+00 | \* |
| 336 | 65 | G | G | T | 1.45 | 5.338849e-01 |  |
| 337 | 66 | C | C | A | 1.94 | 5.348241e-01 |  |
| 337 | 66 | C | C | C | 91.07 | 0.000000e+00 | \* |
| 337 | 66 | C | C | G | 1.94 | 4.265952e-01 |  |
| 337 | 66 | C | C | T | 5.05 | 3.105000e-02 |  |
| 338 | 67 | T | T | A | 0.00 | 8.611111e-01 |  |
| 338 | 67 | T | T | C | 1.78 | 2.871756e-01 |  |
| 338 | 67 | T | T | G | 1.78 | 4.657958e-01 |  |
| 338 | 67 | T | T | T | 96.44 | 0.000000e+00 | \* |
| 339 | 68 | C | C | A | 2.39 | 4.470216e-01 |  |
| 339 | 68 | C | C | C | 95.04 | 0.000000e+00 | \* |
| 339 | 68 | C | C | G | 0.51 | 8.359916e-01 |  |
| 339 | 68 | C | C | T | 2.05 | 3.570280e-01 |  |
| 340 | 69 | C | C | A | 1.92 | 5.391658e-01 |  |
| 340 | 69 | C | C | C | 94.38 | 0.000000e+00 | \* |
| 340 | 69 | C | C | G | 0.55 | 8.253543e-01 |  |
| 340 | 69 | C | C | T | 3.16 | 1.546227e-01 |  |
| 341 | 70 | C | C | A | 2.64 | 4.025678e-01 |  |
| 341 | 70 | C | C | C | 90.65 | 0.000000e+00 | \* |
| 341 | 70 | C | C | G | 4.27 | 1.070167e-01 |  |
| 341 | 70 | C | C | T | 2.44 | 2.693839e-01 |  |
| 342 | 71 | G | G | A | 5.43 | 1.047690e-01 |  |
| 342 | 71 | G | G | C | 3.02 | 4.180293e-02 |  |
| 342 | 71 | G | G | G | 90.95 | 0.000000e+00 | \* |
| 342 | 71 | G | G | T | 0.60 | 8.229607e-01 |  |
| 343 | 72 | C | C | A | 3.70 | 2.496831e-01 |  |
| 343 | 72 | C | C | C | 90.66 | 0.000000e+00 | \* |
| 343 | 72 | C | C | G | 3.11 | 2.177718e-01 |  |
| 343 | 72 | C | C | T | 2.53 | 2.517673e-01 |  |
| 344 | 73 | G | G | A | 7.50 | 3.442224e-02 |  |
| 344 | 73 | G | G | C | 3.33 | 2.376419e-02 |  |
| 344 | 73 | G | G | G | 88.33 | 0.000000e+00 | \* |
| 344 | 73 | G | G | T | 0.83 | 7.464132e-01 |  |
| 345 | 74 | A | A | A | 94.64 | 0.000000e+00 | \* |
| 345 | 74 | A | A | C | 3.12 | 3.460648e-02 |  |
| 345 | 74 | A | A | G | 1.04 | 6.727231e-01 |  |
| 345 | 74 | A | A | T | 1.19 | 6.204904e-01 |  |
| 346 | 75 | C | C | A | 2.59 | 4.113835e-01 |  |
| 346 | 75 | C | C | C | 92.23 | 0.000000e+00 | \* |
| 346 | 75 | C | C | G | 1.52 | 5.318853e-01 |  |
| 346 | 75 | C | C | T | 3.66 | 1.025204e-01 |  |

## For use in R

If you want to work with the results in R, here is output that you can copy and paste in your terminal to get:

The base information:

```
structure(list(focal.base = c("A", "C", "G", "T"), avg.percsignal = c(94.8057903743008, 
93.2784027423945, 93.5425778446083, 94.9519964713811), avg.areasignal = c(601.055555555556, 
589.784615384615, 623.649122807018, 677.232558139535), crit.perc.area = c(9.69146964953835, 
3.79552189360765, 7.88823102101582, 6.30655183676762), mu = c(2.97087138939094, 
1.56447897512198, 2.18257756661812, 1.95534473564703), fillibens = c(0.98950070751286, 
0.994602561397613, 0.993295187710658, 0.996192425443615)), .Names = c("focal.base", 
"avg.percsignal", "avg.areasignal", "crit.perc.area", "mu", "fillibens"
), row.names = c(NA, -4L), class = "data.frame")
```

the data.frame that contains information on the guide region:

```
structure(list(A.area = c(522, 629, 15, 1060, 22, 29, 37, 34, 
19, 20, 514, 21, 1074, 26, 31, 37, 0, 41, 10, 12, 1, 24, 0, 22, 
10, 455, 10, 15, 604, 13, 451, 8, 17, 15, 19, 14, 505, 11, 28, 
21, 0, 43, 629, 17, 41, 31, 903, 21, 24, 21, 13, 311, 553, 14, 
623, 526, 7, 2, 8, 20, 36, 740, 19, 377, 12, 10, 0, 14, 14, 13, 
27, 19, 36, 636, 17), C.area = c(11, 10, 5, 10, 587, 9, 19, 8, 
390, 675, 11, 9, 9, 620, 542, 7, 8, 0, 5, 11, 11, 0, 11, 9, 543, 
18, 656, 15, 11, 614, 13, 547, 8, 6, 7, 450, 14, 700, 617, 595, 
9, 9, 10, 582, 529, 9, 9, 532, 8, 8, 355, 4, 5, 0, 0, 4, 1, 12, 
14, 513, 8, 10, 584, 9, 17, 469, 13, 556, 688, 446, 15, 466, 
16, 21, 605), G.area = c(4, 12, 521, 11, 14, 22, 1165, 740, 11, 
26, 16, 543, 9, 12, 15, 621, 14, 993, 14, 20, 23, 910, 10, 733, 
12, 10, 2, 2, 6, 14, 22, 15, 453, 682, 541, 7, 3, 9, 3, 10, 20, 
837, 22, 21, 25, 487, 8, 13, 372, 666, 9, 4, 17, 460, 11, 11, 
378, 4, 6, 25, 419, 15, 7, 25, 653, 10, 13, 3, 4, 21, 452, 16, 
424, 7, 10), T.area = c(0, 2, 8, 8, 13, 722, 13, 9, 8, 8, 6, 
9, 10, 10, 13, 11, 473, 6, 585, 650, 678, 9, 548, 9, 10, 6, 15, 
723, 12, 13, 0, 15, 6, 1, 6, 5, 7, 12, 16, 15, 668, 11, 95, 7, 
8, 4, 7, 11, 3, 3, 5, 0, 1, 3, 3, 10, 15, 514, 545, 7, 3, 0, 
10, 0, 10, 26, 705, 12, 23, 12, 3, 13, 4, 8, 24), Tot.area = c(537, 
653, 549, 1089, 636, 782, 1234, 791, 428, 729, 547, 582, 1102, 
668, 601, 676, 495, 1040, 614, 693, 713, 943, 569, 773, 575, 
489, 683, 755, 633, 654, 486, 585, 484, 704, 573, 476, 529, 732, 
664, 641, 697, 900, 756, 627, 603, 531, 927, 577, 407, 698, 382, 
319, 576, 477, 637, 551, 401, 532, 573, 565, 466, 765, 620, 411, 
692, 515, 731, 585, 729, 492, 497, 514, 480, 672, 656), A.perc = c(97.2067039106145, 
96.3246554364472, 2.73224043715847, 97.3370064279155, 3.45911949685535, 
3.70843989769821, 2.99837925445705, 4.29835651074589, 4.4392523364486, 
2.74348422496571, 93.9670932358318, 3.60824742268041, 97.459165154265, 
3.89221556886228, 5.15806988352745, 5.47337278106509, 0, 3.94230769230769, 
1.62866449511401, 1.73160173160173, 0.140252454417952, 2.54506892895016, 
0, 2.84605433376455, 1.73913043478261, 93.0470347648262, 1.46412884333821, 
1.98675496688742, 95.4186413902054, 1.98776758409786, 92.798353909465, 
1.36752136752137, 3.51239669421488, 2.13068181818182, 3.31588132635253, 
2.94117647058824, 95.4631379962193, 1.50273224043716, 4.21686746987952, 
3.27613104524181, 0, 4.77777777777778, 83.2010582010582, 2.71132376395534, 
6.79933665008292, 5.83804143126177, 97.411003236246, 3.63951473136915, 
5.8968058968059, 3.00859598853868, 3.40314136125654, 97.4921630094044, 
96.0069444444444, 2.93501048218029, 97.8021978021978, 95.4627949183303, 
1.74563591022444, 0.37593984962406, 1.39616055846422, 3.53982300884956, 
7.72532188841202, 96.7320261437908, 3.06451612903226, 91.7274939172749, 
1.73410404624277, 1.94174757281553, 0, 2.39316239316239, 1.92043895747599, 
2.64227642276423, 5.43259557344064, 3.69649805447471, 7.5, 94.6428571428571, 
2.59146341463415), C.perc = c(2.04841713221601, 1.53139356814701, 
0.910746812386157, 0.918273645546373, 92.2955974842767, 1.15089514066496, 
1.53970826580227, 1.01137800252844, 91.1214953271028, 92.5925925925926, 
2.0109689213894, 1.54639175257732, 0.816696914700545, 92.814371257485, 
90.1830282861897, 1.03550295857988, 1.61616161616162, 0, 0.814332247557003, 
1.58730158730159, 1.54277699859748, 0, 1.93321616871705, 1.16429495472186, 
94.4347826086957, 3.68098159509202, 96.0468521229868, 1.98675496688742, 
1.73775671406003, 93.8837920489297, 2.67489711934156, 93.5042735042735, 
1.65289256198347, 0.852272727272727, 1.2216404886562, 94.5378151260504, 
2.64650283553875, 95.6284153005465, 92.921686746988, 92.8237129485179, 
1.29124820659971, 1, 1.32275132275132, 92.822966507177, 87.7280265339967, 
1.69491525423729, 0.970873786407767, 92.2010398613518, 1.96560196560197, 
1.14613180515759, 92.9319371727749, 1.25391849529781, 0.868055555555556, 
0, 0, 0.725952813067151, 0.249376558603491, 2.25563909774436, 
2.44328097731239, 90.7964601769911, 1.71673819742489, 1.30718954248366, 
94.1935483870968, 2.18978102189781, 2.45664739884393, 91.0679611650485, 
1.77838577291382, 95.042735042735, 94.3758573388203, 90.650406504065, 
3.01810865191147, 90.6614785992218, 3.33333333333333, 3.125, 
92.2256097560976), G.perc = c(0.74487895716946, 1.83767228177642, 
94.8998178506375, 1.01010101010101, 2.20125786163522, 2.81329923273657, 
94.4084278768233, 93.5524652338812, 2.57009345794393, 3.56652949245542, 
2.92504570383912, 93.298969072165, 0.816696914700545, 1.79640718562874, 
2.49584026622296, 91.8639053254438, 2.82828282828283, 95.4807692307692, 
2.28013029315961, 2.88600288600289, 3.2258064516129, 96.5005302226935, 
1.75746924428822, 94.8253557567917, 2.08695652173913, 2.04498977505112, 
0.292825768667643, 0.264900662251656, 0.947867298578199, 2.14067278287462, 
4.52674897119342, 2.56410256410256, 93.595041322314, 96.875, 
94.4153577661431, 1.47058823529412, 0.56710775047259, 1.22950819672131, 
0.451807228915663, 1.5600624024961, 2.86944045911047, 93, 2.91005291005291, 
3.34928229665072, 4.14593698175788, 91.713747645951, 0.862998921251348, 
2.25303292894281, 91.4004914004914, 95.4154727793696, 2.35602094240838, 
1.25391849529781, 2.95138888888889, 96.4360587002096, 1.72684458398744, 
1.99637023593466, 94.2643391521197, 0.75187969924812, 1.04712041884817, 
4.42477876106195, 89.9141630901288, 1.96078431372549, 1.12903225806452, 
6.08272506082725, 94.364161849711, 1.94174757281553, 1.77838577291382, 
0.512820512820513, 0.548696844993141, 4.26829268292683, 90.9456740442656, 
3.11284046692607, 88.3333333333333, 1.04166666666667, 1.52439024390244
), T.perc = c(0, 0.306278713629403, 1.45719489981785, 0.734618916437098, 
2.0440251572327, 92.3273657289003, 1.05348460291734, 1.1378002528445, 
1.86915887850467, 1.09739368998628, 1.09689213893967, 1.54639175257732, 
0.907441016333938, 1.49700598802395, 2.1630615640599, 1.62721893491124, 
95.5555555555556, 0.576923076923077, 95.2768729641694, 93.7950937950938, 
95.0911640953717, 0.95440084835631, 96.3093145869947, 1.16429495472186, 
1.73913043478261, 1.22699386503067, 2.19619326500732, 95.7615894039735, 
1.8957345971564, 1.98776758409786, 0, 2.56410256410256, 1.2396694214876, 
0.142045454545455, 1.04712041884817, 1.05042016806723, 1.32325141776938, 
1.63934426229508, 2.40963855421687, 2.34009360374415, 95.8393113342898, 
1.22222222222222, 12.5661375661376, 1.11642743221691, 1.32669983416252, 
0.753295668549906, 0.75512405609493, 1.90641247833622, 0.737100737100737, 
0.429799426934097, 1.30890052356021, 0, 0.173611111111111, 0.628930817610063, 
0.470957613814757, 1.81488203266788, 3.74064837905237, 96.6165413533835, 
95.1134380453752, 1.23893805309735, 0.643776824034335, 0, 1.61290322580645, 
0, 1.44508670520231, 5.04854368932039, 96.4432284541724, 2.05128205128205, 
3.15500685871056, 2.4390243902439, 0.603621730382294, 2.52918287937743, 
0.833333333333333, 1.19047619047619, 3.65853658536585), base.call = c("A", 
"A", "G", "A", "C", "T", "G", "G", "C", "C", "A", "G", "A", "C", 
"C", "G", "T", "G", "T", "T", "T", "G", "T", "G", "C", "A", "C", 
"T", "A", "C", "A", "C", "G", "G", "G", "C", "A", "C", "C", "C", 
"T", "G", "A", "C", "C", "G", "A", "C", "G", "G", "C", "A", "A", 
"G", "A", "A", "G", "T", "T", "C", "G", "A", "C", "A", "G", "C", 
"T", "C", "C", "C", "G", "C", "G", "A", "C"), index = 272:346, 
    guide.seq = c("A", "A", "G", "A", "C", "T", "G", "G", "C", 
    "C", "A", "G", "A", "C", "C", "G", "T", "G", "T", "T", "T", 
    "G", "T", "G", "C", "A", "C", "T", "A", "C", "A", "C", "G", 
    "G", "G", "C", "A", "C", "C", "C", "T", "G", "A", "C", "C", 
    "G", "A", "C", "G", "G", "C", "A", "A", "G", "A", "A", "G", 
    "T", "T", "C", "G", "A", "C", "A", "G", "C", "T", "C", "C", 
    "C", "G", "C", "G", "A", "C"), T.pval = c(0.936170212765957, 
    0.902296716040191, 0.529912680669291, 0.780246191901842, 
    0.358858821095162, 0, 0.668765382711982, 0.638971228400734, 
    0.405131736217181, 0.653225050509047, 0.653402306975221, 
    0.501142434637233, 0.720463957433423, 0.516962708723675, 
    0.329724525477053, 0.475865319274885, 0, 0.831240255416568, 
    0, 0, 0, 0.703880939231884, 0, 0.629659335478689, 0.442201042343931, 
    0.607765083727343, 0.321953652940933, 0, 0.397832957702098, 
    0.373293985974302, 0.936170212765957, 0.245210017595186, 
    0.603367264234854, 0.928483466311794, 0.67102063605692, 0.66985125276684, 
    0.574657176985623, 0.472141678911971, 0.275343435313156, 
    0.289882629115785, 0, 0.609423294031712, 2.50571481348327e-05, 
    0.64650377280693, 0.573484487991152, 0.77393019619057, 0.77330946037514, 
    0.394927167920646, 0.779409574493963, 0.873256490904596, 
    0.579548086069621, 0.936170212765957, 0.924757768558585, 
    0.814961945177612, 0.862201186009471, 0.42033262567721, 0.0957510186984016, 
    0, 0, 0.603620728057522, 0.810207025540255, 0.936170212765957, 
    0.480284907667934, 0.936170212765957, 0.533884857673086, 
    0.031049997785228, 0, 0.357027979873582, 0.154622710793132, 
    0.269383861117367, 0.822960670133221, 0.251767312539453, 
    0.7464132049875, 0.620490429603347, 0.102520352902471), C.pval = c(0.198654204198465, 
    0.38875083059426, 0.688355889818715, 0.684948063544333, 0, 
    0.572642903806898, 0.385037995901648, 0.641321408083502, 
    0, 0, 0.209507745962436, 0.382067092408815, 0.728996381773044, 
    0, 0, 0.62965518803553, 0.351803464888178, 0.860465116279069, 
    0.729966345381376, 0.36415237786408, 0.383672394654904, 0.860465116279069, 
    0.233489507439636, 0.565948946274664, 0, 0.0124388510767075, 
    0, 0.216765358989371, 0.302572897343664, 0, 0.0751408544452401, 
    0, 0.336446580780793, 0.714078372593418, 0.537279827901416, 
    0, 0.0787572600787649, 0, 0, 0, 0.502656617281006, 0.646779528401367, 
    0.48713427228412, 0, 0, 0.31938766253486, 0.660605498716589, 
    0, 0.223261103690509, 0.575020738224175, 0, 0.521181987229486, 
    0.707274854983005, 0.860465116279069, 0.860465116279069, 
    0.764064509247196, 0.857463343784074, 0.146391656058138, 
    0.109431365231634, 0, 0.310748803998777, 0.49478717782544, 
    0, 0.161618124382713, 0.107134541345956, 0, 0.287175554680228, 
    0, 0, 0, 0.0418029345566659, 0, 0.0237641880303248, 0.0346064795740906, 
    0), G.pval = c(0.765192525703107, 0.451266531993335, 0, 0.682459190718791, 
    0.36968629390231, 0.260120005469017, 0, 0, 0.299726008694834, 
    0.165455324481677, 0.243528323040226, 0, 0.742749174095015, 
    0.461342895291484, 0.312821117528177, 0, 0.257838424661913, 
    0, 0.353672396193025, 0.249214715078871, 0.203492283842345, 
    0, 0.471003803969705, 0, 0.393946787783939, 0.403171052264954, 
    0.896110047888171, 0.902820968577439, 0.701766554981075, 
    0.382389001153362, 0.0909039202669422, 0.30076467546071, 
    0, 0, 0, 0.546659989836392, 0.819836652290616, 0.615890514219098, 
    0.853664347854228, 0.522234925642701, 0.251662554488824, 
    0, 0.245698082092912, 0.188865658062112, 0.115557254998239, 
    0, 0.72826035347662, 0.359107882668002, 0, 0, 0.338814358651915, 
    0.608672234121144, 0.239757453769253, 0, 0.478705954621642, 
    0.414072174047711, 0, 0.763008939032321, 0.671045412925769, 
    0.0969640378756017, 0, 0.422197537292047, 0.646029496805738, 
    0.0332593662305997, 0, 0.426595182592326, 0.465795783252276, 
    0.835991575426938, 0.825354265107939, 0.10701673796716, 0, 
    0.217771809052064, 0, 0.672723088564108, 0.531885309409726
    ), A.pval = c(0, 0, 0.387283531874924, 0, 0.279187329656785, 
    0.248270517212592, 0.344526666441797, 0.18649450818046, 0.173905608936282, 
    0.385402599472961, 0, 0.260332614195818, 0, 0.227371800938053, 
    0.120789316046946, 0.102564490168382, 0.86111111111111, 0.221943283292237, 
    0.59988658725188, 0.578233641652396, 0.857143014646477, 0.419546744156364, 
    0.86111111111111, 0.368546841532502, 0.57665827843676, 0, 
    0.634786884620311, 0.525704269019409, 0, 0.525499900061238, 
    0, 0.655315105822717, 0.272325919964916, 0.49704342714548, 
    0.298337739268704, 0.353404918958734, 0, 0.626581495038945, 
    0.194137400421586, 0.30383614559919, 0.86111111111111, 0.146706111050693, 
    0, 0.390799983215771, 0.0505530330625736, 0.0846810923428288, 
    0, 0.256516766810296, 0.0820877815413834, 0.342958943003793, 
    0.286548644834347, 0, 0, 0.354372125041786, 0, 0, 0.575298053703998, 
    0.836375687560635, 0.649233906646029, 0.268848356761866, 
    0.0303814173355871, 0, 0.334474625243831, 0, 0.577709897852257, 
    0.534824058199853, 0.86111111111111, 0.447021583546581, 0.539165838793634, 
    0.402567821985543, 0.104769035766564, 0.249683058062621, 
    0.0344222372392216, 0, 0.411383545793762), guide.position = 1:75), .Names = c("A.area", 
"C.area", "G.area", "T.area", "Tot.area", "A.perc", "C.perc", 
"G.perc", "T.perc", "base.call", "index", "guide.seq", "T.pval", 
"C.pval", "G.pval", "A.pval", "guide.position"), row.names = 272:346, class = "data.frame")
```

*Report generated using EditR v1.0.8*
