# Supplementary material for: Mechanistic and genetic basis of single-strand templated repair at Cas12a-induced DNA breaks in Chlamydomonas reinhardtii
Source: Nat Commun. 2021 Nov 19;12:6751. doi: 10.1038/s41467-021-27004-1 (PMC8604939; doi:10.1038/s41467-021-27004-1)
Supplement: Supplementary file 22 — Source Data [file 41467_2021_27004_MOESM22_ESM.zip › Source Data/EditR analysis/EditR outputs/Antisense/rep1_ssODN_antisense_0_16_32.html]

EditR v1.0.8 report


# EditR v1.0.8 report

- Data QA
  - Filtering data
  - Percent noise peak area
  - Base information
- Predicted editing
  - Editing bar plot
  - Editing table plot
  - Table of editing results
- For use in R

## Data QA

### Filtering data

What the data looked like prefiltering:

and the post filtering signal / noise plot:

### Percent noise peak area

### Base information

Here’s information about the signal of each base, the critical percent value where any higher value would be called as significant, and Filliben’s correlation for how well the noise was modelled by the zero adjusted gamma distribution.

| Base | Average percent signal | Average peak area | Critical percent value | model mu | Fillibens correlation |
| --- | --- | --- | --- | --- | --- |
| A | 91.52692 | 428.5588 | 11.716500 | 3.346162 | 0.9516255 |
| C | 92.36680 | 447.0149 | 5.846458 | 2.137249 | 0.9955422 |
| G | 93.56324 | 442.2281 | 9.388407 | 2.465722 | 0.9883704 |
| T | 92.88526 | 521.8837 | 7.851218 | 2.562430 | 0.9929096 |

## Predicted editing

### Editing bar plot

### Editing table plot

### Table of editing results


Here’s the entire guide region

| Sanger position | Guide position | Guide sequence | Sanger base call | Focal base | Focal base peak area | p value |  |
| --- | --- | --- | --- | --- | --- | --- | --- |
| 277 | 1 | A | A | A | 94.56 | 0.000000e+00 | \* |
| 277 | 1 | A | A | C | 1.89 | 4.583601e-01 |  |
| 277 | 1 | A | A | G | 0.47 | 8.030719e-01 |  |
| 277 | 1 | A | A | T | 3.07 | 2.780860e-01 |  |
| 278 | 2 | A | A | A | 93.90 | 0.000000e+00 | \* |
| 278 | 2 | A | A | C | 2.03 | 4.145444e-01 |  |
| 278 | 2 | A | A | G | 1.22 | 6.075159e-01 |  |
| 278 | 2 | A | A | T | 2.85 | 3.178409e-01 |  |
| 279 | 3 | G | G | A | 2.23 | 4.730450e-01 |  |
| 279 | 3 | G | G | C | 0.99 | 7.598341e-01 |  |
| 279 | 3 | G | G | G | 95.30 | 0.000000e+00 | \* |
| 279 | 3 | G | G | T | 1.49 | 6.353120e-01 |  |
| 280 | 4 | A | A | A | 94.80 | 0.000000e+00 | \* |
| 280 | 4 | A | A | C | 0.95 | 7.731639e-01 |  |
| 280 | 4 | A | A | G | 0.71 | 7.406008e-01 |  |
| 280 | 4 | A | A | T | 3.55 | 2.083371e-01 |  |
| 281 | 5 | C | C | A | 3.70 | 2.814434e-01 |  |
| 281 | 5 | C | C | C | 93.63 | 0.000000e+00 | \* |
| 281 | 5 | C | C | G | 0.82 | 7.106844e-01 |  |
| 281 | 5 | C | C | T | 1.85 | 5.398478e-01 |  |
| 282 | 6 | T | T | A | 3.83 | 2.679986e-01 |  |
| 282 | 6 | T | T | C | 1.33 | 6.477482e-01 |  |
| 282 | 6 | T | T | G | 2.00 | 4.337974e-01 |  |
| 282 | 6 | T | T | T | 92.85 | 0.000000e+00 | \* |
| 283 | 7 | G | G | A | 2.40 | 4.462454e-01 |  |
| 283 | 7 | G | G | C | 0.00 | 9.047619e-01 |  |
| 283 | 7 | G | G | G | 96.57 | 0.000000e+00 | \* |
| 283 | 7 | G | G | T | 1.03 | 7.544633e-01 |  |
| 284 | 8 | G | G | A | 3.80 | 2.707573e-01 |  |
| 284 | 8 | G | G | C | 1.04 | 7.455798e-01 |  |
| 284 | 8 | G | G | G | 93.44 | 0.000000e+00 | \* |
| 284 | 8 | G | G | T | 1.73 | 5.711923e-01 |  |
| 285 | 9 | C | C | A | 2.72 | 4.004700e-01 |  |
| 285 | 9 | C | C | C | 92.45 | 0.000000e+00 | \* |
| 285 | 9 | C | C | G | 2.11 | 4.111450e-01 |  |
| 285 | 9 | C | C | T | 2.72 | 3.417296e-01 |  |
| 286 | 10 | C | C | A | 2.63 | 4.127762e-01 |  |
| 286 | 10 | C | C | C | 92.81 | 0.000000e+00 | \* |
| 286 | 10 | C | C | G | 2.98 | 2.736192e-01 |  |
| 286 | 10 | C | C | T | 1.58 | 6.103103e-01 |  |
| 287 | 11 | A | A | A | 92.38 | 0.000000e+00 | \* |
| 287 | 11 | A | A | C | 1.97 | 4.350055e-01 |  |
| 287 | 11 | A | A | G | 2.21 | 3.933747e-01 |  |
| 287 | 11 | A | A | T | 3.44 | 2.225840e-01 |  |
| 288 | 12 | G | G | A | 2.40 | 4.469464e-01 |  |
| 288 | 12 | G | G | C | 1.92 | 4.497393e-01 |  |
| 288 | 12 | G | G | G | 95.68 | 0.000000e+00 | \* |
| 288 | 12 | G | G | T | 0.00 | 9.042553e-01 |  |
| 289 | 13 | A | A | A | 94.91 | 0.000000e+00 | \* |
| 289 | 13 | A | A | C | 0.95 | 7.728337e-01 |  |
| 289 | 13 | A | A | G | 0.83 | 7.088095e-01 |  |
| 289 | 13 | A | A | T | 3.31 | 2.405534e-01 |  |
| 290 | 14 | C | C | A | 3.91 | 2.601225e-01 |  |
| 290 | 14 | C | C | C | 92.38 | 0.000000e+00 | \* |
| 290 | 14 | C | C | G | 1.95 | 4.423998e-01 |  |
| 290 | 14 | C | C | T | 1.76 | 5.631773e-01 |  |
| 291 | 15 | C | C | A | 7.05 | 7.411437e-02 |  |
| 291 | 15 | C | C | C | 88.25 | 0.000000e+00 | \* |
| 291 | 15 | C | C | G | 2.14 | 4.070455e-01 |  |
| 291 | 15 | C | C | T | 2.56 | 3.727618e-01 |  |
| 292 | 16 | G | G | A | 0.00 | 8.090909e-01 |  |
| 292 | 16 | G | G | C | 1.73 | 5.104540e-01 |  |
| 292 | 16 | G | G | G | 96.97 | 0.000000e+00 | \* |
| 292 | 16 | G | G | T | 1.30 | 6.849914e-01 |  |
| 293 | 17 | T | T | A | 0.00 | 8.090909e-01 |  |
| 293 | 17 | T | T | C | 1.88 | 4.630115e-01 |  |
| 293 | 17 | T | T | G | 1.88 | 4.578319e-01 |  |
| 293 | 17 | T | T | T | 96.25 | 0.000000e+00 | \* |
| 294 | 18 | G | G | A | 2.72 | 3.996687e-01 |  |
| 294 | 18 | G | G | C | 0.95 | 7.707816e-01 |  |
| 294 | 18 | G | G | G | 95.91 | 0.000000e+00 | \* |
| 294 | 18 | G | G | T | 0.41 | 8.786539e-01 |  |
| 295 | 19 | T | T | A | 0.85 | 7.057922e-01 |  |
| 295 | 19 | T | T | C | 1.49 | 5.919513e-01 |  |
| 295 | 19 | T | T | G | 2.35 | 3.697546e-01 |  |
| 295 | 19 | T | T | T | 95.31 | 0.000000e+00 | \* |
| 296 | 20 | T | T | A | 1.54 | 5.871322e-01 |  |
| 296 | 20 | T | T | C | 2.12 | 3.899923e-01 |  |
| 296 | 20 | T | T | G | 2.12 | 4.110360e-01 |  |
| 296 | 20 | T | T | T | 94.23 | 0.000000e+00 | \* |
| 297 | 21 | T | T | A | 0.00 | 8.090909e-01 |  |
| 297 | 21 | T | T | C | 2.45 | 3.008803e-01 |  |
| 297 | 21 | T | T | G | 2.07 | 4.193212e-01 |  |
| 297 | 21 | T | T | T | 95.48 | 0.000000e+00 | \* |
| 298 | 22 | G | G | A | 1.83 | 5.371012e-01 |  |
| 298 | 22 | G | G | C | 0.00 | 9.047619e-01 |  |
| 298 | 22 | G | G | G | 97.10 | 0.000000e+00 | \* |
| 298 | 22 | G | G | T | 1.07 | 7.447007e-01 |  |
| 299 | 23 | T | T | A | 0.00 | 8.090909e-01 |  |
| 299 | 23 | T | T | C | 2.07 | 4.036362e-01 |  |
| 299 | 23 | T | T | G | 1.84 | 4.655756e-01 |  |
| 299 | 23 | T | T | T | 96.09 | 0.000000e+00 | \* |
| 300 | 24 | G | G | A | 2.23 | 4.726146e-01 |  |
| 300 | 24 | G | G | C | 1.12 | 7.203588e-01 |  |
| 300 | 24 | G | G | G | 95.35 | 0.000000e+00 | \* |
| 300 | 24 | G | G | T | 1.30 | 6.843519e-01 |  |
| 301 | 25 | C | C | A | 1.60 | 5.761986e-01 |  |
| 301 | 25 | C | C | C | 94.28 | 0.000000e+00 | \* |
| 301 | 25 | C | C | G | 1.60 | 5.167900e-01 |  |
| 301 | 25 | C | C | T | 2.52 | 3.825472e-01 |  |
| 302 | 26 | A | A | A | 90.51 | 0.000000e+00 | \* |
| 302 | 26 | A | A | C | 3.52 | 1.153929e-01 |  |
| 302 | 26 | A | A | G | 2.98 | 2.738068e-01 |  |
| 302 | 26 | A | A | T | 2.98 | 2.936892e-01 |  |
| 303 | 27 | C | C | A | 1.74 | 5.530336e-01 |  |
| 303 | 27 | C | C | C | 95.75 | 0.000000e+00 | \* |
| 303 | 27 | C | C | G | 0.39 | 8.251398e-01 |  |
| 303 | 27 | C | C | T | 2.12 | 4.713377e-01 |  |
| 304 | 28 | T | T | A | 1.93 | 5.203065e-01 |  |
| 304 | 28 | T | T | C | 3.34 | 1.373992e-01 |  |
| 304 | 28 | T | T | G | 0.00 | 9.000000e-01 |  |
| 304 | 28 | T | T | T | 94.73 | 0.000000e+00 | \* |
| 305 | 29 | A | A | A | 96.29 | 0.000000e+00 | \* |
| 305 | 29 | A | A | C | 1.53 | 5.795710e-01 |  |
| 305 | 29 | A | A | G | 0.44 | 8.123576e-01 |  |
| 305 | 29 | A | A | T | 1.75 | 5.660681e-01 |  |
| 306 | 30 | C | C | A | 1.81 | 5.400401e-01 |  |
| 306 | 30 | C | C | C | 93.75 | 0.000000e+00 | \* |
| 306 | 30 | C | C | G | 1.41 | 5.607979e-01 |  |
| 306 | 30 | C | C | T | 3.02 | 2.863049e-01 |  |
| 307 | 31 | A | A | A | 90.41 | 0.000000e+00 | \* |
| 307 | 31 | A | A | C | 2.74 | 2.358204e-01 |  |
| 307 | 31 | A | A | G | 3.84 | 1.803224e-01 |  |
| 307 | 31 | A | A | T | 3.01 | 2.880867e-01 |  |
| 308 | 32 | C | C | A | 1.12 | 6.603412e-01 |  |
| 308 | 32 | C | C | C | 94.41 | 0.000000e+00 | \* |
| 308 | 32 | C | C | G | 2.01 | 4.305247e-01 |  |
| 308 | 32 | C | C | T | 2.46 | 3.945193e-01 |  |
| 309 | 33 | G | G | A | 2.96 | 3.681598e-01 |  |
| 309 | 33 | G | G | C | 1.78 | 4.960086e-01 |  |
| 309 | 33 | G | G | G | 95.27 | 0.000000e+00 | \* |
| 309 | 33 | G | G | T | 0.00 | 9.042553e-01 |  |
| 310 | 34 | G | G | A | 0.00 | 8.090909e-01 |  |
| 310 | 34 | G | G | C | 1.23 | 6.824211e-01 |  |
| 310 | 34 | G | G | G | 98.36 | 0.000000e+00 | \* |
| 310 | 34 | G | G | T | 0.41 | 8.785093e-01 |  |
| 311 | 35 | G | G | A | 4.05 | 2.462947e-01 |  |
| 311 | 35 | G | G | C | 1.27 | 6.701042e-01 |  |
| 311 | 35 | G | G | G | 93.42 | 0.000000e+00 | \* |
| 311 | 35 | G | G | T | 1.27 | 6.936828e-01 |  |
| 312 | 36 | C | C | A | 5.10 | 1.639285e-01 |  |
| 312 | 36 | C | C | C | 93.77 | 0.000000e+00 | \* |
| 312 | 36 | C | C | G | 1.13 | 6.292753e-01 |  |
| 312 | 36 | C | C | T | 0.00 | 9.042553e-01 |  |
| 313 | 37 | A | A | A | 92.91 | 0.000000e+00 | \* |
| 313 | 37 | A | A | C | 2.53 | 2.810217e-01 |  |
| 313 | 37 | A | A | G | 0.51 | 7.943663e-01 |  |
| 313 | 37 | A | A | T | 4.05 | 1.509131e-01 |  |
| 314 | 38 | C | C | A | 0.00 | 8.090909e-01 |  |
| 314 | 38 | C | C | C | 97.41 | 0.000000e+00 | \* |
| 314 | 38 | C | C | G | 1.11 | 6.354159e-01 |  |
| 314 | 38 | C | C | T | 1.48 | 6.370223e-01 |  |
| 315 | 39 | C | C | A | 6.58 | 9.020586e-02 |  |
| 315 | 39 | C | C | C | 91.30 | 0.000000e+00 | \* |
| 315 | 39 | C | C | G | 0.00 | 9.000000e-01 |  |
| 315 | 39 | C | C | T | 2.13 | 4.703523e-01 |  |
| 316 | 40 | C | C | A | 5.74 | 1.269898e-01 |  |
| 316 | 40 | C | C | C | 90.57 | 0.000000e+00 | \* |
| 316 | 40 | C | C | G | 0.82 | 7.111324e-01 |  |
| 316 | 40 | C | C | T | 2.87 | 3.135781e-01 |  |
| 317 | 41 | T | T | A | 3.77 | 2.737612e-01 |  |
| 317 | 41 | T | T | C | 2.51 | 2.852636e-01 |  |
| 317 | 41 | T | T | G | 3.95 | 1.703822e-01 |  |
| 317 | 41 | T | T | T | 89.77 | 0.000000e+00 | \* |
| 318 | 42 | G | G | A | 11.61 | 1.046132e-02 |  |
| 318 | 42 | G | G | C | 0.00 | 9.047619e-01 |  |
| 318 | 42 | G | G | G | 86.88 | 0.000000e+00 | \* |
| 318 | 42 | G | G | T | 1.51 | 6.291335e-01 |  |
| 319 | 43 | A | A | A | 69.43 | 1.931788e-14 | \* |
| 319 | 43 | A | A | C | 1.29 | 6.621493e-01 |  |
| 319 | 43 | A | A | G | 2.95 | 2.783712e-01 |  |
| 319 | 43 | A | A | T | 26.34 | 2.419466e-09 | \* |
| 320 | 44 | C | C | A | 6.17 | 1.065476e-01 |  |
| 320 | 44 | C | C | C | 89.36 | 0.000000e+00 | \* |
| 320 | 44 | C | C | G | 3.40 | 2.230128e-01 |  |
| 320 | 44 | C | C | T | 1.06 | 7.459291e-01 |  |
| 321 | 45 | C | C | A | 14.90 | 2.433157e-03 | \* |
| 321 | 45 | C | C | C | 77.76 | 0.000000e+00 | \* |
| 321 | 45 | C | C | G | 4.90 | 1.056683e-01 |  |
| 321 | 45 | C | C | T | 2.45 | 3.970752e-01 |  |
| 322 | 46 | G | G | A | 11.02 | 1.357543e-02 |  |
| 322 | 46 | G | G | C | 1.65 | 5.369383e-01 |  |
| 322 | 46 | G | G | G | 87.33 | 0.000000e+00 | \* |
| 322 | 46 | G | G | T | 0.00 | 9.042553e-01 |  |
| 323 | 47 | A | A | A | 94.01 | 0.000000e+00 | \* |
| 323 | 47 | A | A | C | 1.00 | 7.572480e-01 |  |
| 323 | 47 | A | A | G | 0.71 | 7.395201e-01 |  |
| 323 | 47 | A | A | T | 4.28 | 1.298293e-01 |  |
| 324 | 48 | C | C | A | 5.24 | 1.551348e-01 |  |
| 324 | 48 | C | C | C | 90.24 | 0.000000e+00 | \* |
| 324 | 48 | C | C | G | 2.62 | 3.253201e-01 |  |
| 324 | 48 | C | C | T | 1.90 | 5.253859e-01 |  |
| 325 | 49 | G | G | A | 7.77 | 5.477840e-02 |  |
| 325 | 49 | G | G | C | 1.77 | 4.987703e-01 |  |
| 325 | 49 | G | G | G | 88.69 | 0.000000e+00 | \* |
| 325 | 49 | G | G | T | 1.77 | 5.608418e-01 |  |
| 326 | 50 | G | G | A | 1.75 | 5.508163e-01 |  |
| 326 | 50 | G | G | C | 1.09 | 7.272066e-01 |  |
| 326 | 50 | G | G | G | 97.16 | 0.000000e+00 | \* |
| 326 | 50 | G | G | T | 0.00 | 9.042553e-01 |  |
| 327 | 51 | C | C | A | 2.93 | 3.718536e-01 |  |
| 327 | 51 | C | C | C | 92.31 | 0.000000e+00 | \* |
| 327 | 51 | C | C | G | 2.56 | 3.338486e-01 |  |
| 327 | 51 | C | C | T | 2.20 | 4.537067e-01 |  |
| 328 | 52 | A | A | A | 91.50 | 0.000000e+00 | \* |
| 328 | 52 | A | A | C | 1.62 | 5.483208e-01 |  |
| 328 | 52 | A | A | G | 2.02 | 4.284120e-01 |  |
| 328 | 52 | A | A | T | 4.86 | 8.788608e-02 |  |
| 329 | 53 | A | A | A | 93.22 | 0.000000e+00 | \* |
| 329 | 53 | A | A | C | 0.25 | 9.001395e-01 |  |
| 329 | 53 | A | A | G | 3.52 | 2.109676e-01 |  |
| 329 | 53 | A | A | T | 3.02 | 2.878524e-01 |  |
| 330 | 54 | G | G | A | 4.60 | 1.993105e-01 |  |
| 330 | 54 | G | G | C | 0.00 | 9.047619e-01 |  |
| 330 | 54 | G | G | G | 94.17 | 0.000000e+00 | \* |
| 330 | 54 | G | G | T | 1.23 | 7.038968e-01 |  |
| 331 | 55 | A | A | A | 94.62 | 0.000000e+00 | \* |
| 331 | 55 | A | A | C | 0.00 | 9.047619e-01 |  |
| 331 | 55 | A | A | G | 2.91 | 2.826463e-01 |  |
| 331 | 55 | A | A | T | 2.47 | 3.933351e-01 |  |
| 332 | 56 | A | A | A | 92.04 | 0.000000e+00 | \* |
| 332 | 56 | A | A | C | 0.00 | 9.047619e-01 |  |
| 332 | 56 | A | A | G | 3.71 | 1.915585e-01 |  |
| 332 | 56 | A | A | T | 4.24 | 1.329207e-01 |  |
| 333 | 57 | G | G | A | 1.88 | 5.291593e-01 |  |
| 333 | 57 | G | G | C | 1.88 | 4.620449e-01 |  |
| 333 | 57 | G | G | G | 91.35 | 0.000000e+00 | \* |
| 333 | 57 | G | G | T | 4.89 | 8.616015e-02 |  |
| 334 | 58 | T | T | A | 0.56 | 7.527327e-01 |  |
| 334 | 58 | T | T | C | 2.79 | 2.266856e-01 |  |
| 334 | 58 | T | T | G | 1.11 | 6.341008e-01 |  |
| 334 | 58 | T | T | T | 95.54 | 0.000000e+00 | \* |
| 335 | 59 | T | T | A | 17.91 | 6.237678e-04 | \* |
| 335 | 59 | T | T | C | 3.21 | 1.551772e-01 |  |
| 335 | 59 | T | T | G | 1.87 | 4.588596e-01 |  |
| 335 | 59 | T | T | T | 77.01 | 0.000000e+00 | \* |
| 336 | 60 | C | C | A | 0.80 | 7.138560e-01 |  |
| 336 | 60 | C | C | C | 91.42 | 0.000000e+00 | \* |
| 336 | 60 | C | C | G | 6.97 | 3.611442e-02 |  |
| 336 | 60 | C | C | T | 0.80 | 8.076567e-01 |  |
| 337 | 61 | G | G | A | 5.99 | 1.148487e-01 |  |
| 337 | 61 | G | G | C | 1.41 | 6.210517e-01 |  |
| 337 | 61 | G | G | G | 91.55 | 0.000000e+00 | \* |
| 337 | 61 | G | G | T | 1.06 | 7.478138e-01 |  |
| 338 | 62 | A | A | A | 93.49 | 0.000000e+00 | \* |
| 338 | 62 | A | A | C | 1.67 | 5.301776e-01 |  |
| 338 | 62 | A | A | G | 2.23 | 3.899203e-01 |  |
| 338 | 62 | A | A | T | 2.60 | 3.649442e-01 |  |
| 339 | 63 | C | C | A | 3.60 | 2.919895e-01 |  |
| 339 | 63 | C | C | C | 93.29 | 0.000000e+00 | \* |
| 339 | 63 | C | C | G | 1.44 | 5.542793e-01 |  |
| 339 | 63 | C | C | T | 1.68 | 5.839148e-01 |  |
| 340 | 64 | A | A | A | 86.91 | 0.000000e+00 | \* |
| 340 | 64 | A | A | C | 1.82 | 4.818991e-01 |  |
| 340 | 64 | A | A | G | 9.09 | 1.172703e-02 |  |
| 340 | 64 | A | A | T | 2.18 | 4.574694e-01 |  |
| 341 | 65 | G | G | A | 0.48 | 7.645219e-01 |  |
| 341 | 65 | G | G | C | 4.04 | 6.948424e-02 |  |
| 341 | 65 | G | G | G | 95.49 | 0.000000e+00 | \* |
| 341 | 65 | G | G | T | 0.00 | 9.042553e-01 |  |
| 342 | 66 | C | C | A | 0.57 | 7.510894e-01 |  |
| 342 | 66 | C | C | C | 89.20 | 0.000000e+00 | \* |
| 342 | 66 | C | C | G | 3.69 | 1.934935e-01 |  |
| 342 | 66 | C | C | T | 6.53 | 2.670308e-02 |  |
| 343 | 67 | T | T | A | 0.00 | 8.090909e-01 |  |
| 343 | 67 | T | T | C | 3.50 | 1.185175e-01 |  |
| 343 | 67 | T | T | G | 2.52 | 3.401530e-01 |  |
| 343 | 67 | T | T | T | 93.98 | 0.000000e+00 | \* |
| 344 | 68 | C | C | A | 3.36 | 3.189217e-01 |  |
| 344 | 68 | C | C | C | 94.24 | 0.000000e+00 | \* |
| 344 | 68 | C | C | G | 0.00 | 9.000000e-01 |  |
| 344 | 68 | C | C | T | 2.40 | 4.081601e-01 |  |
| 345 | 69 | C | C | A | 2.73 | 3.990785e-01 |  |
| 345 | 69 | C | C | C | 92.98 | 0.000000e+00 | \* |
| 345 | 69 | C | C | G | 1.17 | 6.200425e-01 |  |
| 345 | 69 | C | C | T | 3.12 | 2.706182e-01 |  |
| 346 | 70 | C | C | A | 4.61 | 1.985581e-01 |  |
| 346 | 70 | C | C | C | 87.32 | 0.000000e+00 | \* |
| 346 | 70 | C | C | G | 5.76 | 6.775419e-02 |  |
| 346 | 70 | C | C | T | 2.31 | 4.288446e-01 |  |
| 347 | 71 | G | G | A | 6.06 | 1.114138e-01 |  |
| 347 | 71 | G | G | C | 5.15 | 2.156756e-02 |  |
| 347 | 71 | G | G | G | 87.88 | 0.000000e+00 | \* |
| 347 | 71 | G | G | T | 0.91 | 7.837317e-01 |  |
| 348 | 72 | C | C | A | 0.00 | 8.090909e-01 |  |
| 348 | 72 | C | C | C | 92.20 | 0.000000e+00 | \* |
| 348 | 72 | C | C | G | 6.07 | 5.782779e-02 |  |
| 348 | 72 | C | C | T | 1.73 | 5.693644e-01 |  |
| 349 | 73 | G | G | A | 8.71 | 3.684103e-02 |  |
| 349 | 73 | G | G | C | 3.23 | 1.527217e-01 |  |
| 349 | 73 | G | G | G | 87.42 | 0.000000e+00 | \* |
| 349 | 73 | G | G | T | 0.65 | 8.404619e-01 |  |
| 350 | 74 | A | A | A | 91.01 | 0.000000e+00 | \* |
| 350 | 74 | A | A | C | 2.57 | 2.723205e-01 |  |
| 350 | 74 | A | A | G | 2.14 | 4.061952e-01 |  |
| 350 | 74 | A | A | T | 4.28 | 1.295668e-01 |  |
| 351 | 75 | C | C | A | 3.55 | 2.973820e-01 |  |
| 351 | 75 | C | C | C | 74.94 | 0.000000e+00 | \* |
| 351 | 75 | C | C | G | 18.18 | 8.214290e-05 | \* |
| 351 | 75 | C | C | T | 3.33 | 2.387457e-01 |  |

## For use in R

If you want to work with the results in R, here is output that you can copy and paste in your terminal to get:

The base information:

```
structure(list(focal.base = c("A", "C", "G", "T"), avg.percsignal = c(91.5269202212091, 
92.3668016548347, 93.5632432984721, 92.8852634742951), avg.areasignal = c(428.558823529412, 
447.014925373134, 442.228070175439, 521.883720930233), crit.perc.area = c(11.7164998745881, 
5.84645842905321, 9.38840678024172, 7.85121800817693), mu = c(3.34616200021648, 
2.13724947362702, 2.46572218947525, 2.56243039159784), fillibens = c(0.95162552533231, 
0.995542247759947, 0.988370396433864, 0.992909640142861)), .Names = c("focal.base", 
"avg.percsignal", "avg.areasignal", "crit.perc.area", "mu", "fillibens"
), row.names = c(NA, -4L), class = "data.frame")
```

the data.frame that contains information on the guide region:

```
structure(list(A.area = c(400, 462, 9, 802, 18, 23, 21, 22, 9, 
15, 376, 10, 802, 20, 33, 0, 0, 20, 4, 8, 0, 12, 0, 12, 7, 334, 
9, 11, 441, 9, 330, 5, 10, 0, 16, 18, 367, 0, 34, 28, 21, 77, 
377, 29, 73, 40, 659, 22, 22, 8, 8, 226, 371, 15, 422, 347, 5, 
2, 67, 3, 17, 503, 15, 239, 2, 2, 0, 14, 14, 16, 20, 0, 27, 425, 
16), C.area = c(8, 10, 4, 8, 456, 8, 0, 6, 306, 529, 8, 8, 8, 
473, 413, 8, 7, 7, 7, 11, 13, 0, 9, 6, 412, 13, 496, 19, 7, 465, 
10, 422, 6, 6, 5, 331, 10, 527, 472, 442, 14, 0, 7, 420, 381, 
6, 7, 379, 5, 5, 252, 4, 1, 0, 0, 0, 5, 10, 12, 341, 4, 9, 389, 
5, 17, 314, 18, 393, 477, 303, 17, 319, 10, 12, 338), G.area = c(2, 
6, 385, 6, 4, 12, 844, 541, 7, 17, 9, 399, 7, 10, 10, 448, 7, 
704, 11, 11, 11, 636, 8, 513, 7, 11, 2, 0, 2, 7, 14, 9, 322, 
480, 369, 4, 2, 6, 0, 4, 22, 576, 16, 16, 24, 317, 5, 11, 251, 
444, 7, 5, 14, 307, 13, 14, 243, 4, 7, 26, 260, 12, 6, 25, 402, 
13, 13, 0, 6, 20, 290, 21, 271, 10, 82), T.area = c(13, 14, 6, 
30, 9, 558, 9, 10, 9, 9, 14, 0, 28, 9, 12, 6, 359, 3, 447, 490, 
507, 7, 418, 7, 11, 11, 11, 539, 8, 15, 11, 11, 0, 2, 5, 0, 16, 
8, 11, 14, 500, 10, 143, 5, 12, 0, 30, 8, 5, 0, 6, 12, 12, 4, 
11, 16, 13, 343, 288, 3, 3, 14, 7, 6, 0, 23, 484, 10, 16, 8, 
3, 6, 2, 20, 15), Tot.area = c(423, 492, 404, 846, 487, 601, 
874, 579, 331, 570, 407, 417, 845, 512, 468, 462, 373, 734, 469, 
520, 531, 655, 435, 538, 437, 369, 518, 569, 458, 496, 365, 447, 
338, 488, 395, 353, 395, 541, 517, 488, 557, 663, 543, 470, 490, 
363, 701, 420, 283, 457, 273, 247, 398, 326, 446, 377, 266, 359, 
374, 373, 284, 538, 417, 275, 421, 352, 515, 417, 513, 347, 330, 
346, 310, 467, 451), A.perc = c(94.5626477541371, 93.9024390243902, 
2.22772277227723, 94.7990543735225, 3.69609856262834, 3.82695507487521, 
2.40274599542334, 3.79965457685665, 2.7190332326284, 2.63157894736842, 
92.3832923832924, 2.39808153477218, 94.9112426035503, 3.90625, 
7.05128205128205, 0, 0, 2.72479564032698, 0.852878464818763, 
1.53846153846154, 0, 1.83206106870229, 0, 2.23048327137546, 1.60183066361556, 
90.5149051490515, 1.73745173745174, 1.93321616871705, 96.2882096069869, 
1.81451612903226, 90.4109589041096, 1.11856823266219, 2.9585798816568, 
0, 4.05063291139241, 5.09915014164306, 92.9113924050633, 0, 6.57640232108317, 
5.73770491803279, 3.77019748653501, 11.6138763197587, 69.4290976058932, 
6.17021276595745, 14.8979591836735, 11.0192837465565, 94.0085592011412, 
5.23809523809524, 7.77385159010601, 1.75054704595186, 2.93040293040293, 
91.497975708502, 93.21608040201, 4.60122699386503, 94.6188340807175, 
92.0424403183024, 1.8796992481203, 0.557103064066852, 17.9144385026738, 
0.804289544235925, 5.98591549295775, 93.4944237918216, 3.59712230215827, 
86.9090909090909, 0.475059382422803, 0.568181818181818, 0, 3.35731414868106, 
2.72904483430799, 4.61095100864553, 6.06060606060606, 0, 8.70967741935484, 
91.0064239828694, 3.54767184035477), C.perc = c(1.89125295508274, 
2.03252032520325, 0.99009900990099, 0.945626477541371, 93.6344969199179, 
1.33111480865225, 0, 1.03626943005181, 92.4471299093656, 92.8070175438596, 
1.96560196560197, 1.91846522781775, 0.946745562130177, 92.3828125, 
88.2478632478633, 1.73160173160173, 1.87667560321716, 0.953678474114441, 
1.49253731343284, 2.11538461538462, 2.44821092278719, 0, 2.06896551724138, 
1.11524163568773, 94.279176201373, 3.5230352303523, 95.7528957528958, 
3.33919156414763, 1.52838427947598, 93.75, 2.73972602739726, 
94.407158836689, 1.77514792899408, 1.22950819672131, 1.26582278481013, 
93.7677053824363, 2.53164556962025, 97.4121996303142, 91.2959381044487, 
90.5737704918033, 2.51346499102334, 0, 1.28913443830571, 89.3617021276596, 
77.7551020408163, 1.65289256198347, 0.998573466476462, 90.2380952380952, 
1.76678445229682, 1.09409190371991, 92.3076923076923, 1.61943319838057, 
0.251256281407035, 0, 0, 0, 1.8796992481203, 2.78551532033426, 
3.20855614973262, 91.4209115281501, 1.40845070422535, 1.6728624535316, 
93.2853717026379, 1.81818181818182, 4.03800475059382, 89.2045454545455, 
3.49514563106796, 94.2446043165468, 92.9824561403509, 87.3198847262248, 
5.15151515151515, 92.1965317919075, 3.2258064516129, 2.56959314775161, 
74.9445676274945), G.perc = c(0.472813238770686, 1.21951219512195, 
95.2970297029703, 0.709219858156028, 0.82135523613963, 1.99667221297837, 
96.5675057208238, 93.4369602763385, 2.11480362537764, 2.98245614035088, 
2.21130221130221, 95.6834532374101, 0.828402366863905, 1.953125, 
2.13675213675214, 96.969696969697, 1.87667560321716, 95.9128065395095, 
2.3454157782516, 2.11538461538462, 2.07156308851224, 97.0992366412214, 
1.83908045977011, 95.3531598513011, 1.60183066361556, 2.9810298102981, 
0.386100386100386, 0, 0.436681222707424, 1.41129032258065, 3.83561643835616, 
2.01342281879195, 95.2662721893491, 98.3606557377049, 93.4177215189873, 
1.13314447592068, 0.506329113924051, 1.1090573012939, 0, 0.819672131147541, 
3.94973070017953, 86.8778280542986, 2.94659300184162, 3.40425531914894, 
4.89795918367347, 87.3278236914601, 0.713266761768902, 2.61904761904762, 
88.6925795053004, 97.1553610503282, 2.56410256410256, 2.02429149797571, 
3.51758793969849, 94.1717791411043, 2.91479820627803, 3.71352785145889, 
91.3533834586466, 1.1142061281337, 1.8716577540107, 6.97050938337802, 
91.5492957746479, 2.23048327137546, 1.43884892086331, 9.09090909090909, 
95.4869358669834, 3.69318181818182, 2.52427184466019, 0, 1.16959064327485, 
5.76368876080692, 87.8787878787879, 6.06936416184971, 87.4193548387097, 
2.14132762312634, 18.1818181818182), T.perc = c(3.07328605200946, 
2.84552845528455, 1.48514851485149, 3.54609929078014, 1.84804928131417, 
92.8452579034942, 1.02974828375286, 1.72711571675302, 2.7190332326284, 
1.57894736842105, 3.43980343980344, 0, 3.31360946745562, 1.7578125, 
2.56410256410256, 1.2987012987013, 96.2466487935657, 0.408719346049046, 
95.3091684434968, 94.2307692307692, 95.4802259887006, 1.06870229007634, 
96.0919540229885, 1.30111524163569, 2.51716247139588, 2.9810298102981, 
2.12355212355212, 94.7275922671353, 1.74672489082969, 3.0241935483871, 
3.01369863013699, 2.46085011185682, 0, 0.409836065573771, 1.26582278481013, 
0, 4.05063291139241, 1.47874306839187, 2.12765957446809, 2.86885245901639, 
89.7666068222621, 1.50829562594268, 26.3351749539595, 1.06382978723404, 
2.44897959183673, 0, 4.27960057061341, 1.9047619047619, 1.76678445229682, 
0, 2.1978021978022, 4.8582995951417, 3.01507537688442, 1.22699386503067, 
2.46636771300448, 4.24403183023873, 4.88721804511278, 95.5431754874652, 
77.0053475935829, 0.804289544235925, 1.05633802816901, 2.60223048327138, 
1.67865707434053, 2.18181818181818, 0, 6.53409090909091, 93.9805825242718, 
2.39808153477218, 3.11890838206628, 2.30547550432277, 0.909090909090909, 
1.73410404624277, 0.645161290322581, 4.28265524625268, 3.32594235033259
), base.call = c("A", "A", "G", "A", "C", "T", "G", "G", "C", 
"C", "A", "G", "A", "C", "C", "G", "T", "G", "T", "T", "T", "G", 
"T", "G", "C", "A", "C", "T", "A", "C", "A", "C", "G", "G", "G", 
"C", "A", "C", "C", "C", "T", "G", "A", "C", "C", "G", "A", "C", 
"G", "G", "C", "A", "A", "G", "A", "A", "G", "T", "T", "C", "G", 
"A", "C", "A", "G", "C", "T", "C", "C", "C", "G", "C", "G", "A", 
"C"), index = 277:351, guide.seq = c("A", "A", "G", "A", "C", 
"T", "G", "G", "C", "C", "A", "G", "A", "C", "C", "G", "T", "G", 
"T", "T", "T", "G", "T", "G", "C", "A", "C", "T", "A", "C", "A", 
"C", "G", "G", "G", "C", "A", "C", "C", "C", "T", "G", "A", "C", 
"C", "G", "A", "C", "G", "G", "C", "A", "A", "G", "A", "A", "G", 
"T", "T", "C", "G", "A", "C", "A", "G", "C", "T", "C", "C", "C", 
"G", "C", "G", "A", "C"), T.pval = c(0.278086045742941, 0.317840911219394, 
0.63531203000606, 0.208337087118373, 0.539847822405939, 0, 0.75446332812699, 
0.571192287689539, 0.341729590944325, 0.610310337311268, 0.222583982049721, 
0.904255319142527, 0.240553438004072, 0.563177320051943, 0.372761778473793, 
0.684991375103836, 0, 0.878653903544696, 0, 0, 0, 0.74470072727049, 
0, 0.684351926137424, 0.382547223014439, 0.293689161139928, 0.471337672574877, 
0, 0.566068073897448, 0.286304901717129, 0.288086680678018, 0.394519311777118, 
0.904255319142527, 0.878509295001477, 0.693682806734558, 0.904255319142527, 
0.150913131866237, 0.637022338049292, 0.470352332997049, 0.313578087600734, 
0, 0.629133481277172, 2.41946618295685e-09, 0.745929050245218, 
0.397075185999482, 0.904255319142527, 0.129829262346727, 0.525385932913116, 
0.560841790254305, 0.904255319142527, 0.45370665603056, 0.0878860794326184, 
0.287852444231191, 0.703896755313519, 0.3933351036768, 0.132920672539543, 
0.0861601479510785, 0, 0, 0.807656657421718, 0.747813772041361, 
0.364944166529847, 0.583914813214923, 0.457469393413018, 0.904255319142527, 
0.0267030819745336, 0, 0.408160099234828, 0.270618217765834, 
0.428844576554993, 0.783731673056213, 0.569364436962841, 0.840461919957455, 
0.129566809878274, 0.238745653014235), C.pval = c(0.458360115545944, 
0.414544359344069, 0.759834070986423, 0.773163886455827, 0, 0.647748171849025, 
0.904761904754824, 0.745579794426137, 0, 0, 0.435005504384216, 
0.449739316365156, 0.772833650461847, 0, 0, 0.510454036013443, 
0.463011535285256, 0.770781639611375, 0.591951281834002, 0.389992302394222, 
0.300880304688323, 0.904761904754824, 0.403636212038679, 0.720358810394534, 
0, 0.115392920342554, 0, 0.137399165829339, 0.579571006569072, 
0, 0.235820437179996, 0, 0.496008574487092, 0.682421110038805, 
0.67010420973598, 0, 0.281021685371787, 0, 0, 0, 0.285263559567859, 
0.904761904754824, 0.662149289606126, 0, 0, 0.536938348910333, 
0.757248014390612, 0, 0.49877034385872, 0.72720663541074, 0, 
0.548320775704933, 0.900139455113832, 0.904761904754824, 0.904761904754824, 
0.904761904754824, 0.462044855579032, 0.226685619112904, 0.155177150875848, 
0, 0.621051670860166, 0.530177601027356, 0, 0.481899078885894, 
0.0694842385522857, 0, 0.118517543619735, 0, 0, 0, 0.0215675597185663, 
0, 0.152721664942411, 0.272320546806029, 0), G.pval = c(0.803071867704042, 
0.607515861396607, 0, 0.740600838042369, 0.710684425213195, 0.433797385630843, 
0, 0, 0.411144966772657, 0.27361919718117, 0.39337470073177, 
0, 0.708809450964029, 0.442399757938037, 0.407045510957934, 0, 
0.457831904097918, 0, 0.369754582737622, 0.411036012554602, 0.41932118023569, 
0, 0.465575569774855, 0, 0.516790049748461, 0.27380678163817, 
0.825139833609549, 0.899999999997222, 0.81235757824356, 0.560797894874784, 
0.180322420187384, 0.43052470271673, 0, 0, 0, 0.629275258164641, 
0.79436632562125, 0.635415892355843, 0.899999999997222, 0.711132372415876, 
0.170382221458533, 0, 0.278371189556985, 0.223012810244534, 0.105668289328701, 
0, 0.739520082010014, 0.325320125903326, 0, 0, 0.333848639663293, 
0.428411966653185, 0.210967641189314, 0, 0.282646277235063, 0.191558486683636, 
0, 0.634100757081818, 0.458859555374103, 0.0361144248877315, 
0, 0.389920268051609, 0.554279346306283, 0.011727026285705, 0, 
0.193493504818684, 0.340153044267349, 0.899999999997222, 0.62004249451715, 
0.0677541919216277, 0, 0.0578277924819022, 0, 0.406195208353839, 
8.21429040827049e-05), A.pval = c(0, 0, 0.473045024917405, 0, 
0.281443355315604, 0.267998635301368, 0.446245423334653, 0.270757315820481, 
0.400469987221193, 0.412776155461256, 0, 0.446946408246861, 0, 
0.260122549441149, 0.074114367693025, 0.80909090909091, 0.80909090909091, 
0.399668668741819, 0.705792155830046, 0.58713219769666, 0.80909090909091, 
0.537101162644108, 0.80909090909091, 0.472614556612691, 0.576198631402144, 
0, 0.553033583419677, 0.520306524492118, 0, 0.540040117271659, 
0, 0.660341233716173, 0.368159766554546, 0.80909090909091, 0.246294708648202, 
0.163928466081358, 0, 0.80909090909091, 0.0902058590760367, 0.126989816636301, 
0.273761163567026, 0.0104613163271655, 1.93178806284777e-14, 
0.106547602664621, 0.00243315675259048, 0.0135754275164121, 0, 
0.15513480405569, 0.0547784041945012, 0.550816288155109, 0.371853623990717, 
0, 0, 0.199310542119804, 0, 0, 0.529159320568435, 0.752732745384356, 
0.000623767766588346, 0.713856014270827, 0.114848723022935, 0, 
0.291989450550795, 0, 0.764521873167327, 0.751089394573155, 0.80909090909091, 
0.318921683247775, 0.399078534910108, 0.198558106829527, 0.111413834831275, 
0.80909090909091, 0.0368410290465899, 0, 0.297381999135045), 
    guide.position = 1:75), .Names = c("A.area", "C.area", "G.area", 
"T.area", "Tot.area", "A.perc", "C.perc", "G.perc", "T.perc", 
"base.call", "index", "guide.seq", "T.pval", "C.pval", "G.pval", 
"A.pval", "guide.position"), row.names = 277:351, class = "data.frame")
```

*Report generated using EditR v1.0.8*
