# Supplementary material for: Mechanistic and genetic basis of single-strand templated repair at Cas12a-induced DNA breaks in Chlamydomonas reinhardtii
Source: Nat Commun. 2021 Nov 19;12:6751. doi: 10.1038/s41467-021-27004-1 (PMC8604939; doi:10.1038/s41467-021-27004-1)
Supplement: Supplementary file 22 — Source Data [file 41467_2021_27004_MOESM22_ESM.zip › Source Data/EditR analysis/EditR outputs/Antisense/rep1_ssODN_antisense_16.html]

EditR v1.0.8 report


# EditR v1.0.8 report

- Data QA
  - Filtering data
  - Percent noise peak area
  - Base information
- Predicted editing
  - Editing bar plot
  - Editing table plot
  - Table of editing results
- For use in R

## Data QA

### Filtering data

What the data looked like prefiltering:

and the post filtering signal / noise plot:

### Percent noise peak area

### Base information

Here’s information about the signal of each base, the critical percent value where any higher value would be called as significant, and Filliben’s correlation for how well the noise was modelled by the zero adjusted gamma distribution.

| Base | Average percent signal | Average peak area | Critical percent value | model mu | Fillibens correlation |
| --- | --- | --- | --- | --- | --- |
| A | 92.30842 | 553.6176 | 10.946644 | 3.271642 | 0.9691550 |
| C | 92.31755 | 573.5224 | 5.790482 | 2.372988 | 0.9928352 |
| G | 93.58571 | 575.8750 | 9.376427 | 2.549636 | 0.9899429 |
| T | 92.46135 | 667.9318 | 7.991690 | 2.597201 | 0.9975629 |

## Predicted editing

### Editing bar plot

### Editing table plot

### Table of editing results


Here’s the entire guide region

| Sanger position | Guide position | Guide sequence | Sanger base call | Focal base | Focal base peak area | p value |  |
| --- | --- | --- | --- | --- | --- | --- | --- |
| 276 | 1 | A | A | A | 95.00 | 0.0000000000 | \* |
| 276 | 1 | A | A | C | 1.92 | 0.5334021778 |  |
| 276 | 1 | A | A | G | 0.58 | 0.7730369261 |  |
| 276 | 1 | A | A | T | 2.50 | 0.3972314579 |  |
| 277 | 2 | A | A | A | 93.51 | 0.0000000000 | \* |
| 277 | 2 | A | A | C | 2.60 | 0.3242107195 |  |
| 277 | 2 | A | A | G | 1.46 | 0.5571991767 |  |
| 277 | 2 | A | A | T | 2.44 | 0.4112343466 |  |
| 278 | 3 | G | G | A | 1.99 | 0.5556746346 |  |
| 278 | 3 | G | G | C | 1.39 | 0.7099338271 |  |
| 278 | 3 | G | G | G | 95.42 | 0.0000000000 | \* |
| 278 | 3 | G | G | T | 1.20 | 0.7230910197 |  |
| 279 | 4 | A | A | A | 95.03 | 0.0000000000 | \* |
| 279 | 4 | A | A | C | 0.96 | 0.8255006030 |  |
| 279 | 4 | A | A | G | 0.76 | 0.7269752743 |  |
| 279 | 4 | A | A | T | 3.25 | 0.2600235240 |  |
| 280 | 5 | C | C | A | 3.53 | 0.3139911546 |  |
| 280 | 5 | C | C | C | 93.11 | 0.0000000000 | \* |
| 280 | 5 | C | C | G | 1.44 | 0.5615096141 |  |
| 280 | 5 | C | C | T | 1.92 | 0.5322467080 |  |
| 281 | 6 | T | T | A | 4.62 | 0.1991525377 |  |
| 281 | 6 | T | T | C | 1.41 | 0.7049914750 |  |
| 281 | 6 | T | T | G | 2.05 | 0.4323583196 |  |
| 281 | 6 | T | T | T | 91.92 | 0.0000000000 | \* |
| 282 | 7 | G | G | A | 2.10 | 0.5362119499 |  |
| 282 | 7 | G | G | C | 1.14 | 0.7836887567 |  |
| 282 | 7 | G | G | G | 95.63 | 0.0000000000 | \* |
| 282 | 7 | G | G | T | 1.14 | 0.7382455885 |  |
| 283 | 8 | G | G | A | 3.08 | 0.3740164473 |  |
| 283 | 8 | G | G | C | 0.94 | 0.8288693958 |  |
| 283 | 8 | G | G | G | 94.37 | 0.0000000000 | \* |
| 283 | 8 | G | G | T | 1.61 | 0.6138212785 |  |
| 284 | 9 | C | C | A | 3.56 | 0.3093025189 |  |
| 284 | 9 | C | C | C | 92.40 | 0.0000000000 | \* |
| 284 | 9 | C | C | G | 1.90 | 0.4621790489 |  |
| 284 | 9 | C | C | T | 2.14 | 0.4793596289 |  |
| 285 | 10 | C | C | A | 2.54 | 0.4596206663 |  |
| 285 | 10 | C | C | C | 94.23 | 0.0000000000 | \* |
| 285 | 10 | C | C | G | 3.24 | 0.2483503714 |  |
| 285 | 10 | C | C | T | 0.00 | 0.9139784946 |  |
| 286 | 11 | A | A | A | 92.84 | 0.0000000000 | \* |
| 286 | 11 | A | A | C | 1.93 | 0.5296160173 |  |
| 286 | 11 | A | A | G | 1.93 | 0.4553356353 |  |
| 286 | 11 | A | A | T | 3.29 | 0.2537791517 |  |
| 287 | 12 | G | G | A | 2.21 | 0.5166984999 |  |
| 287 | 12 | G | G | C | 3.12 | 0.2027819944 |  |
| 287 | 12 | G | G | G | 92.83 | 0.0000000000 | \* |
| 287 | 12 | G | G | T | 1.84 | 0.5538558665 |  |
| 288 | 13 | A | A | A | 95.13 | 0.0000000000 | \* |
| 288 | 13 | A | A | C | 0.94 | 0.8290932858 |  |
| 288 | 13 | A | A | G | 0.66 | 0.7537499544 |  |
| 288 | 13 | A | A | T | 3.28 | 0.2549893047 |  |
| 289 | 14 | C | C | A | 4.28 | 0.2297106071 |  |
| 289 | 14 | C | C | C | 91.90 | 0.0000000000 | \* |
| 289 | 14 | C | C | G | 1.83 | 0.4755507930 |  |
| 289 | 14 | C | C | T | 1.99 | 0.5160227425 |  |
| 290 | 15 | C | C | A | 8.86 | 0.0278921202 |  |
| 290 | 15 | C | C | C | 88.29 | 0.0000000000 | \* |
| 290 | 15 | C | C | G | 1.34 | 0.5859065656 |  |
| 290 | 15 | C | C | T | 1.51 | 0.6412978104 |  |
| 291 | 16 | G | G | A | 4.28 | 0.2299464423 |  |
| 291 | 16 | G | G | C | 2.22 | 0.4357807149 |  |
| 291 | 16 | G | G | G | 92.08 | 0.0000000000 | \* |
| 291 | 16 | G | G | T | 1.43 | 0.6622287983 |  |
| 292 | 17 | T | T | A | 5.18 | 0.1554663292 |  |
| 292 | 17 | T | T | C | 2.69 | 0.3006492551 |  |
| 292 | 17 | T | T | G | 1.92 | 0.4583154323 |  |
| 292 | 17 | T | T | T | 90.21 | 0.0000000000 | \* |
| 293 | 18 | G | G | A | 2.76 | 0.4224523878 |  |
| 293 | 18 | G | G | C | 1.43 | 0.6978395072 |  |
| 293 | 18 | G | G | G | 95.19 | 0.0000000000 | \* |
| 293 | 18 | G | G | T | 0.61 | 0.8562182508 |  |
| 294 | 19 | T | T | A | 0.00 | 0.8636363636 |  |
| 294 | 19 | T | T | C | 1.60 | 0.6441321972 |  |
| 294 | 19 | T | T | G | 4.63 | 0.1241361211 |  |
| 294 | 19 | T | T | T | 93.77 | 0.0000000000 | \* |
| 295 | 20 | T | T | A | 1.64 | 0.6218403359 |  |
| 295 | 20 | T | T | C | 2.69 | 0.3007959432 |  |
| 295 | 20 | T | T | G | 0.00 | 0.8791208791 |  |
| 295 | 20 | T | T | T | 95.67 | 0.0000000000 | \* |
| 296 | 21 | T | T | A | 0.00 | 0.8636363636 |  |
| 296 | 21 | T | T | C | 3.00 | 0.2278401255 |  |
| 296 | 21 | T | T | G | 1.71 | 0.5009387719 |  |
| 296 | 21 | T | T | T | 95.29 | 0.0000000000 | \* |
| 297 | 22 | G | G | A | 1.64 | 0.6226766153 |  |
| 297 | 22 | G | G | C | 0.00 | 0.8928571429 |  |
| 297 | 22 | G | G | G | 97.43 | 0.0000000000 | \* |
| 297 | 22 | G | G | T | 0.94 | 0.7878786385 |  |
| 298 | 23 | T | T | A | 0.00 | 0.8636363636 |  |
| 298 | 23 | T | T | C | 3.11 | 0.2048609482 |  |
| 298 | 23 | T | T | G | 1.73 | 0.4975552141 |  |
| 298 | 23 | T | T | T | 95.16 | 0.0000000000 | \* |
| 299 | 24 | G | G | A | 1.86 | 0.5808825173 |  |
| 299 | 24 | G | G | C | 1.14 | 0.7820193618 |  |
| 299 | 24 | G | G | G | 95.71 | 0.0000000000 | \* |
| 299 | 24 | G | G | T | 1.29 | 0.6994490597 |  |
| 300 | 25 | C | C | A | 1.59 | 0.6312139464 |  |
| 300 | 25 | C | C | C | 93.45 | 0.0000000000 | \* |
| 300 | 25 | C | C | G | 1.95 | 0.4528054357 |  |
| 300 | 25 | C | C | T | 3.01 | 0.2990373254 |  |
| 301 | 26 | A | A | A | 89.10 | 0.0000000000 | \* |
| 301 | 26 | A | A | C | 3.63 | 0.1227153218 |  |
| 301 | 26 | A | A | G | 4.27 | 0.1489054414 |  |
| 301 | 26 | A | A | T | 2.99 | 0.3020551897 |  |
| 302 | 27 | C | C | A | 2.24 | 0.5102266823 |  |
| 302 | 27 | C | C | C | 95.81 | 0.0000000000 | \* |
| 302 | 27 | C | C | G | 0.00 | 0.8791208791 |  |
| 302 | 27 | C | C | T | 1.94 | 0.5271756269 |  |
| 303 | 28 | T | T | A | 1.59 | 0.6322927242 |  |
| 303 | 28 | T | T | C | 4.37 | 0.0554762790 |  |
| 303 | 28 | T | T | G | 0.40 | 0.8149687051 |  |
| 303 | 28 | T | T | T | 93.65 | 0.0000000000 | \* |
| 304 | 29 | A | A | A | 95.88 | 0.0000000000 | \* |
| 304 | 29 | A | A | C | 1.37 | 0.7160738716 |  |
| 304 | 29 | A | A | G | 1.03 | 0.6603812686 |  |
| 304 | 29 | A | A | T | 1.72 | 0.5849724242 |  |
| 305 | 30 | C | C | A | 1.88 | 0.5769703819 |  |
| 305 | 30 | C | C | C | 93.11 | 0.0000000000 | \* |
| 305 | 30 | C | C | G | 1.88 | 0.4667095570 |  |
| 305 | 30 | C | C | T | 3.13 | 0.2786931197 |  |
| 306 | 31 | A | A | A | 89.10 | 0.0000000000 | \* |
| 306 | 31 | A | A | C | 2.78 | 0.2780567978 |  |
| 306 | 31 | A | A | G | 5.34 | 0.0862247964 |  |
| 306 | 31 | A | A | T | 2.78 | 0.3410749841 |  |
| 307 | 32 | C | C | A | 1.23 | 0.7010748251 |  |
| 307 | 32 | C | C | C | 95.26 | 0.0000000000 | \* |
| 307 | 32 | C | C | G | 1.75 | 0.4923920870 |  |
| 307 | 32 | C | C | T | 1.75 | 0.5755350582 |  |
| 308 | 33 | G | G | A | 2.70 | 0.4321546601 |  |
| 308 | 33 | G | G | C | 1.80 | 0.5747642845 |  |
| 308 | 33 | G | G | G | 93.92 | 0.0000000000 | \* |
| 308 | 33 | G | G | T | 1.58 | 0.6222958014 |  |
| 309 | 34 | G | G | A | 1.38 | 0.6712327745 |  |
| 309 | 34 | G | G | C | 1.85 | 0.5596071756 |  |
| 309 | 34 | G | G | G | 96.31 | 0.0000000000 | \* |
| 309 | 34 | G | G | T | 0.46 | 0.8812015526 |  |
| 310 | 35 | G | G | A | 3.25 | 0.3503521819 |  |
| 310 | 35 | G | G | C | 1.34 | 0.7270799281 |  |
| 310 | 35 | G | G | G | 94.07 | 0.0000000000 | \* |
| 310 | 35 | G | G | T | 1.34 | 0.6855407996 |  |
| 311 | 36 | C | C | A | 3.88 | 0.2717952920 |  |
| 311 | 36 | C | C | C | 92.89 | 0.0000000000 | \* |
| 311 | 36 | C | C | G | 1.29 | 0.5965079889 |  |
| 311 | 36 | C | C | T | 1.94 | 0.5280671067 |  |
| 312 | 37 | A | A | A | 91.90 | 0.0000000000 | \* |
| 312 | 37 | A | A | C | 2.77 | 0.2807284960 |  |
| 312 | 37 | A | A | G | 1.19 | 0.6223430684 |  |
| 312 | 37 | A | A | T | 4.15 | 0.1485822841 |  |
| 313 | 38 | C | C | A | 3.01 | 0.3847853400 |  |
| 313 | 38 | C | C | C | 94.12 | 0.0000000000 | \* |
| 313 | 38 | C | C | G | 1.23 | 0.6113497082 |  |
| 313 | 38 | C | C | T | 1.64 | 0.6051023964 |  |
| 314 | 39 | C | C | A | 7.32 | 0.0582607828 |  |
| 314 | 39 | C | C | C | 89.39 | 0.0000000000 | \* |
| 314 | 39 | C | C | G | 1.05 | 0.6565631790 |  |
| 314 | 39 | C | C | T | 2.24 | 0.4547154672 |  |
| 315 | 40 | C | C | A | 4.65 | 0.1964178174 |  |
| 315 | 40 | C | C | C | 91.19 | 0.0000000000 | \* |
| 315 | 40 | C | C | G | 1.92 | 0.4575734273 |  |
| 315 | 40 | C | C | T | 2.24 | 0.4543814378 |  |
| 316 | 41 | T | T | A | 3.30 | 0.3429515783 |  |
| 316 | 41 | T | T | C | 3.59 | 0.1279398317 |  |
| 316 | 41 | T | T | G | 2.30 | 0.3867377081 |  |
| 316 | 41 | T | T | T | 90.80 | 0.0000000000 | \* |
| 317 | 42 | G | G | A | 7.03 | 0.0668034696 |  |
| 317 | 42 | G | G | C | 0.26 | 0.8921230298 |  |
| 317 | 42 | G | G | G | 91.43 | 0.0000000000 | \* |
| 317 | 42 | G | G | T | 1.28 | 0.7012742500 |  |
| 318 | 43 | A | A | A | 92.41 | 0.0000000000 | \* |
| 318 | 43 | A | A | C | 1.16 | 0.7780050711 |  |
| 318 | 43 | A | A | G | 2.96 | 0.2840264550 |  |
| 318 | 43 | A | A | T | 3.47 | 0.2267815956 |  |
| 319 | 44 | C | C | A | 8.79 | 0.0289524242 |  |
| 319 | 44 | C | C | C | 91.21 | 0.0000000000 | \* |
| 319 | 44 | C | C | G | 0.00 | 0.8791208791 |  |
| 319 | 44 | C | C | T | 0.00 | 0.9139784946 |  |
| 320 | 45 | C | C | A | 10.17 | 0.0147067724 |  |
| 320 | 45 | C | C | C | 82.20 | 0.0000000000 | \* |
| 320 | 45 | C | C | G | 4.92 | 0.1074362839 |  |
| 320 | 45 | C | C | T | 2.71 | 0.3538452470 |  |
| 321 | 46 | G | G | A | 6.69 | 0.0782681076 |  |
| 321 | 46 | G | G | C | 3.04 | 0.2190473037 |  |
| 321 | 46 | G | G | G | 89.25 | 0.0000000000 | \* |
| 321 | 46 | G | G | T | 1.01 | 0.7689089957 |  |
| 322 | 47 | A | A | A | 94.10 | 0.0000000000 | \* |
| 322 | 47 | A | A | C | 0.60 | 0.8769579993 |  |
| 322 | 47 | A | A | G | 0.84 | 0.7074233986 |  |
| 322 | 47 | A | A | T | 4.45 | 0.1221030675 |  |
| 323 | 48 | C | C | A | 5.39 | 0.1420254951 |  |
| 323 | 48 | C | C | C | 90.48 | 0.0000000000 | \* |
| 323 | 48 | C | C | G | 1.62 | 0.5223580895 |  |
| 323 | 48 | C | C | T | 2.51 | 0.3943687716 |  |
| 324 | 49 | G | G | A | 8.80 | 0.0287558152 |  |
| 324 | 49 | G | G | C | 1.87 | 0.5526049902 |  |
| 324 | 49 | G | G | G | 86.93 | 0.0000000000 | \* |
| 324 | 49 | G | G | T | 2.40 | 0.4189307283 |  |
| 325 | 50 | G | G | A | 1.99 | 0.5553350658 |  |
| 325 | 50 | G | G | C | 2.15 | 0.4587361353 |  |
| 325 | 50 | G | G | G | 94.79 | 0.0000000000 | \* |
| 325 | 50 | G | G | T | 1.07 | 0.7541467627 |  |
| 326 | 51 | C | C | A | 3.60 | 0.3045648072 |  |
| 326 | 51 | C | C | C | 93.91 | 0.0000000000 | \* |
| 326 | 51 | C | C | G | 2.49 | 0.3537470106 |  |
| 326 | 51 | C | C | T | 0.00 | 0.9139784946 |  |
| 327 | 52 | A | A | A | 92.43 | 0.0000000000 | \* |
| 327 | 52 | A | A | C | 1.26 | 0.7495983479 |  |
| 327 | 52 | A | A | G | 2.21 | 0.4029736757 |  |
| 327 | 52 | A | A | T | 4.10 | 0.1533527507 |  |
| 328 | 53 | A | A | A | 93.26 | 0.0000000000 | \* |
| 328 | 53 | A | A | C | 1.87 | 0.5505609507 |  |
| 328 | 53 | A | A | G | 2.06 | 0.4306974882 |  |
| 328 | 53 | A | A | T | 2.81 | 0.3351490390 |  |
| 329 | 54 | G | G | A | 3.59 | 0.3061213980 |  |
| 329 | 54 | G | G | C | 0.00 | 0.8928571429 |  |
| 329 | 54 | G | G | G | 96.41 | 0.0000000000 | \* |
| 329 | 54 | G | G | T | 0.00 | 0.9139784946 |  |
| 330 | 55 | A | A | A | 93.19 | 0.0000000000 | \* |
| 330 | 55 | A | A | C | 0.00 | 0.8928571429 |  |
| 330 | 55 | A | A | G | 3.92 | 0.1779181914 |  |
| 330 | 55 | A | A | T | 2.90 | 0.3190234427 |  |
| 331 | 56 | A | A | A | 93.28 | 0.0000000000 | \* |
| 331 | 56 | A | A | C | 0.20 | 0.8925841397 |  |
| 331 | 56 | A | A | G | 1.78 | 0.4872691515 |  |
| 331 | 56 | A | A | T | 4.74 | 0.1007521583 |  |
| 332 | 57 | G | G | A | 1.74 | 0.6032418036 |  |
| 332 | 57 | G | G | C | 1.16 | 0.7777118080 |  |
| 332 | 57 | G | G | G | 92.17 | 0.0000000000 | \* |
| 332 | 57 | G | G | T | 4.93 | 0.0890365307 |  |
| 333 | 58 | T | T | A | 1.18 | 0.7103667138 |  |
| 333 | 58 | T | T | C | 2.36 | 0.3927492424 |  |
| 333 | 58 | T | T | G | 0.59 | 0.7700227604 |  |
| 333 | 58 | T | T | T | 95.87 | 0.0000000000 | \* |
| 334 | 59 | T | T | A | 17.50 | 0.0003501525 | \* |
| 334 | 59 | T | T | C | 2.88 | 0.2529980386 |  |
| 334 | 59 | T | T | G | 2.50 | 0.3526153339 |  |
| 334 | 59 | T | T | T | 77.12 | 0.0000000000 | \* |
| 335 | 60 | C | C | A | 1.93 | 0.5678158159 |  |
| 335 | 60 | C | C | C | 91.71 | 0.0000000000 | \* |
| 335 | 60 | C | C | G | 5.39 | 0.0838837596 |  |
| 335 | 60 | C | C | T | 0.96 | 0.7812598727 |  |
| 336 | 61 | G | G | A | 4.90 | 0.1762482467 |  |
| 336 | 61 | G | G | C | 1.80 | 0.5739705242 |  |
| 336 | 61 | G | G | G | 91.24 | 0.0000000000 | \* |
| 336 | 61 | G | G | T | 2.06 | 0.4977388557 |  |
| 337 | 62 | A | A | A | 94.33 | 0.0000000000 | \* |
| 337 | 62 | A | A | C | 1.11 | 0.7912223040 |  |
| 337 | 62 | A | A | G | 2.07 | 0.4278719487 |  |
| 337 | 62 | A | A | T | 2.49 | 0.3994465090 |  |
| 338 | 63 | C | C | A | 3.89 | 0.2701716880 |  |
| 338 | 63 | C | C | C | 94.87 | 0.0000000000 | \* |
| 338 | 63 | C | C | G | 0.00 | 0.8791208791 |  |
| 338 | 63 | C | C | T | 1.24 | 0.7117132109 |  |
| 339 | 64 | A | A | A | 89.07 | 0.0000000000 | \* |
| 339 | 64 | A | A | C | 1.91 | 0.5369719419 |  |
| 339 | 64 | A | A | G | 6.28 | 0.0526888652 |  |
| 339 | 64 | A | A | T | 2.73 | 0.3498605028 |  |
| 340 | 65 | G | G | A | 0.52 | 0.8207986486 |  |
| 340 | 65 | G | G | C | 3.31 | 0.1697159262 |  |
| 340 | 65 | G | G | G | 95.30 | 0.0000000000 | \* |
| 340 | 65 | G | G | T | 0.87 | 0.8029340650 |  |
| 341 | 66 | C | C | A | 0.61 | 0.8076604301 |  |
| 341 | 66 | C | C | C | 90.37 | 0.0000000000 | \* |
| 341 | 66 | C | C | G | 3.89 | 0.1801269532 |  |
| 341 | 66 | C | C | T | 5.12 | 0.0780073557 |  |
| 342 | 67 | T | T | A | 0.00 | 0.8636363636 |  |
| 342 | 67 | T | T | C | 3.18 | 0.1917674390 |  |
| 342 | 67 | T | T | G | 0.00 | 0.8791208791 |  |
| 342 | 67 | T | T | T | 96.82 | 0.0000000000 | \* |
| 343 | 68 | C | C | A | 3.43 | 0.3263249933 |  |
| 343 | 68 | C | C | C | 94.22 | 0.0000000000 | \* |
| 343 | 68 | C | C | G | 0.00 | 0.8791208791 |  |
| 343 | 68 | C | C | T | 2.35 | 0.4308370773 |  |
| 344 | 69 | C | C | A | 3.12 | 0.3686181366 |  |
| 344 | 69 | C | C | C | 91.91 | 0.0000000000 | \* |
| 344 | 69 | C | C | G | 0.57 | 0.7753376414 |  |
| 344 | 69 | C | C | T | 4.40 | 0.1266040260 |  |
| 345 | 70 | C | C | A | 4.13 | 0.2448025138 |  |
| 345 | 70 | C | C | C | 89.13 | 0.0000000000 | \* |
| 345 | 70 | C | C | G | 5.22 | 0.0919601376 |  |
| 345 | 70 | C | C | T | 1.52 | 0.6368527536 |  |
| 346 | 71 | G | G | A | 5.30 | 0.1475964732 |  |
| 346 | 71 | G | G | C | 3.69 | 0.1160187370 |  |
| 346 | 71 | G | G | G | 90.32 | 0.0000000000 | \* |
| 346 | 71 | G | G | T | 0.69 | 0.8415204444 |  |
| 347 | 72 | C | C | A | 5.28 | 0.1485821686 |  |
| 347 | 72 | C | C | C | 88.41 | 0.0000000000 | \* |
| 347 | 72 | C | C | G | 4.67 | 0.1214948481 |  |
| 347 | 72 | C | C | T | 1.63 | 0.6092122583 |  |
| 348 | 73 | G | G | A | 6.92 | 0.0703969941 |  |
| 348 | 73 | G | G | C | 3.58 | 0.1295229814 |  |
| 348 | 73 | G | G | G | 89.02 | 0.0000000000 | \* |
| 348 | 73 | G | G | T | 0.48 | 0.8788960168 |  |
| 349 | 74 | A | A | A | 92.30 | 0.0000000000 | \* |
| 349 | 74 | A | A | C | 3.05 | 0.2175946526 |  |
| 349 | 74 | A | A | G | 0.80 | 0.7173711808 |  |
| 349 | 74 | A | A | T | 3.85 | 0.1795626566 |  |
| 350 | 75 | C | C | A | 0.00 | 0.8636363636 |  |
| 350 | 75 | C | C | C | 94.84 | 0.0000000000 | \* |
| 350 | 75 | C | C | G | 1.16 | 0.6274649043 |  |
| 350 | 75 | C | C | T | 3.99 | 0.1642505890 |  |

## For use in R

If you want to work with the results in R, here is output that you can copy and paste in your terminal to get:

The base information:

```
structure(list(focal.base = c("A", "C", "G", "T"), avg.percsignal = c(92.3084168713404, 
92.3175524034474, 93.5857075511838, 92.4613507459454), avg.areasignal = c(553.617647058824, 
573.522388059701, 575.875, 667.931818181818), crit.perc.area = c(10.9466438001523, 
5.79048223777924, 9.37642676962956, 7.99169025743598), mu = c(3.27164225423307, 
2.37298813875003, 2.54963608704538, 2.59720069562305), fillibens = c(0.969154971851743, 
0.99283522658871, 0.989942933692905, 0.997562892735817)), .Names = c("focal.base", 
"avg.percsignal", "avg.areasignal", "crit.perc.area", "mu", "fillibens"
), row.names = c(NA, -4L), class = "data.frame")
```

the data.frame that contains information on the guide region:

```
structure(list(A.area = c(494, 576, 10, 995, 22, 36, 24, 23, 
15, 18, 480, 12, 1015, 28, 53, 27, 27, 27, 0, 11, 0, 14, 0, 13, 
9, 417, 15, 12, 558, 12, 417, 7, 12, 9, 17, 18, 465, 22, 49, 
29, 23, 55, 718, 55, 60, 33, 782, 30, 33, 13, 13, 293, 498, 15, 
547, 472, 6, 6, 91, 10, 19, 682, 22, 326, 3, 3, 0, 19, 22, 19, 
23, 26, 29, 575, 0), C.area = c(10, 16, 7, 10, 581, 11, 13, 7, 
389, 669, 10, 17, 10, 601, 528, 14, 14, 14, 10, 18, 21, 0, 18, 
8, 528, 17, 641, 33, 8, 595, 13, 543, 8, 12, 7, 431, 14, 688, 
598, 569, 25, 2, 9, 571, 485, 15, 5, 504, 7, 14, 339, 4, 10, 
0, 0, 1, 4, 12, 15, 476, 7, 8, 536, 7, 19, 441, 22, 522, 648, 
410, 16, 435, 15, 19, 570), G.area = c(3, 9, 479, 8, 9, 16, 1094, 
704, 8, 23, 10, 505, 7, 12, 8, 581, 10, 930, 29, 0, 12, 833, 
10, 670, 11, 20, 0, 3, 6, 12, 25, 10, 417, 626, 492, 6, 6, 9, 
7, 12, 16, 715, 23, 0, 29, 440, 7, 9, 326, 618, 9, 7, 11, 403, 
23, 9, 318, 3, 13, 28, 354, 15, 0, 23, 547, 19, 0, 0, 4, 24, 
392, 23, 373, 5, 7), T.area = c(13, 15, 6, 34, 12, 717, 13, 12, 
9, 0, 17, 10, 35, 13, 9, 9, 470, 6, 587, 641, 667, 8, 550, 9, 
17, 14, 13, 708, 10, 20, 13, 10, 7, 3, 7, 9, 21, 12, 15, 14, 
632, 10, 27, 0, 16, 5, 37, 14, 9, 7, 0, 13, 15, 0, 17, 24, 17, 
488, 401, 5, 8, 18, 7, 10, 5, 25, 669, 13, 31, 7, 3, 8, 2, 24, 
24), Tot.area = c(520, 616, 502, 1047, 624, 780, 1144, 746, 421, 
710, 517, 544, 1067, 654, 598, 631, 521, 977, 626, 670, 700, 
855, 578, 700, 565, 468, 669, 756, 582, 639, 468, 570, 444, 650, 
523, 464, 506, 731, 669, 624, 696, 782, 777, 626, 590, 493, 831, 
557, 375, 652, 361, 317, 534, 418, 587, 506, 345, 509, 520, 519, 
388, 723, 565, 366, 574, 488, 691, 554, 705, 460, 434, 492, 419, 
623, 601), A.perc = c(95, 93.5064935064935, 1.99203187250996, 
95.0334288443171, 3.52564102564103, 4.61538461538461, 2.0979020979021, 
3.08310991957105, 3.56294536817102, 2.53521126760563, 92.8433268858801, 
2.20588235294118, 95.1265229615745, 4.28134556574924, 8.8628762541806, 
4.27892234548336, 5.18234165067178, 2.76356192425793, 0, 1.64179104477612, 
0, 1.6374269005848, 0, 1.85714285714286, 1.5929203539823, 89.1025641025641, 
2.24215246636771, 1.58730158730159, 95.8762886597938, 1.87793427230047, 
89.1025641025641, 1.2280701754386, 2.7027027027027, 1.38461538461538, 
3.25047801147228, 3.87931034482759, 91.897233201581, 3.00957592339261, 
7.32436472346786, 4.6474358974359, 3.30459770114943, 7.03324808184143, 
92.4066924066924, 8.78594249201278, 10.1694915254237, 6.69371196754564, 
94.1034897713598, 5.38599640933573, 8.8, 1.99386503067485, 3.601108033241, 
92.4290220820189, 93.2584269662921, 3.58851674641148, 93.1856899488927, 
93.2806324110672, 1.73913043478261, 1.17878192534381, 17.5, 1.92678227360308, 
4.89690721649485, 94.32918395574, 3.89380530973451, 89.0710382513661, 
0.522648083623693, 0.614754098360656, 0, 3.42960288808664, 3.12056737588652, 
4.1304347826087, 5.29953917050691, 5.28455284552845, 6.92124105011933, 
92.2953451043339, 0), C.perc = c(1.92307692307692, 2.5974025974026, 
1.39442231075697, 0.955109837631328, 93.1089743589744, 1.41025641025641, 
1.13636363636364, 0.938337801608579, 92.3990498812352, 94.2253521126761, 
1.93423597678917, 3.125, 0.937207122774133, 91.8960244648318, 
88.2943143812709, 2.21870047543582, 2.68714011516315, 1.43295803480041, 
1.59744408945687, 2.6865671641791, 3, 0, 3.11418685121107, 1.14285714285714, 
93.4513274336283, 3.63247863247863, 95.8146487294469, 4.36507936507936, 
1.3745704467354, 93.1142410015649, 2.77777777777778, 95.2631578947368, 
1.8018018018018, 1.84615384615385, 1.33843212237094, 92.8879310344828, 
2.76679841897233, 94.1176470588235, 89.3871449925262, 91.1858974358974, 
3.59195402298851, 0.255754475703325, 1.15830115830116, 91.2140575079872, 
82.2033898305085, 3.04259634888438, 0.601684717208183, 90.4847396768402, 
1.86666666666667, 2.14723926380368, 93.9058171745152, 1.26182965299685, 
1.87265917602996, 0, 0, 0.197628458498024, 1.15942028985507, 
2.35756385068762, 2.88461538461538, 91.7148362235067, 1.80412371134021, 
1.10650069156293, 94.8672566371681, 1.91256830601093, 3.31010452961672, 
90.3688524590164, 3.18379160636758, 94.2238267148014, 91.9148936170213, 
89.1304347826087, 3.68663594470046, 88.4146341463415, 3.5799522673031, 
3.04975922953451, 94.8419301164725), G.perc = c(0.576923076923077, 
1.46103896103896, 95.4183266932271, 0.764087870105062, 1.44230769230769, 
2.05128205128205, 95.6293706293706, 94.3699731903485, 1.90023752969121, 
3.23943661971831, 1.93423597678917, 92.8308823529412, 0.656044985941893, 
1.8348623853211, 1.33779264214047, 92.0760697305864, 1.91938579654511, 
95.1893551688843, 4.63258785942492, 0, 1.71428571428571, 97.4269005847953, 
1.73010380622837, 95.7142857142857, 1.94690265486726, 4.27350427350427, 
0, 0.396825396825397, 1.03092783505155, 1.87793427230047, 5.34188034188034, 
1.75438596491228, 93.9189189189189, 96.3076923076923, 94.0726577437859, 
1.29310344827586, 1.18577075098814, 1.2311901504788, 1.04633781763827, 
1.92307692307692, 2.29885057471264, 91.4322250639386, 2.96010296010296, 
0, 4.91525423728814, 89.2494929006085, 0.842358604091456, 1.61579892280072, 
86.9333333333333, 94.7852760736196, 2.49307479224377, 2.20820189274448, 
2.05992509363296, 96.4114832535885, 3.91822827938671, 1.77865612648221, 
92.1739130434783, 0.589390962671906, 2.5, 5.39499036608863, 91.2371134020619, 
2.0746887966805, 0, 6.28415300546448, 95.2961672473868, 3.89344262295082, 
0, 0, 0.567375886524823, 5.21739130434783, 90.3225806451613, 
4.67479674796748, 89.0214797136038, 0.802568218298555, 1.16472545757072
), T.perc = c(2.5, 2.43506493506493, 1.19521912350598, 3.24737344794651, 
1.92307692307692, 91.9230769230769, 1.13636363636364, 1.60857908847185, 
2.13776722090261, 0, 3.28820116054159, 1.83823529411765, 3.28022492970947, 
1.98776758409786, 1.50501672240803, 1.42630744849445, 90.21113243762, 
0.614124872057318, 93.7699680511182, 95.6716417910448, 95.2857142857143, 
0.935672514619883, 95.1557093425606, 1.28571428571429, 3.00884955752212, 
2.99145299145299, 1.94319880418535, 93.6507936507936, 1.71821305841924, 
3.12989045383412, 2.77777777777778, 1.75438596491228, 1.57657657657658, 
0.461538461538462, 1.33843212237094, 1.93965517241379, 4.1501976284585, 
1.64158686730506, 2.24215246636771, 2.24358974358974, 90.8045977011494, 
1.27877237851662, 3.47490347490348, 0, 2.71186440677966, 1.01419878296146, 
4.45246690734055, 2.51346499102334, 2.4, 1.07361963190184, 0, 
4.10094637223975, 2.80898876404494, 0, 2.89608177172061, 4.74308300395257, 
4.92753623188406, 95.8742632612967, 77.1153846153846, 0.963391136801541, 
2.06185567010309, 2.4896265560166, 1.23893805309735, 2.73224043715847, 
0.871080139372822, 5.12295081967213, 96.8162083936324, 2.34657039711191, 
4.39716312056738, 1.52173913043478, 0.691244239631336, 1.6260162601626, 
0.477326968973747, 3.85232744783307, 3.99334442595674), base.call = c("A", 
"A", "G", "A", "C", "T", "G", "G", "C", "C", "A", "G", "A", "C", 
"C", "G", "T", "G", "T", "T", "T", "G", "T", "G", "C", "A", "C", 
"T", "A", "C", "A", "C", "G", "G", "G", "C", "A", "C", "C", "C", 
"T", "G", "A", "C", "C", "G", "A", "C", "G", "G", "C", "A", "A", 
"G", "A", "A", "G", "T", "T", "C", "G", "A", "C", "A", "G", "C", 
"T", "C", "C", "C", "G", "C", "G", "A", "C"), index = 276:350, 
    guide.seq = c("A", "A", "G", "A", "C", "T", "G", "G", "C", 
    "C", "A", "G", "A", "C", "C", "G", "T", "G", "T", "T", "T", 
    "G", "T", "G", "C", "A", "C", "T", "A", "C", "A", "C", "G", 
    "G", "G", "C", "A", "C", "C", "C", "T", "G", "A", "C", "C", 
    "G", "A", "C", "G", "G", "C", "A", "A", "G", "A", "A", "G", 
    "T", "T", "C", "G", "A", "C", "A", "G", "C", "T", "C", "C", 
    "C", "G", "C", "G", "A", "C"), T.pval = c(0.397231457856343, 
    0.411234346601611, 0.723091019733231, 0.260023524047133, 
    0.532246708004145, 0, 0.738245588546081, 0.61382127846974, 
    0.479359628944426, 0.913978494580568, 0.253779151663234, 
    0.553855866463238, 0.254989304749155, 0.516022742549487, 
    0.641297810392822, 0.662228798313576, 0, 0.85621825083822, 
    0, 0, 0, 0.787878638541858, 0, 0.699449059717089, 0.299037325412762, 
    0.302055189715575, 0.527175626936664, 0, 0.584972424240835, 
    0.278693119666762, 0.341074984075509, 0.575535058150625, 
    0.622295801356202, 0.881201552602946, 0.685540799617681, 
    0.528067106737919, 0.148582284076679, 0.605102396416757, 
    0.454715467203477, 0.454381437772895, 0, 0.701274250028756, 
    0.226781595621325, 0.913978494580568, 0.353845247034423, 
    0.768908995732043, 0.122103067482479, 0.39436877155921, 0.418930728265314, 
    0.754146762678869, 0.913978494580568, 0.153352750671244, 
    0.335149039005113, 0.913978494580568, 0.319023442673563, 
    0.100752158346873, 0.0890365306905924, 0, 0, 0.781259872741778, 
    0.497738855699441, 0.399446508953019, 0.71171321088199, 0.349860502781521, 
    0.802934065047812, 0.0780073557029362, 0, 0.430837077253893, 
    0.126604026048206, 0.636852753568375, 0.841520444351691, 
    0.609212258269193, 0.878896016793033, 0.179562656637713, 
    0.164250588960243), C.pval = c(0.533402177844488, 0.324210719497976, 
    0.709933827131361, 0.825500602977646, 0, 0.704991475011605, 
    0.783688756732056, 0.828869395756559, 0, 0, 0.529616017319319, 
    0.202781994375706, 0.829093285829592, 0, 0, 0.435780714943823, 
    0.300649255063711, 0.697839507150857, 0.644132197191233, 
    0.300795943189278, 0.227840125501113, 0.892857142856464, 
    0.204860948164015, 0.782019361802523, 0, 0.122715321839276, 
    0, 0.0554762789547468, 0.716073871624271, 0, 0.278056797800939, 
    0, 0.574764284487437, 0.559607175570143, 0.727079928078661, 
    0, 0.280728495993157, 0, 0, 0, 0.127939831744001, 0.892123029840068, 
    0.778005071130535, 0, 0, 0.21904730366395, 0.876957999307982, 
    0, 0.552604990196244, 0.458736135340343, 0, 0.749598347930075, 
    0.550560950684156, 0.892857142856464, 0.892857142856464, 
    0.892584139696054, 0.777711807978341, 0.392749242443636, 
    0.252998038634562, 0, 0.573970524207533, 0.791222303971727, 
    0, 0.536971941915289, 0.169715926247225, 0, 0.191767439026642, 
    0, 0, 0, 0.116018736967301, 0, 0.129522981415506, 0.217594652595829, 
    0), G.pval = c(0.773036926118535, 0.55719917668374, 0, 0.726975274308417, 
    0.56150961405013, 0.432358319572866, 0, 0, 0.462179048902481, 
    0.248350371380451, 0.455335635310993, 0, 0.753749954411796, 
    0.475550793025891, 0.58590656559177, 0, 0.458315432311607, 
    0, 0.124136121146201, 0.879120879120835, 0.500938771914442, 
    0, 0.497555214055604, 0, 0.452805435691161, 0.148905441374191, 
    0.879120879120835, 0.814968705051215, 0.660381268565092, 
    0.466709556997774, 0.086224796353104, 0.492392087002588, 
    0, 0, 0, 0.596507988942114, 0.62234306835923, 0.611349708246198, 
    0.656563178951379, 0.45757342731016, 0.386737708056653, 0, 
    0.284026455008967, 0.879120879120835, 0.107436283933514, 
    0, 0.707423398605889, 0.522358089461755, 0, 0, 0.353747010615113, 
    0.402973675684304, 0.430697488229117, 0, 0.17791819142362, 
    0.487269151523825, 0, 0.770022760368736, 0.35261533389156, 
    0.0838837596109125, 0, 0.427871948679742, 0.879120879120835, 
    0.0526888651846131, 0, 0.180126953161887, 0.879120879120835, 
    0.879120879120835, 0.775337641429497, 0.0919601375703878, 
    0, 0.121494848077647, 0, 0.717371180785335, 0.627464904340898
    ), A.pval = c(0, 0, 0.555674634640398, 0, 0.313991154645335, 
    0.199152537721903, 0.53621194988451, 0.374016447328302, 0.309302518908323, 
    0.459620666345016, 0, 0.516698499947535, 0, 0.229710607106627, 
    0.0278921201894914, 0.229946442319394, 0.155466329198054, 
    0.422452387822647, 0.863636363636362, 0.62184033586988, 0.863636363636362, 
    0.62267661534104, 0.863636363636362, 0.580882517279385, 0.63121394639898, 
    0, 0.510226682257052, 0.632292724150608, 0, 0.576970381866964, 
    0, 0.701074825069745, 0.432154660135855, 0.671232774515324, 
    0.35035218188387, 0.271795292005316, 0, 0.384785339959347, 
    0.0582607828317072, 0.196417817357698, 0.342951578300291, 
    0.0668034696068979, 0, 0.0289524242308878, 0.0147067724450893, 
    0.078268107606306, 0, 0.142025495132119, 0.0287558151630469, 
    0.555335065804187, 0.304564807195984, 0, 0, 0.306121398032549, 
    0, 0, 0.603241803597411, 0.710366713826131, 0.000350152503322598, 
    0.567815815912239, 0.176248246715156, 0, 0.270171688027014, 
    0, 0.820798648564676, 0.807660430124918, 0.863636363636362, 
    0.326324993262877, 0.368618136620164, 0.244802513755481, 
    0.147596473206323, 0.148582168631439, 0.0703969940643694, 
    0, 0.863636363636362), guide.position = 1:75), .Names = c("A.area", 
"C.area", "G.area", "T.area", "Tot.area", "A.perc", "C.perc", 
"G.perc", "T.perc", "base.call", "index", "guide.seq", "T.pval", 
"C.pval", "G.pval", "A.pval", "guide.position"), row.names = 276:350, class = "data.frame")
```

*Report generated using EditR v1.0.8*
