# Supplementary material for: Mechanistic and genetic basis of single-strand templated repair at Cas12a-induced DNA breaks in Chlamydomonas reinhardtii
Source: Nat Commun. 2021 Nov 19;12:6751. doi: 10.1038/s41467-021-27004-1 (PMC8604939; doi:10.1038/s41467-021-27004-1)
Supplement: Supplementary file 22 — Source Data [file 41467_2021_27004_MOESM22_ESM.zip › Source Data/EditR analysis/EditR outputs/Antisense/rep1_ssODN_antisense_-16.html]

EditR v1.0.8 report


# EditR v1.0.8 report

- Data QA
  - Filtering data
  - Percent noise peak area
  - Base information
- Predicted editing
  - Editing bar plot
  - Editing table plot
  - Table of editing results
- For use in R

## Data QA

### Filtering data

What the data looked like prefiltering:

and the post filtering signal / noise plot:

### Percent noise peak area

### Base information

Here’s information about the signal of each base, the critical percent value where any higher value would be called as significant, and Filliben’s correlation for how well the noise was modelled by the zero adjusted gamma distribution.

| Base | Average percent signal | Average peak area | Critical percent value | model mu | Fillibens correlation |
| --- | --- | --- | --- | --- | --- |
| A | 94.93608 | 221.2353 | 7.606764 | 2.388234 | 0.9429688 |
| C | 93.16993 | 233.1176 | 4.614143 | 1.959339 | 0.9964634 |
| G | 94.44410 | 211.1250 | 5.534134 | 1.781770 | 0.9940690 |
| T | 94.64809 | 243.6047 | 6.451728 | 2.544215 | 0.9891264 |

## Predicted editing

### Editing bar plot

### Editing table plot

### Table of editing results


Here’s the entire guide region

| Sanger position | Guide position | Guide sequence | Sanger base call | Focal base | Focal base peak area | p value |  |
| --- | --- | --- | --- | --- | --- | --- | --- |
| 278 | 1 | A | A | A | 95.35 | 0.0000000000 | \* |
| 278 | 1 | A | A | C | 2.33 | 0.2651105419 |  |
| 278 | 1 | A | A | G | 2.33 | 0.2334708939 |  |
| 278 | 1 | A | A | T | 0.00 | 0.8829787234 |  |
| 279 | 2 | A | A | A | 95.38 | 0.0000000000 | \* |
| 279 | 2 | A | A | C | 0.84 | 0.8228664262 |  |
| 279 | 2 | A | A | G | 1.26 | 0.5355196799 |  |
| 279 | 2 | A | A | T | 2.52 | 0.3866177231 |  |
| 280 | 3 | G | G | A | 3.77 | 0.1491420214 |  |
| 280 | 3 | G | G | C | 1.89 | 0.4186224025 |  |
| 280 | 3 | G | G | G | 93.71 | 0.0000000000 | \* |
| 280 | 3 | G | G | T | 0.63 | 0.8629272387 |  |
| 281 | 4 | A | A | A | 96.56 | 0.0000000000 | \* |
| 281 | 4 | A | A | C | 1.15 | 0.7254250275 |  |
| 281 | 4 | A | A | G | 0.92 | 0.6626172734 |  |
| 281 | 4 | A | A | T | 1.38 | 0.7188600397 |  |
| 282 | 5 | C | C | A | 3.59 | 0.1683611898 |  |
| 282 | 5 | C | C | C | 92.43 | 0.0000000000 | \* |
| 282 | 5 | C | C | G | 0.80 | 0.7073028023 |  |
| 282 | 5 | C | C | T | 3.19 | 0.2357608702 |  |
| 283 | 6 | T | T | A | 3.23 | 0.2113412033 |  |
| 283 | 6 | T | T | C | 2.02 | 0.3690451542 |  |
| 283 | 6 | T | T | G | 3.23 | 0.1030376100 |  |
| 283 | 6 | T | T | T | 91.53 | 0.0000000000 | \* |
| 284 | 7 | G | G | A | 1.43 | 0.5727927608 |  |
| 284 | 7 | G | G | C | 0.71 | 0.8509424206 |  |
| 284 | 7 | G | G | G | 96.67 | 0.0000000000 | \* |
| 284 | 7 | G | G | T | 1.19 | 0.7670912755 |  |
| 285 | 8 | G | G | A | 2.69 | 0.2923902860 |  |
| 285 | 8 | G | G | C | 1.35 | 0.6463000620 |  |
| 285 | 8 | G | G | G | 94.17 | 0.0000000000 | \* |
| 285 | 8 | G | G | T | 1.79 | 0.5974177349 |  |
| 286 | 9 | C | C | A | 2.33 | 0.3607114300 |  |
| 286 | 9 | C | C | C | 91.86 | 0.0000000000 | \* |
| 286 | 9 | C | C | G | 2.33 | 0.2334708939 |  |
| 286 | 9 | C | C | T | 3.49 | 0.1841838395 |  |
| 287 | 10 | C | C | A | 2.89 | 0.2599268730 |  |
| 287 | 10 | C | C | C | 92.06 | 0.0000000000 | \* |
| 287 | 10 | C | C | G | 1.81 | 0.3591237946 |  |
| 287 | 10 | C | C | T | 3.25 | 0.2243430862 |  |
| 288 | 11 | A | A | A | 94.61 | 0.0000000000 | \* |
| 288 | 11 | A | A | C | 2.45 | 0.2294165807 |  |
| 288 | 11 | A | A | G | 1.96 | 0.3170342083 |  |
| 288 | 11 | A | A | T | 0.98 | 0.8119818481 |  |
| 289 | 12 | G | G | A | 5.23 | 0.0557933757 |  |
| 289 | 12 | G | G | C | 0.65 | 0.8611347043 |  |
| 289 | 12 | G | G | G | 92.81 | 0.0000000000 | \* |
| 289 | 12 | G | G | T | 1.31 | 0.7371879501 |  |
| 290 | 13 | A | A | A | 96.02 | 0.0000000000 | \* |
| 290 | 13 | A | A | C | 1.24 | 0.6878270205 |  |
| 290 | 13 | A | A | G | 1.00 | 0.6335510534 |  |
| 290 | 13 | A | A | T | 1.74 | 0.6132354378 |  |
| 291 | 14 | C | C | A | 3.91 | 0.1367836287 |  |
| 291 | 14 | C | C | C | 92.58 | 0.0000000000 | \* |
| 291 | 14 | C | C | G | 0.39 | 0.8381312016 |  |
| 291 | 14 | C | C | T | 3.12 | 0.2476953343 |  |
| 292 | 15 | C | C | A | 4.13 | 0.1181464938 |  |
| 292 | 15 | C | C | C | 90.83 | 0.0000000000 | \* |
| 292 | 15 | C | C | G | 1.83 | 0.3507641823 |  |
| 292 | 15 | C | C | T | 3.21 | 0.2313232373 |  |
| 293 | 16 | G | G | A | 4.26 | 0.1085743342 |  |
| 293 | 16 | G | G | C | 1.70 | 0.4942181658 |  |
| 293 | 16 | G | G | G | 92.34 | 0.0000000000 | \* |
| 293 | 16 | G | G | T | 1.70 | 0.6250003897 |  |
| 294 | 17 | T | T | A | 0.00 | 0.8545454545 |  |
| 294 | 17 | T | T | C | 1.71 | 0.4891067668 |  |
| 294 | 17 | T | T | G | 3.43 | 0.0848984364 |  |
| 294 | 17 | T | T | T | 94.86 | 0.0000000000 | \* |
| 295 | 18 | G | G | A | 1.45 | 0.5675931717 |  |
| 295 | 18 | G | G | C | 0.00 | 0.8915662651 |  |
| 295 | 18 | G | G | G | 97.69 | 0.0000000000 | \* |
| 295 | 18 | G | G | T | 0.87 | 0.8321060040 |  |
| 296 | 19 | T | T | A | 0.00 | 0.8545454545 |  |
| 296 | 19 | T | T | C | 1.50 | 0.5805725708 |  |
| 296 | 19 | T | T | G | 4.00 | 0.0485036312 |  |
| 296 | 19 | T | T | T | 94.50 | 0.0000000000 | \* |
| 297 | 20 | T | T | A | 1.73 | 0.4946675152 |  |
| 297 | 20 | T | T | C | 0.00 | 0.8915662651 |  |
| 297 | 20 | T | T | G | 2.60 | 0.1838949370 |  |
| 297 | 20 | T | T | T | 95.67 | 0.0000000000 | \* |
| 298 | 21 | T | T | A | 0.00 | 0.8545454545 |  |
| 298 | 21 | T | T | C | 1.32 | 0.6585504677 |  |
| 298 | 21 | T | T | G | 2.19 | 0.2614748291 |  |
| 298 | 21 | T | T | T | 96.49 | 0.0000000000 | \* |
| 299 | 22 | G | G | A | 1.45 | 0.5651273651 |  |
| 299 | 22 | G | G | C | 0.36 | 0.8880354978 |  |
| 299 | 22 | G | G | G | 97.45 | 0.0000000000 | \* |
| 299 | 22 | G | G | T | 0.73 | 0.8521207411 |  |
| 300 | 23 | T | T | A | 0.00 | 0.8545454545 |  |
| 300 | 23 | T | T | C | 1.48 | 0.5900842693 |  |
| 300 | 23 | T | T | G | 0.99 | 0.6372259174 |  |
| 300 | 23 | T | T | T | 97.54 | 0.0000000000 | \* |
| 301 | 24 | G | G | A | 2.85 | 0.2666615754 |  |
| 301 | 24 | G | G | C | 2.03 | 0.3630000554 |  |
| 301 | 24 | G | G | G | 93.50 | 0.0000000000 | \* |
| 301 | 24 | G | G | T | 1.63 | 0.6476707946 |  |
| 302 | 25 | C | C | A | 2.22 | 0.3820489630 |  |
| 302 | 25 | C | C | C | 92.00 | 0.0000000000 | \* |
| 302 | 25 | C | C | G | 1.78 | 0.3668988994 |  |
| 302 | 25 | C | C | T | 4.00 | 0.1178431409 |  |
| 303 | 26 | A | A | A | 93.64 | 0.0000000000 | \* |
| 303 | 26 | A | A | C | 4.05 | 0.0248788538 |  |
| 303 | 26 | A | A | G | 2.31 | 0.2361911589 |  |
| 303 | 26 | A | A | T | 0.00 | 0.8829787234 |  |
| 304 | 27 | C | C | A | 4.04 | 0.1256102446 |  |
| 304 | 27 | C | C | C | 84.30 | 0.0000000000 | \* |
| 304 | 27 | C | C | G | 8.97 | 0.0002320833 | \* |
| 304 | 27 | C | C | T | 2.69 | 0.3434700437 |  |
| 305 | 28 | T | T | A | 2.29 | 0.3672247621 |  |
| 305 | 28 | T | T | C | 2.75 | 0.1585771417 |  |
| 305 | 28 | T | T | G | 0.00 | 0.8901098901 |  |
| 305 | 28 | T | T | T | 94.95 | 0.0000000000 | \* |
| 306 | 29 | A | A | A | 95.83 | 0.0000000000 | \* |
| 306 | 29 | A | A | C | 1.67 | 0.5092060512 |  |
| 306 | 29 | A | A | G | 0.83 | 0.6938846737 |  |
| 306 | 29 | A | A | T | 1.67 | 0.6355993748 |  |
| 307 | 30 | C | C | A | 3.15 | 0.2210860579 |  |
| 307 | 30 | C | C | C | 94.14 | 0.0000000000 | \* |
| 307 | 30 | C | C | G | 0.00 | 0.8901098901 |  |
| 307 | 30 | C | C | T | 2.70 | 0.3405051843 |  |
| 308 | 31 | A | A | A | 94.68 | 0.0000000000 | \* |
| 308 | 31 | A | A | C | 2.13 | 0.3290600186 |  |
| 308 | 31 | A | A | G | 3.19 | 0.1064391519 |  |
| 308 | 31 | A | A | T | 0.00 | 0.8829787234 |  |
| 309 | 32 | C | C | A | 1.43 | 0.5719056353 |  |
| 309 | 32 | C | C | C | 92.86 | 0.0000000000 | \* |
| 309 | 32 | C | C | G | 2.38 | 0.2225415249 |  |
| 309 | 32 | C | C | T | 3.33 | 0.2094864984 |  |
| 310 | 33 | G | G | A | 2.89 | 0.2596002767 |  |
| 310 | 33 | G | G | C | 2.31 | 0.2691600698 |  |
| 310 | 33 | G | G | G | 93.06 | 0.0000000000 | \* |
| 310 | 33 | G | G | T | 1.73 | 0.6153989756 |  |
| 311 | 34 | G | G | A | 1.36 | 0.5905620308 |  |
| 311 | 34 | G | G | C | 0.45 | 0.8834082641 |  |
| 311 | 34 | G | G | G | 96.83 | 0.0000000000 | \* |
| 311 | 34 | G | G | T | 1.36 | 0.7238946799 |  |
| 312 | 35 | G | G | A | 0.00 | 0.8545454545 |  |
| 312 | 35 | G | G | C | 3.03 | 0.1100893075 |  |
| 312 | 35 | G | G | G | 94.55 | 0.0000000000 | \* |
| 312 | 35 | G | G | T | 2.42 | 0.4125583086 |  |
| 313 | 36 | C | C | A | 2.14 | 0.3998519125 |  |
| 313 | 36 | C | C | C | 94.12 | 0.0000000000 | \* |
| 313 | 36 | C | C | G | 1.60 | 0.4191259548 |  |
| 313 | 36 | C | C | T | 2.14 | 0.4936795548 |  |
| 314 | 37 | A | A | A | 93.85 | 0.0000000000 | \* |
| 314 | 37 | A | A | C | 3.08 | 0.1033424178 |  |
| 314 | 37 | A | A | G | 0.00 | 0.8901098901 |  |
| 314 | 37 | A | A | T | 3.08 | 0.2572184804 |  |
| 315 | 38 | C | C | A | 2.12 | 0.4043010661 |  |
| 315 | 38 | C | C | C | 94.92 | 0.0000000000 | \* |
| 315 | 38 | C | C | G | 0.42 | 0.8294925911 |  |
| 315 | 38 | C | C | T | 2.54 | 0.3810152578 |  |
| 316 | 39 | C | C | A | 3.98 | 0.1301236711 |  |
| 316 | 39 | C | C | C | 91.59 | 0.0000000000 | \* |
| 316 | 39 | C | C | G | 1.33 | 0.5116880330 |  |
| 316 | 39 | C | C | T | 3.10 | 0.2531405458 |  |
| 317 | 40 | C | C | A | 4.41 | 0.0981820167 |  |
| 317 | 40 | C | C | C | 90.75 | 0.0000000000 | \* |
| 317 | 40 | C | C | G | 2.20 | 0.2593453396 |  |
| 317 | 40 | C | C | T | 2.64 | 0.3552236834 |  |
| 318 | 41 | T | T | A | 1.87 | 0.4613228832 |  |
| 318 | 41 | T | T | C | 1.87 | 0.4256212024 |  |
| 318 | 41 | T | T | G | 2.80 | 0.1526308571 |  |
| 318 | 41 | T | T | T | 93.46 | 0.0000000000 | \* |
| 319 | 42 | G | G | A | 1.66 | 0.5135942121 |  |
| 319 | 42 | G | G | C | 0.99 | 0.7789538486 |  |
| 319 | 42 | G | G | G | 96.36 | 0.0000000000 | \* |
| 319 | 42 | G | G | T | 0.99 | 0.8094644719 |  |
| 320 | 43 | A | A | A | 94.21 | 0.0000000000 | \* |
| 320 | 43 | A | A | C | 1.65 | 0.5150561913 |  |
| 320 | 43 | A | A | G | 3.31 | 0.0954939188 |  |
| 320 | 43 | A | A | T | 0.83 | 0.8384791278 |  |
| 321 | 44 | C | C | A | 2.83 | 0.2691237962 |  |
| 321 | 44 | C | C | C | 91.51 | 0.0000000000 | \* |
| 321 | 44 | C | C | G | 2.36 | 0.2269220286 |  |
| 321 | 44 | C | C | T | 3.30 | 0.2149397891 |  |
| 322 | 45 | C | C | A | 6.53 | 0.0220216552 |  |
| 322 | 45 | C | C | C | 88.44 | 0.0000000000 | \* |
| 322 | 45 | C | C | G | 2.51 | 0.1982918518 |  |
| 322 | 45 | C | C | T | 2.51 | 0.3888451750 |  |
| 323 | 46 | G | G | A | 4.42 | 0.0972207519 |  |
| 323 | 46 | G | G | C | 2.21 | 0.3013542276 |  |
| 323 | 46 | G | G | G | 92.27 | 0.0000000000 | \* |
| 323 | 46 | G | G | T | 1.10 | 0.7861545355 |  |
| 324 | 47 | A | A | A | 96.63 | 0.0000000000 | \* |
| 324 | 47 | A | A | C | 1.50 | 0.5813766174 |  |
| 324 | 47 | A | A | G | 1.12 | 0.5855625903 |  |
| 324 | 47 | A | A | T | 0.75 | 0.8493594826 |  |
| 325 | 48 | C | C | A | 3.17 | 0.2181698548 |  |
| 325 | 48 | C | C | C | 91.53 | 0.0000000000 | \* |
| 325 | 48 | C | C | G | 1.06 | 0.6099012600 |  |
| 325 | 48 | C | C | T | 4.23 | 0.0952295311 |  |
| 326 | 49 | G | G | A | 3.94 | 0.1340537816 |  |
| 326 | 49 | G | G | C | 2.36 | 0.2542974755 |  |
| 326 | 49 | G | G | G | 93.70 | 0.0000000000 | \* |
| 326 | 49 | G | G | T | 0.00 | 0.8829787234 |  |
| 327 | 50 | G | G | A | 1.06 | 0.6680524621 |  |
| 327 | 50 | G | G | C | 1.06 | 0.7554092937 |  |
| 327 | 50 | G | G | G | 97.34 | 0.0000000000 | \* |
| 327 | 50 | G | G | T | 0.53 | 0.8709926273 |  |
| 328 | 51 | C | C | A | 1.54 | 0.5433984454 |  |
| 328 | 51 | C | C | C | 92.31 | 0.0000000000 | \* |
| 328 | 51 | C | C | G | 2.31 | 0.2370967488 |  |
| 328 | 51 | C | C | T | 3.85 | 0.1352231048 |  |
| 329 | 52 | A | A | A | 90.48 | 0.0000000000 | \* |
| 329 | 52 | A | A | C | 1.90 | 0.4115459343 |  |
| 329 | 52 | A | A | G | 2.86 | 0.1453489571 |  |
| 329 | 52 | A | A | T | 4.76 | 0.0575373314 |  |
| 330 | 53 | A | A | A | 98.24 | 0.0000000000 | \* |
| 330 | 53 | A | A | C | 0.00 | 0.8915662651 |  |
| 330 | 53 | A | A | G | 1.18 | 0.5660607047 |  |
| 330 | 53 | A | A | T | 0.59 | 0.8666169511 |  |
| 331 | 54 | G | G | A | 2.63 | 0.3026947393 |  |
| 331 | 54 | G | G | C | 0.88 | 0.8132712468 |  |
| 331 | 54 | G | G | G | 95.61 | 0.0000000000 | \* |
| 331 | 54 | G | G | T | 0.88 | 0.8304441707 |  |
| 332 | 55 | A | A | A | 97.17 | 0.0000000000 | \* |
| 332 | 55 | A | A | C | 0.00 | 0.8915662651 |  |
| 332 | 55 | A | A | G | 1.42 | 0.4812592478 |  |
| 332 | 55 | A | A | T | 1.42 | 0.7082098619 |  |
| 333 | 56 | A | A | A | 95.58 | 0.0000000000 | \* |
| 333 | 56 | A | A | C | 1.66 | 0.5131153236 |  |
| 333 | 56 | A | A | G | 1.66 | 0.4026149059 |  |
| 333 | 56 | A | A | T | 1.10 | 0.7861545355 |  |
| 334 | 57 | G | G | A | 1.87 | 0.4613228832 |  |
| 334 | 57 | G | G | C | 2.80 | 0.1484660279 |  |
| 334 | 57 | G | G | G | 91.59 | 0.0000000000 | \* |
| 334 | 57 | G | G | T | 3.74 | 0.1486693900 |  |
| 335 | 58 | T | T | A | 1.73 | 0.4940499965 |  |
| 335 | 58 | T | T | C | 1.73 | 0.4808083164 |  |
| 335 | 58 | T | T | G | 0.58 | 0.7834570863 |  |
| 335 | 58 | T | T | T | 95.95 | 0.0000000000 | \* |
| 336 | 59 | T | T | A | 1.09 | 0.6619893947 |  |
| 336 | 59 | T | T | C | 1.63 | 0.5246233188 |  |
| 336 | 59 | T | T | G | 1.09 | 0.5991747891 |  |
| 336 | 59 | T | T | T | 96.20 | 0.0000000000 | \* |
| 337 | 60 | C | C | A | 1.09 | 0.6604294946 |  |
| 337 | 60 | C | C | C | 95.08 | 0.0000000000 | \* |
| 337 | 60 | C | C | G | 1.09 | 0.5969634897 |  |
| 337 | 60 | C | C | T | 2.73 | 0.3333478749 |  |
| 338 | 61 | G | G | A | 5.41 | 0.0493014430 |  |
| 338 | 61 | G | G | C | 2.70 | 0.1688488074 |  |
| 338 | 61 | G | G | G | 87.39 | 0.0000000000 | \* |
| 338 | 61 | G | G | T | 4.50 | 0.0737556500 |  |
| 339 | 62 | A | A | A | 96.20 | 0.0000000000 | \* |
| 339 | 62 | A | A | C | 1.27 | 0.6789712328 |  |
| 339 | 62 | A | A | G | 0.84 | 0.6899872005 |  |
| 339 | 62 | A | A | T | 1.69 | 0.6293004721 |  |
| 340 | 63 | C | C | A | 3.52 | 0.1758470134 |  |
| 340 | 63 | C | C | C | 93.97 | 0.0000000000 | \* |
| 340 | 63 | C | C | G | 0.00 | 0.8901098901 |  |
| 340 | 63 | C | C | T | 2.51 | 0.3888451750 |  |
| 341 | 64 | A | A | A | 96.04 | 0.0000000000 | \* |
| 341 | 64 | A | A | C | 1.44 | 0.6067623605 |  |
| 341 | 64 | A | A | G | 1.44 | 0.4731817092 |  |
| 341 | 64 | A | A | T | 1.08 | 0.7918092432 |  |
| 342 | 65 | G | G | A | 2.72 | 0.2871732120 |  |
| 342 | 65 | G | G | C | 2.04 | 0.3599621046 |  |
| 342 | 65 | G | G | G | 94.56 | 0.0000000000 | \* |
| 342 | 65 | G | G | T | 0.68 | 0.8576224165 |  |
| 343 | 66 | C | C | A | 1.32 | 0.6015499557 |  |
| 343 | 66 | C | C | C | 94.74 | 0.0000000000 | \* |
| 343 | 66 | C | C | G | 1.32 | 0.5157991136 |  |
| 343 | 66 | C | C | T | 2.63 | 0.3581349475 |  |
| 344 | 67 | T | T | A | 1.08 | 0.6650557235 |  |
| 344 | 67 | T | T | C | 1.08 | 0.7514119052 |  |
| 344 | 67 | T | T | G | 0.54 | 0.7963095376 |  |
| 344 | 67 | T | T | T | 97.31 | 0.0000000000 | \* |
| 345 | 68 | C | C | A | 0.55 | 0.7907234334 |  |
| 345 | 68 | C | C | C | 92.82 | 0.0000000000 | \* |
| 345 | 68 | C | C | G | 3.87 | 0.0553250994 |  |
| 345 | 68 | C | C | T | 2.76 | 0.3261336068 |  |
| 346 | 69 | C | C | A | 1.03 | 0.6780211200 |  |
| 346 | 69 | C | C | C | 94.36 | 0.0000000000 | \* |
| 346 | 69 | C | C | G | 0.51 | 0.8039468862 |  |
| 346 | 69 | C | C | T | 4.10 | 0.1073601780 |  |
| 347 | 70 | C | C | A | 2.68 | 0.2934285604 |  |
| 347 | 70 | C | C | C | 91.28 | 0.0000000000 | \* |
| 347 | 70 | C | C | G | 1.34 | 0.5064683125 |  |
| 347 | 70 | C | C | T | 4.70 | 0.0612298705 |  |
| 348 | 71 | G | G | A | 2.36 | 0.3533612046 |  |
| 348 | 71 | G | G | C | 2.36 | 0.2542974755 |  |
| 348 | 71 | G | G | G | 92.91 | 0.0000000000 | \* |
| 348 | 71 | G | G | T | 2.36 | 0.4296508749 |  |
| 349 | 72 | C | C | A | 2.74 | 0.2840226155 |  |
| 349 | 72 | C | C | C | 93.84 | 0.0000000000 | \* |
| 349 | 72 | C | C | G | 0.68 | 0.7473788571 |  |
| 349 | 72 | C | C | T | 2.74 | 0.3315495554 |  |
| 350 | 73 | G | G | A | 3.97 | 0.1313312077 |  |
| 350 | 73 | G | G | C | 4.76 | 0.0078306280 | \* |
| 350 | 73 | G | G | G | 90.48 | 0.0000000000 | \* |
| 350 | 73 | G | G | T | 0.79 | 0.8432943599 |  |
| 351 | 74 | A | A | A | 96.39 | 0.0000000000 | \* |
| 351 | 74 | A | A | C | 2.58 | 0.1972126232 |  |
| 351 | 74 | A | A | G | 1.03 | 0.6201006537 |  |
| 351 | 74 | A | A | T | 0.00 | 0.8829787234 |  |
| 352 | 75 | C | C | A | 1.73 | 0.4940499965 |  |
| 352 | 75 | C | C | C | 92.49 | 0.0000000000 | \* |
| 352 | 75 | C | C | G | 0.58 | 0.7834570863 |  |
| 352 | 75 | C | C | T | 5.20 | 0.0371486343 |  |

## For use in R

If you want to work with the results in R, here is output that you can copy and paste in your terminal to get:

The base information:

```
structure(list(focal.base = c("A", "C", "G", "T"), avg.percsignal = c(94.9360778454264, 
93.1699339846895, 94.4441009006448, 94.6480895472376), avg.areasignal = c(221.235294117647, 
233.117647058824, 211.125, 243.604651162791), crit.perc.area = c(7.60676433756292, 
4.61414349110036, 5.5341334858211, 6.45172834852222), mu = c(2.38823389181938, 
1.95933866880757, 1.78176976230375, 2.54421536971399), fillibens = c(0.942968837786113, 
0.996463380429302, 0.9940689608119, 0.989126370614387)), .Names = c("focal.base", 
"avg.percsignal", "avg.areasignal", "crit.perc.area", "mu", "fillibens"
), row.names = c(NA, -4L), class = "data.frame")
```

the data.frame that contains information on the guide region:

```
structure(list(A.area = c(205, 227, 6, 421, 9, 8, 6, 6, 4, 8, 
193, 8, 386, 10, 9, 10, 0, 5, 0, 4, 0, 4, 0, 7, 5, 162, 9, 5, 
230, 7, 178, 3, 5, 3, 0, 4, 183, 5, 9, 10, 4, 5, 228, 6, 13, 
8, 258, 6, 5, 2, 2, 95, 167, 3, 206, 173, 2, 3, 2, 2, 6, 228, 
7, 267, 4, 2, 2, 1, 2, 4, 3, 4, 5, 187, 3), C.area = c(5, 2, 
3, 5, 232, 5, 3, 3, 158, 255, 5, 1, 5, 237, 198, 4, 3, 0, 3, 
0, 3, 1, 3, 5, 207, 7, 188, 6, 4, 209, 4, 195, 4, 1, 5, 176, 
6, 224, 207, 206, 4, 3, 4, 194, 176, 4, 4, 173, 3, 2, 120, 2, 
0, 1, 0, 3, 3, 3, 3, 174, 3, 3, 187, 4, 3, 144, 2, 168, 184, 
136, 3, 137, 6, 5, 160), G.area = c(5, 3, 149, 4, 2, 8, 407, 
210, 4, 5, 4, 142, 4, 1, 4, 217, 6, 338, 8, 6, 5, 268, 2, 230, 
4, 4, 20, 0, 2, 0, 6, 5, 161, 214, 156, 3, 0, 1, 3, 5, 6, 291, 
8, 5, 5, 167, 3, 2, 119, 183, 3, 3, 2, 109, 3, 3, 98, 1, 2, 2, 
97, 2, 0, 4, 139, 2, 1, 7, 1, 2, 118, 1, 114, 2, 1), T.area = c(0, 
6, 1, 6, 8, 227, 5, 4, 6, 9, 2, 2, 7, 8, 7, 4, 166, 3, 189, 221, 
220, 2, 198, 4, 9, 0, 6, 207, 4, 6, 0, 7, 3, 3, 4, 4, 6, 6, 7, 
6, 200, 3, 2, 7, 5, 2, 2, 8, 0, 1, 5, 5, 1, 1, 3, 2, 4, 166, 
177, 5, 5, 4, 5, 3, 1, 4, 181, 5, 8, 7, 3, 4, 1, 0, 9), Tot.area = c(215, 
238, 159, 436, 251, 248, 421, 223, 172, 277, 204, 153, 402, 256, 
218, 235, 175, 346, 200, 231, 228, 275, 203, 246, 225, 173, 223, 
218, 240, 222, 188, 210, 173, 221, 165, 187, 195, 236, 226, 227, 
214, 302, 242, 212, 199, 181, 267, 189, 127, 188, 130, 105, 170, 
114, 212, 181, 107, 173, 184, 183, 111, 237, 199, 278, 147, 152, 
186, 181, 195, 149, 127, 146, 126, 194, 173), A.perc = c(95.3488372093023, 
95.3781512605042, 3.77358490566038, 96.5596330275229, 3.58565737051793, 
3.2258064516129, 1.42517814726841, 2.69058295964126, 2.32558139534884, 
2.88808664259928, 94.6078431372549, 5.22875816993464, 96.0199004975124, 
3.90625, 4.12844036697248, 4.25531914893617, 0, 1.44508670520231, 
0, 1.73160173160173, 0, 1.45454545454545, 0, 2.84552845528455, 
2.22222222222222, 93.6416184971098, 4.03587443946188, 2.29357798165138, 
95.8333333333333, 3.15315315315315, 94.6808510638298, 1.42857142857143, 
2.89017341040462, 1.35746606334842, 0, 2.13903743315508, 93.8461538461538, 
2.11864406779661, 3.98230088495575, 4.40528634361233, 1.86915887850467, 
1.65562913907285, 94.2148760330578, 2.83018867924528, 6.53266331658291, 
4.41988950276243, 96.6292134831461, 3.17460317460317, 3.93700787401575, 
1.06382978723404, 1.53846153846154, 90.4761904761905, 98.2352941176471, 
2.63157894736842, 97.1698113207547, 95.5801104972376, 1.86915887850467, 
1.73410404624277, 1.08695652173913, 1.09289617486339, 5.40540540540541, 
96.2025316455696, 3.51758793969849, 96.0431654676259, 2.72108843537415, 
1.31578947368421, 1.0752688172043, 0.552486187845304, 1.02564102564103, 
2.68456375838926, 2.36220472440945, 2.73972602739726, 3.96825396825397, 
96.3917525773196, 1.73410404624277), C.perc = c(2.32558139534884, 
0.840336134453782, 1.88679245283019, 1.14678899082569, 92.4302788844622, 
2.01612903225806, 0.712589073634204, 1.34529147982063, 91.8604651162791, 
92.057761732852, 2.45098039215686, 0.65359477124183, 1.24378109452736, 
92.578125, 90.8256880733945, 1.70212765957447, 1.71428571428571, 
0, 1.5, 0, 1.31578947368421, 0.363636363636364, 1.47783251231527, 
2.03252032520325, 92, 4.04624277456647, 84.304932735426, 2.75229357798165, 
1.66666666666667, 94.1441441441441, 2.12765957446809, 92.8571428571429, 
2.3121387283237, 0.452488687782805, 3.03030303030303, 94.1176470588235, 
3.07692307692308, 94.9152542372881, 91.5929203539823, 90.7488986784141, 
1.86915887850467, 0.993377483443709, 1.65289256198347, 91.5094339622642, 
88.4422110552764, 2.20994475138122, 1.49812734082397, 91.5343915343915, 
2.36220472440945, 1.06382978723404, 92.3076923076923, 1.9047619047619, 
0, 0.87719298245614, 0, 1.65745856353591, 2.80373831775701, 1.73410404624277, 
1.6304347826087, 95.0819672131148, 2.7027027027027, 1.26582278481013, 
93.9698492462311, 1.43884892086331, 2.04081632653061, 94.7368421052632, 
1.0752688172043, 92.8176795580111, 94.3589743589744, 91.2751677852349, 
2.36220472440945, 93.8356164383562, 4.76190476190476, 2.57731958762887, 
92.485549132948), G.perc = c(2.32558139534884, 1.26050420168067, 
93.7106918238994, 0.917431192660551, 0.796812749003984, 3.2258064516129, 
96.6745843230404, 94.1704035874439, 2.32558139534884, 1.80505415162455, 
1.96078431372549, 92.8104575163399, 0.995024875621891, 0.390625, 
1.8348623853211, 92.3404255319149, 3.42857142857143, 97.6878612716763, 
4, 2.5974025974026, 2.19298245614035, 97.4545454545455, 0.985221674876847, 
93.4959349593496, 1.77777777777778, 2.3121387283237, 8.96860986547085, 
0, 0.833333333333333, 0, 3.19148936170213, 2.38095238095238, 
93.0635838150289, 96.8325791855204, 94.5454545454545, 1.60427807486631, 
0, 0.423728813559322, 1.32743362831858, 2.20264317180617, 2.80373831775701, 
96.3576158940397, 3.30578512396694, 2.35849056603774, 2.51256281407035, 
92.2651933701657, 1.12359550561798, 1.05820105820106, 93.7007874015748, 
97.3404255319149, 2.30769230769231, 2.85714285714286, 1.17647058823529, 
95.6140350877193, 1.41509433962264, 1.65745856353591, 91.588785046729, 
0.578034682080925, 1.08695652173913, 1.09289617486339, 87.3873873873874, 
0.843881856540084, 0, 1.43884892086331, 94.5578231292517, 1.31578947368421, 
0.537634408602151, 3.86740331491713, 0.512820512820513, 1.34228187919463, 
92.9133858267717, 0.684931506849315, 90.4761904761905, 1.03092783505155, 
0.578034682080925), T.perc = c(0, 2.52100840336134, 0.628930817610063, 
1.37614678899083, 3.18725099601594, 91.5322580645161, 1.18764845605701, 
1.79372197309417, 3.48837209302326, 3.24909747292419, 0.980392156862745, 
1.30718954248366, 1.74129353233831, 3.125, 3.21100917431193, 
1.70212765957447, 94.8571428571429, 0.867052023121387, 94.5, 
95.6709956709957, 96.4912280701754, 0.727272727272727, 97.5369458128079, 
1.6260162601626, 4, 0, 2.69058295964126, 94.954128440367, 1.66666666666667, 
2.7027027027027, 0, 3.33333333333333, 1.73410404624277, 1.35746606334842, 
2.42424242424242, 2.13903743315508, 3.07692307692308, 2.54237288135593, 
3.09734513274336, 2.6431718061674, 93.4579439252336, 0.993377483443709, 
0.826446280991736, 3.30188679245283, 2.51256281407035, 1.10497237569061, 
0.749063670411985, 4.23280423280423, 0, 0.531914893617021, 3.84615384615385, 
4.76190476190476, 0.588235294117647, 0.87719298245614, 1.41509433962264, 
1.10497237569061, 3.73831775700935, 95.9537572254335, 96.195652173913, 
2.73224043715847, 4.5045045045045, 1.68776371308017, 2.51256281407035, 
1.07913669064748, 0.680272108843537, 2.63157894736842, 97.3118279569892, 
2.76243093922652, 4.1025641025641, 4.69798657718121, 2.36220472440945, 
2.73972602739726, 0.793650793650794, 0, 5.20231213872832), base.call = c("A", 
"A", "G", "A", "C", "T", "G", "G", "C", "C", "A", "G", "A", "C", 
"C", "G", "T", "G", "T", "T", "T", "G", "T", "G", "C", "A", "C", 
"T", "A", "C", "A", "C", "G", "G", "G", "C", "A", "C", "C", "C", 
"T", "G", "A", "C", "C", "G", "A", "C", "G", "G", "C", "A", "A", 
"G", "A", "A", "G", "T", "T", "C", "G", "A", "C", "A", "G", "C", 
"T", "C", "C", "C", "G", "C", "G", "A", "C"), index = 278:352, 
    guide.seq = c("A", "A", "G", "A", "C", "T", "G", "G", "C", 
    "C", "A", "G", "A", "C", "C", "G", "T", "G", "T", "T", "T", 
    "G", "T", "G", "C", "A", "C", "T", "A", "C", "A", "C", "G", 
    "G", "G", "C", "A", "C", "C", "C", "T", "G", "A", "C", "C", 
    "G", "A", "C", "G", "G", "C", "A", "A", "G", "A", "A", "G", 
    "T", "T", "C", "G", "A", "C", "A", "G", "C", "T", "C", "C", 
    "C", "G", "C", "G", "A", "C"), T.pval = c(0.88297872340416, 
    0.386617723128664, 0.862927238684433, 0.718860039699248, 
    0.235760870196469, 0, 0.767091275542865, 0.597417734903808, 
    0.184183839460287, 0.224343086175196, 0.811981848108527, 
    0.737187950091948, 0.613235437819094, 0.247695334257322, 
    0.231323237326413, 0.625000389727306, 0, 0.832106003988072, 
    0, 0, 0, 0.852120741119751, 0, 0.647670794555306, 0.117843140867154, 
    0.88297872340416, 0.343470043680181, 0, 0.635599374804117, 
    0.3405051843491, 0.88297872340416, 0.209486498430065, 0.615398975583219, 
    0.723894679949317, 0.412558308617966, 0.493679554770719, 
    0.257218480417634, 0.381015257789195, 0.253140545838632, 
    0.355223683356865, 0, 0.809464471929121, 0.83847912778016, 
    0.214939789148337, 0.388845174975947, 0.786154535477157, 
    0.849359482624487, 0.0952295311470166, 0.88297872340416, 
    0.870992627347905, 0.135223104801795, 0.0575373314175638, 
    0.86661695106435, 0.830444170682779, 0.708209861941048, 0.786154535477157, 
    0.148669389950122, 0, 0, 0.33334787487489, 0.0737556499997878, 
    0.629300472127489, 0.388845174975947, 0.791809243233515, 
    0.857622416489014, 0.35813494749911, 0, 0.326133606796248, 
    0.107360177995766, 0.0612298705003598, 0.429650874872389, 
    0.331549555425153, 0.843294359937127, 0.88297872340416, 0.0371486342725376
    ), C.pval = c(0.265110541945359, 0.822866426176315, 0.418622402542678, 
    0.725425027539914, 0, 0.369045154191704, 0.85094242055008, 
    0.646300061977961, 0, 0, 0.229416580695909, 0.861134704320645, 
    0.687827020520578, 0, 0, 0.494218165848406, 0.489106766763866, 
    0.891566265059716, 0.580572570808755, 0.891566265059716, 
    0.658550467703181, 0.88803549783863, 0.59008426930261, 0.363000055380255, 
    0, 0.02487885379974, 0, 0.158577141658224, 0.509206051210271, 
    0, 0.329060018568003, 0, 0.269160069844694, 0.883408264078275, 
    0.110089307502682, 0, 0.103342417843748, 0, 0, 0, 0.425621202414603, 
    0.77895384863701, 0.515056191266366, 0, 0, 0.30135422763068, 
    0.581376617423302, 0, 0.25429747554872, 0.755409293713533, 
    0, 0.411545934312624, 0.891566265059716, 0.813271246843021, 
    0.891566265059716, 0.513115323629177, 0.148466027931985, 
    0.480808316431293, 0.52462331875211, 0, 0.168848807429845, 
    0.678971232771142, 0, 0.606762360494326, 0.359962104598348, 
    0, 0.75141190522445, 0, 0, 0, 0.25429747554872, 0, 0.00783062799919809, 
    0.197212623245984, 0), G.pval = c(0.233470893886287, 0.535519679939513, 
    0, 0.662617273383523, 0.707302802273753, 0.103037610035563, 
    0, 0, 0.233470893886287, 0.359123794616112, 0.317034208348602, 
    0, 0.633551053449189, 0.838131201637504, 0.350764182284542, 
    0, 0.0848984363903298, 0, 0.0485036312398208, 0.183894937008899, 
    0.26147482906613, 0, 0.637225917399858, 0, 0.366898899418765, 
    0.236191158903696, 0.000232083345478862, 0.890109890109496, 
    0.693884673745256, 0.890109890109496, 0.106439151937249, 
    0.222541524913432, 0, 0, 0, 0.419125954820527, 0.890109890109496, 
    0.82949259114079, 0.511688032958903, 0.259345339570728, 0.152630857092671, 
    0, 0.0954939188385489, 0.226922028612718, 0.198291851822878, 
    0, 0.585562590273363, 0.609901260034793, 0, 0, 0.237096748810784, 
    0.145348957117251, 0.566060704713637, 0, 0.481259247775762, 
    0.402614905891771, 0, 0.783457086277806, 0.599174789097498, 
    0.596963489710836, 0, 0.689987200539139, 0.890109890109496, 
    0.473181709174679, 0, 0.515799113605271, 0.796309537600839, 
    0.0553250993895977, 0.803946886150784, 0.506468312450683, 
    0, 0.747378857094196, 0, 0.620100653652916, 0.783457086277806
    ), A.pval = c(0, 0, 0.149142021438844, 0, 0.168361189786787, 
    0.211341203262509, 0.572792760813698, 0.292390286041813, 
    0.360711430023031, 0.259926872978834, 0, 0.055793375670862, 
    0, 0.136783628700863, 0.118146493812715, 0.10857433416328, 
    0.854545454545454, 0.567593171693413, 0.854545454545454, 
    0.494667515239366, 0.854545454545454, 0.565127365055085, 
    0.854545454545454, 0.266661575417124, 0.382048963039821, 
    0, 0.12561024459411, 0.367224762101191, 0, 0.221086057850273, 
    0, 0.571905635313595, 0.25960027671892, 0.590562030752353, 
    0.854545454545454, 0.399851912460315, 0, 0.404301066086956, 
    0.130123671095051, 0.0981820166962231, 0.461322883232954, 
    0.513594212052908, 0, 0.269123796195995, 0.022021655166595, 
    0.0972207519361028, 0, 0.218169854780578, 0.13405378159473, 
    0.668052462125224, 0.543398445439391, 0, 0, 0.302694739260598, 
    0, 0, 0.461322883232954, 0.494049996495983, 0.66198939465182, 
    0.660429494610515, 0.0493014430016785, 0, 0.175847013393771, 
    0, 0.287173211966147, 0.601549955693965, 0.665055723501423, 
    0.790723433360134, 0.678021119984147, 0.293428560350228, 
    0.353361204586434, 0.284022615519864, 0.131331207670336, 
    0, 0.494049996495983), guide.position = 1:75), .Names = c("A.area", 
"C.area", "G.area", "T.area", "Tot.area", "A.perc", "C.perc", 
"G.perc", "T.perc", "base.call", "index", "guide.seq", "T.pval", 
"C.pval", "G.pval", "A.pval", "guide.position"), row.names = 278:352, class = "data.frame")
```

*Report generated using EditR v1.0.8*
