# Supplementary material for: Mechanistic and genetic basis of single-strand templated repair at Cas12a-induced DNA breaks in Chlamydomonas reinhardtii
Source: Nat Commun. 2021 Nov 19;12:6751. doi: 10.1038/s41467-021-27004-1 (PMC8604939; doi:10.1038/s41467-021-27004-1)
Supplement: Supplementary file 22 — Source Data [file 41467_2021_27004_MOESM22_ESM.zip › Source Data/EditR analysis/EditR outputs/Antisense/rep1_ssODN_antisense_32.html]

EditR v1.0.8 report


# EditR v1.0.8 report

- Data QA
  - Filtering data
  - Percent noise peak area
  - Base information
- Predicted editing
  - Editing bar plot
  - Editing table plot
  - Table of editing results
- For use in R

## Data QA

### Filtering data

What the data looked like prefiltering:

and the post filtering signal / noise plot:

### Percent noise peak area

### Base information

Here’s information about the signal of each base, the critical percent value where any higher value would be called as significant, and Filliben’s correlation for how well the noise was modelled by the zero adjusted gamma distribution.

| Base | Average percent signal | Average peak area | Critical percent value | model mu | Fillibens correlation |
| --- | --- | --- | --- | --- | --- |
| A | 92.49543 | 529.2941 | 11.955658 | 3.373167 | 0.9656538 |
| C | 91.96959 | 527.2239 | 5.382135 | 1.981334 | 0.9941835 |
| G | 93.68134 | 534.0357 | 8.449633 | 2.255921 | 0.9925880 |
| T | 93.61016 | 649.3636 | 8.111994 | 2.626999 | 0.9963590 |

## Predicted editing

### Editing bar plot

### Editing table plot

### Table of editing results


Here’s the entire guide region

| Sanger position | Guide position | Guide sequence | Sanger base call | Focal base | Focal base peak area | p value |  |
| --- | --- | --- | --- | --- | --- | --- | --- |
| 276 | 1 | A | A | A | 94.01 | 0.000000e+00 | \* |
| 276 | 1 | A | A | C | 1.80 | 4.510522e-01 |  |
| 276 | 1 | A | A | G | 0.60 | 7.929430e-01 |  |
| 276 | 1 | A | A | T | 3.59 | 2.166837e-01 |  |
| 277 | 2 | A | A | A | 93.51 | 0.000000e+00 | \* |
| 277 | 2 | A | A | C | 2.00 | 3.846467e-01 |  |
| 277 | 2 | A | A | G | 1.16 | 6.254889e-01 |  |
| 277 | 2 | A | A | T | 3.33 | 2.536969e-01 |  |
| 278 | 3 | G | G | A | 2.35 | 4.822634e-01 |  |
| 278 | 3 | G | G | C | 1.07 | 7.211697e-01 |  |
| 278 | 3 | G | G | G | 95.30 | 0.000000e+00 | \* |
| 278 | 3 | G | G | T | 1.28 | 7.028248e-01 |  |
| 279 | 4 | A | A | A | 94.72 | 0.000000e+00 | \* |
| 279 | 4 | A | A | C | 0.88 | 7.858616e-01 |  |
| 279 | 4 | A | A | G | 0.78 | 7.379783e-01 |  |
| 279 | 4 | A | A | T | 3.62 | 2.135696e-01 |  |
| 280 | 5 | C | C | A | 4.39 | 2.319660e-01 |  |
| 280 | 5 | C | C | C | 92.81 | 0.000000e+00 | \* |
| 280 | 5 | C | C | G | 0.88 | 7.094504e-01 |  |
| 280 | 5 | C | C | T | 1.93 | 5.350681e-01 |  |
| 281 | 6 | T | T | A | 4.24 | 2.453398e-01 |  |
| 281 | 6 | T | T | C | 1.19 | 6.755587e-01 |  |
| 281 | 6 | T | T | G | 1.85 | 4.490118e-01 |  |
| 281 | 6 | T | T | T | 92.72 | 0.000000e+00 | \* |
| 282 | 7 | G | G | A | 2.69 | 4.293912e-01 |  |
| 282 | 7 | G | G | C | 0.00 | 9.166667e-01 |  |
| 282 | 7 | G | G | G | 96.15 | 0.000000e+00 | \* |
| 282 | 7 | G | G | T | 1.15 | 7.354322e-01 |  |
| 283 | 8 | G | G | A | 3.75 | 2.943501e-01 |  |
| 283 | 8 | G | G | C | 0.72 | 8.328529e-01 |  |
| 283 | 8 | G | G | G | 93.65 | 0.000000e+00 | \* |
| 283 | 8 | G | G | T | 1.88 | 5.485967e-01 |  |
| 284 | 9 | C | C | A | 3.59 | 3.124369e-01 |  |
| 284 | 9 | C | C | C | 91.03 | 0.000000e+00 | \* |
| 284 | 9 | C | C | G | 2.31 | 3.556643e-01 |  |
| 284 | 9 | C | C | T | 3.08 | 2.933813e-01 |  |
| 285 | 10 | C | C | A | 2.71 | 4.276464e-01 |  |
| 285 | 10 | C | C | C | 92.33 | 0.000000e+00 | \* |
| 285 | 10 | C | C | G | 3.16 | 2.250226e-01 |  |
| 285 | 10 | C | C | T | 1.80 | 5.667143e-01 |  |
| 286 | 11 | A | A | A | 92.54 | 0.000000e+00 | \* |
| 286 | 11 | A | A | C | 1.81 | 4.448046e-01 |  |
| 286 | 11 | A | A | G | 1.81 | 4.580700e-01 |  |
| 286 | 11 | A | A | T | 3.83 | 1.874740e-01 |  |
| 287 | 12 | G | G | A | 2.40 | 4.743937e-01 |  |
| 287 | 12 | G | G | C | 2.20 | 3.238281e-01 |  |
| 287 | 12 | G | G | G | 93.60 | 0.000000e+00 | \* |
| 287 | 12 | G | G | T | 1.80 | 5.678664e-01 |  |
| 288 | 13 | A | A | A | 94.69 | 0.000000e+00 | \* |
| 288 | 13 | A | A | C | 0.87 | 7.891234e-01 |  |
| 288 | 13 | A | A | G | 0.77 | 7.407039e-01 |  |
| 288 | 13 | A | A | T | 3.67 | 2.066159e-01 |  |
| 289 | 14 | C | C | A | 4.42 | 2.290633e-01 |  |
| 289 | 14 | C | C | C | 91.82 | 0.000000e+00 | \* |
| 289 | 14 | C | C | G | 1.64 | 5.002929e-01 |  |
| 289 | 14 | C | C | T | 2.13 | 4.867842e-01 |  |
| 290 | 15 | C | C | A | 7.75 | 6.020999e-02 |  |
| 290 | 15 | C | C | C | 88.56 | 0.000000e+00 | \* |
| 290 | 15 | C | C | G | 1.66 | 4.944657e-01 |  |
| 290 | 15 | C | C | T | 2.03 | 5.104540e-01 |  |
| 291 | 16 | G | G | A | 4.41 | 2.301325e-01 |  |
| 291 | 16 | G | G | C | 1.36 | 6.134076e-01 |  |
| 291 | 16 | G | G | G | 92.88 | 0.000000e+00 | \* |
| 291 | 16 | G | G | T | 1.36 | 6.835885e-01 |  |
| 292 | 17 | T | T | A | 0.00 | 8.545455e-01 |  |
| 292 | 17 | T | T | C | 2.11 | 3.512968e-01 |  |
| 292 | 17 | T | T | G | 1.68 | 4.887280e-01 |  |
| 292 | 17 | T | T | T | 96.21 | 0.000000e+00 | \* |
| 293 | 18 | G | G | A | 3.34 | 3.424914e-01 |  |
| 293 | 18 | G | G | C | 1.11 | 7.051598e-01 |  |
| 293 | 18 | G | G | G | 94.99 | 0.000000e+00 | \* |
| 293 | 18 | G | G | T | 0.56 | 8.665943e-01 |  |
| 294 | 19 | T | T | A | 1.52 | 6.250779e-01 |  |
| 294 | 19 | T | T | C | 1.69 | 4.889202e-01 |  |
| 294 | 19 | T | T | G | 2.20 | 3.770158e-01 |  |
| 294 | 19 | T | T | T | 94.59 | 0.000000e+00 | \* |
| 295 | 20 | T | T | A | 1.21 | 6.814007e-01 |  |
| 295 | 20 | T | T | C | 1.97 | 3.932314e-01 |  |
| 295 | 20 | T | T | G | 3.18 | 2.220750e-01 |  |
| 295 | 20 | T | T | T | 93.64 | 0.000000e+00 | \* |
| 296 | 21 | T | T | A | 0.00 | 8.545455e-01 |  |
| 296 | 21 | T | T | C | 2.52 | 2.428116e-01 |  |
| 296 | 21 | T | T | G | 1.78 | 4.665598e-01 |  |
| 296 | 21 | T | T | T | 95.70 | 0.000000e+00 | \* |
| 297 | 22 | G | G | A | 1.65 | 6.024450e-01 |  |
| 297 | 22 | G | G | C | 0.00 | 9.166667e-01 |  |
| 297 | 22 | G | G | G | 97.22 | 0.000000e+00 | \* |
| 297 | 22 | G | G | T | 1.14 | 7.394049e-01 |  |
| 298 | 23 | T | T | A | 0.00 | 8.545455e-01 |  |
| 298 | 23 | T | T | C | 2.30 | 2.962771e-01 |  |
| 298 | 23 | T | T | G | 1.59 | 5.111084e-01 |  |
| 298 | 23 | T | T | T | 96.11 | 0.000000e+00 | \* |
| 299 | 24 | G | G | A | 1.83 | 5.703009e-01 |  |
| 299 | 24 | G | G | C | 1.22 | 6.659302e-01 |  |
| 299 | 24 | G | G | G | 95.43 | 0.000000e+00 | \* |
| 299 | 24 | G | G | T | 1.52 | 6.400328e-01 |  |
| 300 | 25 | C | C | A | 1.32 | 6.615027e-01 |  |
| 300 | 25 | C | C | C | 93.77 | 0.000000e+00 | \* |
| 300 | 25 | C | C | G | 1.89 | 4.417207e-01 |  |
| 300 | 25 | C | C | T | 3.02 | 3.032367e-01 |  |
| 301 | 26 | A | A | A | 90.16 | 0.000000e+00 | \* |
| 301 | 26 | A | A | C | 3.36 | 1.047754e-01 |  |
| 301 | 26 | A | A | G | 2.91 | 2.579816e-01 |  |
| 301 | 26 | A | A | T | 3.58 | 2.184387e-01 |  |
| 302 | 27 | C | C | A | 1.94 | 5.496895e-01 |  |
| 302 | 27 | C | C | C | 95.79 | 0.000000e+00 | \* |
| 302 | 27 | C | C | G | 0.00 | 9.340659e-01 |  |
| 302 | 27 | C | C | T | 2.27 | 4.537672e-01 |  |
| 303 | 28 | T | T | A | 1.79 | 5.766120e-01 |  |
| 303 | 28 | T | T | C | 3.03 | 1.470724e-01 |  |
| 303 | 28 | T | T | G | 0.41 | 8.468999e-01 |  |
| 303 | 28 | T | T | T | 94.77 | 0.000000e+00 | \* |
| 304 | 29 | A | A | A | 95.46 | 0.000000e+00 | \* |
| 304 | 29 | A | A | C | 1.57 | 5.322855e-01 |  |
| 304 | 29 | A | A | G | 1.22 | 6.094569e-01 |  |
| 304 | 29 | A | A | T | 1.75 | 5.819238e-01 |  |
| 305 | 30 | C | C | A | 1.86 | 5.636598e-01 |  |
| 305 | 30 | C | C | C | 93.39 | 0.000000e+00 | \* |
| 305 | 30 | C | C | G | 1.36 | 5.725835e-01 |  |
| 305 | 30 | C | C | T | 3.39 | 2.445913e-01 |  |
| 306 | 31 | A | A | A | 89.51 | 0.000000e+00 | \* |
| 306 | 31 | A | A | C | 2.68 | 2.085525e-01 |  |
| 306 | 31 | A | A | G | 4.24 | 1.223482e-01 |  |
| 306 | 31 | A | A | T | 3.57 | 2.194912e-01 |  |
| 307 | 32 | C | C | A | 1.51 | 6.270463e-01 |  |
| 307 | 32 | C | C | C | 94.72 | 0.000000e+00 | \* |
| 307 | 32 | C | C | G | 1.51 | 5.322176e-01 |  |
| 307 | 32 | C | C | T | 2.26 | 4.548872e-01 |  |
| 308 | 33 | G | G | A | 3.36 | 3.399956e-01 |  |
| 308 | 33 | G | G | C | 1.92 | 4.098538e-01 |  |
| 308 | 33 | G | G | G | 92.33 | 0.000000e+00 | \* |
| 308 | 33 | G | G | T | 2.40 | 4.248399e-01 |  |
| 309 | 34 | G | G | A | 0.00 | 8.545455e-01 |  |
| 309 | 34 | G | G | C | 1.52 | 5.501454e-01 |  |
| 309 | 34 | G | G | G | 98.14 | 0.000000e+00 | \* |
| 309 | 34 | G | G | T | 0.34 | 8.966575e-01 |  |
| 310 | 35 | G | G | A | 3.67 | 3.038140e-01 |  |
| 310 | 35 | G | G | C | 1.22 | 6.642936e-01 |  |
| 310 | 35 | G | G | G | 95.11 | 0.000000e+00 | \* |
| 310 | 35 | G | G | T | 0.00 | 9.139785e-01 |  |
| 311 | 36 | C | C | A | 4.06 | 2.626823e-01 |  |
| 311 | 36 | C | C | C | 94.51 | 0.000000e+00 | \* |
| 311 | 36 | C | C | G | 1.43 | 5.523385e-01 |  |
| 311 | 36 | C | C | T | 0.00 | 9.139785e-01 |  |
| 312 | 37 | A | A | A | 92.56 | 0.000000e+00 | \* |
| 312 | 37 | A | A | C | 2.69 | 2.070725e-01 |  |
| 312 | 37 | A | A | G | 0.83 | 7.246370e-01 |  |
| 312 | 37 | A | A | T | 3.93 | 1.767995e-01 |  |
| 313 | 38 | C | C | A | 2.92 | 3.973833e-01 |  |
| 313 | 38 | C | C | C | 95.55 | 0.000000e+00 | \* |
| 313 | 38 | C | C | G | 1.54 | 5.254097e-01 |  |
| 313 | 38 | C | C | T | 0.00 | 9.139785e-01 |  |
| 314 | 39 | C | C | A | 7.52 | 6.631717e-02 |  |
| 314 | 39 | C | C | C | 89.54 | 0.000000e+00 | \* |
| 314 | 39 | C | C | G | 0.82 | 7.274726e-01 |  |
| 314 | 39 | C | C | T | 2.12 | 4.876126e-01 |  |
| 315 | 40 | C | C | A | 5.17 | 1.711072e-01 |  |
| 315 | 40 | C | C | C | 90.69 | 0.000000e+00 | \* |
| 315 | 40 | C | C | G | 1.55 | 5.214488e-01 |  |
| 315 | 40 | C | C | T | 2.59 | 3.848632e-01 |  |
| 316 | 41 | T | T | A | 2.84 | 4.084082e-01 |  |
| 316 | 41 | T | T | C | 2.84 | 1.780844e-01 |  |
| 316 | 41 | T | T | G | 2.24 | 3.680589e-01 |  |
| 316 | 41 | T | T | T | 92.08 | 0.000000e+00 | \* |
| 317 | 42 | G | G | A | 5.96 | 1.250679e-01 |  |
| 317 | 42 | G | G | C | 0.00 | 9.166667e-01 |  |
| 317 | 42 | G | G | G | 92.68 | 0.000000e+00 | \* |
| 317 | 42 | G | G | T | 1.36 | 6.838285e-01 |  |
| 318 | 43 | A | A | A | 93.69 | 0.000000e+00 | \* |
| 318 | 43 | A | A | C | 0.96 | 7.591967e-01 |  |
| 318 | 43 | A | A | G | 2.06 | 4.049305e-01 |  |
| 318 | 43 | A | A | T | 3.29 | 2.590474e-01 |  |
| 319 | 44 | C | C | A | 7.67 | 6.232268e-02 |  |
| 319 | 44 | C | C | C | 90.29 | 0.000000e+00 | \* |
| 319 | 44 | C | C | G | 0.00 | 9.340659e-01 |  |
| 319 | 44 | C | C | T | 2.04 | 5.068540e-01 |  |
| 320 | 45 | C | C | A | 9.76 | 2.582557e-02 |  |
| 320 | 45 | C | C | C | 83.11 | 0.000000e+00 | \* |
| 320 | 45 | C | C | G | 5.25 | 6.803501e-02 |  |
| 320 | 45 | C | C | T | 1.88 | 5.485285e-01 |  |
| 321 | 46 | G | G | A | 7.68 | 6.208137e-02 |  |
| 321 | 46 | G | G | C | 1.97 | 3.919551e-01 |  |
| 321 | 46 | G | G | G | 90.35 | 0.000000e+00 | \* |
| 321 | 46 | G | G | T | 0.00 | 9.139785e-01 |  |
| 322 | 47 | A | A | A | 94.20 | 0.000000e+00 | \* |
| 322 | 47 | A | A | C | 0.86 | 7.911658e-01 |  |
| 322 | 47 | A | A | G | 0.62 | 7.876605e-01 |  |
| 322 | 47 | A | A | T | 4.32 | 1.383640e-01 |  |
| 323 | 48 | C | C | A | 5.25 | 1.658066e-01 |  |
| 323 | 48 | C | C | C | 93.13 | 0.000000e+00 | \* |
| 323 | 48 | C | C | G | 1.62 | 5.053407e-01 |  |
| 323 | 48 | C | C | T | 0.00 | 9.139785e-01 |  |
| 324 | 49 | G | G | A | 8.24 | 4.914937e-02 |  |
| 324 | 49 | G | G | C | 1.76 | 4.620962e-01 |  |
| 324 | 49 | G | G | G | 87.35 | 0.000000e+00 | \* |
| 324 | 49 | G | G | T | 2.65 | 3.725078e-01 |  |
| 325 | 50 | G | G | A | 2.02 | 5.367819e-01 |  |
| 325 | 50 | G | G | C | 1.18 | 6.806535e-01 |  |
| 325 | 50 | G | G | G | 96.80 | 0.000000e+00 | \* |
| 325 | 50 | G | G | T | 0.00 | 9.139785e-01 |  |
| 326 | 51 | C | C | A | 2.95 | 3.930576e-01 |  |
| 326 | 51 | C | C | C | 91.74 | 0.000000e+00 | \* |
| 326 | 51 | C | C | G | 2.95 | 2.522043e-01 |  |
| 326 | 51 | C | C | T | 2.36 | 4.332788e-01 |  |
| 327 | 52 | A | A | A | 93.47 | 0.000000e+00 | \* |
| 327 | 52 | A | A | C | 1.37 | 6.063044e-01 |  |
| 327 | 52 | A | A | G | 2.06 | 4.040497e-01 |  |
| 327 | 52 | A | A | T | 3.09 | 2.907334e-01 |  |
| 328 | 53 | A | A | A | 93.89 | 0.000000e+00 | \* |
| 328 | 53 | A | A | C | 0.79 | 8.138238e-01 |  |
| 328 | 53 | A | A | G | 2.37 | 3.447824e-01 |  |
| 328 | 53 | A | A | T | 2.96 | 3.137435e-01 |  |
| 329 | 54 | G | G | A | 3.53 | 3.197507e-01 |  |
| 329 | 54 | G | G | C | 0.00 | 9.166667e-01 |  |
| 329 | 54 | G | G | G | 95.21 | 0.000000e+00 | \* |
| 329 | 54 | G | G | T | 1.26 | 7.086769e-01 |  |
| 330 | 55 | A | A | A | 93.26 | 0.000000e+00 | \* |
| 330 | 55 | A | A | C | 0.00 | 9.166667e-01 |  |
| 330 | 55 | A | A | G | 3.19 | 2.208935e-01 |  |
| 330 | 55 | A | A | T | 3.55 | 2.228562e-01 |  |
| 331 | 56 | A | A | A | 92.28 | 0.000000e+00 | \* |
| 331 | 56 | A | A | C | 0.00 | 9.166667e-01 |  |
| 331 | 56 | A | A | G | 2.64 | 2.978487e-01 |  |
| 331 | 56 | A | A | T | 5.08 | 8.401781e-02 |  |
| 332 | 57 | G | G | A | 2.15 | 5.153924e-01 |  |
| 332 | 57 | G | G | C | 1.23 | 6.624074e-01 |  |
| 332 | 57 | G | G | G | 92.64 | 0.000000e+00 | \* |
| 332 | 57 | G | G | T | 3.99 | 1.701097e-01 |  |
| 333 | 58 | T | T | A | 0.80 | 7.561141e-01 |  |
| 333 | 58 | T | T | C | 2.39 | 2.744802e-01 |  |
| 333 | 58 | T | T | G | 0.99 | 6.748205e-01 |  |
| 333 | 58 | T | T | T | 95.83 | 0.000000e+00 | \* |
| 334 | 59 | T | T | A | 0.93 | 7.316754e-01 |  |
| 334 | 59 | T | T | C | 1.87 | 4.262335e-01 |  |
| 334 | 59 | T | T | G | 1.68 | 4.892025e-01 |  |
| 334 | 59 | T | T | T | 95.51 | 0.000000e+00 | \* |
| 335 | 60 | C | C | A | 0.00 | 8.545455e-01 |  |
| 335 | 60 | C | C | C | 92.11 | 0.000000e+00 | \* |
| 335 | 60 | C | C | G | 6.40 | 3.457795e-02 |  |
| 335 | 60 | C | C | T | 1.49 | 6.477817e-01 |  |
| 336 | 61 | G | G | A | 6.08 | 1.194737e-01 |  |
| 336 | 61 | G | G | C | 1.52 | 5.515920e-01 |  |
| 336 | 61 | G | G | G | 90.38 | 0.000000e+00 | \* |
| 336 | 61 | G | G | T | 2.03 | 5.114807e-01 |  |
| 337 | 62 | A | A | A | 93.45 | 0.000000e+00 | \* |
| 337 | 62 | A | A | C | 1.16 | 6.858687e-01 |  |
| 337 | 62 | A | A | G | 2.47 | 3.257184e-01 |  |
| 337 | 62 | A | A | T | 2.91 | 3.221952e-01 |  |
| 338 | 63 | C | C | A | 4.36 | 2.346537e-01 |  |
| 338 | 63 | C | C | C | 91.29 | 0.000000e+00 | \* |
| 338 | 63 | C | C | G | 2.18 | 3.805717e-01 |  |
| 338 | 63 | C | C | T | 2.18 | 4.749096e-01 |  |
| 339 | 64 | A | A | A | 88.56 | 0.000000e+00 | \* |
| 339 | 64 | A | A | C | 1.91 | 4.135118e-01 |  |
| 339 | 64 | A | A | G | 6.27 | 3.735680e-02 |  |
| 339 | 64 | A | A | T | 3.27 | 2.624647e-01 |  |
| 340 | 65 | G | G | A | 1.97 | 5.445331e-01 |  |
| 340 | 65 | G | G | C | 2.69 | 2.056671e-01 |  |
| 340 | 65 | G | G | G | 95.33 | 0.000000e+00 | \* |
| 340 | 65 | G | G | T | 0.00 | 9.139785e-01 |  |
| 341 | 66 | C | C | A | 2.86 | 4.059903e-01 |  |
| 341 | 66 | C | C | C | 88.13 | 0.000000e+00 | \* |
| 341 | 66 | C | C | G | 3.74 | 1.629979e-01 |  |
| 341 | 66 | C | C | T | 5.27 | 7.383394e-02 |  |
| 342 | 67 | T | T | A | 0.74 | 7.652346e-01 |  |
| 342 | 67 | T | T | C | 2.52 | 2.419638e-01 |  |
| 342 | 67 | T | T | G | 0.00 | 9.340659e-01 |  |
| 342 | 67 | T | T | T | 96.74 | 0.000000e+00 | \* |
| 343 | 68 | C | C | A | 3.08 | 3.758512e-01 |  |
| 343 | 68 | C | C | C | 92.31 | 0.000000e+00 | \* |
| 343 | 68 | C | C | G | 1.35 | 5.752208e-01 |  |
| 343 | 68 | C | C | T | 3.27 | 2.625450e-01 |  |
| 344 | 69 | C | C | A | 2.86 | 4.059903e-01 |  |
| 344 | 69 | C | C | C | 93.49 | 0.000000e+00 | \* |
| 344 | 69 | C | C | G | 0.48 | 8.289621e-01 |  |
| 344 | 69 | C | C | T | 3.17 | 2.773742e-01 |  |
| 345 | 70 | C | C | A | 4.04 | 2.645894e-01 |  |
| 345 | 70 | C | C | C | 88.36 | 0.000000e+00 | \* |
| 345 | 70 | C | C | G | 4.51 | 1.046439e-01 |  |
| 345 | 70 | C | C | T | 3.09 | 2.915490e-01 |  |
| 346 | 71 | G | G | A | 6.04 | 1.212795e-01 |  |
| 346 | 71 | G | G | C | 4.35 | 3.460845e-02 |  |
| 346 | 71 | G | G | G | 88.41 | 0.000000e+00 | \* |
| 346 | 71 | G | G | T | 1.21 | 7.219870e-01 |  |
| 347 | 72 | C | C | A | 0.00 | 8.545455e-01 |  |
| 347 | 72 | C | C | C | 93.01 | 0.000000e+00 | \* |
| 347 | 72 | C | C | G | 5.13 | 7.320408e-02 |  |
| 347 | 72 | C | C | T | 1.86 | 5.513989e-01 |  |
| 348 | 73 | G | G | A | 6.48 | 1.016049e-01 |  |
| 348 | 73 | G | G | C | 3.37 | 1.034273e-01 |  |
| 348 | 73 | G | G | G | 89.90 | 0.000000e+00 | \* |
| 348 | 73 | G | G | T | 0.26 | 9.041131e-01 |  |
| 349 | 74 | A | A | A | 92.72 | 0.000000e+00 | \* |
| 349 | 74 | A | A | C | 1.52 | 5.501454e-01 |  |
| 349 | 74 | A | A | G | 1.52 | 5.287864e-01 |  |
| 349 | 74 | A | A | T | 4.23 | 1.460897e-01 |  |
| 350 | 75 | C | C | A | 3.50 | 3.225400e-01 |  |
| 350 | 75 | C | C | C | 74.61 | 0.000000e+00 | \* |
| 350 | 75 | C | C | G | 18.04 | 2.487675e-05 | \* |
| 350 | 75 | C | C | T | 3.85 | 1.849248e-01 |  |

## For use in R

If you want to work with the results in R, here is output that you can copy and paste in your terminal to get:

The base information:

```
structure(list(focal.base = c("A", "C", "G", "T"), avg.percsignal = c(92.4954313412268, 
91.9695915964188, 93.6813355408679, 93.6101644408874), avg.areasignal = c(529.294117647059, 
527.223880597015, 534.035714285714, 649.363636363636), crit.perc.area = c(11.9556578331072, 
5.38213467658534, 8.44963299743092, 8.11199406041975), mu = c(3.37316736275594, 
1.98133445677709, 2.25592058047569, 2.62699920814759), fillibens = c(0.965653792037269, 
0.994183527375128, 0.992588046586574, 0.996358986581355)), .Names = c("focal.base", 
"avg.percsignal", "avg.areasignal", "crit.perc.area", "mu", "fillibens"
), row.names = c(NA, -4L), class = "data.frame")
```

the data.frame that contains information on the guide region:

```
structure(list(A.area = c(471, 562, 11, 969, 25, 32, 28, 26, 
14, 18, 459, 12, 980, 27, 42, 26, 0, 30, 9, 8, 0, 13, 0, 12, 
7, 403, 12, 13, 547, 11, 401, 8, 14, 0, 18, 17, 448, 19, 46, 
30, 19, 44, 683, 45, 52, 35, 764, 26, 28, 12, 10, 272, 476, 14, 
526, 454, 7, 4, 5, 0, 24, 642, 24, 325, 11, 13, 5, 16, 18, 17, 
25, 0, 25, 548, 20), C.area = c(9, 12, 5, 9, 529, 9, 0, 5, 355, 
614, 9, 11, 9, 561, 480, 8, 10, 10, 10, 13, 17, 0, 13, 8, 497, 
15, 591, 22, 9, 551, 12, 502, 8, 9, 6, 396, 13, 622, 548, 526, 
19, 0, 7, 530, 443, 9, 7, 461, 6, 7, 311, 4, 4, 0, 0, 0, 4, 12, 
10, 432, 6, 8, 503, 7, 15, 401, 17, 480, 589, 372, 18, 399, 13, 
9, 426), G.area = c(3, 7, 446, 8, 5, 14, 999, 649, 9, 21, 9, 
468, 8, 10, 9, 548, 8, 854, 13, 21, 12, 768, 9, 627, 10, 13, 
0, 3, 7, 8, 19, 8, 385, 580, 467, 6, 4, 10, 5, 9, 15, 684, 15, 
0, 28, 412, 5, 8, 297, 575, 10, 6, 12, 378, 18, 13, 302, 5, 9, 
30, 357, 17, 12, 23, 531, 17, 0, 7, 3, 19, 366, 22, 347, 9, 103
), T.area = c(18, 20, 6, 37, 11, 700, 12, 13, 12, 12, 19, 9, 
38, 13, 11, 8, 457, 5, 560, 618, 646, 9, 543, 10, 16, 16, 14, 
688, 10, 20, 16, 12, 10, 2, 0, 0, 19, 0, 13, 15, 616, 10, 24, 
12, 10, 0, 35, 0, 9, 0, 8, 9, 15, 5, 20, 25, 13, 482, 511, 7, 
8, 20, 12, 12, 0, 24, 652, 17, 20, 13, 5, 8, 1, 25, 22), Tot.area = c(501, 
601, 468, 1023, 570, 755, 1039, 693, 390, 665, 496, 500, 1035, 
611, 542, 590, 475, 899, 592, 660, 675, 790, 565, 657, 530, 447, 
617, 726, 573, 590, 448, 530, 417, 591, 491, 419, 484, 651, 612, 
580, 669, 738, 729, 587, 533, 456, 811, 495, 340, 594, 339, 291, 
507, 397, 564, 492, 326, 503, 535, 469, 395, 687, 551, 367, 557, 
455, 674, 520, 630, 421, 414, 429, 386, 591, 571), A.perc = c(94.0119760479042, 
93.5108153078203, 2.35042735042735, 94.7214076246334, 4.3859649122807, 
4.23841059602649, 2.6948989412897, 3.75180375180375, 3.58974358974359, 
2.70676691729323, 92.5403225806452, 2.4, 94.6859903381642, 4.4189852700491, 
7.74907749077491, 4.40677966101695, 0, 3.33704115684093, 1.52027027027027, 
1.21212121212121, 0, 1.64556962025316, 0, 1.82648401826484, 1.32075471698113, 
90.1565995525727, 1.94489465153971, 1.79063360881543, 95.4624781849913, 
1.86440677966102, 89.5089285714286, 1.50943396226415, 3.35731414868106, 
0, 3.66598778004073, 4.05727923627685, 92.5619834710744, 2.91858678955453, 
7.51633986928105, 5.17241379310345, 2.84005979073244, 5.96205962059621, 
93.6899862825789, 7.66609880749574, 9.75609756097561, 7.67543859649123, 
94.2046855733662, 5.25252525252525, 8.23529411764706, 2.02020202020202, 
2.94985250737463, 93.4707903780069, 93.8856015779093, 3.5264483627204, 
93.2624113475177, 92.2764227642276, 2.14723926380368, 0.795228628230616, 
0.934579439252336, 0, 6.07594936708861, 93.4497816593886, 4.3557168784029, 
88.5558583106267, 1.97486535008977, 2.85714285714286, 0.741839762611276, 
3.07692307692308, 2.85714285714286, 4.03800475059382, 6.03864734299517, 
0, 6.47668393782383, 92.7241962774958, 3.50262697022767), C.perc = c(1.79640718562874, 
1.99667221297837, 1.06837606837607, 0.879765395894428, 92.8070175438596, 
1.19205298013245, 0, 0.721500721500722, 91.025641025641, 92.3308270676692, 
1.81451612903226, 2.2, 0.869565217391304, 91.8166939443535, 88.5608856088561, 
1.35593220338983, 2.10526315789474, 1.11234705228031, 1.68918918918919, 
1.96969696969697, 2.51851851851852, 0, 2.30088495575221, 1.21765601217656, 
93.7735849056604, 3.35570469798658, 95.7860615883306, 3.03030303030303, 
1.57068062827225, 93.3898305084746, 2.67857142857143, 94.7169811320755, 
1.91846522781775, 1.52284263959391, 1.22199592668024, 94.5107398568019, 
2.68595041322314, 95.5453149001536, 89.5424836601307, 90.6896551724138, 
2.84005979073244, 0, 0.960219478737997, 90.2896081771721, 83.1144465290807, 
1.97368421052632, 0.863131935881628, 93.1313131313131, 1.76470588235294, 
1.17845117845118, 91.740412979351, 1.3745704467354, 0.788954635108481, 
0, 0, 0, 1.22699386503067, 2.38568588469185, 1.86915887850467, 
92.1108742004264, 1.51898734177215, 1.16448326055313, 91.2885662431942, 
1.90735694822888, 2.69299820466786, 88.1318681318681, 2.52225519287834, 
92.3076923076923, 93.4920634920635, 88.3610451306413, 4.34782608695652, 
93.006993006993, 3.36787564766839, 1.52284263959391, 74.6059544658494
), G.perc = c(0.598802395209581, 1.16472545757072, 95.2991452991453, 
0.782013685239492, 0.87719298245614, 1.85430463576159, 96.1501443695861, 
93.6507936507936, 2.30769230769231, 3.15789473684211, 1.81451612903226, 
93.6, 0.772946859903382, 1.6366612111293, 1.66051660516605, 92.8813559322034, 
1.68421052631579, 94.9944382647386, 2.19594594594595, 3.18181818181818, 
1.77777777777778, 97.2151898734177, 1.5929203539823, 95.4337899543379, 
1.88679245283019, 2.9082774049217, 0, 0.413223140495868, 1.2216404886562, 
1.35593220338983, 4.24107142857143, 1.50943396226415, 92.326139088729, 
98.1387478849408, 95.112016293279, 1.43198090692124, 0.826446280991736, 
1.53609831029186, 0.816993464052288, 1.55172413793103, 2.24215246636771, 
92.6829268292683, 2.05761316872428, 0, 5.25328330206379, 90.3508771929825, 
0.61652281134402, 1.61616161616162, 87.3529411764706, 96.8013468013468, 
2.94985250737463, 2.06185567010309, 2.36686390532544, 95.2141057934509, 
3.19148936170213, 2.64227642276423, 92.638036809816, 0.99403578528827, 
1.68224299065421, 6.39658848614072, 90.379746835443, 2.4745269286754, 
2.17785843920145, 6.26702997275204, 95.3321364452424, 3.73626373626374, 
0, 1.34615384615385, 0.476190476190476, 4.51306413301663, 88.4057971014493, 
5.12820512820513, 89.8963730569948, 1.52284263959391, 18.0385288966725
), T.perc = c(3.59281437125748, 3.32778702163062, 1.28205128205128, 
3.61681329423265, 1.92982456140351, 92.7152317880795, 1.15495668912416, 
1.87590187590188, 3.07692307692308, 1.80451127819549, 3.83064516129032, 
1.8, 3.67149758454106, 2.12765957446809, 2.02952029520295, 1.35593220338983, 
96.2105263157895, 0.556173526140156, 94.5945945945946, 93.6363636363636, 
95.7037037037037, 1.13924050632911, 96.1061946902655, 1.5220700152207, 
3.0188679245283, 3.57941834451902, 2.26904376012966, 94.7658402203857, 
1.74520069808028, 3.38983050847458, 3.57142857142857, 2.26415094339623, 
2.39808153477218, 0.338409475465313, 0, 0, 3.92561983471074, 
0, 2.12418300653595, 2.58620689655172, 92.0777279521674, 1.3550135501355, 
3.29218106995885, 2.0442930153322, 1.87617260787993, 0, 4.31565967940814, 
0, 2.64705882352941, 0, 2.3598820058997, 3.09278350515464, 2.9585798816568, 
1.25944584382872, 3.54609929078014, 5.08130081300813, 3.98773006134969, 
95.8250497017893, 95.5140186915888, 1.49253731343284, 2.0253164556962, 
2.91120815138282, 2.17785843920145, 3.26975476839237, 0, 5.27472527472527, 
96.7359050445104, 3.26923076923077, 3.17460317460317, 3.08788598574822, 
1.20772946859903, 1.86480186480186, 0.259067357512953, 4.23011844331641, 
3.85288966725044), base.call = c("A", "A", "G", "A", "C", "T", 
"G", "G", "C", "C", "A", "G", "A", "C", "C", "G", "T", "G", "T", 
"T", "T", "G", "T", "G", "C", "A", "C", "T", "A", "C", "A", "C", 
"G", "G", "G", "C", "A", "C", "C", "C", "T", "G", "A", "C", "C", 
"G", "A", "C", "G", "G", "C", "A", "A", "G", "A", "A", "G", "T", 
"T", "C", "G", "A", "C", "A", "G", "C", "T", "C", "C", "C", "G", 
"C", "G", "A", "C"), index = 276:350, guide.seq = c("A", "A", 
"G", "A", "C", "T", "G", "G", "C", "C", "A", "G", "A", "C", "C", 
"G", "T", "G", "T", "T", "T", "G", "T", "G", "C", "A", "C", "T", 
"A", "C", "A", "C", "G", "G", "G", "C", "A", "C", "C", "C", "T", 
"G", "A", "C", "C", "G", "A", "C", "G", "G", "C", "A", "A", "G", 
"A", "A", "G", "T", "T", "C", "G", "A", "C", "A", "G", "C", "T", 
"C", "C", "C", "G", "C", "G", "A", "C"), T.pval = c(0.216683719020228, 
0.253696872383191, 0.702824775253092, 0.2135696022127, 0.535068120593795, 
0, 0.735432237619675, 0.548596739990194, 0.293381342100665, 0.566714298987372, 
0.187473978258114, 0.567866432289354, 0.20661594992163, 0.486784211966435, 
0.510453990117528, 0.683588526315973, 0, 0.866594321537338, 0, 
0, 0, 0.739404869695861, 0, 0.640032784938445, 0.303236672784306, 
0.218438719339728, 0.45376716107138, 0, 0.581923840459897, 0.244591260261268, 
0.219491183046174, 0.454887217464385, 0.424839935718337, 0.896657464389295, 
0.913978494580569, 0.913978494580569, 0.176799461623035, 0.913978494580569, 
0.487612581716188, 0.384863211234222, 0, 0.683828544566551, 0.25904741790173, 
0.506854040150182, 0.548528466530182, 0.913978494580569, 0.138363984753014, 
0.913978494580569, 0.372507754171374, 0.913978494580569, 0.433278792221022, 
0.290733397531259, 0.313743461743047, 0.708676880501944, 0.222856163497884, 
0.0840178134264059, 0.170109655427717, 0, 0, 0.647781688236816, 
0.511480735047821, 0.322195195414723, 0.474909610385191, 0.26246466793089, 
0.913978494580569, 0.0738339354931401, 0, 0.262544951646865, 
0.277374200197982, 0.291549021974569, 0.721987045883744, 0.551398878994113, 
0.904113083125409, 0.146089711947169, 0.184924759533489), C.pval = c(0.451052199428269, 
0.384646653398073, 0.721169711339068, 0.78586155546188, 0, 0.675558719149989, 
0.916666666593762, 0.832852922881948, 0, 0, 0.444804552242467, 
0.323828091032268, 0.789123394739055, 0, 0, 0.613407556489433, 
0.351296788905976, 0.705159768689193, 0.488920185576654, 0.393231376020402, 
0.242811616635787, 0.916666666593762, 0.296277083783286, 0.665930166471372, 
0, 0.104775422558449, 0, 0.147072449971626, 0.53228545451366, 
0, 0.208552462506761, 0, 0.409853757950083, 0.550145439209618, 
0.664293571285672, 0, 0.207072545832584, 0, 0, 0, 0.178084423171449, 
0.916666666593762, 0.759196686091719, 0, 0, 0.39195509638794, 
0.791165760211691, 0, 0.462096173833688, 0.680653477335871, 0, 
0.606304396390714, 0.813823759260488, 0.916666666593762, 0.916666666593762, 
0.916666666593762, 0.66240736963565, 0.274480205632598, 0.426233462381063, 
0, 0.551591972492065, 0.685868699254128, 0, 0.41351178686846, 
0.205667065326033, 0, 0.241963824973631, 0, 0, 0, 0.0346084476014255, 
0, 0.103427335660354, 0.550145439209618, 0), G.pval = c(0.792942991313963, 
0.625488867523247, 0, 0.737978311297745, 0.709450418977129, 0.449011759922408, 
0, 0, 0.355664335841162, 0.225022600557276, 0.458070009533757, 
0, 0.740703919178624, 0.500292875149647, 0.494465671916797, 0, 
0.488727999150991, 0, 0.377015791497866, 0.222075000256262, 0.466559825117588, 
0, 0.511108442021009, 0, 0.441720698726068, 0.257981630421168, 
0.934065934065934, 0.84689987834141, 0.609456915662282, 0.572583475135495, 
0.122348183285009, 0.532217573283385, 0, 0, 0, 0.552338462469495, 
0.724637045719961, 0.525409657931421, 0.727472586398918, 0.521448758351368, 
0.368058869415009, 0, 0.404930544346893, 0.934065934065934, 0.0680350075807965, 
0, 0.787660458980629, 0.505340654760887, 0, 0, 0.252204348849495, 
0.404049656869183, 0.344782412735587, 0, 0.220893509706952, 0.29784865812777, 
0, 0.674820487124049, 0.489202549932742, 0.034577950433238, 0, 
0.325718410139018, 0.380571671616088, 0.0373568012826258, 0, 
0.162997940218854, 0.934065934065934, 0.575220841465795, 0.82896206404497, 
0.104643904420715, 0, 0.0732040841732349, 0, 0.528786391285889, 
2.48767489035018e-05), A.pval = c(0, 0, 0.482263378317416, 0, 
0.231966039702189, 0.245339785411823, 0.429391200671223, 0.294350084208411, 
0.31243692897805, 0.427646420055972, 0, 0.474393708629254, 0, 
0.229063309786373, 0.0602099924535682, 0.230132484395735, 0.854545454545455, 
0.34249144011364, 0.625077890371162, 0.68140065356242, 0.854545454545455, 
0.602445037754296, 0.854545454545455, 0.570300937820873, 0.661502703697975, 
0, 0.549689518197863, 0.576612044341363, 0, 0.563659756124424, 
0, 0.627046336557289, 0.339995569974992, 0.854545454545455, 0.303813960239671, 
0.262682329839595, 0, 0.397383328296701, 0.066317167663941, 0.171107245129608, 
0.408408244549401, 0.125067875191041, 0, 0.0623226830541985, 
0.0258255695537792, 0.0620813745746311, 0, 0.165806613069444, 
0.0491493679478258, 0.536781944781286, 0.393057611125132, 0, 
0, 0.31975069985057, 0, 0, 0.515392431266109, 0.756114116994236, 
0.731675397310131, 0.854545454545455, 0.119473659547617, 0, 0.234653710052841, 
0, 0.544533064728061, 0.405990315924465, 0.765234592833787, 0.375851207735946, 
0.405990315924465, 0.264589410504281, 0.121279527609496, 0.854545454545455, 
0.101604930904666, 0, 0.32254004756413), guide.position = 1:75), .Names = c("A.area", 
"C.area", "G.area", "T.area", "Tot.area", "A.perc", "C.perc", 
"G.perc", "T.perc", "base.call", "index", "guide.seq", "T.pval", 
"C.pval", "G.pval", "A.pval", "guide.position"), row.names = 276:350, class = "data.frame")
```

*Report generated using EditR v1.0.8*
