# Supplementary material for: Mechanistic and genetic basis of single-strand templated repair at Cas12a-induced DNA breaks in Chlamydomonas reinhardtii
Source: Nat Commun. 2021 Nov 19;12:6751. doi: 10.1038/s41467-021-27004-1 (PMC8604939; doi:10.1038/s41467-021-27004-1)
Supplement: Supplementary file 22 — Source Data [file 41467_2021_27004_MOESM22_ESM.zip › Source Data/EditR analysis/EditR outputs/Antisense/rep1_ssODN_antisense_-32.html]

EditR v1.0.8 report


# EditR v1.0.8 report

- Data QA
  - Filtering data
  - Percent noise peak area
  - Base information
- Predicted editing
  - Editing bar plot
  - Editing table plot
  - Table of editing results
- For use in R

## Data QA

### Filtering data

What the data looked like prefiltering:

and the post filtering signal / noise plot:

### Percent noise peak area

### Base information

Here’s information about the signal of each base, the critical percent value where any higher value would be called as significant, and Filliben’s correlation for how well the noise was modelled by the zero adjusted gamma distribution.

| Base | Average percent signal | Average peak area | Critical percent value | model mu | Fillibens correlation |
| --- | --- | --- | --- | --- | --- |
| A | 95.26383 | 299.8438 | 5.604304 | 1.714259 | 0.9894380 |
| C | 94.64183 | 313.4638 | 4.795851 | 1.883770 | 0.9876276 |
| G | 95.28383 | 305.0351 | 4.764226 | 1.739034 | 0.9944006 |
| T | 94.95155 | 313.9535 | 5.358131 | 1.946202 | 0.9948532 |

## Predicted editing

### Editing bar plot

### Editing table plot

### Table of editing results


Here’s the entire guide region

| Sanger position | Guide position | Guide sequence | Sanger base call | Focal base | Focal base peak area | p value |  |
| --- | --- | --- | --- | --- | --- | --- | --- |
| 282 | 1 | A | A | A | 96.99 | 0.0000000000 | \* |
| 282 | 1 | A | A | C | 2.63 | 0.1802739376 |  |
| 282 | 1 | A | A | G | 0.00 | 0.9222222222 |  |
| 282 | 1 | A | A | T | 0.38 | 0.9388311719 |  |
| 283 | 2 | A | A | A | 96.36 | 0.0000000000 | \* |
| 283 | 2 | A | A | C | 1.66 | 0.4822994019 |  |
| 283 | 2 | A | A | G | 1.66 | 0.4220462909 |  |
| 283 | 2 | A | A | T | 0.33 | 0.9441900241 |  |
| 284 | 3 | G | G | A | 4.15 | 0.0414581521 |  |
| 284 | 3 | G | G | C | 1.38 | 0.5961711954 |  |
| 284 | 3 | G | G | G | 93.55 | 0.0000000000 | \* |
| 284 | 3 | G | G | T | 0.92 | 0.7978357708 |  |
| 285 | 4 | A | A | A | 96.84 | 0.0000000000 | \* |
| 285 | 4 | A | A | C | 1.23 | 0.6603857935 |  |
| 285 | 4 | A | A | G | 1.05 | 0.6731129475 |  |
| 285 | 4 | A | A | T | 0.88 | 0.8129826289 |  |
| 286 | 5 | C | C | A | 3.36 | 0.0863196881 |  |
| 286 | 5 | C | C | C | 92.97 | 0.0000000000 | \* |
| 286 | 5 | C | C | G | 1.83 | 0.3575349822 |  |
| 286 | 5 | C | C | T | 1.83 | 0.4446126692 |  |
| 287 | 6 | T | T | A | 2.80 | 0.1437874606 |  |
| 287 | 6 | T | T | C | 2.10 | 0.3206582474 |  |
| 287 | 6 | T | T | G | 4.20 | 0.0217104749 |  |
| 287 | 6 | T | T | T | 90.91 | 0.0000000000 | \* |
| 288 | 7 | G | G | A | 1.56 | 0.3978016773 |  |
| 288 | 7 | G | G | C | 0.35 | 0.9063802408 |  |
| 288 | 7 | G | G | G | 97.22 | 0.0000000000 | \* |
| 288 | 7 | G | G | T | 0.87 | 0.8166749063 |  |
| 289 | 8 | G | G | A | 1.97 | 0.2888487025 |  |
| 289 | 8 | G | G | C | 1.64 | 0.4867057705 |  |
| 289 | 8 | G | G | G | 96.05 | 0.0000000000 | \* |
| 289 | 8 | G | G | T | 0.33 | 0.9444242468 |  |
| 290 | 9 | C | C | A | 0.90 | 0.6227915134 |  |
| 290 | 9 | C | C | C | 95.02 | 0.0000000000 | \* |
| 290 | 9 | C | C | G | 1.81 | 0.3660950938 |  |
| 290 | 9 | C | C | T | 2.26 | 0.3090459417 |  |
| 291 | 10 | C | C | A | 2.08 | 0.2655807807 |  |
| 291 | 10 | C | C | C | 95.55 | 0.0000000000 | \* |
| 291 | 10 | C | C | G | 1.19 | 0.6157985083 |  |
| 291 | 10 | C | C | T | 1.19 | 0.6963935745 |  |
| 292 | 11 | A | A | A | 87.65 | 0.0000000000 | \* |
| 292 | 11 | A | A | C | 2.39 | 0.2361407437 |  |
| 292 | 11 | A | A | G | 1.59 | 0.4458630415 |  |
| 292 | 11 | A | A | T | 8.37 | 0.0001969207 | \* |
| 293 | 12 | G | G | A | 3.72 | 0.0620142396 |  |
| 293 | 12 | G | G | C | 0.93 | 0.7775379327 |  |
| 293 | 12 | G | G | G | 95.35 | 0.0000000000 | \* |
| 293 | 12 | G | G | T | 0.00 | 0.9574468085 |  |
| 294 | 13 | A | A | A | 97.07 | 0.0000000000 | \* |
| 294 | 13 | A | A | C | 1.37 | 0.6026588416 |  |
| 294 | 13 | A | A | G | 0.59 | 0.8495967227 |  |
| 294 | 13 | A | A | T | 0.98 | 0.7778175861 |  |
| 295 | 14 | C | C | A | 2.74 | 0.1517999281 |  |
| 295 | 14 | C | C | C | 94.83 | 0.0000000000 | \* |
| 295 | 14 | C | C | G | 0.61 | 0.8431058406 |  |
| 295 | 14 | C | C | T | 1.82 | 0.4485720430 |  |
| 296 | 15 | C | C | A | 1.99 | 0.2846046787 |  |
| 296 | 15 | C | C | C | 92.83 | 0.0000000000 | \* |
| 296 | 15 | C | C | G | 2.39 | 0.2019599181 |  |
| 296 | 15 | C | C | T | 2.79 | 0.1873069462 |  |
| 297 | 16 | G | G | A | 2.85 | 0.1374627845 |  |
| 297 | 16 | G | G | C | 1.27 | 0.6454892844 |  |
| 297 | 16 | G | G | G | 93.99 | 0.0000000000 | \* |
| 297 | 16 | G | G | T | 1.90 | 0.4223268898 |  |
| 298 | 17 | T | T | A | 2.09 | 0.2623620554 |  |
| 298 | 17 | T | T | C | 0.42 | 0.8997867124 |  |
| 298 | 17 | T | T | G | 2.09 | 0.2771437249 |  |
| 298 | 17 | T | T | T | 95.40 | 0.0000000000 | \* |
| 299 | 18 | G | G | A | 1.06 | 0.5665699348 |  |
| 299 | 18 | G | G | C | 0.21 | 0.9130350070 |  |
| 299 | 18 | G | G | G | 98.10 | 0.0000000000 | \* |
| 299 | 18 | G | G | T | 0.63 | 0.8879573306 |  |
| 300 | 19 | T | T | A | 0.00 | 0.8750000000 |  |
| 300 | 19 | T | T | C | 1.56 | 0.5204362399 |  |
| 300 | 19 | T | T | G | 3.52 | 0.0528436466 |  |
| 300 | 19 | T | T | T | 94.92 | 0.0000000000 | \* |
| 301 | 20 | T | T | A | 0.33 | 0.8222644819 |  |
| 301 | 20 | T | T | C | 1.66 | 0.4822994019 |  |
| 301 | 20 | T | T | G | 0.33 | 0.9048639693 |  |
| 301 | 20 | T | T | T | 97.68 | 0.0000000000 | \* |
| 302 | 21 | T | T | A | 0.00 | 0.8750000000 |  |
| 302 | 21 | T | T | C | 1.98 | 0.3599371234 |  |
| 302 | 21 | T | T | G | 2.31 | 0.2203318712 |  |
| 302 | 21 | T | T | T | 95.71 | 0.0000000000 | \* |
| 303 | 22 | G | G | A | 1.03 | 0.5751524983 |  |
| 303 | 22 | G | G | C | 0.00 | 0.9146341463 |  |
| 303 | 22 | G | G | G | 98.45 | 0.0000000000 | \* |
| 303 | 22 | G | G | T | 0.52 | 0.9152106078 |  |
| 304 | 23 | T | T | A | 0.36 | 0.8138731966 |  |
| 304 | 23 | T | T | C | 2.53 | 0.2030151453 |  |
| 304 | 23 | T | T | G | 1.08 | 0.6608279173 |  |
| 304 | 23 | T | T | T | 96.03 | 0.0000000000 | \* |
| 305 | 24 | G | G | A | 1.96 | 0.2918633992 |  |
| 305 | 24 | G | G | C | 1.68 | 0.4722294289 |  |
| 305 | 24 | G | G | G | 94.96 | 0.0000000000 | \* |
| 305 | 24 | G | G | T | 1.40 | 0.6105305730 |  |
| 306 | 25 | C | C | A | 1.64 | 0.3754014441 |  |
| 306 | 25 | C | C | C | 93.44 | 0.0000000000 | \* |
| 306 | 25 | C | C | G | 1.97 | 0.3143317063 |  |
| 306 | 25 | C | C | T | 2.95 | 0.1590388415 |  |
| 307 | 26 | A | A | A | 94.76 | 0.0000000000 | \* |
| 307 | 26 | A | A | C | 4.37 | 0.0186381881 |  |
| 307 | 26 | A | A | G | 0.87 | 0.7486027473 |  |
| 307 | 26 | A | A | T | 0.00 | 0.9574468085 |  |
| 308 | 27 | C | C | A | 1.51 | 0.4148573896 |  |
| 308 | 27 | C | C | C | 96.08 | 0.0000000000 | \* |
| 308 | 27 | C | C | G | 0.00 | 0.9222222222 |  |
| 308 | 27 | C | C | T | 2.41 | 0.2700800812 |  |
| 309 | 28 | T | T | A | 2.88 | 0.1330827468 |  |
| 309 | 28 | T | T | C | 2.56 | 0.1947067649 |  |
| 309 | 28 | T | T | G | 0.00 | 0.9222222222 |  |
| 309 | 28 | T | T | T | 94.55 | 0.0000000000 | \* |
| 310 | 29 | A | A | A | 94.86 | 0.0000000000 | \* |
| 310 | 29 | A | A | C | 1.71 | 0.4588546742 |  |
| 310 | 29 | A | A | G | 1.71 | 0.4002026664 |  |
| 310 | 29 | A | A | T | 1.71 | 0.4884144033 |  |
| 311 | 30 | C | C | A | 1.91 | 0.3037798649 |  |
| 311 | 30 | C | C | C | 92.99 | 0.0000000000 | \* |
| 311 | 30 | C | C | G | 2.23 | 0.2402162738 |  |
| 311 | 30 | C | C | T | 2.87 | 0.1733089414 |  |
| 312 | 31 | A | A | A | 93.28 | 0.0000000000 | \* |
| 312 | 31 | A | A | C | 2.52 | 0.2044033051 |  |
| 312 | 31 | A | A | G | 2.94 | 0.1074017984 |  |
| 312 | 31 | A | A | T | 1.26 | 0.6669136646 |  |
| 313 | 32 | C | C | A | 1.41 | 0.4454823189 |  |
| 313 | 32 | C | C | C | 93.66 | 0.0000000000 | \* |
| 313 | 32 | C | C | G | 2.11 | 0.2713318122 |  |
| 313 | 32 | C | C | T | 2.82 | 0.1821263083 |  |
| 314 | 33 | G | G | A | 3.08 | 0.1120515813 |  |
| 314 | 33 | G | G | C | 2.31 | 0.2581422897 |  |
| 314 | 33 | G | G | G | 94.23 | 0.0000000000 | \* |
| 314 | 33 | G | G | T | 0.38 | 0.9376736797 |  |
| 315 | 34 | G | G | A | 1.54 | 0.4050003399 |  |
| 315 | 34 | G | G | C | 1.54 | 0.5304298896 |  |
| 315 | 34 | G | G | G | 96.62 | 0.0000000000 | \* |
| 315 | 34 | G | G | T | 0.31 | 0.9465835048 |  |
| 316 | 35 | G | G | A | 1.26 | 0.4963778880 |  |
| 316 | 35 | G | G | C | 2.09 | 0.3225406733 |  |
| 316 | 35 | G | G | G | 95.82 | 0.0000000000 | \* |
| 316 | 35 | G | G | T | 0.84 | 0.8272871731 |  |
| 317 | 36 | C | C | A | 1.53 | 0.4085501526 |  |
| 317 | 36 | C | C | C | 94.66 | 0.0000000000 | \* |
| 317 | 36 | C | C | G | 1.53 | 0.4723441735 |  |
| 317 | 36 | C | C | T | 2.29 | 0.3014275939 |  |
| 318 | 37 | A | A | A | 94.44 | 0.0000000000 | \* |
| 318 | 37 | A | A | C | 2.78 | 0.1520078165 |  |
| 318 | 37 | A | A | G | 0.40 | 0.8943806369 |  |
| 318 | 37 | A | A | T | 2.38 | 0.2773631905 |  |
| 319 | 38 | C | C | A | 1.51 | 0.4134648955 |  |
| 319 | 38 | C | C | C | 96.07 | 0.0000000000 | \* |
| 319 | 38 | C | C | G | 0.60 | 0.8442082124 |  |
| 319 | 38 | C | C | T | 1.81 | 0.4525027785 |  |
| 320 | 39 | C | C | A | 3.46 | 0.0790829556 |  |
| 320 | 39 | C | C | C | 93.40 | 0.0000000000 | \* |
| 320 | 39 | C | C | G | 0.63 | 0.8366672519 |  |
| 320 | 39 | C | C | T | 2.52 | 0.2444312473 |  |
| 321 | 40 | C | C | A | 3.17 | 0.1025854770 |  |
| 321 | 40 | C | C | C | 93.02 | 0.0000000000 | \* |
| 321 | 40 | C | C | G | 0.63 | 0.8347941845 |  |
| 321 | 40 | C | C | T | 3.17 | 0.1260703513 |  |
| 322 | 41 | T | T | A | 2.38 | 0.2061743628 |  |
| 322 | 41 | T | T | C | 2.72 | 0.1624941968 |  |
| 322 | 41 | T | T | G | 2.72 | 0.1391358277 |  |
| 322 | 41 | T | T | T | 92.18 | 0.0000000000 | \* |
| 323 | 42 | G | G | A | 1.57 | 0.3967624533 |  |
| 323 | 42 | G | G | C | 0.89 | 0.7897833206 |  |
| 323 | 42 | G | G | G | 96.42 | 0.0000000000 | \* |
| 323 | 42 | G | G | T | 1.12 | 0.7234668737 |  |
| 324 | 43 | A | A | A | 94.63 | 0.0000000000 | \* |
| 324 | 43 | A | A | C | 1.69 | 0.4665417781 |  |
| 324 | 43 | A | A | G | 2.82 | 0.1232714829 |  |
| 324 | 43 | A | A | T | 0.85 | 0.8237052465 |  |
| 325 | 44 | C | C | A | 1.99 | 0.2858200295 |  |
| 325 | 44 | C | C | C | 94.70 | 0.0000000000 | \* |
| 325 | 44 | C | C | G | 0.99 | 0.6991444452 |  |
| 325 | 44 | C | C | T | 2.32 | 0.2939039751 |  |
| 326 | 45 | C | C | A | 4.73 | 0.0237198195 |  |
| 326 | 45 | C | C | C | 91.27 | 0.0000000000 | \* |
| 326 | 45 | C | C | G | 1.82 | 0.3632528208 |  |
| 326 | 45 | C | C | T | 2.18 | 0.3320802692 |  |
| 327 | 46 | G | G | A | 3.80 | 0.0574641071 |  |
| 327 | 46 | G | G | C | 1.90 | 0.3879457481 |  |
| 327 | 46 | G | G | G | 94.30 | 0.0000000000 | \* |
| 327 | 46 | G | G | T | 0.00 | 0.9574468085 |  |
| 328 | 47 | A | A | A | 96.93 | 0.0000000000 | \* |
| 328 | 47 | A | A | C | 1.28 | 0.6400449236 |  |
| 328 | 47 | A | A | G | 1.28 | 0.5760469856 |  |
| 328 | 47 | A | A | T | 0.51 | 0.9162806908 |  |
| 329 | 48 | C | C | A | 2.61 | 0.1690944191 |  |
| 329 | 48 | C | C | C | 94.40 | 0.0000000000 | \* |
| 329 | 48 | C | C | G | 0.75 | 0.7971016472 |  |
| 329 | 48 | C | C | T | 2.24 | 0.3156746261 |  |
| 330 | 49 | G | G | A | 8.09 | 0.0007926935 | \* |
| 330 | 49 | G | G | C | 2.31 | 0.2569220980 |  |
| 330 | 49 | G | G | G | 89.60 | 0.0000000000 | \* |
| 330 | 49 | G | G | T | 0.00 | 0.9574468085 |  |
| 331 | 50 | G | G | A | 1.03 | 0.5761285814 |  |
| 331 | 50 | G | G | C | 1.03 | 0.7405056691 |  |
| 331 | 50 | G | G | G | 97.25 | 0.0000000000 | \* |
| 331 | 50 | G | G | T | 0.69 | 0.8736044057 |  |
| 332 | 51 | C | C | A | 1.57 | 0.3953725848 |  |
| 332 | 51 | C | C | C | 93.72 | 0.0000000000 | \* |
| 332 | 51 | C | C | G | 2.09 | 0.2765220039 |  |
| 332 | 51 | C | C | T | 2.62 | 0.2216179766 |  |
| 333 | 52 | A | A | A | 92.44 | 0.0000000000 | \* |
| 333 | 52 | A | A | C | 2.52 | 0.2044033051 |  |
| 333 | 52 | A | A | G | 1.68 | 0.4126362663 |  |
| 333 | 52 | A | A | T | 3.36 | 0.1033276783 |  |
| 334 | 53 | A | A | A | 95.74 | 0.0000000000 | \* |
| 334 | 53 | A | A | C | 1.94 | 0.3747348373 |  |
| 334 | 53 | A | A | G | 1.55 | 0.4628877116 |  |
| 334 | 53 | A | A | T | 0.78 | 0.8473344266 |  |
| 335 | 54 | G | G | A | 1.17 | 0.5261790521 |  |
| 335 | 54 | G | G | C | 0.00 | 0.9146341463 |  |
| 335 | 54 | G | G | G | 97.08 | 0.0000000000 | \* |
| 335 | 54 | G | G | T | 1.75 | 0.4736089743 |  |
| 336 | 55 | A | A | A | 98.45 | 0.0000000000 | \* |
| 336 | 55 | A | A | C | 0.00 | 0.9146341463 |  |
| 336 | 55 | A | A | G | 0.93 | 0.7261005331 |  |
| 336 | 55 | A | A | T | 0.62 | 0.8918092344 |  |
| 337 | 56 | A | A | A | 97.36 | 0.0000000000 | \* |
| 337 | 56 | A | A | C | 0.38 | 0.9038819543 |  |
| 337 | 56 | A | A | G | 0.75 | 0.7940394765 |  |
| 337 | 56 | A | A | T | 1.51 | 0.5671517943 |  |
| 338 | 57 | G | G | A | 2.70 | 0.1562342336 |  |
| 338 | 57 | G | G | C | 0.68 | 0.8543047988 |  |
| 338 | 57 | G | G | G | 93.24 | 0.0000000000 | \* |
| 338 | 57 | G | G | T | 3.38 | 0.1014477974 |  |
| 339 | 58 | T | T | A | 1.14 | 0.5379727739 |  |
| 339 | 58 | T | T | C | 1.14 | 0.6989785636 |  |
| 339 | 58 | T | T | G | 0.76 | 0.7929974656 |  |
| 339 | 58 | T | T | T | 96.97 | 0.0000000000 | \* |
| 340 | 59 | T | T | A | 0.73 | 0.6881993051 |  |
| 340 | 59 | T | T | C | 1.82 | 0.4161531875 |  |
| 340 | 59 | T | T | G | 1.09 | 0.6557091454 |  |
| 340 | 59 | T | T | T | 96.35 | 0.0000000000 | \* |
| 341 | 60 | C | C | A | 0.75 | 0.6789823241 |  |
| 341 | 60 | C | C | C | 95.47 | 0.0000000000 | \* |
| 341 | 60 | C | C | G | 1.13 | 0.6396124480 |  |
| 341 | 60 | C | C | T | 2.64 | 0.2165744923 |  |
| 342 | 61 | G | G | A | 4.35 | 0.0342281098 |  |
| 342 | 61 | G | G | C | 3.11 | 0.1019907935 |  |
| 342 | 61 | G | G | G | 91.93 | 0.0000000000 | \* |
| 342 | 61 | G | G | T | 0.62 | 0.8913228559 |  |
| 343 | 62 | A | A | A | 96.79 | 0.0000000000 | \* |
| 343 | 62 | A | A | C | 1.75 | 0.4451180725 |  |
| 343 | 62 | A | A | G | 1.17 | 0.6248130118 |  |
| 343 | 62 | A | A | T | 0.29 | 0.9480727675 |  |
| 344 | 63 | C | C | A | 2.82 | 0.1413089724 |  |
| 344 | 63 | C | C | C | 94.37 | 0.0000000000 | \* |
| 344 | 63 | C | C | G | 0.35 | 0.9018101517 |  |
| 344 | 63 | C | C | T | 2.46 | 0.2564953688 |  |
| 345 | 64 | A | A | A | 94.63 | 0.0000000000 | \* |
| 345 | 64 | A | A | C | 2.93 | 0.1271288570 |  |
| 345 | 64 | A | A | G | 1.95 | 0.3193465252 |  |
| 345 | 64 | A | A | T | 0.49 | 0.9209055859 |  |
| 346 | 65 | G | G | A | 1.24 | 0.5017252974 |  |
| 346 | 65 | G | G | C | 2.48 | 0.2141424350 |  |
| 346 | 65 | G | G | G | 96.28 | 0.0000000000 | \* |
| 346 | 65 | G | G | T | 0.00 | 0.9574468085 |  |
| 347 | 66 | C | C | A | 0.80 | 0.6608736817 |  |
| 347 | 66 | C | C | C | 95.18 | 0.0000000000 | \* |
| 347 | 66 | C | C | G | 1.61 | 0.4408872974 |  |
| 347 | 66 | C | C | T | 2.41 | 0.2700800812 |  |
| 348 | 67 | T | T | A | 0.66 | 0.7123253804 |  |
| 348 | 67 | T | T | C | 1.00 | 0.7534325815 |  |
| 348 | 67 | T | T | G | 0.33 | 0.9047107602 |  |
| 348 | 67 | T | T | T | 98.01 | 0.0000000000 | \* |
| 349 | 68 | C | C | A | 0.00 | 0.8750000000 |  |
| 349 | 68 | C | C | C | 96.44 | 0.0000000000 | \* |
| 349 | 68 | C | C | G | 1.42 | 0.5146057121 |  |
| 349 | 68 | C | C | T | 2.14 | 0.3459368713 |  |
| 350 | 69 | C | C | A | 0.65 | 0.7185937395 |  |
| 350 | 69 | C | C | C | 95.47 | 0.0000000000 | \* |
| 350 | 69 | C | C | G | 1.62 | 0.4363667235 |  |
| 350 | 69 | C | C | T | 2.27 | 0.3082318198 |  |
| 351 | 70 | C | C | A | 0.00 | 0.8750000000 |  |
| 351 | 70 | C | C | C | 95.17 | 0.0000000000 | \* |
| 351 | 70 | C | C | G | 1.93 | 0.3253307324 |  |
| 351 | 70 | C | C | T | 2.90 | 0.1677338314 |  |
| 352 | 71 | G | G | A | 2.73 | 0.1529077345 |  |
| 352 | 71 | G | G | C | 0.91 | 0.7849084014 |  |
| 352 | 71 | G | G | G | 96.36 | 0.0000000000 | \* |
| 352 | 71 | G | G | T | 0.00 | 0.9574468085 |  |
| 353 | 72 | C | C | A | 0.82 | 0.6547137736 |  |
| 353 | 72 | C | C | C | 95.90 | 0.0000000000 | \* |
| 353 | 72 | C | C | G | 1.23 | 0.5973355679 |  |
| 353 | 72 | C | C | T | 2.05 | 0.3725767697 |  |
| 354 | 73 | G | G | A | 3.74 | 0.0610139056 |  |
| 354 | 73 | G | G | C | 1.87 | 0.3996304371 |  |
| 354 | 73 | G | G | G | 93.46 | 0.0000000000 | \* |
| 354 | 73 | G | G | T | 0.93 | 0.7931863463 |  |
| 355 | 74 | A | A | A | 95.33 | 0.0000000000 | \* |
| 355 | 74 | A | A | C | 1.56 | 0.5224555626 |  |
| 355 | 74 | A | A | G | 1.25 | 0.5901504037 |  |
| 355 | 74 | A | A | T | 1.87 | 0.4325632370 |  |
| 356 | 75 | C | C | A | 1.70 | 0.3581956770 |  |
| 356 | 75 | C | C | C | 91.50 | 0.0000000000 | \* |
| 356 | 75 | C | C | G | 1.70 | 0.4052078159 |  |
| 356 | 75 | C | C | T | 5.10 | 0.0137162554 |  |

## For use in R

If you want to work with the results in R, here is output that you can copy and paste in your terminal to get:

The base information:

```
structure(list(focal.base = c("A", "C", "G", "T"), avg.percsignal = c(95.2638315154935, 
94.6418336387441, 95.2838317986101, 94.951552954557), avg.areasignal = c(299.84375, 
313.463768115942, 305.035087719298, 313.953488372093), crit.perc.area = c(5.60430394085162, 
4.79585065136648, 4.76422588556689, 5.35813128914901), mu = c(1.7142590750905, 
1.88377027918769, 1.73903378878017, 1.94620177062478), fillibens = c(0.989437998127868, 
0.987627608636948, 0.994400570129443, 0.994853212532975)), .Names = c("focal.base", 
"avg.percsignal", "avg.areasignal", "crit.perc.area", "mu", "fillibens"
), row.names = c(NA, -4L), class = "data.frame")
```

the data.frame that contains information on the guide region:

```
structure(list(A.area = c(258, 291, 9, 551, 11, 8, 9, 6, 2, 7, 
220, 8, 497, 9, 5, 9, 5, 5, 0, 1, 0, 4, 1, 7, 5, 217, 5, 9, 332, 
6, 222, 4, 8, 5, 3, 4, 238, 5, 11, 10, 7, 7, 335, 6, 13, 10, 
379, 7, 14, 3, 3, 110, 247, 2, 318, 258, 4, 3, 2, 2, 7, 332, 
8, 194, 3, 2, 2, 0, 2, 0, 6, 2, 8, 306, 5), C.area = c(7, 5, 
3, 7, 304, 6, 2, 5, 210, 322, 6, 2, 7, 312, 233, 4, 1, 1, 4, 
5, 6, 0, 7, 6, 285, 10, 319, 8, 6, 292, 6, 266, 6, 5, 5, 248, 
7, 318, 297, 293, 8, 4, 6, 286, 251, 5, 5, 253, 4, 3, 179, 3, 
5, 0, 0, 1, 1, 3, 5, 253, 5, 6, 268, 6, 6, 237, 3, 271, 295, 
197, 2, 234, 4, 5, 269), G.area = c(0, 5, 203, 6, 6, 12, 560, 
292, 4, 4, 4, 205, 3, 2, 6, 297, 5, 464, 9, 1, 7, 381, 3, 339, 
6, 2, 0, 0, 6, 7, 7, 6, 245, 314, 229, 4, 1, 2, 2, 2, 8, 431, 
10, 3, 5, 248, 5, 2, 155, 283, 4, 2, 4, 166, 3, 2, 138, 2, 3, 
3, 148, 4, 1, 4, 233, 4, 1, 4, 5, 4, 212, 3, 200, 4, 5), T.area = c(1, 
1, 2, 5, 6, 260, 5, 1, 5, 4, 21, 0, 5, 6, 7, 6, 228, 3, 243, 
295, 290, 2, 266, 5, 9, 0, 8, 295, 6, 9, 3, 8, 1, 1, 2, 6, 6, 
6, 8, 10, 271, 5, 3, 7, 6, 0, 2, 6, 0, 2, 5, 4, 2, 3, 2, 4, 5, 
256, 264, 7, 1, 1, 7, 1, 0, 6, 295, 6, 7, 6, 0, 5, 2, 6, 15), 
    Tot.area = c(266, 302, 217, 569, 327, 286, 576, 304, 221, 
    337, 251, 215, 512, 329, 251, 316, 239, 473, 256, 302, 303, 
    387, 277, 357, 305, 229, 332, 312, 350, 314, 238, 284, 260, 
    325, 239, 262, 252, 331, 318, 315, 294, 447, 354, 302, 275, 
    263, 391, 268, 173, 291, 191, 119, 258, 171, 323, 265, 148, 
    264, 274, 265, 161, 343, 284, 205, 242, 249, 301, 281, 309, 
    207, 220, 244, 214, 321, 294), A.perc = c(96.9924812030075, 
    96.3576158940397, 4.14746543778802, 96.8365553602812, 3.36391437308869, 
    2.7972027972028, 1.5625, 1.97368421052632, 0.904977375565611, 
    2.07715133531157, 87.6494023904382, 3.72093023255814, 97.0703125, 
    2.73556231003039, 1.99203187250996, 2.84810126582278, 2.09205020920502, 
    1.05708245243129, 0, 0.33112582781457, 0, 1.03359173126615, 
    0.36101083032491, 1.96078431372549, 1.63934426229508, 94.7598253275109, 
    1.50602409638554, 2.88461538461538, 94.8571428571429, 1.91082802547771, 
    93.2773109243697, 1.40845070422535, 3.07692307692308, 1.53846153846154, 
    1.25523012552301, 1.52671755725191, 94.4444444444444, 1.51057401812689, 
    3.45911949685535, 3.17460317460317, 2.38095238095238, 1.56599552572707, 
    94.6327683615819, 1.98675496688742, 4.72727272727273, 3.80228136882129, 
    96.9309462915601, 2.61194029850746, 8.09248554913295, 1.03092783505155, 
    1.57068062827225, 92.436974789916, 95.7364341085271, 1.16959064327485, 
    98.4520123839009, 97.3584905660377, 2.7027027027027, 1.13636363636364, 
    0.72992700729927, 0.754716981132076, 4.34782608695652, 96.7930029154519, 
    2.8169014084507, 94.6341463414634, 1.2396694214876, 0.803212851405622, 
    0.664451827242525, 0, 0.647249190938511, 0, 2.72727272727273, 
    0.819672131147541, 3.73831775700935, 95.3271028037383, 1.70068027210884
    ), C.perc = c(2.63157894736842, 1.65562913907285, 1.38248847926267, 
    1.23022847100176, 92.9663608562691, 2.0979020979021, 0.347222222222222, 
    1.64473684210526, 95.0226244343891, 95.5489614243323, 2.39043824701195, 
    0.930232558139535, 1.3671875, 94.8328267477204, 92.8286852589641, 
    1.26582278481013, 0.418410041841004, 0.211416490486258, 1.5625, 
    1.65562913907285, 1.98019801980198, 0, 2.52707581227437, 
    1.68067226890756, 93.4426229508197, 4.36681222707424, 96.0843373493976, 
    2.56410256410256, 1.71428571428571, 92.9936305732484, 2.52100840336134, 
    93.6619718309859, 2.30769230769231, 1.53846153846154, 2.09205020920502, 
    94.6564885496183, 2.77777777777778, 96.0725075528701, 93.3962264150943, 
    93.015873015873, 2.72108843537415, 0.894854586129754, 1.69491525423729, 
    94.7019867549669, 91.2727272727273, 1.90114068441065, 1.27877237851662, 
    94.4029850746269, 2.3121387283237, 1.03092783505155, 93.717277486911, 
    2.52100840336134, 1.93798449612403, 0, 0, 0.377358490566038, 
    0.675675675675676, 1.13636363636364, 1.82481751824818, 95.4716981132076, 
    3.1055900621118, 1.74927113702624, 94.3661971830986, 2.92682926829268, 
    2.47933884297521, 95.1807228915663, 0.996677740863787, 96.44128113879, 
    95.4692556634304, 95.1690821256039, 0.909090909090909, 95.9016393442623, 
    1.86915887850467, 1.55763239875389, 91.4965986394558), G.perc = c(0, 
    1.65562913907285, 93.5483870967742, 1.05448154657293, 1.8348623853211, 
    4.1958041958042, 97.2222222222222, 96.0526315789474, 1.80995475113122, 
    1.18694362017804, 1.59362549800797, 95.3488372093023, 0.5859375, 
    0.60790273556231, 2.39043824701195, 93.9873417721519, 2.09205020920502, 
    98.0972515856237, 3.515625, 0.33112582781457, 2.31023102310231, 
    98.4496124031008, 1.08303249097473, 94.9579831932773, 1.9672131147541, 
    0.873362445414847, 0, 0, 1.71428571428571, 2.22929936305732, 
    2.94117647058824, 2.11267605633803, 94.2307692307692, 96.6153846153846, 
    95.81589958159, 1.52671755725191, 0.396825396825397, 0.604229607250755, 
    0.628930817610063, 0.634920634920635, 2.72108843537415, 96.420581655481, 
    2.82485875706215, 0.993377483443709, 1.81818181818182, 94.2965779467681, 
    1.27877237851662, 0.746268656716418, 89.5953757225434, 97.2508591065292, 
    2.09424083769634, 1.68067226890756, 1.55038759689922, 97.0760233918129, 
    0.928792569659443, 0.754716981132076, 93.2432432432432, 0.757575757575758, 
    1.09489051094891, 1.13207547169811, 91.9254658385093, 1.16618075801749, 
    0.352112676056338, 1.95121951219512, 96.2809917355372, 1.60642570281124, 
    0.332225913621262, 1.42348754448399, 1.61812297734628, 1.93236714975845, 
    96.3636363636364, 1.22950819672131, 93.4579439252336, 1.24610591900312, 
    1.70068027210884), T.perc = c(0.37593984962406, 0.33112582781457, 
    0.921658986175115, 0.878734622144112, 1.8348623853211, 90.9090909090909, 
    0.868055555555556, 0.328947368421053, 2.26244343891403, 1.18694362017804, 
    8.36653386454183, 0, 0.9765625, 1.82370820668693, 2.78884462151394, 
    1.89873417721519, 95.397489539749, 0.634249471458774, 94.921875, 
    97.682119205298, 95.7095709570957, 0.516795865633075, 96.028880866426, 
    1.40056022408964, 2.95081967213115, 0, 2.40963855421687, 
    94.5512820512821, 1.71428571428571, 2.86624203821656, 1.26050420168067, 
    2.8169014084507, 0.384615384615385, 0.307692307692308, 0.836820083682008, 
    2.29007633587786, 2.38095238095238, 1.81268882175227, 2.51572327044025, 
    3.17460317460317, 92.1768707482993, 1.11856823266219, 0.847457627118644, 
    2.31788079470199, 2.18181818181818, 0, 0.51150895140665, 
    2.23880597014925, 0, 0.687285223367698, 2.61780104712042, 
    3.36134453781513, 0.775193798449612, 1.75438596491228, 0.619195046439629, 
    1.50943396226415, 3.37837837837838, 96.969696969697, 96.3503649635037, 
    2.64150943396226, 0.62111801242236, 0.291545189504373, 2.46478873239437, 
    0.48780487804878, 0, 2.40963855421687, 98.0066445182724, 
    2.13523131672598, 2.26537216828479, 2.89855072463768, 0, 
    2.04918032786885, 0.934579439252336, 1.86915887850467, 5.10204081632653
    ), base.call = c("A", "A", "G", "A", "C", "T", "G", "G", 
    "C", "C", "A", "G", "A", "C", "C", "G", "T", "G", "T", "T", 
    "T", "G", "T", "G", "C", "A", "C", "T", "A", "C", "A", "C", 
    "G", "G", "G", "C", "A", "C", "C", "C", "T", "G", "A", "C", 
    "C", "G", "A", "C", "G", "G", "C", "A", "A", "G", "A", "A", 
    "G", "T", "T", "C", "G", "A", "C", "A", "G", "C", "T", "C", 
    "C", "C", "G", "C", "G", "A", "C"), index = 282:356, guide.seq = c("A", 
    "A", "G", "A", "C", "T", "G", "G", "C", "C", "A", "G", "A", 
    "C", "C", "G", "T", "G", "T", "T", "T", "G", "T", "G", "C", 
    "A", "C", "T", "A", "C", "A", "C", "G", "G", "G", "C", "A", 
    "C", "C", "C", "T", "G", "A", "C", "C", "G", "A", "C", "G", 
    "G", "C", "A", "A", "G", "A", "A", "G", "T", "T", "C", "G", 
    "A", "C", "A", "G", "C", "T", "C", "C", "C", "G", "C", "G", 
    "A", "C"), T.pval = c(0.938831171874272, 0.944190024125835, 
    0.797835770807053, 0.812982628927288, 0.444612669170249, 
    0, 0.816674906279113, 0.944424246813348, 0.309045941708892, 
    0.696393574517426, 0.000196920667668965, 0.957446808510052, 
    0.777817586079951, 0.448572043019375, 0.187306946151512, 
    0.422326889783262, 0, 0.88795733063924, 0, 0, 0, 0.915210607790266, 
    0, 0.610530573009336, 0.159038841461226, 0.957446808510052, 
    0.270080081174403, 0, 0.48841440333694, 0.173308941409123, 
    0.66691366458225, 0.182126308293937, 0.937673679686051, 0.946583504776928, 
    0.827287173057935, 0.301427593875168, 0.277363190467605, 
    0.452502778533841, 0.244431247301708, 0.126070351315922, 
    0, 0.723466873652172, 0.823705246480932, 0.293903975089859, 
    0.332080269178166, 0.957446808510052, 0.916280690779524, 
    0.315674626123651, 0.957446808510052, 0.873604405675759, 
    0.221617976621622, 0.103327678268739, 0.847334426577872, 
    0.473608974286976, 0.891809234422554, 0.567151794291877, 
    0.101447797422053, 0, 0, 0.21657449228049, 0.891322855914392, 
    0.948072767526622, 0.2564953688167, 0.920905585887552, 0.957446808510052, 
    0.270080081174403, 0, 0.34593687134648, 0.308231819761806, 
    0.167733831416567, 0.957446808510052, 0.372576769741455, 
    0.793186346309587, 0.4325632370119, 0.0137162554374035), 
    C.pval = c(0.180273937607552, 0.482299401900653, 0.596171195383132, 
    0.660385793522072, 0, 0.320658247365919, 0.906380240845712, 
    0.486705770539838, 0, 0, 0.236140743726828, 0.777537932698179, 
    0.602658841608264, 0, 0, 0.645489284398094, 0.899786712401909, 
    0.91303500704078, 0.520436239907901, 0.482299401900653, 0.359937123422043, 
    0.914634146292478, 0.203015145334061, 0.472229428870724, 
    0, 0.0186381881340745, 0, 0.194706764934744, 0.458854674165997, 
    0, 0.204403305138517, 0, 0.258142289677975, 0.530429889625218, 
    0.322540673262208, 0, 0.152007816480039, 0, 0, 0, 0.162494196814646, 
    0.789783320623485, 0.46654177814444, 0, 0, 0.387945748066105, 
    0.640044923566033, 0, 0.256922098002218, 0.74050566913103, 
    0, 0.204403305138517, 0.374734837260384, 0.914634146292478, 
    0.914634146292478, 0.903881954261257, 0.854304798826677, 
    0.698978563622787, 0.416153187497922, 0, 0.10199079351074, 
    0.445118072481696, 0, 0.127128857038954, 0.214142435022812, 
    0, 0.75343258148698, 0, 0, 0, 0.784908401409813, 0, 0.39963043712648, 
    0.522455562593644, 0), G.pval = c(0.922222222222223, 0.422046290850882, 
    0, 0.673112947493409, 0.35753498222231, 0.0217104748953949, 
    0, 0, 0.366095093779763, 0.615798508308242, 0.445863041540234, 
    0, 0.849596722696996, 0.843105840618786, 0.201959918140039, 
    0, 0.277143724905044, 0, 0.0528436465906932, 0.904863969334994, 
    0.220331871191923, 0, 0.660827917310731, 0, 0.314331706285679, 
    0.748602747283656, 0.922222222222223, 0.922222222222223, 
    0.400202666384557, 0.240216273802977, 0.107401798381968, 
    0.271331812202626, 0, 0, 0, 0.47234417351046, 0.894380636922083, 
    0.844208212421694, 0.836667251883085, 0.834794184496957, 
    0.139135827746933, 0, 0.123271482853411, 0.699144445230564, 
    0.363252820762668, 0, 0.576046985552926, 0.797101647179736, 
    0, 0, 0.276522003936935, 0.412636266318299, 0.462887711630937, 
    0, 0.726100533148912, 0.794039476520586, 0, 0.79299746562121, 
    0.655709145438258, 0.639612447991377, 0, 0.624813011750321, 
    0.901810151686369, 0.319346525225396, 0, 0.440887297406017, 
    0.904710760222044, 0.514605712127223, 0.436366723472017, 
    0.325330732375748, 0, 0.597335567861974, 0, 0.590150403726534, 
    0.405207815942303), A.pval = c(0, 0, 0.0414581520962983, 
    0, 0.0863196881124573, 0.143787460558734, 0.397801677347629, 
    0.288848702532256, 0.622791513437731, 0.265580780703554, 
    0, 0.0620142395525323, 0, 0.151799928137901, 0.284604678748184, 
    0.137462784450333, 0.262362055434241, 0.566569934786377, 
    0.874999999999981, 0.822264481875622, 0.874999999999981, 
    0.575152498349636, 0.813873196621453, 0.291863399182326, 
    0.375401444058555, 0, 0.414857389638924, 0.133082746790923, 
    0, 0.303779864899192, 0, 0.445482318903471, 0.11205158127442, 
    0.405000339879112, 0.49637788802683, 0.408550152646436, 0, 
    0.413464895508212, 0.0790829556433656, 0.102585476974833, 
    0.206174362767926, 0.396762453252932, 0, 0.285820029516569, 
    0.0237198194595545, 0.0574641071241705, 0, 0.169094419132202, 
    0.000792693483758433, 0.576128581442574, 0.395372584762558, 
    0, 0, 0.526179052085091, 0, 0, 0.156234233644413, 0.53797277392778, 
    0.688199305110593, 0.678982324116469, 0.0342281098125601, 
    0, 0.141308972425507, 0, 0.501725297438162, 0.660873681680954, 
    0.712325380446661, 0.874999999999981, 0.718593739460612, 
    0.874999999999981, 0.152907734487642, 0.654713773560343, 
    0.0610139055563482, 0, 0.35819567695014), guide.position = 1:75), .Names = c("A.area", 
"C.area", "G.area", "T.area", "Tot.area", "A.perc", "C.perc", 
"G.perc", "T.perc", "base.call", "index", "guide.seq", "T.pval", 
"C.pval", "G.pval", "A.pval", "guide.position"), row.names = 282:356, class = "data.frame")
```

*Report generated using EditR v1.0.8*
