# Supplementary material for: Mechanistic and genetic basis of single-strand templated repair at Cas12a-induced DNA breaks in Chlamydomonas reinhardtii
Source: Nat Commun. 2021 Nov 19;12:6751. doi: 10.1038/s41467-021-27004-1 (PMC8604939; doi:10.1038/s41467-021-27004-1)
Supplement: Supplementary file 22 — Source Data [file 41467_2021_27004_MOESM22_ESM.zip › Source Data/EditR analysis/EditR outputs/Antisense/rep1_ssODN_antisense_-32_-16_0.html]

EditR v1.0.8 report


# EditR v1.0.8 report

- Data QA
  - Filtering data
  - Percent noise peak area
  - Base information
- Predicted editing
  - Editing bar plot
  - Editing table plot
  - Table of editing results
- For use in R

## Data QA

### Filtering data

What the data looked like prefiltering:

and the post filtering signal / noise plot:

### Percent noise peak area

### Base information

Here’s information about the signal of each base, the critical percent value where any higher value would be called as significant, and Filliben’s correlation for how well the noise was modelled by the zero adjusted gamma distribution.

| Base | Average percent signal | Average peak area | Critical percent value | model mu | Fillibens correlation |
| --- | --- | --- | --- | --- | --- |
| A | 92.68993 | 539.4706 | 8.762644 | 3.362551 | 0.9926094 |
| C | 92.50511 | 556.4478 | 5.542673 | 1.984410 | 0.9937582 |
| G | 94.51992 | 569.0179 | 5.049207 | 1.773750 | 0.9912966 |
| T | 94.04697 | 611.6136 | 5.236906 | 1.979207 | 0.9956455 |

## Predicted editing

### Editing bar plot

### Editing table plot

### Table of editing results


Here’s the entire guide region

| Sanger position | Guide position | Guide sequence | Sanger base call | Focal base | Focal base peak area | p value |  |
| --- | --- | --- | --- | --- | --- | --- | --- |
| 276 | 1 | A | A | A | 93.18 | 0.000000e+00 | \* |
| 276 | 1 | A | A | C | 1.95 | 4.193843e-01 |  |
| 276 | 1 | A | A | G | 1.56 | 4.880490e-01 |  |
| 276 | 1 | A | A | T | 3.31 | 1.037584e-01 |  |
| 277 | 2 | A | A | A | 94.42 | 0.000000e+00 | \* |
| 277 | 2 | A | A | C | 1.18 | 7.059943e-01 |  |
| 277 | 2 | A | A | G | 1.35 | 5.727560e-01 |  |
| 277 | 2 | A | A | T | 3.05 | 1.390633e-01 |  |
| 278 | 3 | G | G | A | 2.35 | 6.048522e-01 |  |
| 278 | 3 | G | G | C | 1.50 | 5.839649e-01 |  |
| 278 | 3 | G | G | G | 96.15 | 0.000000e+00 | \* |
| 278 | 3 | G | G | T | 0.00 | 9.139785e-01 |  |
| 279 | 4 | A | A | A | 94.43 | 0.000000e+00 | \* |
| 279 | 4 | A | A | C | 1.00 | 7.779354e-01 |  |
| 279 | 4 | A | A | G | 1.00 | 7.278165e-01 |  |
| 279 | 4 | A | A | T | 3.58 | 7.662855e-02 |  |
| 280 | 5 | C | C | A | 4.63 | 1.896044e-01 |  |
| 280 | 5 | C | C | C | 91.40 | 0.000000e+00 | \* |
| 280 | 5 | C | C | G | 1.82 | 3.916298e-01 |  |
| 280 | 5 | C | C | T | 2.15 | 3.372592e-01 |  |
| 281 | 6 | T | T | A | 3.98 | 2.769862e-01 |  |
| 281 | 6 | T | T | C | 1.28 | 6.684519e-01 |  |
| 281 | 6 | T | T | G | 2.28 | 2.544736e-01 |  |
| 281 | 6 | T | T | T | 92.46 | 0.000000e+00 | \* |
| 282 | 7 | G | G | A | 2.60 | 5.480923e-01 |  |
| 282 | 7 | G | G | C | 0.00 | 9.642857e-01 |  |
| 282 | 7 | G | G | G | 96.38 | 0.000000e+00 | \* |
| 282 | 7 | G | G | T | 1.02 | 7.460220e-01 |  |
| 283 | 8 | G | G | A | 4.10 | 2.589258e-01 |  |
| 283 | 8 | G | G | C | 1.27 | 6.713017e-01 |  |
| 283 | 8 | G | G | G | 93.78 | 0.000000e+00 | \* |
| 283 | 8 | G | G | T | 0.85 | 8.029427e-01 |  |
| 284 | 9 | C | C | A | 6.81 | 4.361852e-02 |  |
| 284 | 9 | C | C | C | 89.05 | 0.000000e+00 | \* |
| 284 | 9 | C | C | G | 1.95 | 3.487679e-01 |  |
| 284 | 9 | C | C | T | 2.19 | 3.251083e-01 |  |
| 285 | 10 | C | C | A | 3.63 | 3.352621e-01 |  |
| 285 | 10 | C | C | C | 92.30 | 0.000000e+00 | \* |
| 285 | 10 | C | C | G | 2.47 | 2.090002e-01 |  |
| 285 | 10 | C | C | T | 1.60 | 5.264986e-01 |  |
| 286 | 11 | A | A | A | 75.34 | 0.000000e+00 | \* |
| 286 | 11 | A | A | C | 1.94 | 4.218969e-01 |  |
| 286 | 11 | A | A | G | 1.75 | 4.166642e-01 |  |
| 286 | 11 | A | A | T | 20.97 | 5.434542e-13 | \* |
| 287 | 12 | G | G | A | 2.28 | 6.207355e-01 |  |
| 287 | 12 | G | G | C | 2.28 | 3.188330e-01 |  |
| 287 | 12 | G | G | G | 94.91 | 0.000000e+00 | \* |
| 287 | 12 | G | G | T | 0.53 | 8.807563e-01 |  |
| 288 | 13 | A | A | A | 94.92 | 0.000000e+00 | \* |
| 288 | 13 | A | A | C | 0.83 | 8.358104e-01 |  |
| 288 | 13 | A | A | G | 0.93 | 7.542547e-01 |  |
| 288 | 13 | A | A | T | 3.32 | 1.035024e-01 |  |
| 289 | 14 | C | C | A | 4.21 | 2.427242e-01 |  |
| 289 | 14 | C | C | C | 92.22 | 0.000000e+00 | \* |
| 289 | 14 | C | C | G | 1.94 | 3.492740e-01 |  |
| 289 | 14 | C | C | T | 1.62 | 5.182004e-01 |  |
| 290 | 15 | C | C | A | 5.65 | 9.803986e-02 |  |
| 290 | 15 | C | C | C | 91.34 | 0.000000e+00 | \* |
| 290 | 15 | C | C | G | 1.59 | 4.759806e-01 |  |
| 290 | 15 | C | C | T | 1.41 | 5.979623e-01 |  |
| 291 | 16 | G | G | A | 4.87 | 1.628209e-01 |  |
| 291 | 16 | G | G | C | 1.18 | 7.090937e-01 |  |
| 291 | 16 | G | G | G | 92.77 | 0.000000e+00 | \* |
| 291 | 16 | G | G | T | 1.18 | 6.893132e-01 |  |
| 292 | 17 | T | T | A | 0.00 | 9.000000e-01 |  |
| 292 | 17 | T | T | C | 3.37 | 1.130883e-01 |  |
| 292 | 17 | T | T | G | 2.02 | 3.250041e-01 |  |
| 292 | 17 | T | T | T | 94.61 | 0.000000e+00 | \* |
| 293 | 18 | G | G | A | 2.92 | 4.767240e-01 |  |
| 293 | 18 | G | G | C | 0.00 | 9.642857e-01 |  |
| 293 | 18 | G | G | G | 96.63 | 0.000000e+00 | \* |
| 293 | 18 | G | G | T | 0.45 | 8.924480e-01 |  |
| 294 | 19 | T | T | A | 0.00 | 9.000000e-01 |  |
| 294 | 19 | T | T | C | 1.78 | 4.779247e-01 |  |
| 294 | 19 | T | T | G | 3.74 | 5.087222e-02 |  |
| 294 | 19 | T | T | T | 94.48 | 0.000000e+00 | \* |
| 295 | 20 | T | T | A | 1.46 | 7.929432e-01 |  |
| 295 | 20 | T | T | C | 2.43 | 2.804341e-01 |  |
| 295 | 20 | T | T | G | 2.91 | 1.306935e-01 |  |
| 295 | 20 | T | T | T | 93.20 | 0.000000e+00 | \* |
| 296 | 21 | T | T | A | 0.00 | 9.000000e-01 |  |
| 296 | 21 | T | T | C | 2.87 | 1.867125e-01 |  |
| 296 | 21 | T | T | G | 2.07 | 3.107428e-01 |  |
| 296 | 21 | T | T | T | 95.06 | 0.000000e+00 | \* |
| 297 | 22 | G | G | A | 2.19 | 6.407519e-01 |  |
| 297 | 22 | G | G | C | 0.00 | 9.642857e-01 |  |
| 297 | 22 | G | G | G | 96.95 | 0.000000e+00 | \* |
| 297 | 22 | G | G | T | 0.85 | 8.017340e-01 |  |
| 298 | 23 | T | T | A | 0.58 | 8.914154e-01 |  |
| 298 | 23 | T | T | C | 3.29 | 1.222317e-01 |  |
| 298 | 23 | T | T | G | 1.94 | 3.514974e-01 |  |
| 298 | 23 | T | T | T | 94.19 | 0.000000e+00 | \* |
| 299 | 24 | G | G | A | 2.25 | 6.272017e-01 |  |
| 299 | 24 | G | G | C | 1.05 | 7.571360e-01 |  |
| 299 | 24 | G | G | G | 95.50 | 0.000000e+00 | \* |
| 299 | 24 | G | G | T | 1.20 | 6.799530e-01 |  |
| 300 | 25 | C | C | A | 2.03 | 6.772013e-01 |  |
| 300 | 25 | C | C | C | 94.28 | 0.000000e+00 | \* |
| 300 | 25 | C | C | G | 1.48 | 5.216742e-01 |  |
| 300 | 25 | C | C | T | 2.21 | 3.180710e-01 |  |
| 301 | 26 | A | A | A | 90.29 | 0.000000e+00 | \* |
| 301 | 26 | A | A | C | 3.53 | 9.573387e-02 |  |
| 301 | 26 | A | A | G | 2.43 | 2.183322e-01 |  |
| 301 | 26 | A | A | T | 3.75 | 6.289030e-02 |  |
| 302 | 27 | C | C | A | 2.99 | 4.630086e-01 |  |
| 302 | 27 | C | C | C | 87.40 | 0.000000e+00 | \* |
| 302 | 27 | C | C | G | 7.96 | 2.000422e-04 | \* |
| 302 | 27 | C | C | T | 1.66 | 5.040516e-01 |  |
| 303 | 28 | T | T | A | 1.44 | 7.965885e-01 |  |
| 303 | 28 | T | T | C | 3.35 | 1.156044e-01 |  |
| 303 | 28 | T | T | G | 0.00 | 9.670330e-01 |  |
| 303 | 28 | T | T | T | 95.22 | 0.000000e+00 | \* |
| 304 | 29 | A | A | A | 94.83 | 0.000000e+00 | \* |
| 304 | 29 | A | A | C | 1.60 | 5.423760e-01 |  |
| 304 | 29 | A | A | G | 2.14 | 2.909174e-01 |  |
| 304 | 29 | A | A | T | 1.43 | 5.930643e-01 |  |
| 305 | 30 | C | C | A | 1.85 | 7.150180e-01 |  |
| 305 | 30 | C | C | C | 94.27 | 0.000000e+00 | \* |
| 305 | 30 | C | C | G | 1.69 | 4.391748e-01 |  |
| 305 | 30 | C | C | T | 2.19 | 3.243888e-01 |  |
| 306 | 31 | A | A | A | 89.28 | 0.000000e+00 | \* |
| 306 | 31 | A | A | C | 2.63 | 2.341979e-01 |  |
| 306 | 31 | A | A | G | 4.38 | 2.337881e-02 |  |
| 306 | 31 | A | A | T | 3.72 | 6.534541e-02 |  |
| 307 | 32 | C | C | A | 1.48 | 7.884861e-01 |  |
| 307 | 32 | C | C | C | 94.44 | 0.000000e+00 | \* |
| 307 | 32 | C | C | G | 2.22 | 2.683205e-01 |  |
| 307 | 32 | C | C | T | 1.85 | 4.339037e-01 |  |
| 308 | 33 | G | G | A | 1.89 | 7.082488e-01 |  |
| 308 | 33 | G | G | C | 1.89 | 4.404094e-01 |  |
| 308 | 33 | G | G | G | 94.34 | 0.000000e+00 | \* |
| 308 | 33 | G | G | T | 1.89 | 4.217824e-01 |  |
| 309 | 34 | G | G | A | 1.30 | 8.190864e-01 |  |
| 309 | 34 | G | G | C | 0.97 | 7.855920e-01 |  |
| 309 | 34 | G | G | G | 96.92 | 0.000000e+00 | \* |
| 309 | 34 | G | G | T | 0.81 | 8.139646e-01 |  |
| 310 | 35 | G | G | A | 2.83 | 4.957845e-01 |  |
| 310 | 35 | G | G | C | 1.21 | 6.942257e-01 |  |
| 310 | 35 | G | G | G | 94.94 | 0.000000e+00 | \* |
| 310 | 35 | G | G | T | 1.01 | 7.492501e-01 |  |
| 311 | 36 | C | C | A | 2.69 | 5.276456e-01 |  |
| 311 | 36 | C | C | C | 94.62 | 0.000000e+00 | \* |
| 311 | 36 | C | C | G | 1.12 | 6.733026e-01 |  |
| 311 | 36 | C | C | T | 1.57 | 5.376706e-01 |  |
| 312 | 37 | A | A | A | 93.25 | 0.000000e+00 | \* |
| 312 | 37 | A | A | C | 2.74 | 2.100171e-01 |  |
| 312 | 37 | A | A | G | 0.00 | 9.670330e-01 |  |
| 312 | 37 | A | A | T | 4.01 | 4.648659e-02 |  |
| 313 | 38 | C | C | A | 2.20 | 6.391773e-01 |  |
| 313 | 38 | C | C | C | 95.45 | 0.000000e+00 | \* |
| 313 | 38 | C | C | G | 1.17 | 6.506751e-01 |  |
| 313 | 38 | C | C | T | 1.17 | 6.906136e-01 |  |
| 314 | 39 | C | C | A | 4.67 | 1.848120e-01 |  |
| 314 | 39 | C | C | C | 92.11 | 0.000000e+00 | \* |
| 314 | 39 | C | C | G | 0.97 | 7.401032e-01 |  |
| 314 | 39 | C | C | T | 2.25 | 3.065787e-01 |  |
| 315 | 40 | C | C | A | 3.73 | 3.177712e-01 |  |
| 315 | 40 | C | C | C | 92.05 | 0.000000e+00 | \* |
| 315 | 40 | C | C | G | 1.95 | 3.482615e-01 |  |
| 315 | 40 | C | C | T | 2.27 | 3.014722e-01 |  |
| 316 | 41 | T | T | A | 0.00 | 9.000000e-01 |  |
| 316 | 41 | T | T | C | 3.03 | 1.591936e-01 |  |
| 316 | 41 | T | T | G | 1.59 | 4.741091e-01 |  |
| 316 | 41 | T | T | T | 95.37 | 0.000000e+00 | \* |
| 317 | 42 | G | G | A | 2.83 | 4.971580e-01 |  |
| 317 | 42 | G | G | C | 0.26 | 9.570503e-01 |  |
| 317 | 42 | G | G | G | 95.89 | 0.000000e+00 | \* |
| 317 | 42 | G | G | T | 1.03 | 7.435838e-01 |  |
| 318 | 43 | A | A | A | 87.26 | 0.000000e+00 | \* |
| 318 | 43 | A | A | C | 1.16 | 7.163654e-01 |  |
| 318 | 43 | A | A | G | 2.32 | 2.446586e-01 |  |
| 318 | 43 | A | A | T | 9.26 | 3.683889e-05 | \* |
| 319 | 44 | C | C | A | 3.56 | 3.486766e-01 |  |
| 319 | 44 | C | C | C | 92.20 | 0.000000e+00 | \* |
| 319 | 44 | C | C | G | 2.88 | 1.352391e-01 |  |
| 319 | 44 | C | C | T | 1.36 | 6.203251e-01 |  |
| 320 | 45 | C | C | A | 6.08 | 7.342569e-02 |  |
| 320 | 45 | C | C | C | 90.61 | 0.000000e+00 | \* |
| 320 | 45 | C | C | G | 1.66 | 4.500424e-01 |  |
| 320 | 45 | C | C | T | 1.66 | 5.043945e-01 |  |
| 321 | 46 | G | G | A | 2.86 | 4.907080e-01 |  |
| 321 | 46 | G | G | C | 2.20 | 3.422164e-01 |  |
| 321 | 46 | G | G | G | 94.95 | 0.000000e+00 | \* |
| 321 | 46 | G | G | T | 0.00 | 9.139785e-01 |  |
| 322 | 47 | A | A | A | 95.28 | 0.000000e+00 | \* |
| 322 | 47 | A | A | C | 0.83 | 8.369695e-01 |  |
| 322 | 47 | A | A | G | 0.71 | 8.430126e-01 |  |
| 322 | 47 | A | A | T | 3.18 | 1.197318e-01 |  |
| 323 | 48 | C | C | A | 3.98 | 2.766922e-01 |  |
| 323 | 48 | C | C | C | 93.74 | 0.000000e+00 | \* |
| 323 | 48 | C | C | G | 0.76 | 8.239514e-01 |  |
| 323 | 48 | C | C | T | 1.52 | 5.574281e-01 |  |
| 324 | 49 | G | G | A | 7.23 | 3.226051e-02 |  |
| 324 | 49 | G | G | C | 1.73 | 4.942181e-01 |  |
| 324 | 49 | G | G | G | 89.88 | 0.000000e+00 | \* |
| 324 | 49 | G | G | T | 1.16 | 6.969817e-01 |  |
| 325 | 50 | G | G | A | 2.20 | 6.395416e-01 |  |
| 325 | 50 | G | G | C | 1.57 | 5.554511e-01 |  |
| 325 | 50 | G | G | G | 95.29 | 0.000000e+00 | \* |
| 325 | 50 | G | G | T | 0.94 | 7.732104e-01 |  |
| 326 | 51 | C | C | A | 3.38 | 3.823453e-01 |  |
| 326 | 51 | C | C | C | 94.65 | 0.000000e+00 | \* |
| 326 | 51 | C | C | G | 1.97 | 3.407033e-01 |  |
| 326 | 51 | C | C | T | 0.00 | 9.139785e-01 |  |
| 327 | 52 | A | A | A | 92.83 | 0.000000e+00 | \* |
| 327 | 52 | A | A | C | 1.37 | 6.350011e-01 |  |
| 327 | 52 | A | A | G | 2.05 | 3.173605e-01 |  |
| 327 | 52 | A | A | T | 3.75 | 6.277973e-02 |  |
| 328 | 53 | A | A | A | 95.11 | 0.000000e+00 | \* |
| 328 | 53 | A | A | C | 0.56 | 9.103521e-01 |  |
| 328 | 53 | A | A | G | 0.94 | 7.512277e-01 |  |
| 328 | 53 | A | A | T | 3.38 | 9.599791e-02 |  |
| 329 | 54 | G | G | A | 1.90 | 7.053700e-01 |  |
| 329 | 54 | G | G | C | 1.43 | 6.114518e-01 |  |
| 329 | 54 | G | G | G | 95.96 | 0.000000e+00 | \* |
| 329 | 54 | G | G | T | 0.71 | 8.410485e-01 |  |
| 330 | 55 | A | A | A | 95.49 | 0.000000e+00 | \* |
| 330 | 55 | A | A | C | 0.00 | 9.642857e-01 |  |
| 330 | 55 | A | A | G | 1.22 | 6.322898e-01 |  |
| 330 | 55 | A | A | T | 3.30 | 1.055284e-01 |  |
| 331 | 56 | A | A | A | 94.00 | 0.000000e+00 | \* |
| 331 | 56 | A | A | C | 0.39 | 9.430543e-01 |  |
| 331 | 56 | A | A | G | 0.77 | 8.183560e-01 |  |
| 331 | 56 | A | A | T | 4.84 | 1.674252e-02 |  |
| 332 | 57 | G | G | A | 1.71 | 7.441160e-01 |  |
| 332 | 57 | G | G | C | 0.57 | 9.086301e-01 |  |
| 332 | 57 | G | G | G | 95.43 | 0.000000e+00 | \* |
| 332 | 57 | G | G | T | 2.29 | 2.978852e-01 |  |
| 333 | 58 | T | T | A | 1.02 | 8.567832e-01 |  |
| 333 | 58 | T | T | C | 3.06 | 1.544178e-01 |  |
| 333 | 58 | T | T | G | 0.82 | 8.017524e-01 |  |
| 333 | 58 | T | T | T | 95.10 | 0.000000e+00 | \* |
| 334 | 59 | T | T | A | 1.34 | 8.127967e-01 |  |
| 334 | 59 | T | T | C | 1.34 | 6.455324e-01 |  |
| 334 | 59 | T | T | G | 2.29 | 2.498422e-01 |  |
| 334 | 59 | T | T | T | 95.03 | 0.000000e+00 | \* |
| 335 | 60 | C | C | A | 4.92 | 1.577503e-01 |  |
| 335 | 60 | C | C | C | 91.48 | 0.000000e+00 | \* |
| 335 | 60 | C | C | G | 2.46 | 2.108988e-01 |  |
| 335 | 60 | C | C | T | 1.14 | 7.043380e-01 |  |
| 336 | 61 | G | G | A | 6.57 | 5.194982e-02 |  |
| 336 | 61 | G | G | C | 1.95 | 4.203278e-01 |  |
| 336 | 61 | G | G | G | 91.00 | 0.000000e+00 | \* |
| 336 | 61 | G | G | T | 0.49 | 8.871456e-01 |  |
| 337 | 62 | A | A | A | 95.11 | 0.000000e+00 | \* |
| 337 | 62 | A | A | C | 1.12 | 7.319593e-01 |  |
| 337 | 62 | A | A | G | 1.12 | 6.749386e-01 |  |
| 337 | 62 | A | A | T | 2.65 | 2.089230e-01 |  |
| 338 | 63 | C | C | A | 3.90 | 2.903916e-01 |  |
| 338 | 63 | C | C | C | 93.39 | 0.000000e+00 | \* |
| 338 | 63 | C | C | G | 0.68 | 8.535414e-01 |  |
| 338 | 63 | C | C | T | 2.03 | 3.728794e-01 |  |
| 339 | 64 | A | A | A | 91.93 | 0.000000e+00 | \* |
| 339 | 64 | A | A | C | 1.82 | 4.625107e-01 |  |
| 339 | 64 | A | A | G | 2.86 | 1.377347e-01 |  |
| 339 | 64 | A | A | T | 3.39 | 9.578732e-02 |  |
| 340 | 65 | G | G | A | 1.76 | 7.348226e-01 |  |
| 340 | 65 | G | G | C | 2.08 | 3.774949e-01 |  |
| 340 | 65 | G | G | G | 95.04 | 0.000000e+00 | \* |
| 340 | 65 | G | G | T | 1.12 | 7.104052e-01 |  |
| 341 | 66 | C | C | A | 3.53 | 3.532646e-01 |  |
| 341 | 66 | C | C | C | 92.31 | 0.000000e+00 | \* |
| 341 | 66 | C | C | G | 1.46 | 5.301839e-01 |  |
| 341 | 66 | C | C | T | 2.70 | 1.988477e-01 |  |
| 342 | 67 | T | T | A | 0.00 | 9.000000e-01 |  |
| 342 | 67 | T | T | C | 1.36 | 6.356114e-01 |  |
| 342 | 67 | T | T | G | 1.36 | 5.685157e-01 |  |
| 342 | 67 | T | T | T | 97.27 | 0.000000e+00 | \* |
| 343 | 68 | C | C | A | 4.45 | 2.113771e-01 |  |
| 343 | 68 | C | C | C | 92.70 | 0.000000e+00 | \* |
| 343 | 68 | C | C | G | 1.25 | 6.191565e-01 |  |
| 343 | 68 | C | C | T | 1.60 | 5.255168e-01 |  |
| 344 | 69 | C | C | A | 3.85 | 2.979805e-01 |  |
| 344 | 69 | C | C | C | 92.72 | 0.000000e+00 | \* |
| 344 | 69 | C | C | G | 0.43 | 9.264276e-01 |  |
| 344 | 69 | C | C | T | 3.00 | 1.466828e-01 |  |
| 345 | 70 | C | C | A | 5.05 | 1.451918e-01 |  |
| 345 | 70 | C | C | C | 90.99 | 0.000000e+00 | \* |
| 345 | 70 | C | C | G | 1.54 | 4.964052e-01 |  |
| 345 | 70 | C | C | T | 2.42 | 2.632080e-01 |  |
| 346 | 71 | G | G | A | 5.66 | 9.788196e-02 |  |
| 346 | 71 | G | G | C | 1.36 | 6.380393e-01 |  |
| 346 | 71 | G | G | G | 92.53 | 0.000000e+00 | \* |
| 346 | 71 | G | G | T | 0.45 | 8.920378e-01 |  |
| 347 | 72 | C | C | A | 5.74 | 9.264278e-02 |  |
| 347 | 72 | C | C | C | 91.19 | 0.000000e+00 | \* |
| 347 | 72 | C | C | G | 0.82 | 8.004285e-01 |  |
| 347 | 72 | C | C | T | 2.25 | 3.066713e-01 |  |
| 348 | 73 | G | G | A | 6.56 | 5.239517e-02 |  |
| 348 | 73 | G | G | C | 3.04 | 1.569858e-01 |  |
| 348 | 73 | G | G | G | 89.70 | 0.000000e+00 | \* |
| 348 | 73 | G | G | T | 0.70 | 8.435702e-01 |  |
| 349 | 74 | A | A | A | 94.52 | 0.000000e+00 | \* |
| 349 | 74 | A | A | C | 1.29 | 6.644785e-01 |  |
| 349 | 74 | A | A | G | 0.81 | 8.056429e-01 |  |
| 349 | 74 | A | A | T | 3.39 | 9.560696e-02 |  |
| 350 | 75 | C | C | A | 4.42 | 2.151244e-01 |  |
| 350 | 75 | C | C | C | 93.13 | 0.000000e+00 | \* |
| 350 | 75 | C | C | G | 0.33 | 9.457837e-01 |  |
| 350 | 75 | C | C | T | 2.13 | 3.436276e-01 |  |

## For use in R

If you want to work with the results in R, here is output that you can copy and paste in your terminal to get:

The base information:

```
structure(list(focal.base = c("A", "C", "G", "T"), avg.percsignal = c(92.6899307376402, 
92.5051093571898, 94.5199237064938, 94.0469726160606), avg.areasignal = c(539.470588235294, 
556.44776119403, 569.017857142857, 611.613636363636), crit.perc.area = c(8.7626439207613, 
5.54267278477351, 5.0492066125571, 5.23690570636206), mu = c(3.36255049537521, 
1.98441016368639, 1.7737504225502, 1.97920681318258), fillibens = c(0.992609381159278, 
0.993758238024031, 0.99129659138987, 0.995645520247513)), .Names = c("focal.base", 
"avg.percsignal", "avg.areasignal", "crit.perc.area", "mu", "fillibens"
), row.names = c(NA, -4L), class = "data.frame")
```

the data.frame that contains information on the guide region:

```
structure(list(A.area = c(478, 558, 11, 949, 28, 28, 28, 29, 
28, 25, 388, 13, 916, 26, 32, 29, 0, 26, 0, 9, 0, 18, 3, 15, 
11, 409, 18, 9, 532, 11, 408, 8, 8, 8, 14, 12, 442, 15, 29, 23, 
0, 22, 603, 21, 33, 13, 808, 21, 25, 14, 12, 272, 506, 8, 550, 
486, 6, 5, 7, 26, 27, 681, 23, 353, 11, 17, 0, 25, 27, 23, 25, 
28, 28, 586, 27), C.area = c(10, 7, 7, 10, 553, 9, 0, 9, 366, 
635, 10, 13, 8, 569, 517, 7, 15, 0, 10, 15, 18, 0, 17, 7, 511, 
16, 527, 21, 9, 559, 12, 510, 8, 6, 6, 422, 13, 651, 572, 567, 
19, 2, 8, 544, 492, 10, 7, 494, 6, 10, 336, 4, 3, 6, 0, 2, 2, 
15, 7, 483, 8, 8, 551, 7, 13, 444, 9, 521, 650, 414, 6, 445, 
13, 8, 569), G.area = c(8, 8, 450, 10, 11, 16, 1038, 663, 8, 
17, 9, 541, 9, 12, 9, 552, 9, 860, 21, 18, 13, 796, 10, 636, 
8, 11, 48, 0, 12, 10, 20, 12, 400, 597, 469, 5, 0, 8, 6, 12, 
10, 746, 16, 17, 9, 432, 6, 4, 311, 607, 7, 6, 5, 404, 7, 4, 
334, 4, 12, 13, 374, 8, 4, 11, 594, 7, 9, 7, 3, 7, 409, 4, 383, 
5, 2), T.area = c(17, 18, 0, 36, 13, 650, 11, 6, 9, 11, 108, 
3, 32, 10, 8, 7, 421, 4, 531, 576, 597, 7, 486, 8, 12, 17, 10, 
597, 8, 13, 17, 10, 8, 5, 5, 7, 19, 8, 14, 14, 598, 8, 64, 8, 
9, 0, 27, 8, 4, 6, 0, 11, 18, 3, 19, 25, 8, 466, 497, 6, 2, 19, 
12, 13, 7, 13, 642, 9, 21, 11, 2, 11, 3, 21, 13), Tot.area = c(513, 
591, 468, 1005, 605, 703, 1077, 707, 411, 688, 515, 570, 965, 
617, 566, 595, 445, 890, 562, 618, 628, 821, 516, 666, 542, 453, 
603, 627, 561, 593, 457, 540, 424, 616, 494, 446, 474, 682, 621, 
616, 627, 778, 691, 590, 543, 455, 848, 527, 346, 637, 355, 293, 
532, 421, 576, 517, 350, 490, 523, 528, 411, 716, 590, 384, 625, 
481, 660, 562, 701, 455, 442, 488, 427, 620, 611), A.perc = c(93.17738791423, 
94.4162436548223, 2.35042735042735, 94.4278606965174, 4.62809917355372, 
3.98293029871977, 2.59981429897864, 4.1018387553041, 6.81265206812652, 
3.63372093023256, 75.3398058252427, 2.28070175438596, 94.9222797927461, 
4.21393841166937, 5.65371024734982, 4.87394957983193, 0, 2.92134831460674, 
0, 1.45631067961165, 0, 2.19244823386114, 0.581395348837209, 
2.25225225225225, 2.02952029520295, 90.2869757174393, 2.98507462686567, 
1.43540669856459, 94.8306595365419, 1.85497470489039, 89.2778993435449, 
1.48148148148148, 1.88679245283019, 1.2987012987013, 2.83400809716599, 
2.69058295964126, 93.2489451476793, 2.19941348973607, 4.66988727858293, 
3.73376623376623, 0, 2.82776349614396, 87.2648335745297, 3.5593220338983, 
6.07734806629834, 2.85714285714286, 95.2830188679245, 3.98481973434535, 
7.22543352601156, 2.1978021978022, 3.38028169014085, 92.8327645051195, 
95.1127819548872, 1.90023752969121, 95.4861111111111, 94.0038684719536, 
1.71428571428571, 1.02040816326531, 1.33843212237094, 4.92424242424242, 
6.56934306569343, 95.1117318435754, 3.89830508474576, 91.9270833333333, 
1.76, 3.53430353430353, 0, 4.44839857651246, 3.85164051355207, 
5.05494505494505, 5.65610859728507, 5.73770491803279, 6.55737704918033, 
94.5161290322581, 4.4189852700491), C.perc = c(1.94931773879142, 
1.1844331641286, 1.4957264957265, 0.995024875621891, 91.404958677686, 
1.28022759601707, 0, 1.27298444130127, 89.051094890511, 92.296511627907, 
1.94174757281553, 2.28070175438596, 0.829015544041451, 92.2204213938412, 
91.3427561837456, 1.17647058823529, 3.37078651685393, 0, 1.77935943060498, 
2.42718446601942, 2.86624203821656, 0, 3.29457364341085, 1.05105105105105, 
94.280442804428, 3.53200883002207, 87.3963515754561, 3.34928229665072, 
1.60427807486631, 94.2664418212479, 2.62582056892779, 94.4444444444444, 
1.88679245283019, 0.974025974025974, 1.21457489878543, 94.6188340807175, 
2.74261603375527, 95.4545454545455, 92.109500805153, 92.0454545454545, 
3.03030303030303, 0.25706940874036, 1.15774240231548, 92.2033898305085, 
90.6077348066298, 2.1978021978022, 0.825471698113208, 93.7381404174573, 
1.73410404624277, 1.56985871271586, 94.6478873239437, 1.36518771331058, 
0.56390977443609, 1.42517814726841, 0, 0.386847195357834, 0.571428571428571, 
3.06122448979592, 1.33843212237094, 91.4772727272727, 1.94647201946472, 
1.11731843575419, 93.3898305084746, 1.82291666666667, 2.08, 92.3076923076923, 
1.36363636363636, 92.7046263345196, 92.7246790299572, 90.989010989011, 
1.35746606334842, 91.1885245901639, 3.04449648711944, 1.29032258064516, 
93.126022913257), G.perc = c(1.55945419103314, 1.35363790186125, 
96.1538461538462, 0.995024875621891, 1.81818181818182, 2.27596017069701, 
96.3788300835655, 93.7765205091938, 1.94647201946472, 2.47093023255814, 
1.74757281553398, 94.9122807017544, 0.932642487046632, 1.94489465153971, 
1.59010600706714, 92.7731092436975, 2.02247191011236, 96.6292134831461, 
3.73665480427046, 2.9126213592233, 2.07006369426752, 96.9549330085262, 
1.93798449612403, 95.4954954954955, 1.4760147601476, 2.42825607064018, 
7.96019900497512, 0, 2.13903743315508, 1.68634064080944, 4.37636761487965, 
2.22222222222222, 94.3396226415094, 96.9155844155844, 94.9392712550607, 
1.12107623318386, 0, 1.17302052785924, 0.966183574879227, 1.94805194805195, 
1.59489633173844, 95.8868894601542, 2.31548480463097, 2.88135593220339, 
1.65745856353591, 94.9450549450549, 0.707547169811321, 0.759013282732448, 
89.8843930635838, 95.2904238618524, 1.97183098591549, 2.04778156996587, 
0.93984962406015, 95.9619952494062, 1.21527777777778, 0.773694390715667, 
95.4285714285714, 0.816326530612245, 2.29445506692161, 2.46212121212121, 
90.9975669099757, 1.11731843575419, 0.677966101694915, 2.86458333333333, 
95.04, 1.45530145530146, 1.36363636363636, 1.24555160142349, 
0.427960057061341, 1.53846153846154, 92.5339366515837, 0.819672131147541, 
89.6955503512881, 0.806451612903226, 0.327332242225859), T.perc = c(3.31384015594542, 
3.04568527918782, 0, 3.58208955223881, 2.14876033057851, 92.4608819345661, 
1.0213556174559, 0.848656294200849, 2.18978102189781, 1.59883720930233, 
20.9708737864078, 0.526315789473684, 3.3160621761658, 1.62074554294976, 
1.41342756183746, 1.17647058823529, 94.6067415730337, 0.449438202247191, 
94.4839857651246, 93.2038834951456, 95.0636942675159, 0.852618757612667, 
94.1860465116279, 1.2012012012012, 2.2140221402214, 3.75275938189845, 
1.65837479270315, 95.2153110047847, 1.42602495543672, 2.19224283305228, 
3.7199124726477, 1.85185185185185, 1.88679245283019, 0.811688311688312, 
1.01214574898785, 1.5695067264574, 4.0084388185654, 1.17302052785924, 
2.25442834138486, 2.27272727272727, 95.3748006379585, 1.02827763496144, 
9.26193921852388, 1.35593220338983, 1.65745856353591, 0, 3.18396226415094, 
1.5180265654649, 1.15606936416185, 0.941915227629513, 0, 3.7542662116041, 
3.38345864661654, 0.712589073634204, 3.29861111111111, 4.83558994197292, 
2.28571428571429, 95.1020408163265, 95.0286806883365, 1.13636363636364, 
0.48661800486618, 2.6536312849162, 2.03389830508475, 3.38541666666667, 
1.12, 2.7027027027027, 97.2727272727273, 1.60142348754448, 2.99572039942939, 
2.41758241758242, 0.452488687782805, 2.25409836065574, 0.702576112412178, 
3.38709677419355, 2.12765957446809), base.call = c("A", "A", 
"G", "A", "C", "T", "G", "G", "C", "C", "A", "G", "A", "C", "C", 
"G", "T", "G", "T", "T", "T", "G", "T", "G", "C", "A", "C", "T", 
"A", "C", "A", "C", "G", "G", "G", "C", "A", "C", "C", "C", "T", 
"G", "A", "C", "C", "G", "A", "C", "G", "G", "C", "A", "A", "G", 
"A", "A", "G", "T", "T", "C", "G", "A", "C", "A", "G", "C", "T", 
"C", "C", "C", "G", "C", "G", "A", "C"), index = 276:350, guide.seq = c("A", 
"A", "G", "A", "C", "T", "G", "G", "C", "C", "A", "G", "A", "C", 
"C", "G", "T", "G", "T", "T", "T", "G", "T", "G", "C", "A", "C", 
"T", "A", "C", "A", "C", "G", "G", "G", "C", "A", "C", "C", "C", 
"T", "G", "A", "C", "C", "G", "A", "C", "G", "G", "C", "A", "A", 
"G", "A", "A", "G", "T", "T", "C", "G", "A", "C", "A", "G", "C", 
"T", "C", "C", "C", "G", "C", "G", "A", "C"), T.pval = c(0.103758400049205, 
0.13906327770259, 0.913978494580568, 0.076628547593799, 0.337259221789438, 
0, 0.746022043715554, 0.802942670420961, 0.325108294360757, 0.526498560052393, 
5.43454170554014e-13, 0.880756276952531, 0.103502351520985, 0.5182003669354, 
0.597962305952075, 0.689313216990009, 0, 0.892448030391997, 0, 
0, 0, 0.801733972292302, 0, 0.679952970213245, 0.31807098246191, 
0.0628903017934015, 0.504051610923586, 0, 0.593064273253887, 
0.324388750860071, 0.0653454104685864, 0.43390369003573, 0.421782373981439, 
0.813964597120501, 0.7492501314655, 0.537670621202837, 0.0464865910971018, 
0.690613582165869, 0.306578691095249, 0.301472197516382, 0, 0.743583799656063, 
3.6838892662705e-05, 0.620325062949957, 0.504394453536251, 0.913978494580568, 
0.1197317584125, 0.557428120573963, 0.696981723222871, 0.773210374379579, 
0.913978494580568, 0.0627797262360842, 0.0959979071570903, 0.841048535399721, 
0.105528433744155, 0.0167425164104877, 0.297885191683845, 0, 
0, 0.704337986166225, 0.887145637648518, 0.208923041241946, 0.372879389208879, 
0.0957873233805765, 0.710405225614917, 0.198847724312429, 0, 
0.525516788937392, 0.146682811184286, 0.263207969208483, 0.89203778706001, 
0.306671337135779, 0.843570151002521, 0.0956069574633803, 0.343627554154194
), C.pval = c(0.419384339209747, 0.705994276746416, 0.583964874343139, 
0.777935360947919, 0, 0.668451856479405, 0.964285714274344, 0.67130165422034, 
0, 0, 0.421896925048894, 0.318832958954789, 0.835810376546796, 
0, 0, 0.709093705179072, 0.113088340780482, 0.964285714274344, 
0.477924661165141, 0.280434087960173, 0.186712540263383, 0.964285714274344, 
0.122231744272821, 0.757135973673241, 0, 0.095733868433198, 0, 
0.115604419602149, 0.542375968035693, 0, 0.234197898913899, 0, 
0.440409416516068, 0.785592032959214, 0.694225684881691, 0, 0.210017069358859, 
0, 0, 0, 0.159193613319125, 0.957050275938569, 0.716365444894075, 
0, 0, 0.34221635673414, 0.836969515418538, 0, 0.494218126013301, 
0.555451115215477, 0, 0.635001073429322, 0.910352147592591, 0.611451837899771, 
0.964285714274344, 0.943054329827054, 0.9086301286018, 0.154417817479543, 
0.645532362912852, 0, 0.420327771405897, 0.731959276232002, 0, 
0.462510687323721, 0.377494902750307, 0, 0.635611388456094, 0, 
0, 0, 0.638039285887117, 0, 0.156985818705459, 0.664478485069868, 
0), G.pval = c(0.488049046583186, 0.57275595045657, 0, 0.727816466191467, 
0.391629823873537, 0.25447361959495, 0, 0, 0.348767929198172, 
0.209000163235625, 0.416664221244158, 0, 0.754254659201402, 0.349274035037679, 
0.475980588119052, 0, 0.325004137035057, 0, 0.0508722188532005, 
0.130693528419708, 0.310742789926323, 0, 0.351497359253481, 0, 
0.521674181807673, 0.218332228892934, 0.000200042182288862, 0.967032966994691, 
0.290917422833733, 0.439174834877771, 0.0233788087028459, 0.268320492893101, 
0, 0, 0, 0.673302580539411, 0.967032966994691, 0.650675100574953, 
0.740103156382618, 0.348261526576504, 0.474109075639616, 0, 0.244658611491335, 
0.135239094285158, 0.450042357611712, 0, 0.843012575821107, 0.823951403750357, 
0, 0, 0.340703296146202, 0.317360502875843, 0.751227723051935, 
0, 0.632289832373721, 0.818356040878438, 0, 0.801752375675665, 
0.2498421891324, 0.210898836875151, 0, 0.674938641313628, 0.853541373746217, 
0.137734713510403, 0, 0.530183888194332, 0.568515738176019, 0.61915654225029, 
0.926427550072025, 0.496405193758487, 0, 0.800428470539778, 0, 
0.805642944092607, 0.945783653507763), A.pval = c(0, 0, 0.604852173465332, 
0, 0.189604386845236, 0.276986214525548, 0.548092251305352, 0.258925849331444, 
0.04361852119747, 0.33526205175445, 0, 0.620735491166551, 0, 
0.242724234771743, 0.0980398568351968, 0.162820935671648, 0.899999999997222, 
0.476724049008157, 0.899999999997222, 0.792943245061187, 0.899999999997222, 
0.640751920005626, 0.89141539897952, 0.627201711251061, 0.677201274131619, 
0, 0.463008626653054, 0.796588496415393, 0, 0.71501797499296, 
0, 0.788486131663956, 0.708248765846651, 0.819086350186153, 0.495784508592272, 
0.52764562737619, 0, 0.639177273919063, 0.184811996756196, 0.317771206524446, 
0.899999999997222, 0.497158037704018, 0, 0.348676563665735, 0.0734256855145722, 
0.490707981824248, 0, 0.27669215808215, 0.0322605118989983, 0.639541632519802, 
0.382345307688693, 0, 0, 0.705369952718932, 0, 0, 0.744115978593733, 
0.856783217862926, 0.81279665300186, 0.157750320629625, 0.0519498241677999, 
0, 0.290391641054288, 0, 0.73482257170186, 0.353264602387565, 
0.899999999997222, 0.211377058419511, 0.297980487115685, 0.14519181137681, 
0.0978819604802402, 0.0926427811059523, 0.0523951672688037, 0, 
0.21512436740691), guide.position = 1:75), .Names = c("A.area", 
"C.area", "G.area", "T.area", "Tot.area", "A.perc", "C.perc", 
"G.perc", "T.perc", "base.call", "index", "guide.seq", "T.pval", 
"C.pval", "G.pval", "A.pval", "guide.position"), row.names = 276:350, class = "data.frame")
```

*Report generated using EditR v1.0.8*
