# Supplementary material for: Mechanistic and genetic basis of single-strand templated repair at Cas12a-induced DNA breaks in Chlamydomonas reinhardtii
Source: Nat Commun. 2021 Nov 19;12:6751. doi: 10.1038/s41467-021-27004-1 (PMC8604939; doi:10.1038/s41467-021-27004-1)
Supplement: Supplementary file 22 — Source Data [file 41467_2021_27004_MOESM22_ESM.zip › Source Data/EditR analysis/EditR outputs/Antisense/rep1_ssODN_antisense_-32_-16_0_16_32.html]

EditR v1.0.8 report


# EditR v1.0.8 report

- Data QA
  - Filtering data
  - Percent noise peak area
  - Base information
- Predicted editing
  - Editing bar plot
  - Editing table plot
  - Table of editing results
- For use in R

## Data QA

### Filtering data

What the data looked like prefiltering:

and the post filtering signal / noise plot:

### Percent noise peak area

### Base information

Here’s information about the signal of each base, the critical percent value where any higher value would be called as significant, and Filliben’s correlation for how well the noise was modelled by the zero adjusted gamma distribution.

| Base | Average percent signal | Average peak area | Critical percent value | model mu | Fillibens correlation |
| --- | --- | --- | --- | --- | --- |
| A | 91.18494 | 396.3824 | 10.492097 | 3.119441 | 0.9376365 |
| C | 92.17446 | 432.7059 | 5.586492 | 1.994537 | 0.9921947 |
| G | 93.86033 | 435.7143 | 8.706983 | 2.328671 | 0.9895955 |
| T | 93.10071 | 492.3256 | 7.506488 | 2.516136 | 0.9946771 |

## Predicted editing

### Editing bar plot

### Editing table plot

### Table of editing results


Here’s the entire guide region

| Sanger position | Guide position | Guide sequence | Sanger base call | Focal base | Focal base peak area | p value |  |
| --- | --- | --- | --- | --- | --- | --- | --- |
| 278 | 1 | A | A | A | 95.21 | 0.000000e+00 | \* |
| 278 | 1 | A | A | C | 2.13 | 3.473338e-01 |  |
| 278 | 1 | A | A | G | 0.27 | 8.667062e-01 |  |
| 278 | 1 | A | A | T | 2.39 | 4.129152e-01 |  |
| 279 | 2 | A | A | A | 93.83 | 0.000000e+00 | \* |
| 279 | 2 | A | A | C | 1.91 | 4.109559e-01 |  |
| 279 | 2 | A | A | G | 1.28 | 5.900001e-01 |  |
| 279 | 2 | A | A | T | 2.98 | 2.907920e-01 |  |
| 280 | 3 | G | G | A | 1.83 | 5.878178e-01 |  |
| 280 | 3 | G | G | C | 1.31 | 6.238793e-01 |  |
| 280 | 3 | G | G | G | 95.04 | 0.000000e+00 | \* |
| 280 | 3 | G | G | T | 1.83 | 5.582168e-01 |  |
| 281 | 4 | A | A | A | 94.63 | 0.000000e+00 | \* |
| 281 | 4 | A | A | C | 1.05 | 7.178950e-01 |  |
| 281 | 4 | A | A | G | 0.65 | 7.629248e-01 |  |
| 281 | 4 | A | A | T | 3.66 | 1.856773e-01 |  |
| 282 | 5 | C | C | A | 3.90 | 2.562957e-01 |  |
| 282 | 5 | C | C | C | 92.84 | 0.000000e+00 | \* |
| 282 | 5 | C | C | G | 1.30 | 5.834680e-01 |  |
| 282 | 5 | C | C | T | 1.95 | 5.243507e-01 |  |
| 283 | 6 | T | T | A | 3.55 | 2.986585e-01 |  |
| 283 | 6 | T | T | C | 1.42 | 5.813620e-01 |  |
| 283 | 6 | T | T | G | 2.13 | 3.935026e-01 |  |
| 283 | 6 | T | T | T | 92.90 | 0.000000e+00 | \* |
| 284 | 7 | G | G | A | 2.61 | 4.396877e-01 |  |
| 284 | 7 | G | G | C | 0.71 | 8.251735e-01 |  |
| 284 | 7 | G | G | G | 95.61 | 0.000000e+00 | \* |
| 284 | 7 | G | G | T | 1.07 | 7.693050e-01 |  |
| 285 | 8 | G | G | A | 3.59 | 2.937893e-01 |  |
| 285 | 8 | G | G | C | 1.08 | 7.072133e-01 |  |
| 285 | 8 | G | G | G | 94.08 | 0.000000e+00 | \* |
| 285 | 8 | G | G | T | 1.26 | 7.185483e-01 |  |
| 286 | 9 | C | C | A | 5.02 | 1.548724e-01 |  |
| 286 | 9 | C | C | C | 92.79 | 0.000000e+00 | \* |
| 286 | 9 | C | C | G | 2.19 | 3.813715e-01 |  |
| 286 | 9 | C | C | T | 0.00 | 9.255319e-01 |  |
| 287 | 10 | C | C | A | 2.96 | 3.831016e-01 |  |
| 287 | 10 | C | C | C | 92.98 | 0.000000e+00 | \* |
| 287 | 10 | C | C | G | 2.96 | 2.579217e-01 |  |
| 287 | 10 | C | C | T | 1.11 | 7.587006e-01 |  |
| 288 | 11 | A | A | A | 59.05 | 1.187939e-14 | \* |
| 288 | 11 | A | A | C | 1.81 | 4.448014e-01 |  |
| 288 | 11 | A | A | G | 1.81 | 4.604888e-01 |  |
| 288 | 11 | A | A | T | 37.33 | 1.387779e-14 | \* |
| 289 | 12 | G | G | A | 2.57 | 4.463657e-01 |  |
| 289 | 12 | G | G | C | 2.38 | 2.820455e-01 |  |
| 289 | 12 | G | G | G | 95.05 | 0.000000e+00 | \* |
| 289 | 12 | G | G | T | 0.00 | 9.255319e-01 |  |
| 290 | 13 | A | A | A | 94.73 | 0.000000e+00 | \* |
| 290 | 13 | A | A | C | 0.83 | 7.899003e-01 |  |
| 290 | 13 | A | A | G | 1.11 | 6.348204e-01 |  |
| 290 | 13 | A | A | T | 3.33 | 2.323317e-01 |  |
| 291 | 14 | C | C | A | 5.21 | 1.415365e-01 |  |
| 291 | 14 | C | C | C | 91.67 | 0.000000e+00 | \* |
| 291 | 14 | C | C | G | 1.46 | 5.433919e-01 |  |
| 291 | 14 | C | C | T | 1.67 | 6.030385e-01 |  |
| 292 | 15 | C | C | A | 4.70 | 1.793761e-01 |  |
| 292 | 15 | C | C | C | 91.95 | 0.000000e+00 | \* |
| 292 | 15 | C | C | G | 1.79 | 4.649903e-01 |  |
| 292 | 15 | C | C | T | 1.57 | 6.314500e-01 |  |
| 293 | 16 | G | G | A | 4.19 | 2.253934e-01 |  |
| 293 | 16 | G | G | C | 1.32 | 6.168607e-01 |  |
| 293 | 16 | G | G | G | 93.16 | 0.000000e+00 | \* |
| 293 | 16 | G | G | T | 1.32 | 6.996720e-01 |  |
| 294 | 17 | T | T | A | 0.00 | 8.909091e-01 |  |
| 294 | 17 | T | T | C | 2.57 | 2.376477e-01 |  |
| 294 | 17 | T | T | G | 2.00 | 4.198718e-01 |  |
| 294 | 17 | T | T | T | 95.43 | 0.000000e+00 | \* |
| 295 | 18 | G | G | A | 2.90 | 3.917113e-01 |  |
| 295 | 18 | G | G | C | 1.31 | 6.235996e-01 |  |
| 295 | 18 | G | G | G | 95.21 | 0.000000e+00 | \* |
| 295 | 18 | G | G | T | 0.58 | 8.782573e-01 |  |
| 296 | 19 | T | T | A | 1.59 | 6.363642e-01 |  |
| 296 | 19 | T | T | C | 1.36 | 6.024313e-01 |  |
| 296 | 19 | T | T | G | 2.05 | 4.105945e-01 |  |
| 296 | 19 | T | T | T | 95.00 | 0.000000e+00 | \* |
| 297 | 20 | T | T | A | 2.26 | 5.034845e-01 |  |
| 297 | 20 | T | T | C | 1.85 | 4.323376e-01 |  |
| 297 | 20 | T | T | G | 2.46 | 3.328791e-01 |  |
| 297 | 20 | T | T | T | 93.43 | 0.000000e+00 | \* |
| 298 | 21 | T | T | A | 0.00 | 8.909091e-01 |  |
| 298 | 21 | T | T | C | 0.00 | 9.156627e-01 |  |
| 298 | 21 | T | T | G | 2.24 | 3.727072e-01 |  |
| 298 | 21 | T | T | T | 97.76 | 0.000000e+00 | \* |
| 299 | 22 | G | G | A | 2.05 | 5.427952e-01 |  |
| 299 | 22 | G | G | C | 0.00 | 9.156627e-01 |  |
| 299 | 22 | G | G | G | 97.00 | 0.000000e+00 | \* |
| 299 | 22 | G | G | T | 0.95 | 8.001315e-01 |  |
| 300 | 23 | T | T | A | 0.00 | 8.909091e-01 |  |
| 300 | 23 | T | T | C | 2.48 | 2.587825e-01 |  |
| 300 | 23 | T | T | G | 1.98 | 4.239660e-01 |  |
| 300 | 23 | T | T | T | 95.54 | 0.000000e+00 | \* |
| 301 | 24 | G | G | A | 5.04 | 1.533193e-01 |  |
| 301 | 24 | G | G | C | 1.12 | 6.920599e-01 |  |
| 301 | 24 | G | G | G | 92.54 | 0.000000e+00 | \* |
| 301 | 24 | G | G | T | 1.31 | 7.048546e-01 |  |
| 302 | 25 | C | C | A | 2.42 | 4.745698e-01 |  |
| 302 | 25 | C | C | C | 93.24 | 0.000000e+00 | \* |
| 302 | 25 | C | C | G | 1.93 | 4.339874e-01 |  |
| 302 | 25 | C | C | T | 2.42 | 4.078061e-01 |  |
| 303 | 26 | A | A | A | 91.87 | 0.000000e+00 | \* |
| 303 | 26 | A | A | C | 3.31 | 1.178254e-01 |  |
| 303 | 26 | A | A | G | 1.51 | 5.315703e-01 |  |
| 303 | 26 | A | A | T | 3.31 | 2.346933e-01 |  |
| 304 | 27 | C | C | A | 2.23 | 5.094293e-01 |  |
| 304 | 27 | C | C | C | 78.40 | 0.000000e+00 | \* |
| 304 | 27 | C | C | G | 17.82 | 4.161838e-05 | \* |
| 304 | 27 | C | C | T | 1.56 | 6.334245e-01 |  |
| 305 | 28 | T | T | A | 1.30 | 6.958920e-01 |  |
| 305 | 28 | T | T | C | 2.61 | 2.298386e-01 |  |
| 305 | 28 | T | T | G | 0.22 | 8.777968e-01 |  |
| 305 | 28 | T | T | T | 95.87 | 0.000000e+00 | \* |
| 306 | 29 | A | A | A | 96.33 | 0.000000e+00 | \* |
| 306 | 29 | A | A | C | 1.57 | 5.256716e-01 |  |
| 306 | 29 | A | A | G | 0.52 | 7.992750e-01 |  |
| 306 | 29 | A | A | T | 1.57 | 6.289575e-01 |  |
| 307 | 30 | C | C | A | 1.79 | 5.947073e-01 |  |
| 307 | 30 | C | C | C | 94.17 | 0.000000e+00 | \* |
| 307 | 30 | C | C | G | 1.12 | 6.316785e-01 |  |
| 307 | 30 | C | C | T | 2.91 | 3.026297e-01 |  |
| 308 | 31 | A | A | A | 89.77 | 0.000000e+00 | \* |
| 308 | 31 | A | A | C | 2.92 | 1.718189e-01 |  |
| 308 | 31 | A | A | G | 4.39 | 1.192917e-01 |  |
| 308 | 31 | A | A | T | 2.92 | 3.009075e-01 |  |
| 309 | 32 | C | C | A | 1.43 | 6.700836e-01 |  |
| 309 | 32 | C | C | C | 95.00 | 0.000000e+00 | \* |
| 309 | 32 | C | C | G | 1.43 | 5.508577e-01 |  |
| 309 | 32 | C | C | T | 2.14 | 4.744221e-01 |  |
| 310 | 33 | G | G | A | 2.48 | 4.635450e-01 |  |
| 310 | 33 | G | G | C | 1.86 | 4.292488e-01 |  |
| 310 | 33 | G | G | G | 95.67 | 0.000000e+00 | \* |
| 310 | 33 | G | G | T | 0.00 | 9.255319e-01 |  |
| 311 | 34 | G | G | A | 1.04 | 7.495648e-01 |  |
| 311 | 34 | G | G | C | 1.25 | 6.443406e-01 |  |
| 311 | 34 | G | G | G | 97.50 | 0.000000e+00 | \* |
| 311 | 34 | G | G | T | 0.21 | 9.207834e-01 |  |
| 312 | 35 | G | G | A | 3.08 | 3.636205e-01 |  |
| 312 | 35 | G | G | C | 1.29 | 6.313092e-01 |  |
| 312 | 35 | G | G | G | 93.83 | 0.000000e+00 | \* |
| 312 | 35 | G | G | T | 1.80 | 5.659893e-01 |  |
| 313 | 36 | C | C | A | 3.29 | 3.333773e-01 |  |
| 313 | 36 | C | C | C | 93.41 | 0.000000e+00 | \* |
| 313 | 36 | C | C | G | 1.50 | 5.337922e-01 |  |
| 313 | 36 | C | C | T | 1.80 | 5.668404e-01 |  |
| 314 | 37 | A | A | A | 92.82 | 0.000000e+00 | \* |
| 314 | 37 | A | A | C | 2.87 | 1.801706e-01 |  |
| 314 | 37 | A | A | G | 0.29 | 8.616042e-01 |  |
| 314 | 37 | A | A | T | 4.02 | 1.450839e-01 |  |
| 315 | 38 | C | C | A | 2.10 | 5.347057e-01 |  |
| 315 | 38 | C | C | C | 95.43 | 0.000000e+00 | \* |
| 315 | 38 | C | C | G | 1.14 | 6.257517e-01 |  |
| 315 | 38 | C | C | T | 1.33 | 6.971980e-01 |  |
| 316 | 39 | C | C | A | 4.93 | 1.613024e-01 |  |
| 316 | 39 | C | C | C | 92.40 | 0.000000e+00 | \* |
| 316 | 39 | C | C | G | 0.21 | 8.804296e-01 |  |
| 316 | 39 | C | C | T | 2.46 | 3.965854e-01 |  |
| 317 | 40 | C | C | A | 4.09 | 2.356146e-01 |  |
| 317 | 40 | C | C | C | 91.81 | 0.000000e+00 | \* |
| 317 | 40 | C | C | G | 1.29 | 5.856687e-01 |  |
| 317 | 40 | C | C | T | 2.80 | 3.244707e-01 |  |
| 318 | 41 | T | T | A | 2.90 | 3.928235e-01 |  |
| 318 | 41 | T | T | C | 2.12 | 3.484955e-01 |  |
| 318 | 41 | T | T | G | 3.86 | 1.590884e-01 |  |
| 318 | 41 | T | T | T | 91.12 | 0.000000e+00 | \* |
| 319 | 42 | G | G | A | 9.16 | 1.994685e-02 |  |
| 319 | 42 | G | G | C | 1.74 | 4.689469e-01 |  |
| 319 | 42 | G | G | G | 87.68 | 0.000000e+00 | \* |
| 319 | 42 | G | G | T | 1.42 | 6.722821e-01 |  |
| 320 | 43 | A | A | A | 77.63 | 0.000000e+00 | \* |
| 320 | 43 | A | A | C | 1.50 | 5.511908e-01 |  |
| 320 | 43 | A | A | G | 3.01 | 2.512361e-01 |  |
| 320 | 43 | A | A | T | 17.86 | 1.150005e-06 | \* |
| 321 | 44 | C | C | A | 5.61 | 1.173174e-01 |  |
| 321 | 44 | C | C | C | 89.24 | 0.000000e+00 | \* |
| 321 | 44 | C | C | G | 4.04 | 1.446131e-01 |  |
| 321 | 44 | C | C | T | 1.12 | 7.554976e-01 |  |
| 322 | 45 | C | C | A | 10.66 | 9.169267e-03 | \* |
| 322 | 45 | C | C | C | 82.31 | 0.000000e+00 | \* |
| 322 | 45 | C | C | G | 4.08 | 1.410357e-01 |  |
| 322 | 45 | C | C | T | 2.95 | 2.964642e-01 |  |
| 323 | 46 | G | G | A | 9.40 | 1.763359e-02 |  |
| 323 | 46 | G | G | C | 1.42 | 5.800639e-01 |  |
| 323 | 46 | G | G | G | 88.89 | 0.000000e+00 | \* |
| 323 | 46 | G | G | T | 0.28 | 9.157087e-01 |  |
| 324 | 47 | A | A | A | 94.77 | 0.000000e+00 | \* |
| 324 | 47 | A | A | C | 0.79 | 8.021006e-01 |  |
| 324 | 47 | A | A | G | 0.63 | 7.687387e-01 |  |
| 324 | 47 | A | A | T | 3.80 | 1.689254e-01 |  |
| 325 | 48 | C | C | A | 4.70 | 1.789661e-01 |  |
| 325 | 48 | C | C | C | 91.34 | 0.000000e+00 | \* |
| 325 | 48 | C | C | G | 2.48 | 3.309885e-01 |  |
| 325 | 48 | C | C | T | 1.49 | 6.543516e-01 |  |
| 326 | 49 | G | G | A | 5.09 | 1.495337e-01 |  |
| 326 | 49 | G | G | C | 1.82 | 4.420930e-01 |  |
| 326 | 49 | G | G | G | 91.64 | 0.000000e+00 | \* |
| 326 | 49 | G | G | T | 1.45 | 6.630189e-01 |  |
| 327 | 50 | G | G | A | 2.81 | 4.069919e-01 |  |
| 327 | 50 | G | G | C | 1.51 | 5.482548e-01 |  |
| 327 | 50 | G | G | G | 94.60 | 0.000000e+00 | \* |
| 327 | 50 | G | G | T | 1.08 | 7.664098e-01 |  |
| 328 | 51 | C | C | A | 3.41 | 3.174867e-01 |  |
| 328 | 51 | C | C | C | 92.80 | 0.000000e+00 | \* |
| 328 | 51 | C | C | G | 1.89 | 4.421743e-01 |  |
| 328 | 51 | C | C | T | 1.89 | 5.401010e-01 |  |
| 329 | 52 | A | A | A | 93.86 | 0.000000e+00 | \* |
| 329 | 52 | A | A | C | 1.75 | 4.633269e-01 |  |
| 329 | 52 | A | A | G | 0.88 | 6.996141e-01 |  |
| 329 | 52 | A | A | T | 3.51 | 2.062485e-01 |  |
| 330 | 53 | A | A | A | 93.95 | 0.000000e+00 | \* |
| 330 | 53 | A | A | C | 0.53 | 8.701874e-01 |  |
| 330 | 53 | A | A | G | 2.89 | 2.665333e-01 |  |
| 330 | 53 | A | A | T | 2.63 | 3.595218e-01 |  |
| 331 | 54 | G | G | A | 3.36 | 3.236183e-01 |  |
| 331 | 54 | G | G | C | 0.00 | 9.156627e-01 |  |
| 331 | 54 | G | G | G | 95.72 | 0.000000e+00 | \* |
| 331 | 54 | G | G | T | 0.92 | 8.075744e-01 |  |
| 332 | 55 | A | A | A | 95.85 | 0.000000e+00 | \* |
| 332 | 55 | A | A | C | 0.23 | 9.100205e-01 |  |
| 332 | 55 | A | A | G | 1.15 | 6.232521e-01 |  |
| 332 | 55 | A | A | T | 2.76 | 3.318182e-01 |  |
| 333 | 56 | A | A | A | 91.83 | 0.000000e+00 | \* |
| 333 | 56 | A | A | C | 0.27 | 9.068840e-01 |  |
| 333 | 56 | A | A | G | 3.00 | 2.525920e-01 |  |
| 333 | 56 | A | A | T | 4.90 | 7.679237e-02 |  |
| 334 | 57 | G | G | A | 1.53 | 6.496771e-01 |  |
| 334 | 57 | G | G | C | 0.00 | 9.156627e-01 |  |
| 334 | 57 | G | G | G | 94.27 | 0.000000e+00 | \* |
| 334 | 57 | G | G | T | 4.20 | 1.282179e-01 |  |
| 335 | 58 | T | T | A | 0.58 | 8.345835e-01 |  |
| 335 | 58 | T | T | C | 2.31 | 2.979348e-01 |  |
| 335 | 58 | T | T | G | 0.87 | 7.024863e-01 |  |
| 335 | 58 | T | T | T | 96.24 | 0.000000e+00 | \* |
| 336 | 59 | T | T | A | 14.17 | 1.411817e-03 | \* |
| 336 | 59 | T | T | C | 3.00 | 1.602658e-01 |  |
| 336 | 59 | T | T | G | 1.91 | 4.393020e-01 |  |
| 336 | 59 | T | T | T | 80.93 | 0.000000e+00 | \* |
| 337 | 60 | C | C | A | 2.58 | 4.446760e-01 |  |
| 337 | 60 | C | C | C | 91.21 | 0.000000e+00 | \* |
| 337 | 60 | C | C | G | 5.17 | 7.712434e-02 |  |
| 337 | 60 | C | C | T | 1.03 | 7.784798e-01 |  |
| 338 | 61 | G | G | A | 6.48 | 7.674370e-02 |  |
| 338 | 61 | G | G | C | 1.37 | 6.018598e-01 |  |
| 338 | 61 | G | G | G | 91.13 | 0.000000e+00 | \* |
| 338 | 61 | G | G | T | 1.02 | 7.809768e-01 |  |
| 339 | 62 | A | A | A | 94.69 | 0.000000e+00 | \* |
| 339 | 62 | A | A | C | 1.33 | 6.154698e-01 |  |
| 339 | 62 | A | A | G | 1.33 | 5.765039e-01 |  |
| 339 | 62 | A | A | T | 2.66 | 3.542138e-01 |  |
| 340 | 63 | C | C | A | 3.57 | 2.962287e-01 |  |
| 340 | 63 | C | C | C | 94.05 | 0.000000e+00 | \* |
| 340 | 63 | C | C | G | 0.71 | 7.459281e-01 |  |
| 340 | 63 | C | C | T | 1.67 | 6.030385e-01 |  |
| 341 | 64 | A | A | A | 87.45 | 0.000000e+00 | \* |
| 341 | 64 | A | A | C | 1.85 | 4.333220e-01 |  |
| 341 | 64 | A | A | G | 7.38 | 2.172654e-02 |  |
| 341 | 64 | A | A | T | 3.32 | 2.335022e-01 |  |
| 342 | 65 | G | G | A | 0.68 | 8.178622e-01 |  |
| 342 | 65 | G | G | C | 3.39 | 1.087549e-01 |  |
| 342 | 65 | G | G | G | 94.57 | 0.000000e+00 | \* |
| 342 | 65 | G | G | T | 1.36 | 6.904228e-01 |  |
| 343 | 66 | C | C | A | 0.89 | 7.792505e-01 |  |
| 343 | 66 | C | C | C | 91.69 | 0.000000e+00 | \* |
| 343 | 66 | C | C | G | 1.78 | 4.670680e-01 |  |
| 343 | 66 | C | C | T | 5.64 | 4.412427e-02 |  |
| 344 | 67 | T | T | A | 0.00 | 8.909091e-01 |  |
| 344 | 67 | T | T | C | 2.41 | 2.740249e-01 |  |
| 344 | 67 | T | T | G | 1.61 | 5.072657e-01 |  |
| 344 | 67 | T | T | T | 95.98 | 0.000000e+00 | \* |
| 345 | 68 | C | C | A | 2.66 | 4.310287e-01 |  |
| 345 | 68 | C | C | C | 94.92 | 0.000000e+00 | \* |
| 345 | 68 | C | C | G | 0.00 | 9.120879e-01 |  |
| 345 | 68 | C | C | T | 2.42 | 4.064450e-01 |  |
| 346 | 69 | C | C | A | 2.15 | 5.244338e-01 |  |
| 346 | 69 | C | C | C | 93.55 | 0.000000e+00 | \* |
| 346 | 69 | C | C | G | 0.59 | 7.822599e-01 |  |
| 346 | 69 | C | C | T | 3.71 | 1.799616e-01 |  |
| 347 | 70 | C | C | A | 2.66 | 4.311504e-01 |  |
| 347 | 70 | C | C | C | 88.76 | 0.000000e+00 | \* |
| 347 | 70 | C | C | G | 5.03 | 8.335849e-02 |  |
| 347 | 70 | C | C | T | 3.55 | 2.005972e-01 |  |
| 348 | 71 | G | G | A | 5.18 | 1.432328e-01 |  |
| 348 | 71 | G | G | C | 4.27 | 4.373631e-02 |  |
| 348 | 71 | G | G | G | 89.94 | 0.000000e+00 | \* |
| 348 | 71 | G | G | T | 0.61 | 8.731545e-01 |  |
| 349 | 72 | C | C | A | 4.48 | 1.979901e-01 |  |
| 349 | 72 | C | C | C | 89.08 | 0.000000e+00 | \* |
| 349 | 72 | C | C | G | 3.36 | 2.082805e-01 |  |
| 349 | 72 | C | C | T | 3.08 | 2.725690e-01 |  |
| 350 | 73 | G | G | A | 7.17 | 5.484330e-02 |  |
| 350 | 73 | G | G | C | 2.28 | 3.061152e-01 |  |
| 350 | 73 | G | G | G | 89.90 | 0.000000e+00 | \* |
| 350 | 73 | G | G | T | 0.65 | 8.654840e-01 |  |
| 351 | 74 | A | A | A | 91.82 | 0.000000e+00 | \* |
| 351 | 74 | A | A | C | 1.82 | 4.420930e-01 |  |
| 351 | 74 | A | A | G | 2.05 | 4.105945e-01 |  |
| 351 | 74 | A | A | T | 4.32 | 1.177423e-01 |  |
| 352 | 75 | C | C | A | 2.48 | 4.634206e-01 |  |
| 352 | 75 | C | C | C | 78.83 | 0.000000e+00 | \* |
| 352 | 75 | C | C | G | 14.86 | 2.508347e-04 | \* |
| 352 | 75 | C | C | T | 3.83 | 1.660080e-01 |  |

## For use in R

If you want to work with the results in R, here is output that you can copy and paste in your terminal to get:

The base information:

```
structure(list(focal.base = c("A", "C", "G", "T"), avg.percsignal = c(91.1849376301824, 
92.1744566303702, 93.8603278252797, 93.1007105652809), avg.areasignal = c(396.382352941176, 
432.705882352941, 435.714285714286, 492.325581395349), crit.perc.area = c(10.4920964895851, 
5.58649193784106, 8.7069824627391, 7.50648814426703), mu = c(3.11944131926733, 
1.99453684708888, 2.32867063359262, 2.51613634023054), fillibens = c(0.937636539201853, 
0.992194658218938, 0.989595512682814, 0.994677060940231)), .Names = c("focal.base", 
"avg.percsignal", "avg.areasignal", "crit.perc.area", "mu", "fillibens"
), row.names = c(NA, -4L), class = "data.frame")
```

the data.frame that contains information on the guide region:

```
structure(list(A.area = c(358, 441, 7, 723, 18, 20, 22, 20, 16, 
16, 261, 13, 683, 25, 21, 19, 0, 20, 7, 11, 0, 13, 0, 27, 10, 
305, 10, 6, 367, 8, 307, 6, 8, 5, 12, 11, 323, 11, 24, 19, 15, 
58, 413, 25, 47, 33, 598, 19, 14, 13, 9, 214, 357, 11, 416, 337, 
4, 2, 52, 10, 19, 499, 15, 237, 3, 3, 0, 11, 11, 9, 17, 16, 22, 
404, 11), C.area = c(8, 9, 5, 8, 428, 8, 6, 6, 296, 503, 8, 12, 
6, 440, 411, 6, 9, 9, 6, 9, 0, 0, 10, 6, 386, 11, 352, 12, 6, 
420, 10, 399, 6, 6, 5, 312, 10, 501, 450, 426, 11, 11, 8, 398, 
363, 5, 5, 369, 5, 7, 245, 4, 2, 0, 1, 1, 0, 8, 11, 353, 4, 7, 
395, 5, 15, 309, 12, 392, 479, 300, 14, 318, 7, 8, 350), G.area = c(1, 
6, 364, 5, 6, 12, 805, 524, 7, 16, 8, 480, 8, 7, 8, 422, 7, 656, 
9, 12, 11, 614, 8, 496, 8, 5, 80, 1, 2, 5, 15, 6, 309, 468, 365, 
5, 1, 6, 1, 6, 20, 555, 16, 18, 18, 312, 4, 10, 252, 438, 5, 
2, 11, 313, 5, 11, 247, 3, 7, 20, 267, 7, 3, 20, 418, 6, 8, 0, 
3, 17, 295, 12, 276, 9, 66), T.area = c(9, 14, 7, 28, 9, 523, 
9, 7, 0, 6, 165, 0, 24, 8, 7, 6, 334, 4, 418, 455, 480, 6, 386, 
7, 10, 11, 7, 441, 6, 13, 10, 9, 0, 1, 7, 6, 14, 7, 12, 13, 472, 
9, 95, 5, 13, 1, 24, 6, 4, 5, 5, 8, 10, 3, 12, 18, 11, 333, 297, 
4, 3, 14, 7, 9, 6, 19, 478, 10, 19, 12, 2, 11, 2, 19, 17), Tot.area = c(376, 
470, 383, 764, 461, 563, 842, 557, 319, 541, 442, 505, 721, 480, 
447, 453, 350, 689, 440, 487, 491, 633, 404, 536, 414, 332, 449, 
460, 381, 446, 342, 420, 323, 480, 389, 334, 348, 525, 487, 464, 
518, 633, 532, 446, 441, 351, 631, 404, 275, 463, 264, 228, 380, 
327, 434, 367, 262, 346, 367, 387, 293, 527, 420, 271, 442, 337, 
498, 413, 512, 338, 328, 357, 307, 440, 444), A.perc = c(95.2127659574468, 
93.8297872340426, 1.82767624020888, 94.6335078534031, 3.90455531453362, 
3.55239786856128, 2.61282660332542, 3.59066427289048, 5.01567398119122, 
2.95748613678373, 59.0497737556561, 2.57425742574257, 94.7295423023578, 
5.20833333333333, 4.69798657718121, 4.19426048565121, 0, 2.90275761973875, 
1.59090909090909, 2.25872689938398, 0, 2.05371248025276, 0, 5.03731343283582, 
2.41545893719807, 91.8674698795181, 2.2271714922049, 1.30434782608696, 
96.3254593175853, 1.79372197309417, 89.766081871345, 1.42857142857143, 
2.47678018575851, 1.04166666666667, 3.08483290488432, 3.29341317365269, 
92.816091954023, 2.0952380952381, 4.92813141683778, 4.0948275862069, 
2.8957528957529, 9.16271721958926, 77.6315789473684, 5.60538116591928, 
10.6575963718821, 9.4017094017094, 94.770206022187, 4.7029702970297, 
5.09090909090909, 2.80777537796976, 3.40909090909091, 93.859649122807, 
93.9473684210526, 3.36391437308869, 95.852534562212, 91.8256130790191, 
1.52671755725191, 0.578034682080925, 14.1689373297003, 2.58397932816537, 
6.48464163822526, 94.6869070208729, 3.57142857142857, 87.4538745387454, 
0.678733031674208, 0.890207715133531, 0, 2.6634382566586, 2.1484375, 
2.66272189349112, 5.18292682926829, 4.48179271708683, 7.16612377850163, 
91.8181818181818, 2.47747747747748), C.perc = c(2.12765957446809, 
1.91489361702128, 1.30548302872063, 1.04712041884817, 92.8416485900217, 
1.42095914742451, 0.712589073634204, 1.07719928186715, 92.7899686520376, 
92.9759704251386, 1.80995475113122, 2.37623762376238, 0.832177531206657, 
91.6666666666667, 91.9463087248322, 1.32450331125828, 2.57142857142857, 
1.30624092888244, 1.36363636363636, 1.84804928131417, 0, 0, 2.47524752475248, 
1.11940298507463, 93.2367149758454, 3.31325301204819, 78.3964365256125, 
2.60869565217391, 1.5748031496063, 94.1704035874439, 2.92397660818713, 
95, 1.85758513931889, 1.25, 1.2853470437018, 93.4131736526946, 
2.8735632183908, 95.4285714285714, 92.4024640657084, 91.8103448275862, 
2.12355212355212, 1.73775671406003, 1.50375939849624, 89.237668161435, 
82.312925170068, 1.42450142450142, 0.792393026941363, 91.3366336633663, 
1.81818181818182, 1.51187904967603, 92.8030303030303, 1.75438596491228, 
0.526315789473684, 0, 0.230414746543779, 0.272479564032698, 0, 
2.3121387283237, 2.99727520435967, 91.2144702842377, 1.36518771331058, 
1.32827324478178, 94.0476190476191, 1.8450184501845, 3.39366515837104, 
91.6913946587537, 2.40963855421687, 94.9152542372881, 93.5546875, 
88.7573964497041, 4.26829268292683, 89.0756302521008, 2.28013029315961, 
1.81818181818182, 78.8288288288288), G.perc = c(0.265957446808511, 
1.27659574468085, 95.0391644908616, 0.654450261780105, 1.30151843817787, 
2.13143872113677, 95.6057007125891, 94.0754039497307, 2.19435736677116, 
2.95748613678373, 1.80995475113122, 95.049504950495, 1.10957004160888, 
1.45833333333333, 1.78970917225951, 93.1567328918322, 2, 95.2104499274311, 
2.04545454545455, 2.46406570841889, 2.24032586558045, 96.998420221169, 
1.98019801980198, 92.5373134328358, 1.93236714975845, 1.50602409638554, 
17.8173719376392, 0.217391304347826, 0.5249343832021, 1.12107623318386, 
4.3859649122807, 1.42857142857143, 95.6656346749226, 97.5, 93.8303341902314, 
1.49700598802395, 0.28735632183908, 1.14285714285714, 0.205338809034908, 
1.29310344827586, 3.86100386100386, 87.6777251184834, 3.00751879699248, 
4.03587443946188, 4.08163265306122, 88.8888888888889, 0.63391442155309, 
2.47524752475248, 91.6363636363636, 94.6004319654428, 1.89393939393939, 
0.87719298245614, 2.89473684210526, 95.7186544342508, 1.15207373271889, 
2.99727520435967, 94.2748091603053, 0.867052023121387, 1.90735694822888, 
5.16795865633075, 91.1262798634812, 1.32827324478178, 0.714285714285714, 
7.38007380073801, 94.5701357466063, 1.78041543026706, 1.60642570281124, 
0, 0.5859375, 5.02958579881657, 89.9390243902439, 3.36134453781513, 
89.9022801302932, 2.04545454545455, 14.8648648648649), T.perc = c(2.3936170212766, 
2.97872340425532, 1.82767624020888, 3.66492146596859, 1.95227765726681, 
92.8952042628774, 1.06888361045131, 1.25673249551167, 0, 1.1090573012939, 
37.3303167420815, 0, 3.32871012482663, 1.66666666666667, 1.56599552572707, 
1.32450331125828, 95.4285714285714, 0.58055152394775, 95, 93.429158110883, 
97.7596741344196, 0.947867298578199, 95.5445544554455, 1.30597014925373, 
2.41545893719807, 3.31325301204819, 1.55902004454343, 95.8695652173913, 
1.5748031496063, 2.91479820627803, 2.92397660818713, 2.14285714285714, 
0, 0.208333333333333, 1.79948586118252, 1.79640718562874, 4.02298850574713, 
1.33333333333333, 2.46406570841889, 2.80172413793103, 91.1196911196911, 
1.4218009478673, 17.8571428571429, 1.12107623318386, 2.94784580498866, 
0.284900284900285, 3.80348652931854, 1.48514851485149, 1.45454545454545, 
1.07991360691145, 1.89393939393939, 3.50877192982456, 2.63157894736842, 
0.917431192660551, 2.76497695852535, 4.90463215258856, 4.19847328244275, 
96.242774566474, 80.9264305177112, 1.03359173126615, 1.02389078498294, 
2.65654648956357, 1.66666666666667, 3.3210332103321, 1.35746606334842, 
5.6379821958457, 95.9839357429719, 2.42130750605327, 3.7109375, 
3.55029585798817, 0.609756097560976, 3.0812324929972, 0.651465798045603, 
4.31818181818182, 3.82882882882883), base.call = c("A", "A", 
"G", "A", "C", "T", "G", "G", "C", "C", "A", "G", "A", "C", "C", 
"G", "T", "G", "T", "T", "T", "G", "T", "G", "C", "A", "C", "T", 
"A", "C", "A", "C", "G", "G", "G", "C", "A", "C", "C", "C", "T", 
"G", "A", "C", "C", "G", "A", "C", "G", "G", "C", "A", "A", "G", 
"A", "A", "G", "T", "T", "C", "G", "A", "C", "A", "G", "C", "T", 
"C", "C", "C", "G", "C", "G", "A", "C"), index = 278:352, guide.seq = c("A", 
"A", "G", "A", "C", "T", "G", "G", "C", "C", "A", "G", "A", "C", 
"C", "G", "T", "G", "T", "T", "T", "G", "T", "G", "C", "A", "C", 
"T", "A", "C", "A", "C", "G", "G", "G", "C", "A", "C", "C", "C", 
"T", "G", "A", "C", "C", "G", "A", "C", "G", "G", "C", "A", "A", 
"G", "A", "A", "G", "T", "T", "C", "G", "A", "C", "A", "G", "C", 
"T", "C", "C", "C", "G", "C", "G", "A", "C"), T.pval = c(0.412915206522588, 
0.290792019842357, 0.558216764787575, 0.185677261429536, 0.524350696718937, 
0, 0.769304994060743, 0.718548296144196, 0.925531914893617, 0.758700640849046, 
1.38777878078145e-14, 0.925531914893617, 0.23233169055037, 0.603038539782493, 
0.631449959657711, 0.699671955856246, 0, 0.878257328750774, 0, 
0, 0, 0.800131459924127, 0, 0.704854565619895, 0.407806085008385, 
0.234693344328921, 0.633424511555492, 0, 0.628957527560303, 0.302629733420693, 
0.300907525928063, 0.474422055158196, 0.925531914893617, 0.920783412218157, 
0.565989320129899, 0.56684035401066, 0.14508392192239, 0.697198028604725, 
0.39658535314804, 0.324470686950371, 0, 0.672282131447101, 1.15000516343056e-06, 
0.755497605042718, 0.29646421914407, 0.915708719632386, 0.168925386933391, 
0.654351597624737, 0.66301891709501, 0.766409755469816, 0.540101047760684, 
0.206248491117928, 0.359521824414343, 0.807574428723447, 0.331818156193547, 
0.076792365202408, 0.128217872279307, 0, 0, 0.778479816270405, 
0.780976808161634, 0.354213765240887, 0.603038539782493, 0.233502157799687, 
0.690422808452504, 0.0441242655590037, 0, 0.406445044069531, 
0.179961560884544, 0.20059721702632, 0.873154528737662, 0.272569040969803, 
0.865484024596751, 0.117742261598134, 0.166008040722854), C.pval = c(0.347333830869648, 
0.410955867316063, 0.623879280380945, 0.717894996956547, 0, 0.58136198924833, 
0.825173522062678, 0.707213320721487, 0, 0, 0.444801408288514, 
0.282045497764302, 0.789900268930403, 0, 0, 0.616860705346215, 
0.237647731174822, 0.623599598272923, 0.602431315579872, 0.43233762739667, 
0.915662650542506, 0.915662650542506, 0.258782504052832, 0.692059889815002, 
0, 0.117825393467459, 0, 0.229838610217846, 0.525671640872609, 
0, 0.171818939747205, 0, 0.429248792943426, 0.644340585342064, 
0.631309183173894, 0, 0.180170600440289, 0, 0, 0, 0.348495522489341, 
0.468946929930779, 0.551190843360883, 0, 0, 0.580063926640962, 
0.802100630757714, 0, 0.442093049229808, 0.548254765643551, 0, 
0.463326880040299, 0.870187442083551, 0.915662650542506, 0.910020532961236, 
0.906883977015176, 0.915662650542506, 0.297934833894875, 0.160265811670016, 
0, 0.601859839359674, 0.615469792489743, 0, 0.433322004353823, 
0.10875492725342, 0, 0.274024885834006, 0, 0, 0, 0.0437363065098094, 
0, 0.306115179140362, 0.442093049229808, 0), G.pval = c(0.866706199769482, 
0.590000113518215, 0, 0.762924840129486, 0.583467981497702, 0.393502632110643, 
0, 0, 0.381371521779038, 0.257921665911274, 0.460488797703482, 
0, 0.634820350753518, 0.543391866803911, 0.464990331385638, 0, 
0.419871798578303, 0, 0.410594549475919, 0.332879140549994, 0.372707208987022, 
0, 0.423965967198085, 0, 0.433987362664049, 0.531570338005518, 
4.16183801418279e-05, 0.877796810208954, 0.799275036547121, 0.631678491090015, 
0.119291738668086, 0.550857651921832, 0, 0, 0, 0.533792231459458, 
0.861604222244521, 0.625751679828064, 0.880429582262296, 0.585668714439991, 
0.159088423549397, 0, 0.251236054122349, 0.144613061527821, 0.14103570458901, 
0, 0.768738699784857, 0.330988513300463, 0, 0, 0.442174318702322, 
0.699614086265901, 0.26653328766816, 0, 0.623252066572141, 0.252591953830224, 
0, 0.702486341852524, 0.439301982473488, 0.0771243358841144, 
0, 0.576503887837113, 0.74592812534034, 0.0217265368491819, 0, 
0.467068019941334, 0.507265742319013, 0.912087912058151, 0.782259889177169, 
0.0833584869427572, 0, 0.208280454610767, 0, 0.410594549475919, 
0.000250834730133209), A.pval = c(0, 0, 0.587817767353526, 0, 
0.256295698754005, 0.298658509997634, 0.439687710142484, 0.293789285686609, 
0.154872375340069, 0.38310156466993, 1.18793863634892e-14, 0.446365734810172, 
0, 0.141536481628592, 0.17937610495396, 0.225393411848687, 0.89090909090863, 
0.391711281704449, 0.636364162718045, 0.503484487742799, 0.89090909090863, 
0.542795218536622, 0.89090909090863, 0.153319326122766, 0.474569845614728, 
0, 0.509429291800889, 0.69589200972669, 0, 0.594707325084922, 
0, 0.670083580863458, 0.463545026997042, 0.749564760394818, 0.363620487270599, 
0.333377301600299, 0, 0.534705690131781, 0.161302392783557, 0.235614594253786, 
0.392823542617811, 0.0199468538741497, 0, 0.117317425584745, 
0.00916926673417873, 0.0176335855249318, 0, 0.178966147488955, 
0.149533728026248, 0.406991871594024, 0.317486676358168, 0, 0, 
0.323618348952578, 0, 0, 0.649677098383228, 0.834583474549025, 
0.00141181745030694, 0.444676011035281, 0.0767437017244983, 0, 
0.296228669004345, 0, 0.817862197410311, 0.779250519504811, 0.89090909090863, 
0.43102870621522, 0.524433751002236, 0.431150437766784, 0.14323284889633, 
0.197990113096009, 0.0548432981851827, 0, 0.463420619530962), 
    guide.position = 1:75), .Names = c("A.area", "C.area", "G.area", 
"T.area", "Tot.area", "A.perc", "C.perc", "G.perc", "T.perc", 
"base.call", "index", "guide.seq", "T.pval", "C.pval", "G.pval", 
"A.pval", "guide.position"), row.names = 278:352, class = "data.frame")
```

*Report generated using EditR v1.0.8*
