# Supplementary material for: Mechanistic and genetic basis of single-strand templated repair at Cas12a-induced DNA breaks in Chlamydomonas reinhardtii
Source: Nat Commun. 2021 Nov 19;12:6751. doi: 10.1038/s41467-021-27004-1 (PMC8604939; doi:10.1038/s41467-021-27004-1)
Supplement: Supplementary file 22 — Source Data [file 41467_2021_27004_MOESM22_ESM.zip › Source Data/EditR analysis/EditR outputs/Antisense/rep2_ssODN_antisense_0.html]

EditR v1.0.8 report


# EditR v1.0.8 report

- Data QA
  - Filtering data
  - Percent noise peak area
  - Base information
- Predicted editing
  - Editing bar plot
  - Editing table plot
  - Table of editing results
- For use in R

## Data QA

### Filtering data

What the data looked like prefiltering:

and the post filtering signal / noise plot:

### Percent noise peak area

### Base information

Here’s information about the signal of each base, the critical percent value where any higher value would be called as significant, and Filliben’s correlation for how well the noise was modelled by the zero adjusted gamma distribution.

| Base | Average percent signal | Average peak area | Critical percent value | model mu | Fillibens correlation |
| --- | --- | --- | --- | --- | --- |
| A | 92.28139 | 348.7353 | 8.870529 | 3.016446 | 0.9952855 |
| C | 92.95346 | 367.4848 | 5.647706 | 2.102620 | 0.9946737 |
| G | 93.05056 | 352.4386 | 5.770350 | 2.037110 | 0.9922794 |
| T | 94.24966 | 413.0000 | 6.722417 | 2.452492 | 0.9920912 |

## Predicted editing

### Editing bar plot

### Editing table plot

### Table of editing results


Here’s the entire guide region

| Sanger position | Guide position | Guide sequence | Sanger base call | Focal base | Focal base peak area | p value |  |
| --- | --- | --- | --- | --- | --- | --- | --- |
| 275 | 1 | A | A | A | 95.20 | 0.000000000 | \* |
| 275 | 1 | A | A | C | 1.80 | 0.502190365 |  |
| 275 | 1 | A | A | G | 1.20 | 0.698809667 |  |
| 275 | 1 | A | A | T | 1.80 | 0.563501209 |  |
| 276 | 2 | A | A | A | 93.02 | 0.000000000 | \* |
| 276 | 2 | A | A | C | 0.75 | 0.864734028 |  |
| 276 | 2 | A | A | G | 1.25 | 0.681749417 |  |
| 276 | 2 | A | A | T | 4.99 | 0.050987646 |  |
| 277 | 3 | G | G | A | 2.25 | 0.538177808 |  |
| 277 | 3 | G | G | C | 2.57 | 0.270948828 |  |
| 277 | 3 | G | G | G | 94.53 | 0.000000000 | \* |
| 277 | 3 | G | G | T | 0.64 | 0.863610212 |  |
| 278 | 4 | A | A | A | 93.05 | 0.000000000 | \* |
| 278 | 4 | A | A | C | 1.06 | 0.773061306 |  |
| 278 | 4 | A | A | G | 2.11 | 0.379260840 |  |
| 278 | 4 | A | A | T | 3.78 | 0.143898338 |  |
| 279 | 5 | C | C | A | 4.48 | 0.172286350 |  |
| 279 | 5 | C | C | C | 89.05 | 0.000000000 | \* |
| 279 | 5 | C | C | G | 4.48 | 0.040957272 |  |
| 279 | 5 | C | C | T | 1.99 | 0.508131020 |  |
| 280 | 6 | T | T | A | 4.17 | 0.205833499 |  |
| 280 | 6 | T | T | C | 3.33 | 0.131802109 |  |
| 280 | 6 | T | T | G | 2.50 | 0.276950881 |  |
| 280 | 6 | T | T | T | 90.00 | 0.000000000 | \* |
| 281 | 7 | G | G | A | 3.75 | 0.258962198 |  |
| 281 | 7 | G | G | C | 1.73 | 0.527274873 |  |
| 281 | 7 | G | G | G | 93.36 | 0.000000000 | \* |
| 281 | 7 | G | G | T | 1.15 | 0.752774700 |  |
| 282 | 8 | G | G | A | 4.87 | 0.136587335 |  |
| 282 | 8 | G | G | C | 0.93 | 0.814660592 |  |
| 282 | 8 | G | G | G | 93.27 | 0.000000000 | \* |
| 282 | 8 | G | G | T | 0.93 | 0.809057758 |  |
| 283 | 9 | C | C | A | 5.00 | 0.126536047 |  |
| 283 | 9 | C | C | C | 88.46 | 0.000000000 | \* |
| 283 | 9 | C | C | G | 4.23 | 0.053012974 |  |
| 283 | 9 | C | C | T | 2.31 | 0.420014042 |  |
| 284 | 10 | C | C | A | 4.88 | 0.136126999 |  |
| 284 | 10 | C | C | C | 90.69 | 0.000000000 | \* |
| 284 | 10 | C | C | G | 3.10 | 0.161463772 |  |
| 284 | 10 | C | C | T | 1.33 | 0.703759035 |  |
| 285 | 11 | A | A | A | 93.89 | 0.000000000 | \* |
| 285 | 11 | A | A | C | 2.25 | 0.356364127 |  |
| 285 | 11 | A | A | G | 1.29 | 0.667017597 |  |
| 285 | 11 | A | A | T | 2.57 | 0.353623510 |  |
| 286 | 12 | G | G | A | 2.88 | 0.404123094 |  |
| 286 | 12 | G | G | C | 3.21 | 0.149717697 |  |
| 286 | 12 | G | G | G | 93.91 | 0.000000000 | \* |
| 286 | 12 | G | G | T | 0.00 | 0.903225806 |  |
| 287 | 13 | A | A | A | 94.02 | 0.000000000 | \* |
| 287 | 13 | A | A | C | 0.97 | 0.801833095 |  |
| 287 | 13 | A | A | G | 1.13 | 0.724840014 |  |
| 287 | 13 | A | A | T | 3.88 | 0.132554886 |  |
| 288 | 14 | C | C | A | 4.03 | 0.222207737 |  |
| 288 | 14 | C | C | C | 91.44 | 0.000000000 | \* |
| 288 | 14 | C | C | G | 2.77 | 0.218820124 |  |
| 288 | 14 | C | C | T | 1.76 | 0.575023740 |  |
| 289 | 15 | C | C | A | 4.53 | 0.166873433 |  |
| 289 | 15 | C | C | C | 90.65 | 0.000000000 | \* |
| 289 | 15 | C | C | G | 3.40 | 0.122013973 |  |
| 289 | 15 | C | C | T | 1.42 | 0.678716843 |  |
| 290 | 16 | G | G | A | 4.83 | 0.140435204 |  |
| 290 | 16 | G | G | C | 1.34 | 0.672049963 |  |
| 290 | 16 | G | G | G | 91.69 | 0.000000000 | \* |
| 290 | 16 | G | G | T | 2.14 | 0.464202892 |  |
| 291 | 17 | T | T | A | 0.00 | 0.909090909 |  |
| 291 | 17 | T | T | C | 1.79 | 0.507896186 |  |
| 291 | 17 | T | T | G | 2.14 | 0.371018536 |  |
| 291 | 17 | T | T | T | 96.07 | 0.000000000 | \* |
| 292 | 18 | G | G | A | 4.15 | 0.208355023 |  |
| 292 | 18 | G | G | C | 0.86 | 0.833791055 |  |
| 292 | 18 | G | G | G | 93.26 | 0.000000000 | \* |
| 292 | 18 | G | G | T | 1.73 | 0.585841591 |  |
| 293 | 19 | T | T | A | 0.00 | 0.909090909 |  |
| 293 | 19 | T | T | C | 0.87 | 0.830593176 |  |
| 293 | 19 | T | T | G | 5.83 | 0.009341987 | \* |
| 293 | 19 | T | T | T | 93.29 | 0.000000000 | \* |
| 294 | 20 | T | T | A | 0.52 | 0.886044031 |  |
| 294 | 20 | T | T | C | 2.08 | 0.407401273 |  |
| 294 | 20 | T | T | G | 4.43 | 0.043193241 |  |
| 294 | 20 | T | T | T | 92.97 | 0.000000000 | \* |
| 295 | 21 | T | T | A | 0.00 | 0.909090909 |  |
| 295 | 21 | T | T | C | 0.00 | 0.941176471 |  |
| 295 | 21 | T | T | G | 3.10 | 0.161982000 |  |
| 295 | 21 | T | T | T | 96.90 | 0.000000000 | \* |
| 296 | 22 | G | G | A | 2.35 | 0.515286201 |  |
| 296 | 22 | G | G | C | 2.55 | 0.276580627 |  |
| 296 | 22 | G | G | G | 94.31 | 0.000000000 | \* |
| 296 | 22 | G | G | T | 0.78 | 0.839237010 |  |
| 297 | 23 | T | T | A | 0.31 | 0.902147640 |  |
| 297 | 23 | T | T | C | 4.35 | 0.044988471 |  |
| 297 | 23 | T | T | G | 1.24 | 0.683488753 |  |
| 297 | 23 | T | T | T | 94.10 | 0.000000000 | \* |
| 298 | 24 | G | G | A | 3.90 | 0.238508558 |  |
| 298 | 24 | G | G | C | 1.46 | 0.626241875 |  |
| 298 | 24 | G | G | G | 93.41 | 0.000000000 | \* |
| 298 | 24 | G | G | T | 1.22 | 0.735057609 |  |
| 299 | 25 | C | C | A | 1.24 | 0.769246678 |  |
| 299 | 25 | C | C | C | 96.59 | 0.000000000 | \* |
| 299 | 25 | C | C | G | 2.17 | 0.363971113 |  |
| 299 | 25 | C | C | T | 0.00 | 0.903225806 |  |
| 300 | 26 | A | A | A | 87.97 | 0.000000000 | \* |
| 300 | 26 | A | A | C | 4.12 | 0.057557060 |  |
| 300 | 26 | A | A | G | 3.78 | 0.083921151 |  |
| 300 | 26 | A | A | T | 4.12 | 0.108050588 |  |
| 301 | 27 | C | C | A | 2.66 | 0.449878700 |  |
| 301 | 27 | C | C | C | 94.20 | 0.000000000 | \* |
| 301 | 27 | C | C | G | 0.48 | 0.917728796 |  |
| 301 | 27 | C | C | T | 2.66 | 0.333920663 |  |
| 302 | 28 | T | T | A | 1.64 | 0.679900420 |  |
| 302 | 28 | T | T | C | 5.15 | 0.017977852 |  |
| 302 | 28 | T | T | G | 1.41 | 0.622350264 |  |
| 302 | 28 | T | T | T | 91.80 | 0.000000000 | \* |
| 303 | 29 | A | A | A | 94.63 | 0.000000000 | \* |
| 303 | 29 | A | A | C | 1.69 | 0.540562160 |  |
| 303 | 29 | A | A | G | 1.98 | 0.421373880 |  |
| 303 | 29 | A | A | T | 1.69 | 0.595507566 |  |
| 304 | 30 | C | C | A | 1.55 | 0.699465552 |  |
| 304 | 30 | C | C | C | 95.34 | 0.000000000 | \* |
| 304 | 30 | C | C | G | 2.33 | 0.318787321 |  |
| 304 | 30 | C | C | T | 0.78 | 0.840595962 |  |
| 305 | 31 | A | A | A | 88.01 | 0.000000000 | \* |
| 305 | 31 | A | A | C | 2.74 | 0.233085925 |  |
| 305 | 31 | A | A | G | 6.16 | 0.006404225 | \* |
| 305 | 31 | A | A | T | 3.08 | 0.246685545 |  |
| 306 | 32 | C | C | A | 1.42 | 0.728857269 |  |
| 306 | 32 | C | C | C | 93.45 | 0.000000000 | \* |
| 306 | 32 | C | C | G | 2.85 | 0.204023099 |  |
| 306 | 32 | C | C | T | 2.28 | 0.427571148 |  |
| 307 | 33 | G | G | A | 2.67 | 0.446824696 |  |
| 307 | 33 | G | G | C | 3.44 | 0.118937783 |  |
| 307 | 33 | G | G | G | 93.89 | 0.000000000 | \* |
| 307 | 33 | G | G | T | 0.00 | 0.903225806 |  |
| 308 | 34 | G | G | A | 1.35 | 0.745814935 |  |
| 308 | 34 | G | G | C | 1.89 | 0.472515983 |  |
| 308 | 34 | G | G | G | 96.77 | 0.000000000 | \* |
| 308 | 34 | G | G | T | 0.00 | 0.903225806 |  |
| 309 | 35 | G | G | A | 4.13 | 0.210488213 |  |
| 309 | 35 | G | G | C | 2.86 | 0.209122168 |  |
| 309 | 35 | G | G | G | 91.75 | 0.000000000 | \* |
| 309 | 35 | G | G | T | 1.27 | 0.721006779 |  |
| 310 | 36 | C | C | A | 2.15 | 0.561005623 |  |
| 310 | 36 | C | C | C | 95.34 | 0.000000000 | \* |
| 310 | 36 | C | C | G | 1.43 | 0.611674092 |  |
| 310 | 36 | C | C | T | 1.08 | 0.773484463 |  |
| 311 | 37 | A | A | A | 92.16 | 0.000000000 | \* |
| 311 | 37 | A | A | C | 2.94 | 0.193233018 |  |
| 311 | 37 | A | A | G | 0.00 | 0.955555556 |  |
| 311 | 37 | A | A | T | 4.90 | 0.055042956 |  |
| 312 | 38 | C | C | A | 2.31 | 0.523784345 |  |
| 312 | 38 | C | C | C | 94.68 | 0.000000000 | \* |
| 312 | 38 | C | C | G | 0.00 | 0.955555556 |  |
| 312 | 38 | C | C | T | 3.01 | 0.260263974 |  |
| 313 | 39 | C | C | A | 4.85 | 0.138674779 |  |
| 313 | 39 | C | C | C | 91.33 | 0.000000000 | \* |
| 313 | 39 | C | C | G | 3.83 | 0.080101041 |  |
| 313 | 39 | C | C | T | 0.00 | 0.903225806 |  |
| 314 | 40 | C | C | A | 4.91 | 0.133588719 |  |
| 314 | 40 | C | C | C | 89.66 | 0.000000000 | \* |
| 314 | 40 | C | C | G | 2.07 | 0.393531156 |  |
| 314 | 40 | C | C | T | 3.36 | 0.200144682 |  |
| 315 | 41 | T | T | A | 0.76 | 0.855870241 |  |
| 315 | 41 | T | T | C | 3.82 | 0.080039038 |  |
| 315 | 41 | T | T | G | 1.53 | 0.577135034 |  |
| 315 | 41 | T | T | T | 93.89 | 0.000000000 | \* |
| 316 | 42 | G | G | A | 3.00 | 0.381291332 |  |
| 316 | 42 | G | G | C | 3.22 | 0.147699854 |  |
| 316 | 42 | G | G | G | 92.49 | 0.000000000 | \* |
| 316 | 42 | G | G | T | 1.29 | 0.715996721 |  |
| 317 | 43 | A | A | A | 90.16 | 0.000000000 | \* |
| 317 | 43 | A | A | C | 1.17 | 0.733756698 |  |
| 317 | 43 | A | A | G | 1.41 | 0.622350264 |  |
| 317 | 43 | A | A | T | 7.26 | 0.005893568 | \* |
| 318 | 44 | C | C | A | 3.01 | 0.381071948 |  |
| 318 | 44 | C | C | C | 93.44 | 0.000000000 | \* |
| 318 | 44 | C | C | G | 1.37 | 0.636988500 |  |
| 318 | 44 | C | C | T | 2.19 | 0.452861195 |  |
| 319 | 45 | C | C | A | 5.00 | 0.126536047 |  |
| 319 | 45 | C | C | C | 89.71 | 0.000000000 | \* |
| 319 | 45 | C | C | G | 4.12 | 0.059580067 |  |
| 319 | 45 | C | C | T | 1.18 | 0.746832560 |  |
| 320 | 46 | G | G | A | 3.93 | 0.235097643 |  |
| 320 | 46 | G | G | C | 2.14 | 0.388766478 |  |
| 320 | 46 | G | G | G | 93.93 | 0.000000000 | \* |
| 320 | 46 | G | G | T | 0.00 | 0.903225806 |  |
| 321 | 47 | A | A | A | 94.51 | 0.000000000 | \* |
| 321 | 47 | A | A | C | 0.98 | 0.798310178 |  |
| 321 | 47 | A | A | G | 1.18 | 0.708000584 |  |
| 321 | 47 | A | A | T | 3.33 | 0.204159516 |  |
| 322 | 48 | C | C | A | 2.87 | 0.407704943 |  |
| 322 | 48 | C | C | C | 96.18 | 0.000000000 | \* |
| 322 | 48 | C | C | G | 0.96 | 0.787415172 |  |
| 322 | 48 | C | C | T | 0.00 | 0.903225806 |  |
| 323 | 49 | G | G | A | 9.35 | 0.007173885 | \* |
| 323 | 49 | G | G | C | 1.87 | 0.478604497 |  |
| 323 | 49 | G | G | G | 86.92 | 0.000000000 | \* |
| 323 | 49 | G | G | T | 1.87 | 0.543501307 |  |
| 324 | 50 | G | G | A | 3.17 | 0.350324564 |  |
| 324 | 50 | G | G | C | 2.12 | 0.396982716 |  |
| 324 | 50 | G | G | G | 93.65 | 0.000000000 | \* |
| 324 | 50 | G | G | T | 1.06 | 0.777818028 |  |
| 325 | 51 | C | C | A | 1.35 | 0.745019030 |  |
| 325 | 51 | C | C | C | 93.24 | 0.000000000 | \* |
| 325 | 51 | C | C | G | 3.60 | 0.099991252 |  |
| 325 | 51 | C | C | T | 1.80 | 0.563501209 |  |
| 326 | 52 | A | A | A | 87.23 | 0.000000000 | \* |
| 326 | 52 | A | A | C | 1.60 | 0.576970029 |  |
| 326 | 52 | A | A | G | 4.26 | 0.051679845 |  |
| 326 | 52 | A | A | T | 6.91 | 0.008284240 | \* |
| 327 | 53 | A | A | A | 95.18 | 0.000000000 | \* |
| 327 | 53 | A | A | C | 0.60 | 0.896771016 |  |
| 327 | 53 | A | A | G | 1.20 | 0.697462053 |  |
| 327 | 53 | A | A | T | 3.01 | 0.259734325 |  |
| 328 | 54 | G | G | A | 1.18 | 0.781126859 |  |
| 328 | 54 | G | G | C | 1.57 | 0.584730269 |  |
| 328 | 54 | G | G | G | 97.24 | 0.000000000 | \* |
| 328 | 54 | G | G | T | 0.00 | 0.903225806 |  |
| 329 | 55 | A | A | A | 92.92 | 0.000000000 | \* |
| 329 | 55 | A | A | C | 0.57 | 0.903320859 |  |
| 329 | 55 | A | A | G | 1.98 | 0.419601547 |  |
| 329 | 55 | A | A | T | 4.53 | 0.076198531 |  |
| 330 | 56 | A | A | A | 93.53 | 0.000000000 | \* |
| 330 | 56 | A | A | C | 0.00 | 0.941176471 |  |
| 330 | 56 | A | A | G | 0.97 | 0.782087801 |  |
| 330 | 56 | A | A | T | 5.50 | 0.031922992 |  |
| 331 | 57 | G | G | A | 1.89 | 0.622188794 |  |
| 331 | 57 | G | G | C | 2.83 | 0.214439010 |  |
| 331 | 57 | G | G | G | 91.98 | 0.000000000 | \* |
| 331 | 57 | G | G | T | 3.30 | 0.209134248 |  |
| 332 | 58 | T | T | A | 0.35 | 0.899746979 |  |
| 332 | 58 | T | T | C | 3.52 | 0.108941459 |  |
| 332 | 58 | T | T | G | 0.00 | 0.955555556 |  |
| 332 | 58 | T | T | T | 96.13 | 0.000000000 | \* |
| 333 | 59 | T | T | A | 1.25 | 0.766803301 |  |
| 333 | 59 | T | T | C | 1.25 | 0.705318140 |  |
| 333 | 59 | T | T | G | 4.06 | 0.063048487 |  |
| 333 | 59 | T | T | T | 93.44 | 0.000000000 | \* |
| 334 | 60 | C | C | A | 3.46 | 0.302672769 |  |
| 334 | 60 | C | C | C | 93.08 | 0.000000000 | \* |
| 334 | 60 | C | C | G | 2.20 | 0.354255495 |  |
| 334 | 60 | C | C | T | 1.26 | 0.724376803 |  |
| 335 | 61 | G | G | A | 7.35 | 0.028289173 |  |
| 335 | 61 | G | G | C | 1.63 | 0.563347401 |  |
| 335 | 61 | G | G | G | 88.98 | 0.000000000 | \* |
| 335 | 61 | G | G | T | 2.04 | 0.493531807 |  |
| 336 | 62 | A | A | A | 93.74 | 0.000000000 | \* |
| 336 | 62 | A | A | C | 1.16 | 0.737606375 |  |
| 336 | 62 | A | A | G | 0.93 | 0.796724994 |  |
| 336 | 62 | A | A | T | 4.18 | 0.103372288 |  |
| 337 | 63 | C | C | A | 2.59 | 0.464698949 |  |
| 337 | 63 | C | C | C | 94.83 | 0.000000000 | \* |
| 337 | 63 | C | C | G | 1.15 | 0.718004677 |  |
| 337 | 63 | C | C | T | 1.44 | 0.672722732 |  |
| 338 | 64 | A | A | A | 89.14 | 0.000000000 | \* |
| 338 | 64 | A | A | C | 1.81 | 0.499309006 |  |
| 338 | 64 | A | A | G | 3.17 | 0.152187922 |  |
| 338 | 64 | A | A | T | 5.88 | 0.022377524 |  |
| 339 | 65 | G | G | A | 1.10 | 0.797608474 |  |
| 339 | 65 | G | G | C | 2.20 | 0.372045609 |  |
| 339 | 65 | G | G | G | 95.88 | 0.000000000 | \* |
| 339 | 65 | G | G | T | 0.82 | 0.831376350 |  |
| 340 | 66 | C | C | A | 1.77 | 0.648780517 |  |
| 340 | 66 | C | C | C | 93.62 | 0.000000000 | \* |
| 340 | 66 | C | C | G | 2.13 | 0.375468458 |  |
| 340 | 66 | C | C | T | 2.48 | 0.375420836 |  |
| 341 | 67 | T | T | A | 0.25 | 0.905059208 |  |
| 341 | 67 | T | T | C | 0.74 | 0.866106249 |  |
| 341 | 67 | T | T | G | 2.72 | 0.228332824 |  |
| 341 | 67 | T | T | T | 96.29 | 0.000000000 | \* |
| 342 | 68 | C | C | A | 3.00 | 0.381532556 |  |
| 342 | 68 | C | C | C | 91.89 | 0.000000000 | \* |
| 342 | 68 | C | C | G | 3.30 | 0.133807067 |  |
| 342 | 68 | C | C | T | 1.80 | 0.563501209 |  |
| 343 | 69 | C | C | A | 2.70 | 0.440456260 |  |
| 343 | 69 | C | C | C | 93.61 | 0.000000000 | \* |
| 343 | 69 | C | C | G | 0.98 | 0.777948440 |  |
| 343 | 69 | C | C | T | 2.70 | 0.323605251 |  |
| 344 | 70 | C | C | A | 0.00 | 0.909090909 |  |
| 344 | 70 | C | C | C | 95.44 | 0.000000000 | \* |
| 344 | 70 | C | C | G | 2.28 | 0.332137547 |  |
| 344 | 70 | C | C | T | 2.28 | 0.426993825 |  |
| 345 | 71 | G | G | A | 8.20 | 0.015849533 |  |
| 345 | 71 | G | G | C | 0.39 | 0.927427489 |  |
| 345 | 71 | G | G | G | 91.02 | 0.000000000 | \* |
| 345 | 71 | G | G | T | 0.39 | 0.892343767 |  |
| 346 | 72 | C | C | A | 0.00 | 0.909090909 |  |
| 346 | 72 | C | C | C | 96.34 | 0.000000000 | \* |
| 346 | 72 | C | C | G | 0.73 | 0.858078244 |  |
| 346 | 72 | C | C | T | 2.93 | 0.275585044 |  |
| 347 | 73 | G | G | A | 7.82 | 0.020590362 |  |
| 347 | 73 | G | G | C | 2.06 | 0.415616528 |  |
| 347 | 73 | G | G | G | 89.71 | 0.000000000 | \* |
| 347 | 73 | G | G | T | 0.41 | 0.890709728 |  |
| 348 | 74 | A | A | A | 94.18 | 0.000000000 | \* |
| 348 | 74 | A | A | C | 1.66 | 0.552555742 |  |
| 348 | 74 | A | A | G | 0.55 | 0.903361895 |  |
| 348 | 74 | A | A | T | 3.60 | 0.165633465 |  |
| 349 | 75 | C | C | A | 1.67 | 0.673566104 |  |
| 349 | 75 | C | C | C | 92.78 | 0.000000000 | \* |
| 349 | 75 | C | C | G | 2.22 | 0.348369149 |  |
| 349 | 75 | C | C | T | 3.33 | 0.204159516 |  |

## For use in R

If you want to work with the results in R, here is output that you can copy and paste in your terminal to get:

The base information:

```
structure(list(focal.base = c("A", "C", "G", "T"), avg.percsignal = c(92.2813910117813, 
92.9534647812924, 93.050563465753, 94.2496614945792), avg.areasignal = c(348.735294117647, 
367.484848484848, 352.438596491228, 413), crit.perc.area = c(8.87052941807297, 
5.64770563957746, 5.77034969058603, 6.72241675490619), mu = c(3.0164464028139, 
2.10261971064844, 2.03710946287654, 2.45249247780382), fillibens = c(0.995285469735132, 
0.994673673234652, 0.992279358287884, 0.992091239926587)), .Names = c("focal.base", 
"avg.percsignal", "avg.areasignal", "crit.perc.area", "mu", "fillibens"
), row.names = c(NA, -4L), class = "data.frame")
```

the data.frame that contains information on the guide region:

```
structure(list(A.area = c(317, 373, 7, 616, 18, 20, 26, 21, 13, 
22, 292, 9, 582, 16, 16, 18, 0, 24, 0, 2, 0, 12, 1, 16, 4, 256, 
11, 7, 335, 6, 257, 5, 7, 5, 13, 6, 282, 10, 19, 19, 3, 14, 385, 
11, 17, 11, 482, 9, 20, 12, 3, 164, 316, 3, 328, 289, 4, 1, 4, 
11, 18, 404, 9, 197, 4, 5, 1, 10, 11, 0, 21, 0, 19, 340, 6), 
    C.area = c(6, 3, 8, 7, 358, 16, 12, 4, 230, 409, 7, 10, 6, 
    363, 320, 5, 5, 5, 3, 8, 0, 13, 14, 6, 312, 12, 390, 22, 
    6, 368, 8, 328, 9, 7, 9, 266, 9, 409, 358, 347, 15, 15, 5, 
    342, 305, 6, 5, 302, 4, 8, 207, 3, 2, 4, 2, 0, 6, 10, 4, 
    296, 4, 5, 330, 4, 8, 264, 3, 306, 381, 251, 1, 263, 5, 6, 
    334), G.area = c(4, 5, 294, 14, 18, 12, 647, 402, 11, 14, 
    4, 293, 7, 11, 12, 342, 6, 540, 20, 17, 12, 481, 4, 383, 
    7, 11, 2, 6, 7, 9, 18, 10, 246, 359, 289, 4, 0, 0, 15, 8, 
    6, 431, 6, 5, 14, 263, 6, 3, 186, 354, 8, 8, 4, 247, 7, 3, 
    195, 0, 13, 7, 218, 4, 4, 7, 349, 6, 11, 11, 4, 6, 233, 2, 
    218, 2, 8), T.area = c(6, 20, 2, 25, 8, 432, 8, 4, 6, 6, 
    8, 0, 24, 7, 5, 8, 269, 10, 320, 357, 375, 4, 303, 5, 0, 
    12, 11, 392, 6, 3, 9, 8, 0, 0, 4, 3, 15, 13, 0, 13, 369, 
    6, 31, 8, 4, 0, 17, 0, 4, 4, 4, 13, 10, 0, 16, 17, 7, 273, 
    299, 4, 5, 18, 5, 13, 3, 7, 389, 6, 11, 6, 1, 8, 1, 13, 12
    ), Tot.area = c(333, 401, 311, 662, 402, 480, 693, 431, 260, 
    451, 311, 312, 619, 397, 353, 373, 280, 579, 343, 384, 387, 
    510, 322, 410, 323, 291, 414, 427, 354, 386, 292, 351, 262, 
    371, 315, 279, 306, 432, 392, 387, 393, 466, 427, 366, 340, 
    280, 510, 314, 214, 378, 222, 188, 332, 254, 353, 309, 212, 
    284, 320, 318, 245, 431, 348, 221, 364, 282, 404, 333, 407, 
    263, 256, 273, 243, 361, 360), A.perc = c(95.1951951951952, 
    93.0174563591022, 2.2508038585209, 93.0513595166163, 4.47761194029851, 
    4.16666666666667, 3.75180375180375, 4.87238979118329, 5, 
    4.8780487804878, 93.8906752411576, 2.88461538461538, 94.0226171243942, 
    4.03022670025189, 4.53257790368272, 4.82573726541555, 0, 
    4.14507772020725, 0, 0.520833333333333, 0, 2.35294117647059, 
    0.31055900621118, 3.90243902439024, 1.23839009287926, 87.9725085910653, 
    2.65700483091787, 1.63934426229508, 94.6327683615819, 1.55440414507772, 
    88.013698630137, 1.42450142450142, 2.67175572519084, 1.34770889487871, 
    4.12698412698413, 2.1505376344086, 92.156862745098, 2.31481481481481, 
    4.8469387755102, 4.90956072351421, 0.763358778625954, 3.00429184549356, 
    90.1639344262295, 3.00546448087432, 5, 3.92857142857143, 
    94.5098039215686, 2.86624203821656, 9.34579439252336, 3.17460317460317, 
    1.35135135135135, 87.2340425531915, 95.1807228915663, 1.18110236220472, 
    92.9178470254957, 93.5275080906149, 1.88679245283019, 0.352112676056338, 
    1.25, 3.45911949685535, 7.3469387755102, 93.7354988399072, 
    2.58620689655172, 89.1402714932127, 1.0989010989011, 1.77304964539007, 
    0.247524752475248, 3.003003003003, 2.7027027027027, 0, 8.203125, 
    0, 7.81893004115226, 94.1828254847645, 1.66666666666667), 
    C.perc = c(1.8018018018018, 0.748129675810474, 2.57234726688103, 
    1.05740181268882, 89.0547263681592, 3.33333333333333, 1.73160173160173, 
    0.928074245939675, 88.4615384615385, 90.6873614190687, 2.2508038585209, 
    3.20512820512821, 0.969305331179321, 91.4357682619647, 90.6515580736544, 
    1.34048257372654, 1.78571428571429, 0.863557858376511, 0.87463556851312, 
    2.08333333333333, 0, 2.54901960784314, 4.34782608695652, 
    1.46341463414634, 96.5944272445821, 4.12371134020619, 94.2028985507246, 
    5.15222482435597, 1.69491525423729, 95.3367875647668, 2.73972602739726, 
    93.4472934472934, 3.43511450381679, 1.88679245283019, 2.85714285714286, 
    95.3405017921147, 2.94117647058824, 94.6759259259259, 91.3265306122449, 
    89.6640826873385, 3.81679389312977, 3.21888412017167, 1.17096018735363, 
    93.4426229508197, 89.7058823529412, 2.14285714285714, 0.980392156862745, 
    96.1783439490446, 1.86915887850467, 2.11640211640212, 93.2432432432432, 
    1.59574468085106, 0.602409638554217, 1.5748031496063, 0.56657223796034, 
    0, 2.83018867924528, 3.52112676056338, 1.25, 93.0817610062893, 
    1.63265306122449, 1.16009280742459, 94.8275862068966, 1.80995475113122, 
    2.1978021978022, 93.6170212765958, 0.742574257425743, 91.8918918918919, 
    93.6117936117936, 95.4372623574145, 0.390625, 96.3369963369963, 
    2.05761316872428, 1.66204986149584, 92.7777777777778), G.perc = c(1.2012012012012, 
    1.24688279301746, 94.5337620578778, 2.11480362537764, 4.47761194029851, 
    2.5, 93.3621933621934, 93.2714617169374, 4.23076923076923, 
    3.10421286031042, 1.28617363344051, 93.9102564102564, 1.13085621970921, 
    2.77078085642317, 3.39943342776204, 91.6890080428954, 2.14285714285714, 
    93.2642487046632, 5.83090379008746, 4.42708333333333, 3.10077519379845, 
    94.3137254901961, 1.24223602484472, 93.4146341463415, 2.1671826625387, 
    3.78006872852234, 0.483091787439614, 1.40515222482436, 1.9774011299435, 
    2.33160621761658, 6.16438356164384, 2.84900284900285, 93.8931297709924, 
    96.7654986522911, 91.7460317460317, 1.4336917562724, 0, 0, 
    3.8265306122449, 2.0671834625323, 1.52671755725191, 92.4892703862661, 
    1.40515222482436, 1.36612021857923, 4.11764705882353, 93.9285714285714, 
    1.17647058823529, 0.955414012738854, 86.9158878504673, 93.6507936507936, 
    3.6036036036036, 4.25531914893617, 1.20481927710843, 97.244094488189, 
    1.98300283286119, 0.970873786407767, 91.9811320754717, 0, 
    4.0625, 2.20125786163522, 88.9795918367347, 0.928074245939675, 
    1.14942528735632, 3.16742081447964, 95.8791208791209, 2.12765957446809, 
    2.72277227722772, 3.3033033033033, 0.982800982800983, 2.28136882129278, 
    91.015625, 0.732600732600733, 89.7119341563786, 0.554016620498615, 
    2.22222222222222), T.perc = c(1.8018018018018, 4.98753117206983, 
    0.643086816720257, 3.77643504531722, 1.99004975124378, 90, 
    1.15440115440115, 0.928074245939675, 2.30769230769231, 1.33037694013304, 
    2.57234726688103, 0, 3.87722132471729, 1.7632241813602, 1.41643059490085, 
    2.14477211796247, 96.0714285714286, 1.72711571675302, 93.2944606413994, 
    92.96875, 96.8992248062015, 0.784313725490196, 94.0993788819876, 
    1.21951219512195, 0, 4.12371134020619, 2.65700483091787, 
    91.8032786885246, 1.69491525423729, 0.77720207253886, 3.08219178082192, 
    2.27920227920228, 0, 0, 1.26984126984127, 1.0752688172043, 
    4.90196078431373, 3.00925925925926, 0, 3.35917312661499, 
    93.8931297709924, 1.28755364806867, 7.25995316159251, 2.18579234972678, 
    1.17647058823529, 0, 3.33333333333333, 0, 1.86915887850467, 
    1.05820105820106, 1.8018018018018, 6.91489361702128, 3.01204819277108, 
    0, 4.53257790368272, 5.50161812297735, 3.30188679245283, 
    96.1267605633803, 93.4375, 1.25786163522013, 2.04081632653061, 
    4.17633410672854, 1.4367816091954, 5.88235294117647, 0.824175824175824, 
    2.4822695035461, 96.2871287128713, 1.8018018018018, 2.7027027027027, 
    2.28136882129278, 0.390625, 2.93040293040293, 0.411522633744856, 
    3.601108033241, 3.33333333333333), base.call = c("A", "A", 
    "G", "A", "C", "T", "G", "G", "C", "C", "A", "G", "A", "C", 
    "C", "G", "T", "G", "T", "T", "T", "G", "T", "G", "C", "A", 
    "C", "T", "A", "C", "A", "C", "G", "G", "G", "C", "A", "C", 
    "C", "C", "T", "G", "A", "C", "C", "G", "A", "C", "G", "G", 
    "C", "A", "A", "G", "A", "A", "G", "T", "T", "C", "G", "A", 
    "C", "A", "G", "C", "T", "C", "C", "C", "G", "C", "G", "A", 
    "C"), index = 275:349, guide.seq = c("A", "A", "G", "A", 
    "C", "T", "G", "G", "C", "C", "A", "G", "A", "C", "C", "G", 
    "T", "G", "T", "T", "T", "G", "T", "G", "C", "A", "C", "T", 
    "A", "C", "A", "C", "G", "G", "G", "C", "A", "C", "C", "C", 
    "T", "G", "A", "C", "C", "G", "A", "C", "G", "G", "C", "A", 
    "A", "G", "A", "A", "G", "T", "T", "C", "G", "A", "C", "A", 
    "G", "C", "T", "C", "C", "C", "G", "C", "G", "A", "C"), T.pval = c(0.563501209176513, 
    0.0509876464229206, 0.863610211943073, 0.143898337887681, 
    0.508131020139906, 0, 0.75277469974235, 0.809057757706433, 
    0.420014041580419, 0.703759035145803, 0.353623509726871, 
    0.903225806446376, 0.132554885656108, 0.575023740041829, 
    0.678716843486632, 0.4642028922426, 0, 0.585841590545235, 
    0, 0, 0, 0.839237010128772, 0, 0.735057608655211, 0.903225806446376, 
    0.108050587575535, 0.333920662754729, 0, 0.595507565731883, 
    0.840595961946106, 0.246685545113341, 0.427571147685691, 
    0.903225806446376, 0.903225806446376, 0.721006779442576, 
    0.773484462668194, 0.0550429555312841, 0.260263974013986, 
    0.903225806446376, 0.200144682002221, 0, 0.715996721255062, 
    0.00589356797076079, 0.452861195157328, 0.746832559786968, 
    0.903225806446376, 0.204159515547842, 0.903225806446376, 
    0.543501307006385, 0.777818027919869, 0.563501209176513, 
    0.00828423973135939, 0.259734325271741, 0.903225806446376, 
    0.0761985310673441, 0.0319229920529813, 0.209134248388744, 
    0, 0, 0.724376803140164, 0.493531806688977, 0.103372287898106, 
    0.672722732041883, 0.0223775244461646, 0.831376349528448, 
    0.375420835850269, 0, 0.563501209176513, 0.323605251014888, 
    0.426993825492515, 0.892343767123824, 0.275585044015826, 
    0.890709728101278, 0.16563346510726, 0.204159515547842), 
    C.pval = c(0.502190365116071, 0.864734028109216, 0.270948827556596, 
    0.773061305914867, 0, 0.131802109372947, 0.527274873120231, 
    0.814660591972556, 0, 0, 0.356364127019733, 0.14971769658157, 
    0.801833095067961, 0, 0, 0.672049962576459, 0.507896186269846, 
    0.83379105512349, 0.830593175766379, 0.407401272911892, 0.941176470588235, 
    0.276580627094233, 0.0449884712901413, 0.626241874611582, 
    0, 0.0575570602033464, 0, 0.0179778516477617, 0.540562159699, 
    0, 0.23308592524723, 0, 0.118937783443455, 0.472515982671196, 
    0.209122168058936, 0, 0.193233017994763, 0, 0, 0, 0.080039037639696, 
    0.147699853691328, 0.733756698316694, 0, 0, 0.388766478044965, 
    0.798310178456127, 0, 0.47860449731719, 0.396982715847811, 
    0, 0.576970028958919, 0.896771015778593, 0.584730268754343, 
    0.90332085912778, 0.941176470588235, 0.214439009570096, 0.108941459363995, 
    0.705318140039743, 0, 0.563347401397309, 0.737606375419916, 
    0, 0.499309006285909, 0.372045609321518, 0, 0.866106249463543, 
    0, 0, 0, 0.927427489395045, 0, 0.415616527619015, 0.552555742008015, 
    0), G.pval = c(0.698809667310449, 0.68174941665041, 0, 0.379260840446384, 
    0.0409572721881953, 0.276950881388236, 0, 0, 0.0530129744910893, 
    0.161463772108236, 0.667017596928035, 0, 0.724840014193282, 
    0.218820124035102, 0.122013972757538, 0, 0.371018536013196, 
    0, 0.00934198720345714, 0.0431932406544736, 0.161982000131568, 
    0, 0.683488752728764, 0, 0.363971112785023, 0.0839211510421791, 
    0.917728796185029, 0.622350263691713, 0.421373880303829, 
    0.318787321266095, 0.00640422545004105, 0.204023099341162, 
    0, 0, 0, 0.611674092185426, 0.955555555555295, 0.955555555555295, 
    0.0801010408346703, 0.393531156149698, 0.577135034149487, 
    0, 0.622350263691713, 0.636988499994198, 0.05958006715108, 
    0, 0.708000583813649, 0.787415172413594, 0, 0, 0.099991252182718, 
    0.051679845432971, 0.697462053268096, 0, 0.419601546816631, 
    0.782087800994604, 0, 0.955555555555295, 0.0630484872251218, 
    0.354255494568792, 0, 0.796724994166875, 0.718004676712127, 
    0.152187921837643, 0, 0.375468457696151, 0.228332823662755, 
    0.133807067462527, 0.777948439800273, 0.33213754654188, 0, 
    0.858078243880609, 0, 0.903361895280137, 0.348369149220753
    ), A.pval = c(0, 0, 0.538177807606047, 0, 0.172286349783231, 
    0.205833499056418, 0.258962197803792, 0.136587334771805, 
    0.12653604742351, 0.136126998558278, 0, 0.404123094449004, 
    0, 0.222207736889519, 0.166873432760018, 0.140435204465732, 
    0.909090909074362, 0.208355023019296, 0.909090909074362, 
    0.886044031416404, 0.909090909074362, 0.515286200851465, 
    0.902147639811867, 0.238508557522837, 0.769246678466955, 
    0, 0.44987870046935, 0.679900419718524, 0, 0.699465552397126, 
    0, 0.728857269264133, 0.446824695696687, 0.745814935443685, 
    0.210488212903449, 0.561005622848355, 0, 0.523784345159513, 
    0.138674778559707, 0.133588719327082, 0.855870240898754, 
    0.381291332469408, 0, 0.381071947970466, 0.12653604742351, 
    0.235097643060376, 0, 0.407704943246599, 0.00717388456363743, 
    0.35032456407943, 0.745019029641502, 0, 0, 0.781126859095018, 
    0, 0, 0.622188794399128, 0.899746978577367, 0.766803300958611, 
    0.302672769014197, 0.0282891733702341, 0, 0.46469894917486, 
    0, 0.797608474069787, 0.648780517327049, 0.905059207645999, 
    0.38153255555183, 0.440456259764858, 0.909090909074362, 0.0158495325509955, 
    0.909090909074362, 0.0205903618112868, 0, 0.673566104141046
    ), guide.position = 1:75), .Names = c("A.area", "C.area", 
"G.area", "T.area", "Tot.area", "A.perc", "C.perc", "G.perc", 
"T.perc", "base.call", "index", "guide.seq", "T.pval", "C.pval", 
"G.pval", "A.pval", "guide.position"), row.names = 275:349, class = "data.frame")
```

*Report generated using EditR v1.0.8*
