# Supplementary material for: Mechanistic and genetic basis of single-strand templated repair at Cas12a-induced DNA breaks in Chlamydomonas reinhardtii
Source: Nat Commun. 2021 Nov 19;12:6751. doi: 10.1038/s41467-021-27004-1 (PMC8604939; doi:10.1038/s41467-021-27004-1)
Supplement: Supplementary file 22 — Source Data [file 41467_2021_27004_MOESM22_ESM.zip › Source Data/EditR analysis/EditR outputs/Antisense/rep2_ssODN_antisense_0_16_32.html]

EditR v1.0.8 report


# EditR v1.0.8 report

- Data QA
  - Filtering data
  - Percent noise peak area
  - Base information
- Predicted editing
  - Editing bar plot
  - Editing table plot
  - Table of editing results
- For use in R

## Data QA

### Filtering data

What the data looked like prefiltering:

and the post filtering signal / noise plot:

### Percent noise peak area

### Base information

Here’s information about the signal of each base, the critical percent value where any higher value would be called as significant, and Filliben’s correlation for how well the noise was modelled by the zero adjusted gamma distribution.

| Base | Average percent signal | Average peak area | Critical percent value | model mu | Fillibens correlation |
| --- | --- | --- | --- | --- | --- |
| A | 95.09729 | 449.3235 | 8.947117 | 2.986727 | 0.9974912 |
| C | 93.13161 | 461.4030 | 4.366624 | 1.872869 | 0.9951369 |
| G | 92.36223 | 446.6607 | 5.363391 | 2.063693 | 0.9971579 |
| T | 94.88304 | 543.4545 | 5.652260 | 2.247687 | 0.9947451 |

## Predicted editing

### Editing bar plot

### Editing table plot

### Table of editing results


Here’s the entire guide region

| Sanger position | Guide position | Guide sequence | Sanger base call | Focal base | Focal base peak area | p value |  |
| --- | --- | --- | --- | --- | --- | --- | --- |
| 276 | 1 | A | A | A | 97.04 | 0.00000000 | \* |
| 276 | 1 | A | A | C | 1.98 | 0.37494246 |  |
| 276 | 1 | A | A | G | 0.99 | 0.81718478 |  |
| 276 | 1 | A | A | T | 0.00 | 0.92473118 |  |
| 277 | 2 | A | A | A | 95.77 | 0.00000000 | \* |
| 277 | 2 | A | A | C | 1.01 | 0.81920296 |  |
| 277 | 2 | A | A | G | 1.61 | 0.58025242 |  |
| 277 | 2 | A | A | T | 1.61 | 0.62211508 |  |
| 278 | 3 | G | G | A | 2.83 | 0.39442036 |  |
| 278 | 3 | G | G | C | 3.86 | 0.02445883 |  |
| 278 | 3 | G | G | G | 92.29 | 0.00000000 | \* |
| 278 | 3 | G | G | T | 1.03 | 0.81505821 |  |
| 279 | 4 | A | A | A | 95.27 | 0.00000000 | \* |
| 279 | 4 | A | A | C | 1.21 | 0.73186795 |  |
| 279 | 4 | A | A | G | 1.09 | 0.78121139 |  |
| 279 | 4 | A | A | T | 2.43 | 0.34545604 |  |
| 280 | 5 | C | C | A | 4.39 | 0.17438143 |  |
| 280 | 5 | C | C | C | 90.62 | 0.00000000 | \* |
| 280 | 5 | C | C | G | 2.79 | 0.20898526 |  |
| 280 | 5 | C | C | T | 2.20 | 0.41645725 |  |
| 281 | 6 | T | T | A | 4.33 | 0.18008594 |  |
| 281 | 6 | T | T | C | 2.09 | 0.33060287 |  |
| 281 | 6 | T | T | G | 2.41 | 0.30363702 |  |
| 281 | 6 | T | T | T | 91.17 | 0.00000000 | \* |
| 282 | 7 | G | G | A | 3.83 | 0.23762544 |  |
| 282 | 7 | G | G | C | 1.51 | 0.59050360 |  |
| 282 | 7 | G | G | G | 93.39 | 0.00000000 | \* |
| 282 | 7 | G | G | T | 1.28 | 0.73948171 |  |
| 283 | 8 | G | G | A | 5.14 | 0.11306445 |  |
| 283 | 8 | G | G | C | 1.60 | 0.54769635 |  |
| 283 | 8 | G | G | G | 92.02 | 0.00000000 | \* |
| 283 | 8 | G | G | T | 1.24 | 0.75096696 |  |
| 284 | 9 | C | C | A | 4.59 | 0.15605824 |  |
| 284 | 9 | C | C | C | 89.91 | 0.00000000 | \* |
| 284 | 9 | C | C | G | 2.14 | 0.38501596 |  |
| 284 | 9 | C | C | T | 3.36 | 0.14346630 |  |
| 285 | 10 | C | C | A | 1.99 | 0.56819037 |  |
| 285 | 10 | C | C | C | 92.41 | 0.00000000 | \* |
| 285 | 10 | C | C | G | 3.07 | 0.15655506 |  |
| 285 | 10 | C | C | T | 2.53 | 0.31610498 |  |
| 286 | 11 | A | A | A | 94.71 | 0.00000000 | \* |
| 286 | 11 | A | A | C | 2.02 | 0.35870312 |  |
| 286 | 11 | A | A | G | 2.02 | 0.42763777 |  |
| 286 | 11 | A | A | T | 1.26 | 0.74498051 |  |
| 287 | 12 | G | G | A | 3.40 | 0.29698222 |  |
| 287 | 12 | G | G | C | 2.36 | 0.23811065 |  |
| 287 | 12 | G | G | G | 93.46 | 0.00000000 | \* |
| 287 | 12 | G | G | T | 0.79 | 0.87194430 |  |
| 288 | 13 | A | A | A | 98.21 | 0.00000000 | \* |
| 288 | 13 | A | A | C | 1.15 | 0.76115013 |  |
| 288 | 13 | A | A | G | 0.64 | 0.91083443 |  |
| 288 | 13 | A | A | T | 0.00 | 0.92473118 |  |
| 289 | 14 | C | C | A | 4.02 | 0.21373555 |  |
| 289 | 14 | C | C | C | 94.16 | 0.00000000 | \* |
| 289 | 14 | C | C | G | 0.20 | 0.95475892 |  |
| 289 | 14 | C | C | T | 1.61 | 0.62211508 |  |
| 290 | 15 | C | C | A | 5.00 | 0.12290629 |  |
| 290 | 15 | C | C | C | 91.36 | 0.00000000 | \* |
| 290 | 15 | C | C | G | 2.05 | 0.41709859 |  |
| 290 | 15 | C | C | T | 1.59 | 0.62892777 |  |
| 291 | 16 | G | G | A | 5.12 | 0.11471680 |  |
| 291 | 16 | G | G | C | 1.49 | 0.59814676 |  |
| 291 | 16 | G | G | G | 92.11 | 0.00000000 | \* |
| 291 | 16 | G | G | T | 1.28 | 0.73841448 |  |
| 292 | 17 | T | T | A | 0.00 | 0.88181818 |  |
| 292 | 17 | T | T | C | 0.00 | 0.95238095 |  |
| 292 | 17 | T | T | G | 2.47 | 0.28768563 |  |
| 292 | 17 | T | T | T | 97.53 | 0.00000000 | \* |
| 293 | 18 | G | G | A | 4.08 | 0.20774693 |  |
| 293 | 18 | G | G | C | 0.95 | 0.83929347 |  |
| 293 | 18 | G | G | G | 94.16 | 0.00000000 | \* |
| 293 | 18 | G | G | T | 0.82 | 0.86605259 |  |
| 294 | 19 | T | T | A | 1.89 | 0.59112601 |  |
| 294 | 19 | T | T | C | 0.21 | 0.95204075 |  |
| 294 | 19 | T | T | G | 4.40 | 0.03391695 |  |
| 294 | 19 | T | T | T | 93.50 | 0.00000000 | \* |
| 295 | 20 | T | T | A | 0.97 | 0.78725854 |  |
| 295 | 20 | T | T | C | 0.58 | 0.93009916 |  |
| 295 | 20 | T | T | G | 3.31 | 0.12178320 |  |
| 295 | 20 | T | T | T | 95.14 | 0.00000000 | \* |
| 296 | 21 | T | T | A | 0.00 | 0.88181818 |  |
| 296 | 21 | T | T | C | 1.90 | 0.40474327 |  |
| 296 | 21 | T | T | G | 2.48 | 0.28488398 |  |
| 296 | 21 | T | T | T | 95.62 | 0.00000000 | \* |
| 297 | 22 | G | G | A | 2.31 | 0.49783011 |  |
| 297 | 22 | G | G | C | 1.39 | 0.64989717 |  |
| 297 | 22 | G | G | G | 95.53 | 0.00000000 | \* |
| 297 | 22 | G | G | T | 0.77 | 0.87476385 |  |
| 298 | 23 | T | T | A | 0.00 | 0.88181818 |  |
| 298 | 23 | T | T | C | 1.42 | 0.63608448 |  |
| 298 | 23 | T | T | G | 1.42 | 0.65773322 |  |
| 298 | 23 | T | T | T | 97.17 | 0.00000000 | \* |
| 299 | 24 | G | G | A | 3.80 | 0.24189074 |  |
| 299 | 24 | G | G | C | 1.33 | 0.67813802 |  |
| 299 | 24 | G | G | G | 93.55 | 0.00000000 | \* |
| 299 | 24 | G | G | T | 1.33 | 0.72195490 |  |
| 300 | 25 | C | C | A | 1.42 | 0.69611424 |  |
| 300 | 25 | C | C | C | 94.56 | 0.00000000 | \* |
| 300 | 25 | C | C | G | 1.89 | 0.47212931 |  |
| 300 | 25 | C | C | T | 2.13 | 0.43871638 |  |
| 301 | 26 | A | A | A | 89.92 | 0.00000000 | \* |
| 301 | 26 | A | A | C | 3.64 | 0.03510026 |  |
| 301 | 26 | A | A | G | 3.92 | 0.06052797 |  |
| 301 | 26 | A | A | T | 2.52 | 0.31901441 |  |
| 302 | 27 | C | C | A | 2.02 | 0.56127296 |  |
| 302 | 27 | C | C | C | 95.76 | 0.00000000 | \* |
| 302 | 27 | C | C | G | 0.00 | 0.95604396 |  |
| 302 | 27 | C | C | T | 2.22 | 0.40790516 |  |
| 303 | 28 | T | T | A | 1.42 | 0.69666434 |  |
| 303 | 28 | T | T | C | 2.65 | 0.15978837 |  |
| 303 | 28 | T | T | G | 1.77 | 0.51773685 |  |
| 303 | 28 | T | T | T | 94.16 | 0.00000000 | \* |
| 304 | 29 | A | A | A | 94.47 | 0.00000000 | \* |
| 304 | 29 | A | A | C | 1.49 | 0.59970456 |  |
| 304 | 29 | A | A | G | 2.13 | 0.38930946 |  |
| 304 | 29 | A | A | T | 1.91 | 0.51183679 |  |
| 305 | 30 | C | C | A | 1.67 | 0.64077351 |  |
| 305 | 30 | C | C | C | 95.00 | 0.00000000 | \* |
| 305 | 30 | C | C | G | 1.25 | 0.72252222 |  |
| 305 | 30 | C | C | T | 2.08 | 0.45354787 |  |
| 306 | 31 | A | A | A | 92.35 | 0.00000000 | \* |
| 306 | 31 | A | A | C | 2.55 | 0.18460422 |  |
| 306 | 31 | A | A | G | 5.10 | 0.01409528 |  |
| 306 | 31 | A | A | T | 0.00 | 0.92473118 |  |
| 307 | 32 | C | C | A | 1.64 | 0.64778940 |  |
| 307 | 32 | C | C | C | 92.99 | 0.00000000 | \* |
| 307 | 32 | C | C | G | 2.10 | 0.39759143 |  |
| 307 | 32 | C | C | T | 3.27 | 0.15769034 |  |
| 308 | 33 | G | G | A | 4.48 | 0.16608343 |  |
| 308 | 33 | G | G | C | 2.69 | 0.15287483 |  |
| 308 | 33 | G | G | G | 90.75 | 0.00000000 | \* |
| 308 | 33 | G | G | T | 2.09 | 0.45145293 |  |
| 309 | 34 | G | G | A | 2.36 | 0.48846790 |  |
| 309 | 34 | G | G | C | 1.07 | 0.79377804 |  |
| 309 | 34 | G | G | G | 95.72 | 0.00000000 | \* |
| 309 | 34 | G | G | T | 0.86 | 0.85737110 |  |
| 310 | 35 | G | G | A | 4.10 | 0.20474903 |  |
| 310 | 35 | G | G | C | 1.54 | 0.57563786 |  |
| 310 | 35 | G | G | G | 93.59 | 0.00000000 | \* |
| 310 | 35 | G | G | T | 0.77 | 0.87498422 |  |
| 311 | 36 | C | C | A | 2.63 | 0.43206684 |  |
| 311 | 36 | C | C | C | 96.20 | 0.00000000 | \* |
| 311 | 36 | C | C | G | 1.17 | 0.75298052 |  |
| 311 | 36 | C | C | T | 0.00 | 0.92473118 |  |
| 312 | 37 | A | A | A | 95.48 | 0.00000000 | \* |
| 312 | 37 | A | A | C | 3.19 | 0.07239927 |  |
| 312 | 37 | A | A | G | 0.00 | 0.95604396 |  |
| 312 | 37 | A | A | T | 1.33 | 0.72143988 |  |
| 313 | 38 | C | C | A | 2.40 | 0.47850539 |  |
| 313 | 38 | C | C | C | 95.01 | 0.00000000 | \* |
| 313 | 38 | C | C | G | 1.11 | 0.77517585 |  |
| 313 | 38 | C | C | T | 1.48 | 0.66936996 |  |
| 314 | 39 | C | C | A | 5.50 | 0.09136734 |  |
| 314 | 39 | C | C | C | 91.04 | 0.00000000 | \* |
| 314 | 39 | C | C | G | 2.24 | 0.35312802 |  |
| 314 | 39 | C | C | T | 1.22 | 0.75715517 |  |
| 315 | 40 | C | C | A | 4.14 | 0.20048575 |  |
| 315 | 40 | C | C | C | 90.89 | 0.00000000 | \* |
| 315 | 40 | C | C | G | 2.28 | 0.34171113 |  |
| 315 | 40 | C | C | T | 2.69 | 0.27457664 |  |
| 316 | 41 | T | T | A | 1.55 | 0.66755516 |  |
| 316 | 41 | T | T | C | 1.16 | 0.75563855 |  |
| 316 | 41 | T | T | G | 1.74 | 0.52892396 |  |
| 316 | 41 | T | T | T | 95.55 | 0.00000000 | \* |
| 317 | 42 | G | G | A | 3.16 | 0.33550683 |  |
| 317 | 42 | G | G | C | 1.66 | 0.51483219 |  |
| 317 | 42 | G | G | G | 93.84 | 0.00000000 | \* |
| 317 | 42 | G | G | T | 1.33 | 0.72098799 |  |
| 318 | 43 | A | A | A | 90.79 | 0.00000000 | \* |
| 318 | 43 | A | A | C | 1.29 | 0.69677027 |  |
| 318 | 43 | A | A | G | 1.66 | 0.56139833 |  |
| 318 | 43 | A | A | T | 6.26 | 0.00455943 | \* |
| 319 | 44 | C | C | A | 2.43 | 0.47324086 |  |
| 319 | 44 | C | C | C | 97.57 | 0.00000000 | \* |
| 319 | 44 | C | C | G | 0.00 | 0.95604396 |  |
| 319 | 44 | C | C | T | 0.00 | 0.92473118 |  |
| 320 | 45 | C | C | A | 6.32 | 0.05509555 |  |
| 320 | 45 | C | C | C | 88.06 | 0.00000000 | \* |
| 320 | 45 | C | C | G | 4.45 | 0.03200433 |  |
| 320 | 45 | C | C | T | 1.17 | 0.77328671 |  |
| 321 | 46 | G | G | A | 5.60 | 0.08596194 |  |
| 321 | 46 | G | G | C | 2.40 | 0.22502162 |  |
| 321 | 46 | G | G | G | 92.00 | 0.00000000 | \* |
| 321 | 46 | G | G | T | 0.00 | 0.92473118 |  |
| 322 | 47 | A | A | A | 96.93 | 0.00000000 | \* |
| 322 | 47 | A | A | C | 1.07 | 0.79257242 |  |
| 322 | 47 | A | A | G | 0.61 | 0.91544625 |  |
| 322 | 47 | A | A | T | 1.38 | 0.70404843 |  |
| 323 | 48 | C | C | A | 2.92 | 0.37752868 |  |
| 323 | 48 | C | C | C | 94.16 | 0.00000000 | \* |
| 323 | 48 | C | C | G | 0.73 | 0.89091062 |  |
| 323 | 48 | C | C | T | 2.19 | 0.41834272 |  |
| 324 | 49 | G | G | A | 8.55 | 0.01304131 |  |
| 324 | 49 | G | G | C | 2.23 | 0.27857545 |  |
| 324 | 49 | G | G | G | 87.73 | 0.00000000 | \* |
| 324 | 49 | G | G | T | 1.49 | 0.66642279 |  |
| 325 | 50 | G | G | A | 3.81 | 0.24027320 |  |
| 325 | 50 | G | G | C | 1.20 | 0.73694797 |  |
| 325 | 50 | G | G | G | 93.59 | 0.00000000 | \* |
| 325 | 50 | G | G | T | 1.40 | 0.69622835 |  |
| 326 | 51 | C | C | A | 3.45 | 0.29017791 |  |
| 326 | 51 | C | C | C | 91.03 | 0.00000000 | \* |
| 326 | 51 | C | C | G | 3.10 | 0.15176593 |  |
| 326 | 51 | C | C | T | 2.41 | 0.34934294 |  |
| 327 | 52 | A | A | A | 97.71 | 0.00000000 | \* |
| 327 | 52 | A | A | C | 1.38 | 0.65504603 |  |
| 327 | 52 | A | A | G | 0.92 | 0.83960307 |  |
| 327 | 52 | A | A | T | 0.00 | 0.92473118 |  |
| 328 | 53 | A | A | A | 98.52 | 0.00000000 | \* |
| 328 | 53 | A | A | C | 0.25 | 0.95169343 |  |
| 328 | 53 | A | A | G | 1.23 | 0.72960368 |  |
| 328 | 53 | A | A | T | 0.00 | 0.92473118 |  |
| 329 | 54 | G | G | A | 1.85 | 0.59899093 |  |
| 329 | 54 | G | G | C | 2.16 | 0.30314454 |  |
| 329 | 54 | G | G | G | 94.44 | 0.00000000 | \* |
| 329 | 54 | G | G | T | 1.54 | 0.64620643 |  |
| 330 | 55 | A | A | A | 97.07 | 0.00000000 | \* |
| 330 | 55 | A | A | C | 0.00 | 0.95238095 |  |
| 330 | 55 | A | A | G | 2.25 | 0.34943118 |  |
| 330 | 55 | A | A | T | 0.68 | 0.89071012 |  |
| 331 | 56 | A | A | A | 96.02 | 0.00000000 | \* |
| 331 | 56 | A | A | C | 1.00 | 0.82334659 |  |
| 331 | 56 | A | A | G | 1.00 | 0.81474509 |  |
| 331 | 56 | A | A | T | 1.99 | 0.48548473 |  |
| 332 | 57 | G | G | A | 2.17 | 0.52741079 |  |
| 332 | 57 | G | G | C | 2.90 | 0.11275884 |  |
| 332 | 57 | G | G | G | 91.67 | 0.00000000 | \* |
| 332 | 57 | G | G | T | 3.26 | 0.15931529 |  |
| 333 | 58 | T | T | A | 1.00 | 0.78266817 |  |
| 333 | 58 | T | T | C | 2.74 | 0.14115066 |  |
| 333 | 58 | T | T | G | 0.50 | 0.93360247 |  |
| 333 | 58 | T | T | T | 95.76 | 0.00000000 | \* |
| 334 | 59 | T | T | A | 3.40 | 0.29725319 |  |
| 334 | 59 | T | T | C | 1.36 | 0.66260249 |  |
| 334 | 59 | T | T | G | 3.63 | 0.08506707 |  |
| 334 | 59 | T | T | T | 91.61 | 0.00000000 | \* |
| 335 | 60 | C | C | A | 3.55 | 0.27481334 |  |
| 335 | 60 | C | C | C | 92.39 | 0.00000000 | \* |
| 335 | 60 | C | C | G | 2.54 | 0.26868726 |  |
| 335 | 60 | C | C | T | 1.52 | 0.65355245 |  |
| 336 | 61 | G | G | A | 6.71 | 0.04315936 |  |
| 336 | 61 | G | G | C | 2.35 | 0.24025408 |  |
| 336 | 61 | G | G | G | 89.60 | 0.00000000 | \* |
| 336 | 61 | G | G | T | 1.34 | 0.71717668 |  |
| 337 | 62 | A | A | A | 97.41 | 0.00000000 | \* |
| 337 | 62 | A | A | C | 1.29 | 0.69451562 |  |
| 337 | 62 | A | A | G | 1.29 | 0.70552606 |  |
| 337 | 62 | A | A | T | 0.00 | 0.92473118 |  |
| 338 | 63 | C | C | A | 2.40 | 0.47985093 |  |
| 338 | 63 | C | C | C | 93.90 | 0.00000000 | \* |
| 338 | 63 | C | C | G | 1.53 | 0.61388550 |  |
| 338 | 63 | C | C | T | 2.18 | 0.42195677 |  |
| 339 | 64 | A | A | A | 95.09 | 0.00000000 | \* |
| 339 | 64 | A | A | C | 2.11 | 0.32354143 |  |
| 339 | 64 | A | A | G | 2.81 | 0.20634720 |  |
| 339 | 64 | A | A | T | 0.00 | 0.92473118 |  |
| 340 | 65 | G | G | A | 2.32 | 0.49582691 |  |
| 340 | 65 | G | G | C | 2.32 | 0.24903688 |  |
| 340 | 65 | G | G | G | 93.04 | 0.00000000 | \* |
| 340 | 65 | G | G | T | 2.32 | 0.37713146 |  |
| 341 | 66 | C | C | A | 2.17 | 0.52869522 |  |
| 341 | 66 | C | C | C | 93.77 | 0.00000000 | \* |
| 341 | 66 | C | C | G | 1.90 | 0.47000877 |  |
| 341 | 66 | C | C | T | 2.17 | 0.42542233 |  |
| 342 | 67 | T | T | A | 1.11 | 0.76114395 |  |
| 342 | 67 | T | T | C | 0.92 | 0.84856336 |  |
| 342 | 67 | T | T | G | 2.40 | 0.30496921 |  |
| 342 | 67 | T | T | T | 95.56 | 0.00000000 | \* |
| 343 | 68 | C | C | A | 2.13 | 0.53752589 |  |
| 343 | 68 | C | C | C | 93.85 | 0.00000000 | \* |
| 343 | 68 | C | C | G | 2.36 | 0.31604093 |  |
| 343 | 68 | C | C | T | 1.65 | 0.60566811 |  |
| 344 | 69 | C | C | A | 1.52 | 0.67281573 |  |
| 344 | 69 | C | C | C | 93.71 | 0.00000000 | \* |
| 344 | 69 | C | C | G | 0.57 | 0.92280569 |  |
| 344 | 69 | C | C | T | 4.19 | 0.05853119 |  |
| 345 | 70 | C | C | A | 0.00 | 0.88181818 |  |
| 345 | 70 | C | C | C | 96.13 | 0.00000000 | \* |
| 345 | 70 | C | C | G | 1.49 | 0.62863114 |  |
| 345 | 70 | C | C | T | 2.38 | 0.35899261 |  |
| 346 | 71 | G | G | A | 6.78 | 0.04120044 |  |
| 346 | 71 | G | G | C | 0.88 | 0.86141233 |  |
| 346 | 71 | G | G | G | 89.97 | 0.00000000 | \* |
| 346 | 71 | G | G | T | 2.36 | 0.36527083 |  |
| 347 | 72 | C | C | A | 0.00 | 0.88181818 |  |
| 347 | 72 | C | C | C | 96.29 | 0.00000000 | \* |
| 347 | 72 | C | C | G | 0.86 | 0.85754902 |  |
| 347 | 72 | C | C | T | 2.86 | 0.23589948 |  |
| 348 | 73 | G | G | A | 8.59 | 0.01270866 |  |
| 348 | 73 | G | G | C | 3.99 | 0.01950726 |  |
| 348 | 73 | G | G | G | 85.58 | 0.00000000 | \* |
| 348 | 73 | G | G | T | 1.84 | 0.53836295 |  |
| 349 | 74 | A | A | A | 98.06 | 0.00000000 | \* |
| 349 | 74 | A | A | C | 1.29 | 0.69620856 |  |
| 349 | 74 | A | A | G | 0.65 | 0.90937316 |  |
| 349 | 74 | A | A | T | 0.00 | 0.92473118 |  |
| 350 | 75 | C | C | A | 2.16 | 0.52946311 |  |
| 350 | 75 | C | C | C | 90.26 | 0.00000000 | \* |
| 350 | 75 | C | C | G | 4.55 | 0.02842405 |  |
| 350 | 75 | C | C | T | 3.03 | 0.20008628 |  |

## For use in R

If you want to work with the results in R, here is output that you can copy and paste in your terminal to get:

The base information:

```
structure(list(focal.base = c("A", "C", "G", "T"), avg.percsignal = c(95.0972872236909, 
93.1316098405754, 92.3622289106034, 94.8830440833364), avg.areasignal = c(449.323529411765, 
461.402985074627, 446.660714285714, 543.454545454545), crit.perc.area = c(8.94711725392743, 
4.36662398187748, 5.36339107788231, 5.6522605476484), mu = c(2.98672679381033, 
1.87286912287796, 2.06369336851522, 2.24768696096196), fillibens = c(0.997491202720124, 
0.995136930737645, 0.99715786219044, 0.994745138096446)), .Names = c("focal.base", 
"avg.percsignal", "avg.areasignal", "crit.perc.area", "mu", "fillibens"
), row.names = c(NA, -4L), class = "data.frame")
```

the data.frame that contains information on the guide region:

```
structure(list(A.area = c(393, 476, 11, 785, 22, 27, 33, 29, 
15, 11, 376, 13, 770, 20, 22, 24, 0, 30, 9, 5, 0, 15, 0, 20, 
6, 321, 10, 8, 444, 8, 326, 7, 15, 11, 16, 9, 359, 13, 27, 20, 
8, 19, 493, 11, 27, 21, 632, 12, 23, 19, 10, 213, 400, 6, 431, 
386, 6, 4, 15, 14, 20, 527, 11, 271, 11, 8, 6, 9, 8, 0, 23, 0, 
28, 456, 10), C.area = c(8, 5, 15, 10, 454, 13, 13, 9, 294, 511, 
8, 9, 9, 468, 402, 7, 0, 7, 1, 3, 10, 9, 6, 7, 400, 13, 474, 
15, 7, 456, 9, 398, 9, 5, 6, 329, 12, 514, 447, 439, 6, 10, 7, 
442, 376, 9, 7, 387, 6, 6, 264, 3, 1, 7, 0, 4, 8, 11, 6, 364, 
7, 7, 431, 6, 11, 346, 5, 397, 492, 323, 3, 337, 13, 6, 417), 
    G.area = c(4, 8, 359, 9, 14, 15, 805, 519, 7, 17, 8, 357, 
    5, 1, 9, 432, 9, 693, 21, 17, 13, 620, 6, 493, 8, 14, 0, 
    10, 10, 6, 18, 9, 304, 447, 365, 4, 0, 6, 11, 11, 9, 564, 
    9, 0, 19, 345, 4, 3, 236, 467, 9, 2, 5, 306, 10, 4, 253, 
    2, 16, 10, 267, 7, 7, 8, 441, 7, 13, 10, 3, 5, 305, 3, 279, 
    3, 21), T.area = c(0, 8, 4, 20, 11, 568, 11, 7, 11, 14, 5, 
    3, 0, 8, 7, 6, 356, 6, 446, 489, 502, 5, 412, 7, 9, 9, 11, 
    532, 9, 10, 0, 14, 7, 4, 3, 0, 5, 8, 6, 13, 494, 8, 34, 0, 
    5, 0, 9, 9, 4, 7, 7, 0, 0, 5, 3, 8, 9, 384, 404, 6, 4, 0, 
    10, 0, 11, 8, 517, 7, 22, 8, 8, 10, 6, 0, 14), Tot.area = c(405, 
    497, 389, 824, 501, 623, 862, 564, 327, 553, 397, 382, 784, 
    497, 440, 469, 365, 736, 477, 514, 525, 649, 424, 527, 423, 
    357, 495, 565, 470, 480, 353, 428, 335, 467, 390, 342, 376, 
    541, 491, 483, 517, 601, 543, 453, 427, 375, 652, 411, 269, 
    499, 290, 218, 406, 324, 444, 402, 276, 401, 441, 394, 298, 
    541, 459, 285, 474, 369, 541, 423, 525, 336, 339, 350, 326, 
    465, 462), A.perc = c(97.037037037037, 95.7746478873239, 
    2.82776349614396, 95.2669902912621, 4.39121756487026, 4.3338683788122, 
    3.82830626450116, 5.14184397163121, 4.58715596330275, 1.98915009041591, 
    94.7103274559194, 3.40314136125654, 98.2142857142857, 4.02414486921529, 
    5, 5.11727078891258, 0, 4.07608695652174, 1.88679245283019, 
    0.972762645914397, 0, 2.31124807395994, 0, 3.79506641366224, 
    1.41843971631206, 89.9159663865546, 2.02020202020202, 1.41592920353982, 
    94.468085106383, 1.66666666666667, 92.3512747875354, 1.63551401869159, 
    4.47761194029851, 2.35546038543897, 4.1025641025641, 2.63157894736842, 
    95.4787234042553, 2.40295748613678, 5.4989816700611, 4.1407867494824, 
    1.54738878143133, 3.16139767054908, 90.7918968692449, 2.42825607064018, 
    6.3231850117096, 5.6, 96.9325153374233, 2.91970802919708, 
    8.55018587360595, 3.80761523046092, 3.44827586206897, 97.7064220183486, 
    98.5221674876847, 1.85185185185185, 97.0720720720721, 96.0199004975124, 
    2.17391304347826, 0.997506234413965, 3.40136054421769, 3.55329949238579, 
    6.71140939597315, 97.4121996303142, 2.39651416122004, 95.0877192982456, 
    2.32067510548523, 2.1680216802168, 1.1090573012939, 2.12765957446809, 
    1.52380952380952, 0, 6.78466076696165, 0, 8.58895705521472, 
    98.0645161290323, 2.16450216450216), C.perc = c(1.97530864197531, 
    1.00603621730382, 3.8560411311054, 1.21359223300971, 90.6187624750499, 
    2.08667736757624, 1.50812064965197, 1.59574468085106, 89.9082568807339, 
    92.4050632911392, 2.01511335012594, 2.35602094240838, 1.14795918367347, 
    94.1649899396378, 91.3636363636364, 1.49253731343284, 0, 
    0.951086956521739, 0.209643605870021, 0.583657587548638, 
    1.9047619047619, 1.38674884437596, 1.41509433962264, 1.32827324478178, 
    94.5626477541371, 3.64145658263305, 95.7575757575758, 2.65486725663717, 
    1.48936170212766, 95, 2.54957507082153, 92.9906542056075, 
    2.6865671641791, 1.07066381156317, 1.53846153846154, 96.1988304093567, 
    3.19148936170213, 95.0092421441774, 91.0386965376782, 90.8902691511387, 
    1.1605415860735, 1.66389351081531, 1.28913443830571, 97.5717439293598, 
    88.056206088993, 2.4, 1.07361963190184, 94.1605839416058, 
    2.23048327137546, 1.20240480961924, 91.0344827586207, 1.37614678899083, 
    0.246305418719212, 2.16049382716049, 0, 0.995024875621891, 
    2.89855072463768, 2.7431421446384, 1.36054421768707, 92.3857868020305, 
    2.3489932885906, 1.29390018484288, 93.8997821350763, 2.10526315789474, 
    2.32067510548523, 93.7669376693767, 0.924214417744917, 93.8534278959811, 
    93.7142857142857, 96.1309523809524, 0.884955752212389, 96.2857142857143, 
    3.98773006134969, 1.29032258064516, 90.2597402597403), G.perc = c(0.987654320987654, 
    1.60965794768612, 92.2879177377892, 1.09223300970874, 2.79441117764471, 
    2.40770465489567, 93.3874709976798, 92.0212765957447, 2.14067278287462, 
    3.07414104882459, 2.01511335012594, 93.455497382199, 0.637755102040816, 
    0.201207243460765, 2.04545454545455, 92.1108742004264, 2.46575342465753, 
    94.1576086956522, 4.40251572327044, 3.30739299610895, 2.47619047619048, 
    95.5315870570108, 1.41509433962264, 93.5483870967742, 1.89125295508274, 
    3.92156862745098, 0, 1.76991150442478, 2.12765957446809, 
    1.25, 5.09915014164306, 2.10280373831776, 90.7462686567164, 
    95.7173447537473, 93.5897435897436, 1.16959064327485, 0, 
    1.1090573012939, 2.24032586558045, 2.27743271221532, 1.74081237911025, 
    93.8435940099834, 1.65745856353591, 0, 4.44964871194379, 
    92, 0.613496932515337, 0.72992700729927, 87.7323420074349, 
    93.5871743486974, 3.10344827586207, 0.917431192660551, 1.23152709359606, 
    94.4444444444444, 2.25225225225225, 0.995024875621891, 91.6666666666667, 
    0.498753117206983, 3.6281179138322, 2.53807106598985, 89.5973154362416, 
    1.29390018484288, 1.52505446623094, 2.80701754385965, 93.0379746835443, 
    1.8970189701897, 2.40295748613678, 2.36406619385343, 0.571428571428571, 
    1.48809523809524, 89.9705014749263, 0.857142857142857, 85.5828220858896, 
    0.645161290322581, 4.54545454545455), T.perc = c(0, 1.60965794768612, 
    1.02827763496144, 2.42718446601942, 2.19560878243513, 91.1717495987159, 
    1.27610208816705, 1.24113475177305, 3.36391437308869, 2.53164556962025, 
    1.25944584382872, 0.785340314136126, 0, 1.60965794768612, 
    1.59090909090909, 1.27931769722815, 97.5342465753425, 0.815217391304348, 
    93.5010482180294, 95.136186770428, 95.6190476190476, 0.770416024653313, 
    97.1698113207547, 1.32827324478178, 2.12765957446809, 2.52100840336134, 
    2.22222222222222, 94.1592920353982, 1.91489361702128, 2.08333333333333, 
    0, 3.27102803738318, 2.08955223880597, 0.856531049250535, 
    0.769230769230769, 0, 1.32978723404255, 1.47874306839187, 
    1.22199592668024, 2.69151138716356, 95.5512572533849, 1.33111480865225, 
    6.26151012891344, 0, 1.17096018735363, 0, 1.38036809815951, 
    2.18978102189781, 1.48698884758364, 1.40280561122244, 2.41379310344828, 
    0, 0, 1.54320987654321, 0.675675675675676, 1.99004975124378, 
    3.26086956521739, 95.7605985037407, 91.609977324263, 1.52284263959391, 
    1.34228187919463, 0, 2.17864923747277, 0, 2.32067510548523, 
    2.1680216802168, 95.5637707948244, 1.6548463356974, 4.19047619047619, 
    2.38095238095238, 2.3598820058997, 2.85714285714286, 1.84049079754601, 
    0, 3.03030303030303), base.call = c("A", "A", "G", "A", "C", 
    "T", "G", "G", "C", "C", "A", "G", "A", "C", "C", "G", "T", 
    "G", "T", "T", "T", "G", "T", "G", "C", "A", "C", "T", "A", 
    "C", "A", "C", "G", "G", "G", "C", "A", "C", "C", "C", "T", 
    "G", "A", "C", "C", "G", "A", "C", "G", "G", "C", "A", "A", 
    "G", "A", "A", "G", "T", "T", "C", "G", "A", "C", "A", "G", 
    "C", "T", "C", "C", "C", "G", "C", "G", "A", "C"), index = 276:350, 
    guide.seq = c("A", "A", "G", "A", "C", "T", "G", "G", "C", 
    "C", "A", "G", "A", "C", "C", "G", "T", "G", "T", "T", "T", 
    "G", "T", "G", "C", "A", "C", "T", "A", "C", "A", "C", "G", 
    "G", "G", "C", "A", "C", "C", "C", "T", "G", "A", "C", "C", 
    "G", "A", "C", "G", "G", "C", "A", "A", "G", "A", "A", "G", 
    "T", "T", "C", "G", "A", "C", "A", "G", "C", "T", "C", "C", 
    "C", "G", "C", "G", "A", "C"), T.pval = c(0.924731182795698, 
    0.622115076844041, 0.815058209472588, 0.3454560393832, 0.416457247800519, 
    0, 0.739481713370298, 0.750966957426973, 0.14346630279098, 
    0.316104975937831, 0.744980506693328, 0.871944301425691, 
    0.924731182795698, 0.622115076844041, 0.628927768464629, 
    0.738414477008309, 0, 0.866052585852882, 0, 0, 0, 0.874763849835091, 
    0, 0.721954901190216, 0.438716380884259, 0.319014413553922, 
    0.407905162598676, 0, 0.511836785325952, 0.453547874284038, 
    0.924731182795698, 0.157690335988218, 0.45145292841562, 0.857371104133405, 
    0.874984219933738, 0.924731182795698, 0.72143988046229, 0.669369960319787, 
    0.757155166730461, 0.27457663918912, 0, 0.720987993203566, 
    0.00455942957668931, 0.924731182795698, 0.773286706393291, 
    0.924731182795698, 0.704048430474835, 0.418342720321418, 
    0.666422788883157, 0.696228350787153, 0.349342944704271, 
    0.924731182795698, 0.924731182795698, 0.646206431627606, 
    0.890710123440457, 0.485484730009784, 0.15931528814639, 0, 
    0, 0.653552454323807, 0.717176683312309, 0.924731182795698, 
    0.421956767220894, 0.924731182795698, 0.377131456606465, 
    0.425422330865788, 0, 0.605668105937907, 0.0585311924430955, 
    0.358992612270206, 0.365270828958384, 0.235899483483128, 
    0.538362950619033, 0.924731182795698, 0.200086279050257), 
    C.pval = c(0.374942461792781, 0.819202961886632, 0.0244588335237119, 
    0.731867954866387, 0, 0.330602867116658, 0.590503601868975, 
    0.547696353882752, 0, 0, 0.358703118126945, 0.23811064562415, 
    0.761150133433782, 0, 0, 0.598146755111978, 0.952380952380885, 
    0.839293471282088, 0.952040746208553, 0.930099155821558, 
    0.404743273115925, 0.649897169708245, 0.636084475824526, 
    0.678138018078222, 0, 0.0351002568437341, 0, 0.159788366091259, 
    0.599704555984135, 0, 0.184604222957666, 0, 0.152874833210526, 
    0.793778042316883, 0.575637856603574, 0, 0.0723992650833194, 
    0, 0, 0, 0.755638554916707, 0.514832192821946, 0.696770269036624, 
    0, 0, 0.225021619711774, 0.792572417780873, 0, 0.278575449278869, 
    0.736947973281098, 0, 0.655046028417565, 0.951693431239703, 
    0.303144544745024, 0.952380952380885, 0.823346589888478, 
    0.112758835220538, 0.141150655007549, 0.662602490200914, 
    0, 0.240254084680346, 0.694515616531532, 0, 0.323541427541135, 
    0.24903687633227, 0, 0.848563360914936, 0, 0, 0, 0.861412326142397, 
    0, 0.0195072562141646, 0.696208557785295, 0), G.pval = c(0.817184782154234, 
    0.580252420355334, 0, 0.781211392474467, 0.208985262479427, 
    0.303637021860857, 0, 0, 0.385015963615037, 0.156555060483466, 
    0.427637766493072, 0, 0.910834425307128, 0.954758916495091, 
    0.417098590196218, 0, 0.287685630933491, 0, 0.0339169525532397, 
    0.121783204683562, 0.284883976084692, 0, 0.657733220271572, 
    0, 0.472129313519284, 0.0605279712728917, 0.956043956043634, 
    0.517736847763027, 0.389309464321535, 0.722522215198131, 
    0.0140952768005298, 0.39759142683713, 0, 0, 0, 0.7529805222528, 
    0.956043956043634, 0.775175849442335, 0.353128018689233, 
    0.34171112783586, 0.52892396178411, 0, 0.561398333140406, 
    0.956043956043634, 0.0320043313730151, 0, 0.915446251936135, 
    0.890910620985517, 0, 0, 0.151765925054976, 0.839603074733536, 
    0.729603684574021, 0, 0.349431184927371, 0.814745087149889, 
    0, 0.933602466840807, 0.0850670703517201, 0.26868726248748, 
    0, 0.705526063130162, 0.613885500199531, 0.206347195849089, 
    0, 0.470008772883293, 0.304969214267647, 0.316040928027174, 
    0.922805692055492, 0.628631136224355, 0, 0.857549017699703, 
    0, 0.909373164971377, 0.0284240549399165), A.pval = c(0, 
    0, 0.39442035791571, 0, 0.174381432597891, 0.180085937269813, 
    0.237625439112266, 0.113064449104981, 0.156058239338939, 
    0.568190367499759, 0, 0.296982222822, 0, 0.213735548327183, 
    0.122906292045727, 0.114716799619331, 0.881818181818107, 
    0.207746930538054, 0.591126006767401, 0.787258541317944, 
    0.881818181818107, 0.497830110524387, 0.881818181818107, 
    0.241890740420538, 0.696114238092838, 0, 0.56127295731725, 
    0.696664336991583, 0, 0.640773509892125, 0, 0.64778940020026, 
    0.166083425935276, 0.488467899776354, 0.20474902943913, 0.432066835669645, 
    0, 0.478505391638406, 0.091367343228574, 0.200485748879694, 
    0.667555159639842, 0.33550682679091, 0, 0.47324086032592, 
    0.0550955543626888, 0.0859619378722787, 0, 0.377528680701144, 
    0.0130413143948624, 0.240273195443091, 0.290177910485764, 
    0, 0, 0.59899093078015, 0, 0, 0.527410789574843, 0.782668172223129, 
    0.297253186391316, 0.274813340424956, 0.0431593631105023, 
    0, 0.479850926403141, 0, 0.495826914738447, 0.528695222377941, 
    0.761143950503289, 0.537525893037491, 0.672815730296129, 
    0.881818181818107, 0.041200441617968, 0.881818181818107, 
    0.0127086600551604, 0, 0.529463107305317), guide.position = 1:75), .Names = c("A.area", 
"C.area", "G.area", "T.area", "Tot.area", "A.perc", "C.perc", 
"G.perc", "T.perc", "base.call", "index", "guide.seq", "T.pval", 
"C.pval", "G.pval", "A.pval", "guide.position"), row.names = 276:350, class = "data.frame")
```

*Report generated using EditR v1.0.8*
