# Supplementary material for: Mechanistic and genetic basis of single-strand templated repair at Cas12a-induced DNA breaks in Chlamydomonas reinhardtii
Source: Nat Commun. 2021 Nov 19;12:6751. doi: 10.1038/s41467-021-27004-1 (PMC8604939; doi:10.1038/s41467-021-27004-1)
Supplement: Supplementary file 22 — Source Data [file 41467_2021_27004_MOESM22_ESM.zip › Source Data/EditR analysis/EditR outputs/Antisense/rep2_ssODN_antisense_16.html]

EditR v1.0.8 report


# EditR v1.0.8 report

- Data QA
  - Filtering data
  - Percent noise peak area
  - Base information
- Predicted editing
  - Editing bar plot
  - Editing table plot
  - Table of editing results
- For use in R

## Data QA

### Filtering data

What the data looked like prefiltering:

and the post filtering signal / noise plot:

### Percent noise peak area

### Base information

Here’s information about the signal of each base, the critical percent value where any higher value would be called as significant, and Filliben’s correlation for how well the noise was modelled by the zero adjusted gamma distribution.

| Base | Average percent signal | Average peak area | Critical percent value | model mu | Fillibens correlation |
| --- | --- | --- | --- | --- | --- |
| A | 93.27861 | 232.7353 | 9.156231 | 3.314129 | 0.9938584 |
| C | 91.68325 | 251.1642 | 6.239446 | 2.409816 | 0.9841478 |
| G | 92.74683 | 240.6316 | 6.537110 | 2.495213 | 0.9934446 |
| T | 93.87110 | 292.6744 | 7.339984 | 2.639486 | 0.9889453 |

## Predicted editing

### Editing bar plot

### Editing table plot

### Table of editing results


Here’s the entire guide region

| Sanger position | Guide position | Guide sequence | Sanger base call | Focal base | Focal base peak area | p value |  |
| --- | --- | --- | --- | --- | --- | --- | --- |
| 277 | 1 | A | A | A | 91.52 | 0.000000000 | \* |
| 277 | 1 | A | A | C | 3.12 | 0.228370690 |  |
| 277 | 1 | A | A | G | 3.12 | 0.246467594 |  |
| 277 | 1 | A | A | T | 2.23 | 0.469367919 |  |
| 278 | 2 | A | A | A | 93.21 | 0.000000000 | \* |
| 278 | 2 | A | A | C | 1.89 | 0.561098775 |  |
| 278 | 2 | A | A | G | 1.13 | 0.788763029 |  |
| 278 | 2 | A | A | T | 3.77 | 0.177143076 |  |
| 279 | 3 | G | G | A | 2.31 | 0.540205189 |  |
| 279 | 3 | G | G | C | 6.48 | 0.007617369 | \* |
| 279 | 3 | G | G | G | 89.81 | 0.000000000 | \* |
| 279 | 3 | G | G | T | 1.39 | 0.692058513 |  |
| 280 | 4 | A | A | A | 93.16 | 0.000000000 | \* |
| 280 | 4 | A | A | C | 0.88 | 0.859014024 |  |
| 280 | 4 | A | A | G | 1.99 | 0.536175823 |  |
| 280 | 4 | A | A | T | 3.97 | 0.153515605 |  |
| 281 | 5 | C | C | A | 7.42 | 0.032299107 |  |
| 281 | 5 | C | C | C | 86.57 | 0.000000000 | \* |
| 281 | 5 | C | C | G | 2.83 | 0.308812226 |  |
| 281 | 5 | C | C | T | 3.18 | 0.265656426 |  |
| 282 | 6 | T | T | A | 0.00 | 0.836363636 |  |
| 282 | 6 | T | T | C | 2.35 | 0.416495900 |  |
| 282 | 6 | T | T | G | 2.35 | 0.429925182 |  |
| 282 | 6 | T | T | T | 95.30 | 0.000000000 | \* |
| 283 | 7 | G | G | A | 4.40 | 0.203142746 |  |
| 283 | 7 | G | G | C | 0.00 | 0.928571429 |  |
| 283 | 7 | G | G | G | 94.51 | 0.000000000 | \* |
| 283 | 7 | G | G | T | 1.10 | 0.762814332 |  |
| 284 | 8 | G | G | A | 5.99 | 0.080120073 |  |
| 284 | 8 | G | G | C | 1.58 | 0.663442061 |  |
| 284 | 8 | G | G | G | 91.17 | 0.000000000 | \* |
| 284 | 8 | G | G | T | 1.26 | 0.724148403 |  |
| 285 | 9 | C | C | A | 5.08 | 0.137899198 |  |
| 285 | 9 | C | C | C | 87.01 | 0.000000000 | \* |
| 285 | 9 | C | C | G | 5.65 | 0.024887322 |  |
| 285 | 9 | C | C | T | 2.26 | 0.462404223 |  |
| 286 | 10 | C | C | A | 2.01 | 0.601227914 |  |
| 286 | 10 | C | C | C | 90.60 | 0.000000000 | \* |
| 286 | 10 | C | C | G | 4.03 | 0.116168032 |  |
| 286 | 10 | C | C | T | 3.36 | 0.236433719 |  |
| 287 | 11 | A | A | A | 94.20 | 0.000000000 | \* |
| 287 | 11 | A | A | C | 1.93 | 0.546157621 |  |
| 287 | 11 | A | A | G | 3.86 | 0.133883267 |  |
| 287 | 11 | A | A | T | 0.00 | 0.882978723 |  |
| 288 | 12 | G | G | A | 3.86 | 0.268977647 |  |
| 288 | 12 | G | G | C | 2.42 | 0.397392027 |  |
| 288 | 12 | G | G | G | 92.27 | 0.000000000 | \* |
| 288 | 12 | G | G | T | 1.45 | 0.676377121 |  |
| 289 | 13 | A | A | A | 94.04 | 0.000000000 | \* |
| 289 | 13 | A | A | C | 0.92 | 0.851751458 |  |
| 289 | 13 | A | A | G | 0.92 | 0.837613162 |  |
| 289 | 13 | A | A | T | 4.13 | 0.137117530 |  |
| 290 | 14 | C | C | A | 6.25 | 0.068366315 |  |
| 290 | 14 | C | C | C | 87.85 | 0.000000000 | \* |
| 290 | 14 | C | C | G | 2.78 | 0.320070276 |  |
| 290 | 14 | C | C | T | 3.12 | 0.275403706 |  |
| 291 | 15 | C | C | A | 5.74 | 0.093661495 |  |
| 291 | 15 | C | C | C | 90.16 | 0.000000000 | \* |
| 291 | 15 | C | C | G | 3.28 | 0.218332101 |  |
| 291 | 15 | C | C | T | 0.82 | 0.819520377 |  |
| 292 | 16 | G | G | A | 5.51 | 0.107282525 |  |
| 292 | 16 | G | G | C | 3.15 | 0.223683431 |  |
| 292 | 16 | G | G | G | 90.16 | 0.000000000 | \* |
| 292 | 16 | G | G | T | 1.18 | 0.743714350 |  |
| 293 | 17 | T | T | A | 0.00 | 0.836363636 |  |
| 293 | 17 | T | T | C | 2.51 | 0.370427054 |  |
| 293 | 17 | T | T | G | 2.51 | 0.385676736 |  |
| 293 | 17 | T | T | T | 94.97 | 0.000000000 | \* |
| 294 | 18 | G | G | A | 2.80 | 0.444882477 |  |
| 294 | 18 | G | G | C | 1.27 | 0.759527958 |  |
| 294 | 18 | G | G | G | 95.42 | 0.000000000 | \* |
| 294 | 18 | G | G | T | 0.51 | 0.862760261 |  |
| 295 | 19 | T | T | A | 3.29 | 0.356502744 |  |
| 295 | 19 | T | T | C | 0.00 | 0.928571429 |  |
| 295 | 19 | T | T | G | 6.17 | 0.014608423 |  |
| 295 | 19 | T | T | T | 90.53 | 0.000000000 | \* |
| 296 | 20 | T | T | A | 0.00 | 0.836363636 |  |
| 296 | 20 | T | T | C | 2.63 | 0.338982791 |  |
| 296 | 20 | T | T | G | 3.76 | 0.146605220 |  |
| 296 | 20 | T | T | T | 93.61 | 0.000000000 | \* |
| 297 | 21 | T | T | A | 0.00 | 0.836363636 |  |
| 297 | 21 | T | T | C | 0.77 | 0.879959451 |  |
| 297 | 21 | T | T | G | 2.70 | 0.337832855 |  |
| 297 | 21 | T | T | T | 96.53 | 0.000000000 | \* |
| 298 | 22 | G | G | A | 1.80 | 0.644512982 |  |
| 298 | 22 | G | G | C | 0.60 | 0.904861483 |  |
| 298 | 22 | G | G | G | 94.91 | 0.000000000 | \* |
| 298 | 22 | G | G | T | 2.69 | 0.360597661 |  |
| 299 | 23 | T | T | A | 0.00 | 0.836363636 |  |
| 299 | 23 | T | T | C | 1.35 | 0.737482214 |  |
| 299 | 23 | T | T | G | 2.69 | 0.340761208 |  |
| 299 | 23 | T | T | T | 95.96 | 0.000000000 | \* |
| 300 | 24 | G | G | A | 3.31 | 0.353719026 |  |
| 300 | 24 | G | G | C | 1.10 | 0.807092121 |  |
| 300 | 24 | G | G | G | 93.01 | 0.000000000 | \* |
| 300 | 24 | G | G | T | 2.57 | 0.387459331 |  |
| 301 | 25 | C | C | A | 1.35 | 0.727694746 |  |
| 301 | 25 | C | C | C | 90.13 | 0.000000000 | \* |
| 301 | 25 | C | C | G | 4.04 | 0.115244529 |  |
| 301 | 25 | C | C | T | 4.48 | 0.105132513 |  |
| 302 | 26 | A | A | A | 91.30 | 0.000000000 | \* |
| 302 | 26 | A | A | C | 3.80 | 0.124929138 |  |
| 302 | 26 | A | A | G | 4.89 | 0.052373270 |  |
| 302 | 26 | A | A | T | 0.00 | 0.882978723 |  |
| 303 | 27 | C | C | A | 4.74 | 0.167591995 |  |
| 303 | 27 | C | C | C | 92.09 | 0.000000000 | \* |
| 303 | 27 | C | C | G | 0.00 | 0.911111111 |  |
| 303 | 27 | C | C | T | 3.16 | 0.268832358 |  |
| 304 | 28 | T | T | A | 1.48 | 0.704973348 |  |
| 304 | 28 | T | T | C | 4.06 | 0.098209274 |  |
| 304 | 28 | T | T | G | 1.85 | 0.579782639 |  |
| 304 | 28 | T | T | T | 92.62 | 0.000000000 | \* |
| 305 | 29 | A | A | A | 92.34 | 0.000000000 | \* |
| 305 | 29 | A | A | C | 4.44 | 0.067981847 |  |
| 305 | 29 | A | A | G | 0.81 | 0.858368978 |  |
| 305 | 29 | A | A | T | 2.42 | 0.423384665 |  |
| 306 | 30 | C | C | A | 3.31 | 0.354226212 |  |
| 306 | 30 | C | C | C | 93.80 | 0.000000000 | \* |
| 306 | 30 | C | C | G | 0.83 | 0.854879430 |  |
| 306 | 30 | C | C | T | 2.07 | 0.511973652 |  |
| 307 | 31 | A | A | A | 90.86 | 0.000000000 | \* |
| 307 | 31 | A | A | C | 2.86 | 0.284445842 |  |
| 307 | 31 | A | A | G | 4.00 | 0.118952275 |  |
| 307 | 31 | A | A | T | 2.29 | 0.455966095 |  |
| 308 | 32 | C | C | A | 2.22 | 0.558943983 |  |
| 308 | 32 | C | C | C | 91.11 | 0.000000000 | \* |
| 308 | 32 | C | C | G | 1.78 | 0.600626852 |  |
| 308 | 32 | C | C | T | 4.89 | 0.077005948 |  |
| 309 | 33 | G | G | A | 5.65 | 0.098771265 |  |
| 309 | 33 | G | G | C | 3.95 | 0.108466705 |  |
| 309 | 33 | G | G | G | 85.31 | 0.000000000 | \* |
| 309 | 33 | G | G | T | 5.08 | 0.066019629 |  |
| 310 | 34 | G | G | A | 0.42 | 0.829814756 |  |
| 310 | 34 | G | G | C | 1.67 | 0.631659871 |  |
| 310 | 34 | G | G | G | 97.07 | 0.000000000 | \* |
| 310 | 34 | G | G | T | 0.84 | 0.816469484 |  |
| 311 | 35 | G | G | A | 3.94 | 0.258637594 |  |
| 311 | 35 | G | G | C | 1.48 | 0.695761763 |  |
| 311 | 35 | G | G | G | 92.61 | 0.000000000 | \* |
| 311 | 35 | G | G | T | 1.97 | 0.537133483 |  |
| 312 | 36 | C | C | A | 3.49 | 0.324582269 |  |
| 312 | 36 | C | C | C | 94.19 | 0.000000000 | \* |
| 312 | 36 | C | C | G | 1.16 | 0.780963364 |  |
| 312 | 36 | C | C | T | 1.16 | 0.748046168 |  |
| 313 | 37 | A | A | A | 90.10 | 0.000000000 | \* |
| 313 | 37 | A | A | C | 2.60 | 0.346064622 |  |
| 313 | 37 | A | A | G | 3.65 | 0.161461777 |  |
| 313 | 37 | A | A | T | 3.65 | 0.193793678 |  |
| 314 | 38 | C | C | A | 3.86 | 0.269679014 |  |
| 314 | 38 | C | C | C | 89.47 | 0.000000000 | \* |
| 314 | 38 | C | C | G | 1.75 | 0.607883578 |  |
| 314 | 38 | C | C | T | 4.91 | 0.075611123 |  |
| 315 | 39 | C | C | A | 5.86 | 0.086986992 |  |
| 315 | 39 | C | C | C | 91.02 | 0.000000000 | \* |
| 315 | 39 | C | C | G | 1.95 | 0.546459697 |  |
| 315 | 39 | C | C | T | 1.17 | 0.745902426 |  |
| 316 | 40 | C | C | A | 4.02 | 0.248728821 |  |
| 316 | 40 | C | C | C | 91.57 | 0.000000000 | \* |
| 316 | 40 | C | C | G | 0.00 | 0.911111111 |  |
| 316 | 40 | C | C | T | 4.42 | 0.110561878 |  |
| 317 | 41 | T | T | A | 0.81 | 0.801340636 |  |
| 317 | 41 | T | T | C | 3.25 | 0.204985312 |  |
| 317 | 41 | T | T | G | 2.03 | 0.522265424 |  |
| 317 | 41 | T | T | T | 93.90 | 0.000000000 | \* |
| 318 | 42 | G | G | A | 3.41 | 0.336617023 |  |
| 318 | 42 | G | G | C | 2.05 | 0.508821428 |  |
| 318 | 42 | G | G | G | 93.52 | 0.000000000 | \* |
| 318 | 42 | G | G | T | 1.02 | 0.779394743 |  |
| 319 | 43 | A | A | A | 95.34 | 0.000000000 | \* |
| 319 | 43 | A | A | C | 1.43 | 0.709861379 |  |
| 319 | 43 | A | A | G | 1.08 | 0.802695225 |  |
| 319 | 43 | A | A | T | 2.15 | 0.490120811 |  |
| 320 | 44 | C | C | A | 3.83 | 0.273825259 |  |
| 320 | 44 | C | C | C | 91.49 | 0.000000000 | \* |
| 320 | 44 | C | C | G | 3.83 | 0.137994551 |  |
| 320 | 44 | C | C | T | 0.85 | 0.813887699 |  |
| 321 | 45 | C | C | A | 4.78 | 0.163707811 |  |
| 321 | 45 | C | C | C | 89.47 | 0.000000000 | \* |
| 321 | 45 | C | C | G | 4.31 | 0.090425167 |  |
| 321 | 45 | C | C | T | 1.44 | 0.679998689 |  |
| 322 | 46 | G | G | A | 5.35 | 0.118262736 |  |
| 322 | 46 | G | G | C | 2.14 | 0.479964268 |  |
| 322 | 46 | G | G | G | 92.51 | 0.000000000 | \* |
| 322 | 46 | G | G | T | 0.00 | 0.882978723 |  |
| 323 | 47 | A | A | A | 95.85 | 0.000000000 | \* |
| 323 | 47 | A | A | C | 0.96 | 0.842652721 |  |
| 323 | 47 | A | A | G | 0.64 | 0.883040375 |  |
| 323 | 47 | A | A | T | 2.56 | 0.391469298 |  |
| 324 | 48 | C | C | A | 3.83 | 0.274109674 |  |
| 324 | 48 | C | C | C | 92.82 | 0.000000000 | \* |
| 324 | 48 | C | C | G | 0.48 | 0.898878117 |  |
| 324 | 48 | C | C | T | 2.87 | 0.323748699 |  |
| 325 | 49 | G | G | A | 9.56 | 0.007556966 | \* |
| 325 | 49 | G | G | C | 2.21 | 0.459280272 |  |
| 325 | 49 | G | G | G | 84.56 | 0.000000000 | \* |
| 325 | 49 | G | G | T | 3.68 | 0.189686110 |  |
| 326 | 50 | G | G | A | 3.63 | 0.302925295 |  |
| 326 | 50 | G | G | C | 0.81 | 0.873891164 |  |
| 326 | 50 | G | G | G | 95.16 | 0.000000000 | \* |
| 326 | 50 | G | G | T | 0.40 | 0.871813129 |  |
| 327 | 51 | C | C | A | 3.42 | 0.334732256 |  |
| 327 | 51 | C | C | C | 90.41 | 0.000000000 | \* |
| 327 | 51 | C | C | G | 2.05 | 0.515534917 |  |
| 327 | 51 | C | C | T | 4.11 | 0.139027828 |  |
| 328 | 52 | A | A | A | 92.04 | 0.000000000 | \* |
| 328 | 52 | A | A | C | 0.88 | 0.858611220 |  |
| 328 | 52 | A | A | G | 2.65 | 0.349488262 |  |
| 328 | 52 | A | A | T | 4.42 | 0.109971040 |  |
| 329 | 53 | A | A | A | 95.05 | 0.000000000 | \* |
| 329 | 53 | A | A | C | 0.00 | 0.928571429 |  |
| 329 | 53 | A | A | G | 0.99 | 0.822273995 |  |
| 329 | 53 | A | A | T | 3.96 | 0.154978216 |  |
| 330 | 54 | G | G | A | 1.23 | 0.746933444 |  |
| 330 | 54 | G | G | C | 3.07 | 0.239626979 |  |
| 330 | 54 | G | G | G | 95.09 | 0.000000000 | \* |
| 330 | 54 | G | G | T | 0.61 | 0.850900403 |  |
| 331 | 55 | A | A | A | 96.10 | 0.000000000 | \* |
| 331 | 55 | A | A | C | 0.00 | 0.928571429 |  |
| 331 | 55 | A | A | G | 0.87 | 0.847685944 |  |
| 331 | 55 | A | A | T | 3.03 | 0.292746683 |  |
| 332 | 56 | A | A | A | 95.59 | 0.000000000 | \* |
| 332 | 56 | A | A | C | 0.49 | 0.915490429 |  |
| 332 | 56 | A | A | G | 0.98 | 0.824398524 |  |
| 332 | 56 | A | A | T | 2.94 | 0.309793963 |  |
| 333 | 57 | G | G | A | 2.29 | 0.545204911 |  |
| 333 | 57 | G | G | C | 2.29 | 0.433847195 |  |
| 333 | 57 | G | G | G | 93.89 | 0.000000000 | \* |
| 333 | 57 | G | G | T | 1.53 | 0.655970757 |  |
| 334 | 58 | T | T | A | 2.59 | 0.485137437 |  |
| 334 | 58 | T | T | C | 2.07 | 0.500927725 |  |
| 334 | 58 | T | T | G | 0.00 | 0.911111111 |  |
| 334 | 58 | T | T | T | 95.34 | 0.000000000 | \* |
| 335 | 59 | T | T | A | 2.00 | 0.603934280 |  |
| 335 | 59 | T | T | C | 1.00 | 0.832973115 |  |
| 335 | 59 | T | T | G | 3.50 | 0.182377215 |  |
| 335 | 59 | T | T | T | 93.50 | 0.000000000 | \* |
| 336 | 60 | C | C | A | 1.57 | 0.687716271 |  |
| 336 | 60 | C | C | C | 94.24 | 0.000000000 | \* |
| 336 | 60 | C | C | G | 2.62 | 0.358698035 |  |
| 336 | 60 | C | C | T | 1.57 | 0.644273875 |  |
| 337 | 61 | G | G | A | 4.67 | 0.174928318 |  |
| 337 | 61 | G | G | C | 2.00 | 0.524178827 |  |
| 337 | 61 | G | G | G | 90.00 | 0.000000000 | \* |
| 337 | 61 | G | G | T | 3.33 | 0.240011774 |  |
| 338 | 62 | A | A | A | 94.30 | 0.000000000 | \* |
| 338 | 62 | A | A | C | 1.14 | 0.796984492 |  |
| 338 | 62 | A | A | G | 1.52 | 0.679642431 |  |
| 338 | 62 | A | A | T | 3.04 | 0.290594181 |  |
| 339 | 63 | C | C | A | 2.62 | 0.479370002 |  |
| 339 | 63 | C | C | C | 94.76 | 0.000000000 | \* |
| 339 | 63 | C | C | G | 1.31 | 0.741310845 |  |
| 339 | 63 | C | C | T | 1.31 | 0.712137389 |  |
| 340 | 64 | A | A | A | 93.80 | 0.000000000 | \* |
| 340 | 64 | A | A | C | 1.55 | 0.672245646 |  |
| 340 | 64 | A | A | G | 3.10 | 0.251147455 |  |
| 340 | 64 | A | A | T | 1.55 | 0.649681469 |  |
| 341 | 65 | G | G | A | 0.85 | 0.796916200 |  |
| 341 | 65 | G | G | C | 2.99 | 0.255160286 |  |
| 341 | 65 | G | G | G | 96.15 | 0.000000000 | \* |
| 341 | 65 | G | G | T | 0.00 | 0.882978723 |  |
| 342 | 66 | C | C | A | 1.57 | 0.687716271 |  |
| 342 | 66 | C | C | C | 91.62 | 0.000000000 | \* |
| 342 | 66 | C | C | G | 2.62 | 0.358698035 |  |
| 342 | 66 | C | C | T | 4.19 | 0.131185039 |  |
| 343 | 67 | T | T | A | 0.00 | 0.836363636 |  |
| 343 | 67 | T | T | C | 0.40 | 0.921490031 |  |
| 343 | 67 | T | T | G | 2.81 | 0.312362644 |  |
| 343 | 67 | T | T | T | 96.79 | 0.000000000 | \* |
| 344 | 68 | C | C | A | 2.86 | 0.433915255 |  |
| 344 | 68 | C | C | C | 90.95 | 0.000000000 | \* |
| 344 | 68 | C | C | G | 1.43 | 0.707196405 |  |
| 344 | 68 | C | C | T | 4.76 | 0.084995449 |  |
| 345 | 69 | C | C | A | 1.18 | 0.754712608 |  |
| 345 | 69 | C | C | C | 95.29 | 0.000000000 | \* |
| 345 | 69 | C | C | G | 0.00 | 0.911111111 |  |
| 345 | 69 | C | C | T | 3.53 | 0.210079068 |  |
| 346 | 70 | C | C | A | 2.38 | 0.526874464 |  |
| 346 | 70 | C | C | C | 94.05 | 0.000000000 | \* |
| 346 | 70 | C | C | G | 0.60 | 0.888111008 |  |
| 346 | 70 | C | C | T | 2.98 | 0.303012942 |  |
| 347 | 71 | G | G | A | 4.55 | 0.187115139 |  |
| 347 | 71 | G | G | C | 1.95 | 0.541038074 |  |
| 347 | 71 | G | G | G | 93.51 | 0.000000000 | \* |
| 347 | 71 | G | G | T | 0.00 | 0.882978723 |  |
| 348 | 72 | C | C | A | 0.00 | 0.836363636 |  |
| 348 | 72 | C | C | C | 96.07 | 0.000000000 | \* |
| 348 | 72 | C | C | G | 0.56 | 0.891602755 |  |
| 348 | 72 | C | C | T | 3.37 | 0.234045521 |  |
| 349 | 73 | G | G | A | 6.04 | 0.077856105 |  |
| 349 | 73 | G | G | C | 4.03 | 0.101281740 |  |
| 349 | 73 | G | G | G | 89.26 | 0.000000000 | \* |
| 349 | 73 | G | G | T | 0.67 | 0.843160365 |  |
| 350 | 74 | A | A | A | 96.77 | 0.000000000 | \* |
| 350 | 74 | A | A | C | 1.38 | 0.725971363 |  |
| 350 | 74 | A | A | G | 0.00 | 0.911111111 |  |
| 350 | 74 | A | A | T | 1.84 | 0.571040827 |  |
| 351 | 75 | C | C | A | 2.63 | 0.477123557 |  |
| 351 | 75 | C | C | C | 92.54 | 0.000000000 | \* |
| 351 | 75 | C | C | G | 1.32 | 0.739696792 |  |
| 351 | 75 | C | C | T | 3.51 | 0.213079987 |  |

## For use in R

If you want to work with the results in R, here is output that you can copy and paste in your terminal to get:

The base information:

```
structure(list(focal.base = c("A", "C", "G", "T"), avg.percsignal = c(93.2786098583282, 
91.6832544547693, 92.7468345845094, 93.8711026334972), avg.areasignal = c(232.735294117647, 
251.164179104478, 240.631578947368, 292.674418604651), crit.perc.area = c(9.15623084911376, 
6.23944585703671, 6.5371096763449, 7.33998375432346), mu = c(3.31412932216173, 
2.40981552663955, 2.49521245486343, 2.63948622826718), fillibens = c(0.99385839152866, 
0.984147761741744, 0.993444590648898, 0.988945290389615)), .Names = c("focal.base", 
"avg.percsignal", "avg.areasignal", "crit.perc.area", "mu", "fillibens"
), row.names = c(NA, -4L), class = "data.frame")
```

the data.frame that contains information on the guide region:

```
structure(list(A.area = c(205, 247, 5, 422, 21, 0, 20, 19, 9, 
6, 195, 8, 410, 18, 14, 14, 0, 11, 8, 0, 0, 6, 0, 9, 3, 168, 
12, 4, 229, 8, 159, 5, 10, 1, 8, 6, 173, 11, 15, 10, 2, 10, 266, 
9, 10, 10, 300, 8, 13, 9, 5, 104, 192, 2, 222, 195, 3, 5, 4, 
3, 7, 248, 6, 121, 2, 3, 0, 6, 3, 4, 7, 0, 9, 210, 6), C.area = c(7, 
5, 14, 4, 245, 7, 0, 5, 154, 270, 4, 5, 4, 253, 220, 8, 5, 5, 
0, 7, 2, 2, 3, 3, 201, 7, 233, 11, 11, 227, 5, 205, 7, 4, 3, 
162, 5, 255, 233, 228, 8, 6, 4, 215, 187, 4, 3, 194, 3, 2, 132, 
1, 0, 5, 0, 1, 3, 4, 2, 180, 3, 3, 217, 2, 7, 175, 1, 191, 243, 
158, 3, 171, 6, 3, 211), G.area = c(7, 3, 194, 9, 8, 7, 430, 
289, 10, 12, 8, 191, 4, 8, 8, 229, 5, 375, 15, 10, 7, 317, 6, 
253, 9, 9, 0, 5, 2, 2, 7, 4, 151, 232, 188, 2, 7, 5, 5, 0, 5, 
274, 3, 9, 9, 173, 2, 1, 115, 236, 3, 3, 2, 155, 2, 2, 123, 0, 
7, 5, 135, 4, 3, 4, 225, 5, 7, 3, 0, 1, 144, 1, 133, 0, 3), T.area = c(5, 
10, 3, 18, 9, 284, 5, 4, 4, 10, 0, 3, 18, 9, 2, 3, 189, 2, 220, 
249, 250, 9, 214, 7, 10, 0, 8, 251, 6, 5, 4, 11, 9, 2, 4, 2, 
7, 14, 3, 11, 231, 3, 6, 2, 3, 0, 8, 6, 5, 1, 6, 5, 8, 1, 7, 
6, 2, 184, 187, 3, 5, 8, 3, 2, 0, 8, 241, 10, 9, 5, 0, 6, 1, 
4, 8), Tot.area = c(224, 265, 216, 453, 283, 298, 455, 317, 177, 
298, 207, 207, 436, 288, 244, 254, 199, 393, 243, 266, 259, 334, 
223, 272, 223, 184, 253, 271, 248, 242, 175, 225, 177, 239, 203, 
172, 192, 285, 256, 249, 246, 293, 279, 235, 209, 187, 313, 209, 
136, 248, 146, 113, 202, 163, 231, 204, 131, 193, 200, 191, 150, 
263, 229, 129, 234, 191, 249, 210, 255, 168, 154, 178, 149, 217, 
228), A.perc = c(91.5178571428571, 93.2075471698113, 2.31481481481481, 
93.1567328918322, 7.42049469964664, 0, 4.3956043956044, 5.99369085173502, 
5.08474576271186, 2.01342281879195, 94.2028985507246, 3.86473429951691, 
94.0366972477064, 6.25, 5.73770491803279, 5.51181102362205, 0, 
2.79898218829517, 3.29218106995885, 0, 0, 1.79640718562874, 0, 
3.30882352941176, 1.34529147982063, 91.304347826087, 4.74308300395257, 
1.4760147601476, 92.3387096774194, 3.30578512396694, 90.8571428571429, 
2.22222222222222, 5.64971751412429, 0.418410041841004, 3.94088669950739, 
3.48837209302326, 90.1041666666667, 3.85964912280702, 5.859375, 
4.01606425702811, 0.813008130081301, 3.41296928327645, 95.3405017921147, 
3.82978723404255, 4.78468899521531, 5.3475935828877, 95.8466453674121, 
3.82775119617225, 9.55882352941176, 3.62903225806452, 3.42465753424658, 
92.0353982300885, 95.049504950495, 1.22699386503067, 96.1038961038961, 
95.5882352941177, 2.29007633587786, 2.59067357512953, 2, 1.57068062827225, 
4.66666666666667, 94.2965779467681, 2.62008733624454, 93.7984496124031, 
0.854700854700855, 1.57068062827225, 0, 2.85714285714286, 1.17647058823529, 
2.38095238095238, 4.54545454545455, 0, 6.04026845637584, 96.7741935483871, 
2.63157894736842), C.perc = c(3.125, 1.88679245283019, 6.48148148148148, 
0.883002207505519, 86.5724381625442, 2.3489932885906, 0, 1.57728706624606, 
87.0056497175141, 90.6040268456376, 1.93236714975845, 2.41545893719807, 
0.917431192660551, 87.8472222222222, 90.1639344262295, 3.1496062992126, 
2.51256281407035, 1.27226463104326, 0, 2.63157894736842, 0.772200772200772, 
0.598802395209581, 1.34529147982063, 1.10294117647059, 90.1345291479821, 
3.80434782608696, 92.094861660079, 4.0590405904059, 4.43548387096774, 
93.801652892562, 2.85714285714286, 91.1111111111111, 3.95480225988701, 
1.67364016736402, 1.47783251231527, 94.1860465116279, 2.60416666666667, 
89.4736842105263, 91.015625, 91.566265060241, 3.2520325203252, 
2.04778156996587, 1.4336917562724, 91.4893617021277, 89.4736842105263, 
2.13903743315508, 0.958466453674121, 92.822966507177, 2.20588235294118, 
0.806451612903226, 90.4109589041096, 0.884955752212389, 0, 3.06748466257669, 
0, 0.490196078431373, 2.29007633587786, 2.07253886010363, 1, 
94.2408376963351, 2, 1.14068441064639, 94.7598253275109, 1.55038759689922, 
2.99145299145299, 91.6230366492147, 0.401606425702811, 90.9523809523809, 
95.2941176470588, 94.0476190476191, 1.94805194805195, 96.0674157303371, 
4.02684563758389, 1.38248847926267, 92.5438596491228), G.perc = c(3.125, 
1.13207547169811, 89.8148148148148, 1.98675496688742, 2.82685512367491, 
2.3489932885906, 94.5054945054945, 91.1671924290221, 5.64971751412429, 
4.02684563758389, 3.86473429951691, 92.2705314009662, 0.917431192660551, 
2.77777777777778, 3.27868852459016, 90.1574803149606, 2.51256281407035, 
95.4198473282443, 6.17283950617284, 3.7593984962406, 2.7027027027027, 
94.9101796407186, 2.69058295964126, 93.0147058823529, 4.03587443946188, 
4.89130434782609, 0, 1.8450184501845, 0.806451612903226, 0.826446280991736, 
4, 1.77777777777778, 85.3107344632768, 97.071129707113, 92.6108374384236, 
1.16279069767442, 3.64583333333333, 1.75438596491228, 1.953125, 
0, 2.03252032520325, 93.5153583617747, 1.0752688172043, 3.82978723404255, 
4.30622009569378, 92.5133689839572, 0.638977635782748, 0.478468899521531, 
84.5588235294118, 95.1612903225806, 2.05479452054795, 2.65486725663717, 
0.99009900990099, 95.0920245398773, 0.865800865800866, 0.980392156862745, 
93.8931297709924, 0, 3.5, 2.61780104712042, 90, 1.52091254752852, 
1.31004366812227, 3.10077519379845, 96.1538461538462, 2.61780104712042, 
2.81124497991968, 1.42857142857143, 0, 0.595238095238095, 93.5064935064935, 
0.561797752808989, 89.2617449664429, 0, 1.31578947368421), T.perc = c(2.23214285714286, 
3.77358490566038, 1.38888888888889, 3.97350993377483, 3.18021201413428, 
95.3020134228188, 1.0989010989011, 1.26182965299685, 2.25988700564972, 
3.35570469798658, 0, 1.44927536231884, 4.12844036697248, 3.125, 
0.819672131147541, 1.18110236220472, 94.9748743718593, 0.508905852417303, 
90.5349794238683, 93.609022556391, 96.5250965250965, 2.69461077844311, 
95.9641255605381, 2.57352941176471, 4.48430493273543, 0, 3.16205533596838, 
92.619926199262, 2.41935483870968, 2.06611570247934, 2.28571428571429, 
4.88888888888889, 5.08474576271186, 0.836820083682008, 1.97044334975369, 
1.16279069767442, 3.64583333333333, 4.91228070175439, 1.171875, 
4.41767068273092, 93.9024390243902, 1.02389078498294, 2.1505376344086, 
0.851063829787234, 1.43540669856459, 0, 2.55591054313099, 2.87081339712919, 
3.67647058823529, 0.403225806451613, 4.10958904109589, 4.42477876106195, 
3.96039603960396, 0.613496932515337, 3.03030303030303, 2.94117647058824, 
1.52671755725191, 95.3367875647668, 93.5, 1.57068062827225, 3.33333333333333, 
3.04182509505703, 1.31004366812227, 1.55038759689922, 0, 4.18848167539267, 
96.7871485943775, 4.76190476190476, 3.52941176470588, 2.97619047619048, 
0, 3.37078651685393, 0.671140939597315, 1.84331797235023, 3.50877192982456
), base.call = c("A", "A", "G", "A", "C", "T", "G", "G", "C", 
"C", "A", "G", "A", "C", "C", "G", "T", "G", "T", "T", "T", "G", 
"T", "G", "C", "A", "C", "T", "A", "C", "A", "C", "G", "G", "G", 
"C", "A", "C", "C", "C", "T", "G", "A", "C", "C", "G", "A", "C", 
"G", "G", "C", "A", "A", "G", "A", "A", "G", "T", "T", "C", "G", 
"A", "C", "A", "G", "C", "T", "C", "C", "C", "G", "C", "G", "A", 
"C"), index = 277:351, guide.seq = c("A", "A", "G", "A", "C", 
"T", "G", "G", "C", "C", "A", "G", "A", "C", "C", "G", "T", "G", 
"T", "T", "T", "G", "T", "G", "C", "A", "C", "T", "A", "C", "A", 
"C", "G", "G", "G", "C", "A", "C", "C", "C", "T", "G", "A", "C", 
"C", "G", "A", "C", "G", "G", "C", "A", "A", "G", "A", "A", "G", 
"T", "T", "C", "G", "A", "C", "A", "G", "C", "T", "C", "C", "C", 
"G", "C", "G", "A", "C"), T.pval = c(0.469367919097455, 0.17714307596351, 
0.692058513142332, 0.153515605144814, 0.265656426350895, 0, 0.762814331889312, 
0.72414840267699, 0.462404223053847, 0.236433718923106, 0.88297872340416, 
0.676377120511359, 0.137117530482684, 0.275403706459853, 0.819520376796607, 
0.743714349527406, 0, 0.862760261296224, 0, 0, 0, 0.36059766066555, 
0, 0.387459330835059, 0.105132513157093, 0.88297872340416, 0.268832358488409, 
0, 0.423384665397299, 0.511973652013435, 0.455966095031372, 0.0770059475853042, 
0.0660196294116674, 0.816469483547123, 0.537133483468168, 0.748046168126022, 
0.193793677860088, 0.0756111226387955, 0.745902426338212, 0.110561877842729, 
0, 0.779394743258122, 0.490120811083429, 0.81388769917981, 0.67999868926974, 
0.88297872340416, 0.391469297714947, 0.323748699487653, 0.189686110332286, 
0.871813129381829, 0.139027828010078, 0.109971040082759, 0.154978215521896, 
0.850900403433068, 0.292746683493754, 0.309793963476181, 0.655970756853677, 
0, 0, 0.644273875309595, 0.240011773785945, 0.290594181363172, 
0.712137388689985, 0.649681468583325, 0.88297872340416, 0.131185039407337, 
0, 0.0849954493818809, 0.210079067799219, 0.303012942434784, 
0.88297872340416, 0.234045520563064, 0.843160365327043, 0.571040827154567, 
0.213079987355005), C.pval = c(0.228370690081398, 0.561098775367359, 
0.00761736919579936, 0.859014023689555, 0, 0.416495899943031, 
0.928571428571429, 0.663442061269089, 0, 0, 0.546157620628374, 
0.397392027179537, 0.851751458283381, 0, 0, 0.223683431473649, 
0.370427053669028, 0.759527957829768, 0.928571428571429, 0.338982791049832, 
0.879959450654766, 0.904861482538614, 0.737482214499809, 0.80709212105563, 
0, 0.124929138451373, 0, 0.0982092739713861, 0.0679818468151613, 
0, 0.284445842195243, 0, 0.108466704919493, 0.631659870518845, 
0.695761763400108, 0, 0.346064621582097, 0, 0, 0, 0.204985312279595, 
0.508821428496218, 0.709861378621188, 0, 0, 0.479964268013048, 
0.842652720735582, 0, 0.459280271736778, 0.873891164470056, 0, 
0.858611219779485, 0.928571428571429, 0.239626978758187, 0.928571428571429, 
0.915490429225087, 0.433847194542493, 0.500927725259298, 0.832973115272538, 
0, 0.524178827051943, 0.796984492269473, 0, 0.672245645501535, 
0.255160286489896, 0, 0.921490031153875, 0, 0, 0, 0.541038074110539, 
0, 0.101281739656137, 0.725971363282755, 0), G.pval = c(0.246467593827046, 
0.788763029282392, 0, 0.536175823343401, 0.308812225654613, 0.429925181711351, 
0, 0, 0.0248873215885916, 0.116168032175814, 0.133883267191576, 
0, 0.837613162215701, 0.320070275901948, 0.218332100610157, 0, 
0.385676736361828, 0, 0.0146084231589531, 0.146605220178797, 
0.337832854715753, 0, 0.340761207521004, 0, 0.115244528677618, 
0.0523732695481807, 0.91111111108653, 0.579782638522224, 0.858368978280519, 
0.854879429508339, 0.118952274535882, 0.600626851916512, 0, 0, 
0, 0.780963363828599, 0.161461776695687, 0.607883577588251, 0.546459696514468, 
0.91111111108653, 0.522265423781187, 0, 0.802695224539106, 0.137994550768248, 
0.090425167497075, 0, 0.88304037493241, 0.898878116843694, 0, 
0, 0.515534916978565, 0.349488262050553, 0.822273995065059, 0, 
0.847685944140852, 0.824398524205168, 0, 0.91111111108653, 0.182377214510164, 
0.358698034772011, 0, 0.679642430703253, 0.741310845036316, 0.251147454800601, 
0, 0.358698034772011, 0.312362643590933, 0.707196405399808, 0.91111111108653, 
0.888111007969416, 0, 0.891602755236779, 0, 0.91111111108653, 
0.739696792288723), A.pval = c(0, 0, 0.540205188842035, 0, 0.0322991072309833, 
0.836363636363636, 0.203142745539137, 0.0801200725912106, 0.137899198237405, 
0.601227914168149, 0, 0.268977647381802, 0, 0.0683663145649089, 
0.0936614946694438, 0.107282525070885, 0.836363636363636, 0.444882477178526, 
0.356502744006089, 0.836363636363636, 0.836363636363636, 0.644512981603701, 
0.836363636363636, 0.353719025837371, 0.727694745822861, 0, 0.167591995038181, 
0.704973347649039, 0, 0.354226212439328, 0, 0.558943982649582, 
0.0987712654818622, 0.829814756028491, 0.258637594340835, 0.324582269048993, 
0, 0.269679013930027, 0.0869869923779996, 0.248728821050433, 
0.801340635558239, 0.336617023329193, 0, 0.273825258554809, 0.163707810601686, 
0.118262736151286, 0, 0.274109674455457, 0.00755696643787274, 
0.302925294508548, 0.334732256262806, 0, 0, 0.746933443914673, 
0, 0, 0.545204910803498, 0.485137436518192, 0.603934280337201, 
0.687716270940829, 0.174928318157473, 0, 0.47937000171156, 0, 
0.796916199796981, 0.687716270940829, 0.836363636363636, 0.43391525536843, 
0.754712608116038, 0.526874463596999, 0.187115138693634, 0.836363636363636, 
0.0778561053317826, 0, 0.477123557486993), guide.position = 1:75), .Names = c("A.area", 
"C.area", "G.area", "T.area", "Tot.area", "A.perc", "C.perc", 
"G.perc", "T.perc", "base.call", "index", "guide.seq", "T.pval", 
"C.pval", "G.pval", "A.pval", "guide.position"), row.names = 277:351, class = "data.frame")
```

*Report generated using EditR v1.0.8*
