# Supplementary material for: Mechanistic and genetic basis of single-strand templated repair at Cas12a-induced DNA breaks in Chlamydomonas reinhardtii
Source: Nat Commun. 2021 Nov 19;12:6751. doi: 10.1038/s41467-021-27004-1 (PMC8604939; doi:10.1038/s41467-021-27004-1)
Supplement: Supplementary file 22 — Source Data [file 41467_2021_27004_MOESM22_ESM.zip › Source Data/EditR analysis/EditR outputs/Antisense/rep2_ssODN_antisense_-16.html]

EditR v1.0.8 report


# EditR v1.0.8 report

- Data QA
  - Filtering data
  - Percent noise peak area
  - Base information
- Predicted editing
  - Editing bar plot
  - Editing table plot
  - Table of editing results
- For use in R

## Data QA

### Filtering data

What the data looked like prefiltering:

and the post filtering signal / noise plot:

### Percent noise peak area

### Base information

Here’s information about the signal of each base, the critical percent value where any higher value would be called as significant, and Filliben’s correlation for how well the noise was modelled by the zero adjusted gamma distribution.

| Base | Average percent signal | Average peak area | Critical percent value | model mu | Fillibens correlation |
| --- | --- | --- | --- | --- | --- |
| A | 92.94001 | 286.0000 | 9.905248 | 3.636829 | 0.9889775 |
| C | 92.67891 | 316.7164 | 6.273910 | 2.378613 | 0.9953839 |
| G | 93.09961 | 284.6667 | 5.514116 | 2.069393 | 0.9941622 |
| T | 93.56787 | 338.8605 | 6.007860 | 2.193058 | 0.9925825 |

## Predicted editing

### Editing bar plot

### Editing table plot

### Table of editing results


Here’s the entire guide region

| Sanger position | Guide position | Guide sequence | Sanger base call | Focal base | Focal base peak area | p value |  |
| --- | --- | --- | --- | --- | --- | --- | --- |
| 277 | 1 | A | A | A | 94.33 | 0.0000000000 | \* |
| 277 | 1 | A | A | C | 1.77 | 0.5929845296 |  |
| 277 | 1 | A | A | G | 1.42 | 0.6317319943 |  |
| 277 | 1 | A | A | T | 2.48 | 0.3042911585 |  |
| 278 | 2 | A | A | A | 93.39 | 0.0000000000 | \* |
| 278 | 2 | A | A | C | 1.50 | 0.6836893167 |  |
| 278 | 2 | A | A | G | 2.10 | 0.3881819146 |  |
| 278 | 2 | A | A | T | 3.00 | 0.1982975087 |  |
| 279 | 3 | G | G | A | 2.81 | 0.4945643129 |  |
| 279 | 3 | G | G | C | 3.21 | 0.2096652456 |  |
| 279 | 3 | G | G | G | 93.98 | 0.0000000000 | \* |
| 279 | 3 | G | G | T | 0.00 | 0.8936170213 |  |
| 280 | 4 | A | A | A | 93.45 | 0.0000000000 | \* |
| 280 | 4 | A | A | C | 1.27 | 0.7569888630 |  |
| 280 | 4 | A | A | G | 1.64 | 0.5496663645 |  |
| 280 | 4 | A | A | T | 3.64 | 0.1123365034 |  |
| 281 | 5 | C | C | A | 5.19 | 0.1671362702 |  |
| 281 | 5 | C | C | C | 92.51 | 0.0000000000 | \* |
| 281 | 5 | C | C | G | 0.00 | 0.9333333333 |  |
| 281 | 5 | C | C | T | 2.31 | 0.3484380722 |  |
| 282 | 6 | T | T | A | 4.81 | 0.2027274794 |  |
| 282 | 6 | T | T | C | 1.60 | 0.6495123634 |  |
| 282 | 6 | T | T | G | 2.94 | 0.1820773321 |  |
| 282 | 6 | T | T | T | 90.64 | 0.0000000000 | \* |
| 283 | 7 | G | G | A | 3.11 | 0.4404626186 |  |
| 283 | 7 | G | G | C | 0.92 | 0.8546071603 |  |
| 283 | 7 | G | G | G | 94.87 | 0.0000000000 | \* |
| 283 | 7 | G | G | T | 1.10 | 0.7242420788 |  |
| 284 | 8 | G | G | A | 4.24 | 0.2690398933 |  |
| 284 | 8 | G | G | C | 1.41 | 0.7128263787 |  |
| 284 | 8 | G | G | G | 93.50 | 0.0000000000 | \* |
| 284 | 8 | G | G | T | 0.85 | 0.7956290313 |  |
| 285 | 9 | C | C | A | 6.41 | 0.0854815922 |  |
| 285 | 9 | C | C | C | 87.61 | 0.0000000000 | \* |
| 285 | 9 | C | C | G | 4.27 | 0.0438152946 |  |
| 285 | 9 | C | C | T | 1.71 | 0.5250498355 |  |
| 286 | 10 | C | C | A | 2.93 | 0.4724259206 |  |
| 286 | 10 | C | C | C | 92.27 | 0.0000000000 | \* |
| 286 | 10 | C | C | G | 2.67 | 0.2368153549 |  |
| 286 | 10 | C | C | T | 2.13 | 0.3953461225 |  |
| 287 | 11 | A | A | A | 91.76 | 0.0000000000 | \* |
| 287 | 11 | A | A | C | 2.35 | 0.4099798427 |  |
| 287 | 11 | A | A | G | 2.75 | 0.2199768556 |  |
| 287 | 11 | A | A | T | 3.14 | 0.1764825721 |  |
| 288 | 12 | G | G | A | 3.53 | 0.3708926145 |  |
| 288 | 12 | G | G | C | 2.35 | 0.4099798427 |  |
| 288 | 12 | G | G | G | 94.12 | 0.0000000000 | \* |
| 288 | 12 | G | G | T | 0.00 | 0.8936170213 |  |
| 289 | 13 | A | A | A | 94.26 | 0.0000000000 | \* |
| 289 | 13 | A | A | C | 1.15 | 0.7943085477 |  |
| 289 | 13 | A | A | G | 0.76 | 0.8525540461 |  |
| 289 | 13 | A | A | T | 3.82 | 0.0941532678 |  |
| 290 | 14 | C | C | A | 5.00 | 0.1842310143 |  |
| 290 | 14 | C | C | C | 94.12 | 0.0000000000 | \* |
| 290 | 14 | C | C | G | 0.29 | 0.9274482170 |  |
| 290 | 14 | C | C | T | 0.59 | 0.8519209449 |  |
| 291 | 15 | C | C | A | 6.98 | 0.0613208519 |  |
| 291 | 15 | C | C | C | 89.84 | 0.0000000000 | \* |
| 291 | 15 | C | C | G | 2.86 | 0.1976041326 |  |
| 291 | 15 | C | C | T | 0.32 | 0.8852224014 |  |
| 292 | 16 | G | G | A | 4.71 | 0.2131068345 |  |
| 292 | 16 | G | G | C | 1.68 | 0.6229753555 |  |
| 292 | 16 | G | G | G | 92.26 | 0.0000000000 | \* |
| 292 | 16 | G | G | T | 1.35 | 0.6448018448 |  |
| 293 | 17 | T | T | A | 2.59 | 0.5361106887 |  |
| 293 | 17 | T | T | C | 2.59 | 0.3461464244 |  |
| 293 | 17 | T | T | G | 3.02 | 0.1689113503 |  |
| 293 | 17 | T | T | T | 91.81 | 0.0000000000 | \* |
| 294 | 18 | G | G | A | 3.67 | 0.3486512806 |  |
| 294 | 18 | G | G | C | 1.30 | 0.7498293839 |  |
| 294 | 18 | G | G | G | 93.95 | 0.0000000000 | \* |
| 294 | 18 | G | G | T | 1.08 | 0.7300438705 |  |
| 295 | 19 | T | T | A | 1.76 | 0.6868014535 |  |
| 295 | 19 | T | T | C | 2.11 | 0.4823587982 |  |
| 295 | 19 | T | T | G | 4.58 | 0.0308389547 |  |
| 295 | 19 | T | T | T | 91.55 | 0.0000000000 | \* |
| 296 | 20 | T | T | A | 0.32 | 0.8341981680 |  |
| 296 | 20 | T | T | C | 1.95 | 0.5350858017 |  |
| 296 | 20 | T | T | G | 3.57 | 0.0953243770 |  |
| 296 | 20 | T | T | T | 94.16 | 0.0000000000 | \* |
| 297 | 21 | T | T | A | 0.00 | 0.8363636364 |  |
| 297 | 21 | T | T | C | 2.88 | 0.2747636360 |  |
| 297 | 21 | T | T | G | 2.24 | 0.3452421254 |  |
| 297 | 21 | T | T | T | 94.87 | 0.0000000000 | \* |
| 298 | 22 | G | G | A | 2.49 | 0.5545076940 |  |
| 298 | 22 | G | G | C | 1.74 | 0.6036077621 |  |
| 298 | 22 | G | G | G | 95.02 | 0.0000000000 | \* |
| 298 | 22 | G | G | T | 0.75 | 0.8201084568 |  |
| 299 | 23 | T | T | A | 0.00 | 0.8363636364 |  |
| 299 | 23 | T | T | C | 0.82 | 0.8748093954 |  |
| 299 | 23 | T | T | G | 1.65 | 0.5460536909 |  |
| 299 | 23 | T | T | T | 97.53 | 0.0000000000 | \* |
| 300 | 24 | G | G | A | 2.74 | 0.5084469632 |  |
| 300 | 24 | G | G | C | 3.04 | 0.2423292845 |  |
| 300 | 24 | G | G | G | 93.01 | 0.0000000000 | \* |
| 300 | 24 | G | G | T | 1.22 | 0.6874869065 |  |
| 301 | 25 | C | C | A | 0.00 | 0.8363636364 |  |
| 301 | 25 | C | C | C | 96.39 | 0.0000000000 | \* |
| 301 | 25 | C | C | G | 1.44 | 0.6220394628 |  |
| 301 | 25 | C | C | T | 2.17 | 0.3861390750 |  |
| 302 | 26 | A | A | A | 88.79 | 0.0000000000 | \* |
| 302 | 26 | A | A | C | 3.88 | 0.1159102654 |  |
| 302 | 26 | A | A | G | 5.17 | 0.0151857542 |  |
| 302 | 26 | A | A | T | 2.16 | 0.3891883906 |  |
| 303 | 27 | C | C | A | 2.88 | 0.4812182221 |  |
| 303 | 27 | C | C | C | 88.14 | 0.0000000000 | \* |
| 303 | 27 | C | C | G | 7.37 | 0.0009286876 | \* |
| 303 | 27 | C | C | T | 1.60 | 0.5600240102 |  |
| 304 | 28 | T | T | A | 1.65 | 0.7052481791 |  |
| 304 | 28 | T | T | C | 1.98 | 0.5246288290 |  |
| 304 | 28 | T | T | G | 0.00 | 0.9333333333 |  |
| 304 | 28 | T | T | T | 96.37 | 0.0000000000 | \* |
| 305 | 29 | A | A | A | 94.41 | 0.0000000000 | \* |
| 305 | 29 | A | A | C | 1.75 | 0.6012784021 |  |
| 305 | 29 | A | A | G | 2.45 | 0.2892095885 |  |
| 305 | 29 | A | A | T | 1.40 | 0.6276734385 |  |
| 306 | 30 | C | C | A | 1.93 | 0.6573991639 |  |
| 306 | 30 | C | C | C | 95.50 | 0.0000000000 | \* |
| 306 | 30 | C | C | G | 1.93 | 0.4448741983 |  |
| 306 | 30 | C | C | T | 0.64 | 0.8418408898 |  |
| 307 | 31 | A | A | A | 92.69 | 0.0000000000 | \* |
| 307 | 31 | A | A | C | 2.74 | 0.3079533808 |  |
| 307 | 31 | A | A | G | 3.65 | 0.0873349744 |  |
| 307 | 31 | A | A | T | 0.91 | 0.7782484877 |  |
| 308 | 32 | C | C | A | 1.39 | 0.7448740564 |  |
| 308 | 32 | C | C | C | 94.43 | 0.0000000000 | \* |
| 308 | 32 | C | C | G | 2.09 | 0.3918166453 |  |
| 308 | 32 | C | C | T | 2.09 | 0.4075646714 |  |
| 309 | 33 | G | G | A | 4.41 | 0.2473889015 |  |
| 309 | 33 | G | G | C | 1.96 | 0.5309357956 |  |
| 309 | 33 | G | G | G | 93.63 | 0.0000000000 | \* |
| 309 | 33 | G | G | T | 0.00 | 0.8936170213 |  |
| 310 | 34 | G | G | A | 2.35 | 0.5803972429 |  |
| 310 | 34 | G | G | C | 2.01 | 0.5138967837 |  |
| 310 | 34 | G | G | G | 95.30 | 0.0000000000 | \* |
| 310 | 34 | G | G | T | 0.34 | 0.8838608890 |  |
| 311 | 35 | G | G | A | 2.92 | 0.4754271909 |  |
| 311 | 35 | G | G | C | 1.25 | 0.7639372223 |  |
| 311 | 35 | G | G | G | 95.83 | 0.0000000000 | \* |
| 311 | 35 | G | G | T | 0.00 | 0.8936170213 |  |
| 312 | 36 | C | C | A | 3.20 | 0.4261132865 |  |
| 312 | 36 | C | C | C | 93.61 | 0.0000000000 | \* |
| 312 | 36 | C | C | G | 0.91 | 0.8105903693 |  |
| 312 | 36 | C | C | T | 2.28 | 0.3543191473 |  |
| 313 | 37 | A | A | A | 89.71 | 0.0000000000 | \* |
| 313 | 37 | A | A | C | 3.29 | 0.1959483715 |  |
| 313 | 37 | A | A | G | 2.47 | 0.2836917973 |  |
| 313 | 37 | A | A | T | 4.53 | 0.0473333896 |  |
| 314 | 38 | C | C | A | 2.25 | 0.5993635490 |  |
| 314 | 38 | C | C | C | 96.07 | 0.0000000000 | \* |
| 314 | 38 | C | C | G | 0.56 | 0.8965858357 |  |
| 314 | 38 | C | C | T | 1.12 | 0.7166189392 |  |
| 315 | 39 | C | C | A | 4.39 | 0.2501707783 |  |
| 315 | 39 | C | C | C | 92.16 | 0.0000000000 | \* |
| 315 | 39 | C | C | G | 2.19 | 0.3598129051 |  |
| 315 | 39 | C | C | T | 1.25 | 0.6751936558 |  |
| 316 | 40 | C | C | A | 4.05 | 0.2938325154 |  |
| 316 | 40 | C | C | C | 91.90 | 0.0000000000 | \* |
| 316 | 40 | C | C | G | 0.93 | 0.8039933268 |  |
| 316 | 40 | C | C | T | 3.12 | 0.1799119663 |  |
| 317 | 41 | T | T | A | 0.67 | 0.8210484179 |  |
| 317 | 41 | T | T | C | 1.33 | 0.7381110264 |  |
| 317 | 41 | T | T | G | 3.33 | 0.1225092781 |  |
| 317 | 41 | T | T | T | 94.67 | 0.0000000000 | \* |
| 318 | 42 | G | G | A | 3.92 | 0.3117146270 |  |
| 318 | 42 | G | G | C | 1.96 | 0.5309357956 |  |
| 318 | 42 | G | G | G | 93.00 | 0.0000000000 | \* |
| 318 | 42 | G | G | T | 1.12 | 0.7175951855 |  |
| 319 | 43 | A | A | A | 93.45 | 0.0000000000 | \* |
| 319 | 43 | A | A | C | 1.42 | 0.7089133817 |  |
| 319 | 43 | A | A | G | 1.14 | 0.7350657280 |  |
| 319 | 43 | A | A | T | 3.99 | 0.0804413587 |  |
| 320 | 44 | C | C | A | 2.33 | 0.5847663987 |  |
| 320 | 44 | C | C | C | 96.01 | 0.0000000000 | \* |
| 320 | 44 | C | C | G | 0.00 | 0.9333333333 |  |
| 320 | 44 | C | C | T | 1.66 | 0.5407804104 |  |
| 321 | 45 | C | C | A | 5.13 | 0.1723818454 |  |
| 321 | 45 | C | C | C | 89.01 | 0.0000000000 | \* |
| 321 | 45 | C | C | G | 3.30 | 0.1272472535 |  |
| 321 | 45 | C | C | T | 2.56 | 0.2852733960 |  |
| 322 | 46 | G | G | A | 6.61 | 0.0763250134 |  |
| 322 | 46 | G | G | C | 2.64 | 0.3316128533 |  |
| 322 | 46 | G | G | G | 90.31 | 0.0000000000 | \* |
| 322 | 46 | G | G | T | 0.44 | 0.8735466136 |  |
| 323 | 47 | A | A | A | 95.12 | 0.0000000000 | \* |
| 323 | 47 | A | A | C | 1.03 | 0.8268887441 |  |
| 323 | 47 | A | A | G | 0.77 | 0.8509024986 |  |
| 323 | 47 | A | A | T | 3.08 | 0.1847497330 |  |
| 324 | 48 | C | C | A | 3.75 | 0.3374844261 |  |
| 324 | 48 | C | C | C | 94.76 | 0.0000000000 | \* |
| 324 | 48 | C | C | G | 0.75 | 0.8565614650 |  |
| 324 | 48 | C | C | T | 0.75 | 0.8194723777 |  |
| 325 | 49 | G | G | A | 8.54 | 0.0239081902 |  |
| 325 | 49 | G | G | C | 2.44 | 0.3856337720 |  |
| 325 | 49 | G | G | G | 87.80 | 0.0000000000 | \* |
| 325 | 49 | G | G | T | 1.22 | 0.6862969309 |  |
| 326 | 50 | G | G | A | 2.34 | 0.5818637293 |  |
| 326 | 50 | G | G | C | 1.34 | 0.7367036356 |  |
| 326 | 50 | G | G | G | 95.32 | 0.0000000000 | \* |
| 326 | 50 | G | G | T | 1.00 | 0.7528473608 |  |
| 327 | 51 | C | C | A | 1.72 | 0.6929659566 |  |
| 327 | 51 | C | C | C | 94.25 | 0.0000000000 | \* |
| 327 | 51 | C | C | G | 4.02 | 0.0581582645 |  |
| 327 | 51 | C | C | T | 0.00 | 0.8936170213 |  |
| 328 | 52 | A | A | A | 89.66 | 0.0000000000 | \* |
| 328 | 52 | A | A | C | 1.38 | 0.7234924441 |  |
| 328 | 52 | A | A | G | 3.45 | 0.1086316265 |  |
| 328 | 52 | A | A | T | 5.52 | 0.0169543964 |  |
| 329 | 53 | A | A | A | 94.25 | 0.0000000000 | \* |
| 329 | 53 | A | A | C | 1.92 | 0.5456732237 |  |
| 329 | 53 | A | A | G | 1.53 | 0.5885646730 |  |
| 329 | 53 | A | A | T | 2.30 | 0.3501729108 |  |
| 330 | 54 | G | G | A | 0.99 | 0.7953866341 |  |
| 330 | 54 | G | G | C | 2.46 | 0.3789995880 |  |
| 330 | 54 | G | G | G | 95.57 | 0.0000000000 | \* |
| 330 | 54 | G | G | T | 0.99 | 0.7580905294 |  |
| 331 | 55 | A | A | A | 95.26 | 0.0000000000 | \* |
| 331 | 55 | A | A | C | 0.00 | 0.9404761905 |  |
| 331 | 55 | A | A | G | 1.46 | 0.6160532779 |  |
| 331 | 55 | A | A | T | 3.28 | 0.1548872989 |  |
| 332 | 56 | A | A | A | 94.72 | 0.0000000000 | \* |
| 332 | 56 | A | A | C | 0.41 | 0.9313223410 |  |
| 332 | 56 | A | A | G | 1.22 | 0.7061643556 |  |
| 332 | 56 | A | A | T | 3.66 | 0.1100372861 |  |
| 333 | 57 | G | G | A | 1.78 | 0.6843131225 |  |
| 333 | 57 | G | G | C | 2.96 | 0.2588868677 |  |
| 333 | 57 | G | G | G | 92.90 | 0.0000000000 | \* |
| 333 | 57 | G | G | T | 2.37 | 0.3326374317 |  |
| 334 | 58 | T | T | A | 0.90 | 0.8034439477 |  |
| 334 | 58 | T | T | C | 3.60 | 0.1490682852 |  |
| 334 | 58 | T | T | G | 0.00 | 0.9333333333 |  |
| 334 | 58 | T | T | T | 95.50 | 0.0000000000 | \* |
| 335 | 59 | T | T | A | 1.23 | 0.7666460806 |  |
| 335 | 59 | T | T | C | 1.65 | 0.6355158291 |  |
| 335 | 59 | T | T | G | 4.12 | 0.0524363404 |  |
| 335 | 59 | T | T | T | 93.00 | 0.0000000000 | \* |
| 336 | 60 | C | C | A | 3.92 | 0.3117146270 |  |
| 336 | 60 | C | C | C | 91.76 | 0.0000000000 | \* |
| 336 | 60 | C | C | G | 3.14 | 0.1497749275 |  |
| 336 | 60 | C | C | T | 1.18 | 0.7000309881 |  |
| 337 | 61 | G | G | A | 9.28 | 0.0149683855 |  |
| 337 | 61 | G | G | C | 4.12 | 0.0921227102 |  |
| 337 | 61 | G | G | G | 86.08 | 0.0000000000 | \* |
| 337 | 61 | G | G | T | 0.52 | 0.8636050006 |  |
| 338 | 62 | A | A | A | 94.72 | 0.0000000000 | \* |
| 338 | 62 | A | A | C | 1.17 | 0.7868608243 |  |
| 338 | 62 | A | A | G | 0.29 | 0.9274993261 |  |
| 338 | 62 | A | A | T | 3.81 | 0.0952111184 |  |
| 339 | 63 | C | C | A | 2.33 | 0.5847663987 |  |
| 339 | 63 | C | C | C | 93.02 | 0.0000000000 | \* |
| 339 | 63 | C | C | G | 1.99 | 0.4233366231 |  |
| 339 | 63 | C | C | T | 2.66 | 0.2645981014 |  |
| 340 | 64 | A | A | A | 90.91 | 0.0000000000 | \* |
| 340 | 64 | A | A | C | 1.70 | 0.6159202396 |  |
| 340 | 64 | A | A | G | 2.27 | 0.3368083550 |  |
| 340 | 64 | A | A | T | 5.11 | 0.0259407345 |  |
| 341 | 65 | G | G | A | 1.77 | 0.6857414213 |  |
| 341 | 65 | G | G | C | 2.83 | 0.2876620474 |  |
| 341 | 65 | G | G | G | 93.99 | 0.0000000000 | \* |
| 341 | 65 | G | G | T | 1.41 | 0.6227568850 |  |
| 342 | 66 | C | C | A | 4.41 | 0.2481683667 |  |
| 342 | 66 | C | C | C | 92.07 | 0.0000000000 | \* |
| 342 | 66 | C | C | G | 1.76 | 0.5035615577 |  |
| 342 | 66 | C | C | T | 1.76 | 0.5080390319 |  |
| 343 | 67 | T | T | A | 1.33 | 0.7534199374 |  |
| 343 | 67 | T | T | C | 0.33 | 0.9354528330 |  |
| 343 | 67 | T | T | G | 2.33 | 0.3197224685 |  |
| 343 | 67 | T | T | T | 96.00 | 0.0000000000 | \* |
| 344 | 68 | C | C | A | 3.69 | 0.3458486254 |  |
| 344 | 68 | C | C | C | 90.77 | 0.0000000000 | \* |
| 344 | 68 | C | C | G | 3.69 | 0.0839061512 |  |
| 344 | 68 | C | C | T | 1.85 | 0.4817071309 |  |
| 345 | 69 | C | C | A | 3.49 | 0.3774637647 |  |
| 345 | 69 | C | C | C | 92.44 | 0.0000000000 | \* |
| 345 | 69 | C | C | G | 1.74 | 0.5100465219 |  |
| 345 | 69 | C | C | T | 2.33 | 0.3432083033 |  |
| 346 | 70 | C | C | A | 4.42 | 0.2458287411 |  |
| 346 | 70 | C | C | C | 89.38 | 0.0000000000 | \* |
| 346 | 70 | C | C | G | 3.10 | 0.1559225685 |  |
| 346 | 70 | C | C | T | 3.10 | 0.1827476739 |  |
| 347 | 71 | G | G | A | 6.00 | 0.1077030556 |  |
| 347 | 71 | G | G | C | 1.00 | 0.8341599710 |  |
| 347 | 71 | G | G | G | 92.00 | 0.0000000000 | \* |
| 347 | 71 | G | G | T | 1.00 | 0.7538197086 |  |
| 348 | 72 | C | C | A | 0.00 | 0.8363636364 |  |
| 348 | 72 | C | C | C | 96.35 | 0.0000000000 | \* |
| 348 | 72 | C | C | G | 0.91 | 0.8105903693 |  |
| 348 | 72 | C | C | T | 2.74 | 0.2474777384 |  |
| 349 | 73 | G | G | A | 9.28 | 0.0149683855 |  |
| 349 | 73 | G | G | C | 1.03 | 0.8261973393 |  |
| 349 | 73 | G | G | G | 88.66 | 0.0000000000 | \* |
| 349 | 73 | G | G | T | 1.03 | 0.7447491400 |  |
| 350 | 74 | A | A | A | 95.68 | 0.0000000000 | \* |
| 350 | 74 | A | A | C | 1.08 | 0.8133351575 |  |
| 350 | 74 | A | A | G | 0.36 | 0.9227133375 |  |
| 350 | 74 | A | A | T | 2.88 | 0.2206152685 |  |
| 351 | 75 | C | C | A | 2.45 | 0.5619829220 |  |
| 351 | 75 | C | C | C | 91.96 | 0.0000000000 | \* |
| 351 | 75 | C | C | G | 2.80 | 0.2093305141 |  |
| 351 | 75 | C | C | T | 2.80 | 0.2359897914 |  |

## For use in R

If you want to work with the results in R, here is output that you can copy and paste in your terminal to get:

The base information:

```
structure(list(focal.base = c("A", "C", "G", "T"), avg.percsignal = c(92.940005433265, 
92.6789143837688, 93.0996119786422, 93.567871557614), avg.areasignal = c(286, 
316.716417910448, 284.666666666667, 338.860465116279), crit.perc.area = c(9.90524813966007, 
6.27390968762134, 5.51411575432483, 6.00785949785977), mu = c(3.63682853064213, 
2.37861272582836, 2.06939275286162, 2.19305822772326), fillibens = c(0.988977538452076, 
0.995383862246658, 0.994162179229307, 0.992582476339279)), .Names = c("focal.base", 
"avg.percsignal", "avg.areasignal", "crit.perc.area", "mu", "fillibens"
), row.names = c(NA, -4L), class = "data.frame")
```

the data.frame that contains information on the guide region:

```
structure(list(A.area = c(266, 311, 7, 514, 18, 18, 17, 15, 15, 
11, 234, 9, 493, 17, 22, 14, 6, 17, 5, 1, 0, 10, 0, 9, 0, 206, 
9, 5, 270, 6, 203, 4, 9, 7, 7, 7, 218, 8, 14, 13, 2, 14, 328, 
7, 14, 15, 370, 10, 14, 7, 3, 130, 246, 2, 261, 233, 3, 2, 3, 
10, 18, 323, 7, 160, 5, 10, 4, 10, 12, 10, 12, 0, 18, 266, 7), 
    C.area = c(5, 5, 8, 7, 321, 6, 5, 5, 205, 346, 6, 6, 6, 320, 
    283, 5, 6, 6, 6, 6, 9, 7, 2, 10, 267, 9, 275, 6, 5, 297, 
    6, 271, 4, 6, 3, 205, 8, 342, 294, 295, 4, 7, 5, 289, 243, 
    6, 4, 253, 4, 4, 164, 2, 5, 5, 0, 1, 5, 8, 4, 234, 8, 4, 
    280, 3, 8, 209, 1, 246, 318, 202, 2, 211, 2, 3, 263), G.area = c(4, 
    7, 234, 9, 0, 11, 518, 331, 10, 10, 7, 240, 4, 1, 9, 274, 
    7, 435, 13, 11, 7, 382, 4, 306, 4, 12, 23, 0, 7, 6, 8, 6, 
    191, 284, 230, 2, 6, 2, 7, 3, 10, 332, 4, 0, 9, 205, 3, 2, 
    144, 285, 7, 5, 4, 194, 4, 3, 157, 0, 10, 8, 167, 1, 6, 4, 
    266, 4, 7, 10, 6, 7, 184, 2, 172, 1, 8), T.area = c(7, 10, 
    0, 20, 8, 339, 6, 3, 4, 8, 8, 0, 20, 2, 1, 4, 213, 5, 260, 
    290, 296, 3, 237, 4, 6, 5, 5, 292, 4, 2, 2, 6, 0, 1, 0, 5, 
    11, 4, 4, 10, 284, 4, 14, 5, 7, 1, 12, 2, 2, 3, 0, 8, 6, 
    2, 9, 9, 4, 212, 226, 3, 1, 13, 8, 9, 4, 4, 288, 5, 8, 7, 
    2, 6, 2, 8, 8), Tot.area = c(282, 333, 249, 550, 347, 374, 
    546, 354, 234, 375, 255, 255, 523, 340, 315, 297, 232, 463, 
    284, 308, 312, 402, 243, 329, 277, 232, 312, 303, 286, 311, 
    219, 287, 204, 298, 240, 219, 243, 356, 319, 321, 300, 357, 
    351, 301, 273, 227, 389, 267, 164, 299, 174, 145, 261, 203, 
    274, 246, 169, 222, 243, 255, 194, 341, 301, 176, 283, 227, 
    300, 271, 344, 226, 200, 219, 194, 278, 286), A.perc = c(94.3262411347518, 
    93.3933933933934, 2.81124497991968, 93.4545454545455, 5.18731988472623, 
    4.81283422459893, 3.11355311355311, 4.23728813559322, 6.41025641025641, 
    2.93333333333333, 91.7647058823529, 3.52941176470588, 94.263862332696, 
    5, 6.98412698412698, 4.71380471380471, 2.58620689655172, 
    3.67170626349892, 1.76056338028169, 0.324675324675325, 0, 
    2.48756218905473, 0, 2.73556231003039, 0, 88.7931034482759, 
    2.88461538461538, 1.65016501650165, 94.4055944055944, 1.92926045016077, 
    92.6940639269406, 1.39372822299652, 4.41176470588235, 2.3489932885906, 
    2.91666666666667, 3.19634703196347, 89.7119341563786, 2.24719101123596, 
    4.38871473354232, 4.04984423676012, 0.666666666666667, 3.92156862745098, 
    93.4472934472934, 2.32558139534884, 5.12820512820513, 6.6079295154185, 
    95.1156812339332, 3.74531835205992, 8.53658536585366, 2.34113712374582, 
    1.72413793103448, 89.6551724137931, 94.2528735632184, 0.985221674876847, 
    95.2554744525548, 94.7154471544715, 1.77514792899408, 0.900900900900901, 
    1.23456790123457, 3.92156862745098, 9.27835051546392, 94.7214076246334, 
    2.32558139534884, 90.9090909090909, 1.76678445229682, 4.40528634361233, 
    1.33333333333333, 3.690036900369, 3.48837209302326, 4.42477876106195, 
    6, 0, 9.27835051546392, 95.6834532374101, 2.44755244755245
    ), C.perc = c(1.77304964539007, 1.5015015015015, 3.21285140562249, 
    1.27272727272727, 92.507204610951, 1.60427807486631, 0.915750915750916, 
    1.41242937853107, 87.6068376068376, 92.2666666666667, 2.35294117647059, 
    2.35294117647059, 1.1472275334608, 94.1176470588235, 89.8412698412698, 
    1.68350168350168, 2.58620689655172, 1.29589632829374, 2.11267605633803, 
    1.94805194805195, 2.88461538461538, 1.74129353233831, 0.823045267489712, 
    3.03951367781155, 96.3898916967509, 3.87931034482759, 88.1410256410256, 
    1.98019801980198, 1.74825174825175, 95.4983922829582, 2.73972602739726, 
    94.4250871080139, 1.96078431372549, 2.01342281879195, 1.25, 
    93.6073059360731, 3.29218106995885, 96.0674157303371, 92.1630094043887, 
    91.9003115264798, 1.33333333333333, 1.96078431372549, 1.42450142450142, 
    96.0132890365448, 89.010989010989, 2.6431718061674, 1.02827763496144, 
    94.7565543071161, 2.4390243902439, 1.33779264214047, 94.2528735632184, 
    1.37931034482759, 1.91570881226054, 2.46305418719212, 0, 
    0.40650406504065, 2.9585798816568, 3.6036036036036, 1.64609053497942, 
    91.7647058823529, 4.12371134020619, 1.17302052785924, 93.0232558139535, 
    1.70454545454545, 2.82685512367491, 92.0704845814978, 0.333333333333333, 
    90.7749077490775, 92.4418604651163, 89.3805309734513, 1, 
    96.3470319634703, 1.03092783505155, 1.07913669064748, 91.958041958042
    ), G.perc = c(1.41843971631206, 2.1021021021021, 93.9759036144578, 
    1.63636363636364, 0, 2.94117647058824, 94.8717948717949, 
    93.5028248587571, 4.27350427350427, 2.66666666666667, 2.74509803921569, 
    94.1176470588235, 0.764818355640535, 0.294117647058824, 2.85714285714286, 
    92.2558922558922, 3.01724137931034, 93.9524838012959, 4.57746478873239, 
    3.57142857142857, 2.24358974358974, 95.0248756218905, 1.64609053497942, 
    93.0091185410334, 1.44404332129964, 5.17241379310345, 7.37179487179487, 
    0, 2.44755244755245, 1.92926045016077, 3.65296803652968, 
    2.09059233449477, 93.6274509803922, 95.3020134228188, 95.8333333333333, 
    0.91324200913242, 2.46913580246914, 0.561797752808989, 2.19435736677116, 
    0.934579439252336, 3.33333333333333, 92.9971988795518, 1.13960113960114, 
    0, 3.2967032967033, 90.3083700440529, 0.77120822622108, 0.749063670411985, 
    87.8048780487805, 95.3177257525084, 4.02298850574713, 3.44827586206897, 
    1.53256704980843, 95.5665024630542, 1.45985401459854, 1.21951219512195, 
    92.8994082840237, 0, 4.11522633744856, 3.13725490196078, 
    86.0824742268041, 0.293255131964809, 1.99335548172757, 2.27272727272727, 
    93.9929328621908, 1.76211453744493, 2.33333333333333, 3.690036900369, 
    1.74418604651163, 3.09734513274336, 92, 0.91324200913242, 
    88.659793814433, 0.359712230215827, 2.7972027972028), T.perc = c(2.4822695035461, 
    3.003003003003, 0, 3.63636363636364, 2.30547550432277, 90.6417112299465, 
    1.0989010989011, 0.847457627118644, 1.70940170940171, 2.13333333333333, 
    3.13725490196078, 0, 3.82409177820268, 0.588235294117647, 
    0.317460317460317, 1.34680134680135, 91.8103448275862, 1.07991360691145, 
    91.5492957746479, 94.1558441558442, 94.8717948717949, 0.746268656716418, 
    97.5308641975309, 1.21580547112462, 2.16606498194946, 2.1551724137931, 
    1.6025641025641, 96.3696369636964, 1.3986013986014, 0.643086816720257, 
    0.91324200913242, 2.09059233449477, 0, 0.335570469798658, 
    0, 2.28310502283105, 4.52674897119342, 1.12359550561798, 
    1.25391849529781, 3.11526479750779, 94.6666666666667, 1.12044817927171, 
    3.98860398860399, 1.66112956810631, 2.56410256410256, 0.440528634361233, 
    3.08483290488432, 0.749063670411985, 1.21951219512195, 1.00334448160535, 
    0, 5.51724137931035, 2.29885057471264, 0.985221674876847, 
    3.28467153284672, 3.65853658536585, 2.36686390532544, 95.4954954954955, 
    93.0041152263374, 1.17647058823529, 0.515463917525773, 3.81231671554252, 
    2.6578073089701, 5.11363636363636, 1.41342756183746, 1.76211453744493, 
    96, 1.8450184501845, 2.32558139534884, 3.09734513274336, 
    1, 2.73972602739726, 1.03092783505155, 2.87769784172662, 
    2.7972027972028), base.call = c("A", "A", "G", "A", "C", 
    "T", "G", "G", "C", "C", "A", "G", "A", "C", "C", "G", "T", 
    "G", "T", "T", "T", "G", "T", "G", "C", "A", "C", "T", "A", 
    "C", "A", "C", "G", "G", "G", "C", "A", "C", "C", "C", "T", 
    "G", "A", "C", "C", "G", "A", "C", "G", "G", "C", "A", "A", 
    "G", "A", "A", "G", "T", "T", "C", "G", "A", "C", "A", "G", 
    "C", "T", "C", "C", "C", "G", "C", "G", "A", "C"), index = 277:351, 
    guide.seq = c("A", "A", "G", "A", "C", "T", "G", "G", "C", 
    "C", "A", "G", "A", "C", "C", "G", "T", "G", "T", "T", "T", 
    "G", "T", "G", "C", "A", "C", "T", "A", "C", "A", "C", "G", 
    "G", "G", "C", "A", "C", "C", "C", "T", "G", "A", "C", "C", 
    "G", "A", "C", "G", "G", "C", "A", "A", "G", "A", "A", "G", 
    "T", "T", "C", "G", "A", "C", "A", "G", "C", "T", "C", "C", 
    "C", "G", "C", "G", "A", "C"), T.pval = c(0.304291158497432, 
    0.198297508691949, 0.893617021275807, 0.112336503396231, 
    0.348438072210447, 0, 0.724242078836734, 0.795629031302461, 
    0.525049835539073, 0.395346122470539, 0.176482572110291, 
    0.893617021275807, 0.0941532678363068, 0.851920944902876, 
    0.885222401448349, 0.644801844830531, 0, 0.73004387050014, 
    0, 0, 0, 0.820108456772139, 0, 0.687486906523518, 0.386139074959375, 
    0.389188390632113, 0.560024010216423, 0, 0.627673438522245, 
    0.841840889801741, 0.778248487678381, 0.407564671441435, 
    0.893617021275807, 0.883860888978317, 0.893617021275807, 
    0.354319147264239, 0.0473333895724019, 0.716618939182276, 
    0.675193655847439, 0.179911966309191, 0, 0.717595185475947, 
    0.0804413586518756, 0.540780410407464, 0.285273395977298, 
    0.873546613603105, 0.184749732979946, 0.819472377701871, 
    0.686296930865198, 0.752847360790791, 0.893617021275807, 
    0.0169543963969894, 0.350172910818461, 0.758090529384595, 
    0.154887298851678, 0.110037286078744, 0.332637431722042, 
    0, 0, 0.700030988131238, 0.863605000632731, 0.0952111183740119, 
    0.264598101442443, 0.0259407345359215, 0.622756884996259, 
    0.508039031857312, 0, 0.481707130868743, 0.343208303254108, 
    0.182747673861434, 0.753819708649868, 0.247477738353303, 
    0.744749140039918, 0.220615268472725, 0.235989791390604), 
    C.pval = c(0.592984529608174, 0.683689316690293, 0.209665245579063, 
    0.756988862968904, 0, 0.649512363363794, 0.854607160255023, 
    0.712826378694316, 0, 0, 0.409979842740829, 0.409979842740829, 
    0.794308547740869, 0, 0, 0.62297535551755, 0.346146424428463, 
    0.749829383871055, 0.482358798207096, 0.535085801699802, 
    0.274763635995335, 0.60360776206061, 0.874809395384633, 0.242329284470511, 
    0, 0.115910265395782, 0, 0.524628828972075, 0.601278402096748, 
    0, 0.307953380778749, 0, 0.530935795575204, 0.513896783712563, 
    0.763937222348967, 0, 0.195948371542714, 0, 0, 0, 0.738111026375161, 
    0.530935795575204, 0.708913381663396, 0, 0, 0.331612853303072, 
    0.82688874411085, 0, 0.385633772047503, 0.736703635649128, 
    0, 0.723492444053862, 0.545673223736334, 0.378999587960613, 
    0.94047619047619, 0.931322341046585, 0.258886867675022, 0.149068285217316, 
    0.635515829116962, 0, 0.0921227102158192, 0.786860824342766, 
    0, 0.615920239627653, 0.287662047448323, 0, 0.935452833010913, 
    0, 0, 0, 0.834159971034586, 0, 0.826197339251123, 0.813335157504167, 
    0), G.pval = c(0.631731994257947, 0.388181914610143, 0, 0.549666364493707, 
    0.933333333333334, 0.182077332146822, 0, 0, 0.0438152946332477, 
    0.236815354895683, 0.219976855589225, 0, 0.852554046138026, 
    0.92744821704283, 0.197604132611955, 0, 0.168911350321093, 
    0, 0.0308389547328534, 0.0953243769693095, 0.345242125421357, 
    0, 0.54605369085935, 0, 0.622039462772715, 0.0151857541878406, 
    0.000928687642469006, 0.933333333333334, 0.28920958849605, 
    0.444874198268078, 0.0873349744296604, 0.391816645344099, 
    0, 0, 0, 0.810590369270938, 0.283691797289737, 0.896585835684384, 
    0.359812905143652, 0.803993326812817, 0.122509278125234, 
    0, 0.735065727952858, 0.933333333333334, 0.127247253464748, 
    0, 0.850902498559305, 0.856561464983971, 0, 0, 0.0581582645289384, 
    0.108631626497463, 0.588564672954953, 0, 0.61605327786321, 
    0.706164355555237, 0, 0.933333333333334, 0.0524363403778219, 
    0.149774927493729, 0, 0.927499326065739, 0.423336623087143, 
    0.336808355032847, 0, 0.503561557724933, 0.319722468459489, 
    0.0839061512084953, 0.510046521903439, 0.155922568496352, 
    0, 0.810590369270938, 0, 0.922713337507972, 0.209330514124715
    ), A.pval = c(0, 0, 0.494564312919137, 0, 0.167136270159584, 
    0.202727479440955, 0.440462618560806, 0.269039893261237, 
    0.0854815921888782, 0.472425920565159, 0, 0.370892614521039, 
    0, 0.184231014346465, 0.0613208518608676, 0.213106834507614, 
    0.536110688683254, 0.348651280556811, 0.686801453502439, 
    0.834198168005945, 0.836363636363636, 0.554507693979048, 
    0.836363636363636, 0.508446963198741, 0.836363636363636, 
    0, 0.481218222082859, 0.705248179119362, 0, 0.657399163864602, 
    0, 0.744874056385023, 0.247388901467568, 0.580397242883265, 
    0.475427190931479, 0.426113286512454, 0, 0.599363549040296, 
    0.250170778293301, 0.293832515392709, 0.821048417865267, 
    0.311714626979381, 0, 0.584766398713173, 0.172381845360071, 
    0.0763250133609935, 0, 0.337484426060124, 0.0239081901702061, 
    0.581863729301878, 0.692965956560408, 0, 0, 0.795386634051556, 
    0, 0, 0.684313122496885, 0.803443947743962, 0.766646080648728, 
    0.311714626979381, 0.0149683854819581, 0, 0.584766398713173, 
    0, 0.685741421274811, 0.248168366718951, 0.753419937408343, 
    0.345848625405017, 0.377463764735855, 0.245828741108898, 
    0.107703055595111, 0.836363636363636, 0.0149683854819581, 
    0, 0.561982922029641), guide.position = 1:75), .Names = c("A.area", 
"C.area", "G.area", "T.area", "Tot.area", "A.perc", "C.perc", 
"G.perc", "T.perc", "base.call", "index", "guide.seq", "T.pval", 
"C.pval", "G.pval", "A.pval", "guide.position"), row.names = 277:351, class = "data.frame")
```

*Report generated using EditR v1.0.8*
