# Supplementary material for: Mechanistic and genetic basis of single-strand templated repair at Cas12a-induced DNA breaks in Chlamydomonas reinhardtii
Source: Nat Commun. 2021 Nov 19;12:6751. doi: 10.1038/s41467-021-27004-1 (PMC8604939; doi:10.1038/s41467-021-27004-1)
Supplement: Supplementary file 22 — Source Data [file 41467_2021_27004_MOESM22_ESM.zip › Source Data/EditR analysis/EditR outputs/Antisense/rep2_ssODN_antisense_32.html]

EditR v1.0.8 report


# EditR v1.0.8 report

- Data QA
  - Filtering data
  - Percent noise peak area
  - Base information
- Predicted editing
  - Editing bar plot
  - Editing table plot
  - Table of editing results
- For use in R

## Data QA

### Filtering data

What the data looked like prefiltering:

and the post filtering signal / noise plot:

### Percent noise peak area

### Base information

Here’s information about the signal of each base, the critical percent value where any higher value would be called as significant, and Filliben’s correlation for how well the noise was modelled by the zero adjusted gamma distribution.

| Base | Average percent signal | Average peak area | Critical percent value | model mu | Fillibens correlation |
| --- | --- | --- | --- | --- | --- |
| A | 92.93530 | 323.3143 | 10.253339 | 3.408272 | 0.9925186 |
| C | 91.87302 | 336.4091 | 6.400505 | 2.179188 | 0.9925800 |
| G | 92.61130 | 313.1579 | 6.662454 | 2.098165 | 0.9935329 |
| T | 93.90183 | 378.4419 | 6.641680 | 2.685948 | 0.9928794 |

## Predicted editing

### Editing bar plot

### Editing table plot

### Table of editing results


Here’s the entire guide region

| Sanger position | Guide position | Guide sequence | Sanger base call | Focal base | Focal base peak area | p value |  |
| --- | --- | --- | --- | --- | --- | --- | --- |
| 274 | 1 | A | A | A | 92.72 | 0.0000000000 | \* |
| 274 | 1 | A | A | C | 2.22 | 0.3784695486 |  |
| 274 | 1 | A | A | G | 1.90 | 0.4455149938 |  |
| 274 | 1 | A | A | T | 3.16 | 0.2623297692 |  |
| 275 | 2 | A | A | A | 91.49 | 0.0000000000 | \* |
| 275 | 2 | A | A | C | 2.93 | 0.2242440831 |  |
| 275 | 2 | A | A | G | 1.06 | 0.7128529720 |  |
| 275 | 2 | A | A | T | 4.52 | 0.0837095726 |  |
| 276 | 3 | G | G | A | 3.31 | 0.3719139894 |  |
| 276 | 3 | G | G | C | 1.47 | 0.6022941566 |  |
| 276 | 3 | G | G | G | 95.22 | 0.0000000000 | \* |
| 276 | 3 | G | G | T | 0.00 | 0.8510638298 |  |
| 277 | 4 | A | A | A | 94.20 | 0.0000000000 | \* |
| 277 | 4 | A | A | C | 0.94 | 0.7745964333 |  |
| 277 | 4 | A | A | G | 0.94 | 0.7544306319 |  |
| 277 | 4 | A | A | T | 3.92 | 0.1429788795 |  |
| 278 | 5 | C | C | A | 5.65 | 0.1252037814 |  |
| 278 | 5 | C | C | C | 91.67 | 0.0000000000 | \* |
| 278 | 5 | C | C | G | 0.81 | 0.7983103642 |  |
| 278 | 5 | C | C | T | 1.88 | 0.5860551986 |  |
| 279 | 6 | T | T | A | 5.59 | 0.1284515100 |  |
| 279 | 6 | T | T | C | 1.86 | 0.4771506842 |  |
| 279 | 6 | T | T | G | 2.33 | 0.3349111953 |  |
| 279 | 6 | T | T | T | 90.21 | 0.0000000000 | \* |
| 280 | 7 | G | G | A | 4.17 | 0.2556094330 |  |
| 280 | 7 | G | G | C | 2.08 | 0.4139392836 |  |
| 280 | 7 | G | G | G | 92.63 | 0.0000000000 | \* |
| 280 | 7 | G | G | T | 1.12 | 0.7732363262 |  |
| 281 | 8 | G | G | A | 4.98 | 0.1745433074 |  |
| 281 | 8 | G | G | C | 2.24 | 0.3723401457 |  |
| 281 | 8 | G | G | G | 91.04 | 0.0000000000 | \* |
| 281 | 8 | G | G | T | 1.74 | 0.6250718014 |  |
| 282 | 9 | C | C | A | 6.30 | 0.0894955937 |  |
| 282 | 9 | C | C | C | 85.43 | 0.0000000000 | \* |
| 282 | 9 | C | C | G | 5.51 | 0.0272230438 |  |
| 282 | 9 | C | C | T | 2.76 | 0.3514478888 |  |
| 283 | 10 | C | C | A | 3.92 | 0.2855177171 |  |
| 283 | 10 | C | C | C | 90.93 | 0.0000000000 | \* |
| 283 | 10 | C | C | G | 2.70 | 0.2591433528 |  |
| 283 | 10 | C | C | T | 2.45 | 0.4283414241 |  |
| 284 | 11 | A | A | A | 92.12 | 0.0000000000 | \* |
| 284 | 11 | A | A | C | 2.05 | 0.4218893074 |  |
| 284 | 11 | A | A | G | 2.05 | 0.4029463956 |  |
| 284 | 11 | A | A | T | 3.77 | 0.1624981400 |  |
| 285 | 12 | G | G | A | 3.94 | 0.2828417875 |  |
| 285 | 12 | G | G | C | 2.87 | 0.2345867223 |  |
| 285 | 12 | G | G | G | 92.47 | 0.0000000000 | \* |
| 285 | 12 | G | G | T | 0.72 | 0.8300752482 |  |
| 286 | 13 | A | A | A | 94.26 | 0.0000000000 | \* |
| 286 | 13 | A | A | C | 0.98 | 0.7613462577 |  |
| 286 | 13 | A | A | G | 0.49 | 0.8893056813 |  |
| 286 | 13 | A | A | T | 4.26 | 0.1058593172 |  |
| 287 | 14 | C | C | A | 4.55 | 0.2144031739 |  |
| 287 | 14 | C | C | C | 93.05 | 0.0000000000 | \* |
| 287 | 14 | C | C | G | 0.00 | 0.9555555556 |  |
| 287 | 14 | C | C | T | 2.41 | 0.4401945463 |  |
| 288 | 15 | C | C | A | 9.25 | 0.0177568138 |  |
| 288 | 15 | C | C | C | 88.06 | 0.0000000000 | \* |
| 288 | 15 | C | C | G | 2.69 | 0.2609209850 |  |
| 288 | 15 | C | C | T | 0.00 | 0.8510638298 |  |
| 289 | 16 | G | G | A | 7.01 | 0.0613986714 |  |
| 289 | 16 | G | G | C | 2.74 | 0.2578475805 |  |
| 289 | 16 | G | G | G | 88.41 | 0.0000000000 | \* |
| 289 | 16 | G | G | T | 1.83 | 0.6007239219 |  |
| 290 | 17 | T | T | A | 0.00 | 0.8623853211 |  |
| 290 | 17 | T | T | C | 2.31 | 0.3548554511 |  |
| 290 | 17 | T | T | G | 2.69 | 0.2598468808 |  |
| 290 | 17 | T | T | T | 95.00 | 0.0000000000 | \* |
| 291 | 18 | G | G | A | 4.46 | 0.2234591345 |  |
| 291 | 18 | G | G | C | 1.16 | 0.7040816285 |  |
| 291 | 18 | G | G | G | 93.41 | 0.0000000000 | \* |
| 291 | 18 | G | G | T | 0.97 | 0.7992783923 |  |
| 292 | 19 | T | T | A | 2.11 | 0.5827615924 |  |
| 292 | 19 | T | T | C | 0.91 | 0.7848615762 |  |
| 292 | 19 | T | T | G | 4.83 | 0.0483079532 |  |
| 292 | 19 | T | T | T | 92.15 | 0.0000000000 | \* |
| 293 | 20 | T | T | A | 1.13 | 0.7645974334 |  |
| 293 | 20 | T | T | C | 0.00 | 0.9294117647 |  |
| 293 | 20 | T | T | G | 3.94 | 0.1000551105 |  |
| 293 | 20 | T | T | T | 94.93 | 0.0000000000 | \* |
| 294 | 21 | T | T | A | 0.27 | 0.8575859048 |  |
| 294 | 21 | T | T | C | 3.20 | 0.1804286562 |  |
| 294 | 21 | T | T | G | 2.13 | 0.3826447320 |  |
| 294 | 21 | T | T | T | 94.40 | 0.0000000000 | \* |
| 295 | 22 | G | G | A | 3.10 | 0.4055583384 |  |
| 295 | 22 | G | G | C | 1.99 | 0.4399572563 |  |
| 295 | 22 | G | G | G | 93.58 | 0.0000000000 | \* |
| 295 | 22 | G | G | T | 1.33 | 0.7304344350 |  |
| 296 | 23 | T | T | A | 0.00 | 0.8623853211 |  |
| 296 | 23 | T | T | C | 0.68 | 0.8481359918 |  |
| 296 | 23 | T | T | G | 1.35 | 0.6155648956 |  |
| 296 | 23 | T | T | T | 97.97 | 0.0000000000 | \* |
| 297 | 24 | G | G | A | 4.10 | 0.2636823429 |  |
| 297 | 24 | G | G | C | 1.37 | 0.6368693732 |  |
| 297 | 24 | G | G | G | 92.08 | 0.0000000000 | \* |
| 297 | 24 | G | G | T | 2.46 | 0.4262186599 |  |
| 298 | 25 | C | C | A | 2.89 | 0.4396691229 |  |
| 298 | 25 | C | C | C | 92.60 | 0.0000000000 | \* |
| 298 | 25 | C | C | G | 1.29 | 0.6374195502 |  |
| 298 | 25 | C | C | T | 3.22 | 0.2524530435 |  |
| 299 | 26 | A | A | A | 88.01 | 0.0000000000 | \* |
| 299 | 26 | A | A | C | 3.75 | 0.1148720775 |  |
| 299 | 26 | A | A | G | 4.12 | 0.0868554221 |  |
| 299 | 26 | A | A | T | 4.12 | 0.1200916164 |  |
| 300 | 27 | C | C | A | 3.81 | 0.2993817633 |  |
| 300 | 27 | C | C | C | 93.46 | 0.0000000000 | \* |
| 300 | 27 | C | C | G | 1.09 | 0.7039936600 |  |
| 300 | 27 | C | C | T | 1.63 | 0.6538861576 |  |
| 301 | 28 | T | T | A | 1.57 | 0.6859429136 |  |
| 301 | 28 | T | T | C | 4.46 | 0.0614696470 |  |
| 301 | 28 | T | T | G | 0.52 | 0.8809130247 |  |
| 301 | 28 | T | T | T | 93.44 | 0.0000000000 | \* |
| 302 | 29 | A | A | A | 95.47 | 0.0000000000 | \* |
| 302 | 29 | A | A | C | 1.81 | 0.4929717243 |  |
| 302 | 29 | A | A | G | 1.21 | 0.6637010480 |  |
| 302 | 29 | A | A | T | 1.51 | 0.6862667024 |  |
| 303 | 30 | C | C | A | 2.40 | 0.5294925601 |  |
| 303 | 30 | C | C | C | 94.61 | 0.0000000000 | \* |
| 303 | 30 | C | C | G | 1.20 | 0.6673850550 |  |
| 303 | 30 | C | C | T | 1.80 | 0.6098621586 |  |
| 304 | 31 | A | A | A | 90.00 | 0.0000000000 | \* |
| 304 | 31 | A | A | C | 2.69 | 0.2681016648 |  |
| 304 | 31 | A | A | G | 3.46 | 0.1461902589 |  |
| 304 | 31 | A | A | T | 3.85 | 0.1520493656 |  |
| 305 | 32 | C | C | A | 1.88 | 0.6277741990 |  |
| 305 | 32 | C | C | C | 92.16 | 0.0000000000 | \* |
| 305 | 32 | C | C | G | 1.88 | 0.4505712357 |  |
| 305 | 32 | C | C | T | 4.08 | 0.1248741673 |  |
| 306 | 33 | G | G | A | 5.65 | 0.1247608980 |  |
| 306 | 33 | G | G | C | 2.61 | 0.2854030428 |  |
| 306 | 33 | G | G | G | 90.00 | 0.0000000000 | \* |
| 306 | 33 | G | G | T | 1.74 | 0.6256653067 |  |
| 307 | 34 | G | G | A | 1.54 | 0.6926965620 |  |
| 307 | 34 | G | G | C | 2.46 | 0.3179448121 |  |
| 307 | 34 | G | G | G | 93.85 | 0.0000000000 | \* |
| 307 | 34 | G | G | T | 2.15 | 0.5094959153 |  |
| 308 | 35 | G | G | A | 3.70 | 0.3143222190 |  |
| 308 | 35 | G | G | C | 1.48 | 0.5987060272 |  |
| 308 | 35 | G | G | G | 92.59 | 0.0000000000 | \* |
| 308 | 35 | G | G | T | 2.22 | 0.4904457405 |  |
| 309 | 36 | C | C | A | 4.02 | 0.2736767902 |  |
| 309 | 36 | C | C | C | 93.17 | 0.0000000000 | \* |
| 309 | 36 | C | C | G | 0.80 | 0.7993459241 |  |
| 309 | 36 | C | C | T | 2.01 | 0.5504976306 |  |
| 310 | 37 | A | A | A | 87.25 | 0.0000000000 | \* |
| 310 | 37 | A | A | C | 2.68 | 0.2696683646 |  |
| 310 | 37 | A | A | G | 4.03 | 0.0936070106 |  |
| 310 | 37 | A | A | T | 6.04 | 0.0188359517 |  |
| 311 | 38 | C | C | A | 4.12 | 0.2606627748 |  |
| 311 | 38 | C | C | C | 93.30 | 0.0000000000 | \* |
| 311 | 38 | C | C | G | 1.03 | 0.7240027775 |  |
| 311 | 38 | C | C | T | 1.55 | 0.6771046932 |  |
| 312 | 39 | C | C | A | 7.41 | 0.0496162776 |  |
| 312 | 39 | C | C | C | 90.03 | 0.0000000000 | \* |
| 312 | 39 | C | C | G | 0.00 | 0.9555555556 |  |
| 312 | 39 | C | C | T | 2.56 | 0.3989055058 |  |
| 313 | 40 | C | C | A | 5.13 | 0.1619734869 |  |
| 313 | 40 | C | C | C | 90.31 | 0.0000000000 | \* |
| 313 | 40 | C | C | G | 0.28 | 0.9322655648 |  |
| 313 | 40 | C | C | T | 4.27 | 0.1048040368 |  |
| 314 | 41 | T | T | A | 1.18 | 0.7565021473 |  |
| 314 | 41 | T | T | C | 3.53 | 0.1377485069 |  |
| 314 | 41 | T | T | G | 1.76 | 0.4843645036 |  |
| 314 | 41 | T | T | T | 93.53 | 0.0000000000 | \* |
| 315 | 42 | G | G | A | 3.23 | 0.3848940019 |  |
| 315 | 42 | G | G | C | 0.99 | 0.7585670011 |  |
| 315 | 42 | G | G | G | 94.54 | 0.0000000000 | \* |
| 315 | 42 | G | G | T | 1.24 | 0.7494778504 |  |
| 316 | 43 | A | A | A | 93.90 | 0.0000000000 | \* |
| 316 | 43 | A | A | C | 1.22 | 0.6854509126 |  |
| 316 | 43 | A | A | G | 1.22 | 0.6599522835 |  |
| 316 | 43 | A | A | T | 3.66 | 0.1778056770 |  |
| 317 | 44 | C | C | A | 3.06 | 0.4120079628 |  |
| 317 | 44 | C | C | C | 95.41 | 0.0000000000 | \* |
| 317 | 44 | C | C | G | 1.53 | 0.5573308575 |  |
| 317 | 44 | C | C | T | 0.00 | 0.8510638298 |  |
| 318 | 45 | C | C | A | 7.26 | 0.0537166686 |  |
| 318 | 45 | C | C | C | 86.47 | 0.0000000000 | \* |
| 318 | 45 | C | C | G | 4.29 | 0.0756371930 |  |
| 318 | 45 | C | C | T | 1.98 | 0.5583461959 |  |
| 319 | 46 | G | G | A | 5.68 | 0.1229042032 |  |
| 319 | 46 | G | G | C | 3.79 | 0.1107876504 |  |
| 319 | 46 | G | G | G | 90.53 | 0.0000000000 | \* |
| 319 | 46 | G | G | T | 0.00 | 0.8510638298 |  |
| 320 | 47 | A | A | A | 95.01 | 0.0000000000 | \* |
| 320 | 47 | A | A | C | 0.87 | 0.7962634505 |  |
| 320 | 47 | A | A | G | 0.65 | 0.8461469125 |  |
| 320 | 47 | A | A | T | 3.47 | 0.2069964941 |  |
| 321 | 48 | C | C | A | 5.03 | 0.1696486998 |  |
| 321 | 48 | C | C | C | 89.93 | 0.0000000000 | \* |
| 321 | 48 | C | C | G | 0.67 | 0.8401455297 |  |
| 321 | 48 | C | C | T | 4.36 | 0.0967528451 |  |
| 322 | 49 | G | G | A | 9.09 | 0.0194769627 |  |
| 322 | 49 | G | G | C | 2.14 | 0.3986991339 |  |
| 322 | 49 | G | G | G | 85.56 | 0.0000000000 | \* |
| 322 | 49 | G | G | T | 3.21 | 0.2537722619 |  |
| 323 | 50 | G | G | A | 0.00 | 0.8623853211 |  |
| 323 | 50 | G | G | C | 1.79 | 0.4996195774 |  |
| 323 | 50 | G | G | G | 96.72 | 0.0000000000 | \* |
| 323 | 50 | G | G | T | 1.49 | 0.6908226407 |  |
| 324 | 51 | C | C | A | 2.05 | 0.5949695603 |  |
| 324 | 51 | C | C | C | 91.79 | 0.0000000000 | \* |
| 324 | 51 | C | C | G | 3.59 | 0.1323299053 |  |
| 324 | 51 | C | C | T | 2.56 | 0.3989055058 |  |
| 325 | 52 | A | A | A | 89.38 | 0.0000000000 | \* |
| 325 | 52 | A | A | C | 1.25 | 0.6753815907 |  |
| 325 | 52 | A | A | G | 1.88 | 0.4522437595 |  |
| 325 | 52 | A | A | T | 7.50 | 0.0039242736 | \* |
| 326 | 53 | A | A | A | 97.52 | 0.0000000000 | \* |
| 326 | 53 | A | A | C | 1.06 | 0.7360890757 |  |
| 326 | 53 | A | A | G | 1.42 | 0.5933165886 |  |
| 326 | 53 | A | A | T | 0.00 | 0.8510638298 |  |
| 327 | 54 | G | G | A | 3.12 | 0.4010512362 |  |
| 327 | 54 | G | G | C | 0.00 | 0.9294117647 |  |
| 327 | 54 | G | G | G | 94.64 | 0.0000000000 | \* |
| 327 | 54 | G | G | T | 2.23 | 0.4876961604 |  |
| 328 | 55 | A | A | A | 95.25 | 0.0000000000 | \* |
| 328 | 55 | A | A | C | 0.63 | 0.8583777684 |  |
| 328 | 55 | A | A | G | 1.58 | 0.5403769079 |  |
| 328 | 55 | A | A | T | 2.53 | 0.4072498514 |  |
| 329 | 56 | A | A | A | 93.13 | 0.0000000000 | \* |
| 329 | 56 | A | A | C | 0.69 | 0.8452632150 |  |
| 329 | 56 | A | A | G | 0.69 | 0.8353290656 |  |
| 329 | 56 | A | A | T | 5.50 | 0.0327095915 |  |
| 330 | 57 | G | G | A | 2.67 | 0.4783322211 |  |
| 330 | 57 | G | G | C | 3.21 | 0.1791894354 |  |
| 330 | 57 | G | G | G | 90.91 | 0.0000000000 | \* |
| 330 | 57 | G | G | T | 3.21 | 0.2537722619 |  |
| 331 | 58 | T | T | A | 2.92 | 0.4352502163 |  |
| 331 | 58 | T | T | C | 2.92 | 0.2252626589 |  |
| 331 | 58 | T | T | G | 0.73 | 0.8223607043 |  |
| 331 | 58 | T | T | T | 93.43 | 0.0000000000 | \* |
| 332 | 59 | T | T | A | 0.00 | 0.8623853211 |  |
| 332 | 59 | T | T | C | 1.74 | 0.5147917245 |  |
| 332 | 59 | T | T | G | 4.18 | 0.0826540236 |  |
| 332 | 59 | T | T | T | 94.08 | 0.0000000000 | \* |
| 333 | 60 | C | C | A | 3.23 | 0.3848940019 |  |
| 333 | 60 | C | C | C | 92.83 | 0.0000000000 | \* |
| 333 | 60 | C | C | G | 2.87 | 0.2288289665 |  |
| 333 | 60 | C | C | T | 1.08 | 0.7817249971 |  |
| 334 | 61 | G | G | A | 6.70 | 0.0725595378 |  |
| 334 | 61 | G | G | C | 1.91 | 0.4625027405 |  |
| 334 | 61 | G | G | G | 87.56 | 0.0000000000 | \* |
| 334 | 61 | G | G | T | 3.83 | 0.1544314564 |  |
| 335 | 62 | A | A | A | 94.47 | 0.0000000000 | \* |
| 335 | 62 | A | A | C | 1.32 | 0.6535763501 |  |
| 335 | 62 | A | A | G | 1.32 | 0.6274635550 |  |
| 335 | 62 | A | A | T | 2.89 | 0.3192566361 |  |
| 336 | 63 | C | C | A | 2.85 | 0.4475686291 |  |
| 336 | 63 | C | C | C | 94.30 | 0.0000000000 | \* |
| 336 | 63 | C | C | G | 1.90 | 0.4455149938 |  |
| 336 | 63 | C | C | T | 0.95 | 0.8022302986 |  |
| 337 | 64 | A | A | A | 91.30 | 0.0000000000 | \* |
| 337 | 64 | A | A | C | 1.93 | 0.4570487109 |  |
| 337 | 64 | A | A | G | 2.90 | 0.2236514378 |  |
| 337 | 64 | A | A | T | 3.86 | 0.1496752269 |  |
| 338 | 65 | G | G | A | 3.06 | 0.4120079628 |  |
| 338 | 65 | G | G | C | 2.75 | 0.2562100201 |  |
| 338 | 65 | G | G | G | 92.66 | 0.0000000000 | \* |
| 338 | 65 | G | G | T | 1.53 | 0.6815586560 |  |
| 339 | 66 | C | C | A | 3.79 | 0.3029431059 |  |
| 339 | 66 | C | C | C | 90.91 | 0.0000000000 | \* |
| 339 | 66 | C | C | G | 2.27 | 0.3484838783 |  |
| 339 | 66 | C | C | T | 3.03 | 0.2897041430 |  |
| 340 | 67 | T | T | A | 0.00 | 0.8623853211 |  |
| 340 | 67 | T | T | C | 0.82 | 0.8087536725 |  |
| 340 | 67 | T | T | G | 2.47 | 0.3036683201 |  |
| 340 | 67 | T | T | T | 96.70 | 0.0000000000 | \* |
| 341 | 68 | C | C | A | 3.39 | 0.3595369665 |  |
| 341 | 68 | C | C | C | 91.86 | 0.0000000000 | \* |
| 341 | 68 | C | C | G | 2.71 | 0.2562153372 |  |
| 341 | 68 | C | C | T | 2.03 | 0.5432047822 |  |
| 342 | 69 | C | C | A | 2.20 | 0.5668671636 |  |
| 342 | 69 | C | C | C | 93.41 | 0.0000000000 | \* |
| 342 | 69 | C | C | G | 0.00 | 0.9555555556 |  |
| 342 | 69 | C | C | T | 4.40 | 0.0938892026 |  |
| 343 | 70 | C | C | A | 0.00 | 0.8623853211 |  |
| 343 | 70 | C | C | C | 94.42 | 0.0000000000 | \* |
| 343 | 70 | C | C | G | 1.29 | 0.6369547763 |  |
| 343 | 70 | C | C | T | 4.29 | 0.1030970249 |  |
| 344 | 71 | G | G | A | 5.75 | 0.1185943617 |  |
| 344 | 71 | G | G | C | 4.42 | 0.0635435253 |  |
| 344 | 71 | G | G | G | 88.50 | 0.0000000000 | \* |
| 344 | 71 | G | G | T | 1.33 | 0.7304344350 |  |
| 345 | 72 | C | C | A | 2.02 | 0.6017353852 |  |
| 345 | 72 | C | C | C | 93.95 | 0.0000000000 | \* |
| 345 | 72 | C | C | G | 0.40 | 0.9098586435 |  |
| 345 | 72 | C | C | T | 3.63 | 0.1821601944 |  |
| 346 | 73 | G | G | A | 8.07 | 0.0344752557 |  |
| 346 | 73 | G | G | C | 2.69 | 0.2684499737 |  |
| 346 | 73 | G | G | G | 87.44 | 0.0000000000 | \* |
| 346 | 73 | G | G | T | 1.79 | 0.6106067866 |  |
| 347 | 74 | A | A | A | 97.78 | 0.0000000000 | \* |
| 347 | 74 | A | A | C | 1.90 | 0.4652041413 |  |
| 347 | 74 | A | A | G | 0.32 | 0.9267407142 |  |
| 347 | 74 | A | A | T | 0.00 | 0.8510638298 |  |
| 348 | 75 | C | C | A | 3.10 | 0.4057824251 |  |
| 348 | 75 | C | C | C | 83.28 | 0.0000000000 | \* |
| 348 | 75 | C | C | G | 10.53 | 0.0002943278 | \* |
| 348 | 75 | C | C | T | 3.10 | 0.2760762890 |  |

## For use in R

If you want to work with the results in R, here is output that you can copy and paste in your terminal to get:

The base information:

```
structure(list(focal.base = c("A", "C", "G", "T"), avg.percsignal = c(92.9352998093197, 
91.873022035783, 92.6112975875289, 93.9018253155937), avg.areasignal = c(323.314285714286, 
336.409090909091, 313.157894736842, 378.441860465116), crit.perc.area = c(10.2533385969626, 
6.40050505606821, 6.66245429916494, 6.64167992496608), mu = c(3.40827230094786, 
2.17918785227356, 2.0981652234923, 2.68594789333706), fillibens = c(0.992518572111596, 
0.992580045871785, 0.993532917233391, 0.992879369588763)), .Names = c("focal.base", 
"avg.percsignal", "avg.areasignal", "crit.perc.area", "mu", "fillibens"
), row.names = c(NA, -4L), class = "data.frame")
```

the data.frame that contains information on the guide region:

```
structure(list(A.area = c(293, 344, 9, 601, 21, 24, 26, 20, 16, 
16, 269, 11, 575, 17, 31, 23, 0, 23, 7, 4, 1, 14, 0, 15, 9, 235, 
14, 6, 316, 8, 234, 6, 13, 5, 10, 10, 260, 16, 26, 18, 4, 13, 
385, 10, 22, 15, 438, 15, 17, 0, 4, 143, 275, 7, 301, 271, 5, 
8, 0, 9, 14, 359, 9, 189, 10, 10, 0, 10, 8, 0, 13, 5, 18, 308, 
10), C.area = c(7, 11, 4, 6, 341, 8, 13, 9, 217, 371, 6, 8, 6, 
348, 295, 9, 6, 6, 3, 0, 12, 9, 2, 5, 288, 10, 343, 17, 6, 316, 
7, 294, 6, 8, 4, 232, 8, 362, 316, 317, 12, 4, 5, 312, 262, 10, 
4, 268, 4, 6, 179, 2, 3, 0, 2, 2, 6, 8, 5, 259, 4, 5, 298, 4, 
9, 240, 3, 271, 340, 220, 10, 233, 6, 6, 269), G.area = c(6, 
4, 259, 6, 3, 10, 578, 366, 14, 11, 6, 258, 3, 0, 9, 290, 7, 
482, 16, 14, 8, 423, 4, 337, 4, 11, 4, 2, 4, 4, 9, 6, 207, 305, 
250, 2, 12, 4, 0, 1, 6, 381, 5, 5, 13, 239, 3, 2, 160, 324, 7, 
3, 4, 212, 5, 2, 170, 2, 12, 8, 183, 5, 6, 6, 303, 6, 9, 8, 0, 
3, 200, 1, 195, 1, 34), T.area = c(10, 17, 0, 25, 7, 387, 7, 
7, 7, 10, 11, 2, 26, 9, 0, 6, 247, 5, 305, 337, 354, 6, 290, 
9, 10, 11, 6, 356, 5, 6, 10, 13, 4, 7, 6, 5, 18, 6, 9, 15, 318, 
5, 15, 0, 6, 0, 16, 13, 6, 5, 5, 12, 0, 5, 8, 16, 6, 256, 270, 
3, 8, 11, 3, 8, 5, 8, 352, 6, 16, 10, 3, 9, 4, 0, 10), Tot.area = c(316, 
376, 272, 638, 372, 429, 624, 402, 254, 408, 292, 279, 610, 374, 
335, 328, 260, 516, 331, 355, 375, 452, 296, 366, 311, 267, 367, 
381, 331, 334, 260, 319, 230, 325, 270, 249, 298, 388, 351, 351, 
340, 403, 410, 327, 303, 264, 461, 298, 187, 335, 195, 160, 282, 
224, 316, 291, 187, 274, 287, 279, 209, 380, 316, 207, 327, 264, 
364, 295, 364, 233, 226, 248, 223, 315, 323), A.perc = c(92.7215189873418, 
91.4893617021277, 3.30882352941176, 94.2006269592476, 5.64516129032258, 
5.59440559440559, 4.16666666666667, 4.97512437810945, 6.2992125984252, 
3.92156862745098, 92.1232876712329, 3.9426523297491, 94.2622950819672, 
4.54545454545455, 9.25373134328358, 7.01219512195122, 0, 4.45736434108527, 
2.11480362537764, 1.12676056338028, 0.266666666666667, 3.09734513274336, 
0, 4.0983606557377, 2.89389067524116, 88.0149812734082, 3.81471389645777, 
1.5748031496063, 95.4682779456193, 2.39520958083832, 90, 1.88087774294671, 
5.65217391304348, 1.53846153846154, 3.7037037037037, 4.01606425702811, 
87.248322147651, 4.12371134020619, 7.40740740740741, 5.12820512820513, 
1.17647058823529, 3.2258064516129, 93.9024390243902, 3.05810397553517, 
7.26072607260726, 5.68181818181818, 95.0108459869848, 5.03355704697987, 
9.09090909090909, 0, 2.05128205128205, 89.375, 97.5177304964539, 
3.125, 95.253164556962, 93.127147766323, 2.67379679144385, 2.91970802919708, 
0, 3.2258064516129, 6.69856459330144, 94.4736842105263, 2.84810126582278, 
91.304347826087, 3.05810397553517, 3.78787878787879, 0, 3.38983050847458, 
2.1978021978022, 0, 5.75221238938053, 2.01612903225806, 8.07174887892377, 
97.7777777777778, 3.09597523219814), C.perc = c(2.21518987341772, 
2.92553191489362, 1.47058823529412, 0.940438871473354, 91.6666666666667, 
1.86480186480186, 2.08333333333333, 2.23880597014925, 85.4330708661417, 
90.9313725490196, 2.05479452054795, 2.8673835125448, 0.983606557377049, 
93.048128342246, 88.0597014925373, 2.74390243902439, 2.30769230769231, 
1.16279069767442, 0.906344410876133, 0, 3.2, 1.99115044247788, 
0.675675675675676, 1.36612021857923, 92.604501607717, 3.74531835205992, 
93.4604904632153, 4.46194225721785, 1.81268882175227, 94.6107784431138, 
2.69230769230769, 92.1630094043887, 2.60869565217391, 2.46153846153846, 
1.48148148148148, 93.1726907630522, 2.68456375838926, 93.298969072165, 
90.02849002849, 90.3133903133903, 3.52941176470588, 0.992555831265509, 
1.21951219512195, 95.4128440366973, 86.4686468646865, 3.78787878787879, 
0.867678958785249, 89.9328859060403, 2.13903743315508, 1.7910447761194, 
91.7948717948718, 1.25, 1.06382978723404, 0, 0.632911392405063, 
0.687285223367698, 3.20855614973262, 2.91970802919708, 1.74216027874564, 
92.831541218638, 1.91387559808612, 1.31578947368421, 94.3037974683544, 
1.93236714975845, 2.75229357798165, 90.9090909090909, 0.824175824175824, 
91.864406779661, 93.4065934065934, 94.4206008583691, 4.42477876106195, 
93.9516129032258, 2.69058295964126, 1.9047619047619, 83.28173374613
), G.perc = c(1.89873417721519, 1.06382978723404, 95.2205882352941, 
0.940438871473354, 0.806451612903226, 2.33100233100233, 92.6282051282051, 
91.044776119403, 5.51181102362205, 2.69607843137255, 2.05479452054795, 
92.4731182795699, 0.491803278688525, 0, 2.6865671641791, 88.4146341463415, 
2.69230769230769, 93.4108527131783, 4.83383685800604, 3.94366197183099, 
2.13333333333333, 93.5840707964602, 1.35135135135135, 92.0765027322404, 
1.28617363344051, 4.11985018726592, 1.08991825613079, 0.5249343832021, 
1.20845921450151, 1.19760479041916, 3.46153846153846, 1.88087774294671, 
90, 93.8461538461538, 92.5925925925926, 0.803212851405622, 4.02684563758389, 
1.03092783505155, 0, 0.284900284900285, 1.76470588235294, 94.5409429280397, 
1.21951219512195, 1.52905198776758, 4.29042904290429, 90.530303030303, 
0.650759219088937, 0.671140939597315, 85.5614973262032, 96.7164179104478, 
3.58974358974359, 1.875, 1.41843971631206, 94.6428571428571, 
1.58227848101266, 0.687285223367698, 90.9090909090909, 0.72992700729927, 
4.18118466898955, 2.8673835125448, 87.5598086124402, 1.31578947368421, 
1.89873417721519, 2.89855072463768, 92.6605504587156, 2.27272727272727, 
2.47252747252747, 2.71186440677966, 0, 1.28755364806867, 88.4955752212389, 
0.403225806451613, 87.4439461883408, 0.317460317460317, 10.5263157894737
), T.perc = c(3.16455696202532, 4.52127659574468, 0, 3.91849529780564, 
1.88172043010753, 90.2097902097902, 1.12179487179487, 1.74129353233831, 
2.75590551181102, 2.45098039215686, 3.76712328767123, 0.716845878136201, 
4.26229508196721, 2.40641711229947, 0, 1.82926829268293, 95, 
0.968992248062015, 92.1450151057402, 94.9295774647887, 94.4, 
1.32743362831858, 97.972972972973, 2.45901639344262, 3.21543408360129, 
4.11985018726592, 1.63487738419619, 93.4383202099738, 1.51057401812689, 
1.79640718562874, 3.84615384615385, 4.07523510971787, 1.73913043478261, 
2.15384615384615, 2.22222222222222, 2.00803212851406, 6.04026845637584, 
1.54639175257732, 2.56410256410256, 4.27350427350427, 93.5294117647059, 
1.24069478908189, 3.65853658536585, 0, 1.98019801980198, 0, 3.470715835141, 
4.36241610738255, 3.20855614973262, 1.49253731343284, 2.56410256410256, 
7.5, 0, 2.23214285714286, 2.53164556962025, 5.49828178694158, 
3.20855614973262, 93.4306569343066, 94.0766550522648, 1.0752688172043, 
3.82775119617225, 2.89473684210526, 0.949367088607595, 3.86473429951691, 
1.52905198776758, 3.03030303030303, 96.7032967032967, 2.03389830508475, 
4.3956043956044, 4.29184549356223, 1.32743362831858, 3.62903225806452, 
1.79372197309417, 0, 3.09597523219814), base.call = c("A", "A", 
"G", "A", "C", "T", "G", "G", "C", "C", "A", "G", "A", "C", "C", 
"G", "T", "G", "T", "T", "T", "G", "T", "G", "C", "A", "C", "T", 
"A", "C", "A", "C", "G", "G", "G", "C", "A", "C", "C", "C", "T", 
"G", "A", "C", "C", "G", "A", "C", "G", "G", "C", "A", "A", "G", 
"A", "A", "G", "T", "T", "C", "G", "A", "C", "A", "G", "C", "T", 
"C", "C", "C", "G", "C", "G", "A", "C"), index = 274:348, guide.seq = c("A", 
"A", "G", "A", "C", "T", "G", "G", "C", "C", "A", "G", "A", "C", 
"C", "G", "T", "G", "T", "T", "T", "G", "T", "G", "C", "A", "C", 
"T", "A", "C", "A", "C", "G", "G", "G", "C", "A", "C", "C", "C", 
"T", "G", "A", "C", "C", "G", "A", "C", "G", "G", "C", "A", "A", 
"G", "A", "A", "G", "T", "T", "C", "G", "A", "C", "A", "G", "C", 
"T", "C", "C", "C", "G", "C", "G", "A", "C"), T.pval = c(0.262329769226327, 
0.0837095725938913, 0.851063829787234, 0.142978879504219, 0.586055198624948, 
0, 0.773236326246857, 0.625071801381544, 0.351447888782524, 0.428341424143114, 
0.162498139970981, 0.830075248167096, 0.105859317178581, 0.440194546304203, 
0.851063829787234, 0.600723921861652, 0, 0.799278392293715, 0, 
0, 0, 0.73043443497895, 0, 0.426218659913412, 0.252453043481341, 
0.120091616370439, 0.653886157604517, 0, 0.686266702427323, 0.609862158633327, 
0.152049365581631, 0.124874167282566, 0.625665306708198, 0.509495915292415, 
0.490445740525585, 0.550497630603438, 0.0188359517160768, 0.677104693179192, 
0.398905505768244, 0.104804036833353, 0, 0.749477850395756, 0.177805677049166, 
0.851063829787234, 0.55834619587977, 0.851063829787234, 0.206996494064654, 
0.096752845066696, 0.253772261926999, 0.69082264067694, 0.398905505768244, 
0.00392427359868897, 0.851063829787234, 0.487696160410504, 0.407249851423525, 
0.0327095914804779, 0.253772261926999, 0, 0, 0.781724997116271, 
0.154431456428025, 0.319256636085687, 0.802230298609712, 0.149675226947936, 
0.681558656002449, 0.289704143027625, 0, 0.543204782150137, 0.0938892026293884, 
0.103097024870771, 0.73043443497895, 0.182160194437886, 0.610606786577636, 
0.851063829787234, 0.276076289046652), C.pval = c(0.378469548564884, 
0.224244083145014, 0.602294156582465, 0.77459643326023, 0, 0.477150684191435, 
0.413939283622855, 0.372340145733364, 0, 0, 0.4218893073843, 
0.234586722341716, 0.761346257695379, 0, 0, 0.257847580540918, 
0.354855451146023, 0.704081628489602, 0.784861576205684, 0.929411764705882, 
0.180428656171352, 0.439957256280338, 0.848135991829667, 0.636869373243742, 
0, 0.114872077460302, 0, 0.0614696469871472, 0.492971724303107, 
0, 0.268101664826913, 0, 0.285403042774445, 0.31794481205887, 
0.598706027213253, 0, 0.269668364599763, 0, 0, 0, 0.13774850692164, 
0.758567001119817, 0.685450912590686, 0, 0, 0.110787650444242, 
0.796263450477037, 0, 0.398699133863807, 0.499619577430347, 0, 
0.675381590697684, 0.736089075686251, 0.929411764705882, 0.858377768396144, 
0.84526321496844, 0.179189435427415, 0.225262658863077, 0.514791724465584, 
0, 0.462502740522143, 0.653576350134616, 0, 0.457048710912091, 
0.256210020124101, 0, 0.808753672503589, 0, 0, 0, 0.0635435252525196, 
0, 0.268449973664714, 0.465204141340111, 0), G.pval = c(0.445514993843927, 
0.712852971954359, 0, 0.754430631884146, 0.798310364230677, 0.334911195270961, 
0, 0, 0.0272230438338438, 0.259143352798811, 0.402946395614586, 
0, 0.889305681332811, 0.955555555555295, 0.260920985001134, 0, 
0.259846880799636, 0, 0.0483079531969816, 0.10005511046934, 0.382644732020501, 
0, 0.615564895573866, 0, 0.637419550166528, 0.0868554220916217, 
0.703993660010128, 0.880913024702994, 0.663701048007689, 0.667385055009712, 
0.146190258935034, 0.450571235657773, 0, 0, 0, 0.799345924050882, 
0.0936070106072142, 0.724002777522713, 0.955555555555295, 0.932265564811562, 
0.484364503636345, 0, 0.659952283528643, 0.557330857512521, 0.0756371929821311, 
0, 0.846146912454107, 0.840145529675939, 0, 0, 0.132329905334327, 
0.452243759478047, 0.593316588568614, 0, 0.540376907897605, 0.835329065631938, 
0, 0.822360704325282, 0.0826540235674817, 0.228828966465997, 
0, 0.627463554950868, 0.445514993843927, 0.223651437834872, 0, 
0.348483878344305, 0.303668320101727, 0.25621533716643, 0.955555555555295, 
0.636954776269927, 0, 0.909858643532962, 0, 0.926740714183876, 
0.000294327813995188), A.pval = c(0, 0, 0.371913989448147, 0, 
0.125203781363022, 0.128451510022864, 0.255609432956887, 0.174543307423848, 
0.0894955936903371, 0.285517717066126, 0, 0.28284178746457, 0, 
0.214403173937227, 0.0177568138392452, 0.0613986714057041, 0.862385321100916, 
0.223459134545762, 0.582761592387529, 0.764597433351818, 0.857585904809909, 
0.405558338423279, 0.862385321100916, 0.263682342935385, 0.439669122898626, 
0, 0.299381763313301, 0.685942913626049, 0, 0.529492560136098, 
0, 0.627774198996027, 0.12476089801796, 0.692696562048053, 0.314322219030597, 
0.273676790174243, 0, 0.260662774757599, 0.0496162776400992, 
0.161973486900338, 0.756502147295735, 0.384894001864588, 0, 0.412007962833008, 
0.053716668644455, 0.122904203164586, 0, 0.169648699789276, 0.0194769627391884, 
0.862385321100916, 0.594969560295928, 0, 0, 0.401051236233156, 
0, 0, 0.47833222105408, 0.435250216332954, 0.862385321100916, 
0.384894001864588, 0.0725595377897362, 0, 0.447568629135198, 
0, 0.412007962833008, 0.30294310588789, 0.862385321100916, 0.359536966492711, 
0.566867163577435, 0.862385321100916, 0.118594361663987, 0.601735385235259, 
0.034475255702808, 0, 0.405782425064147), guide.position = 1:75), .Names = c("A.area", 
"C.area", "G.area", "T.area", "Tot.area", "A.perc", "C.perc", 
"G.perc", "T.perc", "base.call", "index", "guide.seq", "T.pval", 
"C.pval", "G.pval", "A.pval", "guide.position"), row.names = 274:348, class = "data.frame")
```

*Report generated using EditR v1.0.8*
