# Supplementary material for: Mechanistic and genetic basis of single-strand templated repair at Cas12a-induced DNA breaks in Chlamydomonas reinhardtii
Source: Nat Commun. 2021 Nov 19;12:6751. doi: 10.1038/s41467-021-27004-1 (PMC8604939; doi:10.1038/s41467-021-27004-1)
Supplement: Supplementary file 22 — Source Data [file 41467_2021_27004_MOESM22_ESM.zip › Source Data/EditR analysis/EditR outputs/Antisense/rep2_ssODN_antisense_-32.html]

EditR v1.0.8 report


# EditR v1.0.8 report

- Data QA
  - Filtering data
  - Percent noise peak area
  - Base information
- Predicted editing
  - Editing bar plot
  - Editing table plot
  - Table of editing results
- For use in R

## Data QA

### Filtering data

What the data looked like prefiltering:

and the post filtering signal / noise plot:

### Percent noise peak area

### Base information

Here’s information about the signal of each base, the critical percent value where any higher value would be called as significant, and Filliben’s correlation for how well the noise was modelled by the zero adjusted gamma distribution.

| Base | Average percent signal | Average peak area | Critical percent value | model mu | Fillibens correlation |
| --- | --- | --- | --- | --- | --- |
| A | 93.14154 | 356.2059 | 9.173953 | 2.877731 | 0.9965207 |
| C | 93.92964 | 370.2239 | 5.566731 | 1.967489 | 0.9945900 |
| G | 92.92584 | 354.1964 | 4.542480 | 1.736138 | 0.9903109 |
| T | 95.02298 | 418.7500 | 6.026890 | 2.264676 | 0.9944186 |

## Predicted editing

### Editing bar plot

### Editing table plot

### Table of editing results


Here’s the entire guide region

| Sanger position | Guide position | Guide sequence | Sanger base call | Focal base | Focal base peak area | p value |  |
| --- | --- | --- | --- | --- | --- | --- | --- |
| 276 | 1 | A | A | A | 93.98 | 0.0000000000 | \* |
| 276 | 1 | A | A | C | 2.11 | 0.3628046638 |  |
| 276 | 1 | A | A | G | 0.60 | 0.8705566035 |  |
| 276 | 1 | A | A | T | 3.31 | 0.1672880754 |  |
| 277 | 2 | A | A | A | 92.56 | 0.0000000000 | \* |
| 277 | 2 | A | A | C | 1.74 | 0.4852179923 |  |
| 277 | 2 | A | A | G | 1.24 | 0.6157956323 |  |
| 277 | 2 | A | A | T | 4.47 | 0.0543216561 |  |
| 278 | 3 | G | G | A | 2.90 | 0.3248704101 |  |
| 278 | 3 | G | G | C | 3.55 | 0.0936414359 |  |
| 278 | 3 | G | G | G | 93.55 | 0.0000000000 | \* |
| 278 | 3 | G | G | T | 0.00 | 0.9354838710 |  |
| 279 | 4 | A | A | A | 94.46 | 0.0000000000 | \* |
| 279 | 4 | A | A | C | 1.02 | 0.7598171026 |  |
| 279 | 4 | A | A | G | 0.73 | 0.8314897658 |  |
| 279 | 4 | A | A | T | 3.79 | 0.1068527790 |  |
| 280 | 5 | C | C | A | 3.81 | 0.2091088227 |  |
| 280 | 5 | C | C | C | 91.88 | 0.0000000000 | \* |
| 280 | 5 | C | C | G | 2.54 | 0.1643443305 |  |
| 280 | 5 | C | C | T | 1.78 | 0.5562165112 |  |
| 281 | 6 | T | T | A | 4.28 | 0.1637202645 |  |
| 281 | 6 | T | T | C | 3.21 | 0.1316934909 |  |
| 281 | 6 | T | T | G | 2.14 | 0.2616733708 |  |
| 281 | 6 | T | T | T | 90.36 | 0.0000000000 | \* |
| 282 | 7 | G | G | A | 5.21 | 0.0997453364 |  |
| 282 | 7 | G | G | C | 0.00 | 0.9642857143 |  |
| 282 | 7 | G | G | G | 93.75 | 0.0000000000 | \* |
| 282 | 7 | G | G | T | 1.04 | 0.8010470615 |  |
| 283 | 8 | G | G | A | 5.12 | 0.1045320553 |  |
| 283 | 8 | G | G | C | 2.00 | 0.3948237259 |  |
| 283 | 8 | G | G | G | 91.54 | 0.0000000000 | \* |
| 283 | 8 | G | G | T | 1.34 | 0.7078777586 |  |
| 284 | 9 | C | C | A | 3.11 | 0.2942985704 |  |
| 284 | 9 | C | C | C | 90.27 | 0.0000000000 | \* |
| 284 | 9 | C | C | G | 4.28 | 0.0148635776 |  |
| 284 | 9 | C | C | T | 2.33 | 0.3794005181 |  |
| 285 | 10 | C | C | A | 1.83 | 0.5168172015 |  |
| 285 | 10 | C | C | C | 93.38 | 0.0000000000 | \* |
| 285 | 10 | C | C | G | 2.74 | 0.1278181317 |  |
| 285 | 10 | C | C | T | 2.05 | 0.4639886570 |  |
| 286 | 11 | A | A | A | 85.98 | 0.0000000000 | \* |
| 286 | 11 | A | A | C | 2.18 | 0.3416424210 |  |
| 286 | 11 | A | A | G | 2.18 | 0.2503592086 |  |
| 286 | 11 | A | A | T | 9.66 | 0.0001290843 | \* |
| 287 | 12 | G | G | A | 2.79 | 0.3429233277 |  |
| 287 | 12 | G | G | C | 2.48 | 0.2642970476 |  |
| 287 | 12 | G | G | G | 94.74 | 0.0000000000 | \* |
| 287 | 12 | G | G | T | 0.00 | 0.9354838710 |  |
| 288 | 13 | A | A | A | 94.65 | 0.0000000000 | \* |
| 288 | 13 | A | A | C | 0.94 | 0.7877105134 |  |
| 288 | 13 | A | A | G | 0.63 | 0.8627978407 |  |
| 288 | 13 | A | A | T | 3.78 | 0.1079490609 |  |
| 289 | 14 | C | C | A | 3.77 | 0.2131933080 |  |
| 289 | 14 | C | C | C | 92.46 | 0.0000000000 | \* |
| 289 | 14 | C | C | G | 2.26 | 0.2283650544 |  |
| 289 | 14 | C | C | T | 1.51 | 0.6492936819 |  |
| 290 | 15 | C | C | A | 3.77 | 0.2132717074 |  |
| 290 | 15 | C | C | C | 94.20 | 0.0000000000 | \* |
| 290 | 15 | C | C | G | 1.74 | 0.3993370782 |  |
| 290 | 15 | C | C | T | 0.29 | 0.9312488707 |  |
| 291 | 16 | G | G | A | 6.06 | 0.0620964024 |  |
| 291 | 16 | G | G | C | 2.20 | 0.3350461302 |  |
| 291 | 16 | G | G | G | 90.36 | 0.0000000000 | \* |
| 291 | 16 | G | G | T | 1.38 | 0.6939860890 |  |
| 292 | 17 | T | T | A | 0.00 | 0.8090909091 |  |
| 292 | 17 | T | T | C | 2.51 | 0.2567862303 |  |
| 292 | 17 | T | T | G | 2.51 | 0.1702854352 |  |
| 292 | 17 | T | T | T | 94.98 | 0.0000000000 | \* |
| 293 | 18 | G | G | A | 4.25 | 0.1667263422 |  |
| 293 | 18 | G | G | C | 1.24 | 0.6754010271 |  |
| 293 | 18 | G | G | G | 94.16 | 0.0000000000 | \* |
| 293 | 18 | G | G | T | 0.35 | 0.9277969542 |  |
| 294 | 19 | T | T | A | 0.00 | 0.8090909091 |  |
| 294 | 19 | T | T | C | 0.85 | 0.8203856709 |  |
| 294 | 19 | T | T | G | 4.26 | 0.0152875699 |  |
| 294 | 19 | T | T | T | 94.89 | 0.0000000000 | \* |
| 295 | 20 | T | T | A | 0.26 | 0.7974297098 |  |
| 295 | 20 | T | T | C | 0.00 | 0.9642857143 |  |
| 295 | 20 | T | T | G | 2.62 | 0.1476937078 |  |
| 295 | 20 | T | T | T | 97.11 | 0.0000000000 | \* |
| 296 | 21 | T | T | A | 0.00 | 0.8090909091 |  |
| 296 | 21 | T | T | C | 2.52 | 0.2545029178 |  |
| 296 | 21 | T | T | G | 2.02 | 0.3005556417 |  |
| 296 | 21 | T | T | T | 95.47 | 0.0000000000 | \* |
| 297 | 22 | G | G | A | 3.56 | 0.2370174010 |  |
| 297 | 22 | G | G | C | 1.98 | 0.4038106874 |  |
| 297 | 22 | G | G | G | 93.68 | 0.0000000000 | \* |
| 297 | 22 | G | G | T | 0.79 | 0.8656381640 |  |
| 298 | 23 | T | T | A | 0.00 | 0.8090909091 |  |
| 298 | 23 | T | T | C | 1.85 | 0.4469933446 |  |
| 298 | 23 | T | T | G | 1.54 | 0.4820057311 |  |
| 298 | 23 | T | T | T | 96.62 | 0.0000000000 | \* |
| 299 | 24 | G | G | A | 3.63 | 0.2283660151 |  |
| 299 | 24 | G | G | C | 1.21 | 0.6865009693 |  |
| 299 | 24 | G | G | G | 93.70 | 0.0000000000 | \* |
| 299 | 24 | G | G | T | 1.45 | 0.6682006874 |  |
| 300 | 25 | C | C | A | 1.21 | 0.6437550415 |  |
| 300 | 25 | C | C | C | 95.17 | 0.0000000000 | \* |
| 300 | 25 | C | C | G | 1.81 | 0.3712089363 |  |
| 300 | 25 | C | C | T | 1.81 | 0.5439319757 |  |
| 301 | 26 | A | A | A | 90.07 | 0.0000000000 | \* |
| 301 | 26 | A | A | C | 3.77 | 0.0745626362 |  |
| 301 | 26 | A | A | G | 2.40 | 0.1947680844 |  |
| 301 | 26 | A | A | T | 3.77 | 0.1092495290 |  |
| 302 | 27 | C | C | A | 2.53 | 0.3858664483 |  |
| 302 | 27 | C | C | C | 95.45 | 0.0000000000 | \* |
| 302 | 27 | C | C | G | 0.00 | 0.9340659341 |  |
| 302 | 27 | C | C | T | 2.02 | 0.4750802658 |  |
| 303 | 28 | T | T | A | 1.65 | 0.5517046266 |  |
| 303 | 28 | T | T | C | 3.07 | 0.1510189664 |  |
| 303 | 28 | T | T | G | 0.71 | 0.8381067751 |  |
| 303 | 28 | T | T | T | 94.56 | 0.0000000000 | \* |
| 304 | 29 | A | A | A | 95.39 | 0.0000000000 | \* |
| 304 | 29 | A | A | C | 1.63 | 0.5257219183 |  |
| 304 | 29 | A | A | G | 1.36 | 0.5634902534 |  |
| 304 | 29 | A | A | T | 1.63 | 0.6081810234 |  |
| 305 | 30 | C | C | A | 1.90 | 0.5016861636 |  |
| 305 | 30 | C | C | C | 96.47 | 0.0000000000 | \* |
| 305 | 30 | C | C | G | 0.00 | 0.9340659341 |  |
| 305 | 30 | C | C | T | 1.63 | 0.6066478678 |  |
| 306 | 31 | A | A | A | 91.23 | 0.0000000000 | \* |
| 306 | 31 | A | A | C | 2.81 | 0.1950958394 |  |
| 306 | 31 | A | A | G | 2.46 | 0.1815238157 |  |
| 306 | 31 | A | A | T | 3.51 | 0.1396532914 |  |
| 307 | 32 | C | C | A | 1.18 | 0.6488478692 |  |
| 307 | 32 | C | C | C | 94.08 | 0.0000000000 | \* |
| 307 | 32 | C | C | G | 1.18 | 0.6420675207 |  |
| 307 | 32 | C | C | T | 3.55 | 0.1343187681 |  |
| 308 | 33 | G | G | A | 4.37 | 0.1568045196 |  |
| 308 | 33 | G | G | C | 1.98 | 0.4012955433 |  |
| 308 | 33 | G | G | G | 92.86 | 0.0000000000 | \* |
| 308 | 33 | G | G | T | 0.79 | 0.8649459407 |  |
| 309 | 34 | G | G | A | 2.36 | 0.4155645678 |  |
| 309 | 34 | G | G | C | 1.57 | 0.5464644611 |  |
| 309 | 34 | G | G | G | 94.76 | 0.0000000000 | \* |
| 309 | 34 | G | G | T | 1.31 | 0.7170526437 |  |
| 310 | 35 | G | G | A | 4.93 | 0.1157802398 |  |
| 310 | 35 | G | G | C | 1.32 | 0.6451833250 |  |
| 310 | 35 | G | G | G | 93.75 | 0.0000000000 | \* |
| 310 | 35 | G | G | T | 0.00 | 0.9354838710 |  |
| 311 | 36 | C | C | A | 3.33 | 0.2646249855 |  |
| 311 | 36 | C | C | C | 93.33 | 0.0000000000 | \* |
| 311 | 36 | C | C | G | 1.11 | 0.6750255143 |  |
| 311 | 36 | C | C | T | 2.22 | 0.4122117422 |  |
| 312 | 37 | A | A | A | 89.27 | 0.0000000000 | \* |
| 312 | 37 | A | A | C | 2.84 | 0.1892599070 |  |
| 312 | 37 | A | A | G | 2.84 | 0.1125596510 |  |
| 312 | 37 | A | A | T | 5.05 | 0.0294727585 |  |
| 313 | 38 | C | C | A | 1.92 | 0.4984534712 |  |
| 313 | 38 | C | C | C | 95.44 | 0.0000000000 | \* |
| 313 | 38 | C | C | G | 1.20 | 0.6349139938 |  |
| 313 | 38 | C | C | T | 1.44 | 0.6729928945 |  |
| 314 | 39 | C | C | A | 3.91 | 0.1988361565 |  |
| 314 | 39 | C | C | C | 91.67 | 0.0000000000 | \* |
| 314 | 39 | C | C | G | 2.08 | 0.2790449296 |  |
| 314 | 39 | C | C | T | 2.34 | 0.3768117823 |  |
| 315 | 40 | C | C | A | 2.91 | 0.3238380320 |  |
| 315 | 40 | C | C | C | 93.65 | 0.0000000000 | \* |
| 315 | 40 | C | C | G | 0.00 | 0.9340659341 |  |
| 315 | 40 | C | C | T | 3.44 | 0.1490075301 |  |
| 316 | 41 | T | T | A | 1.28 | 0.6286847749 |  |
| 316 | 41 | T | T | C | 2.82 | 0.1926240297 |  |
| 316 | 41 | T | T | G | 1.79 | 0.3779060961 |  |
| 316 | 41 | T | T | T | 94.10 | 0.0000000000 | \* |
| 317 | 42 | G | G | A | 4.33 | 0.1597987570 |  |
| 317 | 42 | G | G | C | 1.52 | 0.5675882711 |  |
| 317 | 42 | G | G | G | 93.07 | 0.0000000000 | \* |
| 317 | 42 | G | G | T | 1.08 | 0.7891049139 |  |
| 318 | 43 | A | A | A | 93.79 | 0.0000000000 | \* |
| 318 | 43 | A | A | C | 1.28 | 0.6573716526 |  |
| 318 | 43 | A | A | G | 1.07 | 0.6932405883 |  |
| 318 | 43 | A | A | T | 3.85 | 0.1003879011 |  |
| 319 | 44 | C | C | A | 2.52 | 0.3865936346 |  |
| 319 | 44 | C | C | C | 95.80 | 0.0000000000 | \* |
| 319 | 44 | C | C | G | 0.00 | 0.9340659341 |  |
| 319 | 44 | C | C | T | 1.68 | 0.5892415152 |  |
| 320 | 45 | C | C | A | 5.20 | 0.1002680648 |  |
| 320 | 45 | C | C | C | 89.91 | 0.0000000000 | \* |
| 320 | 45 | C | C | G | 3.67 | 0.0363111773 |  |
| 320 | 45 | C | C | T | 1.22 | 0.7451879508 |  |
| 321 | 46 | G | G | A | 6.83 | 0.0401161805 |  |
| 321 | 46 | G | G | C | 2.73 | 0.2096446289 |  |
| 321 | 46 | G | G | G | 90.44 | 0.0000000000 | \* |
| 321 | 46 | G | G | T | 0.00 | 0.9354838710 |  |
| 322 | 47 | A | A | A | 94.96 | 0.0000000000 | \* |
| 322 | 47 | A | A | C | 1.21 | 0.6868836767 |  |
| 322 | 47 | A | A | G | 0.81 | 0.8036104654 |  |
| 322 | 47 | A | A | T | 3.02 | 0.2164608433 |  |
| 323 | 48 | C | C | A | 3.03 | 0.3060592464 |  |
| 323 | 48 | C | C | C | 92.42 | 0.0000000000 | \* |
| 323 | 48 | C | C | G | 0.61 | 0.8695520142 |  |
| 323 | 48 | C | C | T | 3.94 | 0.0923774274 |  |
| 324 | 49 | G | G | A | 10.43 | 0.0046615363 | \* |
| 324 | 49 | G | G | C | 2.37 | 0.2905466667 |  |
| 324 | 49 | G | G | G | 85.78 | 0.0000000000 | \* |
| 324 | 49 | G | G | T | 1.42 | 0.6788415740 |  |
| 325 | 50 | G | G | A | 4.35 | 0.1582302597 |  |
| 325 | 50 | G | G | C | 1.28 | 0.6597410918 |  |
| 325 | 50 | G | G | G | 94.37 | 0.0000000000 | \* |
| 325 | 50 | G | G | T | 0.00 | 0.9354838710 |  |
| 326 | 51 | C | C | A | 1.33 | 0.6193375996 |  |
| 326 | 51 | C | C | C | 92.92 | 0.0000000000 | \* |
| 326 | 51 | C | C | G | 2.65 | 0.1422364816 |  |
| 326 | 51 | C | C | T | 3.10 | 0.2030197713 |  |
| 327 | 52 | A | A | A | 95.81 | 0.0000000000 | \* |
| 327 | 52 | A | A | C | 1.80 | 0.4641952622 |  |
| 327 | 52 | A | A | G | 2.40 | 0.1952432677 |  |
| 327 | 52 | A | A | T | 0.00 | 0.9354838710 |  |
| 328 | 53 | A | A | A | 94.85 | 0.0000000000 | \* |
| 328 | 53 | A | A | C | 0.30 | 0.9514317503 |  |
| 328 | 53 | A | A | G | 0.91 | 0.7630636016 |  |
| 328 | 53 | A | A | T | 3.94 | 0.0923774274 |  |
| 329 | 54 | G | G | A | 2.79 | 0.3425347116 |  |
| 329 | 54 | G | G | C | 2.39 | 0.2853049873 |  |
| 329 | 54 | G | G | G | 93.63 | 0.0000000000 | \* |
| 329 | 54 | G | G | T | 1.20 | 0.7541803718 |  |
| 330 | 55 | A | A | A | 95.86 | 0.0000000000 | \* |
| 330 | 55 | A | A | C | 0.55 | 0.9082967592 |  |
| 330 | 55 | A | A | G | 1.10 | 0.6778025119 |  |
| 330 | 55 | A | A | T | 2.49 | 0.3378583843 |  |
| 331 | 56 | A | A | A | 93.96 | 0.0000000000 | \* |
| 331 | 56 | A | A | C | 0.60 | 0.8955742086 |  |
| 331 | 56 | A | A | G | 0.91 | 0.7641947369 |  |
| 331 | 56 | A | A | T | 4.53 | 0.0507820877 |  |
| 332 | 57 | G | G | A | 3.76 | 0.2145938314 |  |
| 332 | 57 | G | G | C | 0.94 | 0.7898523449 |  |
| 332 | 57 | G | G | G | 92.96 | 0.0000000000 | \* |
| 332 | 57 | G | G | T | 2.35 | 0.3757737783 |  |
| 333 | 58 | T | T | A | 2.69 | 0.3577717533 |  |
| 333 | 58 | T | T | C | 2.69 | 0.2169397471 |  |
| 333 | 58 | T | T | G | 0.67 | 0.8496732655 |  |
| 333 | 58 | T | T | T | 93.94 | 0.0000000000 | \* |
| 334 | 59 | T | T | A | 0.31 | 0.7928980764 |  |
| 334 | 59 | T | T | C | 1.25 | 0.6710544836 |  |
| 334 | 59 | T | T | G | 2.50 | 0.1721499881 |  |
| 334 | 59 | T | T | T | 95.94 | 0.0000000000 | \* |
| 335 | 60 | C | C | A | 1.96 | 0.4900987138 |  |
| 335 | 60 | C | C | C | 95.10 | 0.0000000000 | \* |
| 335 | 60 | C | C | G | 1.31 | 0.5853021300 |  |
| 335 | 60 | C | C | T | 1.63 | 0.6054155251 |  |
| 336 | 61 | G | G | A | 7.50 | 0.0271041768 |  |
| 336 | 61 | G | G | C | 2.50 | 0.2588603677 |  |
| 336 | 61 | G | G | G | 86.67 | 0.0000000000 | \* |
| 336 | 61 | G | G | T | 3.33 | 0.1642510200 |  |
| 337 | 62 | A | A | A | 94.17 | 0.0000000000 | \* |
| 337 | 62 | A | A | C | 1.35 | 0.6335927315 |  |
| 337 | 62 | A | A | G | 1.35 | 0.5679120265 |  |
| 337 | 62 | A | A | T | 3.14 | 0.1956726552 |  |
| 338 | 63 | C | C | A | 2.56 | 0.3804869326 |  |
| 338 | 63 | C | C | C | 93.18 | 0.0000000000 | \* |
| 338 | 63 | C | C | G | 1.99 | 0.3092185762 |  |
| 338 | 63 | C | C | T | 2.27 | 0.3972656110 |  |
| 339 | 64 | A | A | A | 92.64 | 0.0000000000 | \* |
| 339 | 64 | A | A | C | 1.73 | 0.4871420173 |  |
| 339 | 64 | A | A | G | 2.16 | 0.2549651543 |  |
| 339 | 64 | A | A | T | 3.46 | 0.1457169727 |  |
| 340 | 65 | G | G | A | 2.18 | 0.4479510314 |  |
| 340 | 65 | G | G | C | 2.45 | 0.2701222123 |  |
| 340 | 65 | G | G | G | 94.28 | 0.0000000000 | \* |
| 340 | 65 | G | G | T | 1.09 | 0.7868099616 |  |
| 341 | 66 | C | C | A | 2.08 | 0.4662794186 |  |
| 341 | 66 | C | C | C | 90.62 | 0.0000000000 | \* |
| 341 | 66 | C | C | G | 2.78 | 0.1217756205 |  |
| 341 | 66 | C | C | T | 4.51 | 0.0517281072 |  |
| 342 | 67 | T | T | A | 0.49 | 0.7716368282 |  |
| 342 | 67 | T | T | C | 0.99 | 0.7720302929 |  |
| 342 | 67 | T | T | G | 1.98 | 0.3136457136 |  |
| 342 | 67 | T | T | T | 96.54 | 0.0000000000 | \* |
| 343 | 68 | C | C | A | 1.53 | 0.5766562222 |  |
| 343 | 68 | C | C | C | 94.79 | 0.0000000000 | \* |
| 343 | 68 | C | C | G | 1.53 | 0.4840423226 |  |
| 343 | 68 | C | C | T | 2.15 | 0.4349915236 |  |
| 344 | 69 | C | C | A | 1.47 | 0.5897242535 |  |
| 344 | 69 | C | C | C | 94.12 | 0.0000000000 | \* |
| 344 | 69 | C | C | G | 0.00 | 0.9340659341 |  |
| 344 | 69 | C | C | T | 4.41 | 0.0574664652 |  |
| 345 | 70 | C | C | A | 0.00 | 0.8090909091 |  |
| 345 | 70 | C | C | C | 98.43 | 0.0000000000 | \* |
| 345 | 70 | C | C | G | 1.57 | 0.4664513481 |  |
| 345 | 70 | C | C | T | 0.00 | 0.9354838710 |  |
| 346 | 71 | G | G | A | 5.77 | 0.0731385610 |  |
| 346 | 71 | G | G | C | 1.54 | 0.5586861762 |  |
| 346 | 71 | G | G | G | 91.54 | 0.0000000000 | \* |
| 346 | 71 | G | G | T | 1.15 | 0.7672332442 |  |
| 347 | 72 | C | C | A | 0.00 | 0.8090909091 |  |
| 347 | 72 | C | C | C | 96.04 | 0.0000000000 | \* |
| 347 | 72 | C | C | G | 0.72 | 0.8346928004 |  |
| 347 | 72 | C | C | T | 3.24 | 0.1791888532 |  |
| 348 | 73 | G | G | A | 9.09 | 0.0105141365 |  |
| 348 | 73 | G | G | C | 2.77 | 0.2026212641 |  |
| 348 | 73 | G | G | G | 88.14 | 0.0000000000 | \* |
| 348 | 73 | G | G | T | 0.00 | 0.9354838710 |  |
| 349 | 74 | A | A | A | 94.81 | 0.0000000000 | \* |
| 349 | 74 | A | A | C | 1.37 | 0.6254223175 |  |
| 349 | 74 | A | A | G | 0.55 | 0.8849666560 |  |
| 349 | 74 | A | A | T | 3.28 | 0.1726266615 |  |
| 350 | 75 | C | C | A | 1.67 | 0.5483299055 |  |
| 350 | 75 | C | C | C | 93.59 | 0.0000000000 | \* |
| 350 | 75 | C | C | G | 1.39 | 0.5463987431 |  |
| 350 | 75 | C | C | T | 3.34 | 0.1628625363 |  |

## For use in R

If you want to work with the results in R, here is output that you can copy and paste in your terminal to get:

The base information:

```
structure(list(focal.base = c("A", "C", "G", "T"), avg.percsignal = c(93.141538733258, 
93.9296373172427, 92.9258394699552, 95.022980363033), avg.areasignal = c(356.205882352941, 
370.223880597015, 354.196428571429, 418.75), crit.perc.area = c(9.17395333538441, 
5.56673109297917, 4.54247983763881, 6.02689027217293), mu = c(2.87773085845679, 
1.96748953065933, 1.73613805546457, 2.26467577421016), fillibens = c(0.99652072701696, 
0.994589985590343, 0.990310915699324, 0.994418574828444)), .Names = c("focal.base", 
"avg.percsignal", "avg.areasignal", "crit.perc.area", "mu", "fillibens"
), row.names = c(NA, -4L), class = "data.frame")
```

the data.frame that contains information on the guide region:

```
structure(list(A.area = c(312, 373, 9, 648, 15, 20, 35, 23, 8, 
8, 276, 9, 601, 15, 13, 22, 0, 24, 0, 1, 0, 18, 0, 15, 4, 263, 
10, 7, 352, 7, 260, 4, 11, 9, 15, 9, 283, 8, 15, 11, 5, 20, 438, 
9, 17, 20, 471, 10, 22, 17, 3, 160, 313, 7, 347, 311, 8, 8, 1, 
6, 18, 420, 9, 214, 8, 6, 2, 5, 6, 0, 15, 0, 23, 347, 6), C.area = c(7, 
7, 11, 7, 362, 15, 0, 9, 232, 409, 7, 8, 6, 368, 325, 8, 7, 7, 
3, 0, 10, 10, 6, 5, 315, 11, 378, 13, 6, 355, 8, 318, 5, 6, 4, 
252, 9, 398, 352, 354, 11, 7, 6, 342, 294, 8, 6, 305, 5, 5, 210, 
3, 1, 6, 2, 2, 2, 8, 4, 291, 6, 6, 328, 4, 9, 261, 4, 309, 384, 
250, 4, 267, 7, 5, 336), G.area = c(2, 5, 290, 5, 10, 10, 630, 
411, 11, 12, 7, 306, 4, 9, 6, 328, 7, 532, 15, 10, 8, 474, 5, 
387, 6, 7, 0, 3, 5, 0, 7, 4, 234, 362, 285, 3, 9, 5, 8, 0, 7, 
430, 5, 0, 12, 265, 4, 2, 181, 369, 6, 4, 3, 235, 4, 3, 198, 
2, 8, 4, 208, 6, 7, 5, 346, 8, 8, 5, 0, 4, 238, 2, 223, 2, 5), 
    T.area = c(11, 18, 0, 26, 7, 422, 7, 6, 6, 9, 31, 0, 24, 
    6, 1, 5, 265, 2, 334, 370, 379, 4, 314, 6, 6, 11, 8, 400, 
    6, 6, 10, 12, 2, 5, 0, 6, 16, 6, 9, 13, 367, 5, 18, 6, 4, 
    0, 15, 13, 3, 0, 7, 0, 13, 3, 9, 15, 5, 279, 307, 5, 8, 14, 
    8, 8, 4, 13, 391, 7, 18, 0, 3, 9, 0, 12, 12), Tot.area = c(332, 
    403, 310, 686, 394, 467, 672, 449, 257, 438, 321, 323, 635, 
    398, 345, 363, 279, 565, 352, 381, 397, 506, 325, 413, 331, 
    292, 396, 423, 369, 368, 285, 338, 252, 382, 304, 270, 317, 
    417, 384, 378, 390, 462, 467, 357, 327, 293, 496, 330, 211, 
    391, 226, 167, 330, 251, 362, 331, 213, 297, 320, 306, 240, 
    446, 352, 231, 367, 288, 405, 326, 408, 254, 260, 278, 253, 
    366, 359), A.perc = c(93.9759036144578, 92.5558312655087, 
    2.90322580645161, 94.4606413994169, 3.80710659898477, 4.28265524625268, 
    5.20833333333333, 5.12249443207127, 3.11284046692607, 1.82648401826484, 
    85.981308411215, 2.78637770897833, 94.6456692913386, 3.76884422110553, 
    3.76811594202899, 6.06060606060606, 0, 4.24778761061947, 
    0, 0.26246719160105, 0, 3.55731225296443, 0, 3.6319612590799, 
    1.20845921450151, 90.0684931506849, 2.52525252525253, 1.6548463356974, 
    95.3929539295393, 1.90217391304348, 91.2280701754386, 1.18343195266272, 
    4.36507936507936, 2.35602094240838, 4.93421052631579, 3.33333333333333, 
    89.2744479495268, 1.91846522781775, 3.90625, 2.91005291005291, 
    1.28205128205128, 4.32900432900433, 93.7901498929336, 2.52100840336134, 
    5.19877675840979, 6.8259385665529, 94.9596774193548, 3.03030303030303, 
    10.4265402843602, 4.34782608695652, 1.32743362831858, 95.8083832335329, 
    94.8484848484848, 2.78884462151394, 95.8563535911602, 93.9577039274924, 
    3.75586854460094, 2.69360269360269, 0.3125, 1.96078431372549, 
    7.5, 94.1704035874439, 2.55681818181818, 92.6406926406926, 
    2.17983651226158, 2.08333333333333, 0.493827160493827, 1.53374233128834, 
    1.47058823529412, 0, 5.76923076923077, 0, 9.09090909090909, 
    94.8087431693989, 1.67130919220056), C.perc = c(2.10843373493976, 
    1.73697270471464, 3.54838709677419, 1.02040816326531, 91.8781725888325, 
    3.21199143468951, 0, 2.00445434298441, 90.272373540856, 93.37899543379, 
    2.18068535825545, 2.47678018575851, 0.94488188976378, 92.462311557789, 
    94.2028985507246, 2.20385674931129, 2.5089605734767, 1.23893805309735, 
    0.852272727272727, 0, 2.51889168765743, 1.97628458498024, 
    1.84615384615385, 1.21065375302663, 95.166163141994, 3.76712328767123, 
    95.4545454545455, 3.07328605200946, 1.6260162601626, 96.4673913043478, 
    2.80701754385965, 94.0828402366864, 1.98412698412698, 1.57068062827225, 
    1.31578947368421, 93.3333333333333, 2.8391167192429, 95.4436450839329, 
    91.6666666666667, 93.6507936507936, 2.82051282051282, 1.51515151515152, 
    1.2847965738758, 95.7983193277311, 89.9082568807339, 2.73037542662116, 
    1.20967741935484, 92.4242424242424, 2.3696682464455, 1.27877237851662, 
    92.9203539823009, 1.79640718562874, 0.303030303030303, 2.39043824701195, 
    0.552486187845304, 0.604229607250755, 0.938967136150235, 
    2.69360269360269, 1.25, 95.0980392156863, 2.5, 1.34529147982063, 
    93.1818181818182, 1.73160173160173, 2.45231607629428, 90.625, 
    0.987654320987654, 94.7852760736196, 94.1176470588235, 98.4251968503937, 
    1.53846153846154, 96.0431654676259, 2.76679841897233, 1.36612021857923, 
    93.5933147632312), G.perc = c(0.602409638554217, 1.24069478908189, 
    93.5483870967742, 0.728862973760933, 2.53807106598985, 2.14132762312634, 
    93.75, 91.5367483296214, 4.28015564202335, 2.73972602739726, 
    2.18068535825545, 94.7368421052632, 0.62992125984252, 2.26130653266332, 
    1.73913043478261, 90.3581267217631, 2.5089605734767, 94.1592920353982, 
    4.26136363636364, 2.6246719160105, 2.01511335012594, 93.6758893280632, 
    1.53846153846154, 93.7046004842615, 1.81268882175227, 2.3972602739726, 
    0, 0.709219858156028, 1.3550135501355, 0, 2.45614035087719, 
    1.18343195266272, 92.8571428571429, 94.7643979057592, 93.75, 
    1.11111111111111, 2.8391167192429, 1.19904076738609, 2.08333333333333, 
    0, 1.79487179487179, 93.0735930735931, 1.07066381156317, 
    0, 3.6697247706422, 90.4436860068259, 0.806451612903226, 
    0.606060606060606, 85.781990521327, 94.3734015345268, 2.65486725663717, 
    2.39520958083832, 0.909090909090909, 93.6254980079681, 1.10497237569061, 
    0.906344410876133, 92.9577464788732, 0.673400673400673, 2.5, 
    1.30718954248366, 86.6666666666667, 1.34529147982063, 1.98863636363636, 
    2.16450216450216, 94.2779291553133, 2.77777777777778, 1.97530864197531, 
    1.53374233128834, 0, 1.5748031496063, 91.5384615384615, 0.719424460431655, 
    88.1422924901186, 0.546448087431694, 1.39275766016713), T.perc = c(3.31325301204819, 
    4.46650124069479, 0, 3.79008746355685, 1.77664974619289, 
    90.3640256959315, 1.04166666666667, 1.33630289532294, 2.33463035019455, 
    2.05479452054795, 9.65732087227414, 0, 3.77952755905512, 
    1.50753768844221, 0.289855072463768, 1.37741046831956, 94.9820788530466, 
    0.353982300884956, 94.8863636363636, 97.1128608923885, 95.4659949622166, 
    0.790513833992095, 96.6153846153846, 1.45278450363196, 1.81268882175227, 
    3.76712328767123, 2.02020202020202, 94.5626477541371, 1.6260162601626, 
    1.6304347826087, 3.50877192982456, 3.55029585798817, 0.793650793650794, 
    1.30890052356021, 0, 2.22222222222222, 5.04731861198738, 
    1.43884892086331, 2.34375, 3.43915343915344, 94.1025641025641, 
    1.08225108225108, 3.85438972162741, 1.68067226890756, 1.22324159021407, 
    0, 3.0241935483871, 3.93939393939394, 1.4218009478673, 0, 
    3.09734513274336, 0, 3.93939393939394, 1.19521912350598, 
    2.48618784530387, 4.53172205438066, 2.34741784037559, 93.9393939393939, 
    95.9375, 1.63398692810458, 3.33333333333333, 3.1390134529148, 
    2.27272727272727, 3.46320346320346, 1.08991825613079, 4.51388888888889, 
    96.5432098765432, 2.14723926380368, 4.41176470588235, 0, 
    1.15384615384615, 3.23741007194245, 0, 3.27868852459016, 
    3.34261838440111), base.call = c("A", "A", "G", "A", "C", 
    "T", "G", "G", "C", "C", "A", "G", "A", "C", "C", "G", "T", 
    "G", "T", "T", "T", "G", "T", "G", "C", "A", "C", "T", "A", 
    "C", "A", "C", "G", "G", "G", "C", "A", "C", "C", "C", "T", 
    "G", "A", "C", "C", "G", "A", "C", "G", "G", "C", "A", "A", 
    "G", "A", "A", "G", "T", "T", "C", "G", "A", "C", "A", "G", 
    "C", "T", "C", "C", "C", "G", "C", "G", "A", "C"), index = 276:350, 
    guide.seq = c("A", "A", "G", "A", "C", "T", "G", "G", "C", 
    "C", "A", "G", "A", "C", "C", "G", "T", "G", "T", "T", "T", 
    "G", "T", "G", "C", "A", "C", "T", "A", "C", "A", "C", "G", 
    "G", "G", "C", "A", "C", "C", "C", "T", "G", "A", "C", "C", 
    "G", "A", "C", "G", "G", "C", "A", "A", "G", "A", "A", "G", 
    "T", "T", "C", "G", "A", "C", "A", "G", "C", "T", "C", "C", 
    "C", "G", "C", "G", "A", "C"), T.pval = c(0.167288075394795, 
    0.0543216560764588, 0.935483870967742, 0.106852778952942, 
    0.556216511211454, 0, 0.801047061529615, 0.70787775864738, 
    0.37940051808412, 0.463988656950274, 0.000129084267444846, 
    0.935483870967742, 0.107949060932161, 0.649293681916156, 
    0.931248870697952, 0.693986089019472, 0, 0.927796954214374, 
    0, 0, 0, 0.865638163978527, 0, 0.668200687392656, 0.543931975713543, 
    0.109249529016319, 0.47508026581392, 0, 0.608181023442675, 
    0.606647867766295, 0.139653291371407, 0.134318768145018, 
    0.864945940665595, 0.71705264370671, 0.935483870967742, 0.412211742171626, 
    0.0294727584670835, 0.672992894459246, 0.376811782257824, 
    0.149007530074815, 0, 0.789104913940756, 0.100387901105415, 
    0.5892415152164, 0.745187950848121, 0.935483870967742, 0.216460843259385, 
    0.0923774274361757, 0.678841573955535, 0.935483870967742, 
    0.203019771325727, 0.935483870967742, 0.0923774274361757, 
    0.754180371764809, 0.337858384346092, 0.0507820876762011, 
    0.375773778341195, 0, 0, 0.605415525109339, 0.164251019965422, 
    0.19567265524487, 0.397265610975216, 0.145716972702896, 0.786809961621117, 
    0.0517281071622098, 0, 0.434991523634217, 0.0574664652059602, 
    0.935483870967742, 0.767233244230967, 0.179188853170652, 
    0.935483870967742, 0.172626661513452, 0.162862536348225), 
    C.pval = c(0.362804663752873, 0.485217992255108, 0.0936414358729307, 
    0.759817102602495, 0, 0.131693490904828, 0.964285714274344, 
    0.394823725909253, 0, 0, 0.341642421017129, 0.264297047645829, 
    0.787710513404313, 0, 0, 0.335046130202113, 0.256786230271109, 
    0.675401027083706, 0.820385670933894, 0.964285714274344, 
    0.254502917792362, 0.40381068736454, 0.446993344628231, 0.686500969261188, 
    0, 0.0745626361551092, 0, 0.151018966397777, 0.525721918310412, 
    0, 0.195095839411581, 0, 0.401295543342, 0.546464461085143, 
    0.645183324968612, 0, 0.189259906958955, 0, 0, 0, 0.192624029714695, 
    0.56758827110363, 0.657371652645304, 0, 0, 0.209644628854614, 
    0.686883676726978, 0, 0.29054666666212, 0.659741091774452, 
    0, 0.464195262190813, 0.951431750277018, 0.285304987330452, 
    0.908296759198947, 0.895574208623301, 0.789852344946516, 
    0.216939747054547, 0.671054483601562, 0, 0.258860367720658, 
    0.633592731549861, 0, 0.487142017266067, 0.270122212299034, 
    0, 0.772030292929174, 0, 0, 0, 0.558686176161558, 0, 0.202621264109955, 
    0.625422317533689, 0), G.pval = c(0.870556603531986, 0.615795632291755, 
    0, 0.831489765833941, 0.164344330519642, 0.261673370777715, 
    0, 0, 0.0148635776430976, 0.12781813170426, 0.250359208557714, 
    0, 0.862797840736458, 0.228365054434863, 0.399337078155567, 
    0, 0.170285435167105, 0, 0.0152875699350001, 0.14769370778347, 
    0.300555641728352, 0, 0.48200573112328, 0, 0.371208936319363, 
    0.194768084359908, 0.934065934065934, 0.838106775066746, 
    0.563490253421695, 0.934065934065934, 0.181523815725096, 
    0.642067520748207, 0, 0, 0, 0.675025514343037, 0.112559651035729, 
    0.634913993812552, 0.279044929602191, 0.934065934065934, 
    0.37790609606967, 0, 0.693240588283048, 0.934065934065934, 
    0.0363111772636899, 0, 0.803610465372552, 0.869552014214865, 
    0, 0, 0.142236481581201, 0.1952432677023, 0.763063601642664, 
    0, 0.677802511853011, 0.764194736940585, 0, 0.849673265460568, 
    0.172149988072943, 0.585302129978335, 0, 0.567912026517875, 
    0.309218576172056, 0.254965154302071, 0, 0.121775620510825, 
    0.313645713574074, 0.484042322579438, 0.934065934065934, 
    0.466451348050809, 0, 0.83469280042559, 0, 0.884966655978525, 
    0.546398743058), A.pval = c(0, 0, 0.324870410050975, 0, 0.209108822673265, 
    0.163720264494612, 0.09974533635048, 0.104532055325668, 0.29429857036831, 
    0.516817201481687, 0, 0.342923327738624, 0, 0.213193307960778, 
    0.213271707392123, 0.0620964024214493, 0.80909090909091, 
    0.166726342223479, 0.80909090909091, 0.79742970975525, 0.80909090909091, 
    0.237017401022566, 0.80909090909091, 0.228366015104326, 0.643755041453696, 
    0, 0.385866448341247, 0.551704626593316, 0, 0.501686163616064, 
    0, 0.648847869172879, 0.156804519634643, 0.415564567821327, 
    0.115780239790685, 0.264624985516074, 0, 0.498453471169814, 
    0.198836156510245, 0.323838031958871, 0.628684774933257, 
    0.159798757047009, 0, 0.3865936346452, 0.100268064838965, 
    0.0401161805158001, 0, 0.306059246361509, 0.00466153634431854, 
    0.158230259664139, 0.619337599628234, 0, 0, 0.342534711560093, 
    0, 0, 0.214593831414468, 0.357771753273103, 0.792898076373203, 
    0.490098713768902, 0.0271041768096046, 0, 0.380486932551196, 
    0, 0.447951031427074, 0.466279418605815, 0.77163682819592, 
    0.576656222153961, 0.589724253546913, 0.80909090909091, 0.0731385609966513, 
    0.80909090909091, 0.0105141364690376, 0, 0.548329905539978
    ), guide.position = 1:75), .Names = c("A.area", "C.area", 
"G.area", "T.area", "Tot.area", "A.perc", "C.perc", "G.perc", 
"T.perc", "base.call", "index", "guide.seq", "T.pval", "C.pval", 
"G.pval", "A.pval", "guide.position"), row.names = 276:350, class = "data.frame")
```

*Report generated using EditR v1.0.8*
