# Supplementary material for: Mechanistic and genetic basis of single-strand templated repair at Cas12a-induced DNA breaks in Chlamydomonas reinhardtii
Source: Nat Commun. 2021 Nov 19;12:6751. doi: 10.1038/s41467-021-27004-1 (PMC8604939; doi:10.1038/s41467-021-27004-1)
Supplement: Supplementary file 22 — Source Data [file 41467_2021_27004_MOESM22_ESM.zip › Source Data/EditR analysis/EditR outputs/Antisense/rep2_ssODN_antisense_-32_-16_0.html]

EditR v1.0.8 report


# EditR v1.0.8 report

- Data QA
  - Filtering data
  - Percent noise peak area
  - Base information
- Predicted editing
  - Editing bar plot
  - Editing table plot
  - Table of editing results
- For use in R

## Data QA

### Filtering data

What the data looked like prefiltering:

and the post filtering signal / noise plot:

### Percent noise peak area

### Base information

Here’s information about the signal of each base, the critical percent value where any higher value would be called as significant, and Filliben’s correlation for how well the noise was modelled by the zero adjusted gamma distribution.

| Base | Average percent signal | Average peak area | Critical percent value | model mu | Fillibens correlation |
| --- | --- | --- | --- | --- | --- |
| A | 90.49740 | 394.3030 | 13.576317 | 3.874107 | 0.9963671 |
| C | 90.18907 | 393.5714 | 10.055470 | 3.047484 | 0.9887668 |
| G | 90.58671 | 405.3636 | 6.697685 | 2.405025 | 0.9786927 |
| T | 92.93041 | 441.5581 | 11.836423 | 3.730411 | 0.9969483 |

## Predicted editing

### Editing bar plot

### Editing table plot

### Table of editing results


Here’s the entire guide region

| Sanger position | Guide position | Guide sequence | Sanger base call | Focal base | Focal base peak area | p value |  |
| --- | --- | --- | --- | --- | --- | --- | --- |
| 292 | 1 | A | A | A | 91.15 | 2.220446e-16 | \* |
| 292 | 1 | A | A | C | 6.97 | 5.386652e-02 |  |
| 292 | 1 | A | A | G | 0.00 | 9.347826e-01 |  |
| 292 | 1 | A | A | T | 1.88 | 7.175329e-01 |  |
| 293 | 2 | A | A | A | 91.02 | 2.220446e-16 | \* |
| 293 | 2 | A | A | C | 5.83 | 9.783751e-02 |  |
| 293 | 2 | A | A | G | 1.21 | 7.511436e-01 |  |
| 293 | 2 | A | A | T | 1.94 | 7.050775e-01 |  |
| 294 | 3 | G | G | A | 4.56 | 2.634385e-01 |  |
| 294 | 3 | G | G | C | 5.41 | 1.206329e-01 |  |
| 294 | 3 | G | G | G | 83.76 | 0.000000e+00 | \* |
| 294 | 3 | G | G | T | 6.27 | 1.392408e-01 |  |
| 295 | 4 | A | A | A | 91.21 | 2.220446e-16 | \* |
| 295 | 4 | A | A | C | 0.89 | 8.026566e-01 |  |
| 295 | 4 | A | A | G | 1.79 | 5.731115e-01 |  |
| 295 | 4 | A | A | T | 6.11 | 1.491470e-01 |  |
| 296 | 5 | C | C | A | 8.55 | 6.555876e-02 |  |
| 296 | 5 | C | C | C | 87.17 | 0.000000e+00 | \* |
| 296 | 5 | C | C | G | 2.85 | 2.906267e-01 |  |
| 296 | 5 | C | C | T | 1.43 | 8.018905e-01 |  |
| 297 | 6 | T | T | A | 6.99 | 1.146947e-01 |  |
| 297 | 6 | T | T | C | 3.60 | 2.890879e-01 |  |
| 297 | 6 | T | T | G | 2.33 | 4.148732e-01 |  |
| 297 | 6 | T | T | T | 87.08 | 0.000000e+00 | \* |
| 298 | 7 | G | G | A | 4.97 | 2.297053e-01 |  |
| 298 | 7 | G | G | C | 1.88 | 5.904846e-01 |  |
| 298 | 7 | G | G | G | 92.34 | 0.000000e+00 | \* |
| 298 | 7 | G | G | T | 0.81 | 9.006035e-01 |  |
| 299 | 8 | G | G | A | 6.68 | 1.279818e-01 |  |
| 299 | 8 | G | G | C | 1.01 | 7.787866e-01 |  |
| 299 | 8 | G | G | G | 89.07 | 0.000000e+00 | \* |
| 299 | 8 | G | G | T | 3.24 | 4.687335e-01 |  |
| 300 | 9 | C | C | A | 7.67 | 9.024361e-02 |  |
| 300 | 9 | C | C | C | 84.32 | 0.000000e+00 | \* |
| 300 | 9 | C | C | G | 0.00 | 9.347826e-01 |  |
| 300 | 9 | C | C | T | 8.01 | 6.327906e-02 |  |
| 301 | 10 | C | C | A | 2.79 | 4.570294e-01 |  |
| 301 | 10 | C | C | C | 88.20 | 0.000000e+00 | \* |
| 301 | 10 | C | C | G | 3.65 | 1.577309e-01 |  |
| 301 | 10 | C | C | T | 5.36 | 2.050994e-01 |  |
| 302 | 11 | A | A | A | 70.93 | 7.835954e-13 | \* |
| 302 | 11 | A | A | C | 2.62 | 4.439311e-01 |  |
| 302 | 11 | A | A | G | 7.27 | 5.707455e-03 | \* |
| 302 | 11 | A | A | T | 19.19 | 2.267496e-04 | \* |
| 303 | 12 | G | G | A | 4.86 | 2.385609e-01 |  |
| 303 | 12 | G | G | C | 3.32 | 3.275041e-01 |  |
| 303 | 12 | G | G | G | 90.54 | 0.000000e+00 | \* |
| 303 | 12 | G | G | T | 1.28 | 8.276999e-01 |  |
| 304 | 13 | A | A | A | 92.72 | 1.110223e-16 | \* |
| 304 | 13 | A | A | C | 1.08 | 7.638875e-01 |  |
| 304 | 13 | A | A | G | 1.24 | 7.438155e-01 |  |
| 304 | 13 | A | A | T | 4.95 | 2.432079e-01 |  |
| 305 | 14 | C | C | A | 3.89 | 3.264480e-01 |  |
| 305 | 14 | C | C | C | 92.46 | 0.000000e+00 | \* |
| 305 | 14 | C | C | G | 0.00 | 9.347826e-01 |  |
| 305 | 14 | C | C | T | 3.65 | 4.044145e-01 |  |
| 306 | 15 | C | C | A | 8.75 | 6.097910e-02 |  |
| 306 | 15 | C | C | C | 85.25 | 0.000000e+00 | \* |
| 306 | 15 | C | C | G | 3.25 | 2.158034e-01 |  |
| 306 | 15 | C | C | T | 2.75 | 5.530813e-01 |  |
| 307 | 16 | G | G | A | 3.31 | 3.915640e-01 |  |
| 307 | 16 | G | G | C | 4.58 | 1.822408e-01 |  |
| 307 | 16 | G | G | G | 88.55 | 0.000000e+00 | \* |
| 307 | 16 | G | G | T | 3.56 | 4.175462e-01 |  |
| 308 | 17 | T | T | A | 0.00 | 8.288288e-01 |  |
| 308 | 17 | T | T | C | 3.55 | 2.964907e-01 |  |
| 308 | 17 | T | T | G | 2.48 | 3.754731e-01 |  |
| 308 | 17 | T | T | T | 93.97 | 0.000000e+00 | \* |
| 309 | 18 | G | G | A | 5.20 | 2.128158e-01 |  |
| 309 | 18 | G | G | C | 1.01 | 7.799076e-01 |  |
| 309 | 18 | G | G | G | 91.11 | 0.000000e+00 | \* |
| 309 | 18 | G | G | T | 2.68 | 5.649297e-01 |  |
| 310 | 19 | T | T | A | 0.27 | 8.141626e-01 |  |
| 310 | 19 | T | T | C | 3.46 | 3.086160e-01 |  |
| 310 | 19 | T | T | G | 3.46 | 1.836327e-01 |  |
| 310 | 19 | T | T | T | 92.82 | 0.000000e+00 | \* |
| 311 | 20 | T | T | A | 0.00 | 8.288288e-01 |  |
| 311 | 20 | T | T | C | 0.27 | 9.002336e-01 |  |
| 311 | 20 | T | T | G | 5.31 | 3.746159e-02 |  |
| 311 | 20 | T | T | T | 94.43 | 0.000000e+00 | \* |
| 312 | 21 | T | T | A | 0.25 | 8.156764e-01 |  |
| 312 | 21 | T | T | C | 1.99 | 5.685550e-01 |  |
| 312 | 21 | T | T | G | 3.47 | 1.812566e-01 |  |
| 312 | 21 | T | T | T | 94.29 | 0.000000e+00 | \* |
| 313 | 22 | G | G | A | 4.38 | 2.792624e-01 |  |
| 313 | 22 | G | G | C | 0.91 | 7.990381e-01 |  |
| 313 | 22 | G | G | G | 90.88 | 0.000000e+00 | \* |
| 313 | 22 | G | G | T | 3.83 | 3.779212e-01 |  |
| 314 | 23 | T | T | A | 0.00 | 8.288288e-01 |  |
| 314 | 23 | T | T | C | 0.00 | 9.135802e-01 |  |
| 314 | 23 | T | T | G | 1.52 | 6.587693e-01 |  |
| 314 | 23 | T | T | T | 98.48 | 0.000000e+00 | \* |
| 315 | 24 | G | G | A | 4.85 | 2.396328e-01 |  |
| 315 | 24 | G | G | C | 1.76 | 6.162300e-01 |  |
| 315 | 24 | G | G | G | 89.21 | 0.000000e+00 | \* |
| 315 | 24 | G | G | T | 4.19 | 3.303784e-01 |  |
| 316 | 25 | C | C | A | 2.68 | 4.716678e-01 |  |
| 316 | 25 | C | C | C | 83.11 | 0.000000e+00 | \* |
| 316 | 25 | C | C | G | 1.61 | 6.293987e-01 |  |
| 316 | 25 | C | C | T | 12.60 | 6.823952e-03 | \* |
| 317 | 26 | A | A | A | 85.48 | 1.887379e-15 | \* |
| 317 | 26 | A | A | C | 3.30 | 3.310887e-01 |  |
| 317 | 26 | A | A | G | 4.62 | 6.934022e-02 |  |
| 317 | 26 | A | A | T | 6.60 | 1.202432e-01 |  |
| 318 | 27 | C | C | A | 1.34 | 6.708394e-01 |  |
| 318 | 27 | C | C | C | 89.01 | 0.000000e+00 | \* |
| 318 | 27 | C | C | G | 7.77 | 3.436026e-03 | \* |
| 318 | 27 | C | C | T | 1.88 | 7.175329e-01 |  |
| 319 | 28 | T | T | A | 2.58 | 4.849756e-01 |  |
| 319 | 28 | T | T | C | 2.58 | 4.498592e-01 |  |
| 319 | 28 | T | T | G | 0.26 | 9.304831e-01 |  |
| 319 | 28 | T | T | T | 94.57 | 0.000000e+00 | \* |
| 320 | 29 | A | A | A | 91.55 | 2.220446e-16 | \* |
| 320 | 29 | A | A | C | 5.99 | 8.969196e-02 |  |
| 320 | 29 | A | A | G | 0.82 | 8.549522e-01 |  |
| 320 | 29 | A | A | T | 1.63 | 7.633501e-01 |  |
| 321 | 30 | C | C | A | 3.47 | 3.731187e-01 |  |
| 321 | 30 | C | C | C | 87.87 | 0.000000e+00 | \* |
| 321 | 30 | C | C | G | 2.72 | 3.182844e-01 |  |
| 321 | 30 | C | C | T | 5.94 | 1.605241e-01 |  |
| 322 | 31 | A | A | A | 90.73 | 2.220446e-16 | \* |
| 322 | 31 | A | A | C | 3.31 | 3.294845e-01 |  |
| 322 | 31 | A | A | G | 5.96 | 2.032502e-02 |  |
| 322 | 31 | A | A | T | 0.00 | 9.574468e-01 |  |
| 323 | 32 | C | C | A | 1.42 | 6.584971e-01 |  |
| 323 | 32 | C | C | C | 93.75 | 0.000000e+00 | \* |
| 323 | 32 | C | C | G | 1.70 | 5.992597e-01 |  |
| 323 | 32 | C | C | T | 3.12 | 4.876666e-01 |  |
| 324 | 33 | G | G | A | 5.56 | 1.887957e-01 |  |
| 324 | 33 | G | G | C | 5.21 | 1.336989e-01 |  |
| 324 | 33 | G | G | G | 84.72 | 0.000000e+00 | \* |
| 324 | 33 | G | G | T | 4.51 | 2.904026e-01 |  |
| 325 | 34 | G | G | A | 3.93 | 3.225131e-01 |  |
| 325 | 34 | G | G | C | 4.67 | 1.745876e-01 |  |
| 325 | 34 | G | G | G | 91.40 | 0.000000e+00 | \* |
| 325 | 34 | G | G | T | 0.00 | 9.574468e-01 |  |
| 326 | 35 | G | G | A | 0.99 | 7.238794e-01 |  |
| 326 | 35 | G | G | C | 2.64 | 4.395631e-01 |  |
| 326 | 35 | G | G | G | 96.37 | 0.000000e+00 | \* |
| 326 | 35 | G | G | T | 0.00 | 9.574468e-01 |  |
| 327 | 36 | C | C | A | 6.14 | 1.546385e-01 |  |
| 327 | 36 | C | C | C | 92.06 | 0.000000e+00 | \* |
| 327 | 36 | C | C | G | 0.00 | 9.347826e-01 |  |
| 327 | 36 | C | C | T | 1.81 | 7.311993e-01 |  |
| 328 | 37 | A | A | A | 89.84 | 2.220446e-16 | \* |
| 328 | 37 | A | A | C | 3.17 | 3.499912e-01 |  |
| 328 | 37 | A | A | G | 2.22 | 4.444628e-01 |  |
| 328 | 37 | A | A | T | 4.76 | 2.629275e-01 |  |
| 329 | 38 | C | C | A | 2.33 | 5.207418e-01 |  |
| 329 | 38 | C | C | C | 95.10 | 0.000000e+00 | \* |
| 329 | 38 | C | C | G | 0.00 | 9.347826e-01 |  |
| 329 | 38 | C | C | T | 2.56 | 5.870305e-01 |  |
| 330 | 39 | C | C | A | 6.38 | 1.419911e-01 |  |
| 330 | 39 | C | C | C | 83.22 | 0.000000e+00 | \* |
| 330 | 39 | C | C | G | 5.67 | 2.661859e-02 |  |
| 330 | 39 | C | C | T | 4.73 | 2.665372e-01 |  |
| 331 | 40 | C | C | A | 3.80 | 3.364387e-01 |  |
| 331 | 40 | C | C | C | 92.66 | 0.000000e+00 | \* |
| 331 | 40 | C | C | G | 0.76 | 8.672727e-01 |  |
| 331 | 40 | C | C | T | 2.78 | 5.468266e-01 |  |
| 332 | 41 | T | T | A | 0.79 | 7.529283e-01 |  |
| 332 | 41 | T | T | C | 6.56 | 6.680689e-02 |  |
| 332 | 41 | T | T | G | 1.84 | 5.579874e-01 |  |
| 332 | 41 | T | T | T | 90.81 | 0.000000e+00 | \* |
| 333 | 42 | G | G | A | 4.89 | 2.359595e-01 |  |
| 333 | 42 | G | G | C | 3.91 | 2.503169e-01 |  |
| 333 | 42 | G | G | G | 90.61 | 0.000000e+00 | \* |
| 333 | 42 | G | G | T | 0.59 | 9.264784e-01 |  |
| 334 | 43 | A | A | A | 84.60 | 2.664535e-15 | \* |
| 334 | 43 | A | A | C | 1.42 | 6.907405e-01 |  |
| 334 | 43 | A | A | G | 1.42 | 6.879359e-01 |  |
| 334 | 43 | A | A | T | 12.56 | 6.966965e-03 | \* |
| 335 | 44 | C | C | A | 3.24 | 3.993337e-01 |  |
| 335 | 44 | C | C | C | 91.62 | 0.000000e+00 | \* |
| 335 | 44 | C | C | G | 4.59 | 7.093437e-02 |  |
| 335 | 44 | C | C | T | 0.54 | 9.311096e-01 |  |
| 336 | 45 | C | C | A | 8.78 | 6.027455e-02 |  |
| 336 | 45 | C | C | C | 84.14 | 0.000000e+00 | \* |
| 336 | 45 | C | C | G | 4.25 | 9.567632e-02 |  |
| 336 | 45 | C | C | T | 2.83 | 5.382503e-01 |  |
| 337 | 46 | G | G | A | 2.80 | 4.551615e-01 |  |
| 337 | 46 | G | G | C | 6.23 | 7.939405e-02 |  |
| 337 | 46 | G | G | G | 90.97 | 0.000000e+00 | \* |
| 337 | 46 | G | G | T | 0.00 | 9.574468e-01 |  |
| 338 | 47 | A | A | A | 93.61 | 1.110223e-16 | \* |
| 338 | 47 | A | A | C | 0.91 | 7.990381e-01 |  |
| 338 | 47 | A | A | G | 2.19 | 4.535532e-01 |  |
| 338 | 47 | A | A | T | 3.28 | 4.612485e-01 |  |
| 339 | 48 | C | C | A | 5.22 | 2.116708e-01 |  |
| 339 | 48 | C | C | C | 89.28 | 0.000000e+00 | \* |
| 339 | 48 | C | C | G | 0.87 | 8.431250e-01 |  |
| 339 | 48 | C | C | T | 4.64 | 2.764079e-01 |  |
| 340 | 49 | G | G | A | 8.30 | 7.188968e-02 |  |
| 340 | 49 | G | G | C | 1.31 | 7.152170e-01 |  |
| 340 | 49 | G | G | G | 84.72 | 0.000000e+00 | \* |
| 340 | 49 | G | G | T | 5.68 | 1.797491e-01 |  |
| 341 | 50 | G | G | A | 3.54 | 3.641256e-01 |  |
| 341 | 50 | G | G | C | 1.77 | 6.140561e-01 |  |
| 341 | 50 | G | G | G | 92.41 | 0.000000e+00 | \* |
| 341 | 50 | G | G | T | 2.28 | 6.406542e-01 |  |
| 342 | 51 | C | C | A | 6.25 | 1.487096e-01 |  |
| 342 | 51 | C | C | C | 85.83 | 0.000000e+00 | \* |
| 342 | 51 | C | C | G | 2.50 | 3.710340e-01 |  |
| 342 | 51 | C | C | T | 5.42 | 2.006814e-01 |  |
| 343 | 52 | A | A | A | 92.00 | 1.110223e-16 | \* |
| 343 | 52 | A | A | C | 2.50 | 4.655160e-01 |  |
| 343 | 52 | A | A | G | 3.00 | 2.605449e-01 |  |
| 343 | 52 | A | A | T | 2.50 | 5.989301e-01 |  |
| 344 | 53 | A | A | A | 96.26 | 0.000000e+00 | \* |
| 344 | 53 | A | A | C | 1.72 | 6.244741e-01 |  |
| 344 | 53 | A | A | G | 1.15 | 7.697669e-01 |  |
| 344 | 53 | A | A | T | 0.86 | 8.930980e-01 |  |
| 345 | 54 | G | G | A | 4.81 | 2.421067e-01 |  |
| 345 | 54 | G | G | C | 0.00 | 9.135802e-01 |  |
| 345 | 54 | G | G | G | 93.33 | 0.000000e+00 | \* |
| 345 | 54 | G | G | T | 1.85 | 7.222758e-01 |  |
| 346 | 55 | A | A | A | 94.51 | 0.000000e+00 | \* |
| 346 | 55 | A | A | C | 0.00 | 9.135802e-01 |  |
| 346 | 55 | A | A | G | 0.82 | 8.534621e-01 |  |
| 346 | 55 | A | A | T | 4.67 | 2.728108e-01 |  |
| 347 | 56 | A | A | A | 93.62 | 1.110223e-16 | \* |
| 347 | 56 | A | A | C | 0.00 | 9.135802e-01 |  |
| 347 | 56 | A | A | G | 0.87 | 8.431250e-01 |  |
| 347 | 56 | A | A | T | 5.51 | 1.931638e-01 |  |
| 348 | 57 | G | G | A | 5.86 | 1.703833e-01 |  |
| 348 | 57 | G | G | C | 0.90 | 8.013266e-01 |  |
| 348 | 57 | G | G | G | 90.99 | 0.000000e+00 | \* |
| 348 | 57 | G | G | T | 2.25 | 6.456439e-01 |  |
| 349 | 58 | T | T | A | 3.02 | 4.270255e-01 |  |
| 349 | 58 | T | T | C | 0.34 | 8.930553e-01 |  |
| 349 | 58 | T | T | G | 3.69 | 1.523169e-01 |  |
| 349 | 58 | T | T | T | 92.95 | 0.000000e+00 | \* |
| 350 | 59 | T | T | A | 0.95 | 7.302866e-01 |  |
| 350 | 59 | T | T | C | 1.26 | 7.257123e-01 |  |
| 350 | 59 | T | T | G | 0.00 | 9.347826e-01 |  |
| 350 | 59 | T | T | T | 97.79 | 0.000000e+00 | \* |
| 351 | 60 | C | C | A | 0.00 | 8.288288e-01 |  |
| 351 | 60 | C | C | C | 93.69 | 0.000000e+00 | \* |
| 351 | 60 | C | C | G | 1.66 | 6.128780e-01 |  |
| 351 | 60 | C | C | T | 4.65 | 2.749179e-01 |  |
| 352 | 61 | G | G | A | 5.45 | 1.958524e-01 |  |
| 352 | 61 | G | G | C | 2.72 | 4.245904e-01 |  |
| 352 | 61 | G | G | G | 87.16 | 0.000000e+00 | \* |
| 352 | 61 | G | G | T | 4.67 | 2.729280e-01 |  |
| 353 | 62 | A | A | A | 91.92 | 1.110223e-16 | \* |
| 353 | 62 | A | A | C | 1.90 | 5.865329e-01 |  |
| 353 | 62 | A | A | G | 1.43 | 6.868864e-01 |  |
| 353 | 62 | A | A | T | 4.75 | 2.641319e-01 |  |
| 354 | 63 | C | C | A | 2.45 | 5.034063e-01 |  |
| 354 | 63 | C | C | C | 91.28 | 0.000000e+00 | \* |
| 354 | 63 | C | C | G | 2.45 | 3.830514e-01 |  |
| 354 | 63 | C | C | T | 3.81 | 3.803917e-01 |  |
| 355 | 64 | A | A | A | 97.38 | 0.000000e+00 | \* |
| 355 | 64 | A | A | C | 0.00 | 9.135802e-01 |  |
| 355 | 64 | A | A | G | 2.62 | 3.418979e-01 |  |
| 355 | 64 | A | A | T | 0.00 | 9.574468e-01 |  |
| 356 | 65 | G | G | A | 0.54 | 7.846407e-01 |  |
| 356 | 65 | G | G | C | 4.09 | 2.307684e-01 |  |
| 356 | 65 | G | G | G | 94.82 | 0.000000e+00 | \* |
| 356 | 65 | G | G | T | 0.54 | 9.306840e-01 |  |
| 357 | 66 | C | C | A | 3.87 | 3.284899e-01 |  |
| 357 | 66 | C | C | C | 93.66 | 0.000000e+00 | \* |
| 357 | 66 | C | C | G | 0.70 | 8.781556e-01 |  |
| 357 | 66 | C | C | T | 1.76 | 7.396561e-01 |  |
| 358 | 67 | T | T | A | 1.00 | 7.231535e-01 |  |
| 358 | 67 | T | T | C | 4.23 | 2.157821e-01 |  |
| 358 | 67 | T | T | G | 1.74 | 5.877687e-01 |  |
| 358 | 67 | T | T | T | 93.03 | 0.000000e+00 | \* |
| 359 | 68 | C | C | A | 0.59 | 7.786444e-01 |  |
| 359 | 68 | C | C | C | 91.69 | 0.000000e+00 | \* |
| 359 | 68 | C | C | G | 2.37 | 4.033584e-01 |  |
| 359 | 68 | C | C | T | 5.34 | 2.071344e-01 |  |
| 360 | 69 | C | C | A | 0.00 | 8.288288e-01 |  |
| 360 | 69 | C | C | C | 92.34 | 0.000000e+00 | \* |
| 360 | 69 | C | C | G | 0.24 | 9.313022e-01 |  |
| 360 | 69 | C | C | T | 7.42 | 8.330440e-02 |  |
| 361 | 70 | C | C | A | 0.00 | 8.288288e-01 |  |
| 361 | 70 | C | C | C | 96.90 | 0.000000e+00 | \* |
| 361 | 70 | C | C | G | 1.38 | 7.010910e-01 |  |
| 361 | 70 | C | C | T | 1.72 | 7.465559e-01 |  |
| 362 | 71 | G | G | A | 0.77 | 7.554385e-01 |  |
| 362 | 71 | G | G | C | 7.31 | 4.502754e-02 |  |
| 362 | 71 | G | G | G | 88.85 | 0.000000e+00 | \* |
| 362 | 71 | G | G | T | 3.08 | 4.957970e-01 |  |
| 363 | 72 | C | C | A | 0.00 | 8.288288e-01 |  |
| 363 | 72 | C | C | C | 97.17 | 0.000000e+00 | \* |
| 363 | 72 | C | C | G | 0.71 | 8.776846e-01 |  |
| 363 | 72 | C | C | T | 2.12 | 6.708795e-01 |  |
| 364 | 73 | G | G | A | 3.94 | 3.219196e-01 |  |
| 364 | 73 | G | G | C | 9.06 | 1.743087e-02 |  |
| 364 | 73 | G | G | G | 86.22 | 0.000000e+00 | \* |
| 364 | 73 | G | G | T | 0.79 | 9.030930e-01 |  |
| 365 | 74 | A | A | A | 91.51 | 2.220446e-16 | \* |
| 365 | 74 | A | A | C | 3.98 | 2.428376e-01 |  |
| 365 | 74 | A | A | G | 0.80 | 8.596677e-01 |  |
| 365 | 74 | A | A | T | 3.71 | 3.949905e-01 |  |
| 366 | 75 | C | C | A | 1.09 | 7.085836e-01 |  |
| 366 | 75 | C | C | C | 92.35 | 0.000000e+00 | \* |
| 366 | 75 | C | C | G | 1.37 | 7.051538e-01 |  |
| 366 | 75 | C | C | T | 5.19 | 2.205038e-01 |  |

## For use in R

If you want to work with the results in R, here is output that you can copy and paste in your terminal to get:

The base information:

```
structure(list(focal.base = c("A", "C", "G", "T"), avg.percsignal = c(90.4973989920218, 
90.1890724508796, 90.5867072918634, 92.9304108683028), avg.areasignal = c(394.30303030303, 
393.571428571429, 405.363636363636, 441.558139534884), crit.perc.area = c(13.576316864596, 
10.0554696994845, 6.69768447609175, 11.8364234491836), mu = c(3.87410731823635, 
3.04748393425821, 2.40502535425283, 3.73041076184648), fillibens = c(0.996367133683335, 
0.988766816186451, 0.978692723606411, 0.996948333774665)), .Names = c("focal.base", 
"avg.percsignal", "avg.areasignal", "crit.perc.area", "mu", "fillibens"
), row.names = c(NA, -4L), class = "data.frame")
```

the data.frame that contains information on the guide region:

```
structure(list(A.area = c(340, 375, 16, 612, 36, 33, 37, 33, 
22, 13, 244, 19, 599, 16, 35, 13, 0, 31, 1, 0, 1, 24, 0, 22, 
10, 259, 5, 10, 336, 14, 274, 5, 16, 16, 3, 17, 283, 10, 27, 
15, 3, 25, 357, 12, 31, 9, 513, 18, 19, 14, 15, 184, 335, 13, 
344, 323, 13, 9, 3, 0, 14, 387, 9, 223, 2, 11, 4, 2, 0, 0, 2, 
0, 10, 345, 4), C.area = c(26, 24, 19, 6, 367, 17, 14, 5, 242, 
411, 9, 13, 7, 380, 341, 18, 10, 6, 13, 1, 8, 5, 0, 8, 310, 10, 
332, 10, 22, 355, 10, 330, 15, 19, 8, 255, 10, 408, 352, 366, 
25, 20, 6, 339, 297, 20, 5, 308, 3, 7, 206, 5, 6, 0, 0, 0, 2, 
1, 4, 282, 7, 8, 335, 0, 15, 266, 17, 309, 386, 281, 19, 275, 
23, 15, 338), G.area = c(0, 5, 294, 12, 12, 11, 687, 440, 0, 
17, 25, 354, 8, 0, 13, 348, 7, 543, 13, 20, 14, 498, 5, 405, 
6, 14, 29, 1, 3, 11, 18, 6, 244, 372, 292, 0, 7, 0, 24, 3, 7, 
463, 6, 17, 15, 292, 12, 3, 194, 365, 6, 6, 4, 252, 3, 3, 202, 
11, 0, 5, 224, 6, 9, 6, 348, 2, 7, 8, 1, 4, 231, 2, 219, 3, 5
), T.area = c(7, 8, 22, 41, 6, 411, 6, 16, 23, 25, 66, 5, 32, 
15, 11, 14, 265, 16, 349, 356, 380, 21, 325, 19, 47, 20, 7, 366, 
6, 24, 0, 11, 13, 0, 0, 5, 15, 11, 20, 11, 346, 3, 53, 2, 10, 
0, 18, 16, 13, 9, 13, 5, 3, 5, 17, 19, 5, 277, 310, 14, 12, 20, 
14, 0, 2, 5, 374, 18, 31, 5, 8, 6, 2, 14, 19), Tot.area = c(373, 
412, 351, 671, 421, 472, 744, 494, 287, 466, 344, 391, 646, 411, 
400, 393, 282, 596, 376, 377, 403, 548, 330, 454, 373, 303, 373, 
387, 367, 404, 302, 352, 288, 407, 303, 277, 315, 429, 423, 395, 
381, 511, 422, 370, 353, 321, 548, 345, 229, 395, 240, 200, 348, 
270, 364, 345, 222, 298, 317, 301, 257, 421, 367, 229, 367, 284, 
402, 337, 418, 290, 260, 283, 254, 377, 366), A.perc = c(91.1528150134048, 
91.0194174757282, 4.55840455840456, 91.2071535022355, 8.55106888361045, 
6.99152542372881, 4.97311827956989, 6.68016194331984, 7.66550522648084, 
2.78969957081545, 70.9302325581395, 4.85933503836317, 92.7244582043344, 
3.89294403892944, 8.75, 3.30788804071247, 0, 5.2013422818792, 
0.265957446808511, 0, 0.248138957816377, 4.37956204379562, 0, 
4.84581497797357, 2.68096514745308, 85.4785478547855, 1.34048257372654, 
2.58397932816537, 91.5531335149864, 3.46534653465347, 90.728476821192, 
1.42045454545455, 5.55555555555556, 3.93120393120393, 0.99009900990099, 
6.13718411552347, 89.8412698412698, 2.33100233100233, 6.38297872340426, 
3.79746835443038, 0.78740157480315, 4.89236790606654, 84.5971563981043, 
3.24324324324324, 8.78186968838527, 2.80373831775701, 93.6131386861314, 
5.21739130434783, 8.29694323144105, 3.54430379746835, 6.25, 92, 
96.2643678160919, 4.81481481481481, 94.5054945054945, 93.6231884057971, 
5.85585585585586, 3.02013422818792, 0.946372239747634, 0, 5.44747081712062, 
91.9239904988124, 2.45231607629428, 97.3799126637555, 0.544959128065395, 
3.87323943661972, 0.995024875621891, 0.593471810089021, 0, 0, 
0.769230769230769, 0, 3.93700787401575, 91.5119363395225, 1.09289617486339
), C.perc = c(6.97050938337802, 5.8252427184466, 5.41310541310541, 
0.894187779433681, 87.1733966745843, 3.60169491525424, 1.88172043010753, 
1.01214574898785, 84.3205574912892, 88.1974248927039, 2.61627906976744, 
3.32480818414322, 1.08359133126935, 92.4574209245742, 85.25, 
4.58015267175572, 3.54609929078014, 1.00671140939597, 3.45744680851064, 
0.26525198938992, 1.98511166253102, 0.912408759124088, 0, 1.76211453744493, 
83.1099195710456, 3.3003300330033, 89.0080428954424, 2.58397932816537, 
5.99455040871935, 87.8712871287129, 3.3112582781457, 93.75, 5.20833333333333, 
4.66830466830467, 2.64026402640264, 92.057761732852, 3.17460317460317, 
95.1048951048951, 83.2151300236407, 92.6582278481013, 6.56167979002625, 
3.91389432485323, 1.4218009478673, 91.6216216216216, 84.1359773371105, 
6.23052959501558, 0.912408759124088, 89.2753623188406, 1.31004366812227, 
1.77215189873418, 85.8333333333333, 2.5, 1.72413793103448, 0, 
0, 0, 0.900900900900901, 0.335570469798658, 1.26182965299685, 
93.687707641196, 2.72373540856031, 1.90023752969121, 91.2806539509537, 
0, 4.08719346049046, 93.6619718309859, 4.22885572139303, 91.6913946587537, 
92.3444976076555, 96.8965517241379, 7.30769230769231, 97.1731448763251, 
9.05511811023622, 3.97877984084881, 92.3497267759563), G.perc = c(0, 
1.21359223300971, 83.7606837606838, 1.78837555886736, 2.85035629453682, 
2.33050847457627, 92.3387096774194, 89.0688259109312, 0, 3.6480686695279, 
7.26744186046512, 90.537084398977, 1.23839009287926, 0, 3.25, 
88.5496183206107, 2.4822695035461, 91.1073825503356, 3.45744680851064, 
5.30503978779841, 3.47394540942928, 90.8759124087591, 1.51515151515152, 
89.2070484581498, 1.60857908847185, 4.62046204620462, 7.77479892761394, 
0.258397932816537, 0.817438692098093, 2.72277227722772, 5.96026490066225, 
1.70454545454545, 84.7222222222222, 91.4004914004914, 96.3696369636964, 
0, 2.22222222222222, 0, 5.67375886524823, 0.759493670886076, 
1.83727034120735, 90.6066536203522, 1.4218009478673, 4.59459459459459, 
4.24929178470255, 90.9657320872274, 2.18978102189781, 0.869565217391304, 
84.7161572052402, 92.4050632911392, 2.5, 3, 1.14942528735632, 
93.3333333333333, 0.824175824175824, 0.869565217391304, 90.990990990991, 
3.69127516778523, 0, 1.66112956810631, 87.15953307393, 1.42517814726841, 
2.45231607629428, 2.62008733624454, 94.8228882833787, 0.704225352112676, 
1.74129353233831, 2.37388724035608, 0.239234449760766, 1.37931034482759, 
88.8461538461538, 0.706713780918728, 86.2204724409449, 0.795755968169761, 
1.36612021857923), T.perc = c(1.87667560321716, 1.94174757281553, 
6.26780626780627, 6.11028315946349, 1.42517814726841, 87.0762711864407, 
0.806451612903226, 3.23886639676113, 8.01393728222997, 5.36480686695279, 
19.1860465116279, 1.27877237851662, 4.95356037151703, 3.64963503649635, 
2.75, 3.56234096692112, 93.9716312056738, 2.68456375838926, 92.8191489361702, 
94.4297082228117, 94.2928039702233, 3.83211678832117, 98.4848484848485, 
4.18502202643172, 12.6005361930295, 6.6006600660066, 1.87667560321716, 
94.5736434108527, 1.63487738419619, 5.94059405940594, 0, 3.125, 
4.51388888888889, 0, 0, 1.80505415162455, 4.76190476190476, 2.56410256410256, 
4.72813238770686, 2.78481012658228, 90.8136482939633, 0.587084148727984, 
12.5592417061611, 0.540540540540541, 2.8328611898017, 0, 3.28467153284672, 
4.63768115942029, 5.67685589519651, 2.27848101265823, 5.41666666666667, 
2.5, 0.862068965517241, 1.85185185185185, 4.67032967032967, 5.50724637681159, 
2.25225225225225, 92.9530201342282, 97.7917981072555, 4.65116279069767, 
4.66926070038911, 4.75059382422803, 3.81471389645777, 0, 0.544959128065395, 
1.76056338028169, 93.0348258706468, 5.34124629080119, 7.41626794258373, 
1.72413793103448, 3.07692307692308, 2.12014134275618, 0.78740157480315, 
3.71352785145889, 5.19125683060109), base.call = c("A", "A", 
"G", "A", "C", "T", "G", "G", "C", "C", "A", "G", "A", "C", "C", 
"G", "T", "G", "T", "T", "T", "G", "T", "G", "C", "A", "C", "T", 
"A", "C", "A", "C", "G", "G", "G", "C", "A", "C", "C", "C", "T", 
"G", "A", "C", "C", "G", "A", "C", "G", "G", "C", "A", "A", "G", 
"A", "A", "G", "T", "T", "C", "G", "A", "C", "A", "G", "C", "T", 
"C", "C", "C", "G", "C", "G", "A", "C"), index = 292:366, guide.seq = c("A", 
"A", "G", "A", "C", "T", "G", "G", "C", "C", "A", "G", "A", "C", 
"C", "G", "T", "G", "T", "T", "T", "G", "T", "G", "C", "A", "C", 
"T", "A", "C", "A", "C", "G", "G", "G", "C", "A", "C", "C", "C", 
"T", "G", "A", "C", "C", "G", "A", "C", "G", "G", "C", "A", "A", 
"G", "A", "A", "G", "T", "T", "C", "G", "A", "C", "A", "G", "C", 
"T", "C", "C", "C", "G", "C", "G", "A", "C"), T.pval = c(0.717532938389744, 
0.70507751557245, 0.139240751009133, 0.149147035275646, 0.80189051875779, 
0, 0.900603522511326, 0.46873351303241, 0.0632790644203617, 0.205099431263158, 
0.000226749607318499, 0.827699901085038, 0.243207886978083, 0.404414518596138, 
0.553081266783288, 0.417546179375141, 0, 0.56492969341438, 0, 
0, 0, 0.377921181073791, 0, 0.330378429626412, 0.00682395219546872, 
0.120243233838026, 0.717532938389744, 0, 0.763350051167198, 0.160524077191955, 
0.957446808510052, 0.487666572328715, 0.290402649269638, 0.957446808510052, 
0.957446808510052, 0.731199340877997, 0.262927505149035, 0.587030477488835, 
0.266537235702979, 0.546826597628741, 0, 0.92647841995953, 0.0069669650989832, 
0.931109619437256, 0.538250259053242, 0.957446808510052, 0.461248492141857, 
0.27640793775048, 0.179749126330087, 0.640654240606968, 0.200681437528657, 
0.598930099850582, 0.893097960005009, 0.722275846291558, 0.272810834008065, 
0.1931637819479, 0.64564391384391, 0, 0, 0.274917865521196, 0.27292799468832, 
0.264131907300857, 0.380391664006516, 0.957446808510052, 0.93068403215179, 
0.73965607722118, 0, 0.207134370724666, 0.0833044036496674, 0.746555921779484, 
0.495797000045584, 0.670879482360611, 0.903093014863853, 0.394990502910489, 
0.220503791393486), C.pval = c(0.0538665237546145, 0.0978375117388164, 
0.120632857730279, 0.802656609008745, 0, 0.289087874542327, 0.590484561787426, 
0.778786613519354, 0, 0, 0.443931079808464, 0.32750407615819, 
0.763887462954614, 0, 0, 0.18224075651477, 0.296490717013466, 
0.779907637024615, 0.308616002283603, 0.90023360242093, 0.568555030910117, 
0.799038107426795, 0.913580246873723, 0.616230024433444, 0, 0.33108873310711, 
0, 0.449859222174251, 0.0896919578570309, 0, 0.329484536850049, 
0, 0.133698857037143, 0.174587610675231, 0.439563071654581, 0, 
0.349991154111421, 0, 0, 0, 0.0668068878254974, 0.250316880760481, 
0.690740492476696, 0, 0, 0.0793940487899866, 0.799038107426795, 
0, 0.715216963813185, 0.614056129906429, 0, 0.46551599138378, 
0.624474118611804, 0.913580246873723, 0.913580246873723, 0.913580246873723, 
0.801326646517095, 0.89305525852345, 0.725712255500989, 0, 0.424590364317163, 
0.586532892941607, 0, 0.913580246873723, 0.230768395721291, 0, 
0.215782073141641, 0, 0, 0, 0.0450275386576604, 0, 0.0174308712219137, 
0.242837591442051, 0), G.pval = c(0.934782608695653, 0.751143573999971, 
0, 0.573111523510233, 0.290626663004889, 0.414873173301787, 0, 
0, 0.934782608695653, 0.157730948654971, 0.00570745450940224, 
0, 0.743815463141862, 0.934782608695653, 0.215803403834963, 0, 
0.375473062562552, 0, 0.183632744101332, 0.0374615884001638, 
0.18125664649476, 0, 0.658769255350254, 0, 0.629398683703748, 
0.0693402200990538, 0.00343602644251029, 0.930483079280482, 0.854952181212696, 
0.31828442088973, 0.0203250196583015, 0.599259664940277, 0, 0, 
0, 0.934782608695653, 0.444462762075548, 0.934782608695653, 0.026618593816057, 
0.867272735617172, 0.5579873923306, 0, 0.687935901712474, 0.070934368005926, 
0.0956763236920561, 0, 0.453553178419058, 0.843125011062512, 
0, 0, 0.371034027497039, 0.260544874503933, 0.76976690102946, 
0, 0.853462072153929, 0.843125011062512, 0, 0.152316896414166, 
0.934782608695653, 0.612878001120429, 0, 0.6868864042341, 0.383051360123637, 
0.34189787997563, 0, 0.878155603965737, 0.587768698052432, 0.403358444047857, 
0.931302214926789, 0.701090960222827, 0, 0.877684601161325, 0, 
0.859667705995091, 0.70515384614784), A.pval = c(2.22044604925031e-16, 
2.22044604925031e-16, 0.26343853020707, 2.22044604925031e-16, 
0.0655587629121066, 0.114694686018623, 0.229705327602357, 0.127981825682187, 
0.0902436142904235, 0.457029390952657, 7.83595410780435e-13, 
0.238560851214331, 1.11022302462516e-16, 0.326448045033312, 0.0609791027813213, 
0.391563998711092, 0.828828828828829, 0.212815752121767, 0.814162582646996, 
0.828828828828829, 0.815676424321011, 0.279262403420202, 0.828828828828829, 
0.239632808236428, 0.471667843050086, 1.88737914186277e-15, 0.670839405106437, 
0.484975623649441, 2.22044604925031e-16, 0.373118717044391, 2.22044604925031e-16, 
0.658497114491855, 0.188795662267996, 0.322513072155328, 0.723879372027101, 
0.154638490317825, 2.22044604925031e-16, 0.520741823151448, 0.14199114901278, 
0.336438731664504, 0.752928266521351, 0.235959506100078, 2.66453525910038e-15, 
0.399333742461948, 0.0602745500278126, 0.455161534814905, 1.11022302462516e-16, 
0.211670782478075, 0.0718896808606704, 0.364125555562675, 0.148709626640046, 
1.11022302462516e-16, 0, 0.242106678108578, 0, 1.11022302462516e-16, 
0.170383296639648, 0.427025450081467, 0.730286648670399, 0.828828828828829, 
0.195852369213326, 1.11022302462516e-16, 0.503406332804301, 0, 
0.784640651962559, 0.328489908013191, 0.723153540505563, 0.778644354624153, 
0.828828828828829, 0.828828828828829, 0.755438515565501, 0.828828828828829, 
0.321919558137974, 2.22044604925031e-16, 0.708583553680128), 
    guide.position = 1:75), .Names = c("A.area", "C.area", "G.area", 
"T.area", "Tot.area", "A.perc", "C.perc", "G.perc", "T.perc", 
"base.call", "index", "guide.seq", "T.pval", "C.pval", "G.pval", 
"A.pval", "guide.position"), row.names = 292:366, class = "data.frame")
```

*Report generated using EditR v1.0.8*
