# Supplementary material for: Mechanistic and genetic basis of single-strand templated repair at Cas12a-induced DNA breaks in Chlamydomonas reinhardtii
Source: Nat Commun. 2021 Nov 19;12:6751. doi: 10.1038/s41467-021-27004-1 (PMC8604939; doi:10.1038/s41467-021-27004-1)
Supplement: Supplementary file 22 — Source Data [file 41467_2021_27004_MOESM22_ESM.zip › Source Data/EditR analysis/EditR outputs/Antisense/rep2_ssODN_antisense_-32_-16_0_16_32.html]

EditR v1.0.8 report


# EditR v1.0.8 report

- Data QA
  - Filtering data
  - Percent noise peak area
  - Base information
- Predicted editing
  - Editing bar plot
  - Editing table plot
  - Table of editing results
- For use in R

## Data QA

### Filtering data

What the data looked like prefiltering:

and the post filtering signal / noise plot:

### Percent noise peak area

### Base information

Here’s information about the signal of each base, the critical percent value where any higher value would be called as significant, and Filliben’s correlation for how well the noise was modelled by the zero adjusted gamma distribution.

| Base | Average percent signal | Average peak area | Critical percent value | model mu | Fillibens correlation |
| --- | --- | --- | --- | --- | --- |
| A | 92.32650 | 337.1471 | 10.147514 | 3.152453 | 0.9971947 |
| C | 93.84767 | 355.7612 | 4.780183 | 1.855167 | 0.9901730 |
| G | 93.47487 | 335.5439 | 4.181917 | 1.584319 | 0.9841633 |
| T | 95.03191 | 413.7674 | 6.870696 | 2.305276 | 0.9931063 |

## Predicted editing

### Editing bar plot

### Editing table plot

### Table of editing results


Here’s the entire guide region

| Sanger position | Guide position | Guide sequence | Sanger base call | Focal base | Focal base peak area | p value |  |
| --- | --- | --- | --- | --- | --- | --- | --- |
| 277 | 1 | A | A | A | 93.35 | 0.000000e+00 | \* |
| 277 | 1 | A | A | C | 2.72 | 1.576506e-01 |  |
| 277 | 1 | A | A | G | 0.30 | 9.205641e-01 |  |
| 277 | 1 | A | A | T | 3.63 | 1.503153e-01 |  |
| 278 | 2 | A | A | A | 92.25 | 0.000000e+00 | \* |
| 278 | 2 | A | A | C | 2.07 | 3.211920e-01 |  |
| 278 | 2 | A | A | G | 1.81 | 3.114202e-01 |  |
| 278 | 2 | A | A | T | 3.88 | 1.240480e-01 |  |
| 279 | 3 | G | G | A | 3.41 | 3.036560e-01 |  |
| 279 | 3 | G | G | C | 2.73 | 1.555660e-01 |  |
| 279 | 3 | G | G | G | 93.52 | 0.000000e+00 | \* |
| 279 | 3 | G | G | T | 0.34 | 8.969413e-01 |  |
| 280 | 4 | A | A | A | 93.27 | 0.000000e+00 | \* |
| 280 | 4 | A | A | C | 0.92 | 7.740229e-01 |  |
| 280 | 4 | A | A | G | 0.76 | 7.873515e-01 |  |
| 280 | 4 | A | A | T | 5.05 | 4.834024e-02 |  |
| 281 | 5 | C | C | A | 4.18 | 2.156739e-01 |  |
| 281 | 5 | C | C | C | 91.91 | 0.000000e+00 | \* |
| 281 | 5 | C | C | G | 1.83 | 3.046821e-01 |  |
| 281 | 5 | C | C | T | 2.09 | 4.349801e-01 |  |
| 282 | 6 | T | T | A | 4.35 | 1.993363e-01 |  |
| 282 | 6 | T | T | C | 2.39 | 2.286383e-01 |  |
| 282 | 6 | T | T | G | 2.17 | 1.995343e-01 |  |
| 282 | 6 | T | T | T | 91.09 | 0.000000e+00 | \* |
| 283 | 7 | G | G | A | 3.79 | 2.576835e-01 |  |
| 283 | 7 | G | G | C | 0.00 | 9.166667e-01 |  |
| 283 | 7 | G | G | G | 95.11 | 0.000000e+00 | \* |
| 283 | 7 | G | G | T | 1.10 | 7.239790e-01 |  |
| 284 | 8 | G | G | A | 5.43 | 1.187399e-01 |  |
| 284 | 8 | G | G | C | 1.58 | 5.000172e-01 |  |
| 284 | 8 | G | G | G | 90.95 | 0.000000e+00 | \* |
| 284 | 8 | G | G | T | 2.04 | 4.489622e-01 |  |
| 285 | 9 | C | C | A | 4.47 | 1.881502e-01 |  |
| 285 | 9 | C | C | C | 90.65 | 0.000000e+00 | \* |
| 285 | 9 | C | C | G | 4.07 | 1.210463e-02 |  |
| 285 | 9 | C | C | T | 0.81 | 8.050898e-01 |  |
| 286 | 10 | C | C | A | 2.35 | 4.693428e-01 |  |
| 286 | 10 | C | C | C | 93.90 | 0.000000e+00 | \* |
| 286 | 10 | C | C | G | 1.88 | 2.872680e-01 |  |
| 286 | 10 | C | C | T | 1.88 | 4.925713e-01 |  |
| 287 | 11 | A | A | A | 80.38 | 0.000000e+00 | \* |
| 287 | 11 | A | A | C | 2.22 | 2.760535e-01 |  |
| 287 | 11 | A | A | G | 1.90 | 2.802788e-01 |  |
| 287 | 11 | A | A | T | 15.51 | 2.713841e-06 | \* |
| 288 | 12 | G | G | A | 2.79 | 3.950439e-01 |  |
| 288 | 12 | G | G | C | 2.48 | 2.080325e-01 |  |
| 288 | 12 | G | G | G | 94.43 | 0.000000e+00 | \* |
| 288 | 12 | G | G | T | 0.31 | 9.004665e-01 |  |
| 289 | 13 | A | A | A | 93.55 | 0.000000e+00 | \* |
| 289 | 13 | A | A | C | 0.97 | 7.554544e-01 |  |
| 289 | 13 | A | A | G | 0.65 | 8.349258e-01 |  |
| 289 | 13 | A | A | T | 4.84 | 5.738975e-02 |  |
| 290 | 14 | C | C | A | 4.16 | 2.178366e-01 |  |
| 290 | 14 | C | C | C | 93.51 | 0.000000e+00 | \* |
| 290 | 14 | C | C | G | 0.26 | 9.251629e-01 |  |
| 290 | 14 | C | C | T | 2.08 | 4.378442e-01 |  |
| 291 | 15 | C | C | A | 6.15 | 8.316238e-02 |  |
| 291 | 15 | C | C | C | 90.78 | 0.000000e+00 | \* |
| 291 | 15 | C | C | G | 2.51 | 1.270889e-01 |  |
| 291 | 15 | C | C | T | 0.56 | 8.628820e-01 |  |
| 292 | 16 | G | G | A | 0.00 | 8.363636e-01 |  |
| 292 | 16 | G | G | C | 2.11 | 3.061398e-01 |  |
| 292 | 16 | G | G | G | 96.07 | 0.000000e+00 | \* |
| 292 | 16 | G | G | T | 1.81 | 5.111594e-01 |  |
| 293 | 17 | T | T | A | 0.00 | 8.363636e-01 |  |
| 293 | 17 | T | T | C | 1.89 | 3.800962e-01 |  |
| 293 | 17 | T | T | G | 1.89 | 2.818786e-01 |  |
| 293 | 17 | T | T | T | 96.21 | 0.000000e+00 | \* |
| 294 | 18 | G | G | A | 4.81 | 1.604957e-01 |  |
| 294 | 18 | G | G | C | 0.00 | 9.166667e-01 |  |
| 294 | 18 | G | G | G | 94.81 | 0.000000e+00 | \* |
| 294 | 18 | G | G | T | 0.38 | 8.915007e-01 |  |
| 295 | 19 | T | T | A | 1.44 | 6.414238e-01 |  |
| 295 | 19 | T | T | C | 0.86 | 7.926513e-01 |  |
| 295 | 19 | T | T | G | 4.32 | 7.928564e-03 | \* |
| 295 | 19 | T | T | T | 93.37 | 0.000000e+00 | \* |
| 296 | 20 | T | T | A | 0.80 | 7.594327e-01 |  |
| 296 | 20 | T | T | C | 0.80 | 8.153314e-01 |  |
| 296 | 20 | T | T | G | 2.92 | 7.172926e-02 |  |
| 296 | 20 | T | T | T | 95.49 | 0.000000e+00 | \* |
| 297 | 21 | T | T | A | 0.00 | 8.363636e-01 |  |
| 297 | 21 | T | T | C | 1.98 | 3.516335e-01 |  |
| 297 | 21 | T | T | G | 1.98 | 2.556408e-01 |  |
| 297 | 21 | T | T | T | 96.05 | 0.000000e+00 | \* |
| 298 | 22 | G | G | A | 2.49 | 4.444081e-01 |  |
| 298 | 22 | G | G | C | 1.66 | 4.691665e-01 |  |
| 298 | 22 | G | G | G | 95.02 | 0.000000e+00 | \* |
| 298 | 22 | G | G | T | 0.83 | 8.007369e-01 |  |
| 299 | 23 | T | T | A | 0.00 | 8.363636e-01 |  |
| 299 | 23 | T | T | C | 1.90 | 3.783810e-01 |  |
| 299 | 23 | T | T | G | 1.27 | 5.459920e-01 |  |
| 299 | 23 | T | T | T | 96.84 | 0.000000e+00 | \* |
| 300 | 24 | G | G | A | 3.33 | 3.142837e-01 |  |
| 300 | 24 | G | G | C | 1.54 | 5.187212e-01 |  |
| 300 | 24 | G | G | G | 93.08 | 0.000000e+00 | \* |
| 300 | 24 | G | G | T | 2.05 | 4.449240e-01 |  |
| 301 | 25 | C | C | A | 2.50 | 4.426204e-01 |  |
| 301 | 25 | C | C | C | 95.62 | 0.000000e+00 | \* |
| 301 | 25 | C | C | G | 1.88 | 2.882642e-01 |  |
| 301 | 25 | C | C | T | 0.00 | 9.148936e-01 |  |
| 302 | 26 | A | A | A | 89.74 | 0.000000e+00 | \* |
| 302 | 26 | A | A | C | 3.66 | 4.795215e-02 |  |
| 302 | 26 | A | A | G | 4.03 | 1.282873e-02 |  |
| 302 | 26 | A | A | T | 2.56 | 3.213304e-01 |  |
| 303 | 27 | C | C | A | 2.45 | 4.508737e-01 |  |
| 303 | 27 | C | C | C | 90.19 | 0.000000e+00 | \* |
| 303 | 27 | C | C | G | 5.45 | 1.161398e-03 | \* |
| 303 | 27 | C | C | T | 1.91 | 4.843002e-01 |  |
| 304 | 28 | T | T | A | 1.51 | 6.284522e-01 |  |
| 304 | 28 | T | T | C | 3.02 | 1.104102e-01 |  |
| 304 | 28 | T | T | G | 0.00 | 9.333333e-01 |  |
| 304 | 28 | T | T | T | 95.48 | 0.000000e+00 | \* |
| 305 | 29 | A | A | A | 95.09 | 0.000000e+00 | \* |
| 305 | 29 | A | A | C | 1.84 | 3.995291e-01 |  |
| 305 | 29 | A | A | G | 1.23 | 5.650781e-01 |  |
| 305 | 29 | A | A | T | 1.84 | 5.031984e-01 |  |
| 306 | 30 | C | C | A | 1.69 | 5.937670e-01 |  |
| 306 | 30 | C | C | C | 95.51 | 0.000000e+00 | \* |
| 306 | 30 | C | C | G | 0.84 | 7.527627e-01 |  |
| 306 | 30 | C | C | T | 1.97 | 4.679527e-01 |  |
| 307 | 31 | A | A | A | 91.04 | 0.000000e+00 | \* |
| 307 | 31 | A | A | C | 2.51 | 2.006694e-01 |  |
| 307 | 31 | A | A | G | 2.87 | 7.718077e-02 |  |
| 307 | 31 | A | A | T | 3.58 | 1.550701e-01 |  |
| 308 | 32 | C | C | A | 1.76 | 5.793619e-01 |  |
| 308 | 32 | C | C | C | 93.55 | 0.000000e+00 | \* |
| 308 | 32 | C | C | G | 1.47 | 4.511777e-01 |  |
| 308 | 32 | C | C | T | 3.23 | 2.023428e-01 |  |
| 309 | 33 | G | G | A | 2.93 | 3.727019e-01 |  |
| 309 | 33 | G | G | C | 2.09 | 3.132695e-01 |  |
| 309 | 33 | G | G | G | 94.98 | 0.000000e+00 | \* |
| 309 | 33 | G | G | T | 0.00 | 9.148936e-01 |  |
| 310 | 34 | G | G | A | 1.99 | 5.353988e-01 |  |
| 310 | 34 | G | G | C | 1.42 | 5.683904e-01 |  |
| 310 | 34 | G | G | G | 96.02 | 0.000000e+00 | \* |
| 310 | 34 | G | G | T | 0.57 | 8.610290e-01 |  |
| 311 | 35 | G | G | A | 2.77 | 3.979643e-01 |  |
| 311 | 35 | G | G | C | 1.38 | 5.838678e-01 |  |
| 311 | 35 | G | G | G | 95.50 | 0.000000e+00 | \* |
| 311 | 35 | G | G | T | 0.35 | 8.963827e-01 |  |
| 312 | 36 | C | C | A | 1.95 | 5.421416e-01 |  |
| 312 | 36 | C | C | C | 94.14 | 0.000000e+00 | \* |
| 312 | 36 | C | C | G | 1.95 | 2.626024e-01 |  |
| 312 | 36 | C | C | T | 1.95 | 4.715789e-01 |  |
| 313 | 37 | A | A | A | 90.03 | 0.000000e+00 | \* |
| 313 | 37 | A | A | C | 2.99 | 1.138639e-01 |  |
| 313 | 37 | A | A | G | 1.66 | 3.676839e-01 |  |
| 313 | 37 | A | A | T | 5.32 | 3.856112e-02 |  |
| 314 | 38 | C | C | A | 1.49 | 6.313761e-01 |  |
| 314 | 38 | C | C | C | 96.77 | 0.000000e+00 | \* |
| 314 | 38 | C | C | G | 0.50 | 8.821774e-01 |  |
| 314 | 38 | C | C | T | 1.24 | 6.821049e-01 |  |
| 315 | 39 | C | C | A | 3.24 | 3.266524e-01 |  |
| 315 | 39 | C | C | C | 94.32 | 0.000000e+00 | \* |
| 315 | 39 | C | C | G | 1.35 | 5.046523e-01 |  |
| 315 | 39 | C | C | T | 1.08 | 7.307555e-01 |  |
| 316 | 40 | C | C | A | 2.42 | 4.566314e-01 |  |
| 316 | 40 | C | C | C | 93.01 | 0.000000e+00 | \* |
| 316 | 40 | C | C | G | 1.61 | 3.874108e-01 |  |
| 316 | 40 | C | C | T | 2.96 | 2.453773e-01 |  |
| 317 | 41 | T | T | A | 0.00 | 8.363636e-01 |  |
| 317 | 41 | T | T | C | 2.60 | 1.801119e-01 |  |
| 317 | 41 | T | T | G | 1.56 | 4.087073e-01 |  |
| 317 | 41 | T | T | T | 95.83 | 0.000000e+00 | \* |
| 318 | 42 | G | G | A | 3.64 | 2.743493e-01 |  |
| 318 | 42 | G | G | C | 1.37 | 5.912605e-01 |  |
| 318 | 42 | G | G | G | 93.85 | 0.000000e+00 | \* |
| 318 | 42 | G | G | T | 1.14 | 7.136411e-01 |  |
| 319 | 43 | A | A | A | 86.89 | 0.000000e+00 | \* |
| 319 | 43 | A | A | C | 1.21 | 6.563815e-01 |  |
| 319 | 43 | A | A | G | 1.70 | 3.526406e-01 |  |
| 319 | 43 | A | A | T | 10.19 | 4.688077e-04 | \* |
| 320 | 44 | C | C | A | 2.64 | 4.190432e-01 |  |
| 320 | 44 | C | C | C | 95.01 | 0.000000e+00 | \* |
| 320 | 44 | C | C | G | 2.35 | 1.594222e-01 |  |
| 320 | 44 | C | C | T | 0.00 | 9.148936e-01 |  |
| 321 | 45 | C | C | A | 5.23 | 1.308809e-01 |  |
| 321 | 45 | C | C | C | 91.38 | 0.000000e+00 | \* |
| 321 | 45 | C | C | G | 1.54 | 4.191003e-01 |  |
| 321 | 45 | C | C | T | 1.85 | 5.015840e-01 |  |
| 322 | 46 | G | G | A | 3.07 | 3.521907e-01 |  |
| 322 | 46 | G | G | C | 3.83 | 3.821270e-02 |  |
| 322 | 46 | G | G | G | 92.34 | 0.000000e+00 | \* |
| 322 | 46 | G | G | T | 0.77 | 8.168554e-01 |  |
| 323 | 47 | A | A | A | 94.41 | 0.000000e+00 | \* |
| 323 | 47 | A | A | C | 1.00 | 7.439339e-01 |  |
| 323 | 47 | A | A | G | 0.60 | 8.512916e-01 |  |
| 323 | 47 | A | A | T | 3.99 | 1.133402e-01 |  |
| 324 | 48 | C | C | A | 3.44 | 3.004398e-01 |  |
| 324 | 48 | C | C | C | 93.44 | 0.000000e+00 | \* |
| 324 | 48 | C | C | G | 0.62 | 8.422034e-01 |  |
| 324 | 48 | C | C | T | 2.50 | 3.352667e-01 |  |
| 325 | 49 | G | G | A | 8.00 | 3.184291e-02 |  |
| 325 | 49 | G | G | C | 2.50 | 2.026983e-01 |  |
| 325 | 49 | G | G | G | 87.50 | 0.000000e+00 | \* |
| 325 | 49 | G | G | T | 2.00 | 4.587402e-01 |  |
| 326 | 50 | G | G | A | 0.56 | 7.939267e-01 |  |
| 326 | 50 | G | G | C | 1.41 | 5.734931e-01 |  |
| 326 | 50 | G | G | G | 97.18 | 0.000000e+00 | \* |
| 326 | 50 | G | G | T | 0.85 | 7.967704e-01 |  |
| 327 | 51 | C | C | A | 1.40 | 6.502708e-01 |  |
| 327 | 51 | C | C | C | 93.95 | 0.000000e+00 | \* |
| 327 | 51 | C | C | G | 2.33 | 1.638037e-01 |  |
| 327 | 51 | C | C | T | 2.33 | 3.754054e-01 |  |
| 328 | 52 | A | A | A | 91.57 | 0.000000e+00 | \* |
| 328 | 52 | A | A | C | 1.69 | 4.589505e-01 |  |
| 328 | 52 | A | A | G | 1.69 | 3.580058e-01 |  |
| 328 | 52 | A | A | T | 5.06 | 4.792702e-02 |  |
| 329 | 53 | A | A | A | 93.46 | 0.000000e+00 | \* |
| 329 | 53 | A | A | C | 0.62 | 8.630063e-01 |  |
| 329 | 53 | A | A | G | 1.25 | 5.556635e-01 |  |
| 329 | 53 | A | A | T | 4.67 | 6.575169e-02 |  |
| 330 | 54 | G | G | A | 1.65 | 6.000978e-01 |  |
| 330 | 54 | G | G | C | 0.83 | 8.054454e-01 |  |
| 330 | 54 | G | G | G | 96.69 | 0.000000e+00 | \* |
| 330 | 54 | G | G | T | 0.83 | 8.016262e-01 |  |
| 331 | 55 | A | A | A | 95.28 | 0.000000e+00 | \* |
| 331 | 55 | A | A | C | 0.00 | 9.166667e-01 |  |
| 331 | 55 | A | A | G | 0.88 | 7.332024e-01 |  |
| 331 | 55 | A | A | T | 3.83 | 1.280590e-01 |  |
| 332 | 56 | A | A | A | 93.85 | 0.000000e+00 | \* |
| 332 | 56 | A | A | C | 0.65 | 8.572076e-01 |  |
| 332 | 56 | A | A | G | 0.97 | 6.920625e-01 |  |
| 332 | 56 | A | A | T | 4.53 | 7.381030e-02 |  |
| 333 | 57 | G | G | A | 1.50 | 6.299216e-01 |  |
| 333 | 57 | G | G | C | 0.00 | 9.166667e-01 |  |
| 333 | 57 | G | G | G | 96.00 | 0.000000e+00 | \* |
| 333 | 57 | G | G | T | 2.50 | 3.352667e-01 |  |
| 334 | 58 | T | T | A | 3.56 | 2.849415e-01 |  |
| 334 | 58 | T | T | C | 1.07 | 7.165764e-01 |  |
| 334 | 58 | T | T | G | 0.71 | 8.092709e-01 |  |
| 334 | 58 | T | T | T | 94.66 | 0.000000e+00 | \* |
| 335 | 59 | T | T | A | 4.03 | 2.310785e-01 |  |
| 335 | 59 | T | T | C | 1.34 | 6.016958e-01 |  |
| 335 | 59 | T | T | G | 3.02 | 6.171896e-02 |  |
| 335 | 59 | T | T | T | 91.61 | 0.000000e+00 | \* |
| 336 | 60 | C | C | A | 1.35 | 6.587811e-01 |  |
| 336 | 60 | C | C | C | 94.59 | 0.000000e+00 | \* |
| 336 | 60 | C | C | G | 2.70 | 9.770515e-02 |  |
| 336 | 60 | C | C | T | 1.35 | 6.493386e-01 |  |
| 337 | 61 | G | G | A | 4.65 | 1.728901e-01 |  |
| 337 | 61 | G | G | C | 1.86 | 3.922003e-01 |  |
| 337 | 61 | G | G | G | 93.02 | 0.000000e+00 | \* |
| 337 | 61 | G | G | T | 0.47 | 8.795531e-01 |  |
| 338 | 62 | A | A | A | 95.27 | 0.000000e+00 | \* |
| 338 | 62 | A | A | C | 1.24 | 6.436259e-01 |  |
| 338 | 62 | A | A | G | 0.75 | 7.950778e-01 |  |
| 338 | 62 | A | A | T | 2.74 | 2.860552e-01 |  |
| 339 | 63 | C | C | A | 2.40 | 4.608759e-01 |  |
| 339 | 63 | C | C | C | 95.21 | 0.000000e+00 | \* |
| 339 | 63 | C | C | G | 0.90 | 7.269675e-01 |  |
| 339 | 63 | C | C | T | 1.50 | 6.048991e-01 |  |
| 340 | 64 | A | A | A | 91.00 | 0.000000e+00 | \* |
| 340 | 64 | A | A | C | 1.90 | 3.794534e-01 |  |
| 340 | 64 | A | A | G | 2.84 | 7.988180e-02 |  |
| 340 | 64 | A | A | T | 4.27 | 9.134694e-02 |  |
| 341 | 65 | G | G | A | 0.87 | 7.467942e-01 |  |
| 341 | 65 | G | G | C | 2.33 | 2.455412e-01 |  |
| 341 | 65 | G | G | G | 95.35 | 0.000000e+00 | \* |
| 341 | 65 | G | G | T | 1.45 | 6.181484e-01 |  |
| 342 | 66 | C | C | A | 1.48 | 6.335302e-01 |  |
| 342 | 66 | C | C | C | 93.70 | 0.000000e+00 | \* |
| 342 | 66 | C | C | G | 1.85 | 2.962124e-01 |  |
| 342 | 66 | C | C | T | 2.96 | 2.443449e-01 |  |
| 343 | 67 | T | T | A | 0.00 | 8.363636e-01 |  |
| 343 | 67 | T | T | C | 0.25 | 9.130865e-01 |  |
| 343 | 67 | T | T | G | 1.01 | 6.715784e-01 |  |
| 343 | 67 | T | T | T | 98.73 | 0.000000e+00 | \* |
| 344 | 68 | C | C | A | 2.22 | 4.919181e-01 |  |
| 344 | 68 | C | C | C | 93.33 | 0.000000e+00 | \* |
| 344 | 68 | C | C | G | 1.59 | 3.981430e-01 |  |
| 344 | 68 | C | C | T | 2.86 | 2.631649e-01 |  |
| 345 | 69 | C | C | A | 2.03 | 5.284672e-01 |  |
| 345 | 69 | C | C | C | 93.92 | 0.000000e+00 | \* |
| 345 | 69 | C | C | G | 0.25 | 9.257664e-01 |  |
| 345 | 69 | C | C | T | 3.80 | 1.317978e-01 |  |
| 346 | 70 | C | C | A | 2.32 | 4.748473e-01 |  |
| 346 | 70 | C | C | C | 93.44 | 0.000000e+00 | \* |
| 346 | 70 | C | C | G | 0.77 | 7.840606e-01 |  |
| 346 | 70 | C | C | T | 3.47 | 1.683499e-01 |  |
| 347 | 71 | G | G | A | 6.64 | 6.470853e-02 |  |
| 347 | 71 | G | G | C | 1.24 | 6.431886e-01 |  |
| 347 | 71 | G | G | G | 91.70 | 0.000000e+00 | \* |
| 347 | 71 | G | G | T | 0.41 | 8.872747e-01 |  |
| 348 | 72 | C | C | A | 0.00 | 8.363636e-01 |  |
| 348 | 72 | C | C | C | 96.93 | 0.000000e+00 | \* |
| 348 | 72 | C | C | G | 0.77 | 7.866000e-01 |  |
| 348 | 72 | C | C | T | 2.30 | 3.818416e-01 |  |
| 349 | 73 | G | G | A | 8.61 | 2.305598e-02 |  |
| 349 | 73 | G | G | C | 2.87 | 1.319396e-01 |  |
| 349 | 73 | G | G | G | 86.48 | 0.000000e+00 | \* |
| 349 | 73 | G | G | T | 2.05 | 4.454854e-01 |  |
| 350 | 74 | A | A | A | 95.60 | 0.000000e+00 | \* |
| 350 | 74 | A | A | C | 1.47 | 5.489817e-01 |  |
| 350 | 74 | A | A | G | 0.59 | 8.554072e-01 |  |
| 350 | 74 | A | A | T | 2.35 | 3.705299e-01 |  |
| 351 | 75 | C | C | A | 2.91 | 3.760757e-01 |  |
| 351 | 75 | C | C | C | 85.76 | 0.000000e+00 | \* |
| 351 | 75 | C | C | G | 8.14 | 8.735226e-06 | \* |
| 351 | 75 | C | C | T | 3.20 | 2.065267e-01 |  |

## For use in R

If you want to work with the results in R, here is output that you can copy and paste in your terminal to get:

The base information:

```
structure(list(focal.base = c("A", "C", "G", "T"), avg.percsignal = c(92.3264978111306, 
93.8476672069164, 93.4748651350263, 95.031913096515), avg.areasignal = c(337.147058823529, 
355.761194029851, 335.543859649123, 413.767441860465), crit.perc.area = c(10.1475136040565, 
4.78018257510928, 4.18191667193591, 6.87069635947951), mu = c(3.15245289378729, 
1.85516711971654, 1.58431891499079, 2.3052754942869), fillibens = c(0.997194670554706, 
0.990172952666437, 0.984163298671519, 0.993106263968579)), .Names = c("focal.base", 
"avg.percsignal", "avg.areasignal", "crit.perc.area", "mu", "fillibens"
), row.names = c(NA, -4L), class = "data.frame")
```

the data.frame that contains information on the guide region:

```
structure(list(A.area = c(309, 357, 10, 610, 16, 20, 24, 24, 
11, 10, 254, 9, 580, 16, 22, 0, 0, 25, 5, 3, 0, 12, 0, 13, 8, 
245, 9, 6, 310, 6, 254, 6, 7, 7, 8, 5, 271, 6, 12, 9, 0, 16, 
358, 9, 17, 8, 473, 11, 16, 2, 3, 163, 300, 4, 323, 290, 3, 10, 
12, 4, 10, 383, 8, 192, 3, 4, 0, 7, 8, 6, 16, 0, 21, 326, 10), 
    C.area = c(9, 8, 8, 6, 352, 11, 0, 7, 223, 400, 7, 8, 6, 
    360, 325, 7, 5, 0, 3, 3, 8, 8, 6, 6, 306, 10, 331, 12, 6, 
    340, 7, 319, 5, 5, 4, 241, 9, 389, 349, 346, 10, 6, 5, 324, 
    297, 10, 5, 299, 5, 5, 202, 3, 2, 2, 0, 2, 0, 3, 4, 280, 
    4, 5, 318, 4, 8, 253, 1, 294, 371, 242, 3, 253, 7, 5, 295
    ), G.area = c(1, 7, 274, 5, 7, 10, 603, 402, 10, 8, 6, 305, 
    4, 1, 9, 318, 5, 493, 15, 11, 8, 458, 4, 363, 6, 11, 20, 
    0, 4, 3, 8, 5, 227, 338, 276, 5, 5, 2, 5, 6, 6, 412, 7, 8, 
    5, 241, 3, 2, 175, 345, 5, 3, 4, 234, 3, 3, 192, 2, 9, 8, 
    200, 3, 3, 6, 328, 5, 4, 5, 1, 2, 221, 2, 211, 2, 28), T.area = c(12, 
    15, 1, 33, 8, 419, 7, 9, 2, 8, 49, 1, 30, 8, 2, 6, 254, 2, 
    324, 360, 389, 4, 306, 8, 0, 7, 7, 380, 6, 7, 10, 11, 0, 
    2, 1, 5, 16, 5, 4, 11, 368, 5, 42, 0, 6, 2, 20, 8, 4, 3, 
    5, 9, 15, 2, 13, 14, 5, 266, 273, 4, 1, 11, 5, 9, 5, 8, 390, 
    9, 15, 9, 1, 6, 5, 8, 11), Tot.area = c(331, 387, 293, 654, 
    383, 460, 634, 442, 246, 426, 316, 323, 620, 385, 358, 331, 
    264, 520, 347, 377, 405, 482, 316, 390, 320, 273, 367, 398, 
    326, 356, 279, 341, 239, 352, 289, 256, 301, 402, 370, 372, 
    384, 439, 412, 341, 325, 261, 501, 320, 200, 355, 215, 178, 
    321, 242, 339, 309, 200, 281, 298, 296, 215, 402, 334, 211, 
    344, 270, 395, 315, 395, 259, 241, 261, 244, 341, 344), A.perc = c(93.3534743202417, 
    92.2480620155039, 3.41296928327645, 93.2721712538226, 4.17754569190601, 
    4.34782608695652, 3.78548895899054, 5.42986425339367, 4.47154471544715, 
    2.34741784037559, 80.379746835443, 2.78637770897833, 93.5483870967742, 
    4.15584415584416, 6.14525139664805, 0, 0, 4.80769230769231, 
    1.44092219020173, 0.795755968169761, 0, 2.4896265560166, 
    0, 3.33333333333333, 2.5, 89.7435897435897, 2.45231607629428, 
    1.50753768844221, 95.0920245398773, 1.68539325842697, 91.0394265232975, 
    1.75953079178886, 2.92887029288703, 1.98863636363636, 2.7681660899654, 
    1.953125, 90.0332225913621, 1.49253731343284, 3.24324324324324, 
    2.41935483870968, 0, 3.64464692482916, 86.8932038834951, 
    2.63929618768328, 5.23076923076923, 3.06513409961686, 94.4111776447106, 
    3.4375, 8, 0.563380281690141, 1.3953488372093, 91.5730337078652, 
    93.4579439252336, 1.65289256198347, 95.2802359882006, 93.8511326860841, 
    1.5, 3.55871886120996, 4.02684563758389, 1.35135135135135, 
    4.65116279069767, 95.273631840796, 2.39520958083832, 90.9952606635071, 
    0.872093023255814, 1.48148148148148, 0, 2.22222222222222, 
    2.0253164556962, 2.31660231660232, 6.63900414937759, 0, 8.60655737704918, 
    95.6011730205279, 2.90697674418605), C.perc = c(2.7190332326284, 
    2.0671834625323, 2.73037542662116, 0.917431192660551, 91.9060052219321, 
    2.39130434782609, 0, 1.58371040723982, 90.650406504065, 93.8967136150235, 
    2.21518987341772, 2.47678018575851, 0.967741935483871, 93.5064935064935, 
    90.7821229050279, 2.11480362537764, 1.89393939393939, 0, 
    0.864553314121037, 0.795755968169761, 1.97530864197531, 1.6597510373444, 
    1.89873417721519, 1.53846153846154, 95.625, 3.66300366300366, 
    90.1907356948229, 3.01507537688442, 1.84049079754601, 95.5056179775281, 
    2.5089605734767, 93.5483870967742, 2.09205020920502, 1.42045454545455, 
    1.3840830449827, 94.140625, 2.99003322259136, 96.7661691542289, 
    94.3243243243243, 93.010752688172, 2.60416666666667, 1.36674259681093, 
    1.21359223300971, 95.0146627565982, 91.3846153846154, 3.83141762452107, 
    0.998003992015968, 93.4375, 2.5, 1.40845070422535, 93.953488372093, 
    1.68539325842697, 0.623052959501558, 0.826446280991736, 0, 
    0.647249190938511, 0, 1.06761565836299, 1.34228187919463, 
    94.5945945945946, 1.86046511627907, 1.24378109452736, 95.2095808383233, 
    1.8957345971564, 2.32558139534884, 93.7037037037037, 0.253164556962025, 
    93.3333333333333, 93.9240506329114, 93.4362934362934, 1.2448132780083, 
    96.9348659003831, 2.86885245901639, 1.46627565982405, 85.7558139534884
    ), G.perc = c(0.302114803625378, 1.80878552971576, 93.5153583617747, 
    0.764525993883792, 1.82767624020888, 2.17391304347826, 95.1104100946372, 
    90.9502262443439, 4.0650406504065, 1.87793427230047, 1.89873417721519, 
    94.4272445820433, 0.645161290322581, 0.25974025974026, 2.51396648044693, 
    96.0725075528701, 1.89393939393939, 94.8076923076923, 4.32276657060519, 
    2.91777188328912, 1.97530864197531, 95.0207468879668, 1.26582278481013, 
    93.0769230769231, 1.875, 4.02930402930403, 5.44959128065395, 
    0, 1.22699386503067, 0.842696629213483, 2.8673835125448, 
    1.46627565982405, 94.9790794979079, 96.0227272727273, 95.5017301038062, 
    1.953125, 1.66112956810631, 0.497512437810945, 1.35135135135135, 
    1.61290322580645, 1.5625, 93.8496583143508, 1.69902912621359, 
    2.34604105571848, 1.53846153846154, 92.3371647509579, 0.598802395209581, 
    0.625, 87.5, 97.1830985915493, 2.32558139534884, 1.68539325842697, 
    1.24610591900312, 96.6942148760331, 0.884955752212389, 0.970873786407767, 
    96, 0.711743772241993, 3.02013422818792, 2.7027027027027, 
    93.0232558139535, 0.746268656716418, 0.898203592814371, 2.8436018957346, 
    95.3488372093023, 1.85185185185185, 1.0126582278481, 1.58730158730159, 
    0.253164556962025, 0.772200772200772, 91.701244813278, 0.766283524904215, 
    86.4754098360656, 0.586510263929619, 8.13953488372093), T.perc = c(3.62537764350453, 
    3.87596899224806, 0.341296928327645, 5.04587155963303, 2.088772845953, 
    91.0869565217391, 1.10410094637224, 2.03619909502262, 0.813008130081301, 
    1.87793427230047, 15.506329113924, 0.309597523219814, 4.83870967741935, 
    2.07792207792208, 0.558659217877095, 1.81268882175227, 96.2121212121212, 
    0.384615384615385, 93.371757925072, 95.4907161803714, 96.0493827160494, 
    0.829875518672199, 96.8354430379747, 2.05128205128205, 0, 
    2.56410256410256, 1.90735694822888, 95.4773869346734, 1.84049079754601, 
    1.96629213483146, 3.584229390681, 3.2258064516129, 0, 0.568181818181818, 
    0.346020761245675, 1.953125, 5.3156146179402, 1.24378109452736, 
    1.08108108108108, 2.95698924731183, 95.8333333333333, 1.13895216400911, 
    10.1941747572816, 0, 1.84615384615385, 0.766283524904215, 
    3.99201596806387, 2.5, 2, 0.845070422535211, 2.32558139534884, 
    5.0561797752809, 4.67289719626168, 0.826446280991736, 3.83480825958702, 
    4.53074433656958, 2.5, 94.661921708185, 91.6107382550336, 
    1.35135135135135, 0.465116279069767, 2.7363184079602, 1.49700598802395, 
    4.2654028436019, 1.45348837209302, 2.96296296296296, 98.7341772151899, 
    2.85714285714286, 3.79746835443038, 3.47490347490348, 0.4149377593361, 
    2.29885057471264, 2.04918032786885, 2.34604105571848, 3.19767441860465
    ), base.call = c("A", "A", "G", "A", "C", "T", "G", "G", 
    "C", "C", "A", "G", "A", "C", "C", "G", "T", "G", "T", "T", 
    "T", "G", "T", "G", "C", "A", "C", "T", "A", "C", "A", "C", 
    "G", "G", "G", "C", "A", "C", "C", "C", "T", "G", "A", "C", 
    "C", "G", "A", "C", "G", "G", "C", "A", "A", "G", "A", "A", 
    "G", "T", "T", "C", "G", "A", "C", "A", "G", "C", "T", "C", 
    "C", "C", "G", "C", "G", "A", "C"), index = 277:351, guide.seq = c("A", 
    "A", "G", "A", "C", "T", "G", "G", "C", "C", "A", "G", "A", 
    "C", "C", "G", "T", "G", "T", "T", "T", "G", "T", "G", "C", 
    "A", "C", "T", "A", "C", "A", "C", "G", "G", "G", "C", "A", 
    "C", "C", "C", "T", "G", "A", "C", "C", "G", "A", "C", "G", 
    "G", "C", "A", "A", "G", "A", "A", "G", "T", "T", "C", "G", 
    "A", "C", "A", "G", "C", "T", "C", "C", "C", "G", "C", "G", 
    "A", "C"), T.pval = c(0.15031533963972, 0.124047966826789, 
    0.896941258936708, 0.0483402419620262, 0.434980077976908, 
    0, 0.723978971155468, 0.448962153376093, 0.8050897587957, 
    0.492571345396784, 2.71384144356901e-06, 0.900466515308204, 
    0.0573897526466952, 0.437844248352798, 0.862882038033267, 
    0.5111594095932, 0, 0.891500650453243, 0, 0, 0, 0.800736879650822, 
    0, 0.444923973179662, 0.91489361696974, 0.321330370501822, 
    0.484300154001278, 0, 0.503198395353219, 0.467952737622027, 
    0.15507013120696, 0.202342779435237, 0.91489361696974, 0.861029021837296, 
    0.896382660566216, 0.471578901532135, 0.0385611220387064, 
    0.682104881338484, 0.730755488085469, 0.245377301140438, 
    0, 0.713641076989604, 0.000468807749362643, 0.91489361696974, 
    0.501584046544506, 0.816855427415154, 0.113340246979588, 
    0.335266711555412, 0.458740198317709, 0.796770448874546, 
    0.375405372346898, 0.0479270190070559, 0.065751685163878, 
    0.80162617829586, 0.128059002348643, 0.0738103031377383, 
    0.335266711555412, 0, 0, 0.649338587674561, 0.879553120080747, 
    0.286055228964475, 0.604899078827622, 0.0913469393637977, 
    0.618148382550122, 0.244344867269324, 0, 0.263164940838794, 
    0.131797751596751, 0.168349912086901, 0.887274697861386, 
    0.381841579983532, 0.445485386091774, 0.370529881492134, 
    0.206526658100764), C.pval = c(0.157650631869672, 0.321191995956539, 
    0.155565959257096, 0.774022915280789, 0, 0.228638263383915, 
    0.916666666593763, 0.500017191169818, 0, 0, 0.276053477545173, 
    0.20803245461078, 0.75545441025797, 0, 0, 0.306139812708974, 
    0.380096193344658, 0.916666666593763, 0.79265133006316, 0.815331366503469, 
    0.351633498685029, 0.469166464308963, 0.378380956115229, 
    0.518721161603196, 0, 0.0479521487405901, 0, 0.110410231664533, 
    0.39952907108057, 0, 0.200669406386532, 0, 0.313269518621849, 
    0.568390436838286, 0.583867846051313, 0, 0.113863941770708, 
    0, 0, 0, 0.180111899268212, 0.591260475937047, 0.656381452010674, 
    0, 0, 0.0382127006151851, 0.74393388958332, 0, 0.202698261265703, 
    0.573493074559051, 0, 0.45895045534736, 0.863006314215149, 
    0.805445386479989, 0.916666666593763, 0.857207615595299, 
    0.916666666593763, 0.716576365342724, 0.601695821715801, 
    0, 0.392200305025419, 0.643625882083516, 0, 0.37945344700559, 
    0.245541213582304, 0, 0.913086535567106, 0, 0, 0, 0.643188606326723, 
    0, 0.131939622415103, 0.548981664384105, 0), G.pval = c(0.92056411141113, 
    0.31142024279316, 0, 0.787351453070367, 0.304682057577945, 
    0.199534337998711, 0, 0, 0.0121046255414486, 0.28726798131288, 
    0.280278779202796, 0, 0.834925760133857, 0.925162916806057, 
    0.127088886031548, 0, 0.281878635107524, 0, 0.00792856405636322, 
    0.0717292592084124, 0.255640776436803, 0, 0.545991991040206, 
    0, 0.288264211443343, 0.0128287330736373, 0.00116139760636103, 
    0.933333333333334, 0.565078147417176, 0.75276265289156, 0.0771807690537624, 
    0.451177690699391, 0, 0, 0, 0.262602402195989, 0.367683891027495, 
    0.882177407531016, 0.504652280542623, 0.387410813848585, 
    0.408707313930446, 0, 0.352640583629971, 0.159422225090078, 
    0.419100258403423, 0, 0.851291607995931, 0.84220335957223, 
    0, 0, 0.163803670980219, 0.358005839323982, 0.55566345256741, 
    0, 0.733202405582551, 0.692062494410129, 0, 0.809270867049494, 
    0.0617189570762873, 0.0977051489922415, 0, 0.795077769863908, 
    0.726967515446561, 0.0798817964260613, 0, 0.296212360046534, 
    0.671578397272875, 0.398143004776223, 0.925766398308927, 
    0.78406063384435, 0, 0.786600049681014, 0, 0.855407193405628, 
    8.73522579780683e-06), A.pval = c(0, 0, 0.303655990500708, 
    0, 0.215673917203952, 0.199336332276165, 0.257683504426905, 
    0.118739904571643, 0.188150222489081, 0.469342757918067, 
    0, 0.395043889828415, 0, 0.217836649422383, 0.0831623782201059, 
    0.836363636363636, 0.836363636363636, 0.160495731937409, 
    0.641423830846842, 0.759432726379347, 0.836363636363636, 
    0.444408085125467, 0.836363636363636, 0.314283726225084, 
    0.442620361733808, 0, 0.450873672106419, 0.628452178698871, 
    0, 0.593766988800296, 0, 0.579361856154184, 0.372701931875432, 
    0.535398773953982, 0.397964342165297, 0.542141585242538, 
    0, 0.631376110211062, 0.326652359902963, 0.456631355649064, 
    0.836363636363636, 0.274349330284944, 0, 0.419043208642679, 
    0.130880908064687, 0.352190728990866, 0, 0.300439773854403, 
    0.0318429132797093, 0.793926732198669, 0.650270787750467, 
    0, 0, 0.600097782816179, 0, 0, 0.629921623300808, 0.284941506203242, 
    0.231078526403097, 0.658781097301547, 0.172890138040267, 
    0, 0.460875871676935, 0, 0.746794188191274, 0.633530213470581, 
    0.836363636363636, 0.491918067199663, 0.528467247842917, 
    0.474847304093793, 0.0647085277086206, 0.836363636363636, 
    0.0230559810978508, 0, 0.376075670866017), guide.position = 1:75), .Names = c("A.area", 
"C.area", "G.area", "T.area", "Tot.area", "A.perc", "C.perc", 
"G.perc", "T.perc", "base.call", "index", "guide.seq", "T.pval", 
"C.pval", "G.pval", "A.pval", "guide.position"), row.names = 277:351, class = "data.frame")
```

*Report generated using EditR v1.0.8*
