# Supplementary material for: Mechanistic and genetic basis of single-strand templated repair at Cas12a-induced DNA breaks in Chlamydomonas reinhardtii
Source: Nat Commun. 2021 Nov 19;12:6751. doi: 10.1038/s41467-021-27004-1 (PMC8604939; doi:10.1038/s41467-021-27004-1)
Supplement: Supplementary file 22 — Source Data [file 41467_2021_27004_MOESM22_ESM.zip › Source Data/EditR analysis/EditR outputs/Antisense/rep3_ssODN_antisense_0.html]

EditR v1.0.8 report


# EditR v1.0.8 report

- Data QA
  - Filtering data
  - Percent noise peak area
  - Base information
- Predicted editing
  - Editing bar plot
  - Editing table plot
  - Table of editing results
- For use in R

## Data QA

### Filtering data

What the data looked like prefiltering:

and the post filtering signal / noise plot:

### Percent noise peak area

### Base information

Here’s information about the signal of each base, the critical percent value where any higher value would be called as significant, and Filliben’s correlation for how well the noise was modelled by the zero adjusted gamma distribution.

| Base | Average percent signal | Average peak area | Critical percent value | model mu | Fillibens correlation |
| --- | --- | --- | --- | --- | --- |
| A | 92.29053 | 352.0882 | 9.949588 | 3.279279 | 0.9957344 |
| C | 93.29447 | 371.7463 | 5.188153 | 2.003616 | 0.9966297 |
| G | 92.88646 | 355.0357 | 5.884576 | 1.912811 | 0.9945610 |
| T | 94.73298 | 429.7045 | 6.347384 | 2.241763 | 0.9919078 |

## Predicted editing

### Editing bar plot

### Editing table plot

### Table of editing results


Here’s the entire guide region

| Sanger position | Guide position | Guide sequence | Sanger base call | Focal base | Focal base peak area | p value |  |
| --- | --- | --- | --- | --- | --- | --- | --- |
| 276 | 1 | A | A | A | 94.22 | 0.000000e+00 | \* |
| 276 | 1 | A | A | C | 1.82 | 4.630857e-01 |  |
| 276 | 1 | A | A | G | 0.61 | 8.219869e-01 |  |
| 276 | 1 | A | A | T | 3.34 | 1.602556e-01 |  |
| 277 | 2 | A | A | A | 93.38 | 0.000000e+00 | \* |
| 277 | 2 | A | A | C | 1.23 | 6.969784e-01 |  |
| 277 | 2 | A | A | G | 1.47 | 5.193972e-01 |  |
| 277 | 2 | A | A | T | 3.92 | 9.810497e-02 |  |
| 278 | 3 | G | G | A | 2.84 | 4.145575e-01 |  |
| 278 | 3 | G | G | C | 3.47 | 8.796058e-02 |  |
| 278 | 3 | G | G | G | 93.69 | 0.000000e+00 | \* |
| 278 | 3 | G | G | T | 0.00 | 8.817204e-01 |  |
| 279 | 4 | A | A | A | 93.98 | 0.000000e+00 | \* |
| 279 | 4 | A | A | C | 1.05 | 7.613360e-01 |  |
| 279 | 4 | A | A | G | 1.20 | 6.139615e-01 |  |
| 279 | 4 | A | A | T | 3.76 | 1.128983e-01 |  |
| 280 | 5 | C | C | A | 5.68 | 1.086059e-01 |  |
| 280 | 5 | C | C | C | 92.10 | 0.000000e+00 | \* |
| 280 | 5 | C | C | G | 0.00 | 9.230769e-01 |  |
| 280 | 5 | C | C | T | 2.22 | 3.755268e-01 |  |
| 281 | 6 | T | T | A | 4.62 | 1.855974e-01 |  |
| 281 | 6 | T | T | C | 1.41 | 6.260130e-01 |  |
| 281 | 6 | T | T | G | 2.41 | 2.587252e-01 |  |
| 281 | 6 | T | T | T | 91.57 | 0.000000e+00 | \* |
| 282 | 7 | G | G | A | 4.24 | 2.227947e-01 |  |
| 282 | 7 | G | G | C | 0.00 | 9.285714e-01 |  |
| 282 | 7 | G | G | G | 94.59 | 0.000000e+00 | \* |
| 282 | 7 | G | G | T | 1.17 | 6.869271e-01 |  |
| 283 | 8 | G | G | A | 4.63 | 1.849561e-01 |  |
| 283 | 8 | G | G | C | 2.20 | 3.344839e-01 |  |
| 283 | 8 | G | G | G | 92.51 | 0.000000e+00 | \* |
| 283 | 8 | G | G | T | 0.66 | 8.204075e-01 |  |
| 284 | 9 | C | C | A | 3.82 | 2.714315e-01 |  |
| 284 | 9 | C | C | C | 91.98 | 0.000000e+00 | \* |
| 284 | 9 | C | C | G | 1.91 | 3.820650e-01 |  |
| 284 | 9 | C | C | T | 2.29 | 3.584007e-01 |  |
| 285 | 10 | C | C | A | 1.98 | 5.707004e-01 |  |
| 285 | 10 | C | C | C | 92.29 | 0.000000e+00 | \* |
| 285 | 10 | C | C | G | 2.86 | 1.770815e-01 |  |
| 285 | 10 | C | C | T | 2.86 | 2.351652e-01 |  |
| 286 | 11 | A | A | A | 92.64 | 0.000000e+00 | \* |
| 286 | 11 | A | A | C | 1.84 | 4.568992e-01 |  |
| 286 | 11 | A | A | G | 1.84 | 4.016290e-01 |  |
| 286 | 11 | A | A | T | 3.68 | 1.207421e-01 |  |
| 287 | 12 | G | G | A | 3.76 | 2.783398e-01 |  |
| 287 | 12 | G | G | C | 1.25 | 6.859539e-01 |  |
| 287 | 12 | G | G | G | 94.67 | 0.000000e+00 | \* |
| 287 | 12 | G | G | T | 0.31 | 8.712643e-01 |  |
| 288 | 13 | A | A | A | 94.10 | 0.000000e+00 | \* |
| 288 | 13 | A | A | C | 1.06 | 7.590685e-01 |  |
| 288 | 13 | A | A | G | 1.06 | 6.664321e-01 |  |
| 288 | 13 | A | A | T | 3.78 | 1.107093e-01 |  |
| 289 | 14 | C | C | A | 4.19 | 2.284208e-01 |  |
| 289 | 14 | C | C | C | 92.36 | 0.000000e+00 | \* |
| 289 | 14 | C | C | G | 2.46 | 2.477183e-01 |  |
| 289 | 14 | C | C | T | 0.99 | 7.406556e-01 |  |
| 290 | 15 | C | C | A | 5.88 | 9.766159e-02 |  |
| 290 | 15 | C | C | C | 89.30 | 0.000000e+00 | \* |
| 290 | 15 | C | C | G | 2.67 | 2.080252e-01 |  |
| 290 | 15 | C | C | T | 2.14 | 3.972271e-01 |  |
| 291 | 16 | G | G | A | 5.32 | 1.307316e-01 |  |
| 291 | 16 | G | G | C | 1.60 | 5.504226e-01 |  |
| 291 | 16 | G | G | G | 91.49 | 0.000000e+00 | \* |
| 291 | 16 | G | G | T | 1.60 | 5.549782e-01 |  |
| 292 | 17 | T | T | A | 3.39 | 3.286280e-01 |  |
| 292 | 17 | T | T | C | 0.34 | 9.211424e-01 |  |
| 292 | 17 | T | T | G | 2.03 | 3.477017e-01 |  |
| 292 | 17 | T | T | T | 94.24 | 0.000000e+00 | \* |
| 293 | 18 | G | G | A | 3.84 | 2.686283e-01 |  |
| 293 | 18 | G | G | C | 0.00 | 9.285714e-01 |  |
| 293 | 18 | G | G | G | 95.29 | 0.000000e+00 | \* |
| 293 | 18 | G | G | T | 0.87 | 7.709848e-01 |  |
| 294 | 19 | T | T | A | 1.87 | 5.929670e-01 |  |
| 294 | 19 | T | T | C | 0.00 | 9.285714e-01 |  |
| 294 | 19 | T | T | G | 4.27 | 4.933723e-02 |  |
| 294 | 19 | T | T | T | 93.87 | 0.000000e+00 | \* |
| 295 | 20 | T | T | A | 0.74 | 7.877710e-01 |  |
| 295 | 20 | T | T | C | 0.74 | 8.573489e-01 |  |
| 295 | 20 | T | T | G | 2.72 | 2.007544e-01 |  |
| 295 | 20 | T | T | T | 95.80 | 0.000000e+00 | \* |
| 296 | 21 | T | T | A | 0.00 | 8.363636e-01 |  |
| 296 | 21 | T | T | C | 0.00 | 9.285714e-01 |  |
| 296 | 21 | T | T | G | 2.49 | 2.415670e-01 |  |
| 296 | 21 | T | T | T | 97.51 | 0.000000e+00 | \* |
| 297 | 22 | G | G | A | 2.71 | 4.369332e-01 |  |
| 297 | 22 | G | G | C | 1.93 | 4.231032e-01 |  |
| 297 | 22 | G | G | G | 94.58 | 0.000000e+00 | \* |
| 297 | 22 | G | G | T | 0.77 | 7.954733e-01 |  |
| 298 | 23 | T | T | A | 0.00 | 8.363636e-01 |  |
| 298 | 23 | T | T | C | 0.87 | 8.202020e-01 |  |
| 298 | 23 | T | T | G | 1.46 | 5.237975e-01 |  |
| 298 | 23 | T | T | T | 97.67 | 0.000000e+00 | \* |
| 299 | 24 | G | G | A | 3.29 | 3.437375e-01 |  |
| 299 | 24 | G | G | C | 1.64 | 5.318216e-01 |  |
| 299 | 24 | G | G | G | 92.72 | 0.000000e+00 | \* |
| 299 | 24 | G | G | T | 2.35 | 3.443403e-01 |  |
| 300 | 25 | C | C | A | 2.08 | 5.525467e-01 |  |
| 300 | 25 | C | C | C | 93.47 | 0.000000e+00 | \* |
| 300 | 25 | C | C | G | 1.48 | 5.149369e-01 |  |
| 300 | 25 | C | C | T | 2.97 | 2.168874e-01 |  |
| 301 | 26 | A | A | A | 87.97 | 0.000000e+00 | \* |
| 301 | 26 | A | A | C | 3.78 | 6.085935e-02 |  |
| 301 | 26 | A | A | G | 4.47 | 4.070714e-02 |  |
| 301 | 26 | A | A | T | 3.78 | 1.109078e-01 |  |
| 302 | 27 | C | C | A | 2.73 | 4.332027e-01 |  |
| 302 | 27 | C | C | C | 94.54 | 0.000000e+00 | \* |
| 302 | 27 | C | C | G | 0.00 | 9.230769e-01 |  |
| 302 | 27 | C | C | T | 2.73 | 2.605098e-01 |  |
| 303 | 28 | T | T | A | 1.57 | 6.505886e-01 |  |
| 303 | 28 | T | T | C | 3.80 | 5.918466e-02 |  |
| 303 | 28 | T | T | G | 2.01 | 3.531481e-01 |  |
| 303 | 28 | T | T | T | 92.62 | 0.000000e+00 | \* |
| 304 | 29 | A | A | A | 93.66 | 0.000000e+00 | \* |
| 304 | 29 | A | A | C | 1.93 | 4.251766e-01 |  |
| 304 | 29 | A | A | G | 1.65 | 4.591049e-01 |  |
| 304 | 29 | A | A | T | 2.75 | 2.555653e-01 |  |
| 305 | 30 | C | C | A | 2.27 | 5.155458e-01 |  |
| 305 | 30 | C | C | C | 92.68 | 0.000000e+00 | \* |
| 305 | 30 | C | C | G | 2.02 | 3.513379e-01 |  |
| 305 | 30 | C | C | T | 3.03 | 2.063916e-01 |  |
| 306 | 31 | A | A | A | 89.08 | 0.000000e+00 | \* |
| 306 | 31 | A | A | C | 2.39 | 2.807752e-01 |  |
| 306 | 31 | A | A | G | 4.44 | 4.191911e-02 |  |
| 306 | 31 | A | A | T | 4.10 | 8.419760e-02 |  |
| 307 | 32 | C | C | A | 1.40 | 6.822297e-01 |  |
| 307 | 32 | C | C | C | 92.46 | 0.000000e+00 | \* |
| 307 | 32 | C | C | G | 2.23 | 2.976046e-01 |  |
| 307 | 32 | C | C | T | 3.91 | 9.904638e-02 |  |
| 308 | 33 | G | G | A | 3.01 | 3.868698e-01 |  |
| 308 | 33 | G | G | C | 2.26 | 3.185430e-01 |  |
| 308 | 33 | G | G | G | 92.86 | 0.000000e+00 | \* |
| 308 | 33 | G | G | T | 1.88 | 4.694921e-01 |  |
| 309 | 34 | G | G | A | 1.58 | 6.481339e-01 |  |
| 309 | 34 | G | G | C | 2.11 | 3.651467e-01 |  |
| 309 | 34 | G | G | G | 95.79 | 0.000000e+00 | \* |
| 309 | 34 | G | G | T | 0.53 | 8.451079e-01 |  |
| 310 | 35 | G | G | A | 3.75 | 2.798330e-01 |  |
| 310 | 35 | G | G | C | 1.88 | 4.443046e-01 |  |
| 310 | 35 | G | G | G | 94.06 | 0.000000e+00 | \* |
| 310 | 35 | G | G | T | 0.31 | 8.713459e-01 |  |
| 311 | 36 | C | C | A | 3.17 | 3.614685e-01 |  |
| 311 | 36 | C | C | C | 92.25 | 0.000000e+00 | \* |
| 311 | 36 | C | C | G | 1.41 | 5.408134e-01 |  |
| 311 | 36 | C | C | T | 3.17 | 1.847398e-01 |  |
| 312 | 37 | A | A | A | 89.34 | 0.000000e+00 | \* |
| 312 | 37 | A | A | C | 2.82 | 1.813023e-01 |  |
| 312 | 37 | A | A | G | 2.51 | 2.387914e-01 |  |
| 312 | 37 | A | A | T | 5.33 | 2.699672e-02 |  |
| 313 | 38 | C | C | A | 2.28 | 5.145774e-01 |  |
| 313 | 38 | C | C | C | 94.99 | 0.000000e+00 | \* |
| 313 | 38 | C | C | G | 0.91 | 7.200188e-01 |  |
| 313 | 38 | C | C | T | 1.82 | 4.863175e-01 |  |
| 314 | 39 | C | C | A | 5.37 | 1.276479e-01 |  |
| 314 | 39 | C | C | C | 89.51 | 0.000000e+00 | \* |
| 314 | 39 | C | C | G | 3.17 | 1.354648e-01 |  |
| 314 | 39 | C | C | T | 1.95 | 4.489137e-01 |  |
| 315 | 40 | C | C | A | 5.15 | 1.421305e-01 |  |
| 315 | 40 | C | C | C | 89.95 | 0.000000e+00 | \* |
| 315 | 40 | C | C | G | 1.29 | 5.831026e-01 |  |
| 315 | 40 | C | C | T | 3.61 | 1.284459e-01 |  |
| 316 | 41 | T | T | A | 1.93 | 5.803155e-01 |  |
| 316 | 41 | T | T | C | 2.17 | 3.433471e-01 |  |
| 316 | 41 | T | T | G | 2.17 | 3.121305e-01 |  |
| 316 | 41 | T | T | T | 93.72 | 0.000000e+00 | \* |
| 317 | 42 | G | G | A | 3.31 | 3.398580e-01 |  |
| 317 | 42 | G | G | C | 1.86 | 4.485355e-01 |  |
| 317 | 42 | G | G | G | 93.58 | 0.000000e+00 | \* |
| 317 | 42 | G | G | T | 1.24 | 6.648265e-01 |  |
| 318 | 43 | A | A | A | 82.01 | 0.000000e+00 | \* |
| 318 | 43 | A | A | C | 1.20 | 7.071466e-01 |  |
| 318 | 43 | A | A | G | 2.16 | 3.159595e-01 |  |
| 318 | 43 | A | A | T | 14.63 | 1.345370e-06 | \* |
| 319 | 44 | C | C | A | 2.58 | 4.596028e-01 |  |
| 319 | 44 | C | C | C | 97.42 | 0.000000e+00 | \* |
| 319 | 44 | C | C | G | 0.00 | 9.230769e-01 |  |
| 319 | 44 | C | C | T | 0.00 | 8.817204e-01 |  |
| 320 | 45 | C | C | A | 7.26 | 4.639343e-02 |  |
| 320 | 45 | C | C | C | 86.87 | 0.000000e+00 | \* |
| 320 | 45 | C | C | G | 4.75 | 3.099611e-02 |  |
| 320 | 45 | C | C | T | 1.12 | 7.025592e-01 |  |
| 321 | 46 | G | G | A | 6.62 | 6.590816e-02 |  |
| 321 | 46 | G | G | C | 2.09 | 3.699173e-01 |  |
| 321 | 46 | G | G | G | 90.94 | 0.000000e+00 | \* |
| 321 | 46 | G | G | T | 0.35 | 8.681195e-01 |  |
| 322 | 47 | A | A | A | 94.86 | 0.000000e+00 | \* |
| 322 | 47 | A | A | C | 0.95 | 7.956500e-01 |  |
| 322 | 47 | A | A | G | 0.57 | 8.328713e-01 |  |
| 322 | 47 | A | A | T | 3.62 | 1.272752e-01 |  |
| 323 | 48 | C | C | A | 3.86 | 2.664014e-01 |  |
| 323 | 48 | C | C | C | 92.58 | 0.000000e+00 | \* |
| 323 | 48 | C | C | G | 0.89 | 7.275058e-01 |  |
| 323 | 48 | C | C | T | 2.67 | 2.723132e-01 |  |
| 324 | 49 | G | G | A | 9.63 | 1.203562e-02 |  |
| 324 | 49 | G | G | C | 2.29 | 3.074588e-01 |  |
| 324 | 49 | G | G | G | 86.70 | 0.000000e+00 | \* |
| 324 | 49 | G | G | T | 1.38 | 6.233571e-01 |  |
| 325 | 50 | G | G | A | 3.17 | 3.606101e-01 |  |
| 325 | 50 | G | G | C | 0.79 | 8.435122e-01 |  |
| 325 | 50 | G | G | G | 94.71 | 0.000000e+00 | \* |
| 325 | 50 | G | G | T | 1.32 | 6.399699e-01 |  |
| 326 | 51 | C | C | A | 3.62 | 2.967750e-01 |  |
| 326 | 51 | C | C | C | 90.95 | 0.000000e+00 | \* |
| 326 | 51 | C | C | G | 2.71 | 2.009438e-01 |  |
| 326 | 51 | C | C | T | 2.71 | 2.633970e-01 |  |
| 327 | 52 | A | A | A | 88.83 | 0.000000e+00 | \* |
| 327 | 52 | A | A | C | 1.02 | 7.744518e-01 |  |
| 327 | 52 | A | A | G | 3.05 | 1.512140e-01 |  |
| 327 | 52 | A | A | T | 7.11 | 4.657749e-03 | \* |
| 328 | 53 | A | A | A | 94.66 | 0.000000e+00 | \* |
| 328 | 53 | A | A | C | 0.30 | 9.236848e-01 |  |
| 328 | 53 | A | A | G | 1.19 | 6.197898e-01 |  |
| 328 | 53 | A | A | T | 3.86 | 1.037215e-01 |  |
| 329 | 54 | G | G | A | 2.26 | 5.171514e-01 |  |
| 329 | 54 | G | G | C | 2.64 | 2.185609e-01 |  |
| 329 | 54 | G | G | G | 95.09 | 0.000000e+00 | \* |
| 329 | 54 | G | G | T | 0.00 | 8.817204e-01 |  |
| 330 | 55 | A | A | A | 94.35 | 0.000000e+00 | \* |
| 330 | 55 | A | A | C | 0.28 | 9.243936e-01 |  |
| 330 | 55 | A | A | G | 2.54 | 2.320917e-01 |  |
| 330 | 55 | A | A | T | 2.82 | 2.422571e-01 |  |
| 331 | 56 | A | A | A | 95.13 | 0.000000e+00 | \* |
| 331 | 56 | A | A | C | 0.00 | 9.285714e-01 |  |
| 331 | 56 | A | A | G | 1.30 | 5.795142e-01 |  |
| 331 | 56 | A | A | T | 3.57 | 1.325085e-01 |  |
| 332 | 57 | G | G | A | 1.88 | 5.907964e-01 |  |
| 332 | 57 | G | G | C | 1.41 | 6.248854e-01 |  |
| 332 | 57 | G | G | G | 93.90 | 0.000000e+00 | \* |
| 332 | 57 | G | G | T | 2.82 | 2.437409e-01 |  |
| 333 | 58 | T | T | A | 0.00 | 8.363636e-01 |  |
| 333 | 58 | T | T | C | 3.37 | 9.911806e-02 |  |
| 333 | 58 | T | T | G | 1.01 | 6.842564e-01 |  |
| 333 | 58 | T | T | T | 95.62 | 0.000000e+00 | \* |
| 334 | 59 | T | T | A | 1.52 | 6.584454e-01 |  |
| 334 | 59 | T | T | C | 1.52 | 5.786655e-01 |  |
| 334 | 59 | T | T | G | 3.35 | 1.150774e-01 |  |
| 334 | 59 | T | T | T | 93.60 | 0.000000e+00 | \* |
| 335 | 60 | C | C | A | 3.86 | 2.662846e-01 |  |
| 335 | 60 | C | C | C | 91.64 | 0.000000e+00 | \* |
| 335 | 60 | C | C | G | 3.22 | 1.302022e-01 |  |
| 335 | 60 | C | C | T | 1.29 | 6.512987e-01 |  |
| 336 | 61 | G | G | A | 7.82 | 3.403561e-02 |  |
| 336 | 61 | G | G | C | 2.47 | 2.597482e-01 |  |
| 336 | 61 | G | G | G | 89.30 | 0.000000e+00 | \* |
| 336 | 61 | G | G | T | 0.41 | 8.612880e-01 |  |
| 337 | 62 | A | A | A | 94.15 | 0.000000e+00 | \* |
| 337 | 62 | A | A | C | 1.41 | 6.262005e-01 |  |
| 337 | 62 | A | A | G | 1.64 | 4.634428e-01 |  |
| 337 | 62 | A | A | T | 2.81 | 2.449765e-01 |  |
| 338 | 63 | C | C | A | 2.55 | 4.648159e-01 |  |
| 338 | 63 | C | C | C | 94.05 | 0.000000e+00 | \* |
| 338 | 63 | C | C | G | 1.98 | 3.613550e-01 |  |
| 338 | 63 | C | C | T | 1.42 | 6.107894e-01 |  |
| 339 | 64 | A | A | A | 91.42 | 0.000000e+00 | \* |
| 339 | 64 | A | A | C | 1.72 | 5.033780e-01 |  |
| 339 | 64 | A | A | G | 2.58 | 2.258876e-01 |  |
| 339 | 64 | A | A | T | 4.29 | 7.068138e-02 |  |
| 340 | 65 | G | G | A | 3.24 | 3.501825e-01 |  |
| 340 | 65 | G | G | C | 2.70 | 2.052454e-01 |  |
| 340 | 65 | G | G | G | 92.70 | 0.000000e+00 | \* |
| 340 | 65 | G | G | T | 1.35 | 6.310804e-01 |  |
| 341 | 66 | C | C | A | 2.84 | 4.149333e-01 |  |
| 341 | 66 | C | C | C | 91.13 | 0.000000e+00 | \* |
| 341 | 66 | C | C | G | 2.48 | 2.438546e-01 |  |
| 341 | 66 | C | C | T | 3.55 | 1.353685e-01 |  |
| 342 | 67 | T | T | A | 0.00 | 8.363636e-01 |  |
| 342 | 67 | T | T | C | 1.47 | 6.000949e-01 |  |
| 342 | 67 | T | T | G | 1.72 | 4.393206e-01 |  |
| 342 | 67 | T | T | T | 96.81 | 0.000000e+00 | \* |
| 343 | 68 | C | C | A | 2.51 | 4.723127e-01 |  |
| 343 | 68 | C | C | C | 93.73 | 0.000000e+00 | \* |
| 343 | 68 | C | C | G | 2.19 | 3.071795e-01 |  |
| 343 | 68 | C | C | T | 1.57 | 5.637527e-01 |  |
| 344 | 69 | C | C | A | 2.18 | 5.321500e-01 |  |
| 344 | 69 | C | C | C | 93.69 | 0.000000e+00 | \* |
| 344 | 69 | C | C | G | 0.73 | 7.835859e-01 |  |
| 344 | 69 | C | C | T | 3.40 | 1.531887e-01 |  |
| 345 | 70 | C | C | A | 0.00 | 8.363636e-01 |  |
| 345 | 70 | C | C | C | 97.36 | 0.000000e+00 | \* |
| 345 | 70 | C | C | G | 1.13 | 6.397613e-01 |  |
| 345 | 70 | C | C | T | 1.51 | 5.817700e-01 |  |
| 346 | 71 | G | G | A | 6.20 | 8.250235e-02 |  |
| 346 | 71 | G | G | C | 1.94 | 4.217800e-01 |  |
| 346 | 71 | G | G | G | 89.53 | 0.000000e+00 | \* |
| 346 | 71 | G | G | T | 2.33 | 3.496497e-01 |  |
| 347 | 72 | C | C | A | 3.56 | 3.050103e-01 |  |
| 347 | 72 | C | C | C | 92.53 | 0.000000e+00 | \* |
| 347 | 72 | C | C | G | 0.71 | 7.890236e-01 |  |
| 347 | 72 | C | C | T | 3.20 | 1.797580e-01 |  |
| 348 | 73 | G | G | A | 9.02 | 1.721403e-02 |  |
| 348 | 73 | G | G | C | 3.69 | 6.794191e-02 |  |
| 348 | 73 | G | G | G | 87.30 | 0.000000e+00 | \* |
| 348 | 73 | G | G | T | 0.00 | 8.817204e-01 |  |
| 349 | 74 | A | A | A | 94.75 | 0.000000e+00 | \* |
| 349 | 74 | A | A | C | 1.66 | 5.262642e-01 |  |
| 349 | 74 | A | A | G | 0.83 | 7.492127e-01 |  |
| 349 | 74 | A | A | T | 2.76 | 2.540921e-01 |  |
| 350 | 75 | C | C | A | 2.52 | 4.699406e-01 |  |
| 350 | 75 | C | C | C | 93.56 | 0.000000e+00 | \* |
| 350 | 75 | C | C | G | 0.00 | 9.230769e-01 |  |
| 350 | 75 | C | C | T | 3.92 | 9.810497e-02 |  |

## For use in R

If you want to work with the results in R, here is output that you can copy and paste in your terminal to get:

The base information:

```
structure(list(focal.base = c("A", "C", "G", "T"), avg.percsignal = c(92.2905299425723, 
93.2944717004083, 92.8864575254146, 94.7329828599931), avg.areasignal = c(352.088235294118, 
371.746268656716, 355.035714285714, 429.704545454545), crit.perc.area = c(9.94958832514891, 
5.18815337373941, 5.88457608687205, 6.3473837034962), mu = c(3.2792795086245, 
2.0036156749508, 1.91281113702503, 2.24176252469423), fillibens = c(0.995734429626359, 
0.996629713834685, 0.994560994808505, 0.991907808064103)), .Names = c("focal.base", 
"avg.percsignal", "avg.areasignal", "crit.perc.area", "mu", "fillibens"
), row.names = c(NA, -4L), class = "data.frame")
```

the data.frame that contains information on the guide region:

```
structure(list(A.area = c(310, 381, 9, 625, 23, 23, 29, 21, 10, 
9, 302, 12, 622, 17, 22, 20, 10, 22, 7, 3, 0, 14, 0, 14, 7, 256, 
11, 7, 340, 9, 261, 5, 8, 6, 12, 9, 285, 10, 22, 20, 8, 16, 342, 
9, 26, 19, 498, 13, 21, 12, 8, 175, 319, 6, 334, 293, 4, 0, 5, 
12, 19, 402, 9, 213, 12, 8, 0, 8, 9, 0, 16, 10, 22, 343, 9), 
    C.area = c(6, 5, 11, 7, 373, 7, 0, 10, 241, 419, 6, 4, 7, 
    375, 334, 6, 1, 0, 0, 3, 0, 10, 3, 7, 315, 11, 381, 17, 7, 
    367, 7, 331, 6, 8, 6, 262, 9, 417, 367, 349, 9, 9, 5, 340, 
    311, 6, 5, 312, 5, 3, 201, 2, 1, 7, 1, 0, 3, 10, 5, 285, 
    6, 6, 332, 4, 10, 257, 6, 299, 386, 258, 5, 260, 9, 6, 334
    ), G.area = c(2, 6, 297, 8, 0, 12, 647, 420, 5, 13, 6, 302, 
    7, 10, 10, 344, 6, 546, 16, 11, 10, 489, 5, 395, 5, 13, 0, 
    9, 6, 8, 13, 8, 247, 364, 301, 4, 8, 4, 13, 5, 9, 452, 9, 
    0, 17, 261, 3, 3, 189, 358, 6, 6, 4, 252, 9, 4, 200, 3, 11, 
    10, 217, 7, 7, 6, 343, 7, 7, 7, 3, 3, 231, 2, 213, 3, 0), 
    T.area = c(11, 16, 0, 25, 9, 456, 8, 3, 6, 13, 12, 1, 25, 
    4, 8, 6, 278, 5, 352, 388, 391, 4, 335, 10, 10, 11, 11, 414, 
    10, 12, 12, 14, 5, 2, 1, 9, 17, 8, 8, 14, 388, 6, 61, 0, 
    4, 1, 19, 9, 3, 5, 6, 14, 13, 0, 10, 11, 6, 284, 307, 4, 
    1, 12, 5, 10, 5, 10, 395, 5, 14, 4, 6, 9, 0, 10, 14), Tot.area = c(329, 
    408, 317, 665, 405, 498, 684, 454, 262, 454, 326, 319, 661, 
    406, 374, 376, 295, 573, 375, 405, 401, 517, 343, 426, 337, 
    291, 403, 447, 363, 396, 293, 358, 266, 380, 320, 284, 319, 
    439, 410, 388, 414, 483, 417, 349, 358, 287, 525, 337, 218, 
    378, 221, 197, 337, 265, 354, 308, 213, 297, 328, 311, 243, 
    427, 353, 233, 370, 282, 408, 319, 412, 265, 258, 281, 244, 
    362, 357), A.perc = c(94.2249240121581, 93.3823529411765, 
    2.8391167192429, 93.984962406015, 5.67901234567901, 4.61847389558233, 
    4.23976608187134, 4.62555066079295, 3.81679389312977, 1.98237885462555, 
    92.638036809816, 3.76175548589342, 94.0998487140696, 4.1871921182266, 
    5.88235294117647, 5.31914893617021, 3.38983050847458, 3.83944153577661, 
    1.86666666666667, 0.740740740740741, 0, 2.70793036750484, 
    0, 3.28638497652582, 2.07715133531157, 87.9725085910653, 
    2.72952853598015, 1.56599552572707, 93.66391184573, 2.27272727272727, 
    89.0784982935154, 1.39664804469274, 3.00751879699248, 1.57894736842105, 
    3.75, 3.16901408450704, 89.3416927899686, 2.27790432801822, 
    5.36585365853659, 5.15463917525773, 1.93236714975845, 3.31262939958592, 
    82.0143884892086, 2.57879656160458, 7.26256983240223, 6.62020905923345, 
    94.8571428571429, 3.85756676557863, 9.63302752293578, 3.17460317460317, 
    3.61990950226244, 88.8324873096447, 94.6587537091988, 2.26415094339623, 
    94.3502824858757, 95.1298701298701, 1.87793427230047, 0, 
    1.52439024390244, 3.85852090032154, 7.81893004115226, 94.1451990632319, 
    2.54957507082153, 91.4163090128755, 3.24324324324324, 2.83687943262411, 
    0, 2.50783699059561, 2.18446601941748, 0, 6.2015503875969, 
    3.55871886120996, 9.01639344262295, 94.7513812154696, 2.52100840336134
    ), C.perc = c(1.82370820668693, 1.22549019607843, 3.47003154574133, 
    1.05263157894737, 92.0987654320988, 1.40562248995984, 0, 
    2.20264317180617, 91.9847328244275, 92.2907488986784, 1.84049079754601, 
    1.25391849529781, 1.0590015128593, 92.3645320197044, 89.3048128342246, 
    1.59574468085106, 0.338983050847458, 0, 0, 0.740740740740741, 
    0, 1.93423597678917, 0.87463556851312, 1.64319248826291, 
    93.4718100890208, 3.78006872852234, 94.5409429280397, 3.80313199105145, 
    1.92837465564738, 92.6767676767677, 2.38907849829352, 92.4581005586592, 
    2.25563909774436, 2.10526315789474, 1.875, 92.2535211267606, 
    2.82131661442006, 94.9886104783599, 89.5121951219512, 89.9484536082474, 
    2.17391304347826, 1.86335403726708, 1.19904076738609, 97.4212034383954, 
    86.8715083798883, 2.09059233449477, 0.952380952380952, 92.5816023738872, 
    2.29357798165138, 0.793650793650794, 90.9502262443439, 1.01522842639594, 
    0.29673590504451, 2.64150943396226, 0.282485875706215, 0, 
    1.40845070422535, 3.36700336700337, 1.52439024390244, 91.6398713826367, 
    2.46913580246914, 1.40515222482436, 94.0509915014164, 1.71673819742489, 
    2.7027027027027, 91.1347517730496, 1.47058823529412, 93.730407523511, 
    93.6893203883495, 97.3584905660377, 1.93798449612403, 92.5266903914591, 
    3.68852459016393, 1.65745856353591, 93.5574229691877), G.perc = c(0.60790273556231, 
    1.47058823529412, 93.6908517350158, 1.20300751879699, 0, 
    2.40963855421687, 94.5906432748538, 92.511013215859, 1.90839694656489, 
    2.86343612334802, 1.84049079754601, 94.6708463949843, 1.0590015128593, 
    2.46305418719212, 2.67379679144385, 91.4893617021277, 2.03389830508475, 
    95.2879581151832, 4.26666666666667, 2.71604938271605, 2.49376558603491, 
    94.5841392649903, 1.45772594752187, 92.7230046948357, 1.48367952522255, 
    4.46735395189003, 0, 2.01342281879195, 1.65289256198347, 
    2.02020202020202, 4.43686006825939, 2.23463687150838, 92.8571428571429, 
    95.7894736842105, 94.0625, 1.40845070422535, 2.50783699059561, 
    0.911161731207289, 3.17073170731707, 1.28865979381443, 2.17391304347826, 
    93.5817805383023, 2.15827338129496, 0, 4.74860335195531, 
    90.9407665505227, 0.571428571428571, 0.890207715133531, 86.697247706422, 
    94.7089947089947, 2.71493212669683, 3.04568527918782, 1.18694362017804, 
    95.0943396226415, 2.54237288135593, 1.2987012987013, 93.8967136150235, 
    1.01010101010101, 3.35365853658537, 3.21543408360129, 89.3004115226338, 
    1.63934426229508, 1.98300283286119, 2.57510729613734, 92.7027027027027, 
    2.4822695035461, 1.7156862745098, 2.19435736677116, 0.728155339805825, 
    1.13207547169811, 89.5348837209302, 0.711743772241993, 87.2950819672131, 
    0.828729281767956, 0), T.perc = c(3.34346504559271, 3.92156862745098, 
    0, 3.7593984962406, 2.22222222222222, 91.566265060241, 1.16959064327485, 
    0.66079295154185, 2.29007633587786, 2.86343612334802, 3.68098159509202, 
    0.313479623824451, 3.7821482602118, 0.985221674876847, 2.13903743315508, 
    1.59574468085106, 94.2372881355932, 0.87260034904014, 93.8666666666667, 
    95.8024691358025, 97.5062344139651, 0.773694390715667, 97.667638483965, 
    2.34741784037559, 2.9673590504451, 3.78006872852234, 2.72952853598015, 
    92.6174496644295, 2.75482093663912, 3.03030303030303, 4.09556313993174, 
    3.91061452513966, 1.8796992481203, 0.526315789473684, 0.3125, 
    3.16901408450704, 5.32915360501567, 1.82232346241458, 1.95121951219512, 
    3.60824742268041, 93.719806763285, 1.24223602484472, 14.6282973621103, 
    0, 1.11731843575419, 0.348432055749129, 3.61904761904762, 
    2.67062314540059, 1.37614678899083, 1.32275132275132, 2.71493212669683, 
    7.10659898477157, 3.85756676557863, 0, 2.82485875706215, 
    3.57142857142857, 2.8169014084507, 95.6228956228956, 93.5975609756098, 
    1.28617363344051, 0.411522633744856, 2.81030444964871, 1.41643059490085, 
    4.29184549356223, 1.35135135135135, 3.54609929078014, 96.8137254901961, 
    1.56739811912226, 3.39805825242718, 1.50943396226415, 2.32558139534884, 
    3.20284697508897, 0, 2.76243093922652, 3.92156862745098), 
    base.call = c("A", "A", "G", "A", "C", "T", "G", "G", "C", 
    "C", "A", "G", "A", "C", "C", "G", "T", "G", "T", "T", "T", 
    "G", "T", "G", "C", "A", "C", "T", "A", "C", "A", "C", "G", 
    "G", "G", "C", "A", "C", "C", "C", "T", "G", "A", "C", "C", 
    "G", "A", "C", "G", "G", "C", "A", "A", "G", "A", "A", "G", 
    "T", "T", "C", "G", "A", "C", "A", "G", "C", "T", "C", "C", 
    "C", "G", "C", "G", "A", "C"), index = 276:350, guide.seq = c("A", 
    "A", "G", "A", "C", "T", "G", "G", "C", "C", "A", "G", "A", 
    "C", "C", "G", "T", "G", "T", "T", "T", "G", "T", "G", "C", 
    "A", "C", "T", "A", "C", "A", "C", "G", "G", "G", "C", "A", 
    "C", "C", "C", "T", "G", "A", "C", "C", "G", "A", "C", "G", 
    "G", "C", "A", "A", "G", "A", "A", "G", "T", "T", "C", "G", 
    "A", "C", "A", "G", "C", "T", "C", "C", "C", "G", "C", "G", 
    "A", "C"), T.pval = c(0.160255564157252, 0.098104967208, 
    0.881720430107452, 0.112898265780212, 0.375526771359895, 
    0, 0.686927121058451, 0.820407472329734, 0.358400703272699, 
    0.235165155274692, 0.12074214551544, 0.871264309763626, 0.110709289443403, 
    0.740655629509951, 0.397227094759649, 0.554978227279565, 
    0, 0.770984830300088, 0, 0, 0, 0.79547329845375, 0, 0.344340261040977, 
    0.216887408844569, 0.110907795290004, 0.260509826946116, 
    0, 0.255565340677559, 0.206391648404137, 0.0841975962548307, 
    0.0990463787047747, 0.469492089311955, 0.845107852530592, 
    0.871345899192207, 0.184739803458708, 0.0269967200787368, 
    0.486317523886303, 0.448913737304717, 0.128445859008985, 
    0, 0.664826480722198, 1.34536954166098e-06, 0.881720430107452, 
    0.70255922034873, 0.868119532399202, 0.12727517550396, 0.27231322111524, 
    0.623357106437723, 0.63996990068191, 0.263397022207679, 0.00465774909543926, 
    0.103721484103189, 0.881720430107452, 0.242257080247167, 
    0.132508459201138, 0.243740892355124, 0, 0, 0.651298705898863, 
    0.861288030848853, 0.244976490627438, 0.610789394375677, 
    0.0706813832067937, 0.631080372723421, 0.135368492364958, 
    0, 0.563752660126351, 0.153188724784051, 0.581769974276345, 
    0.349649743347885, 0.179757995441976, 0.881720430107452, 
    0.254092088011152, 0.098104967208), C.pval = c(0.463085729950252, 
    0.696978449924022, 0.0879605775947089, 0.761336002521573, 
    0, 0.626013003731402, 0.928571428571429, 0.334483932799867, 
    0, 0, 0.456899227023045, 0.685953947140278, 0.75906845269433, 
    0, 0, 0.550422635290708, 0.921142410585117, 0.928571428571429, 
    0.928571428571429, 0.857348892945994, 0.928571428571429, 
    0.423103178412507, 0.820201957470621, 0.53182156670121, 0, 
    0.060859345390497, 0, 0.0591846597287684, 0.425176608024565, 
    0, 0.280775166923628, 0, 0.318542992659995, 0.365146667224127, 
    0.444304561884527, 0, 0.181302271314985, 0, 0, 0, 0.343347149496904, 
    0.448535545706745, 0.707146567156348, 0, 0, 0.369917292003877, 
    0.795649956222663, 0, 0.307458835991005, 0.843512235930084, 
    0, 0.774451829626653, 0.923684821343386, 0.218560877540407, 
    0.924393590619214, 0.928571428571429, 0.624885382427695, 
    0.0991180553767859, 0.57866547783203, 0, 0.259748234183339, 
    0.626200485736144, 0, 0.503378039060389, 0.205245405543076, 
    0, 0.600094883552585, 0, 0, 0, 0.421780027539721, 0, 0.0679419064833153, 
    0.526264245826149, 0), G.pval = c(0.821986857229023, 0.519397178990304, 
    0, 0.61396150419831, 0.923076923076922, 0.258725189842896, 
    0, 0, 0.382064995030434, 0.177081485021759, 0.401629026959429, 
    0, 0.66643213648435, 0.247718266980999, 0.208025187547202, 
    0, 0.34770166406619, 0, 0.0493372346555098, 0.200754374631969, 
    0.241567041430788, 0, 0.523797469811677, 0, 0.514936900078389, 
    0.0407071390363041, 0.923076923076922, 0.353148052203466, 
    0.459104871806655, 0.351337894994411, 0.0419191070718521, 
    0.297604635753103, 0, 0, 0, 0.540813408122673, 0.23879138049767, 
    0.720018817863155, 0.135464778400763, 0.583102633471296, 
    0.312130466197715, 0, 0.315959502421036, 0.923076923076922, 
    0.0309961141871753, 0, 0.832871298797563, 0.727505776266822, 
    0, 0, 0.200943786069033, 0.151214047096483, 0.619789801103156, 
    0, 0.23209165762461, 0.579514174668596, 0, 0.684256414643245, 
    0.115077411548035, 0.130202245723227, 0, 0.463442805377038, 
    0.361354982761127, 0.225887638820163, 0, 0.243854584361842, 
    0.439320560753815, 0.307179494008212, 0.783585890568694, 
    0.639761253029087, 0, 0.789023565402911, 0, 0.749212709168552, 
    0.923076923076922), A.pval = c(0, 0, 0.414557517388526, 0, 
    0.108605908305554, 0.185597443500528, 0.222794730537497, 
    0.184956059887004, 0.271431530960412, 0.570700377051131, 
    0, 0.278339804227521, 0, 0.228420805594629, 0.0976615882060285, 
    0.130731612681181, 0.328628011210238, 0.268628334392213, 
    0.592967034510256, 0.787770991621219, 0.836363636363636, 
    0.436933229373363, 0.836363636363636, 0.343737457709929, 
    0.552546687127822, 0, 0.433202711853452, 0.650588635686178, 
    0, 0.515545760605853, 0, 0.682229729005962, 0.386869800084968, 
    0.648133940502933, 0.279832994412239, 0.36146845973455, 0, 
    0.51457739421257, 0.12764785288366, 0.142130462861044, 0.580315540956635, 
    0.339858025006794, 0, 0.459602759227664, 0.0463934280215764, 
    0.0659081636760706, 0, 0.26640141685664, 0.0120356180704617, 
    0.360610067218834, 0.296774991717152, 0, 0, 0.517151389600419, 
    0, 0, 0.590796418400497, 0.836363636363636, 0.658445361538626, 
    0.266284595400301, 0.034035611529738, 0, 0.464815932486682, 
    0, 0.350182531188069, 0.414933333997659, 0.836363636363636, 
    0.472312709170125, 0.532149998802105, 0.836363636363636, 
    0.0825023527356316, 0.305010274372144, 0.0172140348334543, 
    0, 0.469940605164629), guide.position = 1:75), .Names = c("A.area", 
"C.area", "G.area", "T.area", "Tot.area", "A.perc", "C.perc", 
"G.perc", "T.perc", "base.call", "index", "guide.seq", "T.pval", 
"C.pval", "G.pval", "A.pval", "guide.position"), row.names = 276:350, class = "data.frame")
```

*Report generated using EditR v1.0.8*
