# Supplementary material for: Mechanistic and genetic basis of single-strand templated repair at Cas12a-induced DNA breaks in Chlamydomonas reinhardtii
Source: Nat Commun. 2021 Nov 19;12:6751. doi: 10.1038/s41467-021-27004-1 (PMC8604939; doi:10.1038/s41467-021-27004-1)
Supplement: Supplementary file 22 — Source Data [file 41467_2021_27004_MOESM22_ESM.zip › Source Data/EditR analysis/EditR outputs/Antisense/rep3_ssODN_antisense_0_16_32.html]

EditR v1.0.8 report


# EditR v1.0.8 report

- Data QA
  - Filtering data
  - Percent noise peak area
  - Base information
- Predicted editing
  - Editing bar plot
  - Editing table plot
  - Table of editing results
- For use in R

## Data QA

### Filtering data

What the data looked like prefiltering:

and the post filtering signal / noise plot:

### Percent noise peak area

### Base information

Here’s information about the signal of each base, the critical percent value where any higher value would be called as significant, and Filliben’s correlation for how well the noise was modelled by the zero adjusted gamma distribution.

| Base | Average percent signal | Average peak area | Critical percent value | model mu | Fillibens correlation |
| --- | --- | --- | --- | --- | --- |
| A | 92.92568 | 309.1176 | 10.393906 | 3.307645 | 0.9821031 |
| C | 92.91139 | 329.9254 | 5.445906 | 1.985375 | 0.9950451 |
| G | 93.39363 | 310.9649 | 4.947312 | 1.917259 | 0.9907799 |
| T | 94.25501 | 357.0465 | 6.546533 | 2.199967 | 0.9951610 |

## Predicted editing

### Editing bar plot

### Editing table plot

### Table of editing results


Here’s the entire guide region

| Sanger position | Guide position | Guide sequence | Sanger base call | Focal base | Focal base peak area | p value |  |
| --- | --- | --- | --- | --- | --- | --- | --- |
| 277 | 1 | A | A | A | 91.56 | 0.000000e+00 | \* |
| 277 | 1 | A | A | C | 1.56 | 5.487755e-01 |  |
| 277 | 1 | A | A | G | 2.19 | 3.074370e-01 |  |
| 277 | 1 | A | A | T | 4.69 | 5.378652e-02 |  |
| 278 | 2 | A | A | A | 93.75 | 0.000000e+00 | \* |
| 278 | 2 | A | A | C | 0.57 | 8.908179e-01 |  |
| 278 | 2 | A | A | G | 1.70 | 4.760964e-01 |  |
| 278 | 2 | A | A | T | 3.98 | 9.844875e-02 |  |
| 279 | 3 | G | G | A | 2.56 | 4.578998e-01 |  |
| 279 | 3 | G | G | C | 3.66 | 7.885245e-02 |  |
| 279 | 3 | G | G | G | 91.94 | 0.000000e+00 | \* |
| 279 | 3 | G | G | T | 1.83 | 4.813461e-01 |  |
| 280 | 4 | A | A | A | 93.81 | 0.000000e+00 | \* |
| 280 | 4 | A | A | C | 1.00 | 7.618963e-01 |  |
| 280 | 4 | A | A | G | 0.84 | 8.157956e-01 |  |
| 280 | 4 | A | A | T | 4.35 | 7.205792e-02 |  |
| 281 | 5 | C | C | A | 3.76 | 2.825936e-01 |  |
| 281 | 5 | C | C | C | 93.64 | 0.000000e+00 | \* |
| 281 | 5 | C | C | G | 0.00 | 9.222222e-01 |  |
| 281 | 5 | C | C | T | 2.60 | 2.881088e-01 |  |
| 282 | 6 | T | T | A | 4.33 | 2.198623e-01 |  |
| 282 | 6 | T | T | C | 2.80 | 1.922665e-01 |  |
| 282 | 6 | T | T | G | 2.54 | 2.128237e-01 |  |
| 282 | 6 | T | T | T | 90.33 | 0.000000e+00 | \* |
| 283 | 7 | G | G | A | 3.70 | 2.899514e-01 |  |
| 283 | 7 | G | G | C | 0.00 | 9.404762e-01 |  |
| 283 | 7 | G | G | G | 95.13 | 0.000000e+00 | \* |
| 283 | 7 | G | G | T | 1.18 | 6.860588e-01 |  |
| 284 | 8 | G | G | A | 4.58 | 1.958275e-01 |  |
| 284 | 8 | G | G | C | 2.04 | 3.830441e-01 |  |
| 284 | 8 | G | G | G | 92.88 | 0.000000e+00 | \* |
| 284 | 8 | G | G | T | 0.51 | 8.680722e-01 |  |
| 285 | 9 | C | C | A | 4.98 | 1.627052e-01 |  |
| 285 | 9 | C | C | C | 89.21 | 0.000000e+00 | \* |
| 285 | 9 | C | C | G | 2.07 | 3.428182e-01 |  |
| 285 | 9 | C | C | T | 3.73 | 1.202525e-01 |  |
| 286 | 10 | C | C | A | 3.04 | 3.812259e-01 |  |
| 286 | 10 | C | C | C | 93.16 | 0.000000e+00 | \* |
| 286 | 10 | C | C | G | 1.27 | 6.553657e-01 |  |
| 286 | 10 | C | C | T | 2.53 | 3.027547e-01 |  |
| 287 | 11 | A | A | A | 92.68 | 0.000000e+00 | \* |
| 287 | 11 | A | A | C | 2.09 | 3.659682e-01 |  |
| 287 | 11 | A | A | G | 2.44 | 2.380677e-01 |  |
| 287 | 11 | A | A | T | 2.79 | 2.515892e-01 |  |
| 288 | 12 | G | G | A | 3.28 | 3.449193e-01 |  |
| 288 | 12 | G | G | C | 1.46 | 5.880298e-01 |  |
| 288 | 12 | G | G | G | 95.26 | 0.000000e+00 | \* |
| 288 | 12 | G | G | T | 0.00 | 9.148936e-01 |  |
| 289 | 13 | A | A | A | 94.67 | 0.000000e+00 | \* |
| 289 | 13 | A | A | C | 1.20 | 6.875499e-01 |  |
| 289 | 13 | A | A | G | 0.69 | 8.577455e-01 |  |
| 289 | 13 | A | A | T | 3.44 | 1.528835e-01 |  |
| 290 | 14 | C | C | A | 4.02 | 2.518261e-01 |  |
| 290 | 14 | C | C | C | 90.88 | 0.000000e+00 | \* |
| 290 | 14 | C | C | G | 2.68 | 1.833966e-01 |  |
| 290 | 14 | C | C | T | 2.41 | 3.290881e-01 |  |
| 291 | 15 | C | C | A | 4.78 | 1.788147e-01 |  |
| 291 | 15 | C | C | C | 91.40 | 0.000000e+00 | \* |
| 291 | 15 | C | C | G | 2.23 | 2.949974e-01 |  |
| 291 | 15 | C | C | T | 1.59 | 5.537611e-01 |  |
| 292 | 16 | G | G | A | 3.94 | 2.610749e-01 |  |
| 292 | 16 | G | G | C | 1.52 | 5.667957e-01 |  |
| 292 | 16 | G | G | G | 93.03 | 0.000000e+00 | \* |
| 292 | 16 | G | G | T | 1.52 | 5.779799e-01 |  |
| 293 | 17 | T | T | A | 0.82 | 7.691677e-01 |  |
| 293 | 17 | T | T | C | 1.63 | 5.224258e-01 |  |
| 293 | 17 | T | T | G | 2.45 | 2.355903e-01 |  |
| 293 | 17 | T | T | T | 95.10 | 0.000000e+00 | \* |
| 294 | 18 | G | G | A | 3.68 | 2.918608e-01 |  |
| 294 | 18 | G | G | C | 0.78 | 8.373731e-01 |  |
| 294 | 18 | G | G | G | 93.80 | 0.000000e+00 | \* |
| 294 | 18 | G | G | T | 1.74 | 5.072553e-01 |  |
| 295 | 19 | T | T | A | 0.96 | 7.474311e-01 |  |
| 295 | 19 | T | T | C | 1.60 | 5.355932e-01 |  |
| 295 | 19 | T | T | G | 3.83 | 4.541288e-02 |  |
| 295 | 19 | T | T | T | 93.61 | 0.000000e+00 | \* |
| 296 | 20 | T | T | A | 0.31 | 8.258913e-01 |  |
| 296 | 20 | T | T | C | 0.00 | 9.404762e-01 |  |
| 296 | 20 | T | T | G | 2.45 | 2.362096e-01 |  |
| 296 | 20 | T | T | T | 97.25 | 0.000000e+00 | \* |
| 297 | 21 | T | T | A | 0.00 | 8.363636e-01 |  |
| 297 | 21 | T | T | C | 1.78 | 4.686764e-01 |  |
| 297 | 21 | T | T | G | 2.37 | 2.547757e-01 |  |
| 297 | 21 | T | T | T | 95.85 | 0.000000e+00 | \* |
| 298 | 22 | G | G | A | 3.37 | 3.328534e-01 |  |
| 298 | 22 | G | G | C | 0.00 | 9.404762e-01 |  |
| 298 | 22 | G | G | G | 95.73 | 0.000000e+00 | \* |
| 298 | 22 | G | G | T | 0.90 | 7.711988e-01 |  |
| 299 | 23 | T | T | A | 0.00 | 8.363636e-01 |  |
| 299 | 23 | T | T | C | 4.30 | 3.873332e-02 |  |
| 299 | 23 | T | T | G | 1.43 | 5.858535e-01 |  |
| 299 | 23 | T | T | T | 94.27 | 0.000000e+00 | \* |
| 300 | 24 | G | G | A | 3.71 | 2.879603e-01 |  |
| 300 | 24 | G | G | C | 3.18 | 1.309226e-01 |  |
| 300 | 24 | G | G | G | 92.04 | 0.000000e+00 | \* |
| 300 | 24 | G | G | T | 1.06 | 7.223360e-01 |  |
| 301 | 25 | C | C | A | 2.24 | 5.155801e-01 |  |
| 301 | 25 | C | C | C | 93.61 | 0.000000e+00 | \* |
| 301 | 25 | C | C | G | 1.28 | 6.503753e-01 |  |
| 301 | 25 | C | C | T | 2.88 | 2.356986e-01 |  |
| 302 | 26 | A | A | A | 86.97 | 0.000000e+00 | \* |
| 302 | 26 | A | A | C | 3.83 | 6.560576e-02 |  |
| 302 | 26 | A | A | G | 3.45 | 7.416973e-02 |  |
| 302 | 26 | A | A | T | 5.75 | 2.091860e-02 |  |
| 303 | 27 | C | C | A | 2.57 | 4.566498e-01 |  |
| 303 | 27 | C | C | C | 95.14 | 0.000000e+00 | \* |
| 303 | 27 | C | C | G | 0.29 | 9.174105e-01 |  |
| 303 | 27 | C | C | T | 2.00 | 4.334019e-01 |  |
| 304 | 28 | T | T | A | 1.71 | 6.133309e-01 |  |
| 304 | 28 | T | T | C | 3.70 | 7.544448e-02 |  |
| 304 | 28 | T | T | G | 1.14 | 7.064228e-01 |  |
| 304 | 28 | T | T | T | 93.45 | 0.000000e+00 | \* |
| 305 | 29 | A | A | A | 96.05 | 0.000000e+00 | \* |
| 305 | 29 | A | A | C | 1.52 | 5.650358e-01 |  |
| 305 | 29 | A | A | G | 1.22 | 6.758127e-01 |  |
| 305 | 29 | A | A | T | 1.22 | 6.735402e-01 |  |
| 306 | 30 | C | C | A | 1.78 | 5.999977e-01 |  |
| 306 | 30 | C | C | C | 94.66 | 0.000000e+00 | \* |
| 306 | 30 | C | C | G | 1.48 | 5.651772e-01 |  |
| 306 | 30 | C | C | T | 2.08 | 4.124384e-01 |  |
| 307 | 31 | A | A | A | 91.09 | 0.000000e+00 | \* |
| 307 | 31 | A | A | C | 2.83 | 1.858117e-01 |  |
| 307 | 31 | A | A | G | 3.64 | 5.798909e-02 |  |
| 307 | 31 | A | A | T | 2.43 | 3.253805e-01 |  |
| 308 | 32 | C | C | A | 2.35 | 4.953981e-01 |  |
| 308 | 32 | C | C | C | 93.29 | 0.000000e+00 | \* |
| 308 | 32 | C | C | G | 2.35 | 2.613913e-01 |  |
| 308 | 32 | C | C | T | 2.01 | 4.297083e-01 |  |
| 309 | 33 | G | G | A | 3.08 | 3.743032e-01 |  |
| 309 | 33 | G | G | C | 2.20 | 3.327610e-01 |  |
| 309 | 33 | G | G | G | 93.83 | 0.000000e+00 | \* |
| 309 | 33 | G | G | T | 0.88 | 7.763559e-01 |  |
| 310 | 34 | G | G | A | 1.89 | 5.800564e-01 |  |
| 310 | 34 | G | G | C | 2.52 | 2.515463e-01 |  |
| 310 | 34 | G | G | G | 95.28 | 0.000000e+00 | \* |
| 310 | 34 | G | G | T | 0.31 | 8.984952e-01 |  |
| 311 | 35 | G | G | A | 4.48 | 2.052278e-01 |  |
| 311 | 35 | G | G | C | 1.49 | 5.754579e-01 |  |
| 311 | 35 | G | G | G | 93.66 | 0.000000e+00 | \* |
| 311 | 35 | G | G | T | 0.37 | 8.908962e-01 |  |
| 312 | 36 | C | C | A | 2.87 | 4.075786e-01 |  |
| 312 | 36 | C | C | C | 94.67 | 0.000000e+00 | \* |
| 312 | 36 | C | C | G | 1.23 | 6.702340e-01 |  |
| 312 | 36 | C | C | T | 1.23 | 6.691671e-01 |  |
| 313 | 37 | A | A | A | 90.68 | 0.000000e+00 | \* |
| 313 | 37 | A | A | C | 2.51 | 2.531222e-01 |  |
| 313 | 37 | A | A | G | 1.79 | 4.424223e-01 |  |
| 313 | 37 | A | A | T | 5.02 | 4.026032e-02 |  |
| 314 | 38 | C | C | A | 3.22 | 3.545998e-01 |  |
| 314 | 38 | C | C | C | 94.64 | 0.000000e+00 | \* |
| 314 | 38 | C | C | G | 0.27 | 9.182985e-01 |  |
| 314 | 38 | C | C | T | 1.88 | 4.682155e-01 |  |
| 315 | 39 | C | C | A | 6.71 | 7.001912e-02 |  |
| 315 | 39 | C | C | C | 90.09 | 0.000000e+00 | \* |
| 315 | 39 | C | C | G | 1.46 | 5.759002e-01 |  |
| 315 | 39 | C | C | T | 1.75 | 5.057282e-01 |  |
| 316 | 40 | C | C | A | 4.55 | 1.989661e-01 |  |
| 316 | 40 | C | C | C | 90.61 | 0.000000e+00 | \* |
| 316 | 40 | C | C | G | 1.21 | 6.773093e-01 |  |
| 316 | 40 | C | C | T | 3.64 | 1.302294e-01 |  |
| 317 | 41 | T | T | A | 3.64 | 2.976330e-01 |  |
| 317 | 41 | T | T | C | 3.64 | 8.115842e-02 |  |
| 317 | 41 | T | T | G | 2.12 | 3.279093e-01 |  |
| 317 | 41 | T | T | T | 90.61 | 0.000000e+00 | \* |
| 318 | 42 | G | G | A | 3.15 | 3.647608e-01 |  |
| 318 | 42 | G | G | C | 2.91 | 1.731881e-01 |  |
| 318 | 42 | G | G | G | 92.98 | 0.000000e+00 | \* |
| 318 | 42 | G | G | T | 0.97 | 7.505963e-01 |  |
| 319 | 43 | A | A | A | 82.30 | 0.000000e+00 | \* |
| 319 | 43 | A | A | C | 1.12 | 7.176486e-01 |  |
| 319 | 43 | A | A | G | 1.69 | 4.836085e-01 |  |
| 319 | 43 | A | A | T | 14.89 | 2.380206e-06 | \* |
| 320 | 44 | C | C | A | 3.50 | 3.149197e-01 |  |
| 320 | 44 | C | C | C | 94.59 | 0.000000e+00 | \* |
| 320 | 44 | C | C | G | 1.59 | 5.207448e-01 |  |
| 320 | 44 | C | C | T | 0.32 | 8.980222e-01 |  |
| 321 | 45 | C | C | A | 9.97 | 1.260860e-02 |  |
| 321 | 45 | C | C | C | 84.57 | 0.000000e+00 | \* |
| 321 | 45 | C | C | G | 2.89 | 1.442266e-01 |  |
| 321 | 45 | C | C | T | 2.57 | 2.941107e-01 |  |
| 322 | 46 | G | G | A | 5.20 | 1.465946e-01 |  |
| 322 | 46 | G | G | C | 2.00 | 3.943805e-01 |  |
| 322 | 46 | G | G | G | 91.20 | 0.000000e+00 | \* |
| 322 | 46 | G | G | T | 1.60 | 5.513818e-01 |  |
| 323 | 47 | A | A | A | 95.06 | 0.000000e+00 | \* |
| 323 | 47 | A | A | C | 0.90 | 7.981513e-01 |  |
| 323 | 47 | A | A | G | 0.90 | 7.954212e-01 |  |
| 323 | 47 | A | A | T | 3.15 | 1.919253e-01 |  |
| 324 | 48 | C | C | A | 4.68 | 1.873566e-01 |  |
| 324 | 48 | C | C | C | 93.53 | 0.000000e+00 | \* |
| 324 | 48 | C | C | G | 0.72 | 8.495159e-01 |  |
| 324 | 48 | C | C | T | 1.08 | 7.167008e-01 |  |
| 325 | 49 | G | G | A | 7.87 | 3.861855e-02 |  |
| 325 | 49 | G | G | C | 2.25 | 3.201642e-01 |  |
| 325 | 49 | G | G | G | 87.64 | 0.000000e+00 | \* |
| 325 | 49 | G | G | T | 2.25 | 3.685883e-01 |  |
| 326 | 50 | G | G | A | 3.70 | 2.891779e-01 |  |
| 326 | 50 | G | G | C | 0.93 | 7.890036e-01 |  |
| 326 | 50 | G | G | G | 95.06 | 0.000000e+00 | \* |
| 326 | 50 | G | G | T | 0.31 | 8.991706e-01 |  |
| 327 | 51 | C | C | A | 2.08 | 5.435336e-01 |  |
| 327 | 51 | C | C | C | 92.71 | 0.000000e+00 | \* |
| 327 | 51 | C | C | G | 2.08 | 3.400145e-01 |  |
| 327 | 51 | C | C | T | 3.12 | 1.950655e-01 |  |
| 328 | 52 | A | A | A | 92.57 | 0.000000e+00 | \* |
| 328 | 52 | A | A | C | 1.35 | 6.300566e-01 |  |
| 328 | 52 | A | A | G | 2.03 | 3.585477e-01 |  |
| 328 | 52 | A | A | T | 4.05 | 9.234387e-02 |  |
| 329 | 53 | A | A | A | 95.60 | 0.000000e+00 | \* |
| 329 | 53 | A | A | C | 0.37 | 9.244190e-01 |  |
| 329 | 53 | A | A | G | 1.10 | 7.224094e-01 |  |
| 329 | 53 | A | A | T | 2.93 | 2.261861e-01 |  |
| 330 | 54 | G | G | A | 1.89 | 5.800564e-01 |  |
| 330 | 54 | G | G | C | 0.47 | 9.093393e-01 |  |
| 330 | 54 | G | G | G | 97.17 | 0.000000e+00 | \* |
| 330 | 54 | G | G | T | 0.47 | 8.750157e-01 |  |
| 331 | 55 | A | A | A | 95.97 | 0.000000e+00 | \* |
| 331 | 55 | A | A | C | 0.00 | 9.404762e-01 |  |
| 331 | 55 | A | A | G | 2.35 | 2.613913e-01 |  |
| 331 | 55 | A | A | T | 1.68 | 5.273660e-01 |  |
| 332 | 56 | A | A | A | 94.30 | 0.000000e+00 | \* |
| 332 | 56 | A | A | C | 0.00 | 9.404762e-01 |  |
| 332 | 56 | A | A | G | 1.14 | 7.059934e-01 |  |
| 332 | 56 | A | A | T | 4.56 | 5.992533e-02 |  |
| 333 | 57 | G | G | A | 2.33 | 4.995663e-01 |  |
| 333 | 57 | G | G | C | 1.16 | 7.028164e-01 |  |
| 333 | 57 | G | G | G | 94.77 | 0.000000e+00 | \* |
| 333 | 57 | G | G | T | 1.74 | 5.072553e-01 |  |
| 334 | 58 | T | T | A | 0.44 | 8.153431e-01 |  |
| 334 | 58 | T | T | C | 2.62 | 2.281963e-01 |  |
| 334 | 58 | T | T | G | 0.87 | 8.038770e-01 |  |
| 334 | 58 | T | T | T | 96.07 | 0.000000e+00 | \* |
| 335 | 59 | T | T | A | 8.33 | 3.022052e-02 |  |
| 335 | 59 | T | T | C | 1.59 | 5.394082e-01 |  |
| 335 | 59 | T | T | G | 1.98 | 3.730929e-01 |  |
| 335 | 59 | T | T | T | 88.10 | 0.000000e+00 | \* |
| 336 | 60 | C | C | A | 3.50 | 3.150841e-01 |  |
| 336 | 60 | C | C | C | 92.22 | 0.000000e+00 | \* |
| 336 | 60 | C | C | G | 3.11 | 1.116169e-01 |  |
| 336 | 60 | C | C | T | 1.17 | 6.889636e-01 |  |
| 337 | 61 | G | G | A | 5.41 | 1.329003e-01 |  |
| 337 | 61 | G | G | C | 2.16 | 3.445075e-01 |  |
| 337 | 61 | G | G | G | 91.89 | 0.000000e+00 | \* |
| 337 | 61 | G | G | T | 0.54 | 8.617878e-01 |  |
| 338 | 62 | A | A | A | 94.23 | 0.000000e+00 | \* |
| 338 | 62 | A | A | C | 1.10 | 7.269051e-01 |  |
| 338 | 62 | A | A | G | 1.92 | 3.944070e-01 |  |
| 338 | 62 | A | A | T | 2.75 | 2.591381e-01 |  |
| 339 | 63 | C | C | A | 2.67 | 4.405752e-01 |  |
| 339 | 63 | C | C | C | 92.00 | 0.000000e+00 | \* |
| 339 | 63 | C | C | G | 2.33 | 2.656186e-01 |  |
| 339 | 63 | C | C | T | 3.00 | 2.146090e-01 |  |
| 340 | 64 | A | A | A | 95.53 | 0.000000e+00 | \* |
| 340 | 64 | A | A | C | 2.23 | 3.236791e-01 |  |
| 340 | 64 | A | A | G | 2.23 | 2.934352e-01 |  |
| 340 | 64 | A | A | T | 0.00 | 9.148936e-01 |  |
| 341 | 65 | G | G | A | 0.69 | 7.864383e-01 |  |
| 341 | 65 | G | G | C | 2.08 | 3.704130e-01 |  |
| 341 | 65 | G | G | G | 95.50 | 0.000000e+00 | \* |
| 341 | 65 | G | G | T | 1.73 | 5.114953e-01 |  |
| 342 | 66 | C | C | A | 2.86 | 4.094461e-01 |  |
| 342 | 66 | C | C | C | 93.06 | 0.000000e+00 | \* |
| 342 | 66 | C | C | G | 2.04 | 3.539500e-01 |  |
| 342 | 66 | C | C | T | 2.04 | 4.222306e-01 |  |
| 343 | 67 | T | T | A | 1.61 | 6.314254e-01 |  |
| 343 | 67 | T | T | C | 0.97 | 7.745213e-01 |  |
| 343 | 67 | T | T | G | 0.32 | 9.151552e-01 |  |
| 343 | 67 | T | T | T | 97.10 | 0.000000e+00 | \* |
| 344 | 68 | C | C | A | 2.93 | 3.978529e-01 |  |
| 344 | 68 | C | C | C | 93.04 | 0.000000e+00 | \* |
| 344 | 68 | C | C | G | 1.10 | 7.224094e-01 |  |
| 344 | 68 | C | C | T | 2.93 | 2.261861e-01 |  |
| 345 | 69 | C | C | A | 2.95 | 3.948116e-01 |  |
| 345 | 69 | C | C | C | 94.99 | 0.000000e+00 | \* |
| 345 | 69 | C | C | G | 0.00 | 9.222222e-01 |  |
| 345 | 69 | C | C | T | 2.06 | 4.157247e-01 |  |
| 346 | 70 | C | C | A | 4.21 | 2.320478e-01 |  |
| 346 | 70 | C | C | C | 94.39 | 0.000000e+00 | \* |
| 346 | 70 | C | C | G | 1.40 | 5.990544e-01 |  |
| 346 | 70 | C | C | T | 0.00 | 9.148936e-01 |  |
| 347 | 71 | G | G | A | 5.42 | 1.320537e-01 |  |
| 347 | 71 | G | G | C | 0.99 | 7.683546e-01 |  |
| 347 | 71 | G | G | G | 92.61 | 0.000000e+00 | \* |
| 347 | 71 | G | G | T | 0.99 | 7.455622e-01 |  |
| 348 | 72 | C | C | A | 3.51 | 3.141793e-01 |  |
| 348 | 72 | C | C | C | 93.86 | 0.000000e+00 | \* |
| 348 | 72 | C | C | G | 0.88 | 8.026222e-01 |  |
| 348 | 72 | C | C | T | 1.75 | 5.041944e-01 |  |
| 349 | 73 | G | G | A | 9.52 | 1.602811e-02 |  |
| 349 | 73 | G | G | C | 2.86 | 1.816486e-01 |  |
| 349 | 73 | G | G | G | 86.67 | 0.000000e+00 | \* |
| 349 | 73 | G | G | T | 0.95 | 7.554300e-01 |  |
| 350 | 74 | A | A | A | 95.71 | 0.000000e+00 | \* |
| 350 | 74 | A | A | C | 1.98 | 4.007724e-01 |  |
| 350 | 74 | A | A | G | 0.66 | 8.643327e-01 |  |
| 350 | 74 | A | A | T | 1.65 | 5.358591e-01 |  |
| 351 | 75 | C | C | A | 2.41 | 4.839463e-01 |  |
| 351 | 75 | C | C | C | 84.14 | 0.000000e+00 | \* |
| 351 | 75 | C | C | G | 9.66 | 7.107034e-06 | \* |
| 351 | 75 | C | C | T | 3.79 | 1.146186e-01 |  |

## For use in R

If you want to work with the results in R, here is output that you can copy and paste in your terminal to get:

The base information:

```
structure(list(focal.base = c("A", "C", "G", "T"), avg.percsignal = c(92.9256807853585, 
92.9113854856468, 93.3936278003092, 94.2550106400649), avg.areasignal = c(309.117647058824, 
329.925373134328, 310.964912280702, 357.046511627907), crit.perc.area = c(10.3939057956162, 
5.44590588124068, 4.94731202255287, 6.54653340107372), mu = c(3.30764464250543, 
1.98537541767752, 1.9172589871455, 2.19996694672371), fillibens = c(0.982103146048334, 
0.995045109043599, 0.990779949503488, 0.995161019409939)), .Names = c("focal.base", 
"avg.percsignal", "avg.areasignal", "crit.perc.area", "mu", "fillibens"
), row.names = c(NA, -4L), class = "data.frame")
```

the data.frame that contains information on the guide region:

```
structure(list(A.area = c(293, 330, 7, 561, 13, 17, 22, 18, 12, 
12, 266, 9, 551, 15, 15, 13, 2, 19, 3, 1, 0, 15, 0, 14, 7, 227, 
9, 6, 316, 6, 225, 7, 7, 6, 12, 7, 253, 12, 23, 15, 12, 13, 293, 
11, 31, 13, 423, 13, 14, 12, 4, 137, 261, 4, 286, 248, 4, 1, 
21, 9, 10, 343, 8, 171, 2, 7, 5, 8, 10, 9, 11, 8, 20, 290, 7), 
    C.area = c(5, 2, 10, 6, 324, 11, 0, 8, 215, 368, 6, 4, 7, 
    339, 287, 5, 4, 4, 5, 0, 6, 0, 12, 12, 293, 10, 333, 13, 
    5, 319, 7, 278, 5, 8, 4, 231, 7, 353, 309, 299, 12, 12, 4, 
    297, 263, 5, 4, 260, 4, 3, 178, 2, 1, 1, 0, 0, 2, 6, 4, 237, 
    4, 4, 276, 4, 6, 228, 3, 254, 322, 202, 2, 214, 6, 6, 244
    ), G.area = c(7, 6, 251, 5, 0, 10, 566, 365, 5, 5, 7, 261, 
    4, 10, 7, 307, 6, 484, 12, 8, 8, 426, 4, 347, 4, 9, 1, 4, 
    4, 5, 9, 7, 213, 303, 251, 3, 5, 1, 5, 4, 7, 384, 6, 5, 9, 
    228, 4, 2, 156, 308, 4, 3, 3, 206, 7, 3, 163, 2, 5, 8, 170, 
    7, 7, 4, 276, 5, 1, 3, 0, 3, 188, 2, 182, 2, 28), T.area = c(15, 
    14, 5, 26, 9, 355, 7, 2, 9, 10, 8, 0, 20, 9, 5, 5, 233, 9, 
    293, 318, 323, 4, 263, 4, 9, 15, 7, 328, 4, 7, 6, 6, 2, 1, 
    1, 3, 14, 7, 6, 12, 299, 4, 53, 1, 8, 4, 14, 3, 4, 1, 6, 
    6, 8, 1, 5, 12, 3, 220, 222, 3, 1, 10, 9, 0, 5, 5, 301, 8, 
    7, 0, 2, 4, 2, 5, 11), Tot.area = c(320, 352, 273, 598, 346, 
    393, 595, 393, 241, 395, 287, 274, 582, 373, 314, 330, 245, 
    516, 313, 327, 337, 445, 279, 377, 313, 261, 350, 351, 329, 
    337, 247, 298, 227, 318, 268, 244, 279, 373, 343, 330, 330, 
    413, 356, 314, 311, 250, 445, 278, 178, 324, 192, 148, 273, 
    212, 298, 263, 172, 229, 252, 257, 185, 364, 300, 179, 289, 
    245, 310, 273, 339, 214, 203, 228, 210, 303, 290), A.perc = c(91.5625, 
    93.75, 2.56410256410256, 93.8127090301003, 3.75722543352601, 
    4.32569974554707, 3.69747899159664, 4.58015267175572, 4.9792531120332, 
    3.0379746835443, 92.6829268292683, 3.28467153284672, 94.6735395189003, 
    4.02144772117962, 4.77707006369427, 3.93939393939394, 0.816326530612245, 
    3.68217054263566, 0.958466453674121, 0.305810397553517, 0, 
    3.37078651685393, 0, 3.71352785145889, 2.23642172523962, 
    86.9731800766284, 2.57142857142857, 1.70940170940171, 96.048632218845, 
    1.78041543026706, 91.0931174089069, 2.3489932885906, 3.08370044052863, 
    1.88679245283019, 4.47761194029851, 2.86885245901639, 90.6810035842294, 
    3.2171581769437, 6.70553935860058, 4.54545454545455, 3.63636363636364, 
    3.14769975786925, 82.3033707865169, 3.5031847133758, 9.96784565916399, 
    5.2, 95.0561797752809, 4.67625899280576, 7.86516853932584, 
    3.7037037037037, 2.08333333333333, 92.5675675675676, 95.6043956043956, 
    1.88679245283019, 95.9731543624161, 94.2965779467681, 2.32558139534884, 
    0.436681222707424, 8.33333333333333, 3.50194552529183, 5.40540540540541, 
    94.2307692307692, 2.66666666666667, 95.5307262569832, 0.692041522491349, 
    2.85714285714286, 1.61290322580645, 2.93040293040293, 2.94985250737463, 
    4.20560747663551, 5.41871921182266, 3.50877192982456, 9.52380952380952, 
    95.7095709570957, 2.41379310344828), C.perc = c(1.5625, 0.568181818181818, 
    3.66300366300366, 1.00334448160535, 93.6416184971098, 2.79898218829517, 
    0, 2.03562340966921, 89.2116182572614, 93.1645569620253, 
    2.09059233449477, 1.45985401459854, 1.20274914089347, 90.8847184986595, 
    91.4012738853503, 1.51515151515152, 1.63265306122449, 0.775193798449612, 
    1.59744408945687, 0, 1.78041543026706, 0, 4.3010752688172, 
    3.18302387267904, 93.6102236421725, 3.83141762452107, 95.1428571428571, 
    3.7037037037037, 1.51975683890578, 94.6587537091988, 2.83400809716599, 
    93.2885906040269, 2.20264317180617, 2.51572327044025, 1.49253731343284, 
    94.672131147541, 2.5089605734767, 94.6380697050938, 90.0874635568513, 
    90.6060606060606, 3.63636363636364, 2.90556900726392, 1.12359550561798, 
    94.5859872611465, 84.5659163987138, 2, 0.898876404494382, 
    93.5251798561151, 2.24719101123596, 0.925925925925926, 92.7083333333333, 
    1.35135135135135, 0.366300366300366, 0.471698113207547, 0, 
    0, 1.16279069767442, 2.62008733624454, 1.58730158730159, 
    92.2178988326848, 2.16216216216216, 1.0989010989011, 92, 
    2.23463687150838, 2.07612456747405, 93.0612244897959, 0.967741935483871, 
    93.040293040293, 94.9852507374631, 94.392523364486, 0.985221674876847, 
    93.859649122807, 2.85714285714286, 1.98019801980198, 84.1379310344828
    ), G.perc = c(2.1875, 1.70454545454545, 91.9413919413919, 
    0.836120401337793, 0, 2.54452926208651, 95.1260504201681, 
    92.8753180661578, 2.0746887966805, 1.26582278481013, 2.4390243902439, 
    95.2554744525548, 0.687285223367698, 2.68096514745308, 2.22929936305732, 
    93.030303030303, 2.44897959183673, 93.7984496124031, 3.83386581469649, 
    2.44648318042813, 2.37388724035608, 95.7303370786517, 1.4336917562724, 
    92.0424403183024, 1.2779552715655, 3.44827586206897, 0.285714285714286, 
    1.13960113960114, 1.21580547112462, 1.48367952522255, 3.64372469635628, 
    2.3489932885906, 93.8325991189427, 95.2830188679245, 93.6567164179104, 
    1.22950819672131, 1.7921146953405, 0.268096514745308, 1.45772594752187, 
    1.21212121212121, 2.12121212121212, 92.9782082324455, 1.68539325842697, 
    1.59235668789809, 2.89389067524116, 91.2, 0.898876404494382, 
    0.719424460431655, 87.6404494382023, 95.0617283950617, 2.08333333333333, 
    2.02702702702703, 1.0989010989011, 97.1698113207547, 2.3489932885906, 
    1.14068441064639, 94.7674418604651, 0.873362445414847, 1.98412698412698, 
    3.11284046692607, 91.8918918918919, 1.92307692307692, 2.33333333333333, 
    2.23463687150838, 95.5017301038062, 2.04081632653061, 0.32258064516129, 
    1.0989010989011, 0, 1.4018691588785, 92.6108374384236, 0.87719298245614, 
    86.6666666666667, 0.66006600660066, 9.6551724137931), T.perc = c(4.6875, 
    3.97727272727273, 1.83150183150183, 4.34782608695652, 2.60115606936416, 
    90.3307888040712, 1.17647058823529, 0.508905852417303, 3.7344398340249, 
    2.53164556962025, 2.78745644599303, 0, 3.43642611683849, 
    2.41286863270777, 1.59235668789809, 1.51515151515152, 95.1020408163265, 
    1.74418604651163, 93.6102236421725, 97.2477064220184, 95.8456973293769, 
    0.898876404494382, 94.2652329749104, 1.06100795755968, 2.87539936102236, 
    5.74712643678161, 2, 93.4472934472934, 1.21580547112462, 
    2.07715133531157, 2.42914979757085, 2.01342281879195, 0.881057268722467, 
    0.314465408805031, 0.373134328358209, 1.22950819672131, 5.01792114695341, 
    1.87667560321716, 1.74927113702624, 3.63636363636364, 90.6060606060606, 
    0.968523002421308, 14.8876404494382, 0.318471337579618, 2.57234726688103, 
    1.6, 3.14606741573034, 1.07913669064748, 2.24719101123596, 
    0.308641975308642, 3.125, 4.05405405405405, 2.93040293040293, 
    0.471698113207547, 1.67785234899329, 4.56273764258555, 1.74418604651163, 
    96.0698689956332, 88.0952380952381, 1.16731517509728, 0.540540540540541, 
    2.74725274725275, 3, 0, 1.73010380622837, 2.04081632653061, 
    97.0967741935484, 2.93040293040293, 2.06489675516224, 0, 
    0.985221674876847, 1.75438596491228, 0.952380952380952, 1.65016501650165, 
    3.79310344827586), base.call = c("A", "A", "G", "A", "C", 
    "T", "G", "G", "C", "C", "A", "G", "A", "C", "C", "G", "T", 
    "G", "T", "T", "T", "G", "T", "G", "C", "A", "C", "T", "A", 
    "C", "A", "C", "G", "G", "G", "C", "A", "C", "C", "C", "T", 
    "G", "A", "C", "C", "G", "A", "C", "G", "G", "C", "A", "A", 
    "G", "A", "A", "G", "T", "T", "C", "G", "A", "C", "A", "G", 
    "C", "T", "C", "C", "C", "G", "C", "G", "A", "C"), index = 277:351, 
    guide.seq = c("A", "A", "G", "A", "C", "T", "G", "G", "C", 
    "C", "A", "G", "A", "C", "C", "G", "T", "G", "T", "T", "T", 
    "G", "T", "G", "C", "A", "C", "T", "A", "C", "A", "C", "G", 
    "G", "G", "C", "A", "C", "C", "C", "T", "G", "A", "C", "C", 
    "G", "A", "C", "G", "G", "C", "A", "A", "G", "A", "A", "G", 
    "T", "T", "C", "G", "A", "C", "A", "G", "C", "T", "C", "C", 
    "C", "G", "C", "G", "A", "C"), T.pval = c(0.0537865203386781, 
    0.0984487453702355, 0.481346104032927, 0.0720579182126793, 
    0.288108787106797, 0, 0.686058790203372, 0.868072234038207, 
    0.120252491081936, 0.302754658857797, 0.251589178008852, 
    0.91489361696974, 0.152883474459544, 0.329088075708301, 0.553761100111065, 
    0.577979939490846, 0, 0.507255280935267, 0, 0, 0, 0.771198831682095, 
    0, 0.722336016020206, 0.23569855511156, 0.0209186011735774, 
    0.433401914838842, 0, 0.673540227300021, 0.412438416279178, 
    0.325380462259757, 0.429708317241075, 0.776355897503608, 
    0.898495188575933, 0.890896151001024, 0.669167101413079, 
    0.0402603175543866, 0.468215451287598, 0.505728229931949, 
    0.13022939297922, 0, 0.750596348976922, 2.3802064128553e-06, 
    0.898022228304154, 0.29411068068337, 0.551381785867534, 0.191925260042055, 
    0.716700825535683, 0.368588321072026, 0.899170569661006, 
    0.195065454377383, 0.092343868630425, 0.226186063707124, 
    0.875015720178368, 0.527366018397957, 0.0599253252808659, 
    0.507255280935267, 0, 0, 0.688963622848991, 0.86178784862691, 
    0.259138134958535, 0.21460897040917, 0.91489361696974, 0.511495347342426, 
    0.422230584579814, 0, 0.226186063707124, 0.415724749984659, 
    0.91489361696974, 0.745562179473062, 0.504194434947348, 0.755430044855882, 
    0.535859090862847, 0.114618626707667), C.pval = c(0.54877551246571, 
    0.890817875828886, 0.0788524478881963, 0.76189632371745, 
    0, 0.19226654477848, 0.94047619047619, 0.383044085832972, 
    0, 0, 0.365968188578992, 0.588029774315449, 0.687549872834117, 
    0, 0, 0.566795728215217, 0.522425798404791, 0.837373065947618, 
    0.535593191320097, 0.94047619047619, 0.468676431834767, 0.94047619047619, 
    0.0387333205791758, 0.130922595942508, 0, 0.0656057595810087, 
    0, 0.0754444810805317, 0.565035800719446, 0, 0.185811699613336, 
    0, 0.332760961180335, 0.251546313762593, 0.575457896251503, 
    0, 0.253122183414249, 0, 0, 0, 0.0811584244412022, 0.173188073572383, 
    0.717648607190075, 0, 0, 0.394380466283461, 0.798151292668513, 
    0, 0.320164238895719, 0.789003584674685, 0, 0.630056643398533, 
    0.924419038046079, 0.90933932964728, 0.94047619047619, 0.94047619047619, 
    0.702816435644212, 0.22819627885115, 0.539408200841539, 0, 
    0.344507474747602, 0.726905089602335, 0, 0.323679099044357, 
    0.370413019647777, 0, 0.774521257225423, 0, 0, 0, 0.768354610090192, 
    0, 0.181648588776015, 0.400772377162099, 0), G.pval = c(0.307437024664335, 
    0.476096393949101, 0, 0.815795610422081, 0.922222222222223, 
    0.21282366107275, 0, 0, 0.342818214756618, 0.655365731218349, 
    0.238067653252559, 0, 0.857745479965168, 0.183396579897632, 
    0.29499738160672, 0, 0.235590294417058, 0, 0.0454128844972119, 
    0.236209641975406, 0.254775665612843, 0, 0.585853478860677, 
    0, 0.650375349053791, 0.0741697317480526, 0.917410545723198, 
    0.706422784667321, 0.675812719525966, 0.565177210417226, 
    0.057989094987799, 0.261391297912377, 0, 0, 0, 0.670233974358762, 
    0.442422340235429, 0.918298484124711, 0.57590015814105, 0.677309342138475, 
    0.327909253175449, 0, 0.483608514002962, 0.520744756900975, 
    0.144226560696, 0, 0.795421210257029, 0.849515895593358, 
    0, 0, 0.340014516747385, 0.358547695638971, 0.722409429891196, 
    0, 0.261391297912377, 0.705993431035964, 0, 0.803877015347967, 
    0.373092869156925, 0.111616855524956, 0, 0.394407049844301, 
    0.265618645099629, 0.293435159407384, 0, 0.35394995970039, 
    0.915155156496858, 0.722409429891196, 0.922222222222223, 
    0.599054350101987, 0, 0.80262216801523, 0, 0.864332711670402, 
    7.1070338033552e-06), A.pval = c(0, 0, 0.457899780339862, 
    0, 0.28259361505457, 0.219862309556866, 0.289951415073008, 
    0.19582747854765, 0.162705198767038, 0.381225939959884, 0, 
    0.344919337117256, 0, 0.251826133996516, 0.178814707076685, 
    0.261074930544494, 0.769167747600683, 0.291860761348224, 
    0.747431053844786, 0.825891301711952, 0.836363636363636, 
    0.332853407122238, 0.836363636363636, 0.287960294185129, 
    0.515580064825383, 0, 0.456649841891379, 0.613330896478261, 
    0, 0.599997727321623, 0, 0.495398116143425, 0.374303188019269, 
    0.580056380512596, 0.205227799402492, 0.407578636344795, 
    0, 0.354599755217236, 0.070019116226581, 0.198966053164334, 
    0.297632985207129, 0.364760778695124, 0, 0.314919689057874, 
    0.0126086003265116, 0.146594582282526, 0, 0.1873565607739, 
    0.0386185463798197, 0.289177854861372, 0.543533574728295, 
    0, 0, 0.580056380512596, 0, 0, 0.499566334379937, 0.815343106807073, 
    0.0302205221236402, 0.315084075547234, 0.132900281648033, 
    0, 0.440575165494006, 0, 0.786438313971437, 0.409446107486363, 
    0.631425388274593, 0.397852940385462, 0.394811554926745, 
    0.232047750360314, 0.132053732140658, 0.314179319972751, 
    0.0160281102967372, 0, 0.483946296330566), guide.position = 1:75), .Names = c("A.area", 
"C.area", "G.area", "T.area", "Tot.area", "A.perc", "C.perc", 
"G.perc", "T.perc", "base.call", "index", "guide.seq", "T.pval", 
"C.pval", "G.pval", "A.pval", "guide.position"), row.names = 277:351, class = "data.frame")
```

*Report generated using EditR v1.0.8*
