# Supplementary material for: Mechanistic and genetic basis of single-strand templated repair at Cas12a-induced DNA breaks in Chlamydomonas reinhardtii
Source: Nat Commun. 2021 Nov 19;12:6751. doi: 10.1038/s41467-021-27004-1 (PMC8604939; doi:10.1038/s41467-021-27004-1)
Supplement: Supplementary file 22 — Source Data [file 41467_2021_27004_MOESM22_ESM.zip › Source Data/EditR analysis/EditR outputs/Antisense/rep3_ssODN_antisense_16.html]

EditR v1.0.8 report


# EditR v1.0.8 report

- Data QA
  - Filtering data
  - Percent noise peak area
  - Base information
- Predicted editing
  - Editing bar plot
  - Editing table plot
  - Table of editing results
- For use in R

## Data QA

### Filtering data

What the data looked like prefiltering:

and the post filtering signal / noise plot:

### Percent noise peak area

### Base information

Here’s information about the signal of each base, the critical percent value where any higher value would be called as significant, and Filliben’s correlation for how well the noise was modelled by the zero adjusted gamma distribution.

| Base | Average percent signal | Average peak area | Critical percent value | model mu | Fillibens correlation |
| --- | --- | --- | --- | --- | --- |
| A | 93.71075 | 372.7059 | 11.378711 | 3.378301 | 0.9869434 |
| C | 94.21017 | 391.3636 | 5.000097 | 1.828885 | 0.9969638 |
| G | 93.39378 | 367.5789 | 5.004424 | 1.693554 | 0.9958461 |
| T | 94.66050 | 430.7273 | 6.129975 | 2.149411 | 0.9943451 |

## Predicted editing

### Editing bar plot

### Editing table plot

### Table of editing results


Here’s the entire guide region

| Sanger position | Guide position | Guide sequence | Sanger base call | Focal base | Focal base peak area | p value |  |
| --- | --- | --- | --- | --- | --- | --- | --- |
| 275 | 1 | A | A | A | 94.72 | 0.00000000 | \* |
| 275 | 1 | A | A | C | 1.67 | 0.45336581 |  |
| 275 | 1 | A | A | G | 0.56 | 0.80798429 |  |
| 275 | 1 | A | A | T | 3.06 | 0.17910555 |  |
| 276 | 2 | A | A | A | 93.21 | 0.00000000 | \* |
| 276 | 2 | A | A | C | 1.17 | 0.65397896 |  |
| 276 | 2 | A | A | G | 1.17 | 0.56810140 |  |
| 276 | 2 | A | A | T | 4.45 | 0.05189242 |  |
| 277 | 3 | G | G | A | 3.04 | 0.35373131 |  |
| 277 | 3 | G | G | C | 1.22 | 0.63530169 |  |
| 277 | 3 | G | G | G | 95.74 | 0.00000000 | \* |
| 277 | 3 | G | G | T | 0.00 | 0.86021505 |  |
| 278 | 4 | A | A | A | 94.00 | 0.00000000 | \* |
| 278 | 4 | A | A | C | 1.14 | 0.66564427 |  |
| 278 | 4 | A | A | G | 1.43 | 0.46745874 |  |
| 278 | 4 | A | A | T | 3.43 | 0.13059935 |  |
| 279 | 5 | C | C | A | 3.84 | 0.26250612 |  |
| 279 | 5 | C | C | C | 93.76 | 0.00000000 | \* |
| 279 | 5 | C | C | G | 0.00 | 0.90000000 |  |
| 279 | 5 | C | C | T | 2.40 | 0.30094179 |  |
| 280 | 6 | T | T | A | 4.84 | 0.17666385 |  |
| 280 | 6 | T | T | C | 3.23 | 0.09237104 |  |
| 280 | 6 | T | T | G | 2.02 | 0.27968738 |  |
| 280 | 6 | T | T | T | 89.92 | 0.00000000 | \* |
| 281 | 7 | G | G | A | 4.52 | 0.20083434 |  |
| 281 | 7 | G | G | C | 0.00 | 0.92941176 |  |
| 281 | 7 | G | G | G | 94.35 | 0.00000000 | \* |
| 281 | 7 | G | G | T | 1.13 | 0.66328913 |  |
| 282 | 8 | G | G | A | 5.45 | 0.13747520 |  |
| 282 | 8 | G | G | C | 1.68 | 0.44942557 |  |
| 282 | 8 | G | G | G | 92.45 | 0.00000000 | \* |
| 282 | 8 | G | G | T | 0.42 | 0.83532745 |  |
| 283 | 9 | C | C | A | 5.43 | 0.13838675 |  |
| 283 | 9 | C | C | C | 90.58 | 0.00000000 | \* |
| 283 | 9 | C | C | G | 3.26 | 0.07723427 |  |
| 283 | 9 | C | C | T | 0.72 | 0.77660378 |  |
| 284 | 10 | C | C | A | 1.52 | 0.58409236 |  |
| 284 | 10 | C | C | C | 96.53 | 0.00000000 | \* |
| 284 | 10 | C | C | G | 1.95 | 0.29686416 |  |
| 284 | 10 | C | C | T | 0.00 | 0.86021505 |  |
| 285 | 11 | A | A | A | 95.83 | 0.00000000 | \* |
| 285 | 11 | A | A | C | 1.92 | 0.36263177 |  |
| 285 | 11 | A | A | G | 1.92 | 0.30498432 |  |
| 285 | 11 | A | A | T | 0.32 | 0.84707229 |  |
| 286 | 12 | G | G | A | 3.92 | 0.25465716 |  |
| 286 | 12 | G | G | C | 1.20 | 0.63988195 |  |
| 286 | 12 | G | G | G | 94.58 | 0.00000000 | \* |
| 286 | 12 | G | G | T | 0.30 | 0.84891153 |  |
| 287 | 13 | A | A | A | 93.57 | 0.00000000 | \* |
| 287 | 13 | A | A | C | 1.00 | 0.72389030 |  |
| 287 | 13 | A | A | G | 0.86 | 0.69617923 |  |
| 287 | 13 | A | A | T | 4.57 | 0.04626810 |  |
| 288 | 14 | C | C | A | 3.70 | 0.27624869 |  |
| 288 | 14 | C | C | C | 93.52 | 0.00000000 | \* |
| 288 | 14 | C | C | G | 1.16 | 0.57359253 |  |
| 288 | 14 | C | C | T | 1.62 | 0.51015875 |  |
| 289 | 15 | C | C | A | 6.35 | 0.09439547 |  |
| 289 | 15 | C | C | C | 89.85 | 0.00000000 | \* |
| 289 | 15 | C | C | G | 1.78 | 0.34822878 |  |
| 289 | 15 | C | C | T | 2.03 | 0.39165904 |  |
| 290 | 16 | G | G | A | 5.30 | 0.14613024 |  |
| 290 | 16 | G | G | C | 1.01 | 0.71985526 |  |
| 290 | 16 | G | G | G | 91.92 | 0.00000000 | \* |
| 290 | 16 | G | G | T | 1.77 | 0.46583899 |  |
| 291 | 17 | T | T | A | 0.00 | 0.78181818 |  |
| 291 | 17 | T | T | C | 1.38 | 0.56537158 |  |
| 291 | 17 | T | T | G | 2.42 | 0.18821119 |  |
| 291 | 17 | T | T | T | 96.19 | 0.00000000 | \* |
| 292 | 18 | G | G | A | 4.35 | 0.21504094 |  |
| 292 | 18 | G | G | C | 0.50 | 0.88558485 |  |
| 292 | 18 | G | G | G | 94.65 | 0.00000000 | \* |
| 292 | 18 | G | G | T | 0.50 | 0.82262976 |  |
| 293 | 19 | T | T | A | 1.32 | 0.61747676 |  |
| 293 | 19 | T | T | C | 0.26 | 0.92140466 |  |
| 293 | 19 | T | T | G | 4.75 | 0.01364105 |  |
| 293 | 19 | T | T | T | 93.67 | 0.00000000 | \* |
| 294 | 20 | T | T | A | 1.49 | 0.58967281 |  |
| 294 | 20 | T | T | C | 2.23 | 0.27164912 |  |
| 294 | 20 | T | T | G | 1.98 | 0.28925578 |  |
| 294 | 20 | T | T | T | 94.31 | 0.00000000 | \* |
| 295 | 21 | T | T | A | 0.00 | 0.78181818 |  |
| 295 | 21 | T | T | C | 2.23 | 0.27164912 |  |
| 295 | 21 | T | T | G | 1.73 | 0.36203816 |  |
| 295 | 21 | T | T | T | 96.04 | 0.00000000 | \* |
| 296 | 22 | G | G | A | 3.54 | 0.29341452 |  |
| 296 | 22 | G | G | C | 0.75 | 0.81761723 |  |
| 296 | 22 | G | G | G | 94.78 | 0.00000000 | \* |
| 296 | 22 | G | G | T | 0.93 | 0.72176573 |  |
| 297 | 23 | T | T | A | 0.00 | 0.78181818 |  |
| 297 | 23 | T | T | C | 0.00 | 0.92941176 |  |
| 297 | 23 | T | T | G | 1.79 | 0.34379067 |  |
| 297 | 23 | T | T | T | 98.21 | 0.00000000 | \* |
| 298 | 24 | G | G | A | 4.48 | 0.20369543 |  |
| 298 | 24 | G | G | C | 1.35 | 0.58139220 |  |
| 298 | 24 | G | G | G | 93.05 | 0.00000000 | \* |
| 298 | 24 | G | G | T | 1.12 | 0.66600754 |  |
| 299 | 25 | C | C | A | 2.20 | 0.47348763 |  |
| 299 | 25 | C | C | C | 95.05 | 0.00000000 | \* |
| 299 | 25 | C | C | G | 1.92 | 0.30498432 |  |
| 299 | 25 | C | C | T | 0.82 | 0.75153287 |  |
| 300 | 26 | A | A | A | 90.13 | 0.00000000 | \* |
| 300 | 26 | A | A | C | 3.62 | 0.05800589 |  |
| 300 | 26 | A | A | G | 3.95 | 0.03538158 |  |
| 300 | 26 | A | A | T | 2.30 | 0.32292617 |  |
| 301 | 27 | C | C | A | 2.78 | 0.38845614 |  |
| 301 | 27 | C | C | C | 95.14 | 0.00000000 | \* |
| 301 | 27 | C | C | G | 0.46 | 0.83619961 |  |
| 301 | 27 | C | C | T | 1.62 | 0.51015875 |  |
| 302 | 28 | T | T | A | 1.83 | 0.53291146 |  |
| 302 | 28 | T | T | C | 4.11 | 0.03164407 |  |
| 302 | 28 | T | T | G | 0.68 | 0.76290157 |  |
| 302 | 28 | T | T | T | 93.38 | 0.00000000 | \* |
| 303 | 29 | A | A | A | 96.60 | 0.00000000 | \* |
| 303 | 29 | A | A | C | 1.31 | 0.59649711 |  |
| 303 | 29 | A | A | G | 0.79 | 0.72466601 |  |
| 303 | 29 | A | A | T | 1.31 | 0.60748817 |  |
| 304 | 30 | C | C | A | 1.80 | 0.53658359 |  |
| 304 | 30 | C | C | C | 95.36 | 0.00000000 | \* |
| 304 | 30 | C | C | G | 1.55 | 0.42463733 |  |
| 304 | 30 | C | C | T | 1.29 | 0.61385732 |  |
| 305 | 31 | A | A | A | 93.45 | 0.00000000 | \* |
| 305 | 31 | A | A | C | 2.76 | 0.15635985 |  |
| 305 | 31 | A | A | G | 3.79 | 0.04230190 |  |
| 305 | 31 | A | A | T | 0.00 | 0.86021505 |  |
| 306 | 32 | C | C | A | 1.15 | 0.64570031 |  |
| 306 | 32 | C | C | C | 95.98 | 0.00000000 | \* |
| 306 | 32 | C | C | G | 2.01 | 0.28090758 |  |
| 306 | 32 | C | C | T | 0.86 | 0.74140665 |  |
| 307 | 33 | G | G | A | 3.69 | 0.27769161 |  |
| 307 | 33 | G | G | C | 2.21 | 0.27533564 |  |
| 307 | 33 | G | G | G | 94.10 | 0.00000000 | \* |
| 307 | 33 | G | G | T | 0.00 | 0.86021505 |  |
| 308 | 34 | G | G | A | 3.03 | 0.35491305 |  |
| 308 | 34 | G | G | C | 0.00 | 0.92941176 |  |
| 308 | 34 | G | G | G | 95.96 | 0.00000000 | \* |
| 308 | 34 | G | G | T | 1.01 | 0.69940238 |  |
| 309 | 35 | G | G | A | 2.15 | 0.48138174 |  |
| 309 | 35 | G | G | C | 1.53 | 0.50480053 |  |
| 309 | 35 | G | G | G | 94.17 | 0.00000000 | \* |
| 309 | 35 | G | G | T | 2.15 | 0.36109162 |  |
| 310 | 36 | C | C | A | 2.49 | 0.42915440 |  |
| 310 | 36 | C | C | C | 96.80 | 0.00000000 | \* |
| 310 | 36 | C | C | G | 0.71 | 0.75291795 |  |
| 310 | 36 | C | C | T | 0.00 | 0.86021505 |  |
| 311 | 37 | A | A | A | 91.10 | 0.00000000 | \* |
| 311 | 37 | A | A | C | 2.76 | 0.15599962 |  |
| 311 | 37 | A | A | G | 1.84 | 0.32885605 |  |
| 311 | 37 | A | A | T | 4.29 | 0.05998350 |  |
| 312 | 38 | C | C | A | 2.92 | 0.36911555 |  |
| 312 | 38 | C | C | C | 94.16 | 0.00000000 | \* |
| 312 | 38 | C | C | G | 0.90 | 0.67932885 |  |
| 312 | 38 | C | C | T | 2.02 | 0.39380611 |  |
| 313 | 39 | C | C | A | 5.42 | 0.13931032 |  |
| 313 | 39 | C | C | C | 93.10 | 0.00000000 | \* |
| 313 | 39 | C | C | G | 1.48 | 0.44927021 |  |
| 313 | 39 | C | C | T | 0.00 | 0.86021505 |  |
| 314 | 40 | C | C | A | 3.83 | 0.26355793 |  |
| 314 | 40 | C | C | C | 93.37 | 0.00000000 | \* |
| 314 | 40 | C | C | G | 1.28 | 0.52629019 |  |
| 314 | 40 | C | C | T | 1.53 | 0.53786836 |  |
| 315 | 41 | T | T | A | 1.54 | 0.58073791 |  |
| 315 | 41 | T | T | C | 1.03 | 0.71361697 |  |
| 315 | 41 | T | T | G | 2.31 | 0.21102869 |  |
| 315 | 41 | T | T | T | 95.13 | 0.00000000 | \* |
| 316 | 42 | G | G | A | 4.00 | 0.24647018 |  |
| 316 | 42 | G | G | C | 2.32 | 0.24885078 |  |
| 316 | 42 | G | G | G | 92.84 | 0.00000000 | \* |
| 316 | 42 | G | G | T | 0.84 | 0.74677815 |  |
| 317 | 43 | A | A | A | 93.70 | 0.00000000 | \* |
| 317 | 43 | A | A | C | 1.26 | 0.61665697 |  |
| 317 | 43 | A | A | G | 1.26 | 0.53222258 |  |
| 317 | 43 | A | A | T | 3.78 | 0.09575680 |  |
| 318 | 44 | C | C | A | 2.58 | 0.41662948 |  |
| 318 | 44 | C | C | C | 95.88 | 0.00000000 | \* |
| 318 | 44 | C | C | G | 0.00 | 0.90000000 |  |
| 318 | 44 | C | C | T | 1.55 | 0.53296644 |  |
| 319 | 45 | C | C | A | 7.65 | 0.05368723 |  |
| 319 | 45 | C | C | C | 86.61 | 0.00000000 | \* |
| 319 | 45 | C | C | G | 4.92 | 0.01111277 |  |
| 319 | 45 | C | C | T | 0.82 | 0.75271654 |  |
| 320 | 46 | G | G | A | 6.51 | 0.08781003 |  |
| 320 | 46 | G | G | C | 0.98 | 0.73293656 |  |
| 320 | 46 | G | G | G | 91.86 | 0.00000000 | \* |
| 320 | 46 | G | G | T | 0.65 | 0.79340369 |  |
| 321 | 47 | A | A | A | 94.36 | 0.00000000 | \* |
| 321 | 47 | A | A | C | 0.94 | 0.74754776 |  |
| 321 | 47 | A | A | G | 0.75 | 0.73765066 |  |
| 321 | 47 | A | A | T | 3.95 | 0.08248321 |  |
| 322 | 48 | C | C | A | 4.19 | 0.22869943 |  |
| 322 | 48 | C | C | C | 94.91 | 0.00000000 | \* |
| 322 | 48 | C | C | G | 0.90 | 0.67960180 |  |
| 322 | 48 | C | C | T | 0.00 | 0.86021505 |  |
| 323 | 49 | G | G | A | 9.59 | 0.02263355 |  |
| 323 | 49 | G | G | C | 1.83 | 0.39537251 |  |
| 323 | 49 | G | G | G | 88.58 | 0.00000000 | \* |
| 323 | 49 | G | G | T | 0.00 | 0.86021505 |  |
| 324 | 50 | G | G | A | 3.50 | 0.29840476 |  |
| 324 | 50 | G | G | C | 0.75 | 0.81637877 |  |
| 324 | 50 | G | G | G | 94.75 | 0.00000000 | \* |
| 324 | 50 | G | G | T | 1.00 | 0.70237334 |  |
| 325 | 51 | C | C | A | 4.72 | 0.18526271 |  |
| 325 | 51 | C | C | C | 90.56 | 0.00000000 | \* |
| 325 | 51 | C | C | G | 1.72 | 0.36713502 |  |
| 325 | 51 | C | C | T | 3.00 | 0.18684653 |  |
| 326 | 52 | A | A | A | 96.72 | 0.00000000 | \* |
| 326 | 52 | A | A | C | 1.09 | 0.68625642 |  |
| 326 | 52 | A | A | G | 2.19 | 0.23783207 |  |
| 326 | 52 | A | A | T | 0.00 | 0.86021505 |  |
| 327 | 53 | A | A | A | 96.41 | 0.00000000 | \* |
| 327 | 53 | A | A | C | 0.00 | 0.92941176 |  |
| 327 | 53 | A | A | G | 1.20 | 0.55734788 |  |
| 327 | 53 | A | A | T | 2.40 | 0.30158694 |  |
| 328 | 54 | G | G | A | 2.33 | 0.45248534 |  |
| 328 | 54 | G | G | C | 0.00 | 0.92941176 |  |
| 328 | 54 | G | G | G | 97.28 | 0.00000000 | \* |
| 328 | 54 | G | G | T | 0.39 | 0.83932748 |  |
| 329 | 55 | A | A | A | 92.95 | 0.00000000 | \* |
| 329 | 55 | A | A | C | 0.00 | 0.92941176 |  |
| 329 | 55 | A | A | G | 3.79 | 0.04225637 |  |
| 329 | 55 | A | A | T | 3.25 | 0.15191060 |  |
| 330 | 56 | A | A | A | 95.00 | 0.00000000 | \* |
| 330 | 56 | A | A | C | 0.00 | 0.92941176 |  |
| 330 | 56 | A | A | G | 0.59 | 0.79714781 |  |
| 330 | 56 | A | A | T | 4.41 | 0.05376825 |  |
| 331 | 57 | G | G | A | 1.80 | 0.53696540 |  |
| 331 | 57 | G | G | C | 1.35 | 0.57888337 |  |
| 331 | 57 | G | G | G | 94.59 | 0.00000000 | \* |
| 331 | 57 | G | G | T | 2.25 | 0.33497955 |  |
| 332 | 58 | T | T | A | 0.34 | 0.76085500 |  |
| 332 | 58 | T | T | C | 3.06 | 0.11160138 |  |
| 332 | 58 | T | T | G | 0.68 | 0.76461720 |  |
| 332 | 58 | T | T | T | 95.92 | 0.00000000 | \* |
| 333 | 59 | T | T | A | 10.16 | 0.01748175 |  |
| 333 | 59 | T | T | C | 0.95 | 0.74267600 |  |
| 333 | 59 | T | T | G | 3.49 | 0.05963587 |  |
| 333 | 59 | T | T | T | 85.40 | 0.00000000 | \* |
| 334 | 60 | C | C | A | 1.57 | 0.57589414 |  |
| 334 | 60 | C | C | C | 94.67 | 0.00000000 | \* |
| 334 | 60 | C | C | G | 2.82 | 0.12446457 |  |
| 334 | 60 | C | C | T | 0.94 | 0.71960448 |  |
| 335 | 61 | G | G | A | 5.69 | 0.12439277 |  |
| 335 | 61 | G | G | C | 2.44 | 0.21950580 |  |
| 335 | 61 | G | G | G | 91.06 | 0.00000000 | \* |
| 335 | 61 | G | G | T | 0.81 | 0.75445995 |  |
| 336 | 62 | A | A | A | 93.44 | 0.00000000 | \* |
| 336 | 62 | A | A | C | 1.09 | 0.68576550 |  |
| 336 | 62 | A | A | G | 1.53 | 0.42983679 |  |
| 336 | 62 | A | A | T | 3.94 | 0.08313083 |  |
| 337 | 63 | C | C | A | 2.16 | 0.47904473 |  |
| 337 | 63 | C | C | C | 93.78 | 0.00000000 | \* |
| 337 | 63 | C | C | G | 2.70 | 0.14104473 |  |
| 337 | 63 | C | C | T | 1.35 | 0.59411464 |  |
| 338 | 64 | A | A | A | 91.67 | 0.00000000 | \* |
| 338 | 64 | A | A | C | 2.92 | 0.13134359 |  |
| 338 | 64 | A | A | G | 2.08 | 0.26245805 |  |
| 338 | 64 | A | A | T | 3.33 | 0.14174633 |  |
| 339 | 65 | G | G | A | 2.18 | 0.47628457 |  |
| 339 | 65 | G | G | C | 1.91 | 0.36783572 |  |
| 339 | 65 | G | G | G | 94.82 | 0.00000000 | \* |
| 339 | 65 | G | G | T | 1.09 | 0.67550756 |  |
| 340 | 66 | C | C | A | 0.68 | 0.71906445 |  |
| 340 | 66 | C | C | C | 93.56 | 0.00000000 | \* |
| 340 | 66 | C | C | G | 2.71 | 0.13969754 |  |
| 340 | 66 | C | C | T | 3.05 | 0.17980501 |  |
| 341 | 67 | T | T | A | 0.00 | 0.78181818 |  |
| 341 | 67 | T | T | C | 1.50 | 0.51974508 |  |
| 341 | 67 | T | T | G | 1.25 | 0.53762936 |  |
| 341 | 67 | T | T | T | 97.26 | 0.00000000 | \* |
| 342 | 68 | C | C | A | 4.12 | 0.23542462 |  |
| 342 | 68 | C | C | C | 92.35 | 0.00000000 | \* |
| 342 | 68 | C | C | G | 1.18 | 0.56587273 |  |
| 342 | 68 | C | C | T | 2.35 | 0.31119929 |  |
| 343 | 69 | C | C | A | 2.13 | 0.48445698 |  |
| 343 | 69 | C | C | C | 94.80 | 0.00000000 | \* |
| 343 | 69 | C | C | G | 0.00 | 0.90000000 |  |
| 343 | 69 | C | C | T | 3.07 | 0.17649206 |  |
| 344 | 70 | C | C | A | 0.00 | 0.78181818 |  |
| 344 | 70 | C | C | C | 98.53 | 0.00000000 | \* |
| 344 | 70 | C | C | G | 1.47 | 0.45389603 |  |
| 344 | 70 | C | C | T | 0.00 | 0.86021505 |  |
| 345 | 71 | G | G | A | 5.58 | 0.13049468 |  |
| 345 | 71 | G | G | C | 1.86 | 0.38423888 |  |
| 345 | 71 | G | G | G | 91.08 | 0.00000000 | \* |
| 345 | 71 | G | G | T | 1.49 | 0.55147552 |  |
| 346 | 72 | C | C | A | 0.00 | 0.78181818 |  |
| 346 | 72 | C | C | C | 97.45 | 0.00000000 | \* |
| 346 | 72 | C | C | G | 2.55 | 0.16444291 |  |
| 346 | 72 | C | C | T | 0.00 | 0.86021505 |  |
| 347 | 73 | G | G | A | 9.62 | 0.02236572 |  |
| 347 | 73 | G | G | C | 4.23 | 0.02715342 |  |
| 347 | 73 | G | G | G | 86.15 | 0.00000000 | \* |
| 347 | 73 | G | G | T | 0.00 | 0.86021505 |  |
| 348 | 74 | A | A | A | 94.50 | 0.00000000 | \* |
| 348 | 74 | A | A | C | 1.31 | 0.59649711 |  |
| 348 | 74 | A | A | G | 0.79 | 0.72466601 |  |
| 348 | 74 | A | A | T | 3.40 | 0.13349774 |  |
| 349 | 75 | C | C | A | 2.44 | 0.43683522 |  |
| 349 | 75 | C | C | C | 94.85 | 0.00000000 | \* |
| 349 | 75 | C | C | G | 0.00 | 0.90000000 |  |
| 349 | 75 | C | C | T | 2.71 | 0.23685258 |  |

## For use in R

If you want to work with the results in R, here is output that you can copy and paste in your terminal to get:

The base information:

```
structure(list(focal.base = c("A", "C", "G", "T"), avg.percsignal = c(93.7107519083965, 
94.2101677044492, 93.3937778960846, 94.6605011187459), avg.areasignal = c(372.705882352941, 
391.363636363636, 367.578947368421, 430.727272727273), crit.perc.area = c(11.3787108687544, 
5.0000972229123, 5.00442357894334, 6.12997449915228), mu = c(3.3783009216473, 
1.82888517962231, 1.69355425071851, 2.14941113231839), fillibens = c(0.986943374999102, 
0.996963790720135, 0.995846074956368, 0.994345060154427)), .Names = c("focal.base", 
"avg.percsignal", "avg.areasignal", "crit.perc.area", "mu", "fillibens"
), row.names = c(NA, -4L), class = "data.frame")
```

the data.frame that contains information on the guide region:

```
structure(list(A.area = c(341, 398, 10, 658, 16, 24, 32, 26, 
15, 7, 299, 13, 655, 16, 25, 21, 0, 26, 5, 6, 0, 19, 0, 20, 8, 
274, 12, 8, 369, 7, 271, 4, 10, 12, 7, 7, 297, 13, 22, 15, 6, 
19, 446, 10, 28, 20, 502, 14, 21, 14, 11, 177, 322, 6, 343, 323, 
4, 1, 32, 5, 14, 427, 8, 220, 8, 2, 0, 14, 9, 0, 15, 0, 25, 361, 
9), C.area = c(6, 5, 4, 8, 391, 16, 0, 8, 250, 445, 6, 4, 7, 
404, 354, 4, 4, 3, 1, 9, 9, 4, 0, 6, 346, 11, 411, 18, 5, 370, 
8, 334, 6, 0, 5, 272, 9, 419, 378, 366, 4, 11, 6, 372, 317, 3, 
5, 317, 4, 3, 211, 2, 0, 0, 0, 0, 3, 9, 3, 302, 6, 5, 347, 7, 
7, 276, 6, 314, 401, 269, 5, 267, 11, 5, 350), G.area = c(2, 
5, 315, 10, 0, 10, 668, 441, 9, 9, 6, 314, 6, 5, 7, 364, 7, 566, 
18, 8, 7, 508, 6, 415, 7, 12, 2, 3, 3, 6, 11, 7, 255, 380, 307, 
2, 6, 4, 6, 5, 9, 441, 6, 0, 18, 282, 4, 3, 194, 379, 4, 4, 4, 
250, 14, 2, 210, 2, 11, 9, 224, 7, 10, 5, 348, 8, 5, 4, 0, 4, 
245, 7, 224, 3, 0), T.area = c(11, 19, 0, 24, 10, 446, 8, 2, 
2, 0, 1, 1, 32, 7, 8, 7, 278, 3, 355, 381, 388, 5, 329, 5, 3, 
7, 7, 409, 5, 5, 0, 3, 0, 4, 7, 0, 14, 9, 0, 6, 371, 4, 18, 6, 
3, 2, 21, 0, 0, 4, 7, 0, 8, 1, 12, 15, 5, 282, 269, 3, 2, 18, 
5, 8, 4, 9, 390, 8, 13, 0, 4, 0, 0, 13, 10), Tot.area = c(360, 
427, 329, 700, 417, 496, 708, 477, 276, 461, 312, 332, 700, 432, 
394, 396, 289, 598, 379, 404, 404, 536, 335, 446, 364, 304, 432, 
438, 382, 388, 290, 348, 271, 396, 326, 281, 326, 445, 406, 392, 
390, 475, 476, 388, 366, 307, 532, 334, 219, 400, 233, 183, 334, 
257, 369, 340, 222, 294, 315, 319, 246, 457, 370, 240, 367, 295, 
401, 340, 423, 273, 269, 274, 260, 382, 369), A.perc = c(94.7222222222222, 
93.2084309133489, 3.03951367781155, 94, 3.83693045563549, 4.83870967741935, 
4.51977401129944, 5.45073375262055, 5.43478260869565, 1.51843817787419, 
95.8333333333333, 3.91566265060241, 93.5714285714286, 3.7037037037037, 
6.34517766497462, 5.3030303030303, 0, 4.34782608695652, 1.31926121372032, 
1.48514851485149, 0, 3.54477611940298, 0, 4.48430493273543, 2.1978021978022, 
90.1315789473684, 2.77777777777778, 1.82648401826484, 96.5968586387435, 
1.80412371134021, 93.448275862069, 1.14942528735632, 3.690036900369, 
3.03030303030303, 2.14723926380368, 2.49110320284698, 91.1042944785276, 
2.92134831460674, 5.41871921182266, 3.8265306122449, 1.53846153846154, 
4, 93.6974789915966, 2.57731958762887, 7.65027322404372, 6.51465798045603, 
94.3609022556391, 4.19161676646707, 9.58904109589041, 3.5, 4.72103004291846, 
96.7213114754098, 96.4071856287425, 2.33463035019455, 92.9539295392954, 
95, 1.8018018018018, 0.340136054421769, 10.1587301587302, 1.56739811912226, 
5.69105691056911, 93.4354485776805, 2.16216216216216, 91.6666666666667, 
2.17983651226158, 0.677966101694915, 0, 4.11764705882353, 2.12765957446809, 
0, 5.57620817843866, 0, 9.61538461538461, 94.5026178010471, 2.4390243902439
), C.perc = c(1.66666666666667, 1.17096018735363, 1.21580547112462, 
1.14285714285714, 93.7649880095923, 3.2258064516129, 0, 1.67714884696017, 
90.5797101449275, 96.529284164859, 1.92307692307692, 1.20481927710843, 
1, 93.5185185185185, 89.8477157360406, 1.01010101010101, 1.3840830449827, 
0.501672240802676, 0.263852242744063, 2.22772277227723, 2.22772277227723, 
0.746268656716418, 0, 1.34529147982063, 95.0549450549451, 3.61842105263158, 
95.1388888888889, 4.10958904109589, 1.30890052356021, 95.360824742268, 
2.75862068965517, 95.9770114942529, 2.2140221402214, 0, 1.53374233128834, 
96.797153024911, 2.76073619631902, 94.1573033707865, 93.1034482758621, 
93.3673469387755, 1.02564102564103, 2.31578947368421, 1.26050420168067, 
95.8762886597938, 86.6120218579235, 0.977198697068404, 0.93984962406015, 
94.9101796407186, 1.82648401826484, 0.75, 90.5579399141631, 1.09289617486339, 
0, 0, 0, 0, 1.35135135135135, 3.06122448979592, 0.952380952380952, 
94.6708463949843, 2.4390243902439, 1.09409190371991, 93.7837837837838, 
2.91666666666667, 1.90735694822888, 93.5593220338983, 1.49625935162095, 
92.3529411764706, 94.7990543735225, 98.5347985347985, 1.85873605947955, 
97.4452554744526, 4.23076923076923, 1.30890052356021, 94.8509485094851
), G.perc = c(0.555555555555556, 1.17096018735363, 95.7446808510638, 
1.42857142857143, 0, 2.01612903225806, 94.3502824858757, 92.4528301886792, 
3.26086956521739, 1.95227765726681, 1.92307692307692, 94.578313253012, 
0.857142857142857, 1.15740740740741, 1.77664974619289, 91.9191919191919, 
2.42214532871972, 94.6488294314381, 4.74934036939314, 1.98019801980198, 
1.73267326732673, 94.7761194029851, 1.7910447761194, 93.0493273542601, 
1.92307692307692, 3.94736842105263, 0.462962962962963, 0.684931506849315, 
0.785340314136126, 1.54639175257732, 3.79310344827586, 2.01149425287356, 
94.0959409594096, 95.959595959596, 94.1717791411043, 0.711743772241993, 
1.84049079754601, 0.898876404494382, 1.47783251231527, 1.27551020408163, 
2.30769230769231, 92.8421052631579, 1.26050420168067, 0, 4.91803278688525, 
91.85667752443, 0.75187969924812, 0.898203592814371, 88.5844748858448, 
94.75, 1.71673819742489, 2.18579234972678, 1.19760479041916, 
97.2762645914397, 3.7940379403794, 0.588235294117647, 94.5945945945946, 
0.680272108843537, 3.49206349206349, 2.82131661442006, 91.0569105691057, 
1.53172866520788, 2.7027027027027, 2.08333333333333, 94.8228882833787, 
2.71186440677966, 1.24688279301746, 1.17647058823529, 0, 1.46520146520147, 
91.0780669144981, 2.55474452554745, 86.1538461538462, 0.785340314136126, 
0), T.perc = c(3.05555555555556, 4.44964871194379, 0, 3.42857142857143, 
2.39808153477218, 89.9193548387097, 1.12994350282486, 0.419287211740042, 
0.72463768115942, 0, 0.320512820512821, 0.301204819277108, 4.57142857142857, 
1.62037037037037, 2.03045685279188, 1.76767676767677, 96.1937716262976, 
0.501672240802676, 93.6675461741425, 94.3069306930693, 96.039603960396, 
0.932835820895522, 98.2089552238806, 1.12107623318386, 0.824175824175824, 
2.30263157894737, 1.62037037037037, 93.37899543379, 1.30890052356021, 
1.28865979381443, 0, 0.862068965517241, 0, 1.01010101010101, 
2.14723926380368, 0, 4.29447852760736, 2.02247191011236, 0, 1.53061224489796, 
95.1282051282051, 0.842105263157895, 3.78151260504202, 1.54639175257732, 
0.819672131147541, 0.651465798045603, 3.94736842105263, 0, 0, 
1, 3.00429184549356, 0, 2.39520958083832, 0.389105058365759, 
3.2520325203252, 4.41176470588235, 2.25225225225225, 95.9183673469388, 
85.3968253968254, 0.940438871473354, 0.813008130081301, 3.93873085339168, 
1.35135135135135, 3.33333333333333, 1.08991825613079, 3.05084745762712, 
97.2568578553616, 2.35294117647059, 3.07328605200946, 0, 1.48698884758364, 
0, 0, 3.40314136125654, 2.710027100271), base.call = c("A", "A", 
"G", "A", "C", "T", "G", "G", "C", "C", "A", "G", "A", "C", "C", 
"G", "T", "G", "T", "T", "T", "G", "T", "G", "C", "A", "C", "T", 
"A", "C", "A", "C", "G", "G", "G", "C", "A", "C", "C", "C", "T", 
"G", "A", "C", "C", "G", "A", "C", "G", "G", "C", "A", "A", "G", 
"A", "A", "G", "T", "T", "C", "G", "A", "C", "A", "G", "C", "T", 
"C", "C", "C", "G", "C", "G", "A", "C"), index = 275:349, guide.seq = c("A", 
"A", "G", "A", "C", "T", "G", "G", "C", "C", "A", "G", "A", "C", 
"C", "G", "T", "G", "T", "T", "T", "G", "T", "G", "C", "A", "C", 
"T", "A", "C", "A", "C", "G", "G", "G", "C", "A", "C", "C", "C", 
"T", "G", "A", "C", "C", "G", "A", "C", "G", "G", "C", "A", "A", 
"G", "A", "A", "G", "T", "T", "C", "G", "A", "C", "A", "G", "C", 
"T", "C", "C", "C", "G", "C", "G", "A", "C"), T.pval = c(0.17910555311564, 
0.0518924163831133, 0.860215053763439, 0.130599348701117, 0.300941785643023, 
0, 0.663289128659079, 0.835327449697509, 0.776603784001167, 0.860215053763439, 
0.847072288937049, 0.848911528381448, 0.0462681020353299, 0.510158746455973, 
0.391659044422077, 0.465838989967863, 0, 0.822629758358693, 0, 
0, 0, 0.721765730463932, 0, 0.666007537325004, 0.751532869902711, 
0.322926172876621, 0.510158746455973, 0, 0.607488169813862, 0.613857324685247, 
0.860215053763439, 0.741406652550622, 0.860215053763439, 0.699402379054655, 
0.361091622025968, 0.860215053763439, 0.0599835032492683, 0.393806111832303, 
0.860215053763439, 0.537868356841012, 0, 0.746778145016476, 0.0957567992441819, 
0.532966438614034, 0.752716537834261, 0.793403688563188, 0.0824832088542046, 
0.860215053763439, 0.860215053763439, 0.702373337889763, 0.186846526387325, 
0.860215053763439, 0.301586937439055, 0.839327478464377, 0.151910603072278, 
0.053768250968517, 0.334979545926838, 0, 0, 0.719604480836076, 
0.754459945152407, 0.0831308269068126, 0.594114637748839, 0.141746325655566, 
0.675507559468029, 0.179805009971739, 0, 0.311199286733872, 0.176492057642506, 
0.860215053763439, 0.551475519589731, 0.860215053763439, 0.860215053763439, 
0.133497742996774, 0.236852576689837), C.pval = c(0.453365812633658, 
0.653978959683625, 0.63530169108596, 0.665644267148041, 0, 0.0923710351038716, 
0.929411764705882, 0.449425567688998, 0, 0, 0.362631769727302, 
0.639881951736342, 0.723890299641293, 0, 0, 0.719855258458212, 
0.565371584146343, 0.885584848563466, 0.921404655012937, 0.271649123035624, 
0.271649123035624, 0.817617226048926, 0.929411764705882, 0.581392204146186, 
0, 0.0580058851224863, 0, 0.0316440659776651, 0.596497105248072, 
0, 0.156359848853515, 0, 0.275335636872525, 0.929411764705882, 
0.504800527154002, 0, 0.155999620865143, 0, 0, 0, 0.713616968691558, 
0.248850778428813, 0.616656973270886, 0, 0, 0.732936556932712, 
0.747547756152456, 0, 0.3953725104421, 0.816378767166605, 0, 
0.6862564229677, 0.929411764705882, 0.929411764705882, 0.929411764705882, 
0.929411764705882, 0.578883371223139, 0.111601378179665, 0.742676004907636, 
0, 0.219505803168379, 0.685765497115798, 0, 0.131343588508065, 
0.367835715480548, 0, 0.519745075512331, 0, 0, 0, 0.384238878667907, 
0, 0.0271534164044689, 0.596497105248072, 0), G.pval = c(0.807984293457344, 
0.56810139719541, 0, 0.467458737226441, 0.899999999997222, 0.279687382052078, 
0, 0, 0.0772342696492569, 0.296864155908964, 0.304984320867001, 
0, 0.696179227150964, 0.573592525817557, 0.348228775712375, 0, 
0.188211190584224, 0, 0.013641052211722, 0.289255783266798, 0.362038158578386, 
0, 0.343790666177054, 0, 0.304984320867001, 0.0353815812911307, 
0.836199609185737, 0.762901569308662, 0.724666007060199, 0.424637328182136, 
0.0423019015204572, 0.280907581712088, 0, 0, 0, 0.752917946186942, 
0.328856053383104, 0.679328852954599, 0.449270210978329, 0.526290188879787, 
0.211028691424262, 0, 0.532222575536857, 0.899999999997222, 0.0111127717063815, 
0, 0.737650664847309, 0.679601803259409, 0, 0, 0.367135018786272, 
0.237832072698651, 0.557347875297352, 0, 0.0422563680801545, 
0.797147810179161, 0, 0.764617199275661, 0.0596358687226781, 
0.124464574052816, 0, 0.429836792627647, 0.141044730627419, 0.262458048835198, 
0, 0.139697543117766, 0.537629357669316, 0.565872726125454, 0.899999999997222, 
0.453896033214142, 0, 0.164442907544596, 0, 0.724666007060199, 
0.899999999997222), A.pval = c(0, 0, 0.353731313416583, 0, 0.262506119556554, 
0.176663848687212, 0.200834338139539, 0.137475201273117, 0.138386754692054, 
0.584092355677918, 0, 0.254657158561594, 0, 0.276248692496572, 
0.0943954736302313, 0.146130242000285, 0.781818181814129, 0.215040941609223, 
0.617476757235001, 0.58967281111815, 0.781818181814129, 0.293414522722495, 
0.781818181814129, 0.203695425827861, 0.473487634144006, 0, 0.388456135520306, 
0.532911458186381, 0, 0.536583586993399, 0, 0.645700314806116, 
0.277691605914953, 0.35491304902795, 0.481381737764281, 0.429154396501888, 
0, 0.369115551877702, 0.139310318098777, 0.263557929951805, 0.580737912241641, 
0.246470178455553, 0, 0.416629477935335, 0.0536872339608653, 
0.0878100324301887, 0, 0.228699434302715, 0.0226335483938866, 
0.298404760104627, 0.18526271234255, 0, 0, 0.452485339672094, 
0, 0, 0.536965396451047, 0.760854998930925, 0.0174817489050207, 
0.575894144646254, 0.124392768630487, 0, 0.4790447312734, 0, 
0.476284574923904, 0.719064450606573, 0.781818181814129, 0.235424618190021, 
0.484456979927156, 0.781818181814129, 0.130494676385036, 0.781818181814129, 
0.0223657248697171, 0, 0.436835215844812), guide.position = 1:75), .Names = c("A.area", 
"C.area", "G.area", "T.area", "Tot.area", "A.perc", "C.perc", 
"G.perc", "T.perc", "base.call", "index", "guide.seq", "T.pval", 
"C.pval", "G.pval", "A.pval", "guide.position"), row.names = 275:349, class = "data.frame")
```

*Report generated using EditR v1.0.8*
