# Supplementary material for: Mechanistic and genetic basis of single-strand templated repair at Cas12a-induced DNA breaks in Chlamydomonas reinhardtii
Source: Nat Commun. 2021 Nov 19;12:6751. doi: 10.1038/s41467-021-27004-1 (PMC8604939; doi:10.1038/s41467-021-27004-1)
Supplement: Supplementary file 22 — Source Data [file 41467_2021_27004_MOESM22_ESM.zip › Source Data/EditR analysis/EditR outputs/Antisense/rep3_ssODN_antisense_-16.html]

EditR v1.0.8 report


# EditR v1.0.8 report

- Data QA
  - Filtering data
  - Percent noise peak area
  - Base information
- Predicted editing
  - Editing bar plot
  - Editing table plot
  - Table of editing results
- For use in R

## Data QA

### Filtering data

What the data looked like prefiltering:

and the post filtering signal / noise plot:

### Percent noise peak area

### Base information

Here’s information about the signal of each base, the critical percent value where any higher value would be called as significant, and Filliben’s correlation for how well the noise was modelled by the zero adjusted gamma distribution.

| Base | Average percent signal | Average peak area | Critical percent value | model mu | Fillibens correlation |
| --- | --- | --- | --- | --- | --- |
| A | 95.59283 | 448.9714 | 9.440380 | 3.472476 | 0.9747374 |
| C | 92.76250 | 475.6212 | 4.574167 | 2.044120 | 0.9912102 |
| G | 93.24534 | 462.8947 | 4.477346 | 1.660093 | 0.9967473 |
| T | 94.20076 | 544.6512 | 4.700877 | 1.926897 | 0.9952569 |

## Predicted editing

### Editing bar plot

### Editing table plot

### Table of editing results


Here’s the entire guide region

| Sanger position | Guide position | Guide sequence | Sanger base call | Focal base | Focal base peak area | p value |  |
| --- | --- | --- | --- | --- | --- | --- | --- |
| 274 | 1 | A | A | A | 96.69 | 0.000000e+00 | \* |
| 274 | 1 | A | A | C | 2.13 | 3.872426e-01 |  |
| 274 | 1 | A | A | G | 0.71 | 8.562907e-01 |  |
| 274 | 1 | A | A | T | 0.47 | 9.007456e-01 |  |
| 275 | 2 | A | A | A | 95.77 | 0.000000e+00 | \* |
| 275 | 2 | A | A | C | 1.41 | 7.144950e-01 |  |
| 275 | 2 | A | A | G | 1.81 | 3.574316e-01 |  |
| 275 | 2 | A | A | T | 1.01 | 7.762252e-01 |  |
| 276 | 3 | G | G | A | 2.65 | 5.064896e-01 |  |
| 276 | 3 | G | G | C | 2.89 | 1.511537e-01 |  |
| 276 | 3 | G | G | G | 92.77 | 0.000000e+00 | \* |
| 276 | 3 | G | G | T | 1.69 | 4.943463e-01 |  |
| 277 | 4 | A | A | A | 95.99 | 0.000000e+00 | \* |
| 277 | 4 | A | A | C | 1.09 | 8.384566e-01 |  |
| 277 | 4 | A | A | G | 1.22 | 6.211136e-01 |  |
| 277 | 4 | A | A | T | 1.70 | 4.874817e-01 |  |
| 278 | 5 | C | C | A | 4.59 | 2.049342e-01 |  |
| 278 | 5 | C | C | C | 91.20 | 0.000000e+00 | \* |
| 278 | 5 | C | C | G | 2.10 | 2.592316e-01 |  |
| 278 | 5 | C | C | T | 2.10 | 3.341815e-01 |  |
| 279 | 6 | T | T | A | 3.82 | 3.040243e-01 |  |
| 279 | 6 | T | T | C | 3.02 | 1.260712e-01 |  |
| 279 | 6 | T | T | G | 2.23 | 2.248696e-01 |  |
| 279 | 6 | T | T | T | 90.94 | 0.000000e+00 | \* |
| 280 | 7 | G | G | A | 3.72 | 3.182876e-01 |  |
| 280 | 7 | G | G | C | 0.00 | 9.529412e-01 |  |
| 280 | 7 | G | G | G | 95.15 | 0.000000e+00 | \* |
| 280 | 7 | G | G | T | 1.13 | 7.308835e-01 |  |
| 281 | 8 | G | G | A | 4.54 | 2.105767e-01 |  |
| 281 | 8 | G | G | C | 1.51 | 6.671903e-01 |  |
| 281 | 8 | G | G | G | 93.28 | 0.000000e+00 | \* |
| 281 | 8 | G | G | T | 0.67 | 8.706514e-01 |  |
| 282 | 9 | C | C | A | 4.64 | 1.996629e-01 |  |
| 282 | 9 | C | C | C | 91.01 | 0.000000e+00 | \* |
| 282 | 9 | C | C | G | 2.03 | 2.819820e-01 |  |
| 282 | 9 | C | C | T | 2.32 | 2.656716e-01 |  |
| 283 | 10 | C | C | A | 3.27 | 3.917224e-01 |  |
| 283 | 10 | C | C | C | 92.25 | 0.000000e+00 | \* |
| 283 | 10 | C | C | G | 2.07 | 2.706413e-01 |  |
| 283 | 10 | C | C | T | 2.41 | 2.400595e-01 |  |
| 284 | 11 | A | A | A | 94.84 | 0.000000e+00 | \* |
| 284 | 11 | A | A | C | 2.21 | 3.537960e-01 |  |
| 284 | 11 | A | A | G | 2.70 | 1.247563e-01 |  |
| 284 | 11 | A | A | T | 0.25 | 9.135516e-01 |  |
| 285 | 12 | G | G | A | 2.87 | 4.642443e-01 |  |
| 285 | 12 | G | G | C | 1.44 | 7.024425e-01 |  |
| 285 | 12 | G | G | G | 94.74 | 0.000000e+00 | \* |
| 285 | 12 | G | G | T | 0.96 | 7.931709e-01 |  |
| 286 | 13 | A | A | A | 97.18 | 0.000000e+00 | \* |
| 286 | 13 | A | A | C | 0.98 | 8.730762e-01 |  |
| 286 | 13 | A | A | G | 0.86 | 7.930666e-01 |  |
| 286 | 13 | A | A | T | 0.98 | 7.847727e-01 |  |
| 287 | 14 | C | C | A | 4.99 | 1.647242e-01 |  |
| 287 | 14 | C | C | C | 91.31 | 0.000000e+00 | \* |
| 287 | 14 | C | C | G | 2.03 | 2.806288e-01 |  |
| 287 | 14 | C | C | T | 1.66 | 5.040854e-01 |  |
| 288 | 15 | C | C | A | 6.32 | 7.616416e-02 |  |
| 288 | 15 | C | C | C | 91.58 | 0.000000e+00 | \* |
| 288 | 15 | C | C | G | 2.11 | 2.586355e-01 |  |
| 288 | 15 | C | C | T | 0.00 | 9.148936e-01 |  |
| 289 | 16 | G | G | A | 5.61 | 1.158083e-01 |  |
| 289 | 16 | G | G | C | 1.40 | 7.169992e-01 |  |
| 289 | 16 | G | G | G | 91.58 | 0.000000e+00 | \* |
| 289 | 16 | G | G | T | 1.40 | 6.162235e-01 |  |
| 290 | 17 | T | T | A | 0.00 | 8.440367e-01 |  |
| 290 | 17 | T | T | C | 2.08 | 4.079223e-01 |  |
| 290 | 17 | T | T | G | 2.34 | 1.967862e-01 |  |
| 290 | 17 | T | T | T | 95.58 | 0.000000e+00 | \* |
| 291 | 18 | G | G | A | 3.94 | 2.864874e-01 |  |
| 291 | 18 | G | G | C | 0.00 | 9.529412e-01 |  |
| 291 | 18 | G | G | G | 95.28 | 0.000000e+00 | \* |
| 291 | 18 | G | G | T | 0.79 | 8.439763e-01 |  |
| 292 | 19 | T | T | A | 1.80 | 6.718695e-01 |  |
| 292 | 19 | T | T | C | 1.40 | 7.169992e-01 |  |
| 292 | 19 | T | T | G | 4.41 | 1.109873e-02 |  |
| 292 | 19 | T | T | T | 92.38 | 0.000000e+00 | \* |
| 293 | 20 | T | T | A | 0.95 | 8.027609e-01 |  |
| 293 | 20 | T | T | C | 1.89 | 4.888124e-01 |  |
| 293 | 20 | T | T | G | 2.65 | 1.332219e-01 |  |
| 293 | 20 | T | T | T | 94.51 | 0.000000e+00 | \* |
| 294 | 21 | T | T | A | 0.00 | 8.440367e-01 |  |
| 294 | 21 | T | T | C | 0.00 | 9.529412e-01 |  |
| 294 | 21 | T | T | G | 2.64 | 1.349342e-01 |  |
| 294 | 21 | T | T | T | 97.36 | 0.000000e+00 | \* |
| 295 | 22 | G | G | A | 2.06 | 6.224569e-01 |  |
| 295 | 22 | G | G | C | 1.91 | 4.794409e-01 |  |
| 295 | 22 | G | G | G | 95.14 | 0.000000e+00 | \* |
| 295 | 22 | G | G | T | 0.88 | 8.166932e-01 |  |
| 296 | 23 | T | T | A | 0.44 | 8.384451e-01 |  |
| 296 | 23 | T | T | C | 2.86 | 1.571300e-01 |  |
| 296 | 23 | T | T | G | 1.54 | 4.677965e-01 |  |
| 296 | 23 | T | T | T | 95.15 | 0.000000e+00 | \* |
| 297 | 24 | G | G | A | 3.26 | 3.933477e-01 |  |
| 297 | 24 | G | G | C | 1.45 | 6.961845e-01 |  |
| 297 | 24 | G | G | G | 93.84 | 0.000000e+00 | \* |
| 297 | 24 | G | G | T | 1.45 | 5.961301e-01 |  |
| 298 | 25 | C | C | A | 3.02 | 4.369589e-01 |  |
| 298 | 25 | C | C | C | 91.59 | 0.000000e+00 | \* |
| 298 | 25 | C | C | G | 2.16 | 2.441934e-01 |  |
| 298 | 25 | C | C | T | 3.23 | 8.618864e-02 |  |
| 299 | 26 | A | A | A | 91.34 | 0.000000e+00 | \* |
| 299 | 26 | A | A | C | 3.91 | 3.159747e-02 |  |
| 299 | 26 | A | A | G | 4.75 | 6.592895e-03 | \* |
| 299 | 26 | A | A | T | 0.00 | 9.148936e-01 |  |
| 300 | 27 | C | C | A | 2.83 | 4.723002e-01 |  |
| 300 | 27 | C | C | C | 83.03 | 0.000000e+00 | \* |
| 300 | 27 | C | C | G | 12.32 | 1.581170e-08 | \* |
| 300 | 27 | C | C | T | 1.82 | 4.404331e-01 |  |
| 301 | 28 | T | T | A | 1.51 | 7.246662e-01 |  |
| 301 | 28 | T | T | C | 3.95 | 2.934758e-02 |  |
| 301 | 28 | T | T | G | 0.94 | 7.549315e-01 |  |
| 301 | 28 | T | T | T | 93.60 | 0.000000e+00 | \* |
| 302 | 29 | A | A | A | 93.86 | 0.000000e+00 | \* |
| 302 | 29 | A | A | C | 1.97 | 4.529844e-01 |  |
| 302 | 29 | A | A | G | 2.19 | 2.336835e-01 |  |
| 302 | 29 | A | A | T | 1.97 | 3.804186e-01 |  |
| 303 | 30 | C | C | A | 2.37 | 5.622964e-01 |  |
| 303 | 30 | C | C | C | 92.70 | 0.000000e+00 | \* |
| 303 | 30 | C | C | G | 2.56 | 1.488404e-01 |  |
| 303 | 30 | C | C | T | 2.37 | 2.518863e-01 |  |
| 304 | 31 | A | A | A | 92.54 | 0.000000e+00 | \* |
| 304 | 31 | A | A | C | 2.76 | 1.801828e-01 |  |
| 304 | 31 | A | A | G | 4.70 | 7.149568e-03 | \* |
| 304 | 31 | A | A | T | 0.00 | 9.148936e-01 |  |
| 305 | 32 | C | C | A | 1.77 | 6.788427e-01 |  |
| 305 | 32 | C | C | C | 93.60 | 0.000000e+00 | \* |
| 305 | 32 | C | C | G | 2.43 | 1.762376e-01 |  |
| 305 | 32 | C | C | T | 2.21 | 2.997046e-01 |  |
| 306 | 33 | G | G | A | 3.48 | 3.565928e-01 |  |
| 306 | 33 | G | G | C | 2.32 | 3.134238e-01 |  |
| 306 | 33 | G | G | G | 94.20 | 0.000000e+00 | \* |
| 306 | 33 | G | G | T | 0.00 | 9.148936e-01 |  |
| 307 | 34 | G | G | A | 2.04 | 6.257464e-01 |  |
| 307 | 34 | G | G | C | 0.41 | 9.506616e-01 |  |
| 307 | 34 | G | G | G | 96.52 | 0.000000e+00 | \* |
| 307 | 34 | G | G | T | 1.02 | 7.703498e-01 |  |
| 308 | 35 | G | G | A | 2.95 | 4.496952e-01 |  |
| 308 | 35 | G | G | C | 1.47 | 6.848489e-01 |  |
| 308 | 35 | G | G | G | 94.35 | 0.000000e+00 | \* |
| 308 | 35 | G | G | T | 1.23 | 6.901641e-01 |  |
| 309 | 36 | C | C | A | 2.48 | 5.400618e-01 |  |
| 309 | 36 | C | C | C | 94.49 | 0.000000e+00 | \* |
| 309 | 36 | C | C | G | 1.38 | 5.433195e-01 |  |
| 309 | 36 | C | C | T | 1.65 | 5.086018e-01 |  |
| 310 | 37 | A | A | A | 94.71 | 0.000000e+00 | \* |
| 310 | 37 | A | A | C | 3.17 | 1.008237e-01 |  |
| 310 | 37 | A | A | G | 2.12 | 2.553556e-01 |  |
| 310 | 37 | A | A | T | 0.00 | 9.148936e-01 |  |
| 311 | 38 | C | C | A | 2.54 | 5.279552e-01 |  |
| 311 | 38 | C | C | C | 95.83 | 0.000000e+00 | \* |
| 311 | 38 | C | C | G | 0.00 | 9.777778e-01 |  |
| 311 | 38 | C | C | T | 1.63 | 5.168672e-01 |  |
| 312 | 39 | C | C | A | 4.42 | 2.243350e-01 |  |
| 312 | 39 | C | C | C | 93.17 | 0.000000e+00 | \* |
| 312 | 39 | C | C | G | 2.41 | 1.803066e-01 |  |
| 312 | 39 | C | C | T | 0.00 | 9.148936e-01 |  |
| 313 | 40 | C | C | A | 3.55 | 3.450980e-01 |  |
| 313 | 40 | C | C | C | 93.74 | 0.000000e+00 | \* |
| 313 | 40 | C | C | G | 0.00 | 9.777778e-01 |  |
| 313 | 40 | C | C | T | 2.71 | 1.677920e-01 |  |
| 314 | 41 | T | T | A | 0.76 | 8.198142e-01 |  |
| 314 | 41 | T | T | C | 2.49 | 2.569968e-01 |  |
| 314 | 41 | T | T | G | 1.91 | 3.208227e-01 |  |
| 314 | 41 | T | T | T | 94.84 | 0.000000e+00 | \* |
| 315 | 42 | G | G | A | 3.31 | 3.846378e-01 |  |
| 315 | 42 | G | G | C | 0.33 | 9.520893e-01 |  |
| 315 | 42 | G | G | G | 95.03 | 0.000000e+00 | \* |
| 315 | 42 | G | G | T | 1.32 | 6.498332e-01 |  |
| 316 | 43 | A | A | A | 97.00 | 0.000000e+00 | \* |
| 316 | 43 | A | A | C | 1.41 | 7.122814e-01 |  |
| 316 | 43 | A | A | G | 1.59 | 4.466900e-01 |  |
| 316 | 43 | A | A | T | 0.00 | 9.148936e-01 |  |
| 317 | 44 | C | C | A | 3.27 | 3.925763e-01 |  |
| 317 | 44 | C | C | C | 94.69 | 0.000000e+00 | \* |
| 317 | 44 | C | C | G | 0.00 | 9.777778e-01 |  |
| 317 | 44 | C | C | T | 2.04 | 3.560060e-01 |  |
| 318 | 45 | C | C | A | 5.82 | 1.026929e-01 |  |
| 318 | 45 | C | C | C | 88.37 | 0.000000e+00 | \* |
| 318 | 45 | C | C | G | 4.03 | 1.968308e-02 |  |
| 318 | 45 | C | C | T | 1.79 | 4.518930e-01 |  |
| 319 | 46 | G | G | A | 5.61 | 1.157394e-01 |  |
| 319 | 46 | G | G | C | 2.04 | 4.237083e-01 |  |
| 319 | 46 | G | G | G | 91.58 | 0.000000e+00 | \* |
| 319 | 46 | G | G | T | 0.77 | 8.496150e-01 |  |
| 320 | 47 | A | A | A | 96.37 | 0.000000e+00 | \* |
| 320 | 47 | A | A | C | 1.10 | 8.353669e-01 |  |
| 320 | 47 | A | A | G | 0.79 | 8.238375e-01 |  |
| 320 | 47 | A | A | T | 1.74 | 4.742603e-01 |  |
| 321 | 48 | C | C | A | 3.90 | 2.918849e-01 |  |
| 321 | 48 | C | C | C | 92.66 | 0.000000e+00 | \* |
| 321 | 48 | C | C | G | 0.92 | 7.662593e-01 |  |
| 321 | 48 | C | C | T | 2.52 | 2.107644e-01 |  |
| 322 | 49 | G | G | A | 7.72 | 3.151055e-02 |  |
| 322 | 49 | G | G | C | 2.46 | 2.664223e-01 |  |
| 322 | 49 | G | G | G | 89.82 | 0.000000e+00 | \* |
| 322 | 49 | G | G | T | 0.00 | 9.148936e-01 |  |
| 323 | 50 | G | G | A | 2.52 | 5.321736e-01 |  |
| 323 | 50 | G | G | C | 1.36 | 7.372148e-01 |  |
| 323 | 50 | G | G | G | 95.54 | 0.000000e+00 | \* |
| 323 | 50 | G | G | T | 0.58 | 8.868733e-01 |  |
| 324 | 51 | C | C | A | 2.35 | 5.658351e-01 |  |
| 324 | 51 | C | C | C | 92.28 | 0.000000e+00 | \* |
| 324 | 51 | C | C | G | 2.68 | 1.277006e-01 |  |
| 324 | 51 | C | C | T | 2.68 | 1.739047e-01 |  |
| 325 | 52 | A | A | A | 96.49 | 0.000000e+00 | \* |
| 325 | 52 | A | A | C | 1.32 | 7.545884e-01 |  |
| 325 | 52 | A | A | G | 1.32 | 5.727869e-01 |  |
| 325 | 52 | A | A | T | 0.88 | 8.186571e-01 |  |
| 326 | 53 | A | A | A | 97.12 | 0.000000e+00 | \* |
| 326 | 53 | A | A | C | 1.20 | 8.013146e-01 |  |
| 326 | 53 | A | A | G | 1.68 | 4.093946e-01 |  |
| 326 | 53 | A | A | T | 0.00 | 9.148936e-01 |  |
| 327 | 54 | G | G | A | 1.45 | 7.334482e-01 |  |
| 327 | 54 | G | G | C | 1.45 | 6.942762e-01 |  |
| 327 | 54 | G | G | G | 96.22 | 0.000000e+00 | \* |
| 327 | 54 | G | G | T | 0.87 | 8.201947e-01 |  |
| 328 | 55 | A | A | A | 97.53 | 0.000000e+00 | \* |
| 328 | 55 | A | A | C | 0.00 | 9.529412e-01 |  |
| 328 | 55 | A | A | G | 2.02 | 2.854930e-01 |  |
| 328 | 55 | A | A | T | 0.45 | 9.030829e-01 |  |
| 329 | 56 | A | A | A | 96.68 | 0.000000e+00 | \* |
| 329 | 56 | A | A | C | 0.51 | 9.468179e-01 |  |
| 329 | 56 | A | A | G | 1.28 | 5.922973e-01 |  |
| 329 | 56 | A | A | T | 1.53 | 5.609374e-01 |  |
| 330 | 57 | G | G | A | 1.41 | 7.399143e-01 |  |
| 330 | 57 | G | G | C | 1.41 | 7.122814e-01 |  |
| 330 | 57 | G | G | G | 94.70 | 0.000000e+00 | \* |
| 330 | 57 | G | G | T | 2.47 | 2.231882e-01 |  |
| 331 | 58 | T | T | A | 0.74 | 8.218562e-01 |  |
| 331 | 58 | T | T | C | 3.69 | 4.505407e-02 |  |
| 331 | 58 | T | T | G | 0.99 | 7.342123e-01 |  |
| 331 | 58 | T | T | T | 94.58 | 0.000000e+00 | \* |
| 332 | 59 | T | T | A | 0.93 | 8.045206e-01 |  |
| 332 | 59 | T | T | C | 1.63 | 6.132166e-01 |  |
| 332 | 59 | T | T | G | 3.49 | 4.297204e-02 |  |
| 332 | 59 | T | T | T | 93.95 | 0.000000e+00 | \* |
| 333 | 60 | C | C | A | 4.03 | 2.737740e-01 |  |
| 333 | 60 | C | C | C | 92.18 | 0.000000e+00 | \* |
| 333 | 60 | C | C | G | 2.37 | 1.893094e-01 |  |
| 333 | 60 | C | C | T | 1.42 | 6.080173e-01 |  |
| 334 | 61 | G | G | A | 6.85 | 5.496580e-02 |  |
| 334 | 61 | G | G | C | 3.27 | 8.696354e-02 |  |
| 334 | 61 | G | G | G | 88.69 | 0.000000e+00 | \* |
| 334 | 61 | G | G | T | 1.19 | 7.057326e-01 |  |
| 335 | 62 | A | A | A | 97.24 | 0.000000e+00 | \* |
| 335 | 62 | A | A | C | 1.29 | 7.656696e-01 |  |
| 335 | 62 | A | A | G | 1.47 | 4.986524e-01 |  |
| 335 | 62 | A | A | T | 0.00 | 9.148936e-01 |  |
| 336 | 63 | C | C | A | 2.87 | 4.646145e-01 |  |
| 336 | 63 | C | C | C | 93.24 | 0.000000e+00 | \* |
| 336 | 63 | C | C | G | 1.43 | 5.165640e-01 |  |
| 336 | 63 | C | C | T | 2.46 | 2.269323e-01 |  |
| 337 | 64 | A | A | A | 94.88 | 0.000000e+00 | \* |
| 337 | 64 | A | A | C | 2.05 | 4.207226e-01 |  |
| 337 | 64 | A | A | G | 3.07 | 7.658739e-02 |  |
| 337 | 64 | A | A | T | 0.00 | 9.148936e-01 |  |
| 338 | 65 | G | G | A | 2.75 | 4.871702e-01 |  |
| 338 | 65 | G | G | C | 2.55 | 2.360921e-01 |  |
| 338 | 65 | G | G | G | 93.52 | 0.000000e+00 | \* |
| 338 | 65 | G | G | T | 1.18 | 7.104630e-01 |  |
| 339 | 66 | C | C | A | 4.12 | 2.609739e-01 |  |
| 339 | 66 | C | C | C | 91.24 | 0.000000e+00 | \* |
| 339 | 66 | C | C | G | 2.06 | 2.717307e-01 |  |
| 339 | 66 | C | C | T | 2.58 | 1.977287e-01 |  |
| 340 | 67 | T | T | A | 1.66 | 6.985452e-01 |  |
| 340 | 67 | T | T | C | 0.74 | 9.249720e-01 |  |
| 340 | 67 | T | T | G | 1.84 | 3.460099e-01 |  |
| 340 | 67 | T | T | T | 95.76 | 0.000000e+00 | \* |
| 341 | 68 | C | C | A | 4.09 | 2.653293e-01 |  |
| 341 | 68 | C | C | C | 91.14 | 0.000000e+00 | \* |
| 341 | 68 | C | C | G | 2.50 | 1.612766e-01 |  |
| 341 | 68 | C | C | T | 2.27 | 2.794160e-01 |  |
| 342 | 69 | C | C | A | 3.39 | 3.717913e-01 |  |
| 342 | 69 | C | C | C | 91.80 | 0.000000e+00 | \* |
| 342 | 69 | C | C | G | 1.07 | 6.933572e-01 |  |
| 342 | 69 | C | C | T | 3.74 | 4.234269e-02 |  |
| 343 | 70 | C | C | A | 0.00 | 8.440367e-01 |  |
| 343 | 70 | C | C | C | 96.17 | 0.000000e+00 | \* |
| 343 | 70 | C | C | G | 1.47 | 4.979077e-01 |  |
| 343 | 70 | C | C | T | 2.36 | 2.538571e-01 |  |
| 344 | 71 | G | G | A | 5.97 | 9.399952e-02 |  |
| 344 | 71 | G | G | C | 0.85 | 9.044497e-01 |  |
| 344 | 71 | G | G | G | 91.19 | 0.000000e+00 | \* |
| 344 | 71 | G | G | T | 1.99 | 3.748987e-01 |  |
| 345 | 72 | C | C | A | 0.00 | 8.440367e-01 |  |
| 345 | 72 | C | C | C | 97.01 | 0.000000e+00 | \* |
| 345 | 72 | C | C | G | 1.09 | 6.848186e-01 |  |
| 345 | 72 | C | C | T | 1.90 | 4.074464e-01 |  |
| 346 | 73 | G | G | A | 8.70 | 1.655484e-02 |  |
| 346 | 73 | G | G | C | 3.48 | 6.354937e-02 |  |
| 346 | 73 | G | G | G | 86.67 | 0.000000e+00 | \* |
| 346 | 73 | G | G | T | 1.16 | 7.182296e-01 |  |
| 347 | 74 | A | A | A | 97.62 | 0.000000e+00 | \* |
| 347 | 74 | A | A | C | 1.51 | 6.675260e-01 |  |
| 347 | 74 | A | A | G | 0.86 | 7.907984e-01 |  |
| 347 | 74 | A | A | T | 0.00 | 9.148936e-01 |  |
| 348 | 75 | C | C | A | 3.85 | 2.995389e-01 |  |
| 348 | 75 | C | C | C | 91.90 | 0.000000e+00 | \* |
| 348 | 75 | C | C | G | 0.81 | 8.147901e-01 |  |
| 348 | 75 | C | C | T | 3.44 | 6.485186e-02 |  |

## For use in R

If you want to work with the results in R, here is output that you can copy and paste in your terminal to get:

The base information:

```
structure(list(focal.base = c("A", "C", "G", "T"), avg.percsignal = c(95.5928306689908, 
92.7624999408948, 93.2453408613498, 94.2007583203027), avg.areasignal = c(448.971428571429, 
475.621212121212, 462.894736842105, 544.651162790698), crit.perc.area = c(9.44037976795199, 
4.57416745875224, 4.47734642973485, 4.70087660959313), mu = c(3.47247633269191, 
2.04412025529494, 1.66009261564516, 1.92689663204411), fillibens = c(0.974737391653839, 
0.991210192407589, 0.996747329368431, 0.995256870277819)), .Names = c("focal.base", 
"avg.percsignal", "avg.areasignal", "crit.perc.area", "mu", "fillibens"
), row.names = c(NA, -4L), class = "data.frame")
```

the data.frame that contains information on the guide region:

```
structure(list(A.area = c(409, 476, 11, 789, 24, 24, 33, 27, 
16, 19, 386, 12, 792, 27, 30, 28, 0, 30, 9, 5, 0, 14, 2, 18, 
14, 327, 14, 8, 428, 12, 335, 8, 12, 10, 12, 9, 358, 14, 22, 
17, 4, 20, 549, 16, 26, 22, 611, 17, 22, 13, 7, 220, 405, 5, 
435, 379, 4, 3, 4, 17, 23, 528, 14, 278, 14, 16, 9, 18, 19, 0, 
21, 0, 30, 452, 19), C.area = c(9, 7, 12, 9, 477, 19, 0, 9, 314, 
536, 9, 6, 8, 494, 435, 7, 8, 0, 7, 10, 0, 13, 13, 8, 425, 14, 
411, 21, 9, 470, 10, 424, 8, 2, 6, 343, 12, 528, 464, 449, 13, 
2, 8, 464, 395, 8, 7, 404, 7, 7, 275, 3, 5, 5, 0, 2, 4, 15, 7, 
389, 11, 7, 455, 6, 13, 354, 4, 401, 515, 326, 3, 357, 12, 7, 
454), G.area = c(3, 9, 385, 10, 11, 14, 844, 555, 7, 12, 11, 
396, 7, 11, 10, 457, 9, 726, 22, 14, 14, 646, 7, 518, 10, 17, 
61, 5, 10, 13, 17, 11, 325, 472, 384, 5, 8, 0, 12, 0, 10, 574, 
9, 0, 18, 359, 5, 4, 256, 493, 8, 3, 7, 331, 9, 5, 268, 4, 15, 
10, 298, 8, 7, 9, 476, 8, 10, 11, 6, 5, 321, 4, 299, 4, 4), T.area = c(2, 
5, 7, 14, 11, 572, 10, 4, 8, 14, 1, 4, 8, 9, 0, 7, 368, 6, 461, 
499, 516, 6, 432, 8, 15, 0, 9, 497, 9, 12, 0, 10, 0, 5, 5, 6, 
0, 9, 0, 13, 496, 8, 0, 10, 8, 3, 11, 11, 0, 3, 8, 2, 0, 3, 2, 
6, 7, 384, 404, 6, 4, 0, 12, 0, 6, 10, 520, 10, 21, 8, 7, 7, 
4, 0, 17), Tot.area = c(423, 497, 415, 822, 523, 629, 887, 595, 
345, 581, 407, 418, 815, 541, 475, 499, 385, 762, 499, 528, 530, 
679, 454, 552, 464, 358, 495, 531, 456, 507, 362, 453, 345, 489, 
407, 363, 378, 551, 498, 479, 523, 604, 566, 490, 447, 392, 634, 
436, 285, 516, 298, 228, 417, 344, 446, 392, 283, 406, 430, 422, 
336, 543, 488, 293, 509, 388, 543, 440, 561, 339, 352, 368, 345, 
463, 494), A.perc = c(96.6903073286052, 95.7746478873239, 2.65060240963855, 
95.985401459854, 4.58891013384321, 3.81558028616852, 3.72040586245772, 
4.53781512605042, 4.63768115942029, 3.27022375215146, 94.8402948402948, 
2.87081339712919, 97.1779141104294, 4.99075785582255, 6.31578947368421, 
5.61122244488978, 0, 3.93700787401575, 1.80360721442886, 0.946969696969697, 
0, 2.06185567010309, 0.440528634361233, 3.26086956521739, 3.01724137931034, 
91.340782122905, 2.82828282828283, 1.50659133709981, 93.859649122807, 
2.36686390532544, 92.5414364640884, 1.76600441501104, 3.47826086956522, 
2.04498977505112, 2.94840294840295, 2.47933884297521, 94.7089947089947, 
2.54083484573503, 4.41767068273092, 3.54906054279749, 0.764818355640535, 
3.3112582781457, 96.9964664310954, 3.26530612244898, 5.8165548098434, 
5.61224489795918, 96.3722397476341, 3.89908256880734, 7.71929824561404, 
2.51937984496124, 2.3489932885906, 96.4912280701754, 97.1223021582734, 
1.45348837209302, 97.5336322869955, 96.6836734693878, 1.41342756183746, 
0.738916256157635, 0.930232558139535, 4.02843601895735, 6.84523809523809, 
97.2375690607735, 2.86885245901639, 94.8805460750853, 2.75049115913556, 
4.12371134020619, 1.65745856353591, 4.09090909090909, 3.38680926916221, 
0, 5.96590909090909, 0, 8.69565217391304, 97.6241900647948, 3.84615384615385
), C.perc = c(2.12765957446809, 1.40845070422535, 2.89156626506024, 
1.09489051094891, 91.2045889101339, 3.02066772655008, 0, 1.51260504201681, 
91.0144927536232, 92.2547332185886, 2.21130221130221, 1.43540669856459, 
0.98159509202454, 91.3123844731978, 91.5789473684211, 1.40280561122244, 
2.07792207792208, 0, 1.40280561122244, 1.89393939393939, 0, 1.91458026509573, 
2.86343612334802, 1.44927536231884, 91.5948275862069, 3.91061452513966, 
83.030303030303, 3.95480225988701, 1.97368421052632, 92.7021696252466, 
2.76243093922652, 93.598233995585, 2.31884057971015, 0.408997955010225, 
1.47420147420147, 94.4903581267218, 3.17460317460317, 95.8257713248639, 
93.1726907630522, 93.7369519832985, 2.48565965583174, 0.33112582781457, 
1.41342756183746, 94.6938775510204, 88.3668903803132, 2.04081632653061, 
1.10410094637224, 92.6605504587156, 2.45614035087719, 1.35658914728682, 
92.2818791946309, 1.31578947368421, 1.19904076738609, 1.45348837209302, 
0, 0.510204081632653, 1.41342756183746, 3.69458128078818, 1.62790697674419, 
92.1800947867299, 3.27380952380952, 1.28913443830571, 93.2377049180328, 
2.04778156996587, 2.55402750491159, 91.2371134020619, 0.736648250460405, 
91.1363636363636, 91.8003565062389, 96.165191740413, 0.852272727272727, 
97.0108695652174, 3.47826086956522, 1.51187904967603, 91.9028340080972
), G.perc = c(0.709219858156028, 1.81086519114688, 92.7710843373494, 
1.21654501216545, 2.10325047801147, 2.22575516693164, 95.152198421646, 
93.2773109243697, 2.02898550724638, 2.06540447504303, 2.7027027027027, 
94.7368421052632, 0.858895705521472, 2.03327171903882, 2.10526315789474, 
91.5831663326653, 2.33766233766234, 95.2755905511811, 4.40881763527054, 
2.65151515151515, 2.64150943396226, 95.139911634757, 1.54185022026432, 
93.8405797101449, 2.1551724137931, 4.74860335195531, 12.3232323232323, 
0.941619585687382, 2.19298245614035, 2.56410256410256, 4.69613259668508, 
2.42825607064018, 94.2028985507246, 96.5235173824131, 94.3488943488943, 
1.37741046831956, 2.11640211640212, 0, 2.40963855421687, 0, 1.91204588910134, 
95.0331125827815, 1.59010600706714, 0, 4.02684563758389, 91.5816326530612, 
0.788643533123028, 0.917431192660551, 89.8245614035088, 95.5426356589147, 
2.68456375838926, 1.31578947368421, 1.67865707434053, 96.2209302325581, 
2.01793721973094, 1.27551020408163, 94.6996466431095, 0.985221674876847, 
3.48837209302326, 2.3696682464455, 88.6904761904762, 1.47329650092081, 
1.4344262295082, 3.07167235494881, 93.516699410609, 2.06185567010309, 
1.84162062615101, 2.5, 1.06951871657754, 1.47492625368732, 91.1931818181818, 
1.08695652173913, 86.6666666666667, 0.863930885529158, 0.809716599190283
), T.perc = c(0.472813238770686, 1.00603621730382, 1.68674698795181, 
1.70316301703163, 2.10325047801147, 90.9379968203498, 1.12739571589628, 
0.672268907563025, 2.31884057971015, 2.40963855421687, 0.245700245700246, 
0.956937799043062, 0.98159509202454, 1.66358595194085, 0, 1.40280561122244, 
95.5844155844156, 0.78740157480315, 92.3847695390781, 94.5075757575758, 
97.3584905660377, 0.883652430044183, 95.1541850220264, 1.44927536231884, 
3.23275862068966, 0, 1.81818181818182, 93.5969868173258, 1.97368421052632, 
2.36686390532544, 0, 2.2075055187638, 0, 1.02249488752556, 1.22850122850123, 
1.65289256198347, 0, 1.63339382940109, 0, 2.71398747390397, 94.8374760994264, 
1.32450331125828, 0, 2.04081632653061, 1.78970917225951, 0.76530612244898, 
1.73501577287066, 2.52293577981651, 0, 0.581395348837209, 2.68456375838926, 
0.87719298245614, 0, 0.872093023255814, 0.448430493273543, 1.53061224489796, 
2.47349823321555, 94.5812807881773, 93.953488372093, 1.4218009478673, 
1.19047619047619, 0, 2.45901639344262, 0, 1.17878192534381, 2.57731958762887, 
95.7642725598527, 2.27272727272727, 3.74331550802139, 2.3598820058997, 
1.98863636363636, 1.90217391304348, 1.15942028985507, 0, 3.4412955465587
), base.call = c("A", "A", "G", "A", "C", "T", "G", "G", "C", 
"C", "A", "G", "A", "C", "C", "G", "T", "G", "T", "T", "T", "G", 
"T", "G", "C", "A", "C", "T", "A", "C", "A", "C", "G", "G", "G", 
"C", "A", "C", "C", "C", "T", "G", "A", "C", "C", "G", "A", "C", 
"G", "G", "C", "A", "A", "G", "A", "A", "G", "T", "T", "C", "G", 
"A", "C", "A", "G", "C", "T", "C", "C", "C", "G", "C", "G", "A", 
"C"), index = 274:348, guide.seq = c("A", "A", "G", "A", "C", 
"T", "G", "G", "C", "C", "A", "G", "A", "C", "C", "G", "T", "G", 
"T", "T", "T", "G", "T", "G", "C", "A", "C", "T", "A", "C", "A", 
"C", "G", "G", "G", "C", "A", "C", "C", "C", "T", "G", "A", "C", 
"C", "G", "A", "C", "G", "G", "C", "A", "A", "G", "A", "A", "G", 
"T", "T", "C", "G", "A", "C", "A", "G", "C", "T", "C", "C", "C", 
"G", "C", "G", "A", "C"), T.pval = c(0.900745606381717, 0.776225212618779, 
0.494346313107639, 0.487481721366606, 0.334181467465558, 0, 0.730883497640554, 
0.870651417627092, 0.265671640524044, 0.240059540474935, 0.913551570857217, 
0.793170940342893, 0.784772738032348, 0.504085351223794, 0.91489361696974, 
0.616223517230759, 0, 0.843976304055098, 0, 0, 0, 0.816693156896756, 
0, 0.596130063522749, 0.0861886385213715, 0.91489361696974, 0.440433070478253, 
0, 0.380418625752163, 0.25188629724976, 0.91489361696974, 0.299704646521432, 
0.91489361696974, 0.770349773766586, 0.690164130330984, 0.508601784209974, 
0.91489361696974, 0.516867229618708, 0.91489361696974, 0.167792045547085, 
0, 0.649833196373669, 0.91489361696974, 0.356006013409505, 0.451893033421568, 
0.849614966920683, 0.474260312338729, 0.210764425385951, 0.91489361696974, 
0.886873317039636, 0.173904747396962, 0.81865711362454, 0.91489361696974, 
0.820194682177452, 0.903082927353768, 0.560937373268679, 0.223188160464491, 
0, 0, 0.608017308180566, 0.705732610602043, 0.91489361696974, 
0.226932314189369, 0.91489361696974, 0.710462973856295, 0.197728675346849, 
0, 0.279416024749151, 0.0423426887004944, 0.253857071445914, 
0.374898666641605, 0.407446412833567, 0.718229649974289, 0.91489361696974, 
0.0648518584932863), C.pval = c(0.387242580446469, 0.714494994980576, 
0.151153655845828, 0.838456591580318, 0, 0.126071240312448, 0.952941176470502, 
0.667190277031211, 0, 0, 0.353796031818487, 0.702442477211223, 
0.873076221281085, 0, 0, 0.716999192098713, 0.407922307481169, 
0.952941176470502, 0.716999192098713, 0.488812376402618, 0.952941176470502, 
0.479440905155546, 0.157130047434741, 0.696184455261611, 0, 0.0315974666019261, 
0, 0.0293475787211261, 0.452984374867557, 0, 0.180182812085939, 
0, 0.313423759669951, 0.950661649963491, 0.684848851921002, 0, 
0.100823722479598, 0, 0, 0, 0.256996751361437, 0.952089319531264, 
0.712281363091934, 0, 0, 0.423708281282773, 0.835366937776538, 
0, 0.266422253860342, 0.737214810074673, 0, 0.754588414267709, 
0.801314589924895, 0.694276227152543, 0.952941176470502, 0.946817880670553, 
0.712281363091934, 0.0450540687968263, 0.613216615849704, 0, 
0.0869635370410924, 0.76566960399064, 0, 0.420722614657829, 0.236092092327786, 
0, 0.924972024384692, 0, 0, 0, 0.904449691795564, 0, 0.0635493694410373, 
0.667526029457443, 0), G.pval = c(0.856290673471983, 0.357431571749339, 
0, 0.621113647843757, 0.259231597627773, 0.224869625010307, 0, 
0, 0.281982043381406, 0.27064128654229, 0.124756327026641, 0, 
0.793066587552072, 0.280628826153915, 0.258635463156608, 0, 0.196786248738733, 
0, 0.0110987253380018, 0.133221945086824, 0.134934207963468, 
0, 0.467796497752869, 0, 0.244193355429584, 0.00659289468802438, 
1.58116958415278e-08, 0.754931460856707, 0.233683458317957, 0.148840366212202, 
0.00714956832765523, 0.176237648221382, 0, 0, 0, 0.543319514835139, 
0.255355553630935, 0.977777777777777, 0.180306562040514, 0.977777777777777, 
0.320822671044779, 0, 0.446689974118709, 0.977777777777777, 0.0196830752191667, 
0, 0.823837503499652, 0.766259342460387, 0, 0, 0.127700625688752, 
0.572786913904612, 0.40939464082875, 0, 0.285492981621993, 0.592297275272009, 
0, 0.734212274491864, 0.0429720401001787, 0.18930940163273, 0, 
0.498652436742984, 0.516564046235712, 0.0765873898668193, 0, 
0.271730736246452, 0.34600989360619, 0.161276586156269, 0.693357190655096, 
0.497907732210536, 0, 0.684818613103052, 0, 0.7907984003226, 
0.814790082498511), A.pval = c(0, 0, 0.506489620375881, 0, 0.204934181904002, 
0.304024343938366, 0.31828755945013, 0.210576653116846, 0.199662855165991, 
0.391722354069831, 0, 0.464244285248697, 0, 0.164724165253155, 
0.0761641598152325, 0.115808254661939, 0.844036697247707, 0.286487417606664, 
0.671869503448413, 0.802760877058637, 0.844036697247707, 0.622456898956806, 
0.838445115974426, 0.393347685531163, 0.436958916673456, 0, 0.472300238964848, 
0.724666207542664, 0, 0.562296419697003, 0, 0.67884270588247, 
0.356592767160663, 0.625746378318117, 0.449695152018661, 0.540061818668798, 
0, 0.527955226972947, 0.224334972743338, 0.345097987616406, 0.81981418450416, 
0.384637831185728, 0, 0.392576338538228, 0.102692871011249, 0.115739447554138, 
0, 0.291884885623528, 0.0315105476906695, 0.532173578426238, 
0.565835079867003, 0, 0, 0.733448194528086, 0, 0, 0.739914284560945, 
0.821856194498081, 0.804520581591556, 0.273773969029455, 0.0549658021357361, 
0, 0.464614501380544, 0, 0.487170216159259, 0.26097393700904, 
0.69854519757593, 0.26532928707338, 0.371791330924276, 0.844036697247707, 
0.0939995205583665, 0.844036697247707, 0.0165548360360739, 0, 
0.299538862293043), guide.position = 1:75), .Names = c("A.area", 
"C.area", "G.area", "T.area", "Tot.area", "A.perc", "C.perc", 
"G.perc", "T.perc", "base.call", "index", "guide.seq", "T.pval", 
"C.pval", "G.pval", "A.pval", "guide.position"), row.names = 274:348, class = "data.frame")
```

*Report generated using EditR v1.0.8*
