# Supplementary material for: Mechanistic and genetic basis of single-strand templated repair at Cas12a-induced DNA breaks in Chlamydomonas reinhardtii
Source: Nat Commun. 2021 Nov 19;12:6751. doi: 10.1038/s41467-021-27004-1 (PMC8604939; doi:10.1038/s41467-021-27004-1)
Supplement: Supplementary file 22 — Source Data [file 41467_2021_27004_MOESM22_ESM.zip › Source Data/EditR analysis/EditR outputs/Antisense/rep3_ssODN_antisense_32.html]

EditR v1.0.8 report


# EditR v1.0.8 report

- Data QA
  - Filtering data
  - Percent noise peak area
  - Base information
- Predicted editing
  - Editing bar plot
  - Editing table plot
  - Table of editing results
- For use in R

## Data QA

### Filtering data

What the data looked like prefiltering:

and the post filtering signal / noise plot:

### Percent noise peak area

### Base information

Here’s information about the signal of each base, the critical percent value where any higher value would be called as significant, and Filliben’s correlation for how well the noise was modelled by the zero adjusted gamma distribution.

| Base | Average percent signal | Average peak area | Critical percent value | model mu | Fillibens correlation |
| --- | --- | --- | --- | --- | --- |
| A | 92.53063 | 369.0588 | 7.994021 | 2.788153 | 0.9574587 |
| C | 92.77527 | 393.8696 | 4.397848 | 1.774900 | 0.9928875 |
| G | 94.36960 | 390.8364 | 5.306312 | 1.918112 | 0.9964369 |
| T | 95.04745 | 462.3023 | 5.770930 | 2.207595 | 0.9972054 |

## Predicted editing

### Editing bar plot

### Editing table plot

### Table of editing results


Here’s the entire guide region

| Sanger position | Guide position | Guide sequence | Sanger base call | Focal base | Focal base peak area | p value |  |
| --- | --- | --- | --- | --- | --- | --- | --- |
| 279 | 1 | A | A | A | 92.09 | 0.000000e+00 | \* |
| 279 | 1 | A | A | C | 1.98 | 3.282626e-01 |  |
| 279 | 1 | A | A | G | 0.85 | 7.668183e-01 |  |
| 279 | 1 | A | A | T | 5.08 | 2.226667e-02 |  |
| 280 | 2 | A | A | A | 91.96 | 0.000000e+00 | \* |
| 280 | 2 | A | A | C | 0.95 | 7.840120e-01 |  |
| 280 | 2 | A | A | G | 1.42 | 5.564409e-01 |  |
| 280 | 2 | A | A | T | 5.67 | 1.122021e-02 |  |
| 281 | 3 | G | G | A | 2.69 | 4.011676e-01 |  |
| 281 | 3 | G | G | C | 4.19 | 1.405549e-02 |  |
| 281 | 3 | G | G | G | 93.11 | 0.000000e+00 | \* |
| 281 | 3 | G | G | T | 0.00 | 9.042553e-01 |  |
| 282 | 4 | A | A | A | 92.96 | 0.000000e+00 | \* |
| 282 | 4 | A | A | C | 1.13 | 7.054947e-01 |  |
| 282 | 4 | A | A | G | 0.70 | 8.110793e-01 |  |
| 282 | 4 | A | A | T | 5.21 | 1.925536e-02 |  |
| 283 | 5 | C | C | A | 4.66 | 1.188732e-01 |  |
| 283 | 5 | C | C | C | 91.14 | 0.000000e+00 | \* |
| 283 | 5 | C | C | G | 2.33 | 2.660705e-01 |  |
| 283 | 5 | C | C | T | 1.86 | 4.961429e-01 |  |
| 284 | 6 | T | T | A | 3.90 | 1.968181e-01 |  |
| 284 | 6 | T | T | C | 1.36 | 5.935628e-01 |  |
| 284 | 6 | T | T | G | 2.34 | 2.640812e-01 |  |
| 284 | 6 | T | T | T | 92.40 | 0.000000e+00 | \* |
| 285 | 7 | G | G | A | 2.31 | 4.883266e-01 |  |
| 285 | 7 | G | G | C | 0.00 | 9.390244e-01 |  |
| 285 | 7 | G | G | G | 96.60 | 0.000000e+00 | \* |
| 285 | 7 | G | G | T | 1.09 | 7.579302e-01 |  |
| 286 | 8 | G | G | A | 3.43 | 2.626708e-01 |  |
| 286 | 8 | G | G | C | 1.82 | 3.896587e-01 |  |
| 286 | 8 | G | G | G | 93.33 | 0.000000e+00 | \* |
| 286 | 8 | G | G | T | 1.41 | 6.515495e-01 |  |
| 287 | 9 | C | C | A | 4.63 | 1.217940e-01 |  |
| 287 | 9 | C | C | C | 90.04 | 0.000000e+00 | \* |
| 287 | 9 | C | C | G | 2.14 | 3.171422e-01 |  |
| 287 | 9 | C | C | T | 3.20 | 1.635602e-01 |  |
| 288 | 10 | C | C | A | 3.26 | 2.913640e-01 |  |
| 288 | 10 | C | C | C | 94.35 | 0.000000e+00 | \* |
| 288 | 10 | C | C | G | 2.39 | 2.516725e-01 |  |
| 288 | 10 | C | C | T | 0.00 | 9.042553e-01 |  |
| 289 | 11 | A | A | A | 91.67 | 0.000000e+00 | \* |
| 289 | 11 | A | A | C | 2.08 | 2.911621e-01 |  |
| 289 | 11 | A | A | G | 2.98 | 1.420490e-01 |  |
| 289 | 11 | A | A | T | 3.27 | 1.528190e-01 |  |
| 290 | 12 | G | G | A | 2.86 | 3.671415e-01 |  |
| 290 | 12 | G | G | C | 0.86 | 8.181914e-01 |  |
| 290 | 12 | G | G | G | 94.00 | 0.000000e+00 | \* |
| 290 | 12 | G | G | T | 2.29 | 3.646502e-01 |  |
| 291 | 13 | A | A | A | 93.46 | 0.000000e+00 | \* |
| 291 | 13 | A | A | C | 1.02 | 7.540396e-01 |  |
| 291 | 13 | A | A | G | 0.87 | 7.586145e-01 |  |
| 291 | 13 | A | A | T | 4.65 | 3.631139e-02 |  |
| 292 | 14 | C | C | A | 4.62 | 1.224076e-01 |  |
| 292 | 14 | C | C | C | 91.92 | 0.000000e+00 | \* |
| 292 | 14 | C | C | G | 1.62 | 4.834022e-01 |  |
| 292 | 14 | C | C | T | 1.85 | 5.019270e-01 |  |
| 293 | 15 | C | C | A | 5.43 | 6.966850e-02 |  |
| 293 | 15 | C | C | C | 91.47 | 0.000000e+00 | \* |
| 293 | 15 | C | C | G | 1.81 | 4.169229e-01 |  |
| 293 | 15 | C | C | T | 1.29 | 6.929964e-01 |  |
| 294 | 16 | G | G | A | 3.06 | 3.272585e-01 |  |
| 294 | 16 | G | G | C | 2.04 | 3.056845e-01 |  |
| 294 | 16 | G | G | G | 93.62 | 0.000000e+00 | \* |
| 294 | 16 | G | G | T | 1.28 | 6.984781e-01 |  |
| 295 | 17 | T | T | A | 2.46 | 4.532654e-01 |  |
| 295 | 17 | T | T | C | 1.23 | 6.571217e-01 |  |
| 295 | 17 | T | T | G | 2.46 | 2.356790e-01 |  |
| 295 | 17 | T | T | T | 93.85 | 0.000000e+00 | \* |
| 296 | 18 | G | G | A | 1.93 | 5.830828e-01 |  |
| 296 | 18 | G | G | C | 0.00 | 9.390244e-01 |  |
| 296 | 18 | G | G | G | 97.58 | 0.000000e+00 | \* |
| 296 | 18 | G | G | T | 0.48 | 8.865668e-01 |  |
| 297 | 19 | T | T | A | 2.72 | 3.965674e-01 |  |
| 297 | 19 | T | T | C | 0.00 | 9.390244e-01 |  |
| 297 | 19 | T | T | G | 3.21 | 1.114526e-01 |  |
| 297 | 19 | T | T | T | 94.07 | 0.000000e+00 | \* |
| 298 | 20 | T | T | A | 0.00 | 9.090909e-01 |  |
| 298 | 20 | T | T | C | 0.94 | 7.858083e-01 |  |
| 298 | 20 | T | T | G | 2.82 | 1.657746e-01 |  |
| 298 | 20 | T | T | T | 96.24 | 0.000000e+00 | \* |
| 299 | 21 | T | T | A | 0.00 | 9.090909e-01 |  |
| 299 | 21 | T | T | C | 0.00 | 9.390244e-01 |  |
| 299 | 21 | T | T | G | 2.15 | 3.136158e-01 |  |
| 299 | 21 | T | T | T | 97.85 | 0.000000e+00 | \* |
| 300 | 22 | G | G | A | 0.56 | 8.811266e-01 |  |
| 300 | 22 | G | G | C | 0.19 | 9.381926e-01 |  |
| 300 | 22 | G | G | G | 98.50 | 0.000000e+00 | \* |
| 300 | 22 | G | G | T | 0.75 | 8.445725e-01 |  |
| 301 | 23 | T | T | A | 0.00 | 9.090909e-01 |  |
| 301 | 23 | T | T | C | 2.18 | 2.600413e-01 |  |
| 301 | 23 | T | T | G | 1.09 | 6.808871e-01 |  |
| 301 | 23 | T | T | T | 96.73 | 0.000000e+00 | \* |
| 302 | 24 | G | G | A | 0.68 | 8.658099e-01 |  |
| 302 | 24 | G | G | C | 1.14 | 7.010995e-01 |  |
| 302 | 24 | G | G | G | 95.91 | 0.000000e+00 | \* |
| 302 | 24 | G | G | T | 2.27 | 3.683734e-01 |  |
| 303 | 25 | C | C | A | 2.50 | 4.444146e-01 |  |
| 303 | 25 | C | C | C | 92.22 | 0.000000e+00 | \* |
| 303 | 25 | C | C | G | 1.67 | 4.656127e-01 |  |
| 303 | 25 | C | C | T | 3.61 | 1.096175e-01 |  |
| 304 | 26 | A | A | A | 85.58 | 0.000000e+00 | \* |
| 304 | 26 | A | A | C | 3.85 | 2.453838e-02 |  |
| 304 | 26 | A | A | G | 3.21 | 1.120111e-01 |  |
| 304 | 26 | A | A | T | 7.37 | 1.402257e-03 | \* |
| 305 | 27 | C | C | A | 2.39 | 4.694247e-01 |  |
| 305 | 27 | C | C | C | 95.93 | 0.000000e+00 | \* |
| 305 | 27 | C | C | G | 0.00 | 9.021739e-01 |  |
| 305 | 27 | C | C | T | 1.67 | 5.610924e-01 |  |
| 306 | 28 | T | T | A | 1.97 | 5.747388e-01 |  |
| 306 | 28 | T | T | C | 3.06 | 8.136121e-02 |  |
| 306 | 28 | T | T | G | 0.66 | 8.246893e-01 |  |
| 306 | 28 | T | T | T | 94.32 | 0.000000e+00 | \* |
| 307 | 29 | A | A | A | 93.98 | 0.000000e+00 | \* |
| 307 | 29 | A | A | C | 3.66 | 3.263333e-02 |  |
| 307 | 29 | A | A | G | 0.79 | 7.867854e-01 |  |
| 307 | 29 | A | A | T | 1.57 | 5.972413e-01 |  |
| 308 | 30 | C | C | A | 2.22 | 5.103403e-01 |  |
| 308 | 30 | C | C | C | 95.56 | 0.000000e+00 | \* |
| 308 | 30 | C | C | G | 0.00 | 9.021739e-01 |  |
| 308 | 30 | C | C | T | 2.22 | 3.830743e-01 |  |
| 309 | 31 | A | A | A | 89.63 | 0.000000e+00 | \* |
| 309 | 31 | A | A | C | 2.68 | 1.384577e-01 |  |
| 309 | 31 | A | A | G | 3.68 | 6.716467e-02 |  |
| 309 | 31 | A | A | T | 4.01 | 7.243268e-02 |  |
| 310 | 32 | C | C | A | 1.37 | 7.251926e-01 |  |
| 310 | 32 | C | C | C | 93.96 | 0.000000e+00 | \* |
| 310 | 32 | C | C | G | 1.92 | 3.799739e-01 |  |
| 310 | 32 | C | C | T | 2.75 | 2.483022e-01 |  |
| 311 | 33 | G | G | A | 1.10 | 7.882224e-01 |  |
| 311 | 33 | G | G | C | 2.21 | 2.520788e-01 |  |
| 311 | 33 | G | G | G | 95.96 | 0.000000e+00 | \* |
| 311 | 33 | G | G | T | 0.74 | 8.479352e-01 |  |
| 312 | 34 | G | G | A | 0.00 | 9.090909e-01 |  |
| 312 | 34 | G | G | C | 0.75 | 8.551983e-01 |  |
| 312 | 34 | G | G | G | 98.00 | 0.000000e+00 | \* |
| 312 | 34 | G | G | T | 1.25 | 7.079226e-01 |  |
| 313 | 35 | G | G | A | 0.00 | 9.090909e-01 |  |
| 313 | 35 | G | G | C | 1.22 | 6.606694e-01 |  |
| 313 | 35 | G | G | G | 95.72 | 0.000000e+00 | \* |
| 313 | 35 | G | G | T | 3.06 | 1.874332e-01 |  |
| 314 | 36 | C | C | A | 3.81 | 2.087063e-01 |  |
| 314 | 36 | C | C | C | 93.77 | 0.000000e+00 | \* |
| 314 | 36 | C | C | G | 1.04 | 7.000547e-01 |  |
| 314 | 36 | C | C | T | 1.38 | 6.618596e-01 |  |
| 315 | 37 | A | A | A | 90.03 | 0.000000e+00 | \* |
| 315 | 37 | A | A | C | 2.80 | 1.162654e-01 |  |
| 315 | 37 | A | A | G | 1.87 | 3.971458e-01 |  |
| 315 | 37 | A | A | T | 5.30 | 1.746026e-02 |  |
| 316 | 38 | C | C | A | 3.36 | 2.753930e-01 |  |
| 316 | 38 | C | C | C | 95.97 | 0.000000e+00 | \* |
| 316 | 38 | C | C | G | 0.67 | 8.203313e-01 |  |
| 316 | 38 | C | C | T | 0.00 | 9.042553e-01 |  |
| 317 | 39 | C | C | A | 5.84 | 5.161358e-02 |  |
| 317 | 39 | C | C | C | 89.25 | 0.000000e+00 | \* |
| 317 | 39 | C | C | G | 2.80 | 1.690856e-01 |  |
| 317 | 39 | C | C | T | 2.10 | 4.191877e-01 |  |
| 318 | 40 | C | C | A | 4.14 | 1.688403e-01 |  |
| 318 | 40 | C | C | C | 89.54 | 0.000000e+00 | \* |
| 318 | 40 | C | C | G | 2.19 | 3.022417e-01 |  |
| 318 | 40 | C | C | T | 4.14 | 6.359982e-02 |  |
| 319 | 41 | T | T | A | 2.52 | 4.404963e-01 |  |
| 319 | 41 | T | T | C | 0.92 | 7.960862e-01 |  |
| 319 | 41 | T | T | G | 2.06 | 3.386871e-01 |  |
| 319 | 41 | T | T | T | 94.51 | 0.000000e+00 | \* |
| 320 | 42 | G | G | A | 1.79 | 6.188963e-01 |  |
| 320 | 42 | G | G | C | 1.79 | 4.000286e-01 |  |
| 320 | 42 | G | G | G | 95.02 | 0.000000e+00 | \* |
| 320 | 42 | G | G | T | 1.39 | 6.583199e-01 |  |
| 321 | 43 | A | A | A | 93.86 | 0.000000e+00 | \* |
| 321 | 43 | A | A | C | 1.27 | 6.379932e-01 |  |
| 321 | 43 | A | A | G | 1.48 | 5.322501e-01 |  |
| 321 | 43 | A | A | T | 3.39 | 1.365472e-01 |  |
| 322 | 44 | C | C | A | 3.01 | 3.374424e-01 |  |
| 322 | 44 | C | C | C | 94.49 | 0.000000e+00 | \* |
| 322 | 44 | C | C | G | 0.00 | 9.021739e-01 |  |
| 322 | 44 | C | C | T | 2.51 | 3.051261e-01 |  |
| 323 | 45 | C | C | A | 8.87 | 4.969734e-03 | \* |
| 323 | 45 | C | C | C | 86.29 | 0.000000e+00 | \* |
| 323 | 45 | C | C | G | 4.30 | 3.318950e-02 |  |
| 323 | 45 | C | C | T | 0.54 | 8.802421e-01 |  |
| 324 | 46 | G | G | A | 3.16 | 3.082941e-01 |  |
| 324 | 46 | G | G | C | 3.16 | 6.957199e-02 |  |
| 324 | 46 | G | G | G | 92.41 | 0.000000e+00 | \* |
| 324 | 46 | G | G | T | 1.27 | 7.016856e-01 |  |
| 325 | 47 | A | A | A | 93.15 | 0.000000e+00 | \* |
| 325 | 47 | A | A | C | 1.11 | 7.126182e-01 |  |
| 325 | 47 | A | A | G | 0.74 | 8.004129e-01 |  |
| 325 | 47 | A | A | T | 5.00 | 2.452726e-02 |  |
| 326 | 48 | C | C | A | 3.88 | 1.994077e-01 |  |
| 326 | 48 | C | C | C | 91.97 | 0.000000e+00 | \* |
| 326 | 48 | C | C | G | 0.83 | 7.722042e-01 |  |
| 326 | 48 | C | C | T | 3.32 | 1.455737e-01 |  |
| 327 | 49 | G | G | A | 8.47 | 6.829262e-03 | \* |
| 327 | 49 | G | G | C | 1.69 | 4.414801e-01 |  |
| 327 | 49 | G | G | G | 88.14 | 0.000000e+00 | \* |
| 327 | 49 | G | G | T | 1.69 | 5.540750e-01 |  |
| 328 | 50 | G | G | A | 1.85 | 6.037181e-01 |  |
| 328 | 50 | G | G | C | 0.69 | 8.710485e-01 |  |
| 328 | 50 | G | G | G | 96.30 | 0.000000e+00 | \* |
| 328 | 50 | G | G | T | 1.16 | 7.366913e-01 |  |
| 329 | 51 | C | C | A | 3.90 | 1.971360e-01 |  |
| 329 | 51 | C | C | C | 93.07 | 0.000000e+00 | \* |
| 329 | 51 | C | C | G | 3.03 | 1.343757e-01 |  |
| 329 | 51 | C | C | T | 0.00 | 9.042553e-01 |  |
| 330 | 52 | A | A | A | 92.39 | 0.000000e+00 | \* |
| 330 | 52 | A | A | C | 1.02 | 7.549891e-01 |  |
| 330 | 52 | A | A | G | 1.02 | 7.083895e-01 |  |
| 330 | 52 | A | A | T | 5.58 | 1.247666e-02 |  |
| 331 | 53 | A | A | A | 94.80 | 0.000000e+00 | \* |
| 331 | 53 | A | A | C | 0.29 | 9.350571e-01 |  |
| 331 | 53 | A | A | G | 1.16 | 6.560648e-01 |  |
| 331 | 53 | A | A | T | 3.76 | 9.450218e-02 |  |
| 332 | 54 | G | G | A | 3.18 | 3.054933e-01 |  |
| 332 | 54 | G | G | C | 0.00 | 9.390244e-01 |  |
| 332 | 54 | G | G | G | 96.11 | 0.000000e+00 | \* |
| 332 | 54 | G | G | T | 0.71 | 8.535005e-01 |  |
| 333 | 55 | A | A | A | 93.64 | 0.000000e+00 | \* |
| 333 | 55 | A | A | C | 0.25 | 9.365058e-01 |  |
| 333 | 55 | A | A | G | 2.54 | 2.178353e-01 |  |
| 333 | 55 | A | A | T | 3.56 | 1.151169e-01 |  |
| 334 | 56 | A | A | A | 94.83 | 0.000000e+00 | \* |
| 334 | 56 | A | A | C | 0.00 | 9.390244e-01 |  |
| 334 | 56 | A | A | G | 0.91 | 7.450705e-01 |  |
| 334 | 56 | A | A | T | 4.26 | 5.598963e-02 |  |
| 335 | 57 | G | G | A | 1.69 | 6.459271e-01 |  |
| 335 | 57 | G | G | C | 2.11 | 2.824039e-01 |  |
| 335 | 57 | G | G | G | 92.83 | 0.000000e+00 | \* |
| 335 | 57 | G | G | T | 3.38 | 1.384694e-01 |  |
| 336 | 58 | T | T | A | 1.58 | 6.742052e-01 |  |
| 336 | 58 | T | T | C | 3.47 | 4.406880e-02 |  |
| 336 | 58 | T | T | G | 0.63 | 8.310159e-01 |  |
| 336 | 58 | T | T | T | 94.32 | 0.000000e+00 | \* |
| 337 | 59 | T | T | A | 1.13 | 7.815729e-01 |  |
| 337 | 59 | T | T | C | 1.42 | 5.688926e-01 |  |
| 337 | 59 | T | T | G | 3.40 | 9.109187e-02 |  |
| 337 | 59 | T | T | T | 94.05 | 0.000000e+00 | \* |
| 338 | 60 | C | C | A | 3.87 | 2.005613e-01 |  |
| 338 | 60 | C | C | C | 92.86 | 0.000000e+00 | \* |
| 338 | 60 | C | C | G | 1.79 | 4.246289e-01 |  |
| 338 | 60 | C | C | T | 1.49 | 6.259797e-01 |  |
| 339 | 61 | G | G | A | 3.80 | 2.092272e-01 |  |
| 339 | 61 | G | G | C | 1.52 | 5.198262e-01 |  |
| 339 | 61 | G | G | G | 93.92 | 0.000000e+00 | \* |
| 339 | 61 | G | G | T | 0.76 | 8.427958e-01 |  |
| 340 | 62 | A | A | A | 94.35 | 0.000000e+00 | \* |
| 340 | 62 | A | A | C | 1.30 | 6.222273e-01 |  |
| 340 | 62 | A | A | G | 1.96 | 3.695619e-01 |  |
| 340 | 62 | A | A | T | 2.39 | 3.352691e-01 |  |
| 341 | 63 | C | C | A | 3.43 | 2.633486e-01 |  |
| 341 | 63 | C | C | C | 94.46 | 0.000000e+00 | \* |
| 341 | 63 | C | C | G | 1.06 | 6.936772e-01 |  |
| 341 | 63 | C | C | T | 1.06 | 7.677598e-01 |  |
| 342 | 64 | A | A | A | 91.06 | 0.000000e+00 | \* |
| 342 | 64 | A | A | C | 2.03 | 3.085759e-01 |  |
| 342 | 64 | A | A | G | 2.44 | 2.407158e-01 |  |
| 342 | 64 | A | A | T | 4.47 | 4.427112e-02 |  |
| 343 | 65 | G | G | A | 1.76 | 6.276562e-01 |  |
| 343 | 65 | G | G | C | 2.01 | 3.165017e-01 |  |
| 343 | 65 | G | G | G | 94.72 | 0.000000e+00 | \* |
| 343 | 65 | G | G | T | 1.51 | 6.192243e-01 |  |
| 344 | 66 | C | C | A | 3.30 | 2.846346e-01 |  |
| 344 | 66 | C | C | C | 92.41 | 0.000000e+00 | \* |
| 344 | 66 | C | C | G | 1.98 | 3.623039e-01 |  |
| 344 | 66 | C | C | T | 2.31 | 3.576864e-01 |  |
| 345 | 67 | T | T | A | 0.90 | 8.293698e-01 |  |
| 345 | 67 | T | T | C | 1.80 | 3.963407e-01 |  |
| 345 | 67 | T | T | G | 2.03 | 3.482273e-01 |  |
| 345 | 67 | T | T | T | 95.27 | 0.000000e+00 | \* |
| 346 | 68 | C | C | A | 3.41 | 2.667050e-01 |  |
| 346 | 68 | C | C | C | 92.33 | 0.000000e+00 | \* |
| 346 | 68 | C | C | G | 2.56 | 2.152884e-01 |  |
| 346 | 68 | C | C | T | 1.70 | 5.507474e-01 |  |
| 347 | 69 | C | C | A | 3.20 | 3.022819e-01 |  |
| 347 | 69 | C | C | C | 92.11 | 0.000000e+00 | \* |
| 347 | 69 | C | C | G | 0.64 | 8.287480e-01 |  |
| 347 | 69 | C | C | T | 4.05 | 6.960390e-02 |  |
| 348 | 70 | C | C | A | 3.64 | 2.312464e-01 |  |
| 348 | 70 | C | C | C | 91.06 | 0.000000e+00 | \* |
| 348 | 70 | C | C | G | 0.99 | 7.162955e-01 |  |
| 348 | 70 | C | C | T | 4.30 | 5.308970e-02 |  |
| 349 | 71 | G | G | A | 3.81 | 2.087063e-01 |  |
| 349 | 71 | G | G | C | 1.04 | 7.451231e-01 |  |
| 349 | 71 | G | G | G | 92.39 | 0.000000e+00 | \* |
| 349 | 71 | G | G | T | 2.77 | 2.437755e-01 |  |
| 350 | 72 | C | C | A | 4.56 | 1.273733e-01 |  |
| 350 | 72 | C | C | C | 91.86 | 0.000000e+00 | \* |
| 350 | 72 | C | C | G | 1.30 | 6.002373e-01 |  |
| 350 | 72 | C | C | T | 2.28 | 3.662482e-01 |  |
| 351 | 73 | G | G | A | 5.45 | 6.827664e-02 |  |
| 351 | 73 | G | G | C | 3.64 | 3.411602e-02 |  |
| 351 | 73 | G | G | G | 89.45 | 0.000000e+00 | \* |
| 351 | 73 | G | G | T | 1.45 | 6.376091e-01 |  |
| 352 | 74 | A | A | A | 92.93 | 0.000000e+00 | \* |
| 352 | 74 | A | A | C | 1.26 | 6.420542e-01 |  |
| 352 | 74 | A | A | G | 0.76 | 7.953435e-01 |  |
| 352 | 74 | A | A | T | 5.05 | 2.315525e-02 |  |
| 353 | 75 | C | C | A | 3.05 | 3.287216e-01 |  |
| 353 | 75 | C | C | C | 79.64 | 0.000000e+00 | \* |
| 353 | 75 | C | C | G | 13.74 | 1.167900e-07 | \* |
| 353 | 75 | C | C | T | 3.56 | 1.151169e-01 |  |

## For use in R

If you want to work with the results in R, here is output that you can copy and paste in your terminal to get:

The base information:

```
structure(list(focal.base = c("A", "C", "G", "T"), avg.percsignal = c(92.5306307068815, 
92.7752670997377, 94.3695984597682, 95.0474535035694), avg.areasignal = c(369.058823529412, 
393.869565217391, 390.836363636364, 462.302325581395), crit.perc.area = c(7.99402062022708, 
4.39784765903177, 5.30631228877825, 5.77093000787554), mu = c(2.78815272233596, 
1.77489991451837, 1.91811203272341, 2.2075945676942), fillibens = c(0.957458741965303, 
0.992887472346013, 0.996436916986668, 0.997205385015152)), .Names = c("focal.base", 
"avg.percsignal", "avg.areasignal", "crit.perc.area", "mu", "fillibens"
), row.names = c(NA, -4L), class = "data.frame")
```

the data.frame that contains information on the guide region:

```
structure(list(A.area = c(326, 389, 9, 660, 20, 20, 17, 17, 13, 
15, 308, 10, 643, 20, 21, 12, 8, 12, 11, 0, 0, 3, 0, 3, 9, 267, 
10, 9, 359, 9, 268, 5, 3, 0, 0, 11, 289, 15, 25, 17, 11, 9, 443, 
12, 33, 10, 503, 14, 20, 8, 9, 182, 328, 9, 368, 312, 4, 5, 4, 
13, 10, 434, 13, 224, 7, 10, 4, 12, 15, 11, 11, 14, 15, 368, 
12), C.area = c(7, 4, 14, 8, 391, 7, 0, 9, 253, 434, 7, 3, 7, 
398, 354, 8, 4, 0, 0, 4, 0, 1, 8, 5, 332, 12, 401, 14, 14, 387, 
8, 342, 6, 3, 4, 271, 9, 429, 382, 368, 4, 9, 6, 377, 321, 10, 
6, 332, 4, 3, 215, 2, 1, 0, 1, 0, 5, 11, 5, 312, 4, 6, 358, 5, 
8, 280, 8, 325, 432, 275, 3, 282, 10, 5, 313), G.area = c(3, 
6, 311, 5, 10, 12, 710, 462, 6, 11, 10, 329, 6, 7, 7, 367, 8, 
606, 13, 12, 9, 524, 4, 422, 6, 10, 0, 3, 3, 0, 11, 7, 261, 393, 
313, 3, 6, 3, 12, 9, 9, 477, 7, 0, 16, 292, 4, 3, 208, 416, 7, 
2, 4, 272, 10, 3, 220, 2, 12, 6, 247, 9, 4, 6, 377, 6, 9, 9, 
3, 3, 267, 4, 246, 3, 54), T.area = c(18, 24, 0, 37, 8, 474, 
8, 7, 9, 0, 11, 8, 32, 8, 5, 5, 305, 3, 381, 409, 410, 4, 355, 
10, 13, 23, 7, 432, 6, 9, 12, 10, 2, 5, 10, 4, 17, 0, 9, 17, 
413, 7, 16, 10, 2, 4, 27, 12, 4, 5, 0, 11, 13, 2, 14, 14, 8, 
299, 332, 5, 2, 11, 4, 11, 6, 7, 423, 6, 19, 13, 8, 7, 4, 20, 
14), Tot.area = c(354, 423, 334, 710, 429, 513, 735, 495, 281, 
460, 336, 350, 688, 433, 387, 392, 325, 621, 405, 425, 419, 532, 
367, 440, 360, 312, 418, 458, 382, 405, 299, 364, 272, 401, 327, 
289, 321, 447, 428, 411, 437, 502, 472, 399, 372, 316, 540, 361, 
236, 432, 231, 197, 346, 283, 393, 329, 237, 317, 353, 336, 263, 
460, 379, 246, 398, 303, 444, 352, 469, 302, 289, 307, 275, 396, 
393), A.perc = c(92.090395480226, 91.9621749408984, 2.69461077844311, 
92.9577464788732, 4.66200466200466, 3.89863547758285, 2.31292517006803, 
3.43434343434343, 4.62633451957295, 3.26086956521739, 91.6666666666667, 
2.85714285714286, 93.4593023255814, 4.6189376443418, 5.42635658914729, 
3.06122448979592, 2.46153846153846, 1.93236714975845, 2.71604938271605, 
0, 0, 0.56390977443609, 0, 0.681818181818182, 2.5, 85.5769230769231, 
2.39234449760766, 1.96506550218341, 93.979057591623, 2.22222222222222, 
89.6321070234114, 1.37362637362637, 1.10294117647059, 0, 0, 3.80622837370242, 
90.0311526479751, 3.35570469798658, 5.8411214953271, 4.13625304136253, 
2.51716247139588, 1.79282868525896, 93.8559322033898, 3.00751879699248, 
8.87096774193548, 3.16455696202532, 93.1481481481482, 3.8781163434903, 
8.47457627118644, 1.85185185185185, 3.8961038961039, 92.3857868020305, 
94.7976878612717, 3.18021201413428, 93.6386768447837, 94.8328267477204, 
1.68776371308017, 1.57728706624606, 1.13314447592068, 3.86904761904762, 
3.80228136882129, 94.3478260869565, 3.43007915567282, 91.0569105691057, 
1.75879396984925, 3.3003300330033, 0.900900900900901, 3.40909090909091, 
3.19829424307036, 3.64238410596026, 3.80622837370242, 4.56026058631922, 
5.45454545454545, 92.9292929292929, 3.05343511450382), C.perc = c(1.9774011299435, 
0.945626477541371, 4.19161676646707, 1.12676056338028, 91.1421911421911, 
1.364522417154, 0, 1.81818181818182, 90.0355871886121, 94.3478260869565, 
2.08333333333333, 0.857142857142857, 1.01744186046512, 91.9168591224018, 
91.4728682170543, 2.04081632653061, 1.23076923076923, 0, 0, 0.941176470588235, 
0, 0.18796992481203, 2.17983651226158, 1.13636363636364, 92.2222222222222, 
3.84615384615385, 95.933014354067, 3.05676855895197, 3.66492146596859, 
95.5555555555556, 2.67558528428094, 93.956043956044, 2.20588235294118, 
0.748129675810474, 1.22324159021407, 93.7716262975779, 2.80373831775701, 
95.9731543624161, 89.2523364485981, 89.5377128953771, 0.91533180778032, 
1.79282868525896, 1.27118644067797, 94.4862155388471, 86.2903225806452, 
3.16455696202532, 1.11111111111111, 91.9667590027701, 1.69491525423729, 
0.694444444444444, 93.0735930735931, 1.01522842639594, 0.289017341040462, 
0, 0.254452926208651, 0, 2.10970464135021, 3.47003154574133, 
1.41643059490085, 92.8571428571429, 1.52091254752852, 1.30434782608696, 
94.4591029023747, 2.03252032520325, 2.01005025125628, 92.4092409240924, 
1.8018018018018, 92.3295454545455, 92.1108742004264, 91.0596026490066, 
1.03806228373702, 91.85667752443, 3.63636363636364, 1.26262626262626, 
79.6437659033079), G.perc = c(0.847457627118644, 1.41843971631206, 
93.1137724550898, 0.704225352112676, 2.33100233100233, 2.33918128654971, 
96.5986394557823, 93.3333333333333, 2.13523131672598, 2.39130434782609, 
2.97619047619048, 94, 0.872093023255814, 1.61662817551963, 1.80878552971576, 
93.6224489795918, 2.46153846153846, 97.5845410628019, 3.20987654320988, 
2.82352941176471, 2.14797136038186, 98.4962406015038, 1.08991825613079, 
95.9090909090909, 1.66666666666667, 3.20512820512821, 0, 0.655021834061135, 
0.785340314136126, 0, 3.67892976588629, 1.92307692307692, 95.9558823529412, 
98.0049875311721, 95.7186544342508, 1.03806228373702, 1.86915887850467, 
0.671140939597315, 2.80373831775701, 2.18978102189781, 2.05949656750572, 
95.0199203187251, 1.48305084745763, 0, 4.3010752688172, 92.4050632911392, 
0.740740740740741, 0.831024930747922, 88.135593220339, 96.2962962962963, 
3.03030303030303, 1.01522842639594, 1.15606936416185, 96.113074204947, 
2.54452926208651, 0.911854103343465, 92.8270042194093, 0.630914826498423, 
3.39943342776204, 1.78571428571429, 93.9163498098859, 1.95652173913043, 
1.05540897097625, 2.4390243902439, 94.7236180904523, 1.98019801980198, 
2.02702702702703, 2.55681818181818, 0.639658848614073, 0.993377483443709, 
92.3875432525952, 1.30293159609121, 89.4545454545455, 0.757575757575758, 
13.7404580152672), T.perc = c(5.08474576271186, 5.67375886524823, 
0, 5.2112676056338, 1.86480186480186, 92.3976608187134, 1.08843537414966, 
1.41414141414141, 3.20284697508897, 0, 3.27380952380952, 2.28571428571429, 
4.65116279069767, 1.84757505773672, 1.29198966408269, 1.27551020408163, 
93.8461538461538, 0.483091787439614, 94.0740740740741, 96.2352941176471, 
97.8520286396181, 0.75187969924812, 96.7302452316076, 2.27272727272727, 
3.61111111111111, 7.37179487179487, 1.67464114832536, 94.3231441048035, 
1.57068062827225, 2.22222222222222, 4.0133779264214, 2.74725274725275, 
0.735294117647059, 1.24688279301746, 3.05810397553517, 1.3840830449827, 
5.29595015576324, 0, 2.10280373831776, 4.13625304136253, 94.5080091533181, 
1.39442231075697, 3.38983050847458, 2.5062656641604, 0.537634408602151, 
1.26582278481013, 5, 3.32409972299169, 1.69491525423729, 1.15740740740741, 
0, 5.58375634517767, 3.75722543352601, 0.706713780918728, 3.56234096692112, 
4.25531914893617, 3.37552742616034, 94.3217665615142, 94.0509915014164, 
1.48809523809524, 0.760456273764259, 2.39130434782609, 1.05540897097625, 
4.47154471544715, 1.50753768844221, 2.31023102310231, 95.2702702702703, 
1.70454545454545, 4.05117270788913, 4.3046357615894, 2.7681660899654, 
2.28013029315961, 1.45454545454545, 5.05050505050505, 3.56234096692112
), base.call = c("A", "A", "G", "A", "C", "T", "G", "G", "C", 
"C", "A", "G", "A", "C", "C", "G", "T", "G", "T", "T", "T", "G", 
"T", "G", "C", "A", "C", "T", "A", "C", "A", "C", "G", "G", "G", 
"C", "A", "C", "C", "C", "T", "G", "A", "C", "C", "G", "A", "C", 
"G", "G", "C", "A", "A", "G", "A", "A", "G", "T", "T", "C", "G", 
"A", "C", "A", "G", "C", "T", "C", "C", "C", "G", "C", "G", "A", 
"C"), index = 279:353, guide.seq = c("A", "A", "G", "A", "C", 
"T", "G", "G", "C", "C", "A", "G", "A", "C", "C", "G", "T", "G", 
"T", "T", "T", "G", "T", "G", "C", "A", "C", "T", "A", "C", "A", 
"C", "G", "G", "G", "C", "A", "C", "C", "C", "T", "G", "A", "C", 
"C", "G", "A", "C", "G", "G", "C", "A", "A", "G", "A", "A", "G", 
"T", "T", "C", "G", "A", "C", "A", "G", "C", "T", "C", "C", "C", 
"G", "C", "G", "A", "C"), T.pval = c(0.022266673941934, 0.011220211276209, 
0.904255319142527, 0.0192553612979316, 0.496142871004974, 0, 
0.757930225444671, 0.651549530290083, 0.163560170468306, 0.904255319142527, 
0.152819012409499, 0.364650245626681, 0.0363113942916949, 0.501926990588078, 
0.692996355976146, 0.698478126019502, 0, 0.886566836590246, 0, 
0, 0, 0.844572515088243, 0, 0.36837338242709, 0.109617548483133, 
0.0014022565970947, 0.561092356265645, 0, 0.59724127441621, 0.383074347061663, 
0.0724326840939414, 0.248302246828541, 0.847935236092194, 0.707922615012164, 
0.187433227491474, 0.661859643196389, 0.0174602595148653, 0.904255319142527, 
0.41918768917209, 0.063599821254978, 0, 0.658319939008535, 0.136547233559813, 
0.305126075547318, 0.880242116401382, 0.701685588834324, 0.024527258883272, 
0.145573689828087, 0.554074969749366, 0.736691271898029, 0.904255319142527, 
0.0124766633548254, 0.0945021751286065, 0.853500481923571, 0.115116915604926, 
0.055989630780902, 0.138469445763861, 0, 0, 0.625979727112838, 
0.842795811542022, 0.335269118153217, 0.767759779137045, 0.0442711167208001, 
0.619224300403917, 0.3576863657682, 0, 0.550747367750481, 0.0696038966712191, 
0.0530897007038719, 0.243775527153875, 0.366248181579826, 0.637609104263597, 
0.0231552465919832, 0.115116915604926), C.pval = c(0.328262574615417, 
0.784012041986751, 0.0140554865721927, 0.705494668841411, 0, 
0.593562842353682, 0.939024390243902, 0.389658684041407, 0, 0, 
0.291162108206829, 0.818191420948718, 0.754039629071412, 0, 0, 
0.305684531161544, 0.657121716302523, 0.939024390243902, 0.939024390243902, 
0.785808295279265, 0.939024390243902, 0.938192634145033, 0.260041318091513, 
0.701099546376481, 0, 0.0245383773971978, 0, 0.0813612136459603, 
0.0326333292574917, 0, 0.138457654087198, 0, 0.252078839642308, 
0.855198319857617, 0.660669412992965, 0, 0.116265357595049, 0, 
0, 0, 0.796086225131228, 0.400028626820107, 0.637993166386307, 
0, 0, 0.069571994103368, 0.712618210209542, 0, 0.441480055010367, 
0.871048490232094, 0, 0.754989122072317, 0.935057085745997, 0.939024390243902, 
0.93650575311551, 0.939024390243902, 0.282403905882899, 0.0440687974454027, 
0.568892566088456, 0, 0.5198262295164, 0.622227309756103, 0, 
0.308575919793517, 0.316501741963347, 0, 0.39634065149982, 0, 
0, 0, 0.745123115521603, 0, 0.0341160244609436, 0.642054172489147, 
0), G.pval = c(0.766818318950934, 0.556440869391629, 0, 0.811079336085605, 
0.266070513271119, 0.264081229381243, 0, 0, 0.317142183922588, 
0.251672492559076, 0.142048965345827, 0, 0.758614541299287, 0.483402205345852, 
0.416922932942433, 0, 0.235679024964171, 0, 0.111452592494327, 
0.165774638626715, 0.313615795602015, 0, 0.680887142410843, 0, 
0.465612721102427, 0.112011056938783, 0.902173913039219, 0.824689252285377, 
0.786785360028805, 0.902173913039219, 0.0671646717218179, 0.379973922462988, 
0, 0, 0, 0.700054659028977, 0.397145776848281, 0.82033130362237, 
0.169085585384974, 0.302241713015197, 0.338687065961338, 0, 0.532250067218747, 
0.902173913039219, 0.0331894959845903, 0, 0.800412916503477, 
0.772204195706975, 0, 0, 0.134375671585613, 0.708389521449999, 
0.65606481069503, 0, 0.217835296500953, 0.745070473821845, 0, 
0.831015877562972, 0.0910918683458533, 0.424628902964708, 0, 
0.369561915723724, 0.693677249810127, 0.240715770588852, 0, 0.362303864191427, 
0.348227328093822, 0.21528840006919, 0.828748010690442, 0.716295474344133, 
0, 0.600237310320573, 0, 0.795343473078559, 1.16790042192783e-07
), A.pval = c(0, 0, 0.401167578887493, 0, 0.118873246345047, 
0.196818054916119, 0.488326642385079, 0.2626707606995, 0.121793952289646, 
0.291364016583155, 0, 0.367141522506657, 0, 0.122407557337453, 
0.0696684975775401, 0.327258456687722, 0.453265436817829, 0.583082835535492, 
0.396567421061523, 0.909090909074362, 0.909090909074362, 0.881126594174283, 
0.909090909074362, 0.865809850792056, 0.444414577659564, 0, 0.469424705965046, 
0.574738763182633, 0, 0.510340313728753, 0, 0.725192587314358, 
0.78822242902503, 0.909090909074362, 0.909090909074362, 0.208706342751862, 
0, 0.27539297959946, 0.0516135767295629, 0.168840272922471, 0.440496327013107, 
0.618896325461714, 0, 0.337442379434405, 0.00496973446190907, 
0.308294057677904, 0, 0.199407716534997, 0.00682926159088004, 
0.60371807384629, 0.197136025005552, 0, 0, 0.30549325441387, 
0, 0, 0.645927131237774, 0.674205229141338, 0.781572935907054, 
0.200561317303367, 0.209227160971431, 0, 0.263348615166593, 0, 
0.627656225900066, 0.284634592602849, 0.829369767344993, 0.266704966536214, 
0.302281874472509, 0.231246361804894, 0.208706342751862, 0.127373299487117, 
0.0682766437819955, 0, 0.328721641705476), guide.position = 1:75), .Names = c("A.area", 
"C.area", "G.area", "T.area", "Tot.area", "A.perc", "C.perc", 
"G.perc", "T.perc", "base.call", "index", "guide.seq", "T.pval", 
"C.pval", "G.pval", "A.pval", "guide.position"), row.names = 279:353, class = "data.frame")
```

*Report generated using EditR v1.0.8*
