# Supplementary material for: Mechanistic and genetic basis of single-strand templated repair at Cas12a-induced DNA breaks in Chlamydomonas reinhardtii
Source: Nat Commun. 2021 Nov 19;12:6751. doi: 10.1038/s41467-021-27004-1 (PMC8604939; doi:10.1038/s41467-021-27004-1)
Supplement: Supplementary file 22 — Source Data [file 41467_2021_27004_MOESM22_ESM.zip › Source Data/EditR analysis/EditR outputs/Antisense/rep3_ssODN_antisense_-32.html]

EditR v1.0.8 report


# EditR v1.0.8 report

- Data QA
  - Filtering data
  - Percent noise peak area
  - Base information
- Predicted editing
  - Editing bar plot
  - Editing table plot
  - Table of editing results
- For use in R

## Data QA

### Filtering data

What the data looked like prefiltering:

and the post filtering signal / noise plot:

### Percent noise peak area

### Base information

Here’s information about the signal of each base, the critical percent value where any higher value would be called as significant, and Filliben’s correlation for how well the noise was modelled by the zero adjusted gamma distribution.

| Base | Average percent signal | Average peak area | Critical percent value | model mu | Fillibens correlation |
| --- | --- | --- | --- | --- | --- |
| A | 94.48098 | 428.1176 | 7.503169 | 2.485666 | 0.9665952 |
| C | 93.07097 | 445.1045 | 4.689581 | 1.905620 | 0.9955949 |
| G | 94.38553 | 428.7143 | 5.530114 | 1.924862 | 0.9949764 |
| T | 94.86365 | 522.3636 | 5.462447 | 1.950170 | 0.9874063 |

## Predicted editing

### Editing bar plot

### Editing table plot

### Table of editing results


Here’s the entire guide region

| Sanger position | Guide position | Guide sequence | Sanger base call | Focal base | Focal base peak area | p value |  |
| --- | --- | --- | --- | --- | --- | --- | --- |
| 276 | 1 | A | A | A | 96.85 | 0.000000e+00 | \* |
| 276 | 1 | A | A | C | 1.94 | 3.913284e-01 |  |
| 276 | 1 | A | A | G | 0.73 | 8.426536e-01 |  |
| 276 | 1 | A | A | T | 0.48 | 8.639060e-01 |  |
| 277 | 2 | A | A | A | 96.38 | 0.000000e+00 | \* |
| 277 | 2 | A | A | C | 1.06 | 7.590948e-01 |  |
| 277 | 2 | A | A | G | 1.70 | 4.768424e-01 |  |
| 277 | 2 | A | A | T | 0.85 | 7.663794e-01 |  |
| 278 | 3 | G | G | A | 2.68 | 3.424305e-01 |  |
| 278 | 3 | G | G | C | 2.41 | 2.363806e-01 |  |
| 278 | 3 | G | G | G | 94.10 | 0.000000e+00 | \* |
| 278 | 3 | G | G | T | 0.80 | 7.812944e-01 |  |
| 279 | 4 | A | A | A | 95.71 | 0.000000e+00 | \* |
| 279 | 4 | A | A | C | 1.13 | 7.308626e-01 |  |
| 279 | 4 | A | A | G | 1.01 | 7.423120e-01 |  |
| 279 | 4 | A | A | T | 2.14 | 3.248376e-01 |  |
| 280 | 5 | C | C | A | 4.12 | 1.325792e-01 |  |
| 280 | 5 | C | C | C | 91.34 | 0.000000e+00 | \* |
| 280 | 5 | C | C | G | 2.27 | 3.018330e-01 |  |
| 280 | 5 | C | C | T | 2.27 | 2.920795e-01 |  |
| 281 | 6 | T | T | A | 4.27 | 1.198801e-01 |  |
| 281 | 6 | T | T | C | 1.54 | 5.575440e-01 |  |
| 281 | 6 | T | T | G | 2.39 | 2.714809e-01 |  |
| 281 | 6 | T | T | T | 91.81 | 0.000000e+00 | \* |
| 282 | 7 | G | G | A | 1.74 | 5.748363e-01 |  |
| 282 | 7 | G | G | C | 0.00 | 9.285714e-01 |  |
| 282 | 7 | G | G | G | 97.02 | 0.000000e+00 | \* |
| 282 | 7 | G | G | T | 1.24 | 6.279130e-01 |  |
| 283 | 8 | G | G | A | 2.39 | 4.063376e-01 |  |
| 283 | 8 | G | G | C | 1.84 | 4.298567e-01 |  |
| 283 | 8 | G | G | G | 95.04 | 0.000000e+00 | \* |
| 283 | 8 | G | G | T | 0.74 | 8.021937e-01 |  |
| 284 | 9 | C | C | A | 4.86 | 7.782344e-02 |  |
| 284 | 9 | C | C | C | 90.27 | 0.000000e+00 | \* |
| 284 | 9 | C | C | G | 2.13 | 3.401895e-01 |  |
| 284 | 9 | C | C | T | 2.74 | 1.910371e-01 |  |
| 285 | 10 | C | C | A | 2.41 | 4.022357e-01 |  |
| 285 | 10 | C | C | C | 92.96 | 0.000000e+00 | \* |
| 285 | 10 | C | C | G | 2.22 | 3.139774e-01 |  |
| 285 | 10 | C | C | T | 2.41 | 2.583572e-01 |  |
| 286 | 11 | A | A | A | 67.14 | 0.000000e+00 | \* |
| 286 | 11 | A | A | C | 1.90 | 4.037021e-01 |  |
| 286 | 11 | A | A | G | 2.38 | 2.734409e-01 |  |
| 286 | 11 | A | A | T | 28.57 | 4.440892e-16 | \* |
| 287 | 12 | G | G | A | 2.44 | 3.937431e-01 |  |
| 287 | 12 | G | G | C | 1.78 | 4.543392e-01 |  |
| 287 | 12 | G | G | G | 95.56 | 0.000000e+00 | \* |
| 287 | 12 | G | G | T | 0.22 | 8.977355e-01 |  |
| 288 | 13 | A | A | A | 96.68 | 0.000000e+00 | \* |
| 288 | 13 | A | A | C | 0.97 | 7.946463e-01 |  |
| 288 | 13 | A | A | G | 0.83 | 8.079686e-01 |  |
| 288 | 13 | A | A | T | 1.52 | 5.245487e-01 |  |
| 289 | 14 | C | C | A | 3.54 | 1.977443e-01 |  |
| 289 | 14 | C | C | C | 93.96 | 0.000000e+00 | \* |
| 289 | 14 | C | C | G | 2.50 | 2.458254e-01 |  |
| 289 | 14 | C | C | T | 0.00 | 9.032258e-01 |  |
| 290 | 15 | C | C | A | 4.72 | 8.650040e-02 |  |
| 290 | 15 | C | C | C | 92.36 | 0.000000e+00 | \* |
| 290 | 15 | C | C | G | 1.57 | 5.237474e-01 |  |
| 290 | 15 | C | C | T | 1.35 | 5.879440e-01 |  |
| 291 | 16 | G | G | A | 4.35 | 1.131117e-01 |  |
| 291 | 16 | G | G | C | 1.37 | 6.292769e-01 |  |
| 291 | 16 | G | G | G | 92.68 | 0.000000e+00 | \* |
| 291 | 16 | G | G | T | 1.60 | 4.958944e-01 |  |
| 292 | 17 | T | T | A | 1.63 | 6.060279e-01 |  |
| 292 | 17 | T | T | C | 0.81 | 8.447526e-01 |  |
| 292 | 17 | T | T | G | 2.17 | 3.288119e-01 |  |
| 292 | 17 | T | T | T | 95.39 | 0.000000e+00 | \* |
| 293 | 18 | G | G | A | 1.68 | 5.916889e-01 |  |
| 293 | 18 | G | G | C | 0.61 | 8.927566e-01 |  |
| 293 | 18 | G | G | G | 96.95 | 0.000000e+00 | \* |
| 293 | 18 | G | G | T | 0.76 | 7.942127e-01 |  |
| 294 | 19 | T | T | A | 0.00 | 9.272727e-01 |  |
| 294 | 19 | T | T | C | 1.09 | 7.481896e-01 |  |
| 294 | 19 | T | T | G | 3.93 | 5.947687e-02 |  |
| 294 | 19 | T | T | T | 94.98 | 0.000000e+00 | \* |
| 295 | 20 | T | T | A | 0.63 | 8.659456e-01 |  |
| 295 | 20 | T | T | C | 2.11 | 3.289960e-01 |  |
| 295 | 20 | T | T | G | 0.00 | 9.560440e-01 |  |
| 295 | 20 | T | T | T | 97.26 | 0.000000e+00 | \* |
| 296 | 21 | T | T | A | 0.41 | 9.027876e-01 |  |
| 296 | 21 | T | T | C | 0.00 | 9.285714e-01 |  |
| 296 | 21 | T | T | G | 2.04 | 3.668753e-01 |  |
| 296 | 21 | T | T | T | 97.56 | 0.000000e+00 | \* |
| 297 | 22 | G | G | A | 0.50 | 8.898394e-01 |  |
| 297 | 22 | G | G | C | 1.49 | 5.765778e-01 |  |
| 297 | 22 | G | G | G | 97.01 | 0.000000e+00 | \* |
| 297 | 22 | G | G | T | 1.00 | 7.174826e-01 |  |
| 298 | 23 | T | T | A | 0.49 | 8.909833e-01 |  |
| 298 | 23 | T | T | C | 0.74 | 8.656440e-01 |  |
| 298 | 23 | T | T | G | 1.47 | 5.623692e-01 |  |
| 298 | 23 | T | T | T | 97.30 | 0.000000e+00 | \* |
| 299 | 24 | G | G | A | 2.01 | 5.011767e-01 |  |
| 299 | 24 | G | G | C | 1.20 | 7.019392e-01 |  |
| 299 | 24 | G | G | G | 95.38 | 0.000000e+00 | \* |
| 299 | 24 | G | G | T | 1.41 | 5.667696e-01 |  |
| 300 | 25 | C | C | A | 1.68 | 5.911747e-01 |  |
| 300 | 25 | C | C | C | 93.53 | 0.000000e+00 | \* |
| 300 | 25 | C | C | G | 2.16 | 3.315339e-01 |  |
| 300 | 25 | C | C | T | 2.64 | 2.093692e-01 |  |
| 301 | 26 | A | A | A | 90.88 | 0.000000e+00 | \* |
| 301 | 26 | A | A | C | 3.70 | 4.402856e-02 |  |
| 301 | 26 | A | A | G | 4.27 | 4.111173e-02 |  |
| 301 | 26 | A | A | T | 1.14 | 6.653037e-01 |  |
| 302 | 27 | C | C | A | 2.30 | 4.272451e-01 |  |
| 302 | 27 | C | C | C | 95.40 | 0.000000e+00 | \* |
| 302 | 27 | C | C | G | 0.21 | 9.499428e-01 |  |
| 302 | 27 | C | C | T | 2.09 | 3.392147e-01 |  |
| 303 | 28 | T | T | A | 1.66 | 5.954180e-01 |  |
| 303 | 28 | T | T | C | 3.33 | 7.458718e-02 |  |
| 303 | 28 | T | T | G | 0.92 | 7.740665e-01 |  |
| 303 | 28 | T | T | T | 94.09 | 0.000000e+00 | \* |
| 304 | 29 | A | A | A | 95.33 | 0.000000e+00 | \* |
| 304 | 29 | A | A | C | 1.56 | 5.489084e-01 |  |
| 304 | 29 | A | A | G | 1.11 | 7.028074e-01 |  |
| 304 | 29 | A | A | T | 2.00 | 3.658856e-01 |  |
| 305 | 30 | C | C | A | 1.93 | 5.215731e-01 |  |
| 305 | 30 | C | C | C | 94.42 | 0.000000e+00 | \* |
| 305 | 30 | C | C | G | 1.29 | 6.334836e-01 |  |
| 305 | 30 | C | C | T | 2.36 | 2.693512e-01 |  |
| 306 | 31 | A | A | A | 93.04 | 0.000000e+00 | \* |
| 306 | 31 | A | A | C | 2.61 | 1.879925e-01 |  |
| 306 | 31 | A | A | G | 4.35 | 3.791161e-02 |  |
| 306 | 31 | A | A | T | 0.00 | 9.032258e-01 |  |
| 307 | 32 | C | C | A | 1.93 | 5.212948e-01 |  |
| 307 | 32 | C | C | C | 93.48 | 0.000000e+00 | \* |
| 307 | 32 | C | C | G | 1.69 | 4.808617e-01 |  |
| 307 | 32 | C | C | T | 2.90 | 1.634462e-01 |  |
| 308 | 33 | G | G | A | 3.48 | 2.059149e-01 |  |
| 308 | 33 | G | G | C | 2.22 | 2.942906e-01 |  |
| 308 | 33 | G | G | G | 94.30 | 0.000000e+00 | \* |
| 308 | 33 | G | G | T | 0.00 | 9.032258e-01 |  |
| 309 | 34 | G | G | A | 0.00 | 9.272727e-01 |  |
| 309 | 34 | G | G | C | 1.13 | 7.334019e-01 |  |
| 309 | 34 | G | G | G | 97.74 | 0.000000e+00 | \* |
| 309 | 34 | G | G | T | 1.13 | 6.693173e-01 |  |
| 310 | 35 | G | G | A | 2.95 | 2.904364e-01 |  |
| 310 | 35 | G | G | C | 1.34 | 6.435423e-01 |  |
| 310 | 35 | G | G | G | 95.71 | 0.000000e+00 | \* |
| 310 | 35 | G | G | T | 0.00 | 9.032258e-01 |  |
| 311 | 36 | C | C | A | 3.26 | 2.375137e-01 |  |
| 311 | 36 | C | C | C | 93.18 | 0.000000e+00 | \* |
| 311 | 36 | C | C | G | 1.48 | 5.573749e-01 |  |
| 311 | 36 | C | C | T | 2.08 | 3.434384e-01 |  |
| 312 | 37 | A | A | A | 95.16 | 0.000000e+00 | \* |
| 312 | 37 | A | A | C | 2.96 | 1.219080e-01 |  |
| 312 | 37 | A | A | G | 1.88 | 4.155604e-01 |  |
| 312 | 37 | A | A | T | 0.00 | 9.032258e-01 |  |
| 313 | 38 | C | C | A | 2.94 | 2.918728e-01 |  |
| 313 | 38 | C | C | C | 95.29 | 0.000000e+00 | \* |
| 313 | 38 | C | C | G | 0.20 | 9.508751e-01 |  |
| 313 | 38 | C | C | T | 1.57 | 5.076527e-01 |  |
| 314 | 39 | C | C | A | 5.93 | 3.470800e-02 |  |
| 314 | 39 | C | C | C | 90.89 | 0.000000e+00 | \* |
| 314 | 39 | C | C | G | 1.69 | 4.794042e-01 |  |
| 314 | 39 | C | C | T | 1.48 | 5.384367e-01 |  |
| 315 | 40 | C | C | A | 5.32 | 5.531319e-02 |  |
| 315 | 40 | C | C | C | 90.69 | 0.000000e+00 | \* |
| 315 | 40 | C | C | G | 0.67 | 8.616125e-01 |  |
| 315 | 40 | C | C | T | 3.33 | 1.068227e-01 |  |
| 316 | 41 | T | T | A | 2.87 | 3.053080e-01 |  |
| 316 | 41 | T | T | C | 1.23 | 6.914978e-01 |  |
| 316 | 41 | T | T | G | 1.84 | 4.279275e-01 |  |
| 316 | 41 | T | T | T | 94.06 | 0.000000e+00 | \* |
| 317 | 42 | G | G | A | 2.35 | 4.164487e-01 |  |
| 317 | 42 | G | G | C | 1.81 | 4.432137e-01 |  |
| 317 | 42 | G | G | G | 94.58 | 0.000000e+00 | \* |
| 317 | 42 | G | G | T | 1.26 | 6.194255e-01 |  |
| 318 | 43 | A | A | A | 97.07 | 0.000000e+00 | \* |
| 318 | 43 | A | A | C | 1.28 | 6.689694e-01 |  |
| 318 | 43 | A | A | G | 1.65 | 4.961167e-01 |  |
| 318 | 43 | A | A | T | 0.00 | 9.032258e-01 |  |
| 319 | 44 | C | C | A | 3.78 | 1.685374e-01 |  |
| 319 | 44 | C | C | C | 93.78 | 0.000000e+00 | \* |
| 319 | 44 | C | C | G | 0.00 | 9.560440e-01 |  |
| 319 | 44 | C | C | T | 2.44 | 2.499237e-01 |  |
| 320 | 45 | C | C | A | 9.41 | 2.069312e-03 | \* |
| 320 | 45 | C | C | C | 84.47 | 0.000000e+00 | \* |
| 320 | 45 | C | C | G | 4.71 | 2.553035e-02 |  |
| 320 | 45 | C | C | T | 1.41 | 5.645089e-01 |  |
| 321 | 46 | G | G | A | 4.02 | 1.422695e-01 |  |
| 321 | 46 | G | G | C | 2.30 | 2.686472e-01 |  |
| 321 | 46 | G | G | G | 93.39 | 0.000000e+00 | \* |
| 321 | 46 | G | G | T | 0.29 | 8.924765e-01 |  |
| 322 | 47 | A | A | A | 96.84 | 0.000000e+00 | \* |
| 322 | 47 | A | A | C | 1.00 | 7.837772e-01 |  |
| 322 | 47 | A | A | G | 0.83 | 8.072452e-01 |  |
| 322 | 47 | A | A | T | 1.33 | 5.943215e-01 |  |
| 323 | 48 | C | C | A | 4.50 | 1.014108e-01 |  |
| 323 | 48 | C | C | C | 92.75 | 0.000000e+00 | \* |
| 323 | 48 | C | C | G | 0.75 | 8.350117e-01 |  |
| 323 | 48 | C | C | T | 2.00 | 3.658856e-01 |  |
| 324 | 49 | G | G | A | 7.58 | 9.428952e-03 | \* |
| 324 | 49 | G | G | C | 2.27 | 2.764750e-01 |  |
| 324 | 49 | G | G | G | 89.77 | 0.000000e+00 | \* |
| 324 | 49 | G | G | T | 0.38 | 8.815871e-01 |  |
| 325 | 50 | G | G | A | 1.68 | 5.915990e-01 |  |
| 325 | 50 | G | G | C | 0.84 | 8.372326e-01 |  |
| 325 | 50 | G | G | G | 96.86 | 0.000000e+00 | \* |
| 325 | 50 | G | G | T | 0.63 | 8.313740e-01 |  |
| 326 | 51 | C | C | A | 2.22 | 4.464934e-01 |  |
| 326 | 51 | C | C | C | 91.11 | 0.000000e+00 | \* |
| 326 | 51 | C | C | G | 3.70 | 7.549793e-02 |  |
| 326 | 51 | C | C | T | 2.96 | 1.535209e-01 |  |
| 327 | 52 | A | A | A | 96.79 | 0.000000e+00 | \* |
| 327 | 52 | A | A | C | 1.38 | 6.278923e-01 |  |
| 327 | 52 | A | A | G | 1.83 | 4.310663e-01 |  |
| 327 | 52 | A | A | T | 0.00 | 9.032258e-01 |  |
| 328 | 53 | A | A | A | 98.46 | 0.000000e+00 | \* |
| 328 | 53 | A | A | C | 0.26 | 9.267344e-01 |  |
| 328 | 53 | A | A | G | 1.28 | 6.356464e-01 |  |
| 328 | 53 | A | A | T | 0.00 | 9.032258e-01 |  |
| 329 | 54 | G | G | A | 3.15 | 2.549399e-01 |  |
| 329 | 54 | G | G | C | 0.95 | 8.022999e-01 |  |
| 329 | 54 | G | G | G | 94.64 | 0.000000e+00 | \* |
| 329 | 54 | G | G | T | 1.26 | 6.200603e-01 |  |
| 330 | 55 | A | A | A | 96.25 | 0.000000e+00 | \* |
| 330 | 55 | A | A | C | 0.00 | 9.285714e-01 |  |
| 330 | 55 | A | A | G | 3.51 | 9.199073e-02 |  |
| 330 | 55 | A | A | T | 0.23 | 8.969197e-01 |  |
| 331 | 56 | A | A | A | 95.12 | 0.000000e+00 | \* |
| 331 | 56 | A | A | C | 1.54 | 5.546596e-01 |  |
| 331 | 56 | A | A | G | 1.03 | 7.348767e-01 |  |
| 331 | 56 | A | A | T | 2.31 | 2.807013e-01 |  |
| 332 | 57 | G | G | A | 2.67 | 3.443330e-01 |  |
| 332 | 57 | G | G | C | 1.53 | 5.615450e-01 |  |
| 332 | 57 | G | G | G | 93.13 | 0.000000e+00 | \* |
| 332 | 57 | G | G | T | 2.67 | 2.028548e-01 |  |
| 333 | 58 | T | T | A | 1.85 | 5.431031e-01 |  |
| 333 | 58 | T | T | C | 2.91 | 1.294633e-01 |  |
| 333 | 58 | T | T | G | 0.79 | 8.204470e-01 |  |
| 333 | 58 | T | T | T | 94.44 | 0.000000e+00 | \* |
| 334 | 59 | T | T | A | 0.98 | 7.860264e-01 |  |
| 334 | 59 | T | T | C | 1.96 | 3.823690e-01 |  |
| 334 | 59 | T | T | G | 3.68 | 7.767255e-02 |  |
| 334 | 59 | T | T | T | 93.38 | 0.000000e+00 | \* |
| 335 | 60 | C | C | A | 3.18 | 2.503155e-01 |  |
| 335 | 60 | C | C | C | 92.31 | 0.000000e+00 | \* |
| 335 | 60 | C | C | G | 2.92 | 1.663995e-01 |  |
| 335 | 60 | C | C | T | 1.59 | 4.995364e-01 |  |
| 336 | 61 | G | G | A | 4.45 | 1.049725e-01 |  |
| 336 | 61 | G | G | C | 3.08 | 1.035850e-01 |  |
| 336 | 61 | G | G | G | 90.75 | 0.000000e+00 | \* |
| 336 | 61 | G | G | T | 1.71 | 4.576525e-01 |  |
| 337 | 62 | A | A | A | 96.50 | 0.000000e+00 | \* |
| 337 | 62 | A | A | C | 1.17 | 7.185238e-01 |  |
| 337 | 62 | A | A | G | 2.33 | 2.859601e-01 |  |
| 337 | 62 | A | A | T | 0.00 | 9.032258e-01 |  |
| 338 | 63 | C | C | A | 3.20 | 2.470040e-01 |  |
| 338 | 63 | C | C | C | 91.76 | 0.000000e+00 | \* |
| 338 | 63 | C | C | G | 2.29 | 2.965710e-01 |  |
| 338 | 63 | C | C | T | 2.75 | 1.891606e-01 |  |
| 339 | 64 | A | A | A | 94.29 | 0.000000e+00 | \* |
| 339 | 64 | A | A | C | 2.14 | 3.178055e-01 |  |
| 339 | 64 | A | A | G | 2.86 | 1.763543e-01 |  |
| 339 | 64 | A | A | T | 0.71 | 8.082674e-01 |  |
| 340 | 65 | G | G | A | 2.28 | 4.328773e-01 |  |
| 340 | 65 | G | G | C | 2.05 | 3.497524e-01 |  |
| 340 | 65 | G | G | G | 94.08 | 0.000000e+00 | \* |
| 340 | 65 | G | G | T | 1.59 | 4.984689e-01 |  |
| 341 | 66 | C | C | A | 2.91 | 2.981671e-01 |  |
| 341 | 66 | C | C | C | 91.57 | 0.000000e+00 | \* |
| 341 | 66 | C | C | G | 2.91 | 1.681356e-01 |  |
| 341 | 66 | C | C | T | 2.62 | 2.136153e-01 |  |
| 342 | 67 | T | T | A | 1.42 | 6.638958e-01 |  |
| 342 | 67 | T | T | C | 1.42 | 6.073528e-01 |  |
| 342 | 67 | T | T | G | 0.00 | 9.560440e-01 |  |
| 342 | 67 | T | T | T | 97.15 | 0.000000e+00 | \* |
| 343 | 68 | C | C | A | 2.31 | 4.257007e-01 |  |
| 343 | 68 | C | C | C | 92.82 | 0.000000e+00 | \* |
| 343 | 68 | C | C | G | 2.56 | 2.319144e-01 |  |
| 343 | 68 | C | C | T | 2.31 | 2.821628e-01 |  |
| 344 | 69 | C | C | A | 2.21 | 4.498053e-01 |  |
| 344 | 69 | C | C | C | 93.57 | 0.000000e+00 | \* |
| 344 | 69 | C | C | G | 0.40 | 9.255848e-01 |  |
| 344 | 69 | C | C | T | 3.82 | 6.405844e-02 |  |
| 345 | 70 | C | C | A | 3.46 | 2.089338e-01 |  |
| 345 | 70 | C | C | C | 94.65 | 0.000000e+00 | \* |
| 345 | 70 | C | C | G | 1.89 | 4.139033e-01 |  |
| 345 | 70 | C | C | T | 0.00 | 9.032258e-01 |  |
| 346 | 71 | G | G | A | 3.58 | 1.923287e-01 |  |
| 346 | 71 | G | G | C | 1.63 | 5.171575e-01 |  |
| 346 | 71 | G | G | G | 93.16 | 0.000000e+00 | \* |
| 346 | 71 | G | G | T | 1.63 | 4.864770e-01 |  |
| 347 | 72 | C | C | A | 0.00 | 9.272727e-01 |  |
| 347 | 72 | C | C | C | 94.83 | 0.000000e+00 | \* |
| 347 | 72 | C | C | G | 2.74 | 1.978617e-01 |  |
| 347 | 72 | C | C | T | 2.43 | 2.528212e-01 |  |
| 348 | 73 | G | G | A | 6.19 | 2.851317e-02 |  |
| 348 | 73 | G | G | C | 3.44 | 6.417761e-02 |  |
| 348 | 73 | G | G | G | 89.69 | 0.000000e+00 | \* |
| 348 | 73 | G | G | T | 0.69 | 8.158576e-01 |  |
| 349 | 74 | A | A | A | 97.66 | 0.000000e+00 | \* |
| 349 | 74 | A | A | C | 1.40 | 6.165668e-01 |  |
| 349 | 74 | A | A | G | 0.93 | 7.702360e-01 |  |
| 349 | 74 | A | A | T | 0.00 | 9.032258e-01 |  |
| 350 | 75 | C | C | A | 0.00 | 9.272727e-01 |  |
| 350 | 75 | C | C | C | 94.61 | 0.000000e+00 | \* |
| 350 | 75 | C | C | G | 1.41 | 5.875502e-01 |  |
| 350 | 75 | C | C | T | 3.98 | 5.358535e-02 |  |

## For use in R

If you want to work with the results in R, here is output that you can copy and paste in your terminal to get:

The base information:

```
structure(list(focal.base = c("A", "C", "G", "T"), avg.percsignal = c(94.4809849426022, 
93.0709658150642, 94.385529167967, 94.8636505435599), avg.areasignal = c(428.117647058824, 
445.10447761194, 428.714285714286, 522.363636363636), crit.perc.area = c(7.50316901063492, 
4.68958103164133, 5.53011406951047, 5.4624471477622), mu = c(2.48566575386628, 
1.90562022741226, 1.9248623084389, 1.95016963113212), fillibens = c(0.966595150119976, 
0.99559493243552, 0.994976366010089, 0.987406302717505)), .Names = c("focal.base", 
"avg.percsignal", "avg.areasignal", "crit.perc.area", "mu", "fillibens"
), row.names = c(NA, -4L), class = "data.frame")
```

the data.frame that contains information on the guide region:

```
structure(list(A.area = c(400, 453, 10, 759, 20, 25, 14, 13, 
16, 13, 282, 11, 699, 17, 21, 19, 6, 11, 0, 3, 2, 3, 2, 10, 7, 
319, 11, 9, 429, 9, 321, 8, 11, 0, 11, 11, 354, 15, 28, 24, 14, 
13, 530, 17, 40, 14, 582, 18, 20, 8, 6, 211, 384, 10, 411, 370, 
7, 7, 4, 12, 13, 497, 14, 264, 10, 10, 7, 9, 11, 11, 11, 0, 18, 
418, 0), C.area = c(8, 5, 9, 9, 443, 9, 0, 10, 297, 502, 8, 8, 
7, 451, 411, 6, 3, 4, 5, 10, 0, 9, 3, 6, 390, 13, 456, 18, 7, 
440, 9, 387, 7, 5, 5, 314, 11, 486, 429, 409, 6, 10, 7, 422, 
359, 8, 6, 371, 6, 4, 246, 3, 1, 3, 0, 6, 4, 11, 8, 348, 9, 6, 
401, 6, 9, 315, 7, 362, 466, 301, 5, 312, 10, 6, 404), G.area = c(3, 
8, 351, 8, 11, 14, 782, 517, 7, 12, 10, 430, 6, 12, 7, 405, 8, 
636, 18, 0, 10, 585, 6, 475, 9, 15, 1, 5, 5, 6, 15, 7, 298, 433, 
357, 5, 7, 1, 8, 3, 9, 524, 9, 0, 20, 325, 5, 3, 237, 462, 10, 
4, 5, 300, 15, 4, 244, 3, 15, 11, 265, 12, 10, 8, 413, 10, 0, 
10, 2, 6, 286, 9, 261, 4, 6), T.area = c(2, 4, 3, 17, 11, 538, 
10, 4, 9, 13, 120, 1, 11, 0, 6, 7, 352, 5, 435, 461, 479, 6, 
397, 7, 11, 4, 10, 509, 9, 11, 0, 12, 0, 5, 0, 7, 0, 8, 7, 15, 
459, 7, 0, 11, 6, 1, 8, 8, 1, 3, 8, 0, 0, 4, 1, 9, 7, 357, 381, 
6, 5, 0, 12, 2, 7, 9, 478, 9, 19, 0, 5, 8, 2, 0, 17), Tot.area = c(413, 
470, 373, 793, 485, 586, 806, 544, 329, 540, 420, 450, 723, 480, 
445, 437, 369, 656, 458, 474, 491, 603, 408, 498, 417, 351, 478, 
541, 450, 466, 345, 414, 316, 443, 373, 337, 372, 510, 472, 451, 
488, 554, 546, 450, 425, 348, 601, 400, 264, 477, 270, 218, 390, 
317, 427, 389, 262, 378, 408, 377, 292, 515, 437, 280, 439, 344, 
492, 390, 498, 318, 307, 329, 291, 428, 427), A.perc = c(96.8523002421308, 
96.3829787234042, 2.68096514745308, 95.7124842370744, 4.12371134020619, 
4.26621160409556, 1.73697270471464, 2.38970588235294, 4.86322188449848, 
2.40740740740741, 67.1428571428571, 2.44444444444444, 96.6804979253112, 
3.54166666666667, 4.71910112359551, 4.34782608695652, 1.6260162601626, 
1.67682926829268, 0, 0.632911392405063, 0.407331975560081, 0.497512437810945, 
0.490196078431373, 2.00803212851406, 1.67865707434053, 90.8831908831909, 
2.30125523012552, 1.66358595194085, 95.3333333333333, 1.931330472103, 
93.0434782608696, 1.93236714975845, 3.48101265822785, 0, 2.94906166219839, 
3.26409495548961, 95.1612903225806, 2.94117647058824, 5.93220338983051, 
5.32150776053215, 2.86885245901639, 2.34657039711191, 97.0695970695971, 
3.77777777777778, 9.41176470588235, 4.02298850574713, 96.8386023294509, 
4.5, 7.57575757575758, 1.67714884696017, 2.22222222222222, 96.7889908256881, 
98.4615384615385, 3.15457413249211, 96.2529274004684, 95.1156812339332, 
2.67175572519084, 1.85185185185185, 0.980392156862745, 3.18302387267904, 
4.45205479452055, 96.504854368932, 3.20366132723112, 94.2857142857143, 
2.27790432801822, 2.90697674418605, 1.42276422764228, 2.30769230769231, 
2.20883534136546, 3.45911949685535, 3.58306188925081, 0, 6.18556701030928, 
97.6635514018692, 0), C.perc = c(1.93704600484262, 1.06382978723404, 
2.41286863270777, 1.13493064312736, 91.340206185567, 1.5358361774744, 
0, 1.83823529411765, 90.273556231003, 92.962962962963, 1.9047619047619, 
1.77777777777778, 0.968188105117566, 93.9583333333333, 92.3595505617977, 
1.37299771167048, 0.813008130081301, 0.609756097560976, 1.09170305676856, 
2.10970464135021, 0, 1.49253731343284, 0.735294117647059, 1.20481927710843, 
93.5251798561151, 3.7037037037037, 95.397489539749, 3.3271719038817, 
1.55555555555556, 94.4206008583691, 2.60869565217391, 93.4782608695652, 
2.21518987341772, 1.12866817155756, 1.34048257372654, 93.1750741839763, 
2.95698924731183, 95.2941176470588, 90.8898305084746, 90.6873614190687, 
1.22950819672131, 1.80505415162455, 1.28205128205128, 93.7777777777778, 
84.4705882352941, 2.29885057471264, 0.998336106489185, 92.75, 
2.27272727272727, 0.838574423480084, 91.1111111111111, 1.37614678899083, 
0.256410256410256, 0.946372239747634, 0, 1.54241645244216, 1.52671755725191, 
2.91005291005291, 1.96078431372549, 92.3076923076923, 3.08219178082192, 
1.16504854368932, 91.7620137299771, 2.14285714285714, 2.0501138952164, 
91.5697674418605, 1.42276422764228, 92.8205128205128, 93.574297188755, 
94.6540880503145, 1.62866449511401, 94.8328267477204, 3.43642611683849, 
1.4018691588785, 94.6135831381733), G.perc = c(0.726392251815981, 
1.70212765957447, 94.1018766756032, 1.00882723833544, 2.2680412371134, 
2.38907849829352, 97.0223325062035, 95.0367647058823, 2.12765957446809, 
2.22222222222222, 2.38095238095238, 95.5555555555556, 0.829875518672199, 
2.5, 1.57303370786517, 92.6773455377574, 2.1680216802168, 96.9512195121951, 
3.93013100436681, 0, 2.03665987780041, 97.0149253731343, 1.47058823529412, 
95.3815261044177, 2.15827338129496, 4.27350427350427, 0.209205020920502, 
0.924214417744917, 1.11111111111111, 1.28755364806867, 4.34782608695652, 
1.69082125603865, 94.3037974683544, 97.7426636568849, 95.7104557640751, 
1.48367952522255, 1.88172043010753, 0.196078431372549, 1.69491525423729, 
0.665188470066519, 1.84426229508197, 94.5848375451263, 1.64835164835165, 
0, 4.70588235294118, 93.3908045977011, 0.831946755407654, 0.75, 
89.7727272727273, 96.8553459119497, 3.7037037037037, 1.8348623853211, 
1.28205128205128, 94.6372239747634, 3.51288056206089, 1.02827763496144, 
93.1297709923664, 0.793650793650794, 3.67647058823529, 2.91777188328912, 
90.7534246575343, 2.33009708737864, 2.2883295194508, 2.85714285714286, 
94.0774487471526, 2.90697674418605, 0, 2.56410256410256, 0.401606425702811, 
1.88679245283019, 93.1596091205212, 2.73556231003039, 89.6907216494845, 
0.934579439252336, 1.40515222482436), T.perc = c(0.484261501210654, 
0.851063829787234, 0.804289544235925, 2.1437578814628, 2.2680412371134, 
91.8088737201365, 1.24069478908189, 0.735294117647059, 2.73556231003039, 
2.40740740740741, 28.5714285714286, 0.222222222222222, 1.52143845089903, 
0, 1.34831460674157, 1.60183066361556, 95.3929539295393, 0.76219512195122, 
94.9781659388646, 97.2573839662447, 97.5560081466395, 0.995024875621891, 
97.3039215686274, 1.40562248995984, 2.6378896882494, 1.13960113960114, 
2.09205020920502, 94.0850277264325, 2, 2.36051502145923, 0, 2.89855072463768, 
0, 1.12866817155756, 0, 2.07715133531157, 0, 1.56862745098039, 
1.48305084745763, 3.32594235033259, 94.0573770491803, 1.26353790613718, 
0, 2.44444444444444, 1.41176470588235, 0.28735632183908, 1.33111480865225, 
2, 0.378787878787879, 0.628930817610063, 2.96296296296296, 0, 
0, 1.26182965299685, 0.234192037470726, 2.31362467866324, 2.67175572519084, 
94.4444444444444, 93.3823529411765, 1.59151193633952, 1.71232876712329, 
0, 2.74599542334096, 0.714285714285714, 1.59453302961276, 2.61627906976744, 
97.1544715447154, 2.30769230769231, 3.81526104417671, 0, 1.62866449511401, 
2.43161094224924, 0.687285223367698, 0, 3.98126463700234), base.call = c("A", 
"A", "G", "A", "C", "T", "G", "G", "C", "C", "A", "G", "A", "C", 
"C", "G", "T", "G", "T", "T", "T", "G", "T", "G", "C", "A", "C", 
"T", "A", "C", "A", "C", "G", "G", "G", "C", "A", "C", "C", "C", 
"T", "G", "A", "C", "C", "G", "A", "C", "G", "G", "C", "A", "A", 
"G", "A", "A", "G", "T", "T", "C", "G", "A", "C", "A", "G", "C", 
"T", "C", "C", "C", "G", "C", "G", "A", "C"), index = 276:350, 
    guide.seq = c("A", "A", "G", "A", "C", "T", "G", "G", "C", 
    "C", "A", "G", "A", "C", "C", "G", "T", "G", "T", "T", "T", 
    "G", "T", "G", "C", "A", "C", "T", "A", "C", "A", "C", "G", 
    "G", "G", "C", "A", "C", "C", "C", "T", "G", "A", "C", "C", 
    "G", "A", "C", "G", "G", "C", "A", "A", "G", "A", "A", "G", 
    "T", "T", "C", "G", "A", "C", "A", "G", "C", "T", "C", "C", 
    "C", "G", "C", "G", "A", "C"), T.pval = c(0.863905950556384, 
    0.766379423246694, 0.781294397087026, 0.324837642804146, 
    0.292079466356495, 0, 0.627912999054817, 0.802193716046403, 
    0.191037149430006, 0.258357231796061, 4.44089209850063e-16, 
    0.897735458734405, 0.524548749532354, 0.903225806446376, 
    0.587944031276715, 0.495894350591462, 0, 0.794212678990008, 
    0, 0, 0, 0.71748260753745, 0, 0.566769611572961, 0.209369176013608, 
    0.665303731955444, 0.339214697797448, 0, 0.365885614123751, 
    0.269351181298725, 0.903225806446376, 0.163446190185139, 
    0.903225806446376, 0.669317275825572, 0.903225806446376, 
    0.343438404918661, 0.903225806446376, 0.507652672592326, 
    0.538436726681844, 0.10682267843644, 0, 0.619425476626896, 
    0.903225806446376, 0.249923743869026, 0.564508878093013, 
    0.892476471956978, 0.594321504892898, 0.365885614123751, 
    0.881587125727928, 0.831373953299972, 0.153520917213137, 
    0.903225806446376, 0.903225806446376, 0.620060342445371, 
    0.896919718991835, 0.280701293747136, 0.202854834258462, 
    0, 0, 0.499536357451465, 0.457652493759335, 0.903225806446376, 
    0.189160573421046, 0.808267399587913, 0.498468899117903, 
    0.213615319312366, 0, 0.282162820732607, 0.0640584408748652, 
    0.903225806446376, 0.486477049390383, 0.252821155021375, 
    0.815857602534324, 0.903225806446376, 0.0535853519318827), 
    C.pval = c(0.391328431124047, 0.759094767804002, 0.23638064488534, 
    0.730862581753154, 0, 0.557543959185498, 0.928571428571429, 
    0.429856666713072, 0, 0, 0.403702100972138, 0.454339159674702, 
    0.794646309287624, 0, 0, 0.629276944394578, 0.844752604162067, 
    0.892756616154865, 0.748189579605209, 0.328995958583866, 
    0.928571428571429, 0.576577760233847, 0.865643972839184, 
    0.70193918266475, 0, 0.044028564307507, 0, 0.0745871843386634, 
    0.54890840864103, 0, 0.187992544368106, 0, 0.294290561241003, 
    0.733401895074618, 0.643542316771232, 0, 0.121908003306026, 
    0, 0, 0, 0.691497848588743, 0.443213654665025, 0.668969377701855, 
    0, 0, 0.26864721863719, 0.783777238070107, 0, 0.276474988183777, 
    0.837232648500611, 0, 0.627892253892822, 0.92673444670105, 
    0.802299928783026, 0.928571428571429, 0.554659607938479, 
    0.561545037148836, 0.129463335630508, 0.382368997900062, 
    0, 0.103584971535454, 0.718523833609066, 0, 0.317805516537719, 
    0.349752400705131, 0, 0.607352818544987, 0, 0, 0, 0.517157496127363, 
    0, 0.0641776086624083, 0.616566802595691, 0), G.pval = c(0.842653592597255, 
    0.476842390714131, 0, 0.742311951568893, 0.3018330458233, 
    0.271480888461796, 0, 0, 0.340189451771141, 0.313977441024393, 
    0.273440863799315, 0, 0.807968637659444, 0.245825386233186, 
    0.52374741461354, 0, 0.328811868436067, 0, 0.0594768734535746, 
    0.956043956043634, 0.366875344523818, 0, 0.562369171229227, 
    0, 0.331533928923951, 0.0411117337274441, 0.949942821108327, 
    0.77406654090108, 0.702807392446613, 0.633483582523955, 0.0379116131205484, 
    0.480861677141413, 0, 0, 0, 0.557374943010815, 0.415560369291523, 
    0.950875067631948, 0.479404235670281, 0.861612532126317, 
    0.427927482371252, 0, 0.496116655768963, 0.956043956043634, 
    0.0255303489476816, 0, 0.807245228412434, 0.835011746382468, 
    0, 0, 0.0754979328767209, 0.431066302875963, 0.635646380497593, 
    0, 0.0919907282185358, 0.734876698112021, 0, 0.820446988305274, 
    0.0776725540666436, 0.16639945649108, 0, 0.285960070894356, 
    0.296571046549506, 0.176354263976552, 0, 0.168135592153961, 
    0.956043956043634, 0.231914353606782, 0.925584842845427, 
    0.413903285411054, 0, 0.197861692577419, 0, 0.770235993674063, 
    0.587550182899387), A.pval = c(0, 0, 0.342430509404017, 0, 
    0.132579175879841, 0.119880063339435, 0.574836291471527, 
    0.4063376259461, 0.0778234381176761, 0.402235688906875, 0, 
    0.393743134583697, 0, 0.197744265563951, 0.0865004019248095, 
    0.113111652605389, 0.606027887137986, 0.591688879766666, 
    0.927272727272727, 0.865945629880498, 0.902787578974393, 
    0.889839397113776, 0.890983273251757, 0.501176672139584, 
    0.591174680917016, 0, 0.427245136985048, 0.595417973808572, 
    0, 0.521573071529611, 0, 0.521294810444485, 0.205914859667056, 
    0.927272727272727, 0.290436436649279, 0.23751372669353, 0, 
    0.291872788749081, 0.0347080018028453, 0.0553131851119174, 
    0.305307978729199, 0.416448746478286, 0, 0.168537410397127, 
    0.00206931164508595, 0.142269527415186, 0, 0.10141084583254, 
    0.00942895169902702, 0.591598967259371, 0.446493370596063, 
    0, 0, 0.254939947252143, 0, 0, 0.344333032405111, 0.543103078646084, 
    0.786026406893233, 0.250315464809409, 0.104972536910966, 
    0, 0.247004028727498, 0, 0.432877333363993, 0.298167132710078, 
    0.663895766876035, 0.425700713489417, 0.449805316836814, 
    0.208933834418896, 0.192328650924965, 0.927272727272727, 
    0.0285131695618074, 0, 0.927272727272727), guide.position = 1:75), .Names = c("A.area", 
"C.area", "G.area", "T.area", "Tot.area", "A.perc", "C.perc", 
"G.perc", "T.perc", "base.call", "index", "guide.seq", "T.pval", 
"C.pval", "G.pval", "A.pval", "guide.position"), row.names = 276:350, class = "data.frame")
```

*Report generated using EditR v1.0.8*
