# Supplementary material for: Mechanistic and genetic basis of single-strand templated repair at Cas12a-induced DNA breaks in Chlamydomonas reinhardtii
Source: Nat Commun. 2021 Nov 19;12:6751. doi: 10.1038/s41467-021-27004-1 (PMC8604939; doi:10.1038/s41467-021-27004-1)
Supplement: Supplementary file 22 — Source Data [file 41467_2021_27004_MOESM22_ESM.zip › Source Data/EditR analysis/EditR outputs/Antisense/rep3_ssODN_antisense_-32_-16_0.html]

EditR v1.0.8 report


# EditR v1.0.8 report

- Data QA
  - Filtering data
  - Percent noise peak area
  - Base information
- Predicted editing
  - Editing bar plot
  - Editing table plot
  - Table of editing results
- For use in R

## Data QA

### Filtering data

What the data looked like prefiltering:

and the post filtering signal / noise plot:

### Percent noise peak area

### Base information

Here’s information about the signal of each base, the critical percent value where any higher value would be called as significant, and Filliben’s correlation for how well the noise was modelled by the zero adjusted gamma distribution.

| Base | Average percent signal | Average peak area | Critical percent value | model mu | Fillibens correlation |
| --- | --- | --- | --- | --- | --- |
| A | 92.59141 | 330.9412 | 10.365798 | 3.248485 | 0.9951040 |
| C | 93.95543 | 343.1818 | 4.222026 | 1.786873 | 0.9898112 |
| G | 93.07661 | 323.8070 | 4.449079 | 1.572860 | 0.9925330 |
| T | 95.11455 | 389.8182 | 6.682825 | 2.219043 | 0.9937079 |

## Predicted editing

### Editing bar plot

### Editing table plot

### Table of editing results


Here’s the entire guide region

| Sanger position | Guide position | Guide sequence | Sanger base call | Focal base | Focal base peak area | p value |  |
| --- | --- | --- | --- | --- | --- | --- | --- |
| 275 | 1 | A | A | A | 95.53 | 0.000000e+00 | \* |
| 275 | 1 | A | A | C | 1.92 | 3.479231e-01 |  |
| 275 | 1 | A | A | G | 0.96 | 6.497204e-01 |  |
| 275 | 1 | A | A | T | 1.60 | 5.522270e-01 |  |
| 276 | 2 | A | A | A | 93.25 | 0.000000e+00 | \* |
| 276 | 2 | A | A | C | 0.50 | 9.007438e-01 |  |
| 276 | 2 | A | A | G | 1.25 | 5.166278e-01 |  |
| 276 | 2 | A | A | T | 5.00 | 4.412875e-02 |  |
| 277 | 3 | G | G | A | 3.16 | 3.572613e-01 |  |
| 277 | 3 | G | G | C | 1.75 | 4.160143e-01 |  |
| 277 | 3 | G | G | G | 95.09 | 0.000000e+00 | \* |
| 277 | 3 | G | G | T | 0.00 | 9.139785e-01 |  |
| 278 | 4 | A | A | A | 93.97 | 0.000000e+00 | \* |
| 278 | 4 | A | A | C | 1.11 | 7.185250e-01 |  |
| 278 | 4 | A | A | G | 0.95 | 6.525218e-01 |  |
| 278 | 4 | A | A | T | 3.97 | 1.040003e-01 |  |
| 279 | 5 | C | C | A | 4.92 | 1.638106e-01 |  |
| 279 | 5 | C | C | C | 92.90 | 0.000000e+00 | \* |
| 279 | 5 | C | C | G | 0.00 | 9.111111e-01 |  |
| 279 | 5 | C | C | T | 2.19 | 3.872004e-01 |  |
| 280 | 6 | T | T | A | 3.86 | 2.646521e-01 |  |
| 280 | 6 | T | T | C | 2.05 | 2.991816e-01 |  |
| 280 | 6 | T | T | G | 2.73 | 1.033475e-01 |  |
| 280 | 6 | T | T | T | 91.36 | 0.000000e+00 | \* |
| 281 | 7 | G | G | A | 4.29 | 2.192746e-01 |  |
| 281 | 7 | G | G | C | 0.00 | 9.176471e-01 |  |
| 281 | 7 | G | G | G | 94.44 | 0.000000e+00 | \* |
| 281 | 7 | G | G | T | 1.27 | 6.544524e-01 |  |
| 282 | 8 | G | G | A | 4.80 | 1.734244e-01 |  |
| 282 | 8 | G | G | C | 1.44 | 5.633338e-01 |  |
| 282 | 8 | G | G | G | 93.29 | 0.000000e+00 | \* |
| 282 | 8 | G | G | T | 0.48 | 8.706746e-01 |  |
| 283 | 9 | C | C | A | 4.10 | 2.385372e-01 |  |
| 283 | 9 | C | C | C | 91.39 | 0.000000e+00 | \* |
| 283 | 9 | C | C | G | 2.87 | 8.641249e-02 |  |
| 283 | 9 | C | C | T | 1.64 | 5.394901e-01 |  |
| 284 | 10 | C | C | A | 2.18 | 5.204155e-01 |  |
| 284 | 10 | C | C | C | 93.22 | 0.000000e+00 | \* |
| 284 | 10 | C | C | G | 2.42 | 1.504559e-01 |  |
| 284 | 10 | C | C | T | 2.18 | 3.888629e-01 |  |
| 285 | 11 | A | A | A | 77.07 | 0.000000e+00 | \* |
| 285 | 11 | A | A | C | 1.91 | 3.503548e-01 |  |
| 285 | 11 | A | A | G | 1.91 | 2.700968e-01 |  |
| 285 | 11 | A | A | T | 19.11 | 5.041002e-08 | \* |
| 286 | 12 | G | G | A | 2.80 | 4.133123e-01 |  |
| 286 | 12 | G | G | C | 0.93 | 7.924583e-01 |  |
| 286 | 12 | G | G | G | 95.65 | 0.000000e+00 | \* |
| 286 | 12 | G | G | T | 0.62 | 8.410055e-01 |  |
| 287 | 13 | A | A | A | 93.66 | 0.000000e+00 | \* |
| 287 | 13 | A | A | C | 1.20 | 6.786300e-01 |  |
| 287 | 13 | A | A | G | 0.86 | 6.963999e-01 |  |
| 287 | 13 | A | A | T | 4.28 | 8.065214e-02 |  |
| 288 | 14 | C | C | A | 4.97 | 1.595697e-01 |  |
| 288 | 14 | C | C | C | 90.58 | 0.000000e+00 | \* |
| 288 | 14 | C | C | G | 2.09 | 2.203833e-01 |  |
| 288 | 14 | C | C | T | 2.36 | 3.461040e-01 |  |
| 289 | 15 | C | C | A | 3.68 | 2.865574e-01 |  |
| 289 | 15 | C | C | C | 93.25 | 0.000000e+00 | \* |
| 289 | 15 | C | C | G | 2.15 | 2.074895e-01 |  |
| 289 | 15 | C | C | T | 0.92 | 7.617451e-01 |  |
| 290 | 16 | G | G | A | 5.39 | 1.309602e-01 |  |
| 290 | 16 | G | G | C | 1.50 | 5.352418e-01 |  |
| 290 | 16 | G | G | G | 91.92 | 0.000000e+00 | \* |
| 290 | 16 | G | G | T | 1.20 | 6.771599e-01 |  |
| 291 | 17 | T | T | A | 0.00 | 8.454545e-01 |  |
| 291 | 17 | T | T | C | 1.13 | 7.091469e-01 |  |
| 291 | 17 | T | T | G | 3.02 | 7.125841e-02 |  |
| 291 | 17 | T | T | T | 95.85 | 0.000000e+00 | \* |
| 292 | 18 | G | G | A | 4.06 | 2.424474e-01 |  |
| 292 | 18 | G | G | C | 0.00 | 9.176471e-01 |  |
| 292 | 18 | G | G | G | 94.58 | 0.000000e+00 | \* |
| 292 | 18 | G | G | T | 1.35 | 6.279535e-01 |  |
| 293 | 19 | T | T | A | 0.00 | 8.454545e-01 |  |
| 293 | 19 | T | T | C | 0.88 | 8.103080e-01 |  |
| 293 | 19 | T | T | G | 3.53 | 3.618609e-02 |  |
| 293 | 19 | T | T | T | 95.59 | 0.000000e+00 | \* |
| 294 | 20 | T | T | A | 0.55 | 8.083686e-01 |  |
| 294 | 20 | T | T | C | 0.00 | 9.176471e-01 |  |
| 294 | 20 | T | T | G | 2.21 | 1.930471e-01 |  |
| 294 | 20 | T | T | T | 97.24 | 0.000000e+00 | \* |
| 295 | 21 | T | T | A | 0.00 | 8.454545e-01 |  |
| 295 | 21 | T | T | C | 1.63 | 4.741097e-01 |  |
| 295 | 21 | T | T | G | 1.90 | 2.741698e-01 |  |
| 295 | 21 | T | T | T | 96.48 | 0.000000e+00 | \* |
| 296 | 22 | G | G | A | 3.47 | 3.135425e-01 |  |
| 296 | 22 | G | G | C | 0.00 | 9.176471e-01 |  |
| 296 | 22 | G | G | G | 95.23 | 0.000000e+00 | \* |
| 296 | 22 | G | G | T | 1.30 | 6.444731e-01 |  |
| 297 | 23 | T | T | A | 0.33 | 8.313774e-01 |  |
| 297 | 23 | T | T | C | 1.64 | 4.654391e-01 |  |
| 297 | 23 | T | T | G | 2.30 | 1.732512e-01 |  |
| 297 | 23 | T | T | T | 95.72 | 0.000000e+00 | \* |
| 298 | 24 | G | G | A | 4.42 | 2.067137e-01 |  |
| 298 | 24 | G | G | C | 1.56 | 5.058678e-01 |  |
| 298 | 24 | G | G | G | 91.95 | 0.000000e+00 | \* |
| 298 | 24 | G | G | T | 2.08 | 4.149059e-01 |  |
| 299 | 25 | C | C | A | 1.28 | 6.906500e-01 |  |
| 299 | 25 | C | C | C | 96.47 | 0.000000e+00 | \* |
| 299 | 25 | C | C | G | 2.24 | 1.856513e-01 |  |
| 299 | 25 | C | C | T | 0.00 | 9.139785e-01 |  |
| 300 | 26 | A | A | A | 89.53 | 0.000000e+00 | \* |
| 300 | 26 | A | A | C | 3.25 | 5.327675e-02 |  |
| 300 | 26 | A | A | G | 2.53 | 1.323812e-01 |  |
| 300 | 26 | A | A | T | 4.69 | 5.723872e-02 |  |
| 301 | 27 | C | C | A | 2.61 | 4.442380e-01 |  |
| 301 | 27 | C | C | C | 87.54 | 0.000000e+00 | \* |
| 301 | 27 | C | C | G | 7.83 | 5.908751e-05 | \* |
| 301 | 27 | C | C | T | 2.03 | 4.278879e-01 |  |
| 302 | 28 | T | T | A | 1.60 | 6.310975e-01 |  |
| 302 | 28 | T | T | C | 3.46 | 3.784623e-02 |  |
| 302 | 28 | T | T | G | 0.00 | 9.111111e-01 |  |
| 302 | 28 | T | T | T | 94.95 | 0.000000e+00 | \* |
| 303 | 29 | A | A | A | 94.88 | 0.000000e+00 | \* |
| 303 | 29 | A | A | C | 1.81 | 3.931434e-01 |  |
| 303 | 29 | A | A | G | 1.20 | 5.367838e-01 |  |
| 303 | 29 | A | A | T | 2.11 | 4.069410e-01 |  |
| 304 | 30 | C | C | A | 2.04 | 5.461536e-01 |  |
| 304 | 30 | C | C | C | 93.88 | 0.000000e+00 | \* |
| 304 | 30 | C | C | G | 2.04 | 2.340361e-01 |  |
| 304 | 30 | C | C | T | 2.04 | 4.247262e-01 |  |
| 305 | 31 | A | A | A | 90.74 | 0.000000e+00 | \* |
| 305 | 31 | A | A | C | 2.22 | 2.400493e-01 |  |
| 305 | 31 | A | A | G | 4.07 | 1.704132e-02 |  |
| 305 | 31 | A | A | T | 2.96 | 2.258802e-01 |  |
| 306 | 32 | C | C | A | 2.25 | 5.072922e-01 |  |
| 306 | 32 | C | C | C | 94.21 | 0.000000e+00 | \* |
| 306 | 32 | C | C | G | 1.93 | 2.647334e-01 |  |
| 306 | 32 | C | C | T | 1.61 | 5.490938e-01 |  |
| 307 | 33 | G | G | A | 3.73 | 2.800000e-01 |  |
| 307 | 33 | G | G | C | 2.49 | 1.678223e-01 |  |
| 307 | 33 | G | G | G | 92.95 | 0.000000e+00 | \* |
| 307 | 33 | G | G | T | 0.83 | 7.875080e-01 |  |
| 308 | 34 | G | G | A | 3.20 | 3.514635e-01 |  |
| 308 | 34 | G | G | C | 0.29 | 9.155016e-01 |  |
| 308 | 34 | G | G | G | 95.93 | 0.000000e+00 | \* |
| 308 | 34 | G | G | T | 0.58 | 8.499585e-01 |  |
| 309 | 35 | G | G | A | 2.80 | 4.129602e-01 |  |
| 309 | 35 | G | G | C | 1.40 | 5.828539e-01 |  |
| 309 | 35 | G | G | G | 94.41 | 0.000000e+00 | \* |
| 309 | 35 | G | G | T | 1.40 | 6.139202e-01 |  |
| 310 | 36 | C | C | A | 1.57 | 6.363030e-01 |  |
| 310 | 36 | C | C | C | 95.69 | 0.000000e+00 | \* |
| 310 | 36 | C | C | G | 2.75 | 1.010620e-01 |  |
| 310 | 36 | C | C | T | 0.00 | 9.139785e-01 |  |
| 311 | 37 | A | A | A | 90.81 | 0.000000e+00 | \* |
| 311 | 37 | A | A | C | 2.83 | 1.030026e-01 |  |
| 311 | 37 | A | A | G | 2.47 | 1.412842e-01 |  |
| 311 | 37 | A | A | T | 3.89 | 1.110139e-01 |  |
| 312 | 38 | C | C | A | 2.28 | 5.012139e-01 |  |
| 312 | 38 | C | C | C | 94.67 | 0.000000e+00 | \* |
| 312 | 38 | C | C | G | 1.27 | 5.082240e-01 |  |
| 312 | 38 | C | C | T | 1.78 | 4.986369e-01 |  |
| 313 | 39 | C | C | A | 1.16 | 7.125649e-01 |  |
| 313 | 39 | C | C | C | 96.51 | 0.000000e+00 | \* |
| 313 | 39 | C | C | G | 1.45 | 4.301457e-01 |  |
| 313 | 39 | C | C | T | 0.87 | 7.756282e-01 |  |
| 314 | 40 | C | C | A | 1.17 | 7.113321e-01 |  |
| 314 | 40 | C | C | C | 94.44 | 0.000000e+00 | \* |
| 314 | 40 | C | C | G | 2.34 | 1.659342e-01 |  |
| 314 | 40 | C | C | T | 2.05 | 4.231371e-01 |  |
| 315 | 41 | T | T | A | 0.00 | 8.454545e-01 |  |
| 315 | 41 | T | T | C | 1.40 | 5.838018e-01 |  |
| 315 | 41 | T | T | G | 1.96 | 2.572966e-01 |  |
| 315 | 41 | T | T | T | 96.65 | 0.000000e+00 | \* |
| 316 | 42 | G | G | A | 3.53 | 3.058168e-01 |  |
| 316 | 42 | G | G | C | 0.94 | 7.888826e-01 |  |
| 316 | 42 | G | G | G | 93.88 | 0.000000e+00 | \* |
| 316 | 42 | G | G | T | 1.65 | 5.371576e-01 |  |
| 317 | 43 | A | A | A | 86.30 | 0.000000e+00 | \* |
| 317 | 43 | A | A | C | 1.29 | 6.344024e-01 |  |
| 317 | 43 | A | A | G | 1.81 | 3.013133e-01 |  |
| 317 | 43 | A | A | T | 10.59 | 2.512048e-04 | \* |
| 318 | 44 | C | C | A | 3.36 | 3.279860e-01 |  |
| 318 | 44 | C | C | C | 92.97 | 0.000000e+00 | \* |
| 318 | 44 | C | C | G | 3.67 | 2.988509e-02 |  |
| 318 | 44 | C | C | T | 0.00 | 9.139785e-01 |  |
| 319 | 45 | C | C | A | 5.88 | 1.030868e-01 |  |
| 319 | 45 | C | C | C | 90.52 | 0.000000e+00 | \* |
| 319 | 45 | C | C | G | 2.29 | 1.763432e-01 |  |
| 319 | 45 | C | C | T | 1.31 | 6.426863e-01 |  |
| 320 | 46 | G | G | A | 5.73 | 1.113153e-01 |  |
| 320 | 46 | G | G | C | 1.91 | 3.513260e-01 |  |
| 320 | 46 | G | G | G | 92.37 | 0.000000e+00 | \* |
| 320 | 46 | G | G | T | 0.00 | 9.139785e-01 |  |
| 321 | 47 | A | A | A | 94.68 | 0.000000e+00 | \* |
| 321 | 47 | A | A | C | 0.82 | 8.316638e-01 |  |
| 321 | 47 | A | A | G | 1.23 | 5.268561e-01 |  |
| 321 | 47 | A | A | T | 3.27 | 1.793689e-01 |  |
| 322 | 48 | C | C | A | 3.78 | 2.744992e-01 |  |
| 322 | 48 | C | C | C | 94.85 | 0.000000e+00 | \* |
| 322 | 48 | C | C | G | 1.37 | 4.627452e-01 |  |
| 322 | 48 | C | C | T | 0.00 | 9.139785e-01 |  |
| 323 | 49 | G | G | A | 5.82 | 1.062765e-01 |  |
| 323 | 49 | G | G | C | 2.12 | 2.743363e-01 |  |
| 323 | 49 | G | G | G | 92.06 | 0.000000e+00 | \* |
| 323 | 49 | G | G | T | 0.00 | 9.139785e-01 |  |
| 324 | 50 | G | G | A | 4.36 | 2.120436e-01 |  |
| 324 | 50 | G | G | C | 0.82 | 8.318386e-01 |  |
| 324 | 50 | G | G | G | 94.82 | 0.000000e+00 | \* |
| 324 | 50 | G | G | T | 0.00 | 9.139785e-01 |  |
| 325 | 51 | C | C | A | 1.90 | 5.718723e-01 |  |
| 325 | 51 | C | C | C | 91.43 | 0.000000e+00 | \* |
| 325 | 51 | C | C | G | 2.86 | 8.771107e-02 |  |
| 325 | 51 | C | C | T | 3.81 | 1.180848e-01 |  |
| 326 | 52 | A | A | A | 91.81 | 0.000000e+00 | \* |
| 326 | 52 | A | A | C | 1.75 | 4.160143e-01 |  |
| 326 | 52 | A | A | G | 1.75 | 3.190199e-01 |  |
| 326 | 52 | A | A | T | 4.68 | 5.795442e-02 |  |
| 327 | 53 | A | A | A | 95.75 | 0.000000e+00 | \* |
| 327 | 53 | A | A | C | 0.00 | 9.176471e-01 |  |
| 327 | 53 | A | A | G | 0.98 | 6.396133e-01 |  |
| 327 | 53 | A | A | T | 3.27 | 1.799152e-01 |  |
| 328 | 54 | G | G | A | 4.15 | 2.331555e-01 |  |
| 328 | 54 | G | G | C | 2.07 | 2.887638e-01 |  |
| 328 | 54 | G | G | G | 93.78 | 0.000000e+00 | \* |
| 328 | 54 | G | G | T | 0.00 | 9.139785e-01 |  |
| 329 | 55 | A | A | A | 93.62 | 0.000000e+00 | \* |
| 329 | 55 | A | A | C | 0.00 | 9.176471e-01 |  |
| 329 | 55 | A | A | G | 2.61 | 1.197681e-01 |  |
| 329 | 55 | A | A | T | 3.77 | 1.220324e-01 |  |
| 330 | 56 | A | A | A | 95.71 | 0.000000e+00 | \* |
| 330 | 56 | A | A | C | 0.00 | 9.176471e-01 |  |
| 330 | 56 | A | A | G | 1.32 | 4.859564e-01 |  |
| 330 | 56 | A | A | T | 2.97 | 2.246686e-01 |  |
| 331 | 57 | G | G | A | 2.99 | 3.832510e-01 |  |
| 331 | 57 | G | G | C | 0.00 | 9.176471e-01 |  |
| 331 | 57 | G | G | G | 93.53 | 0.000000e+00 | \* |
| 331 | 57 | G | G | T | 3.48 | 1.526329e-01 |  |
| 332 | 58 | T | T | A | 0.74 | 7.836836e-01 |  |
| 332 | 58 | T | T | C | 2.57 | 1.491622e-01 |  |
| 332 | 58 | T | T | G | 0.74 | 7.494014e-01 |  |
| 332 | 58 | T | T | T | 95.96 | 0.000000e+00 | \* |
| 333 | 59 | T | T | A | 0.00 | 8.454545e-01 |  |
| 333 | 59 | T | T | C | 1.69 | 4.425156e-01 |  |
| 333 | 59 | T | T | G | 3.05 | 6.836139e-02 |  |
| 333 | 59 | T | T | T | 95.25 | 0.000000e+00 | \* |
| 334 | 60 | C | C | A | 2.44 | 4.735706e-01 |  |
| 334 | 60 | C | C | C | 93.03 | 0.000000e+00 | \* |
| 334 | 60 | C | C | G | 2.09 | 2.212945e-01 |  |
| 334 | 60 | C | C | T | 2.44 | 3.272508e-01 |  |
| 335 | 61 | G | G | A | 7.11 | 5.573558e-02 |  |
| 335 | 61 | G | G | C | 3.11 | 6.643368e-02 |  |
| 335 | 61 | G | G | G | 89.33 | 0.000000e+00 | \* |
| 335 | 61 | G | G | T | 0.44 | 8.770172e-01 |  |
| 336 | 62 | A | A | A | 92.87 | 0.000000e+00 | \* |
| 336 | 62 | A | A | C | 1.23 | 6.646267e-01 |  |
| 336 | 62 | A | A | G | 1.97 | 2.544029e-01 |  |
| 336 | 62 | A | A | T | 3.93 | 1.071438e-01 |  |
| 337 | 63 | C | C | A | 3.68 | 2.865574e-01 |  |
| 337 | 63 | C | C | C | 94.48 | 0.000000e+00 | \* |
| 337 | 63 | C | C | G | 0.00 | 9.111111e-01 |  |
| 337 | 63 | C | C | T | 1.84 | 4.801685e-01 |  |
| 338 | 64 | A | A | A | 89.33 | 0.000000e+00 | \* |
| 338 | 64 | A | A | C | 2.22 | 2.400493e-01 |  |
| 338 | 64 | A | A | G | 3.11 | 6.319522e-02 |  |
| 338 | 64 | A | A | T | 5.33 | 3.312652e-02 |  |
| 339 | 65 | G | G | A | 3.65 | 2.907370e-01 |  |
| 339 | 65 | G | G | C | 1.52 | 5.243190e-01 |  |
| 339 | 65 | G | G | G | 93.62 | 0.000000e+00 | \* |
| 339 | 65 | G | G | T | 1.22 | 6.714490e-01 |  |
| 340 | 66 | C | C | A | 2.32 | 4.953724e-01 |  |
| 340 | 66 | C | C | C | 94.21 | 0.000000e+00 | \* |
| 340 | 66 | C | C | G | 1.54 | 3.942824e-01 |  |
| 340 | 66 | C | C | T | 1.93 | 4.547661e-01 |  |
| 341 | 67 | T | T | A | 0.81 | 7.722804e-01 |  |
| 341 | 67 | T | T | C | 1.62 | 4.761538e-01 |  |
| 341 | 67 | T | T | G | 1.35 | 4.725769e-01 |  |
| 341 | 67 | T | T | T | 96.22 | 0.000000e+00 | \* |
| 342 | 68 | C | C | A | 0.00 | 8.454545e-01 |  |
| 342 | 68 | C | C | C | 94.58 | 0.000000e+00 | \* |
| 342 | 68 | C | C | G | 1.36 | 4.706290e-01 |  |
| 342 | 68 | C | C | T | 4.07 | 9.596661e-02 |  |
| 343 | 69 | C | C | A | 1.33 | 6.817290e-01 |  |
| 343 | 69 | C | C | C | 95.74 | 0.000000e+00 | \* |
| 343 | 69 | C | C | G | 0.00 | 9.111111e-01 |  |
| 343 | 69 | C | C | T | 2.93 | 2.321490e-01 |  |
| 344 | 70 | C | C | A | 6.08 | 9.336722e-02 |  |
| 344 | 70 | C | C | C | 89.35 | 0.000000e+00 | \* |
| 344 | 70 | C | C | G | 1.14 | 5.658253e-01 |  |
| 344 | 70 | C | C | T | 3.42 | 1.599339e-01 |  |
| 345 | 71 | G | G | A | 2.64 | 4.384114e-01 |  |
| 345 | 71 | G | G | C | 2.20 | 2.461433e-01 |  |
| 345 | 71 | G | G | G | 95.15 | 0.000000e+00 | \* |
| 345 | 71 | G | G | T | 0.00 | 9.139785e-01 |  |
| 346 | 72 | C | C | A | 2.39 | 4.821624e-01 |  |
| 346 | 72 | C | C | C | 92.83 | 0.000000e+00 | \* |
| 346 | 72 | C | C | G | 1.59 | 3.756696e-01 |  |
| 346 | 72 | C | C | T | 3.19 | 1.912230e-01 |  |
| 347 | 73 | G | G | A | 7.83 | 3.858145e-02 |  |
| 347 | 73 | G | G | C | 3.91 | 1.734769e-02 |  |
| 347 | 73 | G | G | G | 88.26 | 0.000000e+00 | \* |
| 347 | 73 | G | G | T | 0.00 | 9.139785e-01 |  |
| 348 | 74 | A | A | A | 94.30 | 0.000000e+00 | \* |
| 348 | 74 | A | A | C | 1.42 | 5.702887e-01 |  |
| 348 | 74 | A | A | G | 1.42 | 4.419680e-01 |  |
| 348 | 74 | A | A | T | 2.85 | 2.454152e-01 |  |
| 349 | 75 | C | C | A | 3.56 | 3.017429e-01 |  |
| 349 | 75 | C | C | C | 92.58 | 0.000000e+00 | \* |
| 349 | 75 | C | C | G | 0.00 | 9.111111e-01 |  |
| 349 | 75 | C | C | T | 3.86 | 1.136495e-01 |  |

## For use in R

If you want to work with the results in R, here is output that you can copy and paste in your terminal to get:

The base information:

```
structure(list(focal.base = c("A", "C", "G", "T"), avg.percsignal = c(92.59141434521, 
93.9554345908784, 93.0766126855635, 95.1145468627405), avg.areasignal = c(330.941176470588, 
343.181818181818, 323.80701754386, 389.818181818182), crit.perc.area = c(10.3657984313244, 
4.22202622149748, 4.44907904387214, 6.68282508203289), mu = c(3.24848528669561, 
1.78687272726925, 1.57286030193087, 2.21904330582038), fillibens = c(0.995104000536441, 
0.989811186750032, 0.992533035943131, 0.993707918522738)), .Names = c("focal.base", 
"avg.percsignal", "avg.areasignal", "crit.perc.area", "mu", "fillibens"
), row.names = c(NA, -4L), class = "data.frame")
```

the data.frame that contains information on the guide region:

```
structure(list(A.area = c(299, 373, 9, 592, 18, 17, 27, 20, 10, 
9, 242, 9, 547, 19, 12, 18, 0, 21, 0, 2, 0, 16, 1, 17, 4, 248, 
9, 6, 315, 7, 245, 7, 9, 11, 8, 4, 257, 9, 4, 4, 0, 15, 334, 
11, 18, 15, 463, 11, 11, 16, 4, 157, 293, 10, 323, 290, 6, 2, 
0, 7, 16, 378, 12, 201, 12, 6, 3, 0, 5, 16, 6, 6, 18, 331, 12
), C.area = c(6, 2, 5, 7, 340, 9, 0, 6, 223, 385, 6, 3, 7, 346, 
304, 5, 3, 0, 3, 0, 6, 0, 5, 6, 301, 9, 302, 13, 6, 322, 6, 293, 
6, 1, 4, 244, 8, 373, 332, 323, 5, 4, 5, 304, 277, 5, 4, 276, 
4, 3, 192, 3, 0, 5, 0, 0, 0, 7, 5, 267, 7, 5, 308, 5, 5, 244, 
6, 279, 360, 235, 5, 233, 9, 5, 312), G.area = c(3, 5, 271, 6, 
0, 12, 595, 389, 7, 10, 6, 308, 5, 8, 7, 307, 8, 489, 12, 8, 
7, 439, 7, 354, 7, 7, 27, 0, 4, 7, 11, 6, 224, 330, 270, 7, 7, 
5, 5, 8, 7, 399, 7, 12, 7, 242, 6, 4, 174, 348, 6, 3, 3, 226, 
9, 4, 188, 2, 9, 6, 201, 8, 0, 7, 308, 4, 5, 4, 0, 3, 216, 4, 
203, 5, 0), T.area = c(5, 20, 0, 25, 8, 402, 8, 2, 4, 9, 60, 
2, 25, 9, 3, 4, 254, 7, 325, 352, 356, 6, 291, 8, 0, 13, 7, 357, 
7, 7, 8, 5, 2, 2, 4, 0, 11, 7, 3, 7, 346, 7, 41, 0, 4, 0, 16, 
0, 0, 0, 8, 8, 10, 0, 13, 9, 7, 261, 281, 7, 1, 16, 6, 12, 4, 
5, 356, 12, 11, 9, 0, 8, 0, 10, 13), Tot.area = c(313, 400, 285, 
630, 366, 440, 630, 417, 244, 413, 314, 322, 584, 382, 326, 334, 
265, 517, 340, 362, 369, 461, 304, 385, 312, 277, 345, 376, 332, 
343, 270, 311, 241, 344, 286, 255, 283, 394, 344, 342, 358, 425, 
387, 327, 306, 262, 489, 291, 189, 367, 210, 171, 306, 241, 345, 
303, 201, 272, 295, 287, 225, 407, 326, 225, 329, 259, 370, 295, 
376, 263, 227, 251, 230, 351, 337), A.perc = c(95.5271565495208, 
93.25, 3.15789473684211, 93.968253968254, 4.91803278688525, 3.86363636363636, 
4.28571428571429, 4.79616306954436, 4.0983606557377, 2.17917675544794, 
77.0700636942675, 2.79503105590062, 93.6643835616438, 4.9738219895288, 
3.68098159509202, 5.38922155688623, 0, 4.06189555125725, 0, 0.552486187845304, 
0, 3.470715835141, 0.328947368421053, 4.41558441558442, 1.28205128205128, 
89.5306859205776, 2.60869565217391, 1.59574468085106, 94.8795180722892, 
2.04081632653061, 90.7407407407407, 2.2508038585209, 3.7344398340249, 
3.19767441860465, 2.7972027972028, 1.56862745098039, 90.8127208480565, 
2.28426395939086, 1.16279069767442, 1.16959064327485, 0, 3.52941176470588, 
86.3049095607235, 3.36391437308869, 5.88235294117647, 5.72519083969466, 
94.6830265848671, 3.78006872852234, 5.82010582010582, 4.35967302452316, 
1.9047619047619, 91.812865497076, 95.7516339869281, 4.149377593361, 
93.6231884057971, 95.7095709570957, 2.98507462686567, 0.735294117647059, 
0, 2.4390243902439, 7.11111111111111, 92.8746928746929, 3.68098159509202, 
89.3333333333333, 3.64741641337386, 2.31660231660232, 0.810810810810811, 
0, 1.32978723404255, 6.08365019011407, 2.6431718061674, 2.39043824701195, 
7.82608695652174, 94.3019943019943, 3.56083086053412), C.perc = c(1.91693290734824, 
0.5, 1.75438596491228, 1.11111111111111, 92.896174863388, 2.04545454545455, 
0, 1.43884892086331, 91.3934426229508, 93.2203389830509, 1.91082802547771, 
0.93167701863354, 1.1986301369863, 90.5759162303665, 93.2515337423313, 
1.49700598802395, 1.13207547169811, 0, 0.882352941176471, 0, 
1.6260162601626, 0, 1.64473684210526, 1.55844155844156, 96.474358974359, 
3.24909747292419, 87.536231884058, 3.45744680851064, 1.80722891566265, 
93.8775510204082, 2.22222222222222, 94.2122186495177, 2.4896265560166, 
0.290697674418605, 1.3986013986014, 95.6862745098039, 2.82685512367491, 
94.6700507614213, 96.5116279069767, 94.4444444444444, 1.39664804469274, 
0.941176470588235, 1.29198966408269, 92.9663608562691, 90.5228758169935, 
1.90839694656489, 0.81799591002045, 94.8453608247423, 2.11640211640212, 
0.817438692098093, 91.4285714285714, 1.75438596491228, 0, 2.0746887966805, 
0, 0, 0, 2.57352941176471, 1.69491525423729, 93.0313588850174, 
3.11111111111111, 1.22850122850123, 94.478527607362, 2.22222222222222, 
1.51975683890578, 94.2084942084942, 1.62162162162162, 94.5762711864407, 
95.7446808510638, 89.3536121673004, 2.20264317180617, 92.8286852589641, 
3.91304347826087, 1.42450142450142, 92.5816023738872), G.perc = c(0.958466453674121, 
1.25, 95.0877192982456, 0.952380952380952, 0, 2.72727272727273, 
94.4444444444444, 93.2853717026379, 2.86885245901639, 2.42130750605327, 
1.91082802547771, 95.6521739130435, 0.856164383561644, 2.09424083769634, 
2.14723926380368, 91.9161676646707, 3.0188679245283, 94.5841392649903, 
3.52941176470588, 2.20994475138122, 1.8970189701897, 95.2277657266811, 
2.30263157894737, 91.948051948052, 2.24358974358974, 2.52707581227437, 
7.82608695652174, 0, 1.20481927710843, 2.04081632653061, 4.07407407407407, 
1.92926045016077, 92.9460580912863, 95.9302325581395, 94.4055944055944, 
2.74509803921569, 2.47349823321555, 1.26903553299492, 1.45348837209302, 
2.33918128654971, 1.95530726256983, 93.8823529411765, 1.80878552971576, 
3.6697247706422, 2.28758169934641, 92.3664122137405, 1.22699386503067, 
1.3745704467354, 92.0634920634921, 94.8228882833787, 2.85714285714286, 
1.75438596491228, 0.980392156862745, 93.7759336099585, 2.60869565217391, 
1.32013201320132, 93.5323383084577, 0.735294117647059, 3.05084745762712, 
2.09059233449477, 89.3333333333333, 1.96560196560197, 0, 3.11111111111111, 
93.6170212765958, 1.54440154440154, 1.35135135135135, 1.35593220338983, 
0, 1.14068441064639, 95.1541850220264, 1.59362549800797, 88.2608695652174, 
1.42450142450142, 0), T.perc = c(1.59744408945687, 5, 0, 3.96825396825397, 
2.18579234972678, 91.3636363636364, 1.26984126984127, 0.479616306954436, 
1.63934426229508, 2.17917675544794, 19.1082802547771, 0.62111801242236, 
4.28082191780822, 2.35602094240838, 0.920245398773006, 1.19760479041916, 
95.8490566037736, 1.35396518375242, 95.5882352941177, 97.2375690607735, 
96.4769647696477, 1.30151843817787, 95.7236842105263, 2.07792207792208, 
0, 4.69314079422383, 2.02898550724638, 94.9468085106383, 2.10843373493976, 
2.04081632653061, 2.96296296296296, 1.60771704180064, 0.829875518672199, 
0.581395348837209, 1.3986013986014, 0, 3.886925795053, 1.77664974619289, 
0.872093023255814, 2.04678362573099, 96.6480446927374, 1.64705882352941, 
10.594315245478, 0, 1.30718954248366, 0, 3.2719836400818, 0, 
0, 0, 3.80952380952381, 4.67836257309941, 3.26797385620915, 0, 
3.76811594202899, 2.97029702970297, 3.48258706467662, 95.9558823529412, 
95.2542372881356, 2.4390243902439, 0.444444444444444, 3.93120393120393, 
1.84049079754601, 5.33333333333333, 1.21580547112462, 1.93050193050193, 
96.2162162162162, 4.06779661016949, 2.92553191489362, 3.42205323193916, 
0, 3.18725099601594, 0, 2.84900284900285, 3.85756676557863), 
    base.call = c("A", "A", "G", "A", "C", "T", "G", "G", "C", 
    "C", "A", "G", "A", "C", "C", "G", "T", "G", "T", "T", "T", 
    "G", "T", "G", "C", "A", "C", "T", "A", "C", "A", "C", "G", 
    "G", "G", "C", "A", "C", "C", "C", "T", "G", "A", "C", "C", 
    "G", "A", "C", "G", "G", "C", "A", "A", "G", "A", "A", "G", 
    "T", "T", "C", "G", "A", "C", "A", "G", "C", "T", "C", "C", 
    "C", "G", "C", "G", "A", "C"), index = 275:349, guide.seq = c("A", 
    "A", "G", "A", "C", "T", "G", "G", "C", "C", "A", "G", "A", 
    "C", "C", "G", "T", "G", "T", "T", "T", "G", "T", "G", "C", 
    "A", "C", "T", "A", "C", "A", "C", "G", "G", "G", "C", "A", 
    "C", "C", "C", "T", "G", "A", "C", "C", "G", "A", "C", "G", 
    "G", "C", "A", "A", "G", "A", "A", "G", "T", "T", "C", "G", 
    "A", "C", "A", "G", "C", "T", "C", "C", "C", "G", "C", "G", 
    "A", "C"), T.pval = c(0.552226975193012, 0.0441287471486461, 
    0.913978494580569, 0.104000255909816, 0.387200413272701, 
    0, 0.654452368879199, 0.870674633755549, 0.539490093888564, 
    0.388862900850215, 5.04100221565551e-08, 0.8410055426034, 
    0.0806521351994427, 0.3461040013599, 0.761745074813782, 0.677159924989343, 
    0, 0.627953525061876, 0, 0, 0, 0.644473142850092, 0, 0.414905932240187, 
    0.913978494580569, 0.0572387153050979, 0.427887893499206, 
    0, 0.406941004794762, 0.424726216728406, 0.225880162975397, 
    0.549093785540992, 0.787507980199267, 0.849958451969151, 
    0.613920181326809, 0.913978494580569, 0.11101387315601, 0.498636910892316, 
    0.775628156536736, 0.42313709291008, 0, 0.537157639502417, 
    0.000251204761158919, 0.913978494580569, 0.642686273702963, 
    0.913978494580569, 0.17936894718102, 0.913978494580569, 0.913978494580569, 
    0.913978494580569, 0.118084773876051, 0.0579544181543767, 
    0.179915248911461, 0.913978494580569, 0.122032383923823, 
    0.224668554116939, 0.152632883049916, 0, 0, 0.327250779638684, 
    0.877017209146629, 0.107143809510931, 0.480168517048071, 
    0.0331265232415904, 0.671449003308811, 0.454766071988185, 
    0, 0.0959666089146227, 0.232149038439543, 0.159933906216918, 
    0.913978494580569, 0.191223007838022, 0.913978494580569, 
    0.245415198159987, 0.113649457322732), C.pval = c(0.34792307199515, 
    0.900743809024331, 0.416014270053902, 0.718525042179349, 
    0, 0.299181565923599, 0.917647058735216, 0.563333793080475, 
    0, 0, 0.350354784778636, 0.792458322137281, 0.67862995005999, 
    0, 0, 0.535241777763892, 0.709146882201614, 0.917647058735216, 
    0.81030803739811, 0.917647058735216, 0.47410973394834, 0.917647058735216, 
    0.465439083871022, 0.505867790519438, 0, 0.0532767522962256, 
    0, 0.0378462333323175, 0.393143374469151, 0, 0.240049262010708, 
    0, 0.167822255979129, 0.915501587060614, 0.582853937604621, 
    0, 0.10300258403362, 0, 0, 0, 0.583801797186642, 0.78888258955356, 
    0.634402357131167, 0, 0, 0.351325999819675, 0.831663838254994, 
    0, 0.274336254752513, 0.831838649022318, 0, 0.416014270053902, 
    0.917647058735216, 0.288763815242264, 0.917647058735216, 
    0.917647058735216, 0.917647058735216, 0.149162238929101, 
    0.442515557423517, 0, 0.0664336784682438, 0.664626704789942, 
    0, 0.240049262010708, 0.524318964117469, 0, 0.476153807412335, 
    0, 0, 0, 0.246143346923018, 0, 0.017347692368497, 0.570288703782655, 
    0), G.pval = c(0.6497204218293, 0.516627793127681, 0, 0.652521829237767, 
    0.91111111108653, 0.103347522186686, 0, 0, 0.086412494414274, 
    0.150455851787017, 0.270096803105712, 0, 0.696399900363301, 
    0.220383280441399, 0.20748949897412, 0, 0.0712584137035517, 
    0, 0.0361860855603874, 0.193047095290427, 0.274169768067634, 
    0, 0.173251230434235, 0, 0.185651326057027, 0.132381218502082, 
    5.9087507668476e-05, 0.91111111108653, 0.536783771518735, 
    0.234036132836346, 0.0170413220011019, 0.264733438217915, 
    0, 0, 0, 0.101062005183205, 0.141284156441893, 0.508224041172509, 
    0.430145717146386, 0.16593422352803, 0.257296562279939, 0, 
    0.301313345857018, 0.0298850945674007, 0.176343197878577, 
    0, 0.526856120822716, 0.462745204104781, 0, 0, 0.0877110695766479, 
    0.319019933639777, 0.639613268745386, 0, 0.119768130107619, 
    0.485956363037719, 0, 0.749401416908, 0.0683613940584641, 
    0.221294548259001, 0, 0.254402863388164, 0.91111111108653, 
    0.0631952189114878, 0, 0.394282353461012, 0.472576856285125, 
    0.4706289599671, 0.91111111108653, 0.565825261761444, 0, 
    0.375669555748085, 0, 0.441967979594789, 0.91111111108653
    ), A.pval = c(0, 0, 0.357261324146096, 0, 0.163810588384796, 
    0.264652118770014, 0.21927458200476, 0.173424446085038, 0.238537197541468, 
    0.520415547041788, 0, 0.413312341233571, 0, 0.159569679568556, 
    0.286557381086687, 0.130960230968632, 0.845454545454545, 
    0.242447393695648, 0.845454545454545, 0.808368601995772, 
    0.845454545454545, 0.313542459313904, 0.831377381288775, 
    0.206713667731892, 0.690649987556681, 0, 0.444237991618762, 
    0.631097526368006, 0, 0.546153643212219, 0, 0.507292240629318, 
    0.280000044820431, 0.351463535571456, 0.41296022903319, 0.636302965220035, 
    0, 0.5012138837531, 0.712564946152498, 0.711332125975694, 
    0.845454545454545, 0.305816757682611, 0, 0.32798602998399, 
    0.103086845874668, 0.111315301187999, 0, 0.274499190733807, 
    0.106276493274634, 0.21204355226817, 0.571872281518647, 0, 
    0, 0.233155535133676, 0, 0, 0.383251034862534, 0.783683553769545, 
    0.845454545454545, 0.473570643901891, 0.0557355770046245, 
    0, 0.286557381086687, 0, 0.290737030228231, 0.495372408230057, 
    0.772280370499388, 0.845454545454545, 0.681728959927647, 
    0.0933672217591911, 0.438411426590774, 0.482162361124343, 
    0.0385814542837972, 0, 0.301742875681269), guide.position = 1:75), .Names = c("A.area", 
"C.area", "G.area", "T.area", "Tot.area", "A.perc", "C.perc", 
"G.perc", "T.perc", "base.call", "index", "guide.seq", "T.pval", 
"C.pval", "G.pval", "A.pval", "guide.position"), row.names = 275:349, class = "data.frame")
```

*Report generated using EditR v1.0.8*
