# Supplementary material for: Mechanistic and genetic basis of single-strand templated repair at Cas12a-induced DNA breaks in Chlamydomonas reinhardtii
Source: Nat Commun. 2021 Nov 19;12:6751. doi: 10.1038/s41467-021-27004-1 (PMC8604939; doi:10.1038/s41467-021-27004-1)
Supplement: Supplementary file 22 — Source Data [file 41467_2021_27004_MOESM22_ESM.zip › Source Data/EditR analysis/EditR outputs/Antisense/rep3_ssODN_antisense_-32_-16_0_16_32.html]

EditR v1.0.8 report


# EditR v1.0.8 report

- Data QA
  - Filtering data
  - Percent noise peak area
  - Base information
- Predicted editing
  - Editing bar plot
  - Editing table plot
  - Table of editing results
- For use in R

## Data QA

### Filtering data

What the data looked like prefiltering:

and the post filtering signal / noise plot:

### Percent noise peak area

### Base information

Here’s information about the signal of each base, the critical percent value where any higher value would be called as significant, and Filliben’s correlation for how well the noise was modelled by the zero adjusted gamma distribution.

| Base | Average percent signal | Average peak area | Critical percent value | model mu | Fillibens correlation |
| --- | --- | --- | --- | --- | --- |
| A | 92.38524 | 287.2059 | 9.872736 | 3.316941 | 0.9849608 |
| C | 92.44543 | 310.0290 | 5.505140 | 2.038907 | 0.9951170 |
| G | 92.48708 | 290.6182 | 5.604429 | 1.934424 | 0.9870708 |
| T | 94.45715 | 334.8140 | 7.905086 | 2.715526 | 0.9934209 |

## Predicted editing

### Editing bar plot

### Editing table plot

### Table of editing results


Here’s the entire guide region

| Sanger position | Guide position | Guide sequence | Sanger base call | Focal base | Focal base peak area | p value |  |
| --- | --- | --- | --- | --- | --- | --- | --- |
| 279 | 1 | A | A | A | 99.61 | 0.000000e+00 | \* |
| 279 | 1 | A | A | C | 0.00 | 9.390244e-01 |  |
| 279 | 1 | A | A | G | 0.39 | 8.615059e-01 |  |
| 279 | 1 | A | A | T | 0.00 | 9.255319e-01 |  |
| 280 | 2 | A | A | A | 92.11 | 0.000000e+00 | \* |
| 280 | 2 | A | A | C | 2.92 | 1.805767e-01 |  |
| 280 | 2 | A | A | G | 1.17 | 6.287572e-01 |  |
| 280 | 2 | A | A | T | 3.80 | 2.014331e-01 |  |
| 281 | 3 | G | G | A | 2.43 | 4.909049e-01 |  |
| 281 | 3 | G | G | C | 2.43 | 2.871063e-01 |  |
| 281 | 3 | G | G | G | 93.93 | 0.000000e+00 | \* |
| 281 | 3 | G | G | T | 1.21 | 7.642337e-01 |  |
| 282 | 4 | A | A | A | 94.50 | 0.000000e+00 | \* |
| 282 | 4 | A | A | C | 0.89 | 8.148135e-01 |  |
| 282 | 4 | A | A | G | 0.89 | 7.283431e-01 |  |
| 282 | 4 | A | A | T | 3.72 | 2.117154e-01 |  |
| 283 | 5 | C | C | A | 4.52 | 1.961214e-01 |  |
| 283 | 5 | C | C | C | 90.06 | 0.000000e+00 | \* |
| 283 | 5 | C | C | G | 3.31 | 1.101888e-01 |  |
| 283 | 5 | C | C | T | 2.11 | 5.305879e-01 |  |
| 284 | 6 | T | T | A | 0.00 | 8.272727e-01 |  |
| 284 | 6 | T | T | C | 3.41 | 1.104140e-01 |  |
| 284 | 6 | T | T | G | 2.27 | 2.835166e-01 |  |
| 284 | 6 | T | T | T | 94.32 | 0.000000e+00 | \* |
| 285 | 7 | G | G | A | 4.89 | 1.632965e-01 |  |
| 285 | 7 | G | G | C | 1.75 | 5.005258e-01 |  |
| 285 | 7 | G | G | G | 92.15 | 0.000000e+00 | \* |
| 285 | 7 | G | G | T | 1.22 | 7.624926e-01 |  |
| 286 | 8 | G | G | A | 4.64 | 1.842486e-01 |  |
| 286 | 8 | G | G | C | 1.09 | 7.451608e-01 |  |
| 286 | 8 | G | G | G | 91.80 | 0.000000e+00 | \* |
| 286 | 8 | G | G | T | 2.46 | 4.446159e-01 |  |
| 287 | 9 | C | C | A | 4.55 | 1.935028e-01 |  |
| 287 | 9 | C | C | C | 90.00 | 0.000000e+00 | \* |
| 287 | 9 | C | C | G | 5.45 | 1.180044e-02 |  |
| 287 | 9 | C | C | T | 0.00 | 9.255319e-01 |  |
| 288 | 10 | C | C | A | 2.47 | 4.829982e-01 |  |
| 288 | 10 | C | C | C | 92.86 | 0.000000e+00 | \* |
| 288 | 10 | C | C | G | 3.30 | 1.119649e-01 |  |
| 288 | 10 | C | C | T | 1.37 | 7.240605e-01 |  |
| 289 | 11 | A | A | A | 74.91 | 0.000000e+00 | \* |
| 289 | 11 | A | A | C | 1.85 | 4.650079e-01 |  |
| 289 | 11 | A | A | G | 1.85 | 3.991240e-01 |  |
| 289 | 11 | A | A | T | 21.40 | 8.761716e-08 | \* |
| 290 | 12 | G | G | A | 2.07 | 5.581801e-01 |  |
| 290 | 12 | G | G | C | 3.45 | 1.059680e-01 |  |
| 290 | 12 | G | G | G | 94.14 | 0.000000e+00 | \* |
| 290 | 12 | G | G | T | 0.34 | 9.151871e-01 |  |
| 291 | 13 | A | A | A | 95.67 | 0.000000e+00 | \* |
| 291 | 13 | A | A | C | 0.98 | 7.830291e-01 |  |
| 291 | 13 | A | A | G | 0.79 | 7.607896e-01 |  |
| 291 | 13 | A | A | T | 2.56 | 4.215917e-01 |  |
| 292 | 14 | C | C | A | 2.96 | 3.986792e-01 |  |
| 292 | 14 | C | C | C | 90.83 | 0.000000e+00 | \* |
| 292 | 14 | C | C | G | 3.85 | 6.500768e-02 |  |
| 292 | 14 | C | C | T | 2.37 | 4.664568e-01 |  |
| 293 | 15 | C | C | A | 0.00 | 8.272727e-01 |  |
| 293 | 15 | C | C | C | 97.08 | 0.000000e+00 | \* |
| 293 | 15 | C | C | G | 1.46 | 5.252287e-01 |  |
| 293 | 15 | C | C | T | 1.46 | 7.016117e-01 |  |
| 294 | 16 | G | G | A | 7.21 | 4.699140e-02 |  |
| 294 | 16 | G | G | C | 3.28 | 1.264214e-01 |  |
| 294 | 16 | G | G | G | 88.20 | 0.000000e+00 | \* |
| 294 | 16 | G | G | T | 1.31 | 7.399817e-01 |  |
| 295 | 17 | T | T | A | 1.74 | 6.208098e-01 |  |
| 295 | 17 | T | T | C | 4.35 | 3.940865e-02 |  |
| 295 | 17 | T | T | G | 2.17 | 3.077357e-01 |  |
| 295 | 17 | T | T | T | 91.74 | 0.000000e+00 | \* |
| 296 | 18 | G | G | A | 3.56 | 3.072476e-01 |  |
| 296 | 18 | G | G | C | 0.00 | 9.390244e-01 |  |
| 296 | 18 | G | G | G | 96.21 | 0.000000e+00 | \* |
| 296 | 18 | G | G | T | 0.22 | 9.219910e-01 |  |
| 297 | 19 | T | T | A | 0.00 | 8.272727e-01 |  |
| 297 | 19 | T | T | C | 1.48 | 5.989723e-01 |  |
| 297 | 19 | T | T | G | 2.22 | 2.957078e-01 |  |
| 297 | 19 | T | T | T | 96.30 | 0.000000e+00 | \* |
| 298 | 20 | T | T | A | 0.00 | 8.272727e-01 |  |
| 298 | 20 | T | T | C | 2.59 | 2.483576e-01 |  |
| 298 | 20 | T | T | G | 5.50 | 1.120368e-02 |  |
| 298 | 20 | T | T | T | 91.91 | 0.000000e+00 | \* |
| 299 | 21 | T | T | A | 0.00 | 8.272727e-01 |  |
| 299 | 21 | T | T | C | 1.98 | 4.190195e-01 |  |
| 299 | 21 | T | T | G | 2.31 | 2.747159e-01 |  |
| 299 | 21 | T | T | T | 95.71 | 0.000000e+00 | \* |
| 300 | 22 | G | G | A | 2.21 | 5.313118e-01 |  |
| 300 | 22 | G | G | C | 1.47 | 6.017493e-01 |  |
| 300 | 22 | G | G | G | 95.58 | 0.000000e+00 | \* |
| 300 | 22 | G | G | T | 0.74 | 8.664608e-01 |  |
| 301 | 23 | T | T | A | 0.00 | 8.272727e-01 |  |
| 301 | 23 | T | T | C | 3.23 | 1.334722e-01 |  |
| 301 | 23 | T | T | G | 1.61 | 4.730087e-01 |  |
| 301 | 23 | T | T | T | 95.16 | 0.000000e+00 | \* |
| 302 | 24 | G | G | A | 3.89 | 2.644638e-01 |  |
| 302 | 24 | G | G | C | 1.20 | 7.068417e-01 |  |
| 302 | 24 | G | G | G | 94.01 | 0.000000e+00 | \* |
| 302 | 24 | G | G | T | 0.90 | 8.360822e-01 |  |
| 303 | 25 | C | C | A | 1.44 | 6.754777e-01 |  |
| 303 | 25 | C | C | C | 94.22 | 0.000000e+00 | \* |
| 303 | 25 | C | C | G | 1.44 | 5.307495e-01 |  |
| 303 | 25 | C | C | T | 2.89 | 3.512875e-01 |  |
| 304 | 26 | A | A | A | 87.34 | 0.000000e+00 | \* |
| 304 | 26 | A | A | C | 3.38 | 1.143528e-01 |  |
| 304 | 26 | A | A | G | 4.22 | 4.434241e-02 |  |
| 304 | 26 | A | A | T | 5.06 | 8.557880e-02 |  |
| 305 | 27 | C | C | A | 2.66 | 4.498679e-01 |  |
| 305 | 27 | C | C | C | 87.04 | 0.000000e+00 | \* |
| 305 | 27 | C | C | G | 8.31 | 4.477342e-04 | \* |
| 305 | 27 | C | C | T | 1.99 | 5.602830e-01 |  |
| 306 | 28 | T | T | A | 1.64 | 6.385541e-01 |  |
| 306 | 28 | T | T | C | 2.96 | 1.742112e-01 |  |
| 306 | 28 | T | T | G | 0.99 | 6.938732e-01 |  |
| 306 | 28 | T | T | T | 94.41 | 0.000000e+00 | \* |
| 307 | 29 | A | A | A | 94.41 | 0.000000e+00 | \* |
| 307 | 29 | A | A | C | 3.15 | 1.446358e-01 |  |
| 307 | 29 | A | A | G | 0.70 | 7.877706e-01 |  |
| 307 | 29 | A | A | T | 1.75 | 6.250161e-01 |  |
| 308 | 30 | C | C | A | 2.01 | 5.687236e-01 |  |
| 308 | 30 | C | C | C | 93.96 | 0.000000e+00 | \* |
| 308 | 30 | C | C | G | 2.01 | 3.502766e-01 |  |
| 308 | 30 | C | C | T | 2.01 | 5.550637e-01 |  |
| 309 | 31 | A | A | A | 90.39 | 0.000000e+00 | \* |
| 309 | 31 | A | A | C | 2.62 | 2.413164e-01 |  |
| 309 | 31 | A | A | G | 2.18 | 3.053438e-01 |  |
| 309 | 31 | A | A | T | 4.80 | 1.027351e-01 |  |
| 310 | 32 | C | C | A | 1.45 | 6.745339e-01 |  |
| 310 | 32 | C | C | C | 95.29 | 0.000000e+00 | \* |
| 310 | 32 | C | C | G | 1.81 | 4.093141e-01 |  |
| 310 | 32 | C | C | T | 1.45 | 7.043845e-01 |  |
| 311 | 33 | G | G | A | 4.35 | 2.130784e-01 |  |
| 311 | 33 | G | G | C | 2.42 | 2.906259e-01 |  |
| 311 | 33 | G | G | G | 93.24 | 0.000000e+00 | \* |
| 311 | 33 | G | G | T | 0.00 | 9.255319e-01 |  |
| 312 | 34 | G | G | A | 2.58 | 4.635320e-01 |  |
| 312 | 34 | G | G | C | 2.58 | 2.502762e-01 |  |
| 312 | 34 | G | G | G | 94.19 | 0.000000e+00 | \* |
| 312 | 34 | G | G | T | 0.65 | 8.813447e-01 |  |
| 313 | 35 | G | G | A | 3.63 | 2.983247e-01 |  |
| 313 | 35 | G | G | C | 1.21 | 7.023394e-01 |  |
| 313 | 35 | G | G | G | 93.15 | 0.000000e+00 | \* |
| 313 | 35 | G | G | T | 2.02 | 5.543611e-01 |  |
| 314 | 36 | C | C | A | 3.07 | 3.806058e-01 |  |
| 314 | 36 | C | C | C | 91.23 | 0.000000e+00 | \* |
| 314 | 36 | C | C | G | 3.95 | 5.865834e-02 |  |
| 314 | 36 | C | C | T | 1.75 | 6.233815e-01 |  |
| 315 | 37 | A | A | A | 90.24 | 0.000000e+00 | \* |
| 315 | 37 | A | A | C | 2.85 | 1.948977e-01 |  |
| 315 | 37 | A | A | G | 2.85 | 1.712887e-01 |  |
| 315 | 37 | A | A | T | 4.07 | 1.696364e-01 |  |
| 316 | 38 | C | C | A | 1.79 | 6.119986e-01 |  |
| 316 | 38 | C | C | C | 96.43 | 0.000000e+00 | \* |
| 316 | 38 | C | C | G | 0.00 | 8.913043e-01 |  |
| 316 | 38 | C | C | T | 1.79 | 6.150397e-01 |  |
| 317 | 39 | C | C | A | 4.23 | 2.250247e-01 |  |
| 317 | 39 | C | C | C | 95.77 | 0.000000e+00 | \* |
| 317 | 39 | C | C | G | 0.00 | 8.913043e-01 |  |
| 317 | 39 | C | C | T | 0.00 | 9.255319e-01 |  |
| 318 | 40 | C | C | A | 4.76 | 1.738286e-01 |  |
| 318 | 40 | C | C | C | 90.79 | 0.000000e+00 | \* |
| 318 | 40 | C | C | G | 0.95 | 7.058614e-01 |  |
| 318 | 40 | C | C | T | 3.49 | 2.449124e-01 |  |
| 319 | 41 | T | T | A | 0.67 | 7.923668e-01 |  |
| 319 | 41 | T | T | C | 4.01 | 5.746252e-02 |  |
| 319 | 41 | T | T | G | 1.67 | 4.534555e-01 |  |
| 319 | 41 | T | T | T | 93.65 | 0.000000e+00 | \* |
| 320 | 42 | G | G | A | 4.02 | 2.489728e-01 |  |
| 320 | 42 | G | G | C | 2.41 | 2.912955e-01 |  |
| 320 | 42 | G | G | G | 92.23 | 0.000000e+00 | \* |
| 320 | 42 | G | G | T | 1.34 | 7.325820e-01 |  |
| 321 | 43 | A | A | A | 84.96 | 0.000000e+00 | \* |
| 321 | 43 | A | A | C | 1.18 | 7.134021e-01 |  |
| 321 | 43 | A | A | G | 1.18 | 6.250293e-01 |  |
| 321 | 43 | A | A | T | 12.68 | 1.913086e-04 | \* |
| 322 | 44 | C | C | A | 2.69 | 4.435984e-01 |  |
| 322 | 44 | C | C | C | 91.25 | 0.000000e+00 | \* |
| 322 | 44 | C | C | G | 4.71 | 2.635385e-02 |  |
| 322 | 44 | C | C | T | 1.35 | 7.309627e-01 |  |
| 323 | 45 | C | C | A | 7.87 | 3.246547e-02 |  |
| 323 | 45 | C | C | C | 88.39 | 0.000000e+00 | \* |
| 323 | 45 | C | C | G | 1.50 | 5.119533e-01 |  |
| 323 | 45 | C | C | T | 2.25 | 4.956619e-01 |  |
| 324 | 46 | G | G | A | 8.05 | 2.918326e-02 |  |
| 324 | 46 | G | G | C | 4.66 | 2.744627e-02 |  |
| 324 | 46 | G | G | G | 87.29 | 0.000000e+00 | \* |
| 324 | 46 | G | G | T | 0.00 | 9.255319e-01 |  |
| 325 | 47 | A | A | A | 95.68 | 0.000000e+00 | \* |
| 325 | 47 | A | A | C | 0.72 | 8.624713e-01 |  |
| 325 | 47 | A | A | G | 0.72 | 7.817876e-01 |  |
| 325 | 47 | A | A | T | 2.88 | 3.533753e-01 |  |
| 326 | 48 | C | C | A | 4.37 | 2.113061e-01 |  |
| 326 | 48 | C | C | C | 94.84 | 0.000000e+00 | \* |
| 326 | 48 | C | C | G | 0.79 | 7.588032e-01 |  |
| 326 | 48 | C | C | T | 0.00 | 9.255319e-01 |  |
| 327 | 49 | G | G | A | 6.98 | 5.363184e-02 |  |
| 327 | 49 | G | G | C | 1.74 | 5.008929e-01 |  |
| 327 | 49 | G | G | G | 87.79 | 0.000000e+00 | \* |
| 327 | 49 | G | G | T | 3.49 | 2.454747e-01 |  |
| 328 | 50 | G | G | A | 7.42 | 4.183760e-02 |  |
| 328 | 50 | G | G | C | 0.97 | 7.885676e-01 |  |
| 328 | 50 | G | G | G | 91.29 | 0.000000e+00 | \* |
| 328 | 50 | G | G | T | 0.32 | 9.167263e-01 |  |
| 329 | 51 | C | C | A | 4.95 | 1.585481e-01 |  |
| 329 | 51 | C | C | C | 89.01 | 0.000000e+00 | \* |
| 329 | 51 | C | C | G | 1.65 | 4.612763e-01 |  |
| 329 | 51 | C | C | T | 4.40 | 1.360029e-01 |  |
| 330 | 52 | A | A | A | 86.16 | 0.000000e+00 | \* |
| 330 | 52 | A | A | C | 1.26 | 6.842404e-01 |  |
| 330 | 52 | A | A | G | 4.40 | 3.663162e-02 |  |
| 330 | 52 | A | A | T | 8.18 | 8.062824e-03 | \* |
| 331 | 53 | A | A | A | 95.45 | 0.000000e+00 | \* |
| 331 | 53 | A | A | C | 0.38 | 9.246484e-01 |  |
| 331 | 53 | A | A | G | 1.14 | 6.407124e-01 |  |
| 331 | 53 | A | A | T | 3.03 | 3.235889e-01 |  |
| 332 | 54 | G | G | A | 2.54 | 4.711545e-01 |  |
| 332 | 54 | G | G | C | 1.52 | 5.832304e-01 |  |
| 332 | 54 | G | G | G | 95.94 | 0.000000e+00 | \* |
| 332 | 54 | G | G | T | 0.00 | 9.255319e-01 |  |
| 333 | 55 | A | A | A | 96.49 | 0.000000e+00 | \* |
| 333 | 55 | A | A | C | 0.00 | 9.390244e-01 |  |
| 333 | 55 | A | A | G | 0.35 | 8.679822e-01 |  |
| 333 | 55 | A | A | T | 3.16 | 3.001401e-01 |  |
| 334 | 56 | A | A | A | 94.07 | 0.000000e+00 | \* |
| 334 | 56 | A | A | C | 0.00 | 9.390244e-01 |  |
| 334 | 56 | A | A | G | 1.19 | 6.229292e-01 |  |
| 334 | 56 | A | A | T | 4.74 | 1.071475e-01 |  |
| 335 | 57 | G | G | A | 3.75 | 2.823726e-01 |  |
| 335 | 57 | G | G | C | 0.62 | 8.848211e-01 |  |
| 335 | 57 | G | G | G | 93.12 | 0.000000e+00 | \* |
| 335 | 57 | G | G | T | 2.50 | 4.350949e-01 |  |
| 336 | 58 | T | T | A | 3.67 | 2.928845e-01 |  |
| 336 | 58 | T | T | C | 3.21 | 1.355056e-01 |  |
| 336 | 58 | T | T | G | 0.46 | 8.486345e-01 |  |
| 336 | 58 | T | T | T | 92.66 | 0.000000e+00 | \* |
| 337 | 59 | T | T | A | 8.37 | 2.429376e-02 |  |
| 337 | 59 | T | T | C | 1.26 | 6.852335e-01 |  |
| 337 | 59 | T | T | G | 2.51 | 2.312972e-01 |  |
| 337 | 59 | T | T | T | 87.87 | 0.000000e+00 | \* |
| 338 | 60 | C | C | A | 1.24 | 7.103701e-01 |  |
| 338 | 60 | C | C | C | 93.78 | 0.000000e+00 | \* |
| 338 | 60 | C | C | G | 3.32 | 1.095248e-01 |  |
| 338 | 60 | C | C | T | 1.66 | 6.486188e-01 |  |
| 339 | 61 | G | G | A | 5.59 | 1.139061e-01 |  |
| 339 | 61 | G | G | C | 1.68 | 5.258191e-01 |  |
| 339 | 61 | G | G | G | 88.27 | 0.000000e+00 | \* |
| 339 | 61 | G | G | T | 4.47 | 1.293631e-01 |  |
| 340 | 62 | A | A | A | 94.19 | 0.000000e+00 | \* |
| 340 | 62 | A | A | C | 1.16 | 7.197383e-01 |  |
| 340 | 62 | A | A | G | 0.58 | 8.202911e-01 |  |
| 340 | 62 | A | A | T | 4.07 | 1.691086e-01 |  |
| 341 | 63 | C | C | A | 2.16 | 5.412897e-01 |  |
| 341 | 63 | C | C | C | 94.60 | 0.000000e+00 | \* |
| 341 | 63 | C | C | G | 1.44 | 5.325678e-01 |  |
| 341 | 63 | C | C | T | 1.80 | 6.116227e-01 |  |
| 342 | 64 | A | A | A | 91.67 | 0.000000e+00 | \* |
| 342 | 64 | A | A | C | 1.67 | 5.292574e-01 |  |
| 342 | 64 | A | A | G | 3.33 | 1.080678e-01 |  |
| 342 | 64 | A | A | T | 3.33 | 2.700443e-01 |  |
| 343 | 65 | G | G | A | 3.36 | 3.368163e-01 |  |
| 343 | 65 | G | G | C | 3.02 | 1.642360e-01 |  |
| 343 | 65 | G | G | G | 90.94 | 0.000000e+00 | \* |
| 343 | 65 | G | G | T | 2.68 | 3.937694e-01 |  |
| 344 | 66 | C | C | A | 2.21 | 5.311077e-01 |  |
| 344 | 66 | C | C | C | 93.81 | 0.000000e+00 | \* |
| 344 | 66 | C | C | G | 1.77 | 4.222426e-01 |  |
| 344 | 66 | C | C | T | 2.21 | 5.043207e-01 |  |
| 345 | 67 | T | T | A | 0.00 | 8.272727e-01 |  |
| 345 | 67 | T | T | C | 0.34 | 9.279690e-01 |  |
| 345 | 67 | T | T | G | 2.07 | 3.351054e-01 |  |
| 345 | 67 | T | T | T | 97.59 | 0.000000e+00 | \* |
| 346 | 68 | C | C | A | 2.38 | 4.997496e-01 |  |
| 346 | 68 | C | C | C | 94.05 | 0.000000e+00 | \* |
| 346 | 68 | C | C | G | 2.38 | 2.586997e-01 |  |
| 346 | 68 | C | C | T | 1.19 | 7.701366e-01 |  |
| 347 | 69 | C | C | A | 1.59 | 6.492528e-01 |  |
| 347 | 69 | C | C | C | 93.65 | 0.000000e+00 | \* |
| 347 | 69 | C | C | G | 0.00 | 8.913043e-01 |  |
| 347 | 69 | C | C | T | 4.76 | 1.057547e-01 |  |
| 348 | 70 | C | C | A | 0.00 | 8.272727e-01 |  |
| 348 | 70 | C | C | C | 92.68 | 0.000000e+00 | \* |
| 348 | 70 | C | C | G | 1.46 | 5.239883e-01 |  |
| 348 | 70 | C | C | T | 5.85 | 4.827652e-02 |  |
| 349 | 71 | G | G | A | 4.98 | 1.561541e-01 |  |
| 349 | 71 | G | G | C | 1.49 | 5.947582e-01 |  |
| 349 | 71 | G | G | G | 92.04 | 0.000000e+00 | \* |
| 349 | 71 | G | G | T | 1.49 | 6.930177e-01 |  |
| 350 | 72 | C | C | A | 2.21 | 5.311077e-01 |  |
| 350 | 72 | C | C | C | 90.71 | 0.000000e+00 | \* |
| 350 | 72 | C | C | G | 0.88 | 7.288708e-01 |  |
| 350 | 72 | C | C | T | 6.19 | 3.745646e-02 |  |
| 351 | 73 | G | G | A | 10.05 | 8.975539e-03 | \* |
| 351 | 73 | G | G | C | 2.65 | 2.356782e-01 |  |
| 351 | 73 | G | G | G | 87.30 | 0.000000e+00 | \* |
| 351 | 73 | G | G | T | 0.00 | 9.255319e-01 |  |
| 352 | 74 | A | A | A | 94.44 | 0.000000e+00 | \* |
| 352 | 74 | A | A | C | 1.04 | 7.633026e-01 |  |
| 352 | 74 | A | A | G | 0.00 | 8.913043e-01 |  |
| 352 | 74 | A | A | T | 4.51 | 1.254835e-01 |  |
| 353 | 75 | C | C | A | 2.79 | 4.273798e-01 |  |
| 353 | 75 | C | C | C | 83.28 | 0.000000e+00 | \* |
| 353 | 75 | C | C | G | 11.50 | 9.427352e-06 | \* |
| 353 | 75 | C | C | T | 2.44 | 4.493040e-01 |  |

## For use in R

If you want to work with the results in R, here is output that you can copy and paste in your terminal to get:

The base information:

```
structure(list(focal.base = c("A", "C", "G", "T"), avg.percsignal = c(92.3852380585322, 
92.4454291841938, 92.4870802015732, 94.45714576544), avg.areasignal = c(287.205882352941, 
310.028985507246, 290.618181818182, 334.813953488372), crit.perc.area = c(9.87273605463335, 
5.50513957622382, 5.60442907788347, 7.90508635693777), mu = c(3.31694100554363, 
2.03890675693293, 1.93442396946846, 2.71552627498901), fillibens = c(0.984960822340607, 
0.995116983840396, 0.987070796897791, 0.993420891863868)), .Names = c("focal.base", 
"avg.percsignal", "avg.areasignal", "crit.perc.area", "mu", "fillibens"
), row.names = c(NA, -4L), class = "data.frame")
```

the data.frame that contains information on the guide region:

```
structure(list(A.area = c(255, 315, 6, 533, 15, 0, 28, 17, 10, 
9, 203, 6, 486, 10, 0, 22, 4, 16, 0, 0, 0, 9, 0, 13, 4, 207, 
8, 5, 270, 6, 207, 4, 9, 8, 9, 7, 222, 6, 13, 15, 2, 15, 288, 
8, 21, 19, 399, 11, 12, 23, 9, 137, 252, 5, 275, 238, 6, 8, 20, 
3, 10, 324, 6, 165, 10, 5, 0, 6, 5, 0, 10, 5, 19, 272, 8), C.area = c(0, 
10, 6, 5, 299, 12, 10, 4, 198, 338, 5, 10, 5, 307, 266, 10, 10, 
0, 4, 8, 6, 6, 8, 4, 261, 8, 262, 9, 9, 280, 6, 263, 5, 8, 3, 
208, 7, 324, 294, 286, 12, 9, 4, 271, 236, 11, 3, 239, 3, 3, 
162, 2, 1, 3, 0, 0, 1, 7, 3, 226, 3, 4, 263, 3, 9, 212, 1, 237, 
295, 190, 3, 205, 5, 3, 239), G.area = c(1, 4, 232, 5, 11, 8, 
528, 336, 12, 12, 5, 273, 4, 13, 4, 269, 5, 432, 6, 17, 7, 389, 
4, 314, 4, 10, 25, 3, 2, 6, 5, 5, 193, 292, 231, 9, 7, 0, 0, 
3, 5, 344, 4, 14, 4, 206, 3, 2, 151, 283, 3, 7, 3, 189, 1, 3, 
149, 1, 6, 8, 158, 2, 4, 6, 271, 4, 6, 6, 0, 3, 185, 2, 165, 
0, 33), T.area = c(0, 13, 3, 21, 7, 332, 7, 9, 0, 5, 58, 1, 13, 
8, 4, 4, 211, 1, 260, 284, 290, 3, 236, 3, 8, 12, 6, 287, 5, 
6, 11, 4, 0, 2, 5, 4, 10, 6, 0, 11, 280, 5, 43, 4, 6, 0, 12, 
0, 6, 1, 8, 13, 8, 0, 9, 12, 4, 202, 210, 4, 8, 14, 5, 6, 8, 
5, 283, 3, 15, 12, 3, 14, 0, 13, 7), Tot.area = c(256, 342, 247, 
564, 332, 352, 573, 366, 220, 364, 271, 290, 508, 338, 274, 305, 
230, 449, 270, 309, 303, 407, 248, 334, 277, 237, 301, 304, 286, 
298, 229, 276, 207, 310, 248, 228, 246, 336, 307, 315, 299, 373, 
339, 297, 267, 236, 417, 252, 172, 310, 182, 159, 264, 197, 285, 
253, 160, 218, 239, 241, 179, 344, 278, 180, 298, 226, 290, 252, 
315, 205, 201, 226, 189, 288, 287), A.perc = c(99.609375, 92.1052631578947, 
2.42914979757085, 94.5035460992908, 4.51807228915663, 0, 4.88656195462478, 
4.6448087431694, 4.54545454545455, 2.47252747252747, 74.9077490774908, 
2.06896551724138, 95.6692913385827, 2.9585798816568, 0, 7.21311475409836, 
1.73913043478261, 3.56347438752784, 0, 0, 0, 2.21130221130221, 
0, 3.89221556886228, 1.44404332129964, 87.3417721518987, 2.6578073089701, 
1.64473684210526, 94.4055944055944, 2.01342281879195, 90.3930131004367, 
1.44927536231884, 4.34782608695652, 2.58064516129032, 3.62903225806452, 
3.07017543859649, 90.2439024390244, 1.78571428571429, 4.23452768729642, 
4.76190476190476, 0.668896321070234, 4.02144772117962, 84.9557522123894, 
2.69360269360269, 7.86516853932584, 8.05084745762712, 95.6834532374101, 
4.36507936507936, 6.97674418604651, 7.41935483870968, 4.94505494505495, 
86.1635220125786, 95.4545454545455, 2.53807106598985, 96.4912280701754, 
94.0711462450593, 3.75, 3.6697247706422, 8.36820083682008, 1.2448132780083, 
5.58659217877095, 94.1860465116279, 2.15827338129496, 91.6666666666667, 
3.35570469798658, 2.21238938053097, 0, 2.38095238095238, 1.58730158730159, 
0, 4.97512437810945, 2.21238938053097, 10.0529100529101, 94.4444444444444, 
2.78745644599303), C.perc = c(0, 2.92397660818713, 2.42914979757085, 
0.886524822695035, 90.0602409638554, 3.40909090909091, 1.74520069808028, 
1.09289617486339, 90, 92.8571428571429, 1.8450184501845, 3.44827586206897, 
0.984251968503937, 90.8284023668639, 97.0802919708029, 3.27868852459016, 
4.34782608695652, 0, 1.48148148148148, 2.58899676375405, 1.98019801980198, 
1.47420147420147, 3.2258064516129, 1.19760479041916, 94.2238267148014, 
3.37552742616034, 87.0431893687708, 2.96052631578947, 3.14685314685315, 
93.9597315436242, 2.62008733624454, 95.2898550724638, 2.41545893719807, 
2.58064516129032, 1.20967741935484, 91.2280701754386, 2.84552845528455, 
96.4285714285714, 95.7654723127036, 90.7936507936508, 4.0133779264214, 
2.41286863270777, 1.17994100294985, 91.2457912457913, 88.3895131086142, 
4.66101694915254, 0.719424460431655, 94.8412698412698, 1.74418604651163, 
0.967741935483871, 89.010989010989, 1.25786163522013, 0.378787878787879, 
1.52284263959391, 0, 0, 0.625, 3.21100917431193, 1.25523012552301, 
93.7759336099585, 1.67597765363128, 1.16279069767442, 94.6043165467626, 
1.66666666666667, 3.02013422818792, 93.8053097345133, 0.344827586206897, 
94.0476190476191, 93.6507936507936, 92.6829268292683, 1.49253731343284, 
90.7079646017699, 2.64550264550265, 1.04166666666667, 83.2752613240418
), G.perc = c(0.390625, 1.16959064327485, 93.9271255060729, 0.886524822695035, 
3.31325301204819, 2.27272727272727, 92.1465968586387, 91.8032786885246, 
5.45454545454545, 3.2967032967033, 1.8450184501845, 94.1379310344828, 
0.78740157480315, 3.84615384615385, 1.45985401459854, 88.1967213114754, 
2.17391304347826, 96.2138084632517, 2.22222222222222, 5.50161812297735, 
2.31023102310231, 95.5773955773956, 1.61290322580645, 94.0119760479042, 
1.44404332129964, 4.21940928270042, 8.30564784053156, 0.986842105263158, 
0.699300699300699, 2.01342281879195, 2.18340611353712, 1.81159420289855, 
93.2367149758454, 94.1935483870968, 93.1451612903226, 3.94736842105263, 
2.84552845528455, 0, 0, 0.952380952380952, 1.67224080267559, 
92.2252010723861, 1.17994100294985, 4.71380471380471, 1.49812734082397, 
87.2881355932203, 0.719424460431655, 0.793650793650794, 87.7906976744186, 
91.2903225806452, 1.64835164835165, 4.40251572327044, 1.13636363636364, 
95.9390862944162, 0.350877192982456, 1.18577075098814, 93.125, 
0.458715596330275, 2.51046025104602, 3.3195020746888, 88.268156424581, 
0.581395348837209, 1.43884892086331, 3.33333333333333, 90.9395973154362, 
1.76991150442478, 2.06896551724138, 2.38095238095238, 0, 1.46341463414634, 
92.0398009950249, 0.884955752212389, 87.3015873015873, 0, 11.4982578397213
), T.perc = c(0, 3.80116959064327, 1.21457489878543, 3.72340425531915, 
2.10843373493976, 94.3181818181818, 1.2216404886562, 2.45901639344262, 
0, 1.37362637362637, 21.4022140221402, 0.344827586206897, 2.55905511811024, 
2.36686390532544, 1.45985401459854, 1.31147540983607, 91.7391304347826, 
0.22271714922049, 96.2962962962963, 91.9093851132686, 95.7095709570957, 
0.737100737100737, 95.1612903225806, 0.898203592814371, 2.88808664259928, 
5.06329113924051, 1.99335548172757, 94.4078947368421, 1.74825174825175, 
2.01342281879195, 4.80349344978166, 1.44927536231884, 0, 0.645161290322581, 
2.01612903225806, 1.75438596491228, 4.0650406504065, 1.78571428571429, 
0, 3.49206349206349, 93.6454849498328, 1.34048257372654, 12.6843657817109, 
1.34680134680135, 2.24719101123596, 0, 2.87769784172662, 0, 3.48837209302326, 
0.32258064516129, 4.3956043956044, 8.17610062893082, 3.03030303030303, 
0, 3.15789473684211, 4.74308300395257, 2.5, 92.6605504587156, 
87.8661087866109, 1.6597510373444, 4.46927374301676, 4.06976744186047, 
1.79856115107914, 3.33333333333333, 2.68456375838926, 2.21238938053097, 
97.5862068965517, 1.19047619047619, 4.76190476190476, 5.85365853658537, 
1.49253731343284, 6.19469026548673, 0, 4.51388888888889, 2.4390243902439
), base.call = c("A", "A", "G", "A", "C", "T", "G", "G", "C", 
"C", "A", "G", "A", "C", "C", "G", "T", "G", "T", "T", "T", "G", 
"T", "G", "C", "A", "C", "T", "A", "C", "A", "C", "G", "G", "G", 
"C", "A", "C", "C", "C", "T", "G", "A", "C", "C", "G", "A", "C", 
"G", "G", "C", "A", "A", "G", "A", "A", "G", "T", "T", "C", "G", 
"A", "C", "A", "G", "C", "T", "C", "C", "C", "G", "C", "G", "A", 
"C"), index = 279:353, guide.seq = c("A", "A", "G", "A", "C", 
"T", "G", "G", "C", "C", "A", "G", "A", "C", "C", "G", "T", "G", 
"T", "T", "T", "G", "T", "G", "C", "A", "C", "T", "A", "C", "A", 
"C", "G", "G", "G", "C", "A", "C", "C", "C", "T", "G", "A", "C", 
"C", "G", "A", "C", "G", "G", "C", "A", "A", "G", "A", "A", "G", 
"T", "T", "C", "G", "A", "C", "A", "G", "C", "T", "C", "C", "C", 
"G", "C", "G", "A", "C"), T.pval = c(0.925531914893617, 0.201433065440302, 
0.764233730403961, 0.211715390736177, 0.530587912416756, 0, 0.762492557458821, 
0.444615947986341, 0.925531914893617, 0.724060490865918, 8.76171591945862e-08, 
0.915187138271151, 0.421591696991457, 0.466456840246584, 0.701611717166195, 
0.739981693406712, 0, 0.921991028847471, 0, 0, 0, 0.866460799822638, 
0, 0.83608217159688, 0.35128750394907, 0.0855787973008197, 0.560283037582765, 
0, 0.625016147596268, 0.555063722447397, 0.102735059987306, 0.704384486059824, 
0.925531914893617, 0.881344681440599, 0.554361099935428, 0.623381540697932, 
0.169636419900811, 0.61503969311665, 0.925531914893617, 0.24491237486452, 
0, 0.732582041304244, 0.000191308629678977, 0.730962698772446, 
0.495661863633866, 0.925531914893617, 0.353375331951659, 0.925531914893617, 
0.245474665761818, 0.916726312622001, 0.136002910516984, 0.00806282413061776, 
0.323588881068147, 0.925531914893617, 0.30014008814162, 0.107147510617526, 
0.435094921991856, 0, 0, 0.648618822723049, 0.12936305395567, 
0.169108625459718, 0.611622655279126, 0.270044302722025, 0.393769399261348, 
0.504320677821409, 0, 0.770136634664773, 0.105754711295886, 0.0482765219328809, 
0.693017671706247, 0.0374564622941135, 0.925531914893617, 0.125483501820277, 
0.449303982875888), C.pval = c(0.939024390243902, 0.180576733800804, 
0.287106266669584, 0.814813465575372, 0, 0.110413962985878, 0.50052577435997, 
0.745160781286129, 0, 0, 0.465007949513578, 0.105968021998726, 
0.783029120606736, 0, 0, 0.126421379514072, 0.0394086502480497, 
0.939024390243902, 0.598972337938507, 0.248357620225415, 0.419019528556439, 
0.601749305459684, 0.133472207254236, 0.706841707055325, 0, 0.114352826075389, 
0, 0.174211224998554, 0.144635790349403, 0, 0.241316372090442, 
0, 0.290625949845921, 0.250276224986206, 0.702339391201313, 0, 
0.19489773846515, 0, 0, 0, 0.0574625201832506, 0.291295466254176, 
0.713402063238474, 0, 0, 0.0274462670676066, 0.862471320535199, 
0, 0.500892864087966, 0.788567551696285, 0, 0.684240366079899, 
0.924648410920359, 0.583230370337284, 0.939024390243902, 0.939024390243902, 
0.88482105151568, 0.135505550833814, 0.685233548264124, 0, 0.525819065888799, 
0.719738321803639, 0, 0.529257419091604, 0.164235977218619, 0, 
0.927969042160526, 0, 0, 0, 0.594758248733786, 0, 0.23567818097343, 
0.763302579060377, 0), G.pval = c(0.86150586215728, 0.628757182375073, 
0, 0.728343070025613, 0.110188805940426, 0.283516590657329, 0, 
0, 0.0118004416931994, 0.111964946804571, 0.399123950221563, 
0, 0.760789597174649, 0.0650076800028565, 0.525228719897524, 
0, 0.307735722679372, 0, 0.295707819178537, 0.0112036809419478, 
0.274715880459378, 0, 0.473008686014418, 0, 0.530749540903732, 
0.0443424102542676, 0.00044773421605504, 0.693873238383352, 0.78777055513271, 
0.350276570160906, 0.30534377933186, 0.409314121813618, 0, 0, 
0, 0.058658342236016, 0.171288728716375, 0.891304347825587, 0.891304347825587, 
0.705861370498214, 0.453455524958706, 0, 0.625029321005233, 0.0263538487008544, 
0.511953349595769, 0, 0.781787599283885, 0.758803233423317, 0, 
0, 0.461276331542843, 0.0366316171819148, 0.640712414881134, 
0, 0.867982235858214, 0.622929186820313, 0, 0.848634547989972, 
0.231297188822977, 0.10952476215018, 0, 0.820291079678378, 0.532567761028458, 
0.108067798200693, 0, 0.42224263251296, 0.335105410630383, 0.258699730072772, 
0.891304347825587, 0.523988281721764, 0, 0.728870788225923, 0, 
0.891304347825587, 9.42735239439774e-06), A.pval = c(0, 0, 0.490904939725713, 
0, 0.196121376606612, 0.827272727272728, 0.163296505969248, 0.184248642520365, 
0.193502820059366, 0.482998229039597, 0, 0.558180055042892, 0, 
0.398679224492729, 0.827272727272728, 0.0469914013853356, 0.620809833697678, 
0.307247555543027, 0.827272727272728, 0.827272727272728, 0.827272727272728, 
0.531311809367432, 0.827272727272728, 0.264463785374687, 0.675477740878181, 
0, 0.449867918219181, 0.638554123106361, 0, 0.568723609885276, 
0, 0.674533941008598, 0.213078410008103, 0.463531969181322, 0.298324715056934, 
0.380605777319998, 0, 0.611998555909412, 0.225024676891071, 0.173828639956487, 
0.792366839249077, 0.248972826896186, 0, 0.44359843699723, 0.0324654674903114, 
0.0291832609815016, 0, 0.211306107595257, 0.0536318354403532, 
0.0418376029636366, 0.158548076805417, 0, 0, 0.471154460020871, 
0, 0, 0.28237264966748, 0.292884479527475, 0.0242937630079398, 
0.710370122448117, 0.11390610203414, 0, 0.541289710573724, 0, 
0.336816267848139, 0.53110772408321, 0.827272727272728, 0.499749634824708, 
0.649252812531156, 0.827272727272728, 0.156154145498469, 0.53110772408321, 
0.00897553870855039, 0, 0.427379847755048), guide.position = 1:75), .Names = c("A.area", 
"C.area", "G.area", "T.area", "Tot.area", "A.perc", "C.perc", 
"G.perc", "T.perc", "base.call", "index", "guide.seq", "T.pval", 
"C.pval", "G.pval", "A.pval", "guide.position"), row.names = 279:353, class = "data.frame")
```

*Report generated using EditR v1.0.8*
