# Supplementary material for: Mechanistic and genetic basis of single-strand templated repair at Cas12a-induced DNA breaks in Chlamydomonas reinhardtii
Source: Nat Commun. 2021 Nov 19;12:6751. doi: 10.1038/s41467-021-27004-1 (PMC8604939; doi:10.1038/s41467-021-27004-1)
Supplement: Supplementary file 22 — Source Data [file 41467_2021_27004_MOESM22_ESM.zip › Source Data/EditR analysis/EditR outputs/Controls for normalization/Negative control.html]

EditR v1.0.8 report


# EditR v1.0.8 report

- Data QA
  - Filtering data
  - Percent noise peak area
  - Base information
- Predicted editing
  - Editing bar plot
  - Editing table plot
  - Table of editing results
- For use in R

## Data QA

### Filtering data

What the data looked like prefiltering:

and the post filtering signal / noise plot:

### Percent noise peak area

### Base information

Here’s information about the signal of each base, the critical percent value where any higher value would be called as significant, and Filliben’s correlation for how well the noise was modelled by the zero adjusted gamma distribution.

| Base | Average percent signal | Average peak area | Critical percent value | model mu | Fillibens correlation |
| --- | --- | --- | --- | --- | --- |
| A | 90.15948 | 598.5806 | 12.32875 | 3.258807 | 0.9972404 |
| C | 90.24655 | 558.9710 | 12.51168 | 3.425951 | 0.9893119 |
| G | 91.06259 | 595.9310 | 16.66642 | 4.525446 | 0.9889866 |
| T | 91.09285 | 604.6279 | 13.60703 | 3.773733 | 0.9909279 |

## Predicted editing

### Editing bar plot

### Editing table plot

### Table of editing results


Here’s the entire guide region

| Sanger position | Guide position | Guide sequence | Sanger base call | Focal base | Focal base peak area | p value |  |
| --- | --- | --- | --- | --- | --- | --- | --- |
| 284 | 1 | A | A | A | 87.34 | 3.330669e-16 | \* |
| 284 | 1 | A | A | C | 4.27 | 2.694578e-01 |  |
| 284 | 1 | A | A | G | 4.43 | 3.375612e-01 |  |
| 284 | 1 | A | A | T | 3.96 | 3.160186e-01 |  |
| 285 | 2 | A | A | A | 93.22 | 0.000000e+00 | \* |
| 285 | 2 | A | A | C | 4.78 | 2.236034e-01 |  |
| 285 | 2 | A | A | G | 2.00 | 6.064329e-01 |  |
| 285 | 2 | A | A | T | 0.00 | 8.510638e-01 |  |
| 286 | 3 | G | G | A | 8.44 | 4.610505e-02 |  |
| 286 | 3 | G | G | C | 1.26 | 7.282335e-01 |  |
| 286 | 3 | G | G | G | 85.28 | 3.805178e-12 | \* |
| 286 | 3 | G | G | T | 5.03 | 2.223626e-01 |  |
| 287 | 4 | A | A | A | 93.48 | 0.000000e+00 | \* |
| 287 | 4 | A | A | C | 0.63 | 8.466328e-01 |  |
| 287 | 4 | A | A | G | 5.89 | 2.294454e-01 |  |
| 287 | 4 | A | A | T | 0.00 | 8.510638e-01 |  |
| 288 | 5 | C | C | A | 8.12 | 5.206489e-02 |  |
| 288 | 5 | C | C | C | 88.59 | 0.000000e+00 | \* |
| 288 | 5 | C | C | G | 0.00 | 8.426966e-01 |  |
| 288 | 5 | C | C | T | 3.29 | 3.899570e-01 |  |
| 289 | 6 | T | T | A | 0.00 | 8.230088e-01 |  |
| 289 | 6 | T | T | C | 6.98 | 9.589156e-02 |  |
| 289 | 6 | T | T | G | 4.07 | 3.702742e-01 |  |
| 289 | 6 | T | T | T | 88.95 | 8.881784e-16 | \* |
| 290 | 7 | G | G | A | 3.15 | 3.307187e-01 |  |
| 290 | 7 | G | G | C | 3.49 | 3.566970e-01 |  |
| 290 | 7 | G | G | G | 92.50 | 3.730349e-13 | \* |
| 290 | 7 | G | G | T | 0.85 | 7.530592e-01 |  |
| 291 | 8 | G | G | A | 3.80 | 2.628322e-01 |  |
| 291 | 8 | G | G | C | 3.93 | 3.052485e-01 |  |
| 291 | 8 | G | G | G | 90.75 | 6.562528e-13 | \* |
| 291 | 8 | G | G | T | 1.52 | 6.450720e-01 |  |
| 292 | 9 | C | C | A | 8.23 | 5.006576e-02 |  |
| 292 | 9 | C | C | C | 78.57 | 1.998401e-15 | \* |
| 292 | 9 | C | C | G | 9.74 | 7.782975e-02 |  |
| 292 | 9 | C | C | T | 3.46 | 3.693330e-01 |  |
| 293 | 10 | C | C | A | 6.85 | 8.477536e-02 |  |
| 293 | 10 | C | C | C | 87.55 | 0.000000e+00 | \* |
| 293 | 10 | C | C | G | 3.50 | 4.275667e-01 |  |
| 293 | 10 | C | C | T | 2.10 | 5.535495e-01 |  |
| 294 | 11 | A | A | A | 83.47 | 1.665335e-15 | \* |
| 294 | 11 | A | A | C | 8.60 | 5.023490e-02 |  |
| 294 | 11 | A | A | G | 3.47 | 4.302606e-01 |  |
| 294 | 11 | A | A | T | 4.46 | 2.680817e-01 |  |
| 295 | 12 | G | G | A | 10.83 | 1.809346e-02 |  |
| 295 | 12 | G | G | C | 0.00 | 9.268293e-01 |  |
| 295 | 12 | G | G | G | 87.55 | 1.836975e-12 | \* |
| 295 | 12 | G | G | T | 1.62 | 6.282700e-01 |  |
| 296 | 13 | A | A | A | 92.22 | 0.000000e+00 | \* |
| 296 | 13 | A | A | C | 0.85 | 8.079122e-01 |  |
| 296 | 13 | A | A | G | 3.97 | 3.794903e-01 |  |
| 296 | 13 | A | A | T | 2.96 | 4.312391e-01 |  |
| 297 | 14 | C | C | A | 0.00 | 8.230088e-01 |  |
| 297 | 14 | C | C | C | 92.84 | 0.000000e+00 | \* |
| 297 | 14 | C | C | G | 0.00 | 8.426966e-01 |  |
| 297 | 14 | C | C | T | 7.16 | 1.067834e-01 |  |
| 298 | 15 | C | C | A | 0.00 | 8.230088e-01 |  |
| 298 | 15 | C | C | C | 84.98 | 1.110223e-16 | \* |
| 298 | 15 | C | C | G | 7.67 | 1.403879e-01 |  |
| 298 | 15 | C | C | T | 7.36 | 9.951034e-02 |  |
| 299 | 16 | G | G | A | 2.95 | 3.552424e-01 |  |
| 299 | 16 | G | G | C | 3.65 | 3.377426e-01 |  |
| 299 | 16 | G | G | G | 84.57 | 4.773737e-12 | \* |
| 299 | 16 | G | G | T | 8.84 | 5.855377e-02 |  |
| 300 | 17 | T | T | A | 4.74 | 1.869365e-01 |  |
| 300 | 17 | T | T | C | 0.21 | 9.113325e-01 |  |
| 300 | 17 | T | T | G | 6.60 | 1.890179e-01 |  |
| 300 | 17 | T | T | T | 88.45 | 9.992007e-16 | \* |
| 301 | 18 | G | G | A | 1.02 | 6.585142e-01 |  |
| 301 | 18 | G | G | C | 0.10 | 9.215664e-01 |  |
| 301 | 18 | G | G | G | 94.41 | 2.020606e-13 | \* |
| 301 | 18 | G | G | T | 4.47 | 2.673147e-01 |  |
| 302 | 19 | T | T | A | 1.66 | 5.437266e-01 |  |
| 302 | 19 | T | T | C | 0.33 | 8.952913e-01 |  |
| 302 | 19 | T | T | G | 11.11 | 5.227436e-02 |  |
| 302 | 19 | T | T | T | 86.90 | 1.998401e-15 | \* |
| 303 | 20 | T | T | A | 0.71 | 7.158662e-01 |  |
| 303 | 20 | T | T | C | 0.00 | 9.268293e-01 |  |
| 303 | 20 | T | T | G | 0.00 | 8.426966e-01 |  |
| 303 | 20 | T | T | T | 99.29 | 0.000000e+00 | \* |
| 304 | 21 | T | T | A | 0.68 | 7.207203e-01 |  |
| 304 | 21 | T | T | C | 0.00 | 9.268293e-01 |  |
| 304 | 21 | T | T | G | 3.06 | 4.754582e-01 |  |
| 304 | 21 | T | T | T | 96.26 | 0.000000e+00 | \* |
| 305 | 22 | G | G | A | 1.51 | 5.693121e-01 |  |
| 305 | 22 | G | G | C | 2.79 | 4.547711e-01 |  |
| 305 | 22 | G | G | G | 93.26 | 2.922107e-13 | \* |
| 305 | 22 | G | G | T | 2.44 | 5.026860e-01 |  |
| 306 | 23 | T | T | A | 2.08 | 4.758933e-01 |  |
| 306 | 23 | T | T | C | 2.83 | 4.482795e-01 |  |
| 306 | 23 | T | T | G | 4.15 | 3.626894e-01 |  |
| 306 | 23 | T | T | T | 90.94 | 3.330669e-16 | \* |
| 307 | 24 | G | G | A | 0.43 | 7.649622e-01 |  |
| 307 | 24 | G | G | C | 4.32 | 2.645045e-01 |  |
| 307 | 24 | G | G | G | 95.24 | 1.544320e-13 | \* |
| 307 | 24 | G | G | T | 0.00 | 8.510638e-01 |  |
| 308 | 25 | C | C | A | 0.90 | 6.802160e-01 |  |
| 308 | 25 | C | C | C | 89.21 | 0.000000e+00 | \* |
| 308 | 25 | C | C | G | 0.00 | 8.426966e-01 |  |
| 308 | 25 | C | C | T | 9.89 | 3.985706e-02 |  |
| 309 | 26 | A | A | A | 83.24 | 1.776357e-15 | \* |
| 309 | 26 | A | A | C | 2.89 | 4.392866e-01 |  |
| 309 | 26 | A | A | G | 7.90 | 1.315418e-01 |  |
| 309 | 26 | A | A | T | 5.97 | 1.612631e-01 |  |
| 310 | 27 | C | C | A | 4.62 | 1.956595e-01 |  |
| 310 | 27 | C | C | C | 92.04 | 0.000000e+00 | \* |
| 310 | 27 | C | C | G | 0.00 | 8.426966e-01 |  |
| 310 | 27 | C | C | T | 3.34 | 3.832901e-01 |  |
| 311 | 28 | T | T | A | 4.45 | 2.081894e-01 |  |
| 311 | 28 | T | T | C | 7.21 | 8.749693e-02 |  |
| 311 | 28 | T | T | G | 0.00 | 8.426966e-01 |  |
| 311 | 28 | T | T | T | 88.34 | 1.110223e-15 | \* |
| 312 | 29 | A | A | A | 98.00 | 0.000000e+00 | \* |
| 312 | 29 | A | A | C | 0.00 | 9.268293e-01 |  |
| 312 | 29 | A | A | G | 0.00 | 8.426966e-01 |  |
| 312 | 29 | A | A | T | 2.00 | 5.692773e-01 |  |
| 313 | 30 | C | C | A | 1.67 | 5.417945e-01 |  |
| 313 | 30 | C | C | C | 89.05 | 0.000000e+00 | \* |
| 313 | 30 | C | C | G | 4.82 | 3.047002e-01 |  |
| 313 | 30 | C | C | T | 4.45 | 2.689726e-01 |  |
| 314 | 31 | A | A | A | 84.75 | 8.881784e-16 | \* |
| 314 | 31 | A | A | C | 3.14 | 4.034714e-01 |  |
| 314 | 31 | A | A | G | 8.97 | 9.713092e-02 |  |
| 314 | 31 | A | A | T | 3.14 | 4.082466e-01 |  |
| 315 | 32 | C | C | A | 1.54 | 5.648459e-01 |  |
| 315 | 32 | C | C | C | 91.94 | 0.000000e+00 | \* |
| 315 | 32 | C | C | G | 6.33 | 2.032103e-01 |  |
| 315 | 32 | C | C | T | 0.19 | 8.397101e-01 |  |
| 316 | 33 | G | G | A | 1.35 | 5.973494e-01 |  |
| 316 | 33 | G | G | C | 5.41 | 1.764056e-01 |  |
| 316 | 33 | G | G | G | 89.41 | 1.007416e-12 | \* |
| 316 | 33 | G | G | T | 3.83 | 3.290972e-01 |  |
| 317 | 34 | G | G | A | 3.18 | 3.272550e-01 |  |
| 317 | 34 | G | G | C | 0.00 | 9.268293e-01 |  |
| 317 | 34 | G | G | G | 95.76 | 1.310063e-13 | \* |
| 317 | 34 | G | G | T | 1.06 | 7.199751e-01 |  |
| 318 | 35 | G | G | A | 3.47 | 2.959559e-01 |  |
| 318 | 35 | G | G | C | 4.82 | 2.202567e-01 |  |
| 318 | 35 | G | G | G | 89.60 | 9.504619e-13 | \* |
| 318 | 35 | G | G | T | 2.12 | 5.502513e-01 |  |
| 319 | 36 | C | C | A | 1.95 | 4.952778e-01 |  |
| 319 | 36 | C | C | C | 86.33 | 1.110223e-16 | \* |
| 319 | 36 | C | C | G | 4.77 | 3.088403e-01 |  |
| 319 | 36 | C | C | T | 6.94 | 1.151477e-01 |  |
| 320 | 37 | A | A | A | 89.74 | 1.110223e-16 | \* |
| 320 | 37 | A | A | C | 3.22 | 3.924239e-01 |  |
| 320 | 37 | A | A | G | 0.00 | 8.426966e-01 |  |
| 320 | 37 | A | A | T | 7.04 | 1.111364e-01 |  |
| 321 | 38 | C | C | A | 0.00 | 8.230088e-01 |  |
| 321 | 38 | C | C | C | 91.07 | 0.000000e+00 | \* |
| 321 | 38 | C | C | G | 0.96 | 7.466067e-01 |  |
| 321 | 38 | C | C | T | 7.97 | 7.983018e-02 |  |
| 322 | 39 | C | C | A | 4.06 | 2.392774e-01 |  |
| 322 | 39 | C | C | C | 88.97 | 0.000000e+00 | \* |
| 322 | 39 | C | C | G | 2.76 | 5.110521e-01 |  |
| 322 | 39 | C | C | T | 4.21 | 2.912235e-01 |  |
| 323 | 40 | C | C | A | 1.87 | 5.086703e-01 |  |
| 323 | 40 | C | C | C | 92.21 | 0.000000e+00 | \* |
| 323 | 40 | C | C | G | 1.87 | 6.241927e-01 |  |
| 323 | 40 | C | C | T | 4.05 | 3.065999e-01 |  |
| 324 | 41 | T | T | A | 0.65 | 7.261820e-01 |  |
| 324 | 41 | T | T | C | 5.85 | 1.485838e-01 |  |
| 324 | 41 | T | T | G | 2.11 | 5.919028e-01 |  |
| 324 | 41 | T | T | T | 91.38 | 3.330669e-16 | \* |
| 325 | 42 | G | G | A | 4.55 | 2.005074e-01 |  |
| 325 | 42 | G | G | C | 0.72 | 8.314565e-01 |  |
| 325 | 42 | G | G | G | 94.73 | 1.822986e-13 | \* |
| 325 | 42 | G | G | T | 0.00 | 8.510638e-01 |  |
| 326 | 43 | A | A | A | 90.36 | 1.110223e-16 | \* |
| 326 | 43 | A | A | C | 0.66 | 8.425310e-01 |  |
| 326 | 43 | A | A | G | 7.45 | 1.493193e-01 |  |
| 326 | 43 | A | A | T | 1.53 | 6.430413e-01 |  |
| 327 | 44 | C | C | A | 3.98 | 2.461836e-01 |  |
| 327 | 44 | C | C | C | 91.65 | 0.000000e+00 | \* |
| 327 | 44 | C | C | G | 0.00 | 8.426966e-01 |  |
| 327 | 44 | C | C | T | 4.36 | 2.768634e-01 |  |
| 328 | 45 | C | C | A | 0.00 | 8.230088e-01 |  |
| 328 | 45 | C | C | C | 100.00 | 0.000000e+00 | \* |
| 328 | 45 | C | C | G | 0.00 | 8.426966e-01 |  |
| 328 | 45 | C | C | T | 0.00 | 8.510638e-01 |  |
| 329 | 46 | G | G | A | 2.33 | 4.376277e-01 |  |
| 329 | 46 | G | G | C | 4.08 | 2.892619e-01 |  |
| 329 | 46 | G | G | G | 93.59 | 2.629008e-13 | \* |
| 329 | 46 | G | G | T | 0.00 | 8.510638e-01 |  |
| 330 | 47 | A | A | A | 93.80 | 0.000000e+00 | \* |
| 330 | 47 | A | A | C | 0.32 | 8.975900e-01 |  |
| 330 | 47 | A | A | G | 2.63 | 5.270103e-01 |  |
| 330 | 47 | A | A | T | 3.26 | 3.938127e-01 |  |
| 331 | 48 | C | C | A | 0.00 | 8.230088e-01 |  |
| 331 | 48 | C | C | C | 92.97 | 0.000000e+00 | \* |
| 331 | 48 | C | C | G | 0.57 | 7.945852e-01 |  |
| 331 | 48 | C | C | T | 6.46 | 1.360746e-01 |  |
| 332 | 49 | G | G | A | 7.66 | 6.236164e-02 |  |
| 332 | 49 | G | G | C | 2.39 | 5.181204e-01 |  |
| 332 | 49 | G | G | G | 89.95 | 8.474332e-13 | \* |
| 332 | 49 | G | G | T | 0.00 | 8.510638e-01 |  |
| 333 | 50 | G | G | A | 3.24 | 3.211991e-01 |  |
| 333 | 50 | G | G | C | 0.59 | 8.546392e-01 |  |
| 333 | 50 | G | G | G | 93.97 | 2.328138e-13 | \* |
| 333 | 50 | G | G | T | 2.21 | 5.371385e-01 |  |
| 334 | 51 | C | C | A | 7.73 | 6.048833e-02 |  |
| 334 | 51 | C | C | C | 86.19 | 1.110223e-16 | \* |
| 334 | 51 | C | C | G | 3.87 | 3.897492e-01 |  |
| 334 | 51 | C | C | T | 2.21 | 5.365265e-01 |  |
| 335 | 52 | A | A | A | 91.82 | 1.110223e-16 | \* |
| 335 | 52 | A | A | C | 0.00 | 9.268293e-01 |  |
| 335 | 52 | A | A | G | 0.91 | 7.528181e-01 |  |
| 335 | 52 | A | A | T | 7.27 | 1.024580e-01 |  |
| 336 | 53 | A | A | A | 90.10 | 1.110223e-16 | \* |
| 336 | 53 | A | A | C | 4.17 | 2.800456e-01 |  |
| 336 | 53 | A | A | G | 0.52 | 8.002287e-01 |  |
| 336 | 53 | A | A | T | 5.21 | 2.092232e-01 |  |
| 337 | 54 | G | G | A | 5.12 | 1.628609e-01 |  |
| 337 | 54 | G | G | C | 0.00 | 9.268293e-01 |  |
| 337 | 54 | G | G | G | 94.88 | 1.735279e-13 | \* |
| 337 | 54 | G | G | T | 0.00 | 8.510638e-01 |  |
| 338 | 55 | A | A | A | 91.19 | 1.110223e-16 | \* |
| 338 | 55 | A | A | C | 1.63 | 6.565227e-01 |  |
| 338 | 55 | A | A | G | 4.20 | 3.581187e-01 |  |
| 338 | 55 | A | A | T | 2.98 | 4.283264e-01 |  |
| 339 | 56 | A | A | A | 96.49 | 0.000000e+00 | \* |
| 339 | 56 | A | A | C | 3.09 | 4.099440e-01 |  |
| 339 | 56 | A | A | G | 0.00 | 8.426966e-01 |  |
| 339 | 56 | A | A | T | 0.41 | 8.157562e-01 |  |
| 340 | 57 | G | G | A | 6.07 | 1.141127e-01 |  |
| 340 | 57 | G | G | C | 4.75 | 2.258872e-01 |  |
| 340 | 57 | G | G | G | 87.60 | 1.805445e-12 | \* |
| 340 | 57 | G | G | T | 1.58 | 6.349752e-01 |  |
| 341 | 58 | T | T | A | 0.23 | 7.981154e-01 |  |
| 341 | 58 | T | T | C | 6.55 | 1.135544e-01 |  |
| 341 | 58 | T | T | G | 0.00 | 8.426966e-01 |  |
| 341 | 58 | T | T | T | 93.23 | 2.220446e-16 | \* |
| 342 | 59 | T | T | A | 1.05 | 6.530078e-01 |  |
| 342 | 59 | T | T | C | 1.05 | 7.693949e-01 |  |
| 342 | 59 | T | T | G | 3.97 | 3.793033e-01 |  |
| 342 | 59 | T | T | T | 93.93 | 1.110223e-16 | \* |
| 343 | 60 | C | C | A | 5.87 | 1.229044e-01 |  |
| 343 | 60 | C | C | C | 91.67 | 0.000000e+00 | \* |
| 343 | 60 | C | C | G | 2.46 | 5.473203e-01 |  |
| 343 | 60 | C | C | T | 0.00 | 8.510638e-01 |  |
| 344 | 61 | G | G | A | 4.52 | 2.025009e-01 |  |
| 344 | 61 | G | G | C | 4.05 | 2.924335e-01 |  |
| 344 | 61 | G | G | G | 91.19 | 5.692113e-13 | \* |
| 344 | 61 | G | G | T | 0.24 | 8.353719e-01 |  |
| 345 | 62 | A | A | A | 96.44 | 0.000000e+00 | \* |
| 345 | 62 | A | A | C | 0.48 | 8.735728e-01 |  |
| 345 | 62 | A | A | G | 1.31 | 7.000635e-01 |  |
| 345 | 62 | A | A | T | 1.78 | 6.030720e-01 |  |
| 346 | 63 | C | C | A | 2.74 | 3.815301e-01 |  |
| 346 | 63 | C | C | C | 95.44 | 0.000000e+00 | \* |
| 346 | 63 | C | C | G | 1.28 | 7.039811e-01 |  |
| 346 | 63 | C | C | T | 0.55 | 7.979988e-01 |  |
| 347 | 64 | A | A | A | 87.45 | 2.220446e-16 | \* |
| 347 | 64 | A | A | C | 6.60 | 1.113775e-01 |  |
| 347 | 64 | A | A | G | 2.98 | 4.849563e-01 |  |
| 347 | 64 | A | A | T | 2.98 | 4.286249e-01 |  |
| 348 | 65 | G | G | A | 3.16 | 3.294497e-01 |  |
| 348 | 65 | G | G | C | 2.26 | 5.408148e-01 |  |
| 348 | 65 | G | G | G | 93.37 | 2.821077e-13 | \* |
| 348 | 65 | G | G | T | 1.20 | 6.966196e-01 |  |
| 349 | 66 | C | C | A | 0.20 | 8.024712e-01 |  |
| 349 | 66 | C | C | C | 89.84 | 0.000000e+00 | \* |
| 349 | 66 | C | C | G | 4.10 | 3.672879e-01 |  |
| 349 | 66 | C | C | T | 5.86 | 1.676830e-01 |  |
| 350 | 67 | T | T | A | 2.27 | 4.460357e-01 |  |
| 350 | 67 | T | T | C | 5.54 | 1.675949e-01 |  |
| 350 | 67 | T | T | G | 0.14 | 8.360024e-01 |  |
| 350 | 67 | T | T | T | 92.05 | 3.330669e-16 | \* |
| 351 | 68 | C | C | A | 0.18 | 8.048725e-01 |  |
| 351 | 68 | C | C | C | 92.70 | 0.000000e+00 | \* |
| 351 | 68 | C | C | G | 4.09 | 3.681346e-01 |  |
| 351 | 68 | C | C | T | 3.02 | 4.226757e-01 |  |
| 352 | 69 | C | C | A | 2.52 | 4.100566e-01 |  |
| 352 | 69 | C | C | C | 92.01 | 0.000000e+00 | \* |
| 352 | 69 | C | C | G | 0.00 | 8.426966e-01 |  |
| 352 | 69 | C | C | T | 5.47 | 1.915198e-01 |  |
| 353 | 70 | C | C | A | 4.09 | 2.368169e-01 |  |
| 353 | 70 | C | C | C | 90.39 | 0.000000e+00 | \* |
| 353 | 70 | C | C | G | 1.78 | 6.361984e-01 |  |
| 353 | 70 | C | C | T | 3.74 | 3.388816e-01 |  |
| 354 | 71 | G | G | A | 2.51 | 4.120055e-01 |  |
| 354 | 71 | G | G | C | 2.93 | 4.335593e-01 |  |
| 354 | 71 | G | G | G | 90.59 | 6.913359e-13 | \* |
| 354 | 71 | G | G | T | 3.97 | 3.140782e-01 |  |
| 355 | 72 | C | C | A | 2.25 | 4.487928e-01 |  |
| 355 | 72 | C | C | C | 91.60 | 0.000000e+00 | \* |
| 355 | 72 | C | C | G | 6.15 | 2.138169e-01 |  |
| 355 | 72 | C | C | T | 0.00 | 8.510638e-01 |  |
| 356 | 73 | G | G | A | 2.52 | 4.112764e-01 |  |
| 356 | 73 | G | G | C | 6.29 | 1.255262e-01 |  |
| 356 | 73 | G | G | G | 88.05 | 1.561751e-12 | \* |
| 356 | 73 | G | G | T | 3.14 | 4.075432e-01 |  |
| 357 | 74 | A | A | A | 95.44 | 0.000000e+00 | \* |
| 357 | 74 | A | A | C | 0.69 | 8.365305e-01 |  |
| 357 | 74 | A | A | G | 0.00 | 8.426966e-01 |  |
| 357 | 74 | A | A | T | 3.87 | 3.250732e-01 |  |
| 358 | 75 | C | C | A | 0.00 | 8.230088e-01 |  |
| 358 | 75 | C | C | C | 92.96 | 0.000000e+00 | \* |
| 358 | 75 | C | C | G | 0.00 | 8.426966e-01 |  |
| 358 | 75 | C | C | T | 7.04 | 1.110417e-01 |  |

## For use in R

If you want to work with the results in R, here is output that you can copy and paste in your terminal to get:

The base information:

```
structure(list(focal.base = c("A", "C", "G", "T"), avg.percsignal = c(90.1594766545967, 
90.2465488576281, 91.0625874435175, 91.0928523041834), avg.areasignal = c(598.58064516129, 
558.971014492754, 595.931034482759, 604.627906976744), crit.perc.area = c(12.32874936516, 
12.5116809253263, 16.6664205726334, 13.6070278223138), mu = c(3.25880740045274, 
3.42595133510131, 4.52544547615018, 3.77373281073774), fillibens = c(0.99724044599022, 
0.989311892967993, 0.988986572684175, 0.990927921503207)), .Names = c("focal.base", 
"avg.percsignal", "avg.areasignal", "crit.perc.area", "mu", "fillibens"
), row.names = c(NA, -4L), class = "data.frame")
```

the data.frame that contains information on the guide region:

```
structure(list(A.area = c(552, 605, 47, 1032, 42, 0, 37, 30, 
38, 49, 505, 60, 1091, 0, 0, 21, 23, 10, 10, 4, 4, 13, 11, 3, 
5, 432, 29, 29, 491, 9, 378, 8, 6, 21, 18, 9, 446, 0, 28, 12, 
4, 38, 825, 21, 0, 12, 893, 0, 32, 22, 28, 303, 519, 22, 673, 
468, 23, 1, 5, 31, 19, 812, 15, 411, 21, 1, 16, 1, 18, 23, 12, 
11, 12, 691, 0), C.area = c(27, 31, 7, 7, 458, 48, 41, 31, 363, 
626, 52, 0, 10, 519, 543, 26, 1, 1, 2, 0, 0, 24, 15, 30, 496, 
15, 578, 47, 0, 480, 14, 479, 24, 0, 25, 398, 16, 571, 613, 592, 
36, 6, 6, 483, 533, 21, 3, 489, 10, 4, 312, 0, 24, 0, 12, 15, 
18, 29, 5, 484, 17, 4, 523, 31, 15, 460, 39, 521, 656, 508, 14, 
447, 30, 5, 541), G.area = c(28, 13, 475, 65, 0, 28, 1086, 716, 
45, 25, 21, 485, 47, 0, 49, 603, 32, 929, 67, 0, 18, 803, 22, 
661, 0, 41, 0, 0, 0, 26, 40, 33, 397, 632, 465, 22, 0, 6, 19, 
12, 13, 791, 68, 0, 0, 482, 25, 3, 376, 639, 14, 3, 3, 408, 31, 
0, 332, 0, 19, 13, 383, 11, 7, 14, 620, 21, 1, 23, 0, 10, 433, 
30, 420, 0, 0), T.area = c(25, 0, 28, 0, 17, 612, 10, 12, 16, 
15, 27, 9, 35, 40, 47, 63, 429, 44, 524, 562, 566, 21, 482, 0, 
55, 31, 21, 576, 10, 24, 14, 1, 17, 7, 11, 32, 35, 50, 29, 26, 
562, 0, 14, 23, 0, 0, 31, 34, 0, 15, 8, 24, 30, 0, 22, 2, 6, 
413, 449, 0, 1, 15, 3, 14, 8, 30, 648, 17, 39, 21, 19, 0, 15, 
28, 41), Tot.area = c(632, 649, 557, 1104, 517, 688, 1174, 789, 
462, 715, 605, 554, 1183, 559, 639, 713, 485, 984, 603, 566, 
588, 861, 530, 694, 556, 519, 628, 652, 501, 539, 446, 521, 444, 
660, 519, 461, 497, 627, 689, 642, 615, 835, 913, 527, 533, 515, 
952, 526, 418, 680, 362, 330, 576, 430, 738, 485, 379, 443, 478, 
528, 420, 842, 548, 470, 664, 512, 704, 562, 713, 562, 478, 488, 
477, 724, 582), A.perc = c(87.3417721518987, 93.2203389830509, 
8.43806104129264, 93.4782608695652, 8.12379110251451, 0, 3.15161839863714, 
3.80228136882129, 8.22510822510823, 6.85314685314685, 83.4710743801653, 
10.8303249097473, 92.2231614539307, 0, 0, 2.945301542777, 4.74226804123711, 
1.01626016260163, 1.65837479270315, 0.706713780918728, 0.680272108843537, 
1.50987224157956, 2.07547169811321, 0.432276657060519, 0.899280575539568, 
83.2369942196532, 4.61783439490446, 4.4478527607362, 98.0039920159681, 
1.66975881261596, 84.7533632286996, 1.53550863723608, 1.35135135135135, 
3.18181818181818, 3.46820809248555, 1.95227765726681, 89.738430583501, 
0, 4.06386066763425, 1.86915887850467, 0.650406504065041, 4.55089820359281, 
90.3614457831325, 3.98481973434535, 0, 2.33009708737864, 93.8025210084034, 
0, 7.6555023923445, 3.23529411764706, 7.73480662983425, 91.8181818181818, 
90.1041666666667, 5.11627906976744, 91.1924119241192, 96.4948453608247, 
6.06860158311346, 0.225733634311512, 1.04602510460251, 5.87121212121212, 
4.52380952380952, 96.437054631829, 2.73722627737226, 87.4468085106383, 
3.16265060240964, 0.1953125, 2.27272727272727, 0.177935943060498, 
2.52454417952314, 4.09252669039146, 2.51046025104602, 2.25409836065574, 
2.51572327044025, 95.4419889502762, 0), C.perc = c(4.27215189873418, 
4.77657935285054, 1.25673249551167, 0.634057971014493, 88.5880077369439, 
6.97674418604651, 3.4923339011925, 3.92902408111534, 78.5714285714286, 
87.5524475524475, 8.59504132231405, 0, 0.84530853761623, 92.8443649373882, 
84.9765258215962, 3.64656381486676, 0.206185567010309, 0.101626016260163, 
0.33167495854063, 0, 0, 2.78745644599303, 2.83018867924528, 4.32276657060519, 
89.2086330935252, 2.89017341040462, 92.0382165605096, 7.20858895705521, 
0, 89.0538033395176, 3.1390134529148, 91.9385796545106, 5.40540540540541, 
0, 4.81695568400771, 86.3340563991323, 3.21931589537223, 91.0685805422648, 
88.9695210449927, 92.2118380062305, 5.85365853658537, 0.718562874251497, 
0.657174151150055, 91.6508538899431, 100, 4.07766990291262, 0.315126050420168, 
92.9657794676806, 2.39234449760766, 0.588235294117647, 86.1878453038674, 
0, 4.16666666666667, 0, 1.6260162601626, 3.09278350515464, 4.74934036939314, 
6.54627539503386, 1.04602510460251, 91.6666666666667, 4.04761904761905, 
0.475059382422803, 95.4379562043796, 6.59574468085106, 2.25903614457831, 
89.84375, 5.53977272727273, 92.7046263345196, 92.0056100981767, 
90.3914590747331, 2.92887029288703, 91.5983606557377, 6.28930817610063, 
0.69060773480663, 92.9553264604811), G.perc = c(4.43037974683544, 
2.00308166409861, 85.278276481149, 5.88768115942029, 0, 4.06976744186047, 
92.5042589437819, 90.7477820025348, 9.74025974025974, 3.4965034965035, 
3.47107438016529, 87.5451263537906, 3.97295012679628, 0, 7.66823161189358, 
84.5722300140253, 6.5979381443299, 94.4105691056911, 11.1111111111111, 
0, 3.06122448979592, 93.2636469221835, 4.15094339622642, 95.2449567723343, 
0, 7.89980732177264, 0, 0, 0, 4.82374768089054, 8.96860986547085, 
6.33397312859885, 89.4144144144144, 95.7575757575758, 89.5953757225434, 
4.77223427331887, 0, 0.956937799043062, 2.75761973875181, 1.86915887850467, 
2.11382113821138, 94.7305389221557, 7.44797371303395, 0, 0, 93.5922330097087, 
2.62605042016807, 0.570342205323194, 89.9521531100478, 93.9705882352941, 
3.86740331491713, 0.909090909090909, 0.520833333333333, 94.8837209302326, 
4.20054200542005, 0, 87.598944591029, 0, 3.97489539748954, 2.46212121212121, 
91.1904761904762, 1.30641330166271, 1.27737226277372, 2.97872340425532, 
93.3734939759036, 4.1015625, 0.142045454545455, 4.09252669039146, 
0, 1.77935943060498, 90.5857740585774, 6.14754098360656, 88.0503144654088, 
0, 0), T.perc = c(3.95569620253165, 0, 5.02692998204668, 0, 3.28820116054159, 
88.953488372093, 0.851788756388416, 1.52091254752852, 3.46320346320346, 
2.0979020979021, 4.46280991735537, 1.62454873646209, 2.9585798816568, 
7.15563506261181, 7.35524256651017, 8.835904628331, 88.4536082474227, 
4.47154471544715, 86.8988391376451, 99.2932862190813, 96.2585034013605, 
2.4390243902439, 90.9433962264151, 0, 9.89208633093525, 5.97302504816956, 
3.34394904458599, 88.3435582822086, 1.99600798403194, 4.45269016697588, 
3.1390134529148, 0.191938579654511, 3.82882882882883, 1.06060606060606, 
2.11946050096339, 6.941431670282, 7.04225352112676, 7.97448165869219, 
4.20899854862119, 4.04984423676012, 91.3821138211382, 0, 1.53340635268346, 
4.36432637571157, 0, 0, 3.2563025210084, 6.4638783269962, 0, 
2.20588235294118, 2.20994475138122, 7.27272727272727, 5.20833333333333, 
0, 2.9810298102981, 0.412371134020619, 1.58311345646438, 93.2279909706546, 
93.9330543933054, 0, 0.238095238095238, 1.78147268408551, 0.547445255474453, 
2.97872340425532, 1.20481927710843, 5.859375, 92.0454545454545, 
3.02491103202847, 5.46984572230014, 3.73665480427046, 3.97489539748954, 
0, 3.14465408805031, 3.86740331491713, 7.0446735395189), base.call = c("A", 
"A", "G", "A", "C", "T", "G", "G", "C", "C", "A", "G", "A", "C", 
"C", "G", "T", "G", "T", "T", "T", "G", "T", "G", "C", "A", "C", 
"T", "A", "C", "A", "C", "G", "G", "G", "C", "A", "C", "C", "C", 
"T", "G", "A", "C", "C", "G", "A", "C", "G", "G", "C", "A", "A", 
"G", "A", "A", "G", "T", "T", "C", "G", "A", "C", "A", "G", "C", 
"T", "C", "C", "C", "G", "C", "G", "A", "C"), index = 284:358, 
    guide.seq = c("A", "A", "G", "A", "C", "T", "G", "G", "C", 
    "C", "A", "G", "A", "C", "C", "G", "T", "G", "T", "T", "T", 
    "G", "T", "G", "C", "A", "C", "T", "A", "C", "A", "C", "G", 
    "G", "G", "C", "A", "C", "C", "C", "T", "G", "A", "C", "C", 
    "G", "A", "C", "G", "G", "C", "A", "A", "G", "A", "A", "G", 
    "T", "T", "C", "G", "A", "C", "A", "G", "C", "T", "C", "C", 
    "C", "G", "C", "G", "A", "C"), T.pval = c(0.316018567281549, 
    0.851063829787234, 0.22236262602344, 0.851063829787234, 0.389956998824095, 
    8.88178419700125e-16, 0.753059174493693, 0.645072043677006, 
    0.369333011730894, 0.553549462340053, 0.268081655727232, 
    0.628269992311271, 0.431239098667244, 0.106783440189537, 
    0.0995103428935635, 0.0585537724448312, 9.99200722162641e-16, 
    0.267314688156716, 1.99840144432528e-15, 0, 0, 0.502686032138853, 
    3.33066907387547e-16, 0.851063829787234, 0.0398570552375416, 
    0.161263139692945, 0.383290068239501, 1.11022302462516e-15, 
    0.569277304637538, 0.268972643583829, 0.408246564306132, 
    0.839710059215561, 0.329097160106182, 0.719975121863907, 
    0.550251309867583, 0.115147740809144, 0.111136382875449, 
    0.0798301786304834, 0.29122345685274, 0.306599878074449, 
    3.33066907387547e-16, 0.851063829787234, 0.643041291384758, 
    0.276863394402381, 0.851063829787234, 0.851063829787234, 
    0.393812716468189, 0.136074610825579, 0.851063829787234, 
    0.537138465544683, 0.536526450675504, 0.102457997786846, 
    0.209223178210324, 0.851063829787234, 0.428326387579897, 
    0.815756232107895, 0.634975181077186, 2.22044604925031e-16, 
    1.11022302462516e-16, 0.851063829787234, 0.83537185081873, 
    0.603071992602989, 0.797998791615971, 0.428624947794834, 
    0.696619581535735, 0.167683015362859, 3.33066907387547e-16, 
    0.422675684861227, 0.191519791801277, 0.338881550501321, 
    0.314078150454161, 0.851063829787234, 0.407543172874813, 
    0.32507321159853, 0.111041743294653), C.pval = c(0.269457756564916, 
    0.223603411047135, 0.728233547861078, 0.846632843707782, 
    0, 0.0958915575468675, 0.356696989786891, 0.305248515251967, 
    1.99840144432528e-15, 0, 0.0502349040390854, 0.926829268292683, 
    0.807912238909763, 0, 1.11022302462516e-16, 0.337742635804783, 
    0.911332467930666, 0.921566421059162, 0.895291288872233, 
    0.926829268292683, 0.926829268292683, 0.454771099091427, 
    0.448279463334394, 0.264504530475254, 0, 0.439286561731438, 
    0, 0.0874969327662178, 0.926829268292683, 0, 0.403471396836279, 
    0, 0.176405588367595, 0.926829268292683, 0.220256735646008, 
    1.11022302462516e-16, 0.392423852839802, 0, 0, 0, 0.148583762184612, 
    0.831456542881921, 0.842530967218928, 0, 0, 0.289261929222078, 
    0.897589990479022, 0, 0.518120387037294, 0.85463922467382, 
    1.11022302462516e-16, 0.926829268292683, 0.280045564077339, 
    0.926829268292683, 0.656522748038537, 0.409943952596328, 
    0.225887172203965, 0.113554438985447, 0.769394894125823, 
    0, 0.292433514171313, 0.873572775184238, 0, 0.111377510206685, 
    0.540814840569912, 0, 0.167594898018422, 0, 0, 0, 0.433559308433086, 
    0, 0.12552617675351, 0.836530517561569, 0), G.pval = c(0.337561198706174, 
    0.606432895160107, 3.80517839460026e-12, 0.229445372520921, 
    0.842696629213482, 0.370274172740719, 3.73034936274053e-13, 
    6.5625282985593e-13, 0.0778297498821698, 0.427566650469482, 
    0.430260552587122, 1.83697501654478e-12, 0.379490264452726, 
    0.842696629213482, 0.140387932507101, 4.77373696128325e-12, 
    0.189017890372664, 2.02060590481778e-13, 0.052274360740427, 
    0.842696629213482, 0.475458211028236, 2.92210700081341e-13, 
    0.36268941612591, 1.54432022725359e-13, 0.842696629213482, 
    0.131541760682923, 0.842696629213482, 0.842696629213482, 
    0.842696629213482, 0.304700234212886, 0.0971309213666836, 
    0.203210271562139, 1.00741637254487e-12, 1.31006316905768e-13, 
    9.50461931381597e-13, 0.308840347696373, 0.842696629213482, 
    0.746606704202121, 0.511052090032846, 0.624192699454656, 
    0.59190275005678, 1.82298620643451e-13, 0.149319273766449, 
    0.842696629213482, 0.842696629213482, 2.62900812231237e-13, 
    0.527010290190403, 0.794585236678228, 8.47433234696382e-13, 
    2.32813768263895e-13, 0.389749151228092, 0.752818058147437, 
    0.800228680047243, 1.73527858748912e-13, 0.358118697250609, 
    0.842696629213482, 1.80544468264543e-12, 0.842696629213482, 
    0.379303266666027, 0.547320311182555, 5.69211344725318e-13, 
    0.700063463554174, 0.703981055924309, 0.484956276800356, 
    2.82107670557252e-13, 0.367287940481951, 0.836002352230184, 
    0.368134571331529, 0.842696629213482, 0.636198420328112, 
    6.91335877434085e-13, 0.213816923557072, 1.56175072874021e-12, 
    0.842696629213482, 0.842696629213482), A.pval = c(3.33066907387547e-16, 
    0, 0.0461050451899198, 0, 0.0520648866715256, 0.823008849557521, 
    0.330718738746457, 0.262832169092152, 0.0500657627159223, 
    0.0847753630630367, 1.66533453693773e-15, 0.0180934642371888, 
    0, 0.823008849557521, 0.823008849557521, 0.355242383111475, 
    0.186936484115165, 0.658514214190127, 0.543726592229574, 
    0.715866209436449, 0.720720312200124, 0.569312091624093, 
    0.475893333893807, 0.764962182342818, 0.680215990898375, 
    1.77635683940025e-15, 0.195659458759314, 0.208189393266996, 
    0, 0.541794522453721, 8.88178419700125e-16, 0.564845924352552, 
    0.597349389533367, 0.327254963351471, 0.295955893115715, 
    0.495277769427836, 1.11022302462516e-16, 0.823008849557521, 
    0.239277409357523, 0.508670289399838, 0.726182024948532, 
    0.200507376565831, 1.11022302462516e-16, 0.246183562557149, 
    0.823008849557521, 0.437627654552293, 0, 0.823008849557521, 
    0.0623616445358152, 0.321199073497741, 0.0604883308449139, 
    1.11022302462516e-16, 1.11022302462516e-16, 0.162860871573992, 
    1.11022302462516e-16, 0, 0.114112655280202, 0.798115351236418, 
    0.653007783268923, 0.122904420068554, 0.202500893696655, 
    0, 0.381530057611429, 2.22044604925031e-16, 0.329449715010277, 
    0.802471180265174, 0.446035749707479, 0.804872472991491, 
    0.41005657153464, 0.236816853628295, 0.41200554431906, 0.44879279039757, 
    0.411276357723768, 0, 0.823008849557521), guide.position = 1:75), .Names = c("A.area", 
"C.area", "G.area", "T.area", "Tot.area", "A.perc", "C.perc", 
"G.perc", "T.perc", "base.call", "index", "guide.seq", "T.pval", 
"C.pval", "G.pval", "A.pval", "guide.position"), row.names = 284:358, class = "data.frame")
```

*Report generated using EditR v1.0.8*
