# Supplementary material for: Mechanistic and genetic basis of single-strand templated repair at Cas12a-induced DNA breaks in Chlamydomonas reinhardtii
Source: Nat Commun. 2021 Nov 19;12:6751. doi: 10.1038/s41467-021-27004-1 (PMC8604939; doi:10.1038/s41467-021-27004-1)
Supplement: Supplementary file 22 — Source Data [file 41467_2021_27004_MOESM22_ESM.zip › Source Data/EditR analysis/EditR outputs/Controls for normalization/Positive control (0).html]

EditR v1.0.8 report


# EditR v1.0.8 report

- Data QA
  - Filtering data
  - Percent noise peak area
  - Base information
- Predicted editing
  - Editing bar plot
  - Editing table plot
  - Table of editing results
- For use in R

## Data QA

### Filtering data

What the data looked like prefiltering:

and the post filtering signal / noise plot:

### Percent noise peak area

### Base information

Here’s information about the signal of each base, the critical percent value where any higher value would be called as significant, and Filliben’s correlation for how well the noise was modelled by the zero adjusted gamma distribution.

| Base | Average percent signal | Average peak area | Critical percent value | model mu | Fillibens correlation |
| --- | --- | --- | --- | --- | --- |
| A | 79.61876 | 351.0172 | 40.61559 | 7.996093 | 0.9904288 |
| C | 79.94352 | 305.2676 | 62.97893 | 13.049721 | 0.9939919 |
| G | 79.15105 | 353.7968 | 57.07208 | 11.421423 | 0.9937731 |
| T | 79.92043 | 318.9528 | 51.33390 | 10.106529 | 0.9923358 |

## Predicted editing

### Editing bar plot

### Editing table plot

### Table of editing results


Here’s the entire guide region

| Sanger position | Guide position | Guide sequence | Sanger base call | Focal base | Focal base peak area | p value |  |
| --- | --- | --- | --- | --- | --- | --- | --- |
| 281 | 1 | A | A | A | 79.41 | 3.110157e-04 | \* |
| 281 | 1 | A | A | C | 0.42 | 6.244511e-01 |  |
| 281 | 1 | A | A | G | 2.94 | 4.459256e-01 |  |
| 281 | 1 | A | A | T | 17.23 | 1.211923e-01 |  |
| 282 | 2 | A | A | A | 87.29 | 1.552882e-04 | \* |
| 282 | 2 | A | A | C | 12.37 | 2.439827e-01 |  |
| 282 | 2 | A | A | G | 0.33 | 6.001474e-01 |  |
| 282 | 2 | A | A | T | 0.00 | 6.343612e-01 |  |
| 283 | 3 | G | G | A | 5.14 | 3.124332e-01 |  |
| 283 | 3 | G | G | C | 7.51 | 3.440501e-01 |  |
| 283 | 3 | G | G | G | 87.35 | 1.441455e-03 | \* |
| 283 | 3 | G | G | T | 0.00 | 6.343612e-01 |  |
| 284 | 4 | A | A | A | 95.61 | 7.473222e-05 | \* |
| 284 | 4 | A | A | C | 0.70 | 6.042415e-01 |  |
| 284 | 4 | A | A | G | 0.88 | 5.559154e-01 |  |
| 284 | 4 | A | A | T | 2.81 | 4.201854e-01 |  |
| 285 | 5 | C | C | A | 0.82 | 5.499259e-01 |  |
| 285 | 5 | C | C | C | 83.01 | 2.980107e-03 | \* |
| 285 | 5 | C | C | G | 15.34 | 1.660136e-01 |  |
| 285 | 5 | C | C | T | 0.82 | 5.340553e-01 |  |
| 286 | 6 | T | T | A | 4.41 | 3.399710e-01 |  |
| 286 | 6 | T | T | C | 8.82 | 3.128844e-01 |  |
| 286 | 6 | T | T | G | 2.06 | 4.866595e-01 |  |
| 286 | 6 | T | T | T | 84.71 | 9.851503e-04 | \* |
| 287 | 7 | G | G | A | 0.95 | 5.386284e-01 |  |
| 287 | 7 | G | G | C | 0.16 | 6.472067e-01 |  |
| 287 | 7 | G | G | G | 95.90 | 8.395229e-04 | \* |
| 287 | 7 | G | G | T | 3.00 | 4.118063e-01 |  |
| 288 | 8 | G | G | A | 1.71 | 4.795796e-01 |  |
| 288 | 8 | G | G | C | 4.16 | 4.437359e-01 |  |
| 288 | 8 | G | G | G | 94.13 | 9.386303e-04 | \* |
| 288 | 8 | G | G | T | 0.00 | 6.343612e-01 |  |
| 289 | 9 | C | C | A | 11.36 | 1.602396e-01 |  |
| 289 | 9 | C | C | C | 78.25 | 3.970234e-03 | \* |
| 289 | 9 | C | C | G | 9.74 | 2.522626e-01 |  |
| 289 | 9 | C | C | T | 0.65 | 5.480895e-01 |  |
| 290 | 10 | C | C | A | 0.00 | 6.680942e-01 |  |
| 290 | 10 | C | C | C | 93.88 | 1.552792e-03 | \* |
| 290 | 10 | C | C | G | 0.53 | 5.823082e-01 |  |
| 290 | 10 | C | C | T | 5.59 | 3.194684e-01 |  |
| 291 | 11 | A | A | A | 81.38 | 2.614983e-04 | \* |
| 291 | 11 | A | A | C | 8.91 | 3.110248e-01 |  |
| 291 | 11 | A | A | G | 9.72 | 2.527241e-01 |  |
| 291 | 11 | A | A | T | 0.00 | 6.343612e-01 |  |
| 292 | 12 | G | G | A | 5.84 | 2.886169e-01 |  |
| 292 | 12 | G | G | C | 0.39 | 6.268828e-01 |  |
| 292 | 12 | G | G | G | 85.99 | 1.571197e-03 | \* |
| 292 | 12 | G | G | T | 7.78 | 2.622291e-01 |  |
| 293 | 13 | A | A | A | 93.55 | 8.958830e-05 | \* |
| 293 | 13 | A | A | C | 1.36 | 5.647178e-01 |  |
| 293 | 13 | A | A | G | 0.51 | 5.842171e-01 |  |
| 293 | 13 | A | A | T | 4.58 | 3.511524e-01 |  |
| 294 | 14 | C | C | A | 0.79 | 5.533724e-01 |  |
| 294 | 14 | C | C | C | 91.36 | 1.805887e-03 | \* |
| 294 | 14 | C | C | G | 1.83 | 4.983131e-01 |  |
| 294 | 14 | C | C | T | 6.02 | 3.068997e-01 |  |
| 295 | 15 | C | C | A | 9.73 | 1.897207e-01 |  |
| 295 | 15 | C | C | C | 80.16 | 3.539107e-03 | \* |
| 295 | 15 | C | C | G | 1.56 | 5.133794e-01 |  |
| 295 | 15 | C | C | T | 8.56 | 2.451140e-01 |  |
| 296 | 16 | G | G | A | 0.00 | 6.680942e-01 |  |
| 296 | 16 | G | G | C | 7.43 | 3.461016e-01 |  |
| 296 | 16 | G | G | G | 91.43 | 1.113552e-03 | \* |
| 296 | 16 | G | G | T | 1.14 | 5.107614e-01 |  |
| 297 | 17 | T | T | A | 0.00 | 6.680942e-01 |  |
| 297 | 17 | T | T | C | 12.54 | 2.412043e-01 |  |
| 297 | 17 | T | T | G | 1.98 | 4.906418e-01 |  |
| 297 | 17 | T | T | T | 85.48 | 9.342765e-04 | \* |
| 298 | 18 | G | G | A | 0.56 | 5.761918e-01 |  |
| 298 | 18 | G | G | C | 3.54 | 4.661615e-01 |  |
| 298 | 18 | G | G | G | 93.10 | 1.002069e-03 | \* |
| 298 | 18 | G | G | T | 2.80 | 4.205679e-01 |  |
| 299 | 19 | T | T | A | 10.73 | 1.709252e-01 |  |
| 299 | 19 | T | T | C | 0.28 | 6.356718e-01 |  |
| 299 | 19 | T | T | G | 0.00 | 6.483791e-01 |  |
| 299 | 19 | T | T | T | 88.98 | 7.347655e-04 | \* |
| 300 | 20 | T | T | A | 0.26 | 6.134627e-01 |  |
| 300 | 20 | T | T | C | 10.23 | 2.831867e-01 |  |
| 300 | 20 | T | T | G | 2.56 | 4.628237e-01 |  |
| 300 | 20 | T | T | T | 86.96 | 8.442178e-04 | \* |
| 301 | 21 | T | T | A | 0.27 | 6.109964e-01 |  |
| 301 | 21 | T | T | C | 0.00 | 6.683938e-01 |  |
| 301 | 21 | T | T | G | 3.01 | 4.432003e-01 |  |
| 301 | 21 | T | T | T | 96.72 | 4.329142e-04 | \* |
| 302 | 22 | G | G | A | 5.70 | 2.930029e-01 |  |
| 302 | 22 | G | G | C | 1.43 | 5.610510e-01 |  |
| 302 | 22 | G | G | G | 88.80 | 1.315233e-03 | \* |
| 302 | 22 | G | G | T | 4.07 | 3.690771e-01 |  |
| 303 | 23 | T | T | A | 0.00 | 6.680942e-01 |  |
| 303 | 23 | T | T | C | 12.46 | 2.424778e-01 |  |
| 303 | 23 | T | T | G | 0.85 | 5.578493e-01 |  |
| 303 | 23 | T | T | T | 86.69 | 8.600481e-04 | \* |
| 304 | 24 | G | G | A | 2.02 | 4.596318e-01 |  |
| 304 | 24 | G | G | C | 4.03 | 4.482292e-01 |  |
| 304 | 24 | G | G | G | 89.67 | 1.244417e-03 | \* |
| 304 | 24 | G | G | T | 4.28 | 3.615889e-01 |  |
| 305 | 25 | C | C | A | 0.25 | 6.138332e-01 |  |
| 305 | 25 | C | C | C | 84.05 | 2.800061e-03 | \* |
| 305 | 25 | C | C | G | 0.00 | 6.483791e-01 |  |
| 305 | 25 | C | C | T | 15.70 | 1.366411e-01 |  |
| 306 | 26 | A | A | A | 81.56 | 2.573349e-04 | \* |
| 306 | 26 | A | A | C | 8.83 | 3.127134e-01 |  |
| 306 | 26 | A | A | G | 9.61 | 2.548080e-01 |  |
| 306 | 26 | A | A | T | 0.00 | 6.343612e-01 |  |
| 307 | 27 | C | C | A | 28.85 | 2.956314e-02 |  |
| 307 | 27 | C | C | C | 67.84 | 7.446708e-03 | \* |
| 307 | 27 | C | C | G | 1.56 | 5.132081e-01 |  |
| 307 | 27 | C | C | T | 1.75 | 4.729899e-01 |  |
| 308 | 28 | T | T | A | 0.00 | 6.680942e-01 |  |
| 308 | 28 | T | T | C | 0.33 | 6.320151e-01 |  |
| 308 | 28 | T | T | G | 2.61 | 4.606322e-01 |  |
| 308 | 28 | T | T | T | 97.07 | 4.227807e-04 | \* |
| 309 | 29 | A | A | A | 94.43 | 8.294321e-05 | \* |
| 309 | 29 | A | A | C | 0.98 | 5.862897e-01 |  |
| 309 | 29 | A | A | G | 4.59 | 3.837068e-01 |  |
| 309 | 29 | A | A | T | 0.00 | 6.343612e-01 |  |
| 310 | 30 | C | C | A | 0.00 | 6.680942e-01 |  |
| 310 | 30 | C | C | C | 97.04 | 1.285618e-03 | \* |
| 310 | 30 | C | C | G | 0.00 | 6.483791e-01 |  |
| 310 | 30 | C | C | T | 2.96 | 4.133871e-01 |  |
| 311 | 31 | A | A | A | 93.00 | 9.400812e-05 | \* |
| 311 | 31 | A | A | C | 3.00 | 4.876630e-01 |  |
| 311 | 31 | A | A | G | 2.50 | 4.654590e-01 |  |
| 311 | 31 | A | A | T | 1.50 | 4.878642e-01 |  |
| 312 | 32 | C | C | A | 0.36 | 5.991783e-01 |  |
| 312 | 32 | C | C | C | 95.29 | 1.427425e-03 | \* |
| 312 | 32 | C | C | G | 1.45 | 5.195178e-01 |  |
| 312 | 32 | C | C | T | 2.90 | 4.161087e-01 |  |
| 313 | 33 | G | G | A | 8.26 | 2.215415e-01 |  |
| 313 | 33 | G | G | C | 0.46 | 6.215007e-01 |  |
| 313 | 33 | G | G | G | 91.28 | 1.123753e-03 | \* |
| 313 | 33 | G | G | T | 0.00 | 6.343612e-01 |  |
| 314 | 34 | G | G | A | 0.83 | 5.495035e-01 |  |
| 314 | 34 | G | G | C | 0.28 | 6.362775e-01 |  |
| 314 | 34 | G | G | G | 94.21 | 9.337307e-04 | \* |
| 314 | 34 | G | G | T | 4.68 | 3.478206e-01 |  |
| 315 | 35 | G | G | A | 1.08 | 5.272189e-01 |  |
| 315 | 35 | G | G | C | 5.76 | 3.919844e-01 |  |
| 315 | 35 | G | G | G | 93.17 | 9.977432e-04 | \* |
| 315 | 35 | G | G | T | 0.00 | 6.343612e-01 |  |
| 316 | 36 | C | C | A | 5.42 | 3.027039e-01 |  |
| 316 | 36 | C | C | C | 93.86 | 1.554667e-03 | \* |
| 316 | 36 | C | C | G | 0.00 | 6.483791e-01 |  |
| 316 | 36 | C | C | T | 0.72 | 5.420215e-01 |  |
| 317 | 37 | A | A | A | 95.00 | 7.886957e-05 | \* |
| 317 | 37 | A | A | C | 2.50 | 5.089006e-01 |  |
| 317 | 37 | A | A | G | 2.50 | 4.654590e-01 |  |
| 317 | 37 | A | A | T | 0.00 | 6.343612e-01 |  |
| 318 | 38 | C | C | A | 2.09 | 4.550177e-01 |  |
| 318 | 38 | C | C | C | 96.34 | 1.340155e-03 | \* |
| 318 | 38 | C | C | G | 0.00 | 6.483791e-01 |  |
| 318 | 38 | C | C | T | 1.57 | 4.838690e-01 |  |
| 319 | 39 | C | C | A | 2.41 | 4.358196e-01 |  |
| 319 | 39 | C | C | C | 96.39 | 1.336881e-03 | \* |
| 319 | 39 | C | C | G | 0.30 | 6.034658e-01 |  |
| 319 | 39 | C | C | T | 0.90 | 5.278212e-01 |  |
| 320 | 40 | C | C | A | 5.99 | 2.835752e-01 |  |
| 320 | 40 | C | C | C | 92.11 | 1.726317e-03 | \* |
| 320 | 40 | C | C | G | 0.95 | 5.511169e-01 |  |
| 320 | 40 | C | C | T | 0.95 | 5.246486e-01 |  |
| 321 | 41 | T | T | A | 3.69 | 3.707149e-01 |  |
| 321 | 41 | T | T | C | 9.02 | 3.086016e-01 |  |
| 321 | 41 | T | T | G | 1.23 | 5.327029e-01 |  |
| 321 | 41 | T | T | T | 86.07 | 8.974020e-04 | \* |
| 322 | 42 | G | G | A | 1.79 | 4.745565e-01 |  |
| 322 | 42 | G | G | C | 0.67 | 6.064105e-01 |  |
| 322 | 42 | G | G | G | 97.10 | 7.783321e-04 | \* |
| 322 | 42 | G | G | T | 0.45 | 5.665960e-01 |  |
| 323 | 43 | A | T | A | 10.82 | 1.694328e-01 |  |
| 323 | 43 | A | T | C | 0.98 | 5.862897e-01 |  |
| 323 | 43 | A | T | G | 1.31 | 5.276851e-01 |  |
| 323 | 43 | A | T | T | 86.89 | 8.483531e-04 | \* |
| 324 | 44 | C | C | A | 0.00 | 6.680942e-01 |  |
| 324 | 44 | C | C | C | 95.29 | 1.427062e-03 | \* |
| 324 | 44 | C | C | G | 4.71 | 3.798482e-01 |  |
| 324 | 44 | C | C | T | 0.00 | 6.343612e-01 |  |
| 325 | 45 | C | C | A | 2.52 | 4.293214e-01 |  |
| 325 | 45 | C | C | C | 87.70 | 2.249507e-03 | \* |
| 325 | 45 | C | C | G | 3.15 | 4.369880e-01 |  |
| 325 | 45 | C | C | T | 6.62 | 2.905544e-01 |  |
| 326 | 46 | G | G | A | 3.66 | 3.720717e-01 |  |
| 326 | 46 | G | G | C | 0.00 | 6.683938e-01 |  |
| 326 | 46 | G | G | G | 78.05 | 2.602889e-03 | \* |
| 326 | 46 | G | G | T | 18.29 | 1.115653e-01 |  |
| 327 | 47 | A | A | A | 92.86 | 9.519502e-05 | \* |
| 327 | 47 | A | A | C | 6.33 | 3.752714e-01 |  |
| 327 | 47 | A | A | G | 0.81 | 5.608097e-01 |  |
| 327 | 47 | A | A | T | 0.00 | 6.343612e-01 |  |
| 328 | 48 | C | C | A | 31.42 | 2.328706e-02 |  |
| 328 | 48 | C | C | C | 66.59 | 8.029683e-03 | \* |
| 328 | 48 | C | C | G | 0.00 | 6.483791e-01 |  |
| 328 | 48 | C | C | T | 1.99 | 4.600069e-01 |  |
| 329 | 49 | G | G | A | 30.47 | 2.542697e-02 |  |
| 329 | 49 | G | G | C | 2.34 | 5.158776e-01 |  |
| 329 | 49 | G | G | G | 66.80 | 5.343475e-03 | \* |
| 329 | 49 | G | G | T | 0.39 | 5.722093e-01 |  |
| 330 | 50 | G | G | A | 2.51 | 4.302630e-01 |  |
| 330 | 50 | G | G | C | 0.84 | 5.954888e-01 |  |
| 330 | 50 | G | G | G | 96.66 | 8.002870e-04 | \* |
| 330 | 50 | G | G | T | 0.00 | 6.343612e-01 |  |
| 331 | 51 | C | C | A | 0.00 | 6.680942e-01 |  |
| 331 | 51 | C | C | C | 98.13 | 1.204679e-03 | \* |
| 331 | 51 | C | C | G | 0.00 | 6.483791e-01 |  |
| 331 | 51 | C | C | T | 1.87 | 4.664092e-01 |  |
| 332 | 52 | A | A | A | 97.00 | 6.617662e-05 | \* |
| 332 | 52 | A | A | C | 0.00 | 6.683938e-01 |  |
| 332 | 52 | A | A | G | 3.00 | 4.434308e-01 |  |
| 332 | 52 | A | A | T | 0.00 | 6.343612e-01 |  |
| 333 | 53 | A | A | A | 98.35 | 5.876860e-05 | \* |
| 333 | 53 | A | A | C | 0.41 | 6.251229e-01 |  |
| 333 | 53 | A | A | G | 0.82 | 5.597683e-01 |  |
| 333 | 53 | A | A | T | 0.41 | 5.700746e-01 |  |
| 334 | 54 | G | G | A | 1.79 | 4.745565e-01 |  |
| 334 | 54 | G | G | C | 0.45 | 6.224335e-01 |  |
| 334 | 54 | G | G | G | 97.77 | 7.461357e-04 | \* |
| 334 | 54 | G | G | T | 0.00 | 6.343612e-01 |  |
| 335 | 55 | A | G | A | 38.11 | 1.257311e-02 |  |
| 335 | 55 | A | G | C | 11.46 | 2.598656e-01 |  |
| 335 | 55 | A | G | G | 50.14 | 1.568688e-02 |  |
| 335 | 55 | A | G | T | 0.29 | 5.835428e-01 |  |
| 336 | 56 | A | C | A | 40.62 | 9.991420e-03 | \* |
| 336 | 56 | A | C | C | 54.69 | 1.658151e-02 |  |
| 336 | 56 | A | C | G | 3.52 | 4.225346e-01 |  |
| 336 | 56 | A | C | T | 1.17 | 5.087970e-01 |  |
| 337 | 57 | G | C | A | 17.26 | 8.906976e-02 |  |
| 337 | 57 | G | C | C | 51.33 | 2.037400e-02 |  |
| 337 | 57 | G | C | G | 30.97 | 5.587574e-02 |  |
| 337 | 57 | G | C | T | 0.44 | 5.669845e-01 |  |
| 338 | 58 | T | T | A | 35.36 | 1.617776e-02 |  |
| 338 | 58 | T | T | C | 16.35 | 1.864035e-01 |  |
| 338 | 58 | T | T | G | 0.00 | 6.483791e-01 |  |
| 338 | 58 | T | T | T | 48.29 | 1.240106e-02 |  |
| 339 | 59 | T | C | A | 3.97 | 3.581608e-01 |  |
| 339 | 59 | T | C | C | 43.05 | 3.395578e-02 |  |
| 339 | 59 | T | C | G | 10.93 | 2.303448e-01 |  |
| 339 | 59 | T | C | T | 42.05 | 1.932657e-02 |  |
| 340 | 60 | C | C | A | 6.32 | 2.733225e-01 |  |
| 340 | 60 | C | C | C | 49.01 | 2.349074e-02 |  |
| 340 | 60 | C | C | G | 27.27 | 7.185705e-02 |  |
| 340 | 60 | C | C | T | 17.39 | 1.196501e-01 |  |
| 341 | 61 | G | C | A | 0.00 | 6.680942e-01 |  |
| 341 | 61 | G | C | C | 66.38 | 8.134388e-03 | \* |
| 341 | 61 | G | C | G | 33.62 | 4.674858e-02 |  |
| 341 | 61 | G | C | T | 0.00 | 6.343612e-01 |  |
| 342 | 62 | A | C | A | 47.76 | 5.231375e-03 | \* |
| 342 | 62 | A | C | C | 52.24 | 1.926005e-02 |  |
| 342 | 62 | A | C | G | 0.00 | 6.483791e-01 |  |
| 342 | 62 | A | C | T | 0.00 | 6.343612e-01 |  |
| 343 | 63 | C | C | A | 3.95 | 3.590629e-01 |  |
| 343 | 63 | C | C | C | 53.36 | 1.798619e-02 |  |
| 343 | 63 | C | C | G | 27.27 | 7.185705e-02 |  |
| 343 | 63 | C | C | T | 15.42 | 1.397078e-01 |  |
| 344 | 64 | A | G | A | 30.42 | 2.554657e-02 |  |
| 344 | 64 | A | G | C | 0.00 | 6.683938e-01 |  |
| 344 | 64 | A | G | G | 51.33 | 1.451818e-02 |  |
| 344 | 64 | A | G | T | 18.25 | 1.119262e-01 |  |
| 345 | 65 | G | G | A | 0.76 | 5.557529e-01 |  |
| 345 | 65 | G | G | C | 15.59 | 1.961409e-01 |  |
| 345 | 65 | G | G | G | 49.81 | 1.603190e-02 |  |
| 345 | 65 | G | G | T | 33.84 | 3.493675e-02 |  |
| 346 | 66 | C | C | A | 11.54 | 1.574034e-01 |  |
| 346 | 66 | C | C | C | 44.06 | 3.189699e-02 |  |
| 346 | 66 | C | C | G | 44.06 | 2.337411e-02 |  |
| 346 | 66 | C | C | T | 0.35 | 5.765208e-01 |  |
| 347 | 67 | T | T | A | 0.69 | 5.626964e-01 |  |
| 347 | 67 | T | T | C | 11.72 | 2.551774e-01 |  |
| 347 | 67 | T | T | G | 42.41 | 2.604511e-02 |  |
| 347 | 67 | T | T | T | 45.17 | 1.547135e-02 |  |
| 348 | 68 | C | C | A | 14.73 | 1.142133e-01 |  |
| 348 | 68 | C | C | C | 50.00 | 2.210534e-02 |  |
| 348 | 68 | C | C | G | 1.16 | 5.368832e-01 |  |
| 348 | 68 | C | C | T | 34.11 | 3.426189e-02 |  |
| 349 | 69 | C | C | A | 0.00 | 6.680942e-01 |  |
| 349 | 69 | C | C | C | 100.00 | 1.077164e-03 | \* |
| 349 | 69 | C | C | G | 0.00 | 6.483791e-01 |  |
| 349 | 69 | C | C | T | 0.00 | 6.343612e-01 |  |
| 350 | 70 | C | C | A | 1.25 | 5.133603e-01 |  |
| 350 | 70 | C | C | C | 76.67 | 4.366917e-03 | \* |
| 350 | 70 | C | C | G | 20.42 | 1.155454e-01 |  |
| 350 | 70 | C | C | T | 1.67 | 4.780024e-01 |  |
| 351 | 71 | G | A | A | 58.22 | 2.043627e-03 | \* |
| 351 | 71 | G | A | C | 0.00 | 6.683938e-01 |  |
| 351 | 71 | G | A | G | 41.78 | 2.715067e-02 |  |
| 351 | 71 | G | A | T | 0.00 | 6.343612e-01 |  |
| 352 | 72 | C | C | A | 13.08 | 1.346941e-01 |  |
| 352 | 72 | C | C | C | 85.00 | 2.644841e-03 | \* |
| 352 | 72 | C | C | G | 0.00 | 6.483791e-01 |  |
| 352 | 72 | C | C | T | 1.92 | 4.636626e-01 |  |
| 353 | 73 | G | T | A | 7.60 | 2.377177e-01 |  |
| 353 | 73 | G | T | C | 0.40 | 6.260241e-01 |  |
| 353 | 73 | G | T | G | 33.20 | 4.808865e-02 |  |
| 353 | 73 | G | T | T | 58.80 | 5.919509e-03 | \* |
| 354 | 74 | A | A | A | 44.31 | 7.148105e-03 | \* |
| 354 | 74 | A | A | C | 12.31 | 2.451079e-01 |  |
| 354 | 74 | A | A | G | 0.00 | 6.483791e-01 |  |
| 354 | 74 | A | A | T | 43.38 | 1.757299e-02 |  |
| 355 | 75 | C | C | A | 0.00 | 6.680942e-01 |  |
| 355 | 75 | C | C | C | 97.21 | 1.272491e-03 | \* |
| 355 | 75 | C | C | G | 0.00 | 6.483791e-01 |  |
| 355 | 75 | C | C | T | 2.79 | 4.210030e-01 |  |

## For use in R

If you want to work with the results in R, here is output that you can copy and paste in your terminal to get:

The base information:

```
structure(list(focal.base = c("A", "C", "G", "T"), avg.percsignal = c(79.6187568725642, 
79.9435212572586, 79.1510501159147, 79.9204294788866), avg.areasignal = c(351.01724137931, 
305.267605633803, 353.79679144385, 318.952755905512), crit.perc.area = c(40.6155850173033, 
62.9789252921424, 57.0720836735556, 51.3338967383008), mu = c(7.99609285439641, 
13.0497210937894, 11.4214229321173, 10.1065290255626), fillibens = c(0.990428848732767, 
0.993991902314401, 0.993773137255059, 0.992335814333557)), .Names = c("focal.base", 
"avg.percsignal", "avg.areasignal", "crit.perc.area", "mu", "fillibens"
), row.names = c(NA, -4L), class = "data.frame")
```

the data.frame that contains information on the guide region:

```
structure(list(A.area = c(189, 261, 13, 545, 3, 15, 6, 7, 35, 
0, 201, 15, 551, 3, 25, 0, 0, 3, 38, 1, 1, 28, 0, 8, 1, 314, 
148, 0, 288, 0, 186, 1, 18, 3, 3, 15, 228, 8, 8, 19, 9, 8, 33, 
0, 8, 9, 689, 142, 78, 9, 0, 97, 239, 4, 133, 104, 39, 93, 12, 
16, 0, 149, 10, 80, 2, 33, 2, 38, 0, 3, 124, 34, 19, 144, 0), 
    C.area = c(1, 37, 19, 4, 303, 30, 1, 17, 241, 353, 22, 1, 
    8, 349, 206, 26, 38, 19, 1, 40, 0, 7, 44, 16, 332, 34, 348, 
    1, 3, 295, 6, 263, 1, 1, 16, 260, 6, 369, 320, 292, 22, 3, 
    3, 324, 278, 0, 47, 301, 6, 3, 262, 0, 1, 1, 40, 140, 116, 
    43, 130, 124, 154, 163, 135, 0, 41, 126, 34, 129, 254, 184, 
    0, 221, 1, 40, 244), G.area = c(7, 1, 221, 5, 56, 7, 608, 
    385, 30, 2, 24, 221, 3, 7, 4, 320, 6, 499, 0, 10, 11, 436, 
    3, 356, 0, 37, 8, 8, 14, 0, 5, 4, 199, 342, 259, 0, 6, 0, 
    1, 3, 3, 435, 4, 16, 10, 192, 6, 0, 171, 347, 0, 3, 2, 219, 
    175, 9, 70, 0, 33, 69, 78, 0, 69, 135, 131, 126, 123, 3, 
    0, 49, 89, 0, 83, 0, 0), T.area = c(41, 0, 0, 16, 3, 288, 
    19, 0, 2, 21, 0, 20, 27, 23, 22, 4, 259, 15, 315, 340, 354, 
    20, 306, 17, 62, 0, 9, 298, 0, 9, 3, 8, 0, 17, 0, 2, 0, 6, 
    3, 3, 210, 2, 265, 0, 21, 45, 0, 9, 1, 0, 5, 0, 1, 0, 1, 
    3, 1, 127, 127, 44, 0, 0, 39, 48, 89, 1, 131, 88, 0, 4, 0, 
    5, 147, 141, 7), Tot.area = c(238, 299, 253, 570, 365, 340, 
    634, 409, 308, 376, 247, 257, 589, 382, 257, 350, 303, 536, 
    354, 391, 366, 491, 353, 397, 395, 385, 513, 307, 305, 304, 
    200, 276, 218, 363, 278, 277, 240, 383, 332, 317, 244, 448, 
    305, 340, 317, 246, 742, 452, 256, 359, 267, 100, 243, 224, 
    349, 256, 226, 263, 302, 253, 232, 312, 253, 263, 263, 286, 
    290, 258, 254, 240, 213, 260, 250, 325, 251), A.perc = c(79.4117647058823, 
    87.2909698996656, 5.13833992094862, 95.6140350877193, 0.821917808219178, 
    4.41176470588235, 0.946372239747634, 1.71149144254279, 11.3636363636364, 
    0, 81.3765182186235, 5.83657587548638, 93.5483870967742, 
    0.785340314136126, 9.72762645914397, 0, 0, 0.559701492537313, 
    10.7344632768362, 0.255754475703325, 0.273224043715847, 5.70264765784114, 
    0, 2.01511335012594, 0.253164556962025, 81.5584415584416, 
    28.8499025341131, 0, 94.4262295081967, 0, 93, 0.36231884057971, 
    8.25688073394495, 0.826446280991736, 1.07913669064748, 5.41516245487365, 
    95, 2.088772845953, 2.40963855421687, 5.99369085173502, 3.68852459016393, 
    1.78571428571429, 10.8196721311475, 0, 2.52365930599369, 
    3.65853658536585, 92.8571428571429, 31.4159292035398, 30.46875, 
    2.50696378830084, 0, 97, 98.3539094650206, 1.78571428571429, 
    38.10888252149, 40.625, 17.2566371681416, 35.361216730038, 
    3.97350993377483, 6.32411067193676, 0, 47.7564102564103, 
    3.95256916996047, 30.4182509505703, 0.760456273764259, 11.5384615384615, 
    0.689655172413793, 14.7286821705426, 0, 1.25, 58.2159624413146, 
    13.0769230769231, 7.6, 44.3076923076923, 0), C.perc = c(0.420168067226891, 
    12.3745819397993, 7.5098814229249, 0.701754385964912, 83.013698630137, 
    8.82352941176471, 0.157728706624606, 4.15647921760391, 78.2467532467532, 
    93.8829787234042, 8.90688259109312, 0.389105058365759, 1.35823429541596, 
    91.3612565445026, 80.1556420233463, 7.42857142857143, 12.5412541254125, 
    3.54477611940298, 0.282485875706215, 10.230179028133, 0, 
    1.42566191446029, 12.4645892351275, 4.03022670025189, 84.0506329113924, 
    8.83116883116883, 67.8362573099415, 0.325732899022801, 0.983606557377049, 
    97.0394736842105, 3, 95.2898550724638, 0.458715596330275, 
    0.275482093663912, 5.75539568345324, 93.8628158844765, 2.5, 
    96.3446475195822, 96.3855421686747, 92.1135646687697, 9.01639344262295, 
    0.669642857142857, 0.983606557377049, 95.2941176470588, 87.6971608832808, 
    0, 6.33423180592992, 66.5929203539823, 2.34375, 0.835654596100279, 
    98.12734082397, 0, 0.411522633744856, 0.446428571428571, 
    11.4613180515759, 54.6875, 51.3274336283186, 16.3498098859316, 
    43.046357615894, 49.0118577075099, 66.3793103448276, 52.2435897435897, 
    53.3596837944664, 0, 15.5893536121673, 44.0559440559441, 
    11.7241379310345, 50, 100, 76.6666666666667, 0, 85, 0.4, 
    12.3076923076923, 97.2111553784861), G.perc = c(2.94117647058824, 
    0.334448160535117, 87.3517786561265, 0.87719298245614, 15.3424657534247, 
    2.05882352941176, 95.8990536277603, 94.1320293398533, 9.74025974025974, 
    0.531914893617021, 9.7165991902834, 85.9922178988327, 0.509337860780985, 
    1.83246073298429, 1.55642023346304, 91.4285714285714, 1.98019801980198, 
    93.0970149253731, 0, 2.55754475703325, 3.00546448087432, 
    88.7983706720978, 0.84985835694051, 89.6725440806045, 0, 
    9.61038961038961, 1.55945419103314, 2.60586319218241, 4.59016393442623, 
    0, 2.5, 1.44927536231884, 91.2844036697248, 94.2148760330578, 
    93.1654676258993, 0, 2.5, 0, 0.301204819277108, 0.946372239747634, 
    1.22950819672131, 97.0982142857143, 1.31147540983607, 4.70588235294118, 
    3.15457413249211, 78.0487804878049, 0.808625336927224, 0, 
    66.796875, 96.6573816155989, 0, 3, 0.823045267489712, 97.7678571428571, 
    50.1432664756447, 3.515625, 30.9734513274336, 0, 10.9271523178808, 
    27.2727272727273, 33.6206896551724, 0, 27.2727272727273, 
    51.3307984790875, 49.8098859315589, 44.0559440559441, 42.4137931034483, 
    1.16279069767442, 0, 20.4166666666667, 41.7840375586854, 
    0, 33.2, 0, 0), T.perc = c(17.2268907563025, 0, 0, 2.80701754385965, 
    0.821917808219178, 84.7058823529412, 2.99684542586751, 0, 
    0.649350649350649, 5.58510638297872, 0, 7.78210116731517, 
    4.58404074702886, 6.02094240837696, 8.56031128404669, 1.14285714285714, 
    85.4785478547855, 2.79850746268657, 88.9830508474576, 86.9565217391304, 
    96.7213114754098, 4.07331975560081, 86.685552407932, 4.28211586901763, 
    15.6962025316456, 0, 1.75438596491228, 97.0684039087948, 
    0, 2.96052631578947, 1.5, 2.89855072463768, 0, 4.6831955922865, 
    0, 0.72202166064982, 0, 1.56657963446475, 0.903614457831325, 
    0.946372239747634, 86.0655737704918, 0.446428571428571, 86.8852459016394, 
    0, 6.62460567823344, 18.2926829268293, 0, 1.99115044247788, 
    0.390625, 0, 1.87265917602996, 0, 0.411522633744856, 0, 0.286532951289398, 
    1.171875, 0.442477876106195, 48.2889733840304, 42.0529801324503, 
    17.3913043478261, 0, 0, 15.4150197628458, 18.2509505703422, 
    33.8403041825095, 0.34965034965035, 45.1724137931034, 34.1085271317829, 
    0, 1.66666666666667, 0, 1.92307692307692, 58.8, 43.3846153846154, 
    2.78884462151394), base.call = c("A", "A", "G", "A", "C", 
    "T", "G", "G", "C", "C", "A", "G", "A", "C", "C", "G", "T", 
    "G", "T", "T", "T", "G", "T", "G", "C", "A", "C", "T", "A", 
    "C", "A", "C", "G", "G", "G", "C", "A", "C", "C", "C", "T", 
    "G", "T", "C", "C", "G", "A", "C", "G", "G", "C", "A", "A", 
    "G", "G", "C", "C", "T", "C", "C", "C", "C", "C", "G", "G", 
    "C", "T", "C", "C", "C", "A", "C", "T", "A", "C"), index = 281:355, 
    guide.seq = c("A", "A", "G", "A", "C", "T", "G", "G", "C", 
    "C", "A", "G", "A", "C", "C", "G", "T", "G", "T", "T", "T", 
    "G", "T", "G", "C", "A", "C", "T", "A", "C", "A", "C", "G", 
    "G", "G", "C", "A", "C", "C", "C", "T", "G", "A", "C", "C", 
    "G", "A", "C", "G", "G", "C", "A", "A", "G", "A", "A", "G", 
    "T", "T", "C", "G", "A", "C", "A", "G", "C", "T", "C", "C", 
    "C", "G", "C", "G", "A", "C"), T.pval = c(0.121192315007533, 
    0.634361233480176, 0.634361233480176, 0.420185358417211, 
    0.534055267101582, 0.00098515026974022, 0.411806250495044, 
    0.634361233480176, 0.54808953675818, 0.319468434629906, 0.634361233480176, 
    0.262229106113896, 0.351152360060578, 0.306899715844476, 
    0.245114032700711, 0.510761406550783, 0.000934276460396122, 
    0.420567908857843, 0.000734765488805911, 0.000844217825250126, 
    0.000432914202940005, 0.369077123740048, 0.000860048072958364, 
    0.361588901290383, 0.13664112258126, 0.634361233480176, 0.472989933424819, 
    0.000422780701835546, 0.634361233480176, 0.413387065336946, 
    0.487864241295126, 0.4161086958139, 0.634361233480176, 0.347820574240725, 
    0.634361233480176, 0.542021522477881, 0.634361233480176, 
    0.483869022087739, 0.527821221637893, 0.524648560843135, 
    0.000897402001916348, 0.566595973108555, 0.000848353143452885, 
    0.634361233480176, 0.290554429045457, 0.111565259613054, 
    0.634361233480176, 0.46000694721577, 0.572209327631496, 0.634361233480176, 
    0.466409229057497, 0.634361233480176, 0.570074601943618, 
    0.634361233480176, 0.583542780512941, 0.508797017983756, 
    0.566984505926146, 0.0124010558083019, 0.0193265697716427, 
    0.119650054460225, 0.634361233480176, 0.634361233480176, 
    0.13970781923698, 0.111926189817971, 0.0349367546003274, 
    0.576520842338681, 0.015471353088423, 0.0342618917819284, 
    0.634361233480176, 0.478002443685791, 0.634361233480176, 
    0.463662589974305, 0.00591950856138523, 0.017572985118296, 
    0.421003018604352), C.pval = c(0.6244511371081, 0.243982704946379, 
    0.344050116671512, 0.604241467043878, 0.0029801070836557, 
    0.312884424358792, 0.647206674603483, 0.443735930893764, 
    0.00397023386410389, 0.00155279185278601, 0.311024773862765, 
    0.62688284400126, 0.564717756388018, 0.00180588712659935, 
    0.00353910703918969, 0.34610160186429, 0.241204311762724, 
    0.466161545981675, 0.635671754387579, 0.283186666720222, 
    0.668393782383419, 0.561051041273277, 0.242477842259176, 
    0.448229202162483, 0.00280006133547817, 0.312713431858522, 
    0.00744670819755378, 0.632015072104339, 0.586289706239784, 
    0.00128561827330698, 0.48766304385942, 0.00142742547731056, 
    0.621500698172882, 0.636277510909505, 0.391984405273106, 
    0.00155466685150696, 0.508900563141202, 0.00134015462813863, 
    0.00133688133982091, 0.0017263165977619, 0.308601584454266, 
    0.606410545754862, 0.586289706239784, 0.00142706154098104, 
    0.00224950729790918, 0.668393782383419, 0.375271389125701, 
    0.00802968335900522, 0.515877585161692, 0.595488774036098, 
    0.00120467917242073, 0.668393782383419, 0.625122858517587, 
    0.622433492183537, 0.259865593515495, 0.0165815063876711, 
    0.0203739964285952, 0.18640347558879, 0.0339557832156741, 
    0.0234907401246989, 0.00813438837573921, 0.0192600511320207, 
    0.0179861874351706, 0.668393782383419, 0.196140893814091, 
    0.0318969943058787, 0.255177434245734, 0.0221053418346777, 
    0.00107716358059595, 0.00436691737280004, 0.668393782383419, 
    0.00264484051033276, 0.626024142387019, 0.245107888473841, 
    0.00127249051949274), G.pval = c(0.445925590178641, 0.600147398920402, 
    0.00144145457150757, 0.555915431726073, 0.166013587446893, 
    0.486659484267194, 0.000839522909428014, 0.000938630309385013, 
    0.252262617838654, 0.582308191801245, 0.252724082020444, 
    0.00157119684834472, 0.58421708926335, 0.498313061586352, 
    0.5133793910436, 0.00111355229406485, 0.490641813740259, 
    0.00100206905201716, 0.648379052369076, 0.462823693575189, 
    0.443200287869576, 0.00131523273490286, 0.557849299437509, 
    0.00124441673106923, 0.648379052369076, 0.254808021453122, 
    0.513208096695771, 0.460632164829593, 0.38370679901371, 0.648379052369076, 
    0.465458985549328, 0.519517841247941, 0.00112375297286871, 
    0.000933730675478639, 0.000997743166715193, 0.648379052369076, 
    0.465458985549328, 0.648379052369076, 0.603465814473017, 
    0.551116863498498, 0.532702934774085, 0.000778332130578541, 
    0.527685135337322, 0.379848213351157, 0.436988046481405, 
    0.00260288884644755, 0.560809669307529, 0.648379052369076, 
    0.00534347450380612, 0.000800287046495818, 0.648379052369076, 
    0.44343081808618, 0.559768307462055, 0.000746135701362061, 
    0.0156868807166863, 0.422534609431746, 0.0558757407460451, 
    0.648379052369076, 0.230344848162505, 0.0718570473840199, 
    0.046748576540758, 0.648379052369076, 0.0718570473840199, 
    0.014518179630342, 0.0160319029625879, 0.0233741115367487, 
    0.0260451066799903, 0.536883196540714, 0.648379052369076, 
    0.115545418121541, 0.0271506657204861, 0.648379052369076, 
    0.0480886498834483, 0.648379052369076, 0.648379052369076), 
    A.pval = c(0.000311015715150798, 0.00015528823540123, 0.312433177938073, 
    7.47322215702839e-05, 0.549925928539991, 0.339970980452131, 
    0.538628413965669, 0.479579624903033, 0.160239581466651, 
    0.668094218415416, 0.000261498330327958, 0.288616897851335, 
    8.95882998481756e-05, 0.553372411387253, 0.189720668942176, 
    0.668094218415416, 0.668094218415416, 0.57619179658025, 0.170925214451749, 
    0.613462719677964, 0.610996362869929, 0.293002915322683, 
    0.668094218415416, 0.459631782909127, 0.61383324090133, 0.000257334932157849, 
    0.0295631361409276, 0.668094218415416, 8.29432110741601e-05, 
    0.668094218415416, 9.40081200482279e-05, 0.599178330820543, 
    0.221541481099046, 0.54950345371129, 0.527218858482398, 0.302703893682658, 
    7.88695675192352e-05, 0.455017728021012, 0.43581960075622, 
    0.28357522308501, 0.370714948410819, 0.474556482642727, 0.169432752731477, 
    0.668094218415416, 0.429321389772583, 0.372071748912687, 
    9.51950190777318e-05, 0.0232870635096759, 0.0254269697597711, 
    0.430262972919884, 0.668094218415416, 6.61766238637673e-05, 
    5.87686038565627e-05, 0.474556482642727, 0.0125731064769747, 
    0.00999142009311615, 0.0890697646098353, 0.0161777558770896, 
    0.358160784597382, 0.273322544608665, 0.668094218415416, 
    0.00523137489027958, 0.359062934611951, 0.0255465671384605, 
    0.555752917735385, 0.157403398572637, 0.562696388328351, 
    0.114213319813477, 0.668094218415416, 0.513360325431439, 
    0.00204362650328471, 0.134694091658965, 0.237717668957252, 
    0.00714810455327508, 0.668094218415416), guide.position = 1:75), .Names = c("A.area", 
"C.area", "G.area", "T.area", "Tot.area", "A.perc", "C.perc", 
"G.perc", "T.perc", "base.call", "index", "guide.seq", "T.pval", 
"C.pval", "G.pval", "A.pval", "guide.position"), row.names = 281:355, class = "data.frame")
```

*Report generated using EditR v1.0.8*
