# Supplementary material for: Mechanistic and genetic basis of single-strand templated repair at Cas12a-induced DNA breaks in Chlamydomonas reinhardtii
Source: Nat Commun. 2021 Nov 19;12:6751. doi: 10.1038/s41467-021-27004-1 (PMC8604939; doi:10.1038/s41467-021-27004-1)
Supplement: Supplementary file 22 — Source Data [file 41467_2021_27004_MOESM22_ESM.zip › Source Data/EditR analysis/EditR outputs/Controls for normalization/Positive control (16).html]

EditR v1.0.8 report


# EditR v1.0.8 report

- Data QA
  - Filtering data
  - Percent noise peak area
  - Base information
- Predicted editing
  - Editing bar plot
  - Editing table plot
  - Table of editing results
- For use in R

## Data QA

### Filtering data

What the data looked like prefiltering:

and the post filtering signal / noise plot:

### Percent noise peak area

### Base information

Here’s information about the signal of each base, the critical percent value where any higher value would be called as significant, and Filliben’s correlation for how well the noise was modelled by the zero adjusted gamma distribution.

| Base | Average percent signal | Average peak area | Critical percent value | model mu | Fillibens correlation |
| --- | --- | --- | --- | --- | --- |
| A | 90.24824 | 221.2059 | 22.89406 | 4.353824 | 0.8852298 |
| C | 89.95135 | 244.9048 | 44.87044 | 6.777231 | 0.8782118 |
| G | 84.75712 | 253.9107 | 39.90686 | 6.514061 | 0.9233450 |
| T | 85.89093 | 248.2708 | 50.63738 | 8.955531 | 0.9554177 |

## Predicted editing

### Editing bar plot

### Editing table plot

### Table of editing results


Here’s the entire guide region

| Sanger position | Guide position | Guide sequence | Sanger base call | Focal base | Focal base peak area | p value |  |
| --- | --- | --- | --- | --- | --- | --- | --- |
| 275 | 1 | A | A | A | 96.41 | 4.496827e-08 | \* |
| 275 | 1 | A | A | C | 0.60 | 6.546285e-01 |  |
| 275 | 1 | A | A | G | 2.40 | 4.731302e-01 |  |
| 275 | 1 | A | A | T | 0.60 | 6.250333e-01 |  |
| 276 | 2 | A | A | A | 96.05 | 4.768153e-08 | \* |
| 276 | 2 | A | A | C | 2.63 | 4.541050e-01 |  |
| 276 | 2 | A | A | G | 1.32 | 5.674749e-01 |  |
| 276 | 2 | A | A | T | 0.00 | 7.500000e-01 |  |
| 277 | 3 | G | G | A | 1.49 | 5.264834e-01 |  |
| 277 | 3 | G | G | C | 0.99 | 5.988023e-01 |  |
| 277 | 3 | G | G | G | 97.52 | 6.230003e-05 | \* |
| 277 | 3 | G | G | T | 0.00 | 7.500000e-01 |  |
| 278 | 4 | A | A | A | 97.12 | 3.998848e-08 | \* |
| 278 | 4 | A | A | C | 0.67 | 6.439052e-01 |  |
| 278 | 4 | A | A | G | 2.22 | 4.865083e-01 |  |
| 278 | 4 | A | A | T | 0.00 | 7.500000e-01 |  |
| 279 | 5 | C | C | A | 1.57 | 5.154860e-01 |  |
| 279 | 5 | C | C | C | 93.70 | 2.452647e-04 | \* |
| 279 | 5 | C | C | G | 1.97 | 5.064333e-01 |  |
| 279 | 5 | C | C | T | 2.76 | 4.605164e-01 |  |
| 280 | 6 | T | T | A | 5.00 | 2.509185e-01 |  |
| 280 | 6 | T | T | C | 0.38 | 6.948065e-01 |  |
| 280 | 6 | T | T | G | 1.92 | 5.102410e-01 |  |
| 280 | 6 | T | T | T | 92.69 | 5.221971e-04 | \* |
| 281 | 7 | G | G | A | 1.47 | 5.279637e-01 |  |
| 281 | 7 | G | G | C | 0.92 | 6.075869e-01 |  |
| 281 | 7 | G | G | G | 97.24 | 6.386220e-05 | \* |
| 281 | 7 | G | G | T | 0.37 | 6.553608e-01 |  |
| 282 | 8 | G | G | A | 2.35 | 4.324476e-01 |  |
| 282 | 8 | G | G | C | 1.47 | 5.462494e-01 |  |
| 282 | 8 | G | G | G | 95.88 | 7.178259e-05 | \* |
| 282 | 8 | G | G | T | 0.29 | 6.668529e-01 |  |
| 283 | 9 | C | C | A | 0.00 | 8.288288e-01 |  |
| 283 | 9 | C | C | C | 93.78 | 2.437851e-04 | \* |
| 283 | 9 | C | C | G | 2.07 | 4.979168e-01 |  |
| 283 | 9 | C | C | T | 4.15 | 3.940993e-01 |  |
| 284 | 10 | C | C | A | 0.74 | 6.352844e-01 |  |
| 284 | 10 | C | C | C | 93.73 | 2.447895e-04 | \* |
| 284 | 10 | C | C | G | 1.48 | 5.510654e-01 |  |
| 284 | 10 | C | C | T | 4.06 | 3.977470e-01 |  |
| 285 | 11 | A | A | A | 95.63 | 5.114410e-08 | \* |
| 285 | 11 | A | A | C | 0.55 | 6.635762e-01 |  |
| 285 | 11 | A | A | G | 1.09 | 5.923331e-01 |  |
| 285 | 11 | A | A | T | 2.73 | 4.618124e-01 |  |
| 286 | 12 | G | G | A | 1.89 | 4.797317e-01 |  |
| 286 | 12 | G | G | C | 2.36 | 4.728005e-01 |  |
| 286 | 12 | G | G | G | 95.28 | 7.559354e-05 | \* |
| 286 | 12 | G | G | T | 0.47 | 6.409397e-01 |  |
| 287 | 13 | A | A | A | 97.10 | 4.007340e-08 | \* |
| 287 | 13 | A | A | C | 0.67 | 6.434413e-01 |  |
| 287 | 13 | A | A | G | 0.89 | 6.174370e-01 |  |
| 287 | 13 | A | A | T | 1.34 | 5.538555e-01 |  |
| 288 | 14 | C | C | A | 0.74 | 6.352844e-01 |  |
| 288 | 14 | C | C | C | 94.46 | 2.317554e-04 | \* |
| 288 | 14 | C | C | G | 1.48 | 5.510654e-01 |  |
| 288 | 14 | C | C | T | 3.32 | 4.313840e-01 |  |
| 289 | 15 | C | C | A | 4.92 | 2.550003e-01 |  |
| 289 | 15 | C | C | C | 89.62 | 3.321429e-04 | \* |
| 289 | 15 | C | C | G | 1.64 | 5.353823e-01 |  |
| 289 | 15 | C | C | T | 3.83 | 4.079363e-01 |  |
| 290 | 16 | G | G | A | 3.16 | 3.641411e-01 |  |
| 290 | 16 | G | G | C | 0.70 | 6.382613e-01 |  |
| 290 | 16 | G | G | G | 95.44 | 7.458515e-05 | \* |
| 290 | 16 | G | G | T | 0.70 | 6.132515e-01 |  |
| 291 | 17 | T | T | A | 0.00 | 8.288288e-01 |  |
| 291 | 17 | T | T | C | 0.00 | 8.735632e-01 |  |
| 291 | 17 | T | T | G | 1.83 | 5.177983e-01 |  |
| 291 | 17 | T | T | T | 98.17 | 3.580046e-04 | \* |
| 292 | 18 | G | G | A | 1.66 | 5.057817e-01 |  |
| 292 | 18 | G | G | C | 0.00 | 8.735632e-01 |  |
| 292 | 18 | G | G | G | 97.93 | 6.016281e-05 | \* |
| 292 | 18 | G | G | T | 0.41 | 6.487816e-01 |  |
| 293 | 19 | T | T | A | 2.58 | 4.113765e-01 |  |
| 293 | 19 | T | T | C | 0.74 | 6.328316e-01 |  |
| 293 | 19 | T | T | G | 0.74 | 6.385607e-01 |  |
| 293 | 19 | T | T | T | 95.94 | 4.173115e-04 | \* |
| 294 | 20 | T | T | A | 1.68 | 5.026045e-01 |  |
| 294 | 20 | T | T | C | 0.67 | 6.426223e-01 |  |
| 294 | 20 | T | T | G | 4.04 | 3.737641e-01 |  |
| 294 | 20 | T | T | T | 93.60 | 4.903765e-04 | \* |
| 295 | 21 | T | T | A | 0.71 | 6.407373e-01 |  |
| 295 | 21 | T | T | C | 0.71 | 6.375090e-01 |  |
| 295 | 21 | T | T | G | 1.06 | 5.962270e-01 |  |
| 295 | 21 | T | T | T | 97.53 | 3.741070e-04 | \* |
| 296 | 22 | G | G | A | 0.00 | 8.288288e-01 |  |
| 296 | 22 | G | G | C | 1.26 | 5.681325e-01 |  |
| 296 | 22 | G | G | G | 97.99 | 5.985064e-05 | \* |
| 296 | 22 | G | G | T | 0.75 | 6.076033e-01 |  |
| 297 | 23 | T | T | A | 1.55 | 5.184465e-01 |  |
| 297 | 23 | T | T | C | 0.39 | 6.941703e-01 |  |
| 297 | 23 | T | T | G | 1.55 | 5.438026e-01 |  |
| 297 | 23 | T | T | T | 96.51 | 4.012107e-04 | \* |
| 298 | 24 | G | G | A | 1.83 | 4.854315e-01 |  |
| 298 | 24 | G | G | C | 0.61 | 6.525066e-01 |  |
| 298 | 24 | G | G | G | 97.25 | 6.380639e-05 | \* |
| 298 | 24 | G | G | T | 0.31 | 6.649642e-01 |  |
| 299 | 25 | C | C | A | 0.38 | 7.048292e-01 |  |
| 299 | 25 | C | C | C | 94.30 | 2.346660e-04 | \* |
| 299 | 25 | C | C | G | 0.38 | 6.994426e-01 |  |
| 299 | 25 | C | C | T | 4.94 | 3.625660e-01 |  |
| 300 | 26 | A | A | A | 94.12 | 6.565068e-08 | \* |
| 300 | 26 | A | A | C | 1.60 | 5.336038e-01 |  |
| 300 | 26 | A | A | G | 4.28 | 3.621024e-01 |  |
| 300 | 26 | A | A | T | 0.00 | 7.500000e-01 |  |
| 301 | 27 | C | C | A | 0.39 | 7.028638e-01 |  |
| 301 | 27 | C | C | C | 95.33 | 2.173536e-04 | \* |
| 301 | 27 | C | C | G | 0.00 | 8.478261e-01 |  |
| 301 | 27 | C | C | T | 4.28 | 3.884760e-01 |  |
| 302 | 28 | T | T | A | 2.01 | 4.667829e-01 |  |
| 302 | 28 | T | T | C | 1.61 | 5.334062e-01 |  |
| 302 | 28 | T | T | G | 0.40 | 6.951399e-01 |  |
| 302 | 28 | T | T | T | 95.98 | 4.160766e-04 | \* |
| 303 | 29 | A | A | A | 97.98 | 3.465581e-08 | \* |
| 303 | 29 | A | A | C | 1.61 | 5.328114e-01 |  |
| 303 | 29 | A | A | G | 0.00 | 8.478261e-01 |  |
| 303 | 29 | A | A | T | 0.40 | 6.503118e-01 |  |
| 304 | 30 | C | C | A | 1.22 | 5.605990e-01 |  |
| 304 | 30 | C | C | C | 93.47 | 2.495101e-04 | \* |
| 304 | 30 | C | C | G | 1.22 | 5.773448e-01 |  |
| 304 | 30 | C | C | T | 4.08 | 3.967841e-01 |  |
| 305 | 31 | A | A | A | 96.32 | 4.562819e-08 | \* |
| 305 | 31 | A | A | C | 1.84 | 5.128273e-01 |  |
| 305 | 31 | A | A | G | 1.84 | 5.173095e-01 |  |
| 305 | 31 | A | A | T | 0.00 | 7.500000e-01 |  |
| 306 | 32 | C | C | A | 0.00 | 8.288288e-01 |  |
| 306 | 32 | C | C | C | 94.37 | 2.334580e-04 | \* |
| 306 | 32 | C | C | G | 1.41 | 5.578521e-01 |  |
| 306 | 32 | C | C | T | 4.23 | 3.907424e-01 |  |
| 307 | 33 | G | G | A | 2.22 | 4.450496e-01 |  |
| 307 | 33 | G | G | C | 0.00 | 8.735632e-01 |  |
| 307 | 33 | G | G | G | 97.78 | 6.095554e-05 | \* |
| 307 | 33 | G | G | T | 0.00 | 7.500000e-01 |  |
| 308 | 34 | G | G | A | 0.70 | 6.420434e-01 |  |
| 308 | 34 | G | G | C | 0.00 | 8.735632e-01 |  |
| 308 | 34 | G | G | G | 98.95 | 5.509243e-05 | \* |
| 308 | 34 | G | G | T | 0.35 | 6.581489e-01 |  |
| 309 | 35 | G | G | A | 1.67 | 5.037536e-01 |  |
| 309 | 35 | G | G | C | 0.84 | 6.187800e-01 |  |
| 309 | 35 | G | G | G | 97.49 | 6.248949e-05 | \* |
| 309 | 35 | G | G | T | 0.00 | 7.500000e-01 |  |
| 310 | 36 | C | C | A | 0.93 | 6.037174e-01 |  |
| 310 | 36 | C | C | C | 92.56 | 2.669623e-04 | \* |
| 310 | 36 | C | C | G | 3.26 | 4.165194e-01 |  |
| 310 | 36 | C | C | T | 3.26 | 4.345809e-01 |  |
| 311 | 37 | A | A | A | 94.79 | 5.872922e-08 | \* |
| 311 | 37 | A | A | C | 2.08 | 4.932255e-01 |  |
| 311 | 37 | A | A | G | 3.12 | 4.243823e-01 |  |
| 311 | 37 | A | A | T | 0.00 | 7.500000e-01 |  |
| 312 | 38 | C | C | A | 0.37 | 7.079459e-01 |  |
| 312 | 38 | C | C | C | 94.87 | 2.248717e-04 | \* |
| 312 | 38 | C | C | G | 2.20 | 4.880161e-01 |  |
| 312 | 38 | C | C | T | 2.56 | 4.712177e-01 |  |
| 313 | 39 | C | C | A | 1.18 | 5.672704e-01 |  |
| 313 | 39 | C | C | C | 93.73 | 2.448158e-04 | \* |
| 313 | 39 | C | C | G | 1.96 | 5.070765e-01 |  |
| 313 | 39 | C | C | T | 3.14 | 4.404971e-01 |  |
| 314 | 40 | C | C | A | 1.16 | 5.691951e-01 |  |
| 314 | 40 | C | C | C | 91.47 | 2.893624e-04 | \* |
| 314 | 40 | C | C | G | 3.49 | 4.030967e-01 |  |
| 314 | 40 | C | C | T | 3.88 | 4.056871e-01 |  |
| 315 | 41 | T | T | A | 2.56 | 4.130588e-01 |  |
| 315 | 41 | T | T | C | 3.59 | 3.979807e-01 |  |
| 315 | 41 | T | T | G | 1.54 | 5.449532e-01 |  |
| 315 | 41 | T | T | T | 92.31 | 5.362584e-04 | \* |
| 316 | 42 | G | G | A | 1.52 | 5.221967e-01 |  |
| 316 | 42 | G | G | C | 0.91 | 6.087508e-01 |  |
| 316 | 42 | G | G | G | 97.26 | 6.371439e-05 | \* |
| 316 | 42 | G | G | T | 0.30 | 6.652624e-01 |  |
| 317 | 43 | A | A | A | 95.02 | 5.659551e-08 | \* |
| 317 | 43 | A | A | C | 0.62 | 6.506357e-01 |  |
| 317 | 43 | A | A | G | 2.18 | 4.893472e-01 |  |
| 317 | 43 | A | A | T | 2.18 | 4.940936e-01 |  |
| 318 | 44 | C | C | A | 0.00 | 8.288288e-01 |  |
| 318 | 44 | C | C | C | 94.40 | 2.328738e-04 | \* |
| 318 | 44 | C | C | G | 2.80 | 4.449940e-01 |  |
| 318 | 44 | C | C | T | 2.80 | 4.581191e-01 |  |
| 319 | 45 | C | C | A | 3.70 | 3.252935e-01 |  |
| 319 | 45 | C | C | C | 88.36 | 3.647218e-04 | \* |
| 319 | 45 | C | C | G | 5.29 | 3.177758e-01 |  |
| 319 | 45 | C | C | T | 2.65 | 4.666204e-01 |  |
| 320 | 46 | G | G | A | 6.25 | 1.968835e-01 |  |
| 320 | 46 | G | G | C | 1.34 | 5.593990e-01 |  |
| 320 | 46 | G | G | G | 92.41 | 9.688204e-05 | \* |
| 320 | 46 | G | G | T | 0.00 | 7.500000e-01 |  |
| 321 | 47 | A | A | A | 99.40 | 2.743054e-08 | \* |
| 321 | 47 | A | A | C | 0.60 | 6.543293e-01 |  |
| 321 | 47 | A | A | G | 0.00 | 8.478261e-01 |  |
| 321 | 47 | A | A | T | 0.00 | 7.500000e-01 |  |
| 322 | 48 | C | C | A | 0.44 | 6.917791e-01 |  |
| 322 | 48 | C | C | C | 94.71 | 2.275225e-04 | \* |
| 322 | 48 | C | C | G | 0.44 | 6.875904e-01 |  |
| 322 | 48 | C | C | T | 4.41 | 3.833758e-01 |  |
| 323 | 49 | G | G | A | 7.97 | 1.421896e-01 |  |
| 323 | 49 | G | G | C | 1.45 | 5.483321e-01 |  |
| 323 | 49 | G | G | G | 89.86 | 1.208526e-04 | \* |
| 323 | 49 | G | G | T | 0.72 | 6.107435e-01 |  |
| 324 | 50 | G | G | A | 0.00 | 8.288288e-01 |  |
| 324 | 50 | G | G | C | 1.23 | 5.708884e-01 |  |
| 324 | 50 | G | G | G | 98.46 | 5.746637e-05 | \* |
| 324 | 50 | G | G | T | 0.31 | 6.646632e-01 |  |
| 325 | 51 | C | C | A | 0.53 | 6.731862e-01 |  |
| 325 | 51 | C | C | C | 95.74 | 2.107903e-04 | \* |
| 325 | 51 | C | C | G | 0.00 | 8.478261e-01 |  |
| 325 | 51 | C | C | T | 3.72 | 4.124982e-01 |  |
| 326 | 52 | A | A | A | 81.11 | 5.651573e-07 | \* |
| 326 | 52 | A | A | C | 2.22 | 4.826991e-01 |  |
| 326 | 52 | A | A | G | 12.22 | 1.443449e-01 |  |
| 326 | 52 | A | A | T | 4.44 | 3.818007e-01 |  |
| 327 | 53 | A | A | A | 97.98 | 3.467913e-08 | \* |
| 327 | 53 | A | A | C | 0.00 | 8.735632e-01 |  |
| 327 | 53 | A | A | G | 0.51 | 6.757803e-01 |  |
| 327 | 53 | A | A | T | 1.52 | 5.398285e-01 |  |
| 328 | 54 | G | G | A | 1.18 | 5.672704e-01 |  |
| 328 | 54 | G | G | C | 1.76 | 5.192934e-01 |  |
| 328 | 54 | G | G | G | 95.88 | 7.178259e-05 | \* |
| 328 | 54 | G | G | T | 1.18 | 5.672159e-01 |  |
| 329 | 55 | A | A | A | 95.90 | 4.888622e-08 | \* |
| 329 | 55 | A | A | C | 0.00 | 8.735632e-01 |  |
| 329 | 55 | A | A | G | 4.10 | 3.708704e-01 |  |
| 329 | 55 | A | A | T | 0.00 | 7.500000e-01 |  |
| 330 | 56 | A | A | A | 99.47 | 2.710853e-08 | \* |
| 330 | 56 | A | A | C | 0.00 | 8.735632e-01 |  |
| 330 | 56 | A | A | G | 0.00 | 8.478261e-01 |  |
| 330 | 56 | A | A | T | 0.53 | 6.335432e-01 |  |
| 331 | 57 | G | G | A | 0.00 | 8.288288e-01 |  |
| 331 | 57 | G | G | C | 0.00 | 8.735632e-01 |  |
| 331 | 57 | G | G | G | 98.39 | 5.783629e-05 | \* |
| 331 | 57 | G | G | T | 1.61 | 5.325206e-01 |  |
| 332 | 58 | T | T | A | 4.96 | 2.528100e-01 |  |
| 332 | 58 | T | T | C | 0.00 | 8.735632e-01 |  |
| 332 | 58 | T | T | G | 0.38 | 6.991468e-01 |  |
| 332 | 58 | T | T | T | 94.66 | 4.559745e-04 | \* |
| 333 | 59 | T | A | A | 97.44 | 3.793943e-08 | \* |
| 333 | 59 | T | A | C | 1.03 | 5.944388e-01 |  |
| 333 | 59 | T | A | G | 1.54 | 5.449532e-01 |  |
| 333 | 59 | T | A | T | 0.00 | 7.500000e-01 |  |
| 334 | 60 | C | C | A | 0.00 | 8.288288e-01 |  |
| 334 | 60 | C | C | C | 93.94 | 2.409632e-04 | \* |
| 334 | 60 | C | C | G | 0.51 | 6.757803e-01 |  |
| 334 | 60 | C | C | T | 5.56 | 3.408056e-01 |  |
| 335 | 61 | G | G | A | 12.50 | 6.211925e-02 |  |
| 335 | 61 | G | G | C | 0.62 | 6.503190e-01 |  |
| 335 | 61 | G | G | G | 83.75 | 2.051966e-04 | \* |
| 335 | 61 | G | G | T | 3.12 | 4.411165e-01 |  |
| 336 | 62 | A | A | A | 97.64 | 3.665738e-08 | \* |
| 336 | 62 | A | A | C | 0.26 | 7.238917e-01 |  |
| 336 | 62 | A | A | G | 0.79 | 6.317897e-01 |  |
| 336 | 62 | A | A | T | 1.31 | 5.560869e-01 |  |
| 337 | 63 | C | C | A | 0.85 | 6.157521e-01 |  |
| 337 | 63 | C | C | C | 94.44 | 2.321079e-04 | \* |
| 337 | 63 | C | C | G | 1.28 | 5.710752e-01 |  |
| 337 | 63 | C | C | T | 3.42 | 4.266651e-01 |  |
| 338 | 64 | A | A | A | 93.20 | 7.635611e-08 | \* |
| 338 | 64 | A | A | C | 0.97 | 6.012018e-01 |  |
| 338 | 64 | A | A | G | 1.94 | 5.086693e-01 |  |
| 338 | 64 | A | A | T | 3.88 | 4.053557e-01 |  |
| 339 | 65 | G | G | A | 2.60 | 4.101043e-01 |  |
| 339 | 65 | G | G | C | 0.87 | 6.148457e-01 |  |
| 339 | 65 | G | G | G | 96.54 | 6.784148e-05 | \* |
| 339 | 65 | G | G | T | 0.00 | 7.500000e-01 |  |
| 340 | 66 | C | C | A | 0.00 | 8.288288e-01 |  |
| 340 | 66 | C | C | C | 95.02 | 2.224295e-04 | \* |
| 340 | 66 | C | C | G | 1.53 | 5.455238e-01 |  |
| 340 | 66 | C | C | T | 3.45 | 4.252596e-01 |  |
| 341 | 67 | T | T | A | 2.83 | 3.898660e-01 |  |
| 341 | 67 | T | T | C | 0.81 | 6.225328e-01 |  |
| 341 | 67 | T | T | G | 2.43 | 4.706531e-01 |  |
| 341 | 67 | T | T | T | 93.93 | 4.795152e-04 | \* |
| 342 | 68 | C | C | A | 0.45 | 6.892697e-01 |  |
| 342 | 68 | C | C | C | 93.67 | 2.459136e-04 | \* |
| 342 | 68 | C | C | G | 5.88 | 2.952705e-01 |  |
| 342 | 68 | C | C | T | 0.00 | 7.500000e-01 |  |
| 343 | 69 | C | C | A | 0.00 | 8.288288e-01 |  |
| 343 | 69 | C | C | C | 94.79 | 2.262109e-04 | \* |
| 343 | 69 | C | C | G | 0.00 | 8.478261e-01 |  |
| 343 | 69 | C | C | T | 5.21 | 3.529023e-01 |  |
| 344 | 70 | C | C | A | 0.00 | 8.288288e-01 |  |
| 344 | 70 | C | C | C | 98.97 | 1.660509e-04 | \* |
| 344 | 70 | C | C | G | 1.03 | 5.997404e-01 |  |
| 344 | 70 | C | C | T | 0.00 | 7.500000e-01 |  |
| 345 | 71 | G | G | A | 4.02 | 3.050278e-01 |  |
| 345 | 71 | G | G | C | 1.01 | 5.969585e-01 |  |
| 345 | 71 | G | G | G | 94.97 | 7.763146e-05 | \* |
| 345 | 71 | G | G | T | 0.00 | 7.500000e-01 |  |
| 346 | 72 | C | C | A | 0.00 | 8.288288e-01 |  |
| 346 | 72 | C | C | C | 95.02 | 2.223353e-04 | \* |
| 346 | 72 | C | C | G | 0.50 | 6.771191e-01 |  |
| 346 | 72 | C | C | T | 4.48 | 3.804742e-01 |  |
| 347 | 73 | G | G | A | 48.72 | 1.247283e-04 | \* |
| 347 | 73 | G | G | C | 1.03 | 5.944388e-01 |  |
| 347 | 73 | G | G | G | 49.74 | 4.104277e-03 | \* |
| 347 | 73 | G | G | T | 0.51 | 6.356026e-01 |  |
| 348 | 74 | A | A | A | 73.58 | 1.973045e-06 | \* |
| 348 | 74 | A | A | C | 1.04 | 5.931516e-01 |  |
| 348 | 74 | A | A | G | 25.39 | 3.873660e-02 |  |
| 348 | 74 | A | A | T | 0.00 | 7.500000e-01 |  |
| 349 | 75 | C | G | A | 0.32 | 7.189820e-01 |  |
| 349 | 75 | C | G | C | 45.86 | 9.251718e-03 | \* |
| 349 | 75 | C | G | G | 51.27 | 3.578022e-03 | \* |
| 349 | 75 | C | G | T | 2.55 | 4.721503e-01 |  |

## For use in R

If you want to work with the results in R, here is output that you can copy and paste in your terminal to get:

The base information:

```
structure(list(focal.base = c("A", "C", "G", "T"), avg.percsignal = c(90.248244922338, 
89.9513541381861, 84.7571222749392, 85.8909293050591), avg.areasignal = c(221.205882352941, 
244.904761904762, 253.910714285714, 248.270833333333), crit.perc.area = c(22.8940637932591, 
44.8704366631808, 39.9068586189995, 50.6373786997772), mu = c(4.35382432677365, 
6.77723111901579, 6.5140611527423, 8.95553110802859), fillibens = c(0.885229769847824, 
0.878211807420843, 0.923344956651763, 0.955417720748319)), .Names = c("focal.base", 
"avg.percsignal", "avg.areasignal", "crit.perc.area", "mu", "fillibens"
), row.names = c(NA, -4L), class = "data.frame")
```

the data.frame that contains information on the guide region:

```
structure(list(A.area = c(161, 219, 3, 438, 4, 13, 8, 8, 0, 2, 
175, 4, 436, 2, 9, 9, 0, 8, 7, 5, 2, 0, 4, 6, 1, 176, 1, 5, 243, 
3, 157, 0, 4, 2, 4, 2, 182, 1, 3, 3, 5, 5, 305, 0, 7, 14, 331, 
1, 11, 0, 1, 73, 194, 2, 234, 188, 0, 13, 190, 0, 20, 373, 2, 
96, 6, 0, 7, 1, 0, 0, 8, 0, 95, 142, 1), C.area = c(1, 6, 2, 
3, 238, 1, 5, 5, 181, 254, 1, 5, 3, 256, 164, 2, 0, 0, 2, 2, 
2, 5, 1, 2, 248, 3, 245, 4, 4, 229, 3, 201, 0, 0, 2, 199, 4, 
259, 239, 236, 7, 3, 2, 236, 167, 3, 2, 215, 2, 4, 180, 2, 0, 
3, 0, 0, 0, 0, 2, 186, 1, 1, 221, 1, 2, 248, 2, 207, 273, 96, 
2, 191, 2, 2, 144), G.area = c(4, 3, 197, 10, 5, 5, 528, 326, 
4, 4, 2, 202, 4, 4, 3, 272, 4, 473, 2, 12, 3, 390, 4, 318, 1, 
8, 0, 1, 0, 3, 3, 3, 176, 283, 233, 7, 6, 6, 5, 9, 3, 320, 7, 
7, 10, 207, 0, 1, 124, 320, 0, 11, 1, 163, 10, 0, 122, 1, 3, 
1, 134, 3, 3, 2, 223, 4, 6, 13, 0, 1, 189, 1, 97, 49, 161), T.area = c(1, 
0, 0, 0, 7, 241, 2, 1, 8, 11, 5, 1, 6, 9, 7, 2, 214, 2, 260, 
278, 276, 3, 249, 1, 13, 0, 11, 239, 1, 10, 0, 9, 0, 1, 0, 7, 
0, 7, 8, 10, 180, 1, 7, 7, 5, 0, 0, 10, 1, 1, 7, 4, 3, 2, 0, 
1, 2, 248, 0, 11, 5, 5, 8, 4, 0, 9, 232, 0, 15, 0, 0, 9, 1, 0, 
8), Tot.area = c(167, 228, 202, 451, 254, 260, 543, 340, 193, 
271, 183, 212, 449, 271, 183, 285, 218, 483, 271, 297, 283, 398, 
258, 327, 263, 187, 257, 249, 248, 245, 163, 213, 180, 286, 239, 
215, 192, 273, 255, 258, 195, 329, 321, 250, 189, 224, 333, 227, 
138, 325, 188, 90, 198, 170, 244, 189, 124, 262, 195, 198, 160, 
382, 234, 103, 231, 261, 247, 221, 288, 97, 199, 201, 195, 193, 
314), A.perc = c(96.4071856287425, 96.0526315789474, 1.48514851485149, 
97.1175166297118, 1.5748031496063, 5, 1.47329650092081, 2.35294117647059, 
0, 0.738007380073801, 95.6284153005465, 1.88679245283019, 97.1046770601336, 
0.738007380073801, 4.91803278688525, 3.15789473684211, 0, 1.65631469979296, 
2.5830258302583, 1.68350168350168, 0.706713780918728, 0, 1.55038759689922, 
1.8348623853211, 0.380228136882129, 94.1176470588235, 0.389105058365759, 
2.00803212851406, 97.9838709677419, 1.22448979591837, 96.319018404908, 
0, 2.22222222222222, 0.699300699300699, 1.67364016736402, 0.930232558139535, 
94.7916666666667, 0.366300366300366, 1.17647058823529, 1.16279069767442, 
2.56410256410256, 1.51975683890578, 95.0155763239875, 0, 3.7037037037037, 
6.25, 99.3993993993994, 0.440528634361233, 7.97101449275362, 
0, 0.531914893617021, 81.1111111111111, 97.979797979798, 1.17647058823529, 
95.9016393442623, 99.4708994708995, 0, 4.9618320610687, 97.4358974358974, 
0, 12.5, 97.6439790575916, 0.854700854700855, 93.2038834951456, 
2.5974025974026, 0, 2.83400809716599, 0.452488687782805, 0, 0, 
4.02010050251256, 0, 48.7179487179487, 73.5751295336788, 0.318471337579618
), C.perc = c(0.598802395209581, 2.63157894736842, 0.99009900990099, 
0.665188470066519, 93.7007874015748, 0.384615384615385, 0.920810313075506, 
1.47058823529412, 93.7823834196891, 93.7269372693727, 0.546448087431694, 
2.35849056603774, 0.66815144766147, 94.4649446494465, 89.6174863387978, 
0.701754385964912, 0, 0, 0.738007380073801, 0.673400673400673, 
0.706713780918728, 1.25628140703518, 0.387596899224806, 0.611620795107034, 
94.2965779467681, 1.60427807486631, 95.3307392996109, 1.60642570281124, 
1.61290322580645, 93.469387755102, 1.84049079754601, 94.3661971830986, 
0, 0, 0.836820083682008, 92.5581395348837, 2.08333333333333, 
94.8717948717949, 93.7254901960784, 91.4728682170543, 3.58974358974359, 
0.911854103343465, 0.623052959501558, 94.4, 88.3597883597884, 
1.33928571428571, 0.600600600600601, 94.7136563876652, 1.44927536231884, 
1.23076923076923, 95.7446808510638, 2.22222222222222, 0, 1.76470588235294, 
0, 0, 0, 0, 1.02564102564103, 93.9393939393939, 0.625, 0.261780104712042, 
94.4444444444444, 0.970873786407767, 0.865800865800866, 95.0191570881226, 
0.809716599190283, 93.6651583710407, 94.7916666666667, 98.9690721649485, 
1.00502512562814, 95.0248756218905, 1.02564102564103, 1.03626943005181, 
45.859872611465), G.perc = c(2.39520958083832, 1.31578947368421, 
97.5247524752475, 2.21729490022173, 1.96850393700787, 1.92307692307692, 
97.2375690607735, 95.8823529411765, 2.07253886010363, 1.4760147601476, 
1.09289617486339, 95.2830188679245, 0.89086859688196, 1.4760147601476, 
1.63934426229508, 95.4385964912281, 1.8348623853211, 97.9296066252588, 
0.738007380073801, 4.04040404040404, 1.06007067137809, 97.9899497487437, 
1.55038759689922, 97.2477064220184, 0.380228136882129, 4.27807486631016, 
0, 0.401606425702811, 0, 1.22448979591837, 1.84049079754601, 
1.40845070422535, 97.7777777777778, 98.9510489510489, 97.489539748954, 
3.25581395348837, 3.125, 2.1978021978022, 1.96078431372549, 3.48837209302326, 
1.53846153846154, 97.2644376899696, 2.18068535825545, 2.8, 5.29100529100529, 
92.4107142857143, 0, 0.440528634361233, 89.8550724637681, 98.4615384615385, 
0, 12.2222222222222, 0.505050505050505, 95.8823529411765, 4.0983606557377, 
0, 98.3870967741936, 0.381679389312977, 1.53846153846154, 0.505050505050505, 
83.75, 0.785340314136126, 1.28205128205128, 1.94174757281553, 
96.5367965367965, 1.53256704980843, 2.42914979757085, 5.88235294117647, 
0, 1.03092783505155, 94.9748743718593, 0.497512437810945, 49.7435897435897, 
25.3886010362694, 51.2738853503185), T.perc = c(0.598802395209581, 
0, 0, 0, 2.75590551181102, 92.6923076923077, 0.368324125230203, 
0.294117647058824, 4.14507772020725, 4.0590405904059, 2.73224043715847, 
0.471698113207547, 1.33630289532294, 3.3210332103321, 3.82513661202186, 
0.701754385964912, 98.1651376146789, 0.41407867494824, 95.9409594095941, 
93.6026936026936, 97.5265017667844, 0.753768844221106, 96.5116279069767, 
0.305810397553517, 4.94296577946768, 0, 4.28015564202335, 95.9839357429719, 
0.403225806451613, 4.08163265306122, 0, 4.22535211267606, 0, 
0.34965034965035, 0, 3.25581395348837, 0, 2.56410256410256, 3.13725490196078, 
3.87596899224806, 92.3076923076923, 0.303951367781155, 2.18068535825545, 
2.8, 2.64550264550265, 0, 0, 4.40528634361233, 0.72463768115942, 
0.307692307692308, 3.72340425531915, 4.44444444444444, 1.51515151515152, 
1.17647058823529, 0, 0.529100529100529, 1.61290322580645, 94.6564885496183, 
0, 5.55555555555556, 3.125, 1.30890052356021, 3.41880341880342, 
3.88349514563107, 0, 3.44827586206897, 93.9271255060729, 0, 5.20833333333333, 
0, 0, 4.47761194029851, 0.512820512820513, 0, 2.54777070063694
), base.call = c("A", "A", "G", "A", "C", "T", "G", "G", "C", 
"C", "A", "G", "A", "C", "C", "G", "T", "G", "T", "T", "T", "G", 
"T", "G", "C", "A", "C", "T", "A", "C", "A", "C", "G", "G", "G", 
"C", "A", "C", "C", "C", "T", "G", "A", "C", "C", "G", "A", "C", 
"G", "G", "C", "A", "A", "G", "A", "A", "G", "T", "A", "C", "G", 
"A", "C", "A", "G", "C", "T", "C", "C", "C", "G", "C", "G", "A", 
"G"), index = 275:349, guide.seq = c("A", "A", "G", "A", "C", 
"T", "G", "G", "C", "C", "A", "G", "A", "C", "C", "G", "T", "G", 
"T", "T", "T", "G", "T", "G", "C", "A", "C", "T", "A", "C", "A", 
"C", "G", "G", "G", "C", "A", "C", "C", "C", "T", "G", "A", "C", 
"C", "G", "A", "C", "G", "G", "C", "A", "A", "G", "A", "A", "G", 
"T", "T", "C", "G", "A", "C", "A", "G", "C", "T", "C", "C", "C", 
"G", "C", "G", "A", "C"), T.pval = c(0.625033294756861, 0.749999999999922, 
0.749999999999922, 0.749999999999922, 0.460516379854526, 0.000522197057414298, 
0.655360789879512, 0.666852915321709, 0.394099266399289, 0.397746992908326, 
0.461812395618293, 0.640939661750975, 0.553855510919997, 0.4313839863904, 
0.407936336398254, 0.613251502967563, 0.000358004568421721, 0.648781607072653, 
0.000417311475021886, 0.000490376530134062, 0.000374107033647375, 
0.607603309354156, 0.000401210680294195, 0.664964232196917, 0.362565998755891, 
0.749999999999922, 0.388475981612825, 0.000416076595860737, 0.650311801724392, 
0.396784077866056, 0.749999999999922, 0.390742445646682, 0.749999999999922, 
0.658148907988542, 0.749999999999922, 0.434580933840537, 0.749999999999922, 
0.471217709186306, 0.440497052152479, 0.405687088614549, 0.000536258382895616, 
0.665262379605723, 0.494093591642759, 0.458119147357223, 0.466620382792909, 
0.749999999999922, 0.749999999999922, 0.383375846418714, 0.610743499506949, 
0.664663220214623, 0.412498212148711, 0.381800731212596, 0.539828501844565, 
0.567215892925161, 0.749999999999922, 0.633543229803137, 0.5325206014382, 
0.000455974458405173, 0.749999999999922, 0.340805649234328, 0.441116469955283, 
0.556086948155578, 0.426665122483338, 0.405355747256437, 0.749999999999922, 
0.42525955647272, 0.000479515242829409, 0.749999999999922, 0.35290226610238, 
0.749999999999922, 0.749999999999922, 0.380474231758338, 0.635602633287485, 
0.749999999999922, 0.472150346730469), C.pval = c(0.654628499295476, 
0.454105040496228, 0.598802281853076, 0.643905221900301, 0.000245264664276768, 
0.694806527889298, 0.607586894848918, 0.546249397087774, 0.000243785066203706, 
0.000244789499359466, 0.663576202493468, 0.47280053154257, 0.643441307221736, 
0.000231755439790482, 0.00033214290376582, 0.638261289935317, 
0.87356321839079, 0.87356321839079, 0.632831633993551, 0.642622342493784, 
0.637509044415074, 0.568132494338376, 0.694170274892797, 0.65250663428703, 
0.000234665973980031, 0.533603751910304, 0.000217353600018177, 
0.533406211146392, 0.532811428593049, 0.000249510146058229, 0.512827280682885, 
0.000233458030437061, 0.87356321839079, 0.87356321839079, 0.618779965422332, 
0.000266962287485795, 0.493225547389149, 0.000224871659868953, 
0.000244815769599782, 0.000289362428357975, 0.397980661128914, 
0.608750821066075, 0.650635657541391, 0.000232873791208621, 0.000364721846978111, 
0.559398984636786, 0.654329277829434, 0.000227522470445551, 0.548332087711843, 
0.570888377014657, 0.00021079034779159, 0.482699075699846, 0.87356321839079, 
0.51929337778733, 0.87356321839079, 0.87356321839079, 0.87356321839079, 
0.87356321839079, 0.594438755002642, 0.000240963244371617, 0.650318977039398, 
0.723891729607466, 0.000232107868412879, 0.601201848642043, 0.614845662041591, 
0.000222429529891932, 0.622532754591279, 0.000245913571370715, 
0.00022621090015762, 0.000166050871583834, 0.596958506220618, 
0.000222335301693244, 0.594438755002642, 0.593151616342988, 0.00925171771275957
), G.pval = c(0.473130187120841, 0.567474886001674, 6.23000300055132e-05, 
0.486508332549405, 0.506433329049751, 0.510240992816356, 6.38621953406027e-05, 
7.1782592150349e-05, 0.49791676487597, 0.551065431316033, 0.592333073458913, 
7.5593538959362e-05, 0.617437048264667, 0.551065431316033, 0.535382301662092, 
7.45851470724812e-05, 0.51779826972535, 6.01628112129049e-05, 
0.638560672119784, 0.373764089147261, 0.596227022480736, 5.9850635005354e-05, 
0.543802613652468, 6.38063871802874e-05, 0.699442594843575, 0.362102383972293, 
0.847826086956522, 0.695139882175834, 0.847826086956522, 0.577344775828254, 
0.5173094516281, 0.557852109364751, 6.09555362061087e-05, 5.50924276765397e-05, 
6.24894876963822e-05, 0.416519353820566, 0.424382289462615, 0.488016092403928, 
0.507076473293878, 0.403096724737949, 0.544953163104837, 6.37143855031219e-05, 
0.489347224544953, 0.444994046148786, 0.317775802434154, 9.68820446645147e-05, 
0.847826086956522, 0.687590392065543, 0.000120852598781251, 5.74663720613877e-05, 
0.847826086956522, 0.144344854786846, 0.675780315542473, 7.1782592150349e-05, 
0.370870426822136, 0.847826086956522, 5.78362886280992e-05, 0.699146754254689, 
0.544953163104837, 0.675780315542473, 0.00020519657306739, 0.631789706172199, 
0.571075181341268, 0.508669290862414, 6.78414756059009e-05, 0.54552377757537, 
0.470653101190798, 0.295270479552875, 0.847826086956522, 0.599740411990201, 
7.76314635506647e-05, 0.67711906346712, 0.00410427651350886, 
0.0387365993844562, 0.00357802224187131), A.pval = c(4.49682663328232e-08, 
4.76815337213665e-08, 0.526483389360016, 3.99884768631509e-08, 
0.515485991045199, 0.250918517966754, 0.527963678064808, 0.432447550706141, 
0.828828828828829, 0.635284424652651, 5.11440996220358e-08, 0.479731675820056, 
4.00733950467469e-08, 0.635284424652651, 0.255000292533354, 0.364141064036988, 
0.828828828828829, 0.505781677780122, 0.411376513016873, 0.502604526203358, 
0.640737295212871, 0.828828828828829, 0.518446487689125, 0.485431487484067, 
0.704829177759576, 6.56506845553295e-08, 0.70286382580552, 0.466782878568683, 
3.46558087782611e-08, 0.560599027705863, 4.56281852301288e-08, 
0.828828828828829, 0.445049596952806, 0.642043391144974, 0.503753601823431, 
0.603717410652099, 5.87292185993959e-08, 0.707945914345925, 0.567270408084174, 
0.569195086919372, 0.413058785284173, 0.522196736586211, 5.6595508168833e-08, 
0.828828828828829, 0.325293518926463, 0.196883547155872, 2.74305438363598e-08, 
0.691779147513996, 0.142189552599671, 0.828828828828829, 0.673186155113027, 
5.65157283638484e-07, 3.46791341199193e-08, 0.567270408084174, 
4.88862169634174e-08, 2.71085348613198e-08, 0.828828828828829, 
0.252809975622795, 3.79394269334199e-08, 0.828828828828829, 0.062119250837971, 
3.66573772447865e-08, 0.615752106599141, 7.63561145422642e-08, 
0.410104281390559, 0.828828828828829, 0.389866043897071, 0.689269711550644, 
0.828828828828829, 0.828828828828829, 0.30502779135546, 0.828828828828829, 
0.000124728325194723, 1.97304514515384e-06, 0.718981980341318
), guide.position = 1:75), .Names = c("A.area", "C.area", "G.area", 
"T.area", "Tot.area", "A.perc", "C.perc", "G.perc", "T.perc", 
"base.call", "index", "guide.seq", "T.pval", "C.pval", "G.pval", 
"A.pval", "guide.position"), row.names = 275:349, class = "data.frame")
```

*Report generated using EditR v1.0.8*
