# Supplementary material for: Mechanistic and genetic basis of single-strand templated repair at Cas12a-induced DNA breaks in Chlamydomonas reinhardtii
Source: Nat Commun. 2021 Nov 19;12:6751. doi: 10.1038/s41467-021-27004-1 (PMC8604939; doi:10.1038/s41467-021-27004-1)
Supplement: Supplementary file 22 — Source Data [file 41467_2021_27004_MOESM22_ESM.zip › Source Data/EditR analysis/EditR outputs/Controls for normalization/Positive control (-16).html]

EditR v1.0.8 report


# EditR v1.0.8 report

- Data QA
  - Filtering data
  - Percent noise peak area
  - Base information
- Predicted editing
  - Editing bar plot
  - Editing table plot
  - Table of editing results
- For use in R

## Data QA

### Filtering data

What the data looked like prefiltering:

and the post filtering signal / noise plot:

### Percent noise peak area

### Base information

Here’s information about the signal of each base, the critical percent value where any higher value would be called as significant, and Filliben’s correlation for how well the noise was modelled by the zero adjusted gamma distribution.

| Base | Average percent signal | Average peak area | Critical percent value | model mu | Fillibens correlation |
| --- | --- | --- | --- | --- | --- |
| A | 85.21017 | 278.7941 | 20.47102 | 4.162550 | 0.9492854 |
| C | 88.87074 | 285.9853 | 52.30478 | 8.481915 | 0.9428042 |
| G | 83.18071 | 283.3571 | 34.78082 | 5.935763 | 0.9556335 |
| T | 86.05984 | 325.4884 | 22.07306 | 4.527405 | 0.9458192 |

## Predicted editing

### Editing bar plot

### Editing table plot

### Table of editing results


Here’s the entire guide region

| Sanger position | Guide position | Guide sequence | Sanger base call | Focal base | Focal base peak area | p value |  |
| --- | --- | --- | --- | --- | --- | --- | --- |
| 277 | 1 | A | A | A | 94.50 | 8.257694e-09 | \* |
| 277 | 1 | A | A | C | 4.00 | 4.438058e-01 |  |
| 277 | 1 | A | A | G | 1.50 | 4.766368e-01 |  |
| 277 | 1 | A | A | T | 0.00 | 7.446809e-01 |  |
| 278 | 2 | A | A | A | 94.30 | 8.578320e-09 | \* |
| 278 | 2 | A | A | C | 2.28 | 5.449791e-01 |  |
| 278 | 2 | A | A | G | 0.76 | 5.566942e-01 |  |
| 278 | 2 | A | A | T | 2.66 | 3.830954e-01 |  |
| 279 | 3 | G | G | A | 1.97 | 4.323844e-01 |  |
| 279 | 3 | G | G | C | 0.39 | 7.429037e-01 |  |
| 279 | 3 | G | G | G | 94.49 | 2.826250e-05 | \* |
| 279 | 3 | G | G | T | 3.15 | 3.458936e-01 |  |
| 280 | 4 | A | A | A | 96.27 | 5.931412e-09 | \* |
| 280 | 4 | A | A | C | 0.59 | 7.109499e-01 |  |
| 280 | 4 | A | A | G | 1.38 | 4.882398e-01 |  |
| 280 | 4 | A | A | T | 1.77 | 4.648032e-01 |  |
| 281 | 5 | C | C | A | 1.00 | 5.491474e-01 |  |
| 281 | 5 | C | C | C | 96.01 | 4.959815e-04 | \* |
| 281 | 5 | C | C | G | 1.99 | 4.357167e-01 |  |
| 281 | 5 | C | C | T | 1.00 | 5.553447e-01 |  |
| 282 | 6 | T | T | A | 3.59 | 2.992442e-01 |  |
| 282 | 6 | T | T | C | 3.29 | 4.810205e-01 |  |
| 282 | 6 | T | T | G | 2.40 | 4.069292e-01 |  |
| 282 | 6 | T | T | T | 90.72 | 5.681722e-08 | \* |
| 283 | 7 | G | G | A | 0.30 | 6.677160e-01 |  |
| 283 | 7 | G | G | C | 2.58 | 5.245217e-01 |  |
| 283 | 7 | G | G | G | 96.66 | 2.293770e-05 | \* |
| 283 | 7 | G | G | T | 0.46 | 6.376133e-01 |  |
| 284 | 8 | G | G | A | 2.21 | 4.084988e-01 |  |
| 284 | 8 | G | G | C | 0.74 | 6.902513e-01 |  |
| 284 | 8 | G | G | G | 96.31 | 2.371501e-05 | \* |
| 284 | 8 | G | G | T | 0.74 | 5.921001e-01 |  |
| 285 | 9 | C | C | A | 0.83 | 5.743594e-01 |  |
| 285 | 9 | C | C | C | 94.21 | 5.600405e-04 | \* |
| 285 | 9 | C | C | G | 3.31 | 3.522517e-01 |  |
| 285 | 9 | C | C | T | 1.65 | 4.769316e-01 |  |
| 286 | 10 | C | C | A | 0.60 | 6.120300e-01 |  |
| 286 | 10 | C | C | C | 96.73 | 4.726819e-04 | \* |
| 286 | 10 | C | C | G | 0.60 | 5.799358e-01 |  |
| 286 | 10 | C | C | T | 2.08 | 4.336634e-01 |  |
| 287 | 11 | A | A | A | 95.71 | 6.578316e-09 | \* |
| 287 | 11 | A | A | C | 2.86 | 5.067829e-01 |  |
| 287 | 11 | A | A | G | 1.43 | 4.832075e-01 |  |
| 287 | 11 | A | A | T | 0.00 | 7.446809e-01 |  |
| 288 | 12 | G | G | A | 1.52 | 4.820442e-01 |  |
| 288 | 12 | G | G | C | 3.41 | 4.745744e-01 |  |
| 288 | 12 | G | G | G | 94.70 | 2.770115e-05 | \* |
| 288 | 12 | G | G | T | 0.38 | 6.513571e-01 |  |
| 289 | 13 | A | A | A | 97.07 | 5.103424e-09 | \* |
| 289 | 13 | A | A | C | 0.59 | 7.114635e-01 |  |
| 289 | 13 | A | A | G | 0.78 | 5.539638e-01 |  |
| 289 | 13 | A | A | T | 1.56 | 4.867451e-01 |  |
| 290 | 14 | C | C | A | 1.18 | 5.239300e-01 |  |
| 290 | 14 | C | C | C | 92.92 | 6.112627e-04 | \* |
| 290 | 14 | C | C | G | 3.54 | 3.400319e-01 |  |
| 290 | 14 | C | C | T | 2.36 | 4.085092e-01 |  |
| 291 | 15 | C | C | A | 4.07 | 2.695048e-01 |  |
| 291 | 15 | C | C | C | 92.31 | 6.371282e-04 | \* |
| 291 | 15 | C | C | G | 3.62 | 3.359943e-01 |  |
| 291 | 15 | C | C | T | 0.00 | 7.446809e-01 |  |
| 292 | 16 | G | G | A | 1.79 | 4.509797e-01 |  |
| 292 | 16 | G | G | C | 0.90 | 6.702893e-01 |  |
| 292 | 16 | G | G | G | 95.82 | 2.486616e-05 | \* |
| 292 | 16 | G | G | T | 1.49 | 4.945337e-01 |  |
| 293 | 17 | T | T | A | 1.04 | 5.432901e-01 |  |
| 293 | 17 | T | T | C | 0.69 | 6.963230e-01 |  |
| 293 | 17 | T | T | G | 2.42 | 4.051181e-01 |  |
| 293 | 17 | T | T | T | 95.85 | 2.325999e-08 | \* |
| 294 | 18 | G | G | A | 0.54 | 6.214773e-01 |  |
| 294 | 18 | G | G | C | 0.00 | 8.780488e-01 |  |
| 294 | 18 | G | G | G | 98.74 | 1.879657e-05 | \* |
| 294 | 18 | G | G | T | 0.72 | 5.943685e-01 |  |
| 295 | 19 | T | T | A | 1.44 | 4.908976e-01 |  |
| 295 | 19 | T | T | C | 0.00 | 8.780488e-01 |  |
| 295 | 19 | T | T | G | 1.73 | 4.567426e-01 |  |
| 295 | 19 | T | T | T | 96.83 | 1.960482e-08 | \* |
| 296 | 20 | T | T | A | 2.39 | 3.922104e-01 |  |
| 296 | 20 | T | T | C | 0.00 | 8.780488e-01 |  |
| 296 | 20 | T | T | G | 2.92 | 3.740317e-01 |  |
| 296 | 20 | T | T | T | 94.69 | 2.842898e-08 | \* |
| 297 | 21 | T | T | A | 0.55 | 6.203308e-01 |  |
| 297 | 21 | T | T | C | 0.82 | 6.793161e-01 |  |
| 297 | 21 | T | T | G | 1.37 | 4.887540e-01 |  |
| 297 | 21 | T | T | T | 97.26 | 1.819026e-08 | \* |
| 298 | 22 | G | G | A | 0.43 | 6.422925e-01 |  |
| 298 | 22 | G | G | C | 1.07 | 6.500071e-01 |  |
| 298 | 22 | G | G | G | 97.64 | 2.088270e-05 | \* |
| 298 | 22 | G | G | T | 0.86 | 5.744180e-01 |  |
| 299 | 23 | T | T | A | 2.16 | 4.133571e-01 |  |
| 299 | 23 | T | T | C | 0.31 | 7.592301e-01 |  |
| 299 | 23 | T | T | G | 1.54 | 4.727516e-01 |  |
| 299 | 23 | T | T | T | 95.99 | 2.270040e-08 | \* |
| 300 | 24 | G | G | A | 1.36 | 5.009732e-01 |  |
| 300 | 24 | G | G | C | 0.82 | 6.801582e-01 |  |
| 300 | 24 | G | G | G | 97.01 | 2.218139e-05 | \* |
| 300 | 24 | G | G | T | 0.82 | 5.806000e-01 |  |
| 301 | 25 | C | C | A | 0.62 | 6.069209e-01 |  |
| 301 | 25 | C | C | C | 93.75 | 5.779166e-04 | \* |
| 301 | 25 | C | C | G | 0.94 | 5.345768e-01 |  |
| 301 | 25 | C | C | T | 4.69 | 2.531840e-01 |  |
| 302 | 26 | A | A | A | 94.50 | 8.257694e-09 | \* |
| 302 | 26 | A | A | C | 1.00 | 6.581157e-01 |  |
| 302 | 26 | A | A | G | 1.00 | 5.273137e-01 |  |
| 302 | 26 | A | A | T | 3.50 | 3.217830e-01 |  |
| 303 | 27 | C | G | A | 0.57 | 6.161802e-01 |  |
| 303 | 27 | C | G | C | 1.71 | 5.888472e-01 |  |
| 303 | 27 | C | G | G | 96.57 | 2.313715e-05 | \* |
| 303 | 27 | C | G | T | 1.14 | 5.362865e-01 |  |
| 304 | 28 | T | T | A | 1.91 | 4.385697e-01 |  |
| 304 | 28 | T | T | C | 0.38 | 7.450989e-01 |  |
| 304 | 28 | T | T | G | 0.38 | 6.155649e-01 |  |
| 304 | 28 | T | T | T | 97.33 | 1.797635e-08 | \* |
| 305 | 29 | A | A | A | 98.71 | 3.752926e-09 | \* |
| 305 | 29 | A | A | C | 1.29 | 6.277350e-01 |  |
| 305 | 29 | A | A | G | 0.00 | 7.500000e-01 |  |
| 305 | 29 | A | A | T | 0.00 | 7.446809e-01 |  |
| 306 | 30 | C | C | A | 1.65 | 4.669539e-01 |  |
| 306 | 30 | C | C | C | 90.95 | 6.986203e-04 | \* |
| 306 | 30 | C | C | G | 4.94 | 2.782553e-01 |  |
| 306 | 30 | C | C | T | 2.47 | 3.990749e-01 |  |
| 307 | 31 | A | A | A | 90.00 | 1.918340e-08 | \* |
| 307 | 31 | A | A | C | 1.90 | 5.732729e-01 |  |
| 307 | 31 | A | A | G | 5.24 | 2.670388e-01 |  |
| 307 | 31 | A | A | T | 2.86 | 3.676443e-01 |  |
| 308 | 32 | C | C | A | 0.38 | 6.524623e-01 |  |
| 308 | 32 | C | C | C | 96.23 | 4.888971e-04 | \* |
| 308 | 32 | C | C | G | 1.13 | 5.127455e-01 |  |
| 308 | 32 | C | C | T | 2.26 | 4.170040e-01 |  |
| 309 | 33 | G | G | A | 2.33 | 3.978278e-01 |  |
| 309 | 33 | G | G | C | 0.93 | 6.661663e-01 |  |
| 309 | 33 | G | G | G | 96.74 | 2.275657e-05 | \* |
| 309 | 33 | G | G | T | 0.00 | 7.446809e-01 |  |
| 310 | 34 | G | G | A | 0.29 | 6.697245e-01 |  |
| 310 | 34 | G | G | C | 0.29 | 7.622271e-01 |  |
| 310 | 34 | G | G | G | 99.12 | 1.812177e-05 | \* |
| 310 | 34 | G | G | T | 0.29 | 6.675502e-01 |  |
| 311 | 35 | G | G | A | 0.00 | 7.545455e-01 |  |
| 311 | 35 | G | G | C | 0.73 | 6.908490e-01 |  |
| 311 | 35 | G | G | G | 99.27 | 1.786337e-05 | \* |
| 311 | 35 | G | G | T | 0.00 | 7.446809e-01 |  |
| 312 | 36 | C | C | A | 0.40 | 6.470165e-01 |  |
| 312 | 36 | C | C | C | 95.95 | 4.980575e-04 | \* |
| 312 | 36 | C | C | G | 0.81 | 5.502873e-01 |  |
| 312 | 36 | C | C | T | 2.83 | 3.694329e-01 |  |
| 313 | 37 | A | A | A | 53.07 | 1.987845e-05 | \* |
| 313 | 37 | A | A | C | 1.32 | 6.249588e-01 |  |
| 313 | 37 | A | A | G | 45.61 | 3.343909e-03 | \* |
| 313 | 37 | A | A | T | 0.00 | 7.446809e-01 |  |
| 314 | 38 | C | C | A | 0.56 | 6.178753e-01 |  |
| 314 | 38 | C | C | C | 55.06 | 8.241031e-03 | \* |
| 314 | 38 | C | C | G | 43.54 | 4.117278e-03 | \* |
| 314 | 38 | C | C | T | 0.84 | 5.766498e-01 |  |
| 315 | 39 | C | C | A | 0.34 | 6.595467e-01 |  |
| 315 | 39 | C | C | C | 61.64 | 5.202234e-03 | \* |
| 315 | 39 | C | C | G | 37.67 | 7.447767e-03 | \* |
| 315 | 39 | C | C | T | 0.34 | 6.581647e-01 |  |
| 316 | 40 | C | C | A | 0.36 | 6.554909e-01 |  |
| 316 | 40 | C | C | C | 61.96 | 5.090381e-03 | \* |
| 316 | 40 | C | C | G | 0.72 | 5.614909e-01 |  |
| 316 | 40 | C | C | T | 36.96 | 7.025368e-04 | \* |
| 317 | 41 | T | T | A | 34.68 | 6.515531e-04 | \* |
| 317 | 41 | T | T | C | 0.40 | 7.411875e-01 |  |
| 317 | 41 | T | T | G | 0.81 | 5.507055e-01 |  |
| 317 | 41 | T | T | T | 64.11 | 5.911137e-06 | \* |
| 318 | 42 | G | G | A | 0.52 | 6.251967e-01 |  |
| 318 | 42 | G | G | C | 34.90 | 3.486615e-02 |  |
| 318 | 42 | G | G | G | 64.32 | 5.256028e-04 | \* |
| 318 | 42 | G | G | T | 0.26 | 6.743428e-01 |  |
| 319 | 43 | A | A | A | 99.22 | 3.409766e-09 | \* |
| 319 | 43 | A | A | C | 0.39 | 7.440142e-01 |  |
| 319 | 43 | A | A | G | 0.39 | 6.144599e-01 |  |
| 319 | 43 | A | A | T | 0.00 | 7.446809e-01 |  |
| 320 | 44 | C | C | A | 0.37 | 6.544137e-01 |  |
| 320 | 44 | C | C | C | 63.60 | 4.540535e-03 | \* |
| 320 | 44 | C | C | G | 33.82 | 1.103021e-02 |  |
| 320 | 44 | C | C | T | 2.21 | 4.222828e-01 |  |
| 321 | 45 | C | C | A | 0.40 | 6.470165e-01 |  |
| 321 | 45 | C | C | C | 93.52 | 5.868828e-04 | \* |
| 321 | 45 | C | C | G | 4.45 | 2.977646e-01 |  |
| 321 | 45 | C | C | T | 1.62 | 4.805322e-01 |  |
| 322 | 46 | G | G | A | 1.72 | 4.582613e-01 |  |
| 322 | 46 | G | G | C | 2.59 | 5.240900e-01 |  |
| 322 | 46 | G | G | G | 95.69 | 2.518156e-05 | \* |
| 322 | 46 | G | G | T | 0.00 | 7.446809e-01 |  |
| 323 | 47 | A | A | A | 99.03 | 3.534882e-09 | \* |
| 323 | 47 | A | A | C | 0.65 | 7.028569e-01 |  |
| 323 | 47 | A | A | G | 0.32 | 6.270973e-01 |  |
| 323 | 47 | A | A | T | 0.00 | 7.446809e-01 |  |
| 324 | 48 | C | C | A | 0.63 | 6.062525e-01 |  |
| 324 | 48 | C | C | C | 96.54 | 4.786300e-04 | \* |
| 324 | 48 | C | C | G | 0.31 | 6.287587e-01 |  |
| 324 | 48 | C | C | T | 2.52 | 3.951335e-01 |  |
| 325 | 49 | G | G | A | 7.78 | 1.237748e-01 |  |
| 325 | 49 | G | G | C | 0.56 | 7.160481e-01 |  |
| 325 | 49 | G | G | G | 53.89 | 1.467681e-03 | \* |
| 325 | 49 | G | G | T | 37.78 | 6.074374e-04 | \* |
| 326 | 50 | G | G | A | 29.47 | 1.763595e-03 | \* |
| 326 | 50 | G | G | C | 0.33 | 7.547220e-01 |  |
| 326 | 50 | G | G | G | 70.20 | 2.960733e-04 | \* |
| 326 | 50 | G | G | T | 0.00 | 7.446809e-01 |  |
| 327 | 51 | C | C | A | 34.93 | 6.211117e-04 | \* |
| 327 | 51 | C | C | C | 62.68 | 4.841064e-03 | \* |
| 327 | 51 | C | C | G | 0.48 | 5.984336e-01 |  |
| 327 | 51 | C | C | T | 1.91 | 4.500559e-01 |  |
| 328 | 52 | A | A | A | 50.43 | 3.271241e-05 | \* |
| 328 | 52 | A | A | C | 0.00 | 8.780488e-01 |  |
| 328 | 52 | A | A | G | 0.00 | 7.500000e-01 |  |
| 328 | 52 | A | A | T | 49.57 | 7.590127e-05 | \* |
| 329 | 53 | A | A | A | 97.36 | 4.836915e-09 | \* |
| 329 | 53 | A | A | C | 1.32 | 6.243929e-01 |  |
| 329 | 53 | A | A | G | 0.00 | 7.500000e-01 |  |
| 329 | 53 | A | A | T | 1.32 | 5.143248e-01 |  |
| 330 | 54 | G | G | A | 0.96 | 5.548676e-01 |  |
| 330 | 54 | G | G | C | 46.89 | 1.467266e-02 |  |
| 330 | 54 | G | G | G | 50.72 | 2.010055e-03 | \* |
| 330 | 54 | G | G | T | 1.44 | 5.010244e-01 |  |
| 331 | 55 | A | A | A | 100.00 | 2.949309e-09 | \* |
| 331 | 55 | A | A | C | 0.00 | 8.780488e-01 |  |
| 331 | 55 | A | A | G | 0.00 | 7.500000e-01 |  |
| 331 | 55 | A | A | T | 0.00 | 7.446809e-01 |  |
| 332 | 56 | A | A | A | 59.28 | 6.162525e-06 | \* |
| 332 | 56 | A | A | C | 0.45 | 7.326120e-01 |  |
| 332 | 56 | A | A | G | 39.37 | 6.271218e-03 | \* |
| 332 | 56 | A | A | T | 0.90 | 5.678683e-01 |  |
| 333 | 57 | G | G | A | 0.00 | 7.545455e-01 |  |
| 333 | 57 | G | G | C | 46.11 | 1.551321e-02 |  |
| 333 | 57 | G | G | G | 52.69 | 1.651973e-03 | \* |
| 333 | 57 | G | G | T | 1.20 | 5.294111e-01 |  |
| 334 | 58 | T | T | A | 0.00 | 7.545455e-01 |  |
| 334 | 58 | T | T | C | 32.54 | 4.146331e-02 |  |
| 334 | 58 | T | T | G | 0.00 | 7.500000e-01 |  |
| 334 | 58 | T | T | T | 67.46 | 3.292107e-06 | \* |
| 335 | 59 | T | T | A | 23.85 | 5.202330e-03 | \* |
| 335 | 59 | T | T | C | 1.15 | 6.413533e-01 |  |
| 335 | 59 | T | T | G | 1.54 | 4.731753e-01 |  |
| 335 | 59 | T | T | T | 73.46 | 1.152610e-06 | \* |
| 336 | 60 | C | C | A | 7.07 | 1.431613e-01 |  |
| 336 | 60 | C | C | C | 91.58 | 6.691769e-04 | \* |
| 336 | 60 | C | C | G | 1.35 | 4.909706e-01 |  |
| 336 | 60 | C | C | T | 0.00 | 7.446809e-01 |  |
| 337 | 61 | G | G | A | 0.00 | 7.545455e-01 |  |
| 337 | 61 | G | G | C | 40.39 | 2.337115e-02 |  |
| 337 | 61 | G | G | G | 56.16 | 1.172851e-03 | \* |
| 337 | 61 | G | G | T | 3.45 | 3.252170e-01 |  |
| 338 | 62 | A | A | A | 98.82 | 3.678894e-09 | \* |
| 338 | 62 | A | A | C | 1.18 | 6.385108e-01 |  |
| 338 | 62 | A | A | G | 0.00 | 7.500000e-01 |  |
| 338 | 62 | A | A | T | 0.00 | 7.446809e-01 |  |
| 339 | 63 | C | C | A | 0.74 | 5.886860e-01 |  |
| 339 | 63 | C | C | C | 93.38 | 5.924609e-04 | \* |
| 339 | 63 | C | C | G | 2.21 | 4.200611e-01 |  |
| 339 | 63 | C | C | T | 3.68 | 3.103757e-01 |  |
| 340 | 64 | A | A | A | 50.27 | 3.374615e-05 | \* |
| 340 | 64 | A | A | C | 45.95 | 1.569322e-02 |  |
| 340 | 64 | A | A | G | 0.54 | 5.883480e-01 |  |
| 340 | 64 | A | A | T | 3.24 | 3.392513e-01 |  |
| 341 | 65 | G | G | A | 28.40 | 2.165456e-03 | \* |
| 341 | 65 | G | G | C | 0.80 | 6.820841e-01 |  |
| 341 | 65 | G | G | G | 70.80 | 2.792256e-04 | \* |
| 341 | 65 | G | G | T | 0.00 | 7.446809e-01 |  |
| 342 | 66 | C | C | A | 0.00 | 7.545455e-01 |  |
| 342 | 66 | C | C | C | 96.53 | 4.791402e-04 | \* |
| 342 | 66 | C | C | G | 0.39 | 6.147386e-01 |  |
| 342 | 66 | C | C | T | 3.09 | 3.502881e-01 |  |
| 343 | 67 | T | T | A | 1.11 | 5.331876e-01 |  |
| 343 | 67 | T | T | C | 29.26 | 5.293404e-02 |  |
| 343 | 67 | T | T | G | 0.00 | 7.500000e-01 |  |
| 343 | 67 | T | T | T | 69.63 | 2.251718e-06 | \* |
| 344 | 68 | C | C | A | 2.12 | 4.174131e-01 |  |
| 344 | 68 | C | C | C | 62.71 | 4.830172e-03 | \* |
| 344 | 68 | C | C | G | 35.17 | 9.610370e-03 | \* |
| 344 | 68 | C | C | T | 0.00 | 7.446809e-01 |  |
| 345 | 69 | C | C | A | 0.70 | 5.949211e-01 |  |
| 345 | 69 | C | C | C | 94.77 | 5.392950e-04 | \* |
| 345 | 69 | C | C | G | 0.35 | 6.219358e-01 |  |
| 345 | 69 | C | C | T | 4.18 | 2.801997e-01 |  |
| 346 | 70 | C | C | A | 1.02 | 5.465095e-01 |  |
| 346 | 70 | C | C | C | 94.42 | 5.524714e-04 | \* |
| 346 | 70 | C | C | G | 0.51 | 5.936210e-01 |  |
| 346 | 70 | C | C | T | 4.06 | 2.870789e-01 |  |
| 347 | 71 | G | G | A | 27.71 | 2.471807e-03 | \* |
| 347 | 71 | G | G | C | 1.81 | 5.811292e-01 |  |
| 347 | 71 | G | G | G | 70.48 | 2.880133e-04 | \* |
| 347 | 71 | G | G | T | 0.00 | 7.446809e-01 |  |
| 348 | 72 | C | C | A | 2.02 | 4.267432e-01 |  |
| 348 | 72 | C | C | C | 95.55 | 5.118597e-04 | \* |
| 348 | 72 | C | C | G | 0.40 | 6.112832e-01 |  |
| 348 | 72 | C | C | T | 2.02 | 4.392860e-01 |  |
| 349 | 73 | G | G | A | 3.38 | 3.135014e-01 |  |
| 349 | 73 | G | G | C | 38.16 | 2.746692e-02 |  |
| 349 | 73 | G | G | G | 57.97 | 9.807968e-04 | \* |
| 349 | 73 | G | G | T | 0.48 | 6.327798e-01 |  |
| 350 | 74 | A | A | A | 97.01 | 5.156614e-09 | \* |
| 350 | 74 | A | A | C | 0.50 | 7.251682e-01 |  |
| 350 | 74 | A | A | G | 2.49 | 4.007759e-01 |  |
| 350 | 74 | A | A | T | 0.00 | 7.446809e-01 |  |
| 351 | 75 | C | C | A | 0.38 | 6.510139e-01 |  |
| 351 | 75 | C | C | C | 93.85 | 5.741725e-04 | \* |
| 351 | 75 | C | C | G | 1.92 | 4.411287e-01 |  |
| 351 | 75 | C | C | T | 3.85 | 2.998395e-01 |  |

## For use in R

If you want to work with the results in R, here is output that you can copy and paste in your terminal to get:

The base information:

```
structure(list(focal.base = c("A", "C", "G", "T"), avg.percsignal = c(85.2101664864019, 
88.8707385792467, 83.1807133778665, 86.05984296743), avg.areasignal = c(278.794117647059, 
285.985294117647, 283.357142857143, 325.488372093023), crit.perc.area = c(20.4710157680194, 
52.3047829514447, 34.780822267808, 22.073057932485), mu = c(4.16255000189655, 
8.4819151286945, 5.93576278430408, 4.52740531669706), fillibens = c(0.949285411945213, 
0.942804181845222, 0.955633534211706, 0.945819201516209)), .Names = c("focal.base", 
"avg.percsignal", "avg.areasignal", "crit.perc.area", "mu", "fillibens"
), row.names = c(NA, -4L), class = "data.frame")
```

the data.frame that contains information on the guide region:

```
structure(list(A.area = c(189, 248, 5, 490, 3, 12, 2, 9, 2, 2, 
201, 4, 497, 4, 9, 6, 3, 3, 5, 9, 2, 2, 7, 5, 2, 189, 1, 5, 230, 
4, 189, 1, 5, 1, 0, 1, 121, 2, 1, 1, 86, 2, 256, 1, 1, 4, 307, 
2, 14, 89, 73, 58, 221, 2, 206, 131, 0, 0, 62, 21, 0, 251, 2, 
93, 71, 0, 3, 5, 2, 2, 46, 5, 7, 195, 1), C.area = c(8, 6, 1, 
3, 289, 11, 17, 3, 228, 325, 6, 9, 3, 315, 204, 3, 2, 0, 0, 0, 
3, 5, 1, 3, 300, 2, 3, 1, 3, 221, 4, 255, 2, 1, 2, 237, 3, 196, 
180, 171, 1, 134, 1, 173, 231, 6, 2, 307, 1, 1, 131, 0, 3, 98, 
0, 1, 77, 96, 3, 272, 82, 3, 254, 85, 2, 250, 79, 148, 272, 186, 
3, 236, 79, 1, 244), G.area = c(3, 2, 240, 7, 6, 8, 637, 392, 
8, 2, 3, 250, 4, 12, 8, 321, 7, 547, 6, 11, 5, 455, 5, 357, 3, 
2, 169, 1, 0, 12, 11, 3, 208, 337, 271, 2, 104, 155, 110, 2, 
2, 247, 1, 92, 11, 222, 1, 1, 97, 212, 1, 0, 0, 106, 0, 87, 88, 
0, 4, 4, 114, 0, 6, 1, 177, 1, 0, 83, 1, 1, 117, 1, 120, 5, 5
), T.area = c(0, 7, 8, 9, 3, 303, 3, 3, 4, 7, 0, 1, 8, 8, 0, 
5, 277, 4, 336, 357, 355, 4, 311, 3, 15, 7, 2, 255, 0, 6, 6, 
6, 0, 1, 0, 7, 0, 3, 1, 102, 159, 1, 0, 6, 4, 0, 0, 8, 68, 0, 
4, 57, 3, 3, 0, 2, 2, 199, 191, 0, 7, 0, 10, 6, 0, 8, 188, 0, 
12, 8, 0, 5, 1, 0, 10), Tot.area = c(200, 263, 254, 509, 301, 
334, 659, 407, 242, 336, 210, 264, 512, 339, 221, 335, 289, 554, 
347, 377, 365, 466, 324, 368, 320, 200, 175, 262, 233, 243, 210, 
265, 215, 340, 273, 247, 228, 356, 292, 276, 248, 384, 258, 272, 
247, 232, 310, 318, 180, 302, 209, 115, 227, 209, 206, 221, 167, 
295, 260, 297, 203, 254, 272, 185, 250, 259, 270, 236, 287, 197, 
166, 247, 207, 201, 260), A.perc = c(94.5, 94.2965779467681, 
1.96850393700787, 96.2671905697446, 0.996677740863787, 3.59281437125748, 
0.303490136570561, 2.21130221130221, 0.826446280991736, 0.595238095238095, 
95.7142857142857, 1.51515151515152, 97.0703125, 1.17994100294985, 
4.07239819004525, 1.7910447761194, 1.03806228373702, 0.541516245487365, 
1.44092219020173, 2.38726790450928, 0.547945205479452, 0.429184549356223, 
2.16049382716049, 1.35869565217391, 0.625, 94.5, 0.571428571428571, 
1.90839694656489, 98.7124463519313, 1.64609053497942, 90, 0.377358490566038, 
2.32558139534884, 0.294117647058824, 0, 0.404858299595142, 53.0701754385965, 
0.561797752808989, 0.342465753424658, 0.36231884057971, 34.6774193548387, 
0.520833333333333, 99.2248062015504, 0.367647058823529, 0.404858299595142, 
1.72413793103448, 99.0322580645161, 0.628930817610063, 7.77777777777778, 
29.4701986754967, 34.9282296650718, 50.4347826086956, 97.3568281938326, 
0.956937799043062, 100, 59.2760180995475, 0, 0, 23.8461538461538, 
7.07070707070707, 0, 98.8188976377953, 0.735294117647059, 50.2702702702703, 
28.4, 0, 1.11111111111111, 2.11864406779661, 0.696864111498258, 
1.01522842639594, 27.710843373494, 2.02429149797571, 3.38164251207729, 
97.0149253731343, 0.384615384615385), C.perc = c(4, 2.28136882129278, 
0.393700787401575, 0.589390962671906, 96.0132890365448, 3.29341317365269, 
2.57966616084977, 0.737100737100737, 94.2148760330578, 96.7261904761905, 
2.85714285714286, 3.40909090909091, 0.5859375, 92.9203539823009, 
92.3076923076923, 0.895522388059702, 0.692041522491349, 0, 0, 
0, 0.821917808219178, 1.07296137339056, 0.308641975308642, 0.815217391304348, 
93.75, 1, 1.71428571428571, 0.381679389312977, 1.28755364806867, 
90.9465020576132, 1.9047619047619, 96.2264150943396, 0.930232558139535, 
0.294117647058824, 0.732600732600733, 95.9514170040486, 1.31578947368421, 
55.0561797752809, 61.6438356164384, 61.9565217391304, 0.403225806451613, 
34.8958333333333, 0.387596899224806, 63.6029411764706, 93.5222672064777, 
2.58620689655172, 0.645161290322581, 96.5408805031446, 0.555555555555556, 
0.33112582781457, 62.6794258373206, 0, 1.3215859030837, 46.88995215311, 
0, 0.452488687782805, 46.1077844311377, 32.5423728813559, 1.15384615384615, 
91.5824915824916, 40.3940886699507, 1.18110236220472, 93.3823529411765, 
45.9459459459459, 0.8, 96.5250965250965, 29.2592592592593, 62.7118644067797, 
94.7735191637631, 94.4162436548223, 1.80722891566265, 95.5465587044534, 
38.1642512077295, 0.497512437810945, 93.8461538461538), G.perc = c(1.5, 
0.760456273764259, 94.488188976378, 1.37524557956778, 1.99335548172757, 
2.39520958083832, 96.6616084977238, 96.3144963144963, 3.30578512396694, 
0.595238095238095, 1.42857142857143, 94.6969696969697, 0.78125, 
3.53982300884956, 3.61990950226244, 95.8208955223881, 2.42214532871972, 
98.7364620938628, 1.72910662824207, 2.91777188328912, 1.36986301369863, 
97.6394849785408, 1.54320987654321, 97.0108695652174, 0.9375, 
1, 96.5714285714286, 0.381679389312977, 0, 4.93827160493827, 
5.23809523809524, 1.13207547169811, 96.7441860465116, 99.1176470588235, 
99.2673992673993, 0.809716599190283, 45.6140350877193, 43.5393258426966, 
37.6712328767123, 0.72463768115942, 0.806451612903226, 64.3229166666667, 
0.387596899224806, 33.8235294117647, 4.45344129554656, 95.6896551724138, 
0.32258064516129, 0.314465408805031, 53.8888888888889, 70.1986754966887, 
0.478468899521531, 0, 0, 50.7177033492823, 0, 39.3665158371041, 
52.6946107784431, 0, 1.53846153846154, 1.34680134680135, 56.1576354679803, 
0, 2.20588235294118, 0.540540540540541, 70.8, 0.386100386100386, 
0, 35.1694915254237, 0.348432055749129, 0.50761421319797, 70.4819277108434, 
0.404858299595142, 57.9710144927536, 2.48756218905473, 1.92307692307692
), T.perc = c(0, 2.66159695817491, 3.1496062992126, 1.76817288801572, 
0.996677740863787, 90.7185628742515, 0.455235204855842, 0.737100737100737, 
1.65289256198347, 2.08333333333333, 0, 0.378787878787879, 1.5625, 
2.3598820058997, 0, 1.49253731343284, 95.8477508650519, 0.72202166064982, 
96.8299711815562, 94.6949602122016, 97.2602739726027, 0.858369098712446, 
95.9876543209877, 0.815217391304348, 4.6875, 3.5, 1.14285714285714, 
97.3282442748092, 0, 2.46913580246914, 2.85714285714286, 2.26415094339623, 
0, 0.294117647058824, 0, 2.83400809716599, 0, 0.842696629213483, 
0.342465753424658, 36.9565217391304, 64.1129032258064, 0.260416666666667, 
0, 2.20588235294118, 1.61943319838057, 0, 0, 2.51572327044025, 
37.7777777777778, 0, 1.91387559808612, 49.5652173913044, 1.3215859030837, 
1.43540669856459, 0, 0.904977375565611, 1.19760479041916, 67.4576271186441, 
73.4615384615385, 0, 3.44827586206897, 0, 3.67647058823529, 3.24324324324324, 
0, 3.08880308880309, 69.6296296296296, 0, 4.18118466898955, 4.06091370558376, 
0, 2.02429149797571, 0.483091787439614, 0, 3.84615384615385), 
    base.call = c("A", "A", "G", "A", "C", "T", "G", "G", "C", 
    "C", "A", "G", "A", "C", "C", "G", "T", "G", "T", "T", "T", 
    "G", "T", "G", "C", "A", "G", "T", "A", "C", "A", "C", "G", 
    "G", "G", "C", "A", "C", "C", "C", "T", "G", "A", "C", "C", 
    "G", "A", "C", "G", "G", "C", "A", "A", "G", "A", "A", "G", 
    "T", "T", "C", "G", "A", "C", "A", "G", "C", "T", "C", "C", 
    "C", "G", "C", "G", "A", "C"), index = 277:351, guide.seq = c("A", 
    "A", "G", "A", "C", "T", "G", "G", "C", "C", "A", "G", "A", 
    "C", "C", "G", "T", "G", "T", "T", "T", "G", "T", "G", "C", 
    "A", "C", "T", "A", "C", "A", "C", "G", "G", "G", "C", "A", 
    "C", "C", "C", "T", "G", "A", "C", "C", "G", "A", "C", "G", 
    "G", "C", "A", "A", "G", "A", "A", "G", "T", "T", "C", "G", 
    "A", "C", "A", "G", "C", "T", "C", "C", "C", "G", "C", "G", 
    "A", "C"), T.pval = c(0.744680851063792, 0.38309540017966, 
    0.345893610392493, 0.464803165379049, 0.555344657671612, 
    5.68172225934305e-08, 0.637613285036625, 0.59210010599977, 
    0.476931646283277, 0.433663407578647, 0.744680851063792, 
    0.651357130285786, 0.48674507539046, 0.408509238553294, 0.744680851063792, 
    0.494533665573813, 2.32599891569762e-08, 0.594368476406891, 
    1.96048191014597e-08, 2.84289816132599e-08, 1.81902551910085e-08, 
    0.574417960379502, 2.27004028907629e-08, 0.580600039476343, 
    0.253184034878164, 0.32178302315892, 0.536286508630059, 1.79763482988093e-08, 
    0.744680851063792, 0.399074885820735, 0.367644278361197, 
    0.41700404945059, 0.744680851063792, 0.667550229378778, 0.744680851063792, 
    0.369432882363078, 0.744680851063792, 0.576649806982289, 
    0.658164676097041, 0.000702536769286555, 5.91113700354473e-06, 
    0.674342801844377, 0.744680851063792, 0.422282845924879, 
    0.480532176931932, 0.744680851063792, 0.744680851063792, 
    0.395133500389032, 0.000607437391547783, 0.744680851063792, 
    0.450055852233606, 7.59012730354724e-05, 0.514324780712354, 
    0.501024397487124, 0.744680851063792, 0.567868327180555, 
    0.529411112566145, 3.29210708127636e-06, 1.15260953570395e-06, 
    0.744680851063792, 0.325216997025508, 0.744680851063792, 
    0.310375724954261, 0.339251333310524, 0.744680851063792, 
    0.350288108878284, 2.25171751722097e-06, 0.744680851063792, 
    0.280199711510708, 0.287078891200959, 0.744680851063792, 
    0.439285961183888, 0.632779810202487, 0.744680851063792, 
    0.299839454254593), C.pval = c(0.443805783496578, 0.544979072407748, 
    0.742903718380382, 0.710949932963357, 0.000495981517163235, 
    0.481020534782364, 0.5245216942, 0.690251272995231, 0.000560040503456594, 
    0.000472681924368334, 0.506782947062558, 0.474574446740466, 
    0.711463462300683, 0.000611262705578763, 0.000637128241473439, 
    0.670289275389438, 0.696322973722821, 0.878048780487769, 
    0.878048780487769, 0.878048780487769, 0.679316115823771, 
    0.650007086727992, 0.759230092250514, 0.680158240700442, 
    0.000577916637249154, 0.658115700645752, 0.588847199730462, 
    0.745098941102992, 0.627735019628173, 0.000698620300012887, 
    0.573272909474733, 0.00048889709129929, 0.666166270785763, 
    0.762227102100033, 0.690848995007671, 0.000498057505998961, 
    0.624958756045526, 0.00824103117404607, 0.00520223369627804, 
    0.00509038090013814, 0.741187499695139, 0.0348661546856199, 
    0.744014195907993, 0.00454053502959251, 0.000586882789519572, 
    0.524090007334276, 0.702856883454308, 0.000478630037429872, 
    0.716048104224706, 0.754721965657107, 0.00484106380752858, 
    0.878048780487769, 0.624392864380232, 0.0146726579591114, 
    0.878048780487769, 0.732612038999238, 0.015513207998371, 
    0.0414633130779574, 0.641353331972008, 0.000669176945131467, 
    0.0233711483227492, 0.638510767234782, 0.000592460886215673, 
    0.0156932229286466, 0.68208413389696, 0.000479140150162483, 
    0.0529340403660953, 0.00483017220425086, 0.000539294954433056, 
    0.000552471370540042, 0.581129230805151, 0.00051185973278145, 
    0.027466924178741, 0.725168237859858, 0.000574172547810337
    ), G.pval = c(0.476636830246852, 0.556694167951315, 2.82624963690026e-05, 
    0.488239782025261, 0.435716656429053, 0.406929225363295, 
    2.29376955924732e-05, 2.37150098869598e-05, 0.352251695519386, 
    0.579935751978253, 0.483207533077654, 2.77011521601089e-05, 
    0.553963791651147, 0.340031894104118, 0.335994278132987, 
    2.48661568926778e-05, 0.405118132823471, 1.87965744180563e-05, 
    0.456742592976449, 0.374031659641418, 0.488754002415928, 
    2.08827039647197e-05, 0.472751576705573, 2.21813927672176e-05, 
    0.534576821842371, 0.527313740373663, 2.31371471406394e-05, 
    0.615564915587008, 0.749999999999921, 0.278255281130777, 
    0.267038783274757, 0.512745468013344, 2.27565733055268e-05, 
    1.81217693038249e-05, 1.78633702849762e-05, 0.550287339923442, 
    0.00334390851394462, 0.0041172779515819, 0.00744776735509456, 
    0.561490947120579, 0.550705503518783, 0.000525602756307642, 
    0.614459920179359, 0.0110302103260695, 0.297764610251892, 
    2.51815648717457e-05, 0.627097270633051, 0.628758693324169, 
    0.00146768147611187, 0.000296073267509023, 0.598433641948145, 
    0.749999999999921, 0.749999999999921, 0.00201005470003435, 
    0.749999999999921, 0.00627121827688515, 0.00165197303319342, 
    0.749999999999921, 0.473175332127882, 0.490970575020681, 
    0.00117285115476751, 0.749999999999921, 0.420061144153431, 
    0.588348045008994, 0.000279225612547007, 0.614738581892552, 
    0.749999999999921, 0.009610369536597, 0.621935784912873, 
    0.593620971165779, 0.000288013252885744, 0.611283169785483, 
    0.000980796810663653, 0.400775886549636, 0.441128703617497
    ), A.pval = c(8.2576941951018e-09, 8.5783200542977e-09, 0.432384439475287, 
    5.93141213922621e-09, 0.549147397046149, 0.299244165987067, 
    0.667715983297435, 0.40849879474922, 0.574359389188505, 0.612029983538677, 
    6.57831600303638e-09, 0.482044195903267, 5.10342401671693e-09, 
    0.523930012837725, 0.269504834239575, 0.450979650089522, 
    0.543290114358257, 0.621477296806245, 0.49089759277971, 0.392210386317472, 
    0.620330754062477, 0.642292512737691, 0.413357096372009, 
    0.500973227677623, 0.606920920700106, 8.2576941951018e-09, 
    0.616180150548637, 0.438569749227002, 3.75292574794628e-09, 
    0.466953925158339, 1.9183402311107e-08, 0.65246225815789, 
    0.397827796548649, 0.669724545346375, 0.754545454545314, 
    0.647016456928537, 1.9878450050248e-05, 0.617875344217264, 
    0.659546720931005, 0.655490902279084, 0.000651553116930326, 
    0.625196735058081, 3.40976602508647e-09, 0.654413705044118, 
    0.647016456928537, 0.458261267807301, 3.53488194271279e-09, 
    0.606252464300456, 0.12377475606482, 0.00176359459317343, 
    0.000621111654071416, 3.27124140231394e-05, 4.83691542374487e-09, 
    0.554867578894367, 2.94930879718436e-09, 6.16252538754303e-06, 
    0.754545454545314, 0.754545454545314, 0.00520233046417296, 
    0.143161276171069, 0.754545454545314, 3.67889374519592e-09, 
    0.588686045640548, 3.37461474725353e-05, 0.00216545627873732, 
    0.754545454545314, 0.533187609878051, 0.417413140114551, 
    0.594921063318534, 0.546509527077082, 0.00247180714022699, 
    0.426743212787847, 0.313501363721894, 5.156613802626e-09, 
    0.651013853405375), guide.position = 1:75), .Names = c("A.area", 
"C.area", "G.area", "T.area", "Tot.area", "A.perc", "C.perc", 
"G.perc", "T.perc", "base.call", "index", "guide.seq", "T.pval", 
"C.pval", "G.pval", "A.pval", "guide.position"), row.names = 277:351, class = "data.frame")
```

*Report generated using EditR v1.0.8*
