# Supplementary material for: Mechanistic and genetic basis of single-strand templated repair at Cas12a-induced DNA breaks in Chlamydomonas reinhardtii
Source: Nat Commun. 2021 Nov 19;12:6751. doi: 10.1038/s41467-021-27004-1 (PMC8604939; doi:10.1038/s41467-021-27004-1)
Supplement: Supplementary file 22 — Source Data [file 41467_2021_27004_MOESM22_ESM.zip › Source Data/EditR analysis/EditR outputs/Controls for normalization/Positive control (32).html]

EditR v1.0.8 report


# EditR v1.0.8 report

- Data QA
  - Filtering data
  - Percent noise peak area
  - Base information
- Predicted editing
  - Editing bar plot
  - Editing table plot
  - Table of editing results
- For use in R

## Data QA

### Filtering data

What the data looked like prefiltering:

and the post filtering signal / noise plot:

### Percent noise peak area

### Base information

Here’s information about the signal of each base, the critical percent value where any higher value would be called as significant, and Filliben’s correlation for how well the noise was modelled by the zero adjusted gamma distribution.

| Base | Average percent signal | Average peak area | Critical percent value | model mu | Fillibens correlation |
| --- | --- | --- | --- | --- | --- |
| A | 84.32906 | 223.8583 | 42.54817 | 8.929077 | 0.9901823 |
| C | 84.70357 | 200.6519 | 51.05851 | 9.686665 | 0.9879602 |
| G | 86.06337 | 254.9140 | 58.62146 | 11.060628 | 0.9879351 |
| T | 83.77705 | 204.1765 | 40.74515 | 8.522688 | 0.9890411 |

## Predicted editing

### Editing bar plot

### Editing table plot

### Table of editing results


Here’s the entire guide region

| Sanger position | Guide position | Guide sequence | Sanger base call | Focal base | Focal base peak area | p value |  |
| --- | --- | --- | --- | --- | --- | --- | --- |
| 275 | 1 | A | A | A | 96.58 | 1.126764e-04 | \* |
| 275 | 1 | A | A | C | 0.00 | 5.780051e-01 |  |
| 275 | 1 | A | A | G | 3.42 | 3.539187e-01 |  |
| 275 | 1 | A | A | T | 0.00 | 5.862884e-01 |  |
| 276 | 2 | A | A | A | 95.93 | 1.188498e-04 | \* |
| 276 | 2 | A | A | C | 1.16 | 4.428896e-01 |  |
| 276 | 2 | A | A | G | 1.74 | 4.217869e-01 |  |
| 276 | 2 | A | A | T | 1.16 | 4.688526e-01 |  |
| 277 | 3 | G | G | A | 1.42 | 4.439104e-01 |  |
| 277 | 3 | G | G | C | 1.42 | 4.272525e-01 |  |
| 277 | 3 | G | G | G | 95.04 | 1.274149e-03 | \* |
| 277 | 3 | G | G | T | 2.13 | 4.114431e-01 |  |
| 278 | 4 | A | A | A | 96.55 | 1.129488e-04 | \* |
| 278 | 4 | A | A | C | 0.57 | 4.869879e-01 |  |
| 278 | 4 | A | A | G | 2.87 | 3.733983e-01 |  |
| 278 | 4 | A | A | T | 0.00 | 5.862884e-01 |  |
| 279 | 5 | C | C | A | 1.10 | 4.635261e-01 |  |
| 279 | 5 | C | C | C | 95.03 | 5.807081e-04 | \* |
| 279 | 5 | C | C | G | 0.00 | 5.807292e-01 |  |
| 279 | 5 | C | C | T | 3.87 | 3.335248e-01 |  |
| 280 | 6 | T | T | A | 3.31 | 3.522279e-01 |  |
| 280 | 6 | T | T | C | 0.55 | 4.889988e-01 |  |
| 280 | 6 | T | T | G | 2.21 | 4.002212e-01 |  |
| 280 | 6 | T | T | T | 93.92 | 9.534692e-05 | \* |
| 281 | 7 | G | G | A | 0.80 | 4.849332e-01 |  |
| 281 | 7 | G | G | C | 1.06 | 4.493943e-01 |  |
| 281 | 7 | G | G | G | 97.87 | 1.087976e-03 | \* |
| 281 | 7 | G | G | T | 0.27 | 5.439652e-01 |  |
| 282 | 8 | G | G | A | 1.75 | 4.247555e-01 |  |
| 282 | 8 | G | G | C | 1.32 | 4.333470e-01 |  |
| 282 | 8 | G | G | G | 96.93 | 1.146563e-03 | \* |
| 282 | 8 | G | G | T | 0.00 | 5.862884e-01 |  |
| 283 | 9 | C | C | A | 1.46 | 4.414544e-01 |  |
| 283 | 9 | C | C | C | 93.43 | 6.428226e-04 | \* |
| 283 | 9 | C | C | G | 0.00 | 5.807292e-01 |  |
| 283 | 9 | C | C | T | 5.11 | 2.900546e-01 |  |
| 284 | 10 | C | C | A | 0.53 | 5.064925e-01 |  |
| 284 | 10 | C | C | C | 93.68 | 6.325302e-04 | \* |
| 284 | 10 | C | C | G | 1.58 | 4.301508e-01 |  |
| 284 | 10 | C | C | T | 4.21 | 3.206998e-01 |  |
| 285 | 11 | A | A | A | 93.98 | 1.393968e-04 | \* |
| 285 | 11 | A | A | C | 0.75 | 4.720834e-01 |  |
| 285 | 11 | A | A | G | 5.26 | 2.996265e-01 |  |
| 285 | 11 | A | A | T | 0.00 | 5.862884e-01 |  |
| 286 | 12 | G | G | A | 2.04 | 4.096416e-01 |  |
| 286 | 12 | G | G | C | 1.36 | 4.306615e-01 |  |
| 286 | 12 | G | G | G | 95.92 | 1.212989e-03 | \* |
| 286 | 12 | G | G | T | 0.68 | 5.048089e-01 |  |
| 287 | 13 | A | A | A | 97.08 | 1.081999e-04 | \* |
| 287 | 13 | A | A | C | 0.58 | 4.860874e-01 |  |
| 287 | 13 | A | A | G | 2.34 | 3.946791e-01 |  |
| 287 | 13 | A | A | T | 0.00 | 5.862884e-01 |  |
| 288 | 14 | C | C | A | 1.01 | 4.698732e-01 |  |
| 288 | 14 | C | C | C | 90.91 | 7.548504e-04 | \* |
| 288 | 14 | C | C | G | 4.04 | 3.338803e-01 |  |
| 288 | 14 | C | C | T | 4.04 | 3.269734e-01 |  |
| 289 | 15 | C | C | A | 5.04 | 2.915823e-01 |  |
| 289 | 15 | C | C | C | 85.71 | 1.051827e-03 | \* |
| 289 | 15 | C | C | G | 2.52 | 3.871588e-01 |  |
| 289 | 15 | C | C | T | 6.72 | 2.437500e-01 |  |
| 290 | 16 | G | G | A | 2.50 | 3.873111e-01 |  |
| 290 | 16 | G | G | C | 0.00 | 5.780051e-01 |  |
| 290 | 16 | G | G | G | 96.50 | 1.174330e-03 | \* |
| 290 | 16 | G | G | T | 1.00 | 4.802333e-01 |  |
| 291 | 17 | T | T | A | 0.58 | 5.015772e-01 |  |
| 291 | 17 | T | T | C | 0.58 | 4.860874e-01 |  |
| 291 | 17 | T | T | G | 1.75 | 4.212843e-01 |  |
| 291 | 17 | T | T | T | 97.08 | 7.262367e-05 | \* |
| 292 | 18 | G | G | A | 0.31 | 5.263008e-01 |  |
| 292 | 18 | G | G | C | 0.00 | 5.780051e-01 |  |
| 292 | 18 | G | G | G | 99.06 | 1.018298e-03 | \* |
| 292 | 18 | G | G | T | 0.62 | 5.094381e-01 |  |
| 293 | 19 | T | T | A | 1.05 | 4.673709e-01 |  |
| 293 | 19 | T | T | C | 0.00 | 5.780051e-01 |  |
| 293 | 19 | T | T | G | 1.05 | 4.605867e-01 |  |
| 293 | 19 | T | T | T | 97.91 | 6.760634e-05 | \* |
| 294 | 20 | T | T | A | 0.91 | 4.768804e-01 |  |
| 294 | 20 | T | T | C | 0.00 | 5.780051e-01 |  |
| 294 | 20 | T | T | G | 3.18 | 3.621419e-01 |  |
| 294 | 20 | T | T | T | 95.91 | 8.031871e-05 | \* |
| 295 | 21 | T | T | A | 0.97 | 4.725623e-01 |  |
| 295 | 21 | T | T | C | 0.00 | 5.780051e-01 |  |
| 295 | 21 | T | T | G | 1.94 | 4.123080e-01 |  |
| 295 | 21 | T | T | T | 97.09 | 7.255255e-05 | \* |
| 296 | 22 | G | G | A | 0.00 | 5.717489e-01 |  |
| 296 | 22 | G | G | C | 0.74 | 4.731873e-01 |  |
| 296 | 22 | G | G | G | 98.52 | 1.049249e-03 | \* |
| 296 | 22 | G | G | T | 0.74 | 5.001097e-01 |  |
| 297 | 23 | T | T | A | 1.01 | 4.698732e-01 |  |
| 297 | 23 | T | T | C | 0.51 | 4.934173e-01 |  |
| 297 | 23 | T | T | G | 1.01 | 4.629615e-01 |  |
| 297 | 23 | T | T | T | 97.47 | 7.016763e-05 | \* |
| 298 | 24 | G | G | A | 0.90 | 4.771717e-01 |  |
| 298 | 24 | G | G | C | 0.45 | 4.985383e-01 |  |
| 298 | 24 | G | G | G | 98.64 | 1.042355e-03 | \* |
| 298 | 24 | G | G | T | 0.00 | 5.862884e-01 |  |
| 299 | 25 | C | C | A | 0.53 | 5.064925e-01 |  |
| 299 | 25 | C | C | C | 92.63 | 6.763782e-04 | \* |
| 299 | 25 | C | C | G | 0.00 | 5.807292e-01 |  |
| 299 | 25 | C | C | T | 6.84 | 2.406931e-01 |  |
| 300 | 26 | A | A | A | 94.44 | 1.342427e-04 | \* |
| 300 | 26 | A | A | C | 1.39 | 4.289836e-01 |  |
| 300 | 26 | A | A | G | 4.17 | 3.300510e-01 |  |
| 300 | 26 | A | A | T | 0.00 | 5.862884e-01 |  |
| 301 | 27 | C | C | A | 0.00 | 5.717489e-01 |  |
| 301 | 27 | C | C | C | 94.62 | 5.958240e-04 | \* |
| 301 | 27 | C | C | G | 1.08 | 4.588079e-01 |  |
| 301 | 27 | C | C | T | 4.30 | 3.174263e-01 |  |
| 302 | 28 | T | T | A | 1.69 | 4.280257e-01 |  |
| 302 | 28 | T | T | C | 0.56 | 4.878647e-01 |  |
| 302 | 28 | T | T | G | 1.13 | 4.554157e-01 |  |
| 302 | 28 | T | T | T | 96.61 | 7.560271e-05 | \* |
| 303 | 29 | A | A | A | 96.86 | 1.101441e-04 | \* |
| 303 | 29 | A | A | C | 1.05 | 4.505213e-01 |  |
| 303 | 29 | A | A | G | 2.09 | 4.053337e-01 |  |
| 303 | 29 | A | A | T | 0.00 | 5.862884e-01 |  |
| 304 | 30 | C | C | A | 0.58 | 5.018583e-01 |  |
| 304 | 30 | C | C | C | 94.77 | 5.903988e-04 | \* |
| 304 | 30 | C | C | G | 0.00 | 5.807292e-01 |  |
| 304 | 30 | C | C | T | 4.65 | 3.051780e-01 |  |
| 305 | 31 | A | A | A | 92.13 | 1.623589e-04 | \* |
| 305 | 31 | A | A | C | 1.57 | 4.183838e-01 |  |
| 305 | 31 | A | A | G | 6.30 | 2.747071e-01 |  |
| 305 | 31 | A | A | T | 0.00 | 5.862884e-01 |  |
| 306 | 32 | C | C | A | 0.00 | 5.717489e-01 |  |
| 306 | 32 | C | C | C | 90.85 | 7.577155e-04 | \* |
| 306 | 32 | C | C | G | 3.92 | 3.375545e-01 |  |
| 306 | 32 | C | C | T | 5.23 | 2.862769e-01 |  |
| 307 | 33 | G | G | A | 3.91 | 3.296547e-01 |  |
| 307 | 33 | G | G | C | 0.00 | 5.780051e-01 |  |
| 307 | 33 | G | G | G | 96.09 | 1.201199e-03 | \* |
| 307 | 33 | G | G | T | 0.00 | 5.862884e-01 |  |
| 308 | 34 | G | G | A | 0.53 | 5.060142e-01 |  |
| 308 | 34 | G | G | C | 1.60 | 4.172309e-01 |  |
| 308 | 34 | G | G | G | 97.34 | 1.120658e-03 | \* |
| 308 | 34 | G | G | T | 0.53 | 5.175620e-01 |  |
| 309 | 35 | G | G | A | 3.80 | 3.336495e-01 |  |
| 309 | 35 | G | G | C | 0.63 | 4.818844e-01 |  |
| 309 | 35 | G | G | G | 95.57 | 1.236785e-03 | \* |
| 309 | 35 | G | G | T | 0.00 | 5.862884e-01 |  |
| 310 | 36 | C | C | A | 0.66 | 4.956616e-01 |  |
| 310 | 36 | C | C | C | 94.74 | 5.915492e-04 | \* |
| 310 | 36 | C | C | G | 0.66 | 4.880400e-01 |  |
| 310 | 36 | C | C | T | 3.95 | 3.304746e-01 |  |
| 311 | 37 | A | A | A | 97.08 | 1.081621e-04 | \* |
| 311 | 37 | A | A | C | 1.46 | 4.248569e-01 |  |
| 311 | 37 | A | A | G | 1.46 | 4.364589e-01 |  |
| 311 | 37 | A | A | T | 0.00 | 5.862884e-01 |  |
| 312 | 38 | C | C | A | 0.00 | 5.717489e-01 |  |
| 312 | 38 | C | C | C | 94.36 | 6.059427e-04 | \* |
| 312 | 38 | C | C | G | 1.03 | 4.619596e-01 |  |
| 312 | 38 | C | C | T | 4.62 | 3.064010e-01 |  |
| 313 | 39 | C | C | A | 0.57 | 5.026855e-01 |  |
| 313 | 39 | C | C | C | 92.00 | 7.041470e-04 | \* |
| 313 | 39 | C | C | G | 1.71 | 4.232692e-01 |  |
| 313 | 39 | C | C | T | 5.71 | 2.715216e-01 |  |
| 314 | 40 | C | C | A | 2.25 | 3.993401e-01 |  |
| 314 | 40 | C | C | C | 92.13 | 6.981237e-04 | \* |
| 314 | 40 | C | C | G | 1.69 | 4.247143e-01 |  |
| 314 | 40 | C | C | T | 3.93 | 3.310356e-01 |  |
| 315 | 41 | T | T | A | 3.08 | 3.619521e-01 |  |
| 315 | 41 | T | T | C | 0.00 | 5.780051e-01 |  |
| 315 | 41 | T | T | G | 1.54 | 4.322674e-01 |  |
| 315 | 41 | T | T | T | 95.38 | 8.403879e-05 | \* |
| 316 | 42 | G | G | A | 0.90 | 4.774608e-01 |  |
| 316 | 42 | G | G | C | 1.35 | 4.312095e-01 |  |
| 316 | 42 | G | G | G | 97.75 | 1.095544e-03 | \* |
| 316 | 42 | G | G | T | 0.00 | 5.862884e-01 |  |
| 317 | 43 | A | A | A | 96.05 | 1.176637e-04 | \* |
| 317 | 43 | A | A | C | 0.44 | 4.999358e-01 |  |
| 317 | 43 | A | A | G | 3.51 | 3.508868e-01 |  |
| 317 | 43 | A | A | T | 0.00 | 5.862884e-01 |  |
| 318 | 44 | C | C | A | 0.54 | 5.050318e-01 |  |
| 318 | 44 | C | C | C | 91.30 | 7.360671e-04 | \* |
| 318 | 44 | C | C | G | 4.35 | 3.246849e-01 |  |
| 318 | 44 | C | C | T | 3.80 | 3.359574e-01 |  |
| 319 | 45 | C | C | A | 3.23 | 3.558227e-01 |  |
| 319 | 45 | C | C | C | 91.13 | 7.443394e-04 | \* |
| 319 | 45 | C | C | G | 1.61 | 4.283968e-01 |  |
| 319 | 45 | C | C | T | 4.03 | 3.272779e-01 |  |
| 320 | 46 | G | G | A | 7.43 | 2.281193e-01 |  |
| 320 | 46 | G | G | C | 0.68 | 4.782705e-01 |  |
| 320 | 46 | G | G | G | 91.89 | 1.518388e-03 | \* |
| 320 | 46 | G | G | T | 0.00 | 5.862884e-01 |  |
| 321 | 47 | A | A | A | 93.50 | 1.451008e-04 | \* |
| 321 | 47 | A | A | C | 0.41 | 5.032420e-01 |  |
| 321 | 47 | A | A | G | 1.63 | 4.277245e-01 |  |
| 321 | 47 | A | A | T | 4.47 | 3.113830e-01 |  |
| 322 | 48 | C | C | A | 0.00 | 5.717489e-01 |  |
| 322 | 48 | C | C | C | 93.04 | 6.590972e-04 | \* |
| 322 | 48 | C | C | G | 2.53 | 3.867283e-01 |  |
| 322 | 48 | C | C | T | 4.43 | 3.128283e-01 |  |
| 323 | 49 | G | G | A | 16.87 | 9.354533e-02 |  |
| 323 | 49 | G | G | C | 1.20 | 4.402093e-01 |  |
| 323 | 49 | G | G | G | 81.93 | 2.654108e-03 | \* |
| 323 | 49 | G | G | T | 0.00 | 5.862884e-01 |  |
| 324 | 50 | G | G | A | 2.88 | 3.701090e-01 |  |
| 324 | 50 | G | G | C | 0.48 | 4.957515e-01 |  |
| 324 | 50 | G | G | G | 96.63 | 1.165561e-03 | \* |
| 324 | 50 | G | G | T | 0.00 | 5.862884e-01 |  |
| 325 | 51 | C | C | A | 2.19 | 4.021601e-01 |  |
| 325 | 51 | C | C | C | 94.16 | 6.136406e-04 | \* |
| 325 | 51 | C | C | G | 0.00 | 5.807292e-01 |  |
| 325 | 51 | C | C | T | 3.65 | 3.420307e-01 |  |
| 326 | 52 | A | A | A | 95.35 | 1.246501e-04 | \* |
| 326 | 52 | A | A | C | 0.00 | 5.780051e-01 |  |
| 326 | 52 | A | A | G | 0.00 | 5.807292e-01 |  |
| 326 | 52 | A | A | T | 4.65 | 3.051780e-01 |  |
| 327 | 53 | A | A | A | 98.60 | 9.549278e-05 | \* |
| 327 | 53 | A | A | C | 0.00 | 5.780051e-01 |  |
| 327 | 53 | A | A | G | 0.00 | 5.807292e-01 |  |
| 327 | 53 | A | A | T | 1.40 | 4.534054e-01 |  |
| 328 | 54 | G | G | A | 0.90 | 4.774608e-01 |  |
| 328 | 54 | G | G | C | 7.21 | 2.351319e-01 |  |
| 328 | 54 | G | G | G | 91.89 | 1.518388e-03 | \* |
| 328 | 54 | G | G | T | 0.00 | 5.862884e-01 |  |
| 329 | 55 | A | A | A | 99.40 | 8.946652e-05 | \* |
| 329 | 55 | A | A | C | 0.00 | 5.780051e-01 |  |
| 329 | 55 | A | A | G | 0.00 | 5.807292e-01 |  |
| 329 | 55 | A | A | T | 0.60 | 5.113699e-01 |  |
| 330 | 56 | A | A | A | 98.66 | 9.505342e-05 | \* |
| 330 | 56 | A | A | C | 0.00 | 5.780051e-01 |  |
| 330 | 56 | A | A | G | 0.00 | 5.807292e-01 |  |
| 330 | 56 | A | A | T | 1.34 | 4.569944e-01 |  |
| 331 | 57 | G | G | A | 0.00 | 5.717489e-01 |  |
| 331 | 57 | G | G | C | 8.43 | 2.113538e-01 |  |
| 331 | 57 | G | G | G | 89.16 | 1.769231e-03 | \* |
| 331 | 57 | G | G | T | 2.41 | 3.970170e-01 |  |
| 332 | 58 | T | T | A | 3.45 | 3.469471e-01 |  |
| 332 | 58 | T | T | C | 0.00 | 5.780051e-01 |  |
| 332 | 58 | T | T | G | 0.00 | 5.807292e-01 |  |
| 332 | 58 | T | T | T | 96.55 | 7.598501e-05 | \* |
| 333 | 59 | T | T | A | 2.56 | 3.843562e-01 |  |
| 333 | 59 | T | T | C | 0.51 | 4.926810e-01 |  |
| 333 | 59 | T | T | G | 0.00 | 5.807292e-01 |  |
| 333 | 59 | T | T | T | 96.92 | 7.358851e-05 | \* |
| 334 | 60 | C | C | A | 0.00 | 5.717489e-01 |  |
| 334 | 60 | C | C | C | 98.66 | 4.610893e-04 | \* |
| 334 | 60 | C | C | G | 1.34 | 4.429451e-01 |  |
| 334 | 60 | C | C | T | 0.00 | 5.862884e-01 |  |
| 335 | 61 | G | G | A | 39.77 | 1.265891e-02 |  |
| 335 | 61 | G | G | C | 0.57 | 4.875750e-01 |  |
| 335 | 61 | G | G | G | 58.52 | 1.005714e-02 |  |
| 335 | 61 | G | G | T | 1.14 | 4.706569e-01 |  |
| 336 | 62 | A | A | A | 96.60 | 1.124851e-04 | \* |
| 336 | 62 | A | A | C | 0.49 | 4.952988e-01 |  |
| 336 | 62 | A | A | G | 2.91 | 3.719344e-01 |  |
| 336 | 62 | A | A | T | 0.00 | 5.862884e-01 |  |
| 337 | 63 | C | C | A | 0.00 | 5.717489e-01 |  |
| 337 | 63 | C | C | C | 92.44 | 6.846014e-04 | \* |
| 337 | 63 | C | C | G | 3.49 | 3.515701e-01 |  |
| 337 | 63 | C | C | T | 4.07 | 3.258788e-01 |  |
| 338 | 64 | A | A | A | 98.84 | 9.366669e-05 | \* |
| 338 | 64 | A | A | C | 0.00 | 5.780051e-01 |  |
| 338 | 64 | A | A | G | 1.16 | 4.534159e-01 |  |
| 338 | 64 | A | A | T | 0.00 | 5.862884e-01 |  |
| 339 | 65 | G | G | A | 0.60 | 5.007173e-01 |  |
| 339 | 65 | G | G | C | 0.60 | 4.851621e-01 |  |
| 339 | 65 | G | G | G | 97.62 | 1.103417e-03 | \* |
| 339 | 65 | G | G | T | 1.19 | 4.669792e-01 |  |
| 340 | 66 | C | C | A | 0.00 | 5.717489e-01 |  |
| 340 | 66 | C | C | C | 94.84 | 5.877282e-04 | \* |
| 340 | 66 | C | C | G | 0.00 | 5.807292e-01 |  |
| 340 | 66 | C | C | T | 5.16 | 2.884061e-01 |  |
| 341 | 67 | T | T | A | 1.20 | 4.570663e-01 |  |
| 341 | 67 | T | T | C | 0.60 | 4.845310e-01 |  |
| 341 | 67 | T | T | G | 0.00 | 5.807292e-01 |  |
| 341 | 67 | T | T | T | 98.19 | 6.595301e-05 | \* |
| 342 | 68 | C | C | A | 0.00 | 5.717489e-01 |  |
| 342 | 68 | C | C | C | 100.00 | 4.234424e-04 | \* |
| 342 | 68 | C | C | G | 0.00 | 5.807292e-01 |  |
| 342 | 68 | C | C | T | 0.00 | 5.862884e-01 |  |
| 343 | 69 | C | C | A | 0.00 | 5.717489e-01 |  |
| 343 | 69 | C | C | C | 92.59 | 6.780599e-04 | \* |
| 343 | 69 | C | C | G | 2.12 | 4.043429e-01 |  |
| 343 | 69 | C | C | T | 5.29 | 2.843300e-01 |  |
| 344 | 70 | C | C | A | 0.00 | 5.717489e-01 |  |
| 344 | 70 | C | C | C | 91.30 | 7.360671e-04 | \* |
| 344 | 70 | C | C | G | 1.45 | 4.370315e-01 |  |
| 344 | 70 | C | C | T | 7.25 | 2.306768e-01 |  |
| 345 | 71 | G | G | A | 3.42 | 3.481041e-01 |  |
| 345 | 71 | G | G | C | 0.85 | 4.641819e-01 |  |
| 345 | 71 | G | G | G | 95.73 | 1.226023e-03 | \* |
| 345 | 71 | G | G | T | 0.00 | 5.862884e-01 |  |
| 346 | 72 | C | C | A | 1.48 | 4.401831e-01 |  |
| 346 | 72 | C | C | C | 94.07 | 6.170282e-04 | \* |
| 346 | 72 | C | C | G | 0.00 | 5.807292e-01 |  |
| 346 | 72 | C | C | T | 4.44 | 3.123335e-01 |  |
| 347 | 73 | G | G | A | 5.26 | 2.848541e-01 |  |
| 347 | 73 | G | G | C | 0.00 | 5.780051e-01 |  |
| 347 | 73 | G | G | G | 93.86 | 1.360472e-03 | \* |
| 347 | 73 | G | G | T | 0.88 | 4.892817e-01 |  |
| 348 | 74 | A | A | A | 97.63 | 1.033732e-04 | \* |
| 348 | 74 | A | A | C | 1.78 | 4.076607e-01 |  |
| 348 | 74 | A | A | G | 0.59 | 4.934028e-01 |  |
| 348 | 74 | A | A | T | 0.00 | 5.862884e-01 |  |
| 349 | 75 | C | G | A | 0.00 | 5.717489e-01 |  |
| 349 | 75 | C | G | C | 0.63 | 4.822264e-01 |  |
| 349 | 75 | C | G | G | 99.37 | 1.000980e-03 | \* |
| 349 | 75 | C | G | T | 0.00 | 5.862884e-01 |  |

## For use in R

If you want to work with the results in R, here is output that you can copy and paste in your terminal to get:

The base information:

```
structure(list(focal.base = c("A", "C", "G", "T"), avg.percsignal = c(84.3290570095147, 
84.7035655864513, 86.0633738988988, 83.777050508559), avg.areasignal = c(223.858333333333, 
200.651933701657, 254.913978494624, 204.176470588235), crit.perc.area = c(42.5481675578338, 
51.0585108333692, 58.6214577212847, 40.7451474637562), mu = c(8.92907670615443, 
9.68666502056118, 11.0606282230699, 8.52268838335549), fillibens = c(0.990182274664663, 
0.987960191286826, 0.987935058774441, 0.989041094969012)), .Names = c("focal.base", 
"avg.percsignal", "avg.areasignal", "crit.perc.area", "mu", "fillibens"
), row.names = c(NA, -4L), class = "data.frame")
```

the data.frame that contains information on the guide region:

```
structure(list(A.area = c(113, 165, 2, 336, 2, 6, 3, 4, 2, 1, 
125, 3, 332, 2, 6, 5, 1, 1, 2, 2, 2, 0, 2, 2, 1, 136, 0, 3, 185, 
1, 117, 0, 5, 1, 6, 1, 133, 0, 1, 4, 4, 2, 219, 1, 4, 11, 230, 
0, 14, 6, 3, 41, 141, 1, 165, 147, 0, 7, 5, 0, 70, 199, 0, 85, 
1, 0, 2, 0, 0, 0, 4, 2, 6, 165, 0), C.area = c(0, 2, 2, 2, 172, 
1, 4, 3, 128, 178, 1, 2, 2, 180, 102, 0, 1, 0, 0, 0, 0, 2, 1, 
1, 176, 2, 176, 1, 2, 163, 2, 139, 0, 3, 1, 144, 2, 184, 161, 
164, 0, 3, 1, 168, 113, 1, 1, 147, 1, 1, 129, 0, 0, 8, 0, 0, 
7, 0, 1, 147, 1, 1, 159, 0, 1, 147, 1, 140, 175, 63, 1, 127, 
0, 3, 1), G.area = c(4, 3, 134, 10, 0, 4, 368, 221, 0, 3, 7, 
141, 8, 8, 3, 193, 3, 317, 2, 7, 4, 267, 2, 218, 0, 6, 2, 2, 
4, 0, 8, 6, 123, 183, 151, 1, 2, 2, 3, 3, 2, 217, 8, 8, 2, 136, 
4, 4, 68, 201, 0, 0, 0, 102, 0, 0, 74, 0, 0, 2, 103, 6, 6, 1, 
164, 0, 0, 0, 4, 1, 112, 0, 107, 1, 158), T.area = c(0, 2, 3, 
0, 7, 170, 1, 0, 7, 8, 0, 1, 0, 8, 8, 2, 166, 2, 187, 211, 200, 
2, 193, 0, 13, 0, 8, 171, 0, 8, 0, 8, 0, 1, 0, 6, 0, 9, 10, 7, 
124, 0, 0, 7, 5, 0, 11, 7, 0, 0, 5, 2, 2, 0, 1, 2, 2, 196, 189, 
0, 2, 0, 7, 0, 2, 8, 163, 0, 10, 5, 0, 6, 1, 0, 0), Tot.area = c(117, 
172, 141, 348, 181, 181, 376, 228, 137, 190, 133, 147, 342, 198, 
119, 200, 171, 320, 191, 220, 206, 271, 198, 221, 190, 144, 186, 
177, 191, 172, 127, 153, 128, 188, 158, 152, 137, 195, 175, 178, 
130, 222, 228, 184, 124, 148, 246, 158, 83, 208, 137, 43, 143, 
111, 166, 149, 83, 203, 195, 149, 176, 206, 172, 86, 168, 155, 
166, 140, 189, 69, 117, 135, 114, 169, 159), A.perc = c(96.5811965811966, 
95.9302325581395, 1.41843971631206, 96.551724137931, 1.10497237569061, 
3.31491712707182, 0.797872340425532, 1.75438596491228, 1.45985401459854, 
0.526315789473684, 93.984962406015, 2.04081632653061, 97.0760233918129, 
1.01010101010101, 5.04201680672269, 2.5, 0.584795321637427, 0.3125, 
1.04712041884817, 0.909090909090909, 0.970873786407767, 0, 1.01010101010101, 
0.904977375565611, 0.526315789473684, 94.4444444444444, 0, 1.69491525423729, 
96.8586387434555, 0.581395348837209, 92.1259842519685, 0, 3.90625, 
0.531914893617021, 3.79746835443038, 0.657894736842105, 97.0802919708029, 
0, 0.571428571428571, 2.24719101123596, 3.07692307692308, 0.900900900900901, 
96.0526315789474, 0.543478260869565, 3.2258064516129, 7.43243243243243, 
93.4959349593496, 0, 16.8674698795181, 2.88461538461538, 2.18978102189781, 
95.3488372093023, 98.6013986013986, 0.900900900900901, 99.3975903614458, 
98.6577181208054, 0, 3.44827586206897, 2.56410256410256, 0, 39.7727272727273, 
96.6019417475728, 0, 98.8372093023256, 0.595238095238095, 0, 
1.20481927710843, 0, 0, 0, 3.41880341880342, 1.48148148148148, 
5.26315789473684, 97.6331360946746, 0), C.perc = c(0, 1.16279069767442, 
1.41843971631206, 0.574712643678161, 95.0276243093923, 0.552486187845304, 
1.06382978723404, 1.31578947368421, 93.4306569343066, 93.6842105263158, 
0.75187969924812, 1.36054421768707, 0.584795321637427, 90.9090909090909, 
85.7142857142857, 0, 0.584795321637427, 0, 0, 0, 0, 0.738007380073801, 
0.505050505050505, 0.452488687782805, 92.6315789473684, 1.38888888888889, 
94.6236559139785, 0.564971751412429, 1.04712041884817, 94.7674418604651, 
1.5748031496063, 90.8496732026144, 0, 1.59574468085106, 0.632911392405063, 
94.7368421052632, 1.45985401459854, 94.3589743589744, 92, 92.1348314606742, 
0, 1.35135135135135, 0.43859649122807, 91.304347826087, 91.1290322580645, 
0.675675675675676, 0.40650406504065, 93.0379746835443, 1.20481927710843, 
0.480769230769231, 94.1605839416058, 0, 0, 7.20720720720721, 
0, 0, 8.43373493975904, 0, 0.512820512820513, 98.6577181208054, 
0.568181818181818, 0.485436893203884, 92.4418604651163, 0, 0.595238095238095, 
94.8387096774194, 0.602409638554217, 100, 92.5925925925926, 91.304347826087, 
0.854700854700855, 94.0740740740741, 0, 1.77514792899408, 0.628930817610063
), G.perc = c(3.41880341880342, 1.74418604651163, 95.0354609929078, 
2.8735632183908, 0, 2.20994475138122, 97.8723404255319, 96.9298245614035, 
0, 1.57894736842105, 5.26315789473684, 95.9183673469388, 2.33918128654971, 
4.04040404040404, 2.52100840336134, 96.5, 1.75438596491228, 99.0625, 
1.04712041884817, 3.18181818181818, 1.94174757281553, 98.5239852398524, 
1.01010101010101, 98.6425339366516, 0, 4.16666666666667, 1.0752688172043, 
1.12994350282486, 2.09424083769634, 0, 6.2992125984252, 3.92156862745098, 
96.09375, 97.3404255319149, 95.5696202531646, 0.657894736842105, 
1.45985401459854, 1.02564102564103, 1.71428571428571, 1.68539325842697, 
1.53846153846154, 97.7477477477478, 3.50877192982456, 4.34782608695652, 
1.61290322580645, 91.8918918918919, 1.6260162601626, 2.53164556962025, 
81.9277108433735, 96.6346153846154, 0, 0, 0, 91.8918918918919, 
0, 0, 89.1566265060241, 0, 0, 1.34228187919463, 58.5227272727273, 
2.9126213592233, 3.48837209302326, 1.16279069767442, 97.6190476190476, 
0, 0, 0, 2.11640211640212, 1.44927536231884, 95.7264957264957, 
0, 93.859649122807, 0.591715976331361, 99.3710691823899), T.perc = c(0, 
1.16279069767442, 2.12765957446809, 0, 3.86740331491713, 93.9226519337017, 
0.265957446808511, 0, 5.10948905109489, 4.21052631578947, 0, 
0.680272108843537, 0, 4.04040404040404, 6.72268907563025, 1, 
97.0760233918129, 0.625, 97.9057591623037, 95.9090909090909, 
97.0873786407767, 0.738007380073801, 97.4747474747475, 0, 6.84210526315789, 
0, 4.3010752688172, 96.6101694915254, 0, 4.65116279069767, 0, 
5.22875816993464, 0, 0.531914893617021, 0, 3.94736842105263, 
0, 4.61538461538461, 5.71428571428571, 3.93258426966292, 95.3846153846154, 
0, 0, 3.80434782608696, 4.03225806451613, 0, 4.47154471544715, 
4.43037974683544, 0, 0, 3.64963503649635, 4.65116279069767, 1.3986013986014, 
0, 0.602409638554217, 1.34228187919463, 2.40963855421687, 96.551724137931, 
96.9230769230769, 0, 1.13636363636364, 0, 4.06976744186047, 0, 
1.19047619047619, 5.16129032258065, 98.1927710843374, 0, 5.29100529100529, 
7.2463768115942, 0, 4.44444444444444, 0.87719298245614, 0, 0), 
    base.call = c("A", "A", "G", "A", "C", "T", "G", "G", "C", 
    "C", "A", "G", "A", "C", "C", "G", "T", "G", "T", "T", "T", 
    "G", "T", "G", "C", "A", "C", "T", "A", "C", "A", "C", "G", 
    "G", "G", "C", "A", "C", "C", "C", "T", "G", "A", "C", "C", 
    "G", "A", "C", "G", "G", "C", "A", "A", "G", "A", "A", "G", 
    "T", "T", "C", "G", "A", "C", "A", "G", "C", "T", "C", "C", 
    "C", "G", "C", "G", "A", "G"), index = 275:349, guide.seq = c("A", 
    "A", "G", "A", "C", "T", "G", "G", "C", "C", "A", "G", "A", 
    "C", "C", "G", "T", "G", "T", "T", "T", "G", "T", "G", "C", 
    "A", "C", "T", "A", "C", "A", "C", "G", "G", "G", "C", "A", 
    "C", "C", "C", "T", "G", "A", "C", "C", "G", "A", "C", "G", 
    "G", "C", "A", "A", "G", "A", "A", "G", "T", "T", "C", "G", 
    "A", "C", "A", "G", "C", "T", "C", "C", "C", "G", "C", "G", 
    "A", "C"), T.pval = c(0.58628841607565, 0.468852607048829, 
    0.411443081540424, 0.58628841607565, 0.333524839140398, 9.53469177795707e-05, 
    0.543965226175484, 0.58628841607565, 0.290054559731645, 0.320699812571548, 
    0.58628841607565, 0.504808923072773, 0.58628841607565, 0.326973409283974, 
    0.243749974229128, 0.480233253084377, 7.26236732575547e-05, 
    0.509438112770079, 6.76063404827509e-05, 8.03187060640642e-05, 
    7.25525484648326e-05, 0.500109729963793, 7.0167630535467e-05, 
    0.58628841607565, 0.240693103593044, 0.58628841607565, 0.317426338933798, 
    7.56027099373391e-05, 0.58628841607565, 0.305177999945751, 
    0.58628841607565, 0.286276867993474, 0.58628841607565, 0.517562007278708, 
    0.58628841607565, 0.330474580385772, 0.58628841607565, 0.30640099879167, 
    0.271521559146347, 0.33103563826125, 8.40387918932262e-05, 
    0.58628841607565, 0.58628841607565, 0.335957374097722, 0.327277946284949, 
    0.58628841607565, 0.311382998960128, 0.312828325816375, 0.58628841607565, 
    0.58628841607565, 0.342030704915758, 0.305177999945751, 0.453405354889534, 
    0.58628841607565, 0.51136994003451, 0.45699438515646, 0.397017015354077, 
    7.59850109595117e-05, 7.35885148088755e-05, 0.58628841607565, 
    0.470656903886806, 0.58628841607565, 0.32587883726299, 0.58628841607565, 
    0.466979226015285, 0.288406113656477, 6.59530088171767e-05, 
    0.58628841607565, 0.284329965555864, 0.230676781114917, 0.58628841607565, 
    0.312333513789639, 0.489281671405417, 0.58628841607565, 0.58628841607565
    ), C.pval = c(0.578005115089508, 0.442889551629862, 0.427252510579217, 
    0.486987891028291, 0.00058070812185651, 0.488998777360662, 
    0.44939432749048, 0.433347048078725, 0.000642822593153958, 
    0.000632530232689499, 0.472083434241888, 0.430661507284289, 
    0.486087380467105, 0.000754850363380521, 0.00105182725535324, 
    0.578005115089508, 0.486087380467105, 0.578005115089508, 
    0.578005115089508, 0.578005115089508, 0.578005115089508, 
    0.473187266108755, 0.493417254709921, 0.498538263129873, 
    0.000676378195328287, 0.428983566328748, 0.000595824004363421, 
    0.487864738515657, 0.45052126338525, 0.000590398774354961, 
    0.418383790699036, 0.000757715538110881, 0.578005115089508, 
    0.417230944176393, 0.481884424792994, 0.000591549152395454, 
    0.424856945778326, 0.000605942662077141, 0.0007041469618847, 
    0.000698123748744894, 0.578005115089508, 0.4312094736628, 
    0.499935770043317, 0.000736067101771876, 0.000744339446659636, 
    0.478270543008196, 0.503241984944002, 0.000659097160026878, 
    0.440209328270566, 0.49575154574499, 0.000613640585363506, 
    0.578005115089508, 0.578005115089508, 0.235131885968549, 
    0.578005115089508, 0.578005115089508, 0.211353842770354, 
    0.578005115089508, 0.492681030696739, 0.000461089323479769, 
    0.48757502236433, 0.495298764692013, 0.000684601413670105, 
    0.578005115089508, 0.48516214580487, 0.000587728249534569, 
    0.484531032874018, 0.000423442398598706, 0.000678059854220647, 
    0.000736067101771876, 0.464181908432323, 0.000617028158263033, 
    0.578005115089508, 0.407660702368346, 0.482226445448515), 
    G.pval = c(0.353918684478053, 0.421786918181723, 0.00127414919220303, 
    0.373398304599916, 0.580729166666658, 0.400221167268663, 
    0.00108797645293801, 0.00114656272075575, 0.580729166666658, 
    0.430150833674998, 0.29962647385826, 0.0012129894429963, 
    0.39467906888168, 0.333880293279088, 0.387158784192579, 0.00117432962328912, 
    0.421284258366486, 0.00101829777226592, 0.460586722534702, 
    0.36214189902619, 0.41230798960372, 0.00104924871465173, 
    0.462961537510524, 0.00104235453810564, 0.580729166666658, 
    0.33005095025448, 0.458807864377856, 0.455415696971432, 0.405333682504052, 
    0.580729166666658, 0.274707050634248, 0.337554527032466, 
    0.00120119851601863, 0.00112065768497627, 0.00123678457932941, 
    0.488040049018383, 0.436458872742362, 0.461959639433866, 
    0.423269238390405, 0.424714298139804, 0.432267393165063, 
    0.00109554405698209, 0.350886771748, 0.324684900633105, 0.428396818155883, 
    0.00151838842433272, 0.42772449558812, 0.386728285218149, 
    0.0026541077805855, 0.0011655610313569, 0.580729166666658, 
    0.580729166666658, 0.580729166666658, 0.00151838842433272, 
    0.580729166666658, 0.580729166666658, 0.00176923147130525, 
    0.580729166666658, 0.580729166666658, 0.442945115282282, 
    0.0100571432311441, 0.371934414602391, 0.351570070560234, 
    0.453415923800542, 0.00110341702413208, 0.580729166666658, 
    0.580729166666658, 0.580729166666658, 0.40434292897788, 0.437031528396632, 
    0.0012260230434914, 0.580729166666658, 0.00136047187353328, 
    0.493402839527783, 0.00100098039546792), A.pval = c(0.000112676407449719, 
    0.000118849839112589, 0.443910423705496, 0.000112948824136594, 
    0.463526133276981, 0.352227898985506, 0.484933242821042, 
    0.424755529990539, 0.441454377400597, 0.506492536777406, 
    0.000139396795734825, 0.40964163253976, 0.000108199917386975, 
    0.469873167809896, 0.291582278765912, 0.387311086722295, 
    0.501577193611099, 0.526300770959148, 0.467370907882558, 
    0.476880394951604, 0.472562254780827, 0.571748878923765, 
    0.469873167809896, 0.47717165399005, 0.506492536777406, 0.000134242707025001, 
    0.571748878923765, 0.428025671271888, 0.000110144089351394, 
    0.501858284454902, 0.000162358903222581, 0.571748878923765, 
    0.329654726763367, 0.506014241856369, 0.333649548711114, 
    0.495661639059187, 0.000108162088337305, 0.571748878923765, 
    0.502685508726207, 0.399340108339208, 0.361952086474994, 
    0.477460765362698, 0.000117663691507008, 0.505031761450548, 
    0.355822724834622, 0.228119309262393, 0.000145100762456329, 
    0.571748878923765, 0.0935453341428008, 0.370109047844316, 
    0.402160116955894, 0.00012465006652862, 9.54927777514492e-05, 
    0.477460765362698, 8.94665166127906e-05, 9.50534239979595e-05, 
    0.571748878923765, 0.346947091855574, 0.384356164996378, 
    0.571748878923765, 0.0126589059615054, 0.00011248505343131, 
    0.571748878923765, 9.36666873243563e-05, 0.500717287355702, 
    0.571748878923765, 0.457066281086465, 0.571748878923765, 
    0.571748878923765, 0.571748878923765, 0.348104147001026, 
    0.440183076394034, 0.284854106392745, 0.000103373194997203, 
    0.571748878923765), guide.position = 1:75), .Names = c("A.area", 
"C.area", "G.area", "T.area", "Tot.area", "A.perc", "C.perc", 
"G.perc", "T.perc", "base.call", "index", "guide.seq", "T.pval", 
"C.pval", "G.pval", "A.pval", "guide.position"), row.names = 275:349, class = "data.frame")
```

*Report generated using EditR v1.0.8*
