# Supplementary material for: Mechanistic and genetic basis of single-strand templated repair at Cas12a-induced DNA breaks in Chlamydomonas reinhardtii
Source: Nat Commun. 2021 Nov 19;12:6751. doi: 10.1038/s41467-021-27004-1 (PMC8604939; doi:10.1038/s41467-021-27004-1)
Supplement: Supplementary file 22 — Source Data [file 41467_2021_27004_MOESM22_ESM.zip › Source Data/EditR analysis/EditR outputs/Controls for normalization/Positive control (-32).html]

EditR v1.0.8 report


# EditR v1.0.8 report

- Data QA
  - Filtering data
  - Percent noise peak area
  - Base information
- Predicted editing
  - Editing bar plot
  - Editing table plot
  - Table of editing results
- For use in R

## Data QA

### Filtering data

What the data looked like prefiltering:

and the post filtering signal / noise plot:

### Percent noise peak area

### Base information

Here’s information about the signal of each base, the critical percent value where any higher value would be called as significant, and Filliben’s correlation for how well the noise was modelled by the zero adjusted gamma distribution.

| Base | Average percent signal | Average peak area | Critical percent value | model mu | Fillibens correlation |
| --- | --- | --- | --- | --- | --- |
| A | 86.10371 | 177.4706 | 15.93444 | 4.012047 | 0.9690965 |
| C | 87.84079 | 185.1077 | 32.90070 | 5.999267 | 0.9658514 |
| G | 84.41843 | 190.7368 | 20.20653 | 4.133254 | 0.9384428 |
| T | 87.71209 | 203.7333 | 15.13187 | 3.718458 | 0.9650463 |

## Predicted editing

### Editing bar plot

### Editing table plot

### Table of editing results


Here’s the entire guide region

| Sanger position | Guide position | Guide sequence | Sanger base call | Focal base | Focal base peak area | p value |  |
| --- | --- | --- | --- | --- | --- | --- | --- |
| 275 | 1 | A | A | A | 96.84 | 9.299228e-13 | \* |
| 275 | 1 | A | A | C | 0.00 | 8.139535e-01 |  |
| 275 | 1 | A | A | G | 3.16 | 3.454410e-01 |  |
| 275 | 1 | A | A | T | 0.00 | 7.741935e-01 |  |
| 276 | 2 | A | A | A | 95.15 | 1.507017e-12 | \* |
| 276 | 2 | A | A | C | 1.32 | 5.708054e-01 |  |
| 276 | 2 | A | A | G | 3.08 | 3.518307e-01 |  |
| 276 | 2 | A | A | T | 0.44 | 7.062303e-01 |  |
| 277 | 3 | G | G | A | 2.60 | 3.846394e-01 |  |
| 277 | 3 | G | G | C | 1.56 | 5.456863e-01 |  |
| 277 | 3 | G | G | G | 95.83 | 3.529565e-09 | \* |
| 277 | 3 | G | G | T | 0.00 | 7.741935e-01 |  |
| 278 | 4 | A | A | A | 98.61 | 5.589973e-13 | \* |
| 278 | 4 | A | A | C | 0.46 | 6.857942e-01 |  |
| 278 | 4 | A | A | G | 0.93 | 5.897577e-01 |  |
| 278 | 4 | A | A | T | 0.00 | 7.741935e-01 |  |
| 279 | 5 | C | C | A | 2.40 | 4.048877e-01 |  |
| 279 | 5 | C | C | C | 90.38 | 1.504602e-05 | \* |
| 279 | 5 | C | C | G | 3.37 | 3.301318e-01 |  |
| 279 | 5 | C | C | T | 3.85 | 2.810575e-01 |  |
| 280 | 6 | T | T | A | 2.34 | 4.119070e-01 |  |
| 280 | 6 | T | T | C | 0.47 | 6.852324e-01 |  |
| 280 | 6 | T | T | G | 3.27 | 3.372271e-01 |  |
| 280 | 6 | T | T | T | 93.93 | 3.445022e-13 | \* |
| 281 | 7 | G | G | A | 1.26 | 5.383523e-01 |  |
| 281 | 7 | G | G | C | 0.84 | 6.288184e-01 |  |
| 281 | 7 | G | G | G | 97.48 | 2.561998e-09 | \* |
| 281 | 7 | G | G | T | 0.42 | 7.096134e-01 |  |
| 282 | 8 | G | G | A | 1.37 | 5.242839e-01 |  |
| 282 | 8 | G | G | C | 0.68 | 6.506700e-01 |  |
| 282 | 8 | G | G | G | 97.95 | 2.339721e-09 | \* |
| 282 | 8 | G | G | T | 0.00 | 7.741935e-01 |  |
| 283 | 9 | C | C | A | 1.75 | 4.769555e-01 |  |
| 283 | 9 | C | C | C | 91.23 | 1.369890e-05 | \* |
| 283 | 9 | C | C | G | 3.51 | 3.196686e-01 |  |
| 283 | 9 | C | C | T | 3.51 | 3.093521e-01 |  |
| 284 | 10 | C | C | A | 0.00 | 7.064220e-01 |  |
| 284 | 10 | C | C | C | 94.47 | 9.556677e-06 | \* |
| 284 | 10 | C | C | G | 2.98 | 3.603286e-01 |  |
| 284 | 10 | C | C | T | 2.55 | 4.045910e-01 |  |
| 285 | 11 | A | T | A | 1.01 | 5.721772e-01 |  |
| 285 | 11 | A | T | C | 0.50 | 6.792463e-01 |  |
| 285 | 11 | A | T | G | 2.01 | 4.511899e-01 |  |
| 285 | 11 | A | T | T | 96.48 | 1.569855e-13 | \* |
| 286 | 12 | G | G | A | 1.26 | 5.384897e-01 |  |
| 286 | 12 | G | G | C | 0.50 | 6.790344e-01 |  |
| 286 | 12 | G | G | G | 97.73 | 2.438397e-09 | \* |
| 286 | 12 | G | G | T | 0.50 | 6.957123e-01 |  |
| 287 | 13 | A | A | A | 97.58 | 7.502887e-13 | \* |
| 287 | 13 | A | A | C | 0.91 | 6.200840e-01 |  |
| 287 | 13 | A | A | G | 1.51 | 5.089790e-01 |  |
| 287 | 13 | A | A | T | 0.00 | 7.741935e-01 |  |
| 288 | 14 | C | C | A | 1.29 | 5.341316e-01 |  |
| 288 | 14 | C | C | C | 94.40 | 9.632916e-06 | \* |
| 288 | 14 | C | C | G | 2.16 | 4.359651e-01 |  |
| 288 | 14 | C | C | T | 2.16 | 4.516004e-01 |  |
| 289 | 15 | C | C | A | 4.30 | 2.466645e-01 |  |
| 289 | 15 | C | C | C | 91.40 | 1.344273e-05 | \* |
| 289 | 15 | C | C | G | 1.08 | 5.676790e-01 |  |
| 289 | 15 | C | C | T | 3.23 | 3.351269e-01 |  |
| 290 | 16 | G | G | A | 8.64 | 7.613407e-02 |  |
| 290 | 16 | G | G | C | 0.45 | 6.874430e-01 |  |
| 290 | 16 | G | G | G | 90.00 | 1.099341e-08 | \* |
| 290 | 16 | G | G | T | 0.91 | 6.292738e-01 |  |
| 291 | 17 | T | T | A | 1.05 | 5.657788e-01 |  |
| 291 | 17 | T | T | C | 0.00 | 8.139535e-01 |  |
| 291 | 17 | T | T | G | 2.11 | 4.411289e-01 |  |
| 291 | 17 | T | T | T | 96.84 | 1.405542e-13 | \* |
| 292 | 18 | G | G | A | 0.00 | 7.064220e-01 |  |
| 292 | 18 | G | G | C | 0.66 | 6.550059e-01 |  |
| 292 | 18 | G | G | G | 98.69 | 2.024538e-09 | \* |
| 292 | 18 | G | G | T | 0.66 | 6.705190e-01 |  |
| 293 | 19 | T | T | A | 22.75 | 1.468120e-03 | \* |
| 293 | 19 | T | T | C | 12.02 | 1.236261e-01 |  |
| 293 | 19 | T | T | G | 3.43 | 3.251159e-01 |  |
| 293 | 19 | T | T | T | 61.80 | 6.620083e-09 | \* |
| 294 | 20 | T | T | A | 0.00 | 7.064220e-01 |  |
| 294 | 20 | T | T | C | 17.21 | 6.467741e-02 |  |
| 294 | 20 | T | T | G | 2.33 | 4.188782e-01 |  |
| 294 | 20 | T | T | T | 80.47 | 2.154599e-11 | \* |
| 295 | 21 | T | T | A | 0.00 | 7.064220e-01 |  |
| 295 | 21 | T | T | C | 19.35 | 4.978950e-02 |  |
| 295 | 21 | T | T | G | 19.82 | 1.081765e-02 |  |
| 295 | 21 | T | T | T | 60.83 | 8.921526e-09 | \* |
| 296 | 22 | G | G | A | 5.79 | 1.657077e-01 |  |
| 296 | 22 | G | G | C | 7.44 | 2.252419e-01 |  |
| 296 | 22 | G | G | G | 85.54 | 2.623269e-08 | \* |
| 296 | 22 | G | G | T | 1.24 | 5.777154e-01 |  |
| 297 | 23 | T | T | A | 2.65 | 3.805744e-01 |  |
| 297 | 23 | T | T | C | 7.94 | 2.105796e-01 |  |
| 297 | 23 | T | T | G | 2.65 | 3.889105e-01 |  |
| 297 | 23 | T | T | T | 86.77 | 3.103517e-12 | \* |
| 298 | 24 | G | G | A | 6.28 | 1.449292e-01 |  |
| 298 | 24 | G | G | C | 11.11 | 1.388259e-01 |  |
| 298 | 24 | G | G | G | 80.19 | 7.437306e-08 | \* |
| 298 | 24 | G | G | T | 2.42 | 4.203399e-01 |  |
| 299 | 25 | C | C | A | 15.68 | 1.075239e-02 |  |
| 299 | 25 | C | C | C | 60.54 | 4.245987e-04 | \* |
| 299 | 25 | C | C | G | 5.41 | 2.107353e-01 |  |
| 299 | 25 | C | C | T | 18.38 | 3.759644e-03 | \* |
| 300 | 26 | A | A | A | 65.19 | 8.135847e-09 | \* |
| 300 | 26 | A | A | C | 2.21 | 4.870203e-01 |  |
| 300 | 26 | A | A | G | 28.73 | 1.821604e-03 | \* |
| 300 | 26 | A | A | T | 3.87 | 2.793599e-01 |  |
| 301 | 27 | C | C | A | 0.56 | 6.337295e-01 |  |
| 301 | 27 | C | C | C | 73.74 | 9.632889e-05 | \* |
| 301 | 27 | C | C | G | 21.79 | 7.280283e-03 | \* |
| 301 | 27 | C | C | T | 3.91 | 2.759379e-01 |  |
| 302 | 28 | T | T | A | 16.08 | 9.598921e-03 | \* |
| 302 | 28 | T | T | C | 0.00 | 8.139535e-01 |  |
| 302 | 28 | T | T | G | 20.10 | 1.021543e-02 |  |
| 302 | 28 | T | T | T | 63.82 | 3.566954e-09 | \* |
| 303 | 29 | A | A | A | 73.68 | 7.139165e-10 | \* |
| 303 | 29 | A | A | C | 10.53 | 1.497052e-01 |  |
| 303 | 29 | A | A | G | 0.00 | 7.888889e-01 |  |
| 303 | 29 | A | A | T | 15.79 | 8.205287e-03 | \* |
| 304 | 30 | C | C | A | 0.00 | 7.064220e-01 |  |
| 304 | 30 | C | C | C | 98.14 | 6.359602e-06 | \* |
| 304 | 30 | C | C | G | 1.86 | 4.672546e-01 |  |
| 304 | 30 | C | C | T | 0.00 | 7.741935e-01 |  |
| 305 | 31 | A | A | A | 83.49 | 4.286738e-11 | \* |
| 305 | 31 | A | A | C | 12.26 | 1.198012e-01 |  |
| 305 | 31 | A | A | G | 1.42 | 5.211076e-01 |  |
| 305 | 31 | A | A | T | 2.83 | 3.745238e-01 |  |
| 306 | 32 | C | C | A | 0.00 | 7.064220e-01 |  |
| 306 | 32 | C | C | C | 71.09 | 1.296150e-04 | \* |
| 306 | 32 | C | C | G | 6.25 | 1.757582e-01 |  |
| 306 | 32 | C | C | T | 22.66 | 1.030471e-03 | \* |
| 307 | 33 | G | G | A | 21.80 | 1.915907e-03 | \* |
| 307 | 33 | G | G | C | 18.80 | 5.327952e-02 |  |
| 307 | 33 | G | G | G | 59.40 | 4.328520e-06 | \* |
| 307 | 33 | G | G | T | 0.00 | 7.741935e-01 |  |
| 308 | 34 | G | G | A | 0.00 | 7.064220e-01 |  |
| 308 | 34 | G | G | C | 10.27 | 1.547583e-01 |  |
| 308 | 34 | G | G | G | 72.43 | 3.383034e-07 | \* |
| 308 | 34 | G | G | T | 17.30 | 5.209768e-03 | \* |
| 309 | 35 | G | G | A | 12.08 | 2.935203e-02 |  |
| 309 | 35 | G | G | C | 3.36 | 4.046962e-01 |  |
| 309 | 35 | G | G | G | 84.56 | 3.171447e-08 | \* |
| 309 | 35 | G | G | T | 0.00 | 7.741935e-01 |  |
| 310 | 36 | C | C | A | 10.13 | 5.047505e-02 |  |
| 310 | 36 | C | C | C | 68.35 | 1.762459e-04 | \* |
| 310 | 36 | C | C | G | 20.89 | 8.723911e-03 | \* |
| 310 | 36 | C | C | T | 0.63 | 6.742889e-01 |  |
| 311 | 37 | A | A | A | 91.72 | 4.034884e-12 | \* |
| 311 | 37 | A | A | C | 1.38 | 5.645883e-01 |  |
| 311 | 37 | A | A | G | 2.76 | 3.789229e-01 |  |
| 311 | 37 | A | A | T | 4.14 | 2.585823e-01 |  |
| 312 | 38 | C | C | A | 4.65 | 2.247053e-01 |  |
| 312 | 38 | C | C | C | 78.49 | 5.665974e-05 | \* |
| 312 | 38 | C | C | G | 16.86 | 1.963587e-02 |  |
| 312 | 38 | C | C | T | 0.00 | 7.741935e-01 |  |
| 313 | 39 | C | C | A | 0.62 | 6.244635e-01 |  |
| 313 | 39 | C | C | C | 91.88 | 1.274820e-05 | \* |
| 313 | 39 | C | C | G | 5.62 | 2.009834e-01 |  |
| 313 | 39 | C | C | T | 1.88 | 4.874998e-01 |  |
| 314 | 40 | C | C | A | 0.00 | 7.064220e-01 |  |
| 314 | 40 | C | C | C | 79.11 | 5.283569e-05 | \* |
| 314 | 40 | C | C | G | 17.72 | 1.649846e-02 |  |
| 314 | 40 | C | C | T | 3.16 | 3.409639e-01 |  |
| 315 | 41 | T | T | A | 21.14 | 2.309393e-03 | \* |
| 315 | 41 | T | T | C | 0.00 | 8.139535e-01 |  |
| 315 | 41 | T | T | G | 1.14 | 5.579550e-01 |  |
| 315 | 41 | T | T | T | 77.71 | 5.014922e-11 | \* |
| 316 | 42 | G | G | A | 24.84 | 8.123999e-04 | \* |
| 316 | 42 | G | G | C | 6.77 | 2.466204e-01 |  |
| 316 | 42 | G | G | G | 68.39 | 7.457247e-07 | \* |
| 316 | 42 | G | G | T | 0.00 | 7.741935e-01 |  |
| 317 | 43 | A | A | A | 90.62 | 5.531797e-12 | \* |
| 317 | 43 | A | A | C | 0.78 | 6.368996e-01 |  |
| 317 | 43 | A | A | G | 0.00 | 7.888889e-01 |  |
| 317 | 43 | A | A | T | 8.59 | 7.043719e-02 |  |
| 318 | 44 | C | C | A | 0.00 | 7.064220e-01 |  |
| 318 | 44 | C | C | C | 84.15 | 3.013511e-05 | \* |
| 318 | 44 | C | C | G | 0.00 | 7.888889e-01 |  |
| 318 | 44 | C | C | T | 15.85 | 8.048305e-03 | \* |
| 319 | 45 | C | C | A | 17.95 | 5.679829e-03 | \* |
| 319 | 45 | C | C | C | 62.18 | 3.529641e-04 | \* |
| 319 | 45 | C | C | G | 19.87 | 1.069621e-02 |  |
| 319 | 45 | C | C | T | 0.00 | 7.741935e-01 |  |
| 320 | 46 | G | G | A | 17.53 | 6.384970e-03 | \* |
| 320 | 46 | G | G | C | 24.68 | 2.629678e-02 |  |
| 320 | 46 | G | G | G | 56.49 | 7.647656e-06 | \* |
| 320 | 46 | G | G | T | 1.30 | 5.688247e-01 |  |
| 321 | 47 | A | A | A | 80.18 | 1.108132e-10 | \* |
| 321 | 47 | A | A | C | 13.96 | 9.667970e-02 |  |
| 321 | 47 | A | A | G | 5.86 | 1.912465e-01 |  |
| 321 | 47 | A | A | T | 0.00 | 7.741935e-01 |  |
| 322 | 48 | C | C | A | 0.00 | 7.064220e-01 |  |
| 322 | 48 | C | C | C | 96.64 | 7.509854e-06 | \* |
| 322 | 48 | C | C | G | 0.84 | 6.035631e-01 |  |
| 322 | 48 | C | C | T | 2.52 | 4.082227e-01 |  |
| 323 | 49 | G | G | A | 30.84 | 1.480456e-04 | \* |
| 323 | 49 | G | G | C | 0.93 | 6.164358e-01 |  |
| 323 | 49 | G | G | G | 68.22 | 7.698363e-07 | \* |
| 323 | 49 | G | G | T | 0.00 | 7.741935e-01 |  |
| 324 | 50 | G | G | A | 13.04 | 2.244646e-02 |  |
| 324 | 50 | G | G | C | 14.13 | 9.468576e-02 |  |
| 324 | 50 | G | G | G | 72.28 | 3.483497e-07 | \* |
| 324 | 50 | G | G | T | 0.54 | 6.891134e-01 |  |
| 325 | 51 | C | C | A | 14.79 | 1.378445e-02 |  |
| 325 | 51 | C | C | C | 84.51 | 2.894761e-05 | \* |
| 325 | 51 | C | C | G | 0.00 | 7.888889e-01 |  |
| 325 | 51 | C | C | T | 0.70 | 6.625351e-01 |  |
| 326 | 52 | A | A | A | 68.67 | 3.000800e-09 | \* |
| 326 | 52 | A | A | C | 0.00 | 8.139535e-01 |  |
| 326 | 52 | A | A | G | 31.33 | 1.087824e-03 | \* |
| 326 | 52 | A | A | T | 0.00 | 7.741935e-01 |  |
| 327 | 53 | A | A | A | 73.97 | 6.572624e-10 | \* |
| 327 | 53 | A | A | C | 0.00 | 8.139535e-01 |  |
| 327 | 53 | A | A | G | 10.96 | 6.551616e-02 |  |
| 327 | 53 | A | A | T | 15.07 | 1.019237e-02 |  |
| 328 | 54 | G | G | A | 0.00 | 7.064220e-01 |  |
| 328 | 54 | G | G | C | 15.27 | 8.218220e-02 |  |
| 328 | 54 | G | G | G | 84.73 | 3.068618e-08 | \* |
| 328 | 54 | G | G | T | 0.00 | 7.741935e-01 |  |
| 329 | 55 | A | A | A | 85.00 | 2.779854e-11 | \* |
| 329 | 55 | A | A | C | 14.29 | 9.286516e-02 |  |
| 329 | 55 | A | A | G | 0.00 | 7.888889e-01 |  |
| 329 | 55 | A | A | T | 0.71 | 6.608831e-01 |  |
| 330 | 56 | A | A | A | 96.75 | 9.521273e-13 | \* |
| 330 | 56 | A | A | C | 0.00 | 8.139535e-01 |  |
| 330 | 56 | A | A | G | 0.00 | 7.888889e-01 |  |
| 330 | 56 | A | A | T | 3.25 | 3.331522e-01 |  |
| 331 | 57 | G | G | A | 0.00 | 7.064220e-01 |  |
| 331 | 57 | G | G | C | 17.31 | 6.390200e-02 |  |
| 331 | 57 | G | G | G | 61.54 | 2.846852e-06 | \* |
| 331 | 57 | G | G | T | 21.15 | 1.624410e-03 | \* |
| 332 | 58 | T | T | A | 7.87 | 9.408032e-02 |  |
| 332 | 58 | T | T | C | 5.62 | 2.897937e-01 |  |
| 332 | 58 | T | T | G | 19.10 | 1.249095e-02 |  |
| 332 | 58 | T | T | T | 67.42 | 1.183434e-09 | \* |
| 333 | 59 | T | T | A | 10.97 | 3.998458e-02 |  |
| 333 | 59 | T | T | C | 1.29 | 5.742290e-01 |  |
| 333 | 59 | T | T | G | 0.00 | 7.888889e-01 |  |
| 333 | 59 | T | T | T | 87.74 | 2.303935e-12 | \* |
| 334 | 60 | C | C | A | 19.08 | 4.126545e-03 | \* |
| 334 | 60 | C | C | C | 77.86 | 6.076321e-05 | \* |
| 334 | 60 | C | C | G | 3.05 | 3.542570e-01 |  |
| 334 | 60 | C | C | T | 0.00 | 7.741935e-01 |  |
| 335 | 61 | G | G | A | 29.17 | 2.381820e-04 | \* |
| 335 | 61 | G | G | C | 0.00 | 8.139535e-01 |  |
| 335 | 61 | G | G | G | 59.17 | 4.529592e-06 | \* |
| 335 | 61 | G | G | T | 11.67 | 2.825159e-02 |  |
| 336 | 62 | A | A | A | 89.83 | 6.948886e-12 | \* |
| 336 | 62 | A | A | C | 6.21 | 2.664865e-01 |  |
| 336 | 62 | A | A | G | 0.00 | 7.888889e-01 |  |
| 336 | 62 | A | A | T | 3.95 | 2.724797e-01 |  |
| 337 | 63 | C | C | A | 0.63 | 6.233596e-01 |  |
| 337 | 63 | C | C | C | 96.84 | 7.347587e-06 | \* |
| 337 | 63 | C | C | G | 0.00 | 7.888889e-01 |  |
| 337 | 63 | C | C | T | 2.53 | 4.070191e-01 |  |
| 338 | 64 | A | A | A | 74.00 | 6.521198e-10 | \* |
| 338 | 64 | A | A | C | 15.00 | 8.495514e-02 |  |
| 338 | 64 | A | A | G | 11.00 | 6.496271e-02 |  |
| 338 | 64 | A | A | T | 0.00 | 7.741935e-01 |  |
| 339 | 65 | G | G | A | 0.00 | 7.064220e-01 |  |
| 339 | 65 | G | G | C | 25.00 | 2.530192e-02 |  |
| 339 | 65 | G | G | G | 75.00 | 2.049087e-07 | \* |
| 339 | 65 | G | G | T | 0.00 | 7.741935e-01 |  |
| 340 | 66 | C | C | A | 0.00 | 7.064220e-01 |  |
| 340 | 66 | C | C | C | 87.76 | 2.016002e-05 | \* |
| 340 | 66 | C | C | G | 8.84 | 1.016473e-01 |  |
| 340 | 66 | C | C | T | 3.40 | 3.189093e-01 |  |
| 341 | 67 | T | T | A | 1.28 | 5.355600e-01 |  |
| 341 | 67 | T | T | C | 14.74 | 8.770936e-02 |  |
| 341 | 67 | T | T | G | 0.00 | 7.888889e-01 |  |
| 341 | 67 | T | T | T | 83.97 | 7.331802e-12 | \* |
| 342 | 68 | C | C | A | 0.00 | 7.064220e-01 |  |
| 342 | 68 | C | C | C | 86.52 | 2.311974e-05 | \* |
| 342 | 68 | C | C | G | 0.71 | 6.252134e-01 |  |
| 342 | 68 | C | C | T | 12.77 | 2.033728e-02 |  |
| 343 | 69 | C | C | A | 0.00 | 7.064220e-01 |  |
| 343 | 69 | C | C | C | 73.21 | 1.022041e-04 | \* |
| 343 | 69 | C | C | G | 0.00 | 7.888889e-01 |  |
| 343 | 69 | C | C | T | 26.79 | 2.942164e-04 | \* |
| 344 | 70 | C | C | A | 22.55 | 1.552509e-03 | \* |
| 344 | 70 | C | C | C | 56.86 | 6.432626e-04 | \* |
| 344 | 70 | C | C | G | 0.98 | 5.817618e-01 |  |
| 344 | 70 | C | C | T | 19.61 | 2.593136e-03 | \* |
| 345 | 71 | G | G | A | 8.03 | 8.994512e-02 |  |
| 345 | 71 | G | G | C | 30.66 | 1.299365e-02 |  |
| 345 | 71 | G | G | G | 59.85 | 3.959094e-06 | \* |
| 345 | 71 | G | G | T | 1.46 | 5.450742e-01 |  |
| 346 | 72 | C | C | A | 3.15 | 3.340131e-01 |  |
| 346 | 72 | C | C | C | 80.31 | 4.620451e-05 | \* |
| 346 | 72 | C | C | G | 14.96 | 2.886939e-02 |  |
| 346 | 72 | C | C | T | 1.57 | 5.286081e-01 |  |
| 347 | 73 | G | G | A | 5.19 | 1.947669e-01 |  |
| 347 | 73 | G | G | C | 11.85 | 1.262595e-01 |  |
| 347 | 73 | G | G | G | 80.00 | 7.723016e-08 | \* |
| 347 | 73 | G | G | T | 2.96 | 3.608485e-01 |  |
| 348 | 74 | A | A | A | 74.12 | 6.304903e-10 | \* |
| 348 | 74 | A | A | C | 8.82 | 1.870790e-01 |  |
| 348 | 74 | A | A | G | 0.59 | 6.464367e-01 |  |
| 348 | 74 | A | A | T | 16.47 | 6.683895e-03 | \* |
| 349 | 75 | C | C | A | 0.00 | 7.064220e-01 |  |
| 349 | 75 | C | C | C | 77.38 | 6.412365e-05 | \* |
| 349 | 75 | C | C | G | 22.62 | 6.163210e-03 | \* |
| 349 | 75 | C | C | T | 0.00 | 7.741935e-01 |  |

## For use in R

If you want to work with the results in R, here is output that you can copy and paste in your terminal to get:

The base information:

```
structure(list(focal.base = c("A", "C", "G", "T"), avg.percsignal = c(86.1037130346608, 
87.8407917804165, 84.4184283390905, 87.7120896944914), avg.areasignal = c(177.470588235294, 
185.107692307692, 190.736842105263, 203.733333333333), crit.perc.area = c(15.9344365700385, 
32.9006984230845, 20.2065259723014, 15.1318736451794), mu = c(4.01204732049331, 
5.99926727700967, 4.13325437517703, 3.71845764658417), fillibens = c(0.969096461967766, 
0.96585139880141, 0.938442779301347, 0.96504634716245)), .Names = c("focal.base", 
"avg.percsignal", "avg.areasignal", "crit.perc.area", "mu", "fillibens"
), row.names = c(NA, -4L), class = "data.frame")
```

the data.frame that contains information on the guide region:

```
structure(list(A.area = c(153, 216, 5, 425, 5, 5, 6, 4, 3, 0, 
2, 5, 323, 3, 8, 19, 2, 0, 53, 0, 0, 14, 5, 13, 29, 118, 1, 32, 
140, 0, 177, 0, 29, 0, 18, 16, 133, 8, 1, 0, 37, 77, 232, 0, 
28, 27, 178, 0, 33, 24, 21, 57, 108, 0, 119, 149, 0, 14, 17, 
25, 35, 159, 1, 74, 0, 0, 2, 0, 0, 23, 11, 4, 7, 126, 0), C.area = c(0, 
3, 3, 2, 188, 1, 4, 2, 156, 222, 1, 2, 3, 219, 170, 1, 0, 2, 
28, 37, 42, 18, 15, 23, 112, 4, 132, 0, 20, 158, 26, 91, 25, 
19, 5, 108, 2, 135, 147, 125, 0, 21, 2, 138, 97, 38, 31, 115, 
1, 26, 120, 0, 0, 20, 20, 0, 18, 10, 2, 102, 0, 11, 153, 15, 
39, 129, 23, 122, 123, 58, 42, 102, 16, 15, 130), G.area = c(5, 
7, 184, 4, 7, 7, 464, 286, 6, 7, 4, 388, 5, 5, 2, 198, 4, 301, 
8, 5, 43, 207, 5, 166, 10, 52, 39, 40, 0, 3, 3, 8, 79, 134, 126, 
33, 4, 29, 9, 28, 2, 212, 0, 0, 31, 87, 13, 1, 73, 133, 0, 26, 
16, 111, 0, 0, 64, 34, 0, 4, 71, 0, 0, 11, 117, 13, 0, 1, 0, 
1, 82, 19, 108, 1, 38), T.area = c(0, 1, 0, 0, 8, 201, 2, 0, 
6, 6, 192, 2, 0, 5, 6, 2, 184, 2, 144, 173, 132, 3, 164, 5, 34, 
7, 7, 127, 30, 0, 6, 29, 0, 32, 0, 1, 6, 0, 3, 5, 136, 0, 22, 
26, 0, 2, 0, 3, 0, 1, 1, 0, 22, 0, 1, 5, 22, 120, 136, 0, 14, 
7, 4, 0, 0, 5, 131, 18, 45, 20, 2, 2, 4, 28, 0), Tot.area = c(158, 
227, 192, 431, 208, 214, 476, 292, 171, 235, 199, 397, 331, 232, 
186, 220, 190, 305, 233, 215, 217, 242, 189, 207, 185, 181, 179, 
199, 190, 161, 212, 128, 133, 185, 149, 158, 145, 172, 160, 158, 
175, 310, 256, 164, 156, 154, 222, 119, 107, 184, 142, 83, 146, 
131, 140, 154, 104, 178, 155, 131, 120, 177, 158, 100, 156, 147, 
156, 141, 168, 102, 137, 127, 135, 170, 168), A.perc = c(96.8354430379747, 
95.1541850220264, 2.60416666666667, 98.6078886310905, 2.40384615384615, 
2.33644859813084, 1.26050420168067, 1.36986301369863, 1.75438596491228, 
0, 1.00502512562814, 1.25944584382872, 97.583081570997, 1.29310344827586, 
4.3010752688172, 8.63636363636364, 1.05263157894737, 0, 22.7467811158798, 
0, 0, 5.78512396694215, 2.64550264550265, 6.28019323671498, 15.6756756756757, 
65.1933701657459, 0.558659217877095, 16.0804020100502, 73.6842105263158, 
0, 83.4905660377358, 0, 21.8045112781955, 0, 12.0805369127517, 
10.126582278481, 91.7241379310345, 4.65116279069767, 0.625, 0, 
21.1428571428571, 24.8387096774194, 90.625, 0, 17.9487179487179, 
17.5324675324675, 80.1801801801802, 0, 30.8411214953271, 13.0434782608696, 
14.7887323943662, 68.6746987951807, 73.972602739726, 0, 85, 96.7532467532468, 
0, 7.86516853932584, 10.9677419354839, 19.0839694656489, 29.1666666666667, 
89.8305084745763, 0.632911392405063, 74, 0, 0, 1.28205128205128, 
0, 0, 22.5490196078431, 8.02919708029197, 3.1496062992126, 5.18518518518519, 
74.1176470588235, 0), C.perc = c(0, 1.3215859030837, 1.5625, 
0.464037122969838, 90.3846153846154, 0.467289719626168, 0.840336134453782, 
0.684931506849315, 91.2280701754386, 94.468085106383, 0.50251256281407, 
0.503778337531486, 0.906344410876133, 94.3965517241379, 91.3978494623656, 
0.454545454545455, 0, 0.655737704918033, 12.0171673819742, 17.2093023255814, 
19.3548387096774, 7.43801652892562, 7.93650793650794, 11.1111111111111, 
60.5405405405405, 2.20994475138122, 73.7430167597765, 0, 10.5263157894737, 
98.1366459627329, 12.2641509433962, 71.09375, 18.796992481203, 
10.2702702702703, 3.35570469798658, 68.3544303797468, 1.37931034482759, 
78.4883720930233, 91.875, 79.1139240506329, 0, 6.7741935483871, 
0.78125, 84.1463414634146, 62.1794871794872, 24.6753246753247, 
13.963963963964, 96.6386554621849, 0.934579439252336, 14.1304347826087, 
84.5070422535211, 0, 0, 15.2671755725191, 14.2857142857143, 0, 
17.3076923076923, 5.61797752808989, 1.29032258064516, 77.8625954198473, 
0, 6.21468926553672, 96.8354430379747, 15, 25, 87.7551020408163, 
14.7435897435897, 86.5248226950355, 73.2142857142857, 56.8627450980392, 
30.6569343065693, 80.3149606299213, 11.8518518518519, 8.82352941176471, 
77.3809523809524), G.perc = c(3.16455696202532, 3.08370044052863, 
95.8333333333333, 0.928074245939675, 3.36538461538462, 3.27102803738318, 
97.4789915966387, 97.9452054794521, 3.50877192982456, 2.97872340425532, 
2.01005025125628, 97.7329974811083, 1.51057401812689, 2.1551724137931, 
1.0752688172043, 90, 2.10526315789474, 98.6885245901639, 3.43347639484979, 
2.32558139534884, 19.815668202765, 85.5371900826446, 2.64550264550265, 
80.1932367149758, 5.40540540540541, 28.7292817679558, 21.7877094972067, 
20.1005025125628, 0, 1.86335403726708, 1.41509433962264, 6.25, 
59.3984962406015, 72.4324324324324, 84.5637583892617, 20.8860759493671, 
2.75862068965517, 16.8604651162791, 5.625, 17.7215189873418, 
1.14285714285714, 68.3870967741936, 0, 0, 19.8717948717949, 56.4935064935065, 
5.85585585585586, 0.840336134453782, 68.2242990654206, 72.2826086956522, 
0, 31.3253012048193, 10.958904109589, 84.7328244274809, 0, 0, 
61.5384615384615, 19.1011235955056, 0, 3.05343511450382, 59.1666666666667, 
0, 0, 11, 75, 8.84353741496599, 0, 0.709219858156028, 0, 0.980392156862745, 
59.8540145985401, 14.9606299212598, 80, 0.588235294117647, 22.6190476190476
), T.perc = c(0, 0.440528634361233, 0, 0, 3.84615384615385, 93.9252336448598, 
0.420168067226891, 0, 3.50877192982456, 2.5531914893617, 96.4824120603015, 
0.503778337531486, 0, 2.1551724137931, 3.2258064516129, 0.909090909090909, 
96.8421052631579, 0.655737704918033, 61.8025751072961, 80.4651162790698, 
60.8294930875576, 1.2396694214876, 86.7724867724868, 2.41545893719807, 
18.3783783783784, 3.86740331491713, 3.91061452513966, 63.8190954773869, 
15.7894736842105, 0, 2.83018867924528, 22.65625, 0, 17.2972972972973, 
0, 0.632911392405063, 4.13793103448276, 0, 1.875, 3.16455696202532, 
77.7142857142857, 0, 8.59375, 15.8536585365854, 0, 1.2987012987013, 
0, 2.52100840336134, 0, 0.543478260869565, 0.704225352112676, 
0, 15.0684931506849, 0, 0.714285714285714, 3.24675324675325, 
21.1538461538462, 67.4157303370787, 87.741935483871, 0, 11.6666666666667, 
3.95480225988701, 2.53164556962025, 0, 0, 3.40136054421769, 83.974358974359, 
12.7659574468085, 26.7857142857143, 19.6078431372549, 1.45985401459854, 
1.5748031496063, 2.96296296296296, 16.4705882352941, 0), base.call = c("A", 
"A", "G", "A", "C", "T", "G", "G", "C", "C", "T", "G", "A", "C", 
"C", "G", "T", "G", "T", "T", "T", "G", "T", "G", "C", "A", "C", 
"T", "A", "C", "A", "C", "G", "G", "G", "C", "A", "C", "C", "C", 
"T", "G", "A", "C", "C", "G", "A", "C", "G", "G", "C", "A", "A", 
"G", "A", "A", "G", "T", "T", "C", "G", "A", "C", "A", "G", "C", 
"T", "C", "C", "C", "G", "C", "G", "A", "C"), index = 275:349, 
    guide.seq = c("A", "A", "G", "A", "C", "T", "G", "G", "C", 
    "C", "A", "G", "A", "C", "C", "G", "T", "G", "T", "T", "T", 
    "G", "T", "G", "C", "A", "C", "T", "A", "C", "A", "C", "G", 
    "G", "G", "C", "A", "C", "C", "C", "T", "G", "A", "C", "C", 
    "G", "A", "C", "G", "G", "C", "A", "A", "G", "A", "A", "G", 
    "T", "T", "C", "G", "A", "C", "A", "G", "C", "T", "C", "C", 
    "C", "G", "C", "G", "A", "C"), T.pval = c(0.774193548385466, 
    0.706230260398969, 0.774193548385466, 0.774193548385466, 
    0.28105748699513, 3.44502204541186e-13, 0.709613444329055, 
    0.774193548385466, 0.309352129048712, 0.404591003610595, 
    1.56985535681997e-13, 0.695712320645858, 0.774193548385466, 
    0.451600442790979, 0.335126943543216, 0.62927377874003, 1.40554234917545e-13, 
    0.670518952964589, 6.62008270424508e-09, 2.1545987216598e-11, 
    8.92152574039073e-09, 0.577715369929646, 3.10351744303716e-12, 
    0.420339882524087, 0.00375964398984185, 0.279359937180012, 
    0.275937890963057, 3.56695417647046e-09, 0.00820528674895005, 
    0.774193548385466, 0.374523794473407, 0.00103047098875564, 
    0.774193548385466, 0.00520976820498709, 0.774193548385466, 
    0.674288879954452, 0.258582292526608, 0.774193548385466, 
    0.487499766116262, 0.34096391806629, 5.01492181115282e-11, 
    0.774193548385466, 0.070437193327832, 0.0080483052420437, 
    0.774193548385466, 0.568824719347709, 0.774193548385466, 
    0.408222667468552, 0.774193548385466, 0.689113418122269, 
    0.662535089564596, 0.774193548385466, 0.0101923711897012, 
    0.774193548385466, 0.660883115781565, 0.333152197568555, 
    0.00162440950069109, 1.1834336843819e-09, 2.30393482070212e-12, 
    0.774193548385466, 0.0282515875743758, 0.272479690905724, 
    0.407019090810086, 0.774193548385466, 0.774193548385466, 
    0.31890926154905, 7.33180183232207e-12, 0.0203372844603978, 
    0.000294216443878281, 0.0025931356789608, 0.545074239812037, 
    0.528608059994839, 0.360848513013853, 0.00668389514393064, 
    0.774193548385466), C.pval = c(0.813953488372092, 0.570805412838823, 
    0.545686318456415, 0.685794207297123, 1.5046015399145e-05, 
    0.685232353144695, 0.628818421219437, 0.650669959112682, 
    1.36989015655509e-05, 9.55667717272846e-06, 0.679246322301767, 
    0.679034444391643, 0.620084037463236, 9.63291578437442e-06, 
    1.34427271731852e-05, 0.687442967023787, 0.813953488372092, 
    0.655005862418033, 0.123626063135152, 0.064677411421864, 
    0.0497895000384185, 0.225241852256427, 0.210579561775202, 
    0.138825906584476, 0.00042459871888112, 0.487020315967252, 
    9.63288887867453e-05, 0.813953488372092, 0.149705220574128, 
    6.35960165906724e-06, 0.119801186990512, 0.000129614994486049, 
    0.0532795218879309, 0.154758257453925, 0.404696241967863, 
    0.000176245872619996, 0.564588288590275, 5.66597356179699e-05, 
    1.27482032499238e-05, 5.28356937741226e-05, 0.813953488372092, 
    0.24662038931624, 0.63689956133289, 3.01351096039237e-05, 
    0.000352964053053073, 0.0262967757471388, 0.0966796982853912, 
    7.50985361119838e-06, 0.616435776615966, 0.0946857609906928, 
    2.89476102086628e-05, 0.813953488372092, 0.813953488372092, 
    0.0821821952891517, 0.0928651626654764, 0.813953488372092, 
    0.0639020022013626, 0.289793737303325, 0.574229035298083, 
    6.07632057130214e-05, 0.813953488372092, 0.266486486228207, 
    7.34758700193794e-06, 0.0849551357422345, 0.0253019196141684, 
    2.0160020311577e-05, 0.0877093623638405, 2.31197411488804e-05, 
    0.000102204099235559, 0.000643262627004981, 0.0129936458734983, 
    4.62045052120885e-05, 0.126259491687631, 0.187078985085558, 
    6.41236521250477e-05), G.pval = c(0.345441032741641, 0.351830706736953, 
    3.52956486260325e-09, 0.589757685893067, 0.33013179661541, 
    0.337227072346897, 2.56199828019277e-09, 2.33972108354408e-09, 
    0.319668585295249, 0.360328585606297, 0.451189862482803, 
    2.43839681779434e-09, 0.508979043071102, 0.435965128916978, 
    0.567679011961993, 1.0993412513649e-08, 0.44112894258373, 
    2.02453842579331e-09, 0.325115883523414, 0.418878224749307, 
    0.010817648332841, 2.623269423907e-08, 0.388910524652304, 
    7.43730557140054e-08, 0.210735250430276, 0.0018216044940379, 
    0.00728028310489592, 0.0102154261830215, 0.788888888879626, 
    0.467254571152257, 0.521107587098228, 0.175758171504551, 
    4.32851950848967e-06, 3.38303449809452e-07, 3.17144650541223e-08, 
    0.00872391067989775, 0.378922936388718, 0.0196358682586031, 
    0.200983427617314, 0.0164984596132426, 0.557955007881575, 
    7.4572466857159e-07, 0.788888888879626, 0.788888888879626, 
    0.0106962110159904, 7.64765557936808e-06, 0.191246469545383, 
    0.6035630706081, 7.69836328906592e-07, 3.48349669221371e-07, 
    0.788888888879626, 0.00108782400121088, 0.0655161601305182, 
    3.06861820398296e-08, 0.788888888879626, 0.788888888879626, 
    2.84685239615445e-06, 0.0124909530516145, 0.788888888879626, 
    0.354256960315723, 4.52959216712401e-06, 0.788888888879626, 
    0.788888888879626, 0.0649627066033008, 2.0490865626499e-07, 
    0.101647271761052, 0.788888888879626, 0.625213401998952, 
    0.788888888879626, 0.581761751024162, 3.95909366968095e-06, 
    0.0288693895830743, 7.72301557150357e-08, 0.646436684325583, 
    0.0061632098711385), A.pval = c(9.29922805426031e-13, 1.50701673362619e-12, 
    0.384639393257153, 5.58997292898766e-13, 0.404887681297842, 
    0.411906999565058, 0.538352299433671, 0.524283935881381, 
    0.476955524396038, 0.706422018348624, 0.572177181223543, 
    0.538489705988689, 7.50288720041681e-13, 0.534131603672669, 
    0.246664530561979, 0.0761340656899695, 0.565778786969188, 
    0.706422018348624, 0.00146812038573907, 0.706422018348624, 
    0.706422018348624, 0.165707662403761, 0.380574351583384, 
    0.144929203376062, 0.0107523904391114, 8.13584655201538e-09, 
    0.633729478778811, 0.00959892146461072, 7.13916481487331e-10, 
    0.706422018348624, 4.28673763153142e-11, 0.706422018348624, 
    0.00191590743667291, 0.706422018348624, 0.0293520269823507, 
    0.0504750517062847, 4.03488353839521e-12, 0.224705274436972, 
    0.624463465051938, 0.706422018348624, 0.00230939346375159, 
    0.000812399927590524, 5.53179724249730e-12, 0.706422018348624, 
    0.00567982886981555, 0.00638496963741952, 1.10813247466979e-10, 
    0.706422018348624, 0.000148045562890053, 0.0224464569208476, 
    0.013784445302061, 3.00080038595496e-09, 6.57262355652222e-10, 
    0.706422018348624, 2.77985412466819e-11, 9.52127265918534e-13, 
    0.706422018348624, 0.0940803204023842, 0.0399845797665201, 
    0.00412654509476973, 0.000238182046000568, 6.94888591112885e-12, 
    0.623359556077091, 6.52119802602158e-10, 0.706422018348624, 
    0.706422018348624, 0.535560012213963, 0.706422018348624, 
    0.706422018348624, 0.00155250896242565, 0.0899451178223329, 
    0.334013126988147, 0.194766882569341, 6.30490326614108e-10, 
    0.706422018348624), guide.position = 1:75), .Names = c("A.area", 
"C.area", "G.area", "T.area", "Tot.area", "A.perc", "C.perc", 
"G.perc", "T.perc", "base.call", "index", "guide.seq", "T.pval", 
"C.pval", "G.pval", "A.pval", "guide.position"), row.names = 275:349, class = "data.frame")
```

*Report generated using EditR v1.0.8*
