# Supplementary material for: Mechanistic and genetic basis of single-strand templated repair at Cas12a-induced DNA breaks in Chlamydomonas reinhardtii
Source: Nat Commun. 2021 Nov 19;12:6751. doi: 10.1038/s41467-021-27004-1 (PMC8604939; doi:10.1038/s41467-021-27004-1)
Supplement: Supplementary file 22 — Source Data [file 41467_2021_27004_MOESM22_ESM.zip › Source Data/EditR analysis/EditR outputs/Sense/rep1_ssODN_sense_0.html]

EditR v1.0.8 report


# EditR v1.0.8 report

- Data QA
  - Filtering data
  - Percent noise peak area
  - Base information
- Predicted editing
  - Editing bar plot
  - Editing table plot
  - Table of editing results
- For use in R

## Data QA

### Filtering data

What the data looked like prefiltering:

and the post filtering signal / noise plot:

### Percent noise peak area

### Base information

Here’s information about the signal of each base, the critical percent value where any higher value would be called as significant, and Filliben’s correlation for how well the noise was modelled by the zero adjusted gamma distribution.

| Base | Average percent signal | Average peak area | Critical percent value | model mu | Fillibens correlation |
| --- | --- | --- | --- | --- | --- |
| A | 92.44455 | 511.2059 | 6.449356 | 2.048445 | 0.9826421 |
| C | 91.15190 | 523.7015 | 5.450263 | 1.742241 | 0.9802571 |
| G | 94.13127 | 536.2857 | 9.536736 | 2.487129 | 0.9907234 |
| T | 94.78110 | 580.8409 | 13.271935 | 3.551781 | 0.9905267 |

## Predicted editing

### Editing bar plot

### Editing table plot

### Table of editing results


Here’s the entire guide region

| Sanger position | Guide position | Guide sequence | Sanger base call | Focal base | Focal base peak area | p value |  |
| --- | --- | --- | --- | --- | --- | --- | --- |
| 276 | 1 | A | A | A | 94.77 | 0.000000e+00 | \* |
| 276 | 1 | A | A | C | 1.55 | 4.434117e-01 |  |
| 276 | 1 | A | A | G | 1.94 | 4.598236e-01 |  |
| 276 | 1 | A | A | T | 1.74 | 6.602230e-01 |  |
| 277 | 2 | A | A | A | 94.35 | 0.000000e+00 | \* |
| 277 | 2 | A | A | C | 1.16 | 5.856760e-01 |  |
| 277 | 2 | A | A | G | 2.66 | 3.310172e-01 |  |
| 277 | 2 | A | A | T | 1.83 | 6.447188e-01 |  |
| 278 | 3 | G | G | A | 2.39 | 2.534793e-01 |  |
| 278 | 3 | G | G | C | 1.39 | 4.991704e-01 |  |
| 278 | 3 | G | G | G | 95.03 | 0.000000e+00 | \* |
| 278 | 3 | G | G | T | 1.19 | 7.664933e-01 |  |
| 279 | 4 | A | A | A | 95.31 | 0.000000e+00 | \* |
| 279 | 4 | A | A | C | 0.83 | 7.162448e-01 |  |
| 279 | 4 | A | A | G | 1.88 | 4.724291e-01 |  |
| 279 | 4 | A | A | T | 1.98 | 6.164766e-01 |  |
| 280 | 5 | C | C | A | 2.14 | 2.996856e-01 |  |
| 280 | 5 | C | C | C | 92.69 | 0.000000e+00 | \* |
| 280 | 5 | C | C | G | 0.00 | 9.230769e-01 |  |
| 280 | 5 | C | C | T | 5.17 | 2.146365e-01 |  |
| 281 | 6 | T | T | A | 2.36 | 2.580289e-01 |  |
| 281 | 6 | T | T | C | 1.18 | 5.789923e-01 |  |
| 281 | 6 | T | T | G | 3.39 | 2.334412e-01 |  |
| 281 | 6 | T | T | T | 93.07 | 0.000000e+00 | \* |
| 282 | 7 | G | G | A | 1.06 | 5.691826e-01 |  |
| 282 | 7 | G | G | C | 1.25 | 5.524474e-01 |  |
| 282 | 7 | G | G | G | 96.73 | 0.000000e+00 | \* |
| 282 | 7 | G | G | T | 0.96 | 8.117040e-01 |  |
| 283 | 8 | G | G | A | 1.70 | 3.977980e-01 |  |
| 283 | 8 | G | G | C | 0.71 | 7.655869e-01 |  |
| 283 | 8 | G | G | G | 96.89 | 0.000000e+00 | \* |
| 283 | 8 | G | G | T | 0.71 | 8.596618e-01 |  |
| 284 | 9 | C | C | A | 0.50 | 7.170988e-01 |  |
| 284 | 9 | C | C | C | 91.54 | 0.000000e+00 | \* |
| 284 | 9 | C | C | G | 1.99 | 4.492834e-01 |  |
| 284 | 9 | C | C | T | 5.97 | 1.610505e-01 |  |
| 285 | 10 | C | C | A | 1.80 | 3.722047e-01 |  |
| 285 | 10 | C | C | C | 91.13 | 0.000000e+00 | \* |
| 285 | 10 | C | C | G | 2.26 | 3.985488e-01 |  |
| 285 | 10 | C | C | T | 4.81 | 2.434965e-01 |  |
| 286 | 11 | A | A | A | 92.25 | 0.000000e+00 | \* |
| 286 | 11 | A | A | C | 1.74 | 3.809667e-01 |  |
| 286 | 11 | A | A | G | 5.04 | 1.033825e-01 |  |
| 286 | 11 | A | A | T | 0.97 | 8.100853e-01 |  |
| 287 | 12 | G | G | A | 3.62 | 1.019899e-01 |  |
| 287 | 12 | G | G | C | 1.45 | 4.784777e-01 |  |
| 287 | 12 | G | G | G | 94.20 | 0.000000e+00 | \* |
| 287 | 12 | G | G | T | 0.72 | 8.564486e-01 |  |
| 288 | 13 | A | A | A | 95.41 | 0.000000e+00 | \* |
| 288 | 13 | A | A | C | 1.25 | 5.509933e-01 |  |
| 288 | 13 | A | A | G | 1.04 | 6.705045e-01 |  |
| 288 | 13 | A | A | T | 2.30 | 5.609647e-01 |  |
| 289 | 14 | C | C | A | 2.06 | 3.157482e-01 |  |
| 289 | 14 | C | C | C | 90.65 | 0.000000e+00 | \* |
| 289 | 14 | C | C | G | 1.58 | 5.365222e-01 |  |
| 289 | 14 | C | C | T | 5.71 | 1.772157e-01 |  |
| 290 | 15 | C | C | A | 1.98 | 3.330902e-01 |  |
| 290 | 15 | C | C | C | 89.75 | 0.000000e+00 | \* |
| 290 | 15 | C | C | G | 2.88 | 2.985239e-01 |  |
| 290 | 15 | C | C | T | 5.40 | 1.980178e-01 |  |
| 291 | 16 | G | G | A | 1.94 | 3.414615e-01 |  |
| 291 | 16 | G | G | C | 1.41 | 4.921907e-01 |  |
| 291 | 16 | G | G | G | 94.53 | 0.000000e+00 | \* |
| 291 | 16 | G | G | T | 2.12 | 5.922746e-01 |  |
| 292 | 17 | T | T | A | 3.62 | 1.022485e-01 |  |
| 292 | 17 | T | T | C | 0.90 | 6.881117e-01 |  |
| 292 | 17 | T | T | G | 3.85 | 1.871841e-01 |  |
| 292 | 17 | T | T | T | 91.63 | 1.110223e-16 | \* |
| 293 | 18 | G | G | A | 0.00 | 7.818182e-01 |  |
| 293 | 18 | G | G | C | 0.47 | 8.474599e-01 |  |
| 293 | 18 | G | G | G | 98.34 | 0.000000e+00 | \* |
| 293 | 18 | G | G | T | 1.18 | 7.680587e-01 |  |
| 294 | 19 | T | T | A | 0.93 | 6.044964e-01 |  |
| 294 | 19 | T | T | C | 0.00 | 9.285714e-01 |  |
| 294 | 19 | T | T | G | 2.23 | 4.023784e-01 |  |
| 294 | 19 | T | T | T | 96.83 | 0.000000e+00 | \* |
| 295 | 20 | T | T | A | 0.54 | 7.083571e-01 |  |
| 295 | 20 | T | T | C | 0.00 | 9.285714e-01 |  |
| 295 | 20 | T | T | G | 1.43 | 5.732867e-01 |  |
| 295 | 20 | T | T | T | 98.04 | 0.000000e+00 | \* |
| 296 | 21 | T | T | A | 0.00 | 7.818182e-01 |  |
| 296 | 21 | T | T | C | 0.85 | 7.092808e-01 |  |
| 296 | 21 | T | T | G | 3.41 | 2.317758e-01 |  |
| 296 | 21 | T | T | T | 95.74 | 0.000000e+00 | \* |
| 297 | 22 | G | G | A | 1.07 | 5.651610e-01 |  |
| 297 | 22 | G | G | C | 0.80 | 7.284563e-01 |  |
| 297 | 22 | G | G | G | 96.92 | 0.000000e+00 | \* |
| 297 | 22 | G | G | T | 1.20 | 7.641525e-01 |  |
| 298 | 23 | T | T | A | 0.00 | 7.818182e-01 |  |
| 298 | 23 | T | T | C | 0.00 | 9.285714e-01 |  |
| 298 | 23 | T | T | G | 4.05 | 1.689960e-01 |  |
| 298 | 23 | T | T | T | 95.95 | 0.000000e+00 | \* |
| 299 | 24 | G | G | A | 1.13 | 5.483062e-01 |  |
| 299 | 24 | G | G | C | 1.45 | 4.768182e-01 |  |
| 299 | 24 | G | G | G | 96.28 | 0.000000e+00 | \* |
| 299 | 24 | G | G | T | 1.13 | 7.786048e-01 |  |
| 300 | 25 | C | C | A | 1.15 | 5.418551e-01 |  |
| 300 | 25 | C | C | C | 90.00 | 0.000000e+00 | \* |
| 300 | 25 | C | C | G | 2.12 | 4.247136e-01 |  |
| 300 | 25 | C | C | T | 6.73 | 1.220065e-01 |  |
| 301 | 26 | A | A | A | 92.20 | 0.000000e+00 | \* |
| 301 | 26 | A | A | C | 3.07 | 1.162277e-01 |  |
| 301 | 26 | A | A | G | 2.13 | 4.223679e-01 |  |
| 301 | 26 | A | A | T | 2.60 | 5.106658e-01 |  |
| 302 | 27 | C | C | A | 0.83 | 6.327784e-01 |  |
| 302 | 27 | C | C | C | 92.21 | 0.000000e+00 | \* |
| 302 | 27 | C | C | G | 1.16 | 6.400296e-01 |  |
| 302 | 27 | C | C | T | 5.80 | 1.709999e-01 |  |
| 303 | 28 | T | T | A | 2.57 | 2.231358e-01 |  |
| 303 | 28 | T | T | C | 1.77 | 3.744511e-01 |  |
| 303 | 28 | T | T | G | 0.16 | 8.991581e-01 |  |
| 303 | 28 | T | T | T | 95.51 | 0.000000e+00 | \* |
| 304 | 29 | A | A | A | 96.38 | 0.000000e+00 | \* |
| 304 | 29 | A | A | C | 2.29 | 2.410479e-01 |  |
| 304 | 29 | A | A | G | 0.00 | 9.230769e-01 |  |
| 304 | 29 | A | A | T | 1.33 | 7.390585e-01 |  |
| 305 | 30 | C | C | A | 1.74 | 3.869569e-01 |  |
| 305 | 30 | C | C | C | 91.11 | 0.000000e+00 | \* |
| 305 | 30 | C | C | G | 1.39 | 5.817149e-01 |  |
| 305 | 30 | C | C | T | 5.75 | 1.744361e-01 |  |
| 306 | 31 | A | A | A | 92.33 | 0.000000e+00 | \* |
| 306 | 31 | A | A | C | 3.02 | 1.219515e-01 |  |
| 306 | 31 | A | A | G | 2.79 | 3.110232e-01 |  |
| 306 | 31 | A | A | T | 1.86 | 6.385686e-01 |  |
| 307 | 32 | C | C | A | 1.18 | 5.342250e-01 |  |
| 307 | 32 | C | C | C | 91.34 | 0.000000e+00 | \* |
| 307 | 32 | C | C | G | 1.57 | 5.388206e-01 |  |
| 307 | 32 | C | C | T | 5.91 | 1.648621e-01 |  |
| 308 | 33 | G | G | A | 1.71 | 3.953632e-01 |  |
| 308 | 33 | G | G | C | 1.46 | 4.734770e-01 |  |
| 308 | 33 | G | G | G | 96.83 | 0.000000e+00 | \* |
| 308 | 33 | G | G | T | 0.00 | 9.569892e-01 |  |
| 309 | 34 | G | G | A | 0.54 | 7.081335e-01 |  |
| 309 | 34 | G | G | C | 0.36 | 8.807318e-01 |  |
| 309 | 34 | G | G | G | 98.93 | 0.000000e+00 | \* |
| 309 | 34 | G | G | T | 0.18 | 9.430751e-01 |  |
| 310 | 35 | G | G | A | 1.46 | 4.585886e-01 |  |
| 310 | 35 | G | G | C | 2.08 | 2.874769e-01 |  |
| 310 | 35 | G | G | G | 95.83 | 0.000000e+00 | \* |
| 310 | 35 | G | G | T | 0.62 | 8.745826e-01 |  |
| 311 | 36 | C | C | A | 3.02 | 1.605631e-01 |  |
| 311 | 36 | C | C | C | 90.23 | 0.000000e+00 | \* |
| 311 | 36 | C | C | G | 1.40 | 5.813211e-01 |  |
| 311 | 36 | C | C | T | 5.35 | 2.013565e-01 |  |
| 312 | 37 | A | A | A | 93.29 | 0.000000e+00 | \* |
| 312 | 37 | A | A | C | 3.68 | 6.395812e-02 |  |
| 312 | 37 | A | A | G | 0.00 | 9.230769e-01 |  |
| 312 | 37 | A | A | T | 3.03 | 4.453225e-01 |  |
| 313 | 38 | C | C | A | 0.92 | 6.079257e-01 |  |
| 313 | 38 | C | C | C | 91.88 | 0.000000e+00 | \* |
| 313 | 38 | C | C | G | 0.77 | 7.451294e-01 |  |
| 313 | 38 | C | C | T | 6.43 | 1.361406e-01 |  |
| 314 | 39 | C | C | A | 0.85 | 6.265562e-01 |  |
| 314 | 39 | C | C | C | 91.99 | 0.000000e+00 | \* |
| 314 | 39 | C | C | G | 0.85 | 7.218009e-01 |  |
| 314 | 39 | C | C | T | 6.30 | 1.426876e-01 |  |
| 315 | 40 | C | C | A | 0.18 | 7.717975e-01 |  |
| 315 | 40 | C | C | C | 92.42 | 0.000000e+00 | \* |
| 315 | 40 | C | C | G | 0.18 | 8.950416e-01 |  |
| 315 | 40 | C | C | T | 7.22 | 1.018316e-01 |  |
| 316 | 41 | T | T | A | 0.00 | 7.818182e-01 |  |
| 316 | 41 | T | T | C | 1.41 | 4.930867e-01 |  |
| 316 | 41 | T | T | G | 3.35 | 2.388244e-01 |  |
| 316 | 41 | T | T | T | 95.25 | 0.000000e+00 | \* |
| 317 | 42 | G | G | A | 2.82 | 1.867491e-01 |  |
| 317 | 42 | G | G | C | 1.69 | 3.977301e-01 |  |
| 317 | 42 | G | G | G | 93.80 | 0.000000e+00 | \* |
| 317 | 42 | G | G | T | 1.69 | 6.704046e-01 |  |
| 318 | 43 | A | A | A | 70.59 | 0.000000e+00 | \* |
| 318 | 43 | A | A | C | 3.29 | 9.442420e-02 |  |
| 318 | 43 | A | A | G | 2.60 | 3.408318e-01 |  |
| 318 | 43 | A | A | T | 23.53 | 1.648552e-04 | \* |
| 319 | 44 | C | C | A | 0.94 | 6.025400e-01 |  |
| 319 | 44 | C | C | C | 90.81 | 0.000000e+00 | \* |
| 319 | 44 | C | C | G | 2.81 | 3.075927e-01 |  |
| 319 | 44 | C | C | T | 5.44 | 1.948439e-01 |  |
| 320 | 45 | C | C | A | 0.58 | 6.982966e-01 |  |
| 320 | 45 | C | C | C | 87.86 | 0.000000e+00 | \* |
| 320 | 45 | C | C | G | 5.59 | 7.827281e-02 |  |
| 320 | 45 | C | C | T | 5.97 | 1.608829e-01 |  |
| 321 | 46 | G | G | A | 7.21 | 5.184933e-03 | \* |
| 321 | 46 | G | G | C | 2.79 | 1.520598e-01 |  |
| 321 | 46 | G | G | G | 89.53 | 0.000000e+00 | \* |
| 321 | 46 | G | G | T | 0.47 | 9.021202e-01 |  |
| 322 | 47 | A | A | A | 94.77 | 0.000000e+00 | \* |
| 322 | 47 | A | A | C | 2.04 | 2.980998e-01 |  |
| 322 | 47 | A | A | G | 1.02 | 6.766871e-01 |  |
| 322 | 47 | A | A | T | 2.17 | 5.831264e-01 |  |
| 323 | 48 | C | C | A | 2.57 | 2.221859e-01 |  |
| 323 | 48 | C | C | C | 87.33 | 0.000000e+00 | \* |
| 323 | 48 | C | C | G | 3.56 | 2.147802e-01 |  |
| 323 | 48 | C | C | T | 6.53 | 1.311131e-01 |  |
| 324 | 49 | G | G | A | 4.94 | 3.554391e-02 |  |
| 324 | 49 | G | G | C | 3.20 | 1.030452e-01 |  |
| 324 | 49 | G | G | G | 91.86 | 0.000000e+00 | \* |
| 324 | 49 | G | G | T | 0.00 | 9.569892e-01 |  |
| 325 | 50 | G | G | A | 3.01 | 1.616426e-01 |  |
| 325 | 50 | G | G | C | 2.48 | 2.021440e-01 |  |
| 325 | 50 | G | G | G | 94.50 | 0.000000e+00 | \* |
| 325 | 50 | G | G | T | 0.00 | 9.569892e-01 |  |
| 326 | 51 | C | C | A | 3.49 | 1.131318e-01 |  |
| 326 | 51 | C | C | C | 88.95 | 0.000000e+00 | \* |
| 326 | 51 | C | C | G | 2.03 | 4.403645e-01 |  |
| 326 | 51 | C | C | T | 5.52 | 1.891840e-01 |  |
| 327 | 52 | A | A | A | 95.83 | 0.000000e+00 | \* |
| 327 | 52 | A | A | C | 1.39 | 5.001742e-01 |  |
| 327 | 52 | A | A | G | 1.04 | 6.710772e-01 |  |
| 327 | 52 | A | A | T | 1.74 | 6.617398e-01 |  |
| 328 | 53 | A | A | A | 91.05 | 0.000000e+00 | \* |
| 328 | 53 | A | A | C | 0.80 | 7.315881e-01 |  |
| 328 | 53 | A | A | G | 5.37 | 8.752910e-02 |  |
| 328 | 53 | A | A | T | 2.78 | 4.820363e-01 |  |
| 329 | 54 | G | G | A | 6.22 | 1.215526e-02 |  |
| 329 | 54 | G | G | C | 0.69 | 7.716277e-01 |  |
| 329 | 54 | G | G | G | 92.40 | 0.000000e+00 | \* |
| 329 | 54 | G | G | T | 0.69 | 8.625921e-01 |  |
| 330 | 55 | A | A | A | 95.64 | 0.000000e+00 | \* |
| 330 | 55 | A | A | C | 0.57 | 8.162950e-01 |  |
| 330 | 55 | A | A | G | 2.27 | 3.954556e-01 |  |
| 330 | 55 | A | A | T | 1.52 | 7.038094e-01 |  |
| 331 | 56 | A | A | A | 93.72 | 0.000000e+00 | \* |
| 331 | 56 | A | A | C | 0.63 | 7.951863e-01 |  |
| 331 | 56 | A | A | G | 2.51 | 3.545052e-01 |  |
| 331 | 56 | A | A | T | 3.14 | 4.300113e-01 |  |
| 332 | 57 | G | G | A | 3.09 | 1.528273e-01 |  |
| 332 | 57 | G | G | C | 2.25 | 2.493712e-01 |  |
| 332 | 57 | G | G | G | 89.61 | 0.000000e+00 | \* |
| 332 | 57 | G | G | T | 5.06 | 2.234182e-01 |  |
| 333 | 58 | T | T | A | 2.53 | 2.294302e-01 |  |
| 333 | 58 | T | T | C | 0.69 | 7.722263e-01 |  |
| 333 | 58 | T | T | G | 0.69 | 7.657916e-01 |  |
| 333 | 58 | T | T | T | 96.09 | 0.000000e+00 | \* |
| 334 | 59 | T | T | A | 1.60 | 4.224216e-01 |  |
| 334 | 59 | T | T | C | 4.57 | 2.568551e-02 |  |
| 334 | 59 | T | T | G | 0.23 | 8.846484e-01 |  |
| 334 | 59 | T | T | T | 93.61 | 0.000000e+00 | \* |
| 335 | 60 | C | C | A | 0.00 | 7.818182e-01 |  |
| 335 | 60 | C | C | C | 91.91 | 0.000000e+00 | \* |
| 335 | 60 | C | C | G | 4.49 | 1.358422e-01 |  |
| 335 | 60 | C | C | T | 3.60 | 3.697302e-01 |  |
| 336 | 61 | G | G | A | 7.86 | 2.935814e-03 | \* |
| 336 | 61 | G | G | C | 1.90 | 3.363423e-01 |  |
| 336 | 61 | G | G | G | 88.89 | 0.000000e+00 | \* |
| 336 | 61 | G | G | T | 1.36 | 7.348350e-01 |  |
| 337 | 62 | A | A | A | 93.56 | 0.000000e+00 | \* |
| 337 | 62 | A | A | C | 2.97 | 1.282888e-01 |  |
| 337 | 62 | A | A | G | 1.82 | 4.854645e-01 |  |
| 337 | 62 | A | A | T | 1.65 | 6.779796e-01 |  |
| 338 | 63 | C | C | A | 2.04 | 3.190052e-01 |  |
| 338 | 63 | C | C | C | 89.96 | 0.000000e+00 | \* |
| 338 | 63 | C | C | G | 1.49 | 5.593405e-01 |  |
| 338 | 63 | C | C | T | 6.51 | 1.325170e-01 |  |
| 339 | 64 | A | A | A | 86.24 | 0.000000e+00 | \* |
| 339 | 64 | A | A | C | 3.70 | 6.242929e-02 |  |
| 339 | 64 | A | A | G | 8.47 | 1.760165e-02 |  |
| 339 | 64 | A | A | T | 1.59 | 6.899617e-01 |  |
| 340 | 65 | G | G | A | 1.13 | 5.485626e-01 |  |
| 340 | 65 | G | G | C | 5.84 | 6.557515e-03 | \* |
| 340 | 65 | G | G | G | 92.09 | 0.000000e+00 | \* |
| 340 | 65 | G | G | T | 0.94 | 8.153689e-01 |  |
| 341 | 66 | C | C | A | 0.00 | 7.818182e-01 |  |
| 341 | 66 | C | C | C | 88.14 | 0.000000e+00 | \* |
| 341 | 66 | C | C | G | 1.40 | 5.813211e-01 |  |
| 341 | 66 | C | C | T | 10.47 | 2.980132e-02 |  |
| 342 | 67 | T | T | A | 0.87 | 6.203752e-01 |  |
| 342 | 67 | T | T | C | 4.72 | 2.183767e-02 |  |
| 342 | 67 | T | T | G | 0.87 | 7.157724e-01 |  |
| 342 | 67 | T | T | T | 93.53 | 0.000000e+00 | \* |
| 343 | 68 | C | C | A | 0.00 | 7.818182e-01 |  |
| 343 | 68 | C | C | C | 95.41 | 0.000000e+00 | \* |
| 343 | 68 | C | C | G | 0.22 | 8.868734e-01 |  |
| 343 | 68 | C | C | T | 4.37 | 2.843696e-01 |  |
| 344 | 69 | C | C | A | 0.17 | 7.733903e-01 |  |
| 344 | 69 | C | C | C | 92.41 | 0.000000e+00 | \* |
| 344 | 69 | C | C | G | 0.17 | 8.982442e-01 |  |
| 344 | 69 | C | C | T | 7.26 | 1.003129e-01 |  |
| 345 | 70 | C | C | A | 0.00 | 7.818182e-01 |  |
| 345 | 70 | C | C | C | 87.14 | 0.000000e+00 | \* |
| 345 | 70 | C | C | G | 7.52 | 2.881935e-02 |  |
| 345 | 70 | C | C | T | 5.34 | 2.020061e-01 |  |
| 346 | 71 | G | G | A | 5.82 | 1.704833e-02 |  |
| 346 | 71 | G | G | C | 5.57 | 8.786167e-03 | \* |
| 346 | 71 | G | G | G | 88.35 | 0.000000e+00 | \* |
| 346 | 71 | G | G | T | 0.25 | 9.339095e-01 |  |
| 347 | 72 | C | C | A | 4.54 | 4.951258e-02 |  |
| 347 | 72 | C | C | C | 82.94 | 0.000000e+00 | \* |
| 347 | 72 | C | C | G | 5.40 | 8.612885e-02 |  |
| 347 | 72 | C | C | T | 7.13 | 1.053936e-01 |  |
| 348 | 73 | G | G | A | 10.35 | 3.157876e-04 | \* |
| 348 | 73 | G | G | C | 3.28 | 9.482897e-02 |  |
| 348 | 73 | G | G | G | 85.61 | 0.000000e+00 | \* |
| 348 | 73 | G | G | T | 0.76 | 8.503322e-01 |  |
| 349 | 74 | A | A | A | 91.07 | 0.000000e+00 | \* |
| 349 | 74 | A | A | C | 4.46 | 2.858095e-02 |  |
| 349 | 74 | A | A | G | 1.61 | 5.314005e-01 |  |
| 349 | 74 | A | A | T | 2.86 | 4.708234e-01 |  |
| 350 | 75 | C | C | A | 1.24 | 5.175087e-01 |  |
| 350 | 75 | C | C | C | 88.12 | 0.000000e+00 | \* |
| 350 | 75 | C | C | G | 2.84 | 3.043311e-01 |  |
| 350 | 75 | C | C | T | 7.80 | 8.200927e-02 |  |

## For use in R

If you want to work with the results in R, here is output that you can copy and paste in your terminal to get:

The base information:

```
structure(list(focal.base = c("A", "C", "G", "T"), avg.percsignal = c(92.4445547513449, 
91.1518969593175, 94.1312746270077, 94.7811049849221), avg.areasignal = c(511.205882352941, 
523.701492537313, 536.285714285714, 580.840909090909), crit.perc.area = c(6.44935568891289, 
5.45026312803594, 9.53673568021565, 13.2719355351977), mu = c(2.04844496718755, 
1.74224049475812, 2.48712900795949, 3.55178118960684), fillibens = c(0.982642085783611, 
0.980257056024741, 0.990723369800469, 0.990526694736653)), .Names = c("focal.base", 
"avg.percsignal", "avg.areasignal", "crit.perc.area", "mu", "fillibens"
), row.names = c(NA, -4L), class = "data.frame")
```

the data.frame that contains information on the guide region:

```
structure(list(A.area = c(489, 568, 12, 914, 12, 16, 11, 12, 
2, 12, 476, 20, 914, 13, 11, 11, 16, 0, 5, 3, 0, 8, 0, 7, 6, 
390, 5, 16, 506, 10, 397, 6, 7, 3, 7, 13, 431, 6, 5, 1, 0, 20, 
408, 5, 3, 31, 743, 13, 17, 17, 12, 276, 458, 27, 505, 448, 11, 
11, 7, 0, 29, 567, 11, 326, 6, 0, 5, 0, 1, 0, 23, 21, 41, 510, 
7), C.area = c(8, 7, 7, 8, 520, 8, 13, 5, 368, 606, 9, 8, 12, 
572, 499, 8, 4, 4, 0, 0, 5, 6, 0, 9, 468, 13, 556, 11, 12, 523, 
13, 464, 6, 2, 10, 388, 17, 600, 540, 512, 8, 12, 19, 484, 456, 
12, 16, 441, 11, 14, 306, 4, 4, 3, 3, 3, 8, 3, 20, 409, 7, 18, 
484, 14, 31, 379, 27, 437, 560, 359, 22, 384, 13, 25, 497), G.area = c(10, 
16, 478, 18, 0, 23, 1007, 685, 8, 15, 26, 520, 10, 10, 16, 536, 
17, 830, 12, 8, 20, 724, 21, 596, 11, 9, 7, 1, 0, 8, 12, 8, 397, 
553, 460, 6, 0, 5, 5, 1, 19, 666, 15, 15, 29, 385, 8, 18, 316, 
533, 7, 3, 27, 401, 12, 12, 319, 3, 1, 20, 328, 11, 8, 32, 489, 
6, 5, 1, 1, 31, 349, 25, 339, 9, 16), T.area = c(9, 11, 6, 19, 
29, 631, 10, 5, 24, 32, 5, 4, 22, 36, 30, 12, 405, 10, 520, 549, 
562, 9, 497, 7, 35, 11, 35, 595, 7, 33, 8, 30, 0, 1, 3, 23, 14, 
42, 37, 40, 541, 12, 136, 29, 31, 2, 17, 33, 0, 0, 19, 5, 14, 
3, 8, 15, 18, 418, 410, 16, 5, 10, 35, 6, 5, 45, 535, 20, 44, 
22, 1, 33, 3, 16, 44), Tot.area = c(516, 602, 503, 959, 561, 
678, 1041, 707, 402, 665, 516, 552, 958, 631, 556, 567, 442, 
844, 537, 560, 587, 747, 518, 619, 520, 423, 603, 623, 525, 574, 
430, 508, 410, 559, 480, 430, 462, 653, 587, 554, 568, 710, 578, 
533, 519, 430, 784, 505, 344, 564, 344, 288, 503, 434, 528, 478, 
356, 435, 438, 445, 369, 606, 538, 378, 531, 430, 572, 458, 606, 
412, 395, 463, 396, 560, 564), A.perc = c(94.7674418604651, 94.3521594684385, 
2.38568588469185, 95.3076120959333, 2.13903743315508, 2.3598820058997, 
1.0566762728146, 1.6973125884017, 0.497512437810945, 1.80451127819549, 
92.2480620155039, 3.6231884057971, 95.4070981210856, 2.06022187004754, 
1.97841726618705, 1.94003527336861, 3.61990950226244, 0, 0.931098696461825, 
0.535714285714286, 0, 1.07095046854083, 0, 1.13085621970921, 
1.15384615384615, 92.1985815602837, 0.829187396351575, 2.56821829855538, 
96.3809523809524, 1.74216027874564, 92.3255813953488, 1.18110236220472, 
1.70731707317073, 0.536672629695885, 1.45833333333333, 3.02325581395349, 
93.2900432900433, 0.918836140888208, 0.851788756388416, 0.180505415162455, 
0, 2.8169014084507, 70.5882352941177, 0.938086303939963, 0.578034682080925, 
7.2093023255814, 94.7704081632653, 2.57425742574257, 4.94186046511628, 
3.01418439716312, 3.48837209302326, 95.8333333333333, 91.0536779324056, 
6.22119815668203, 95.6439393939394, 93.7238493723849, 3.08988764044944, 
2.52873563218391, 1.59817351598174, 0, 7.85907859078591, 93.5643564356436, 
2.04460966542751, 86.2433862433862, 1.12994350282486, 0, 0.874125874125874, 
0, 0.165016501650165, 0, 5.82278481012658, 4.53563714902808, 
10.3535353535354, 91.0714285714286, 1.24113475177305), C.perc = c(1.55038759689922, 
1.16279069767442, 1.39165009940358, 0.834202294056309, 92.6916221033868, 
1.17994100294985, 1.24879923150817, 0.707213578500707, 91.5422885572139, 
91.1278195488722, 1.74418604651163, 1.44927536231884, 1.25260960334029, 
90.6497622820919, 89.7482014388489, 1.41093474426808, 0.904977375565611, 
0.4739336492891, 0, 0, 0.851788756388416, 0.803212851405622, 
0, 1.45395799676898, 90, 3.07328605200946, 92.2056384742952, 
1.76565008025682, 2.28571428571429, 91.1149825783972, 3.02325581395349, 
91.3385826771653, 1.46341463414634, 0.35778175313059, 2.08333333333333, 
90.2325581395349, 3.67965367965368, 91.8836140888208, 91.9931856899489, 
92.4187725631769, 1.40845070422535, 1.69014084507042, 3.28719723183391, 
90.8067542213884, 87.8612716763006, 2.7906976744186, 2.04081632653061, 
87.3267326732673, 3.19767441860465, 2.4822695035461, 88.953488372093, 
1.38888888888889, 0.795228628230616, 0.691244239631336, 0.568181818181818, 
0.627615062761506, 2.24719101123596, 0.689655172413793, 4.5662100456621, 
91.9101123595506, 1.8970189701897, 2.97029702970297, 89.9628252788104, 
3.7037037037037, 5.83804143126177, 88.1395348837209, 4.72027972027972, 
95.4148471615721, 92.4092409240924, 87.1359223300971, 5.56962025316456, 
82.9373650107991, 3.28282828282828, 4.46428571428571, 88.1205673758865
), G.perc = c(1.93798449612403, 2.6578073089701, 95.0298210735587, 
1.87695516162669, 0, 3.39233038348083, 96.7339097022094, 96.8882602545969, 
1.99004975124378, 2.25563909774436, 5.03875968992248, 94.2028985507246, 
1.04384133611691, 1.58478605388273, 2.87769784172662, 94.5326278659612, 
3.84615384615385, 98.3412322274881, 2.23463687150838, 1.42857142857143, 
3.40715502555366, 96.9210174029451, 4.05405405405405, 96.2843295638126, 
2.11538461538462, 2.12765957446809, 1.16086235489221, 0.160513643659711, 
0, 1.39372822299652, 2.7906976744186, 1.5748031496063, 96.8292682926829, 
98.9266547406082, 95.8333333333333, 1.3953488372093, 0, 0.765696784073507, 
0.851788756388416, 0.180505415162455, 3.34507042253521, 93.8028169014084, 
2.59515570934256, 2.81425891181989, 5.58766859344894, 89.5348837209302, 
1.02040816326531, 3.56435643564356, 91.8604651162791, 94.5035460992908, 
2.03488372093023, 1.04166666666667, 5.36779324055666, 92.3963133640553, 
2.27272727272727, 2.51046025104602, 89.6067415730337, 0.689655172413793, 
0.228310502283105, 4.49438202247191, 88.8888888888889, 1.81518151815182, 
1.48698884758364, 8.46560846560847, 92.090395480226, 1.3953488372093, 
0.874125874125874, 0.218340611353712, 0.165016501650165, 7.52427184466019, 
88.3544303797468, 5.39956803455724, 85.6060606060606, 1.60714285714286, 
2.83687943262411), T.perc = c(1.74418604651163, 1.82724252491694, 
1.19284294234592, 1.98123044838373, 5.16934046345811, 93.0678466076696, 
0.960614793467819, 0.707213578500707, 5.97014925373134, 4.81203007518797, 
0.968992248062015, 0.72463768115942, 2.2964509394572, 5.70522979397781, 
5.39568345323741, 2.11640211640212, 91.6289592760181, 1.18483412322275, 
96.8342644320298, 98.0357142857143, 95.7410562180579, 1.20481927710843, 
95.945945945946, 1.13085621970921, 6.73076923076923, 2.60047281323877, 
5.80431177446103, 95.5056179775281, 1.33333333333333, 5.74912891986063, 
1.86046511627907, 5.90551181102362, 0, 0.178890876565295, 0.625, 
5.34883720930233, 3.03030303030303, 6.43185298621746, 6.30323679727428, 
7.22021660649819, 95.2464788732394, 1.69014084507042, 23.5294117647059, 
5.44090056285178, 5.97302504816956, 0.465116279069767, 2.16836734693878, 
6.53465346534653, 0, 0, 5.52325581395349, 1.73611111111111, 2.78330019880716, 
0.691244239631336, 1.51515151515152, 3.13807531380753, 5.0561797752809, 
96.0919540229885, 93.6073059360731, 3.59550561797753, 1.3550135501355, 
1.65016501650165, 6.50557620817844, 1.58730158730159, 0.941619585687382, 
10.4651162790698, 93.5314685314685, 4.36681222707424, 7.26072607260726, 
5.33980582524272, 0.253164556962025, 7.12742980561555, 0.757575757575758, 
2.85714285714286, 7.80141843971631), base.call = c("A", "A", 
"G", "A", "C", "T", "G", "G", "C", "C", "A", "G", "A", "C", "C", 
"G", "T", "G", "T", "T", "T", "G", "T", "G", "C", "A", "C", "T", 
"A", "C", "A", "C", "G", "G", "G", "C", "A", "C", "C", "C", "T", 
"G", "A", "C", "C", "G", "A", "C", "G", "G", "C", "A", "A", "G", 
"A", "A", "G", "T", "T", "C", "G", "A", "C", "A", "G", "C", "T", 
"C", "C", "C", "G", "C", "G", "A", "C"), index = 276:350, guide.seq = c("A", 
"A", "G", "A", "C", "T", "G", "G", "C", "C", "A", "G", "A", "C", 
"C", "G", "T", "G", "T", "T", "T", "G", "T", "G", "C", "A", "C", 
"T", "A", "C", "A", "C", "G", "G", "G", "C", "A", "C", "C", "C", 
"T", "G", "A", "C", "C", "G", "A", "C", "G", "G", "C", "A", "A", 
"G", "A", "A", "G", "T", "T", "C", "G", "A", "C", "A", "G", "C", 
"T", "C", "C", "C", "G", "C", "G", "A", "C"), T.pval = c(0.660223015677151, 
0.644718844995412, 0.766493346804471, 0.616476629252225, 0.214636492179559, 
0, 0.811703988462279, 0.859661760660842, 0.161050512675189, 0.243496511393013, 
0.810085265119127, 0.856448585300033, 0.56096474751665, 0.177215684978185, 
0.198017787021901, 0.592274569607758, 1.11022302462516e-16, 0.768058671436384, 
0, 0, 0, 0.764152506623048, 0, 0.77860479276474, 0.122006476634742, 
0.510665848833318, 0.170999851413257, 0, 0.739058488316771, 0.174436110667643, 
0.638568561240566, 0.164862074850199, 0.956989247311347, 0.943075071578722, 
0.874582576575661, 0.201356513332207, 0.445322488208059, 0.136140576368317, 
0.142687569236811, 0.101831591908926, 0, 0.670404610443559, 0.000164855181182477, 
0.1948438631184, 0.160882868052686, 0.902120219727226, 0.583126437942661, 
0.131113148426981, 0.956989247311347, 0.956989247311347, 0.189184022608071, 
0.661739756386705, 0.482036259289018, 0.86259208581923, 0.703809389188406, 
0.430011296678281, 0.223418217230178, 0, 0, 0.369730198704253, 
0.734835049373908, 0.677979599956108, 0.132517034962724, 0.689961661057653, 
0.81536885072606, 0.0298013231975125, 0, 0.284369566848289, 0.100312852751226, 
0.202006137436865, 0.933909543047176, 0.10539356581519, 0.850332201105505, 
0.470823390747944, 0.0820092683161444), C.pval = c(0.44341171766617, 
0.58567601381574, 0.49917038110591, 0.716244780220777, 0, 0.578992344498725, 
0.552447384779547, 0.765586855606359, 0, 0, 0.380966685022485, 
0.478477746925765, 0.550993260764476, 0, 0, 0.492190658286527, 
0.688111719150073, 0.847459876447253, 0.928571428571429, 0.928571428571429, 
0.709280796203575, 0.72845632538167, 0.928571428571429, 0.476818209974431, 
0, 0.116227716312608, 0, 0.374451127141344, 0.241047890474606, 
0, 0.121951506920226, 0, 0.473477047780724, 0.880731812885134, 
0.287476934475216, 0, 0.0639581150533534, 0, 0, 0, 0.493086662857101, 
0.397730089297796, 0.0944242008312353, 0, 0, 0.152059833622905, 
0.298099753106209, 0, 0.103045195927781, 0.202144004411572, 0, 
0.500174163071501, 0.731588129787376, 0.771627706923463, 0.816294956492832, 
0.795186283115671, 0.249371212085534, 0.772226258938815, 0.02568550929664, 
0, 0.336342304626557, 0.12828879764051, 0, 0.0624292920194474, 
0.00655751498085444, 0, 0.0218376671321272, 0, 0, 0, 0.00878616725778403, 
0, 0.0948289745432759, 0.0285809548298798, 0), G.pval = c(0.459823565292821, 
0.331017207021344, 0, 0.472429122382498, 0.923076923076923, 0.233441224071597, 
0, 0, 0.449283405570477, 0.39854875219121, 0.1033824858776, 0, 
0.67050446964625, 0.536522204808718, 0.298523869536799, 0, 0.187184099275467, 
0, 0.402378381197133, 0.573286671401718, 0.231775840875007, 0, 
0.16899600680047, 0, 0.424713577082118, 0.422367909113953, 0.640029649985411, 
0.89915809815462, 0.923076923076923, 0.581714897113396, 0.311023156069582, 
0.538820625101573, 0, 0, 0, 0.581321088637837, 0.923076923076923, 
0.74512944285661, 0.721800888359071, 0.895041583912033, 0.238824378192935, 
0, 0.340831793064694, 0.307592701047593, 0.0782728132585272, 
0, 0.676687070275783, 0.214780166896642, 0, 0, 0.440364452493701, 
0.671077187261338, 0.08752910157474, 0, 0.395455599297461, 0.354505234465227, 
0, 0.765791585978576, 0.884648411033706, 0.13584223176264, 0, 
0.485464459294359, 0.559340469762058, 0.0176016480137502, 0, 
0.581321088637837, 0.715772382415053, 0.886873393698937, 0.898244203414347, 
0.028819349258899, 0, 0.0861288457348567, 0, 0.531400534361952, 
0.304331085830123), A.pval = c(0, 0, 0.253479305414766, 0, 0.299685559596404, 
0.258028912209302, 0.569182638777977, 0.397798041042378, 0.717098811830984, 
0.372204666243789, 0, 0.101989906505876, 0, 0.315748225606602, 
0.333090195605916, 0.34146151151596, 0.102248502885969, 0.781818181814129, 
0.604496430174136, 0.708357103669275, 0.781818181814129, 0.565161045393451, 
0.781818181814129, 0.548306215445295, 0.541855128768767, 0, 0.632778405356051, 
0.223135801409755, 0, 0.386956948084686, 0, 0.534224998225601, 
0.39536321393214, 0.708133497415268, 0.458588601558952, 0.1605631313172, 
0, 0.607925708257708, 0.626556206531544, 0.771797533111817, 0.781818181814129, 
0.186749054976746, 0, 0.602539957872312, 0.698296634619465, 0.00518493279165844, 
0, 0.222185887610625, 0.0355439138519168, 0.161642646280651, 
0.113131827070211, 0, 0, 0.0121552621192631, 0, 0, 0.152827270372666, 
0.229430183292083, 0.422421614299191, 0.781818181814129, 0.00293581396550835, 
0, 0.319005216111249, 0, 0.548562575616313, 0.781818181814129, 
0.62037521165564, 0.781818181814129, 0.773390287354515, 0.781818181814129, 
0.0170483254389637, 0.0495125824067136, 0.000315787552290558, 
0, 0.517508676602548), guide.position = 1:75), .Names = c("A.area", 
"C.area", "G.area", "T.area", "Tot.area", "A.perc", "C.perc", 
"G.perc", "T.perc", "base.call", "index", "guide.seq", "T.pval", 
"C.pval", "G.pval", "A.pval", "guide.position"), row.names = 276:350, class = "data.frame")
```

*Report generated using EditR v1.0.8*
