# Supplementary material for: Mechanistic and genetic basis of single-strand templated repair at Cas12a-induced DNA breaks in Chlamydomonas reinhardtii
Source: Nat Commun. 2021 Nov 19;12:6751. doi: 10.1038/s41467-021-27004-1 (PMC8604939; doi:10.1038/s41467-021-27004-1)
Supplement: Supplementary file 22 — Source Data [file 41467_2021_27004_MOESM22_ESM.zip › Source Data/EditR analysis/EditR outputs/Sense/rep1_ssODN_sense_0_16_32.html]

EditR v1.0.8 report


# EditR v1.0.8 report

- Data QA
  - Filtering data
  - Percent noise peak area
  - Base information
- Predicted editing
  - Editing bar plot
  - Editing table plot
  - Table of editing results
- For use in R

## Data QA

### Filtering data

What the data looked like prefiltering:

and the post filtering signal / noise plot:

### Percent noise peak area

### Base information

Here’s information about the signal of each base, the critical percent value where any higher value would be called as significant, and Filliben’s correlation for how well the noise was modelled by the zero adjusted gamma distribution.

| Base | Average percent signal | Average peak area | Critical percent value | model mu | Fillibens correlation |
| --- | --- | --- | --- | --- | --- |
| A | 92.13891 | 271.8824 | 8.588663 | 3.379133 | 0.9897281 |
| C | 91.61522 | 285.9104 | 5.782922 | 2.256861 | 0.9925583 |
| G | 92.83244 | 282.0351 | 6.330004 | 2.199739 | 0.9955694 |
| T | 93.14660 | 302.3488 | 6.342291 | 2.328862 | 0.9958217 |

## Predicted editing

### Editing bar plot

### Editing table plot

### Table of editing results


Here’s the entire guide region

| Sanger position | Guide position | Guide sequence | Sanger base call | Focal base | Focal base peak area | p value |  |
| --- | --- | --- | --- | --- | --- | --- | --- |
| 277 | 1 | A | A | A | 94.42 | 0.000000e+00 | \* |
| 277 | 1 | A | A | C | 2.15 | 4.116029e-01 |  |
| 277 | 1 | A | A | G | 1.29 | 7.221928e-01 |  |
| 277 | 1 | A | A | T | 2.15 | 4.342804e-01 |  |
| 278 | 2 | A | A | A | 95.21 | 0.000000e+00 | \* |
| 278 | 2 | A | A | C | 0.00 | 8.809524e-01 |  |
| 278 | 2 | A | A | G | 1.71 | 5.718156e-01 |  |
| 278 | 2 | A | A | T | 3.08 | 2.177091e-01 |  |
| 279 | 3 | G | G | A | 3.91 | 3.041940e-01 |  |
| 279 | 3 | G | G | C | 2.17 | 4.031777e-01 |  |
| 279 | 3 | G | G | G | 93.91 | 0.000000e+00 | \* |
| 279 | 3 | G | G | T | 0.00 | 9.042553e-01 |  |
| 280 | 4 | A | A | A | 94.83 | 0.000000e+00 | \* |
| 280 | 4 | A | A | C | 1.03 | 7.663575e-01 |  |
| 280 | 4 | A | A | G | 1.21 | 7.506218e-01 |  |
| 280 | 4 | A | A | T | 2.93 | 2.455667e-01 |  |
| 281 | 5 | C | C | A | 6.02 | 7.536684e-02 |  |
| 281 | 5 | C | C | C | 89.97 | 0.000000e+00 | \* |
| 281 | 5 | C | C | G | 2.34 | 3.759423e-01 |  |
| 281 | 5 | C | C | T | 1.67 | 5.785144e-01 |  |
| 282 | 6 | T | T | A | 4.68 | 1.898304e-01 |  |
| 282 | 6 | T | T | C | 2.68 | 2.697235e-01 |  |
| 282 | 6 | T | T | G | 3.68 | 1.273894e-01 |  |
| 282 | 6 | T | T | T | 88.96 | 0.000000e+00 | \* |
| 283 | 7 | G | G | A | 3.44 | 3.947988e-01 |  |
| 283 | 7 | G | G | C | 0.52 | 8.636966e-01 |  |
| 283 | 7 | G | G | G | 95.18 | 0.000000e+00 | \* |
| 283 | 7 | G | G | T | 0.86 | 8.171529e-01 |  |
| 284 | 8 | G | G | A | 5.15 | 1.388244e-01 |  |
| 284 | 8 | G | G | C | 1.03 | 7.673042e-01 |  |
| 284 | 8 | G | G | G | 93.30 | 0.000000e+00 | \* |
| 284 | 8 | G | G | T | 0.52 | 8.795281e-01 |  |
| 285 | 9 | C | C | A | 7.02 | 3.549441e-02 |  |
| 285 | 9 | C | C | C | 86.40 | 0.000000e+00 | \* |
| 285 | 9 | C | C | G | 3.51 | 1.477338e-01 |  |
| 285 | 9 | C | C | T | 3.07 | 2.198267e-01 |  |
| 286 | 10 | C | C | A | 3.85 | 3.161195e-01 |  |
| 286 | 10 | C | C | C | 90.83 | 0.000000e+00 | \* |
| 286 | 10 | C | C | G | 3.25 | 1.834509e-01 |  |
| 286 | 10 | C | C | T | 2.07 | 4.559383e-01 |  |
| 287 | 11 | A | A | A | 89.26 | 0.000000e+00 | \* |
| 287 | 11 | A | A | C | 2.48 | 3.177656e-01 |  |
| 287 | 11 | A | A | G | 4.96 | 3.906328e-02 |  |
| 287 | 11 | A | A | T | 3.31 | 1.812407e-01 |  |
| 288 | 12 | G | G | A | 4.94 | 1.605377e-01 |  |
| 288 | 12 | G | G | C | 0.41 | 8.723109e-01 |  |
| 288 | 12 | G | G | G | 94.24 | 0.000000e+00 | \* |
| 288 | 12 | G | G | T | 0.41 | 8.906489e-01 |  |
| 289 | 13 | A | A | A | 94.49 | 0.000000e+00 | \* |
| 289 | 13 | A | A | C | 0.89 | 8.025575e-01 |  |
| 289 | 13 | A | A | G | 1.78 | 5.499534e-01 |  |
| 289 | 13 | A | A | T | 2.84 | 2.632554e-01 |  |
| 290 | 14 | C | C | A | 5.99 | 7.683184e-02 |  |
| 290 | 14 | C | C | C | 89.27 | 0.000000e+00 | \* |
| 290 | 14 | C | C | G | 2.52 | 3.286000e-01 |  |
| 290 | 14 | C | C | T | 2.21 | 4.167035e-01 |  |
| 291 | 15 | C | C | A | 5.68 | 9.653465e-02 |  |
| 291 | 15 | C | C | C | 89.08 | 0.000000e+00 | \* |
| 291 | 15 | C | C | G | 3.49 | 1.496993e-01 |  |
| 291 | 15 | C | C | T | 1.75 | 5.549449e-01 |  |
| 292 | 16 | G | G | A | 5.17 | 1.371574e-01 |  |
| 292 | 16 | G | G | C | 1.03 | 7.663575e-01 |  |
| 292 | 16 | G | G | G | 92.41 | 0.000000e+00 | \* |
| 292 | 16 | G | G | T | 1.38 | 6.714512e-01 |  |
| 293 | 17 | T | T | A | 3.90 | 3.071837e-01 |  |
| 293 | 17 | T | T | C | 0.87 | 8.075307e-01 |  |
| 293 | 17 | T | T | G | 3.03 | 2.208288e-01 |  |
| 293 | 17 | T | T | T | 92.21 | 0.000000e+00 | \* |
| 294 | 18 | G | G | A | 3.40 | 4.027884e-01 |  |
| 294 | 18 | G | G | C | 0.21 | 8.799321e-01 |  |
| 294 | 18 | G | G | G | 95.32 | 0.000000e+00 | \* |
| 294 | 18 | G | G | T | 1.06 | 7.653070e-01 |  |
| 295 | 19 | T | T | A | 1.11 | 8.986377e-01 |  |
| 295 | 19 | T | T | C | 0.00 | 8.809524e-01 |  |
| 295 | 19 | T | T | G | 3.69 | 1.261524e-01 |  |
| 295 | 19 | T | T | T | 95.20 | 0.000000e+00 | \* |
| 296 | 20 | T | T | A | 1.34 | 8.656613e-01 |  |
| 296 | 20 | T | T | C | 1.01 | 7.736695e-01 |  |
| 296 | 20 | T | T | G | 3.36 | 1.684217e-01 |  |
| 296 | 20 | T | T | T | 94.30 | 0.000000e+00 | \* |
| 297 | 21 | T | T | A | 0.67 | 9.353760e-01 |  |
| 297 | 21 | T | T | C | 1.67 | 5.649574e-01 |  |
| 297 | 21 | T | T | G | 3.68 | 1.273894e-01 |  |
| 297 | 21 | T | T | T | 93.98 | 0.000000e+00 | \* |
| 298 | 22 | G | G | A | 1.73 | 7.941095e-01 |  |
| 298 | 22 | G | G | C | 0.00 | 8.809524e-01 |  |
| 298 | 22 | G | G | G | 96.54 | 0.000000e+00 | \* |
| 298 | 22 | G | G | T | 1.73 | 5.607278e-01 |  |
| 299 | 23 | T | T | A | 0.00 | 9.454545e-01 |  |
| 299 | 23 | T | T | C | 1.92 | 4.816562e-01 |  |
| 299 | 23 | T | T | G | 3.46 | 1.538666e-01 |  |
| 299 | 23 | T | T | T | 94.62 | 0.000000e+00 | \* |
| 300 | 24 | G | G | A | 1.52 | 8.360224e-01 |  |
| 300 | 24 | G | G | C | 1.52 | 6.178079e-01 |  |
| 300 | 24 | G | G | G | 95.15 | 0.000000e+00 | \* |
| 300 | 24 | G | G | T | 1.82 | 5.325424e-01 |  |
| 301 | 25 | C | C | A | 1.49 | 8.401376e-01 |  |
| 301 | 25 | C | C | C | 94.40 | 0.000000e+00 | \* |
| 301 | 25 | C | C | G | 4.10 | 8.704576e-02 |  |
| 301 | 25 | C | C | T | 0.00 | 9.042553e-01 |  |
| 302 | 26 | A | A | A | 87.04 | 0.000000e+00 | \* |
| 302 | 26 | A | A | C | 3.70 | 1.023515e-01 |  |
| 302 | 26 | A | A | G | 5.09 | 3.432302e-02 |  |
| 302 | 26 | A | A | T | 4.17 | 8.520315e-02 |  |
| 303 | 27 | C | C | A | 2.44 | 6.285554e-01 |  |
| 303 | 27 | C | C | C | 93.73 | 0.000000e+00 | \* |
| 303 | 27 | C | C | G | 1.39 | 6.843640e-01 |  |
| 303 | 27 | C | C | T | 2.44 | 3.552711e-01 |  |
| 304 | 28 | T | T | A | 1.81 | 7.764143e-01 |  |
| 304 | 28 | T | T | C | 3.26 | 1.585145e-01 |  |
| 304 | 28 | T | T | G | 1.45 | 6.645191e-01 |  |
| 304 | 28 | T | T | T | 93.48 | 0.000000e+00 | \* |
| 305 | 29 | A | A | A | 93.86 | 0.000000e+00 | \* |
| 305 | 29 | A | A | C | 1.81 | 5.204644e-01 |  |
| 305 | 29 | A | A | G | 2.17 | 4.256286e-01 |  |
| 305 | 29 | A | A | T | 2.17 | 4.285519e-01 |  |
| 306 | 30 | C | C | A | 3.31 | 4.232100e-01 |  |
| 306 | 30 | C | C | C | 92.28 | 0.000000e+00 | \* |
| 306 | 30 | C | C | G | 2.21 | 4.139679e-01 |  |
| 306 | 30 | C | C | T | 2.21 | 4.173509e-01 |  |
| 307 | 31 | A | A | A | 91.22 | 0.000000e+00 | \* |
| 307 | 31 | A | A | C | 2.44 | 3.283150e-01 |  |
| 307 | 31 | A | A | G | 2.93 | 2.401198e-01 |  |
| 307 | 31 | A | A | T | 3.41 | 1.654183e-01 |  |
| 308 | 32 | C | C | A | 2.04 | 7.246708e-01 |  |
| 308 | 32 | C | C | C | 91.43 | 0.000000e+00 | \* |
| 308 | 32 | C | C | G | 3.27 | 1.817846e-01 |  |
| 308 | 32 | C | C | T | 3.27 | 1.874394e-01 |  |
| 309 | 33 | G | G | A | 4.08 | 2.755639e-01 |  |
| 309 | 33 | G | G | C | 2.55 | 2.995774e-01 |  |
| 309 | 33 | G | G | G | 92.35 | 0.000000e+00 | \* |
| 309 | 33 | G | G | T | 1.02 | 7.771139e-01 |  |
| 310 | 34 | G | G | A | 2.01 | 7.310472e-01 |  |
| 310 | 34 | G | G | C | 0.00 | 8.809524e-01 |  |
| 310 | 34 | G | G | G | 96.98 | 0.000000e+00 | \* |
| 310 | 34 | G | G | T | 1.01 | 7.807634e-01 |  |
| 311 | 35 | G | G | A | 2.41 | 6.357732e-01 |  |
| 311 | 35 | G | G | C | 2.81 | 2.397468e-01 |  |
| 311 | 35 | G | G | G | 94.78 | 0.000000e+00 | \* |
| 311 | 35 | G | G | T | 0.00 | 9.042553e-01 |  |
| 312 | 36 | C | C | A | 2.63 | 5.812716e-01 |  |
| 312 | 36 | C | C | C | 92.98 | 0.000000e+00 | \* |
| 312 | 36 | C | C | G | 3.95 | 1.003473e-01 |  |
| 312 | 36 | C | C | T | 0.44 | 8.881080e-01 |  |
| 313 | 37 | A | A | A | 92.27 | 0.000000e+00 | \* |
| 313 | 37 | A | A | C | 2.73 | 2.579913e-01 |  |
| 313 | 37 | A | A | G | 1.82 | 5.357763e-01 |  |
| 313 | 37 | A | A | T | 3.18 | 2.007806e-01 |  |
| 314 | 38 | C | C | A | 2.97 | 4.998149e-01 |  |
| 314 | 38 | C | C | C | 94.06 | 0.000000e+00 | \* |
| 314 | 38 | C | C | G | 1.65 | 5.933940e-01 |  |
| 314 | 38 | C | C | T | 1.32 | 6.898509e-01 |  |
| 315 | 39 | C | C | A | 2.40 | 6.388114e-01 |  |
| 315 | 39 | C | C | C | 91.78 | 0.000000e+00 | \* |
| 315 | 39 | C | C | G | 3.77 | 1.178590e-01 |  |
| 315 | 39 | C | C | T | 2.05 | 4.606950e-01 |  |
| 316 | 40 | C | C | A | 2.78 | 5.456996e-01 |  |
| 316 | 40 | C | C | C | 91.32 | 0.000000e+00 | \* |
| 316 | 40 | C | C | G | 2.43 | 3.521744e-01 |  |
| 316 | 40 | C | C | T | 3.47 | 1.575311e-01 |  |
| 317 | 41 | T | T | A | 0.00 | 9.454545e-01 |  |
| 317 | 41 | T | T | C | 3.91 | 8.250107e-02 |  |
| 317 | 41 | T | T | G | 3.04 | 2.184674e-01 |  |
| 317 | 41 | T | T | T | 93.04 | 0.000000e+00 | \* |
| 318 | 42 | G | G | A | 2.06 | 7.204557e-01 |  |
| 318 | 42 | G | G | C | 2.06 | 4.383882e-01 |  |
| 318 | 42 | G | G | G | 94.12 | 0.000000e+00 | \* |
| 318 | 42 | G | G | T | 1.76 | 5.492853e-01 |  |
| 319 | 43 | A | A | A | 75.40 | 0.000000e+00 | \* |
| 319 | 43 | A | A | C | 1.94 | 4.756080e-01 |  |
| 319 | 43 | A | A | G | 3.88 | 1.062640e-01 |  |
| 319 | 43 | A | A | T | 18.77 | 5.342601e-09 | \* |
| 320 | 44 | C | C | A | 2.30 | 6.628905e-01 |  |
| 320 | 44 | C | C | C | 91.57 | 0.000000e+00 | \* |
| 320 | 44 | C | C | G | 4.21 | 7.870847e-02 |  |
| 320 | 44 | C | C | T | 1.92 | 5.024299e-01 |  |
| 321 | 45 | C | C | A | 0.00 | 9.454545e-01 |  |
| 321 | 45 | C | C | C | 93.67 | 0.000000e+00 | \* |
| 321 | 45 | C | C | G | 4.07 | 8.962327e-02 |  |
| 321 | 45 | C | C | T | 2.26 | 4.017292e-01 |  |
| 322 | 46 | G | G | A | 5.71 | 9.399364e-02 |  |
| 322 | 46 | G | G | C | 3.81 | 9.184318e-02 |  |
| 322 | 46 | G | G | G | 89.52 | 0.000000e+00 | \* |
| 322 | 46 | G | G | T | 0.95 | 7.948591e-01 |  |
| 323 | 47 | A | A | A | 94.42 | 0.000000e+00 | \* |
| 323 | 47 | A | A | C | 0.97 | 7.828103e-01 |  |
| 323 | 47 | A | A | G | 2.18 | 4.202138e-01 |  |
| 323 | 47 | A | A | T | 2.43 | 3.582728e-01 |  |
| 324 | 48 | C | C | A | 7.60 | 2.241924e-02 |  |
| 324 | 48 | C | C | C | 86.80 | 0.000000e+00 | \* |
| 324 | 48 | C | C | G | 2.40 | 3.601723e-01 |  |
| 324 | 48 | C | C | T | 3.20 | 1.978105e-01 |  |
| 325 | 49 | G | G | A | 0.00 | 9.454545e-01 |  |
| 325 | 49 | G | G | C | 4.93 | 2.714036e-02 |  |
| 325 | 49 | G | G | G | 92.96 | 0.000000e+00 | \* |
| 325 | 49 | G | G | T | 2.11 | 4.438246e-01 |  |
| 326 | 50 | G | G | A | 2.74 | 5.559211e-01 |  |
| 326 | 50 | G | G | C | 1.22 | 7.143729e-01 |  |
| 326 | 50 | G | G | G | 94.53 | 0.000000e+00 | \* |
| 326 | 50 | G | G | T | 1.52 | 6.270616e-01 |  |
| 327 | 51 | C | C | A | 2.23 | 6.784926e-01 |  |
| 327 | 51 | C | C | C | 91.06 | 0.000000e+00 | \* |
| 327 | 51 | C | C | G | 3.91 | 1.037143e-01 |  |
| 327 | 51 | C | C | T | 2.79 | 2.733098e-01 |  |
| 328 | 52 | A | A | A | 91.74 | 0.000000e+00 | \* |
| 328 | 52 | A | A | C | 3.67 | 1.059434e-01 |  |
| 328 | 52 | A | A | G | 4.59 | 5.564893e-02 |  |
| 328 | 52 | A | A | T | 0.00 | 9.042553e-01 |  |
| 329 | 53 | A | A | A | 94.62 | 0.000000e+00 | \* |
| 329 | 53 | A | A | C | 0.00 | 8.809524e-01 |  |
| 329 | 53 | A | A | G | 1.79 | 5.440165e-01 |  |
| 329 | 53 | A | A | T | 3.59 | 1.427118e-01 |  |
| 330 | 54 | G | G | A | 4.55 | 2.071954e-01 |  |
| 330 | 54 | G | G | C | 1.70 | 5.540871e-01 |  |
| 330 | 54 | G | G | G | 93.18 | 0.000000e+00 | \* |
| 330 | 54 | G | G | T | 0.57 | 8.724735e-01 |  |
| 331 | 55 | A | A | A | 94.58 | 0.000000e+00 | \* |
| 331 | 55 | A | A | C | 0.00 | 8.809524e-01 |  |
| 331 | 55 | A | A | G | 2.53 | 3.277577e-01 |  |
| 331 | 55 | A | A | T | 2.89 | 2.539726e-01 |  |
| 332 | 56 | A | A | A | 94.17 | 0.000000e+00 | \* |
| 332 | 56 | A | A | C | 0.45 | 8.696887e-01 |  |
| 332 | 56 | A | A | G | 1.35 | 7.016549e-01 |  |
| 332 | 56 | A | A | T | 4.04 | 9.597567e-02 |  |
| 333 | 57 | G | G | A | 6.67 | 4.650172e-02 |  |
| 333 | 57 | G | G | C | 0.00 | 8.809524e-01 |  |
| 333 | 57 | G | G | G | 89.33 | 0.000000e+00 | \* |
| 333 | 57 | G | G | T | 4.00 | 9.913677e-02 |  |
| 334 | 58 | T | T | A | 0.44 | 9.429319e-01 |  |
| 334 | 58 | T | T | C | 0.44 | 8.702866e-01 |  |
| 334 | 58 | T | T | G | 0.00 | 9.888889e-01 |  |
| 334 | 58 | T | T | T | 99.12 | 0.000000e+00 | \* |
| 335 | 59 | T | T | A | 25.11 | 1.468466e-09 | \* |
| 335 | 59 | T | T | C | 1.73 | 5.450009e-01 |  |
| 335 | 59 | T | T | G | 2.60 | 3.107766e-01 |  |
| 335 | 59 | T | T | T | 70.56 | 0.000000e+00 | \* |
| 336 | 60 | C | C | A | 5.43 | 1.148755e-01 |  |
| 336 | 60 | C | C | C | 91.40 | 0.000000e+00 | \* |
| 336 | 60 | C | C | G | 1.36 | 6.973123e-01 |  |
| 336 | 60 | C | C | T | 1.81 | 5.351088e-01 |  |
| 337 | 61 | G | G | A | 6.54 | 5.135323e-02 |  |
| 337 | 61 | G | G | C | 3.27 | 1.574404e-01 |  |
| 337 | 61 | G | G | G | 87.58 | 0.000000e+00 | \* |
| 337 | 61 | G | G | T | 2.61 | 3.127895e-01 |  |
| 338 | 62 | A | A | A | 93.79 | 0.000000e+00 | \* |
| 338 | 62 | A | A | C | 1.48 | 6.297813e-01 |  |
| 338 | 62 | A | A | G | 1.78 | 5.503104e-01 |  |
| 338 | 62 | A | A | T | 2.96 | 2.402909e-01 |  |
| 339 | 63 | C | C | A | 3.94 | 2.999997e-01 |  |
| 339 | 63 | C | C | C | 91.34 | 0.000000e+00 | \* |
| 339 | 63 | C | C | G | 0.79 | 8.851111e-01 |  |
| 339 | 63 | C | C | T | 3.94 | 1.049132e-01 |  |
| 340 | 64 | A | A | A | 87.59 | 0.000000e+00 | \* |
| 340 | 64 | A | A | C | 4.14 | 6.507913e-02 |  |
| 340 | 64 | A | A | G | 4.14 | 8.443024e-02 |  |
| 340 | 64 | A | A | T | 4.14 | 8.747176e-02 |  |
| 341 | 65 | G | G | A | 1.63 | 8.148993e-01 |  |
| 341 | 65 | G | G | C | 2.44 | 3.283150e-01 |  |
| 341 | 65 | G | G | G | 95.12 | 0.000000e+00 | \* |
| 341 | 65 | G | G | T | 0.81 | 8.278729e-01 |  |
| 342 | 66 | C | C | A | 1.31 | 8.707029e-01 |  |
| 342 | 66 | C | C | C | 93.01 | 0.000000e+00 | \* |
| 342 | 66 | C | C | G | 2.62 | 3.054441e-01 |  |
| 342 | 66 | C | C | T | 3.06 | 2.222089e-01 |  |
| 343 | 67 | T | T | A | 0.00 | 9.454545e-01 |  |
| 343 | 67 | T | T | C | 2.71 | 2.611524e-01 |  |
| 343 | 67 | T | T | G | 0.78 | 8.884716e-01 |  |
| 343 | 67 | T | T | T | 96.51 | 0.000000e+00 | \* |
| 344 | 68 | C | C | A | 3.61 | 3.598867e-01 |  |
| 344 | 68 | C | C | C | 90.76 | 0.000000e+00 | \* |
| 344 | 68 | C | C | G | 2.41 | 3.576355e-01 |  |
| 344 | 68 | C | C | T | 3.21 | 1.957330e-01 |  |
| 345 | 69 | C | C | A | 2.37 | 6.447903e-01 |  |
| 345 | 69 | C | C | C | 93.90 | 0.000000e+00 | \* |
| 345 | 69 | C | C | G | 0.68 | 9.136388e-01 |  |
| 345 | 69 | C | C | T | 3.05 | 2.232676e-01 |  |
| 346 | 70 | C | C | A | 0.00 | 9.454545e-01 |  |
| 346 | 70 | C | C | C | 96.08 | 0.000000e+00 | \* |
| 346 | 70 | C | C | G | 2.61 | 3.067793e-01 |  |
| 346 | 70 | C | C | T | 1.31 | 6.938399e-01 |  |
| 347 | 71 | G | G | A | 4.86 | 1.685247e-01 |  |
| 347 | 71 | G | G | C | 3.78 | 9.430730e-02 |  |
| 347 | 71 | G | G | G | 91.35 | 0.000000e+00 | \* |
| 347 | 71 | G | G | T | 0.00 | 9.042553e-01 |  |
| 348 | 72 | C | C | A | 3.32 | 4.213224e-01 |  |
| 348 | 72 | C | C | C | 91.00 | 0.000000e+00 | \* |
| 348 | 72 | C | C | G | 3.32 | 1.739568e-01 |  |
| 348 | 72 | C | C | T | 2.37 | 3.730895e-01 |  |
| 349 | 73 | G | G | A | 7.69 | 2.082038e-02 |  |
| 349 | 73 | G | G | C | 4.95 | 2.666577e-02 |  |
| 349 | 73 | G | G | G | 87.36 | 0.000000e+00 | \* |
| 349 | 73 | G | G | T | 0.00 | 9.042553e-01 |  |
| 350 | 74 | A | A | A | 93.79 | 0.000000e+00 | \* |
| 350 | 74 | A | A | C | 1.38 | 6.627328e-01 |  |
| 350 | 74 | A | A | G | 2.76 | 2.743959e-01 |  |
| 350 | 74 | A | A | T | 2.07 | 4.565357e-01 |  |
| 351 | 75 | C | C | A | 3.82 | 3.214551e-01 |  |
| 351 | 75 | C | C | C | 61.83 | 0.000000e+00 | \* |
| 351 | 75 | C | C | G | 32.44 | 6.661338e-16 | \* |
| 351 | 75 | C | C | T | 1.91 | 5.046658e-01 |  |

## For use in R

If you want to work with the results in R, here is output that you can copy and paste in your terminal to get:

The base information:

```
structure(list(focal.base = c("A", "C", "G", "T"), avg.percsignal = c(92.1389105092151, 
91.6152233105011, 92.832435519834, 93.1465963139004), avg.areasignal = c(271.882352941176, 
285.910447761194, 282.035087719298, 302.348837209302), crit.perc.area = c(8.58866288411247, 
5.78292250121907, 6.33000420323496, 6.34229082978009), mu = c(3.37913278029058, 
2.25686146037978, 2.1997390195368, 2.32886180900301), fillibens = c(0.989728143852579, 
0.992558347713831, 0.995569379710549, 0.995821698397157)), .Names = c("focal.base", 
"avg.percsignal", "avg.areasignal", "crit.perc.area", "mu", "fillibens"
), row.names = c(NA, -4L), class = "data.frame")
```

the data.frame that contains information on the guide region:

```
structure(list(A.area = c(220, 278, 9, 550, 18, 14, 20, 20, 16, 
13, 216, 12, 532, 19, 13, 15, 9, 16, 3, 4, 2, 7, 0, 5, 4, 188, 
7, 5, 260, 9, 187, 5, 8, 6, 6, 6, 203, 9, 7, 8, 0, 7, 233, 6, 
0, 12, 389, 19, 0, 9, 4, 100, 211, 8, 262, 210, 10, 1, 58, 12, 
10, 317, 10, 127, 4, 3, 0, 9, 7, 0, 9, 7, 14, 272, 10), C.area = c(5, 
0, 5, 6, 269, 8, 3, 4, 197, 307, 6, 1, 5, 283, 204, 3, 2, 1, 
0, 3, 5, 0, 5, 5, 253, 8, 269, 9, 5, 251, 5, 224, 5, 0, 7, 212, 
6, 285, 268, 263, 9, 7, 6, 239, 207, 8, 4, 217, 7, 4, 163, 4, 
0, 3, 0, 1, 0, 1, 4, 202, 5, 5, 232, 6, 6, 213, 7, 226, 277, 
147, 7, 192, 9, 4, 162), G.area = c(3, 5, 216, 7, 7, 11, 553, 
362, 8, 11, 12, 229, 10, 8, 8, 268, 7, 448, 10, 10, 11, 391, 
9, 314, 11, 11, 4, 4, 6, 6, 6, 8, 181, 289, 236, 9, 4, 5, 11, 
7, 7, 320, 12, 11, 9, 188, 9, 6, 132, 311, 7, 5, 4, 164, 7, 3, 
134, 0, 6, 3, 134, 6, 2, 6, 234, 6, 2, 6, 2, 4, 169, 7, 159, 
8, 85), T.area = c(5, 9, 0, 17, 5, 266, 5, 2, 7, 7, 8, 1, 16, 
7, 4, 4, 213, 5, 258, 281, 281, 7, 246, 6, 0, 9, 7, 258, 6, 6, 
7, 8, 2, 3, 0, 1, 7, 4, 6, 10, 214, 6, 58, 5, 5, 2, 10, 8, 3, 
5, 5, 0, 8, 1, 8, 9, 6, 225, 163, 4, 4, 10, 10, 6, 2, 7, 249, 
8, 9, 2, 0, 5, 0, 6, 5), Tot.area = c(233, 292, 230, 580, 299, 
299, 581, 388, 228, 338, 242, 243, 563, 317, 229, 290, 231, 470, 
271, 298, 299, 405, 260, 330, 268, 216, 287, 276, 277, 272, 205, 
245, 196, 298, 249, 228, 220, 303, 292, 288, 230, 340, 309, 261, 
221, 210, 412, 250, 142, 329, 179, 109, 223, 176, 277, 223, 150, 
227, 231, 221, 153, 338, 254, 145, 246, 229, 258, 249, 295, 153, 
185, 211, 182, 290, 262), A.perc = c(94.4206008583691, 95.2054794520548, 
3.91304347826087, 94.8275862068966, 6.02006688963211, 4.68227424749164, 
3.44234079173838, 5.15463917525773, 7.01754385964912, 3.84615384615385, 
89.2561983471074, 4.93827160493827, 94.49378330373, 5.99369085173502, 
5.67685589519651, 5.17241379310345, 3.8961038961039, 3.40425531914894, 
1.1070110701107, 1.34228187919463, 0.668896321070234, 1.72839506172839, 
0, 1.51515151515152, 1.49253731343284, 87.037037037037, 2.4390243902439, 
1.81159420289855, 93.8628158844765, 3.30882352941176, 91.219512195122, 
2.04081632653061, 4.08163265306122, 2.01342281879195, 2.40963855421687, 
2.63157894736842, 92.2727272727273, 2.97029702970297, 2.3972602739726, 
2.77777777777778, 0, 2.05882352941176, 75.4045307443366, 2.29885057471264, 
0, 5.71428571428571, 94.4174757281553, 7.6, 0, 2.73556231003039, 
2.23463687150838, 91.743119266055, 94.6188340807175, 4.54545454545455, 
94.5848375451263, 94.1704035874439, 6.66666666666667, 0.440528634361233, 
25.1082251082251, 5.42986425339367, 6.5359477124183, 93.7869822485207, 
3.93700787401575, 87.5862068965517, 1.6260162601626, 1.31004366812227, 
0, 3.6144578313253, 2.3728813559322, 0, 4.86486486486486, 3.3175355450237, 
7.69230769230769, 93.7931034482759, 3.81679389312977), C.perc = c(2.14592274678112, 
0, 2.17391304347826, 1.03448275862069, 89.9665551839465, 2.67558528428094, 
0.516351118760757, 1.03092783505155, 86.4035087719298, 90.8284023668639, 
2.47933884297521, 0.411522633744856, 0.88809946714032, 89.2744479495268, 
89.0829694323144, 1.03448275862069, 0.865800865800866, 0.212765957446809, 
0, 1.00671140939597, 1.67224080267559, 0, 1.92307692307692, 1.51515151515152, 
94.4029850746269, 3.7037037037037, 93.7282229965157, 3.26086956521739, 
1.80505415162455, 92.2794117647059, 2.4390243902439, 91.4285714285714, 
2.55102040816327, 0, 2.81124497991968, 92.9824561403509, 2.72727272727273, 
94.0594059405941, 91.7808219178082, 91.3194444444444, 3.91304347826087, 
2.05882352941176, 1.94174757281553, 91.5708812260536, 93.6651583710407, 
3.80952380952381, 0.970873786407767, 86.8, 4.92957746478873, 
1.21580547112462, 91.0614525139665, 3.6697247706422, 0, 1.70454545454545, 
0, 0.448430493273543, 0, 0.440528634361233, 1.73160173160173, 
91.4027149321267, 3.26797385620915, 1.4792899408284, 91.3385826771653, 
4.13793103448276, 2.4390243902439, 93.0131004366812, 2.71317829457364, 
90.7630522088353, 93.8983050847458, 96.078431372549, 3.78378378378378, 
90.9952606635071, 4.94505494505495, 1.37931034482759, 61.8320610687023
), G.perc = c(1.28755364806867, 1.71232876712329, 93.9130434782609, 
1.20689655172414, 2.34113712374582, 3.67892976588629, 95.1807228915663, 
93.298969072165, 3.50877192982456, 3.25443786982249, 4.95867768595041, 
94.238683127572, 1.77619893428064, 2.52365930599369, 3.49344978165939, 
92.4137931034483, 3.03030303030303, 95.3191489361702, 3.690036900369, 
3.35570469798658, 3.67892976588629, 96.5432098765432, 3.46153846153846, 
95.1515151515152, 4.1044776119403, 5.09259259259259, 1.39372822299652, 
1.44927536231884, 2.16606498194946, 2.20588235294118, 2.92682926829268, 
3.26530612244898, 92.3469387755102, 96.9798657718121, 94.7791164658635, 
3.94736842105263, 1.81818181818182, 1.65016501650165, 3.76712328767123, 
2.43055555555556, 3.04347826086957, 94.1176470588235, 3.88349514563107, 
4.21455938697318, 4.07239819004525, 89.5238095238095, 2.18446601941748, 
2.4, 92.9577464788732, 94.5288753799392, 3.91061452513966, 4.58715596330275, 
1.79372197309417, 93.1818181818182, 2.52707581227437, 1.34529147982063, 
89.3333333333333, 0, 2.5974025974026, 1.35746606334842, 87.5816993464052, 
1.77514792899408, 0.78740157480315, 4.13793103448276, 95.1219512195122, 
2.62008733624454, 0.775193798449612, 2.40963855421687, 0.677966101694915, 
2.61437908496732, 91.3513513513514, 3.3175355450237, 87.3626373626374, 
2.75862068965517, 32.4427480916031), T.perc = c(2.14592274678112, 
3.08219178082192, 0, 2.93103448275862, 1.67224080267559, 88.9632107023411, 
0.860585197934596, 0.515463917525773, 3.07017543859649, 2.07100591715976, 
3.30578512396694, 0.411522633744856, 2.84191829484902, 2.20820189274448, 
1.74672489082969, 1.37931034482759, 92.2077922077922, 1.06382978723404, 
95.2029520295203, 94.2953020134228, 93.9799331103679, 1.72839506172839, 
94.6153846153846, 1.81818181818182, 0, 4.16666666666667, 2.4390243902439, 
93.4782608695652, 2.16606498194946, 2.20588235294118, 3.41463414634146, 
3.26530612244898, 1.02040816326531, 1.00671140939597, 0, 0.43859649122807, 
3.18181818181818, 1.32013201320132, 2.05479452054795, 3.47222222222222, 
93.0434782608696, 1.76470588235294, 18.7702265372168, 1.91570881226054, 
2.26244343891403, 0.952380952380952, 2.42718446601942, 3.2, 2.11267605633803, 
1.51975683890578, 2.79329608938547, 0, 3.58744394618834, 0.568181818181818, 
2.88808664259928, 4.03587443946188, 4, 99.1189427312775, 70.5627705627706, 
1.80995475113122, 2.61437908496732, 2.9585798816568, 3.93700787401575, 
4.13793103448276, 0.813008130081301, 3.05676855895197, 96.5116279069767, 
3.21285140562249, 3.05084745762712, 1.30718954248366, 0, 2.3696682464455, 
0, 2.06896551724138, 1.90839694656489), base.call = c("A", "A", 
"G", "A", "C", "T", "G", "G", "C", "C", "A", "G", "A", "C", "C", 
"G", "T", "G", "T", "T", "T", "G", "T", "G", "C", "A", "C", "T", 
"A", "C", "A", "C", "G", "G", "G", "C", "A", "C", "C", "C", "T", 
"G", "A", "C", "C", "G", "A", "C", "G", "G", "C", "A", "A", "G", 
"A", "A", "G", "T", "T", "C", "G", "A", "C", "A", "G", "C", "T", 
"C", "C", "C", "G", "C", "G", "A", "C"), index = 277:351, guide.seq = c("A", 
"A", "G", "A", "C", "T", "G", "G", "C", "C", "A", "G", "A", "C", 
"C", "G", "T", "G", "T", "T", "T", "G", "T", "G", "C", "A", "C", 
"T", "A", "C", "A", "C", "G", "G", "G", "C", "A", "C", "C", "C", 
"T", "G", "A", "C", "C", "G", "A", "C", "G", "G", "C", "A", "A", 
"G", "A", "A", "G", "T", "T", "C", "G", "A", "C", "A", "G", "C", 
"T", "C", "C", "C", "G", "C", "G", "A", "C"), T.pval = c(0.434280383225915, 
0.217709077996281, 0.904255319142527, 0.245566731992107, 0.57851440380048, 
0, 0.817152909343771, 0.879528125476148, 0.219826723402162, 0.455938343723551, 
0.181240705386146, 0.890648929226224, 0.263255391980749, 0.416703470578331, 
0.554944895735532, 0.671451218510835, 0, 0.765307041948779, 0, 
0, 0, 0.560727783057991, 0, 0.53254240417517, 0.904255319142527, 
0.0852031474459205, 0.355271121929403, 0, 0.428551939764268, 
0.417350861803655, 0.165418281093154, 0.187439435715738, 0.777113863708376, 
0.780763377621943, 0.904255319142527, 0.888107990675862, 0.20078061670738, 
0.689850887217478, 0.460695044669648, 0.157531098385392, 0, 0.549285262296529, 
5.34260091722416e-09, 0.50242989913529, 0.401729159376283, 0.79485913758494, 
0.358272792770785, 0.197810501816572, 0.443824612331569, 0.62706160561347, 
0.273309816677364, 0.904255319142527, 0.142711805101524, 0.872473457543242, 
0.253972641227689, 0.0959756701183583, 0.0991367681471871, 0, 
0, 0.535108771695493, 0.312789474121245, 0.240290890887949, 0.104913245511616, 
0.0874717576453174, 0.827872931392419, 0.222208947920794, 0, 
0.195733019535472, 0.223267625013756, 0.69383988060393, 0.904255319142527, 
0.373089486928586, 0.904255319142527, 0.456535701004638, 0.50466578423465
), C.pval = c(0.411602869166603, 0.880952380952317, 0.403177668334676, 
0.766357464014009, 0, 0.269723478131411, 0.863696617782382, 0.767304234675152, 
0, 0, 0.317765635212083, 0.872310893770634, 0.802557451312406, 
0, 0, 0.766357464014009, 0.807530679600188, 0.879932052730175, 
0.880952380952317, 0.773669484339692, 0.564957351927122, 0.880952380952317, 
0.481656158969073, 0.617807935633774, 0, 0.102351518663226, 0, 
0.158514477712187, 0.520464373017527, 0, 0.328314997097491, 0, 
0.299577397872393, 0.880952380952317, 0.239746847951221, 0, 0.257991326767245, 
0, 0, 0, 0.0825010674185945, 0.438388214283975, 0.475607967438322, 
0, 0, 0.0918431763129223, 0.782810348414715, 0, 0.027140356489928, 
0.714372864124864, 0, 0.105943373018621, 0.880952380952317, 0.554087085425494, 
0.880952380952317, 0.869688696478372, 0.880952380952317, 0.870286617759846, 
0.545000934879003, 0, 0.157440409851186, 0.629781311769757, 0, 
0.0650791287856486, 0.328314997097491, 0, 0.261152449780879, 
0, 0, 0, 0.0943073000218775, 0, 0.0266657718102841, 0.662732840432664, 
0), G.pval = c(0.722192784539483, 0.57181559190857, 0, 0.750621829528081, 
0.375942296353928, 0.127389356250858, 0, 0, 0.147733788708815, 
0.183450928299003, 0.0390632830641376, 0, 0.549953403658735, 
0.328599951401591, 0.14969927007402, 0, 0.220828773440477, 0, 
0.126152373375275, 0.168421730491711, 0.127389356250858, 0, 0.153866574122656, 
0, 0.0870457624200068, 0.034323023921186, 0.684364027213634, 
0.664519141354774, 0.425628554834276, 0.413967853764827, 0.240119846274944, 
0.181784573380147, 0, 0, 0, 0.100347285996458, 0.535776295768237, 
0.59339403849528, 0.117858951755895, 0.352174424071451, 0.218467360746297, 
0, 0.106263971342663, 0.0787084666958611, 0.089623271135999, 
0, 0.420213796221093, 0.360172339348119, 0, 0, 0.10371433122195, 
0.0556489311948595, 0.544016455726834, 0, 0.327757650097535, 
0.701654853092463, 0, 0.98888888880989, 0.310776613052784, 0.697312260993449, 
0, 0.550310363821313, 0.88511107780967, 0.0844302426488441, 0, 
0.305444079820199, 0.88847164086591, 0.357635534059943, 0.913638815430741, 
0.306779293360706, 0, 0.173956772125099, 0, 0.274395886732559, 
6.66133814775094e-16), A.pval = c(0, 0, 0.304194030897435, 0, 
0.0753668443194201, 0.189830441857244, 0.394798848037168, 0.138824358444409, 
0.0354944149384336, 0.316119467952228, 0, 0.160537678634471, 
0, 0.0768318378643974, 0.0965346549269539, 0.137157430540635, 
0.30718374617458, 0.402788415500985, 0.898637744989684, 0.865661345314947, 
0.935376000321946, 0.794109538456086, 0.945454545454541, 0.836022390442985, 
0.840137559349482, 0, 0.628555443681474, 0.776414346910142, 0, 
0.423209990319156, 0, 0.724670814420256, 0.275563862649312, 0.73104715214982, 
0.635773210854477, 0.581271551475114, 0, 0.499814947310693, 0.638811440697229, 
0.545699630344923, 0.945454545454541, 0.720455683784856, 0, 0.662890542753703, 
0.945454545454541, 0.0939936433266054, 0, 0.0224192355988172, 
0.945454545454541, 0.555921115934905, 0.678492571795739, 0, 0, 
0.207195411726833, 0, 0, 0.0465017163326933, 0.94293188452872, 
1.46846645954213e-09, 0.114875535283084, 0.051353230590052, 0, 
0.299999711168472, 0, 0.814899306000855, 0.87070290813751, 0.945454545454541, 
0.359886723725758, 0.644790348500075, 0.945454545454541, 0.168524710825441, 
0.421322400780738, 0.0208203792913779, 0, 0.321455144617), guide.position = 1:75), .Names = c("A.area", 
"C.area", "G.area", "T.area", "Tot.area", "A.perc", "C.perc", 
"G.perc", "T.perc", "base.call", "index", "guide.seq", "T.pval", 
"C.pval", "G.pval", "A.pval", "guide.position"), row.names = 277:351, class = "data.frame")
```

*Report generated using EditR v1.0.8*
