# Supplementary material for: Mechanistic and genetic basis of single-strand templated repair at Cas12a-induced DNA breaks in Chlamydomonas reinhardtii
Source: Nat Commun. 2021 Nov 19;12:6751. doi: 10.1038/s41467-021-27004-1 (PMC8604939; doi:10.1038/s41467-021-27004-1)
Supplement: Supplementary file 22 — Source Data [file 41467_2021_27004_MOESM22_ESM.zip › Source Data/EditR analysis/EditR outputs/Sense/rep1_ssODN_sense_16.html]

EditR v1.0.8 report


# EditR v1.0.8 report

- Data QA
  - Filtering data
  - Percent noise peak area
  - Base information
- Predicted editing
  - Editing bar plot
  - Editing table plot
  - Table of editing results
- For use in R

## Data QA

### Filtering data

What the data looked like prefiltering:

and the post filtering signal / noise plot:

### Percent noise peak area

### Base information

Here’s information about the signal of each base, the critical percent value where any higher value would be called as significant, and Filliben’s correlation for how well the noise was modelled by the zero adjusted gamma distribution.

| Base | Average percent signal | Average peak area | Critical percent value | model mu | Fillibens correlation |
| --- | --- | --- | --- | --- | --- |
| A | 94.49478 | 428.1471 | 4.664716 | 1.572985 | 0.9924423 |
| C | 94.10714 | 453.0000 | 4.636206 | 1.720335 | 0.9845631 |
| G | 95.00792 | 440.3929 | 7.211066 | 2.157081 | 0.9939046 |
| T | 95.18517 | 467.4545 | 6.455706 | 2.087329 | 0.9957121 |

## Predicted editing

### Editing bar plot

### Editing table plot

### Table of editing results


Here’s the entire guide region

| Sanger position | Guide position | Guide sequence | Sanger base call | Focal base | Focal base peak area | p value |  |
| --- | --- | --- | --- | --- | --- | --- | --- |
| 276 | 1 | A | A | A | 94.48 | 0.000000e+00 | \* |
| 276 | 1 | A | A | C | 1.68 | 4.113441e-01 |  |
| 276 | 1 | A | A | G | 3.12 | 1.993285e-01 |  |
| 276 | 1 | A | A | T | 0.72 | 8.056007e-01 |  |
| 277 | 2 | A | A | A | 95.50 | 0.000000e+00 | \* |
| 277 | 2 | A | A | C | 1.84 | 3.518168e-01 |  |
| 277 | 2 | A | A | G | 1.43 | 5.556378e-01 |  |
| 277 | 2 | A | A | T | 1.23 | 6.418308e-01 |  |
| 278 | 3 | G | G | A | 1.53 | 4.016624e-01 |  |
| 278 | 3 | G | G | C | 2.04 | 2.865427e-01 |  |
| 278 | 3 | G | G | G | 95.15 | 0.000000e+00 | \* |
| 278 | 3 | G | G | T | 1.28 | 6.256576e-01 |  |
| 279 | 4 | A | A | A | 95.19 | 0.000000e+00 | \* |
| 279 | 4 | A | A | C | 0.76 | 8.016046e-01 |  |
| 279 | 4 | A | A | G | 2.28 | 3.417763e-01 |  |
| 279 | 4 | A | A | T | 1.77 | 4.681974e-01 |  |
| 280 | 5 | C | C | A | 3.36 | 5.300717e-02 |  |
| 280 | 5 | C | C | C | 92.86 | 0.000000e+00 | \* |
| 280 | 5 | C | C | G | 2.10 | 3.806865e-01 |  |
| 280 | 5 | C | C | T | 1.68 | 4.956109e-01 |  |
| 281 | 6 | T | T | A | 2.86 | 9.663639e-02 |  |
| 281 | 6 | T | T | C | 1.34 | 5.542137e-01 |  |
| 281 | 6 | T | T | G | 2.29 | 3.393489e-01 |  |
| 281 | 6 | T | T | T | 93.51 | 0.000000e+00 | \* |
| 282 | 7 | G | G | A | 1.16 | 5.522447e-01 |  |
| 282 | 7 | G | G | C | 1.63 | 4.311846e-01 |  |
| 282 | 7 | G | G | G | 96.40 | 0.000000e+00 | \* |
| 282 | 7 | G | G | T | 0.81 | 7.770761e-01 |  |
| 283 | 8 | G | G | A | 1.86 | 2.923786e-01 |  |
| 283 | 8 | G | G | C | 0.84 | 7.687882e-01 |  |
| 283 | 8 | G | G | G | 96.96 | 0.000000e+00 | \* |
| 283 | 8 | G | G | T | 0.34 | 8.967628e-01 |  |
| 284 | 9 | C | C | A | 2.13 | 2.209653e-01 |  |
| 284 | 9 | C | C | C | 93.31 | 0.000000e+00 | \* |
| 284 | 9 | C | C | G | 2.13 | 3.746038e-01 |  |
| 284 | 9 | C | C | T | 2.43 | 2.996604e-01 |  |
| 285 | 10 | C | C | A | 2.66 | 1.217299e-01 |  |
| 285 | 10 | C | C | C | 93.07 | 0.000000e+00 | \* |
| 285 | 10 | C | C | G | 2.31 | 3.354048e-01 |  |
| 285 | 10 | C | C | T | 1.95 | 4.165083e-01 |  |
| 286 | 11 | A | A | A | 94.77 | 0.000000e+00 | \* |
| 286 | 11 | A | A | C | 1.43 | 5.152461e-01 |  |
| 286 | 11 | A | A | G | 3.56 | 1.473950e-01 |  |
| 286 | 11 | A | A | T | 0.24 | 9.112575e-01 |  |
| 287 | 12 | G | G | A | 2.43 | 1.591160e-01 |  |
| 287 | 12 | G | G | C | 0.66 | 8.356134e-01 |  |
| 287 | 12 | G | G | G | 95.58 | 0.000000e+00 | \* |
| 287 | 12 | G | G | T | 1.32 | 6.093947e-01 |  |
| 288 | 13 | A | A | A | 96.20 | 0.000000e+00 | \* |
| 288 | 13 | A | A | C | 1.27 | 5.853149e-01 |  |
| 288 | 13 | A | A | G | 1.52 | 5.304339e-01 |  |
| 288 | 13 | A | A | T | 1.01 | 7.132373e-01 |  |
| 289 | 14 | C | C | A | 3.61 | 3.916726e-02 |  |
| 289 | 14 | C | C | C | 94.12 | 0.000000e+00 | \* |
| 289 | 14 | C | C | G | 0.00 | 9.120879e-01 |  |
| 289 | 14 | C | C | T | 2.28 | 3.343692e-01 |  |
| 290 | 15 | C | C | A | 3.29 | 5.788959e-02 |  |
| 290 | 15 | C | C | C | 93.20 | 0.000000e+00 | \* |
| 290 | 15 | C | C | G | 1.97 | 4.105359e-01 |  |
| 290 | 15 | C | C | T | 1.54 | 5.409323e-01 |  |
| 291 | 16 | G | G | A | 2.95 | 8.678299e-02 |  |
| 291 | 16 | G | G | C | 1.90 | 3.318605e-01 |  |
| 291 | 16 | G | G | G | 94.09 | 0.000000e+00 | \* |
| 291 | 16 | G | G | T | 1.05 | 6.992738e-01 |  |
| 292 | 17 | T | T | A | 0.00 | 9.272727e-01 |  |
| 292 | 17 | T | T | C | 0.88 | 7.534878e-01 |  |
| 292 | 17 | T | T | G | 2.06 | 3.903654e-01 |  |
| 292 | 17 | T | T | T | 97.06 | 0.000000e+00 | \* |
| 293 | 18 | G | G | A | 1.00 | 6.234350e-01 |  |
| 293 | 18 | G | G | C | 1.00 | 7.017594e-01 |  |
| 293 | 18 | G | G | G | 96.99 | 0.000000e+00 | \* |
| 293 | 18 | G | G | T | 1.00 | 7.159908e-01 |  |
| 294 | 19 | T | T | A | 1.18 | 5.462225e-01 |  |
| 294 | 19 | T | T | C | 0.00 | 9.285714e-01 |  |
| 294 | 19 | T | T | G | 2.82 | 2.419643e-01 |  |
| 294 | 19 | T | T | T | 96.00 | 0.000000e+00 | \* |
| 295 | 20 | T | T | A | 0.45 | 8.560576e-01 |  |
| 295 | 20 | T | T | C | 0.00 | 9.285714e-01 |  |
| 295 | 20 | T | T | G | 2.02 | 3.999587e-01 |  |
| 295 | 20 | T | T | T | 97.53 | 0.000000e+00 | \* |
| 296 | 21 | T | T | A | 0.00 | 9.272727e-01 |  |
| 296 | 21 | T | T | C | 1.73 | 3.926281e-01 |  |
| 296 | 21 | T | T | G | 2.38 | 3.218250e-01 |  |
| 296 | 21 | T | T | T | 95.90 | 0.000000e+00 | \* |
| 297 | 22 | G | G | A | 0.16 | 9.198974e-01 |  |
| 297 | 22 | G | G | C | 1.94 | 3.196798e-01 |  |
| 297 | 22 | G | G | G | 96.94 | 0.000000e+00 | \* |
| 297 | 22 | G | G | T | 0.97 | 7.279884e-01 |  |
| 298 | 23 | T | T | A | 0.72 | 7.522276e-01 |  |
| 298 | 23 | T | T | C | 1.44 | 5.108454e-01 |  |
| 298 | 23 | T | T | G | 2.87 | 2.346123e-01 |  |
| 298 | 23 | T | T | T | 94.98 | 0.000000e+00 | \* |
| 299 | 24 | G | G | A | 1.19 | 5.411145e-01 |  |
| 299 | 24 | G | G | C | 0.99 | 7.079306e-01 |  |
| 299 | 24 | G | G | G | 95.84 | 0.000000e+00 | \* |
| 299 | 24 | G | G | T | 1.98 | 4.093242e-01 |  |
| 300 | 25 | C | C | A | 0.92 | 6.601212e-01 |  |
| 300 | 25 | C | C | C | 93.53 | 0.000000e+00 | \* |
| 300 | 25 | C | C | G | 2.77 | 2.503052e-01 |  |
| 300 | 25 | C | C | T | 2.77 | 2.334384e-01 |  |
| 301 | 26 | A | A | A | 94.59 | 0.000000e+00 | \* |
| 301 | 26 | A | A | C | 3.30 | 6.332268e-02 |  |
| 301 | 26 | A | A | G | 2.10 | 3.803986e-01 |  |
| 301 | 26 | A | A | T | 0.00 | 9.247312e-01 |  |
| 302 | 27 | C | C | A | 1.63 | 3.660798e-01 |  |
| 302 | 27 | C | C | C | 96.54 | 0.000000e+00 | \* |
| 302 | 27 | C | C | G | 0.00 | 9.120879e-01 |  |
| 302 | 27 | C | C | T | 1.83 | 4.504654e-01 |  |
| 303 | 28 | T | T | A | 1.82 | 3.043562e-01 |  |
| 303 | 28 | T | T | C | 2.63 | 1.480169e-01 |  |
| 303 | 28 | T | T | G | 0.00 | 9.120879e-01 |  |
| 303 | 28 | T | T | T | 95.56 | 0.000000e+00 | \* |
| 304 | 29 | A | A | A | 95.30 | 0.000000e+00 | \* |
| 304 | 29 | A | A | C | 2.91 | 1.049030e-01 |  |
| 304 | 29 | A | A | G | 0.00 | 9.120879e-01 |  |
| 304 | 29 | A | A | T | 1.79 | 4.630383e-01 |  |
| 305 | 30 | C | C | A | 2.13 | 2.198989e-01 |  |
| 305 | 30 | C | C | C | 94.24 | 0.000000e+00 | \* |
| 305 | 30 | C | C | G | 1.07 | 6.659316e-01 |  |
| 305 | 30 | C | C | T | 2.56 | 2.733164e-01 |  |
| 306 | 31 | A | A | A | 92.55 | 0.000000e+00 | \* |
| 306 | 31 | A | A | C | 2.87 | 1.106532e-01 |  |
| 306 | 31 | A | A | G | 3.44 | 1.605185e-01 |  |
| 306 | 31 | A | A | T | 1.15 | 6.688523e-01 |  |
| 307 | 32 | C | C | A | 1.20 | 5.350857e-01 |  |
| 307 | 32 | C | C | C | 94.47 | 0.000000e+00 | \* |
| 307 | 32 | C | C | G | 1.44 | 5.524905e-01 |  |
| 307 | 32 | C | C | T | 2.88 | 2.142739e-01 |  |
| 308 | 33 | G | G | A | 0.93 | 6.565262e-01 |  |
| 308 | 33 | G | G | C | 2.48 | 1.749624e-01 |  |
| 308 | 33 | G | G | G | 96.58 | 0.000000e+00 | \* |
| 308 | 33 | G | G | T | 0.00 | 9.247312e-01 |  |
| 309 | 34 | G | G | A | 0.00 | 9.272727e-01 |  |
| 309 | 34 | G | G | C | 0.43 | 8.973948e-01 |  |
| 309 | 34 | G | G | G | 99.15 | 0.000000e+00 | \* |
| 309 | 34 | G | G | T | 0.43 | 8.799368e-01 |  |
| 310 | 35 | G | G | A | 0.26 | 9.059792e-01 |  |
| 310 | 35 | G | G | C | 1.28 | 5.795404e-01 |  |
| 310 | 35 | G | G | G | 97.70 | 0.000000e+00 | \* |
| 310 | 35 | G | G | T | 0.77 | 7.913611e-01 |  |
| 311 | 36 | C | C | A | 2.23 | 1.969453e-01 |  |
| 311 | 36 | C | C | C | 95.25 | 0.000000e+00 | \* |
| 311 | 36 | C | C | G | 1.12 | 6.501491e-01 |  |
| 311 | 36 | C | C | T | 1.40 | 5.856341e-01 |  |
| 312 | 37 | A | A | A | 95.21 | 0.000000e+00 | \* |
| 312 | 37 | A | A | C | 3.19 | 7.322555e-02 |  |
| 312 | 37 | A | A | G | 0.80 | 7.482203e-01 |  |
| 312 | 37 | A | A | T | 0.80 | 7.820359e-01 |  |
| 313 | 38 | C | C | A | 0.93 | 6.583642e-01 |  |
| 313 | 38 | C | C | C | 95.36 | 0.000000e+00 | \* |
| 313 | 38 | C | C | G | 1.67 | 4.885067e-01 |  |
| 313 | 38 | C | C | T | 2.04 | 3.931346e-01 |  |
| 314 | 39 | C | C | A | 0.82 | 7.087748e-01 |  |
| 314 | 39 | C | C | C | 95.31 | 0.000000e+00 | \* |
| 314 | 39 | C | C | G | 1.43 | 5.564895e-01 |  |
| 314 | 39 | C | C | T | 2.45 | 2.959435e-01 |  |
| 315 | 40 | C | C | A | 1.50 | 4.135542e-01 |  |
| 315 | 40 | C | C | C | 94.65 | 0.000000e+00 | \* |
| 315 | 40 | C | C | G | 0.86 | 7.304369e-01 |  |
| 315 | 40 | C | C | T | 3.00 | 1.964690e-01 |  |
| 316 | 41 | T | T | A | 0.68 | 7.675490e-01 |  |
| 316 | 41 | T | T | C | 2.27 | 2.227602e-01 |  |
| 316 | 41 | T | T | G | 1.82 | 4.492864e-01 |  |
| 316 | 41 | T | T | T | 95.23 | 0.000000e+00 | \* |
| 317 | 42 | G | G | A | 2.20 | 2.040637e-01 |  |
| 317 | 42 | G | G | C | 2.20 | 2.409433e-01 |  |
| 317 | 42 | G | G | G | 94.13 | 0.000000e+00 | \* |
| 317 | 42 | G | G | T | 1.47 | 5.624618e-01 |  |
| 318 | 43 | A | A | A | 95.78 | 0.000000e+00 | \* |
| 318 | 43 | A | A | C | 1.54 | 4.686137e-01 |  |
| 318 | 43 | A | A | G | 2.69 | 2.642911e-01 |  |
| 318 | 43 | A | A | T | 0.00 | 9.247312e-01 |  |
| 319 | 44 | C | C | A | 1.92 | 2.736767e-01 |  |
| 319 | 44 | C | C | C | 91.67 | 0.000000e+00 | \* |
| 319 | 44 | C | C | G | 2.99 | 2.167273e-01 |  |
| 319 | 44 | C | C | T | 3.42 | 1.410893e-01 |  |
| 320 | 45 | C | C | A | 0.49 | 8.420440e-01 |  |
| 320 | 45 | C | C | C | 92.89 | 0.000000e+00 | \* |
| 320 | 45 | C | C | G | 4.66 | 6.801957e-02 |  |
| 320 | 45 | C | C | T | 1.96 | 4.146032e-01 |  |
| 321 | 46 | G | G | A | 3.35 | 5.362233e-02 |  |
| 321 | 46 | G | G | C | 3.35 | 5.941030e-02 |  |
| 321 | 46 | G | G | G | 93.02 | 0.000000e+00 | \* |
| 321 | 46 | G | G | T | 0.28 | 9.058159e-01 |  |
| 322 | 47 | A | A | A | 95.04 | 0.000000e+00 | \* |
| 322 | 47 | A | A | C | 1.20 | 6.163064e-01 |  |
| 322 | 47 | A | A | G | 1.88 | 4.335031e-01 |  |
| 322 | 47 | A | A | T | 1.88 | 4.369557e-01 |  |
| 323 | 48 | C | C | A | 2.48 | 1.509523e-01 |  |
| 323 | 48 | C | C | C | 91.83 | 0.000000e+00 | \* |
| 323 | 48 | C | C | G | 1.49 | 5.401103e-01 |  |
| 323 | 48 | C | C | T | 4.21 | 7.358708e-02 |  |
| 324 | 49 | G | G | A | 3.03 | 7.920174e-02 |  |
| 324 | 49 | G | G | C | 2.65 | 1.436112e-01 |  |
| 324 | 49 | G | G | G | 92.80 | 0.000000e+00 | \* |
| 324 | 49 | G | G | T | 1.52 | 5.472832e-01 |  |
| 325 | 50 | G | G | A | 1.04 | 6.074539e-01 |  |
| 325 | 50 | G | G | C | 1.25 | 5.935442e-01 |  |
| 325 | 50 | G | G | G | 96.88 | 0.000000e+00 | \* |
| 325 | 50 | G | G | T | 0.83 | 7.715888e-01 |  |
| 326 | 51 | C | C | A | 1.85 | 2.942309e-01 |  |
| 326 | 51 | C | C | C | 95.93 | 0.000000e+00 | \* |
| 326 | 51 | C | C | G | 2.22 | 3.537483e-01 |  |
| 326 | 51 | C | C | T | 0.00 | 9.247312e-01 |  |
| 327 | 52 | A | A | A | 96.51 | 0.000000e+00 | \* |
| 327 | 52 | A | A | C | 2.18 | 2.458569e-01 |  |
| 327 | 52 | A | A | G | 0.87 | 7.252984e-01 |  |
| 327 | 52 | A | A | T | 0.44 | 8.779922e-01 |  |
| 328 | 53 | A | A | A | 95.30 | 0.000000e+00 | \* |
| 328 | 53 | A | A | C | 0.99 | 7.079306e-01 |  |
| 328 | 53 | A | A | G | 3.22 | 1.863787e-01 |  |
| 328 | 53 | A | A | T | 0.50 | 8.650823e-01 |  |
| 329 | 54 | G | G | A | 3.87 | 2.808377e-02 |  |
| 329 | 54 | G | G | C | 1.19 | 6.190438e-01 |  |
| 329 | 54 | G | G | G | 94.64 | 0.000000e+00 | \* |
| 329 | 54 | G | G | T | 0.30 | 9.031616e-01 |  |
| 330 | 55 | A | A | A | 96.09 | 0.000000e+00 | \* |
| 330 | 55 | A | A | C | 0.00 | 9.285714e-01 |  |
| 330 | 55 | A | A | G | 2.30 | 3.375213e-01 |  |
| 330 | 55 | A | A | T | 1.61 | 5.176186e-01 |  |
| 331 | 56 | A | A | A | 96.95 | 0.000000e+00 | \* |
| 331 | 56 | A | A | C | 0.00 | 9.285714e-01 |  |
| 331 | 56 | A | A | G | 1.02 | 6.808389e-01 |  |
| 331 | 56 | A | A | T | 2.04 | 3.945040e-01 |  |
| 332 | 57 | G | G | A | 2.08 | 2.316128e-01 |  |
| 332 | 57 | G | G | C | 3.12 | 7.976419e-02 |  |
| 332 | 57 | G | G | G | 90.28 | 0.000000e+00 | \* |
| 332 | 57 | G | G | T | 4.51 | 5.668465e-02 |  |
| 333 | 58 | T | T | A | 1.17 | 5.492481e-01 |  |
| 333 | 58 | T | T | C | 0.00 | 9.285714e-01 |  |
| 333 | 58 | T | T | G | 1.17 | 6.341064e-01 |  |
| 333 | 58 | T | T | T | 97.66 | 0.000000e+00 | \* |
| 334 | 59 | T | T | A | 16.05 | 6.426746e-10 | \* |
| 334 | 59 | T | T | C | 1.72 | 3.958855e-01 |  |
| 334 | 59 | T | T | G | 1.72 | 4.752076e-01 |  |
| 334 | 59 | T | T | T | 80.52 | 0.000000e+00 | \* |
| 335 | 60 | C | C | A | 2.10 | 2.276238e-01 |  |
| 335 | 60 | C | C | C | 94.49 | 0.000000e+00 | \* |
| 335 | 60 | C | C | G | 2.36 | 3.245563e-01 |  |
| 335 | 60 | C | C | T | 1.05 | 7.009274e-01 |  |
| 336 | 61 | G | G | A | 7.32 | 2.603398e-04 | \* |
| 336 | 61 | G | G | C | 1.74 | 3.872884e-01 |  |
| 336 | 61 | G | G | G | 90.24 | 0.000000e+00 | \* |
| 336 | 61 | G | G | T | 0.70 | 8.121589e-01 |  |
| 337 | 62 | A | A | A | 94.72 | 0.000000e+00 | \* |
| 337 | 62 | A | A | C | 1.89 | 3.358872e-01 |  |
| 337 | 62 | A | A | G | 2.26 | 3.447950e-01 |  |
| 337 | 62 | A | A | T | 1.13 | 6.735483e-01 |  |
| 338 | 63 | C | C | A | 2.18 | 2.081577e-01 |  |
| 338 | 63 | C | C | C | 93.45 | 0.000000e+00 | \* |
| 338 | 63 | C | C | G | 2.18 | 3.621976e-01 |  |
| 338 | 63 | C | C | T | 2.18 | 3.568337e-01 |  |
| 339 | 64 | A | A | A | 90.17 | 0.000000e+00 | \* |
| 339 | 64 | A | A | C | 3.05 | 8.767727e-02 |  |
| 339 | 64 | A | A | G | 6.44 | 1.804517e-02 |  |
| 339 | 64 | A | A | T | 0.34 | 8.965696e-01 |  |
| 340 | 65 | G | G | A | 1.14 | 5.627961e-01 |  |
| 340 | 65 | G | G | C | 3.19 | 7.345503e-02 |  |
| 340 | 65 | G | G | G | 95.67 | 0.000000e+00 | \* |
| 340 | 65 | G | G | T | 0.00 | 9.247312e-01 |  |
| 341 | 66 | C | C | A | 0.27 | 9.025913e-01 |  |
| 341 | 66 | C | C | C | 94.26 | 0.000000e+00 | \* |
| 341 | 66 | C | C | G | 1.09 | 6.576685e-01 |  |
| 341 | 66 | C | C | T | 4.37 | 6.403326e-02 |  |
| 342 | 67 | T | T | A | 0.00 | 9.272727e-01 |  |
| 342 | 67 | T | T | C | 3.42 | 5.439713e-02 |  |
| 342 | 67 | T | T | G | 0.85 | 7.309948e-01 |  |
| 342 | 67 | T | T | T | 95.73 | 0.000000e+00 | \* |
| 343 | 68 | C | C | A | 0.25 | 9.068113e-01 |  |
| 343 | 68 | C | C | C | 96.23 | 0.000000e+00 | \* |
| 343 | 68 | C | C | G | 0.25 | 8.873243e-01 |  |
| 343 | 68 | C | C | T | 3.27 | 1.593027e-01 |  |
| 344 | 69 | C | C | A | 0.77 | 7.277595e-01 |  |
| 344 | 69 | C | C | C | 95.55 | 0.000000e+00 | \* |
| 344 | 69 | C | C | G | 0.00 | 9.120879e-01 |  |
| 344 | 69 | C | C | T | 3.68 | 1.146549e-01 |  |
| 345 | 70 | C | C | A | 0.86 | 6.870403e-01 |  |
| 345 | 70 | C | C | C | 91.93 | 0.000000e+00 | \* |
| 345 | 70 | C | C | G | 4.61 | 7.031610e-02 |  |
| 345 | 70 | C | C | T | 2.59 | 2.663910e-01 |  |
| 346 | 71 | G | G | A | 2.76 | 1.088707e-01 |  |
| 346 | 71 | G | G | C | 3.68 | 3.828293e-02 |  |
| 346 | 71 | G | G | G | 92.33 | 0.000000e+00 | \* |
| 346 | 71 | G | G | T | 1.23 | 6.418308e-01 |  |
| 347 | 72 | C | C | A | 4.44 | 1.346279e-02 |  |
| 347 | 72 | C | C | C | 89.30 | 0.000000e+00 | \* |
| 347 | 72 | C | C | G | 2.87 | 2.344207e-01 |  |
| 347 | 72 | C | C | T | 3.39 | 1.438897e-01 |  |
| 348 | 73 | G | G | A | 6.13 | 1.364004e-03 | \* |
| 348 | 73 | G | G | C | 4.91 | 6.726298e-03 | \* |
| 348 | 73 | G | G | G | 86.81 | 0.000000e+00 | \* |
| 348 | 73 | G | G | T | 2.15 | 3.658027e-01 |  |
| 349 | 74 | A | A | A | 92.44 | 0.000000e+00 | \* |
| 349 | 74 | A | A | C | 3.02 | 9.074026e-02 |  |
| 349 | 74 | A | A | G | 1.73 | 4.729008e-01 |  |
| 349 | 74 | A | A | T | 2.81 | 2.271247e-01 |  |
| 350 | 75 | C | C | A | 1.71 | 3.400895e-01 |  |
| 350 | 75 | C | C | C | 91.68 | 0.000000e+00 | \* |
| 350 | 75 | C | C | G | 1.28 | 6.007681e-01 |  |
| 350 | 75 | C | C | T | 5.33 | 2.772952e-02 |  |

## For use in R

If you want to work with the results in R, here is output that you can copy and paste in your terminal to get:

The base information:

```
structure(list(focal.base = c("A", "C", "G", "T"), avg.percsignal = c(94.4947760840782, 
94.1071421792329, 95.0079225131702, 95.1851744103145), avg.areasignal = c(428.147058823529, 
453, 440.392857142857, 467.454545454545), crit.perc.area = c(4.66471602018932, 
4.63620631648523, 7.21106585360817, 6.45570564962029), mu = c(1.57298550723568, 
1.72033487505097, 2.15708134502698, 2.08732930713495), fillibens = c(0.992442309311235, 
0.98456309578863, 0.993904632626383, 0.995712057536594)), .Names = c("focal.base", 
"avg.percsignal", "avg.areasignal", "crit.perc.area", "mu", "fillibens"
), row.names = c(NA, -4L), class = "data.frame")
```

the data.frame that contains information on the guide region:

```
structure(list(A.area = c(394, 467, 6, 752, 16, 15, 10, 11, 7, 
15, 399, 11, 760, 19, 15, 14, 0, 7, 5, 2, 0, 1, 3, 6, 4, 315, 
8, 9, 426, 10, 323, 5, 3, 0, 1, 8, 358, 5, 4, 7, 3, 12, 499, 
9, 2, 12, 556, 10, 8, 5, 5, 221, 385, 13, 418, 381, 6, 4, 56, 
8, 21, 502, 10, 266, 5, 1, 0, 1, 4, 3, 9, 17, 20, 428, 8), C.area = c(7, 
9, 8, 6, 442, 7, 14, 5, 307, 524, 6, 3, 10, 496, 425, 9, 3, 7, 
0, 0, 8, 12, 6, 5, 405, 11, 474, 13, 13, 442, 10, 393, 8, 2, 
5, 341, 12, 514, 467, 442, 10, 12, 8, 429, 379, 12, 7, 371, 7, 
6, 259, 5, 4, 4, 0, 0, 9, 0, 6, 360, 5, 10, 428, 9, 14, 345, 
16, 383, 494, 319, 12, 342, 16, 14, 430), G.area = c(13, 7, 373, 
18, 10, 12, 829, 574, 7, 13, 15, 433, 12, 0, 9, 446, 7, 676, 
12, 9, 11, 601, 12, 484, 12, 7, 0, 0, 0, 5, 12, 6, 311, 464, 
382, 4, 3, 9, 7, 4, 8, 513, 14, 14, 19, 333, 11, 6, 245, 466, 
6, 2, 13, 318, 10, 4, 260, 4, 6, 9, 259, 12, 10, 19, 420, 4, 
4, 1, 0, 16, 301, 11, 283, 8, 6), T.area = c(3, 6, 5, 14, 8, 
490, 7, 2, 8, 11, 1, 6, 8, 12, 7, 5, 330, 7, 408, 435, 444, 6, 
397, 10, 12, 0, 9, 473, 8, 12, 4, 12, 0, 2, 3, 5, 3, 11, 12, 
14, 419, 8, 0, 16, 8, 1, 11, 17, 4, 4, 0, 1, 2, 1, 7, 8, 13, 
334, 281, 4, 2, 6, 10, 1, 0, 16, 448, 13, 19, 9, 4, 13, 7, 13, 
25), Tot.area = c(417, 489, 392, 790, 476, 524, 860, 592, 329, 
563, 421, 453, 790, 527, 456, 474, 340, 697, 425, 446, 463, 620, 
418, 505, 433, 333, 491, 495, 447, 469, 349, 416, 322, 468, 391, 
358, 376, 539, 490, 467, 440, 545, 521, 468, 408, 358, 585, 404, 
264, 481, 270, 229, 404, 336, 435, 393, 288, 342, 349, 381, 287, 
530, 458, 295, 439, 366, 468, 398, 517, 347, 326, 383, 326, 463, 
469), A.perc = c(94.484412470024, 95.5010224948875, 1.53061224489796, 
95.1898734177215, 3.36134453781513, 2.86259541984733, 1.16279069767442, 
1.85810810810811, 2.12765957446809, 2.66429840142096, 94.7743467933492, 
2.42825607064018, 96.2025316455696, 3.60531309297913, 3.28947368421053, 
2.9535864978903, 0, 1.00430416068867, 1.17647058823529, 0.448430493273543, 
0, 0.161290322580645, 0.717703349282297, 1.18811881188119, 0.92378752886836, 
94.5945945945946, 1.62932790224033, 1.81818181818182, 95.3020134228188, 
2.13219616204691, 92.5501432664756, 1.20192307692308, 0.93167701863354, 
0, 0.255754475703325, 2.23463687150838, 95.2127659574468, 0.927643784786642, 
0.816326530612245, 1.49892933618844, 0.681818181818182, 2.20183486238532, 
95.7773512476008, 1.92307692307692, 0.490196078431373, 3.35195530726257, 
95.042735042735, 2.47524752475248, 3.03030303030303, 1.03950103950104, 
1.85185185185185, 96.5065502183406, 95.2970297029703, 3.86904761904762, 
96.0919540229885, 96.9465648854962, 2.08333333333333, 1.16959064327485, 
16.0458452722063, 2.0997375328084, 7.31707317073171, 94.7169811320755, 
2.18340611353712, 90.1694915254237, 1.13895216400911, 0.273224043715847, 
0, 0.251256281407035, 0.773694390715667, 0.864553314121037, 2.76073619631902, 
4.43864229765013, 6.13496932515337, 92.4406047516199, 1.70575692963753
), C.perc = c(1.67865707434053, 1.84049079754601, 2.04081632653061, 
0.759493670886076, 92.8571428571429, 1.33587786259542, 1.62790697674419, 
0.844594594594595, 93.3130699088146, 93.0728241563055, 1.42517814726841, 
0.662251655629139, 1.26582278481013, 94.1176470588235, 93.2017543859649, 
1.89873417721519, 0.882352941176471, 1.00430416068867, 0, 0, 
1.72786177105832, 1.93548387096774, 1.43540669856459, 0.99009900990099, 
93.5334872979215, 3.3033033033033, 96.5376782077393, 2.62626262626263, 
2.9082774049217, 94.2430703624733, 2.86532951289398, 94.4711538461538, 
2.48447204968944, 0.427350427350427, 1.27877237851662, 95.2513966480447, 
3.19148936170213, 95.3617810760668, 95.3061224489796, 94.6466809421841, 
2.27272727272727, 2.20183486238532, 1.53550863723608, 91.6666666666667, 
92.8921568627451, 3.35195530726257, 1.1965811965812, 91.8316831683168, 
2.65151515151515, 1.24740124740125, 95.9259259259259, 2.18340611353712, 
0.99009900990099, 1.19047619047619, 0, 0, 3.125, 0, 1.71919770773639, 
94.488188976378, 1.74216027874564, 1.88679245283019, 93.4497816593886, 
3.05084745762712, 3.18906605922551, 94.2622950819672, 3.41880341880342, 
96.2311557788945, 95.5512572533849, 91.9308357348703, 3.68098159509202, 
89.2950391644909, 4.9079754601227, 3.02375809935205, 91.6844349680171
), G.perc = c(3.11750599520384, 1.43149284253579, 95.1530612244898, 
2.27848101265823, 2.10084033613445, 2.29007633587786, 96.3953488372093, 
96.9594594594595, 2.12765957446809, 2.30905861456483, 3.56294536817102, 
95.5849889624724, 1.51898734177215, 0, 1.97368421052632, 94.0928270042194, 
2.05882352941176, 96.987087517934, 2.82352941176471, 2.01793721973094, 
2.37580993520518, 96.9354838709677, 2.87081339712919, 95.8415841584158, 
2.77136258660508, 2.1021021021021, 0, 0, 0, 1.06609808102345, 
3.43839541547278, 1.44230769230769, 96.583850931677, 99.1452991452991, 
97.6982097186701, 1.11731843575419, 0.797872340425532, 1.66975881261596, 
1.42857142857143, 0.856531049250535, 1.81818181818182, 94.1284403669725, 
2.68714011516315, 2.99145299145299, 4.65686274509804, 93.0167597765363, 
1.88034188034188, 1.48514851485149, 92.8030303030303, 96.8814968814969, 
2.22222222222222, 0.873362445414847, 3.21782178217822, 94.6428571428571, 
2.29885057471264, 1.01781170483461, 90.2777777777778, 1.16959064327485, 
1.71919770773639, 2.36220472440945, 90.2439024390244, 2.26415094339623, 
2.18340611353712, 6.44067796610169, 95.6719817767654, 1.09289617486339, 
0.854700854700855, 0.251256281407035, 0, 4.61095100864553, 92.3312883435583, 
2.87206266318538, 86.8098159509202, 1.72786177105832, 1.27931769722815
), T.perc = c(0.719424460431655, 1.22699386503067, 1.27551020408163, 
1.77215189873418, 1.68067226890756, 93.5114503816794, 0.813953488372093, 
0.337837837837838, 2.43161094224924, 1.9538188277087, 0.237529691211401, 
1.32450331125828, 1.0126582278481, 2.27703984819734, 1.53508771929825, 
1.05485232067511, 97.0588235294118, 1.00430416068867, 96, 97.5336322869955, 
95.8963282937365, 0.967741935483871, 94.9760765550239, 1.98019801980198, 
2.77136258660508, 0, 1.83299389002037, 95.5555555555556, 1.78970917225951, 
2.55863539445629, 1.14613180515759, 2.88461538461538, 0, 0.427350427350427, 
0.767263427109974, 1.39664804469274, 0.797872340425532, 2.04081632653061, 
2.44897959183673, 2.99785867237687, 95.2272727272727, 1.46788990825688, 
0, 3.41880341880342, 1.96078431372549, 0.279329608938547, 1.88034188034188, 
4.20792079207921, 1.51515151515152, 0.831600831600832, 0, 0.436681222707424, 
0.495049504950495, 0.297619047619048, 1.60919540229885, 2.03562340966921, 
4.51388888888889, 97.6608187134503, 80.5157593123209, 1.0498687664042, 
0.696864111498258, 1.13207547169811, 2.18340611353712, 0.338983050847458, 
0, 4.37158469945355, 95.7264957264957, 3.26633165829146, 3.67504835589942, 
2.59365994236311, 1.22699386503067, 3.39425587467363, 2.14723926380368, 
2.80777537796976, 5.33049040511727), base.call = c("A", "A", 
"G", "A", "C", "T", "G", "G", "C", "C", "A", "G", "A", "C", "C", 
"G", "T", "G", "T", "T", "T", "G", "T", "G", "C", "A", "C", "T", 
"A", "C", "A", "C", "G", "G", "G", "C", "A", "C", "C", "C", "T", 
"G", "A", "C", "C", "G", "A", "C", "G", "G", "C", "A", "A", "G", 
"A", "A", "G", "T", "T", "C", "G", "A", "C", "A", "G", "C", "T", 
"C", "C", "C", "G", "C", "G", "A", "C"), index = 276:350, guide.seq = c("A", 
"A", "G", "A", "C", "T", "G", "G", "C", "C", "A", "G", "A", "C", 
"C", "G", "T", "G", "T", "T", "T", "G", "T", "G", "C", "A", "C", 
"T", "A", "C", "A", "C", "G", "G", "G", "C", "A", "C", "C", "C", 
"T", "G", "A", "C", "C", "G", "A", "C", "G", "G", "C", "A", "A", 
"G", "A", "A", "G", "T", "T", "C", "G", "A", "C", "A", "G", "C", 
"T", "C", "C", "C", "G", "C", "G", "A", "C"), T.pval = c(0.805600727556002, 
0.641830806109078, 0.625657590647016, 0.468197404736581, 0.495610938236183, 
0, 0.777076099562001, 0.89676283422072, 0.299660440200043, 0.416508348978706, 
0.91125753708036, 0.60939465101949, 0.713237346103757, 0.334369246648943, 
0.540932292033562, 0.699273816398051, 0, 0.715990812303441, 0, 
0, 0, 0.72798838744884, 0, 0.409324236360065, 0.233438373932121, 
0.924731182795699, 0.450465418866842, 0, 0.463038297508791, 0.273316383172381, 
0.668852300134187, 0.21427389910305, 0.924731182795699, 0.879936839954413, 
0.791361149954678, 0.58563411125822, 0.782035856376316, 0.393134628600592, 
0.295943453910729, 0.196468997585179, 0, 0.562461756158136, 0.924731182795699, 
0.141089273882588, 0.414603244832574, 0.905815912679438, 0.436955677386021, 
0.0735870837634877, 0.547283248489153, 0.7715887638417, 0.924731182795699, 
0.87799217427244, 0.865082331584768, 0.903161561058265, 0.517618623395065, 
0.394503982865341, 0.0566846508711927, 0, 0, 0.700927414207321, 
0.812158904167613, 0.673548253342024, 0.356833698974173, 0.896569623616523, 
0.924731182795699, 0.0640332630175918, 0, 0.159302739603193, 
0.114654886704701, 0.266390952895471, 0.641830806109078, 0.143889676839403, 
0.365802742007512, 0.227124702081387, 0.0277295185431967), C.pval = c(0.411344141391084, 
0.351816799457327, 0.286542676588527, 0.801604645387985, 0, 0.554213675908782, 
0.431184593501227, 0.768788174020979, 0, 0, 0.515246063693439, 
0.835613418286963, 0.585314869626743, 0, 0, 0.331860456375591, 
0.75348782382373, 0.701759441592782, 0.928571428571429, 0.928571428571429, 
0.392628068523792, 0.319679752124952, 0.510845414150445, 0.707930551098543, 
0, 0.0633226807874566, 0, 0.148016903910708, 0.104903048177301, 
0, 0.110653163251815, 0, 0.174962434643962, 0.89739483619724, 
0.579540407999979, 0, 0.0732255529533764, 0, 0, 0, 0.222760194331186, 
0.240943278234617, 0.468613714257827, 0, 0, 0.0594102967524824, 
0.616306373457213, 0, 0.143611223548242, 0.59354419906491, 0, 
0.24585691409455, 0.707930551098543, 0.619043797216537, 0.928571428571429, 
0.928571428571429, 0.0797641860305106, 0.928571428571429, 0.395885517576232, 
0, 0.387288401546166, 0.335887226543705, 0, 0.0876772681804062, 
0.0734550276779852, 0, 0.0543971302081143, 0, 0, 0, 0.0382829272936015, 
0, 0.0067262980416094, 0.0907402618004824, 0), G.pval = c(0.199328539027282, 
0.555637807536568, 0, 0.34177626456668, 0.380686466870414, 0.339348940091052, 
0, 0, 0.374603752528208, 0.335404792466264, 0.147395008623377, 
0, 0.530433868372073, 0.912087912058151, 0.410535875218611, 0, 
0.39036535138585, 0, 0.241964327290174, 0.39995867649061, 0.321824963779369, 
0, 0.234612324616665, 0, 0.250305237124321, 0.380398628523568, 
0.912087912058151, 0.912087912058151, 0.912087912058151, 0.665931644434122, 
0.160518526630176, 0.552490478859176, 0, 0, 0, 0.65014909360715, 
0.748220276065056, 0.488506742154404, 0.556489480023895, 0.73043689090014, 
0.449286404841432, 0, 0.264291120196938, 0.216727295364456, 0.0680195691786013, 
0, 0.433503092818682, 0.540110310327898, 0, 0, 0.353748259383942, 
0.72529844245975, 0.186378748191624, 0, 0.337521270746694, 0.680838946120078, 
0, 0.63410639841273, 0.475207584270506, 0.324556323937277, 0, 
0.344795014397035, 0.362197631159479, 0.0180451745287697, 0, 
0.657668452610044, 0.730994789974463, 0.887324303730871, 0.912087912058151, 
0.0703161023323586, 0, 0.234420732757205, 0, 0.472900780442768, 
0.600768089356713), A.pval = c(0, 0, 0.401662411255149, 0, 0.0530071682986852, 
0.0966363897779765, 0.552244743581013, 0.292378568842376, 0.220965266493863, 
0.121729865088841, 0, 0.159116033043965, 0, 0.0391672636145743, 
0.0578895857272048, 0.0867829880408921, 0.927272727272727, 0.623434969563357, 
0.546222474548858, 0.856057579740754, 0.927272727272727, 0.919897416347101, 
0.752227609618142, 0.541114493352752, 0.660121206966087, 0, 0.366079820852399, 
0.30435622845138, 0, 0.219898915893311, 0, 0.535085735111306, 
0.656526170112542, 0.927272727272727, 0.905979167809476, 0.196945263749917, 
0, 0.658364157134927, 0.708774750986477, 0.413554216269418, 0.767549020417483, 
0.204063661368319, 0, 0.273676691270825, 0.842044011715229, 0.0536223301364878, 
0, 0.150952343861821, 0.0792017385251913, 0.607453902515522, 
0.294230909837614, 0, 0, 0.0280837749682024, 0, 0, 0.231612794046905, 
0.549248128413976, 6.42674580220159e-10, 0.227623811512276, 0.000260339762098827, 
0, 0.208157661813635, 0, 0.562796082310458, 0.902591311785169, 
0.927272727272727, 0.906811267007464, 0.727759545201818, 0.687040313289519, 
0.108870670867873, 0.0134627882506466, 0.00136400397237479, 0, 
0.340089543430979), guide.position = 1:75), .Names = c("A.area", 
"C.area", "G.area", "T.area", "Tot.area", "A.perc", "C.perc", 
"G.perc", "T.perc", "base.call", "index", "guide.seq", "T.pval", 
"C.pval", "G.pval", "A.pval", "guide.position"), row.names = 276:350, class = "data.frame")
```

*Report generated using EditR v1.0.8*
