# Supplementary material for: Mechanistic and genetic basis of single-strand templated repair at Cas12a-induced DNA breaks in Chlamydomonas reinhardtii
Source: Nat Commun. 2021 Nov 19;12:6751. doi: 10.1038/s41467-021-27004-1 (PMC8604939; doi:10.1038/s41467-021-27004-1)
Supplement: Supplementary file 22 — Source Data [file 41467_2021_27004_MOESM22_ESM.zip › Source Data/EditR analysis/EditR outputs/Sense/rep1_ssODN_sense_-16.html]

EditR v1.0.8 report


# EditR v1.0.8 report

- Data QA
  - Filtering data
  - Percent noise peak area
  - Base information
- Predicted editing
  - Editing bar plot
  - Editing table plot
  - Table of editing results
- For use in R

## Data QA

### Filtering data

What the data looked like prefiltering:

and the post filtering signal / noise plot:

### Percent noise peak area

### Base information

Here’s information about the signal of each base, the critical percent value where any higher value would be called as significant, and Filliben’s correlation for how well the noise was modelled by the zero adjusted gamma distribution.

| Base | Average percent signal | Average peak area | Critical percent value | model mu | Fillibens correlation |
| --- | --- | --- | --- | --- | --- |
| A | 93.99020 | 419.6571 | 5.246361 | 1.732136 | 0.9850048 |
| C | 93.41286 | 436.6818 | 5.121627 | 1.713003 | 0.9835717 |
| G | 94.74740 | 437.7719 | 9.243331 | 2.452893 | 0.9942512 |
| T | 95.65124 | 468.6047 | 7.500273 | 2.304165 | 0.9891151 |

## Predicted editing

### Editing bar plot

### Editing table plot

### Table of editing results


Here’s the entire guide region

| Sanger position | Guide position | Guide sequence | Sanger base call | Focal base | Focal base peak area | p value |  |
| --- | --- | --- | --- | --- | --- | --- | --- |
| 274 | 1 | A | A | A | 95.42 | 0.0000000000 | \* |
| 274 | 1 | A | A | C | 1.45 | 0.4614493566 |  |
| 274 | 1 | A | A | G | 2.17 | 0.3900767454 |  |
| 274 | 1 | A | A | T | 0.96 | 0.7387265507 |  |
| 275 | 2 | A | A | A | 94.88 | 0.0000000000 | \* |
| 275 | 2 | A | A | C | 1.43 | 0.4655564138 |  |
| 275 | 2 | A | A | G | 2.46 | 0.3405465008 |  |
| 275 | 2 | A | A | T | 1.23 | 0.6603978049 |  |
| 276 | 3 | G | G | A | 1.77 | 0.3420267865 |  |
| 276 | 3 | G | G | C | 1.26 | 0.5298946708 |  |
| 276 | 3 | G | G | G | 95.71 | 0.0000000000 | \* |
| 276 | 3 | G | G | T | 1.26 | 0.6506106829 |  |
| 277 | 4 | A | A | A | 96.96 | 0.0000000000 | \* |
| 277 | 4 | A | A | C | 0.79 | 0.7151634983 |  |
| 277 | 4 | A | A | G | 0.00 | 0.8777777778 |  |
| 277 | 4 | A | A | T | 2.25 | 0.3886669087 |  |
| 278 | 5 | C | C | A | 2.70 | 0.1479637214 |  |
| 278 | 5 | C | C | C | 95.06 | 0.0000000000 | \* |
| 278 | 5 | C | C | G | 0.00 | 0.8777777778 |  |
| 278 | 5 | C | C | T | 2.25 | 0.3890004399 |  |
| 279 | 6 | T | T | A | 2.97 | 0.1134673512 |  |
| 279 | 6 | T | T | C | 1.11 | 0.5884929620 |  |
| 279 | 6 | T | T | G | 2.78 | 0.2917933928 |  |
| 279 | 6 | T | T | T | 93.14 | 0.0000000000 | \* |
| 280 | 7 | G | G | A | 0.95 | 0.6197022719 |  |
| 280 | 7 | G | G | C | 1.90 | 0.3147004516 |  |
| 280 | 7 | G | G | G | 96.31 | 0.0000000000 | \* |
| 280 | 7 | G | G | T | 0.83 | 0.7763987846 |  |
| 281 | 8 | G | G | A | 2.11 | 0.2559760838 |  |
| 281 | 8 | G | G | C | 1.05 | 0.6126334389 |  |
| 281 | 8 | G | G | G | 95.96 | 0.0000000000 | \* |
| 281 | 8 | G | G | T | 0.88 | 0.7637036015 |  |
| 282 | 9 | C | C | A | 2.71 | 0.1459513496 |  |
| 282 | 9 | C | C | C | 91.87 | 0.0000000000 | \* |
| 282 | 9 | C | C | G | 1.81 | 0.4599158527 |  |
| 282 | 9 | C | C | T | 3.61 | 0.1654178793 |  |
| 283 | 10 | C | C | A | 2.94 | 0.1171917492 |  |
| 283 | 10 | C | C | C | 93.76 | 0.0000000000 | \* |
| 283 | 10 | C | C | G | 1.47 | 0.5338742150 |  |
| 283 | 10 | C | C | T | 1.83 | 0.4894491231 |  |
| 284 | 11 | A | A | A | 94.12 | 0.0000000000 | \* |
| 284 | 11 | A | A | C | 1.47 | 0.4525485327 |  |
| 284 | 11 | A | A | G | 2.70 | 0.3042215431 |  |
| 284 | 11 | A | A | T | 1.72 | 0.5212516745 |  |
| 285 | 12 | G | G | A | 2.86 | 0.1258315365 |  |
| 285 | 12 | G | G | C | 2.20 | 0.2390811503 |  |
| 285 | 12 | G | G | G | 94.05 | 0.0000000000 | \* |
| 285 | 12 | G | G | T | 0.88 | 0.7626015046 |  |
| 286 | 13 | A | A | A | 95.63 | 0.0000000000 | \* |
| 286 | 13 | A | A | C | 0.79 | 0.7151634983 |  |
| 286 | 13 | A | A | G | 1.32 | 0.5678844939 |  |
| 286 | 13 | A | A | T | 2.25 | 0.3886669087 |  |
| 287 | 14 | C | C | A | 3.45 | 0.0694023483 |  |
| 287 | 14 | C | C | C | 93.47 | 0.0000000000 | \* |
| 287 | 14 | C | C | G | 0.19 | 0.8495260040 |  |
| 287 | 14 | C | C | T | 2.88 | 0.2658885879 |  |
| 288 | 15 | C | C | A | 2.73 | 0.1427958569 |  |
| 288 | 15 | C | C | C | 92.94 | 0.0000000000 | \* |
| 288 | 15 | C | C | G | 1.82 | 0.4568139212 |  |
| 288 | 15 | C | C | T | 2.51 | 0.3341430657 |  |
| 289 | 16 | G | G | A | 2.17 | 0.2417956059 |  |
| 289 | 16 | G | G | C | 1.52 | 0.4356564640 |  |
| 289 | 16 | G | G | G | 94.36 | 0.0000000000 | \* |
| 289 | 16 | G | G | T | 1.95 | 0.4592776782 |  |
| 290 | 17 | T | T | A | 0.00 | 0.8532110092 |  |
| 290 | 17 | T | T | C | 2.05 | 0.2747892898 |  |
| 290 | 17 | T | T | G | 2.64 | 0.3125977652 |  |
| 290 | 17 | T | T | T | 95.31 | 0.0000000000 | \* |
| 291 | 18 | G | G | A | 1.75 | 0.3472680899 |  |
| 291 | 18 | G | G | C | 1.02 | 0.6255218598 |  |
| 291 | 18 | G | G | G | 95.63 | 0.0000000000 | \* |
| 291 | 18 | G | G | T | 1.60 | 0.5521792583 |  |
| 292 | 19 | T | T | A | 1.15 | 0.5460931566 |  |
| 292 | 19 | T | T | C | 0.00 | 0.8941176471 |  |
| 292 | 19 | T | T | G | 2.30 | 0.3671632523 |  |
| 292 | 19 | T | T | T | 96.55 | 0.0000000000 | \* |
| 293 | 20 | T | T | A | 0.68 | 0.7190221595 |  |
| 293 | 20 | T | T | C | 0.00 | 0.8941176471 |  |
| 293 | 20 | T | T | G | 0.00 | 0.8777777778 |  |
| 293 | 20 | T | T | T | 99.32 | 0.0000000000 | \* |
| 294 | 21 | T | T | A | 0.00 | 0.8532110092 |  |
| 294 | 21 | T | T | C | 1.91 | 0.3112498636 |  |
| 294 | 21 | T | T | G | 3.19 | 0.2392235417 |  |
| 294 | 21 | T | T | T | 94.89 | 0.0000000000 | \* |
| 295 | 22 | G | G | A | 0.16 | 0.8452585470 |  |
| 295 | 22 | G | G | C | 1.48 | 0.4482371344 |  |
| 295 | 22 | G | G | G | 97.36 | 0.0000000000 | \* |
| 295 | 22 | G | G | T | 0.99 | 0.7315495898 |  |
| 296 | 23 | T | T | A | 0.24 | 0.8350786502 |  |
| 296 | 23 | T | T | C | 0.00 | 0.8941176471 |  |
| 296 | 23 | T | T | G | 3.39 | 0.2169493510 |  |
| 296 | 23 | T | T | T | 96.37 | 0.0000000000 | \* |
| 297 | 24 | G | G | A | 0.40 | 0.8027407443 |  |
| 297 | 24 | G | G | C | 1.20 | 0.5542214937 |  |
| 297 | 24 | G | G | G | 96.60 | 0.0000000000 | \* |
| 297 | 24 | G | G | T | 1.80 | 0.4986334847 |  |
| 298 | 25 | C | C | A | 1.41 | 0.4553247770 |  |
| 298 | 25 | C | C | C | 92.51 | 0.0000000000 | \* |
| 298 | 25 | C | C | G | 2.11 | 0.4012073633 |  |
| 298 | 25 | C | C | T | 3.98 | 0.1292839314 |  |
| 299 | 26 | A | A | A | 93.69 | 0.0000000000 | \* |
| 299 | 26 | A | A | C | 2.70 | 0.1466469199 |  |
| 299 | 26 | A | A | G | 2.10 | 0.4022479602 |  |
| 299 | 26 | A | A | T | 1.50 | 0.5810290520 |  |
| 300 | 27 | C | C | A | 1.36 | 0.4695517746 |  |
| 300 | 27 | C | C | C | 85.45 | 0.0000000000 | \* |
| 300 | 27 | C | C | G | 10.45 | 0.0051633573 | \* |
| 300 | 27 | C | C | T | 2.73 | 0.2921131739 |  |
| 301 | 28 | T | T | A | 1.55 | 0.4080127422 |  |
| 301 | 28 | T | T | C | 1.99 | 0.2906526259 |  |
| 301 | 28 | T | T | G | 0.00 | 0.8777777778 |  |
| 301 | 28 | T | T | T | 96.46 | 0.0000000000 | \* |
| 302 | 29 | A | A | A | 96.39 | 0.0000000000 | \* |
| 302 | 29 | A | A | C | 1.93 | 0.3077123228 |  |
| 302 | 29 | A | A | G | 0.00 | 0.8777777778 |  |
| 302 | 29 | A | A | T | 1.69 | 0.5291421276 |  |
| 303 | 30 | C | C | A | 2.00 | 0.2796828720 |  |
| 303 | 30 | C | C | C | 94.88 | 0.0000000000 | \* |
| 303 | 30 | C | C | G | 0.00 | 0.8777777778 |  |
| 303 | 30 | C | C | T | 3.12 | 0.2286261136 |  |
| 304 | 31 | A | A | A | 93.31 | 0.0000000000 | \* |
| 304 | 31 | A | A | C | 2.91 | 0.1190204550 |  |
| 304 | 31 | A | A | G | 3.78 | 0.1787062530 |  |
| 304 | 31 | A | A | T | 0.00 | 0.9255319149 |  |
| 305 | 32 | C | C | A | 1.19 | 0.5321133760 |  |
| 305 | 32 | C | C | C | 92.87 | 0.0000000000 | \* |
| 305 | 32 | C | C | G | 2.61 | 0.3165713290 |  |
| 305 | 32 | C | C | T | 3.33 | 0.2000214604 |  |
| 306 | 33 | G | G | A | 0.62 | 0.7379167287 |  |
| 306 | 33 | G | G | C | 1.86 | 0.3257842703 |  |
| 306 | 33 | G | G | G | 97.20 | 0.0000000000 | \* |
| 306 | 33 | G | G | T | 0.31 | 0.8984154715 |  |
| 307 | 34 | G | G | A | 0.43 | 0.7955437109 |  |
| 307 | 34 | G | G | C | 0.21 | 0.8814205818 |  |
| 307 | 34 | G | G | G | 99.14 | 0.0000000000 | \* |
| 307 | 34 | G | G | T | 0.21 | 0.9120411884 |  |
| 308 | 35 | G | G | A | 1.02 | 0.5958354489 |  |
| 308 | 35 | G | G | C | 1.78 | 0.3513551594 |  |
| 308 | 35 | G | G | G | 96.19 | 0.0000000000 | \* |
| 308 | 35 | G | G | T | 1.02 | 0.7237142966 |  |
| 309 | 36 | C | C | A | 3.23 | 0.0876943236 |  |
| 309 | 36 | C | C | C | 93.26 | 0.0000000000 | \* |
| 309 | 36 | C | C | G | 1.17 | 0.6043223637 |  |
| 309 | 36 | C | C | T | 2.35 | 0.3672743930 |  |
| 310 | 37 | A | A | A | 93.75 | 0.0000000000 | \* |
| 310 | 37 | A | A | C | 3.53 | 0.0612065712 |  |
| 310 | 37 | A | A | G | 0.27 | 0.8325479199 |  |
| 310 | 37 | A | A | T | 2.45 | 0.3463201312 |  |
| 311 | 38 | C | C | A | 1.13 | 0.5524733767 |  |
| 311 | 38 | C | C | C | 94.72 | 0.0000000000 | \* |
| 311 | 38 | C | C | G | 0.38 | 0.8079360029 |  |
| 311 | 38 | C | C | T | 3.77 | 0.1487478146 |  |
| 312 | 39 | C | C | A | 1.25 | 0.5095751134 |  |
| 312 | 39 | C | C | C | 94.58 | 0.0000000000 | \* |
| 312 | 39 | C | C | G | 0.83 | 0.6909570616 |  |
| 312 | 39 | C | C | T | 3.33 | 0.1989943054 |  |
| 313 | 40 | C | C | A | 1.55 | 0.4080127422 |  |
| 313 | 40 | C | C | C | 94.91 | 0.0000000000 | \* |
| 313 | 40 | C | C | G | 0.22 | 0.8434978897 |  |
| 313 | 40 | C | C | T | 3.32 | 0.2009114623 |  |
| 314 | 41 | T | T | A | 0.00 | 0.8532110092 |  |
| 314 | 41 | T | T | C | 1.51 | 0.4379498537 |  |
| 314 | 41 | T | T | G | 2.59 | 0.3197604463 |  |
| 314 | 41 | T | T | T | 95.90 | 0.0000000000 | \* |
| 315 | 42 | G | G | A | 3.25 | 0.0851403892 |  |
| 315 | 42 | G | G | C | 1.08 | 0.5997132735 |  |
| 315 | 42 | G | G | G | 93.85 | 0.0000000000 | \* |
| 315 | 42 | G | G | T | 1.81 | 0.4964329409 |  |
| 316 | 43 | A | A | A | 95.73 | 0.0000000000 | \* |
| 316 | 43 | A | A | C | 1.75 | 0.3602442400 |  |
| 316 | 43 | A | A | G | 2.52 | 0.3301884554 |  |
| 316 | 43 | A | A | T | 0.00 | 0.9255319149 |  |
| 317 | 44 | C | C | A | 1.92 | 0.3011026021 |  |
| 317 | 44 | C | C | C | 92.96 | 0.0000000000 | \* |
| 317 | 44 | C | C | G | 1.92 | 0.4373357484 |  |
| 317 | 44 | C | C | T | 3.20 | 0.2171603911 |  |
| 318 | 45 | C | C | A | 1.45 | 0.4392904576 |  |
| 318 | 45 | C | C | C | 91.77 | 0.0000000000 | \* |
| 318 | 45 | C | C | G | 3.87 | 0.1703774339 |  |
| 318 | 45 | C | C | T | 2.91 | 0.2615198564 |  |
| 319 | 46 | G | G | A | 4.21 | 0.0311820023 |  |
| 319 | 46 | G | G | C | 2.25 | 0.2292272727 |  |
| 319 | 46 | G | G | G | 92.98 | 0.0000000000 | \* |
| 319 | 46 | G | G | T | 0.56 | 0.8473656542 |  |
| 320 | 47 | A | A | A | 95.72 | 0.0000000000 | \* |
| 320 | 47 | A | A | C | 1.20 | 0.5547577572 |  |
| 320 | 47 | A | A | G | 1.03 | 0.6409050516 |  |
| 320 | 47 | A | A | T | 2.05 | 0.4339348794 |  |
| 321 | 48 | C | C | A | 3.24 | 0.0862769245 |  |
| 321 | 48 | C | C | C | 92.02 | 0.0000000000 | \* |
| 321 | 48 | C | C | G | 1.25 | 0.5861855147 |  |
| 321 | 48 | C | C | T | 3.49 | 0.1794542836 |  |
| 322 | 49 | G | G | A | 3.80 | 0.0483264260 |  |
| 322 | 49 | G | G | C | 1.90 | 0.3150797299 |  |
| 322 | 49 | G | G | G | 94.30 | 0.0000000000 | \* |
| 322 | 49 | G | G | T | 0.00 | 0.9255319149 |  |
| 323 | 50 | G | G | A | 1.69 | 0.3630771892 |  |
| 323 | 50 | G | G | C | 1.48 | 0.4481132558 |  |
| 323 | 50 | G | G | G | 96.61 | 0.0000000000 | \* |
| 323 | 50 | G | G | T | 0.21 | 0.9123120116 |  |
| 324 | 51 | C | C | A | 2.57 | 0.1664396989 |  |
| 324 | 51 | C | C | C | 93.38 | 0.0000000000 | \* |
| 324 | 51 | C | C | G | 1.84 | 0.4535615751 |  |
| 324 | 51 | C | C | T | 2.21 | 0.3983544384 |  |
| 325 | 52 | A | A | A | 97.03 | 0.0000000000 | \* |
| 325 | 52 | A | A | C | 1.27 | 0.5266008844 |  |
| 325 | 52 | A | A | G | 0.42 | 0.7966014959 |  |
| 325 | 52 | A | A | T | 1.27 | 0.6480841589 |  |
| 326 | 53 | A | A | A | 93.17 | 0.0000000000 | \* |
| 326 | 53 | A | A | C | 0.24 | 0.8771687027 |  |
| 326 | 53 | A | A | G | 4.63 | 0.1157709551 |  |
| 326 | 53 | A | A | T | 1.95 | 0.4595441993 |  |
| 327 | 54 | G | G | A | 5.13 | 0.0114150707 |  |
| 327 | 54 | G | G | C | 0.28 | 0.8703177641 |  |
| 327 | 54 | G | G | G | 93.73 | 0.0000000000 | \* |
| 327 | 54 | G | G | T | 0.85 | 0.7700930712 |  |
| 328 | 55 | A | A | A | 96.85 | 0.0000000000 | \* |
| 328 | 55 | A | A | C | 0.00 | 0.8941176471 |  |
| 328 | 55 | A | A | G | 1.80 | 0.4618714670 |  |
| 328 | 55 | A | A | T | 1.35 | 0.6254032170 |  |
| 329 | 56 | A | A | A | 95.30 | 0.0000000000 | \* |
| 329 | 56 | A | A | C | 0.00 | 0.8941176471 |  |
| 329 | 56 | A | A | G | 2.09 | 0.4047221170 |  |
| 329 | 56 | A | A | T | 2.61 | 0.3136091199 |  |
| 330 | 57 | G | G | A | 3.08 | 0.1013274705 |  |
| 330 | 57 | G | G | C | 1.03 | 0.6227254081 |  |
| 330 | 57 | G | G | G | 91.10 | 0.0000000000 | \* |
| 330 | 57 | G | G | T | 4.79 | 0.0735328699 |  |
| 331 | 58 | T | T | A | 0.00 | 0.8532110092 |  |
| 331 | 58 | T | T | C | 0.00 | 0.8941176471 |  |
| 331 | 58 | T | T | G | 2.02 | 0.4182035780 |  |
| 331 | 58 | T | T | T | 97.98 | 0.0000000000 | \* |
| 332 | 59 | T | T | A | 0.00 | 0.8532110092 |  |
| 332 | 59 | T | T | C | 3.36 | 0.0736818794 |  |
| 332 | 59 | T | T | G | 0.56 | 0.7620766254 |  |
| 332 | 59 | T | T | T | 96.08 | 0.0000000000 | \* |
| 333 | 60 | C | C | A | 1.07 | 0.5766812792 |  |
| 333 | 60 | C | C | C | 93.33 | 0.0000000000 | \* |
| 333 | 60 | C | C | G | 3.73 | 0.1828496233 |  |
| 333 | 60 | C | C | T | 1.87 | 0.4811591911 |  |
| 334 | 61 | G | G | A | 7.49 | 0.0007470907 | \* |
| 334 | 61 | G | G | C | 1.30 | 0.5144588995 |  |
| 334 | 61 | G | G | G | 90.55 | 0.0000000000 | \* |
| 334 | 61 | G | G | T | 0.65 | 0.8251823718 |  |
| 335 | 62 | A | A | A | 95.51 | 0.0000000000 | \* |
| 335 | 62 | A | A | C | 2.54 | 0.1727235890 |  |
| 335 | 62 | A | A | G | 1.95 | 0.4306126999 |  |
| 335 | 62 | A | A | T | 0.00 | 0.9255319149 |  |
| 336 | 63 | C | C | A | 4.42 | 0.0248070746 |  |
| 336 | 63 | C | C | C | 92.26 | 0.0000000000 | \* |
| 336 | 63 | C | C | G | 0.22 | 0.8434978897 |  |
| 336 | 63 | C | C | T | 3.10 | 0.2316661307 |  |
| 337 | 64 | A | A | A | 89.14 | 0.0000000000 | \* |
| 337 | 64 | A | A | C | 3.19 | 0.0880299425 |  |
| 337 | 64 | A | A | G | 6.07 | 0.0548195582 |  |
| 337 | 64 | A | A | T | 1.60 | 0.5538734005 |  |
| 338 | 65 | G | G | A | 1.56 | 0.4047210539 |  |
| 338 | 65 | G | G | C | 4.01 | 0.0361196180 |  |
| 338 | 65 | G | G | G | 92.43 | 0.0000000000 | \* |
| 338 | 65 | G | G | T | 2.00 | 0.4462601271 |  |
| 339 | 66 | C | C | A | 0.00 | 0.8532110092 |  |
| 339 | 66 | C | C | C | 91.86 | 0.0000000000 | \* |
| 339 | 66 | C | C | G | 1.45 | 0.5371878188 |  |
| 339 | 66 | C | C | T | 6.69 | 0.0184934756 |  |
| 340 | 67 | T | T | A | 0.00 | 0.8532110092 |  |
| 340 | 67 | T | T | C | 3.43 | 0.0681641725 |  |
| 340 | 67 | T | T | G | 0.64 | 0.7404338408 |  |
| 340 | 67 | T | T | T | 95.92 | 0.0000000000 | \* |
| 341 | 68 | C | C | A | 0.78 | 0.6830498277 |  |
| 341 | 68 | C | C | C | 95.32 | 0.0000000000 | \* |
| 341 | 68 | C | C | G | 0.00 | 0.8777777778 |  |
| 341 | 68 | C | C | T | 3.90 | 0.1369656476 |  |
| 342 | 69 | C | C | A | 1.00 | 0.6015159425 |  |
| 342 | 69 | C | C | C | 95.00 | 0.0000000000 | \* |
| 342 | 69 | C | C | G | 0.00 | 0.8777777778 |  |
| 342 | 69 | C | C | T | 4.00 | 0.1276477347 |  |
| 343 | 70 | C | C | A | 1.76 | 0.3428689494 |  |
| 343 | 70 | C | C | C | 90.00 | 0.0000000000 | \* |
| 343 | 70 | C | C | G | 5.88 | 0.0605166032 |  |
| 343 | 70 | C | C | T | 2.35 | 0.3657925129 |  |
| 344 | 71 | G | G | A | 4.52 | 0.0224085572 |  |
| 344 | 71 | G | G | C | 5.12 | 0.0100135323 |  |
| 344 | 71 | G | G | G | 89.76 | 0.0000000000 | \* |
| 344 | 71 | G | G | T | 0.60 | 0.8375201877 |  |
| 345 | 72 | C | C | A | 6.43 | 0.0025995738 | \* |
| 345 | 72 | C | C | C | 84.58 | 0.0000000000 | \* |
| 345 | 72 | C | C | G | 5.40 | 0.0779568110 |  |
| 345 | 72 | C | C | T | 3.60 | 0.1671267047 |  |
| 346 | 73 | G | G | A | 9.01 | 0.0001218774 | \* |
| 346 | 73 | G | G | C | 4.20 | 0.0289695196 |  |
| 346 | 73 | G | G | G | 85.89 | 0.0000000000 | \* |
| 346 | 73 | G | G | T | 0.90 | 0.7569232999 |  |
| 347 | 74 | A | A | A | 90.79 | 0.0000000000 | \* |
| 347 | 74 | A | A | C | 3.29 | 0.0795889943 |  |
| 347 | 74 | A | A | G | 1.97 | 0.4266049807 |  |
| 347 | 74 | A | A | T | 3.95 | 0.1322930123 |  |
| 348 | 75 | C | C | A | 2.55 | 0.1705515914 |  |
| 348 | 75 | C | C | C | 90.23 | 0.0000000000 | \* |
| 348 | 75 | C | C | G | 1.49 | 0.5296813337 |  |
| 348 | 75 | C | C | T | 5.73 | 0.0374522683 |  |

## For use in R

If you want to work with the results in R, here is output that you can copy and paste in your terminal to get:

The base information:

```
structure(list(focal.base = c("A", "C", "G", "T"), avg.percsignal = c(93.9902029071068, 
93.4128635220073, 94.7474000951761, 95.6512425888321), avg.areasignal = c(419.657142857143, 
436.681818181818, 437.771929824561, 468.604651162791), crit.perc.area = c(5.24636072997967, 
5.12162715148319, 9.24333114111178, 7.50027328555718), mu = c(1.73213614502794, 
1.71300347462476, 2.45289285320085, 2.30416481818206), fillibens = c(0.985004768533681, 
0.983571693588482, 0.994251167070225, 0.989115099241062)), .Names = c("focal.base", 
"avg.percsignal", "avg.areasignal", "crit.perc.area", "mu", "fillibens"
), row.names = c(NA, -4L), class = "data.frame")
```

the data.frame that contains information on the guide region:

```
structure(list(A.area = c(396, 463, 7, 733, 12, 16, 8, 12, 9, 
16, 384, 13, 723, 18, 12, 10, 0, 12, 5, 3, 0, 1, 1, 2, 6, 312, 
6, 7, 400, 9, 321, 5, 2, 2, 4, 11, 345, 6, 6, 7, 0, 18, 493, 
9, 6, 15, 559, 13, 10, 8, 7, 229, 382, 18, 431, 365, 9, 0, 0, 
4, 23, 489, 20, 279, 7, 0, 0, 3, 5, 6, 15, 25, 30, 414, 12), 
    C.area = c(6, 7, 5, 6, 423, 6, 16, 6, 305, 511, 6, 10, 6, 
    487, 408, 7, 7, 7, 0, 0, 9, 9, 0, 6, 395, 9, 376, 9, 8, 426, 
    10, 391, 6, 1, 7, 318, 13, 502, 454, 429, 7, 6, 9, 436, 379, 
    8, 7, 369, 5, 7, 254, 3, 1, 1, 0, 0, 3, 0, 12, 350, 4, 13, 
    417, 10, 18, 316, 16, 367, 475, 306, 17, 329, 14, 15, 425
    ), G.area = c(9, 12, 379, 0, 0, 15, 810, 547, 6, 8, 11, 427, 
    10, 1, 8, 435, 9, 656, 10, 0, 15, 591, 14, 483, 9, 7, 46, 
    0, 0, 0, 13, 11, 313, 463, 379, 4, 1, 2, 4, 1, 12, 519, 13, 
    9, 16, 331, 6, 5, 248, 456, 5, 1, 19, 329, 8, 8, 266, 7, 
    2, 14, 278, 10, 1, 19, 415, 5, 3, 0, 0, 20, 298, 21, 286, 
    9, 7), T.area = c(4, 6, 5, 17, 10, 502, 7, 5, 12, 10, 7, 
    4, 17, 15, 11, 9, 325, 11, 420, 440, 446, 6, 398, 9, 17, 
    5, 12, 436, 7, 14, 0, 14, 1, 1, 4, 8, 9, 20, 16, 15, 444, 
    10, 0, 15, 12, 2, 12, 14, 0, 1, 6, 3, 8, 3, 6, 10, 14, 340, 
    343, 7, 2, 0, 14, 5, 9, 23, 447, 15, 20, 8, 2, 14, 3, 18, 
    27), Tot.area = c(415, 488, 396, 756, 445, 539, 841, 570, 
    332, 545, 408, 454, 756, 521, 439, 461, 341, 686, 435, 443, 
    470, 607, 413, 500, 427, 333, 440, 452, 415, 449, 344, 421, 
    322, 467, 394, 341, 368, 530, 480, 452, 463, 553, 515, 469, 
    413, 356, 584, 401, 263, 472, 272, 236, 410, 351, 445, 383, 
    292, 347, 357, 375, 307, 512, 452, 313, 449, 344, 466, 385, 
    500, 340, 332, 389, 333, 456, 471), A.perc = c(95.421686746988, 
    94.8770491803279, 1.76767676767677, 96.957671957672, 2.69662921348315, 
    2.96846011131725, 0.951248513674197, 2.10526315789474, 2.71084337349398, 
    2.93577981651376, 94.1176470588235, 2.86343612334802, 95.6349206349206, 
    3.45489443378119, 2.73348519362187, 2.16919739696312, 0, 
    1.74927113702624, 1.14942528735632, 0.677200902934537, 0, 
    0.164744645799012, 0.242130750605327, 0.4, 1.40515222482436, 
    93.6936936936937, 1.36363636363636, 1.54867256637168, 96.3855421686747, 
    2.00445434298441, 93.3139534883721, 1.18764845605701, 0.62111801242236, 
    0.428265524625268, 1.01522842639594, 3.2258064516129, 93.75, 
    1.13207547169811, 1.25, 1.54867256637168, 0, 3.25497287522604, 
    95.7281553398058, 1.91897654584222, 1.45278450363196, 4.21348314606742, 
    95.7191780821918, 3.24189526184539, 3.80228136882129, 1.69491525423729, 
    2.57352941176471, 97.0338983050847, 93.1707317073171, 5.12820512820513, 
    96.8539325842697, 95.3002610966057, 3.08219178082192, 0, 
    0, 1.06666666666667, 7.49185667752443, 95.5078125, 4.42477876106195, 
    89.1373801916933, 1.55902004454343, 0, 0, 0.779220779220779, 
    1, 1.76470588235294, 4.51807228915663, 6.426735218509, 9.00900900900901, 
    90.7894736842105, 2.54777070063694), C.perc = c(1.44578313253012, 
    1.4344262295082, 1.26262626262626, 0.793650793650794, 95.0561797752809, 
    1.11317254174397, 1.90249702734839, 1.05263157894737, 91.8674698795181, 
    93.7614678899083, 1.47058823529412, 2.20264317180617, 0.793650793650794, 
    93.4740882917466, 92.9384965831435, 1.51843817787419, 2.05278592375367, 
    1.02040816326531, 0, 0, 1.91489361702128, 1.4827018121911, 
    0, 1.2, 92.5058548009368, 2.7027027027027, 85.4545454545455, 
    1.99115044247788, 1.92771084337349, 94.8775055679287, 2.90697674418605, 
    92.874109263658, 1.86335403726708, 0.214132762312634, 1.77664974619289, 
    93.2551319648094, 3.53260869565217, 94.7169811320755, 94.5833333333333, 
    94.9115044247788, 1.51187904967603, 1.08499095840868, 1.74757281553398, 
    92.9637526652452, 91.7675544794189, 2.24719101123596, 1.1986301369863, 
    92.0199501246883, 1.90114068441065, 1.48305084745763, 93.3823529411765, 
    1.27118644067797, 0.24390243902439, 0.284900284900285, 0, 
    0, 1.02739726027397, 0, 3.36134453781513, 93.3333333333333, 
    1.30293159609121, 2.5390625, 92.2566371681416, 3.19488817891374, 
    4.00890868596882, 91.8604651162791, 3.43347639484979, 95.3246753246753, 
    95, 90, 5.12048192771084, 84.5758354755784, 4.2042042042042, 
    3.28947368421053, 90.2335456475584), G.perc = c(2.16867469879518, 
    2.45901639344262, 95.7070707070707, 0, 0, 2.78293135435993, 
    96.3139120095125, 95.9649122807018, 1.80722891566265, 1.46788990825688, 
    2.69607843137255, 94.0528634361233, 1.32275132275132, 0.191938579654511, 
    1.82232346241458, 94.3600867678959, 2.63929618768328, 95.6268221574344, 
    2.29885057471264, 0, 3.19148936170213, 97.3640856672158, 
    3.38983050847458, 96.6, 2.10772833723653, 2.1021021021021, 
    10.4545454545455, 0, 0, 0, 3.77906976744186, 2.61282660332542, 
    97.2049689440994, 99.1434689507495, 96.1928934010152, 1.17302052785924, 
    0.271739130434783, 0.377358490566038, 0.833333333333333, 
    0.221238938053097, 2.59179265658747, 93.8517179023508, 2.52427184466019, 
    1.91897654584222, 3.87409200968523, 92.9775280898876, 1.02739726027397, 
    1.24688279301746, 94.2965779467681, 96.6101694915254, 1.83823529411765, 
    0.423728813559322, 4.63414634146341, 93.7321937321937, 1.79775280898876, 
    2.088772845953, 91.0958904109589, 2.01729106628242, 0.560224089635854, 
    3.73333333333333, 90.5537459283388, 1.953125, 0.221238938053097, 
    6.0702875399361, 92.4276169265033, 1.45348837209302, 0.643776824034335, 
    0, 0, 5.88235294117647, 89.7590361445783, 5.39845758354756, 
    85.8858858858859, 1.97368421052632, 1.48619957537155), T.perc = c(0.963855421686747, 
    1.22950819672131, 1.26262626262626, 2.24867724867725, 2.24719101123596, 
    93.1354359925788, 0.832342449464923, 0.87719298245614, 3.6144578313253, 
    1.8348623853211, 1.7156862745098, 0.881057268722467, 2.24867724867725, 
    2.87907869481766, 2.50569476082005, 1.95227765726681, 95.3079178885631, 
    1.60349854227405, 96.551724137931, 99.3227990970655, 94.8936170212766, 
    0.988467874794069, 96.3680387409201, 1.8, 3.98126463700234, 
    1.5015015015015, 2.72727272727273, 96.4601769911504, 1.68674698795181, 
    3.11804008908686, 0, 3.32541567695962, 0.31055900621118, 
    0.214132762312634, 1.01522842639594, 2.34604105571848, 2.44565217391304, 
    3.77358490566038, 3.33333333333333, 3.31858407079646, 95.8963282937365, 
    1.80831826401447, 0, 3.19829424307036, 2.90556900726392, 
    0.561797752808989, 2.05479452054795, 3.49127182044888, 0, 
    0.211864406779661, 2.20588235294118, 1.27118644067797, 1.95121951219512, 
    0.854700854700855, 1.34831460674157, 2.61096605744125, 4.79452054794521, 
    97.9827089337176, 96.078431372549, 1.86666666666667, 0.651465798045603, 
    0, 3.09734513274336, 1.59744408945687, 2.00445434298441, 
    6.68604651162791, 95.9227467811159, 3.8961038961039, 4, 2.35294117647059, 
    0.602409638554217, 3.59897172236504, 0.900900900900901, 3.94736842105263, 
    5.73248407643312), base.call = c("A", "A", "G", "A", "C", 
    "T", "G", "G", "C", "C", "A", "G", "A", "C", "C", "G", "T", 
    "G", "T", "T", "T", "G", "T", "G", "C", "A", "C", "T", "A", 
    "C", "A", "C", "G", "G", "G", "C", "A", "C", "C", "C", "T", 
    "G", "A", "C", "C", "G", "A", "C", "G", "G", "C", "A", "A", 
    "G", "A", "A", "G", "T", "T", "C", "G", "A", "C", "A", "G", 
    "C", "T", "C", "C", "C", "G", "C", "G", "A", "C"), index = 274:348, 
    guide.seq = c("A", "A", "G", "A", "C", "T", "G", "G", "C", 
    "C", "A", "G", "A", "C", "C", "G", "T", "G", "T", "T", "T", 
    "G", "T", "G", "C", "A", "C", "T", "A", "C", "A", "C", "G", 
    "G", "G", "C", "A", "C", "C", "C", "T", "G", "A", "C", "C", 
    "G", "A", "C", "G", "G", "C", "A", "A", "G", "A", "A", "G", 
    "T", "T", "C", "G", "A", "C", "A", "G", "C", "T", "C", "C", 
    "C", "G", "C", "G", "A", "C"), T.pval = c(0.738726550651641, 
    0.660397804893476, 0.650610682922015, 0.388666908722207, 
    0.389000439866022, 0, 0.776398784614289, 0.763703601511183, 
    0.16541787931031, 0.489449123145355, 0.521251674491616, 0.762601504646291, 
    0.388666908722207, 0.265888587947994, 0.33414306571241, 0.459277678240407, 
    0, 0.552179258302358, 0, 0, 0, 0.731549589811549, 0, 0.498633484667088, 
    0.129283931373415, 0.581029052049546, 0.292113173947578, 
    0, 0.529142127584376, 0.228626113555451, 0.925531914893617, 
    0.200021460437232, 0.898415471547676, 0.912041188356376, 
    0.723714296574136, 0.367274393024509, 0.34632013119053, 0.148747814640161, 
    0.198994305373468, 0.200911462282361, 0, 0.496432940933165, 
    0.925531914893617, 0.217160391071144, 0.261519856384301, 
    0.847365654205321, 0.43393487936268, 0.179454283609371, 0.925531914893617, 
    0.91231201156435, 0.398354438382178, 0.648084158941539, 0.459544199324341, 
    0.770093071186471, 0.625403216988466, 0.313609119948652, 
    0.0735328699488625, 0, 0, 0.481159191057434, 0.825182371777312, 
    0.925531914893617, 0.23166613071323, 0.553873400511264, 0.446260127063628, 
    0.018493475612723, 0, 0.136965647599378, 0.12764773472929, 
    0.365792512936547, 0.837520187672299, 0.167126704698179, 
    0.756923299934488, 0.13229301233015, 0.0374522683250603), 
    C.pval = c(0.46144935658445, 0.465556413818553, 0.529894670817992, 
    0.715163498339997, 0, 0.588492961977885, 0.314700451617215, 
    0.612633438901091, 0, 0, 0.452548532733963, 0.239081150311206, 
    0.715163498339997, 0, 0, 0.435656463998449, 0.274789289756959, 
    0.625521859826705, 0.894117647057953, 0.894117647057953, 
    0.311249863569274, 0.448237134436599, 0.894117647057953, 
    0.554221493653989, 0, 0.146646919940568, 0, 0.290652625946709, 
    0.307712322802294, 0, 0.119020454975159, 0, 0.325784270347682, 
    0.881420581833408, 0.351355159449825, 0, 0.0612065711915197, 
    0, 0, 0, 0.437949853665054, 0.599713273490371, 0.360244240043436, 
    0, 0, 0.229227272677065, 0.554757757227765, 0, 0.315079729854866, 
    0.448113255796508, 0, 0.526600884350024, 0.877168702677217, 
    0.870317764086685, 0.894117647057953, 0.894117647057953, 
    0.622725408064364, 0.894117647057953, 0.0736818794237185, 
    0, 0.514458899490745, 0.172723589023696, 0, 0.0880299425422699, 
    0.0361196180029941, 0, 0.068164172529639, 0, 0, 0, 0.0100135322900762, 
    0, 0.0289695195971704, 0.0795889943408358, 0), G.pval = c(0.390076745359328, 
    0.340546500778536, 0, 0.877777777777744, 0.877777777777744, 
    0.2917933928447, 0, 0, 0.459915852715928, 0.53387421504423, 
    0.304221543095454, 0, 0.567884493860631, 0.849526004045034, 
    0.45681392116703, 0, 0.312597765191891, 0, 0.367163252266702, 
    0.877777777777744, 0.239223541737931, 0, 0.216949350962413, 
    0, 0.401207363298569, 0.402247960169768, 0.00516335728922934, 
    0.877777777777744, 0.877777777777744, 0.877777777777744, 
    0.178706253014363, 0.316571329027271, 0, 0, 0, 0.604322363746473, 
    0.83254791993848, 0.807936002888248, 0.690957061575314, 0.843497889714727, 
    0.31976044634009, 0, 0.330188455379549, 0.437335748425023, 
    0.170377433866167, 0, 0.640905051587901, 0.586185514721778, 
    0, 0, 0.45356157505835, 0.796601495925461, 0.115770955144374, 
    0, 0.461871466977573, 0.404722117032188, 0, 0.418203577997397, 
    0.762076625368498, 0.182849623307628, 0, 0.430612699851942, 
    0.843497889714727, 0.0548195582048367, 0, 0.537187818782164, 
    0.740433840807653, 0.877777777777744, 0.877777777777744, 
    0.0605166031621116, 0, 0.0779568109823956, 0, 0.426604980673167, 
    0.529681333659816), A.pval = c(0, 0, 0.342026786527509, 0, 
    0.147963721425413, 0.113467351206853, 0.619702271913856, 
    0.255976083841513, 0.145951349603669, 0.117191749190895, 
    0, 0.125831536473896, 0, 0.0694023483034978, 0.142795856873295, 
    0.241795605885488, 0.853211009174312, 0.347268089944414, 
    0.546093156646204, 0.719022159517916, 0.853211009174312, 
    0.845258547042264, 0.835078650228448, 0.802740744310845, 
    0.455324776956951, 0, 0.469551774574842, 0.40801274215678, 
    0, 0.279682871952824, 0, 0.532113375970872, 0.737916728674396, 
    0.795543710855962, 0.595835448883459, 0.0876943236044729, 
    0, 0.552473376731019, 0.509575113448193, 0.40801274215678, 
    0.853211009174312, 0.0851403892213582, 0, 0.301102602114547, 
    0.439290457553141, 0.0311820023375684, 0, 0.0862769244633002, 
    0.0483264259772946, 0.363077189179112, 0.166439698931855, 
    0, 0, 0.0114150707064145, 0, 0, 0.101327470478371, 0.853211009174312, 
    0.853211009174312, 0.576681279209857, 0.000747090676739504, 
    0, 0.0248070745633779, 0, 0.404721053894333, 0.853211009174312, 
    0.853211009174312, 0.68304982767274, 0.601515942457026, 0.342868949380093, 
    0.0224085572017132, 0.00259957383604581, 0.000121877393768122, 
    0, 0.170551591413773), guide.position = 1:75), .Names = c("A.area", 
"C.area", "G.area", "T.area", "Tot.area", "A.perc", "C.perc", 
"G.perc", "T.perc", "base.call", "index", "guide.seq", "T.pval", 
"C.pval", "G.pval", "A.pval", "guide.position"), row.names = 274:348, class = "data.frame")
```

*Report generated using EditR v1.0.8*
