# Supplementary material for: Mechanistic and genetic basis of single-strand templated repair at Cas12a-induced DNA breaks in Chlamydomonas reinhardtii
Source: Nat Commun. 2021 Nov 19;12:6751. doi: 10.1038/s41467-021-27004-1 (PMC8604939; doi:10.1038/s41467-021-27004-1)
Supplement: Supplementary file 22 — Source Data [file 41467_2021_27004_MOESM22_ESM.zip › Source Data/EditR analysis/EditR outputs/Sense/rep1_ssODN_sense_32.html]

EditR v1.0.8 report


# EditR v1.0.8 report

- Data QA
  - Filtering data
  - Percent noise peak area
  - Base information
- Predicted editing
  - Editing bar plot
  - Editing table plot
  - Table of editing results
- For use in R

## Data QA

### Filtering data

What the data looked like prefiltering:

and the post filtering signal / noise plot:

### Percent noise peak area

### Base information

Here’s information about the signal of each base, the critical percent value where any higher value would be called as significant, and Filliben’s correlation for how well the noise was modelled by the zero adjusted gamma distribution.

| Base | Average percent signal | Average peak area | Critical percent value | model mu | Fillibens correlation |
| --- | --- | --- | --- | --- | --- |
| A | 95.04080 | 476.0000 | 4.992518 | 1.583269 | 0.9921266 |
| C | 94.23209 | 462.3939 | 4.460739 | 1.589294 | 0.9725339 |
| G | 94.89492 | 436.0000 | 6.128144 | 2.001735 | 0.9953540 |
| T | 95.66733 | 474.1591 | 5.975932 | 2.022777 | 0.9940042 |

## Predicted editing

### Editing bar plot

### Editing table plot

### Table of editing results


Here’s the entire guide region

| Sanger position | Guide position | Guide sequence | Sanger base call | Focal base | Focal base peak area | p value |  |
| --- | --- | --- | --- | --- | --- | --- | --- |
| 275 | 1 | A | A | A | 94.21 | 0.000000e+00 | \* |
| 275 | 1 | A | A | C | 1.72 | 3.537682e-01 |  |
| 275 | 1 | A | A | G | 3.00 | 1.622106e-01 |  |
| 275 | 1 | A | A | T | 1.07 | 6.823047e-01 |  |
| 276 | 2 | A | A | A | 95.17 | 0.000000e+00 | \* |
| 276 | 2 | A | A | C | 1.86 | 3.045534e-01 |  |
| 276 | 2 | A | A | G | 1.49 | 4.881251e-01 |  |
| 276 | 2 | A | A | T | 1.49 | 5.405559e-01 |  |
| 277 | 3 | G | G | A | 1.75 | 3.224479e-01 |  |
| 277 | 3 | G | G | C | 2.49 | 1.460942e-01 |  |
| 277 | 3 | G | G | G | 94.01 | 0.000000e+00 | \* |
| 277 | 3 | G | G | T | 1.75 | 4.570393e-01 |  |
| 278 | 4 | A | A | A | 96.83 | 0.000000e+00 | \* |
| 278 | 4 | A | A | C | 0.82 | 7.542746e-01 |  |
| 278 | 4 | A | A | G | 1.18 | 5.848112e-01 |  |
| 278 | 4 | A | A | T | 1.18 | 6.472514e-01 |  |
| 279 | 5 | C | C | A | 3.63 | 4.712194e-02 |  |
| 279 | 5 | C | C | C | 90.73 | 0.000000e+00 | \* |
| 279 | 5 | C | C | G | 4.03 | 6.815610e-02 |  |
| 279 | 5 | C | C | T | 1.61 | 4.990821e-01 |  |
| 280 | 6 | T | T | A | 2.46 | 1.629545e-01 |  |
| 280 | 6 | T | T | C | 1.51 | 4.338329e-01 |  |
| 280 | 6 | T | T | G | 2.65 | 2.154248e-01 |  |
| 280 | 6 | T | T | T | 93.38 | 0.000000e+00 | \* |
| 281 | 7 | G | G | A | 0.84 | 6.632245e-01 |  |
| 281 | 7 | G | G | C | 1.91 | 2.875959e-01 |  |
| 281 | 7 | G | G | G | 96.54 | 0.000000e+00 | \* |
| 281 | 7 | G | G | T | 0.72 | 7.962199e-01 |  |
| 282 | 8 | G | G | A | 2.26 | 1.973657e-01 |  |
| 282 | 8 | G | G | C | 1.39 | 4.848634e-01 |  |
| 282 | 8 | G | G | G | 96.34 | 0.000000e+00 | \* |
| 282 | 8 | G | G | T | 0.00 | 9.032258e-01 |  |
| 283 | 9 | C | C | A | 1.17 | 5.248127e-01 |  |
| 283 | 9 | C | C | C | 93.59 | 0.000000e+00 | \* |
| 283 | 9 | C | C | G | 2.04 | 3.380407e-01 |  |
| 283 | 9 | C | C | T | 3.21 | 1.440631e-01 |  |
| 284 | 10 | C | C | A | 1.89 | 2.833406e-01 |  |
| 284 | 10 | C | C | C | 92.62 | 0.000000e+00 | \* |
| 284 | 10 | C | C | G | 2.92 | 1.741603e-01 |  |
| 284 | 10 | C | C | T | 2.57 | 2.460545e-01 |  |
| 285 | 11 | A | A | A | 95.06 | 0.000000e+00 | \* |
| 285 | 11 | A | A | C | 1.50 | 4.380738e-01 |  |
| 285 | 11 | A | A | G | 3.43 | 1.138778e-01 |  |
| 285 | 11 | A | A | T | 0.00 | 9.032258e-01 |  |
| 286 | 12 | G | G | A | 1.97 | 2.612307e-01 |  |
| 286 | 12 | G | G | C | 0.88 | 7.288894e-01 |  |
| 286 | 12 | G | G | G | 95.39 | 0.000000e+00 | \* |
| 286 | 12 | G | G | T | 1.75 | 4.543363e-01 |  |
| 287 | 13 | A | A | A | 95.74 | 0.000000e+00 | \* |
| 287 | 13 | A | A | C | 1.15 | 5.969012e-01 |  |
| 287 | 13 | A | A | G | 1.61 | 4.511068e-01 |  |
| 287 | 13 | A | A | T | 1.50 | 5.369778e-01 |  |
| 288 | 14 | C | C | A | 3.12 | 8.195660e-02 |  |
| 288 | 14 | C | C | C | 94.13 | 0.000000e+00 | \* |
| 288 | 14 | C | C | G | 0.00 | 8.444444e-01 |  |
| 288 | 14 | C | C | T | 2.75 | 2.124061e-01 |  |
| 289 | 15 | C | C | A | 2.97 | 9.578547e-02 |  |
| 289 | 15 | C | C | C | 92.78 | 0.000000e+00 | \* |
| 289 | 15 | C | C | G | 2.12 | 3.187847e-01 |  |
| 289 | 15 | C | C | T | 2.12 | 3.492328e-01 |  |
| 290 | 16 | G | G | A | 3.02 | 9.072058e-02 |  |
| 290 | 16 | G | G | C | 1.30 | 5.291368e-01 |  |
| 290 | 16 | G | G | G | 93.95 | 0.000000e+00 | \* |
| 290 | 16 | G | G | T | 1.73 | 4.625572e-01 |  |
| 291 | 17 | T | T | A | 2.03 | 2.479141e-01 |  |
| 291 | 17 | T | T | C | 0.29 | 9.343327e-01 |  |
| 291 | 17 | T | T | G | 2.32 | 2.763351e-01 |  |
| 291 | 17 | T | T | T | 95.36 | 0.000000e+00 | \* |
| 292 | 18 | G | G | A | 0.87 | 6.484392e-01 |  |
| 292 | 18 | G | G | C | 1.74 | 3.447468e-01 |  |
| 292 | 18 | G | G | G | 95.79 | 0.000000e+00 | \* |
| 292 | 18 | G | G | T | 1.60 | 5.043991e-01 |  |
| 293 | 19 | T | T | A | 1.13 | 5.379144e-01 |  |
| 293 | 19 | T | T | C | 0.00 | 9.529412e-01 |  |
| 293 | 19 | T | T | G | 2.49 | 2.421754e-01 |  |
| 293 | 19 | T | T | T | 96.37 | 0.000000e+00 | \* |
| 294 | 20 | T | T | A | 0.22 | 8.859075e-01 |  |
| 294 | 20 | T | T | C | 0.00 | 9.529412e-01 |  |
| 294 | 20 | T | T | G | 2.41 | 2.587210e-01 |  |
| 294 | 20 | T | T | T | 97.37 | 0.000000e+00 | \* |
| 295 | 21 | T | T | A | 0.00 | 9.090909e-01 |  |
| 295 | 21 | T | T | C | 2.31 | 1.832746e-01 |  |
| 295 | 21 | T | T | G | 2.10 | 3.249403e-01 |  |
| 295 | 21 | T | T | T | 95.60 | 0.000000e+00 | \* |
| 296 | 22 | G | G | A | 0.00 | 9.090909e-01 |  |
| 296 | 22 | G | G | C | 2.35 | 1.733049e-01 |  |
| 296 | 22 | G | G | G | 96.47 | 0.000000e+00 | \* |
| 296 | 22 | G | G | T | 1.18 | 6.467747e-01 |  |
| 297 | 23 | T | T | A | 0.98 | 6.037357e-01 |  |
| 297 | 23 | T | T | C | 0.00 | 9.529412e-01 |  |
| 297 | 23 | T | T | G | 2.44 | 2.525508e-01 |  |
| 297 | 23 | T | T | T | 96.59 | 0.000000e+00 | \* |
| 298 | 24 | G | G | A | 1.19 | 5.141452e-01 |  |
| 298 | 24 | G | G | C | 0.99 | 6.731320e-01 |  |
| 298 | 24 | G | G | G | 95.43 | 0.000000e+00 | \* |
| 298 | 24 | G | G | T | 2.39 | 2.856462e-01 |  |
| 299 | 25 | C | C | A | 1.10 | 5.521853e-01 |  |
| 299 | 25 | C | C | C | 93.63 | 0.000000e+00 | \* |
| 299 | 25 | C | C | G | 1.54 | 4.728151e-01 |  |
| 299 | 25 | C | C | T | 3.74 | 8.957502e-02 |  |
| 300 | 26 | A | A | A | 91.69 | 0.000000e+00 | \* |
| 300 | 26 | A | A | C | 3.38 | 4.640697e-02 |  |
| 300 | 26 | A | A | G | 2.34 | 2.724969e-01 |  |
| 300 | 26 | A | A | T | 2.60 | 2.412161e-01 |  |
| 301 | 27 | C | C | A | 1.38 | 4.433613e-01 |  |
| 301 | 27 | C | C | C | 96.46 | 0.000000e+00 | \* |
| 301 | 27 | C | C | G | 0.00 | 8.444444e-01 |  |
| 301 | 27 | C | C | T | 2.17 | 3.383657e-01 |  |
| 302 | 28 | T | T | A | 2.00 | 2.538571e-01 |  |
| 302 | 28 | T | T | C | 2.61 | 1.272771e-01 |  |
| 302 | 28 | T | T | G | 0.00 | 8.444444e-01 |  |
| 302 | 28 | T | T | T | 95.39 | 0.000000e+00 | \* |
| 303 | 29 | A | A | A | 95.59 | 0.000000e+00 | \* |
| 303 | 29 | A | A | C | 1.60 | 3.969236e-01 |  |
| 303 | 29 | A | A | G | 0.00 | 8.444444e-01 |  |
| 303 | 29 | A | A | T | 2.81 | 2.031776e-01 |  |
| 304 | 30 | C | C | A | 2.04 | 2.451399e-01 |  |
| 304 | 30 | C | C | C | 93.27 | 0.000000e+00 | \* |
| 304 | 30 | C | C | G | 1.43 | 5.057708e-01 |  |
| 304 | 30 | C | C | T | 3.27 | 1.368698e-01 |  |
| 305 | 31 | A | A | A | 92.86 | 0.000000e+00 | \* |
| 305 | 31 | A | A | C | 2.55 | 1.361424e-01 |  |
| 305 | 31 | A | A | G | 2.81 | 1.900776e-01 |  |
| 305 | 31 | A | A | T | 1.79 | 4.447324e-01 |  |
| 306 | 32 | C | C | A | 1.39 | 4.405412e-01 |  |
| 306 | 32 | C | C | C | 94.00 | 0.000000e+00 | \* |
| 306 | 32 | C | C | G | 1.15 | 5.912641e-01 |  |
| 306 | 32 | C | C | T | 3.46 | 1.146774e-01 |  |
| 307 | 33 | G | G | A | 0.94 | 6.199350e-01 |  |
| 307 | 33 | G | G | C | 2.50 | 1.449806e-01 |  |
| 307 | 33 | G | G | G | 95.94 | 0.000000e+00 | \* |
| 307 | 33 | G | G | T | 0.62 | 8.214550e-01 |  |
| 308 | 34 | G | G | A | 0.00 | 9.090909e-01 |  |
| 308 | 34 | G | G | C | 0.85 | 7.411047e-01 |  |
| 308 | 34 | G | G | G | 98.72 | 0.000000e+00 | \* |
| 308 | 34 | G | G | T | 0.43 | 8.665171e-01 |  |
| 309 | 35 | G | G | A | 1.25 | 4.928676e-01 |  |
| 309 | 35 | G | G | C | 1.25 | 5.519299e-01 |  |
| 309 | 35 | G | G | G | 96.51 | 0.000000e+00 | \* |
| 309 | 35 | G | G | T | 1.00 | 7.078286e-01 |  |
| 310 | 36 | C | C | A | 2.13 | 2.243327e-01 |  |
| 310 | 36 | C | C | C | 94.40 | 0.000000e+00 | \* |
| 310 | 36 | C | C | G | 1.60 | 4.548310e-01 |  |
| 310 | 36 | C | C | T | 1.87 | 4.204685e-01 |  |
| 311 | 37 | A | A | A | 95.23 | 0.000000e+00 | \* |
| 311 | 37 | A | A | C | 2.86 | 9.163793e-02 |  |
| 311 | 37 | A | A | G | 1.91 | 3.705145e-01 |  |
| 311 | 37 | A | A | T | 0.00 | 9.032258e-01 |  |
| 312 | 38 | C | C | A | 0.89 | 6.396870e-01 |  |
| 312 | 38 | C | C | C | 95.72 | 0.000000e+00 | \* |
| 312 | 38 | C | C | G | 1.43 | 5.065461e-01 |  |
| 312 | 38 | C | C | T | 1.96 | 3.933066e-01 |  |
| 313 | 39 | C | C | A | 1.37 | 4.473084e-01 |  |
| 313 | 39 | C | C | C | 94.73 | 0.000000e+00 | \* |
| 313 | 39 | C | C | G | 2.15 | 3.130349e-01 |  |
| 313 | 39 | C | C | T | 1.76 | 4.532802e-01 |  |
| 314 | 40 | C | C | A | 1.86 | 2.905896e-01 |  |
| 314 | 40 | C | C | C | 95.04 | 0.000000e+00 | \* |
| 314 | 40 | C | C | G | 0.62 | 7.530065e-01 |  |
| 314 | 40 | C | C | T | 2.48 | 2.652540e-01 |  |
| 315 | 41 | T | T | A | 1.74 | 3.232400e-01 |  |
| 315 | 41 | T | T | C | 1.96 | 2.724692e-01 |  |
| 315 | 41 | T | T | G | 1.74 | 4.145465e-01 |  |
| 315 | 41 | T | T | T | 94.55 | 0.000000e+00 | \* |
| 316 | 42 | G | G | A | 2.90 | 1.035276e-01 |  |
| 316 | 42 | G | G | C | 1.27 | 5.420113e-01 |  |
| 316 | 42 | G | G | G | 94.57 | 0.000000e+00 | \* |
| 316 | 42 | G | G | T | 1.27 | 6.151481e-01 |  |
| 317 | 43 | A | A | A | 95.44 | 0.000000e+00 | \* |
| 317 | 43 | A | A | C | 1.35 | 5.038230e-01 |  |
| 317 | 43 | A | A | G | 2.87 | 1.804404e-01 |  |
| 317 | 43 | A | A | T | 0.34 | 8.811231e-01 |  |
| 318 | 44 | C | C | A | 2.24 | 2.012603e-01 |  |
| 318 | 44 | C | C | C | 94.29 | 0.000000e+00 | \* |
| 318 | 44 | C | C | G | 0.00 | 8.444444e-01 |  |
| 318 | 44 | C | C | T | 3.47 | 1.141449e-01 |  |
| 319 | 45 | C | C | A | 0.70 | 7.207984e-01 |  |
| 319 | 45 | C | C | C | 92.76 | 0.000000e+00 | \* |
| 319 | 45 | C | C | G | 4.21 | 5.853235e-02 |  |
| 319 | 45 | C | C | T | 2.34 | 2.968462e-01 |  |
| 320 | 46 | G | G | A | 4.11 | 2.756101e-02 |  |
| 320 | 46 | G | G | C | 2.47 | 1.511905e-01 |  |
| 320 | 46 | G | G | G | 92.88 | 0.000000e+00 | \* |
| 320 | 46 | G | G | T | 0.55 | 8.406144e-01 |  |
| 321 | 47 | A | A | A | 95.94 | 0.000000e+00 | \* |
| 321 | 47 | A | A | C | 0.90 | 7.170644e-01 |  |
| 321 | 47 | A | A | G | 1.05 | 6.236590e-01 |  |
| 321 | 47 | A | A | T | 2.11 | 3.539098e-01 |  |
| 322 | 48 | C | C | A | 2.83 | 1.112041e-01 |  |
| 322 | 48 | C | C | C | 92.45 | 0.000000e+00 | \* |
| 322 | 48 | C | C | G | 1.42 | 5.098794e-01 |  |
| 322 | 48 | C | C | T | 3.30 | 1.325214e-01 |  |
| 323 | 49 | G | G | A | 3.11 | 8.252057e-02 |  |
| 323 | 49 | G | G | C | 2.72 | 1.096478e-01 |  |
| 323 | 49 | G | G | G | 93.39 | 0.000000e+00 | \* |
| 323 | 49 | G | G | T | 0.78 | 7.781379e-01 |  |
| 324 | 50 | G | G | A | 2.23 | 2.048739e-01 |  |
| 324 | 50 | G | G | C | 1.42 | 4.746032e-01 |  |
| 324 | 50 | G | G | G | 94.94 | 0.000000e+00 | \* |
| 324 | 50 | G | G | T | 1.42 | 5.641415e-01 |  |
| 325 | 51 | C | C | A | 2.39 | 1.745103e-01 |  |
| 325 | 51 | C | C | C | 93.52 | 0.000000e+00 | \* |
| 325 | 51 | C | C | G | 2.05 | 3.363794e-01 |  |
| 325 | 51 | C | C | T | 2.05 | 3.692442e-01 |  |
| 326 | 52 | A | A | A | 96.83 | 0.000000e+00 | \* |
| 326 | 52 | A | A | C | 1.19 | 5.785675e-01 |  |
| 326 | 52 | A | A | G | 0.79 | 7.038416e-01 |  |
| 326 | 52 | A | A | T | 1.19 | 6.419436e-01 |  |
| 327 | 53 | A | A | A | 94.95 | 0.000000e+00 | \* |
| 327 | 53 | A | A | C | 0.88 | 7.279833e-01 |  |
| 327 | 53 | A | A | G | 3.30 | 1.276503e-01 |  |
| 327 | 53 | A | A | T | 0.88 | 7.467031e-01 |  |
| 328 | 54 | G | G | A | 5.20 | 7.824605e-03 | \* |
| 328 | 54 | G | G | C | 0.58 | 8.565193e-01 |  |
| 328 | 54 | G | G | G | 93.93 | 0.000000e+00 | \* |
| 328 | 54 | G | G | T | 0.29 | 8.876808e-01 |  |
| 329 | 55 | A | A | A | 96.49 | 0.000000e+00 | \* |
| 329 | 55 | A | A | C | 0.21 | 9.452728e-01 |  |
| 329 | 55 | A | A | G | 1.44 | 5.012966e-01 |  |
| 329 | 55 | A | A | T | 1.86 | 4.237166e-01 |  |
| 330 | 56 | A | A | A | 97.31 | 0.000000e+00 | \* |
| 330 | 56 | A | A | C | 0.45 | 8.987143e-01 |  |
| 330 | 56 | A | A | G | 0.00 | 8.444444e-01 |  |
| 330 | 56 | A | A | T | 2.24 | 3.192277e-01 |  |
| 331 | 57 | G | G | A | 2.37 | 1.773510e-01 |  |
| 331 | 57 | G | G | C | 2.71 | 1.113095e-01 |  |
| 331 | 57 | G | G | G | 90.85 | 0.000000e+00 | \* |
| 331 | 57 | G | G | T | 4.07 | 6.582320e-02 |  |
| 332 | 58 | T | T | A | 2.20 | 2.107382e-01 |  |
| 332 | 58 | T | T | C | 0.55 | 8.667082e-01 |  |
| 332 | 58 | T | T | G | 1.10 | 6.089842e-01 |  |
| 332 | 58 | T | T | T | 96.15 | 0.000000e+00 | \* |
| 333 | 59 | T | T | A | 2.19 | 2.132158e-01 |  |
| 333 | 59 | T | T | C | 2.19 | 2.111051e-01 |  |
| 333 | 59 | T | T | G | 1.09 | 6.108900e-01 |  |
| 333 | 59 | T | T | T | 94.54 | 0.000000e+00 | \* |
| 334 | 60 | C | C | A | 1.01 | 5.902365e-01 |  |
| 334 | 60 | C | C | C | 94.96 | 0.000000e+00 | \* |
| 334 | 60 | C | C | G | 2.02 | 3.442224e-01 |  |
| 334 | 60 | C | C | T | 2.02 | 3.781601e-01 |  |
| 335 | 61 | G | G | A | 6.06 | 2.827933e-03 | \* |
| 335 | 61 | G | G | C | 1.68 | 3.660559e-01 |  |
| 335 | 61 | G | G | G | 90.57 | 0.000000e+00 | \* |
| 335 | 61 | G | G | T | 1.68 | 4.764839e-01 |  |
| 336 | 62 | A | A | A | 96.55 | 0.000000e+00 | \* |
| 336 | 62 | A | A | C | 2.18 | 2.130581e-01 |  |
| 336 | 62 | A | A | G | 0.00 | 8.444444e-01 |  |
| 336 | 62 | A | A | T | 1.27 | 6.143544e-01 |  |
| 337 | 63 | C | C | A | 2.26 | 1.976440e-01 |  |
| 337 | 63 | C | C | C | 92.80 | 0.000000e+00 | \* |
| 337 | 63 | C | C | G | 3.09 | 1.517526e-01 |  |
| 337 | 63 | C | C | T | 1.85 | 4.248480e-01 |  |
| 338 | 64 | A | A | A | 91.69 | 0.000000e+00 | \* |
| 338 | 64 | A | A | C | 2.77 | 1.034832e-01 |  |
| 338 | 64 | A | A | G | 5.54 | 1.741756e-02 |  |
| 338 | 64 | A | A | T | 0.00 | 9.032258e-01 |  |
| 339 | 65 | G | G | A | 1.59 | 3.711429e-01 |  |
| 339 | 65 | G | G | C | 2.27 | 1.918266e-01 |  |
| 339 | 65 | G | G | G | 96.15 | 0.000000e+00 | \* |
| 339 | 65 | G | G | T | 0.00 | 9.032258e-01 |  |
| 340 | 66 | C | C | A | 0.27 | 8.751935e-01 |  |
| 340 | 66 | C | C | C | 93.88 | 0.000000e+00 | \* |
| 340 | 66 | C | C | G | 1.33 | 5.361756e-01 |  |
| 340 | 66 | C | C | T | 4.52 | 4.271934e-02 |  |
| 341 | 67 | T | T | A | 0.84 | 6.622587e-01 |  |
| 341 | 67 | T | T | C | 2.73 | 1.094212e-01 |  |
| 341 | 67 | T | T | G | 0.21 | 8.336546e-01 |  |
| 341 | 67 | T | T | T | 96.23 | 0.000000e+00 | \* |
| 342 | 68 | C | C | A | 0.00 | 9.090909e-01 |  |
| 342 | 68 | C | C | C | 96.87 | 0.000000e+00 | \* |
| 342 | 68 | C | C | G | 0.00 | 8.444444e-01 |  |
| 342 | 68 | C | C | T | 3.13 | 1.537336e-01 |  |
| 343 | 69 | C | C | A | 0.19 | 8.921273e-01 |  |
| 343 | 69 | C | C | C | 95.70 | 0.000000e+00 | \* |
| 343 | 69 | C | C | G | 0.19 | 8.359695e-01 |  |
| 343 | 69 | C | C | T | 3.93 | 7.521499e-02 |  |
| 344 | 70 | C | C | A | 0.55 | 7.823251e-01 |  |
| 344 | 70 | C | C | C | 91.48 | 0.000000e+00 | \* |
| 344 | 70 | C | C | G | 3.85 | 8.011951e-02 |  |
| 344 | 70 | C | C | T | 4.12 | 6.261370e-02 |  |
| 345 | 71 | G | G | A | 3.27 | 6.986808e-02 |  |
| 345 | 71 | G | G | C | 2.94 | 8.290743e-02 |  |
| 345 | 71 | G | G | G | 92.48 | 0.000000e+00 | \* |
| 345 | 71 | G | G | T | 1.31 | 6.016879e-01 |  |
| 346 | 72 | C | C | A | 2.59 | 1.423849e-01 |  |
| 346 | 72 | C | C | C | 91.71 | 0.000000e+00 | \* |
| 346 | 72 | C | C | G | 2.33 | 2.737272e-01 |  |
| 346 | 72 | C | C | T | 3.37 | 1.249874e-01 |  |
| 347 | 73 | G | G | A | 5.43 | 5.976609e-03 | \* |
| 347 | 73 | G | G | C | 2.56 | 1.353207e-01 |  |
| 347 | 73 | G | G | G | 89.46 | 0.000000e+00 | \* |
| 347 | 73 | G | G | T | 2.56 | 2.494548e-01 |  |
| 348 | 74 | A | A | A | 96.19 | 0.000000e+00 | \* |
| 348 | 74 | A | A | C | 1.40 | 4.808487e-01 |  |
| 348 | 74 | A | A | G | 2.40 | 2.591479e-01 |  |
| 348 | 74 | A | A | T | 0.00 | 9.032258e-01 |  |
| 349 | 75 | C | C | A | 1.43 | 4.240618e-01 |  |
| 349 | 75 | C | C | C | 77.10 | 0.000000e+00 | \* |
| 349 | 75 | C | C | G | 16.97 | 1.582349e-07 | \* |
| 349 | 75 | C | C | T | 4.50 | 4.364883e-02 |  |

## For use in R

If you want to work with the results in R, here is output that you can copy and paste in your terminal to get:

The base information:

```
structure(list(focal.base = c("A", "C", "G", "T"), avg.percsignal = c(95.0407982180913, 
94.2320885521552, 94.8949179809292, 95.6673286810021), avg.areasignal = c(476, 
462.393939393939, 436, 474.159090909091), crit.perc.area = c(4.99251808837531, 
4.46073889611276, 6.1281439519566, 5.97593157556704), mu = c(1.58326910497342, 
1.58929436902803, 2.00173474581892, 2.02277711731425), fillibens = c(0.992126553612273, 
0.97253391798947, 0.995353959357853, 0.994004216749621)), .Names = c("focal.base", 
"avg.percsignal", "avg.areasignal", "crit.perc.area", "mu", "fillibens"
), row.names = c(NA, -4L), class = "data.frame")
```

the data.frame that contains information on the guide region:

```
structure(list(A.area = c(439, 512, 7, 824, 18, 13, 7, 13, 4, 
11, 443, 9, 831, 17, 14, 14, 7, 6, 5, 1, 0, 0, 4, 6, 5, 353, 
7, 10, 477, 10, 364, 6, 3, 0, 5, 8, 399, 5, 7, 9, 8, 16, 565, 
11, 3, 15, 638, 12, 8, 11, 7, 244, 432, 18, 468, 434, 7, 8, 8, 
4, 18, 532, 11, 298, 7, 1, 4, 0, 1, 2, 10, 10, 17, 480, 7), C.area = c(8, 
10, 10, 7, 450, 8, 16, 8, 321, 540, 7, 4, 10, 513, 437, 6, 1, 
12, 0, 0, 11, 14, 0, 5, 426, 13, 490, 13, 8, 457, 10, 407, 8, 
4, 5, 354, 12, 537, 485, 460, 9, 7, 8, 462, 397, 9, 6, 392, 7, 
7, 274, 3, 4, 2, 1, 2, 8, 2, 8, 377, 5, 12, 451, 9, 10, 353, 
13, 402, 512, 333, 9, 354, 8, 7, 377), G.area = c(14, 8, 377, 
10, 20, 14, 808, 553, 7, 17, 16, 435, 14, 0, 10, 435, 8, 660, 
11, 11, 10, 574, 10, 480, 7, 9, 0, 0, 0, 7, 11, 5, 307, 464, 
387, 6, 8, 8, 11, 3, 8, 522, 17, 0, 18, 339, 7, 6, 240, 469, 
6, 2, 15, 325, 7, 0, 268, 4, 4, 8, 269, 0, 15, 18, 424, 5, 1, 
0, 1, 14, 283, 9, 280, 12, 83), T.area = c(5, 8, 7, 10, 8, 494, 
6, 0, 11, 15, 0, 8, 13, 15, 10, 8, 329, 11, 425, 445, 456, 7, 
396, 12, 17, 10, 11, 476, 14, 16, 7, 15, 2, 2, 4, 7, 0, 11, 9, 
12, 434, 7, 2, 17, 10, 2, 14, 14, 2, 7, 6, 3, 4, 1, 9, 10, 12, 
350, 346, 8, 5, 7, 9, 0, 0, 17, 459, 13, 21, 15, 4, 13, 8, 0, 
22), Tot.area = c(466, 538, 401, 851, 496, 529, 837, 574, 343, 
583, 466, 456, 868, 545, 471, 463, 345, 689, 441, 457, 477, 595, 
410, 503, 455, 385, 508, 499, 499, 490, 392, 433, 320, 470, 401, 
375, 419, 561, 512, 484, 459, 552, 592, 490, 428, 365, 665, 424, 
257, 494, 293, 252, 455, 346, 485, 446, 295, 364, 366, 397, 297, 
551, 486, 325, 441, 376, 477, 415, 535, 364, 306, 386, 313, 499, 
489), A.perc = c(94.206008583691, 95.1672862453532, 1.74563591022444, 
96.8272620446534, 3.62903225806452, 2.45746691871456, 0.836320191158901, 
2.26480836236934, 1.16618075801749, 1.88679245283019, 95.0643776824034, 
1.97368421052632, 95.7373271889401, 3.11926605504587, 2.9723991507431, 
3.02375809935205, 2.02898550724638, 0.870827285921626, 1.13378684807256, 
0.218818380743982, 0, 0, 0.975609756097561, 1.19284294234592, 
1.0989010989011, 91.6883116883117, 1.37795275590551, 2.00400801603206, 
95.5911823647295, 2.04081632653061, 92.8571428571429, 1.38568129330254, 
0.9375, 0, 1.24688279301746, 2.13333333333333, 95.2267303102625, 
0.89126559714795, 1.3671875, 1.8595041322314, 1.74291938997821, 
2.89855072463768, 95.4391891891892, 2.24489795918367, 0.700934579439252, 
4.10958904109589, 95.9398496240601, 2.83018867924528, 3.11284046692607, 
2.22672064777328, 2.38907849829352, 96.8253968253968, 94.9450549450549, 
5.20231213872832, 96.4948453608247, 97.3094170403587, 2.3728813559322, 
2.1978021978022, 2.18579234972678, 1.00755667506297, 6.06060606060606, 
96.551724137931, 2.26337448559671, 91.6923076923077, 1.58730158730159, 
0.265957446808511, 0.838574423480084, 0, 0.186915887850467, 0.549450549450549, 
3.26797385620915, 2.59067357512953, 5.43130990415335, 96.1923847695391, 
1.43149284253579), C.perc = c(1.71673819742489, 1.85873605947955, 
2.49376558603491, 0.82256169212691, 90.7258064516129, 1.51228733459357, 
1.9115890083632, 1.39372822299652, 93.5860058309038, 92.6243567753002, 
1.50214592274678, 0.87719298245614, 1.15207373271889, 94.1284403669725, 
92.7813163481953, 1.29589632829374, 0.289855072463768, 1.74165457184325, 
0, 0, 2.30607966457023, 2.35294117647059, 0, 0.99403578528827, 
93.6263736263736, 3.37662337662338, 96.4566929133858, 2.60521042084168, 
1.60320641282565, 93.265306122449, 2.55102040816327, 93.9953810623557, 
2.5, 0.851063829787234, 1.24688279301746, 94.4, 2.86396181384248, 
95.7219251336898, 94.7265625, 95.0413223140496, 1.96078431372549, 
1.26811594202899, 1.35135135135135, 94.2857142857143, 92.7570093457944, 
2.46575342465753, 0.902255639097744, 92.4528301886792, 2.72373540856031, 
1.417004048583, 93.5153583617747, 1.19047619047619, 0.879120879120879, 
0.578034682080925, 0.206185567010309, 0.448430493273543, 2.71186440677966, 
0.549450549450549, 2.18579234972678, 94.9622166246851, 1.68350168350168, 
2.17785843920145, 92.798353909465, 2.76923076923077, 2.26757369614512, 
93.8829787234042, 2.72536687631027, 96.8674698795181, 95.7009345794392, 
91.4835164835165, 2.94117647058824, 91.7098445595855, 2.55591054313099, 
1.40280561122244, 77.0961145194274), G.perc = c(3.00429184549356, 
1.48698884758364, 94.0149625935162, 1.17508813160987, 4.03225806451613, 
2.64650283553875, 96.5352449223417, 96.3414634146341, 2.04081632653061, 
2.91595197255575, 3.43347639484979, 95.3947368421053, 1.61290322580645, 
0, 2.12314225053079, 93.9524838012959, 2.31884057971015, 95.7910014513788, 
2.49433106575964, 2.40700218818381, 2.09643605870021, 96.4705882352941, 
2.4390243902439, 95.427435387674, 1.53846153846154, 2.33766233766234, 
0, 0, 0, 1.42857142857143, 2.80612244897959, 1.15473441108545, 
95.9375, 98.7234042553192, 96.5087281795511, 1.6, 1.90930787589499, 
1.42602495543672, 2.1484375, 0.619834710743802, 1.74291938997821, 
94.5652173913043, 2.87162162162162, 0, 4.20560747663551, 92.8767123287671, 
1.05263157894737, 1.41509433962264, 93.3852140077821, 94.9392712550607, 
2.04778156996587, 0.793650793650794, 3.2967032967033, 93.9306358381503, 
1.44329896907216, 0, 90.8474576271186, 1.0989010989011, 1.09289617486339, 
2.01511335012594, 90.5723905723906, 0, 3.08641975308642, 5.53846153846154, 
96.1451247165533, 1.32978723404255, 0.209643605870021, 0, 0.186915887850467, 
3.84615384615385, 92.483660130719, 2.33160621761658, 89.4568690095847, 
2.40480961923848, 16.9734151329243), T.perc = c(1.07296137339056, 
1.48698884758364, 1.74563591022444, 1.17508813160987, 1.61290322580645, 
93.3837429111531, 0.716845878136201, 0, 3.2069970845481, 2.57289879931389, 
0, 1.75438596491228, 1.49769585253456, 2.75229357798165, 2.12314225053079, 
1.72786177105832, 95.3623188405797, 1.59651669085631, 96.3718820861678, 
97.3741794310722, 95.5974842767296, 1.17647058823529, 96.5853658536585, 
2.38568588469185, 3.73626373626374, 2.5974025974026, 2.16535433070866, 
95.3907815631263, 2.80561122244489, 3.26530612244898, 1.78571428571429, 
3.46420323325635, 0.625, 0.425531914893617, 0.997506234413965, 
1.86666666666667, 0, 1.96078431372549, 1.7578125, 2.47933884297521, 
94.5533769063181, 1.26811594202899, 0.337837837837838, 3.46938775510204, 
2.33644859813084, 0.547945205479452, 2.10526315789474, 3.30188679245283, 
0.778210116731518, 1.417004048583, 2.04778156996587, 1.19047619047619, 
0.879120879120879, 0.289017341040462, 1.85567010309278, 2.24215246636771, 
4.06779661016949, 96.1538461538462, 94.5355191256831, 2.01511335012594, 
1.68350168350168, 1.27041742286751, 1.85185185185185, 0, 0, 4.52127659574468, 
96.2264150943396, 3.13253012048193, 3.92523364485981, 4.12087912087912, 
1.30718954248366, 3.36787564766839, 2.55591054313099, 0, 4.49897750511247
), base.call = c("A", "A", "G", "A", "C", "T", "G", "G", "C", 
"C", "A", "G", "A", "C", "C", "G", "T", "G", "T", "T", "T", "G", 
"T", "G", "C", "A", "C", "T", "A", "C", "A", "C", "G", "G", "G", 
"C", "A", "C", "C", "C", "T", "G", "A", "C", "C", "G", "A", "C", 
"G", "G", "C", "A", "A", "G", "A", "A", "G", "T", "T", "C", "G", 
"A", "C", "A", "G", "C", "T", "C", "C", "C", "G", "C", "G", "A", 
"C"), index = 275:349, guide.seq = c("A", "A", "G", "A", "C", 
"T", "G", "G", "C", "C", "A", "G", "A", "C", "C", "G", "T", "G", 
"T", "T", "T", "G", "T", "G", "C", "A", "C", "T", "A", "C", "A", 
"C", "G", "G", "G", "C", "A", "C", "C", "C", "T", "G", "A", "C", 
"C", "G", "A", "C", "G", "G", "C", "A", "A", "G", "A", "A", "G", 
"T", "T", "C", "G", "A", "C", "A", "G", "C", "T", "C", "C", "C", 
"G", "C", "G", "A", "C"), T.pval = c(0.682304679580776, 0.540555926630336, 
0.457039282349677, 0.647251414034922, 0.499082088869334, 0, 0.796219888779355, 
0.903225806446376, 0.144063102187325, 0.246054459784107, 0.903225806446376, 
0.454336278788512, 0.536977760504365, 0.212406062567505, 0.349232837770535, 
0.462557170223642, 0, 0.504399064197535, 0, 0, 0, 0.64677470993852, 
0, 0.285646230446797, 0.0895750218458207, 0.241216112886518, 
0.338365703109663, 0, 0.203177602969928, 0.136869843912536, 0.444732402423289, 
0.114677427673973, 0.821455007478224, 0.866517068782555, 0.70782855928286, 
0.420468512514108, 0.903225806446376, 0.393306593760423, 0.453280211442661, 
0.265254010093402, 0, 0.615148078351937, 0.881123068364892, 0.11414486695536, 
0.296846188857751, 0.840614432007918, 0.353909848006819, 0.132521426733613, 
0.778137923961861, 0.564141542500153, 0.369244168723531, 0.641943591523219, 
0.74670313919618, 0.887680849460867, 0.423716592078725, 0.319227678902098, 
0.0658231981979596, 0, 0, 0.378160086778234, 0.476483883424669, 
0.614354417496513, 0.42484797275634, 0.903225806446376, 0.903225806446376, 
0.0427193361840881, 0, 0.153733575394696, 0.0752149888117173, 
0.0626136959346342, 0.601687915123266, 0.124987428564539, 0.249454817382917, 
0.903225806446376, 0.0436488345068677), C.pval = c(0.353768164205585, 
0.304553353793885, 0.146094215350016, 0.754274639442195, 0, 0.433832927780845, 
0.287595869815284, 0.484863445626618, 0, 0, 0.438073845249322, 
0.728889360616869, 0.596901167552595, 0, 0, 0.529136792556636, 
0.934332687245141, 0.344746765430015, 0.952941176470502, 0.952941176470502, 
0.18327460567878, 0.173304893947797, 0.952941176470502, 0.67313204569196, 
0, 0.0464069678218396, 0, 0.127277102195632, 0.396923559839861, 
0, 0.136142395637443, 0, 0.144980570486563, 0.741104734845, 0.551929899870514, 
0, 0.0916379251470882, 0, 0, 0, 0.272469160823351, 0.542011280248066, 
0.503823037594177, 0, 0, 0.151190514061729, 0.717064406612146, 
0, 0.109647787970571, 0.474603243144942, 0, 0.578567537942636, 
0.727983275536859, 0.856519333541375, 0.945272753286674, 0.898714347876366, 
0.111309533357646, 0.866708191499591, 0.211105082844917, 0, 0.366055854143368, 
0.213058090043485, 0, 0.103483157714504, 0.191826609709757, 0, 
0.109421153867976, 0, 0, 0, 0.082907427435098, 0, 0.135320651502905, 
0.480848713367323, 0), G.pval = c(0.162210588573086, 0.488125073815012, 
0, 0.584811175351147, 0.0681561024022392, 0.215424780834479, 
0, 0, 0.338040650779337, 0.174160256848712, 0.113877832335364, 
0, 0.451106793943814, 0.844444444444445, 0.318784696633306, 0, 
0.276335108545279, 0, 0.242175404130445, 0.258721011439306, 0.324940266027986, 
0, 0.252550780116941, 0, 0.472815094142304, 0.272496878021646, 
0.844444444444445, 0.844444444444445, 0.844444444444445, 0.505770793667676, 
0.190077556507543, 0.591264131648356, 0, 0, 0, 0.454831024515686, 
0.370514479072009, 0.506546075441812, 0.313034930937055, 0.753006520871132, 
0.414546515284238, 0, 0.18044041893825, 0.844444444444445, 0.0585323513668725, 
0, 0.623658981335124, 0.50987942176618, 0, 0, 0.336379354142041, 
0.703841585790433, 0.127650312658475, 0, 0.501296623383811, 0.844444444444445, 
0, 0.608984189559022, 0.610890036604514, 0.344222420264647, 0, 
0.844444444444445, 0.151752598799894, 0.0174175616692285, 0, 
0.536175592195204, 0.833654620308385, 0.844444444444445, 0.835969471297773, 
0.080119505971722, 0, 0.273727242518954, 0, 0.259147895055155, 
1.58234896407983e-07), A.pval = c(0, 0, 0.322447911721792, 0, 
0.0471219393114786, 0.162954540739551, 0.663224498002557, 0.197365703429265, 
0.524812735015408, 0.283340649879824, 0, 0.261230687470814, 0, 
0.0819566032144312, 0.095785473602551, 0.0907205805435722, 0.247914066364856, 
0.64843921428756, 0.537914355513364, 0.885907549480703, 0.909090909074362, 
0.909090909074362, 0.603735740335047, 0.51414523118769, 0.55218526415518, 
0, 0.443361254522065, 0.253857060325715, 0, 0.245139900435583, 
0, 0.440541179414608, 0.619934957477958, 0.909090909074362, 0.492867570899344, 
0.22433271009545, 0, 0.639686959228108, 0.447308376362149, 0.2905895711039, 
0.323240023420587, 0.10352759060786, 0, 0.201260307251772, 0.720798376233752, 
0.0275610074663253, 0, 0.111204133851505, 0.0825205655164186, 
0.204873863437722, 0.174510257236533, 0, 0, 0.00782460498400317, 
0, 0, 0.177350953868619, 0.210738173496138, 0.213215760692988, 
0.590236476122454, 0.00282793340819554, 0, 0.197643976875357, 
0, 0.371142859741404, 0.875193513419788, 0.662258736550098, 0.909090909074362, 
0.892127343259357, 0.782325056980262, 0.0698680811993262, 0.142384918594921, 
0.00597660911154629, 0, 0.424061809411506), guide.position = 1:75), .Names = c("A.area", 
"C.area", "G.area", "T.area", "Tot.area", "A.perc", "C.perc", 
"G.perc", "T.perc", "base.call", "index", "guide.seq", "T.pval", 
"C.pval", "G.pval", "A.pval", "guide.position"), row.names = 275:349, class = "data.frame")
```

*Report generated using EditR v1.0.8*
