# Supplementary material for: Mechanistic and genetic basis of single-strand templated repair at Cas12a-induced DNA breaks in Chlamydomonas reinhardtii
Source: Nat Commun. 2021 Nov 19;12:6751. doi: 10.1038/s41467-021-27004-1 (PMC8604939; doi:10.1038/s41467-021-27004-1)
Supplement: Supplementary file 22 — Source Data [file 41467_2021_27004_MOESM22_ESM.zip › Source Data/EditR analysis/EditR outputs/Sense/rep1_ssODN_sense_-32.html]

EditR v1.0.8 report


# EditR v1.0.8 report

- Data QA
  - Filtering data
  - Percent noise peak area
  - Base information
- Predicted editing
  - Editing bar plot
  - Editing table plot
  - Table of editing results
- For use in R

## Data QA

### Filtering data

What the data looked like prefiltering:

and the post filtering signal / noise plot:

### Percent noise peak area

### Base information

Here’s information about the signal of each base, the critical percent value where any higher value would be called as significant, and Filliben’s correlation for how well the noise was modelled by the zero adjusted gamma distribution.

| Base | Average percent signal | Average peak area | Critical percent value | model mu | Fillibens correlation |
| --- | --- | --- | --- | --- | --- |
| A | 92.16035 | 439.1429 | 6.972041 | 2.230797 | 0.9683120 |
| C | 92.10765 | 476.6515 | 5.908661 | 2.111546 | 0.9768258 |
| G | 93.12916 | 444.8947 | 11.340236 | 2.624130 | 0.9891428 |
| T | 94.20987 | 497.5581 | 8.099527 | 2.441309 | 0.9834871 |

## Predicted editing

### Editing bar plot

### Editing table plot

### Table of editing results


Here’s the entire guide region

| Sanger position | Guide position | Guide sequence | Sanger base call | Focal base | Focal base peak area | p value |  |
| --- | --- | --- | --- | --- | --- | --- | --- |
| 273 | 1 | A | A | A | 94.26 | 0.000000e+00 | \* |
| 273 | 1 | A | A | C | 1.91 | 4.403122e-01 |  |
| 273 | 1 | A | A | G | 2.34 | 3.905381e-01 |  |
| 273 | 1 | A | A | T | 1.49 | 6.249104e-01 |  |
| 274 | 2 | A | A | A | 94.72 | 0.000000e+00 | \* |
| 274 | 2 | A | A | C | 1.51 | 5.731675e-01 |  |
| 274 | 2 | A | A | G | 2.26 | 4.024636e-01 |  |
| 274 | 2 | A | A | T | 1.51 | 6.192796e-01 |  |
| 275 | 3 | G | G | A | 2.05 | 4.614485e-01 |  |
| 275 | 3 | G | G | C | 1.37 | 6.222365e-01 |  |
| 275 | 3 | G | G | G | 94.08 | 0.000000e+00 | \* |
| 275 | 3 | G | G | T | 2.51 | 3.746271e-01 |  |
| 276 | 4 | A | A | A | 95.02 | 0.000000e+00 | \* |
| 276 | 4 | A | A | C | 0.95 | 7.611966e-01 |  |
| 276 | 4 | A | A | G | 2.13 | 4.238323e-01 |  |
| 276 | 4 | A | A | T | 1.90 | 5.154233e-01 |  |
| 277 | 5 | C | C | A | 3.42 | 1.817231e-01 |  |
| 277 | 5 | C | C | C | 91.46 | 0.000000e+00 | \* |
| 277 | 5 | C | C | G | 3.04 | 2.963290e-01 |  |
| 277 | 5 | C | C | T | 2.09 | 4.678688e-01 |  |
| 278 | 6 | T | T | A | 3.10 | 2.280556e-01 |  |
| 278 | 6 | T | T | C | 1.38 | 6.179051e-01 |  |
| 278 | 6 | T | T | G | 2.59 | 3.543636e-01 |  |
| 278 | 6 | T | T | T | 92.93 | 0.000000e+00 | \* |
| 279 | 7 | G | G | A | 0.99 | 8.014552e-01 |  |
| 279 | 7 | G | G | C | 1.88 | 4.516131e-01 |  |
| 279 | 7 | G | G | G | 96.24 | 0.000000e+00 | \* |
| 279 | 7 | G | G | T | 0.88 | 7.973132e-01 |  |
| 280 | 8 | G | G | A | 2.08 | 4.521310e-01 |  |
| 280 | 8 | G | G | C | 0.80 | 8.036722e-01 |  |
| 280 | 8 | G | G | G | 95.99 | 0.000000e+00 | \* |
| 280 | 8 | G | G | T | 1.12 | 7.300099e-01 |  |
| 281 | 9 | C | C | A | 2.17 | 4.289461e-01 |  |
| 281 | 9 | C | C | C | 90.79 | 0.000000e+00 | \* |
| 281 | 9 | C | C | G | 2.71 | 3.373794e-01 |  |
| 281 | 9 | C | C | T | 4.34 | 1.260187e-01 |  |
| 282 | 10 | C | C | A | 3.24 | 2.072225e-01 |  |
| 282 | 10 | C | C | C | 91.91 | 0.000000e+00 | \* |
| 282 | 10 | C | C | G | 1.78 | 4.865932e-01 |  |
| 282 | 10 | C | C | T | 3.07 | 2.716869e-01 |  |
| 283 | 11 | A | A | A | 67.78 | 0.000000e+00 | \* |
| 283 | 11 | A | A | C | 1.87 | 4.539113e-01 |  |
| 283 | 11 | A | A | G | 2.49 | 3.674250e-01 |  |
| 283 | 11 | A | A | T | 27.86 | 3.641164e-09 | \* |
| 284 | 12 | G | G | A | 2.90 | 2.636902e-01 |  |
| 284 | 12 | G | G | C | 1.99 | 4.166954e-01 |  |
| 284 | 12 | G | G | G | 94.02 | 0.000000e+00 | \* |
| 284 | 12 | G | G | T | 1.09 | 7.400049e-01 |  |
| 285 | 13 | A | A | A | 95.12 | 0.000000e+00 | \* |
| 285 | 13 | A | A | C | 1.41 | 6.059915e-01 |  |
| 285 | 13 | A | A | G | 1.93 | 4.592537e-01 |  |
| 285 | 13 | A | A | T | 1.54 | 6.100679e-01 |  |
| 286 | 14 | C | C | A | 3.50 | 1.711458e-01 |  |
| 286 | 14 | C | C | C | 93.36 | 0.000000e+00 | \* |
| 286 | 14 | C | C | G | 0.00 | 9.333333e-01 |  |
| 286 | 14 | C | C | T | 3.15 | 2.604528e-01 |  |
| 287 | 15 | C | C | A | 3.47 | 1.739137e-01 |  |
| 287 | 15 | C | C | C | 90.93 | 0.000000e+00 | \* |
| 287 | 15 | C | C | G | 2.90 | 3.133656e-01 |  |
| 287 | 15 | C | C | T | 2.70 | 3.359212e-01 |  |
| 288 | 16 | G | G | A | 2.47 | 3.521180e-01 |  |
| 288 | 16 | G | G | C | 1.65 | 5.257663e-01 |  |
| 288 | 16 | G | G | G | 93.61 | 0.000000e+00 | \* |
| 288 | 16 | G | G | T | 2.27 | 4.257537e-01 |  |
| 289 | 17 | T | T | A | 0.00 | 9.999993e-01 |  |
| 289 | 17 | T | T | C | 0.54 | 8.641058e-01 |  |
| 289 | 17 | T | T | G | 3.50 | 2.457560e-01 |  |
| 289 | 17 | T | T | T | 95.96 | 0.000000e+00 | \* |
| 290 | 18 | G | G | A | 1.13 | 7.574989e-01 |  |
| 290 | 18 | G | G | C | 1.13 | 7.039049e-01 |  |
| 290 | 18 | G | G | G | 95.35 | 0.000000e+00 | \* |
| 290 | 18 | G | G | T | 2.39 | 3.979718e-01 |  |
| 291 | 19 | T | T | A | 0.00 | 9.999993e-01 |  |
| 291 | 19 | T | T | C | 0.42 | 8.828327e-01 |  |
| 291 | 19 | T | T | G | 3.16 | 2.815147e-01 |  |
| 291 | 19 | T | T | T | 96.41 | 0.000000e+00 | \* |
| 292 | 20 | T | T | A | 0.61 | 9.151393e-01 |  |
| 292 | 20 | T | T | C | 1.64 | 5.291572e-01 |  |
| 292 | 20 | T | T | G | 0.00 | 9.333333e-01 |  |
| 292 | 20 | T | T | T | 97.75 | 0.000000e+00 | \* |
| 293 | 21 | T | T | A | 0.00 | 9.999993e-01 |  |
| 293 | 21 | T | T | C | 1.61 | 5.391259e-01 |  |
| 293 | 21 | T | T | G | 3.42 | 2.541196e-01 |  |
| 293 | 21 | T | T | T | 94.97 | 0.000000e+00 | \* |
| 294 | 22 | G | G | A | 0.49 | 9.459326e-01 |  |
| 294 | 22 | G | G | C | 0.00 | 9.058824e-01 |  |
| 294 | 22 | G | G | G | 97.73 | 0.000000e+00 | \* |
| 294 | 22 | G | G | T | 1.78 | 5.447707e-01 |  |
| 295 | 23 | T | T | A | 1.30 | 7.003320e-01 |  |
| 295 | 23 | T | T | C | 1.94 | 4.314449e-01 |  |
| 295 | 23 | T | T | G | 4.54 | 1.622217e-01 |  |
| 295 | 23 | T | T | T | 92.22 | 0.000000e+00 | \* |
| 296 | 24 | G | G | A | 0.75 | 8.784275e-01 |  |
| 296 | 24 | G | G | C | 1.50 | 5.779970e-01 |  |
| 296 | 24 | G | G | G | 96.07 | 0.000000e+00 | \* |
| 296 | 24 | G | G | T | 1.68 | 5.716587e-01 |  |
| 297 | 25 | C | C | A | 1.91 | 5.030469e-01 |  |
| 297 | 25 | C | C | C | 91.95 | 0.000000e+00 | \* |
| 297 | 25 | C | C | G | 2.33 | 3.920693e-01 |  |
| 297 | 25 | C | C | T | 3.81 | 1.745498e-01 |  |
| 298 | 26 | A | A | A | 91.18 | 0.000000e+00 | \* |
| 298 | 26 | A | A | C | 3.74 | 9.335762e-02 |  |
| 298 | 26 | A | A | G | 2.14 | 4.227781e-01 |  |
| 298 | 26 | A | A | T | 2.94 | 2.934273e-01 |  |
| 299 | 27 | C | C | A | 1.90 | 5.058058e-01 |  |
| 299 | 27 | C | C | C | 95.07 | 0.000000e+00 | \* |
| 299 | 27 | C | C | G | 0.00 | 9.333333e-01 |  |
| 299 | 27 | C | C | T | 3.04 | 2.778051e-01 |  |
| 300 | 28 | T | T | A | 2.25 | 4.069301e-01 |  |
| 300 | 28 | T | T | C | 3.19 | 1.563463e-01 |  |
| 300 | 28 | T | T | G | 0.00 | 9.333333e-01 |  |
| 300 | 28 | T | T | T | 94.56 | 0.000000e+00 | \* |
| 301 | 29 | A | A | A | 95.20 | 0.000000e+00 | \* |
| 301 | 29 | A | A | C | 2.62 | 2.561491e-01 |  |
| 301 | 29 | A | A | G | 0.00 | 9.333333e-01 |  |
| 301 | 29 | A | A | T | 2.18 | 4.451307e-01 |  |
| 302 | 30 | C | C | A | 2.50 | 3.461561e-01 |  |
| 302 | 30 | C | C | C | 91.54 | 0.000000e+00 | \* |
| 302 | 30 | C | C | G | 2.50 | 3.666701e-01 |  |
| 302 | 30 | C | C | T | 3.46 | 2.161457e-01 |  |
| 303 | 31 | A | A | A | 92.52 | 0.000000e+00 | \* |
| 303 | 31 | A | A | C | 3.60 | 1.068795e-01 |  |
| 303 | 31 | A | A | G | 3.88 | 2.114784e-01 |  |
| 303 | 31 | A | A | T | 0.00 | 9.574468e-01 |  |
| 304 | 32 | C | C | A | 1.55 | 6.157007e-01 |  |
| 304 | 32 | C | C | C | 92.26 | 0.000000e+00 | \* |
| 304 | 32 | C | C | G | 2.43 | 3.764230e-01 |  |
| 304 | 32 | C | C | T | 3.76 | 1.802603e-01 |  |
| 305 | 33 | G | G | A | 0.88 | 8.366837e-01 |  |
| 305 | 33 | G | G | C | 2.36 | 3.160793e-01 |  |
| 305 | 33 | G | G | G | 96.76 | 0.000000e+00 | \* |
| 305 | 33 | G | G | T | 0.00 | 9.574468e-01 |  |
| 306 | 34 | G | G | A | 0.42 | 9.586719e-01 |  |
| 306 | 34 | G | G | C | 0.00 | 9.058824e-01 |  |
| 306 | 34 | G | G | G | 98.94 | 0.000000e+00 | \* |
| 306 | 34 | G | G | T | 0.64 | 8.622467e-01 |  |
| 307 | 35 | G | G | A | 2.20 | 4.217018e-01 |  |
| 307 | 35 | G | G | C | 1.71 | 5.065795e-01 |  |
| 307 | 35 | G | G | G | 95.61 | 0.000000e+00 | \* |
| 307 | 35 | G | G | T | 0.49 | 8.966450e-01 |  |
| 308 | 36 | C | C | A | 3.69 | 1.476068e-01 |  |
| 308 | 36 | C | C | C | 92.08 | 0.000000e+00 | \* |
| 308 | 36 | C | C | G | 1.85 | 4.740350e-01 |  |
| 308 | 36 | C | C | T | 2.37 | 4.022131e-01 |  |
| 309 | 37 | A | A | A | 93.48 | 0.000000e+00 | \* |
| 309 | 37 | A | A | C | 4.01 | 7.209646e-02 |  |
| 309 | 37 | A | A | G | 0.25 | 8.628909e-01 |  |
| 309 | 37 | A | A | T | 2.26 | 4.285550e-01 |  |
| 310 | 38 | C | C | A | 1.89 | 5.070756e-01 |  |
| 310 | 38 | C | C | C | 93.63 | 0.000000e+00 | \* |
| 310 | 38 | C | C | G | 0.52 | 7.856720e-01 |  |
| 310 | 38 | C | C | T | 3.96 | 1.596005e-01 |  |
| 311 | 39 | C | C | A | 2.25 | 4.080238e-01 |  |
| 311 | 39 | C | C | C | 92.32 | 0.000000e+00 | \* |
| 311 | 39 | C | C | G | 2.06 | 4.361207e-01 |  |
| 311 | 39 | C | C | T | 3.37 | 2.281955e-01 |  |
| 312 | 40 | C | C | A | 3.37 | 1.875103e-01 |  |
| 312 | 40 | C | C | C | 92.06 | 0.000000e+00 | \* |
| 312 | 40 | C | C | G | 0.60 | 7.634743e-01 |  |
| 312 | 40 | C | C | T | 3.97 | 1.586575e-01 |  |
| 313 | 41 | T | T | A | 2.42 | 3.650717e-01 |  |
| 313 | 41 | T | T | C | 3.43 | 1.257533e-01 |  |
| 313 | 41 | T | T | G | 2.42 | 3.785526e-01 |  |
| 313 | 41 | T | T | T | 91.73 | 0.000000e+00 | \* |
| 314 | 42 | G | G | A | 4.50 | 7.906010e-02 |  |
| 314 | 42 | G | G | C | 2.42 | 3.008725e-01 |  |
| 314 | 42 | G | G | G | 91.00 | 0.000000e+00 | \* |
| 314 | 42 | G | G | T | 2.08 | 4.705590e-01 |  |
| 315 | 43 | A | A | A | 93.09 | 0.000000e+00 | \* |
| 315 | 43 | A | A | C | 2.73 | 2.341903e-01 |  |
| 315 | 43 | A | A | G | 2.91 | 3.117061e-01 |  |
| 315 | 43 | A | A | T | 1.27 | 6.865918e-01 |  |
| 316 | 44 | C | C | A | 3.47 | 1.739137e-01 |  |
| 316 | 44 | C | C | C | 89.96 | 0.000000e+00 | \* |
| 316 | 44 | C | C | G | 2.90 | 3.133656e-01 |  |
| 316 | 44 | C | C | T | 3.67 | 1.908012e-01 |  |
| 317 | 45 | C | C | A | 2.41 | 3.680369e-01 |  |
| 317 | 45 | C | C | C | 88.62 | 0.000000e+00 | \* |
| 317 | 45 | C | C | G | 5.25 | 1.213777e-01 |  |
| 317 | 45 | C | C | T | 3.72 | 1.848529e-01 |  |
| 318 | 46 | G | G | A | 6.05 | 2.200034e-02 |  |
| 318 | 46 | G | G | C | 4.74 | 3.469612e-02 |  |
| 318 | 46 | G | G | G | 88.16 | 1.110223e-16 | \* |
| 318 | 46 | G | G | T | 1.05 | 7.498239e-01 |  |
| 319 | 47 | A | A | A | 93.52 | 0.000000e+00 | \* |
| 319 | 47 | A | A | C | 2.33 | 3.246907e-01 |  |
| 319 | 47 | A | A | G | 1.66 | 5.096138e-01 |  |
| 319 | 47 | A | A | T | 2.49 | 3.775041e-01 |  |
| 320 | 48 | C | C | A | 3.94 | 1.219590e-01 |  |
| 320 | 48 | C | C | C | 89.79 | 0.000000e+00 | \* |
| 320 | 48 | C | C | G | 2.32 | 3.936694e-01 |  |
| 320 | 48 | C | C | T | 3.94 | 1.610279e-01 |  |
| 321 | 49 | G | G | A | 5.21 | 4.451377e-02 |  |
| 321 | 49 | G | G | C | 4.51 | 4.358881e-02 |  |
| 321 | 49 | G | G | G | 89.58 | 1.110223e-16 | \* |
| 321 | 49 | G | G | T | 0.69 | 8.478542e-01 |  |
| 322 | 50 | G | G | A | 3.44 | 1.782992e-01 |  |
| 322 | 50 | G | G | C | 3.04 | 1.792564e-01 |  |
| 322 | 50 | G | G | G | 93.52 | 0.000000e+00 | \* |
| 322 | 50 | G | G | T | 0.00 | 9.574468e-01 |  |
| 323 | 51 | C | C | A | 5.69 | 2.996298e-02 |  |
| 323 | 51 | C | C | C | 88.96 | 0.000000e+00 | \* |
| 323 | 51 | C | C | G | 2.68 | 3.420234e-01 |  |
| 323 | 51 | C | C | T | 2.68 | 3.410529e-01 |  |
| 324 | 52 | A | A | A | 94.51 | 0.000000e+00 | \* |
| 324 | 52 | A | A | C | 2.35 | 3.178085e-01 |  |
| 324 | 52 | A | A | G | 1.96 | 4.534024e-01 |  |
| 324 | 52 | A | A | T | 1.18 | 7.142872e-01 |  |
| 325 | 53 | A | A | A | 92.97 | 0.000000e+00 | \* |
| 325 | 53 | A | A | C | 1.41 | 6.089991e-01 |  |
| 325 | 53 | A | A | G | 4.68 | 1.527844e-01 |  |
| 325 | 53 | A | A | T | 0.94 | 7.826252e-01 |  |
| 326 | 54 | G | G | A | 8.04 | 3.906589e-03 | \* |
| 326 | 54 | G | G | C | 0.54 | 8.646350e-01 |  |
| 326 | 54 | G | G | G | 90.62 | 0.000000e+00 | \* |
| 326 | 54 | G | G | T | 0.80 | 8.190480e-01 |  |
| 327 | 55 | A | A | A | 94.76 | 0.000000e+00 | \* |
| 327 | 55 | A | A | C | 0.66 | 8.402638e-01 |  |
| 327 | 55 | A | A | G | 3.06 | 2.938908e-01 |  |
| 327 | 55 | A | A | T | 1.53 | 6.139806e-01 |  |
| 328 | 56 | A | A | A | 93.49 | 0.000000e+00 | \* |
| 328 | 56 | A | A | C | 0.24 | 9.004377e-01 |  |
| 328 | 56 | A | A | G | 2.41 | 3.800088e-01 |  |
| 328 | 56 | A | A | T | 3.86 | 1.701132e-01 |  |
| 329 | 57 | G | G | A | 5.32 | 4.074969e-02 |  |
| 329 | 57 | G | G | C | 2.66 | 2.482435e-01 |  |
| 329 | 57 | G | G | G | 86.71 | 2.220446e-16 | \* |
| 329 | 57 | G | G | T | 5.32 | 6.693233e-02 |  |
| 330 | 58 | T | T | A | 0.00 | 9.999993e-01 |  |
| 330 | 58 | T | T | C | 0.28 | 8.977843e-01 |  |
| 330 | 58 | T | T | G | 1.96 | 4.534024e-01 |  |
| 330 | 58 | T | T | T | 97.76 | 0.000000e+00 | \* |
| 331 | 59 | T | T | A | 2.96 | 2.530800e-01 |  |
| 331 | 59 | T | T | C | 3.76 | 9.157403e-02 |  |
| 331 | 59 | T | T | G | 0.81 | 7.062158e-01 |  |
| 331 | 59 | T | T | T | 92.47 | 0.000000e+00 | \* |
| 332 | 60 | C | C | A | 2.03 | 4.670204e-01 |  |
| 332 | 60 | C | C | C | 91.12 | 0.000000e+00 | \* |
| 332 | 60 | C | C | G | 4.82 | 1.444550e-01 |  |
| 332 | 60 | C | C | T | 2.03 | 4.816717e-01 |  |
| 333 | 61 | G | G | A | 8.58 | 2.418915e-03 | \* |
| 333 | 61 | G | G | C | 2.97 | 1.900033e-01 |  |
| 333 | 61 | G | G | G | 87.79 | 1.110223e-16 | \* |
| 333 | 61 | G | G | T | 0.66 | 8.565238e-01 |  |
| 334 | 62 | A | A | A | 92.43 | 0.000000e+00 | \* |
| 334 | 62 | A | A | C | 3.50 | 1.180682e-01 |  |
| 334 | 62 | A | A | G | 2.52 | 3.631643e-01 |  |
| 334 | 62 | A | A | T | 1.55 | 6.070127e-01 |  |
| 335 | 63 | C | C | A | 5.36 | 3.912696e-02 |  |
| 335 | 63 | C | C | C | 92.27 | 0.000000e+00 | \* |
| 335 | 63 | C | C | G | 0.00 | 9.333333e-01 |  |
| 335 | 63 | C | C | T | 2.36 | 4.052819e-01 |  |
| 336 | 64 | A | A | A | 85.27 | 0.000000e+00 | \* |
| 336 | 64 | A | A | C | 3.76 | 9.172221e-02 |  |
| 336 | 64 | A | A | G | 9.09 | 2.525694e-02 |  |
| 336 | 64 | A | A | T | 1.88 | 5.192313e-01 |  |
| 337 | 65 | G | G | A | 2.01 | 4.731720e-01 |  |
| 337 | 65 | G | G | C | 4.91 | 2.897931e-02 |  |
| 337 | 65 | G | G | G | 91.52 | 0.000000e+00 | \* |
| 337 | 65 | G | G | T | 1.56 | 6.044851e-01 |  |
| 338 | 66 | C | C | A | 1.86 | 5.180748e-01 |  |
| 338 | 66 | C | C | C | 90.45 | 0.000000e+00 | \* |
| 338 | 66 | C | C | G | 1.86 | 4.722248e-01 |  |
| 338 | 66 | C | C | T | 5.84 | 4.739614e-02 |  |
| 339 | 67 | T | T | A | 0.42 | 9.600450e-01 |  |
| 339 | 67 | T | T | C | 5.64 | 1.342597e-02 |  |
| 339 | 67 | T | T | G | 1.25 | 5.965861e-01 |  |
| 339 | 67 | T | T | T | 92.69 | 0.000000e+00 | \* |
| 340 | 68 | C | C | A | 2.61 | 3.223154e-01 |  |
| 340 | 68 | C | C | C | 92.42 | 0.000000e+00 | \* |
| 340 | 68 | C | C | G | 0.47 | 7.977717e-01 |  |
| 340 | 68 | C | C | T | 4.50 | 1.133919e-01 |  |
| 341 | 69 | C | C | A | 2.05 | 4.608488e-01 |  |
| 341 | 69 | C | C | C | 94.03 | 0.000000e+00 | \* |
| 341 | 69 | C | C | G | 0.37 | 8.269246e-01 |  |
| 341 | 69 | C | C | T | 3.54 | 2.055882e-01 |  |
| 342 | 70 | C | C | A | 2.73 | 2.959178e-01 |  |
| 342 | 70 | C | C | C | 87.98 | 0.000000e+00 | \* |
| 342 | 70 | C | C | G | 6.83 | 6.379605e-02 |  |
| 342 | 70 | C | C | T | 2.46 | 3.842851e-01 |  |
| 343 | 71 | G | G | A | 5.72 | 2.904253e-02 |  |
| 343 | 71 | G | G | C | 5.42 | 1.690882e-02 |  |
| 343 | 71 | G | G | G | 87.95 | 1.110223e-16 | \* |
| 343 | 71 | G | G | T | 0.90 | 7.918732e-01 |  |
| 344 | 72 | C | C | A | 6.98 | 9.909265e-03 | \* |
| 344 | 72 | C | C | C | 84.29 | 0.000000e+00 | \* |
| 344 | 72 | C | C | G | 5.99 | 9.007785e-02 |  |
| 344 | 72 | C | C | T | 2.74 | 3.283833e-01 |  |
| 345 | 73 | G | G | A | 10.81 | 3.188835e-04 | \* |
| 345 | 73 | G | G | C | 4.50 | 4.400639e-02 |  |
| 345 | 73 | G | G | G | 83.48 | 7.771561e-16 | \* |
| 345 | 73 | G | G | T | 1.20 | 7.071690e-01 |  |
| 346 | 74 | A | A | A | 88.26 | 0.000000e+00 | \* |
| 346 | 74 | A | A | C | 5.45 | 1.639227e-02 |  |
| 346 | 74 | A | A | G | 2.94 | 3.085054e-01 |  |
| 346 | 74 | A | A | T | 3.35 | 2.304467e-01 |  |
| 347 | 75 | C | C | A | 3.61 | 1.575638e-01 |  |
| 347 | 75 | C | C | C | 88.98 | 0.000000e+00 | \* |
| 347 | 75 | C | C | G | 2.20 | 4.120455e-01 |  |
| 347 | 75 | C | C | T | 5.21 | 7.172214e-02 |  |

## For use in R

If you want to work with the results in R, here is output that you can copy and paste in your terminal to get:

The base information:

```
structure(list(focal.base = c("A", "C", "G", "T"), avg.percsignal = c(92.1603539343091, 
92.1076472931577, 93.1291576655272, 94.2098743832403), avg.areasignal = c(439.142857142857, 
476.651515151515, 444.894736842105, 497.558139534884), crit.perc.area = c(6.97204051201733, 
5.90866112987431, 11.3402354707023, 8.09952743685313), mu = c(2.23079658228059, 
2.11154585592288, 2.62413044439866, 2.4413086437878), fillibens = c(0.968312030955591, 
0.976825785437065, 0.989142841371591, 0.983487122506658)), .Names = c("focal.base", 
"avg.percsignal", "avg.areasignal", "crit.perc.area", "mu", "fillibens"
), row.names = c(NA, -4L), class = "data.frame")
```

the data.frame that contains information on the guide region:

```
structure(list(A.area = c(443, 502, 9, 802, 18, 18, 9, 13, 8, 
20, 326, 16, 740, 20, 18, 12, 0, 8, 0, 3, 0, 3, 6, 4, 9, 341, 
10, 12, 436, 13, 334, 7, 3, 2, 9, 14, 373, 11, 12, 17, 12, 26, 
512, 18, 11, 23, 563, 17, 15, 17, 17, 241, 397, 30, 434, 388, 
16, 0, 11, 8, 26, 476, 25, 272, 9, 7, 2, 11, 11, 10, 19, 28, 
36, 421, 18), C.area = c(9, 8, 6, 8, 482, 8, 17, 5, 335, 568, 
9, 11, 11, 534, 471, 8, 2, 8, 2, 8, 8, 0, 9, 8, 434, 14, 501, 
17, 12, 476, 13, 417, 8, 0, 7, 349, 16, 544, 493, 464, 17, 14, 
15, 466, 405, 18, 14, 387, 13, 15, 266, 6, 6, 2, 3, 1, 8, 1, 
14, 359, 9, 18, 430, 12, 22, 341, 27, 390, 504, 322, 18, 338, 
15, 26, 444), G.area = c(11, 12, 413, 18, 16, 15, 871, 599, 10, 
11, 12, 519, 15, 0, 15, 454, 13, 677, 15, 0, 17, 603, 21, 514, 
11, 8, 0, 0, 0, 13, 14, 11, 328, 466, 392, 7, 1, 3, 11, 3, 12, 
526, 16, 15, 24, 335, 10, 10, 258, 462, 8, 5, 20, 338, 14, 10, 
261, 7, 3, 19, 266, 13, 0, 29, 410, 7, 6, 2, 2, 25, 292, 24, 
278, 14, 11), T.area = c(7, 8, 11, 16, 11, 539, 8, 7, 16, 19, 
134, 6, 12, 18, 14, 11, 356, 17, 457, 477, 472, 11, 427, 9, 18, 
11, 16, 504, 10, 18, 0, 17, 0, 3, 2, 9, 9, 23, 18, 20, 455, 12, 
7, 19, 17, 4, 15, 17, 2, 0, 8, 3, 4, 3, 7, 16, 16, 349, 344, 
8, 2, 8, 11, 6, 7, 22, 444, 19, 19, 9, 3, 11, 4, 16, 26), Tot.area = c(470, 
530, 439, 844, 527, 580, 905, 624, 369, 618, 481, 552, 778, 572, 
518, 485, 371, 710, 474, 488, 497, 617, 463, 535, 472, 374, 527, 
533, 458, 520, 361, 452, 339, 471, 410, 379, 399, 581, 534, 504, 
496, 578, 550, 518, 457, 380, 602, 431, 288, 494, 299, 255, 427, 
373, 458, 415, 301, 357, 372, 394, 303, 515, 466, 319, 448, 377, 
479, 422, 536, 366, 332, 401, 333, 477, 499), A.perc = c(94.2553191489362, 
94.7169811320755, 2.0501138952164, 95.0236966824645, 3.41555977229602, 
3.10344827586207, 0.994475138121547, 2.08333333333333, 2.1680216802168, 
3.23624595469256, 67.7754677754678, 2.89855072463768, 95.1156812339332, 
3.4965034965035, 3.47490347490348, 2.47422680412371, 0, 1.12676056338028, 
0, 0.614754098360656, 0, 0.486223662884927, 1.29589632829374, 
0.747663551401869, 1.90677966101695, 91.1764705882353, 1.89753320683112, 
2.25140712945591, 95.1965065502183, 2.5, 92.5207756232687, 1.54867256637168, 
0.884955752212389, 0.424628450106157, 2.19512195121951, 3.69393139841689, 
93.483709273183, 1.89328743545611, 2.24719101123596, 3.37301587301587, 
2.41935483870968, 4.49826989619377, 93.0909090909091, 3.47490347490348, 
2.40700218818381, 6.05263157894737, 93.5215946843854, 3.94431554524362, 
5.20833333333333, 3.4412955465587, 5.68561872909699, 94.5098039215686, 
92.9742388758782, 8.04289544235925, 94.7598253275109, 93.4939759036145, 
5.3156146179402, 0, 2.95698924731183, 2.03045685279188, 8.58085808580858, 
92.4271844660194, 5.36480686695279, 85.2664576802508, 2.00892857142857, 
1.85676392572944, 0.417536534446764, 2.60663507109005, 2.05223880597015, 
2.73224043715847, 5.72289156626506, 6.98254364089776, 10.8108108108108, 
88.2599580712788, 3.60721442885772), C.perc = c(1.91489361702128, 
1.50943396226415, 1.36674259681093, 0.947867298578199, 91.46110056926, 
1.37931034482759, 1.87845303867403, 0.801282051282051, 90.7859078590786, 
91.9093851132686, 1.87110187110187, 1.99275362318841, 1.41388174807198, 
93.3566433566434, 90.9266409266409, 1.64948453608247, 0.539083557951482, 
1.12676056338028, 0.421940928270042, 1.63934426229508, 1.60965794768612, 
0, 1.9438444924406, 1.49532710280374, 91.9491525423729, 3.74331550802139, 
95.0664136622391, 3.18949343339587, 2.62008733624454, 91.5384615384615, 
3.601108033241, 92.2566371681416, 2.3598820058997, 0, 1.70731707317073, 
92.0844327176781, 4.01002506265664, 93.631669535284, 92.3220973782771, 
92.0634920634921, 3.42741935483871, 2.42214532871972, 2.72727272727273, 
89.96138996139, 88.6214442013129, 4.73684210526316, 2.32558139534884, 
89.7911832946636, 4.51388888888889, 3.03643724696356, 88.9632107023411, 
2.35294117647059, 1.40515222482436, 0.536193029490617, 0.655021834061135, 
0.240963855421687, 2.6578073089701, 0.280112044817927, 3.76344086021505, 
91.1167512690355, 2.97029702970297, 3.49514563106796, 92.274678111588, 
3.76175548589342, 4.91071428571429, 90.4509283819629, 5.63674321503132, 
92.4170616113744, 94.0298507462687, 87.9781420765027, 5.42168674698795, 
84.28927680798, 4.5045045045045, 5.45073375262055, 88.9779559118236
), G.perc = c(2.34042553191489, 2.26415094339623, 94.0774487471526, 
2.13270142180095, 3.03605313092979, 2.58620689655172, 96.2430939226519, 
95.9935897435897, 2.710027100271, 1.77993527508091, 2.49480249480249, 
94.0217391304348, 1.9280205655527, 0, 2.8957528957529, 93.6082474226804, 
3.50404312668464, 95.3521126760563, 3.16455696202532, 0, 3.420523138833, 
97.7309562398703, 4.53563714902808, 96.0747663551402, 2.33050847457627, 
2.13903743315508, 0, 0, 0, 2.5, 3.8781163434903, 2.43362831858407, 
96.7551622418879, 98.9384288747346, 95.609756097561, 1.84696569920844, 
0.25062656641604, 0.516351118760757, 2.05992509363296, 0.595238095238095, 
2.41935483870968, 91.0034602076125, 2.90909090909091, 2.8957528957529, 
5.25164113785558, 88.1578947368421, 1.66112956810631, 2.32018561484919, 
89.5833333333333, 93.5222672064777, 2.67558528428094, 1.96078431372549, 
4.68384074941452, 90.6166219839142, 3.05676855895197, 2.40963855421687, 
86.7109634551495, 1.96078431372549, 0.806451612903226, 4.82233502538071, 
87.7887788778878, 2.52427184466019, 0, 9.09090909090909, 91.5178571428571, 
1.85676392572944, 1.25260960334029, 0.4739336492891, 0.373134328358209, 
6.83060109289617, 87.9518072289157, 5.98503740648379, 83.4834834834835, 
2.93501048218029, 2.20440881763527), T.perc = c(1.48936170212766, 
1.50943396226415, 2.50569476082005, 1.8957345971564, 2.08728652751423, 
92.9310344827586, 0.883977900552486, 1.12179487179487, 4.3360433604336, 
3.07443365695793, 27.8586278586279, 1.08695652173913, 1.54241645244216, 
3.14685314685315, 2.7027027027027, 2.2680412371134, 95.9568733153639, 
2.3943661971831, 96.4135021097046, 97.7459016393443, 94.9698189134809, 
1.78282009724473, 92.2246220302376, 1.68224299065421, 3.8135593220339, 
2.94117647058824, 3.03605313092979, 94.5590994371482, 2.18340611353712, 
3.46153846153846, 0, 3.76106194690265, 0, 0.636942675159236, 
0.48780487804878, 2.37467018469657, 2.25563909774436, 3.95869191049914, 
3.37078651685393, 3.96825396825397, 91.7338709677419, 2.07612456747405, 
1.27272727272727, 3.66795366795367, 3.7199124726477, 1.05263157894737, 
2.49169435215947, 3.94431554524362, 0.694444444444444, 0, 2.67558528428094, 
1.17647058823529, 0.936768149882904, 0.804289544235925, 1.52838427947598, 
3.85542168674699, 5.3156146179402, 97.7591036414566, 92.4731182795699, 
2.03045685279188, 0.66006600660066, 1.55339805825243, 2.36051502145923, 
1.88087774294671, 1.5625, 5.83554376657825, 92.6931106471816, 
4.50236966824645, 3.54477611940298, 2.45901639344262, 0.903614457831325, 
2.7431421446384, 1.2012012012012, 3.35429769392034, 5.21042084168337
), base.call = c("A", "A", "G", "A", "C", "T", "G", "G", "C", 
"C", "A", "G", "A", "C", "C", "G", "T", "G", "T", "T", "T", "G", 
"T", "G", "C", "A", "C", "T", "A", "C", "A", "C", "G", "G", "G", 
"C", "A", "C", "C", "C", "T", "G", "A", "C", "C", "G", "A", "C", 
"G", "G", "C", "A", "A", "G", "A", "A", "G", "T", "T", "C", "G", 
"A", "C", "A", "G", "C", "T", "C", "C", "C", "G", "C", "G", "A", 
"C"), index = 273:347, guide.seq = c("A", "A", "G", "A", "C", 
"T", "G", "G", "C", "C", "A", "G", "A", "C", "C", "G", "T", "G", 
"T", "T", "T", "G", "T", "G", "C", "A", "C", "T", "A", "C", "A", 
"C", "G", "G", "G", "C", "A", "C", "C", "C", "T", "G", "A", "C", 
"C", "G", "A", "C", "G", "G", "C", "A", "A", "G", "A", "A", "G", 
"T", "T", "C", "G", "A", "C", "A", "G", "C", "T", "C", "C", "C", 
"G", "C", "G", "A", "C"), T.pval = c(0.62491035005752, 0.619279557747799, 
0.374627063306492, 0.515423344512955, 0.467868844922092, 0, 0.797313206929335, 
0.730009881035962, 0.126018689111077, 0.271686913714369, 3.64116425899397e-09, 
0.740004860199231, 0.61006792517343, 0.260452848382303, 0.335921172942592, 
0.425753683443162, 0, 0.397971760381156, 0, 0, 0, 0.54477072718158, 
0, 0.571658651756151, 0.174549803640149, 0.293427340528987, 0.277805093482965, 
0, 0.445130689462259, 0.216145668761384, 0.957446808510052, 0.180260321444616, 
0.957446808510052, 0.862246684884306, 0.896644975515746, 0.402213066143417, 
0.428555002259507, 0.159600524877681, 0.228195524382575, 0.158657504269133, 
0, 0.470559033232028, 0.686591814159723, 0.190801197377308, 0.184852929269453, 
0.749823887862436, 0.377504143764375, 0.16102789985431, 0.847854172645748, 
0.957446808510052, 0.341052924418234, 0.714287239318929, 0.782625208596409, 
0.819047984915694, 0.613980558659864, 0.170113249958461, 0.0669323328269243, 
0, 0, 0.481671683786744, 0.856523782387887, 0.607012707542956, 
0.405281855674898, 0.51923126314911, 0.604485079614061, 0.0473961382633445, 
0, 0.113391854609164, 0.205588211927767, 0.384285118828333, 0.791873174213512, 
0.328383349733234, 0.707168957684461, 0.230446695809862, 0.0717221394841185
), C.pval = c(0.440312225253716, 0.573167538643013, 0.622236502295237, 
0.761196603392095, 0, 0.6179051427404, 0.45161314899631, 0.803672197719638, 
0, 0, 0.4539112767586, 0.416695438899617, 0.605991497362977, 
0, 0, 0.525766306266042, 0.864105755821846, 0.703904850750261, 
0.882832727563497, 0.529157193434247, 0.539125878223934, 0.905882352932355, 
0.431444874990715, 0.577997001430159, 0, 0.093357621445748, 0, 
0.156346335569585, 0.256149094143316, 0, 0.10687950841845, 0, 
0.316079344910358, 0.905882352932355, 0.506579471923296, 0, 0.0720964603363777, 
0, 0, 0, 0.125753271795937, 0.300872498841314, 0.234190310816124, 
0, 0, 0.0346961249401454, 0.324690671565111, 0, 0.043588813378012, 
0.179256428907229, 0, 0.31780846765587, 0.608999140570858, 0.864634972739297, 
0.840263762778281, 0.900437685631437, 0.248243492003814, 0.89778431587697, 
0.0915740343228799, 0, 0.190003272647487, 0.118068208156729, 
0, 0.0917222077095285, 0.0289793118017405, 0, 0.0134259705648493, 
0, 0, 0, 0.0169088164618691, 0, 0.0440063926928185, 0.0163922728849426, 
0), G.pval = c(0.390538062794982, 0.402463639298176, 0, 0.423832309733872, 
0.29632902392455, 0.354363559084945, 0, 0, 0.33737938089945, 
0.486593181276968, 0.367425020337097, 0, 0.459253717729811, 0.933333333333334, 
0.313365618967289, 0, 0.245755966326559, 0, 0.281514723202237, 
0.933333333333334, 0.254119582979893, 0, 0.162221720539515, 0, 
0.392069337450688, 0.422778102870231, 0.933333333333334, 0.933333333333334, 
0.933333333333334, 0.366670096391284, 0.21147844481506, 0.376422988993672, 
0, 0, 0, 0.474034996224347, 0.862890937289185, 0.785672048466947, 
0.436120716954563, 0.763474271878386, 0.378552571208885, 0, 0.311706079372435, 
0.313365618967289, 0.121377650393631, 1.11022302462516e-16, 0.509613831564055, 
0.393669350159573, 1.11022302462516e-16, 0, 0.342023424984461, 
0.453402366239767, 0.1527844419189, 0, 0.293890807604492, 0.38000880960185, 
2.22044604925031e-16, 0.453402366239767, 0.706215846551823, 0.144455024862375, 
1.11022302462516e-16, 0.363164307792717, 0.933333333333334, 0.0252569376979154, 
0, 0.472224825437357, 0.596586124729633, 0.797771675581462, 0.826924615886419, 
0.0637960473501239, 1.11022302462516e-16, 0.0900778529455906, 
7.7715611723761e-16, 0.308505387483734, 0.412045471623929), A.pval = c(0, 
0, 0.461448546494195, 0, 0.181723116919461, 0.228055642962163, 
0.801455224245302, 0.452130958015173, 0.428946101315051, 0.207222541347919, 
0, 0.263690240994158, 0, 0.171145758265626, 0.173913690698811, 
0.352118030221679, 0.999999321169503, 0.757498903294461, 0.999999321169503, 
0.915139301975687, 0.999999321169503, 0.945932629032511, 0.700331989474515, 
0.878427471799292, 0.503046909387333, 0, 0.505805793539102, 0.406930138248032, 
0, 0.346156129759459, 0, 0.615700735395404, 0.836683706358932, 
0.958671918196533, 0.421701849366553, 0.147606769109008, 0, 0.507075578427698, 
0.408023763828682, 0.187510318434585, 0.365071741350048, 0.0790600974214789, 
0, 0.173913690698811, 0.368036862812843, 0.022000336163131, 0, 
0.121959013933057, 0.0445137719869108, 0.178299162608713, 0.029962976205842, 
0, 0, 0.00390658858644999, 0, 0, 0.0407496943269546, 0.999999321169503, 
0.253080045489121, 0.467020414624534, 0.00241891526098947, 0, 
0.0391269601208851, 0, 0.473171977791495, 0.518074836671615, 
0.960045007990497, 0.322315357667865, 0.460848819853617, 0.295917809712254, 
0.0290425293773521, 0.00990926541542581, 0.00031888353613263, 
0, 0.157563834028684), guide.position = 1:75), .Names = c("A.area", 
"C.area", "G.area", "T.area", "Tot.area", "A.perc", "C.perc", 
"G.perc", "T.perc", "base.call", "index", "guide.seq", "T.pval", 
"C.pval", "G.pval", "A.pval", "guide.position"), row.names = 273:347, class = "data.frame")
```

*Report generated using EditR v1.0.8*
