# Supplementary material for: Mechanistic and genetic basis of single-strand templated repair at Cas12a-induced DNA breaks in Chlamydomonas reinhardtii
Source: Nat Commun. 2021 Nov 19;12:6751. doi: 10.1038/s41467-021-27004-1 (PMC8604939; doi:10.1038/s41467-021-27004-1)
Supplement: Supplementary file 22 — Source Data [file 41467_2021_27004_MOESM22_ESM.zip › Source Data/EditR analysis/EditR outputs/Sense/rep1_ssODN_sense_-32_-16_0.html]

EditR v1.0.8 report


# EditR v1.0.8 report

- Data QA
  - Filtering data
  - Percent noise peak area
  - Base information
- Predicted editing
  - Editing bar plot
  - Editing table plot
  - Table of editing results
- For use in R

## Data QA

### Filtering data

What the data looked like prefiltering:

and the post filtering signal / noise plot:

### Percent noise peak area

### Base information

Here’s information about the signal of each base, the critical percent value where any higher value would be called as significant, and Filliben’s correlation for how well the noise was modelled by the zero adjusted gamma distribution.

| Base | Average percent signal | Average peak area | Critical percent value | model mu | Fillibens correlation |
| --- | --- | --- | --- | --- | --- |
| A | 94.23947 | 525.1429 | 5.457263 | 1.860419 | 0.9846860 |
| C | 93.88873 | 552.1515 | 4.887251 | 1.719737 | 0.9845505 |
| G | 95.05920 | 532.8246 | 6.657926 | 2.049130 | 0.9911859 |
| T | 95.51205 | 560.2558 | 6.067877 | 1.952765 | 0.9945336 |

## Predicted editing

### Editing bar plot

### Editing table plot

### Table of editing results


Here’s the entire guide region

| Sanger position | Guide position | Guide sequence | Sanger base call | Focal base | Focal base peak area | p value |  |
| --- | --- | --- | --- | --- | --- | --- | --- |
| 273 | 1 | A | A | A | 96.13 | 0.000000e+00 | \* |
| 273 | 1 | A | A | C | 1.74 | 3.736168e-01 |  |
| 273 | 1 | A | A | G | 1.35 | 5.354938e-01 |  |
| 273 | 1 | A | A | T | 0.77 | 7.815407e-01 |  |
| 274 | 2 | A | A | A | 95.76 | 0.000000e+00 | \* |
| 274 | 2 | A | A | C | 1.36 | 5.165555e-01 |  |
| 274 | 2 | A | A | G | 2.03 | 3.549945e-01 |  |
| 274 | 2 | A | A | T | 0.85 | 7.562420e-01 |  |
| 275 | 3 | G | G | A | 1.40 | 5.644429e-01 |  |
| 275 | 3 | G | G | C | 1.40 | 5.000911e-01 |  |
| 275 | 3 | G | G | G | 95.81 | 0.000000e+00 | \* |
| 275 | 3 | G | G | T | 1.40 | 5.602802e-01 |  |
| 276 | 4 | A | A | A | 96.54 | 0.000000e+00 | \* |
| 276 | 4 | A | A | C | 0.84 | 7.293874e-01 |  |
| 276 | 4 | A | A | G | 1.05 | 6.288850e-01 |  |
| 276 | 4 | A | A | T | 1.57 | 5.011125e-01 |  |
| 277 | 5 | C | C | A | 3.11 | 1.270857e-01 |  |
| 277 | 5 | C | C | C | 92.73 | 0.000000e+00 | \* |
| 277 | 5 | C | C | G | 2.42 | 2.741721e-01 |  |
| 277 | 5 | C | C | T | 1.73 | 4.508207e-01 |  |
| 278 | 6 | T | T | A | 3.10 | 1.287925e-01 |  |
| 278 | 6 | T | T | C | 1.24 | 5.636944e-01 |  |
| 278 | 6 | T | T | G | 2.33 | 2.927426e-01 |  |
| 278 | 6 | T | T | T | 93.33 | 0.000000e+00 | \* |
| 279 | 7 | G | G | A | 0.87 | 7.726800e-01 |  |
| 279 | 7 | G | G | C | 1.55 | 4.428321e-01 |  |
| 279 | 7 | G | G | G | 96.62 | 0.000000e+00 | \* |
| 279 | 7 | G | G | T | 0.97 | 7.142739e-01 |  |
| 280 | 8 | G | G | A | 1.71 | 4.501017e-01 |  |
| 280 | 8 | G | G | C | 1.14 | 6.062545e-01 |  |
| 280 | 8 | G | G | G | 97.16 | 0.000000e+00 | \* |
| 280 | 8 | G | G | T | 0.00 | 9.361702e-01 |  |
| 281 | 9 | C | C | A | 2.38 | 2.566508e-01 |  |
| 281 | 9 | C | C | C | 91.92 | 0.000000e+00 | \* |
| 281 | 9 | C | C | G | 2.38 | 2.830577e-01 |  |
| 281 | 9 | C | C | T | 3.33 | 1.284558e-01 |  |
| 282 | 10 | C | C | A | 2.34 | 2.659215e-01 |  |
| 282 | 10 | C | C | C | 94.31 | 0.000000e+00 | \* |
| 282 | 10 | C | C | G | 1.75 | 4.241347e-01 |  |
| 282 | 10 | C | C | T | 1.61 | 4.901729e-01 |  |
| 283 | 11 | A | A | A | 81.94 | 0.000000e+00 | \* |
| 283 | 11 | A | A | C | 1.71 | 3.837220e-01 |  |
| 283 | 11 | A | A | G | 2.85 | 2.030448e-01 |  |
| 283 | 11 | A | A | T | 13.50 | 4.453918e-06 | \* |
| 284 | 12 | G | G | A | 1.53 | 5.155840e-01 |  |
| 284 | 12 | G | G | C | 1.69 | 3.892611e-01 |  |
| 284 | 12 | G | G | G | 95.76 | 0.000000e+00 | \* |
| 284 | 12 | G | G | T | 1.02 | 6.960444e-01 |  |
| 285 | 13 | A | A | A | 96.34 | 0.000000e+00 | \* |
| 285 | 13 | A | A | C | 1.08 | 6.324814e-01 |  |
| 285 | 13 | A | A | G | 1.18 | 5.872747e-01 |  |
| 285 | 13 | A | A | T | 1.40 | 5.600571e-01 |  |
| 286 | 14 | C | C | A | 3.00 | 1.420638e-01 |  |
| 286 | 14 | C | C | C | 94.79 | 0.000000e+00 | \* |
| 286 | 14 | C | C | G | 0.00 | 8.666667e-01 |  |
| 286 | 14 | C | C | T | 2.21 | 3.183152e-01 |  |
| 287 | 15 | C | C | A | 3.25 | 1.110114e-01 |  |
| 287 | 15 | C | C | C | 94.77 | 0.000000e+00 | \* |
| 287 | 15 | C | C | G | 1.99 | 3.662206e-01 |  |
| 287 | 15 | C | C | T | 0.00 | 9.361702e-01 |  |
| 288 | 16 | G | G | A | 2.46 | 2.385058e-01 |  |
| 288 | 16 | G | G | C | 1.05 | 6.419402e-01 |  |
| 288 | 16 | G | G | G | 95.44 | 0.000000e+00 | \* |
| 288 | 16 | G | G | T | 1.05 | 6.831794e-01 |  |
| 289 | 17 | T | T | A | 0.00 | 9.541284e-01 |  |
| 289 | 17 | T | T | C | 0.00 | 9.058824e-01 |  |
| 289 | 17 | T | T | G | 1.75 | 4.257485e-01 |  |
| 289 | 17 | T | T | T | 98.25 | 0.000000e+00 | \* |
| 290 | 18 | G | G | A | 0.95 | 7.417550e-01 |  |
| 290 | 18 | G | G | C | 1.07 | 6.351506e-01 |  |
| 290 | 18 | G | G | G | 97.03 | 0.000000e+00 | \* |
| 290 | 18 | G | G | T | 0.95 | 7.200163e-01 |  |
| 291 | 19 | T | T | A | 1.32 | 5.953420e-01 |  |
| 291 | 19 | T | T | C | 0.56 | 8.263041e-01 |  |
| 291 | 19 | T | T | G | 2.26 | 3.059487e-01 |  |
| 291 | 19 | T | T | T | 95.86 | 0.000000e+00 | \* |
| 292 | 20 | T | T | A | 0.73 | 8.229179e-01 |  |
| 292 | 20 | T | T | C | 0.00 | 9.058824e-01 |  |
| 292 | 20 | T | T | G | 1.10 | 6.137828e-01 |  |
| 292 | 20 | T | T | T | 98.17 | 0.000000e+00 | \* |
| 293 | 21 | T | T | A | 0.00 | 9.541284e-01 |  |
| 293 | 21 | T | T | C | 2.78 | 1.304465e-01 |  |
| 293 | 21 | T | T | G | 2.78 | 2.140171e-01 |  |
| 293 | 21 | T | T | T | 94.44 | 0.000000e+00 | \* |
| 294 | 22 | G | G | A | 0.13 | 9.514781e-01 |  |
| 294 | 22 | G | G | C | 2.12 | 2.610063e-01 |  |
| 294 | 22 | G | G | G | 96.56 | 0.000000e+00 | \* |
| 294 | 22 | G | G | T | 1.19 | 6.328790e-01 |  |
| 295 | 23 | T | T | A | 0.60 | 8.659904e-01 |  |
| 295 | 23 | T | T | C | 2.00 | 2.944383e-01 |  |
| 295 | 23 | T | T | G | 1.80 | 4.126298e-01 |  |
| 295 | 23 | T | T | T | 95.61 | 0.000000e+00 | \* |
| 296 | 24 | G | G | A | 0.64 | 8.519320e-01 |  |
| 296 | 24 | G | G | C | 1.13 | 6.107551e-01 |  |
| 296 | 24 | G | G | G | 96.78 | 0.000000e+00 | \* |
| 296 | 24 | G | G | T | 1.45 | 5.423623e-01 |  |
| 297 | 25 | C | C | A | 1.28 | 6.106044e-01 |  |
| 297 | 25 | C | C | C | 93.60 | 0.000000e+00 | \* |
| 297 | 25 | C | C | G | 1.65 | 4.524587e-01 |  |
| 297 | 25 | C | C | T | 3.47 | 1.129713e-01 |  |
| 298 | 26 | A | A | A | 92.72 | 0.000000e+00 | \* |
| 298 | 26 | A | A | C | 3.29 | 7.303097e-02 |  |
| 298 | 26 | A | A | G | 1.64 | 4.530414e-01 |  |
| 298 | 26 | A | A | T | 2.35 | 2.869139e-01 |  |
| 299 | 27 | C | C | A | 1.56 | 5.018285e-01 |  |
| 299 | 27 | C | C | C | 89.41 | 0.000000e+00 | \* |
| 299 | 27 | C | C | G | 7.29 | 5.855097e-03 | \* |
| 299 | 27 | C | C | T | 1.74 | 4.489687e-01 |  |
| 300 | 28 | T | T | A | 1.59 | 4.906821e-01 |  |
| 300 | 28 | T | T | C | 3.19 | 8.211122e-02 |  |
| 300 | 28 | T | T | G | 0.18 | 8.547677e-01 |  |
| 300 | 28 | T | T | T | 95.04 | 0.000000e+00 | \* |
| 301 | 29 | A | A | A | 96.39 | 0.000000e+00 | \* |
| 301 | 29 | A | A | C | 1.90 | 3.234040e-01 |  |
| 301 | 29 | A | A | G | 0.00 | 8.666667e-01 |  |
| 301 | 29 | A | A | T | 1.71 | 4.577445e-01 |  |
| 302 | 30 | C | C | A | 1.72 | 4.472284e-01 |  |
| 302 | 30 | C | C | C | 94.51 | 0.000000e+00 | \* |
| 302 | 30 | C | C | G | 1.20 | 5.817884e-01 |  |
| 302 | 30 | C | C | T | 2.57 | 2.403114e-01 |  |
| 303 | 31 | A | A | A | 94.04 | 0.000000e+00 | \* |
| 303 | 31 | A | A | C | 2.75 | 1.341728e-01 |  |
| 303 | 31 | A | A | G | 3.21 | 1.564618e-01 |  |
| 303 | 31 | A | A | T | 0.00 | 9.361702e-01 |  |
| 304 | 32 | C | C | A | 1.52 | 5.183499e-01 |  |
| 304 | 32 | C | C | C | 94.88 | 0.000000e+00 | \* |
| 304 | 32 | C | C | G | 1.33 | 5.431509e-01 |  |
| 304 | 32 | C | C | T | 2.28 | 3.028742e-01 |  |
| 305 | 33 | G | G | A | 0.50 | 8.930745e-01 |  |
| 305 | 33 | G | G | C | 2.26 | 2.262248e-01 |  |
| 305 | 33 | G | G | G | 96.23 | 0.000000e+00 | \* |
| 305 | 33 | G | G | T | 1.01 | 7.003357e-01 |  |
| 306 | 34 | G | G | A | 0.00 | 9.541284e-01 |  |
| 306 | 34 | G | G | C | 0.00 | 9.058824e-01 |  |
| 306 | 34 | G | G | G | 99.30 | 0.000000e+00 | \* |
| 306 | 34 | G | G | T | 0.70 | 8.061603e-01 |  |
| 307 | 35 | G | G | A | 2.04 | 3.436568e-01 |  |
| 307 | 35 | G | G | C | 1.43 | 4.877318e-01 |  |
| 307 | 35 | G | G | G | 96.53 | 0.000000e+00 | \* |
| 307 | 35 | G | G | T | 0.00 | 9.361702e-01 |  |
| 308 | 36 | C | C | A | 2.66 | 1.972673e-01 |  |
| 308 | 36 | C | C | C | 93.35 | 0.000000e+00 | \* |
| 308 | 36 | C | C | G | 1.77 | 4.184280e-01 |  |
| 308 | 36 | C | C | T | 2.22 | 3.169673e-01 |  |
| 309 | 37 | A | A | A | 95.86 | 0.000000e+00 | \* |
| 309 | 37 | A | A | C | 3.05 | 9.600520e-02 |  |
| 309 | 37 | A | A | G | 1.09 | 6.161281e-01 |  |
| 309 | 37 | A | A | T | 0.00 | 9.361702e-01 |  |
| 310 | 38 | C | C | A | 1.63 | 4.765395e-01 |  |
| 310 | 38 | C | C | C | 94.96 | 0.000000e+00 | \* |
| 310 | 38 | C | C | G | 1.04 | 6.318804e-01 |  |
| 310 | 38 | C | C | T | 2.37 | 2.810886e-01 |  |
| 311 | 39 | C | C | A | 2.10 | 3.277283e-01 |  |
| 311 | 39 | C | C | C | 94.35 | 0.000000e+00 | \* |
| 311 | 39 | C | C | G | 1.13 | 6.038376e-01 |  |
| 311 | 39 | C | C | T | 2.42 | 2.713057e-01 |  |
| 312 | 40 | C | C | A | 1.87 | 3.964935e-01 |  |
| 312 | 40 | C | C | C | 95.42 | 0.000000e+00 | \* |
| 312 | 40 | C | C | G | 0.34 | 8.275832e-01 |  |
| 312 | 40 | C | C | T | 2.38 | 2.804294e-01 |  |
| 313 | 41 | T | T | A | 0.72 | 8.278804e-01 |  |
| 313 | 41 | T | T | C | 2.33 | 2.108661e-01 |  |
| 313 | 41 | T | T | G | 1.97 | 3.695756e-01 |  |
| 313 | 41 | T | T | T | 94.98 | 0.000000e+00 | \* |
| 314 | 42 | G | G | A | 2.16 | 3.116991e-01 |  |
| 314 | 42 | G | G | C | 1.58 | 4.300424e-01 |  |
| 314 | 42 | G | G | G | 94.97 | 0.000000e+00 | \* |
| 314 | 42 | G | G | T | 1.29 | 5.967850e-01 |  |
| 315 | 43 | A | A | A | 82.31 | 0.000000e+00 | \* |
| 315 | 43 | A | A | C | 1.90 | 3.239516e-01 |  |
| 315 | 43 | A | A | G | 3.00 | 1.823014e-01 |  |
| 315 | 43 | A | A | T | 12.80 | 9.453632e-06 | \* |
| 316 | 44 | C | C | A | 1.62 | 4.802861e-01 |  |
| 316 | 44 | C | C | C | 95.50 | 0.000000e+00 | \* |
| 316 | 44 | C | C | G | 0.00 | 8.666667e-01 |  |
| 316 | 44 | C | C | T | 2.88 | 1.867573e-01 |  |
| 317 | 45 | C | C | A | 1.55 | 5.074127e-01 |  |
| 317 | 45 | C | C | C | 94.58 | 0.000000e+00 | \* |
| 317 | 45 | C | C | G | 3.87 | 9.542706e-02 |  |
| 317 | 45 | C | C | T | 0.00 | 9.361702e-01 |  |
| 318 | 46 | G | G | A | 4.57 | 2.737874e-02 |  |
| 318 | 46 | G | G | C | 2.28 | 2.212369e-01 |  |
| 318 | 46 | G | G | G | 92.47 | 0.000000e+00 | \* |
| 318 | 46 | G | G | T | 0.68 | 8.107892e-01 |  |
| 319 | 47 | A | A | A | 97.65 | 0.000000e+00 | \* |
| 319 | 47 | A | A | C | 1.31 | 5.369586e-01 |  |
| 319 | 47 | A | A | G | 1.04 | 6.300752e-01 |  |
| 319 | 47 | A | A | T | 0.00 | 9.361702e-01 |  |
| 320 | 48 | C | C | A | 3.77 | 6.506346e-02 |  |
| 320 | 48 | C | C | C | 91.90 | 0.000000e+00 | \* |
| 320 | 48 | C | C | G | 1.13 | 6.035561e-01 |  |
| 320 | 48 | C | C | T | 3.20 | 1.428635e-01 |  |
| 321 | 49 | G | G | A | 3.61 | 7.630459e-02 |  |
| 321 | 49 | G | G | C | 3.01 | 1.002667e-01 |  |
| 321 | 49 | G | G | G | 92.77 | 0.000000e+00 | \* |
| 321 | 49 | G | G | T | 0.60 | 8.364188e-01 |  |
| 322 | 50 | G | G | A | 1.79 | 4.213375e-01 |  |
| 322 | 50 | G | G | C | 1.79 | 3.568187e-01 |  |
| 322 | 50 | G | G | G | 95.93 | 0.000000e+00 | \* |
| 322 | 50 | G | G | T | 0.49 | 8.684972e-01 |  |
| 323 | 51 | C | C | A | 2.75 | 1.817439e-01 |  |
| 323 | 51 | C | C | C | 92.31 | 0.000000e+00 | \* |
| 323 | 51 | C | C | G | 2.75 | 2.186950e-01 |  |
| 323 | 51 | C | C | T | 2.20 | 3.216745e-01 |  |
| 324 | 52 | A | A | A | 96.89 | 0.000000e+00 | \* |
| 324 | 52 | A | A | C | 1.38 | 5.053019e-01 |  |
| 324 | 52 | A | A | G | 1.04 | 6.320399e-01 |  |
| 324 | 52 | A | A | T | 0.69 | 8.085044e-01 |  |
| 325 | 53 | A | A | A | 93.70 | 0.000000e+00 | \* |
| 325 | 53 | A | A | C | 1.38 | 5.077439e-01 |  |
| 325 | 53 | A | A | G | 4.13 | 7.774662e-02 |  |
| 325 | 53 | A | A | T | 0.79 | 7.768987e-01 |  |
| 326 | 54 | G | G | A | 3.56 | 8.045978e-02 |  |
| 326 | 54 | G | G | C | 0.89 | 7.085568e-01 |  |
| 326 | 54 | G | G | G | 94.88 | 0.000000e+00 | \* |
| 326 | 54 | G | G | T | 0.67 | 8.161366e-01 |  |
| 327 | 55 | A | A | A | 96.99 | 0.000000e+00 | \* |
| 327 | 55 | A | A | C | 0.00 | 9.058824e-01 |  |
| 327 | 55 | A | A | G | 1.77 | 4.194417e-01 |  |
| 327 | 55 | A | A | T | 1.24 | 6.160692e-01 |  |
| 328 | 56 | A | A | A | 95.38 | 0.000000e+00 | \* |
| 328 | 56 | A | A | C | 0.40 | 8.688584e-01 |  |
| 328 | 56 | A | A | G | 2.01 | 3.609693e-01 |  |
| 328 | 56 | A | A | T | 2.21 | 3.190035e-01 |  |
| 329 | 57 | G | G | A | 3.01 | 1.415221e-01 |  |
| 329 | 57 | G | G | C | 1.09 | 6.251095e-01 |  |
| 329 | 57 | G | G | G | 92.62 | 0.000000e+00 | \* |
| 329 | 57 | G | G | T | 3.28 | 1.337275e-01 |  |
| 330 | 58 | T | T | A | 2.29 | 2.761089e-01 |  |
| 330 | 58 | T | T | C | 0.23 | 8.963947e-01 |  |
| 330 | 58 | T | T | G | 1.38 | 5.289209e-01 |  |
| 330 | 58 | T | T | T | 96.10 | 0.000000e+00 | \* |
| 331 | 59 | T | T | A | 0.92 | 7.519649e-01 |  |
| 331 | 59 | T | T | C | 2.08 | 2.717046e-01 |  |
| 331 | 59 | T | T | G | 0.92 | 6.675122e-01 |  |
| 331 | 59 | T | T | T | 96.07 | 0.000000e+00 | \* |
| 332 | 60 | C | C | A | 1.47 | 5.362496e-01 |  |
| 332 | 60 | C | C | C | 94.54 | 0.000000e+00 | \* |
| 332 | 60 | C | C | G | 2.73 | 2.212070e-01 |  |
| 332 | 60 | C | C | T | 1.26 | 6.083714e-01 |  |
| 333 | 61 | G | G | A | 5.38 | 1.097456e-02 |  |
| 333 | 61 | G | G | C | 1.61 | 4.182333e-01 |  |
| 333 | 61 | G | G | G | 92.47 | 0.000000e+00 | \* |
| 333 | 61 | G | G | T | 0.54 | 8.552032e-01 |  |
| 334 | 62 | A | A | A | 96.22 | 0.000000e+00 | \* |
| 334 | 62 | A | A | C | 1.73 | 3.764924e-01 |  |
| 334 | 62 | A | A | G | 2.05 | 3.519409e-01 |  |
| 334 | 62 | A | A | T | 0.00 | 9.361702e-01 |  |
| 335 | 63 | C | C | A | 2.98 | 1.455010e-01 |  |
| 335 | 63 | C | C | C | 92.82 | 0.000000e+00 | \* |
| 335 | 63 | C | C | G | 2.28 | 3.025228e-01 |  |
| 335 | 63 | C | C | T | 1.93 | 3.928389e-01 |  |
| 336 | 64 | A | A | A | 90.77 | 0.000000e+00 | \* |
| 336 | 64 | A | A | C | 2.37 | 2.012611e-01 |  |
| 336 | 64 | A | A | G | 6.33 | 1.313074e-02 |  |
| 336 | 64 | A | A | T | 0.53 | 8.579637e-01 |  |
| 337 | 65 | G | G | A | 1.46 | 5.413517e-01 |  |
| 337 | 65 | G | G | C | 2.91 | 1.119912e-01 |  |
| 337 | 65 | G | G | G | 94.90 | 0.000000e+00 | \* |
| 337 | 65 | G | G | T | 0.73 | 7.965893e-01 |  |
| 338 | 66 | C | C | A | 2.00 | 3.569453e-01 |  |
| 338 | 66 | C | C | C | 92.24 | 0.000000e+00 | \* |
| 338 | 66 | C | C | G | 1.33 | 5.425222e-01 |  |
| 338 | 66 | C | C | T | 4.43 | 4.756070e-02 |  |
| 339 | 67 | T | T | A | 0.18 | 9.486518e-01 |  |
| 339 | 67 | T | T | C | 2.31 | 2.154160e-01 |  |
| 339 | 67 | T | T | G | 0.36 | 8.243407e-01 |  |
| 339 | 67 | T | T | T | 97.16 | 0.000000e+00 | \* |
| 340 | 68 | C | C | A | 1.39 | 5.676729e-01 |  |
| 340 | 68 | C | C | C | 95.24 | 0.000000e+00 | \* |
| 340 | 68 | C | C | G | 0.79 | 7.073928e-01 |  |
| 340 | 68 | C | C | T | 2.58 | 2.390737e-01 |  |
| 341 | 69 | C | C | A | 1.85 | 4.033911e-01 |  |
| 341 | 69 | C | C | C | 94.46 | 0.000000e+00 | \* |
| 341 | 69 | C | C | G | 0.31 | 8.338711e-01 |  |
| 341 | 69 | C | C | T | 3.38 | 1.220475e-01 |  |
| 342 | 70 | C | C | A | 2.11 | 3.233035e-01 |  |
| 342 | 70 | C | C | C | 91.08 | 0.000000e+00 | \* |
| 342 | 70 | C | C | G | 4.23 | 7.239926e-02 |  |
| 342 | 70 | C | C | T | 2.58 | 2.385405e-01 |  |
| 343 | 71 | G | G | A | 3.95 | 5.351291e-02 |  |
| 343 | 71 | G | G | C | 2.72 | 1.396336e-01 |  |
| 343 | 71 | G | G | G | 92.84 | 0.000000e+00 | \* |
| 343 | 71 | G | G | T | 0.49 | 8.671213e-01 |  |
| 344 | 72 | C | C | A | 4.88 | 1.921939e-02 |  |
| 344 | 72 | C | C | C | 89.60 | 0.000000e+00 | \* |
| 344 | 72 | C | C | G | 2.97 | 1.861874e-01 |  |
| 344 | 72 | C | C | T | 2.55 | 2.451722e-01 |  |
| 345 | 73 | G | G | A | 6.55 | 2.783019e-03 | \* |
| 345 | 73 | G | G | C | 3.27 | 7.404896e-02 |  |
| 345 | 73 | G | G | G | 89.42 | 0.000000e+00 | \* |
| 345 | 73 | G | G | T | 0.76 | 7.875990e-01 |  |
| 346 | 74 | A | A | A | 95.74 | 0.000000e+00 | \* |
| 346 | 74 | A | A | C | 2.49 | 1.788925e-01 |  |
| 346 | 74 | A | A | G | 1.78 | 4.178184e-01 |  |
| 346 | 74 | A | A | T | 0.00 | 9.361702e-01 |  |
| 347 | 75 | C | C | A | 2.49 | 2.317431e-01 |  |
| 347 | 75 | C | C | C | 91.87 | 0.000000e+00 | \* |
| 347 | 75 | C | C | G | 1.49 | 4.950677e-01 |  |
| 347 | 75 | C | C | T | 4.15 | 6.198731e-02 |  |

## For use in R

If you want to work with the results in R, here is output that you can copy and paste in your terminal to get:

The base information:

```
structure(list(focal.base = c("A", "C", "G", "T"), avg.percsignal = c(94.2394715218146, 
93.8887267131487, 95.0591982104002, 95.512053765825), avg.areasignal = c(525.142857142857, 
552.151515151515, 532.824561403509, 560.255813953488), crit.perc.area = c(5.45726354583057, 
4.88725052829722, 6.65792622105791, 6.06787713478139), mu = c(1.8604192060101, 
1.71973661005601, 2.0491299497667, 1.95276525763001), fillibens = c(0.984685983436521, 
0.984550492801554, 0.991185923738218, 0.994533593356241)), .Names = c("focal.base", 
"avg.percsignal", "avg.areasignal", "crit.perc.area", "mu", "fillibens"
), row.names = c(NA, -4L), class = "data.frame")
```

the data.frame that contains information on the guide region:

```
structure(list(A.area = c(497, 565, 7, 921, 18, 20, 9, 12, 10, 
16, 431, 9, 896, 19, 18, 14, 0, 8, 7, 4, 0, 1, 3, 4, 7, 395, 
9, 9, 508, 10, 410, 8, 2, 0, 10, 12, 440, 11, 13, 11, 4, 15, 
521, 9, 8, 20, 748, 20, 12, 11, 10, 280, 476, 16, 548, 475, 11, 
10, 4, 7, 20, 611, 17, 344, 8, 9, 1, 7, 12, 9, 16, 23, 26, 539, 
15), C.area = c(9, 8, 7, 8, 536, 8, 16, 8, 387, 646, 9, 10, 10, 
600, 525, 6, 0, 9, 3, 0, 16, 16, 10, 7, 512, 14, 515, 18, 10, 
551, 12, 500, 9, 0, 7, 421, 14, 640, 585, 562, 13, 11, 12, 530, 
489, 10, 10, 488, 10, 11, 336, 4, 7, 4, 0, 2, 4, 1, 9, 450, 6, 
11, 530, 9, 16, 416, 13, 480, 614, 388, 11, 422, 13, 14, 554), 
    G.area = c(7, 12, 480, 10, 14, 15, 1000, 683, 10, 12, 15, 
    565, 11, 0, 11, 544, 7, 817, 12, 6, 16, 729, 9, 601, 9, 7, 
    42, 1, 0, 7, 14, 7, 383, 568, 473, 8, 5, 7, 7, 2, 11, 661, 
    19, 0, 20, 405, 8, 6, 308, 589, 10, 3, 21, 426, 10, 10, 339, 
    6, 4, 13, 344, 13, 13, 24, 521, 6, 2, 4, 2, 18, 376, 14, 
    355, 10, 9), T.area = c(4, 5, 7, 15, 10, 602, 10, 0, 14, 
    11, 71, 6, 13, 14, 0, 6, 394, 8, 509, 537, 544, 9, 479, 9, 
    19, 10, 10, 537, 9, 15, 0, 12, 4, 4, 0, 10, 0, 16, 15, 14, 
    530, 9, 81, 16, 0, 3, 0, 17, 2, 3, 8, 2, 4, 3, 7, 11, 12, 
    419, 416, 6, 2, 0, 11, 2, 4, 20, 547, 13, 22, 11, 2, 12, 
    3, 0, 25), Tot.area = c(517, 590, 501, 954, 578, 645, 1035, 
    703, 421, 685, 526, 590, 930, 633, 554, 570, 401, 842, 531, 
    547, 576, 755, 501, 621, 547, 426, 576, 565, 527, 583, 436, 
    527, 398, 572, 490, 451, 459, 674, 620, 589, 558, 696, 633, 
    555, 517, 438, 766, 531, 332, 614, 364, 289, 508, 449, 565, 
    498, 366, 436, 433, 476, 372, 635, 571, 379, 549, 451, 563, 
    504, 650, 426, 405, 471, 397, 563, 603), A.perc = c(96.1315280464217, 
    95.7627118644068, 1.39720558882236, 96.5408805031446, 3.11418685121107, 
    3.10077519379845, 0.869565217391304, 1.70697012802276, 2.37529691211401, 
    2.33576642335766, 81.9391634980989, 1.52542372881356, 96.3440860215054, 
    3.00157977883096, 3.24909747292419, 2.45614035087719, 0, 
    0.950118764845606, 1.31826741996234, 0.731261425959781, 0, 
    0.132450331125828, 0.598802395209581, 0.644122383252818, 
    1.27970749542962, 92.7230046948357, 1.5625, 1.5929203539823, 
    96.3946869070209, 1.71526586620926, 94.0366972477064, 1.5180265654649, 
    0.50251256281407, 0, 2.04081632653061, 2.66075388026608, 
    95.8605664488017, 1.63204747774481, 2.09677419354839, 1.86757215619694, 
    0.716845878136201, 2.1551724137931, 82.306477093207, 1.62162162162162, 
    1.54738878143133, 4.5662100456621, 97.6501305483029, 3.76647834274953, 
    3.6144578313253, 1.79153094462541, 2.74725274725275, 96.8858131487889, 
    93.7007874015748, 3.56347438752784, 96.9911504424779, 95.3815261044177, 
    3.00546448087432, 2.29357798165138, 0.92378752886836, 1.47058823529412, 
    5.37634408602151, 96.2204724409449, 2.97723292469352, 90.7651715039578, 
    1.45719489981785, 1.99556541019956, 0.177619893428064, 1.38888888888889, 
    1.84615384615385, 2.11267605633803, 3.95061728395062, 4.88322717622081, 
    6.54911838790932, 95.7371225577265, 2.48756218905473), C.perc = c(1.74081237911025, 
    1.35593220338983, 1.39720558882236, 0.838574423480084, 92.7335640138408, 
    1.24031007751938, 1.54589371980676, 1.13798008534851, 91.9239904988124, 
    94.3065693430657, 1.71102661596958, 1.69491525423729, 1.0752688172043, 
    94.7867298578199, 94.7653429602888, 1.05263157894737, 0, 
    1.06888361045131, 0.564971751412429, 0, 2.77777777777778, 
    2.11920529801325, 1.99600798403194, 1.12721417069243, 93.6014625228519, 
    3.28638497652582, 89.4097222222222, 3.1858407079646, 1.89753320683112, 
    94.5111492281304, 2.75229357798165, 94.876660341556, 2.26130653266332, 
    0, 1.42857142857143, 93.3481152993348, 3.05010893246187, 
    94.9554896142433, 94.3548387096774, 95.4159592529711, 2.32974910394265, 
    1.58045977011494, 1.8957345971564, 95.4954954954955, 94.5841392649903, 
    2.28310502283105, 1.30548302872063, 91.9020715630885, 3.01204819277108, 
    1.79153094462541, 92.3076923076923, 1.3840830449827, 1.37795275590551, 
    0.89086859688196, 0, 0.401606425702811, 1.09289617486339, 
    0.229357798165138, 2.07852193995381, 94.5378151260504, 1.61290322580645, 
    1.73228346456693, 92.8196147110333, 2.37467018469657, 2.9143897996357, 
    92.2394678492239, 2.30905861456483, 95.2380952380952, 94.4615384615385, 
    91.0798122065728, 2.71604938271605, 89.5966029723992, 3.27455919395466, 
    2.4866785079929, 91.8739635157546), G.perc = c(1.35396518375242, 
    2.03389830508475, 95.8083832335329, 1.0482180293501, 2.42214532871972, 
    2.32558139534884, 96.6183574879227, 97.1550497866287, 2.37529691211401, 
    1.75182481751825, 2.85171102661597, 95.7627118644068, 1.18279569892473, 
    0, 1.985559566787, 95.4385964912281, 1.74563591022444, 97.0308788598575, 
    2.25988700564972, 1.09689213893967, 2.77777777777778, 96.5562913907285, 
    1.79640718562874, 96.7793880837359, 1.64533820840951, 1.64319248826291, 
    7.29166666666667, 0.176991150442478, 0, 1.20068610634648, 
    3.21100917431193, 1.32827324478178, 96.2311557788945, 99.3006993006993, 
    96.530612244898, 1.77383592017738, 1.08932461873638, 1.03857566765579, 
    1.12903225806452, 0.33955857385399, 1.97132616487455, 94.9712643678161, 
    3.00157977883096, 0, 3.86847195357834, 92.4657534246575, 
    1.0443864229765, 1.12994350282486, 92.7710843373494, 95.928338762215, 
    2.74725274725275, 1.03806228373702, 4.13385826771654, 94.8775055679287, 
    1.76991150442478, 2.00803212851406, 92.6229508196721, 1.37614678899083, 
    0.92378752886836, 2.73109243697479, 92.4731182795699, 2.04724409448819, 
    2.27670753064799, 6.33245382585752, 94.8998178506375, 1.33037694013304, 
    0.355239786856128, 0.793650793650794, 0.307692307692308, 
    4.22535211267606, 92.8395061728395, 2.9723991507431, 89.4206549118388, 
    1.77619893428064, 1.49253731343284), T.perc = c(0.773694390715667, 
    0.847457627118644, 1.39720558882236, 1.57232704402516, 1.73010380622837, 
    93.3333333333333, 0.966183574879227, 0, 3.32541567695962, 
    1.60583941605839, 13.4980988593156, 1.01694915254237, 1.39784946236559, 
    2.21169036334913, 0, 1.05263157894737, 98.2543640897756, 
    0.950118764845606, 95.8568738229755, 98.1718464351005, 94.4444444444444, 
    1.19205298013245, 95.6087824351297, 1.44927536231884, 3.47349177330896, 
    2.34741784037559, 1.73611111111111, 95.0442477876106, 1.70777988614801, 
    2.57289879931389, 0, 2.27703984819734, 1.00502512562814, 
    0.699300699300699, 0, 2.21729490022173, 0, 2.37388724035608, 
    2.41935483870968, 2.37691001697793, 94.9820788530466, 1.29310344827586, 
    12.7962085308057, 2.88288288288288, 0, 0.684931506849315, 
    0, 3.2015065913371, 0.602409638554217, 0.488599348534202, 
    2.1978021978022, 0.692041522491349, 0.78740157480315, 0.66815144766147, 
    1.23893805309735, 2.20883534136546, 3.27868852459016, 96.1009174311927, 
    96.0739030023095, 1.26050420168067, 0.537634408602151, 0, 
    1.92644483362522, 0.527704485488127, 0.728597449908925, 4.43458980044346, 
    97.158081705151, 2.57936507936508, 3.38461538461538, 2.58215962441315, 
    0.493827160493827, 2.54777070063694, 0.755667506297229, 0, 
    4.14593698175788), base.call = c("A", "A", "G", "A", "C", 
    "T", "G", "G", "C", "C", "A", "G", "A", "C", "C", "G", "T", 
    "G", "T", "T", "T", "G", "T", "G", "C", "A", "C", "T", "A", 
    "C", "A", "C", "G", "G", "G", "C", "A", "C", "C", "C", "T", 
    "G", "A", "C", "C", "G", "A", "C", "G", "G", "C", "A", "A", 
    "G", "A", "A", "G", "T", "T", "C", "G", "A", "C", "A", "G", 
    "C", "T", "C", "C", "C", "G", "C", "G", "A", "C"), index = 273:347, 
    guide.seq = c("A", "A", "G", "A", "C", "T", "G", "G", "C", 
    "C", "A", "G", "A", "C", "C", "G", "T", "G", "T", "T", "T", 
    "G", "T", "G", "C", "A", "C", "T", "A", "C", "A", "C", "G", 
    "G", "G", "C", "A", "C", "C", "C", "T", "G", "A", "C", "C", 
    "G", "A", "C", "G", "G", "C", "A", "A", "G", "A", "A", "G", 
    "T", "T", "C", "G", "A", "C", "A", "G", "C", "T", "C", "C", 
    "C", "G", "C", "G", "A", "C"), T.pval = c(0.78154074187238, 
    0.756242036816206, 0.560280169005597, 0.501112529373133, 
    0.45082068376812, 0, 0.714273942587142, 0.936170212765957, 
    0.128455815842188, 0.490172894126728, 4.4539175221292e-06, 
    0.696044441740429, 0.560057093813876, 0.318315170837222, 
    0.936170212765957, 0.683179350028126, 0, 0.720016290097726, 
    0, 0, 0, 0.632878953387212, 0, 0.542362319953551, 0.112971279356312, 
    0.286913900467926, 0.448968722453229, 0, 0.457744491594613, 
    0.240311439143272, 0.936170212765957, 0.302874203362192, 
    0.700335678224141, 0.806160288250305, 0.936170212765957, 
    0.316967277721893, 0.936170212765957, 0.281088559270257, 
    0.27130573056109, 0.28042942959341, 0, 0.596785022601853, 
    9.4536319572569e-06, 0.186757273057283, 0.936170212765957, 
    0.810789218015525, 0.936170212765957, 0.142863539227216, 
    0.836418846804878, 0.868497242433117, 0.32167446986753, 0.808504377354552, 
    0.77689874901266, 0.81613660780037, 0.616069242337128, 0.319003515802537, 
    0.13372745838246, 0, 0, 0.608371442915441, 0.855203156456091, 
    0.936170212765957, 0.392838886326105, 0.857963696291066, 
    0.796589251566777, 0.0475607046782849, 0, 0.239073739946371, 
    0.122047525426043, 0.238540498178733, 0.867121338424699, 
    0.245172222191753, 0.787599027707344, 0.936170212765957, 
    0.0619873147977656), C.pval = c(0.373616825492151, 0.516555497047567, 
    0.500091078781209, 0.729387404355555, 0, 0.563694395701607, 
    0.44283205388155, 0.60625448315461, 0, 0, 0.383722049288268, 
    0.389261101273096, 0.632481415519354, 0, 0, 0.641940150014326, 
    0.905882352932355, 0.635150626751857, 0.826304127189377, 
    0.905882352932355, 0.130446512068019, 0.261006293604914, 
    0.294438287954638, 0.610755141555789, 0, 0.0730309688649959, 
    0, 0.0821112244061324, 0.323403950230354, 0, 0.134172757585124, 
    0, 0.226224762280439, 0.905882352932355, 0.487731813383576, 
    0, 0.0960051988756482, 0, 0, 0, 0.210866127209051, 0.430042390734251, 
    0.323951632619903, 0, 0, 0.221236919263357, 0.536958628063326, 
    0, 0.100266652969045, 0.356818694455751, 0, 0.505301895578497, 
    0.50774394989559, 0.708556769074915, 0.905882352932355, 0.868858420793363, 
    0.625109511941188, 0.896394727948594, 0.271704601395896, 
    0, 0.4182333184595, 0.376492362075646, 0, 0.201261079188383, 
    0.111991167726246, 0, 0.215415963249593, 0, 0, 0, 0.139633559882769, 
    0, 0.0740489600028383, 0.178892508396759, 0), G.pval = c(0.535493785511753, 
    0.354994508236385, 0, 0.628885042441877, 0.274172062259976, 
    0.292742626535183, 0, 0, 0.283057704949649, 0.424134698606077, 
    0.203044752451926, 0, 0.587274677863431, 0.866666666666663, 
    0.366220591063545, 0, 0.42574852076478, 0, 0.305948718620837, 
    0.613782806397034, 0.214017107142388, 0, 0.412629796068483, 
    0, 0.452458678820611, 0.453041353911478, 0.00585509712716659, 
    0.854767733247063, 0.866666666666663, 0.581788444608979, 
    0.156461807248636, 0.543150948665238, 0, 0, 0, 0.418428007096164, 
    0.616128088179317, 0.63188043549076, 0.60383762680158, 0.827583187632119, 
    0.369575594786596, 0, 0.182301363300779, 0.866666666666663, 
    0.095427058864713, 0, 0.6300752375685, 0.603556074084407, 
    0, 0, 0.21869496931399, 0.632039937565753, 0.0777466158753339, 
    0, 0.419441690603212, 0.360969309324711, 0, 0.528920898446155, 
    0.667512175837124, 0.221206999659303, 0, 0.351940872592973, 
    0.30252282950484, 0.0131307405159312, 0, 0.54252224425205, 
    0.824340678427909, 0.707392759536391, 0.833871110701875, 
    0.0723992560987301, 0, 0.186187398329808, 0, 0.417818429262213, 
    0.49506769637087), A.pval = c(0, 0, 0.564442931291746, 0, 
    0.127085702554031, 0.128792501925947, 0.772679968314115, 
    0.450101712133068, 0.256650848270189, 0.265921457149146, 
    0, 0.515583992587901, 0, 0.142063804201453, 0.111011422553593, 
    0.238505781414863, 0.95412844036683, 0.741755014451829, 0.595341951672475, 
    0.822917896782107, 0.95412844036683, 0.951478067332441, 0.865990440731539, 
    0.851931954310694, 0.61060442132274, 0, 0.501828483641861, 
    0.490682058385957, 0, 0.447228373963987, 0, 0.518349933225564, 
    0.893074464309325, 0.95412844036683, 0.34365679669793, 0.19726732425028, 
    0, 0.47653952016226, 0.327728319435967, 0.396493504555685, 
    0.827880389728106, 0.311699079796795, 0, 0.480286094189479, 
    0.507412748992153, 0.0273787416394717, 0, 0.0650634644507502, 
    0.0763045918486946, 0.421337474988009, 0.181743893281162, 
    0, 0, 0.0804597752432912, 0, 0, 0.141522074660812, 0.276108911902533, 
    0.75196491678769, 0.536249640166561, 0.0109745602102074, 
    0, 0.145500995679047, 0, 0.541351713415586, 0.356945256652805, 
    0.948651841159123, 0.567672876605367, 0.403391129672712, 
    0.323303539260178, 0.0535129107638932, 0.0192193889382799, 
    0.00278301946895065, 0, 0.231743062503589), guide.position = 1:75), .Names = c("A.area", 
"C.area", "G.area", "T.area", "Tot.area", "A.perc", "C.perc", 
"G.perc", "T.perc", "base.call", "index", "guide.seq", "T.pval", 
"C.pval", "G.pval", "A.pval", "guide.position"), row.names = 273:347, class = "data.frame")
```

*Report generated using EditR v1.0.8*
