# Supplementary material for: Mechanistic and genetic basis of single-strand templated repair at Cas12a-induced DNA breaks in Chlamydomonas reinhardtii
Source: Nat Commun. 2021 Nov 19;12:6751. doi: 10.1038/s41467-021-27004-1 (PMC8604939; doi:10.1038/s41467-021-27004-1)
Supplement: Supplementary file 22 — Source Data [file 41467_2021_27004_MOESM22_ESM.zip › Source Data/EditR analysis/EditR outputs/Sense/rep1_ssODN_sense_-32_-16_0_16_32.html]

EditR v1.0.8 report


# EditR v1.0.8 report

- Data QA
  - Filtering data
  - Percent noise peak area
  - Base information
- Predicted editing
  - Editing bar plot
  - Editing table plot
  - Table of editing results
- For use in R

## Data QA

### Filtering data

What the data looked like prefiltering:

and the post filtering signal / noise plot:

### Percent noise peak area

### Base information

Here’s information about the signal of each base, the critical percent value where any higher value would be called as significant, and Filliben’s correlation for how well the noise was modelled by the zero adjusted gamma distribution.

| Base | Average percent signal | Average peak area | Critical percent value | model mu | Fillibens correlation |
| --- | --- | --- | --- | --- | --- |
| A | 93.14437 | 443.1765 | 5.583715 | 1.631065 | 0.9966295 |
| C | 94.04665 | 470.0145 | 4.213181 | 1.559561 | 0.9923569 |
| G | 94.90111 | 498.6182 | 6.351190 | 2.090161 | 0.9951439 |
| T | 96.11603 | 537.2093 | 6.958888 | 2.312501 | 0.9960828 |

## Predicted editing

### Editing bar plot

### Editing table plot

### Table of editing results


Here’s the entire guide region

| Sanger position | Guide position | Guide sequence | Sanger base call | Focal base | Focal base peak area | p value |  |
| --- | --- | --- | --- | --- | --- | --- | --- |
| 279 | 1 | A | A | A | 94.15 | 0.0000000000 | \* |
| 279 | 1 | A | A | C | 1.41 | 0.4612790119 |  |
| 279 | 1 | A | A | G | 3.04 | 0.1739772844 |  |
| 279 | 1 | A | A | T | 1.41 | 0.6467808294 |  |
| 280 | 2 | A | A | A | 92.49 | 0.0000000000 | \* |
| 280 | 2 | A | A | C | 2.84 | 0.0793692831 |  |
| 280 | 2 | A | A | G | 3.65 | 0.1071801144 |  |
| 280 | 2 | A | A | T | 1.01 | 0.7661572513 |  |
| 281 | 3 | G | G | A | 2.80 | 0.1305787818 |  |
| 281 | 3 | G | G | C | 1.17 | 0.5732055825 |  |
| 281 | 3 | G | G | G | 95.79 | 0.0000000000 | \* |
| 281 | 3 | G | G | T | 0.23 | 0.9280257397 |  |
| 282 | 4 | A | A | A | 94.23 | 0.0000000000 | \* |
| 282 | 4 | A | A | C | 0.88 | 0.7146388226 |  |
| 282 | 4 | A | A | G | 3.14 | 0.1618949969 |  |
| 282 | 4 | A | A | T | 1.76 | 0.5396507504 |  |
| 283 | 5 | C | C | A | 2.67 | 0.1467235368 |  |
| 283 | 5 | C | C | C | 91.38 | 0.0000000000 | \* |
| 283 | 5 | C | C | G | 3.70 | 0.1032984817 |  |
| 283 | 5 | C | C | T | 2.26 | 0.4018706288 |  |
| 284 | 6 | T | T | A | 4.19 | 0.0371886424 |  |
| 284 | 6 | T | T | C | 1.01 | 0.6525830620 |  |
| 284 | 6 | T | T | G | 3.69 | 0.1037084797 |  |
| 284 | 6 | T | T | T | 91.11 | 0.0000000000 | \* |
| 285 | 7 | G | G | A | 1.50 | 0.3805826631 |  |
| 285 | 7 | G | G | C | 2.15 | 0.1997226670 |  |
| 285 | 7 | G | G | G | 95.38 | 0.0000000000 | \* |
| 285 | 7 | G | G | T | 0.97 | 0.7799282058 |  |
| 286 | 8 | G | G | A | 2.55 | 0.1629036405 |  |
| 286 | 8 | G | G | C | 1.27 | 0.5221965289 |  |
| 286 | 8 | G | G | G | 95.38 | 0.0000000000 | \* |
| 286 | 8 | G | G | T | 0.80 | 0.8267027616 |  |
| 287 | 9 | C | C | A | 2.06 | 0.2442394620 |  |
| 287 | 9 | C | C | C | 94.10 | 0.0000000000 | \* |
| 287 | 9 | C | C | G | 1.77 | 0.4310816800 |  |
| 287 | 9 | C | C | T | 2.06 | 0.4522760160 |  |
| 288 | 10 | C | C | A | 2.83 | 0.1274142081 |  |
| 288 | 10 | C | C | C | 91.33 | 0.0000000000 | \* |
| 288 | 10 | C | C | G | 4.42 | 0.0559661278 |  |
| 288 | 10 | C | C | T | 1.42 | 0.6434360625 |  |
| 289 | 11 | A | A | A | 90.44 | 0.0000000000 | \* |
| 289 | 11 | A | A | C | 1.17 | 0.5745360605 |  |
| 289 | 11 | A | A | G | 4.66 | 0.0455930043 |  |
| 289 | 11 | A | A | T | 3.73 | 0.1443011838 |  |
| 290 | 12 | G | G | A | 2.85 | 0.1259016319 |  |
| 290 | 12 | G | G | C | 3.05 | 0.0589144403 |  |
| 290 | 12 | G | G | G | 94.11 | 0.0000000000 | \* |
| 290 | 12 | G | G | T | 0.00 | 0.9361702128 |  |
| 291 | 13 | A | A | A | 94.61 | 0.0000000000 | \* |
| 291 | 13 | A | A | C | 1.00 | 0.6546449819 |  |
| 291 | 13 | A | A | G | 2.76 | 0.2167564553 |  |
| 291 | 13 | A | A | T | 1.63 | 0.5778751084 |  |
| 292 | 14 | C | C | A | 2.18 | 0.2217958146 |  |
| 292 | 14 | C | C | C | 91.82 | 0.0000000000 | \* |
| 292 | 14 | C | C | G | 4.00 | 0.0802675097 |  |
| 292 | 14 | C | C | T | 2.00 | 0.4699783033 |  |
| 293 | 15 | C | C | A | 5.42 | 0.0117368645 |  |
| 293 | 15 | C | C | C | 90.62 | 0.0000000000 | \* |
| 293 | 15 | C | C | G | 2.71 | 0.2247907391 |  |
| 293 | 15 | C | C | T | 1.25 | 0.6948922724 |  |
| 294 | 16 | G | G | A | 2.17 | 0.2248425539 |  |
| 294 | 16 | G | G | C | 0.98 | 0.6635803311 |  |
| 294 | 16 | G | G | G | 95.08 | 0.0000000000 | \* |
| 294 | 16 | G | G | T | 1.77 | 0.5352033362 |  |
| 295 | 17 | T | T | A | 0.00 | 0.8636363636 |  |
| 295 | 17 | T | T | C | 1.30 | 0.5120269004 |  |
| 295 | 17 | T | T | G | 3.63 | 0.1093203795 |  |
| 295 | 17 | T | T | T | 95.08 | 0.0000000000 | \* |
| 296 | 18 | G | G | A | 1.56 | 0.3633781108 |  |
| 296 | 18 | G | G | C | 0.39 | 0.8943610488 |  |
| 296 | 18 | G | G | G | 97.00 | 0.0000000000 | \* |
| 296 | 18 | G | G | T | 1.04 | 0.7576773551 |  |
| 297 | 19 | T | T | A | 1.38 | 0.4189686831 |  |
| 297 | 19 | T | T | C | 0.39 | 0.8939710522 |  |
| 297 | 19 | T | T | G | 4.32 | 0.0611066281 |  |
| 297 | 19 | T | T | T | 93.91 | 0.0000000000 | \* |
| 298 | 20 | T | T | A | 0.39 | 0.7725513629 |  |
| 298 | 20 | T | T | C | 0.39 | 0.8936319078 |  |
| 298 | 20 | T | T | G | 1.58 | 0.4849078522 |  |
| 298 | 20 | T | T | T | 97.63 | 0.0000000000 | \* |
| 299 | 21 | T | T | A | 0.00 | 0.8636363636 |  |
| 299 | 21 | T | T | C | 1.36 | 0.4822154871 |  |
| 299 | 21 | T | T | G | 4.08 | 0.0751954528 |  |
| 299 | 21 | T | T | T | 94.56 | 0.0000000000 | \* |
| 300 | 22 | G | G | A | 1.04 | 0.5329663358 |  |
| 300 | 22 | G | G | C | 1.63 | 0.3671332942 |  |
| 300 | 22 | G | G | G | 96.45 | 0.0000000000 | \* |
| 300 | 22 | G | G | T | 0.89 | 0.8022139586 |  |
| 301 | 23 | T | T | A | 0.00 | 0.8636363636 |  |
| 301 | 23 | T | T | C | 0.43 | 0.8846354001 |  |
| 301 | 23 | T | T | G | 3.25 | 0.1484572347 |  |
| 301 | 23 | T | T | T | 96.32 | 0.0000000000 | \* |
| 302 | 24 | G | G | A | 0.72 | 0.6529027360 |  |
| 302 | 24 | G | G | C | 1.25 | 0.5325320083 |  |
| 302 | 24 | G | G | G | 96.96 | 0.0000000000 | \* |
| 302 | 24 | G | G | T | 1.07 | 0.7486699942 |  |
| 303 | 25 | C | C | A | 1.31 | 0.4403890918 |  |
| 303 | 25 | C | C | C | 93.25 | 0.0000000000 | \* |
| 303 | 25 | C | C | G | 1.53 | 0.5002754046 |  |
| 303 | 25 | C | C | T | 3.92 | 0.1246950111 |  |
| 304 | 26 | A | A | A | 90.59 | 0.0000000000 | \* |
| 304 | 26 | A | A | C | 3.23 | 0.0455149927 |  |
| 304 | 26 | A | A | G | 4.30 | 0.0622187944 |  |
| 304 | 26 | A | A | T | 1.88 | 0.5032258628 |  |
| 305 | 27 | C | C | A | 0.79 | 0.6235934317 |  |
| 305 | 27 | C | C | C | 94.85 | 0.0000000000 | \* |
| 305 | 27 | C | C | G | 2.18 | 0.3291811699 |  |
| 305 | 27 | C | C | T | 2.18 | 0.4223453084 |  |
| 306 | 28 | T | T | A | 2.21 | 0.2174079991 |  |
| 306 | 28 | T | T | C | 1.10 | 0.6052331328 |  |
| 306 | 28 | T | T | G | 0.00 | 0.8478260870 |  |
| 306 | 28 | T | T | T | 96.69 | 0.0000000000 | \* |
| 307 | 29 | A | A | A | 94.18 | 0.0000000000 | \* |
| 307 | 29 | A | A | C | 4.31 | 0.0085650785 | \* |
| 307 | 29 | A | A | G | 0.00 | 0.8478260870 |  |
| 307 | 29 | A | A | T | 1.51 | 0.6147483125 |  |
| 308 | 30 | C | C | A | 1.82 | 0.2980950591 |  |
| 308 | 30 | C | C | C | 93.94 | 0.0000000000 | \* |
| 308 | 30 | C | C | G | 1.21 | 0.5945435412 |  |
| 308 | 30 | C | C | T | 3.03 | 0.2404789543 |  |
| 309 | 31 | A | A | A | 93.97 | 0.0000000000 | \* |
| 309 | 31 | A | A | C | 2.47 | 0.1325103163 |  |
| 309 | 31 | A | A | G | 3.56 | 0.1152973620 |  |
| 309 | 31 | A | A | T | 0.00 | 0.9361702128 |  |
| 310 | 32 | C | C | A | 0.94 | 0.5665976177 |  |
| 310 | 32 | C | C | C | 97.41 | 0.0000000000 | \* |
| 310 | 32 | C | C | G | 0.00 | 0.8478260870 |  |
| 310 | 32 | C | C | T | 1.65 | 0.5712512659 |  |
| 311 | 33 | G | G | A | 1.64 | 0.3418462951 |  |
| 311 | 33 | G | G | C | 1.64 | 0.3606104090 |  |
| 311 | 33 | G | G | G | 96.71 | 0.0000000000 | \* |
| 311 | 33 | G | G | T | 0.00 | 0.9361702128 |  |
| 312 | 34 | G | G | A | 0.00 | 0.8636363636 |  |
| 312 | 34 | G | G | C | 2.08 | 0.2164795106 |  |
| 312 | 34 | G | G | G | 97.35 | 0.0000000000 | \* |
| 312 | 34 | G | G | T | 0.57 | 0.8798218491 |  |
| 313 | 35 | G | G | A | 1.37 | 0.4206355294 |  |
| 313 | 35 | G | G | C | 1.14 | 0.5862608604 |  |
| 313 | 35 | G | G | G | 96.35 | 0.0000000000 | \* |
| 313 | 35 | G | G | T | 1.14 | 0.7281287514 |  |
| 314 | 36 | C | C | A | 3.09 | 0.1012702454 |  |
| 314 | 36 | C | C | C | 91.75 | 0.0000000000 | \* |
| 314 | 36 | C | C | G | 2.84 | 0.2043303839 |  |
| 314 | 36 | C | C | T | 2.32 | 0.3868410435 |  |
| 315 | 37 | A | A | A | 91.48 | 0.0000000000 | \* |
| 315 | 37 | A | A | C | 3.01 | 0.0625214956 |  |
| 315 | 37 | A | A | G | 2.76 | 0.2167564553 |  |
| 315 | 37 | A | A | T | 2.76 | 0.2904866650 |  |
| 316 | 38 | C | C | A | 0.70 | 0.6605466999 |  |
| 316 | 38 | C | C | C | 95.30 | 0.0000000000 | \* |
| 316 | 38 | C | C | G | 0.87 | 0.6978983834 |  |
| 316 | 38 | C | C | T | 3.13 | 0.2240323021 |  |
| 317 | 39 | C | C | A | 1.73 | 0.3202555794 |  |
| 317 | 39 | C | C | C | 93.86 | 0.0000000000 | \* |
| 317 | 39 | C | C | G | 1.54 | 0.4972191178 |  |
| 317 | 39 | C | C | T | 2.88 | 0.2671944721 |  |
| 318 | 40 | C | C | A | 1.43 | 0.4035858657 |  |
| 318 | 40 | C | C | C | 94.70 | 0.0000000000 | \* |
| 318 | 40 | C | C | G | 0.61 | 0.7676357955 |  |
| 318 | 40 | C | C | T | 3.26 | 0.2043593386 |  |
| 319 | 41 | T | T | A | 0.00 | 0.8636363636 |  |
| 319 | 41 | T | T | C | 1.15 | 0.5845519026 |  |
| 319 | 41 | T | T | G | 2.10 | 0.3474485674 |  |
| 319 | 41 | T | T | T | 96.76 | 0.0000000000 | \* |
| 320 | 42 | G | G | A | 2.22 | 0.2157315377 |  |
| 320 | 42 | G | G | C | 2.37 | 0.1496874333 |  |
| 320 | 42 | G | G | G | 94.46 | 0.0000000000 | \* |
| 320 | 42 | G | G | T | 0.95 | 0.7848852996 |  |
| 321 | 43 | A | A | A | 86.41 | 0.0000000000 | \* |
| 321 | 43 | A | A | C | 1.92 | 0.2648060170 |  |
| 321 | 43 | A | A | G | 4.88 | 0.0377524111 |  |
| 321 | 43 | A | A | T | 6.79 | 0.0115492553 |  |
| 322 | 44 | C | C | A | 1.47 | 0.3911359466 |  |
| 322 | 44 | C | C | C | 95.81 | 0.0000000000 | \* |
| 322 | 44 | C | C | G | 0.00 | 0.8478260870 |  |
| 322 | 44 | C | C | T | 2.73 | 0.2967495197 |  |
| 323 | 45 | C | C | A | 2.71 | 0.1418074169 |  |
| 323 | 45 | C | C | C | 90.29 | 0.0000000000 | \* |
| 323 | 45 | C | C | G | 4.29 | 0.0628662634 |  |
| 323 | 45 | C | C | T | 2.71 | 0.3000820459 |  |
| 324 | 46 | G | G | A | 3.45 | 0.0736769324 |  |
| 324 | 46 | G | G | C | 2.96 | 0.0673419746 |  |
| 324 | 46 | G | G | G | 93.10 | 0.0000000000 | \* |
| 324 | 46 | G | G | T | 0.49 | 0.8942259774 |  |
| 325 | 47 | A | A | A | 93.88 | 0.0000000000 | \* |
| 325 | 47 | A | A | C | 1.83 | 0.2912346772 |  |
| 325 | 47 | A | A | G | 1.99 | 0.3744311593 |  |
| 325 | 47 | A | A | T | 2.29 | 0.3932164423 |  |
| 326 | 48 | C | C | A | 1.81 | 0.3000506985 |  |
| 326 | 48 | C | C | C | 92.76 | 0.0000000000 | \* |
| 326 | 48 | C | C | G | 1.13 | 0.6193351529 |  |
| 326 | 48 | C | C | T | 4.30 | 0.0930259536 |  |
| 327 | 49 | G | G | A | 6.69 | 0.0034295004 | \* |
| 327 | 49 | G | G | C | 1.27 | 0.5221965289 |  |
| 327 | 49 | G | G | G | 92.04 | 0.0000000000 | \* |
| 327 | 49 | G | G | T | 0.00 | 0.9361702128 |  |
| 328 | 50 | G | G | A | 1.44 | 0.4004035616 |  |
| 328 | 50 | G | G | C | 3.23 | 0.0451286938 |  |
| 328 | 50 | G | G | G | 95.33 | 0.0000000000 | \* |
| 328 | 50 | G | G | T | 0.00 | 0.9361702128 |  |
| 329 | 51 | C | C | A | 1.97 | 0.2630922103 |  |
| 329 | 51 | C | C | C | 94.74 | 0.0000000000 | \* |
| 329 | 51 | C | C | G | 1.97 | 0.3779390093 |  |
| 329 | 51 | C | C | T | 1.32 | 0.6745254618 |  |
| 330 | 52 | A | A | A | 94.65 | 0.0000000000 | \* |
| 330 | 52 | A | A | C | 1.23 | 0.5410163135 |  |
| 330 | 52 | A | A | G | 2.47 | 0.2680246573 |  |
| 330 | 52 | A | A | T | 1.65 | 0.5727188199 |  |
| 331 | 53 | A | A | A | 95.22 | 0.0000000000 | \* |
| 331 | 53 | A | A | C | 0.46 | 0.8788009517 |  |
| 331 | 53 | A | A | G | 3.19 | 0.1553771812 |  |
| 331 | 53 | A | A | T | 1.14 | 0.7289180178 |  |
| 332 | 54 | G | G | A | 4.20 | 0.0370217857 |  |
| 332 | 54 | G | G | C | 0.00 | 0.9268292683 |  |
| 332 | 54 | G | G | G | 95.54 | 0.0000000000 | \* |
| 332 | 54 | G | G | T | 0.26 | 0.9255661212 |  |
| 333 | 55 | A | A | A | 97.01 | 0.0000000000 | \* |
| 333 | 55 | A | A | C | 0.43 | 0.8860043944 |  |
| 333 | 55 | A | A | G | 1.28 | 0.5731513811 |  |
| 333 | 55 | A | A | T | 1.28 | 0.6849820092 |  |
| 334 | 56 | A | A | A | 94.02 | 0.0000000000 | \* |
| 334 | 56 | A | A | C | 0.00 | 0.9268292683 |  |
| 334 | 56 | A | A | G | 2.30 | 0.3026547862 |  |
| 334 | 56 | A | A | T | 3.68 | 0.1500018553 |  |
| 335 | 57 | G | G | A | 2.19 | 0.2194997990 |  |
| 335 | 57 | G | G | C | 0.63 | 0.8232438932 |  |
| 335 | 57 | G | G | G | 92.79 | 0.0000000000 | \* |
| 335 | 57 | G | G | T | 4.39 | 0.0866418808 |  |
| 336 | 58 | T | T | A | 2.13 | 0.2319569536 |  |
| 336 | 58 | T | T | C | 0.00 | 0.9268292683 |  |
| 336 | 58 | T | T | G | 0.47 | 0.7986327251 |  |
| 336 | 58 | T | T | T | 97.40 | 0.0000000000 | \* |
| 337 | 59 | T | T | A | 5.34 | 0.0126321400 |  |
| 337 | 59 | T | T | C | 2.18 | 0.1908381047 |  |
| 337 | 59 | T | T | G | 1.21 | 0.5940928061 |  |
| 337 | 59 | T | T | T | 91.26 | 0.0000000000 | \* |
| 338 | 60 | C | C | A | 0.25 | 0.8181064817 |  |
| 338 | 60 | C | C | C | 96.73 | 0.0000000000 | \* |
| 338 | 60 | C | C | G | 2.27 | 0.3094964932 |  |
| 338 | 60 | C | C | T | 0.76 | 0.8370477672 |  |
| 339 | 61 | G | G | A | 6.25 | 0.0052542770 | \* |
| 339 | 61 | G | G | C | 1.49 | 0.4246647015 |  |
| 339 | 61 | G | G | G | 91.96 | 0.0000000000 | \* |
| 339 | 61 | G | G | T | 0.30 | 0.9221081721 |  |
| 340 | 62 | A | A | A | 95.09 | 0.0000000000 | \* |
| 340 | 62 | A | A | C | 1.45 | 0.4392800096 |  |
| 340 | 62 | A | A | G | 1.82 | 0.4181002289 |  |
| 340 | 62 | A | A | T | 1.64 | 0.5756641376 |  |
| 341 | 63 | C | C | A | 1.89 | 0.2804019376 |  |
| 341 | 63 | C | C | C | 95.58 | 0.0000000000 | \* |
| 341 | 63 | C | C | G | 0.00 | 0.8478260870 |  |
| 341 | 63 | C | C | T | 2.53 | 0.3387353039 |  |
| 342 | 64 | A | A | A | 90.46 | 0.0000000000 | \* |
| 342 | 64 | A | A | C | 1.54 | 0.4032495676 |  |
| 342 | 64 | A | A | G | 6.46 | 0.0090307373 | \* |
| 342 | 64 | A | A | T | 1.54 | 0.6055600692 |  |
| 343 | 65 | G | G | A | 0.40 | 0.7711908743 |  |
| 343 | 65 | G | G | C | 2.99 | 0.0642931707 |  |
| 343 | 65 | G | G | G | 95.22 | 0.0000000000 | \* |
| 343 | 65 | G | G | T | 1.39 | 0.6501119554 |  |
| 344 | 66 | C | C | A | 0.49 | 0.7378827414 |  |
| 344 | 66 | C | C | C | 93.12 | 0.0000000000 | \* |
| 344 | 66 | C | C | G | 1.72 | 0.4447746227 |  |
| 344 | 66 | C | C | T | 4.67 | 0.0693118393 |  |
| 345 | 67 | T | T | A | 0.00 | 0.8636363636 |  |
| 345 | 67 | T | T | C | 2.03 | 0.2301684716 |  |
| 345 | 67 | T | T | G | 0.18 | 0.8408005258 |  |
| 345 | 67 | T | T | T | 97.78 | 0.0000000000 | \* |
| 346 | 68 | C | C | A | 0.00 | 0.8636363636 |  |
| 346 | 68 | C | C | C | 96.98 | 0.0000000000 | \* |
| 346 | 68 | C | C | G | 0.00 | 0.8478260870 |  |
| 346 | 68 | C | C | T | 3.02 | 0.2428667902 |  |
| 347 | 69 | C | C | A | 0.88 | 0.5895690290 |  |
| 347 | 69 | C | C | C | 94.18 | 0.0000000000 | \* |
| 347 | 69 | C | C | G | 0.00 | 0.8478260870 |  |
| 347 | 69 | C | C | T | 4.94 | 0.0556910441 |  |
| 348 | 70 | C | C | A | 0.00 | 0.8636363636 |  |
| 348 | 70 | C | C | C | 94.10 | 0.0000000000 | \* |
| 348 | 70 | C | C | G | 3.65 | 0.1071301288 |  |
| 348 | 70 | C | C | T | 2.25 | 0.4047632051 |  |
| 349 | 71 | G | G | A | 4.32 | 0.0329626544 |  |
| 349 | 71 | G | G | C | 2.70 | 0.0960839076 |  |
| 349 | 71 | G | G | G | 91.62 | 0.0000000000 | \* |
| 349 | 71 | G | G | T | 1.35 | 0.6634873300 |  |
| 350 | 72 | C | C | A | 3.69 | 0.0589225812 |  |
| 350 | 72 | C | C | C | 91.87 | 0.0000000000 | \* |
| 350 | 72 | C | C | G | 1.97 | 0.3787493360 |  |
| 350 | 72 | C | C | T | 2.46 | 0.3529693981 |  |
| 351 | 73 | G | G | A | 6.08 | 0.0062120269 | \* |
| 351 | 73 | G | G | C | 1.66 | 0.3553200388 |  |
| 351 | 73 | G | G | G | 91.99 | 0.0000000000 | \* |
| 351 | 73 | G | G | T | 0.28 | 0.9242707250 |  |
| 352 | 74 | A | A | A | 92.84 | 0.0000000000 | \* |
| 352 | 74 | A | A | C | 3.38 | 0.0362288066 |  |
| 352 | 74 | A | A | G | 0.80 | 0.7191073605 |  |
| 352 | 74 | A | A | T | 2.98 | 0.2487444648 |  |
| 353 | 75 | C | C | A | 0.76 | 0.6340156992 |  |
| 353 | 75 | C | C | C | 83.37 | 0.0000000000 | \* |
| 353 | 75 | C | C | G | 10.13 | 0.0002676343 | \* |
| 353 | 75 | C | C | T | 5.74 | 0.0286962338 |  |

## For use in R

If you want to work with the results in R, here is output that you can copy and paste in your terminal to get:

The base information:

```
structure(list(focal.base = c("A", "C", "G", "T"), avg.percsignal = c(93.1443739808708, 
94.0466497278486, 94.9011062826826, 96.1160252405613), avg.areasignal = c(443.176470588235, 
470.014492753623, 498.618181818182, 537.209302325581), crit.perc.area = c(5.5837154338451, 
4.21318097513766, 6.35119012957956, 6.95888811182574), mu = c(1.63106488838101, 
1.55956137571841, 2.0901614154138, 2.31250102930712), fillibens = c(0.996629543776045, 
0.992356905795086, 0.995143857233486, 0.996082794292603)), .Names = c("focal.base", 
"avg.percsignal", "avg.areasignal", "crit.perc.area", "mu", "fillibens"
), row.names = c(NA, -4L), class = "data.frame")
```

the data.frame that contains information on the guide region:

```
structure(list(A.area = c(402, 456, 12, 751, 13, 25, 14, 16, 
7, 16, 388, 14, 755, 12, 26, 11, 0, 12, 7, 2, 0, 7, 0, 4, 6, 
337, 4, 12, 437, 9, 343, 4, 6, 0, 6, 12, 365, 4, 9, 7, 0, 14, 
496, 7, 12, 14, 614, 8, 21, 8, 6, 230, 418, 16, 454, 409, 7, 
9, 22, 1, 21, 523, 9, 294, 2, 2, 0, 0, 5, 0, 16, 15, 22, 467, 
4), C.area = c(6, 14, 5, 7, 445, 6, 20, 8, 319, 516, 5, 15, 8, 
505, 435, 5, 5, 3, 2, 2, 7, 11, 2, 7, 428, 12, 479, 6, 20, 465, 
9, 413, 6, 11, 5, 356, 12, 548, 489, 465, 6, 15, 11, 457, 400, 
12, 12, 410, 4, 18, 288, 3, 2, 0, 2, 0, 2, 0, 9, 384, 5, 8, 454, 
5, 15, 379, 11, 418, 534, 335, 10, 373, 6, 17, 436), G.area = c(13, 
18, 410, 25, 18, 22, 888, 599, 6, 25, 20, 463, 22, 22, 13, 483, 
14, 744, 22, 8, 21, 652, 15, 542, 7, 16, 11, 0, 0, 6, 13, 0, 
353, 514, 422, 11, 11, 5, 8, 3, 11, 597, 28, 0, 19, 378, 13, 
5, 289, 531, 6, 6, 14, 364, 6, 10, 296, 2, 5, 9, 309, 10, 0, 
21, 478, 7, 1, 0, 0, 13, 339, 8, 333, 4, 53), T.area = c(6, 5, 
1, 14, 11, 543, 9, 5, 7, 8, 16, 0, 13, 11, 6, 9, 367, 8, 478, 
495, 487, 6, 445, 6, 18, 7, 11, 526, 7, 15, 0, 7, 0, 3, 5, 9, 
11, 18, 15, 16, 507, 6, 39, 13, 12, 2, 15, 19, 0, 0, 4, 4, 5, 
1, 6, 16, 14, 412, 376, 3, 1, 9, 12, 5, 7, 19, 529, 13, 28, 8, 
5, 10, 1, 15, 30), Tot.area = c(427, 493, 428, 797, 487, 596, 
931, 628, 339, 565, 429, 492, 798, 550, 480, 508, 386, 767, 509, 
507, 515, 676, 462, 559, 459, 372, 505, 544, 464, 495, 365, 424, 
365, 528, 438, 388, 399, 575, 521, 491, 524, 632, 574, 477, 443, 
406, 654, 442, 314, 557, 304, 243, 439, 381, 468, 435, 319, 423, 
412, 397, 336, 550, 475, 325, 502, 407, 541, 431, 567, 356, 370, 
406, 362, 503, 523), A.perc = c(94.1451990632319, 92.4949290060852, 
2.80373831775701, 94.228356336261, 2.6694045174538, 4.19463087248322, 
1.50375939849624, 2.54777070063694, 2.06489675516224, 2.83185840707965, 
90.4428904428904, 2.84552845528455, 94.6115288220551, 2.18181818181818, 
5.41666666666667, 2.16535433070866, 0, 1.5645371577575, 1.37524557956778, 
0.394477317554241, 0, 1.03550295857988, 0, 0.715563506261181, 
1.30718954248366, 90.5913978494624, 0.792079207920792, 2.20588235294118, 
94.1810344827586, 1.81818181818182, 93.972602739726, 0.943396226415094, 
1.64383561643836, 0, 1.36986301369863, 3.09278350515464, 91.4786967418546, 
0.695652173913043, 1.72744721689059, 1.42566191446029, 0, 2.21518987341772, 
86.411149825784, 1.46750524109015, 2.70880361173815, 3.44827586206897, 
93.8837920489297, 1.80995475113122, 6.68789808917197, 1.43626570915619, 
1.97368421052632, 94.6502057613169, 95.2164009111617, 4.1994750656168, 
97.008547008547, 94.0229885057471, 2.19435736677116, 2.12765957446809, 
5.33980582524272, 0.251889168765743, 6.25, 95.0909090909091, 
1.89473684210526, 90.4615384615385, 0.398406374501992, 0.491400491400491, 
0, 0, 0.881834215167548, 0, 4.32432432432432, 3.69458128078818, 
6.07734806629834, 92.8429423459245, 0.764818355640535), C.perc = c(1.40515222482436, 
2.83975659229209, 1.16822429906542, 0.878293601003764, 91.3757700205339, 
1.00671140939597, 2.14822771213749, 1.27388535031847, 94.1002949852507, 
91.3274336283186, 1.16550116550117, 3.04878048780488, 1.00250626566416, 
91.8181818181818, 90.625, 0.984251968503937, 1.29533678756477, 
0.391134289439374, 0.392927308447937, 0.394477317554241, 1.35922330097087, 
1.62721893491124, 0.432900432900433, 1.25223613595707, 93.2461873638344, 
3.2258064516129, 94.8514851485149, 1.10294117647059, 4.31034482758621, 
93.9393939393939, 2.46575342465753, 97.4056603773585, 1.64383561643836, 
2.08333333333333, 1.14155251141553, 91.7525773195876, 3.00751879699248, 
95.304347826087, 93.8579654510557, 94.7046843177189, 1.14503816793893, 
2.37341772151899, 1.91637630662021, 95.8071278825996, 90.293453724605, 
2.95566502463054, 1.8348623853211, 92.7601809954751, 1.27388535031847, 
3.23159784560144, 94.7368421052632, 1.23456790123457, 0.455580865603645, 
0, 0.427350427350427, 0, 0.626959247648903, 0, 2.18446601941748, 
96.7254408060453, 1.48809523809524, 1.45454545454545, 95.5789473684211, 
1.53846153846154, 2.98804780876494, 93.1203931203931, 2.03327171903882, 
96.9837587006961, 94.1798941798942, 94.1011235955056, 2.7027027027027, 
91.871921182266, 1.65745856353591, 3.37972166998012, 83.3652007648184
), G.perc = c(3.04449648711944, 3.65111561866126, 95.7943925233645, 
3.13676286072773, 3.69609856262834, 3.69127516778523, 95.3813104189044, 
95.3821656050955, 1.76991150442478, 4.42477876106195, 4.66200466200466, 
94.1056910569106, 2.75689223057644, 4, 2.70833333333333, 95.0787401574803, 
3.62694300518135, 97.0013037809648, 4.32220039292731, 1.57790927021696, 
4.07766990291262, 96.4497041420118, 3.24675324675325, 96.95885509839, 
1.52505446623094, 4.3010752688172, 2.17821782178218, 0, 0, 1.21212121212121, 
3.56164383561644, 0, 96.7123287671233, 97.3484848484848, 96.3470319634703, 
2.83505154639175, 2.75689223057644, 0.869565217391304, 1.53550863723608, 
0.610997963340122, 2.09923664122137, 94.4620253164557, 4.8780487804878, 
0, 4.28893905191874, 93.1034482758621, 1.98776758409786, 1.13122171945701, 
92.0382165605096, 95.3321364452424, 1.97368421052632, 2.46913580246914, 
3.18906605922551, 95.5380577427822, 1.28205128205128, 2.29885057471264, 
92.7899686520376, 0.472813238770686, 1.21359223300971, 2.26700251889169, 
91.9642857142857, 1.81818181818182, 0, 6.46153846153846, 95.2191235059761, 
1.71990171990172, 0.184842883548983, 0, 0, 3.65168539325843, 
91.6216216216216, 1.97044334975369, 91.9889502762431, 0.795228628230616, 
10.1338432122371), T.perc = c(1.40515222482436, 1.01419878296146, 
0.233644859813084, 1.75658720200753, 2.25872689938398, 91.1073825503356, 
0.966702470461869, 0.796178343949045, 2.06489675516224, 1.41592920353982, 
3.72960372960373, 0, 1.62907268170426, 2, 1.25, 1.77165354330709, 
95.0777202072539, 1.04302477183833, 93.909626719057, 97.6331360946746, 
94.5631067961165, 0.887573964497041, 96.3203463203463, 1.07334525939177, 
3.92156862745098, 1.88172043010753, 2.17821782178218, 96.6911764705882, 
1.50862068965517, 3.03030303030303, 0, 1.65094339622642, 0, 0.568181818181818, 
1.14155251141553, 2.31958762886598, 2.75689223057644, 3.1304347826087, 
2.87907869481766, 3.25865580448065, 96.7557251908397, 0.949367088607595, 
6.79442508710801, 2.72536687631027, 2.70880361173815, 0.492610837438424, 
2.29357798165138, 4.29864253393665, 0, 0, 1.31578947368421, 1.64609053497942, 
1.13895216400911, 0.26246719160105, 1.28205128205128, 3.67816091954023, 
4.38871473354232, 97.3995271867612, 91.2621359223301, 0.755667506297229, 
0.297619047619048, 1.63636363636364, 2.52631578947368, 1.53846153846154, 
1.39442231075697, 4.66830466830467, 97.7818853974122, 3.01624129930394, 
4.93827160493827, 2.24719101123596, 1.35135135135135, 2.46305418719212, 
0.276243093922652, 2.98210735586481, 5.73613766730401), base.call = c("A", 
"A", "G", "A", "C", "T", "G", "G", "C", "C", "A", "G", "A", "C", 
"C", "G", "T", "G", "T", "T", "T", "G", "T", "G", "C", "A", "C", 
"T", "A", "C", "A", "C", "G", "G", "G", "C", "A", "C", "C", "C", 
"T", "G", "A", "C", "C", "G", "A", "C", "G", "G", "C", "A", "A", 
"G", "A", "A", "G", "T", "T", "C", "G", "A", "C", "A", "G", "C", 
"T", "C", "C", "C", "G", "C", "G", "A", "C"), index = 279:353, 
    guide.seq = c("A", "A", "G", "A", "C", "T", "G", "G", "C", 
    "C", "A", "G", "A", "C", "C", "G", "T", "G", "T", "T", "T", 
    "G", "T", "G", "C", "A", "C", "T", "A", "C", "A", "C", "G", 
    "G", "G", "C", "A", "C", "C", "C", "T", "G", "A", "C", "C", 
    "G", "A", "C", "G", "G", "C", "A", "A", "G", "A", "A", "G", 
    "T", "T", "C", "G", "A", "C", "A", "G", "C", "T", "C", "C", 
    "C", "G", "C", "G", "A", "C"), T.pval = c(0.646780829392201, 
    0.766157251294935, 0.928025739708659, 0.539650750403795, 
    0.40187062882631, 0, 0.779928205775366, 0.826702761609044, 
    0.452276015989642, 0.643436062497991, 0.144301183804531, 
    0.936170212765957, 0.577875108350586, 0.469978303256466, 
    0.694892272422215, 0.535203336165575, 0, 0.757677355059338, 
    0, 0, 0, 0.802213958551802, 0, 0.748669994215343, 0.124695011078676, 
    0.503225862835986, 0.422345308390476, 0, 0.6147483125186, 
    0.24047895431234, 0.936170212765957, 0.571251265862938, 0.936170212765957, 
    0.879821849101061, 0.728128751361097, 0.386841043520827, 
    0.29048666497246, 0.224032302143055, 0.267194472081497, 0.204359338607365, 
    0, 0.784885299606358, 0.0115492552751646, 0.296749519704794, 
    0.300082045949278, 0.894225977397614, 0.393216442340768, 
    0.0930259536413445, 0.936170212765957, 0.936170212765957, 
    0.674525461816549, 0.572718819903437, 0.728918017825473, 
    0.925566121153052, 0.684982009232074, 0.150001855280069, 
    0.0866418808114222, 0, 0, 0.837047767240535, 0.922108172054356, 
    0.57566413762939, 0.338735303900915, 0.60556006922611, 0.65011195538489, 
    0.0693118393006393, 0, 0.242866790166303, 0.0556910441312315, 
    0.404763205120152, 0.663487330033441, 0.352969398088745, 
    0.924270725046085, 0.248744464831544, 0.0286962338241388), 
    C.pval = c(0.461279011858568, 0.0793692831050571, 0.573205582507875, 
    0.714638822591122, 0, 0.652583061977762, 0.199722666993651, 
    0.522196528940787, 0, 0, 0.574536060548892, 0.0589144402695584, 
    0.654644981870679, 0, 0, 0.663580331107253, 0.512026900375594, 
    0.894361048775659, 0.89397105218532, 0.893631907789308, 0.482215487137587, 
    0.367133294204586, 0.884635400148187, 0.532532008281708, 
    0, 0.0455149926850548, 0, 0.6052331327833, 0.00856507851702182, 
    0, 0.132510316305722, 0, 0.360610408985346, 0.216479510644652, 
    0.586260860412315, 0, 0.0625214956043304, 0, 0, 0, 0.584551902597399, 
    0.149687433286432, 0.264806016998962, 0, 0, 0.0673419746118554, 
    0.291234677211864, 0, 0.522196528940787, 0.0451286938467156, 
    0, 0.541016313496341, 0.878800951685846, 0.926829268292683, 
    0.886004394398518, 0.926829268292683, 0.823243893185747, 
    0.926829268292683, 0.190838104721546, 0, 0.42466470154623, 
    0.439280009600362, 0, 0.403249567556368, 0.064293170693996, 
    0, 0.230168471623566, 0, 0, 0, 0.0960839076170301, 0, 0.35532003882894, 
    0.0362288065537004, 0), G.pval = c(0.173977284414149, 0.107180114399727, 
    0, 0.161894996941359, 0.103298481653072, 0.103708479681615, 
    0, 0, 0.431081680048136, 0.0559661277564525, 0.0455930043197652, 
    0, 0.216756455313257, 0.0802675096577948, 0.224790739070062, 
    0, 0.109320379490461, 0, 0.0611066280935878, 0.484907852243024, 
    0.0751954527805626, 0, 0.148457234714441, 0, 0.500275404647237, 
    0.062218794403969, 0.329181169907576, 0.847826086956522, 
    0.847826086956522, 0.594543541238216, 0.115297362031021, 
    0.847826086956522, 0, 0, 0, 0.204330383929991, 0.216756455313257, 
    0.697898383445603, 0.49721911781855, 0.767635795509718, 0.347448567410408, 
    0, 0.0377524110507937, 0.847826086956522, 0.0628662633911508, 
    0, 0.374431159301165, 0.619335152906946, 0, 0, 0.377939009311014, 
    0.268024657300966, 0.155377181185613, 0, 0.573151381142379, 
    0.302654786181908, 0, 0.798632725078799, 0.594092806079206, 
    0.309496493206889, 0, 0.418100228911762, 0.847826086956522, 
    0.00903073728938808, 0, 0.444774622671694, 0.840800525808471, 
    0.847826086956522, 0.847826086956522, 0.107130128824275, 
    0, 0.378749335984298, 0, 0.719107360524778, 0.00026763425071652
    ), A.pval = c(0, 0, 0.130578781818857, 0, 0.146723536795288, 
    0.0371886424267256, 0.380582663123692, 0.162903640521832, 
    0.244239462014323, 0.127414208132741, 0, 0.125901631905582, 
    0, 0.221795814577524, 0.0117368645020197, 0.224842553937341, 
    0.863636363636362, 0.363378110846261, 0.418968683146101, 
    0.772551362933728, 0.863636363636362, 0.53296633582802, 0.863636363636362, 
    0.652902735974068, 0.440389091750201, 0, 0.623593431683829, 
    0.217407999146621, 0, 0.298095059144674, 0, 0.566597617666505, 
    0.341846295143533, 0.863636363636362, 0.420635529412839, 
    0.101270245380396, 0, 0.660546699900844, 0.320255579386406, 
    0.403585865719466, 0.863636363636362, 0.215731537681778, 
    0, 0.391135946572274, 0.141807416931427, 0.0736769324386366, 
    0, 0.300050698531777, 0.00342950040786283, 0.400403561593175, 
    0.263092210253343, 0, 0, 0.0370217857443966, 0, 0, 0.219499799003802, 
    0.231956953644337, 0.0126321400339711, 0.81810648170008, 
    0.00525427699916947, 0, 0.280401937560972, 0, 0.771190874299203, 
    0.737882741364564, 0.863636363636362, 0.863636363636362, 
    0.589569028996585, 0.863636363636362, 0.0329626543942163, 
    0.0589225811529406, 0.00621202688813582, 0, 0.634015699196542
    ), guide.position = 1:75), .Names = c("A.area", "C.area", 
"G.area", "T.area", "Tot.area", "A.perc", "C.perc", "G.perc", 
"T.perc", "base.call", "index", "guide.seq", "T.pval", "C.pval", 
"G.pval", "A.pval", "guide.position"), row.names = 279:353, class = "data.frame")
```

*Report generated using EditR v1.0.8*
