# Supplementary material for: Mechanistic and genetic basis of single-strand templated repair at Cas12a-induced DNA breaks in Chlamydomonas reinhardtii
Source: Nat Commun. 2021 Nov 19;12:6751. doi: 10.1038/s41467-021-27004-1 (PMC8604939; doi:10.1038/s41467-021-27004-1)
Supplement: Supplementary file 22 — Source Data [file 41467_2021_27004_MOESM22_ESM.zip › Source Data/EditR analysis/EditR outputs/Sense/rep2_ssODN_sense_0.html]

EditR v1.0.8 report


# EditR v1.0.8 report

- Data QA
  - Filtering data
  - Percent noise peak area
  - Base information
- Predicted editing
  - Editing bar plot
  - Editing table plot
  - Table of editing results
- For use in R

## Data QA

### Filtering data

What the data looked like prefiltering:

and the post filtering signal / noise plot:

### Percent noise peak area

### Base information

Here’s information about the signal of each base, the critical percent value where any higher value would be called as significant, and Filliben’s correlation for how well the noise was modelled by the zero adjusted gamma distribution.

| Base | Average percent signal | Average peak area | Critical percent value | model mu | Fillibens correlation |
| --- | --- | --- | --- | --- | --- |
| A | 91.33277 | 582.6250 | 7.281139 | 2.646797 | 0.9961720 |
| C | 91.40398 | 610.0000 | 8.566878 | 3.168212 | 0.9909777 |
| G | 93.10164 | 618.6316 | 8.127501 | 2.739425 | 0.9962989 |
| T | 93.18806 | 663.3953 | 8.685973 | 2.891490 | 0.9958472 |

## Predicted editing

### Editing bar plot

### Editing table plot

### Table of editing results


Here’s the entire guide region

| Sanger position | Guide position | Guide sequence | Sanger base call | Focal base | Focal base peak area | p value |  |
| --- | --- | --- | --- | --- | --- | --- | --- |
| 285 | 1 | A | A | A | 94.60 | 0.000000e+00 | \* |
| 285 | 1 | A | A | C | 2.96 | 4.074519e-01 |  |
| 285 | 1 | A | A | G | 2.44 | 4.376901e-01 |  |
| 285 | 1 | A | A | T | 0.00 | 9.148936e-01 |  |
| 286 | 2 | A | A | A | 92.98 | 0.000000e+00 | \* |
| 286 | 2 | A | A | C | 3.72 | 2.729468e-01 |  |
| 286 | 2 | A | A | G | 1.29 | 7.184534e-01 |  |
| 286 | 2 | A | A | T | 2.01 | 5.715148e-01 |  |
| 287 | 3 | G | G | A | 2.11 | 4.553635e-01 |  |
| 287 | 3 | G | G | C | 3.70 | 2.772012e-01 |  |
| 287 | 3 | G | G | G | 94.19 | 0.000000e+00 | \* |
| 287 | 3 | G | G | T | 0.00 | 9.148936e-01 |  |
| 288 | 4 | A | A | A | 93.91 | 0.000000e+00 | \* |
| 288 | 4 | A | A | C | 0.90 | 8.219645e-01 |  |
| 288 | 4 | A | A | G | 2.95 | 3.329038e-01 |  |
| 288 | 4 | A | A | T | 2.24 | 5.172427e-01 |  |
| 289 | 5 | C | C | A | 0.00 | 8.035714e-01 |  |
| 289 | 5 | C | C | C | 98.25 | 0.000000e+00 | \* |
| 289 | 5 | C | C | G | 0.00 | 9.000000e-01 |  |
| 289 | 5 | C | C | T | 1.75 | 6.319622e-01 |  |
| 290 | 6 | T | T | A | 0.00 | 8.035714e-01 |  |
| 290 | 6 | T | T | C | 5.36 | 9.996893e-02 |  |
| 290 | 6 | T | T | G | 2.28 | 4.739895e-01 |  |
| 290 | 6 | T | T | T | 92.36 | 0.000000e+00 | \* |
| 291 | 7 | G | G | A | 1.95 | 4.941615e-01 |  |
| 291 | 7 | G | G | C | 0.00 | 8.658537e-01 |  |
| 291 | 7 | G | G | G | 97.20 | 0.000000e+00 | \* |
| 291 | 7 | G | G | T | 0.85 | 8.358237e-01 |  |
| 292 | 8 | G | G | A | 3.38 | 2.133457e-01 |  |
| 292 | 8 | G | G | C | 2.21 | 5.676529e-01 |  |
| 292 | 8 | G | G | G | 93.36 | 0.000000e+00 | \* |
| 292 | 8 | G | G | T | 1.05 | 7.961580e-01 |  |
| 293 | 9 | C | C | A | 4.49 | 9.703049e-02 |  |
| 293 | 9 | C | C | C | 85.04 | 0.000000e+00 | \* |
| 293 | 9 | C | C | G | 7.48 | 1.621381e-02 |  |
| 293 | 9 | C | C | T | 2.99 | 3.608289e-01 |  |
| 294 | 10 | C | C | A | 3.57 | 1.880349e-01 |  |
| 294 | 10 | C | C | C | 92.87 | 0.000000e+00 | \* |
| 294 | 10 | C | C | G | 0.00 | 9.000000e-01 |  |
| 294 | 10 | C | C | T | 3.57 | 2.660384e-01 |  |
| 295 | 11 | A | A | A | 96.36 | 0.000000e+00 | \* |
| 295 | 11 | A | A | C | 1.92 | 6.344572e-01 |  |
| 295 | 11 | A | A | G | 0.00 | 9.000000e-01 |  |
| 295 | 11 | A | A | T | 1.72 | 6.392934e-01 |  |
| 296 | 12 | G | G | A | 3.81 | 1.588690e-01 |  |
| 296 | 12 | G | G | C | 3.49 | 3.101673e-01 |  |
| 296 | 12 | G | G | G | 91.43 | 0.000000e+00 | \* |
| 296 | 12 | G | G | T | 1.27 | 7.475477e-01 |  |
| 297 | 13 | A | A | A | 93.87 | 0.000000e+00 | \* |
| 297 | 13 | A | A | C | 0.98 | 8.115187e-01 |  |
| 297 | 13 | A | A | G | 2.13 | 5.087192e-01 |  |
| 297 | 13 | A | A | T | 3.02 | 3.556886e-01 |  |
| 298 | 14 | C | C | A | 5.05 | 6.284137e-02 |  |
| 298 | 14 | C | C | C | 89.34 | 0.000000e+00 | \* |
| 298 | 14 | C | C | G | 2.32 | 4.639562e-01 |  |
| 298 | 14 | C | C | T | 3.28 | 3.107494e-01 |  |
| 299 | 15 | C | C | A | 7.32 | 9.694719e-03 | \* |
| 299 | 15 | C | C | C | 88.26 | 0.000000e+00 | \* |
| 299 | 15 | C | C | G | 1.68 | 6.214954e-01 |  |
| 299 | 15 | C | C | T | 2.74 | 4.083910e-01 |  |
| 300 | 16 | G | G | A | 2.14 | 4.495937e-01 |  |
| 300 | 16 | G | G | C | 2.90 | 4.196585e-01 |  |
| 300 | 16 | G | G | G | 91.76 | 0.000000e+00 | \* |
| 300 | 16 | G | G | T | 3.21 | 3.228824e-01 |  |
| 301 | 17 | T | T | A | 0.00 | 8.035714e-01 |  |
| 301 | 17 | T | T | C | 3.52 | 3.055755e-01 |  |
| 301 | 17 | T | T | G | 4.14 | 1.641517e-01 |  |
| 301 | 17 | T | T | T | 92.34 | 0.000000e+00 | \* |
| 302 | 18 | G | G | A | 1.62 | 5.733839e-01 |  |
| 302 | 18 | G | G | C | 0.00 | 8.658537e-01 |  |
| 302 | 18 | G | G | G | 95.44 | 0.000000e+00 | \* |
| 302 | 18 | G | G | T | 2.94 | 3.701620e-01 |  |
| 303 | 19 | T | T | A | 0.00 | 8.035714e-01 |  |
| 303 | 19 | T | T | C | 0.65 | 8.466532e-01 |  |
| 303 | 19 | T | T | G | 7.46 | 1.649400e-02 |  |
| 303 | 19 | T | T | T | 91.90 | 0.000000e+00 | \* |
| 304 | 20 | T | T | A | 0.00 | 8.035714e-01 |  |
| 304 | 20 | T | T | C | 0.00 | 8.658537e-01 |  |
| 304 | 20 | T | T | G | 3.29 | 2.757530e-01 |  |
| 304 | 20 | T | T | T | 96.71 | 0.000000e+00 | \* |
| 305 | 21 | T | T | A | 0.00 | 8.035714e-01 |  |
| 305 | 21 | T | T | C | 1.64 | 6.941841e-01 |  |
| 305 | 21 | T | T | G | 2.53 | 4.170585e-01 |  |
| 305 | 21 | T | T | T | 95.83 | 0.000000e+00 | \* |
| 306 | 22 | G | G | A | 1.00 | 7.136024e-01 |  |
| 306 | 22 | G | G | C | 2.88 | 4.233595e-01 |  |
| 306 | 22 | G | G | G | 94.01 | 0.000000e+00 | \* |
| 306 | 22 | G | G | T | 2.11 | 5.477464e-01 |  |
| 307 | 23 | T | T | A | 0.00 | 8.035714e-01 |  |
| 307 | 23 | T | T | C | 1.69 | 6.838200e-01 |  |
| 307 | 23 | T | T | G | 4.50 | 1.301765e-01 |  |
| 307 | 23 | T | T | T | 93.81 | 0.000000e+00 | \* |
| 308 | 24 | G | G | A | 1.91 | 5.042237e-01 |  |
| 308 | 24 | G | G | C | 1.23 | 7.733947e-01 |  |
| 308 | 24 | G | G | G | 91.55 | 0.000000e+00 | \* |
| 308 | 24 | G | G | T | 5.31 | 9.484054e-02 |  |
| 309 | 25 | C | C | A | 2.41 | 3.878028e-01 |  |
| 309 | 25 | C | C | C | 97.59 | 0.000000e+00 | \* |
| 309 | 25 | C | C | G | 0.00 | 9.000000e-01 |  |
| 309 | 25 | C | C | T | 0.00 | 9.148936e-01 |  |
| 310 | 26 | A | A | A | 84.71 | 0.000000e+00 | \* |
| 310 | 26 | A | A | C | 3.22 | 3.580929e-01 |  |
| 310 | 26 | A | A | G | 8.25 | 9.123732e-03 | \* |
| 310 | 26 | A | A | T | 3.82 | 2.307394e-01 |  |
| 311 | 27 | C | C | A | 4.12 | 1.272991e-01 |  |
| 311 | 27 | C | C | C | 93.04 | 0.000000e+00 | \* |
| 311 | 27 | C | C | G | 0.00 | 9.000000e-01 |  |
| 311 | 27 | C | C | T | 2.84 | 3.892748e-01 |  |
| 312 | 28 | T | T | A | 1.39 | 6.288128e-01 |  |
| 312 | 28 | T | T | C | 5.29 | 1.051509e-01 |  |
| 312 | 28 | T | T | G | 1.39 | 6.935153e-01 |  |
| 312 | 28 | T | T | T | 91.93 | 0.000000e+00 | \* |
| 313 | 29 | A | A | A | 93.50 | 0.000000e+00 | \* |
| 313 | 29 | A | A | C | 2.33 | 5.410747e-01 |  |
| 313 | 29 | A | A | G | 1.67 | 6.240683e-01 |  |
| 313 | 29 | A | A | T | 2.50 | 4.590640e-01 |  |
| 314 | 30 | C | C | A | 3.73 | 1.684670e-01 |  |
| 314 | 30 | C | C | C | 90.16 | 0.000000e+00 | \* |
| 314 | 30 | C | C | G | 2.68 | 3.857826e-01 |  |
| 314 | 30 | C | C | T | 3.43 | 2.869350e-01 |  |
| 315 | 31 | A | A | A | 88.09 | 0.000000e+00 | \* |
| 315 | 31 | A | A | C | 3.08 | 3.842963e-01 |  |
| 315 | 31 | A | A | G | 6.57 | 3.142408e-02 |  |
| 315 | 31 | A | A | T | 2.26 | 5.125427e-01 |  |
| 316 | 32 | C | C | A | 2.09 | 4.614601e-01 |  |
| 316 | 32 | C | C | C | 89.09 | 0.000000e+00 | \* |
| 316 | 32 | C | C | G | 4.98 | 9.529420e-02 |  |
| 316 | 32 | C | C | T | 3.85 | 2.269498e-01 |  |
| 317 | 33 | G | G | A | 2.79 | 3.110588e-01 |  |
| 317 | 33 | G | G | C | 5.36 | 9.977936e-02 |  |
| 317 | 33 | G | G | G | 90.13 | 0.000000e+00 | \* |
| 317 | 33 | G | G | T | 1.72 | 6.410873e-01 |  |
| 318 | 34 | G | G | A | 0.00 | 8.035714e-01 |  |
| 318 | 34 | G | G | C | 2.16 | 5.802543e-01 |  |
| 318 | 34 | G | G | G | 96.55 | 0.000000e+00 | \* |
| 318 | 34 | G | G | T | 1.29 | 7.417747e-01 |  |
| 319 | 35 | G | G | A | 1.50 | 6.023793e-01 |  |
| 319 | 35 | G | G | C | 1.84 | 6.519253e-01 |  |
| 319 | 35 | G | G | G | 94.66 | 0.000000e+00 | \* |
| 319 | 35 | G | G | T | 2.00 | 5.720833e-01 |  |
| 320 | 36 | C | C | A | 3.94 | 1.451481e-01 |  |
| 320 | 36 | C | C | C | 87.99 | 0.000000e+00 | \* |
| 320 | 36 | C | C | G | 5.12 | 8.661402e-02 |  |
| 320 | 36 | C | C | T | 2.95 | 3.679975e-01 |  |
| 321 | 37 | A | A | A | 93.52 | 0.000000e+00 | \* |
| 321 | 37 | A | A | C | 3.24 | 3.545055e-01 |  |
| 321 | 37 | A | A | G | 0.00 | 9.000000e-01 |  |
| 321 | 37 | A | A | T | 3.24 | 3.173626e-01 |  |
| 322 | 38 | C | C | A | 3.59 | 1.845060e-01 |  |
| 322 | 38 | C | C | C | 90.76 | 0.000000e+00 | \* |
| 322 | 38 | C | C | G | 2.57 | 4.098147e-01 |  |
| 322 | 38 | C | C | T | 3.08 | 3.446462e-01 |  |
| 323 | 39 | C | C | A | 4.95 | 6.843258e-02 |  |
| 323 | 39 | C | C | C | 89.01 | 0.000000e+00 | \* |
| 323 | 39 | C | C | G | 3.71 | 2.145595e-01 |  |
| 323 | 39 | C | C | T | 2.34 | 4.952715e-01 |  |
| 324 | 40 | C | C | A | 5.12 | 5.985232e-02 |  |
| 324 | 40 | C | C | C | 90.35 | 0.000000e+00 | \* |
| 324 | 40 | C | C | G | 0.00 | 9.000000e-01 |  |
| 324 | 40 | C | C | T | 4.53 | 1.529018e-01 |  |
| 325 | 41 | T | T | A | 2.04 | 4.722857e-01 |  |
| 325 | 41 | T | T | C | 4.81 | 1.427439e-01 |  |
| 325 | 41 | T | T | G | 2.19 | 4.955850e-01 |  |
| 325 | 41 | T | T | T | 90.96 | 0.000000e+00 | \* |
| 326 | 42 | G | G | A | 2.07 | 4.650692e-01 |  |
| 326 | 42 | G | G | C | 2.19 | 5.739390e-01 |  |
| 326 | 42 | G | G | G | 92.64 | 0.000000e+00 | \* |
| 326 | 42 | G | G | T | 3.11 | 3.400154e-01 |  |
| 327 | 43 | A | A | A | 55.29 | 0.000000e+00 | \* |
| 327 | 43 | A | A | C | 1.44 | 7.339595e-01 |  |
| 327 | 43 | A | A | G | 4.17 | 1.614875e-01 |  |
| 327 | 43 | A | A | T | 39.10 | 5.375700e-13 | \* |
| 328 | 44 | C | C | A | 3.78 | 1.617689e-01 |  |
| 328 | 44 | C | C | C | 96.22 | 0.000000e+00 | \* |
| 328 | 44 | C | C | G | 0.00 | 9.000000e-01 |  |
| 328 | 44 | C | C | T | 0.00 | 9.148936e-01 |  |
| 329 | 45 | C | C | A | 3.63 | 1.799905e-01 |  |
| 329 | 45 | C | C | C | 87.29 | 0.000000e+00 | \* |
| 329 | 45 | C | C | G | 5.45 | 6.931893e-02 |  |
| 329 | 45 | C | C | T | 3.63 | 2.568844e-01 |  |
| 330 | 46 | G | G | A | 3.68 | 1.739586e-01 |  |
| 330 | 46 | G | G | C | 4.11 | 2.185478e-01 |  |
| 330 | 46 | G | G | G | 92.21 | 0.000000e+00 | \* |
| 330 | 46 | G | G | T | 0.00 | 9.148936e-01 |  |
| 331 | 47 | A | A | A | 95.70 | 0.000000e+00 | \* |
| 331 | 47 | A | A | C | 0.84 | 8.284679e-01 |  |
| 331 | 47 | A | A | G | 1.47 | 6.739817e-01 |  |
| 331 | 47 | A | A | T | 1.99 | 5.743753e-01 |  |
| 332 | 48 | C | C | A | 8.76 | 2.728707e-03 | \* |
| 332 | 48 | C | C | C | 90.19 | 0.000000e+00 | \* |
| 332 | 48 | C | C | G | 1.05 | 7.742529e-01 |  |
| 332 | 48 | C | C | T | 0.00 | 9.148936e-01 |  |
| 333 | 49 | G | G | A | 0.00 | 8.035714e-01 |  |
| 333 | 49 | G | G | C | 2.66 | 4.691025e-01 |  |
| 333 | 49 | G | G | G | 97.34 | 0.000000e+00 | \* |
| 333 | 49 | G | G | T | 0.00 | 9.148936e-01 |  |
| 334 | 50 | G | G | A | 2.18 | 4.389402e-01 |  |
| 334 | 50 | G | G | C | 1.75 | 6.713935e-01 |  |
| 334 | 50 | G | G | G | 94.47 | 0.000000e+00 | \* |
| 334 | 50 | G | G | T | 1.60 | 6.690826e-01 |  |
| 335 | 51 | C | C | A | 7.04 | 1.231372e-02 |  |
| 335 | 51 | C | C | C | 87.62 | 0.000000e+00 | \* |
| 335 | 51 | C | C | G | 5.34 | 7.451952e-02 |  |
| 335 | 51 | C | C | T | 0.00 | 9.148936e-01 |  |
| 336 | 52 | A | A | A | 93.18 | 0.000000e+00 | \* |
| 336 | 52 | A | A | C | 0.89 | 8.225743e-01 |  |
| 336 | 52 | A | A | G | 1.19 | 7.430078e-01 |  |
| 336 | 52 | A | A | T | 4.75 | 1.343264e-01 |  |
| 337 | 53 | A | A | A | 95.90 | 0.000000e+00 | \* |
| 337 | 53 | A | A | C | 0.19 | 8.652968e-01 |  |
| 337 | 53 | A | A | G | 0.74 | 8.357457e-01 |  |
| 337 | 53 | A | A | T | 3.17 | 3.297835e-01 |  |
| 338 | 54 | G | G | A | 5.89 | 3.209770e-02 |  |
| 338 | 54 | G | G | C | 2.11 | 5.921481e-01 |  |
| 338 | 54 | G | G | G | 89.89 | 0.000000e+00 | \* |
| 338 | 54 | G | G | T | 2.11 | 5.480198e-01 |  |
| 339 | 55 | A | A | A | 91.32 | 0.000000e+00 | \* |
| 339 | 55 | A | A | C | 0.00 | 8.658537e-01 |  |
| 339 | 55 | A | A | G | 4.50 | 1.302785e-01 |  |
| 339 | 55 | A | A | T | 4.18 | 1.881161e-01 |  |
| 340 | 56 | A | A | A | 94.03 | 0.000000e+00 | \* |
| 340 | 56 | A | A | C | 1.27 | 7.665529e-01 |  |
| 340 | 56 | A | A | G | 0.72 | 8.394842e-01 |  |
| 340 | 56 | A | A | T | 3.98 | 2.112903e-01 |  |
| 341 | 57 | G | G | A | 6.27 | 2.364201e-02 |  |
| 341 | 57 | G | G | C | 2.01 | 6.145930e-01 |  |
| 341 | 57 | G | G | G | 87.72 | 0.000000e+00 | \* |
| 341 | 57 | G | G | T | 4.01 | 2.074934e-01 |  |
| 342 | 58 | T | T | A | 0.00 | 8.035714e-01 |  |
| 342 | 58 | T | T | C | 0.00 | 8.658537e-01 |  |
| 342 | 58 | T | T | G | 0.00 | 9.000000e-01 |  |
| 342 | 58 | T | T | T | 100.00 | 0.000000e+00 | \* |
| 343 | 59 | T | T | A | 1.16 | 6.814850e-01 |  |
| 343 | 59 | T | T | C | 2.50 | 5.031993e-01 |  |
| 343 | 59 | T | T | G | 0.00 | 9.000000e-01 |  |
| 343 | 59 | T | T | T | 96.34 | 0.000000e+00 | \* |
| 344 | 60 | C | C | A | 4.35 | 1.076506e-01 |  |
| 344 | 60 | C | C | C | 91.67 | 0.000000e+00 | \* |
| 344 | 60 | C | C | G | 1.45 | 6.789374e-01 |  |
| 344 | 60 | C | C | T | 2.54 | 4.513093e-01 |  |
| 345 | 61 | G | G | A | 4.19 | 1.211587e-01 |  |
| 345 | 61 | G | G | C | 4.43 | 1.803552e-01 |  |
| 345 | 61 | G | G | G | 91.38 | 0.000000e+00 | \* |
| 345 | 61 | G | G | T | 0.00 | 9.148936e-01 |  |
| 346 | 62 | A | A | A | 92.61 | 0.000000e+00 | \* |
| 346 | 62 | A | A | C | 1.99 | 6.182475e-01 |  |
| 346 | 62 | A | A | G | 3.55 | 2.359454e-01 |  |
| 346 | 62 | A | A | T | 1.85 | 6.096589e-01 |  |
| 347 | 63 | C | C | A | 3.99 | 1.393643e-01 |  |
| 347 | 63 | C | C | C | 88.17 | 0.000000e+00 | \* |
| 347 | 63 | C | C | G | 3.38 | 2.611949e-01 |  |
| 347 | 63 | C | C | T | 4.45 | 1.601116e-01 |  |
| 348 | 64 | A | A | A | 88.97 | 0.000000e+00 | \* |
| 348 | 64 | A | A | C | 7.11 | 2.968997e-02 |  |
| 348 | 64 | A | A | G | 1.72 | 6.116636e-01 |  |
| 348 | 64 | A | A | T | 2.21 | 5.246448e-01 |  |
| 349 | 65 | G | G | A | 2.76 | 3.165612e-01 |  |
| 349 | 65 | G | G | C | 3.99 | 2.350505e-01 |  |
| 349 | 65 | G | G | G | 92.02 | 0.000000e+00 | \* |
| 349 | 65 | G | G | T | 1.23 | 7.572953e-01 |  |
| 350 | 66 | C | C | A | 3.00 | 2.727663e-01 |  |
| 350 | 66 | C | C | C | 90.24 | 0.000000e+00 | \* |
| 350 | 66 | C | C | G | 3.75 | 2.089447e-01 |  |
| 350 | 66 | C | C | T | 3.00 | 3.589150e-01 |  |
| 351 | 67 | T | T | A | 1.02 | 7.095140e-01 |  |
| 351 | 67 | T | T | C | 4.22 | 2.049411e-01 |  |
| 351 | 67 | T | T | G | 2.91 | 3.409305e-01 |  |
| 351 | 67 | T | T | T | 91.85 | 0.000000e+00 | \* |
| 352 | 68 | C | C | A | 2.89 | 2.923037e-01 |  |
| 352 | 68 | C | C | C | 90.14 | 0.000000e+00 | \* |
| 352 | 68 | C | C | G | 3.06 | 3.136130e-01 |  |
| 352 | 68 | C | C | T | 3.91 | 2.194691e-01 |  |
| 353 | 69 | C | C | A | 1.79 | 5.319214e-01 |  |
| 353 | 69 | C | C | C | 93.24 | 0.000000e+00 | \* |
| 353 | 69 | C | C | G | 0.69 | 8.451396e-01 |  |
| 353 | 69 | C | C | T | 4.28 | 1.778994e-01 |  |
| 354 | 70 | C | C | A | 4.62 | 8.809720e-02 |  |
| 354 | 70 | C | C | C | 91.15 | 0.000000e+00 | \* |
| 354 | 70 | C | C | G | 1.35 | 7.045619e-01 |  |
| 354 | 70 | C | C | T | 2.88 | 3.808622e-01 |  |
| 355 | 71 | G | G | A | 3.46 | 2.017433e-01 |  |
| 355 | 71 | G | G | C | 4.55 | 1.684033e-01 |  |
| 355 | 71 | G | G | G | 89.61 | 0.000000e+00 | \* |
| 355 | 71 | G | G | T | 2.38 | 4.850667e-01 |  |
| 356 | 72 | C | C | A | 0.00 | 8.035714e-01 |  |
| 356 | 72 | C | C | C | 92.73 | 0.000000e+00 | \* |
| 356 | 72 | C | C | G | 4.44 | 1.351929e-01 |  |
| 356 | 72 | C | C | T | 2.83 | 3.917284e-01 |  |
| 357 | 73 | G | G | A | 9.98 | 9.004264e-04 | \* |
| 357 | 73 | G | G | C | 5.21 | 1.107308e-01 |  |
| 357 | 73 | G | G | G | 84.82 | 0.000000e+00 | \* |
| 357 | 73 | G | G | T | 0.00 | 9.148936e-01 |  |
| 358 | 74 | A | A | A | 93.29 | 0.000000e+00 | \* |
| 358 | 74 | A | A | C | 1.76 | 6.691399e-01 |  |
| 358 | 74 | A | A | G | 3.51 | 2.411762e-01 |  |
| 358 | 74 | A | A | T | 1.44 | 7.083172e-01 |  |
| 359 | 75 | C | C | A | 4.61 | 8.857120e-02 |  |
| 359 | 75 | C | C | C | 88.48 | 0.000000e+00 | \* |
| 359 | 75 | C | C | G | 2.30 | 4.681451e-01 |  |
| 359 | 75 | C | C | T | 4.61 | 1.460979e-01 |  |

## For use in R

If you want to work with the results in R, here is output that you can copy and paste in your terminal to get:

The base information:

```
structure(list(focal.base = c("A", "C", "G", "T"), avg.percsignal = c(91.3327711624546, 
91.4039814118926, 93.1016414073572, 93.1880567480594), avg.areasignal = c(582.625, 
610, 618.631578947368, 663.395348837209), crit.perc.area = c(7.28113898379275, 
8.56687852383342, 8.12750154253697, 8.68597305936969), mu = c(2.64679680764548, 
3.16821213354988, 2.73942451065087, 2.8914900674118), fillibens = c(0.996171955675203, 
0.990977678483888, 0.996298864428764, 0.995847172199892)), .Names = c("focal.base", 
"avg.percsignal", "avg.areasignal", "crit.perc.area", "mu", "fillibens"
), row.names = c(NA, -4L), class = "data.frame")
```

the data.frame that contains information on the guide region:

```
structure(list(A.area = c(543, 649, 12, 1049, 0, 0, 23, 29, 21, 
27, 503, 24, 1057, 37, 48, 14, 0, 16, 0, 0, 0, 9, 0, 14, 13, 
421, 29, 10, 561, 25, 429, 13, 13, 0, 9, 20, 462, 28, 36, 35, 
14, 18, 345, 21, 22, 17, 912, 50, 0, 15, 29, 314, 515, 28, 568, 
520, 25, 0, 6, 24, 17, 652, 26, 363, 18, 16, 7, 17, 13, 24, 16, 
0, 46, 584, 30), C.area = c(17, 26, 21, 10, 616, 40, 0, 19, 398, 
703, 10, 22, 11, 654, 579, 19, 17, 0, 4, 0, 11, 26, 9, 9, 526, 
16, 655, 38, 14, 605, 15, 555, 25, 15, 11, 447, 16, 707, 648, 
618, 33, 19, 9, 534, 529, 19, 8, 515, 9, 12, 361, 3, 1, 10, 0, 
7, 8, 0, 13, 506, 18, 14, 574, 29, 26, 481, 29, 530, 676, 474, 
21, 459, 24, 11, 576), G.area = c(14, 9, 535, 33, 0, 17, 1147, 
801, 35, 0, 0, 576, 24, 17, 11, 601, 20, 941, 46, 21, 17, 848, 
24, 672, 0, 41, 0, 10, 10, 18, 32, 31, 420, 671, 567, 26, 0, 
20, 27, 0, 15, 805, 26, 0, 33, 426, 14, 6, 329, 649, 22, 4, 4, 
427, 28, 4, 350, 0, 0, 8, 371, 25, 22, 7, 600, 20, 20, 18, 5, 
7, 414, 22, 391, 22, 15), T.area = c(0, 14, 0, 25, 11, 689, 10, 
9, 14, 27, 9, 8, 34, 24, 18, 21, 446, 29, 567, 618, 643, 19, 
500, 39, 0, 19, 20, 661, 15, 23, 11, 24, 8, 9, 12, 15, 16, 24, 
17, 31, 624, 27, 244, 0, 22, 0, 19, 0, 0, 11, 0, 16, 17, 10, 
26, 22, 16, 465, 500, 14, 0, 13, 29, 9, 8, 16, 631, 23, 31, 15, 
11, 14, 0, 9, 30), Tot.area = c(574, 698, 568, 1117, 627, 746, 
1180, 858, 468, 757, 522, 630, 1126, 732, 656, 655, 483, 986, 
617, 639, 671, 902, 533, 734, 539, 497, 704, 719, 600, 671, 487, 
623, 466, 695, 599, 508, 494, 779, 728, 684, 686, 869, 624, 555, 
606, 462, 953, 571, 338, 687, 412, 337, 537, 475, 622, 553, 399, 
465, 519, 552, 406, 704, 651, 408, 652, 533, 687, 588, 725, 520, 
462, 495, 461, 626, 651), A.perc = c(94.5993031358885, 92.9799426934097, 
2.11267605633803, 93.9122649955237, 0, 0, 1.94915254237288, 3.37995337995338, 
4.48717948717949, 3.5667107001321, 96.360153256705, 3.80952380952381, 
93.8721136767318, 5.05464480874317, 7.31707317073171, 2.13740458015267, 
0, 1.62271805273834, 0, 0, 0, 0.997782705099778, 0, 1.90735694822888, 
2.41187384044527, 84.7082494969819, 4.11931818181818, 1.39082058414465, 
93.5, 3.725782414307, 88.0903490759754, 2.08667736757624, 2.78969957081545, 
0, 1.5025041736227, 3.93700787401575, 93.5222672064777, 3.59435173299101, 
4.94505494505495, 5.11695906432749, 2.04081632653061, 2.07134637514384, 
55.2884615384615, 3.78378378378378, 3.63036303630363, 3.67965367965368, 
95.697796432319, 8.75656742556918, 0, 2.18340611353712, 7.03883495145631, 
93.1750741839763, 95.903165735568, 5.89473684210526, 91.3183279742765, 
94.0325497287523, 6.265664160401, 0, 1.15606936416185, 4.34782608695652, 
4.1871921182266, 92.6136363636364, 3.99385560675883, 88.9705882352941, 
2.76073619631902, 3.00187617260788, 1.01892285298399, 2.89115646258503, 
1.79310344827586, 4.61538461538461, 3.46320346320346, 0, 9.97830802603037, 
93.2907348242811, 4.60829493087558), C.perc = c(2.9616724738676, 
3.72492836676218, 3.69718309859155, 0.895255147717099, 98.2456140350877, 
5.36193029490617, 0, 2.21445221445221, 85.042735042735, 92.8665785997358, 
1.91570881226054, 3.49206349206349, 0.976909413854352, 89.344262295082, 
88.2621951219512, 2.90076335877863, 3.51966873706004, 0, 0.648298217179903, 
0, 1.63934426229508, 2.88248337028825, 1.68855534709193, 1.22615803814714, 
97.5881261595547, 3.21931589537223, 93.0397727272727, 5.28511821974965, 
2.33333333333333, 90.1639344262295, 3.08008213552361, 89.0850722311396, 
5.36480686695279, 2.15827338129496, 1.83639398998331, 87.992125984252, 
3.23886639676113, 90.7573812580231, 89.010989010989, 90.3508771929825, 
4.81049562682216, 2.18642117376295, 1.44230769230769, 96.2162162162162, 
87.2937293729373, 4.11255411255411, 0.839454354669465, 90.1926444833625, 
2.66272189349112, 1.74672489082969, 87.621359223301, 0.890207715133531, 
0.186219739292365, 2.10526315789474, 0, 1.26582278481013, 2.00501253132832, 
0, 2.50481695568401, 91.6666666666667, 4.43349753694581, 1.98863636363636, 
88.1720430107527, 7.1078431372549, 3.98773006134969, 90.2439024390244, 
4.22125181950509, 90.1360544217687, 93.2413793103448, 91.1538461538462, 
4.54545454545455, 92.7272727272727, 5.2060737527115, 1.75718849840256, 
88.4792626728111), G.perc = c(2.4390243902439, 1.28939828080229, 
94.1901408450704, 2.95434198746643, 0, 2.27882037533512, 97.2033898305085, 
93.3566433566434, 7.47863247863248, 0, 0, 91.4285714285714, 2.13143872113677, 
2.3224043715847, 1.67682926829268, 91.7557251908397, 4.1407867494824, 
95.4361054766734, 7.45542949756888, 3.28638497652582, 2.53353204172876, 
94.0133037694013, 4.50281425891182, 91.5531335149864, 0, 8.24949698189135, 
0, 1.39082058414465, 1.66666666666667, 2.68256333830104, 6.57084188911704, 
4.97592295345104, 90.1287553648069, 96.5467625899281, 94.6577629382304, 
5.11811023622047, 0, 2.56739409499358, 3.70879120879121, 0, 2.1865889212828, 
92.6352128883774, 4.16666666666667, 0, 5.44554455445545, 92.2077922077922, 
1.46904512067156, 1.0507880910683, 97.3372781065089, 94.4687045123726, 
5.33980582524272, 1.18694362017804, 0.74487895716946, 89.8947368421053, 
4.5016077170418, 0.723327305605787, 87.719298245614, 0, 0, 1.44927536231884, 
91.3793103448276, 3.55113636363636, 3.37941628264209, 1.7156862745098, 
92.0245398773006, 3.75234521575985, 2.91120815138282, 3.06122448979592, 
0.689655172413793, 1.34615384615385, 89.6103896103896, 4.44444444444444, 
84.8156182212581, 3.51437699680511, 2.30414746543779), T.perc = c(0, 
2.00573065902579, 0, 2.23813786929275, 1.75438596491228, 92.3592493297587, 
0.847457627118644, 1.04895104895105, 2.99145299145299, 3.5667107001321, 
1.72413793103448, 1.26984126984127, 3.01953818827709, 3.27868852459016, 
2.74390243902439, 3.20610687022901, 92.3395445134576, 2.94117647058824, 
91.8962722852512, 96.7136150234742, 95.8271236959762, 2.10643015521064, 
93.8086303939963, 5.3133514986376, 0, 3.82293762575453, 2.84090909090909, 
91.9332406119611, 2.5, 3.42771982116244, 2.25872689938398, 3.85232744783307, 
1.71673819742489, 1.29496402877698, 2.00333889816361, 2.95275590551181, 
3.23886639676113, 3.0808729139923, 2.33516483516484, 4.53216374269006, 
90.9620991253644, 3.10701956271577, 39.1025641025641, 0, 3.63036303630363, 
0, 1.99370409233998, 0, 0, 1.60116448326055, 0, 4.74777448071217, 
3.1657355679702, 2.10526315789474, 4.18006430868167, 3.97830018083183, 
4.01002506265664, 100, 96.3391136801541, 2.53623188405797, 0, 
1.84659090909091, 4.45468509984639, 2.20588235294118, 1.22699386503067, 
3.00187617260788, 91.8486171761281, 3.91156462585034, 4.27586206896552, 
2.88461538461538, 2.38095238095238, 2.82828282828283, 0, 1.43769968051118, 
4.60829493087558), base.call = c("A", "A", "G", "A", "C", "T", 
"G", "G", "C", "C", "A", "G", "A", "C", "C", "G", "T", "G", "T", 
"T", "T", "G", "T", "G", "C", "A", "C", "T", "A", "C", "A", "C", 
"G", "G", "G", "C", "A", "C", "C", "C", "T", "G", "A", "C", "C", 
"G", "A", "C", "G", "G", "C", "A", "A", "G", "A", "A", "G", "T", 
"T", "C", "G", "A", "C", "A", "G", "C", "T", "C", "C", "C", "G", 
"C", "G", "A", "C"), index = 285:359, guide.seq = c("A", "A", 
"G", "A", "C", "T", "G", "G", "C", "C", "A", "G", "A", "C", "C", 
"G", "T", "G", "T", "T", "T", "G", "T", "G", "C", "A", "C", "T", 
"A", "C", "A", "C", "G", "G", "G", "C", "A", "C", "C", "C", "T", 
"G", "A", "C", "C", "G", "A", "C", "G", "G", "C", "A", "A", "G", 
"A", "A", "G", "T", "T", "C", "G", "A", "C", "A", "G", "C", "T", 
"C", "C", "C", "G", "C", "G", "A", "C"), T.pval = c(0.91489361696974, 
0.571514772664477, 0.91489361696974, 0.51724271493693, 0.631962235741588, 
0, 0.835823681425898, 0.79615795683146, 0.360828888585375, 0.266038449507386, 
0.639293423461984, 0.747547714603142, 0.355688601981048, 0.310749389117625, 
0.408391034846491, 0.322882357133527, 0, 0.370162002411189, 0, 
0, 0, 0.547746410655188, 0, 0.0948405412358887, 0.91489361696974, 
0.230739400026691, 0.389274811765538, 0, 0.459063989765005, 0.286935009480282, 
0.512542679754079, 0.226949769501801, 0.641087305086865, 0.741774746399826, 
0.572083312072232, 0.367997547558937, 0.31736258896702, 0.344646166603763, 
0.49527145588659, 0.152901754648823, 0, 0.340015433155824, 5.37569988523501e-13, 
0.91489361696974, 0.256884395116388, 0.91489361696974, 0.57437528825297, 
0.91489361696974, 0.91489361696974, 0.669082560840233, 0.91489361696974, 
0.134326379081105, 0.329783480318601, 0.548019823612849, 0.188116094076566, 
0.211290327827656, 0.207493362952848, 0, 0, 0.451309326979969, 
0.91489361696974, 0.609658905628702, 0.160111574852353, 0.5246448473181, 
0.757295265197406, 0.358915044237394, 0, 0.219469103048174, 0.177899421237608, 
0.380862158566957, 0.485066700084039, 0.391728416430248, 0.91489361696974, 
0.708317247769373, 0.146097878002127), C.pval = c(0.407451920477893, 
0.272946810898587, 0.277201182597835, 0.821964468348495, 0, 0.0999689294631709, 
0.865853658536582, 0.567652871634464, 0, 0, 0.634457170849209, 
0.310167317688448, 0.811518742474783, 0, 0, 0.419658484474887, 
0.305575522910921, 0.865853658536582, 0.846653184396677, 0.865853658536582, 
0.694184126743552, 0.423359460771997, 0.683820004262086, 0.773394687980866, 
0, 0.358092883863231, 0, 0.105150868282137, 0.541074693629431, 
0, 0.384296321828053, 0, 0.0997793627612769, 0.580254335884437, 
0.651925314296768, 0, 0.35450550449126, 0, 0, 0, 0.142743886144024, 
0.573939013841801, 0.733959503340265, 0, 0, 0.218547822926783, 
0.82846785240319, 0, 0.46910250873306, 0.671393458964762, 0, 
0.822574275716633, 0.86529677327461, 0.592148132543517, 0.865853658536582, 
0.766552908803632, 0.614592989392588, 0.865853658536582, 0.503199295808264, 
0, 0.180355226148845, 0.618247457200701, 0, 0.0296899744908928, 
0.235050527627386, 0, 0.204941130623228, 0, 0, 0, 0.168403344084067, 
0, 0.110730847034632, 0.669139932568319, 0), G.pval = c(0.437690071337683, 
0.718453378226793, 0, 0.332903798590501, 0.899999999997222, 0.473989474789258, 
0, 0, 0.0162138089756485, 0.899999999997222, 0.899999999997222, 
0, 0.508719181435001, 0.46395619533722, 0.621495401342106, 0, 
0.164151740484392, 0, 0.0164939985730548, 0.275752959735349, 
0.417058461628867, 0, 0.130176536981612, 0, 0.899999999997222, 
0.00912373182191228, 0.899999999997222, 0.693515294736557, 0.624068283597434, 
0.385782604241028, 0.0314240819189784, 0.0952941973014623, 0, 
0, 0, 0.0866140198882367, 0.899999999997222, 0.409814697236787, 
0.214559541647556, 0.899999999997222, 0.495585035507688, 0, 0.161487467967439, 
0.899999999997222, 0.0693189291023423, 0, 0.673981688966288, 
0.774252896995304, 0, 0, 0.0745195172349705, 0.743007826666722, 
0.835745679480422, 0, 0.130278525412168, 0.839484240955754, 0, 
0.899999999997222, 0.899999999997222, 0.678937397996469, 0, 0.235945438123472, 
0.261194923364574, 0.611663560116579, 0, 0.208944738457354, 0.340930483909975, 
0.313612968987341, 0.845139630111036, 0.704561933076586, 0, 0.135192903379127, 
0, 0.241176243711424, 0.468145148855879), A.pval = c(0, 0, 0.455363508354664, 
0, 0.803571428571429, 0.803571428571429, 0.494161516404564, 0.213345712386141, 
0.0970304923765468, 0.188034879180716, 0, 0.158869011944837, 
0, 0.0628413690472012, 0.0096947191941068, 0.449593740902922, 
0.803571428571429, 0.57338393262668, 0.803571428571429, 0.803571428571429, 
0.803571428571429, 0.713602389364718, 0.803571428571429, 0.504223689144445, 
0.387802842420395, 0, 0.12729906908895, 0.628812843407623, 0, 
0.168467018744853, 0, 0.461460125057763, 0.311058799423964, 0.803571428571429, 
0.602379312906387, 0.14514811960126, 0, 0.184505989451375, 0.0684325785579664, 
0.059852321443256, 0.472285692587135, 0.465069210046898, 0, 0.161768862590936, 
0.179990502268123, 0.17395858484808, 0, 0.00272870652454671, 
0.803571428571429, 0.438940243179652, 0.012313717308214, 0, 0, 
0.0320976957469594, 0, 0, 0.0236420065483929, 0.803571428571429, 
0.681485030992085, 0.107650644451327, 0.121158674679587, 0, 0.139364333469311, 
0, 0.316561166327115, 0.272766264226478, 0.709514009892177, 0.292303742731426, 
0.531921402817707, 0.0880971971574219, 0.201743345087066, 0.803571428571429, 
0.000900426447679514, 0, 0.0885712025395583), guide.position = 1:75), .Names = c("A.area", 
"C.area", "G.area", "T.area", "Tot.area", "A.perc", "C.perc", 
"G.perc", "T.perc", "base.call", "index", "guide.seq", "T.pval", 
"C.pval", "G.pval", "A.pval", "guide.position"), row.names = 285:359, class = "data.frame")
```

*Report generated using EditR v1.0.8*
