# Supplementary material for: Mechanistic and genetic basis of single-strand templated repair at Cas12a-induced DNA breaks in Chlamydomonas reinhardtii
Source: Nat Commun. 2021 Nov 19;12:6751. doi: 10.1038/s41467-021-27004-1 (PMC8604939; doi:10.1038/s41467-021-27004-1)
Supplement: Supplementary file 22 — Source Data [file 41467_2021_27004_MOESM22_ESM.zip › Source Data/EditR analysis/EditR outputs/Sense/rep2_ssODN_sense_0_16_32.html]

EditR v1.0.8 report


# EditR v1.0.8 report

- Data QA
  - Filtering data
  - Percent noise peak area
  - Base information
- Predicted editing
  - Editing bar plot
  - Editing table plot
  - Table of editing results
- For use in R

## Data QA

### Filtering data

What the data looked like prefiltering:

and the post filtering signal / noise plot:

### Percent noise peak area

### Base information

Here’s information about the signal of each base, the critical percent value where any higher value would be called as significant, and Filliben’s correlation for how well the noise was modelled by the zero adjusted gamma distribution.

| Base | Average percent signal | Average peak area | Critical percent value | model mu | Fillibens correlation |
| --- | --- | --- | --- | --- | --- |
| A | 92.33074 | 535.2500 | 7.950071 | 2.850227 | 0.9938844 |
| C | 91.87822 | 555.0870 | 6.173849 | 2.250182 | 0.9933169 |
| G | 93.51184 | 580.2069 | 6.904968 | 2.443375 | 0.9961686 |
| T | 93.87348 | 605.4762 | 7.321798 | 2.480713 | 0.9973281 |

## Predicted editing

### Editing bar plot

### Editing table plot

### Table of editing results


Here’s the entire guide region

| Sanger position | Guide position | Guide sequence | Sanger base call | Focal base | Focal base peak area | p value |  |
| --- | --- | --- | --- | --- | --- | --- | --- |
| 283 | 1 | A | A | A | 95.35 | 0.000000e+00 | \* |
| 283 | 1 | A | A | C | 1.01 | 7.881024e-01 |  |
| 283 | 1 | A | A | G | 1.82 | 5.371599e-01 |  |
| 283 | 1 | A | A | T | 1.82 | 5.367785e-01 |  |
| 284 | 2 | A | A | A | 94.18 | 0.000000e+00 | \* |
| 284 | 2 | A | A | C | 2.16 | 4.179773e-01 |  |
| 284 | 2 | A | A | G | 1.50 | 6.293379e-01 |  |
| 284 | 2 | A | A | T | 2.16 | 4.470973e-01 |  |
| 285 | 3 | G | G | A | 2.79 | 3.641338e-01 |  |
| 285 | 3 | G | G | C | 2.99 | 2.206533e-01 |  |
| 285 | 3 | G | G | G | 94.21 | 0.000000e+00 | \* |
| 285 | 3 | G | G | T | 0.00 | 8.947368e-01 |  |
| 286 | 4 | A | A | A | 93.53 | 0.000000e+00 | \* |
| 286 | 4 | A | A | C | 0.90 | 8.198877e-01 |  |
| 286 | 4 | A | A | G | 2.99 | 2.602653e-01 |  |
| 286 | 4 | A | A | T | 2.59 | 3.494470e-01 |  |
| 287 | 5 | C | C | A | 0.00 | 8.392857e-01 |  |
| 287 | 5 | C | C | C | 98.15 | 0.000000e+00 | \* |
| 287 | 5 | C | C | G | 0.00 | 8.876404e-01 |  |
| 287 | 5 | C | C | T | 1.85 | 5.277036e-01 |  |
| 288 | 6 | T | T | A | 0.00 | 8.392857e-01 |  |
| 288 | 6 | T | T | C | 3.45 | 1.485964e-01 |  |
| 288 | 6 | T | T | G | 2.35 | 3.945363e-01 |  |
| 288 | 6 | T | T | T | 94.19 | 0.000000e+00 | \* |
| 289 | 7 | G | G | A | 3.88 | 1.922472e-01 |  |
| 289 | 7 | G | G | C | 2.17 | 4.171038e-01 |  |
| 289 | 7 | G | G | G | 93.41 | 0.000000e+00 | \* |
| 289 | 7 | G | G | T | 0.54 | 8.552318e-01 |  |
| 290 | 8 | G | G | A | 4.55 | 1.240920e-01 |  |
| 290 | 8 | G | G | C | 1.43 | 6.540889e-01 |  |
| 290 | 8 | G | G | G | 93.76 | 0.000000e+00 | \* |
| 290 | 8 | G | G | T | 0.26 | 8.871629e-01 |  |
| 291 | 9 | C | C | A | 6.01 | 4.424290e-02 |  |
| 291 | 9 | C | C | C | 88.46 | 0.000000e+00 | \* |
| 291 | 9 | C | C | G | 3.85 | 1.377799e-01 |  |
| 291 | 9 | C | C | T | 1.68 | 5.738037e-01 |  |
| 292 | 10 | C | C | A | 3.87 | 1.936863e-01 |  |
| 292 | 10 | C | C | C | 91.96 | 0.000000e+00 | \* |
| 292 | 10 | C | C | G | 2.38 | 3.881848e-01 |  |
| 292 | 10 | C | C | T | 1.79 | 5.455815e-01 |  |
| 293 | 11 | A | A | A | 93.02 | 0.000000e+00 | \* |
| 293 | 11 | A | A | C | 1.48 | 6.374432e-01 |  |
| 293 | 11 | A | A | G | 2.96 | 2.648802e-01 |  |
| 293 | 11 | A | A | T | 2.54 | 3.601576e-01 |  |
| 294 | 12 | G | G | A | 3.38 | 2.607905e-01 |  |
| 294 | 12 | G | G | C | 1.50 | 6.294100e-01 |  |
| 294 | 12 | G | G | G | 95.11 | 0.000000e+00 | \* |
| 294 | 12 | G | G | T | 0.00 | 8.947368e-01 |  |
| 295 | 13 | A | A | A | 94.62 | 0.000000e+00 | \* |
| 295 | 13 | A | A | C | 0.91 | 8.152946e-01 |  |
| 295 | 13 | A | A | G | 1.42 | 6.516580e-01 |  |
| 295 | 13 | A | A | T | 3.04 | 2.624540e-01 |  |
| 296 | 14 | C | C | A | 0.00 | 8.392857e-01 |  |
| 296 | 14 | C | C | C | 95.89 | 0.000000e+00 | \* |
| 296 | 14 | C | C | G | 1.81 | 5.397028e-01 |  |
| 296 | 14 | C | C | T | 2.30 | 4.132577e-01 |  |
| 297 | 15 | C | C | A | 3.56 | 2.338761e-01 |  |
| 297 | 15 | C | C | C | 94.00 | 0.000000e+00 | \* |
| 297 | 15 | C | C | G | 1.88 | 5.208122e-01 |  |
| 297 | 15 | C | C | T | 0.56 | 8.518312e-01 |  |
| 298 | 16 | G | G | A | 3.38 | 2.615777e-01 |  |
| 298 | 16 | G | G | C | 2.36 | 3.617571e-01 |  |
| 298 | 16 | G | G | G | 92.57 | 0.000000e+00 | \* |
| 298 | 16 | G | G | T | 1.69 | 5.720126e-01 |  |
| 299 | 17 | T | T | A | 0.00 | 8.392857e-01 |  |
| 299 | 17 | T | T | C | 2.08 | 4.429943e-01 |  |
| 299 | 17 | T | T | G | 3.23 | 2.181900e-01 |  |
| 299 | 17 | T | T | T | 94.69 | 0.000000e+00 | \* |
| 300 | 18 | G | G | A | 2.88 | 3.479160e-01 |  |
| 300 | 18 | G | G | C | 0.00 | 9.268293e-01 |  |
| 300 | 18 | G | G | G | 94.67 | 0.000000e+00 | \* |
| 300 | 18 | G | G | T | 2.45 | 3.788512e-01 |  |
| 301 | 19 | T | T | A | 0.00 | 8.392857e-01 |  |
| 301 | 19 | T | T | C | 0.18 | 9.251976e-01 |  |
| 301 | 19 | T | T | G | 5.47 | 3.593211e-02 |  |
| 301 | 19 | T | T | T | 94.34 | 0.000000e+00 | \* |
| 302 | 20 | T | T | A | 0.33 | 8.335168e-01 |  |
| 302 | 20 | T | T | C | 0.17 | 9.256042e-01 |  |
| 302 | 20 | T | T | G | 2.31 | 4.045521e-01 |  |
| 302 | 20 | T | T | T | 97.19 | 0.000000e+00 | \* |
| 303 | 21 | T | T | A | 0.00 | 8.392857e-01 |  |
| 303 | 21 | T | T | C | 2.18 | 4.137722e-01 |  |
| 303 | 21 | T | T | G | 2.68 | 3.202897e-01 |  |
| 303 | 21 | T | T | T | 95.14 | 0.000000e+00 | \* |
| 304 | 22 | G | G | A | 1.46 | 6.618774e-01 |  |
| 304 | 22 | G | G | C | 2.07 | 4.446050e-01 |  |
| 304 | 22 | G | G | G | 95.00 | 0.000000e+00 | \* |
| 304 | 22 | G | G | T | 1.46 | 6.347383e-01 |  |
| 305 | 23 | T | T | A | 0.20 | 8.376912e-01 |  |
| 305 | 23 | T | T | C | 1.02 | 7.863137e-01 |  |
| 305 | 23 | T | T | G | 3.86 | 1.361192e-01 |  |
| 305 | 23 | T | T | T | 94.92 | 0.000000e+00 | \* |
| 306 | 24 | G | G | A | 2.26 | 4.767704e-01 |  |
| 306 | 24 | G | G | C | 1.21 | 7.278657e-01 |  |
| 306 | 24 | G | G | G | 93.67 | 0.000000e+00 | \* |
| 306 | 24 | G | G | T | 2.87 | 2.939937e-01 |  |
| 307 | 25 | C | C | A | 1.46 | 6.626766e-01 |  |
| 307 | 25 | C | C | C | 89.23 | 0.000000e+00 | \* |
| 307 | 25 | C | C | G | 4.20 | 1.045100e-01 |  |
| 307 | 25 | C | C | T | 5.11 | 5.951190e-02 |  |
| 308 | 26 | A | A | A | 92.18 | 0.000000e+00 | \* |
| 308 | 26 | A | A | C | 3.55 | 1.358199e-01 |  |
| 308 | 26 | A | A | G | 0.47 | 8.646339e-01 |  |
| 308 | 26 | A | A | T | 3.79 | 1.578808e-01 |  |
| 309 | 27 | C | C | A | 2.16 | 4.993777e-01 |  |
| 309 | 27 | C | C | C | 95.67 | 0.000000e+00 | \* |
| 309 | 27 | C | C | G | 0.33 | 8.780495e-01 |  |
| 309 | 27 | C | C | T | 1.83 | 5.335104e-01 |  |
| 310 | 28 | T | T | A | 2.04 | 5.276304e-01 |  |
| 310 | 28 | T | T | C | 4.24 | 7.193207e-02 |  |
| 310 | 28 | T | T | G | 1.10 | 7.402790e-01 |  |
| 310 | 28 | T | T | T | 92.62 | 0.000000e+00 | \* |
| 311 | 29 | A | A | A | 92.99 | 0.000000e+00 | \* |
| 311 | 29 | A | A | C | 1.80 | 5.310682e-01 |  |
| 311 | 29 | A | A | G | 1.26 | 6.971014e-01 |  |
| 311 | 29 | A | A | T | 3.96 | 1.403929e-01 |  |
| 312 | 30 | C | C | A | 2.74 | 3.740009e-01 |  |
| 312 | 30 | C | C | C | 93.48 | 0.000000e+00 | \* |
| 312 | 30 | C | C | G | 1.72 | 5.665170e-01 |  |
| 312 | 30 | C | C | T | 2.06 | 4.734917e-01 |  |
| 313 | 31 | A | A | A | 89.30 | 0.000000e+00 | \* |
| 313 | 31 | A | A | C | 3.02 | 2.153385e-01 |  |
| 313 | 31 | A | A | G | 5.81 | 2.670370e-02 |  |
| 313 | 31 | A | A | T | 1.86 | 5.253914e-01 |  |
| 314 | 32 | C | C | A | 2.05 | 5.258622e-01 |  |
| 314 | 32 | C | C | C | 90.88 | 0.000000e+00 | \* |
| 314 | 32 | C | C | G | 2.79 | 2.969163e-01 |  |
| 314 | 32 | C | C | T | 4.28 | 1.108542e-01 |  |
| 315 | 33 | G | G | A | 1.92 | 5.562386e-01 |  |
| 315 | 33 | G | G | C | 4.56 | 5.284771e-02 |  |
| 315 | 33 | G | G | G | 92.09 | 0.000000e+00 | \* |
| 315 | 33 | G | G | T | 1.44 | 6.415805e-01 |  |
| 316 | 34 | G | G | A | 0.31 | 8.343822e-01 |  |
| 316 | 34 | G | G | C | 0.00 | 9.268293e-01 |  |
| 316 | 34 | G | G | G | 99.38 | 0.000000e+00 | \* |
| 316 | 34 | G | G | T | 0.31 | 8.833047e-01 |  |
| 317 | 35 | G | G | A | 1.72 | 6.033976e-01 |  |
| 317 | 35 | G | G | C | 2.10 | 4.367889e-01 |  |
| 317 | 35 | G | G | G | 95.42 | 0.000000e+00 | \* |
| 317 | 35 | G | G | T | 0.76 | 8.142094e-01 |  |
| 318 | 36 | C | C | A | 2.16 | 4.990474e-01 |  |
| 318 | 36 | C | C | C | 91.56 | 0.000000e+00 | \* |
| 318 | 36 | C | C | G | 4.11 | 1.117910e-01 |  |
| 318 | 36 | C | C | T | 2.16 | 4.467400e-01 |  |
| 319 | 37 | A | A | A | 90.68 | 0.000000e+00 | \* |
| 319 | 37 | A | A | C | 3.18 | 1.889362e-01 |  |
| 319 | 37 | A | A | G | 2.75 | 3.048330e-01 |  |
| 319 | 37 | A | A | T | 3.39 | 2.084222e-01 |  |
| 320 | 38 | C | C | A | 3.30 | 2.734219e-01 |  |
| 320 | 38 | C | C | C | 93.54 | 0.000000e+00 | \* |
| 320 | 38 | C | C | G | 1.20 | 7.129868e-01 |  |
| 320 | 38 | C | C | T | 1.95 | 5.010890e-01 |  |
| 321 | 39 | C | C | A | 3.31 | 2.728156e-01 |  |
| 321 | 39 | C | C | C | 90.39 | 0.000000e+00 | \* |
| 321 | 39 | C | C | G | 3.78 | 1.450567e-01 |  |
| 321 | 39 | C | C | T | 2.52 | 3.639139e-01 |  |
| 322 | 40 | C | C | A | 4.01 | 1.774703e-01 |  |
| 322 | 40 | C | C | C | 92.32 | 0.000000e+00 | \* |
| 322 | 40 | C | C | G | 0.17 | 8.860483e-01 |  |
| 322 | 40 | C | C | T | 3.51 | 1.925757e-01 |  |
| 323 | 41 | T | T | A | 0.83 | 7.851020e-01 |  |
| 323 | 41 | T | T | C | 5.61 | 1.813587e-02 |  |
| 323 | 41 | T | T | G | 1.65 | 5.852490e-01 |  |
| 323 | 41 | T | T | T | 91.91 | 0.000000e+00 | \* |
| 324 | 42 | G | G | A | 2.32 | 4.639518e-01 |  |
| 324 | 42 | G | G | C | 1.80 | 5.292528e-01 |  |
| 324 | 42 | G | G | G | 94.46 | 0.000000e+00 | \* |
| 324 | 42 | G | G | T | 1.42 | 6.475140e-01 |  |
| 325 | 43 | A | A | A | 75.16 | 0.000000e+00 | \* |
| 325 | 43 | A | A | C | 2.03 | 4.573316e-01 |  |
| 325 | 43 | A | A | G | 3.44 | 1.878721e-01 |  |
| 325 | 43 | A | A | T | 19.38 | 1.839550e-07 | \* |
| 326 | 44 | C | C | A | 4.51 | 1.272555e-01 |  |
| 326 | 44 | C | C | C | 90.62 | 0.000000e+00 | \* |
| 326 | 44 | C | C | G | 2.78 | 3.000432e-01 |  |
| 326 | 44 | C | C | T | 2.08 | 4.671146e-01 |  |
| 327 | 45 | C | C | A | 2.10 | 5.140763e-01 |  |
| 327 | 45 | C | C | C | 90.65 | 0.000000e+00 | \* |
| 327 | 45 | C | C | G | 3.24 | 2.164577e-01 |  |
| 327 | 45 | C | C | T | 4.01 | 1.353755e-01 |  |
| 328 | 46 | G | G | A | 3.08 | 3.103486e-01 |  |
| 328 | 46 | G | G | C | 2.64 | 2.929029e-01 |  |
| 328 | 46 | G | G | G | 94.27 | 0.000000e+00 | \* |
| 328 | 46 | G | G | T | 0.00 | 8.947368e-01 |  |
| 329 | 47 | A | A | A | 93.12 | 0.000000e+00 | \* |
| 329 | 47 | A | A | C | 0.83 | 8.365378e-01 |  |
| 329 | 47 | A | A | G | 2.37 | 3.902347e-01 |  |
| 329 | 47 | A | A | T | 3.68 | 1.710341e-01 |  |
| 330 | 48 | C | C | A | 6.27 | 3.637187e-02 |  |
| 330 | 48 | C | C | C | 89.92 | 0.000000e+00 | \* |
| 330 | 48 | C | C | G | 0.76 | 8.189966e-01 |  |
| 330 | 48 | C | C | T | 3.04 | 2.625855e-01 |  |
| 331 | 49 | G | G | A | 0.00 | 8.392857e-01 |  |
| 331 | 49 | G | G | C | 4.26 | 7.078916e-02 |  |
| 331 | 49 | G | G | G | 95.74 | 0.000000e+00 | \* |
| 331 | 49 | G | G | T | 0.00 | 8.947368e-01 |  |
| 332 | 50 | G | G | A | 2.01 | 5.357052e-01 |  |
| 332 | 50 | G | G | C | 1.85 | 5.137695e-01 |  |
| 332 | 50 | G | G | G | 96.14 | 0.000000e+00 | \* |
| 332 | 50 | G | G | T | 0.00 | 8.947368e-01 |  |
| 333 | 51 | C | C | A | 2.92 | 3.404957e-01 |  |
| 333 | 51 | C | C | C | 89.12 | 0.000000e+00 | \* |
| 333 | 51 | C | C | G | 6.10 | 2.070778e-02 |  |
| 333 | 51 | C | C | T | 1.86 | 5.263845e-01 |  |
| 334 | 52 | A | A | A | 92.07 | 0.000000e+00 | \* |
| 334 | 52 | A | A | C | 1.03 | 7.809784e-01 |  |
| 334 | 52 | A | A | G | 1.72 | 5.639722e-01 |  |
| 334 | 52 | A | A | T | 5.17 | 5.669064e-02 |  |
| 335 | 53 | A | A | A | 94.83 | 0.000000e+00 | \* |
| 335 | 53 | A | A | C | 0.99 | 7.927322e-01 |  |
| 335 | 53 | A | A | G | 0.80 | 8.119689e-01 |  |
| 335 | 53 | A | A | T | 3.38 | 2.098535e-01 |  |
| 336 | 54 | G | G | A | 4.05 | 1.728715e-01 |  |
| 336 | 54 | G | G | C | 1.90 | 4.968173e-01 |  |
| 336 | 54 | G | G | G | 90.24 | 0.000000e+00 | \* |
| 336 | 54 | G | G | T | 3.81 | 1.558828e-01 |  |
| 337 | 55 | A | A | A | 92.76 | 0.000000e+00 | \* |
| 337 | 55 | A | A | C | 0.35 | 9.163655e-01 |  |
| 337 | 55 | A | A | G | 1.77 | 5.517734e-01 |  |
| 337 | 55 | A | A | T | 5.12 | 5.886465e-02 |  |
| 338 | 56 | A | A | A | 91.78 | 0.000000e+00 | \* |
| 338 | 56 | A | A | C | 1.96 | 4.803388e-01 |  |
| 338 | 56 | A | A | G | 0.78 | 8.145179e-01 |  |
| 338 | 56 | A | A | T | 5.48 | 4.463331e-02 |  |
| 339 | 57 | G | G | A | 3.06 | 3.153269e-01 |  |
| 339 | 57 | G | G | C | 4.44 | 5.895940e-02 |  |
| 339 | 57 | G | G | G | 89.44 | 0.000000e+00 | \* |
| 339 | 57 | G | G | T | 3.06 | 2.602520e-01 |  |
| 340 | 58 | T | T | A | 2.27 | 4.755085e-01 |  |
| 340 | 58 | T | T | C | 2.47 | 3.334746e-01 |  |
| 340 | 58 | T | T | G | 1.03 | 7.576893e-01 |  |
| 340 | 58 | T | T | T | 94.23 | 0.000000e+00 | \* |
| 341 | 59 | T | T | A | 21.29 | 9.241045e-08 | \* |
| 341 | 59 | T | T | C | 2.61 | 3.004657e-01 |  |
| 341 | 59 | T | T | G | 3.01 | 2.554036e-01 |  |
| 341 | 59 | T | T | T | 73.09 | 0.000000e+00 | \* |
| 342 | 60 | C | C | A | 3.07 | 3.132101e-01 |  |
| 342 | 60 | C | C | C | 94.27 | 0.000000e+00 | \* |
| 342 | 60 | C | C | G | 0.82 | 8.072154e-01 |  |
| 342 | 60 | C | C | T | 1.84 | 5.307593e-01 |  |
| 343 | 61 | G | G | A | 4.07 | 1.709453e-01 |  |
| 343 | 61 | G | G | C | 4.61 | 5.027738e-02 |  |
| 343 | 61 | G | G | G | 89.43 | 0.000000e+00 | \* |
| 343 | 61 | G | G | T | 1.90 | 5.156235e-01 |  |
| 344 | 62 | A | A | A | 91.99 | 0.000000e+00 | \* |
| 344 | 62 | A | A | C | 1.14 | 7.475389e-01 |  |
| 344 | 62 | A | A | G | 2.29 | 4.107900e-01 |  |
| 344 | 62 | A | A | T | 4.58 | 8.910747e-02 |  |
| 345 | 63 | C | C | A | 3.20 | 2.896905e-01 |  |
| 345 | 63 | C | C | C | 90.56 | 0.000000e+00 | \* |
| 345 | 63 | C | C | G | 2.36 | 3.930525e-01 |  |
| 345 | 63 | C | C | T | 3.88 | 1.484434e-01 |  |
| 346 | 64 | A | A | A | 87.06 | 0.000000e+00 | \* |
| 346 | 64 | A | A | C | 6.20 | 9.729709e-03 | \* |
| 346 | 64 | A | A | G | 1.62 | 5.947482e-01 |  |
| 346 | 64 | A | A | T | 5.12 | 5.897283e-02 |  |
| 347 | 65 | G | G | A | 2.71 | 3.805157e-01 |  |
| 347 | 65 | G | G | C | 3.05 | 2.104199e-01 |  |
| 347 | 65 | G | G | G | 92.71 | 0.000000e+00 | \* |
| 347 | 65 | G | G | T | 1.53 | 6.174555e-01 |  |
| 348 | 66 | C | C | A | 0.00 | 8.392857e-01 |  |
| 348 | 66 | C | C | C | 94.73 | 0.000000e+00 | \* |
| 348 | 66 | C | C | G | 1.62 | 5.931694e-01 |  |
| 348 | 66 | C | C | T | 3.65 | 1.741877e-01 |  |
| 349 | 67 | T | T | A | 0.00 | 8.392857e-01 |  |
| 349 | 67 | T | T | C | 4.35 | 6.475434e-02 |  |
| 349 | 67 | T | T | G | 1.86 | 5.244116e-01 |  |
| 349 | 67 | T | T | T | 93.79 | 0.000000e+00 | \* |
| 350 | 68 | C | C | A | 1.50 | 6.540490e-01 |  |
| 350 | 68 | C | C | C | 91.57 | 0.000000e+00 | \* |
| 350 | 68 | C | C | G | 2.62 | 3.328373e-01 |  |
| 350 | 68 | C | C | T | 4.31 | 1.089144e-01 |  |
| 351 | 69 | C | C | A | 1.01 | 7.545158e-01 |  |
| 351 | 69 | C | C | C | 93.21 | 0.000000e+00 | \* |
| 351 | 69 | C | C | G | 0.14 | 8.865579e-01 |  |
| 351 | 69 | C | C | T | 5.64 | 3.946382e-02 |  |
| 352 | 70 | C | C | A | 3.30 | 2.744822e-01 |  |
| 352 | 70 | C | C | C | 92.53 | 0.000000e+00 | \* |
| 352 | 70 | C | C | G | 1.32 | 6.804180e-01 |  |
| 352 | 70 | C | C | T | 2.86 | 2.956027e-01 |  |
| 353 | 71 | G | G | A | 4.55 | 1.245864e-01 |  |
| 353 | 71 | G | G | C | 3.41 | 1.545722e-01 |  |
| 353 | 71 | G | G | G | 89.77 | 0.000000e+00 | \* |
| 353 | 71 | G | G | T | 2.27 | 4.203741e-01 |  |
| 354 | 72 | C | C | A | 0.00 | 8.392857e-01 |  |
| 354 | 72 | C | C | C | 94.74 | 0.000000e+00 | \* |
| 354 | 72 | C | C | G | 2.85 | 2.855253e-01 |  |
| 354 | 72 | C | C | T | 2.41 | 3.878152e-01 |  |
| 355 | 73 | G | G | A | 6.86 | 2.343495e-02 |  |
| 355 | 73 | G | G | C | 6.15 | 1.029580e-02 |  |
| 355 | 73 | G | G | G | 87.00 | 0.000000e+00 | \* |
| 355 | 73 | G | G | T | 0.00 | 8.947368e-01 |  |
| 356 | 74 | A | A | A | 93.43 | 0.000000e+00 | \* |
| 356 | 74 | A | A | C | 1.35 | 6.820399e-01 |  |
| 356 | 74 | A | A | G | 3.03 | 2.521539e-01 |  |
| 356 | 74 | A | A | T | 2.19 | 4.408000e-01 |  |
| 357 | 75 | C | C | A | 2.81 | 3.602013e-01 |  |
| 357 | 75 | C | C | C | 63.25 | 0.000000e+00 | \* |
| 357 | 75 | C | C | G | 30.79 | 1.558753e-13 | \* |
| 357 | 75 | C | C | T | 3.15 | 2.453415e-01 |  |

## For use in R

If you want to work with the results in R, here is output that you can copy and paste in your terminal to get:

The base information:

```
structure(list(focal.base = c("A", "C", "G", "T"), avg.percsignal = c(92.3307391819762, 
91.8782186882864, 93.5118445106871, 93.8734837009024), avg.areasignal = c(535.25, 
555.086956521739, 580.206896551724, 605.47619047619), crit.perc.area = c(7.95007076916177, 
6.173848681947, 6.90496816495847, 7.32179843840871), mu = c(2.85022693772945, 
2.25018211269217, 2.44337476390854, 2.48071342291195), fillibens = c(0.993884383414717, 
0.993316901147812, 0.99616857529516, 0.997328094259559)), .Names = c("focal.base", 
"avg.percsignal", "avg.areasignal", "crit.perc.area", "mu", "fillibens"
), row.names = c(NA, -4L), class = "data.frame")
```

the data.frame that contains information on the guide region:

```
structure(list(A.area = c(472, 566, 14, 940, 0, 0, 43, 35, 25, 
26, 440, 18, 933, 0, 19, 20, 0, 27, 0, 2, 0, 12, 1, 15, 8, 389, 
13, 13, 517, 16, 384, 11, 8, 2, 9, 10, 428, 22, 21, 24, 5, 18, 
481, 26, 11, 14, 785, 33, 0, 13, 11, 267, 477, 17, 525, 469, 
11, 11, 106, 15, 15, 643, 19, 323, 16, 0, 0, 8, 7, 15, 20, 0, 
29, 555, 17), C.area = c(5, 13, 15, 9, 530, 22, 24, 11, 368, 
618, 7, 8, 9, 583, 501, 14, 9, 0, 1, 1, 13, 17, 5, 8, 489, 15, 
575, 27, 10, 545, 13, 488, 19, 0, 11, 423, 15, 623, 574, 553, 
34, 14, 13, 522, 475, 12, 7, 473, 14, 12, 336, 3, 5, 8, 2, 10, 
16, 12, 13, 461, 17, 8, 537, 23, 18, 467, 28, 489, 645, 421, 
15, 432, 26, 8, 382), G.area = c(9, 9, 472, 30, 0, 15, 1035, 
721, 16, 16, 14, 506, 14, 11, 10, 548, 14, 888, 30, 14, 16, 779, 
19, 621, 23, 2, 2, 7, 7, 10, 25, 15, 384, 640, 500, 19, 13, 8, 
24, 1, 10, 733, 22, 16, 17, 428, 20, 4, 315, 623, 23, 5, 4, 379, 
10, 4, 322, 5, 15, 4, 330, 16, 14, 6, 547, 8, 12, 14, 1, 6, 395, 
13, 368, 18, 186), T.area = c(9, 13, 0, 26, 10, 600, 6, 2, 7, 
12, 12, 0, 30, 14, 3, 10, 410, 23, 517, 588, 568, 12, 467, 19, 
28, 16, 11, 590, 22, 12, 8, 23, 6, 2, 4, 10, 16, 13, 16, 21, 
557, 11, 124, 12, 21, 0, 31, 16, 0, 0, 7, 15, 17, 16, 29, 28, 
11, 457, 364, 9, 7, 32, 23, 19, 9, 18, 604, 23, 39, 13, 10, 11, 
0, 13, 19), Tot.area = c(495, 601, 501, 1005, 540, 637, 1108, 
769, 416, 672, 473, 532, 986, 608, 533, 592, 433, 938, 548, 605, 
597, 820, 492, 663, 548, 422, 601, 637, 556, 583, 430, 537, 417, 
644, 524, 462, 472, 666, 635, 599, 606, 776, 640, 576, 524, 454, 
843, 526, 329, 648, 377, 290, 503, 420, 566, 511, 360, 485, 498, 
489, 369, 699, 593, 371, 590, 493, 644, 534, 692, 455, 440, 456, 
423, 594, 604), A.perc = c(95.3535353535353, 94.1763727121464, 
2.79441117764471, 93.5323383084577, 0, 0, 3.88086642599278, 4.55136540962289, 
6.00961538461539, 3.86904761904762, 93.0232558139535, 3.38345864661654, 
94.6247464503043, 0, 3.56472795497186, 3.37837837837838, 0, 2.87846481876333, 
0, 0.330578512396694, 0, 1.46341463414634, 0.203252032520325, 
2.26244343891403, 1.45985401459854, 92.1800947867299, 2.1630615640599, 
2.04081632653061, 92.9856115107914, 2.74442538593482, 89.3023255813954, 
2.04841713221601, 1.91846522781775, 0.31055900621118, 1.7175572519084, 
2.16450216450216, 90.6779661016949, 3.3033033033033, 3.30708661417323, 
4.00667779632721, 0.825082508250825, 2.31958762886598, 75.15625, 
4.51388888888889, 2.09923664122137, 3.08370044052863, 93.1198102016607, 
6.27376425855513, 0, 2.00617283950617, 2.91777188328912, 92.0689655172414, 
94.831013916501, 4.04761904761905, 92.756183745583, 91.7808219178082, 
3.05555555555556, 2.2680412371134, 21.285140562249, 3.06748466257669, 
4.0650406504065, 91.9885550786838, 3.20404721753794, 87.0619946091644, 
2.71186440677966, 0, 0, 1.49812734082397, 1.01156069364162, 3.2967032967033, 
4.54545454545455, 0, 6.85579196217494, 93.4343434343434, 2.81456953642384
), C.perc = c(1.01010101010101, 2.1630615640599, 2.9940119760479, 
0.895522388059702, 98.1481481481482, 3.45368916797488, 2.16606498194946, 
1.43042912873862, 88.4615384615385, 91.9642857142857, 1.47991543340381, 
1.50375939849624, 0.912778904665314, 95.8881578947368, 93.9962476547842, 
2.36486486486486, 2.07852193995381, 0, 0.182481751824818, 0.165289256198347, 
2.17755443886097, 2.07317073170732, 1.01626016260163, 1.20663650075415, 
89.2335766423358, 3.55450236966825, 95.6738768718802, 4.23861852433281, 
1.79856115107914, 93.4819897084048, 3.02325581395349, 90.8752327746741, 
4.55635491606715, 0, 2.09923664122137, 91.5584415584416, 3.17796610169492, 
93.5435435435435, 90.3937007874016, 92.3205342237062, 5.61056105610561, 
1.80412371134021, 2.03125, 90.625, 90.6488549618321, 2.6431718061674, 
0.830367734282325, 89.9239543726236, 4.25531914893617, 1.85185185185185, 
89.1246684350133, 1.03448275862069, 0.99403578528827, 1.9047619047619, 
0.353356890459364, 1.95694716242661, 4.44444444444444, 2.47422680412371, 
2.61044176706827, 94.2740286298569, 4.6070460704607, 1.1444921316166, 
90.5564924114671, 6.19946091644205, 3.05084745762712, 94.7261663286004, 
4.34782608695652, 91.5730337078652, 93.2080924855491, 92.5274725274725, 
3.40909090909091, 94.7368421052632, 6.14657210401891, 1.34680134680135, 
63.2450331125828), G.perc = c(1.81818181818182, 1.49750415973378, 
94.2115768463074, 2.98507462686567, 0, 2.35478806907378, 93.4115523465704, 
93.7581274382315, 3.84615384615385, 2.38095238095238, 2.95983086680761, 
95.1127819548872, 1.41987829614604, 1.80921052631579, 1.87617260787993, 
92.5675675675676, 3.23325635103926, 94.6695095948827, 5.47445255474453, 
2.31404958677686, 2.68006700167504, 95, 3.86178861788618, 93.6651583710407, 
4.1970802919708, 0.4739336492891, 0.332778702163062, 1.0989010989011, 
1.2589928057554, 1.71526586620926, 5.81395348837209, 2.79329608938547, 
92.0863309352518, 99.3788819875776, 95.4198473282443, 4.11255411255411, 
2.75423728813559, 1.2012012012012, 3.77952755905512, 0.1669449081803, 
1.65016501650165, 94.4587628865979, 3.4375, 2.77777777777778, 
3.24427480916031, 94.273127753304, 2.37247924080664, 0.760456273764259, 
95.7446808510638, 96.141975308642, 6.10079575596817, 1.72413793103448, 
0.795228628230616, 90.2380952380952, 1.76678445229682, 0.782778864970646, 
89.4444444444444, 1.03092783505155, 3.01204819277108, 0.81799591002045, 
89.4308943089431, 2.28898426323319, 2.36087689713322, 1.61725067385445, 
92.7118644067797, 1.62271805273834, 1.86335403726708, 2.62172284644195, 
0.144508670520231, 1.31868131868132, 89.7727272727273, 2.85087719298246, 
86.9976359338062, 3.03030303030303, 30.794701986755), T.perc = c(1.81818181818182, 
2.1630615640599, 0, 2.58706467661692, 1.85185185185185, 94.1915227629513, 
0.541516245487365, 0.260078023407022, 1.68269230769231, 1.78571428571429, 
2.5369978858351, 0, 3.04259634888438, 2.30263157894737, 0.562851782363977, 
1.68918918918919, 94.6882217090069, 2.45202558635394, 94.3430656934307, 
97.1900826446281, 95.142378559464, 1.46341463414634, 94.9186991869919, 
2.8657616892911, 5.10948905109489, 3.7914691943128, 1.83028286189684, 
92.6216640502355, 3.9568345323741, 2.05831903945111, 1.86046511627907, 
4.28305400372439, 1.43884892086331, 0.31055900621118, 0.763358778625954, 
2.16450216450216, 3.38983050847458, 1.95195195195195, 2.51968503937008, 
3.50584307178631, 91.9141914191419, 1.41752577319588, 19.375, 
2.08333333333333, 4.00763358778626, 0, 3.6773428232503, 3.04182509505703, 
0, 0, 1.85676392572944, 5.17241379310345, 3.37972166998012, 3.80952380952381, 
5.12367491166078, 5.47945205479452, 3.05555555555556, 94.2268041237113, 
73.0923694779116, 1.84049079754601, 1.8970189701897, 4.57796852646638, 
3.87858347386172, 5.12129380053908, 1.52542372881356, 3.65111561866126, 
93.7888198757764, 4.30711610486891, 5.63583815028902, 2.85714285714286, 
2.27272727272727, 2.41228070175439, 0, 2.18855218855219, 3.14569536423841
), base.call = c("A", "A", "G", "A", "C", "T", "G", "G", "C", 
"C", "A", "G", "A", "C", "C", "G", "T", "G", "T", "T", "T", "G", 
"T", "G", "C", "A", "C", "T", "A", "C", "A", "C", "G", "G", "G", 
"C", "A", "C", "C", "C", "T", "G", "A", "C", "C", "G", "A", "C", 
"G", "G", "C", "A", "A", "G", "A", "A", "G", "T", "T", "C", "G", 
"A", "C", "A", "G", "C", "T", "C", "C", "C", "G", "C", "G", "A", 
"C"), index = 283:357, guide.seq = c("A", "A", "G", "A", "C", 
"T", "G", "G", "C", "C", "A", "G", "A", "C", "C", "G", "T", "G", 
"T", "T", "T", "G", "T", "G", "C", "A", "C", "T", "A", "C", "A", 
"C", "G", "G", "G", "C", "A", "C", "C", "C", "T", "G", "A", "C", 
"C", "G", "A", "C", "G", "G", "C", "A", "A", "G", "A", "A", "G", 
"T", "T", "C", "G", "A", "C", "A", "G", "C", "T", "C", "C", "C", 
"G", "C", "G", "A", "C"), T.pval = c(0.53677854117883, 0.447097269566412, 
0.894736842104279, 0.349447026234132, 0.527703564223005, 0, 0.855231817134479, 
0.887162872982613, 0.573803670713552, 0.545581453688848, 0.360157627960401, 
0.894736842104279, 0.262453975187421, 0.413257661958231, 0.851831248190178, 
0.572012640943214, 0, 0.3788511851526, 0, 0, 0, 0.634738315447226, 
0, 0.293993690622882, 0.059511896976388, 0.157880752107745, 0.533510436094394, 
0, 0.140392850995933, 0.473491680255204, 0.525391401516366, 0.11085418674558, 
0.641580540575405, 0.883304686527095, 0.814209430571856, 0.446740024289801, 
0.208422230946306, 0.501088990718451, 0.363913928440514, 0.192575718342761, 
0, 0.647514021434601, 1.83954953381082e-07, 0.467114644117861, 
0.135375542343221, 0.894736842104279, 0.171034118351774, 0.262585491309856, 
0.894736842104279, 0.894736842104279, 0.526384477553251, 0.0566906398612689, 
0.209853527398292, 0.155882802277546, 0.0588646502800858, 0.0446333112714704, 
0.260251968417934, 0, 0, 0.530759264702353, 0.515623513291205, 
0.0891074682262321, 0.148443400714237, 0.0589728302633336, 0.61745553960075, 
0.174187665410014, 0, 0.108914448942311, 0.0394638201736497, 
0.29560271322225, 0.420374074811405, 0.387815187022049, 0.894736842104279, 
0.440799953075951, 0.245341506689989), C.pval = c(0.788102442859347, 
0.417977297502593, 0.220653253698172, 0.819887729272867, 0, 0.148596400260026, 
0.417103799947605, 0.65408887009338, 0, 0, 0.637443163474182, 
0.629409956470985, 0.815294586283187, 0, 0, 0.36175709047548, 
0.442994261352141, 0.926829268292683, 0.925197572063583, 0.925604222774439, 
0.413772176406884, 0.444605048346307, 0.786313724355811, 0.727865651658746, 
0, 0.135819914757586, 0, 0.0719320695509518, 0.531068186523626, 
0, 0.215338454407103, 0, 0.0528477060213626, 0.926829268292683, 
0.436788894027366, 0, 0.188936226595522, 0, 0, 0, 0.0181358655661692, 
0.529252801178358, 0.457331642456973, 0, 0, 0.292902895457122, 
0.836537804126913, 0, 0.0707891636369389, 0.513769534505651, 
0, 0.78097837786764, 0.792732237440448, 0.496817261980502, 0.916365462281376, 
0.480338801163132, 0.0589594041083656, 0.333474577361228, 0.30046571760955, 
0, 0.0502773778174982, 0.747538887973149, 0, 0.00972970917563298, 
0.210419894611316, 0, 0.0647543399885768, 0, 0, 0, 0.154572202774316, 
0, 0.0102958048893567, 0.682039876493595, 0), G.pval = c(0.537159851321769, 
0.629337866189429, 0, 0.260265304702872, 0.887640449437961, 0.39453626851871, 
0, 0, 0.137779851968173, 0.388184828293819, 0.264880232348419, 
0, 0.651657959346225, 0.539702805491006, 0.520812189192666, 0, 
0.218190012633573, 0, 0.0359321127071659, 0.40455214995981, 0.320289700647782, 
0, 0.136119215626309, 0, 0.104509956058473, 0.864633857590927, 
0.878049486376481, 0.740279045456245, 0.697101439236016, 0.566517030267257, 
0.0267037048425679, 0.296916269786695, 0, 0, 0, 0.111790952393175, 
0.304833029381526, 0.712986804804868, 0.145056718864188, 0.886048257588722, 
0.585248969912306, 0, 0.187872055697743, 0.300043243800163, 0.216457661916649, 
0, 0.390234677501379, 0.818996583048902, 0, 0, 0.0207077830013702, 
0.56397215498593, 0.811968923103396, 0, 0.551773388307653, 0.814517891698872, 
0, 0.757689339495097, 0.255403591409616, 0.807215398446798, 0, 
0.410790049752038, 0.3930524761526, 0.594748194953665, 0, 0.593169396886923, 
0.524411580353494, 0.332837335221886, 0.886557892764, 0.680417971867725, 
0, 0.285525334894922, 0, 0.252153881200874, 1.55875312657372e-13
), A.pval = c(0, 0, 0.364133842474498, 0, 0.839285714285715, 
0.839285714285715, 0.192247183198336, 0.124092043980611, 0.044242895699372, 
0.193686254261931, 0, 0.260790494261847, 0, 0.839285714285715, 
0.23387610626741, 0.261577698526751, 0.839285714285715, 0.347915976466894, 
0.839285714285715, 0.833516762947454, 0.839285714285715, 0.661877434332578, 
0.837691163255761, 0.476770422138127, 0.6626765962446, 0, 0.49937766078373, 
0.527630390186927, 0, 0.374000878459315, 0, 0.52586220257454, 
0.556238551408123, 0.834382227553437, 0.603397581948256, 0.499047390951161, 
0, 0.27342194926115, 0.272815592254191, 0.177470340518462, 0.785102008163113, 
0.463951766348631, 0, 0.127255535610373, 0.514076271481117, 0.310348582623136, 
0, 0.036371874961857, 0.839285714285715, 0.535705196461141, 0.340495720880123, 
0, 0, 0.172871543264795, 0, 0, 0.315326907241335, 0.475508522088266, 
9.24104540667159e-08, 0.313210074190549, 0.170945271991891, 0, 
0.289690508033515, 0, 0.380515720242494, 0.839285714285715, 0.839285714285715, 
0.654048984488402, 0.754515751609575, 0.274482155750594, 0.124586361425469, 
0.839285714285715, 0.0234349546566279, 0, 0.360201251133159), 
    guide.position = 1:75), .Names = c("A.area", "C.area", "G.area", 
"T.area", "Tot.area", "A.perc", "C.perc", "G.perc", "T.perc", 
"base.call", "index", "guide.seq", "T.pval", "C.pval", "G.pval", 
"A.pval", "guide.position"), row.names = 283:357, class = "data.frame")
```

*Report generated using EditR v1.0.8*
