# Supplementary material for: Mechanistic and genetic basis of single-strand templated repair at Cas12a-induced DNA breaks in Chlamydomonas reinhardtii
Source: Nat Commun. 2021 Nov 19;12:6751. doi: 10.1038/s41467-021-27004-1 (PMC8604939; doi:10.1038/s41467-021-27004-1)
Supplement: Supplementary file 22 — Source Data [file 41467_2021_27004_MOESM22_ESM.zip › Source Data/EditR analysis/EditR outputs/Sense/rep2_ssODN_sense_16.html]

EditR v1.0.8 report


# EditR v1.0.8 report

- Data QA
  - Filtering data
  - Percent noise peak area
  - Base information
- Predicted editing
  - Editing bar plot
  - Editing table plot
  - Table of editing results
- For use in R

## Data QA

### Filtering data

What the data looked like prefiltering:

and the post filtering signal / noise plot:

### Percent noise peak area

### Base information

Here’s information about the signal of each base, the critical percent value where any higher value would be called as significant, and Filliben’s correlation for how well the noise was modelled by the zero adjusted gamma distribution.

| Base | Average percent signal | Average peak area | Critical percent value | model mu | Fillibens correlation |
| --- | --- | --- | --- | --- | --- |
| A | 91.37216 | 277.5429 | 10.046039 | 3.593044 | 0.9936371 |
| C | 91.19041 | 284.6515 | 6.725086 | 2.311683 | 0.9748181 |
| G | 91.17013 | 282.0702 | 10.600223 | 3.005553 | 0.9893537 |
| T | 92.08424 | 306.8837 | 8.393375 | 2.754726 | 0.9949864 |

## Predicted editing

### Editing bar plot

### Editing table plot

### Table of editing results


Here’s the entire guide region

| Sanger position | Guide position | Guide sequence | Sanger base call | Focal base | Focal base peak area | p value |  |
| --- | --- | --- | --- | --- | --- | --- | --- |
| 274 | 1 | A | A | A | 94.02 | 0.000000e+00 | \* |
| 274 | 1 | A | A | C | 1.59 | 5.786553e-01 |  |
| 274 | 1 | A | A | G | 0.80 | 8.359513e-01 |  |
| 274 | 1 | A | A | T | 3.59 | 2.343270e-01 |  |
| 275 | 2 | A | A | A | 94.89 | 0.000000e+00 | \* |
| 275 | 2 | A | A | C | 1.28 | 6.749742e-01 |  |
| 275 | 2 | A | A | G | 1.28 | 7.271876e-01 |  |
| 275 | 2 | A | A | T | 2.56 | 4.083612e-01 |  |
| 276 | 3 | G | G | A | 3.35 | 4.347425e-01 |  |
| 276 | 3 | G | G | C | 4.60 | 6.597033e-02 |  |
| 276 | 3 | G | G | G | 92.05 | 0.000000e+00 | \* |
| 276 | 3 | G | G | T | 0.00 | 8.936170e-01 |  |
| 277 | 4 | A | A | A | 94.47 | 0.000000e+00 | \* |
| 277 | 4 | A | A | C | 0.84 | 7.996520e-01 |  |
| 277 | 4 | A | A | G | 1.17 | 7.514471e-01 |  |
| 277 | 4 | A | A | T | 3.52 | 2.436105e-01 |  |
| 278 | 5 | C | C | A | 6.85 | 7.170689e-02 |  |
| 278 | 5 | C | C | C | 85.98 | 0.000000e+00 | \* |
| 278 | 5 | C | C | G | 3.74 | 2.785223e-01 |  |
| 278 | 5 | C | C | T | 3.43 | 2.564624e-01 |  |
| 279 | 6 | T | T | A | 0.00 | 9.266055e-01 |  |
| 279 | 6 | T | T | C | 2.92 | 2.489079e-01 |  |
| 279 | 6 | T | T | G | 3.57 | 2.994933e-01 |  |
| 279 | 6 | T | T | T | 93.51 | 0.000000e+00 | \* |
| 280 | 7 | G | G | A | 3.74 | 3.674399e-01 |  |
| 280 | 7 | G | G | C | 0.51 | 8.687111e-01 |  |
| 280 | 7 | G | G | G | 94.91 | 0.000000e+00 | \* |
| 280 | 7 | G | G | T | 0.85 | 8.025266e-01 |  |
| 281 | 8 | G | G | A | 6.68 | 7.911446e-02 |  |
| 281 | 8 | G | G | C | 1.80 | 5.172321e-01 |  |
| 281 | 8 | G | G | G | 91.00 | 0.000000e+00 | \* |
| 281 | 8 | G | G | T | 0.51 | 8.594580e-01 |  |
| 282 | 9 | C | C | A | 6.14 | 1.077499e-01 |  |
| 282 | 9 | C | C | C | 86.84 | 0.000000e+00 | \* |
| 282 | 9 | C | C | G | 3.07 | 3.706959e-01 |  |
| 282 | 9 | C | C | T | 3.95 | 1.898219e-01 |  |
| 283 | 10 | C | C | A | 3.86 | 3.476985e-01 |  |
| 283 | 10 | C | C | C | 91.10 | 0.000000e+00 | \* |
| 283 | 10 | C | C | G | 2.97 | 3.868940e-01 |  |
| 283 | 10 | C | C | T | 2.08 | 5.133267e-01 |  |
| 284 | 11 | A | A | A | 92.37 | 0.000000e+00 | \* |
| 284 | 11 | A | A | C | 2.41 | 3.550771e-01 |  |
| 284 | 11 | A | A | G | 4.42 | 2.058510e-01 |  |
| 284 | 11 | A | A | T | 0.80 | 8.114805e-01 |  |
| 285 | 12 | G | G | A | 5.16 | 1.834777e-01 |  |
| 285 | 12 | G | G | C | 0.79 | 8.104728e-01 |  |
| 285 | 12 | G | G | G | 93.25 | 0.000000e+00 | \* |
| 285 | 12 | G | G | T | 0.79 | 8.133141e-01 |  |
| 286 | 13 | A | A | A | 94.02 | 0.000000e+00 | \* |
| 286 | 13 | A | A | C | 0.88 | 7.891299e-01 |  |
| 286 | 13 | A | A | G | 1.76 | 6.186700e-01 |  |
| 286 | 13 | A | A | T | 3.34 | 2.693788e-01 |  |
| 287 | 14 | C | C | A | 6.73 | 7.712923e-02 |  |
| 287 | 14 | C | C | C | 88.69 | 0.000000e+00 | \* |
| 287 | 14 | C | C | G | 3.06 | 3.725689e-01 |  |
| 287 | 14 | C | C | T | 1.53 | 6.457744e-01 |  |
| 288 | 15 | C | C | A | 7.20 | 5.853057e-02 |  |
| 288 | 15 | C | C | C | 88.00 | 0.000000e+00 | \* |
| 288 | 15 | C | C | G | 3.60 | 2.958096e-01 |  |
| 288 | 15 | C | C | T | 1.20 | 7.254049e-01 |  |
| 289 | 16 | G | G | A | 5.19 | 1.800433e-01 |  |
| 289 | 16 | G | G | C | 1.95 | 4.746046e-01 |  |
| 289 | 16 | G | G | G | 91.23 | 0.000000e+00 | \* |
| 289 | 16 | G | G | T | 1.62 | 6.225769e-01 |  |
| 290 | 17 | T | T | A | 2.50 | 6.018712e-01 |  |
| 290 | 17 | T | T | C | 0.42 | 8.822770e-01 |  |
| 290 | 17 | T | T | G | 5.00 | 1.576849e-01 |  |
| 290 | 17 | T | T | T | 92.08 | 0.000000e+00 | \* |
| 291 | 18 | G | G | A | 4.45 | 2.623236e-01 |  |
| 291 | 18 | G | G | C | 0.00 | 9.058824e-01 |  |
| 291 | 18 | G | G | G | 93.12 | 0.000000e+00 | \* |
| 291 | 18 | G | G | T | 2.43 | 4.348025e-01 |  |
| 292 | 19 | T | T | A | 0.00 | 9.266055e-01 |  |
| 292 | 19 | T | T | C | 2.13 | 4.255778e-01 |  |
| 292 | 19 | T | T | G | 4.61 | 1.886380e-01 |  |
| 292 | 19 | T | T | T | 93.26 | 0.000000e+00 | \* |
| 293 | 20 | T | T | A | 0.64 | 9.092965e-01 |  |
| 293 | 20 | T | T | C | 1.61 | 5.743862e-01 |  |
| 293 | 20 | T | T | G | 4.50 | 1.981682e-01 |  |
| 293 | 20 | T | T | T | 93.25 | 0.000000e+00 | \* |
| 294 | 21 | T | T | A | 0.00 | 9.266055e-01 |  |
| 294 | 21 | T | T | C | 0.00 | 9.058824e-01 |  |
| 294 | 21 | T | T | G | 4.47 | 2.007713e-01 |  |
| 294 | 21 | T | T | T | 95.53 | 0.000000e+00 | \* |
| 295 | 22 | G | G | A | 1.26 | 8.404769e-01 |  |
| 295 | 22 | G | G | C | 1.26 | 6.815316e-01 |  |
| 295 | 22 | G | G | G | 96.48 | 0.000000e+00 | \* |
| 295 | 22 | G | G | T | 1.01 | 7.697956e-01 |  |
| 296 | 23 | T | T | A | 0.00 | 9.266055e-01 |  |
| 296 | 23 | T | T | C | 1.51 | 6.042863e-01 |  |
| 296 | 23 | T | T | G | 6.42 | 8.074819e-02 |  |
| 296 | 23 | T | T | T | 92.08 | 0.000000e+00 | \* |
| 297 | 24 | G | G | A | 1.25 | 8.414262e-01 |  |
| 297 | 24 | G | G | C | 2.19 | 4.099281e-01 |  |
| 297 | 24 | G | G | G | 94.06 | 0.000000e+00 | \* |
| 297 | 24 | G | G | T | 2.50 | 4.198947e-01 |  |
| 298 | 25 | C | C | A | 1.98 | 7.099421e-01 |  |
| 298 | 25 | C | C | C | 94.86 | 0.000000e+00 | \* |
| 298 | 25 | C | C | G | 0.00 | 9.555556e-01 |  |
| 298 | 25 | C | C | T | 3.16 | 2.970841e-01 |  |
| 299 | 26 | A | A | A | 83.72 | 0.000000e+00 | \* |
| 299 | 26 | A | A | C | 3.26 | 1.945582e-01 |  |
| 299 | 26 | A | A | G | 6.51 | 7.707442e-02 |  |
| 299 | 26 | A | A | T | 6.51 | 3.687610e-02 |  |
| 300 | 27 | C | C | A | 4.78 | 2.231688e-01 |  |
| 300 | 27 | C | C | C | 90.78 | 0.000000e+00 | \* |
| 300 | 27 | C | C | G | 1.71 | 6.299228e-01 |  |
| 300 | 27 | C | C | T | 2.73 | 3.737215e-01 |  |
| 301 | 28 | T | T | A | 1.77 | 7.513637e-01 |  |
| 301 | 28 | T | T | C | 5.65 | 2.646647e-02 |  |
| 301 | 28 | T | T | G | 0.71 | 8.551128e-01 |  |
| 301 | 28 | T | T | T | 91.87 | 0.000000e+00 | \* |
| 302 | 29 | A | A | A | 92.83 | 0.000000e+00 | \* |
| 302 | 29 | A | A | C | 1.89 | 4.919749e-01 |  |
| 302 | 29 | A | A | G | 2.26 | 5.127013e-01 |  |
| 302 | 29 | A | A | T | 3.02 | 3.210616e-01 |  |
| 303 | 30 | C | C | A | 3.91 | 3.400550e-01 |  |
| 303 | 30 | C | C | C | 91.80 | 0.000000e+00 | \* |
| 303 | 30 | C | C | G | 1.95 | 5.764045e-01 |  |
| 303 | 30 | C | C | T | 2.34 | 4.531934e-01 |  |
| 304 | 31 | A | A | A | 88.27 | 0.000000e+00 | \* |
| 304 | 31 | A | A | C | 5.10 | 4.300062e-02 |  |
| 304 | 31 | A | A | G | 4.59 | 1.902002e-01 |  |
| 304 | 31 | A | A | T | 2.04 | 5.218078e-01 |  |
| 305 | 32 | C | C | A | 2.59 | 5.840446e-01 |  |
| 305 | 32 | C | C | C | 92.24 | 0.000000e+00 | \* |
| 305 | 32 | C | C | G | 3.02 | 3.789659e-01 |  |
| 305 | 32 | C | C | T | 2.16 | 4.953318e-01 |  |
| 306 | 33 | G | G | A | 3.41 | 4.225564e-01 |  |
| 306 | 33 | G | G | C | 4.39 | 7.882903e-02 |  |
| 306 | 33 | G | G | G | 89.27 | 0.000000e+00 | \* |
| 306 | 33 | G | G | T | 2.93 | 3.372297e-01 |  |
| 307 | 34 | G | G | A | 2.60 | 5.817364e-01 |  |
| 307 | 34 | G | G | C | 1.30 | 6.686790e-01 |  |
| 307 | 34 | G | G | G | 96.10 | 0.000000e+00 | \* |
| 307 | 34 | G | G | T | 0.00 | 8.936170e-01 |  |
| 308 | 35 | G | G | A | 2.39 | 6.246184e-01 |  |
| 308 | 35 | G | G | C | 1.99 | 4.623283e-01 |  |
| 308 | 35 | G | G | G | 95.62 | 0.000000e+00 | \* |
| 308 | 35 | G | G | T | 0.00 | 8.936170e-01 |  |
| 309 | 36 | C | C | A | 3.74 | 3.669188e-01 |  |
| 309 | 36 | C | C | C | 92.99 | 0.000000e+00 | \* |
| 309 | 36 | C | C | G | 2.80 | 4.138243e-01 |  |
| 309 | 36 | C | C | T | 0.47 | 8.655431e-01 |  |
| 310 | 37 | A | A | A | 92.38 | 0.000000e+00 | \* |
| 310 | 37 | A | A | C | 4.76 | 5.762412e-02 |  |
| 310 | 37 | A | A | G | 0.00 | 9.555556e-01 |  |
| 310 | 37 | A | A | T | 2.86 | 3.498659e-01 |  |
| 311 | 38 | C | C | A | 4.90 | 2.095461e-01 |  |
| 311 | 38 | C | C | C | 93.14 | 0.000000e+00 | \* |
| 311 | 38 | C | C | G | 0.00 | 9.555556e-01 |  |
| 311 | 38 | C | C | T | 1.96 | 5.406944e-01 |  |
| 312 | 39 | C | C | A | 4.68 | 2.342074e-01 |  |
| 312 | 39 | C | C | C | 91.30 | 0.000000e+00 | \* |
| 312 | 39 | C | C | G | 2.01 | 5.651126e-01 |  |
| 312 | 39 | C | C | T | 2.01 | 5.298280e-01 |  |
| 313 | 40 | C | C | A | 4.93 | 2.066026e-01 |  |
| 313 | 40 | C | C | C | 91.90 | 0.000000e+00 | \* |
| 313 | 40 | C | C | G | 0.70 | 8.556322e-01 |  |
| 313 | 40 | C | C | T | 2.46 | 4.272632e-01 |  |
| 314 | 41 | T | T | A | 2.38 | 6.265901e-01 |  |
| 314 | 41 | T | T | C | 6.75 | 9.808125e-03 | \* |
| 314 | 41 | T | T | G | 3.57 | 2.994933e-01 |  |
| 314 | 41 | T | T | T | 87.30 | 0.000000e+00 | \* |
| 315 | 42 | G | G | A | 3.50 | 4.076607e-01 |  |
| 315 | 42 | G | G | C | 3.50 | 1.616295e-01 |  |
| 315 | 42 | G | G | G | 89.50 | 0.000000e+00 | \* |
| 315 | 42 | G | G | T | 3.50 | 2.462617e-01 |  |
| 316 | 43 | A | A | A | 91.30 | 0.000000e+00 | \* |
| 316 | 43 | A | A | C | 1.45 | 6.226849e-01 |  |
| 316 | 43 | A | A | G | 4.93 | 1.630558e-01 |  |
| 316 | 43 | A | A | T | 2.32 | 4.586419e-01 |  |
| 317 | 44 | C | C | A | 7.04 | 6.442237e-02 |  |
| 317 | 44 | C | C | C | 88.89 | 0.000000e+00 | \* |
| 317 | 44 | C | C | G | 3.33 | 3.317372e-01 |  |
| 317 | 44 | C | C | T | 0.74 | 8.231895e-01 |  |
| 318 | 45 | C | C | A | 0.00 | 9.266055e-01 |  |
| 318 | 45 | C | C | C | 94.50 | 0.000000e+00 | \* |
| 318 | 45 | C | C | G | 4.13 | 2.344228e-01 |  |
| 318 | 45 | C | C | T | 1.38 | 6.832027e-01 |  |
| 319 | 46 | G | G | A | 5.05 | 1.945803e-01 |  |
| 319 | 46 | G | G | C | 6.42 | 1.321736e-02 |  |
| 319 | 46 | G | G | G | 87.61 | 0.000000e+00 | \* |
| 319 | 46 | G | G | T | 0.92 | 7.885353e-01 |  |
| 320 | 47 | A | A | A | 93.57 | 0.000000e+00 | \* |
| 320 | 47 | A | A | C | 1.07 | 7.359329e-01 |  |
| 320 | 47 | A | A | G | 3.49 | 3.108420e-01 |  |
| 320 | 47 | A | A | T | 1.88 | 5.608154e-01 |  |
| 321 | 48 | C | C | A | 8.30 | 3.015186e-02 |  |
| 321 | 48 | C | C | C | 86.31 | 0.000000e+00 | \* |
| 321 | 48 | C | C | G | 2.07 | 5.509636e-01 |  |
| 321 | 48 | C | C | T | 3.32 | 2.723529e-01 |  |
| 322 | 49 | G | G | A | 7.28 | 5.566703e-02 |  |
| 322 | 49 | G | G | C | 7.95 | 3.171103e-03 | \* |
| 322 | 49 | G | G | G | 82.78 | 0.000000e+00 | \* |
| 322 | 49 | G | G | T | 1.99 | 5.345360e-01 |  |
| 323 | 50 | G | G | A | 3.45 | 4.165457e-01 |  |
| 323 | 50 | G | G | C | 1.25 | 6.822452e-01 |  |
| 323 | 50 | G | G | G | 95.30 | 0.000000e+00 | \* |
| 323 | 50 | G | G | T | 0.00 | 8.936170e-01 |  |
| 324 | 51 | C | C | A | 6.25 | 1.013143e-01 |  |
| 324 | 51 | C | C | C | 86.36 | 0.000000e+00 | \* |
| 324 | 51 | C | C | G | 4.55 | 1.942591e-01 |  |
| 324 | 51 | C | C | T | 2.84 | 3.528583e-01 |  |
| 325 | 52 | A | A | A | 84.68 | 0.000000e+00 | \* |
| 325 | 52 | A | A | C | 6.45 | 1.286370e-02 |  |
| 325 | 52 | A | A | G | 2.42 | 4.826956e-01 |  |
| 325 | 52 | A | A | T | 6.45 | 3.840029e-02 |  |
| 326 | 53 | A | A | A | 92.41 | 0.000000e+00 | \* |
| 326 | 53 | A | A | C | 0.84 | 7.980498e-01 |  |
| 326 | 53 | A | A | G | 2.11 | 5.437615e-01 |  |
| 326 | 53 | A | A | T | 4.64 | 1.245660e-01 |  |
| 327 | 54 | G | G | A | 8.11 | 3.389478e-02 |  |
| 327 | 54 | G | G | C | 1.62 | 5.701812e-01 |  |
| 327 | 54 | G | G | G | 90.27 | 0.000000e+00 | \* |
| 327 | 54 | G | G | T | 0.00 | 8.936170e-01 |  |
| 328 | 55 | A | A | A | 91.81 | 0.000000e+00 | \* |
| 328 | 55 | A | A | C | 0.00 | 9.058824e-01 |  |
| 328 | 55 | A | A | G | 4.27 | 2.199768e-01 |  |
| 328 | 55 | A | A | T | 3.91 | 1.935330e-01 |  |
| 329 | 56 | A | A | A | 93.01 | 0.000000e+00 | \* |
| 329 | 56 | A | A | C | 0.00 | 9.058824e-01 |  |
| 329 | 56 | A | A | G | 2.18 | 5.287939e-01 |  |
| 329 | 56 | A | A | T | 4.80 | 1.125735e-01 |  |
| 330 | 57 | G | G | A | 7.95 | 3.739496e-02 |  |
| 330 | 57 | G | G | C | 2.65 | 3.019355e-01 |  |
| 330 | 57 | G | G | G | 82.78 | 0.000000e+00 | \* |
| 330 | 57 | G | G | T | 6.62 | 3.421012e-02 |  |
| 331 | 58 | T | T | A | 0.47 | 9.191587e-01 |  |
| 331 | 58 | T | T | C | 0.93 | 7.755423e-01 |  |
| 331 | 58 | T | T | G | 2.79 | 4.160319e-01 |  |
| 331 | 58 | T | T | T | 95.81 | 0.000000e+00 | \* |
| 332 | 59 | T | T | A | 38.22 | 1.174427e-11 | \* |
| 332 | 59 | T | T | C | 1.78 | 5.235957e-01 |  |
| 332 | 59 | T | T | G | 0.89 | 8.157782e-01 |  |
| 332 | 59 | T | T | T | 59.11 | 0.000000e+00 | \* |
| 333 | 60 | C | C | A | 5.43 | 1.589758e-01 |  |
| 333 | 60 | C | C | C | 90.50 | 0.000000e+00 | \* |
| 333 | 60 | C | C | G | 0.45 | 9.044443e-01 |  |
| 333 | 60 | C | C | T | 3.62 | 2.297677e-01 |  |
| 334 | 61 | G | G | A | 6.80 | 7.385643e-02 |  |
| 334 | 61 | G | G | C | 4.76 | 5.762412e-02 |  |
| 334 | 61 | G | G | G | 87.07 | 0.000000e+00 | \* |
| 334 | 61 | G | G | T | 1.36 | 6.869923e-01 |  |
| 335 | 62 | A | A | A | 92.74 | 0.000000e+00 | \* |
| 335 | 62 | A | A | C | 1.61 | 5.728169e-01 |  |
| 335 | 62 | A | A | G | 2.69 | 4.337045e-01 |  |
| 335 | 62 | A | A | T | 2.96 | 3.318663e-01 |  |
| 336 | 63 | C | C | A | 6.48 | 8.904120e-02 |  |
| 336 | 63 | C | C | C | 89.07 | 0.000000e+00 | \* |
| 336 | 63 | C | C | G | 1.21 | 7.417763e-01 |  |
| 336 | 63 | C | C | T | 3.24 | 2.848069e-01 |  |
| 337 | 64 | A | A | A | 83.33 | 0.000000e+00 | \* |
| 337 | 64 | A | A | C | 8.33 | 2.190555e-03 | \* |
| 337 | 64 | A | A | G | 3.85 | 2.656590e-01 |  |
| 337 | 64 | A | A | T | 4.49 | 1.370248e-01 |  |
| 338 | 65 | G | G | A | 1.63 | 7.779364e-01 |  |
| 338 | 65 | G | G | C | 3.25 | 1.951133e-01 |  |
| 338 | 65 | G | G | G | 91.87 | 0.000000e+00 | \* |
| 338 | 65 | G | G | T | 3.25 | 2.827432e-01 |  |
| 339 | 66 | C | C | A | 1.39 | 8.194735e-01 |  |
| 339 | 66 | C | C | C | 93.06 | 0.000000e+00 | \* |
| 339 | 66 | C | C | G | 3.24 | 3.450399e-01 |  |
| 339 | 66 | C | C | T | 2.31 | 4.595259e-01 |  |
| 340 | 67 | T | T | A | 0.00 | 9.266055e-01 |  |
| 340 | 67 | T | T | C | 6.18 | 1.645585e-02 |  |
| 340 | 67 | T | T | G | 1.09 | 7.701672e-01 |  |
| 340 | 67 | T | T | T | 92.73 | 0.000000e+00 | \* |
| 341 | 68 | C | C | A | 2.13 | 6.790717e-01 |  |
| 341 | 68 | C | C | C | 92.77 | 0.000000e+00 | \* |
| 341 | 68 | C | C | G | 3.40 | 3.218400e-01 |  |
| 341 | 68 | C | C | T | 1.70 | 6.032462e-01 |  |
| 342 | 69 | C | C | A | 1.75 | 7.549289e-01 |  |
| 342 | 69 | C | C | C | 95.80 | 0.000000e+00 | \* |
| 342 | 69 | C | C | G | 0.35 | 9.214879e-01 |  |
| 342 | 69 | C | C | T | 2.10 | 5.085113e-01 |  |
| 343 | 70 | C | C | A | 0.00 | 9.266055e-01 |  |
| 343 | 70 | C | C | C | 95.78 | 0.000000e+00 | \* |
| 343 | 70 | C | C | G | 4.22 | 2.253320e-01 |  |
| 343 | 70 | C | C | T | 0.00 | 8.936170e-01 |  |
| 344 | 71 | G | G | A | 4.92 | 2.078289e-01 |  |
| 344 | 71 | G | G | C | 4.92 | 5.041260e-02 |  |
| 344 | 71 | G | G | G | 90.16 | 0.000000e+00 | \* |
| 344 | 71 | G | G | T | 0.00 | 8.936170e-01 |  |
| 345 | 72 | C | C | A | 3.81 | 3.553572e-01 |  |
| 345 | 72 | C | C | C | 91.43 | 0.000000e+00 | \* |
| 345 | 72 | C | C | G | 4.76 | 1.759705e-01 |  |
| 345 | 72 | C | C | T | 0.00 | 8.936170e-01 |  |
| 346 | 73 | G | G | A | 10.27 | 8.648485e-03 | \* |
| 346 | 73 | G | G | C | 7.57 | 4.546918e-03 | \* |
| 346 | 73 | G | G | G | 81.62 | 0.000000e+00 | \* |
| 346 | 73 | G | G | T | 0.54 | 8.557976e-01 |  |
| 347 | 74 | A | A | A | 92.12 | 0.000000e+00 | \* |
| 347 | 74 | A | A | C | 2.05 | 4.451075e-01 |  |
| 347 | 74 | A | A | G | 2.74 | 4.247479e-01 |  |
| 347 | 74 | A | A | T | 3.08 | 3.102817e-01 |  |
| 348 | 75 | C | C | A | 6.57 | 8.449618e-02 |  |
| 348 | 75 | C | C | C | 85.04 | 0.000000e+00 | \* |
| 348 | 75 | C | C | G | 1.46 | 6.854600e-01 |  |
| 348 | 75 | C | C | T | 6.93 | 2.766394e-02 |  |

## For use in R

If you want to work with the results in R, here is output that you can copy and paste in your terminal to get:

The base information:

```
structure(list(focal.base = c("A", "C", "G", "T"), avg.percsignal = c(91.3721557272998, 
91.1904136588375, 91.170134248384, 92.084235947388), avg.areasignal = c(277.542857142857, 
284.651515151515, 282.070175438596, 306.883720930233), crit.perc.area = c(10.0460394079936, 
6.72508632692411, 10.6002226300255, 8.39337458207793), mu = c(3.59304403326438, 
2.31168268048622, 3.00555257914766, 2.75472630246119), fillibens = c(0.993637091790125, 
0.974818091028138, 0.989353740810135, 0.994986420603847)), .Names = c("focal.base", 
"avg.percsignal", "avg.areasignal", "crit.perc.area", "mu", "fillibens"
), row.names = c(NA, -4L), class = "data.frame")
```

the data.frame that contains information on the guide region:

```
structure(list(A.area = c(236, 297, 8, 564, 22, 0, 22, 26, 14, 
13, 230, 13, 535, 22, 18, 16, 6, 22, 0, 2, 0, 5, 0, 4, 5, 180, 
14, 5, 246, 10, 173, 6, 7, 8, 6, 8, 194, 15, 14, 14, 6, 12, 315, 
19, 0, 11, 349, 20, 11, 11, 11, 105, 219, 15, 258, 213, 12, 1, 
86, 12, 10, 345, 16, 130, 4, 3, 0, 5, 5, 0, 9, 8, 19, 269, 18
), C.area = c(4, 4, 11, 5, 276, 9, 3, 7, 198, 307, 6, 2, 5, 290, 
220, 6, 1, 0, 6, 5, 0, 5, 4, 7, 240, 7, 266, 16, 5, 235, 10, 
214, 9, 4, 5, 199, 10, 285, 273, 261, 17, 12, 5, 240, 206, 14, 
4, 208, 12, 4, 152, 8, 2, 3, 0, 0, 4, 2, 4, 200, 7, 6, 220, 13, 
8, 201, 17, 218, 274, 159, 9, 192, 14, 6, 233), G.area = c(2, 
4, 220, 7, 12, 11, 559, 354, 7, 10, 11, 235, 10, 10, 9, 281, 
12, 460, 13, 14, 14, 384, 17, 301, 0, 14, 5, 2, 6, 5, 9, 7, 183, 
296, 240, 6, 0, 0, 6, 2, 9, 307, 17, 9, 9, 191, 13, 5, 125, 304, 
8, 3, 5, 167, 12, 5, 125, 6, 2, 1, 128, 10, 3, 6, 226, 7, 3, 
8, 1, 7, 165, 10, 151, 8, 4), T.area = c(9, 8, 0, 21, 11, 288, 
5, 2, 9, 7, 2, 2, 19, 5, 3, 5, 221, 12, 263, 290, 299, 4, 244, 
8, 8, 14, 8, 260, 8, 6, 4, 5, 6, 0, 0, 1, 6, 6, 6, 7, 220, 12, 
8, 2, 3, 2, 7, 8, 3, 0, 5, 8, 11, 0, 11, 11, 10, 206, 133, 8, 
2, 11, 8, 7, 8, 5, 255, 4, 6, 0, 0, 0, 1, 9, 19), Tot.area = c(251, 
313, 239, 597, 321, 308, 589, 389, 228, 337, 249, 252, 569, 327, 
250, 308, 240, 494, 282, 311, 313, 398, 265, 320, 253, 215, 293, 
283, 265, 256, 196, 232, 205, 308, 251, 214, 210, 306, 299, 284, 
252, 343, 345, 270, 218, 218, 373, 241, 151, 319, 176, 124, 237, 
185, 281, 229, 151, 215, 225, 221, 147, 372, 247, 156, 246, 216, 
275, 235, 286, 166, 183, 210, 185, 292, 274), A.perc = c(94.0239043824701, 
94.888178913738, 3.34728033472803, 94.4723618090452, 6.85358255451713, 
0, 3.73514431239389, 6.68380462724936, 6.14035087719298, 3.85756676557863, 
92.3694779116466, 5.15873015873016, 94.02460456942, 6.72782874617737, 
7.2, 5.19480519480519, 2.5, 4.45344129554656, 0, 0.643086816720257, 
0, 1.25628140703518, 0, 1.25, 1.97628458498024, 83.7209302325581, 
4.77815699658703, 1.76678445229682, 92.8301886792453, 3.90625, 
88.265306122449, 2.58620689655172, 3.41463414634146, 2.5974025974026, 
2.39043824701195, 3.73831775700935, 92.3809523809524, 4.90196078431373, 
4.68227424749164, 4.92957746478873, 2.38095238095238, 3.49854227405248, 
91.304347826087, 7.03703703703704, 0, 5.04587155963303, 93.5656836461126, 
8.29875518672199, 7.28476821192053, 3.44827586206897, 6.25, 84.6774193548387, 
92.4050632911392, 8.10810810810811, 91.8149466192171, 93.0131004366812, 
7.94701986754967, 0.465116279069767, 38.2222222222222, 5.42986425339367, 
6.80272108843537, 92.741935483871, 6.47773279352227, 83.3333333333333, 
1.6260162601626, 1.38888888888889, 0, 2.12765957446809, 1.74825174825175, 
0, 4.91803278688525, 3.80952380952381, 10.2702702702703, 92.1232876712329, 
6.56934306569343), C.perc = c(1.59362549800797, 1.2779552715655, 
4.60251046025105, 0.837520938023451, 85.981308411215, 2.92207792207792, 
0.509337860780985, 1.79948586118252, 86.8421052631579, 91.0979228486647, 
2.40963855421687, 0.793650793650794, 0.878734622144112, 88.6850152905199, 
88, 1.94805194805195, 0.416666666666667, 0, 2.12765957446809, 
1.60771704180064, 0, 1.25628140703518, 1.50943396226415, 2.1875, 
94.8616600790514, 3.25581395348837, 90.7849829351536, 5.65371024734982, 
1.88679245283019, 91.796875, 5.10204081632653, 92.2413793103448, 
4.39024390243902, 1.2987012987013, 1.99203187250996, 92.9906542056075, 
4.76190476190476, 93.1372549019608, 91.304347826087, 91.9014084507042, 
6.74603174603175, 3.49854227405248, 1.44927536231884, 88.8888888888889, 
94.4954128440367, 6.42201834862385, 1.07238605898123, 86.3070539419087, 
7.94701986754967, 1.25391849529781, 86.3636363636364, 6.45161290322581, 
0.843881856540084, 1.62162162162162, 0, 0, 2.64900662251656, 
0.930232558139535, 1.77777777777778, 90.4977375565611, 4.76190476190476, 
1.61290322580645, 89.0688259109312, 8.33333333333333, 3.2520325203252, 
93.0555555555556, 6.18181818181818, 92.7659574468085, 95.8041958041958, 
95.7831325301205, 4.91803278688525, 91.4285714285714, 7.56756756756757, 
2.05479452054795, 85.036496350365), G.perc = c(0.796812749003984, 
1.2779552715655, 92.0502092050209, 1.17252931323283, 3.73831775700935, 
3.57142857142857, 94.9066213921901, 91.0025706940874, 3.07017543859649, 
2.9673590504451, 4.41767068273092, 93.2539682539683, 1.75746924428822, 
3.05810397553517, 3.6, 91.2337662337662, 5, 93.1174089068826, 
4.60992907801418, 4.5016077170418, 4.47284345047923, 96.4824120603015, 
6.41509433962264, 94.0625, 0, 6.51162790697674, 1.70648464163823, 
0.706713780918728, 2.26415094339623, 1.953125, 4.59183673469388, 
3.01724137931034, 89.2682926829268, 96.1038961038961, 95.6175298804781, 
2.80373831775701, 0, 0, 2.0066889632107, 0.704225352112676, 3.57142857142857, 
89.5043731778426, 4.92753623188406, 3.33333333333333, 4.12844036697248, 
87.6146788990826, 3.48525469168901, 2.0746887966805, 82.7814569536424, 
95.2978056426332, 4.54545454545455, 2.41935483870968, 2.10970464135021, 
90.2702702702703, 4.27046263345196, 2.18340611353712, 82.7814569536424, 
2.7906976744186, 0.888888888888889, 0.452488687782805, 87.0748299319728, 
2.68817204301075, 1.21457489878543, 3.84615384615385, 91.869918699187, 
3.24074074074074, 1.09090909090909, 3.40425531914894, 0.34965034965035, 
4.21686746987952, 90.1639344262295, 4.76190476190476, 81.6216216216216, 
2.73972602739726, 1.45985401459854), T.perc = c(3.58565737051793, 
2.55591054313099, 0, 3.51758793969849, 3.42679127725857, 93.5064935064935, 
0.848896434634975, 0.51413881748072, 3.94736842105263, 2.07715133531157, 
0.803212851405622, 0.793650793650794, 3.33919156414763, 1.52905198776758, 
1.2, 1.62337662337662, 92.0833333333333, 2.42914979757085, 93.2624113475177, 
93.2475884244373, 95.5271565495208, 1.00502512562814, 92.0754716981132, 
2.5, 3.16205533596838, 6.51162790697674, 2.73037542662116, 91.8727915194346, 
3.0188679245283, 2.34375, 2.04081632653061, 2.1551724137931, 
2.92682926829268, 0, 0, 0.467289719626168, 2.85714285714286, 
1.96078431372549, 2.0066889632107, 2.46478873239437, 87.3015873015873, 
3.49854227405248, 2.31884057971015, 0.740740740740741, 1.37614678899083, 
0.917431192660551, 1.87667560321716, 3.3195020746888, 1.98675496688742, 
0, 2.84090909090909, 6.45161290322581, 4.64135021097046, 0, 3.91459074733096, 
4.80349344978166, 6.62251655629139, 95.8139534883721, 59.1111111111111, 
3.61990950226244, 1.36054421768707, 2.95698924731183, 3.23886639676113, 
4.48717948717949, 3.2520325203252, 2.31481481481481, 92.7272727272727, 
1.70212765957447, 2.0979020979021, 0, 0, 0, 0.540540540540541, 
3.08219178082192, 6.93430656934307), base.call = c("A", "A", 
"G", "A", "C", "T", "G", "G", "C", "C", "A", "G", "A", "C", "C", 
"G", "T", "G", "T", "T", "T", "G", "T", "G", "C", "A", "C", "T", 
"A", "C", "A", "C", "G", "G", "G", "C", "A", "C", "C", "C", "T", 
"G", "A", "C", "C", "G", "A", "C", "G", "G", "C", "A", "A", "G", 
"A", "A", "G", "T", "T", "C", "G", "A", "C", "A", "G", "C", "T", 
"C", "C", "C", "G", "C", "G", "A", "C"), index = 274:348, guide.seq = c("A", 
"A", "G", "A", "C", "T", "G", "G", "C", "C", "A", "G", "A", "C", 
"C", "G", "T", "G", "T", "T", "T", "G", "T", "G", "C", "A", "C", 
"T", "A", "C", "A", "C", "G", "G", "G", "C", "A", "C", "C", "C", 
"T", "G", "A", "C", "C", "G", "A", "C", "G", "G", "C", "A", "A", 
"G", "A", "A", "G", "T", "T", "C", "G", "A", "C", "A", "G", "C", 
"T", "C", "C", "C", "G", "C", "G", "A", "C"), T.pval = c(0.23432702614046, 
0.408361158072314, 0.893617021275807, 0.243610535676699, 0.256462363590746, 
0, 0.802526624881409, 0.859458034643274, 0.189821863575204, 0.513326738944468, 
0.811480511561419, 0.813314059764454, 0.269378836298132, 0.645774424626606, 
0.725404877043935, 0.622576872577941, 0, 0.434802507852448, 0, 
0, 0, 0.769795631672543, 0, 0.41989469708997, 0.297084112696876, 
0.036876102202575, 0.373721472380046, 0, 0.321061581442946, 0.453193407670361, 
0.521807776482112, 0.495331831367586, 0.337229710582315, 0.893617021275807, 
0.893617021275807, 0.865543139846389, 0.349865859633772, 0.540694441181371, 
0.529828030218353, 0.427263223081766, 0, 0.246261650324218, 0.458641861461324, 
0.823189514005884, 0.683202707935032, 0.788535309182768, 0.560815359157415, 
0.272352868788986, 0.534536033433526, 0.893617021275807, 0.352858292278406, 
0.0384002895902336, 0.124566003221161, 0.893617021275807, 0.193532999106852, 
0.112573450373199, 0.0342101242207512, 0, 0, 0.229767732052082, 
0.686992347529506, 0.331866269178894, 0.284806940159325, 0.13702481076376, 
0.282743186581797, 0.459525910105681, 0, 0.603246241342796, 0.508511271862925, 
0.893617021275807, 0.893617021275807, 0.893617021275807, 0.855797628954481, 
0.310281659308789, 0.0276639378809179), C.pval = c(0.578655252461248, 
0.674974158359026, 0.0659703288238527, 0.799651972685639, 0, 
0.24890794030689, 0.86871110876548, 0.517232126917918, 0, 0, 
0.35507712921785, 0.810472840594165, 0.789129938867004, 0, 0, 
0.474604584277077, 0.88227698347282, 0.905882352932355, 0.425577837471479, 
0.574386234238915, 0.905882352932355, 0.681531640734225, 0.60428634014214, 
0.409928089991095, 0, 0.194558151363257, 0, 0.0264664685954606, 
0.491974903753178, 0, 0.0430006157511054, 0, 0.0788290260699257, 
0.668679039546078, 0.462328342575358, 0, 0.0576241160451528, 
0, 0, 0, 0.00980812530851294, 0.161629527721611, 0.622684870763662, 
0, 0, 0.0132173609469187, 0.735932890226239, 0, 0.00317110313621916, 
0.68224523436296, 0, 0.0128636964491619, 0.798049756477276, 0.570181163866574, 
0.905882352932355, 0.905882352932355, 0.301935499151372, 0.775542281860474, 
0.523595661011496, 0, 0.0576241160451528, 0.572816935075099, 
0, 0.00219055514582334, 0.195113258336846, 0, 0.0164558474491011, 
0, 0, 0, 0.0504125962642964, 0, 0.00454691779065985, 0.445107545381247, 
0), G.pval = c(0.835951277219194, 0.727187559501012, 0, 0.751447055226774, 
0.278522337574057, 0.299493335409756, 0, 0, 0.370695864871071, 
0.386893951892229, 0.205851028981467, 0, 0.618670004737726, 0.372568890512555, 
0.29580963493975, 0, 0.157684914263125, 0, 0.188638042164762, 
0.198168200256155, 0.200771273156608, 0, 0.0807481911195925, 
0, 0.955555555555295, 0.0770744215314506, 0.62992279426291, 0.855112758199773, 
0.512701340263341, 0.576404547883728, 0.190200238128319, 0.37896588095927, 
0, 0, 0, 0.413824253704637, 0.955555555555295, 0.955555555555295, 
0.565112561993662, 0.855632230850605, 0.299493335409756, 0, 0.163055815674481, 
0.331737203376322, 0.234422770330139, 0, 0.310841996957554, 0.550963636838019, 
0, 0, 0.194259057666828, 0.482695570828829, 0.543761528925637, 
0, 0.219976795585085, 0.528793878668418, 0, 0.41603189984231, 
0.815778248055161, 0.904444269057378, 0, 0.433704549310002, 0.741776322848851, 
0.26565902946927, 0, 0.345039882640366, 0.770167204974754, 0.321840042955517, 
0.921487938467422, 0.225332006835139, 0, 0.175970473920781, 0, 
0.424747911639712, 0.685459989488015), A.pval = c(0, 0, 0.434742532099842, 
0, 0.0717068889233365, 0.926605504587155, 0.367439915527555, 
0.0791144550606139, 0.107749896150628, 0.347698453645698, 0, 
0.183477689434011, 0, 0.0771292263688842, 0.058530565065283, 
0.180043303291245, 0.601871206106474, 0.262323594207638, 0.926605504587155, 
0.909296471249448, 0.926605504587155, 0.840476896851525, 0.926605504587155, 
0.841426182483756, 0.709942109598447, 0, 0.223168806018515, 0.751363651994893, 
0, 0.340055019897548, 0, 0.584044599066797, 0.422556428838469, 
0.581736433560932, 0.624618357982492, 0.366918832579406, 0, 0.209546136677172, 
0.234207386299048, 0.206602562292073, 0.626590062447127, 0.407660705803344, 
0, 0.0644223723006051, 0.926605504587155, 0.194580329856017, 
0, 0.0301518645559009, 0.0556670309259022, 0.416545659314365, 
0.101314263817862, 0, 0, 0.0338947803741585, 0, 0, 0.0373949593755174, 
0.919158749397036, 1.17442722213923e-11, 0.158975774939763, 0.0738564343447129, 
0, 0.0890412010639441, 0, 0.77793639133293, 0.819473522113722, 
0.926605504587155, 0.679071686134686, 0.754928858787586, 0.926605504587155, 
0.207828893054057, 0.355357201747892, 0.00864848490209913, 0, 
0.084496184676642), guide.position = 1:75), .Names = c("A.area", 
"C.area", "G.area", "T.area", "Tot.area", "A.perc", "C.perc", 
"G.perc", "T.perc", "base.call", "index", "guide.seq", "T.pval", 
"C.pval", "G.pval", "A.pval", "guide.position"), row.names = 274:348, class = "data.frame")
```

*Report generated using EditR v1.0.8*
