# Supplementary material for: Mechanistic and genetic basis of single-strand templated repair at Cas12a-induced DNA breaks in Chlamydomonas reinhardtii
Source: Nat Commun. 2021 Nov 19;12:6751. doi: 10.1038/s41467-021-27004-1 (PMC8604939; doi:10.1038/s41467-021-27004-1)
Supplement: Supplementary file 22 — Source Data [file 41467_2021_27004_MOESM22_ESM.zip › Source Data/EditR analysis/EditR outputs/Sense/rep2_ssODN_sense_-16.html]

EditR v1.0.8 report


# EditR v1.0.8 report

- Data QA
  - Filtering data
  - Percent noise peak area
  - Base information
- Predicted editing
  - Editing bar plot
  - Editing table plot
  - Table of editing results
- For use in R

## Data QA

### Filtering data

What the data looked like prefiltering:

and the post filtering signal / noise plot:

### Percent noise peak area

### Base information

Here’s information about the signal of each base, the critical percent value where any higher value would be called as significant, and Filliben’s correlation for how well the noise was modelled by the zero adjusted gamma distribution.

| Base | Average percent signal | Average peak area | Critical percent value | model mu | Fillibens correlation |
| --- | --- | --- | --- | --- | --- |
| A | 93.79964 | 622.3750 | 7.484266 | 2.393552 | 0.9919664 |
| C | 91.84176 | 638.1014 | 6.814896 | 2.404525 | 0.9876826 |
| G | 93.77739 | 668.6034 | 7.018410 | 2.261873 | 0.9930012 |
| T | 94.27348 | 702.4762 | 8.230145 | 2.871793 | 0.9969218 |

## Predicted editing

### Editing bar plot

### Editing table plot

### Table of editing results


Here’s the entire guide region

| Sanger position | Guide position | Guide sequence | Sanger base call | Focal base | Focal base peak area | p value |  |
| --- | --- | --- | --- | --- | --- | --- | --- |
| 283 | 1 | A | A | A | 93.82 | 0.000000000 | \* |
| 283 | 1 | A | A | C | 1.50 | 0.621818566 |  |
| 283 | 1 | A | A | G | 2.34 | 0.366347506 |  |
| 283 | 1 | A | A | T | 2.34 | 0.484309384 |  |
| 284 | 2 | A | A | A | 93.28 | 0.000000000 | \* |
| 284 | 2 | A | A | C | 2.63 | 0.323282325 |  |
| 284 | 2 | A | A | G | 1.31 | 0.651470210 |  |
| 284 | 2 | A | A | T | 2.77 | 0.390083073 |  |
| 285 | 3 | G | G | A | 3.21 | 0.207475819 |  |
| 285 | 3 | G | G | C | 3.21 | 0.214690018 |  |
| 285 | 3 | G | G | G | 92.06 | 0.000000000 | \* |
| 285 | 3 | G | G | T | 1.52 | 0.679113828 |  |
| 286 | 4 | A | A | A | 95.02 | 0.000000000 | \* |
| 286 | 4 | A | A | C | 0.90 | 0.786082134 |  |
| 286 | 4 | A | A | G | 2.35 | 0.362727395 |  |
| 286 | 4 | A | A | T | 1.72 | 0.631366864 |  |
| 287 | 5 | C | C | A | 7.61 | 0.009118887 | \* |
| 287 | 5 | C | C | C | 87.32 | 0.000000000 | \* |
| 287 | 5 | C | C | G | 3.24 | 0.199636760 |  |
| 287 | 5 | C | C | T | 1.83 | 0.604350514 |  |
| 288 | 6 | T | T | A | 0.00 | 0.830357143 |  |
| 288 | 6 | T | T | C | 4.02 | 0.114958156 |  |
| 288 | 6 | T | T | G | 2.46 | 0.337817336 |  |
| 288 | 6 | T | T | T | 93.51 | 0.000000000 | \* |
| 289 | 7 | G | G | A | 2.25 | 0.368107744 |  |
| 289 | 7 | G | G | C | 0.00 | 0.890243902 |  |
| 289 | 7 | G | G | G | 96.78 | 0.000000000 | \* |
| 289 | 7 | G | G | T | 0.96 | 0.798769392 |  |
| 290 | 8 | G | G | A | 4.83 | 0.069928756 |  |
| 290 | 8 | G | G | C | 0.92 | 0.782349822 |  |
| 290 | 8 | G | G | G | 94.25 | 0.000000000 | \* |
| 290 | 8 | G | G | T | 0.00 | 0.884210526 |  |
| 291 | 9 | C | C | A | 6.16 | 0.026919317 |  |
| 291 | 9 | C | C | C | 87.06 | 0.000000000 | \* |
| 291 | 9 | C | C | G | 3.70 | 0.143479326 |  |
| 291 | 9 | C | C | T | 3.08 | 0.331268132 |  |
| 292 | 10 | C | C | A | 4.04 | 0.121007968 |  |
| 292 | 10 | C | C | C | 88.90 | 0.000000000 | \* |
| 292 | 10 | C | C | G | 3.40 | 0.177398712 |  |
| 292 | 10 | C | C | T | 3.66 | 0.238342271 |  |
| 293 | 11 | A | A | A | 93.11 | 0.000000000 | \* |
| 293 | 11 | A | A | C | 1.59 | 0.596068911 |  |
| 293 | 11 | A | A | G | 3.53 | 0.161614715 |  |
| 293 | 11 | A | A | T | 1.77 | 0.619909658 |  |
| 294 | 12 | G | G | A | 3.95 | 0.128177371 |  |
| 294 | 12 | G | G | C | 1.90 | 0.507659964 |  |
| 294 | 12 | G | G | G | 92.73 | 0.000000000 | \* |
| 294 | 12 | G | G | T | 1.42 | 0.702175161 |  |
| 295 | 13 | A | A | A | 96.00 | 0.000000000 | \* |
| 295 | 13 | A | A | C | 1.07 | 0.745164340 |  |
| 295 | 13 | A | A | G | 1.07 | 0.727645975 |  |
| 295 | 13 | A | A | T | 1.87 | 0.595303872 |  |
| 296 | 14 | C | C | A | 6.10 | 0.028081574 |  |
| 296 | 14 | C | C | C | 92.51 | 0.000000000 | \* |
| 296 | 14 | C | C | G | 0.00 | 0.932584270 |  |
| 296 | 14 | C | C | T | 1.39 | 0.710205102 |  |
| 297 | 15 | C | C | A | 0.00 | 0.830357143 |  |
| 297 | 15 | C | C | C | 95.63 | 0.000000000 | \* |
| 297 | 15 | C | C | G | 2.18 | 0.402807093 |  |
| 297 | 15 | C | C | T | 2.18 | 0.519578180 |  |
| 298 | 16 | G | G | A | 2.44 | 0.330792803 |  |
| 298 | 16 | G | G | C | 2.01 | 0.476224742 |  |
| 298 | 16 | G | G | G | 93.69 | 0.000000000 | \* |
| 298 | 16 | G | G | T | 1.87 | 0.596076668 |  |
| 299 | 17 | T | T | A | 3.35 | 0.189385173 |  |
| 299 | 17 | T | T | C | 0.99 | 0.766192817 |  |
| 299 | 17 | T | T | G | 2.76 | 0.277798987 |  |
| 299 | 17 | T | T | T | 92.90 | 0.000000000 | \* |
| 300 | 18 | G | G | A | 0.94 | 0.685028481 |  |
| 300 | 18 | G | G | C | 0.00 | 0.890243902 |  |
| 300 | 18 | G | G | G | 96.14 | 0.000000000 | \* |
| 300 | 18 | G | G | T | 2.92 | 0.360853418 |  |
| 301 | 19 | T | T | A | 1.50 | 0.541762905 |  |
| 301 | 19 | T | T | C | 1.20 | 0.708291086 |  |
| 301 | 19 | T | T | G | 4.66 | 0.068818216 |  |
| 301 | 19 | T | T | T | 92.63 | 0.000000000 | \* |
| 302 | 20 | T | T | A | 0.14 | 0.826113893 |  |
| 302 | 20 | T | T | C | 0.00 | 0.890243902 |  |
| 302 | 20 | T | T | G | 3.17 | 0.208993269 |  |
| 302 | 20 | T | T | T | 96.68 | 0.000000000 | \* |
| 303 | 21 | T | T | A | 0.00 | 0.830357143 |  |
| 303 | 21 | T | T | C | 1.86 | 0.518594946 |  |
| 303 | 21 | T | T | G | 2.43 | 0.345670354 |  |
| 303 | 21 | T | T | T | 95.71 | 0.000000000 | \* |
| 304 | 22 | G | G | A | 1.13 | 0.637633155 |  |
| 304 | 22 | G | G | C | 2.47 | 0.359510218 |  |
| 304 | 22 | G | G | G | 94.86 | 0.000000000 | \* |
| 304 | 22 | G | G | T | 1.54 | 0.674053169 |  |
| 305 | 23 | T | T | A | 0.68 | 0.747088592 |  |
| 305 | 23 | T | T | C | 3.21 | 0.214690018 |  |
| 305 | 23 | T | T | G | 2.36 | 0.359997753 |  |
| 305 | 23 | T | T | T | 93.75 | 0.000000000 | \* |
| 306 | 24 | G | G | A | 1.17 | 0.628580980 |  |
| 306 | 24 | G | G | C | 1.30 | 0.682137264 |  |
| 306 | 24 | G | G | G | 94.17 | 0.000000000 | \* |
| 306 | 24 | G | G | T | 3.37 | 0.282003966 |  |
| 307 | 25 | C | C | A | 1.81 | 0.466892621 |  |
| 307 | 25 | C | C | C | 87.50 | 0.000000000 | \* |
| 307 | 25 | C | C | G | 4.07 | 0.108823231 |  |
| 307 | 25 | C | C | T | 6.63 | 0.032870190 |  |
| 308 | 26 | A | A | A | 88.29 | 0.000000000 | \* |
| 308 | 26 | A | A | C | 3.07 | 0.237447530 |  |
| 308 | 26 | A | A | G | 5.18 | 0.045569310 |  |
| 308 | 26 | A | A | T | 3.45 | 0.268255747 |  |
| 309 | 27 | C | C | A | 3.96 | 0.127499050 |  |
| 309 | 27 | C | C | C | 83.11 | 0.000000000 | \* |
| 309 | 27 | C | C | G | 10.05 | 0.000715404 | \* |
| 309 | 27 | C | C | T | 2.89 | 0.366624648 |  |
| 310 | 28 | T | T | A | 1.47 | 0.549091084 |  |
| 310 | 28 | T | T | C | 3.54 | 0.167576022 |  |
| 310 | 28 | T | T | G | 0.00 | 0.932584270 |  |
| 310 | 28 | T | T | T | 94.99 | 0.000000000 | \* |
| 311 | 29 | A | A | A | 96.32 | 0.000000000 | \* |
| 311 | 29 | A | A | C | 2.17 | 0.431920797 |  |
| 311 | 29 | A | A | G | 0.00 | 0.932584270 |  |
| 311 | 29 | A | A | T | 1.51 | 0.682717109 |  |
| 312 | 30 | C | C | A | 2.77 | 0.271526548 |  |
| 312 | 30 | C | C | C | 91.97 | 0.000000000 | \* |
| 312 | 30 | C | C | G | 1.90 | 0.477984051 |  |
| 312 | 30 | C | C | T | 3.36 | 0.283652008 |  |
| 313 | 31 | A | A | A | 90.34 | 0.000000000 | \* |
| 313 | 31 | A | A | C | 2.96 | 0.257319401 |  |
| 313 | 31 | A | A | G | 4.93 | 0.055670954 |  |
| 313 | 31 | A | A | T | 1.78 | 0.617883321 |  |
| 314 | 32 | C | C | A | 2.27 | 0.364366708 |  |
| 314 | 32 | C | C | C | 92.87 | 0.000000000 | \* |
| 314 | 32 | C | C | G | 2.92 | 0.249894655 |  |
| 314 | 32 | C | C | T | 1.94 | 0.576792112 |  |
| 315 | 33 | G | G | A | 2.68 | 0.287142914 |  |
| 315 | 33 | G | G | C | 4.95 | 0.053204012 |  |
| 315 | 33 | G | G | G | 91.75 | 0.000000000 | \* |
| 315 | 33 | G | G | T | 0.62 | 0.851946248 |  |
| 316 | 34 | G | G | A | 0.26 | 0.816465226 |  |
| 316 | 34 | G | G | C | 1.56 | 0.604778895 |  |
| 316 | 34 | G | G | G | 96.36 | 0.000000000 | \* |
| 316 | 34 | G | G | T | 1.82 | 0.606880684 |  |
| 317 | 35 | G | G | A | 1.78 | 0.472729072 |  |
| 317 | 35 | G | G | C | 1.78 | 0.539882241 |  |
| 317 | 35 | G | G | G | 95.62 | 0.000000000 | \* |
| 317 | 35 | G | G | T | 0.81 | 0.825213878 |  |
| 318 | 36 | C | C | A | 3.27 | 0.199773349 |  |
| 318 | 36 | C | C | C | 95.00 | 0.000000000 | \* |
| 318 | 36 | C | C | G | 1.73 | 0.525289199 |  |
| 318 | 36 | C | C | T | 0.00 | 0.884210526 |  |
| 319 | 37 | A | A | A | 94.26 | 0.000000000 | \* |
| 319 | 37 | A | A | C | 3.15 | 0.224539492 |  |
| 319 | 37 | A | A | G | 0.00 | 0.932584270 |  |
| 319 | 37 | A | A | T | 2.59 | 0.427806224 |  |
| 320 | 38 | C | C | A | 3.43 | 0.180105343 |  |
| 320 | 38 | C | C | C | 93.14 | 0.000000000 | \* |
| 320 | 38 | C | C | G | 1.35 | 0.640866620 |  |
| 320 | 38 | C | C | T | 2.08 | 0.543588245 |  |
| 321 | 39 | C | C | A | 3.32 | 0.192919793 |  |
| 321 | 39 | C | C | C | 94.04 | 0.000000000 | \* |
| 321 | 39 | C | C | G | 2.63 | 0.302887957 |  |
| 321 | 39 | C | C | T | 0.00 | 0.884210526 |  |
| 322 | 40 | C | C | A | 3.89 | 0.133462426 |  |
| 322 | 40 | C | C | C | 92.08 | 0.000000000 | \* |
| 322 | 40 | C | C | G | 0.56 | 0.867721315 |  |
| 322 | 40 | C | C | T | 3.47 | 0.265581078 |  |
| 323 | 41 | T | T | A | 1.12 | 0.641013093 |  |
| 323 | 41 | T | T | C | 4.47 | 0.079713490 |  |
| 323 | 41 | T | T | G | 2.09 | 0.425461547 |  |
| 323 | 41 | T | T | T | 92.32 | 0.000000000 | \* |
| 324 | 42 | G | G | A | 1.86 | 0.455368933 |  |
| 324 | 42 | G | G | C | 2.84 | 0.279968873 |  |
| 324 | 42 | G | G | G | 93.34 | 0.000000000 | \* |
| 324 | 42 | G | G | T | 1.97 | 0.571928826 |  |
| 325 | 43 | A | A | A | 96.12 | 0.000000000 | \* |
| 325 | 43 | A | A | C | 1.50 | 0.622002752 |  |
| 325 | 43 | A | A | G | 2.38 | 0.357014561 |  |
| 325 | 43 | A | A | T | 0.00 | 0.884210526 |  |
| 326 | 44 | C | C | A | 4.87 | 0.068245231 |  |
| 326 | 44 | C | C | C | 91.52 | 0.000000000 | \* |
| 326 | 44 | C | C | G | 1.53 | 0.584990416 |  |
| 326 | 44 | C | C | T | 2.09 | 0.542898452 |  |
| 327 | 45 | C | C | A | 2.71 | 0.282621483 |  |
| 327 | 45 | C | C | C | 90.61 | 0.000000000 | \* |
| 327 | 45 | C | C | G | 3.03 | 0.231948477 |  |
| 327 | 45 | C | C | T | 3.66 | 0.237578041 |  |
| 328 | 46 | G | G | A | 2.64 | 0.293974957 |  |
| 328 | 46 | G | G | C | 3.17 | 0.221146950 |  |
| 328 | 46 | G | G | G | 92.08 | 0.000000000 | \* |
| 328 | 46 | G | G | T | 2.11 | 0.536614693 |  |
| 329 | 47 | A | A | A | 94.99 | 0.000000000 | \* |
| 329 | 47 | A | A | C | 1.00 | 0.762415683 |  |
| 329 | 47 | A | A | G | 1.78 | 0.511171579 |  |
| 329 | 47 | A | A | T | 2.22 | 0.510264589 |  |
| 330 | 48 | C | C | A | 5.03 | 0.060814864 |  |
| 330 | 48 | C | C | C | 89.78 | 0.000000000 | \* |
| 330 | 48 | C | C | G | 1.26 | 0.668873243 |  |
| 330 | 48 | C | C | T | 3.93 | 0.202159070 |  |
| 331 | 49 | G | G | A | 0.00 | 0.830357143 |  |
| 331 | 49 | G | G | C | 4.56 | 0.074099180 |  |
| 331 | 49 | G | G | G | 94.18 | 0.000000000 | \* |
| 331 | 49 | G | G | T | 1.27 | 0.737454986 |  |
| 332 | 50 | G | G | A | 1.48 | 0.548687838 |  |
| 332 | 50 | G | G | C | 0.67 | 0.835553380 |  |
| 332 | 50 | G | G | G | 97.85 | 0.000000000 | \* |
| 332 | 50 | G | G | T | 0.00 | 0.884210526 |  |
| 333 | 51 | C | C | A | 4.19 | 0.109296875 |  |
| 333 | 51 | C | C | C | 90.00 | 0.000000000 | \* |
| 333 | 51 | C | C | G | 3.49 | 0.167007127 |  |
| 333 | 51 | C | C | T | 2.33 | 0.486968805 |  |
| 334 | 52 | A | A | A | 93.55 | 0.000000000 | \* |
| 334 | 52 | A | A | C | 1.76 | 0.546606972 |  |
| 334 | 52 | A | A | G | 1.76 | 0.516976754 |  |
| 334 | 52 | A | A | T | 2.93 | 0.358783614 |  |
| 335 | 53 | A | A | A | 97.85 | 0.000000000 | \* |
| 335 | 53 | A | A | C | 0.33 | 0.880133646 |  |
| 335 | 53 | A | A | G | 1.32 | 0.648168327 |  |
| 335 | 53 | A | A | T | 0.50 | 0.864872294 |  |
| 336 | 54 | G | G | A | 4.94 | 0.064734135 |  |
| 336 | 54 | G | G | C | 1.71 | 0.560682330 |  |
| 336 | 54 | G | G | G | 92.21 | 0.000000000 | \* |
| 336 | 54 | G | G | T | 1.14 | 0.764229082 |  |
| 337 | 55 | A | A | A | 94.23 | 0.000000000 | \* |
| 337 | 55 | A | A | C | 0.00 | 0.890243902 |  |
| 337 | 55 | A | A | G | 2.66 | 0.296708044 |  |
| 337 | 55 | A | A | T | 3.11 | 0.326500558 |  |
| 338 | 56 | A | A | A | 94.33 | 0.000000000 | \* |
| 338 | 56 | A | A | C | 0.00 | 0.890243902 |  |
| 338 | 56 | A | A | G | 0.86 | 0.789522164 |  |
| 338 | 56 | A | A | T | 4.81 | 0.115603059 |  |
| 339 | 57 | G | G | A | 5.24 | 0.052473501 |  |
| 339 | 57 | G | G | C | 2.28 | 0.405257753 |  |
| 339 | 57 | G | G | G | 88.15 | 0.000000000 | \* |
| 339 | 57 | G | G | T | 4.33 | 0.157906815 |  |
| 340 | 58 | T | T | A | 4.09 | 0.117006585 |  |
| 340 | 58 | T | T | C | 1.24 | 0.696947673 |  |
| 340 | 58 | T | T | G | 1.24 | 0.673386259 |  |
| 340 | 58 | T | T | T | 93.43 | 0.000000000 | \* |
| 341 | 59 | T | T | A | 1.63 | 0.510389356 |  |
| 341 | 59 | T | T | C | 1.47 | 0.632596477 |  |
| 341 | 59 | T | T | G | 1.30 | 0.654867250 |  |
| 341 | 59 | T | T | T | 95.60 | 0.000000000 | \* |
| 342 | 60 | C | C | A | 3.77 | 0.144364756 |  |
| 342 | 60 | C | C | C | 92.13 | 0.000000000 | \* |
| 342 | 60 | C | C | G | 0.82 | 0.800667782 |  |
| 342 | 60 | C | C | T | 3.28 | 0.296645726 |  |
| 343 | 61 | G | G | A | 3.85 | 0.137309258 |  |
| 343 | 61 | G | G | C | 2.56 | 0.337269740 |  |
| 343 | 61 | G | G | G | 90.81 | 0.000000000 | \* |
| 343 | 61 | G | G | T | 2.78 | 0.389262812 |  |
| 344 | 62 | A | A | A | 93.29 | 0.000000000 | \* |
| 344 | 62 | A | A | C | 1.37 | 0.661610855 |  |
| 344 | 62 | A | A | G | 3.23 | 0.201002262 |  |
| 344 | 62 | A | A | T | 2.11 | 0.536822212 |  |
| 345 | 63 | C | C | A | 2.16 | 0.386166750 |  |
| 345 | 63 | C | C | C | 92.93 | 0.000000000 | \* |
| 345 | 63 | C | C | G | 1.88 | 0.484049464 |  |
| 345 | 63 | C | C | T | 3.03 | 0.340382105 |  |
| 346 | 64 | A | A | A | 89.98 | 0.000000000 | \* |
| 346 | 64 | A | A | C | 4.78 | 0.061229512 |  |
| 346 | 64 | A | A | G | 2.05 | 0.437074807 |  |
| 346 | 64 | A | A | T | 3.19 | 0.311924199 |  |
| 347 | 65 | G | G | A | 1.81 | 0.466692943 |  |
| 347 | 65 | G | G | C | 1.95 | 0.493234268 |  |
| 347 | 65 | G | G | G | 94.58 | 0.000000000 | \* |
| 347 | 65 | G | G | T | 1.67 | 0.643555693 |  |
| 348 | 66 | C | C | A | 2.39 | 0.341208168 |  |
| 348 | 66 | C | C | C | 92.50 | 0.000000000 | \* |
| 348 | 66 | C | C | G | 2.04 | 0.438596627 |  |
| 348 | 66 | C | C | T | 3.07 | 0.333748609 |  |
| 349 | 67 | T | T | A | 0.00 | 0.830357143 |  |
| 349 | 67 | T | T | C | 4.31 | 0.091057001 |  |
| 349 | 67 | T | T | G | 0.00 | 0.932584270 |  |
| 349 | 67 | T | T | T | 95.69 | 0.000000000 | \* |
| 350 | 68 | C | C | A | 1.79 | 0.471339399 |  |
| 350 | 68 | C | C | C | 95.45 | 0.000000000 | \* |
| 350 | 68 | C | C | G | 2.76 | 0.277260539 |  |
| 350 | 68 | C | C | T | 0.00 | 0.884210526 |  |
| 351 | 69 | C | C | A | 1.91 | 0.441844509 |  |
| 351 | 69 | C | C | C | 92.82 | 0.000000000 | \* |
| 351 | 69 | C | C | G | 0.24 | 0.920762235 |  |
| 351 | 69 | C | C | T | 5.02 | 0.100399079 |  |
| 352 | 70 | C | C | A | 4.20 | 0.108035412 |  |
| 352 | 70 | C | C | C | 90.54 | 0.000000000 | \* |
| 352 | 70 | C | C | G | 1.23 | 0.678797916 |  |
| 352 | 70 | C | C | T | 4.03 | 0.190462297 |  |
| 353 | 71 | G | G | A | 2.84 | 0.260708573 |  |
| 353 | 71 | G | G | C | 2.65 | 0.318163405 |  |
| 353 | 71 | G | G | G | 92.23 | 0.000000000 | \* |
| 353 | 71 | G | G | T | 2.27 | 0.499116888 |  |
| 354 | 72 | C | C | A | 5.98 | 0.030656834 |  |
| 354 | 72 | C | C | C | 90.26 | 0.000000000 | \* |
| 354 | 72 | C | C | G | 1.37 | 0.634830197 |  |
| 354 | 72 | C | C | T | 2.39 | 0.471631409 |  |
| 355 | 73 | G | G | A | 8.24 | 0.005622186 | \* |
| 355 | 73 | G | G | C | 5.94 | 0.022281415 |  |
| 355 | 73 | G | G | G | 85.44 | 0.000000000 | \* |
| 355 | 73 | G | G | T | 0.38 | 0.873862929 |  |
| 356 | 74 | A | A | A | 93.81 | 0.000000000 | \* |
| 356 | 74 | A | A | C | 1.83 | 0.526790811 |  |
| 356 | 74 | A | A | G | 2.39 | 0.354068048 |  |
| 356 | 74 | A | A | T | 1.97 | 0.570967137 |  |
| 357 | 75 | C | C | A | 4.20 | 0.108227047 |  |
| 357 | 75 | C | C | C | 89.57 | 0.000000000 | \* |
| 357 | 75 | C | C | G | 1.90 | 0.478202346 |  |
| 357 | 75 | C | C | T | 4.34 | 0.157106098 |  |

## For use in R

If you want to work with the results in R, here is output that you can copy and paste in your terminal to get:

The base information:

```
structure(list(focal.base = c("A", "C", "G", "T"), avg.percsignal = c(93.7996354970731, 
91.841762941827, 93.7773934982175, 94.2734815626338), avg.areasignal = c(622.375, 
638.101449275362, 668.603448275862, 702.47619047619), crit.perc.area = c(7.48426633746417, 
6.81489600972991, 7.01840981360755, 8.23014534666845), mu = c(2.39355187518832, 
2.40452483516025, 2.26187334594017, 2.87179294201626), fillibens = c(0.991966355487168, 
0.987682623693718, 0.993001188183663, 0.996921751331288)), .Names = c("focal.base", 
"avg.percsignal", "avg.areasignal", "crit.perc.area", "mu", "fillibens"
), row.names = c(NA, -4L), class = "data.frame")
```

the data.frame that contains information on the guide region:

```
structure(list(A.area = c(562, 639, 19, 1050, 54, 0, 28, 42, 
30, 32, 527, 25, 1079, 44, 0, 17, 17, 10, 10, 1, 0, 11, 4, 9, 
12, 460, 26, 10, 576, 19, 458, 14, 13, 2, 11, 17, 509, 28, 24, 
28, 8, 17, 768, 35, 17, 15, 854, 32, 0, 11, 18, 319, 591, 26, 
637, 549, 23, 23, 10, 23, 18, 751, 15, 395, 13, 14, 0, 11, 16, 
24, 15, 35, 43, 667, 31), C.area = c(9, 18, 19, 10, 620, 31, 
0, 8, 424, 705, 9, 12, 12, 667, 569, 14, 5, 0, 8, 0, 13, 24, 
19, 10, 581, 16, 546, 24, 13, 630, 15, 573, 24, 12, 11, 494, 
17, 760, 679, 663, 32, 26, 12, 658, 569, 18, 9, 571, 18, 5, 387, 
6, 2, 9, 0, 0, 10, 7, 9, 562, 12, 11, 644, 21, 14, 543, 33, 587, 
776, 517, 14, 528, 31, 13, 661), G.area = c(14, 9, 545, 26, 23, 
19, 1204, 819, 18, 27, 20, 587, 12, 0, 13, 653, 14, 1020, 31, 
22, 17, 923, 14, 727, 27, 27, 66, 0, 0, 13, 25, 18, 445, 741, 
590, 9, 0, 11, 19, 4, 15, 855, 19, 11, 19, 523, 16, 8, 372, 729, 
15, 6, 8, 485, 18, 5, 387, 7, 8, 5, 425, 26, 13, 9, 680, 12, 
0, 17, 2, 7, 487, 8, 446, 17, 14), T.area = c(14, 19, 9, 19, 
13, 721, 12, 0, 15, 29, 10, 9, 21, 10, 13, 13, 471, 31, 616, 
670, 670, 15, 555, 26, 44, 18, 19, 644, 9, 23, 9, 12, 3, 14, 
5, 0, 14, 17, 0, 25, 661, 18, 0, 15, 23, 12, 20, 25, 5, 0, 10, 
10, 3, 6, 21, 28, 19, 526, 587, 20, 13, 17, 21, 14, 12, 18, 733, 
0, 42, 23, 12, 14, 2, 14, 32), Tot.area = c(599, 685, 592, 1105, 
710, 771, 1244, 869, 487, 793, 566, 633, 1124, 721, 595, 697, 
507, 1061, 665, 693, 700, 973, 592, 772, 664, 521, 657, 678, 
598, 685, 507, 617, 485, 769, 617, 520, 540, 816, 722, 720, 716, 
916, 799, 719, 628, 568, 899, 636, 395, 745, 430, 341, 604, 526, 
676, 582, 439, 563, 614, 610, 468, 805, 693, 439, 719, 587, 766, 
615, 836, 571, 528, 585, 522, 711, 738), A.perc = c(93.8230383973289, 
93.2846715328467, 3.20945945945946, 95.0226244343891, 7.6056338028169, 
0, 2.2508038585209, 4.8331415420023, 6.16016427104723, 4.03530895334174, 
93.1095406360424, 3.94944707740916, 95.9964412811388, 6.10263522884882, 
0, 2.4390243902439, 3.35305719921105, 0.942507068803016, 1.50375939849624, 
0.144300144300144, 0, 1.13052415210689, 0.675675675675676, 1.16580310880829, 
1.80722891566265, 88.2917466410749, 3.95738203957382, 1.47492625368732, 
96.3210702341137, 2.77372262773723, 90.3353057199211, 2.26904376012966, 
2.68041237113402, 0.260078023407022, 1.78282009724473, 3.26923076923077, 
94.2592592592593, 3.43137254901961, 3.32409972299169, 3.88888888888889, 
1.11731843575419, 1.85589519650655, 96.1201501877347, 4.86787204450626, 
2.70700636942675, 2.64084507042254, 94.9944382647386, 5.0314465408805, 
0, 1.47651006711409, 4.18604651162791, 93.5483870967742, 97.8476821192053, 
4.94296577946768, 94.2307692307692, 94.3298969072165, 5.23917995444191, 
4.08525754884547, 1.62866449511401, 3.77049180327869, 3.84615384615385, 
93.2919254658385, 2.16450216450216, 89.9772209567198, 1.80806675938804, 
2.38500851788756, 0, 1.78861788617886, 1.91387559808612, 4.2031523642732, 
2.84090909090909, 5.98290598290598, 8.23754789272031, 93.8115330520394, 
4.20054200542005), C.perc = c(1.5025041736227, 2.62773722627737, 
3.20945945945946, 0.904977375565611, 87.3239436619718, 4.02075226977951, 
0, 0.920598388952819, 87.0636550308008, 88.9029003783102, 1.59010600706714, 
1.8957345971564, 1.06761565836299, 92.5104022191401, 95.6302521008403, 
2.00860832137733, 0.986193293885602, 0, 1.20300751879699, 0, 
1.85714285714286, 2.46659815005139, 3.20945945945946, 1.29533678756477, 
87.5, 3.07101727447217, 83.1050228310502, 3.53982300884956, 2.17391304347826, 
91.970802919708, 2.9585798816568, 92.8687196110211, 4.94845360824742, 
1.56046814044213, 1.78282009724473, 95, 3.14814814814815, 93.1372549019608, 
94.0443213296399, 92.0833333333333, 4.46927374301676, 2.83842794759825, 
1.50187734668335, 91.5159944367177, 90.6050955414013, 3.16901408450704, 
1.00111234705228, 89.7798742138365, 4.55696202531646, 0.671140939597315, 
90, 1.75953079178886, 0.33112582781457, 1.71102661596958, 0, 
0, 2.27790432801822, 1.24333925399645, 1.46579804560261, 92.1311475409836, 
2.56410256410256, 1.36645962732919, 92.9292929292929, 4.78359908883827, 
1.9471488178025, 92.5042589437819, 4.30809399477807, 95.4471544715447, 
92.822966507177, 90.5429071803853, 2.65151515151515, 90.2564102564103, 
5.93869731800766, 1.82841068917018, 89.5663956639566), G.perc = c(2.33722871452421, 
1.31386861313869, 92.0608108108108, 2.35294117647059, 3.23943661971831, 
2.46433203631647, 96.7845659163987, 94.2462600690449, 3.69609856262834, 
3.40479192938209, 3.53356890459364, 92.7330173775671, 1.06761565836299, 
0, 2.18487394957983, 93.687230989957, 2.76134122287968, 96.1357210179076, 
4.66165413533835, 3.17460317460317, 2.42857142857143, 94.8612538540596, 
2.36486486486486, 94.1709844559585, 4.06626506024096, 5.18234165067178, 
10.0456621004566, 0, 0, 1.8978102189781, 4.93096646942801, 2.91734197730956, 
91.7525773195876, 96.3589076723017, 95.6239870340357, 1.73076923076923, 
0, 1.34803921568627, 2.63157894736842, 0.555555555555556, 2.09497206703911, 
93.3406113537118, 2.37797246558198, 1.52990264255911, 3.02547770700637, 
92.0774647887324, 1.7797552836485, 1.25786163522013, 94.1772151898734, 
97.8523489932886, 3.48837209302326, 1.75953079178886, 1.32450331125828, 
92.2053231939164, 2.66272189349112, 0.859106529209622, 88.1548974943052, 
1.24333925399645, 1.30293159609121, 0.819672131147541, 90.8119658119658, 
3.22981366459627, 1.87590187590188, 2.0501138952164, 94.5757997218359, 
2.0442930153322, 0, 2.76422764227642, 0.239234449760766, 1.22591943957968, 
92.2348484848485, 1.36752136752137, 85.4406130268199, 2.39099859353024, 
1.8970189701897), T.perc = c(2.33722871452421, 2.77372262773723, 
1.52027027027027, 1.71945701357466, 1.83098591549296, 93.514915693904, 
0.964630225080386, 0, 3.08008213552361, 3.65699873896595, 1.76678445229682, 
1.4218009478673, 1.86832740213523, 1.3869625520111, 2.18487394957983, 
1.86513629842181, 92.8994082840237, 2.92177191328935, 92.6315789473684, 
96.6810966810967, 95.7142857142857, 1.54162384378212, 93.75, 
3.36787564766839, 6.62650602409639, 3.45489443378119, 2.89193302891933, 
94.9852507374631, 1.50501672240803, 3.35766423357664, 1.77514792899408, 
1.94489465153971, 0.618556701030928, 1.82054616384915, 0.810372771474878, 
0, 2.59259259259259, 2.08333333333333, 0, 3.47222222222222, 92.3184357541899, 
1.96506550218341, 0, 2.08623087621697, 3.6624203821656, 2.11267605633803, 
2.22469410456062, 3.93081761006289, 1.26582278481013, 0, 2.32558139534884, 
2.93255131964809, 0.496688741721854, 1.14068441064639, 3.10650887573965, 
4.81099656357388, 4.32801822323462, 93.4280639431616, 95.6026058631922, 
3.27868852459016, 2.77777777777778, 2.11180124223602, 3.03030303030303, 
3.18906605922551, 1.66898470097357, 3.0664395229983, 95.6919060052219, 
0, 5.02392344497608, 4.02802101576182, 2.27272727272727, 2.39316239316239, 
0.383141762452107, 1.9690576652602, 4.3360433604336), base.call = c("A", 
"A", "G", "A", "C", "T", "G", "G", "C", "C", "A", "G", "A", "C", 
"C", "G", "T", "G", "T", "T", "T", "G", "T", "G", "C", "A", "C", 
"T", "A", "C", "A", "C", "G", "G", "G", "C", "A", "C", "C", "C", 
"T", "G", "A", "C", "C", "G", "A", "C", "G", "G", "C", "A", "A", 
"G", "A", "A", "G", "T", "T", "C", "G", "A", "C", "A", "G", "C", 
"T", "C", "C", "C", "G", "C", "G", "A", "C"), index = 283:357, 
    guide.seq = c("A", "A", "G", "A", "C", "T", "G", "G", "C", 
    "C", "A", "G", "A", "C", "C", "G", "T", "G", "T", "T", "T", 
    "G", "T", "G", "C", "A", "C", "T", "A", "C", "A", "C", "G", 
    "G", "G", "C", "A", "C", "C", "C", "T", "G", "A", "C", "C", 
    "G", "A", "C", "G", "G", "C", "A", "A", "G", "A", "A", "G", 
    "T", "T", "C", "G", "A", "C", "A", "G", "C", "T", "C", "C", 
    "C", "G", "C", "G", "A", "C"), T.pval = c(0.484309384453299, 
    0.390083072974634, 0.679113828235155, 0.631366863756145, 
    0.604350514214325, 0, 0.798769392270438, 0.884210526315668, 
    0.331268131732867, 0.238342270919896, 0.619909658278294, 
    0.702175161120306, 0.595303871810043, 0.710205101639301, 
    0.519578179583431, 0.596076667584972, 0, 0.360853418319237, 
    0, 0, 0, 0.67405316891583, 0, 0.282003965879082, 0.032870189604112, 
    0.268255747121068, 0.366624648379382, 0, 0.682717108894218, 
    0.283652008218945, 0.617883321346309, 0.576792111670589, 
    0.851946248461241, 0.60688068357404, 0.825213877542639, 0.884210526315668, 
    0.427806223610042, 0.543588244732893, 0.884210526315668, 
    0.265581077868513, 0, 0.571928825752028, 0.884210526315668, 
    0.542898451992005, 0.237578040861372, 0.536614693254033, 
    0.510264588826008, 0.202159070055254, 0.737454986069562, 
    0.884210526315668, 0.486968804921067, 0.358783613502531, 
    0.864872294001287, 0.764229082429306, 0.326500557539609, 
    0.115603059495922, 0.15790681468988, 0, 0, 0.296645726377222, 
    0.389262812178075, 0.536822211880466, 0.340382104973111, 
    0.311924199181733, 0.643555692940409, 0.333748608527584, 
    0, 0.884210526315668, 0.100399079090341, 0.190462297125258, 
    0.49911688787516, 0.47163140923271, 0.873862928649906, 0.570967137275586, 
    0.157106098027737), C.pval = c(0.621818565770536, 0.323282324886746, 
    0.214690018185977, 0.786082134047147, 0, 0.114958156411832, 
    0.89024390243862, 0.782349822205981, 0, 0, 0.59606891128952, 
    0.507659963810365, 0.745164340267167, 0, 0, 0.476224742269045, 
    0.766192817167948, 0.89024390243862, 0.7082910862968, 0.89024390243862, 
    0.518594946439634, 0.359510217857739, 0.214690018185977, 
    0.682137263857365, 0, 0.23744752970913, 0, 0.167576022042255, 
    0.431920796949792, 0, 0.257319400590611, 0, 0.0532040117316883, 
    0.604778894737479, 0.539882241254346, 0, 0.224539492152484, 
    0, 0, 0, 0.0797134897423241, 0.279968873416394, 0.622002752002741, 
    0, 0, 0.221146950028713, 0.762415683345698, 0, 0.074099180073071, 
    0.835553380458475, 0, 0.546606971721357, 0.880133645922616, 
    0.560682329594401, 0.89024390243862, 0.89024390243862, 0.405257753153369, 
    0.696947672758065, 0.632596476963815, 0, 0.337269739745507, 
    0.661610855403151, 0, 0.0612295117200709, 0.493234268237727, 
    0, 0.0910570006758818, 0, 0, 0, 0.318163405051408, 0, 0.022281415225108, 
    0.526790811468356, 0), G.pval = c(0.366347505982902, 0.651470210234778, 
    0, 0.362727394654685, 0.1996367598799, 0.337817336140617, 
    0, 0, 0.143479326074871, 0.177398711974086, 0.161614714602583, 
    0, 0.727645975367429, 0.932584269662921, 0.402807092939154, 
    0, 0.277798987128743, 0, 0.0688182159031641, 0.208993268553124, 
    0.345670353746353, 0, 0.35999775338613, 0, 0.108823230731348, 
    0.0455693097630727, 0.000715403973835183, 0.932584269662921, 
    0.932584269662921, 0.4779840508733, 0.0556709540463124, 0.249894654669842, 
    0, 0, 0, 0.52528919941424, 0.932584269662921, 0.640866619525439, 
    0.302887957430189, 0.867721314844933, 0.425461547046549, 
    0, 0.357014560897953, 0.584990416011895, 0.231948477096638, 
    0, 0.511171578989171, 0.668873243179709, 0, 0, 0.16700712728948, 
    0.516976754470768, 0.648168326891306, 0, 0.296708044243105, 
    0.789522164232556, 0, 0.673386259386788, 0.654867250133281, 
    0.800667781980448, 0, 0.201002261705864, 0.484049464118376, 
    0.437074807434985, 0, 0.438596627414201, 0.932584269662921, 
    0.277260538662737, 0.92076223493444, 0.678797915875195, 0, 
    0.634830196539328, 0, 0.354068047985446, 0.478202345937556
    ), A.pval = c(0, 0, 0.207475818675819, 0, 0.00911888663385074, 
    0.830357142857142, 0.368107744030727, 0.0699287558072321, 
    0.026919316912801, 0.121007968084068, 0, 0.12817737149267, 
    0, 0.0280815739926137, 0.830357142857142, 0.330792803037647, 
    0.18938517284039, 0.685028481019306, 0.541762904759825, 0.826113893002893, 
    0.830357142857142, 0.637633154754809, 0.747088591694892, 
    0.628580979818991, 0.466892620599122, 0, 0.127499050378297, 
    0.549091084332852, 0, 0.271526548146171, 0, 0.36436670820843, 
    0.287142914061052, 0.816465225555005, 0.472729071778177, 
    0.199773348633657, 0, 0.180105343315135, 0.192919792560501, 
    0.133462425909938, 0.641013092754616, 0.455368932628875, 
    0, 0.0682452306345371, 0.282621483137898, 0.293974957091383, 
    0, 0.0608148639936616, 0.830357142857142, 0.548687838398508, 
    0.109296875338672, 0, 0, 0.0647341350189983, 0, 0, 0.0524735009310089, 
    0.117006585459682, 0.510389355651126, 0.144364756120749, 
    0.137309257837334, 0, 0.386166749716961, 0, 0.466692943092365, 
    0.341208167989569, 0.830357142857142, 0.471339398672179, 
    0.44184450882224, 0.108035411822746, 0.260708573394231, 0.0306568336058017, 
    0.00562218639985512, 0, 0.108227046597222), guide.position = 1:75), .Names = c("A.area", 
"C.area", "G.area", "T.area", "Tot.area", "A.perc", "C.perc", 
"G.perc", "T.perc", "base.call", "index", "guide.seq", "T.pval", 
"C.pval", "G.pval", "A.pval", "guide.position"), row.names = 283:357, class = "data.frame")
```

*Report generated using EditR v1.0.8*
