# Supplementary material for: Mechanistic and genetic basis of single-strand templated repair at Cas12a-induced DNA breaks in Chlamydomonas reinhardtii
Source: Nat Commun. 2021 Nov 19;12:6751. doi: 10.1038/s41467-021-27004-1 (PMC8604939; doi:10.1038/s41467-021-27004-1)
Supplement: Supplementary file 22 — Source Data [file 41467_2021_27004_MOESM22_ESM.zip › Source Data/EditR analysis/EditR outputs/Sense/rep2_ssODN_sense_32.html]

EditR v1.0.8 report


# EditR v1.0.8 report

- Data QA
  - Filtering data
  - Percent noise peak area
  - Base information
- Predicted editing
  - Editing bar plot
  - Editing table plot
  - Table of editing results
- For use in R

## Data QA

### Filtering data

What the data looked like prefiltering:

and the post filtering signal / noise plot:

### Percent noise peak area

### Base information

Here’s information about the signal of each base, the critical percent value where any higher value would be called as significant, and Filliben’s correlation for how well the noise was modelled by the zero adjusted gamma distribution.

| Base | Average percent signal | Average peak area | Critical percent value | model mu | Fillibens correlation |
| --- | --- | --- | --- | --- | --- |
| A | 91.56015 | 242.5455 | 13.329153 | 3.952936 | 0.9943743 |
| C | 90.26388 | 259.0290 | 8.562914 | 2.672194 | 0.9370799 |
| G | 90.19924 | 249.0536 | 8.604998 | 2.972840 | 0.9956084 |
| T | 93.92444 | 290.7442 | 10.167597 | 3.358332 | 0.9946211 |

## Predicted editing

### Editing bar plot

### Editing table plot

### Table of editing results


Here’s the entire guide region

| Sanger position | Guide position | Guide sequence | Sanger base call | Focal base | Focal base peak area | p value |  |
| --- | --- | --- | --- | --- | --- | --- | --- |
| 280 | 1 | A | A | A | 98.60 | 0.000000e+00 | \* |
| 280 | 1 | A | A | C | 1.40 | 6.541671e-01 |  |
| 280 | 1 | A | A | G | 0.00 | 9.120879e-01 |  |
| 280 | 1 | A | A | T | 0.00 | 9.042553e-01 |  |
| 281 | 2 | A | A | A | 92.47 | 0.000000e+00 | \* |
| 281 | 2 | A | A | C | 2.51 | 3.994818e-01 |  |
| 281 | 2 | A | A | G | 2.87 | 4.025799e-01 |  |
| 281 | 2 | A | A | T | 2.15 | 5.979963e-01 |  |
| 282 | 3 | G | G | A | 2.99 | 4.266485e-01 |  |
| 282 | 3 | G | G | C | 6.97 | 2.867242e-02 |  |
| 282 | 3 | G | G | G | 90.05 | 0.000000e+00 | \* |
| 282 | 3 | G | G | T | 0.00 | 9.042553e-01 |  |
| 283 | 4 | A | A | A | 95.50 | 0.000000e+00 | \* |
| 283 | 4 | A | A | C | 1.02 | 7.477498e-01 |  |
| 283 | 4 | A | A | G | 0.41 | 9.000790e-01 |  |
| 283 | 4 | A | A | T | 3.07 | 4.227977e-01 |  |
| 284 | 5 | C | C | A | 7.46 | 9.420662e-02 |  |
| 284 | 5 | C | C | C | 89.55 | 0.000000e+00 | \* |
| 284 | 5 | C | C | G | 1.12 | 7.989581e-01 |  |
| 284 | 5 | C | C | T | 1.87 | 6.563279e-01 |  |
| 285 | 6 | T | T | A | 0.00 | 7.927928e-01 |  |
| 285 | 6 | T | T | C | 1.53 | 6.229983e-01 |  |
| 285 | 6 | T | T | G | 3.05 | 3.663692e-01 |  |
| 285 | 6 | T | T | T | 95.42 | 0.000000e+00 | \* |
| 286 | 7 | G | G | A | 3.56 | 3.588266e-01 |  |
| 286 | 7 | G | G | C | 0.79 | 8.008729e-01 |  |
| 286 | 7 | G | G | G | 94.86 | 0.000000e+00 | \* |
| 286 | 7 | G | G | T | 0.79 | 8.514011e-01 |  |
| 287 | 8 | G | G | A | 6.02 | 1.577735e-01 |  |
| 287 | 8 | G | G | C | 2.71 | 3.608933e-01 |  |
| 287 | 8 | G | G | G | 89.46 | 0.000000e+00 | \* |
| 287 | 8 | G | G | T | 1.81 | 6.682870e-01 |  |
| 288 | 9 | C | C | A | 3.38 | 3.787649e-01 |  |
| 288 | 9 | C | C | C | 85.51 | 0.000000e+00 | \* |
| 288 | 9 | C | C | G | 5.31 | 9.713651e-02 |  |
| 288 | 9 | C | C | T | 5.80 | 1.160579e-01 |  |
| 289 | 10 | C | C | A | 2.31 | 5.164750e-01 |  |
| 289 | 10 | C | C | C | 90.43 | 0.000000e+00 | \* |
| 289 | 10 | C | C | G | 3.30 | 3.218564e-01 |  |
| 289 | 10 | C | C | T | 3.96 | 2.865210e-01 |  |
| 290 | 11 | A | A | A | 89.96 | 0.000000e+00 | \* |
| 290 | 11 | A | A | C | 2.18 | 4.675585e-01 |  |
| 290 | 11 | A | A | G | 2.62 | 4.540681e-01 |  |
| 290 | 11 | A | A | T | 5.24 | 1.544163e-01 |  |
| 291 | 12 | G | G | A | 4.50 | 2.649272e-01 |  |
| 291 | 12 | G | G | C | 1.35 | 6.668015e-01 |  |
| 291 | 12 | G | G | G | 91.89 | 0.000000e+00 | \* |
| 291 | 12 | G | G | T | 2.25 | 5.773552e-01 |  |
| 292 | 13 | A | A | A | 94.56 | 0.000000e+00 | \* |
| 292 | 13 | A | A | C | 1.01 | 7.511905e-01 |  |
| 292 | 13 | A | A | G | 0.81 | 8.548773e-01 |  |
| 292 | 13 | A | A | T | 3.63 | 3.326323e-01 |  |
| 293 | 14 | C | C | A | 5.24 | 2.066544e-01 |  |
| 293 | 14 | C | C | C | 89.86 | 0.000000e+00 | \* |
| 293 | 14 | C | C | G | 1.40 | 7.390344e-01 |  |
| 293 | 14 | C | C | T | 3.50 | 3.525555e-01 |  |
| 294 | 15 | C | C | A | 2.36 | 5.097511e-01 |  |
| 294 | 15 | C | C | C | 91.98 | 0.000000e+00 | \* |
| 294 | 15 | C | C | G | 1.89 | 6.236627e-01 |  |
| 294 | 15 | C | C | T | 3.77 | 3.118687e-01 |  |
| 295 | 16 | G | G | A | 4.82 | 2.386227e-01 |  |
| 295 | 16 | G | G | C | 1.61 | 6.032266e-01 |  |
| 295 | 16 | G | G | G | 91.16 | 0.000000e+00 | \* |
| 295 | 16 | G | G | T | 2.41 | 5.458279e-01 |  |
| 296 | 17 | T | T | A | 2.63 | 4.725091e-01 |  |
| 296 | 17 | T | T | C | 0.00 | 9.024390e-01 |  |
| 296 | 17 | T | T | G | 2.19 | 5.506261e-01 |  |
| 296 | 17 | T | T | T | 95.18 | 0.000000e+00 | \* |
| 297 | 18 | G | G | A | 0.49 | 7.636985e-01 |  |
| 297 | 18 | G | G | C | 0.00 | 9.024390e-01 |  |
| 297 | 18 | G | G | G | 97.33 | 0.000000e+00 | \* |
| 297 | 18 | G | G | T | 2.18 | 5.910926e-01 |  |
| 298 | 19 | T | T | A | 1.15 | 6.821807e-01 |  |
| 298 | 19 | T | T | C | 0.00 | 9.024390e-01 |  |
| 298 | 19 | T | T | G | 4.62 | 1.507103e-01 |  |
| 298 | 19 | T | T | T | 94.23 | 0.000000e+00 | \* |
| 299 | 20 | T | T | A | 0.00 | 7.927928e-01 |  |
| 299 | 20 | T | T | C | 0.00 | 9.024390e-01 |  |
| 299 | 20 | T | T | G | 3.21 | 3.369047e-01 |  |
| 299 | 20 | T | T | T | 96.79 | 0.000000e+00 | \* |
| 300 | 21 | T | T | A | 0.35 | 7.758921e-01 |  |
| 300 | 21 | T | T | C | 2.09 | 4.882146e-01 |  |
| 300 | 21 | T | T | G | 1.74 | 6.584409e-01 |  |
| 300 | 21 | T | T | T | 95.82 | 0.000000e+00 | \* |
| 301 | 22 | G | G | A | 4.26 | 2.874394e-01 |  |
| 301 | 22 | G | G | C | 0.91 | 7.737236e-01 |  |
| 301 | 22 | G | G | G | 93.62 | 0.000000e+00 | \* |
| 301 | 22 | G | G | T | 1.22 | 7.837597e-01 |  |
| 302 | 23 | T | T | A | 2.07 | 5.498019e-01 |  |
| 302 | 23 | T | T | C | 0.83 | 7.922469e-01 |  |
| 302 | 23 | T | T | G | 2.90 | 3.951645e-01 |  |
| 302 | 23 | T | T | T | 94.19 | 0.000000e+00 | \* |
| 303 | 24 | G | G | A | 5.69 | 1.770491e-01 |  |
| 303 | 24 | G | G | C | 1.78 | 5.609631e-01 |  |
| 303 | 24 | G | G | G | 88.61 | 0.000000e+00 | \* |
| 303 | 24 | G | G | T | 3.91 | 2.925822e-01 |  |
| 304 | 25 | C | C | A | 2.51 | 4.888482e-01 |  |
| 304 | 25 | C | C | C | 91.21 | 0.000000e+00 | \* |
| 304 | 25 | C | C | G | 1.67 | 6.748349e-01 |  |
| 304 | 25 | C | C | T | 4.60 | 2.116863e-01 |  |
| 305 | 26 | A | A | A | 80.93 | 3.330669e-15 | \* |
| 305 | 26 | A | A | C | 3.09 | 2.956532e-01 |  |
| 305 | 26 | A | A | G | 6.70 | 3.854594e-02 |  |
| 305 | 26 | A | A | T | 9.28 | 1.683937e-02 |  |
| 306 | 27 | C | C | A | 4.17 | 2.958189e-01 |  |
| 306 | 27 | C | C | C | 88.64 | 0.000000e+00 | \* |
| 306 | 27 | C | C | G | 4.55 | 1.572968e-01 |  |
| 306 | 27 | C | C | T | 2.65 | 4.987239e-01 |  |
| 307 | 28 | T | T | A | 3.04 | 4.195574e-01 |  |
| 307 | 28 | T | T | C | 2.28 | 4.463387e-01 |  |
| 307 | 28 | T | T | G | 4.56 | 1.556460e-01 |  |
| 307 | 28 | T | T | T | 90.11 | 0.000000e+00 | \* |
| 308 | 29 | A | A | A | 93.36 | 0.000000e+00 | \* |
| 308 | 29 | A | A | C | 2.49 | 4.033257e-01 |  |
| 308 | 29 | A | A | G | 2.07 | 5.786222e-01 |  |
| 308 | 29 | A | A | T | 2.07 | 6.134828e-01 |  |
| 309 | 30 | C | C | A | 3.51 | 3.642566e-01 |  |
| 309 | 30 | C | C | C | 92.54 | 0.000000e+00 | \* |
| 309 | 30 | C | C | G | 0.88 | 8.435690e-01 |  |
| 309 | 30 | C | C | T | 3.07 | 4.223305e-01 |  |
| 310 | 31 | A | A | A | 84.97 | 6.661338e-16 | \* |
| 310 | 31 | A | A | C | 1.73 | 5.719210e-01 |  |
| 310 | 31 | A | A | G | 8.67 | 9.534158e-03 | \* |
| 310 | 31 | A | A | T | 4.62 | 2.094666e-01 |  |
| 311 | 32 | C | C | A | 0.48 | 7.643717e-01 |  |
| 311 | 32 | C | C | C | 91.87 | 0.000000e+00 | \* |
| 311 | 32 | C | C | G | 2.39 | 5.045188e-01 |  |
| 311 | 32 | C | C | T | 5.26 | 1.526338e-01 |  |
| 312 | 33 | G | G | A | 7.78 | 8.393692e-02 |  |
| 312 | 33 | G | G | C | 1.67 | 5.883909e-01 |  |
| 312 | 33 | G | G | G | 86.11 | 0.000000e+00 | \* |
| 312 | 33 | G | G | T | 4.44 | 2.284075e-01 |  |
| 313 | 34 | G | G | A | 2.95 | 4.308133e-01 |  |
| 313 | 34 | G | G | C | 3.32 | 2.614031e-01 |  |
| 313 | 34 | G | G | G | 92.25 | 0.000000e+00 | \* |
| 313 | 34 | G | G | T | 1.48 | 7.347819e-01 |  |
| 314 | 35 | G | G | A | 5.86 | 1.673458e-01 |  |
| 314 | 35 | G | G | C | 1.80 | 5.555599e-01 |  |
| 314 | 35 | G | G | G | 91.89 | 0.000000e+00 | \* |
| 314 | 35 | G | G | T | 0.45 | 8.879433e-01 |  |
| 315 | 36 | C | C | A | 5.37 | 1.982700e-01 |  |
| 315 | 36 | C | C | C | 90.73 | 0.000000e+00 | \* |
| 315 | 36 | C | C | G | 3.90 | 2.303492e-01 |  |
| 315 | 36 | C | C | T | 0.00 | 9.042553e-01 |  |
| 316 | 37 | A | A | A | 94.33 | 0.000000e+00 | \* |
| 316 | 37 | A | A | C | 2.58 | 3.860978e-01 |  |
| 316 | 37 | A | A | G | 0.00 | 9.120879e-01 |  |
| 316 | 37 | A | A | T | 3.09 | 4.184179e-01 |  |
| 317 | 38 | C | C | A | 3.30 | 3.886888e-01 |  |
| 317 | 38 | C | C | C | 93.41 | 0.000000e+00 | \* |
| 317 | 38 | C | C | G | 0.00 | 9.120879e-01 |  |
| 317 | 38 | C | C | T | 3.30 | 3.841890e-01 |  |
| 318 | 39 | C | C | A | 4.96 | 2.274649e-01 |  |
| 318 | 39 | C | C | C | 86.26 | 0.000000e+00 | \* |
| 318 | 39 | C | C | G | 4.58 | 1.539980e-01 |  |
| 318 | 39 | C | C | T | 4.20 | 2.566024e-01 |  |
| 319 | 40 | C | C | A | 5.86 | 1.672357e-01 |  |
| 319 | 40 | C | C | C | 91.21 | 0.000000e+00 | \* |
| 319 | 40 | C | C | G | 0.00 | 9.120879e-01 |  |
| 319 | 40 | C | C | T | 2.93 | 4.472988e-01 |  |
| 320 | 41 | T | T | A | 5.19 | 2.102027e-01 |  |
| 320 | 41 | T | T | C | 3.46 | 2.417814e-01 |  |
| 320 | 41 | T | T | G | 3.46 | 2.947373e-01 |  |
| 320 | 41 | T | T | T | 87.88 | 0.000000e+00 | \* |
| 321 | 42 | G | G | A | 4.70 | 2.484827e-01 |  |
| 321 | 42 | G | G | C | 1.68 | 5.856483e-01 |  |
| 321 | 42 | G | G | G | 92.28 | 0.000000e+00 | \* |
| 321 | 42 | G | G | T | 1.34 | 7.604633e-01 |  |
| 322 | 43 | A | A | A | 94.41 | 0.000000e+00 | \* |
| 322 | 43 | A | A | C | 1.05 | 7.414061e-01 |  |
| 322 | 43 | A | A | G | 2.10 | 5.730994e-01 |  |
| 322 | 43 | A | A | T | 2.45 | 5.383259e-01 |  |
| 323 | 44 | C | C | A | 3.86 | 3.260628e-01 |  |
| 323 | 44 | C | C | C | 89.70 | 0.000000e+00 | \* |
| 323 | 44 | C | C | G | 5.58 | 8.178979e-02 |  |
| 323 | 44 | C | C | T | 0.86 | 8.419981e-01 |  |
| 324 | 45 | C | C | A | 5.29 | 2.035930e-01 |  |
| 324 | 45 | C | C | C | 87.98 | 0.000000e+00 | \* |
| 324 | 45 | C | C | G | 4.81 | 1.338275e-01 |  |
| 324 | 45 | C | C | T | 1.92 | 6.445594e-01 |  |
| 325 | 46 | G | G | A | 5.49 | 1.896934e-01 |  |
| 325 | 46 | G | G | C | 2.20 | 4.644020e-01 |  |
| 325 | 46 | G | G | G | 92.31 | 0.000000e+00 | \* |
| 325 | 46 | G | G | T | 0.00 | 9.042553e-01 |  |
| 326 | 47 | A | A | A | 93.60 | 0.000000e+00 | \* |
| 326 | 47 | A | A | C | 0.91 | 7.730835e-01 |  |
| 326 | 47 | A | A | G | 3.05 | 3.672475e-01 |  |
| 326 | 47 | A | A | T | 2.44 | 5.400099e-01 |  |
| 327 | 48 | C | C | A | 7.18 | 1.045250e-01 |  |
| 327 | 48 | C | C | C | 86.60 | 0.000000e+00 | \* |
| 327 | 48 | C | C | G | 0.96 | 8.298248e-01 |  |
| 327 | 48 | C | C | T | 5.26 | 1.526338e-01 |  |
| 328 | 49 | G | G | A | 11.59 | 1.973748e-02 |  |
| 328 | 49 | G | G | C | 1.45 | 6.423147e-01 |  |
| 328 | 49 | G | G | G | 81.88 | 0.000000e+00 | \* |
| 328 | 49 | G | G | T | 5.07 | 1.679883e-01 |  |
| 329 | 50 | G | G | A | 0.00 | 7.927928e-01 |  |
| 329 | 50 | G | G | C | 1.19 | 7.080092e-01 |  |
| 329 | 50 | G | G | G | 98.81 | 0.000000e+00 | \* |
| 329 | 50 | G | G | T | 0.00 | 9.042553e-01 |  |
| 330 | 51 | C | C | A | 6.17 | 1.497270e-01 |  |
| 330 | 51 | C | C | C | 87.04 | 0.000000e+00 | \* |
| 330 | 51 | C | C | G | 4.32 | 1.801532e-01 |  |
| 330 | 51 | C | C | T | 2.47 | 5.340734e-01 |  |
| 331 | 52 | A | A | A | 87.60 | 2.220446e-16 | \* |
| 331 | 52 | A | A | C | 2.48 | 4.053816e-01 |  |
| 331 | 52 | A | A | G | 0.00 | 9.120879e-01 |  |
| 331 | 52 | A | A | T | 9.92 | 1.158956e-02 |  |
| 332 | 53 | A | A | A | 89.32 | 0.000000e+00 | \* |
| 332 | 53 | A | A | C | 0.49 | 8.595043e-01 |  |
| 332 | 53 | A | A | G | 2.91 | 3.935693e-01 |  |
| 332 | 53 | A | A | T | 7.28 | 5.229211e-02 |  |
| 333 | 54 | G | G | A | 4.08 | 3.040420e-01 |  |
| 333 | 54 | G | G | C | 0.68 | 8.239188e-01 |  |
| 333 | 54 | G | G | G | 95.24 | 0.000000e+00 | \* |
| 333 | 54 | G | G | T | 0.00 | 9.042553e-01 |  |
| 334 | 55 | A | A | A | 92.95 | 0.000000e+00 | \* |
| 334 | 55 | A | A | C | 0.00 | 9.024390e-01 |  |
| 334 | 55 | A | A | G | 2.49 | 4.826446e-01 |  |
| 334 | 55 | A | A | T | 4.56 | 2.156293e-01 |  |
| 335 | 56 | A | A | A | 91.67 | 0.000000e+00 | \* |
| 335 | 56 | A | A | C | 0.00 | 9.024390e-01 |  |
| 335 | 56 | A | A | G | 0.98 | 8.255951e-01 |  |
| 335 | 56 | A | A | T | 7.35 | 5.026943e-02 |  |
| 336 | 57 | G | G | A | 2.44 | 4.986208e-01 |  |
| 336 | 57 | G | G | C | 0.81 | 7.959664e-01 |  |
| 336 | 57 | G | G | G | 90.24 | 0.000000e+00 | \* |
| 336 | 57 | G | G | T | 6.50 | 7.985954e-02 |  |
| 337 | 58 | T | T | A | 5.58 | 1.840889e-01 |  |
| 337 | 58 | T | T | C | 0.00 | 9.024390e-01 |  |
| 337 | 58 | T | T | G | 2.33 | 5.197827e-01 |  |
| 337 | 58 | T | T | T | 92.09 | 0.000000e+00 | \* |
| 338 | 59 | T | T | A | 1.81 | 5.879966e-01 |  |
| 338 | 59 | T | T | C | 0.90 | 7.753036e-01 |  |
| 338 | 59 | T | T | G | 0.90 | 8.388956e-01 |  |
| 338 | 59 | T | T | T | 96.38 | 0.000000e+00 | \* |
| 339 | 60 | C | C | A | 5.26 | 2.053605e-01 |  |
| 339 | 60 | C | C | C | 86.12 | 0.000000e+00 | \* |
| 339 | 60 | C | C | G | 0.96 | 8.298248e-01 |  |
| 339 | 60 | C | C | T | 7.66 | 4.248829e-02 |  |
| 340 | 61 | G | G | A | 7.64 | 8.832657e-02 |  |
| 340 | 61 | G | G | C | 2.08 | 4.898519e-01 |  |
| 340 | 61 | G | G | G | 86.11 | 0.000000e+00 | \* |
| 340 | 61 | G | G | T | 4.17 | 2.604474e-01 |  |
| 341 | 62 | A | A | A | 89.56 | 0.000000e+00 | \* |
| 341 | 62 | A | A | C | 1.35 | 6.679391e-01 |  |
| 341 | 62 | A | A | G | 2.02 | 5.916306e-01 |  |
| 341 | 62 | A | A | T | 7.07 | 5.872245e-02 |  |
| 342 | 63 | C | C | A | 2.68 | 4.662517e-01 |  |
| 342 | 63 | C | C | C | 89.73 | 0.000000e+00 | \* |
| 342 | 63 | C | C | G | 1.34 | 7.523342e-01 |  |
| 342 | 63 | C | C | T | 6.25 | 9.146389e-02 |  |
| 343 | 64 | A | A | A | 88.28 | 2.220446e-16 | \* |
| 343 | 64 | A | A | C | 3.12 | 2.906087e-01 |  |
| 343 | 64 | A | A | G | 3.12 | 3.530464e-01 |  |
| 343 | 64 | A | A | T | 5.47 | 1.374820e-01 |  |
| 344 | 65 | G | G | A | 0.00 | 7.927928e-01 |  |
| 344 | 65 | G | G | C | 5.36 | 7.940038e-02 |  |
| 344 | 65 | G | G | G | 89.73 | 0.000000e+00 | \* |
| 344 | 65 | G | G | T | 4.91 | 1.820545e-01 |  |
| 345 | 66 | C | C | A | 3.05 | 4.190779e-01 |  |
| 345 | 66 | C | C | C | 91.37 | 0.000000e+00 | \* |
| 345 | 66 | C | C | G | 1.52 | 7.104558e-01 |  |
| 345 | 66 | C | C | T | 4.06 | 2.735671e-01 |  |
| 346 | 67 | T | T | A | 0.00 | 7.927928e-01 |  |
| 346 | 67 | T | T | C | 3.57 | 2.276913e-01 |  |
| 346 | 67 | T | T | G | 4.37 | 1.754508e-01 |  |
| 346 | 67 | T | T | T | 92.06 | 0.000000e+00 | \* |
| 347 | 68 | C | C | A | 2.78 | 4.531991e-01 |  |
| 347 | 68 | C | C | C | 92.13 | 0.000000e+00 | \* |
| 347 | 68 | C | C | G | 1.85 | 6.320719e-01 |  |
| 347 | 68 | C | C | T | 3.24 | 3.933898e-01 |  |
| 348 | 69 | C | C | A | 0.39 | 7.721193e-01 |  |
| 348 | 69 | C | C | C | 95.28 | 0.000000e+00 | \* |
| 348 | 69 | C | C | G | 0.79 | 8.577714e-01 |  |
| 348 | 69 | C | C | T | 3.54 | 3.454226e-01 |  |
| 349 | 70 | C | C | A | 0.00 | 7.927928e-01 |  |
| 349 | 70 | C | C | C | 93.20 | 0.000000e+00 | \* |
| 349 | 70 | C | C | G | 6.80 | 3.594024e-02 |  |
| 349 | 70 | C | C | T | 0.00 | 9.042553e-01 |  |
| 350 | 71 | G | G | A | 3.25 | 3.946102e-01 |  |
| 350 | 71 | G | G | C | 3.25 | 2.721714e-01 |  |
| 350 | 71 | G | G | G | 93.51 | 0.000000e+00 | \* |
| 350 | 71 | G | G | T | 0.00 | 9.042553e-01 |  |
| 351 | 72 | C | C | A | 1.11 | 6.880668e-01 |  |
| 351 | 72 | C | C | C | 93.89 | 0.000000e+00 | \* |
| 351 | 72 | C | C | G | 1.67 | 6.764979e-01 |  |
| 351 | 72 | C | C | T | 3.33 | 3.782468e-01 |  |
| 352 | 73 | G | G | A | 7.59 | 9.004744e-02 |  |
| 352 | 73 | G | G | C | 6.21 | 4.664216e-02 |  |
| 352 | 73 | G | G | G | 85.52 | 0.000000e+00 | \* |
| 352 | 73 | G | G | T | 0.69 | 8.641780e-01 |  |
| 353 | 74 | A | A | A | 97.31 | 0.000000e+00 | \* |
| 353 | 74 | A | A | C | 0.90 | 7.771627e-01 |  |
| 353 | 74 | A | A | G | 0.90 | 8.402738e-01 |  |
| 353 | 74 | A | A | T | 0.90 | 8.363970e-01 |  |
| 354 | 75 | C | C | A | 4.39 | 2.754466e-01 |  |
| 354 | 75 | C | C | C | 58.77 | 0.000000e+00 | \* |
| 354 | 75 | C | C | G | 28.51 | 1.108239e-09 | \* |
| 354 | 75 | C | C | T | 8.33 | 2.899611e-02 |  |

## For use in R

If you want to work with the results in R, here is output that you can copy and paste in your terminal to get:

The base information:

```
structure(list(focal.base = c("A", "C", "G", "T"), avg.percsignal = c(91.5601502866246, 
90.263881583944, 90.199235074325, 93.9244371068512), avg.areasignal = c(242.545454545455, 
259.028985507246, 249.053571428571, 290.744186046512), crit.perc.area = c(13.3291528129979, 
8.56291452185823, 8.60499792538676, 10.1675974379697), mu = c(3.95293620779635, 
2.67219353923526, 2.97284018657932, 3.35833249315494), fillibens = c(0.994374327323572, 
0.937079945577972, 0.995608360693313, 0.99462110367852)), .Names = c("focal.base", 
"avg.percsignal", "avg.areasignal", "crit.perc.area", "mu", "fillibens"
), row.names = c(NA, -4L), class = "data.frame")
```

the data.frame that contains information on the guide region:

```
structure(list(A.area = c(211, 258, 6, 467, 20, 0, 18, 20, 7, 
7, 206, 10, 469, 15, 5, 12, 6, 2, 3, 0, 1, 14, 5, 16, 6, 157, 
11, 8, 225, 8, 147, 1, 14, 8, 13, 11, 183, 9, 13, 14, 12, 14, 
270, 9, 11, 10, 307, 15, 16, 0, 10, 106, 184, 6, 224, 187, 3, 
12, 4, 11, 11, 266, 6, 113, 0, 6, 0, 6, 1, 0, 5, 2, 11, 217, 
10), C.area = c(3, 7, 14, 5, 240, 4, 4, 9, 177, 274, 5, 3, 5, 
257, 195, 4, 0, 0, 0, 0, 6, 3, 2, 5, 218, 6, 234, 6, 6, 211, 
3, 192, 3, 9, 4, 186, 5, 255, 226, 218, 8, 5, 3, 209, 183, 4, 
3, 181, 2, 3, 141, 3, 1, 1, 0, 0, 1, 0, 2, 180, 3, 4, 201, 4, 
12, 180, 9, 199, 242, 137, 5, 169, 9, 2, 134), G.area = c(0, 
8, 181, 2, 3, 8, 480, 297, 11, 10, 6, 204, 4, 4, 4, 227, 5, 401, 
12, 9, 5, 308, 7, 249, 4, 13, 12, 12, 5, 2, 15, 5, 155, 250, 
204, 8, 0, 0, 12, 0, 8, 275, 6, 13, 10, 168, 10, 2, 113, 250, 
7, 0, 6, 140, 6, 2, 111, 5, 2, 2, 124, 6, 3, 4, 201, 3, 11, 4, 
2, 10, 144, 3, 124, 2, 65), T.area = c(0, 6, 0, 15, 5, 250, 4, 
6, 12, 12, 12, 5, 18, 10, 8, 6, 217, 9, 245, 271, 275, 4, 227, 
11, 11, 18, 7, 237, 5, 7, 8, 11, 8, 4, 1, 0, 6, 9, 11, 7, 203, 
4, 7, 2, 4, 0, 8, 11, 7, 0, 4, 12, 15, 0, 11, 15, 8, 198, 213, 
16, 6, 21, 14, 7, 11, 8, 232, 7, 9, 0, 0, 6, 1, 2, 19), Tot.area = c(214, 
279, 201, 489, 268, 262, 506, 332, 207, 303, 229, 222, 496, 286, 
212, 249, 228, 412, 260, 280, 287, 329, 241, 281, 239, 194, 264, 
263, 241, 228, 173, 209, 180, 271, 222, 205, 194, 273, 262, 239, 
231, 298, 286, 233, 208, 182, 328, 209, 138, 253, 162, 121, 206, 
147, 241, 204, 123, 215, 221, 209, 144, 297, 224, 128, 224, 197, 
252, 216, 254, 147, 154, 180, 145, 223, 228), A.perc = c(98.5981308411215, 
92.4731182795699, 2.98507462686567, 95.5010224948875, 7.46268656716418, 
0, 3.55731225296443, 6.02409638554217, 3.38164251207729, 2.31023102310231, 
89.9563318777293, 4.5045045045045, 94.5564516129032, 5.24475524475525, 
2.35849056603774, 4.81927710843374, 2.63157894736842, 0.485436893203884, 
1.15384615384615, 0, 0.348432055749129, 4.25531914893617, 2.0746887966805, 
5.69395017793594, 2.51046025104602, 80.9278350515464, 4.16666666666667, 
3.04182509505703, 93.3609958506224, 3.50877192982456, 84.971098265896, 
0.478468899521531, 7.77777777777778, 2.9520295202952, 5.85585585585586, 
5.36585365853659, 94.3298969072165, 3.2967032967033, 4.9618320610687, 
5.85774058577406, 5.19480519480519, 4.69798657718121, 94.4055944055944, 
3.86266094420601, 5.28846153846154, 5.49450549450549, 93.5975609756098, 
7.17703349282297, 11.5942028985507, 0, 6.17283950617284, 87.603305785124, 
89.3203883495146, 4.08163265306122, 92.9460580912863, 91.6666666666667, 
2.4390243902439, 5.58139534883721, 1.80995475113122, 5.26315789473684, 
7.63888888888889, 89.5622895622896, 2.67857142857143, 88.28125, 
0, 3.04568527918782, 0, 2.77777777777778, 0.393700787401575, 
0, 3.24675324675325, 1.11111111111111, 7.58620689655172, 97.3094170403587, 
4.3859649122807), C.perc = c(1.4018691588785, 2.5089605734767, 
6.96517412935323, 1.02249488752556, 89.5522388059701, 1.52671755725191, 
0.790513833992095, 2.71084337349398, 85.5072463768116, 90.4290429042904, 
2.18340611353712, 1.35135135135135, 1.00806451612903, 89.8601398601399, 
91.9811320754717, 1.60642570281124, 0, 0, 0, 0, 2.09059233449477, 
0.911854103343465, 0.829875518672199, 1.77935943060498, 91.2133891213389, 
3.09278350515464, 88.6363636363636, 2.28136882129278, 2.4896265560166, 
92.5438596491228, 1.73410404624277, 91.866028708134, 1.66666666666667, 
3.3210332103321, 1.8018018018018, 90.7317073170732, 2.57731958762887, 
93.4065934065934, 86.2595419847328, 91.2133891213389, 3.46320346320346, 
1.67785234899329, 1.04895104895105, 89.6995708154506, 87.9807692307692, 
2.1978021978022, 0.914634146341463, 86.6028708133971, 1.44927536231884, 
1.18577075098814, 87.037037037037, 2.47933884297521, 0.485436893203884, 
0.680272108843537, 0, 0, 0.813008130081301, 0, 0.904977375565611, 
86.1244019138756, 2.08333333333333, 1.34680134680135, 89.7321428571429, 
3.125, 5.35714285714286, 91.3705583756345, 3.57142857142857, 
92.1296296296296, 95.2755905511811, 93.1972789115646, 3.24675324675325, 
93.8888888888889, 6.20689655172414, 0.896860986547085, 58.7719298245614
), G.perc = c(0, 2.8673835125448, 90.0497512437811, 0.408997955010225, 
1.11940298507463, 3.05343511450382, 94.8616600790514, 89.4578313253012, 
5.31400966183575, 3.3003300330033, 2.62008733624454, 91.8918918918919, 
0.806451612903226, 1.3986013986014, 1.88679245283019, 91.1646586345382, 
2.19298245614035, 97.3300970873786, 4.61538461538461, 3.21428571428571, 
1.74216027874564, 93.6170212765958, 2.9045643153527, 88.6120996441281, 
1.67364016736402, 6.70103092783505, 4.54545454545455, 4.56273764258555, 
2.0746887966805, 0.87719298245614, 8.67052023121387, 2.39234449760766, 
86.1111111111111, 92.2509225092251, 91.8918918918919, 3.90243902439024, 
0, 0, 4.58015267175572, 0, 3.46320346320346, 92.2818791946309, 
2.0979020979021, 5.5793991416309, 4.80769230769231, 92.3076923076923, 
3.04878048780488, 0.956937799043062, 81.8840579710145, 98.8142292490119, 
4.32098765432099, 0, 2.9126213592233, 95.2380952380952, 2.4896265560166, 
0.980392156862745, 90.2439024390244, 2.32558139534884, 0.904977375565611, 
0.956937799043062, 86.1111111111111, 2.02020202020202, 1.33928571428571, 
3.125, 89.7321428571429, 1.52284263959391, 4.36507936507936, 
1.85185185185185, 0.78740157480315, 6.80272108843537, 93.5064935064935, 
1.66666666666667, 85.5172413793103, 0.896860986547085, 28.5087719298246
), T.perc = c(0, 2.1505376344086, 0, 3.06748466257669, 1.86567164179104, 
95.4198473282443, 0.790513833992095, 1.80722891566265, 5.79710144927536, 
3.96039603960396, 5.24017467248908, 2.25225225225225, 3.62903225806452, 
3.4965034965035, 3.77358490566038, 2.40963855421687, 95.1754385964912, 
2.18446601941748, 94.2307692307692, 96.7857142857143, 95.8188153310105, 
1.21580547112462, 94.1908713692946, 3.91459074733096, 4.60251046025105, 
9.27835051546392, 2.65151515151515, 90.1140684410646, 2.0746887966805, 
3.07017543859649, 4.6242774566474, 5.26315789473684, 4.44444444444444, 
1.4760147601476, 0.45045045045045, 0, 3.09278350515464, 3.2967032967033, 
4.19847328244275, 2.92887029288703, 87.8787878787879, 1.34228187919463, 
2.44755244755245, 0.858369098712446, 1.92307692307692, 0, 2.4390243902439, 
5.26315789473684, 5.07246376811594, 0, 2.46913580246914, 9.91735537190083, 
7.28155339805825, 0, 4.5643153526971, 7.35294117647059, 6.50406504065041, 
92.0930232558139, 96.3800904977376, 7.6555023923445, 4.16666666666667, 
7.07070707070707, 6.25, 5.46875, 4.91071428571429, 4.06091370558376, 
92.0634920634921, 3.24074074074074, 3.54330708661417, 0, 0, 3.33333333333333, 
0.689655172413793, 0.896860986547085, 8.33333333333333), base.call = c("A", 
"A", "G", "A", "C", "T", "G", "G", "C", "C", "A", "G", "A", "C", 
"C", "G", "T", "G", "T", "T", "T", "G", "T", "G", "C", "A", "C", 
"T", "A", "C", "A", "C", "G", "G", "G", "C", "A", "C", "C", "C", 
"T", "G", "A", "C", "C", "G", "A", "C", "G", "G", "C", "A", "A", 
"G", "A", "A", "G", "T", "T", "C", "G", "A", "C", "A", "G", "C", 
"T", "C", "C", "C", "G", "C", "G", "A", "C"), index = 280:354, 
    guide.seq = c("A", "A", "G", "A", "C", "T", "G", "G", "C", 
    "C", "A", "G", "A", "C", "C", "G", "T", "G", "T", "T", "T", 
    "G", "T", "G", "C", "A", "C", "T", "A", "C", "A", "C", "G", 
    "G", "G", "C", "A", "C", "C", "C", "T", "G", "A", "C", "C", 
    "G", "A", "C", "G", "G", "C", "A", "A", "G", "A", "A", "G", 
    "T", "T", "C", "G", "A", "C", "A", "G", "C", "T", "C", "C", 
    "C", "G", "C", "G", "A", "C"), T.pval = c(0.904255319142527, 
    0.597996315427329, 0.904255319142527, 0.422797656760365, 
    0.656327932064941, 0, 0.851401110062009, 0.668286970214135, 
    0.116057947117681, 0.286521039861083, 0.154416346588028, 
    0.577355167012928, 0.332632264401354, 0.352555530655951, 
    0.311868689413737, 0.545827929829238, 0, 0.591092571227858, 
    0, 0, 0, 0.783759698670182, 0, 0.292582223798993, 0.21168629734723, 
    0.0168393690969707, 0.498723850444714, 0, 0.613482787604678, 
    0.422330453734056, 0.209466581724038, 0.152633799238192, 
    0.228407498837985, 0.734781893318928, 0.887943251324875, 
    0.904255319142527, 0.418417872440625, 0.384188997504715, 
    0.256602368039139, 0.447298838900163, 0, 0.760463255017243, 
    0.538325914639501, 0.841998104424256, 0.644559379767984, 
    0.904255319142527, 0.540009868239547, 0.152633799238192, 
    0.167988270695572, 0.904255319142527, 0.534073380969819, 
    0.011589564640798, 0.0522921126779277, 0.904255319142527, 
    0.215629341771643, 0.050269430218322, 0.0798595448033842, 
    0, 0, 0.0424882865696614, 0.260447403938185, 0.0587224480707871, 
    0.0914638918528664, 0.137481987375974, 0.182054472581178, 
    0.273567071939462, 0, 0.393389779452878, 0.345422567574756, 
    0.904255319142527, 0.904255319142527, 0.378246806245932, 
    0.864178003118715, 0.836396988591594, 0.028996108091395), 
    C.pval = c(0.654167068134433, 0.399481757393459, 0.028672419166905, 
    0.747749782171787, 0, 0.622998292489528, 0.800872913911332, 
    0.360893300321374, 0, 0, 0.467558474528387, 0.666801541016293, 
    0.751190503359397, 0, 0, 0.603226638071199, 0.902439024385758, 
    0.902439024385758, 0.902439024385758, 0.902439024385758, 
    0.488214596088726, 0.773723573549801, 0.792246903821934, 
    0.560963081397738, 0, 0.295653246390872, 0, 0.446338670221452, 
    0.403325704656918, 0, 0.571920999826632, 0, 0.588390907920036, 
    0.26140305524339, 0.555559909908908, 0, 0.386097832050942, 
    0, 0, 0, 0.241781353092849, 0.585648310457821, 0.741406148668871, 
    0, 0, 0.464401960775179, 0.773083546605904, 0, 0.642314729973568, 
    0.708009161660387, 0, 0.405381562844414, 0.859504319923553, 
    0.823918750594237, 0.902439024385758, 0.902439024385758, 
    0.795966410273527, 0.902439024385758, 0.775303625458525, 
    0, 0.489851929459923, 0.667939104622402, 0, 0.290608650498541, 
    0.0794003751466527, 0, 0.22769133850724, 0, 0, 0, 0.272171395256832, 
    0, 0.0466421629522075, 0.777162696462526, 0), G.pval = c(0.912087912058151, 
    0.402579914069349, 0, 0.900078970948601, 0.798958074059311, 
    0.366369229801936, 0, 0, 0.0971365144275915, 0.321856446897638, 
    0.454068059934503, 0, 0.854877335393524, 0.739034398801391, 
    0.623662704611169, 0, 0.550626130910337, 0, 0.150710345554532, 
    0.336904708937451, 0.658440927280614, 0, 0.395164524891175, 
    0, 0.674834889171757, 0.0385459397612107, 0.157296775636744, 
    0.155646043448985, 0.578622218789849, 0.843568972923251, 
    0.00953415826882442, 0.504518847868081, 0, 0, 0, 0.230349220557466, 
    0.912087912058151, 0.912087912058151, 0.153997991508013, 
    0.912087912058151, 0.294737297193836, 0, 0.573099363250368, 
    0.0817897896413411, 0.133827481636757, 0, 0.367247538556607, 
    0.82982481964386, 0, 0, 0.180153153548038, 0.912087912058151, 
    0.393569260143138, 0, 0.482644583250246, 0.825595059078947, 
    0, 0.519782650775001, 0.83889559854631, 0.82982481964386, 
    0, 0.591630616316135, 0.752334247410515, 0.353046407878353, 
    0, 0.710455814413416, 0.175450807729519, 0.632071886380559, 
    0.857771357922039, 0.0359402404049387, 0, 0.676497853230473, 
    0, 0.840273848732825, 1.10823883403555e-09), A.pval = c(0, 
    0, 0.426648501436212, 0, 0.0942066178008063, 0.792792792792792, 
    0.35882663283582, 0.157773478015061, 0.378764923984528, 0.516475038528368, 
    0, 0.264927208518551, 0, 0.206654440775422, 0.509751075358626, 
    0.238622682233101, 0.472509103531837, 0.763698469843599, 
    0.682180741294632, 0.792792792792792, 0.775892130335957, 
    0.287439355200816, 0.549801888058388, 0.177049083773337, 
    0.488848161932661, 3.33066907387547e-15, 0.295818911552556, 
    0.419557395522741, 0, 0.36425656212676, 6.66133814775094e-16, 
    0.764371746264445, 0.0839369209815557, 0.430813281796159, 
    0.167345816427844, 0.19826996799312, 0, 0.38868879713524, 
    0.227464939622797, 0.167235737840714, 0.21020269610834, 0.248482718297993, 
    0, 0.326062782006247, 0.203593008331504, 0.189693355693499, 
    0, 0.104525034369432, 0.0197374849250598, 0.792792792792792, 
    0.149727000938358, 2.22044604925031e-16, 0, 0.304041957144834, 
    0, 0, 0.498620772531033, 0.184088941781769, 0.587996628629524, 
    0.205360515298487, 0.0883265658211423, 0, 0.466251688839345, 
    2.22044604925031e-16, 0.792792792792792, 0.419077893518885, 
    0.792792792792792, 0.453199061485786, 0.772119279550724, 
    0.792792792792792, 0.394610241891681, 0.688066759359186, 
    0.0900474382100602, 0, 0.275446565714679), guide.position = 1:75), .Names = c("A.area", 
"C.area", "G.area", "T.area", "Tot.area", "A.perc", "C.perc", 
"G.perc", "T.perc", "base.call", "index", "guide.seq", "T.pval", 
"C.pval", "G.pval", "A.pval", "guide.position"), row.names = 280:354, class = "data.frame")
```

*Report generated using EditR v1.0.8*
