# Supplementary material for: Mechanistic and genetic basis of single-strand templated repair at Cas12a-induced DNA breaks in Chlamydomonas reinhardtii
Source: Nat Commun. 2021 Nov 19;12:6751. doi: 10.1038/s41467-021-27004-1 (PMC8604939; doi:10.1038/s41467-021-27004-1)
Supplement: Supplementary file 22 — Source Data [file 41467_2021_27004_MOESM22_ESM.zip › Source Data/EditR analysis/EditR outputs/Sense/rep2_ssODN_sense_-32.html]

EditR v1.0.8 report


# EditR v1.0.8 report

- Data QA
  - Filtering data
  - Percent noise peak area
  - Base information
- Predicted editing
  - Editing bar plot
  - Editing table plot
  - Table of editing results
- For use in R

## Data QA

### Filtering data

What the data looked like prefiltering:

and the post filtering signal / noise plot:

### Percent noise peak area

### Base information

Here’s information about the signal of each base, the critical percent value where any higher value would be called as significant, and Filliben’s correlation for how well the noise was modelled by the zero adjusted gamma distribution.

| Base | Average percent signal | Average peak area | Critical percent value | model mu | Fillibens correlation |
| --- | --- | --- | --- | --- | --- |
| A | 92.55899 | 314.9375 | 7.094623 | 2.265746 | 0.9936653 |
| C | 93.99463 | 354.2319 | 7.271032 | 2.551741 | 0.9906841 |
| G | 92.67909 | 322.7895 | 7.151154 | 2.093171 | 0.9977011 |
| T | 93.61684 | 359.3488 | 6.285247 | 2.169623 | 0.9891092 |

## Predicted editing

### Editing bar plot

### Editing table plot

### Table of editing results


Here’s the entire guide region

| Sanger position | Guide position | Guide sequence | Sanger base call | Focal base | Focal base peak area | p value |  |
| --- | --- | --- | --- | --- | --- | --- | --- |
| 282 | 1 | A | A | A | 95.27 | 0.000000e+00 | \* |
| 282 | 1 | A | A | C | 2.52 | 4.016986e-01 |  |
| 282 | 1 | A | A | G | 0.63 | 7.620585e-01 |  |
| 282 | 1 | A | A | T | 1.58 | 5.880654e-01 |  |
| 283 | 2 | A | A | A | 95.94 | 0.000000e+00 | \* |
| 283 | 2 | A | A | C | 1.16 | 7.802455e-01 |  |
| 283 | 2 | A | A | G | 1.74 | 4.391061e-01 |  |
| 283 | 2 | A | A | T | 1.16 | 7.324468e-01 |  |
| 284 | 3 | G | G | A | 4.45 | 8.104722e-02 |  |
| 284 | 3 | G | G | C | 1.62 | 6.497491e-01 |  |
| 284 | 3 | G | G | G | 92.71 | 0.000000e+00 | \* |
| 284 | 3 | G | G | T | 1.21 | 7.135857e-01 |  |
| 285 | 4 | A | A | A | 96.81 | 0.000000e+00 | \* |
| 285 | 4 | A | A | C | 1.28 | 7.485634e-01 |  |
| 285 | 4 | A | A | G | 0.96 | 6.629258e-01 |  |
| 285 | 4 | A | A | T | 0.96 | 7.991053e-01 |  |
| 286 | 5 | C | C | A | 4.32 | 8.929023e-02 |  |
| 286 | 5 | C | C | C | 92.16 | 0.000000e+00 | \* |
| 286 | 5 | C | C | G | 1.35 | 5.443671e-01 |  |
| 286 | 5 | C | C | T | 2.16 | 4.035252e-01 |  |
| 287 | 6 | T | T | A | 4.56 | 7.491754e-02 |  |
| 287 | 6 | T | T | C | 2.41 | 4.290069e-01 |  |
| 287 | 6 | T | T | G | 3.49 | 1.441693e-01 |  |
| 287 | 6 | T | T | T | 89.54 | 0.000000e+00 | \* |
| 288 | 7 | G | G | A | 1.48 | 5.775797e-01 |  |
| 288 | 7 | G | G | C | 1.15 | 7.833912e-01 |  |
| 288 | 7 | G | G | G | 96.07 | 0.000000e+00 | \* |
| 288 | 7 | G | G | T | 1.31 | 6.800805e-01 |  |
| 289 | 8 | G | G | A | 2.45 | 3.291075e-01 |  |
| 289 | 8 | G | G | C | 1.84 | 5.852446e-01 |  |
| 289 | 8 | G | G | G | 95.40 | 0.000000e+00 | \* |
| 289 | 8 | G | G | T | 0.31 | 9.447403e-01 |  |
| 290 | 9 | C | C | A | 2.00 | 4.337044e-01 |  |
| 290 | 9 | C | C | C | 93.60 | 0.000000e+00 | \* |
| 290 | 9 | C | C | G | 3.20 | 1.748803e-01 |  |
| 290 | 9 | C | C | T | 1.20 | 7.185889e-01 |  |
| 291 | 10 | C | C | A | 2.26 | 3.722890e-01 |  |
| 291 | 10 | C | C | C | 94.99 | 0.000000e+00 | \* |
| 291 | 10 | C | C | G | 1.25 | 5.730769e-01 |  |
| 291 | 10 | C | C | T | 1.50 | 6.133269e-01 |  |
| 292 | 11 | A | A | A | 61.98 | 0.000000e+00 | \* |
| 292 | 11 | A | A | C | 2.88 | 3.226840e-01 |  |
| 292 | 11 | A | A | G | 2.24 | 3.261552e-01 |  |
| 292 | 11 | A | A | T | 32.91 | 5.551115e-16 | \* |
| 293 | 12 | G | G | A | 3.80 | 1.315885e-01 |  |
| 293 | 12 | G | G | C | 1.27 | 7.513623e-01 |  |
| 293 | 12 | G | G | G | 93.35 | 0.000000e+00 | \* |
| 293 | 12 | G | G | T | 1.58 | 5.863605e-01 |  |
| 294 | 13 | A | A | A | 95.73 | 0.000000e+00 | \* |
| 294 | 13 | A | A | C | 1.83 | 5.884939e-01 |  |
| 294 | 13 | A | A | G | 1.42 | 5.239610e-01 |  |
| 294 | 13 | A | A | T | 1.02 | 7.801223e-01 |  |
| 295 | 14 | C | C | A | 3.05 | 2.229615e-01 |  |
| 295 | 14 | C | C | C | 93.91 | 0.000000e+00 | \* |
| 295 | 14 | C | C | G | 1.39 | 5.346882e-01 |  |
| 295 | 14 | C | C | T | 1.66 | 5.593244e-01 |  |
| 296 | 15 | C | C | A | 3.63 | 1.489278e-01 |  |
| 296 | 15 | C | C | C | 92.75 | 0.000000e+00 | \* |
| 296 | 15 | C | C | G | 2.42 | 2.914066e-01 |  |
| 296 | 15 | C | C | T | 1.21 | 7.156866e-01 |  |
| 297 | 16 | G | G | A | 4.15 | 1.013309e-01 |  |
| 297 | 16 | G | G | C | 1.78 | 6.026833e-01 |  |
| 297 | 16 | G | G | G | 92.28 | 0.000000e+00 | \* |
| 297 | 16 | G | G | T | 1.78 | 5.200985e-01 |  |
| 298 | 17 | T | T | A | 1.98 | 4.377253e-01 |  |
| 298 | 17 | T | T | C | 0.40 | 9.249384e-01 |  |
| 298 | 17 | T | T | G | 3.17 | 1.778843e-01 |  |
| 298 | 17 | T | T | T | 94.44 | 0.000000e+00 | \* |
| 299 | 18 | G | G | A | 1.66 | 5.247109e-01 |  |
| 299 | 18 | G | G | C | 1.04 | 8.116609e-01 |  |
| 299 | 18 | G | G | G | 95.64 | 0.000000e+00 | \* |
| 299 | 18 | G | G | T | 1.66 | 5.600974e-01 |  |
| 300 | 19 | T | T | A | 0.68 | 8.050323e-01 |  |
| 300 | 19 | T | T | C | 0.00 | 9.390244e-01 |  |
| 300 | 19 | T | T | G | 4.10 | 9.444470e-02 |  |
| 300 | 19 | T | T | T | 95.22 | 0.000000e+00 | \* |
| 301 | 20 | T | T | A | 0.31 | 8.802777e-01 |  |
| 301 | 20 | T | T | C | 0.93 | 8.382729e-01 |  |
| 301 | 20 | T | T | G | 1.54 | 4.905399e-01 |  |
| 301 | 20 | T | T | T | 97.22 | 0.000000e+00 | \* |
| 302 | 21 | T | T | A | 0.00 | 9.017857e-01 |  |
| 302 | 21 | T | T | C | 0.00 | 9.390244e-01 |  |
| 302 | 21 | T | T | G | 2.69 | 2.440509e-01 |  |
| 302 | 21 | T | T | T | 97.31 | 0.000000e+00 | \* |
| 303 | 22 | G | G | A | 1.01 | 7.159200e-01 |  |
| 303 | 22 | G | G | C | 0.50 | 9.137251e-01 |  |
| 303 | 22 | G | G | G | 96.47 | 0.000000e+00 | \* |
| 303 | 22 | G | G | T | 2.02 | 4.464081e-01 |  |
| 304 | 23 | T | T | A | 0.70 | 8.002020e-01 |  |
| 304 | 23 | T | T | C | 0.35 | 9.286833e-01 |  |
| 304 | 23 | T | T | G | 2.46 | 2.842785e-01 |  |
| 304 | 23 | T | T | T | 96.49 | 0.000000e+00 | \* |
| 305 | 24 | G | G | A | 1.13 | 6.808671e-01 |  |
| 305 | 24 | G | G | C | 1.97 | 5.475909e-01 |  |
| 305 | 24 | G | G | G | 94.65 | 0.000000e+00 | \* |
| 305 | 24 | G | G | T | 2.25 | 3.782793e-01 |  |
| 306 | 25 | C | C | A | 1.61 | 5.394255e-01 |  |
| 306 | 25 | C | C | C | 93.89 | 0.000000e+00 | \* |
| 306 | 25 | C | C | G | 2.57 | 2.640190e-01 |  |
| 306 | 25 | C | C | T | 1.93 | 4.726609e-01 |  |
| 307 | 26 | A | A | A | 91.67 | 0.000000e+00 | \* |
| 307 | 26 | A | A | C | 4.58 | 9.463777e-02 |  |
| 307 | 26 | A | A | G | 1.25 | 5.740039e-01 |  |
| 307 | 26 | A | A | T | 2.50 | 3.157155e-01 |  |
| 308 | 27 | C | C | A | 1.70 | 5.135367e-01 |  |
| 308 | 27 | C | C | C | 95.75 | 0.000000e+00 | \* |
| 308 | 27 | C | C | G | 0.28 | 8.524457e-01 |  |
| 308 | 27 | C | C | T | 2.27 | 3.748386e-01 |  |
| 309 | 28 | T | T | A | 3.86 | 1.259766e-01 |  |
| 309 | 28 | T | T | C | 2.97 | 3.040107e-01 |  |
| 309 | 28 | T | T | G | 0.00 | 8.888889e-01 |  |
| 309 | 28 | T | T | T | 93.18 | 0.000000e+00 | \* |
| 310 | 29 | A | A | A | 94.20 | 0.000000e+00 | \* |
| 310 | 29 | A | A | C | 2.03 | 5.314684e-01 |  |
| 310 | 29 | A | A | G | 0.87 | 6.898520e-01 |  |
| 310 | 29 | A | A | T | 2.90 | 2.315829e-01 |  |
| 311 | 30 | C | C | A | 2.53 | 3.132749e-01 |  |
| 311 | 30 | C | C | C | 94.62 | 0.000000e+00 | \* |
| 311 | 30 | C | C | G | 1.58 | 4.799853e-01 |  |
| 311 | 30 | C | C | T | 1.27 | 6.959085e-01 |  |
| 312 | 31 | A | A | A | 94.00 | 0.000000e+00 | \* |
| 312 | 31 | A | A | C | 3.60 | 1.972539e-01 |  |
| 312 | 31 | A | A | G | 2.40 | 2.945272e-01 |  |
| 312 | 31 | A | A | T | 0.00 | 9.574468e-01 |  |
| 313 | 32 | C | C | A | 2.05 | 4.217437e-01 |  |
| 313 | 32 | C | C | C | 95.90 | 0.000000e+00 | \* |
| 313 | 32 | C | C | G | 2.05 | 3.659892e-01 |  |
| 313 | 32 | C | C | T | 0.00 | 9.574468e-01 |  |
| 314 | 33 | G | G | A | 3.61 | 1.500942e-01 |  |
| 314 | 33 | G | G | C | 3.61 | 1.952330e-01 |  |
| 314 | 33 | G | G | G | 91.97 | 0.000000e+00 | \* |
| 314 | 33 | G | G | T | 0.80 | 8.452423e-01 |  |
| 315 | 34 | G | G | A | 0.32 | 8.790356e-01 |  |
| 315 | 34 | G | G | C | 1.59 | 6.591544e-01 |  |
| 315 | 34 | G | G | G | 97.14 | 0.000000e+00 | \* |
| 315 | 34 | G | G | T | 0.95 | 8.005400e-01 |  |
| 316 | 35 | G | G | A | 1.30 | 6.297212e-01 |  |
| 316 | 35 | G | G | C | 2.16 | 4.940427e-01 |  |
| 316 | 35 | G | G | G | 96.10 | 0.000000e+00 | \* |
| 316 | 35 | G | G | T | 0.43 | 9.285113e-01 |  |
| 317 | 36 | C | C | A | 1.90 | 4.591247e-01 |  |
| 317 | 36 | C | C | C | 93.92 | 0.000000e+00 | \* |
| 317 | 36 | C | C | G | 2.28 | 3.171989e-01 |  |
| 317 | 36 | C | C | T | 1.90 | 4.814430e-01 |  |
| 318 | 37 | A | A | A | 93.10 | 0.000000e+00 | \* |
| 318 | 37 | A | A | C | 4.21 | 1.255853e-01 |  |
| 318 | 37 | A | A | G | 0.77 | 7.215056e-01 |  |
| 318 | 37 | A | A | T | 1.92 | 4.768823e-01 |  |
| 319 | 38 | C | C | A | 1.78 | 4.927723e-01 |  |
| 319 | 38 | C | C | C | 96.45 | 0.000000e+00 | \* |
| 319 | 38 | C | C | G | 0.59 | 7.734615e-01 |  |
| 319 | 38 | C | C | T | 1.18 | 7.242605e-01 |  |
| 320 | 39 | C | C | A | 2.45 | 3.306694e-01 |  |
| 320 | 39 | C | C | C | 94.80 | 0.000000e+00 | \* |
| 320 | 39 | C | C | G | 1.22 | 5.819477e-01 |  |
| 320 | 39 | C | C | T | 1.53 | 6.046089e-01 |  |
| 321 | 40 | C | C | A | 2.47 | 3.259719e-01 |  |
| 321 | 40 | C | C | C | 94.14 | 0.000000e+00 | \* |
| 321 | 40 | C | C | G | 1.23 | 5.785796e-01 |  |
| 321 | 40 | C | C | T | 2.16 | 4.039964e-01 |  |
| 322 | 41 | T | T | A | 4.38 | 8.596637e-02 |  |
| 322 | 41 | T | T | C | 4.06 | 1.407770e-01 |  |
| 322 | 41 | T | T | G | 3.12 | 1.838863e-01 |  |
| 322 | 41 | T | T | T | 88.44 | 0.000000e+00 | \* |
| 323 | 42 | G | G | A | 1.86 | 4.698392e-01 |  |
| 323 | 42 | G | G | C | 1.40 | 7.148462e-01 |  |
| 323 | 42 | G | G | G | 94.88 | 0.000000e+00 | \* |
| 323 | 42 | G | G | T | 1.86 | 4.942992e-01 |  |
| 324 | 43 | A | A | A | 90.56 | 0.000000e+00 | \* |
| 324 | 43 | A | A | C | 2.65 | 3.708309e-01 |  |
| 324 | 43 | A | A | G | 5.01 | 4.896593e-02 |  |
| 324 | 43 | A | A | T | 1.77 | 5.235301e-01 |  |
| 325 | 44 | C | C | A | 4.64 | 7.060901e-02 |  |
| 325 | 44 | C | C | C | 92.05 | 0.000000e+00 | \* |
| 325 | 44 | C | C | G | 2.32 | 3.100726e-01 |  |
| 325 | 44 | C | C | T | 0.99 | 7.875092e-01 |  |
| 326 | 45 | C | C | A | 4.21 | 9.719268e-02 |  |
| 326 | 45 | C | C | C | 92.28 | 0.000000e+00 | \* |
| 326 | 45 | C | C | G | 2.46 | 2.842785e-01 |  |
| 326 | 45 | C | C | T | 1.05 | 7.682309e-01 |  |
| 327 | 46 | G | G | A | 5.45 | 3.769461e-02 |  |
| 327 | 46 | G | G | C | 3.50 | 2.114281e-01 |  |
| 327 | 46 | G | G | G | 91.05 | 0.000000e+00 | \* |
| 327 | 46 | G | G | T | 0.00 | 9.574468e-01 |  |
| 328 | 47 | A | A | A | 93.39 | 0.000000e+00 | \* |
| 328 | 47 | A | A | C | 1.93 | 5.599643e-01 |  |
| 328 | 47 | A | A | G | 3.58 | 1.350012e-01 |  |
| 328 | 47 | A | A | T | 1.10 | 7.518550e-01 |  |
| 329 | 48 | C | C | A | 6.13 | 2.190935e-02 |  |
| 329 | 48 | C | C | C | 90.04 | 0.000000e+00 | \* |
| 329 | 48 | C | C | G | 2.30 | 3.137703e-01 |  |
| 329 | 48 | C | C | T | 1.53 | 6.033995e-01 |  |
| 330 | 49 | G | G | A | 8.16 | 4.112096e-03 | \* |
| 330 | 49 | G | G | C | 3.06 | 2.858004e-01 |  |
| 330 | 49 | G | G | G | 87.24 | 0.000000e+00 | \* |
| 330 | 49 | G | G | T | 1.53 | 6.040720e-01 |  |
| 331 | 50 | G | G | A | 3.91 | 1.208535e-01 |  |
| 331 | 50 | G | G | C | 1.42 | 7.067697e-01 |  |
| 331 | 50 | G | G | G | 93.24 | 0.000000e+00 | \* |
| 331 | 50 | G | G | T | 1.42 | 6.411369e-01 |  |
| 332 | 51 | C | C | A | 4.81 | 6.168774e-02 |  |
| 332 | 51 | C | C | C | 89.84 | 0.000000e+00 | \* |
| 332 | 51 | C | C | G | 5.35 | 3.841988e-02 |  |
| 332 | 51 | C | C | T | 0.00 | 9.574468e-01 |  |
| 333 | 52 | A | A | A | 89.73 | 0.000000e+00 | \* |
| 333 | 52 | A | A | C | 2.74 | 3.517441e-01 |  |
| 333 | 52 | A | A | G | 1.37 | 5.390376e-01 |  |
| 333 | 52 | A | A | T | 6.16 | 1.129736e-02 |  |
| 334 | 53 | A | A | A | 95.56 | 0.000000e+00 | \* |
| 334 | 53 | A | A | C | 0.40 | 9.243657e-01 |  |
| 334 | 53 | A | A | G | 2.02 | 3.730326e-01 |  |
| 334 | 53 | A | A | T | 2.02 | 4.461027e-01 |  |
| 335 | 54 | G | G | A | 11.70 | 1.962729e-04 | \* |
| 335 | 54 | G | G | C | 1.06 | 8.050274e-01 |  |
| 335 | 54 | G | G | G | 83.51 | 0.000000e+00 | \* |
| 335 | 54 | G | G | T | 3.72 | 1.154978e-01 |  |
| 336 | 55 | A | A | A | 93.81 | 0.000000e+00 | \* |
| 336 | 55 | A | A | C | 1.03 | 8.132514e-01 |  |
| 336 | 55 | A | A | G | 3.44 | 1.490485e-01 |  |
| 336 | 55 | A | A | T | 1.72 | 5.405650e-01 |  |
| 337 | 56 | A | A | A | 95.28 | 0.000000e+00 | \* |
| 337 | 56 | A | A | C | 0.79 | 8.679398e-01 |  |
| 337 | 56 | A | A | G | 1.57 | 4.819936e-01 |  |
| 337 | 56 | A | A | T | 2.36 | 3.496867e-01 |  |
| 338 | 57 | G | G | A | 7.27 | 8.634554e-03 | \* |
| 338 | 57 | G | G | C | 1.82 | 5.917078e-01 |  |
| 338 | 57 | G | G | G | 85.45 | 0.000000e+00 | \* |
| 338 | 57 | G | G | T | 5.45 | 2.287954e-02 |  |
| 339 | 58 | T | T | A | 2.44 | 3.322274e-01 |  |
| 339 | 58 | T | T | C | 1.63 | 6.478213e-01 |  |
| 339 | 58 | T | T | G | 1.22 | 5.830585e-01 |  |
| 339 | 58 | T | T | T | 94.72 | 0.000000e+00 | \* |
| 340 | 59 | T | T | A | 1.53 | 5.626696e-01 |  |
| 340 | 59 | T | T | C | 2.29 | 4.605347e-01 |  |
| 340 | 59 | T | T | G | 1.53 | 4.950385e-01 |  |
| 340 | 59 | T | T | T | 94.66 | 0.000000e+00 | \* |
| 341 | 60 | C | C | A | 1.47 | 5.789874e-01 |  |
| 341 | 60 | C | C | C | 93.01 | 0.000000e+00 | \* |
| 341 | 60 | C | C | G | 1.47 | 5.105357e-01 |  |
| 341 | 60 | C | C | T | 4.04 | 8.675843e-02 |  |
| 342 | 61 | G | G | A | 7.74 | 5.868766e-03 | \* |
| 342 | 61 | G | G | C | 6.55 | 1.878140e-02 |  |
| 342 | 61 | G | G | G | 82.74 | 0.000000e+00 | \* |
| 342 | 61 | G | G | T | 2.98 | 2.175305e-01 |  |
| 343 | 62 | A | A | A | 93.52 | 0.000000e+00 | \* |
| 343 | 62 | A | A | C | 2.47 | 4.149988e-01 |  |
| 343 | 62 | A | A | G | 2.16 | 3.417496e-01 |  |
| 343 | 62 | A | A | T | 1.85 | 4.970441e-01 |  |
| 344 | 63 | C | C | A | 5.65 | 3.203698e-02 |  |
| 344 | 63 | C | C | C | 91.17 | 0.000000e+00 | \* |
| 344 | 63 | C | C | G | 1.41 | 5.266049e-01 |  |
| 344 | 63 | C | C | T | 1.77 | 5.245537e-01 |  |
| 345 | 64 | A | A | A | 90.37 | 0.000000e+00 | \* |
| 345 | 64 | A | A | C | 4.97 | 6.982818e-02 |  |
| 345 | 64 | A | A | G | 2.48 | 2.792215e-01 |  |
| 345 | 64 | A | A | T | 2.17 | 4.002169e-01 |  |
| 346 | 65 | G | G | A | 5.51 | 3.592865e-02 |  |
| 346 | 65 | G | G | C | 3.81 | 1.691364e-01 |  |
| 346 | 65 | G | G | G | 87.71 | 0.000000e+00 | \* |
| 346 | 65 | G | G | T | 2.97 | 2.193155e-01 |  |
| 347 | 66 | C | C | A | 0.88 | 7.531916e-01 |  |
| 347 | 66 | C | C | C | 91.23 | 0.000000e+00 | \* |
| 347 | 66 | C | C | G | 3.51 | 1.418721e-01 |  |
| 347 | 66 | C | C | T | 4.39 | 6.345463e-02 |  |
| 348 | 67 | T | T | A | 1.42 | 5.942768e-01 |  |
| 348 | 67 | T | T | C | 4.96 | 7.007416e-02 |  |
| 348 | 67 | T | T | G | 0.71 | 7.387797e-01 |  |
| 348 | 67 | T | T | T | 92.91 | 0.000000e+00 | \* |
| 349 | 68 | C | C | A | 1.11 | 6.867147e-01 |  |
| 349 | 68 | C | C | C | 92.99 | 0.000000e+00 | \* |
| 349 | 68 | C | C | G | 1.48 | 5.090250e-01 |  |
| 349 | 68 | C | C | T | 4.43 | 6.102670e-02 |  |
| 350 | 69 | C | C | A | 2.76 | 2.704084e-01 |  |
| 350 | 69 | C | C | C | 93.45 | 0.000000e+00 | \* |
| 350 | 69 | C | C | G | 1.03 | 6.390835e-01 |  |
| 350 | 69 | C | C | T | 2.76 | 2.587869e-01 |  |
| 351 | 70 | C | C | A | 1.81 | 4.833429e-01 |  |
| 351 | 70 | C | C | C | 95.93 | 0.000000e+00 | \* |
| 351 | 70 | C | C | G | 2.26 | 3.209452e-01 |  |
| 351 | 70 | C | C | T | 0.00 | 9.574468e-01 |  |
| 352 | 71 | G | G | A | 4.95 | 5.549682e-02 |  |
| 352 | 71 | G | G | C | 6.44 | 2.067720e-02 |  |
| 352 | 71 | G | G | G | 88.61 | 0.000000e+00 | \* |
| 352 | 71 | G | G | T | 0.00 | 9.574468e-01 |  |
| 353 | 72 | C | C | A | 2.27 | 3.684134e-01 |  |
| 353 | 72 | C | C | C | 92.27 | 0.000000e+00 | \* |
| 353 | 72 | C | C | G | 4.55 | 6.865544e-02 |  |
| 353 | 72 | C | C | T | 0.91 | 8.139839e-01 |  |
| 354 | 73 | G | G | A | 6.32 | 1.886923e-02 |  |
| 354 | 73 | G | G | C | 4.21 | 1.259687e-01 |  |
| 354 | 73 | G | G | G | 86.32 | 0.000000e+00 | \* |
| 354 | 73 | G | G | T | 3.16 | 1.874082e-01 |  |
| 355 | 74 | A | A | A | 92.11 | 0.000000e+00 | \* |
| 355 | 74 | A | A | C | 2.87 | 3.243509e-01 |  |
| 355 | 74 | A | A | G | 3.23 | 1.718750e-01 |  |
| 355 | 74 | A | A | T | 1.79 | 5.162886e-01 |  |
| 356 | 75 | C | C | A | 3.92 | 1.202398e-01 |  |
| 356 | 75 | C | C | C | 92.94 | 0.000000e+00 | \* |
| 356 | 75 | C | C | G | 2.35 | 3.033543e-01 |  |
| 356 | 75 | C | C | T | 0.78 | 8.505451e-01 |  |

## For use in R

If you want to work with the results in R, here is output that you can copy and paste in your terminal to get:

The base information:

```
structure(list(focal.base = c("A", "C", "G", "T"), avg.percsignal = c(92.5589874922434, 
93.9946325823866, 92.6790942357569, 93.6168371567804), avg.areasignal = c(314.9375, 
354.231884057971, 322.789473684211, 359.348837209302), crit.perc.area = c(7.09462256734711, 
7.27103213080397, 7.15115411388922, 6.28524686650173), mu = c(2.26574616556795, 
2.55174072849796, 2.09317135701119, 2.16962279343269), fillibens = c(0.993665261066412, 
0.990684114112226, 0.997701142418655, 0.989109218556126)), .Names = c("focal.base", 
"avg.percsignal", "avg.areasignal", "crit.perc.area", "mu", "fillibens"
), row.names = c(NA, -4L), class = "data.frame")
```

the data.frame that contains information on the guide region:

```
structure(list(A.area = c(302, 331, 11, 607, 16, 17, 9, 8, 5, 
9, 194, 12, 471, 11, 12, 14, 5, 8, 2, 1, 0, 4, 2, 4, 5, 220, 
6, 13, 325, 8, 235, 6, 9, 1, 3, 5, 243, 6, 8, 8, 14, 8, 307, 
14, 12, 14, 339, 16, 16, 11, 9, 131, 237, 22, 273, 242, 12, 6, 
4, 4, 13, 303, 16, 291, 13, 2, 4, 3, 8, 4, 10, 5, 12, 257, 10
), C.area = c(8, 4, 4, 8, 341, 9, 7, 6, 234, 379, 9, 4, 9, 339, 
307, 6, 1, 5, 0, 3, 0, 2, 1, 7, 292, 11, 338, 10, 7, 299, 9, 
281, 9, 5, 5, 247, 11, 326, 310, 305, 13, 6, 9, 278, 263, 9, 
7, 235, 6, 4, 168, 4, 1, 2, 3, 2, 3, 4, 6, 253, 11, 8, 258, 16, 
9, 208, 14, 252, 271, 212, 13, 203, 8, 8, 237), G.area = c(2, 
6, 229, 6, 5, 13, 586, 311, 8, 5, 7, 295, 7, 5, 8, 311, 8, 461, 
12, 5, 9, 383, 7, 336, 8, 3, 1, 0, 3, 5, 6, 6, 229, 306, 222, 
6, 2, 2, 4, 4, 10, 408, 17, 7, 7, 234, 13, 6, 171, 262, 10, 2, 
5, 157, 10, 4, 141, 3, 4, 4, 139, 7, 4, 8, 207, 8, 2, 4, 3, 5, 
179, 10, 164, 9, 6), T.area = c(5, 4, 3, 6, 8, 334, 8, 1, 3, 
6, 103, 5, 5, 6, 4, 6, 238, 8, 279, 315, 325, 8, 275, 8, 6, 6, 
8, 314, 10, 4, 0, 0, 2, 3, 1, 5, 5, 4, 5, 7, 283, 8, 6, 3, 3, 
0, 4, 4, 3, 4, 0, 9, 5, 7, 5, 6, 9, 233, 248, 11, 5, 6, 5, 7, 
7, 10, 262, 12, 8, 0, 0, 2, 6, 5, 2), Tot.area = c(317, 345, 
247, 627, 370, 373, 610, 326, 250, 399, 313, 316, 492, 361, 331, 
337, 252, 482, 293, 324, 334, 397, 285, 355, 311, 240, 353, 337, 
345, 316, 250, 293, 249, 315, 231, 263, 261, 338, 327, 324, 320, 
430, 339, 302, 285, 257, 363, 261, 196, 281, 187, 146, 248, 188, 
291, 254, 165, 246, 262, 272, 168, 324, 283, 322, 236, 228, 282, 
271, 290, 221, 202, 220, 190, 279, 255), A.perc = c(95.2681388012618, 
95.9420289855072, 4.45344129554656, 96.8102073365231, 4.32432432432432, 
4.55764075067024, 1.47540983606557, 2.45398773006135, 2, 2.25563909774436, 
61.9808306709265, 3.79746835443038, 95.7317073170732, 3.04709141274238, 
3.62537764350453, 4.15430267062315, 1.98412698412698, 1.6597510373444, 
0.68259385665529, 0.308641975308642, 0, 1.00755667506297, 0.701754385964912, 
1.12676056338028, 1.60771704180064, 91.6666666666667, 1.69971671388102, 
3.85756676557863, 94.2028985507246, 2.53164556962025, 94, 2.04778156996587, 
3.6144578313253, 0.317460317460317, 1.2987012987013, 1.90114068441065, 
93.1034482758621, 1.77514792899408, 2.44648318042813, 2.46913580246914, 
4.375, 1.86046511627907, 90.5604719764012, 4.63576158940397, 
4.21052631578947, 5.44747081712062, 93.3884297520661, 6.13026819923372, 
8.16326530612245, 3.91459074733096, 4.81283422459893, 89.7260273972603, 
95.5645161290323, 11.7021276595745, 93.8144329896907, 95.2755905511811, 
7.27272727272727, 2.4390243902439, 1.52671755725191, 1.47058823529412, 
7.73809523809524, 93.5185185185185, 5.65371024734982, 90.3726708074534, 
5.50847457627119, 0.87719298245614, 1.41843971631206, 1.1070110701107, 
2.75862068965517, 1.80995475113122, 4.95049504950495, 2.27272727272727, 
6.31578947368421, 92.1146953405018, 3.92156862745098), C.perc = c(2.52365930599369, 
1.15942028985507, 1.61943319838057, 1.27591706539075, 92.1621621621622, 
2.41286863270777, 1.14754098360656, 1.84049079754601, 93.6, 94.9874686716792, 
2.87539936102236, 1.26582278481013, 1.82926829268293, 93.9058171745152, 
92.7492447129909, 1.78041543026706, 0.396825396825397, 1.03734439834025, 
0, 0.925925925925926, 0, 0.503778337531486, 0.350877192982456, 
1.97183098591549, 93.8906752411576, 4.58333333333333, 95.7507082152975, 
2.9673590504451, 2.02898550724638, 94.620253164557, 3.6, 95.9044368600683, 
3.6144578313253, 1.58730158730159, 2.16450216450216, 93.9163498098859, 
4.21455938697318, 96.4497041420118, 94.8012232415902, 94.1358024691358, 
4.0625, 1.3953488372093, 2.65486725663717, 92.0529801324503, 
92.280701754386, 3.50194552529183, 1.92837465564738, 90.0383141762452, 
3.06122448979592, 1.42348754448399, 89.8395721925134, 2.73972602739726, 
0.403225806451613, 1.06382978723404, 1.03092783505155, 0.78740157480315, 
1.81818181818182, 1.6260162601626, 2.29007633587786, 93.0147058823529, 
6.54761904761905, 2.46913580246914, 91.1660777385159, 4.96894409937888, 
3.8135593220339, 91.2280701754386, 4.9645390070922, 92.9889298892989, 
93.448275862069, 95.9276018099548, 6.43564356435644, 92.2727272727273, 
4.21052631578947, 2.8673835125448, 92.9411764705882), G.perc = c(0.630914826498423, 
1.73913043478261, 92.7125506072875, 0.956937799043062, 1.35135135135135, 
3.48525469168901, 96.0655737704918, 95.398773006135, 3.2, 1.2531328320802, 
2.23642172523962, 93.3544303797468, 1.42276422764228, 1.38504155124654, 
2.41691842900302, 92.2848664688427, 3.17460317460317, 95.6431535269709, 
4.09556313993174, 1.54320987654321, 2.69461077844311, 96.4735516372796, 
2.45614035087719, 94.6478873239437, 2.57234726688103, 1.25, 0.28328611898017, 
0, 0.869565217391304, 1.58227848101266, 2.4, 2.04778156996587, 
91.9678714859438, 97.1428571428571, 96.1038961038961, 2.28136882129278, 
0.766283524904215, 0.591715976331361, 1.22324159021407, 1.23456790123457, 
3.125, 94.8837209302326, 5.01474926253687, 2.31788079470199, 
2.45614035087719, 91.0505836575876, 3.58126721763085, 2.29885057471264, 
87.2448979591837, 93.2384341637011, 5.3475935828877, 1.36986301369863, 
2.01612903225806, 83.5106382978723, 3.43642611683849, 1.5748031496063, 
85.4545454545455, 1.21951219512195, 1.52671755725191, 1.47058823529412, 
82.7380952380952, 2.16049382716049, 1.41342756183746, 2.48447204968944, 
87.7118644067797, 3.50877192982456, 0.709219858156028, 1.4760147601476, 
1.03448275862069, 2.26244343891403, 88.6138613861386, 4.54545454545455, 
86.3157894736842, 3.2258064516129, 2.35294117647059), T.perc = c(1.57728706624606, 
1.15942028985507, 1.21457489878543, 0.956937799043062, 2.16216216216216, 
89.544235924933, 1.31147540983607, 0.306748466257669, 1.2, 1.50375939849624, 
32.9073482428115, 1.58227848101266, 1.01626016260163, 1.66204986149584, 
1.20845921450151, 1.78041543026706, 94.4444444444444, 1.6597510373444, 
95.221843003413, 97.2222222222222, 97.3053892215569, 2.01511335012594, 
96.4912280701754, 2.25352112676056, 1.92926045016077, 2.5, 2.26628895184136, 
93.1750741839763, 2.89855072463768, 1.26582278481013, 0, 0, 0.803212851405622, 
0.952380952380952, 0.432900432900433, 1.90114068441065, 1.91570881226054, 
1.18343195266272, 1.52905198776758, 2.16049382716049, 88.4375, 
1.86046511627907, 1.76991150442478, 0.993377483443709, 1.05263157894737, 
0, 1.10192837465565, 1.53256704980843, 1.53061224489796, 1.42348754448399, 
0, 6.16438356164384, 2.01612903225806, 3.72340425531915, 1.71821305841924, 
2.36220472440945, 5.45454545454545, 94.7154471544715, 94.6564885496183, 
4.04411764705882, 2.97619047619048, 1.85185185185185, 1.76678445229682, 
2.17391304347826, 2.96610169491525, 4.3859649122807, 92.9078014184397, 
4.4280442804428, 2.75862068965517, 0, 0, 0.909090909090909, 3.15789473684211, 
1.7921146953405, 0.784313725490196), base.call = c("A", "A", 
"G", "A", "C", "T", "G", "G", "C", "C", "A", "G", "A", "C", "C", 
"G", "T", "G", "T", "T", "T", "G", "T", "G", "C", "A", "C", "T", 
"A", "C", "A", "C", "G", "G", "G", "C", "A", "C", "C", "C", "T", 
"G", "A", "C", "C", "G", "A", "C", "G", "G", "C", "A", "A", "G", 
"A", "A", "G", "T", "T", "C", "G", "A", "C", "A", "G", "C", "T", 
"C", "C", "C", "G", "C", "G", "A", "C"), index = 282:356, guide.seq = c("A", 
"A", "G", "A", "C", "T", "G", "G", "C", "C", "A", "G", "A", "C", 
"C", "G", "T", "G", "T", "T", "T", "G", "T", "G", "C", "A", "C", 
"T", "A", "C", "A", "C", "G", "G", "G", "C", "A", "C", "C", "C", 
"T", "G", "A", "C", "C", "G", "A", "C", "G", "G", "C", "A", "A", 
"G", "A", "A", "G", "T", "T", "C", "G", "A", "C", "A", "G", "C", 
"T", "C", "C", "C", "G", "C", "G", "A", "C"), T.pval = c(0.588065404266183, 
0.732446788881247, 0.713585712472398, 0.799105274350141, 0.403525247922306, 
0, 0.680080463795599, 0.944740314938307, 0.718588898780269, 0.613326868682285, 
5.55111512312578e-16, 0.586360475620423, 0.780122324204444, 0.559324403815872, 
0.715686566040022, 0.520098543153601, 0, 0.560097434954162, 0, 
0, 0, 0.446408052583585, 0, 0.378279285073059, 0.472660917021227, 
0.31571548874721, 0.374838624611142, 0, 0.231582869961802, 0.69590850108525, 
0.957446808510052, 0.957446808510052, 0.845242294345692, 0.800539956562391, 
0.928511328954592, 0.481443046850904, 0.476882277091069, 0.724260496071985, 
0.604608877253068, 0.40399640156312, 0, 0.494299200901673, 0.523530143735732, 
0.787509205011081, 0.768230897168613, 0.957446808510052, 0.751855010107115, 
0.603399456438547, 0.604071973732972, 0.641136937968727, 0.957446808510052, 
0.0112973625465302, 0.44610267365433, 0.115497847941155, 0.540564965193332, 
0.349686736547505, 0.0228795426717047, 0, 0, 0.0867584270892757, 
0.217530459344791, 0.497044094029198, 0.524553719519532, 0.40021689716869, 
0.219315463714306, 0.063454632188977, 0, 0.0610267047063058, 
0.258786885816283, 0.957446808510052, 0.957446808510052, 0.813983867100138, 
0.187408183092525, 0.51628864425976, 0.850545138398131), C.pval = c(0.401698627301158, 
0.780245517714425, 0.649749069882549, 0.748563393258942, 0, 0.429006871663318, 
0.783391205369206, 0.585244609895949, 0, 0, 0.322683965687412, 
0.751362260450304, 0.588493910636794, 0, 0, 0.602683315882895, 
0.924938437046807, 0.8116609214236, 0.939024390243902, 0.838272857626311, 
0.939024390243902, 0.913725050044412, 0.928683271069299, 0.547590933595975, 
0, 0.0946377703279652, 0, 0.304010653469818, 0.531468427588506, 
0, 0.197253916319048, 0, 0.195232954091077, 0.659154415138149, 
0.494042722524741, 0, 0.125585314735404, 0, 0, 0, 0.140777004797234, 
0.714846150470218, 0.370830893616697, 0, 0, 0.211428116401669, 
0.559964333682107, 0, 0.285800366422984, 0.706769720908768, 0, 
0.35174407099727, 0.924365720308211, 0.805027397211832, 0.813251400613818, 
0.867939786719183, 0.591707845446396, 0.647821309055308, 0.460534654801811, 
0, 0.0187814000280919, 0.414998761857724, 0, 0.0698281836571442, 
0.169136353986675, 0, 0.070074156325693, 0, 0, 0, 0.0206771955053932, 
0, 0.125968703752448, 0.324350856541663, 0), G.pval = c(0.762058477049954, 
0.439106083221716, 0, 0.662925845035502, 0.544367137177761, 0.144169296280454, 
0, 0, 0.17488031933704, 0.573076916645257, 0.326155167159725, 
0, 0.523960953654238, 0.534688199821114, 0.291406573217333, 0, 
0.177884325113113, 0, 0.0944447011759773, 0.490539935616684, 
0.24405086875744, 0, 0.284278544539693, 0, 0.264019024381808, 
0.574003902974347, 0.852445718853374, 0.88888888888858, 0.689852049654644, 
0.479985277126618, 0.294527211774541, 0.365989152509218, 0, 0, 
0, 0.317198923127714, 0.721505624668318, 0.77346150335085, 0.581947698339118, 
0.578579606849319, 0.183886326623537, 0, 0.0489659271747437, 
0.310072594158789, 0.284278544539693, 0, 0.13500121239044, 0.313770266294731, 
0, 0, 0.0384198837605005, 0.539037618887772, 0.37303256483205, 
0, 0.149048508253338, 0.481993590918341, 0, 0.583058475352016, 
0.495038502014721, 0.510535689444666, 0, 0.341749577679595, 0.526604941014801, 
0.279221526031745, 0, 0.141872109481447, 0.738779749075324, 0.509025021340864, 
0.639083458638159, 0.320945245166032, 0, 0.0686554369964022, 
0, 0.17187497816944, 0.30335432487034), A.pval = c(0, 0, 0.081047221934777, 
0, 0.0892902279207245, 0.0749175365416058, 0.577579682972623, 
0.329107494398978, 0.433704401209536, 0.372288958600926, 0, 0.13158845606297, 
0, 0.222961499343347, 0.148927790459289, 0.101330877823277, 0.437725307896412, 
0.524710918676207, 0.805032283588843, 0.880277742184957, 0.901785714281768, 
0.71592003215092, 0.800201993898754, 0.680867069738849, 0.539425473057704, 
0, 0.513536656078502, 0.125976592746286, 0, 0.313274899946768, 
0, 0.421743706091076, 0.15009424767645, 0.879035591197266, 0.629721248499654, 
0.459124714478013, 0, 0.492772278530405, 0.33066942351195, 0.325971884763787, 
0.0859663658844679, 0.469839155214095, 0, 0.0706090116837266, 
0.0971926816404683, 0.037694613650195, 0, 0.0219093506288091, 
0.00411209638940446, 0.120853490638165, 0.0616877373858226, 0, 
0, 0.000196272945649612, 0, 0, 0.00863455368038735, 0.332227418121086, 
0.562669555854021, 0.578987418679157, 0.00586876625216604, 0, 
0.0320369765479639, 0, 0.0359286530967796, 0.753191611333976, 
0.594276784601678, 0.686714724185, 0.270408377708828, 0.483342914255701, 
0.0554968219657566, 0.368413446361825, 0.0188692315495229, 0, 
0.120239776955907), guide.position = 1:75), .Names = c("A.area", 
"C.area", "G.area", "T.area", "Tot.area", "A.perc", "C.perc", 
"G.perc", "T.perc", "base.call", "index", "guide.seq", "T.pval", 
"C.pval", "G.pval", "A.pval", "guide.position"), row.names = 282:356, class = "data.frame")
```

*Report generated using EditR v1.0.8*
