# Supplementary material for: Mechanistic and genetic basis of single-strand templated repair at Cas12a-induced DNA breaks in Chlamydomonas reinhardtii
Source: Nat Commun. 2021 Nov 19;12:6751. doi: 10.1038/s41467-021-27004-1 (PMC8604939; doi:10.1038/s41467-021-27004-1)
Supplement: Supplementary file 22 — Source Data [file 41467_2021_27004_MOESM22_ESM.zip › Source Data/EditR analysis/EditR outputs/Sense/rep2_ssODN_sense_-32_-16_0.html]

EditR v1.0.8 report


# EditR v1.0.8 report

- Data QA
  - Filtering data
  - Percent noise peak area
  - Base information
- Predicted editing
  - Editing bar plot
  - Editing table plot
  - Table of editing results
- For use in R

## Data QA

### Filtering data

What the data looked like prefiltering:

and the post filtering signal / noise plot:

### Percent noise peak area

### Base information

Here’s information about the signal of each base, the critical percent value where any higher value would be called as significant, and Filliben’s correlation for how well the noise was modelled by the zero adjusted gamma distribution.

| Base | Average percent signal | Average peak area | Critical percent value | model mu | Fillibens correlation |
| --- | --- | --- | --- | --- | --- |
| A | 89.06477 | 463.1515 | 9.951735 | 3.064004 | 0.9969064 |
| C | 90.40533 | 516.4638 | 9.452164 | 2.944922 | 0.9835546 |
| G | 91.27578 | 501.4821 | 12.172541 | 3.194248 | 0.9939437 |
| T | 93.35700 | 542.2791 | 8.987751 | 3.118776 | 0.9920240 |

## Predicted editing

### Editing bar plot

### Editing table plot

### Table of editing results


Here’s the entire guide region

| Sanger position | Guide position | Guide sequence | Sanger base call | Focal base | Focal base peak area | p value |  |
| --- | --- | --- | --- | --- | --- | --- | --- |
| 280 | 1 | A | A | A | 92.91 | 0.000000e+00 | \* |
| 280 | 1 | A | A | C | 1.82 | 5.999579e-01 |  |
| 280 | 1 | A | A | G | 1.21 | 6.620380e-01 |  |
| 280 | 1 | A | A | T | 4.05 | 2.286766e-01 |  |
| 281 | 2 | A | A | A | 91.50 | 0.000000e+00 | \* |
| 281 | 2 | A | A | C | 3.67 | 2.640354e-01 |  |
| 281 | 2 | A | A | G | 1.17 | 6.715551e-01 |  |
| 281 | 2 | A | A | T | 3.67 | 2.804128e-01 |  |
| 282 | 3 | G | G | A | 4.21 | 1.936260e-01 |  |
| 282 | 3 | G | G | C | 4.21 | 2.005058e-01 |  |
| 282 | 3 | G | G | G | 90.74 | 0.000000e+00 | \* |
| 282 | 3 | G | G | T | 0.84 | 8.279392e-01 |  |
| 283 | 4 | A | A | A | 93.82 | 0.000000e+00 | \* |
| 283 | 4 | A | A | C | 0.98 | 7.905024e-01 |  |
| 283 | 4 | A | A | G | 2.39 | 4.529125e-01 |  |
| 283 | 4 | A | A | T | 2.82 | 4.254066e-01 |  |
| 284 | 5 | C | C | A | 8.68 | 1.991940e-02 |  |
| 284 | 5 | C | C | C | 89.58 | 0.000000e+00 | \* |
| 284 | 5 | C | C | G | 0.00 | 8.791209e-01 |  |
| 284 | 5 | C | C | T | 1.74 | 6.573226e-01 |  |
| 285 | 6 | T | T | A | 0.00 | 8.018018e-01 |  |
| 285 | 6 | T | T | C | 4.28 | 1.938003e-01 |  |
| 285 | 6 | T | T | G | 2.63 | 4.160677e-01 |  |
| 285 | 6 | T | T | T | 93.09 | 0.000000e+00 | \* |
| 286 | 7 | G | G | A | 3.44 | 2.744860e-01 |  |
| 286 | 7 | G | G | C | 0.00 | 9.146341e-01 |  |
| 286 | 7 | G | G | G | 95.34 | 0.000000e+00 | \* |
| 286 | 7 | G | G | T | 1.22 | 7.654111e-01 |  |
| 287 | 8 | G | G | A | 5.21 | 1.197925e-01 |  |
| 287 | 8 | G | G | C | 2.54 | 4.483962e-01 |  |
| 287 | 8 | G | G | G | 91.97 | 0.000000e+00 | \* |
| 287 | 8 | G | G | T | 0.28 | 8.788046e-01 |  |
| 288 | 9 | C | C | A | 5.15 | 1.236240e-01 |  |
| 288 | 9 | C | C | C | 86.27 | 0.000000e+00 | \* |
| 288 | 9 | C | C | G | 4.90 | 1.811052e-01 |  |
| 288 | 9 | C | C | T | 3.68 | 2.789795e-01 |  |
| 289 | 10 | C | C | A | 4.01 | 2.129063e-01 |  |
| 289 | 10 | C | C | C | 93.37 | 0.000000e+00 | \* |
| 289 | 10 | C | C | G | 0.00 | 8.791209e-01 |  |
| 289 | 10 | C | C | T | 2.62 | 4.655185e-01 |  |
| 290 | 11 | A | A | A | 50.91 | 2.882139e-13 | \* |
| 290 | 11 | A | A | C | 3.42 | 2.979035e-01 |  |
| 290 | 11 | A | A | G | 2.21 | 4.803737e-01 |  |
| 290 | 11 | A | A | T | 43.46 | 1.898481e-14 | \* |
| 291 | 12 | G | G | A | 3.61 | 2.549147e-01 |  |
| 291 | 12 | G | G | C | 2.67 | 4.228033e-01 |  |
| 291 | 12 | G | G | G | 91.99 | 0.000000e+00 | \* |
| 291 | 12 | G | G | T | 1.73 | 6.593569e-01 |  |
| 292 | 13 | A | A | A | 91.59 | 0.000000e+00 | \* |
| 292 | 13 | A | A | C | 1.10 | 7.645447e-01 |  |
| 292 | 13 | A | A | G | 3.29 | 3.290591e-01 |  |
| 292 | 13 | A | A | T | 4.02 | 2.317086e-01 |  |
| 293 | 14 | C | C | A | 8.25 | 2.509072e-02 |  |
| 293 | 14 | C | C | C | 89.56 | 0.000000e+00 | \* |
| 293 | 14 | C | C | G | 0.51 | 8.034710e-01 |  |
| 293 | 14 | C | C | T | 1.68 | 6.688429e-01 |  |
| 294 | 15 | C | C | A | 0.00 | 8.018018e-01 |  |
| 294 | 15 | C | C | C | 96.90 | 0.000000e+00 | \* |
| 294 | 15 | C | C | G | 1.55 | 5.969985e-01 |  |
| 294 | 15 | C | C | T | 1.55 | 6.975873e-01 |  |
| 295 | 16 | G | G | A | 4.27 | 1.881459e-01 |  |
| 295 | 16 | G | G | C | 4.08 | 2.146660e-01 |  |
| 295 | 16 | G | G | G | 90.49 | 0.000000e+00 | \* |
| 295 | 16 | G | G | T | 1.17 | 7.748937e-01 |  |
| 296 | 17 | T | T | A | 0.00 | 8.018018e-01 |  |
| 296 | 17 | T | T | C | 2.62 | 4.324514e-01 |  |
| 296 | 17 | T | T | G | 3.93 | 2.610332e-01 |  |
| 296 | 17 | T | T | T | 93.46 | 0.000000e+00 | \* |
| 297 | 18 | G | G | A | 2.62 | 3.892208e-01 |  |
| 297 | 18 | G | G | C | 0.00 | 9.146341e-01 |  |
| 297 | 18 | G | G | G | 93.83 | 0.000000e+00 | \* |
| 297 | 18 | G | G | T | 3.54 | 2.989311e-01 |  |
| 298 | 19 | T | T | A | 0.58 | 7.523143e-01 |  |
| 298 | 19 | T | T | C | 0.97 | 7.908118e-01 |  |
| 298 | 19 | T | T | G | 6.04 | 1.168433e-01 |  |
| 298 | 19 | T | T | T | 92.40 | 0.000000e+00 | \* |
| 299 | 20 | T | T | A | 0.00 | 8.018018e-01 |  |
| 299 | 20 | T | T | C | 0.00 | 9.146341e-01 |  |
| 299 | 20 | T | T | G | 0.57 | 7.908943e-01 |  |
| 299 | 20 | T | T | T | 99.43 | 0.000000e+00 | \* |
| 300 | 21 | T | T | A | 0.00 | 8.018018e-01 |  |
| 300 | 21 | T | T | C | 2.20 | 5.164205e-01 |  |
| 300 | 21 | T | T | G | 2.39 | 4.530361e-01 |  |
| 300 | 21 | T | T | T | 95.41 | 0.000000e+00 | \* |
| 301 | 22 | G | G | A | 2.16 | 4.681153e-01 |  |
| 301 | 22 | G | G | C | 3.16 | 3.373267e-01 |  |
| 301 | 22 | G | G | G | 91.95 | 0.000000e+00 | \* |
| 301 | 22 | G | G | T | 2.73 | 4.431809e-01 |  |
| 302 | 23 | T | T | A | 0.22 | 7.926824e-01 |  |
| 302 | 23 | T | T | C | 1.35 | 7.084106e-01 |  |
| 302 | 23 | T | T | G | 4.94 | 1.782486e-01 |  |
| 302 | 23 | T | T | T | 93.48 | 0.000000e+00 | \* |
| 303 | 24 | G | G | A | 2.50 | 4.100175e-01 |  |
| 303 | 24 | G | G | C | 1.07 | 7.706317e-01 |  |
| 303 | 24 | G | G | G | 91.80 | 0.000000e+00 | \* |
| 303 | 24 | G | G | T | 4.63 | 1.646654e-01 |  |
| 304 | 25 | C | C | A | 1.86 | 5.226224e-01 |  |
| 304 | 25 | C | C | C | 92.16 | 0.000000e+00 | \* |
| 304 | 25 | C | C | G | 0.00 | 8.791209e-01 |  |
| 304 | 25 | C | C | T | 5.98 | 7.331874e-02 |  |
| 305 | 26 | A | A | A | 86.47 | 0.000000e+00 | \* |
| 305 | 26 | A | A | C | 2.76 | 4.064649e-01 |  |
| 305 | 26 | A | A | G | 6.27 | 1.071478e-01 |  |
| 305 | 26 | A | A | T | 4.51 | 1.766950e-01 |  |
| 306 | 27 | C | C | A | 4.50 | 1.691924e-01 |  |
| 306 | 27 | C | C | C | 66.81 | 0.000000e+00 | \* |
| 306 | 27 | C | C | G | 26.34 | 2.669045e-05 | \* |
| 306 | 27 | C | C | T | 2.36 | 5.209902e-01 |  |
| 307 | 28 | T | T | A | 1.57 | 5.767180e-01 |  |
| 307 | 28 | T | T | C | 5.16 | 1.213761e-01 |  |
| 307 | 28 | T | T | G | 0.22 | 8.530423e-01 |  |
| 307 | 28 | T | T | T | 93.05 | 0.000000e+00 | \* |
| 308 | 29 | A | A | A | 94.34 | 0.000000e+00 | \* |
| 308 | 29 | A | A | C | 5.42 | 1.048599e-01 |  |
| 308 | 29 | A | A | G | 0.24 | 8.512132e-01 |  |
| 308 | 29 | A | A | T | 0.00 | 8.829787e-01 |  |
| 309 | 30 | C | C | A | 4.93 | 1.371808e-01 |  |
| 309 | 30 | C | C | C | 89.75 | 0.000000e+00 | \* |
| 309 | 30 | C | C | G | 1.33 | 6.396613e-01 |  |
| 309 | 30 | C | C | T | 3.98 | 2.367381e-01 |  |
| 310 | 31 | A | A | A | 87.25 | 0.000000e+00 | \* |
| 310 | 31 | A | A | C | 5.25 | 1.153792e-01 |  |
| 310 | 31 | A | A | G | 6.00 | 1.188037e-01 |  |
| 310 | 31 | A | A | T | 1.50 | 7.082690e-01 |  |
| 311 | 32 | C | C | A | 3.59 | 2.569650e-01 |  |
| 311 | 32 | C | C | C | 89.62 | 0.000000e+00 | \* |
| 311 | 32 | C | C | G | 3.99 | 2.548022e-01 |  |
| 311 | 32 | C | C | T | 2.79 | 4.304061e-01 |  |
| 312 | 33 | G | G | A | 4.15 | 1.996285e-01 |  |
| 312 | 33 | G | G | C | 9.59 | 9.220299e-03 | \* |
| 312 | 33 | G | G | G | 86.01 | 2.220446e-16 | \* |
| 312 | 33 | G | G | T | 0.26 | 8.795923e-01 |  |
| 313 | 34 | G | G | A | 0.86 | 7.088773e-01 |  |
| 313 | 34 | G | G | C | 2.23 | 5.096852e-01 |  |
| 313 | 34 | G | G | G | 96.56 | 0.000000e+00 | \* |
| 313 | 34 | G | G | T | 0.34 | 8.761538e-01 |  |
| 314 | 35 | G | G | A | 1.24 | 6.386937e-01 |  |
| 314 | 35 | G | G | C | 2.28 | 4.995220e-01 |  |
| 314 | 35 | G | G | G | 93.98 | 0.000000e+00 | \* |
| 314 | 35 | G | G | T | 2.49 | 4.924557e-01 |  |
| 315 | 36 | C | C | A | 4.57 | 1.636951e-01 |  |
| 315 | 36 | C | C | C | 87.90 | 0.000000e+00 | \* |
| 315 | 36 | C | C | G | 4.11 | 2.439272e-01 |  |
| 315 | 36 | C | C | T | 3.42 | 3.175907e-01 |  |
| 316 | 37 | A | A | A | 90.07 | 0.000000e+00 | \* |
| 316 | 37 | A | A | C | 5.44 | 1.041230e-01 |  |
| 316 | 37 | A | A | G | 0.00 | 8.791209e-01 |  |
| 316 | 37 | A | A | T | 4.49 | 1.786699e-01 |  |
| 317 | 38 | C | C | A | 5.24 | 1.182241e-01 |  |
| 317 | 38 | C | C | C | 90.48 | 0.000000e+00 | \* |
| 317 | 38 | C | C | G | 1.11 | 6.826461e-01 |  |
| 317 | 38 | C | C | T | 3.17 | 3.596376e-01 |  |
| 318 | 39 | C | C | A | 3.66 | 2.492594e-01 |  |
| 318 | 39 | C | C | C | 91.68 | 0.000000e+00 | \* |
| 318 | 39 | C | C | G | 2.50 | 4.361307e-01 |  |
| 318 | 39 | C | C | T | 2.16 | 5.628648e-01 |  |
| 319 | 40 | C | C | A | 4.73 | 1.514581e-01 |  |
| 319 | 40 | C | C | C | 91.07 | 0.000000e+00 | \* |
| 319 | 40 | C | C | G | 0.00 | 8.791209e-01 |  |
| 319 | 40 | C | C | T | 4.20 | 2.100602e-01 |  |
| 320 | 41 | T | T | A | 1.09 | 6.671179e-01 |  |
| 320 | 41 | T | T | C | 7.83 | 2.642853e-02 |  |
| 320 | 41 | T | T | G | 2.00 | 5.153579e-01 |  |
| 320 | 41 | T | T | T | 89.07 | 0.000000e+00 | \* |
| 321 | 42 | G | G | A | 3.65 | 2.508359e-01 |  |
| 321 | 42 | G | G | C | 0.00 | 9.146341e-01 |  |
| 321 | 42 | G | G | G | 91.44 | 0.000000e+00 | \* |
| 321 | 42 | G | G | T | 4.91 | 1.404189e-01 |  |
| 322 | 43 | A | A | A | 53.27 | 6.816769e-14 | \* |
| 322 | 43 | A | A | C | 1.50 | 6.747003e-01 |  |
| 322 | 43 | A | A | G | 7.66 | 6.183530e-02 |  |
| 322 | 43 | A | A | T | 37.57 | 2.304601e-12 | \* |
| 323 | 44 | C | C | A | 6.67 | 5.754540e-02 |  |
| 323 | 44 | C | C | C | 87.65 | 0.000000e+00 | \* |
| 323 | 44 | C | C | G | 0.00 | 8.791209e-01 |  |
| 323 | 44 | C | C | T | 5.69 | 8.794694e-02 |  |
| 324 | 45 | C | C | A | 0.00 | 8.018018e-01 |  |
| 324 | 45 | C | C | C | 95.35 | 0.000000e+00 | \* |
| 324 | 45 | C | C | G | 4.65 | 1.991390e-01 |  |
| 324 | 45 | C | C | T | 0.00 | 8.829787e-01 |  |
| 325 | 46 | G | G | A | 3.54 | 2.636537e-01 |  |
| 325 | 46 | G | G | C | 6.82 | 4.778357e-02 |  |
| 325 | 46 | G | G | G | 87.12 | 1.110223e-16 | \* |
| 325 | 46 | G | G | T | 2.53 | 4.849918e-01 |  |
| 326 | 47 | A | A | A | 92.37 | 0.000000e+00 | \* |
| 326 | 47 | A | A | C | 0.76 | 8.327473e-01 |  |
| 326 | 47 | A | A | G | 3.18 | 3.425916e-01 |  |
| 326 | 47 | A | A | T | 3.69 | 2.770735e-01 |  |
| 327 | 48 | C | C | A | 11.73 | 3.742509e-03 | \* |
| 327 | 48 | C | C | C | 83.85 | 0.000000e+00 | \* |
| 327 | 48 | C | C | G | 0.77 | 7.514798e-01 |  |
| 327 | 48 | C | C | T | 3.65 | 2.822957e-01 |  |
| 328 | 49 | G | G | A | 0.00 | 8.018018e-01 |  |
| 328 | 49 | G | G | C | 8.04 | 2.339148e-02 |  |
| 328 | 49 | G | G | G | 90.03 | 0.000000e+00 | \* |
| 328 | 49 | G | G | T | 1.93 | 6.146104e-01 |  |
| 329 | 50 | G | G | A | 3.17 | 3.096927e-01 |  |
| 329 | 50 | G | G | C | 0.70 | 8.435178e-01 |  |
| 329 | 50 | G | G | G | 94.37 | 0.000000e+00 | \* |
| 329 | 50 | G | G | T | 1.76 | 6.519455e-01 |  |
| 330 | 51 | C | C | A | 2.27 | 4.475394e-01 |  |
| 330 | 51 | C | C | C | 87.78 | 0.000000e+00 | \* |
| 330 | 51 | C | C | G | 9.94 | 2.479487e-02 |  |
| 330 | 51 | C | C | T | 0.00 | 8.829787e-01 |  |
| 331 | 52 | A | A | A | 89.93 | 0.000000e+00 | \* |
| 331 | 52 | A | A | C | 4.48 | 1.744915e-01 |  |
| 331 | 52 | A | A | G | 0.37 | 8.279191e-01 |  |
| 331 | 52 | A | A | T | 5.22 | 1.164858e-01 |  |
| 332 | 53 | A | A | A | 96.05 | 0.000000e+00 | \* |
| 332 | 53 | A | A | C | 0.00 | 9.146341e-01 |  |
| 332 | 53 | A | A | G | 0.88 | 7.297376e-01 |  |
| 332 | 53 | A | A | T | 3.07 | 3.782751e-01 |  |
| 333 | 54 | G | G | A | 7.77 | 3.236471e-02 |  |
| 333 | 54 | G | G | C | 3.51 | 2.853751e-01 |  |
| 333 | 54 | G | G | G | 88.72 | 1.110223e-16 | \* |
| 333 | 54 | G | G | T | 0.00 | 8.829787e-01 |  |
| 334 | 55 | A | A | A | 90.04 | 0.000000e+00 | \* |
| 334 | 55 | A | A | C | 0.00 | 9.146341e-01 |  |
| 334 | 55 | A | A | G | 5.86 | 1.254557e-01 |  |
| 334 | 55 | A | A | T | 4.10 | 2.221493e-01 |  |
| 335 | 56 | A | A | A | 94.27 | 0.000000e+00 | \* |
| 335 | 56 | A | A | C | 0.00 | 9.146341e-01 |  |
| 335 | 56 | A | A | G | 0.64 | 7.778428e-01 |  |
| 335 | 56 | A | A | T | 5.10 | 1.257592e-01 |  |
| 336 | 57 | G | G | A | 8.00 | 2.864566e-02 |  |
| 336 | 57 | G | G | C | 2.15 | 5.266583e-01 |  |
| 336 | 57 | G | G | G | 85.23 | 3.330669e-16 | \* |
| 336 | 57 | G | G | T | 4.62 | 1.664907e-01 |  |
| 337 | 58 | T | T | A | 0.00 | 8.018018e-01 |  |
| 337 | 58 | T | T | C | 1.27 | 7.264169e-01 |  |
| 337 | 58 | T | T | G | 0.00 | 8.791209e-01 |  |
| 337 | 58 | T | T | T | 98.73 | 0.000000e+00 | \* |
| 338 | 59 | T | T | A | 1.32 | 6.246698e-01 |  |
| 338 | 59 | T | T | C | 1.54 | 6.647762e-01 |  |
| 338 | 59 | T | T | G | 3.96 | 2.582170e-01 |  |
| 338 | 59 | T | T | T | 93.19 | 0.000000e+00 | \* |
| 339 | 60 | C | C | A | 4.99 | 1.334982e-01 |  |
| 339 | 60 | C | C | C | 88.57 | 0.000000e+00 | \* |
| 339 | 60 | C | C | G | 0.83 | 7.389320e-01 |  |
| 339 | 60 | C | C | T | 5.61 | 9.198114e-02 |  |
| 340 | 61 | G | G | A | 7.89 | 3.028935e-02 |  |
| 340 | 61 | G | G | C | 5.85 | 8.291752e-02 |  |
| 340 | 61 | G | G | G | 83.33 | 5.551115e-16 | \* |
| 340 | 61 | G | G | T | 2.92 | 4.054006e-01 |  |
| 341 | 62 | A | A | A | 90.85 | 0.000000e+00 | \* |
| 341 | 62 | A | A | C | 1.66 | 6.359534e-01 |  |
| 341 | 62 | A | A | G | 4.83 | 1.864489e-01 |  |
| 341 | 62 | A | A | T | 2.66 | 4.567934e-01 |  |
| 342 | 63 | C | C | A | 5.48 | 1.048049e-01 |  |
| 342 | 63 | C | C | C | 91.12 | 0.000000e+00 | \* |
| 342 | 63 | C | C | G | 0.57 | 7.915230e-01 |  |
| 342 | 63 | C | C | T | 2.84 | 4.223735e-01 |  |
| 343 | 64 | A | A | A | 86.30 | 0.000000e+00 | \* |
| 343 | 64 | A | A | C | 8.16 | 2.172135e-02 |  |
| 343 | 64 | A | A | G | 2.04 | 5.090217e-01 |  |
| 343 | 64 | A | A | T | 3.50 | 3.058732e-01 |  |
| 344 | 65 | G | G | A | 5.00 | 1.328248e-01 |  |
| 344 | 65 | G | G | C | 3.46 | 2.920264e-01 |  |
| 344 | 65 | G | G | G | 91.35 | 0.000000e+00 | \* |
| 344 | 65 | G | G | T | 0.19 | 8.813835e-01 |  |
| 345 | 66 | C | C | A | 2.42 | 4.220272e-01 |  |
| 345 | 66 | C | C | C | 89.87 | 0.000000e+00 | \* |
| 345 | 66 | C | C | G | 4.85 | 1.850041e-01 |  |
| 345 | 66 | C | C | T | 2.86 | 4.169748e-01 |  |
| 346 | 67 | T | T | A | 0.00 | 8.018018e-01 |  |
| 346 | 67 | T | T | C | 6.65 | 5.249512e-02 |  |
| 346 | 67 | T | T | G | 1.80 | 5.513188e-01 |  |
| 346 | 67 | T | T | T | 91.55 | 0.000000e+00 | \* |
| 347 | 68 | C | C | A | 0.98 | 6.879429e-01 |  |
| 347 | 68 | C | C | C | 88.26 | 0.000000e+00 | \* |
| 347 | 68 | C | C | G | 3.13 | 3.487302e-01 |  |
| 347 | 68 | C | C | T | 7.63 | 2.513967e-02 |  |
| 348 | 69 | C | C | A | 1.73 | 5.457748e-01 |  |
| 348 | 69 | C | C | C | 92.60 | 0.000000e+00 | \* |
| 348 | 69 | C | C | G | 0.00 | 8.791209e-01 |  |
| 348 | 69 | C | C | T | 5.67 | 8.887122e-02 |  |
| 349 | 70 | C | C | A | 3.56 | 2.612859e-01 |  |
| 349 | 70 | C | C | C | 90.44 | 0.000000e+00 | \* |
| 349 | 70 | C | C | G | 1.56 | 5.960247e-01 |  |
| 349 | 70 | C | C | T | 4.44 | 1.835235e-01 |  |
| 350 | 71 | G | G | A | 3.86 | 2.281022e-01 |  |
| 350 | 71 | G | G | C | 9.25 | 1.127552e-02 |  |
| 350 | 71 | G | G | G | 84.83 | 3.330669e-16 | \* |
| 350 | 71 | G | G | T | 2.06 | 5.863742e-01 |  |
| 351 | 72 | C | C | A | 5.25 | 1.174682e-01 |  |
| 351 | 72 | C | C | C | 88.13 | 0.000000e+00 | \* |
| 351 | 72 | C | C | G | 6.62 | 9.326384e-02 |  |
| 351 | 72 | C | C | T | 0.00 | 8.829787e-01 |  |
| 352 | 73 | G | G | A | 10.03 | 9.603369e-03 | \* |
| 352 | 73 | G | G | C | 9.77 | 8.245273e-03 | \* |
| 352 | 73 | G | G | G | 79.69 | 2.886580e-15 | \* |
| 352 | 73 | G | G | T | 0.51 | 8.649818e-01 |  |
| 353 | 74 | A | A | A | 89.56 | 0.000000e+00 | \* |
| 353 | 74 | A | A | C | 3.04 | 3.576775e-01 |  |
| 353 | 74 | A | A | G | 5.69 | 1.338061e-01 |  |
| 353 | 74 | A | A | T | 1.71 | 6.635355e-01 |  |
| 354 | 75 | C | C | A | 5.83 | 8.800554e-02 |  |
| 354 | 75 | C | C | C | 86.96 | 0.000000e+00 | \* |
| 354 | 75 | C | C | G | 2.06 | 5.060580e-01 |  |
| 354 | 75 | C | C | T | 5.15 | 1.220523e-01 |  |

## For use in R

If you want to work with the results in R, here is output that you can copy and paste in your terminal to get:

The base information:

```
structure(list(focal.base = c("A", "C", "G", "T"), avg.percsignal = c(89.0647683912868, 
90.405333058656, 91.2757840224255, 93.3569961499486), avg.areasignal = c(463.151515151515, 
516.463768115942, 501.482142857143, 542.279069767442), crit.perc.area = c(9.95173489304424, 
9.45216425021705, 12.1725412138716, 8.98775141513151), mu = c(3.06400420356662, 
2.94492196455637, 3.19424832831701, 3.11877600306325), fillibens = c(0.996906362102306, 
0.983554646816425, 0.993943699430722, 0.99202396876765)), .Names = c("focal.base", 
"avg.percsignal", "avg.areasignal", "crit.perc.area", "mu", "fillibens"
), row.names = c(NA, -4L), class = "data.frame")
```

the data.frame that contains information on the guide region:

```
structure(list(A.area = c(459, 549, 20, 865, 50, 0, 34, 37, 21, 
26, 253, 23, 751, 49, 0, 22, 0, 20, 3, 0, 0, 15, 1, 14, 9, 345, 
21, 7, 400, 26, 349, 18, 16, 5, 6, 20, 381, 33, 22, 27, 6, 26, 
285, 34, 0, 14, 726, 61, 0, 18, 8, 241, 438, 31, 461, 444, 26, 
0, 6, 24, 27, 546, 29, 296, 26, 11, 0, 5, 11, 16, 15, 23, 39, 
472, 34), C.area = c(9, 22, 20, 9, 516, 26, 0, 18, 352, 606, 
17, 17, 9, 532, 500, 21, 10, 0, 5, 0, 12, 22, 6, 6, 447, 11, 
312, 23, 23, 473, 21, 449, 37, 13, 11, 385, 23, 570, 551, 520, 
43, 0, 8, 447, 451, 27, 6, 436, 25, 4, 309, 12, 0, 14, 0, 0, 
7, 5, 7, 426, 20, 10, 482, 28, 18, 408, 37, 451, 588, 407, 36, 
386, 38, 16, 507), G.area = c(6, 7, 431, 22, 0, 16, 941, 653, 
20, 0, 11, 586, 27, 3, 8, 466, 15, 715, 31, 3, 13, 640, 22, 515, 
0, 25, 123, 1, 1, 7, 24, 20, 332, 562, 453, 18, 0, 7, 15, 0, 
11, 652, 41, 0, 22, 345, 25, 4, 280, 536, 35, 1, 4, 354, 30, 
3, 277, 0, 18, 4, 285, 29, 3, 7, 475, 22, 10, 16, 0, 7, 330, 
29, 310, 30, 12), T.area = c(20, 22, 4, 26, 10, 566, 12, 2, 15, 
17, 216, 11, 33, 10, 8, 6, 357, 27, 474, 523, 520, 19, 416, 26, 
29, 18, 11, 415, 0, 21, 6, 14, 1, 2, 12, 15, 19, 20, 13, 24, 
489, 35, 201, 29, 0, 10, 29, 19, 6, 10, 0, 14, 14, 0, 21, 24, 
15, 389, 424, 27, 10, 16, 15, 12, 1, 13, 509, 39, 36, 20, 8, 
0, 2, 9, 30), Tot.area = c(494, 600, 475, 922, 576, 608, 987, 
710, 408, 649, 497, 637, 820, 594, 516, 515, 382, 762, 513, 526, 
545, 696, 445, 561, 485, 399, 467, 446, 424, 527, 400, 501, 386, 
582, 482, 438, 423, 630, 601, 571, 549, 713, 535, 510, 473, 396, 
786, 520, 311, 568, 352, 268, 456, 399, 512, 471, 325, 394, 455, 
481, 342, 601, 529, 343, 520, 454, 556, 511, 635, 450, 389, 438, 
389, 527, 583), A.perc = c(92.914979757085, 91.5, 4.21052631578947, 
93.8177874186551, 8.68055555555556, 0, 3.44478216818642, 5.2112676056338, 
5.14705882352941, 4.00616332819723, 50.9054325955734, 3.61067503924647, 
91.5853658536585, 8.24915824915825, 0, 4.27184466019417, 0, 2.6246719160105, 
0.584795321637427, 0, 0, 2.1551724137931, 0.224719101123595, 
2.49554367201426, 1.85567010309278, 86.4661654135338, 4.49678800856531, 
1.5695067264574, 94.3396226415094, 4.93358633776091, 87.25, 3.59281437125748, 
4.14507772020725, 0.859106529209622, 1.2448132780083, 4.5662100456621, 
90.0709219858156, 5.23809523809524, 3.66056572379368, 4.72854640980736, 
1.09289617486339, 3.64656381486676, 53.2710280373832, 6.66666666666667, 
0, 3.53535353535354, 92.3664122137405, 11.7307692307692, 0, 3.16901408450704, 
2.27272727272727, 89.9253731343284, 96.0526315789474, 7.76942355889724, 
90.0390625, 94.2675159235669, 8, 0, 1.31868131868132, 4.98960498960499, 
7.89473684210526, 90.8485856905158, 5.4820415879017, 86.2973760932945, 
5, 2.42290748898678, 0, 0.978473581213307, 1.73228346456693, 
3.55555555555556, 3.8560411311054, 5.25114155251142, 10.025706940874, 
89.5635673624288, 5.83190394511149), C.perc = c(1.82186234817814, 
3.66666666666667, 4.21052631578947, 0.976138828633406, 89.5833333333333, 
4.27631578947368, 0, 2.53521126760563, 86.2745098039216, 93.3744221879815, 
3.420523138833, 2.66875981161695, 1.09756097560976, 89.5622895622896, 
96.8992248062015, 4.07766990291262, 2.61780104712042, 0, 0.974658869395711, 
0, 2.20183486238532, 3.16091954022989, 1.34831460674157, 1.06951871657754, 
92.1649484536082, 2.75689223057644, 66.8094218415418, 5.15695067264574, 
5.42452830188679, 89.753320683112, 5.25, 89.6207584830339, 9.58549222797927, 
2.23367697594502, 2.28215767634855, 87.8995433789954, 5.43735224586288, 
90.4761904761905, 91.6805324459235, 91.0683012259194, 7.83242258652095, 
0, 1.49532710280374, 87.6470588235294, 95.3488372093023, 6.81818181818182, 
0.763358778625954, 83.8461538461538, 8.03858520900322, 0.704225352112676, 
87.7840909090909, 4.47761194029851, 0, 3.50877192982456, 0, 0, 
2.15384615384615, 1.26903553299492, 1.53846153846154, 88.5654885654886, 
5.84795321637427, 1.66389351081531, 91.1153119092628, 8.16326530612245, 
3.46153846153846, 89.8678414096916, 6.65467625899281, 88.2583170254403, 
92.5984251968504, 90.4444444444444, 9.25449871465296, 88.1278538812785, 
9.76863753213368, 3.03605313092979, 86.9639794168096), G.perc = c(1.21457489878543, 
1.16666666666667, 90.7368421052632, 2.38611713665944, 0, 2.63157894736842, 
95.339412360689, 91.9718309859155, 4.90196078431373, 0, 2.21327967806841, 
91.9937205651491, 3.29268292682927, 0.505050505050505, 1.55038759689922, 
90.4854368932039, 3.92670157068063, 93.8320209973753, 6.04288499025341, 
0.570342205323194, 2.38532110091743, 91.9540229885057, 4.9438202247191, 
91.8003565062389, 0, 6.265664160401, 26.338329764454, 0.224215246636771, 
0.235849056603774, 1.32827324478178, 6, 3.99201596806387, 86.0103626943005, 
96.5635738831615, 93.9834024896266, 4.10958904109589, 0, 1.11111111111111, 
2.49584026622296, 0, 2.00364298724954, 91.4446002805049, 7.66355140186916, 
0, 4.65116279069767, 87.1212121212121, 3.18066157760814, 0.769230769230769, 
90.032154340836, 94.3661971830986, 9.94318181818182, 0.373134328358209, 
0.87719298245614, 88.7218045112782, 5.859375, 0.636942675159236, 
85.2307692307692, 0, 3.95604395604396, 0.831600831600832, 83.3333333333333, 
4.82529118136439, 0.56710775047259, 2.04081632653061, 91.3461538461538, 
4.84581497797357, 1.79856115107914, 3.13111545988258, 0, 1.55555555555556, 
84.8329048843188, 6.62100456621005, 79.6915167095116, 5.69259962049336, 
2.05831903945111), T.perc = c(4.04858299595142, 3.66666666666667, 
0.842105263157895, 2.81995661605206, 1.73611111111111, 93.0921052631579, 
1.21580547112462, 0.28169014084507, 3.67647058823529, 2.61941448382126, 
43.4607645875251, 1.72684458398744, 4.02439024390244, 1.68350168350168, 
1.55038759689922, 1.16504854368932, 93.455497382199, 3.54330708661417, 
92.3976608187134, 99.4296577946768, 95.4128440366973, 2.72988505747126, 
93.4831460674157, 4.63458110516934, 5.97938144329897, 4.51127819548872, 
2.35546038543897, 93.0493273542601, 0, 3.98481973434535, 1.5, 
2.79441117764471, 0.259067357512953, 0.343642611683849, 2.4896265560166, 
3.42465753424658, 4.49172576832151, 3.17460317460317, 2.1630615640599, 
4.2031523642732, 89.0710382513661, 4.90883590462833, 37.5700934579439, 
5.68627450980392, 0, 2.52525252525253, 3.68956743002545, 3.65384615384615, 
1.92926045016077, 1.76056338028169, 0, 5.22388059701493, 3.07017543859649, 
0, 4.1015625, 5.09554140127389, 4.61538461538461, 98.7309644670051, 
93.1868131868132, 5.61330561330561, 2.92397660818713, 2.66222961730449, 
2.83553875236295, 3.49854227405248, 0.192307692307692, 2.86343612334802, 
91.5467625899281, 7.6320939334638, 5.66929133858268, 4.44444444444444, 
2.05655526992288, 0, 0.51413881748072, 1.70777988614801, 5.14579759862779
), base.call = c("A", "A", "G", "A", "C", "T", "G", "G", "C", 
"C", "A", "G", "A", "C", "C", "G", "T", "G", "T", "T", "T", "G", 
"T", "G", "C", "A", "C", "T", "A", "C", "A", "C", "G", "G", "G", 
"C", "A", "C", "C", "C", "T", "G", "A", "C", "C", "G", "A", "C", 
"G", "G", "C", "A", "A", "G", "A", "A", "G", "T", "T", "C", "G", 
"A", "C", "A", "G", "C", "T", "C", "C", "C", "G", "C", "G", "A", 
"C"), index = 280:354, guide.seq = c("A", "A", "G", "A", "C", 
"T", "G", "G", "C", "C", "A", "G", "A", "C", "C", "G", "T", "G", 
"T", "T", "T", "G", "T", "G", "C", "A", "C", "T", "A", "C", "A", 
"C", "G", "G", "G", "C", "A", "C", "C", "C", "T", "G", "A", "C", 
"C", "G", "A", "C", "G", "G", "C", "A", "A", "G", "A", "A", "G", 
"T", "T", "C", "G", "A", "C", "A", "G", "C", "T", "C", "C", "C", 
"G", "C", "G", "A", "C"), T.pval = c(0.228676554380674, 0.280412828680748, 
0.827939166246584, 0.425406590019046, 0.657322637264116, 0, 0.765411105121432, 
0.878804556880597, 0.278979497049953, 0.465518467627195, 1.89848137210902e-14, 
0.659356884733281, 0.231708636538148, 0.668842878624421, 0.69758727493323, 
0.774893725575765, 0, 0.298931104584477, 0, 0, 0, 0.443180909152082, 
0, 0.164665393260113, 0.0733187437314712, 0.176694992833459, 
0.520990197345117, 0, 0.88297872340416, 0.236738138389383, 0.708269011742276, 
0.430406062825112, 0.879592315283877, 0.876153826315496, 0.492455726023622, 
0.317590703408841, 0.178669935977807, 0.35963763627241, 0.562864777078138, 
0.210060183202771, 0, 0.140418917782933, 2.3046009545169e-12, 
0.0879469449443405, 0.88297872340416, 0.484991781591689, 0.277073526786118, 
0.282295691447326, 0.614610388027811, 0.651945530741287, 0.88297872340416, 
0.116485817859952, 0.378275126781017, 0.88297872340416, 0.222149318789251, 
0.125759194024063, 0.166490662645596, 0, 0, 0.0919811367472243, 
0.405400606552745, 0.456793363432102, 0.422373502898604, 0.305873198045732, 
0.881383512034131, 0.416974796549863, 0, 0.0251396710323853, 
0.088871221053612, 0.183523489424356, 0.586374200069891, 0.88297872340416, 
0.864981759325506, 0.663535504467912, 0.122052336676734), C.pval = c(0.599957871061131, 
0.264035385697056, 0.200505776601781, 0.790502398135449, 0, 0.193800253640317, 
0.914634146292478, 0.44839624560468, 0, 0, 0.297903532231102, 
0.422803304241466, 0.764544661094481, 0, 0, 0.214665997011954, 
0.432451440854699, 0.914634146292478, 0.79081182945635, 0.914634146292478, 
0.516420488973769, 0.337326652137921, 0.708410629610078, 0.770631711418187, 
0, 0.406464906286225, 0, 0.121376065709168, 0.104859903432463, 
0, 0.115379219897187, 0, 0.00922029927756984, 0.509685168044703, 
0.499521990866612, 0, 0.104122984071568, 0, 0, 0, 0.0264285295164899, 
0.914634146292478, 0.674700288193859, 0, 0, 0.0477835654502375, 
0.83274728884243, 0, 0.0233914781025294, 0.843517777472756, 0, 
0.174491540379326, 0.914634146292478, 0.285375099393666, 0.914634146292478, 
0.914634146292478, 0.526658276170102, 0.726416858752018, 0.664776170658689, 
0, 0.0829175153119984, 0.635953381812487, 0, 0.021721354995405, 
0.292026400260692, 0, 0.0524951166052757, 0, 0, 0, 0.011275523154552, 
0, 0.00824527308020351, 0.357677509677059, 0), G.pval = c(0.662038034384259, 
0.671555116756572, 0, 0.452912461301688, 0.879120879120835, 0.41606765223538, 
0, 0, 0.181105166997211, 0.879120879120835, 0.480373673966454, 
0, 0.329059111318253, 0.803470971877472, 0.596998522120213, 0, 
0.261033159747351, 0, 0.11684326271194, 0.79089433473167, 0.453036060151543, 
0, 0.178248631422864, 0, 0.879120879120835, 0.10714781966262, 
2.66904532137957e-05, 0.853042308420265, 0.85121323273592, 0.639661329735876, 
0.118803747374701, 0.254802246022501, 2.22044604925031e-16, 0, 
0, 0.243927172583126, 0.879120879120835, 0.682646108169577, 0.436130740895196, 
0.879120879120835, 0.515357921848861, 0, 0.0618352997716323, 
0.879120879120835, 0.199138971187871, 1.11022302462516e-16, 0.342591646455819, 
0.751479789421352, 0, 0, 0.0247948691127152, 0.827919115778346, 
0.729737553143033, 1.11022302462516e-16, 0.125455676040878, 0.777842815502104, 
3.33066907387547e-16, 0.879120879120835, 0.258216953415939, 0.738931987349304, 
5.55111512312578e-16, 0.186448949197278, 0.79152298077171, 0.509021698300335, 
0, 0.185004149177248, 0.551318753258531, 0.348730152505061, 0.879120879120835, 
0.596024709538415, 3.33066907387547e-16, 0.0932638391565471, 
2.88657986402541e-15, 0.133806114864849, 0.506058028142997), 
    A.pval = c(0, 0, 0.193626008614737, 0, 0.0199193951936167, 
    0.801801801801802, 0.27448598293407, 0.119792487754242, 0.123623950870624, 
    0.21290625608455, 2.88213897192691e-13, 0.254914697366018, 
    0, 0.0250907229656034, 0.801801801801802, 0.188145884137183, 
    0.801801801801802, 0.389220813566806, 0.752314331511845, 
    0.801801801801802, 0.801801801801802, 0.468115332415584, 
    0.792682388954118, 0.410017534007123, 0.522622382253507, 
    0, 0.169192371783081, 0.576717989276667, 0, 0.137180834759726, 
    0, 0.256964977846232, 0.199628537435805, 0.708877336504611, 
    0.638693740413531, 0.163695084624946, 0, 0.118224108910814, 
    0.249259351989301, 0.151458069962589, 0.667117869875209, 
    0.250835902174332, 6.81676937119846e-14, 0.0575453950975373, 
    0.801801801801802, 0.26365365489977, 0, 0.00374250872009352, 
    0.801801801801802, 0.309692733304765, 0.447539387210283, 
    0, 0, 0.0323647149276909, 0, 0, 0.0286456626901144, 0.801801801801802, 
    0.624669812723946, 0.133498172713532, 0.0302893457203413, 
    0, 0.104804927154101, 0, 0.132824771841552, 0.422027178204934, 
    0.801801801801802, 0.687942906926775, 0.545774811927298, 
    0.261285905705397, 0.228102196120954, 0.117468232468373, 
    0.00960336895989822, 0, 0.0880055437487126), guide.position = 1:75), .Names = c("A.area", 
"C.area", "G.area", "T.area", "Tot.area", "A.perc", "C.perc", 
"G.perc", "T.perc", "base.call", "index", "guide.seq", "T.pval", 
"C.pval", "G.pval", "A.pval", "guide.position"), row.names = 280:354, class = "data.frame")
```

*Report generated using EditR v1.0.8*
