# Supplementary material for: Mechanistic and genetic basis of single-strand templated repair at Cas12a-induced DNA breaks in Chlamydomonas reinhardtii
Source: Nat Commun. 2021 Nov 19;12:6751. doi: 10.1038/s41467-021-27004-1 (PMC8604939; doi:10.1038/s41467-021-27004-1)
Supplement: Supplementary file 22 — Source Data [file 41467_2021_27004_MOESM22_ESM.zip › Source Data/EditR analysis/EditR outputs/Sense/rep2_ssODN_sense_-32_-16_0_16_32.html]

EditR v1.0.8 report


# EditR v1.0.8 report

- Data QA
  - Filtering data
  - Percent noise peak area
  - Base information
- Predicted editing
  - Editing bar plot
  - Editing table plot
  - Table of editing results
- For use in R

## Data QA

### Filtering data

What the data looked like prefiltering:

and the post filtering signal / noise plot:

### Percent noise peak area

### Base information

Here’s information about the signal of each base, the critical percent value where any higher value would be called as significant, and Filliben’s correlation for how well the noise was modelled by the zero adjusted gamma distribution.

| Base | Average percent signal | Average peak area | Critical percent value | model mu | Fillibens correlation |
| --- | --- | --- | --- | --- | --- |
| A | 93.42357 | 574.9688 | 8.565382 | 2.757071 | 0.9941519 |
| C | 92.74722 | 580.8986 | 5.887665 | 2.007409 | 0.9929726 |
| G | 93.67126 | 588.2241 | 6.024333 | 2.044897 | 0.9929311 |
| T | 94.69416 | 640.9762 | 7.286409 | 2.452728 | 0.9973471 |

## Predicted editing

### Editing bar plot

### Editing table plot

### Table of editing results


Here’s the entire guide region

| Sanger position | Guide position | Guide sequence | Sanger base call | Focal base | Focal base peak area | p value |  |
| --- | --- | --- | --- | --- | --- | --- | --- |
| 283 | 1 | A | A | A | 94.05 | 0.000000e+00 | \* |
| 283 | 1 | A | A | C | 0.93 | 7.625169e-01 |  |
| 283 | 1 | A | A | G | 2.60 | 2.386624e-01 |  |
| 283 | 1 | A | A | T | 2.42 | 3.846711e-01 |  |
| 284 | 2 | A | A | A | 92.69 | 0.000000e+00 | \* |
| 284 | 2 | A | A | C | 2.59 | 2.476566e-01 |  |
| 284 | 2 | A | A | G | 1.67 | 4.702901e-01 |  |
| 284 | 2 | A | A | T | 3.04 | 2.595911e-01 |  |
| 285 | 3 | G | G | A | 4.53 | 1.289340e-01 |  |
| 285 | 3 | G | G | C | 2.36 | 2.979006e-01 |  |
| 285 | 3 | G | G | G | 92.52 | 0.000000e+00 | \* |
| 285 | 3 | G | G | T | 0.59 | 8.548744e-01 |  |
| 286 | 4 | A | A | A | 95.54 | 0.000000e+00 | \* |
| 286 | 4 | A | A | C | 1.07 | 7.141225e-01 |  |
| 286 | 4 | A | A | G | 2.04 | 3.668113e-01 |  |
| 286 | 4 | A | A | T | 1.36 | 6.657489e-01 |  |
| 287 | 5 | C | C | A | 7.24 | 2.385927e-02 |  |
| 287 | 5 | C | C | C | 89.29 | 0.000000e+00 | \* |
| 287 | 5 | C | C | G | 1.57 | 5.011667e-01 |  |
| 287 | 5 | C | C | T | 1.89 | 5.166200e-01 |  |
| 288 | 6 | T | T | A | 6.27 | 4.459843e-02 |  |
| 288 | 6 | T | T | C | 2.04 | 3.817670e-01 |  |
| 288 | 6 | T | T | G | 2.45 | 2.686193e-01 |  |
| 288 | 6 | T | T | T | 89.24 | 0.000000e+00 | \* |
| 289 | 7 | G | G | A | 3.20 | 2.708278e-01 |  |
| 289 | 7 | G | G | C | 2.84 | 1.990037e-01 |  |
| 289 | 7 | G | G | G | 93.24 | 0.000000e+00 | \* |
| 289 | 7 | G | G | T | 0.71 | 8.315788e-01 |  |
| 290 | 8 | G | G | A | 4.78 | 1.110415e-01 |  |
| 290 | 8 | G | G | C | 1.20 | 6.676591e-01 |  |
| 290 | 8 | G | G | G | 93.36 | 0.000000e+00 | \* |
| 290 | 8 | G | G | T | 0.66 | 8.411059e-01 |  |
| 291 | 9 | C | C | A | 5.63 | 6.622389e-02 |  |
| 291 | 9 | C | C | C | 89.44 | 0.000000e+00 | \* |
| 291 | 9 | C | C | G | 4.93 | 2.957191e-02 |  |
| 291 | 9 | C | C | T | 0.00 | 9.052632e-01 |  |
| 292 | 10 | C | C | A | 4.28 | 1.485796e-01 |  |
| 292 | 10 | C | C | C | 93.50 | 0.000000e+00 | \* |
| 292 | 10 | C | C | G | 0.00 | 8.764045e-01 |  |
| 292 | 10 | C | C | T | 2.22 | 4.322998e-01 |  |
| 293 | 11 | A | A | A | 86.39 | 0.000000e+00 | \* |
| 293 | 11 | A | A | C | 1.44 | 5.776519e-01 |  |
| 293 | 11 | A | A | G | 0.00 | 8.764045e-01 |  |
| 293 | 11 | A | A | T | 12.16 | 1.391977e-04 | \* |
| 294 | 12 | G | G | A | 2.65 | 3.586550e-01 |  |
| 294 | 12 | G | G | C | 2.83 | 2.020570e-01 |  |
| 294 | 12 | G | G | G | 94.35 | 0.000000e+00 | \* |
| 294 | 12 | G | G | T | 0.18 | 9.019417e-01 |  |
| 295 | 13 | A | A | A | 95.68 | 0.000000e+00 | \* |
| 295 | 13 | A | A | C | 0.90 | 7.713075e-01 |  |
| 295 | 13 | A | A | G | 1.81 | 4.305737e-01 |  |
| 295 | 13 | A | A | T | 1.61 | 5.951917e-01 |  |
| 296 | 14 | C | C | A | 0.00 | 8.303571e-01 |  |
| 296 | 14 | C | C | C | 98.67 | 0.000000e+00 | \* |
| 296 | 14 | C | C | G | 0.00 | 8.764045e-01 |  |
| 296 | 14 | C | C | T | 1.33 | 6.726978e-01 |  |
| 297 | 15 | C | C | A | 7.71 | 1.765964e-02 |  |
| 297 | 15 | C | C | C | 89.21 | 0.000000e+00 | \* |
| 297 | 15 | C | C | G | 1.54 | 5.118265e-01 |  |
| 297 | 15 | C | C | T | 1.54 | 6.137052e-01 |  |
| 298 | 16 | G | G | A | 0.00 | 8.303571e-01 |  |
| 298 | 16 | G | G | C | 1.77 | 4.656277e-01 |  |
| 298 | 16 | G | G | G | 96.99 | 0.000000e+00 | \* |
| 298 | 16 | G | G | T | 1.24 | 6.991955e-01 |  |
| 299 | 17 | T | T | A | 3.08 | 2.878564e-01 |  |
| 299 | 17 | T | T | C | 1.98 | 3.996081e-01 |  |
| 299 | 17 | T | T | G | 2.20 | 3.249248e-01 |  |
| 299 | 17 | T | T | T | 92.73 | 0.000000e+00 | \* |
| 300 | 18 | G | G | A | 3.73 | 2.030176e-01 |  |
| 300 | 18 | G | G | C | 0.00 | 9.390244e-01 |  |
| 300 | 18 | G | G | G | 94.62 | 0.000000e+00 | \* |
| 300 | 18 | G | G | T | 1.65 | 5.838719e-01 |  |
| 301 | 19 | T | T | A | 0.00 | 8.303571e-01 |  |
| 301 | 19 | T | T | C | 0.69 | 8.394813e-01 |  |
| 301 | 19 | T | T | G | 5.16 | 2.354803e-02 |  |
| 301 | 19 | T | T | T | 94.15 | 0.000000e+00 | \* |
| 302 | 20 | T | T | A | 0.65 | 7.716664e-01 |  |
| 302 | 20 | T | T | C | 1.30 | 6.299224e-01 |  |
| 302 | 20 | T | T | G | 2.27 | 3.082979e-01 |  |
| 302 | 20 | T | T | T | 95.78 | 0.000000e+00 | \* |
| 303 | 21 | T | T | A | 0.00 | 8.303571e-01 |  |
| 303 | 21 | T | T | C | 0.00 | 9.390244e-01 |  |
| 303 | 21 | T | T | G | 2.28 | 3.057183e-01 |  |
| 303 | 21 | T | T | T | 97.72 | 0.000000e+00 | \* |
| 304 | 22 | G | G | A | 2.16 | 4.515975e-01 |  |
| 304 | 22 | G | G | C | 2.16 | 3.484349e-01 |  |
| 304 | 22 | G | G | G | 94.71 | 0.000000e+00 | \* |
| 304 | 22 | G | G | T | 0.96 | 7.734398e-01 |  |
| 305 | 23 | T | T | A | 0.00 | 8.303571e-01 |  |
| 305 | 23 | T | T | C | 1.99 | 3.979261e-01 |  |
| 305 | 23 | T | T | G | 2.58 | 2.420600e-01 |  |
| 305 | 23 | T | T | T | 95.43 | 0.000000e+00 | \* |
| 306 | 24 | G | G | A | 2.03 | 4.783776e-01 |  |
| 306 | 24 | G | G | C | 1.25 | 6.469643e-01 |  |
| 306 | 24 | G | G | G | 96.71 | 0.000000e+00 | \* |
| 306 | 24 | G | G | T | 0.00 | 9.052632e-01 |  |
| 307 | 25 | C | C | A | 0.98 | 7.094539e-01 |  |
| 307 | 25 | C | C | C | 99.02 | 0.000000e+00 | \* |
| 307 | 25 | C | C | G | 0.00 | 8.764045e-01 |  |
| 307 | 25 | C | C | T | 0.00 | 9.052632e-01 |  |
| 308 | 26 | A | A | A | 90.21 | 0.000000e+00 | \* |
| 308 | 26 | A | A | C | 2.77 | 2.129354e-01 |  |
| 308 | 26 | A | A | G | 5.11 | 2.490039e-02 |  |
| 308 | 26 | A | A | T | 1.91 | 5.098499e-01 |  |
| 309 | 27 | C | C | A | 2.51 | 3.843184e-01 |  |
| 309 | 27 | C | C | C | 89.63 | 0.000000e+00 | \* |
| 309 | 27 | C | C | G | 5.52 | 1.659708e-02 |  |
| 309 | 27 | C | C | T | 2.34 | 4.021046e-01 |  |
| 310 | 28 | T | T | A | 1.23 | 6.549926e-01 |  |
| 310 | 28 | T | T | C | 4.78 | 3.149003e-02 |  |
| 310 | 28 | T | T | G | 0.00 | 8.764045e-01 |  |
| 310 | 28 | T | T | T | 93.98 | 0.000000e+00 | \* |
| 311 | 29 | A | A | A | 95.99 | 0.000000e+00 | \* |
| 311 | 29 | A | A | C | 1.46 | 5.717390e-01 |  |
| 311 | 29 | A | A | G | 0.91 | 7.163928e-01 |  |
| 311 | 29 | A | A | T | 1.64 | 5.850567e-01 |  |
| 312 | 30 | C | C | A | 2.15 | 4.553356e-01 |  |
| 312 | 30 | C | C | C | 92.90 | 0.000000e+00 | \* |
| 312 | 30 | C | C | G | 1.82 | 4.282596e-01 |  |
| 312 | 30 | C | C | T | 3.14 | 2.444336e-01 |  |
| 313 | 31 | A | A | A | 91.68 | 0.000000e+00 | \* |
| 313 | 31 | A | A | C | 2.41 | 2.873225e-01 |  |
| 313 | 31 | A | A | G | 4.60 | 4.077748e-02 |  |
| 313 | 31 | A | A | T | 1.31 | 6.784600e-01 |  |
| 314 | 32 | C | C | A | 1.28 | 6.455210e-01 |  |
| 314 | 32 | C | C | C | 94.71 | 0.000000e+00 | \* |
| 314 | 32 | C | C | G | 2.01 | 3.746523e-01 |  |
| 314 | 32 | C | C | T | 2.01 | 4.853111e-01 |  |
| 315 | 33 | G | G | A | 3.10 | 2.861303e-01 |  |
| 315 | 33 | G | G | C | 3.33 | 1.286365e-01 |  |
| 315 | 33 | G | G | G | 93.57 | 0.000000e+00 | \* |
| 315 | 33 | G | G | T | 0.00 | 9.052632e-01 |  |
| 316 | 34 | G | G | A | 0.90 | 7.258108e-01 |  |
| 316 | 34 | G | G | C | 1.95 | 4.095569e-01 |  |
| 316 | 34 | G | G | G | 95.95 | 0.000000e+00 | \* |
| 316 | 34 | G | G | T | 1.20 | 7.101662e-01 |  |
| 317 | 35 | G | G | A | 2.20 | 4.437914e-01 |  |
| 317 | 35 | G | G | C | 1.47 | 5.688757e-01 |  |
| 317 | 35 | G | G | G | 95.23 | 0.000000e+00 | \* |
| 317 | 35 | G | G | T | 1.10 | 7.370236e-01 |  |
| 318 | 36 | C | C | A | 2.98 | 3.039361e-01 |  |
| 318 | 36 | C | C | C | 91.28 | 0.000000e+00 | \* |
| 318 | 36 | C | C | G | 4.47 | 4.600826e-02 |  |
| 318 | 36 | C | C | T | 1.28 | 6.886692e-01 |  |
| 319 | 37 | A | A | A | 93.27 | 0.000000e+00 | \* |
| 319 | 37 | A | A | C | 2.86 | 1.968238e-01 |  |
| 319 | 37 | A | A | G | 0.00 | 8.764045e-01 |  |
| 319 | 37 | A | A | T | 3.88 | 1.463603e-01 |  |
| 320 | 38 | C | C | A | 1.84 | 5.203694e-01 |  |
| 320 | 38 | C | C | C | 94.34 | 0.000000e+00 | \* |
| 320 | 38 | C | C | G | 1.27 | 5.991152e-01 |  |
| 320 | 38 | C | C | T | 2.55 | 3.558434e-01 |  |
| 321 | 39 | C | C | A | 3.46 | 2.350950e-01 |  |
| 321 | 39 | C | C | C | 94.33 | 0.000000e+00 | \* |
| 321 | 39 | C | C | G | 2.20 | 3.244218e-01 |  |
| 321 | 39 | C | C | T | 0.00 | 9.052632e-01 |  |
| 322 | 40 | C | C | A | 2.98 | 3.033097e-01 |  |
| 322 | 40 | C | C | C | 93.25 | 0.000000e+00 | \* |
| 322 | 40 | C | C | G | 0.63 | 7.973714e-01 |  |
| 322 | 40 | C | C | T | 3.14 | 2.437200e-01 |  |
| 323 | 41 | T | T | A | 0.93 | 7.193828e-01 |  |
| 323 | 41 | T | T | C | 3.73 | 8.911465e-02 |  |
| 323 | 41 | T | T | G | 1.55 | 5.081163e-01 |  |
| 323 | 41 | T | T | T | 93.79 | 0.000000e+00 | \* |
| 324 | 42 | G | G | A | 2.99 | 3.026417e-01 |  |
| 324 | 42 | G | G | C | 0.00 | 9.390244e-01 |  |
| 324 | 42 | G | G | G | 97.01 | 0.000000e+00 | \* |
| 324 | 42 | G | G | T | 0.00 | 9.052632e-01 |  |
| 325 | 43 | A | A | A | 85.01 | 0.000000e+00 | \* |
| 325 | 43 | A | A | C | 1.13 | 6.908139e-01 |  |
| 325 | 43 | A | A | G | 2.40 | 2.787695e-01 |  |
| 325 | 43 | A | A | T | 11.46 | 2.634783e-04 | \* |
| 326 | 44 | C | C | A | 4.04 | 1.705981e-01 |  |
| 326 | 44 | C | C | C | 95.96 | 0.000000e+00 | \* |
| 326 | 44 | C | C | G | 0.00 | 8.764045e-01 |  |
| 326 | 44 | C | C | T | 0.00 | 9.052632e-01 |  |
| 327 | 45 | C | C | A | 0.00 | 8.303571e-01 |  |
| 327 | 45 | C | C | C | 91.64 | 0.000000e+00 | \* |
| 327 | 45 | C | C | G | 3.35 | 1.280569e-01 |  |
| 327 | 45 | C | C | T | 5.02 | 6.252025e-02 |  |
| 328 | 46 | G | G | A | 2.56 | 3.750819e-01 |  |
| 328 | 46 | G | G | C | 3.20 | 1.454592e-01 |  |
| 328 | 46 | G | G | G | 94.24 | 0.000000e+00 | \* |
| 328 | 46 | G | G | T | 0.00 | 9.052632e-01 |  |
| 329 | 47 | A | A | A | 95.60 | 0.000000e+00 | \* |
| 329 | 47 | A | A | C | 0.81 | 8.022086e-01 |  |
| 329 | 47 | A | A | G | 0.93 | 7.121764e-01 |  |
| 329 | 47 | A | A | T | 2.66 | 3.313528e-01 |  |
| 330 | 48 | C | C | A | 0.00 | 8.303571e-01 |  |
| 330 | 48 | C | C | C | 95.18 | 0.000000e+00 | \* |
| 330 | 48 | C | C | G | 1.67 | 4.716723e-01 |  |
| 330 | 48 | C | C | T | 3.15 | 2.414193e-01 |  |
| 331 | 49 | G | G | A | 10.03 | 3.729596e-03 | \* |
| 331 | 49 | G | G | C | 2.17 | 3.472091e-01 |  |
| 331 | 49 | G | G | G | 86.99 | 0.000000e+00 | \* |
| 331 | 49 | G | G | T | 0.81 | 8.093058e-01 |  |
| 332 | 50 | G | G | A | 1.48 | 6.003393e-01 |  |
| 332 | 50 | G | G | C | 1.78 | 4.639278e-01 |  |
| 332 | 50 | G | G | G | 96.75 | 0.000000e+00 | \* |
| 332 | 50 | G | G | T | 0.00 | 9.052632e-01 |  |
| 333 | 51 | C | C | A | 3.56 | 2.229143e-01 |  |
| 333 | 51 | C | C | C | 89.06 | 0.000000e+00 | \* |
| 333 | 51 | C | C | G | 3.56 | 1.058117e-01 |  |
| 333 | 51 | C | C | T | 3.82 | 1.528472e-01 |  |
| 334 | 52 | A | A | A | 94.17 | 0.000000e+00 | \* |
| 334 | 52 | A | A | C | 0.65 | 8.511509e-01 |  |
| 334 | 52 | A | A | G | 1.94 | 3.924354e-01 |  |
| 334 | 52 | A | A | T | 3.24 | 2.285028e-01 |  |
| 335 | 53 | A | A | A | 95.80 | 0.000000e+00 | \* |
| 335 | 53 | A | A | C | 0.18 | 9.341208e-01 |  |
| 335 | 53 | A | A | G | 1.09 | 6.578935e-01 |  |
| 335 | 53 | A | A | T | 2.92 | 2.814823e-01 |  |
| 336 | 54 | G | G | A | 2.48 | 3.890219e-01 |  |
| 336 | 54 | G | G | C | 2.03 | 3.852193e-01 |  |
| 336 | 54 | G | G | G | 93.68 | 0.000000e+00 | \* |
| 336 | 54 | G | G | T | 1.81 | 5.394915e-01 |  |
| 337 | 55 | A | A | A | 95.52 | 0.000000e+00 | \* |
| 337 | 55 | A | A | C | 0.83 | 7.960638e-01 |  |
| 337 | 55 | A | A | G | 1.49 | 5.273377e-01 |  |
| 337 | 55 | A | A | T | 2.16 | 4.471541e-01 |  |
| 338 | 56 | A | A | A | 94.13 | 0.000000e+00 | \* |
| 338 | 56 | A | A | C | 0.73 | 8.260137e-01 |  |
| 338 | 56 | A | A | G | 0.73 | 7.692666e-01 |  |
| 338 | 56 | A | A | T | 4.40 | 9.966416e-02 |  |
| 339 | 57 | G | G | A | 4.20 | 1.559490e-01 |  |
| 339 | 57 | G | G | C | 2.89 | 1.917549e-01 |  |
| 339 | 57 | G | G | G | 89.50 | 0.000000e+00 | \* |
| 339 | 57 | G | G | T | 3.41 | 2.028086e-01 |  |
| 340 | 58 | T | T | A | 3.12 | 2.826145e-01 |  |
| 340 | 58 | T | T | C | 2.34 | 3.034527e-01 |  |
| 340 | 58 | T | T | G | 0.00 | 8.764045e-01 |  |
| 340 | 58 | T | T | T | 94.54 | 0.000000e+00 | \* |
| 341 | 59 | T | T | A | 11.66 | 1.208310e-03 | \* |
| 341 | 59 | T | T | C | 1.34 | 6.154734e-01 |  |
| 341 | 59 | T | T | G | 2.49 | 2.617108e-01 |  |
| 341 | 59 | T | T | T | 84.51 | 0.000000e+00 | \* |
| 342 | 60 | C | C | A | 3.35 | 2.506374e-01 |  |
| 342 | 60 | C | C | C | 93.68 | 0.000000e+00 | \* |
| 342 | 60 | C | C | G | 1.30 | 5.898208e-01 |  |
| 342 | 60 | C | C | T | 1.67 | 5.764713e-01 |  |
| 343 | 61 | G | G | A | 5.60 | 6.770388e-02 |  |
| 343 | 61 | G | G | C | 3.56 | 1.040475e-01 |  |
| 343 | 61 | G | G | G | 89.06 | 0.000000e+00 | \* |
| 343 | 61 | G | G | T | 1.78 | 5.462975e-01 |  |
| 344 | 62 | A | A | A | 92.92 | 0.000000e+00 | \* |
| 344 | 62 | A | A | C | 1.83 | 4.447945e-01 |  |
| 344 | 62 | A | A | G | 1.97 | 3.858141e-01 |  |
| 344 | 62 | A | A | T | 3.28 | 2.223869e-01 |  |
| 345 | 63 | C | C | A | 2.55 | 3.763226e-01 |  |
| 345 | 63 | C | C | C | 92.03 | 0.000000e+00 | \* |
| 345 | 63 | C | C | G | 1.91 | 4.001587e-01 |  |
| 345 | 63 | C | C | T | 3.51 | 1.897477e-01 |  |
| 346 | 64 | A | A | A | 89.59 | 0.000000e+00 | \* |
| 346 | 64 | A | A | C | 4.82 | 3.028622e-02 |  |
| 346 | 64 | A | A | G | 2.03 | 3.684998e-01 |  |
| 346 | 64 | A | A | T | 3.55 | 1.839790e-01 |  |
| 347 | 65 | G | G | A | 2.87 | 3.219189e-01 |  |
| 347 | 65 | G | G | C | 2.87 | 1.952743e-01 |  |
| 347 | 65 | G | G | G | 92.52 | 0.000000e+00 | \* |
| 347 | 65 | G | G | T | 1.75 | 5.544875e-01 |  |
| 348 | 66 | C | C | A | 2.12 | 4.606398e-01 |  |
| 348 | 66 | C | C | C | 93.64 | 0.000000e+00 | \* |
| 348 | 66 | C | C | G | 1.54 | 5.117217e-01 |  |
| 348 | 66 | C | C | T | 2.70 | 3.241225e-01 |  |
| 349 | 67 | T | T | A | 0.29 | 8.175531e-01 |  |
| 349 | 67 | T | T | C | 3.54 | 1.062616e-01 |  |
| 349 | 67 | T | T | G | 1.62 | 4.862731e-01 |  |
| 349 | 67 | T | T | T | 94.54 | 0.000000e+00 | \* |
| 350 | 68 | C | C | A | 2.33 | 4.175151e-01 |  |
| 350 | 68 | C | C | C | 96.59 | 0.000000e+00 | \* |
| 350 | 68 | C | C | G | 1.08 | 6.636804e-01 |  |
| 350 | 68 | C | C | T | 0.00 | 9.052632e-01 |  |
| 351 | 69 | C | C | A | 1.90 | 5.065974e-01 |  |
| 351 | 69 | C | C | C | 93.07 | 0.000000e+00 | \* |
| 351 | 69 | C | C | G | 0.95 | 7.042670e-01 |  |
| 351 | 69 | C | C | T | 4.08 | 1.268296e-01 |  |
| 352 | 70 | C | C | A | 3.16 | 2.759305e-01 |  |
| 352 | 70 | C | C | C | 95.78 | 0.000000e+00 | \* |
| 352 | 70 | C | C | G | 1.05 | 6.709641e-01 |  |
| 352 | 70 | C | C | T | 0.00 | 9.052632e-01 |  |
| 353 | 71 | G | G | A | 4.72 | 1.151836e-01 |  |
| 353 | 71 | G | G | C | 2.70 | 2.259126e-01 |  |
| 353 | 71 | G | G | G | 91.69 | 0.000000e+00 | \* |
| 353 | 71 | G | G | T | 0.90 | 7.890025e-01 |  |
| 354 | 72 | C | C | A | 0.00 | 8.303571e-01 |  |
| 354 | 72 | C | C | C | 95.35 | 0.000000e+00 | \* |
| 354 | 72 | C | C | G | 2.63 | 2.341210e-01 |  |
| 354 | 72 | C | C | T | 2.02 | 4.819318e-01 |  |
| 355 | 73 | G | G | A | 7.69 | 1.781276e-02 |  |
| 355 | 73 | G | G | C | 3.85 | 7.952285e-02 |  |
| 355 | 73 | G | G | G | 87.56 | 0.000000e+00 | \* |
| 355 | 73 | G | G | T | 0.90 | 7.875131e-01 |  |
| 356 | 74 | A | A | A | 95.72 | 0.000000e+00 | \* |
| 356 | 74 | A | A | C | 1.27 | 6.411745e-01 |  |
| 356 | 74 | A | A | G | 2.06 | 3.606925e-01 |  |
| 356 | 74 | A | A | T | 0.95 | 7.761290e-01 |  |
| 357 | 75 | C | C | A | 0.00 | 8.303571e-01 |  |
| 357 | 75 | C | C | C | 78.43 | 0.000000e+00 | \* |
| 357 | 75 | C | C | G | 18.05 | 1.745455e-08 | \* |
| 357 | 75 | C | C | T | 3.51 | 1.890131e-01 |  |

## For use in R

If you want to work with the results in R, here is output that you can copy and paste in your terminal to get:

The base information:

```
structure(list(focal.base = c("A", "C", "G", "T"), avg.percsignal = c(93.4235714252924, 
92.7472192489602, 93.6712616264444, 94.6941637785675), avg.areasignal = c(574.96875, 
580.898550724638, 588.224137931035, 640.97619047619), crit.perc.area = c(8.56538159549718, 
5.88766505276148, 6.0243334018794, 7.28640867103033), mu = c(2.75707123671027, 
2.0074089064693, 2.04489713722285, 2.45272761457012), fillibens = c(0.994151917756347, 
0.992972565877537, 0.992931124561253, 0.997347116965619)), .Names = c("focal.base", 
"avg.percsignal", "avg.areasignal", "crit.perc.area", "mu", "fillibens"
), row.names = c(NA, -4L), class = "data.frame")
```

the data.frame that contains information on the guide region:

```
structure(list(A.area = c(506, 609, 23, 985, 46, 46, 36, 36, 
24, 29, 419, 15, 953, 0, 45, 0, 14, 34, 0, 4, 0, 18, 0, 13, 5, 
424, 15, 8, 526, 13, 419, 7, 13, 6, 12, 14, 457, 13, 22, 19, 
6, 23, 601, 23, 0, 12, 826, 0, 37, 10, 14, 291, 525, 11, 576, 
513, 16, 16, 61, 18, 22, 709, 16, 353, 18, 11, 2, 13, 14, 15, 
21, 0, 34, 604, 0), C.area = c(5, 17, 12, 11, 567, 15, 32, 9, 
381, 633, 7, 16, 9, 592, 521, 10, 9, 0, 4, 8, 0, 18, 10, 8, 505, 
13, 536, 31, 8, 563, 11, 519, 14, 13, 8, 429, 14, 667, 599, 594, 
24, 0, 8, 546, 493, 15, 7, 513, 8, 12, 350, 2, 1, 9, 5, 4, 11, 
12, 7, 504, 14, 14, 577, 19, 18, 486, 24, 538, 685, 454, 12, 
472, 17, 8, 491), G.area = c(14, 11, 470, 21, 10, 18, 1049, 703, 
21, 0, 0, 534, 18, 0, 9, 548, 10, 862, 30, 14, 14, 788, 13, 618, 
0, 24, 33, 0, 5, 11, 21, 11, 393, 640, 519, 21, 0, 9, 14, 4, 
10, 747, 17, 0, 18, 442, 8, 9, 321, 654, 14, 6, 6, 415, 9, 4, 
341, 0, 13, 7, 350, 15, 12, 8, 581, 8, 11, 6, 7, 5, 408, 13, 
387, 13, 113), T.area = c(13, 20, 3, 14, 12, 655, 8, 5, 0, 15, 
59, 1, 16, 8, 9, 7, 421, 15, 547, 590, 599, 8, 480, 0, 0, 9, 
14, 609, 9, 19, 6, 11, 0, 8, 6, 6, 19, 18, 0, 20, 604, 0, 81, 
0, 27, 0, 23, 17, 3, 0, 15, 10, 16, 8, 13, 24, 13, 485, 442, 
9, 7, 25, 22, 14, 11, 14, 641, 0, 30, 0, 4, 10, 4, 6, 22), Tot.area = c(538, 
657, 508, 1031, 635, 734, 1125, 753, 426, 677, 485, 566, 996, 
600, 584, 565, 454, 911, 581, 616, 613, 832, 503, 639, 510, 470, 
598, 648, 548, 606, 457, 548, 420, 667, 545, 470, 490, 707, 635, 
637, 644, 770, 707, 569, 538, 469, 864, 539, 369, 676, 393, 309, 
548, 443, 603, 545, 381, 513, 523, 538, 393, 763, 627, 394, 628, 
519, 678, 557, 736, 474, 445, 495, 442, 631, 626), A.perc = c(94.0520446096654, 
92.6940639269406, 4.52755905511811, 95.5383123181377, 7.24409448818898, 
6.26702997275204, 3.2, 4.7808764940239, 5.63380281690141, 4.28360413589365, 
86.3917525773196, 2.65017667844523, 95.6827309236948, 0, 7.70547945205479, 
0, 3.08370044052863, 3.73216245883644, 0, 0.649350649350649, 
0, 2.16346153846154, 0, 2.03442879499218, 0.980392156862745, 
90.2127659574468, 2.50836120401338, 1.23456790123457, 95.985401459854, 
2.14521452145215, 91.6849015317287, 1.27737226277372, 3.0952380952381, 
0.899550224887556, 2.20183486238532, 2.97872340425532, 93.265306122449, 
1.83875530410184, 3.46456692913386, 2.98273155416013, 0.93167701863354, 
2.98701298701299, 85.007072135785, 4.04217926186292, 0, 2.55863539445629, 
95.6018518518518, 0, 10.0271002710027, 1.4792899408284, 3.56234096692112, 
94.1747572815534, 95.8029197080292, 2.48306997742664, 95.5223880597015, 
94.1284403669725, 4.1994750656168, 3.11890838206628, 11.6634799235182, 
3.3457249070632, 5.59796437659033, 92.9226736566186, 2.5518341307815, 
89.5939086294416, 2.86624203821656, 2.11946050096339, 0.294985250737463, 
2.33393177737882, 1.90217391304348, 3.16455696202532, 4.71910112359551, 
0, 7.69230769230769, 95.7210776545166, 0), C.perc = c(0.929368029739777, 
2.58751902587519, 2.36220472440945, 1.06692531522793, 89.2913385826772, 
2.04359673024523, 2.84444444444444, 1.19521912350598, 89.4366197183099, 
93.5007385524372, 1.44329896907216, 2.82685512367491, 0.903614457831325, 
98.6666666666667, 89.2123287671233, 1.76991150442478, 1.98237885462555, 
0, 0.688468158347676, 1.2987012987013, 0, 2.16346153846154, 1.98807157057654, 
1.25195618153365, 99.0196078431373, 2.76595744680851, 89.6321070234114, 
4.78395061728395, 1.45985401459854, 92.9042904290429, 2.40700218818381, 
94.7080291970803, 3.33333333333333, 1.94902548725637, 1.46788990825688, 
91.2765957446808, 2.85714285714286, 94.3422913719943, 94.3307086614173, 
93.2496075353218, 3.72670807453416, 0, 1.13154172560113, 95.9578207381371, 
91.635687732342, 3.19829424307036, 0.810185185185185, 95.1762523191095, 
2.1680216802168, 1.77514792899408, 89.058524173028, 0.647249190938511, 
0.182481751824818, 2.03160270880361, 0.829187396351575, 0.73394495412844, 
2.88713910761155, 2.33918128654971, 1.33843212237094, 93.6802973977695, 
3.56234096692112, 1.8348623853211, 92.0255183413078, 4.82233502538071, 
2.86624203821656, 93.6416184971098, 3.53982300884956, 96.588868940754, 
93.070652173913, 95.7805907172996, 2.69662921348315, 95.3535353535353, 
3.84615384615385, 1.26782884310618, 78.4345047923323), G.perc = c(2.60223048327138, 
1.67427701674277, 92.5196850393701, 2.0368574199806, 1.5748031496063, 
2.45231607629428, 93.2444444444444, 93.3598937583001, 4.92957746478873, 
0, 0, 94.3462897526502, 1.80722891566265, 0, 1.54109589041096, 
96.9911504424779, 2.20264317180617, 94.6212952799122, 5.16351118760757, 
2.27272727272727, 2.28384991843393, 94.7115384615385, 2.5844930417495, 
96.7136150234742, 0, 5.1063829787234, 5.51839464882943, 0, 0.912408759124088, 
1.81518151815182, 4.59518599562363, 2.00729927007299, 93.5714285714286, 
95.952023988006, 95.2293577981651, 4.46808510638298, 0, 1.27298444130127, 
2.20472440944882, 0.627943485086342, 1.5527950310559, 97.012987012987, 
2.4045261669024, 0, 3.3457249070632, 94.2430703624733, 0.925925925925926, 
1.66975881261596, 86.9918699186992, 96.7455621301775, 3.56234096692112, 
1.94174757281553, 1.09489051094891, 93.6794582392777, 1.49253731343284, 
0.73394495412844, 89.501312335958, 0, 2.48565965583174, 1.30111524163569, 
89.058524173028, 1.96592398427261, 1.91387559808612, 2.03045685279188, 
92.515923566879, 1.54142581888247, 1.62241887905605, 1.07719928186715, 
0.951086956521739, 1.05485232067511, 91.685393258427, 2.62626262626263, 
87.5565610859728, 2.06022187004754, 18.0511182108626), T.perc = c(2.41635687732342, 
3.0441400304414, 0.590551181102362, 1.35790494665373, 1.88976377952756, 
89.2370572207085, 0.711111111111111, 0.664010624169987, 0, 2.21565731166913, 
12.1649484536082, 0.176678445229682, 1.60642570281124, 1.33333333333333, 
1.54109589041096, 1.23893805309735, 92.7312775330396, 1.64654226125137, 
94.1480206540448, 95.7792207792208, 97.7161500815661, 0.961538461538462, 
95.427435387674, 0, 0, 1.91489361702128, 2.34113712374582, 93.9814814814815, 
1.64233576642336, 3.13531353135314, 1.31291028446389, 2.00729927007299, 
0, 1.19940029985008, 1.10091743119266, 1.27659574468085, 3.87755102040816, 
2.54596888260255, 0, 3.13971742543171, 93.7888198757764, 0, 11.4568599717115, 
0, 5.0185873605948, 0, 2.66203703703704, 3.15398886827458, 0.813008130081301, 
0, 3.81679389312977, 3.23624595469256, 2.91970802919708, 1.8058690744921, 
2.1558872305141, 4.40366972477064, 3.41207349081365, 94.541910331384, 
84.5124282982792, 1.6728624535316, 1.78117048346056, 3.27653997378768, 
3.50877192982456, 3.55329949238579, 1.7515923566879, 2.69749518304432, 
94.5427728613569, 0, 4.07608695652174, 0, 0.898876404494382, 
2.02020202020202, 0.904977375565611, 0.950871632329635, 3.51437699680511
), base.call = c("A", "A", "G", "A", "C", "T", "G", "G", "C", 
"C", "A", "G", "A", "C", "C", "G", "T", "G", "T", "T", "T", "G", 
"T", "G", "C", "A", "C", "T", "A", "C", "A", "C", "G", "G", "G", 
"C", "A", "C", "C", "C", "T", "G", "A", "C", "C", "G", "A", "C", 
"G", "G", "C", "A", "A", "G", "A", "A", "G", "T", "T", "C", "G", 
"A", "C", "A", "G", "C", "T", "C", "C", "C", "G", "C", "G", "A", 
"C"), index = 283:357, guide.seq = c("A", "A", "G", "A", "C", 
"T", "G", "G", "C", "C", "A", "G", "A", "C", "C", "G", "T", "G", 
"T", "T", "T", "G", "T", "G", "C", "A", "C", "T", "A", "C", "A", 
"C", "G", "G", "G", "C", "A", "C", "C", "C", "T", "G", "A", "C", 
"C", "G", "A", "C", "G", "G", "C", "A", "A", "G", "A", "A", "G", 
"T", "T", "C", "G", "A", "C", "A", "G", "C", "T", "C", "C", "C", 
"G", "C", "G", "A", "C"), T.pval = c(0.384671114304207, 0.259591059892505, 
0.854874398571136, 0.665748946625332, 0.516619974274474, 0, 0.831578810317219, 
0.841105938390221, 0.905263157886924, 0.432299799882943, 0.000139197663213797, 
0.901941719867565, 0.595191724599828, 0.672697800842721, 0.613705175603046, 
0.699195469079172, 0, 0.583871904647823, 0, 0, 0, 0.773439754790041, 
0, 0.905263157886924, 0.905263157886924, 0.509849883146663, 0.402104563949106, 
0, 0.585056719112725, 0.244433551242231, 0.678460024939098, 0.485311111370742, 
0.905263157886924, 0.710166199394524, 0.737023598561162, 0.688669188260175, 
0.146360273832431, 0.35584337338443, 0.905263157886924, 0.243719995744634, 
0, 0.905263157886924, 0.000263478301011588, 0.905263157886924, 
0.062520245807881, 0.905263157886924, 0.331352782170354, 0.241419279724301, 
0.809305789433445, 0.905263157886924, 0.15284716394959, 0.228502786205551, 
0.281482348970401, 0.539491471310143, 0.447154069579003, 0.0996641618477143, 
0.202808632476975, 0, 0, 0.576471253798194, 0.546297537761859, 
0.22238692216475, 0.189747653004948, 0.183979033668811, 0.554487456667484, 
0.32412246805661, 0, 0.905263157886924, 0.126829599964663, 0.905263157886924, 
0.789002499215111, 0.481931787842175, 0.787513088925584, 0.776129021874963, 
0.189013094830228), C.pval = c(0.762516934599254, 0.247656586655747, 
0.297900634116959, 0.714122463249445, 0, 0.381766967816507, 0.199003735479368, 
0.667659072084989, 0, 0, 0.577651893406492, 0.202057028807783, 
0.77130749991475, 0, 0, 0.465627664645994, 0.399608115947682, 
0.939024390243902, 0.839481288644878, 0.629922370313168, 0.939024390243902, 
0.348434936385001, 0.397926062073247, 0.646964309053625, 0, 0.21293537614124, 
0, 0.0314900257446455, 0.571739014710621, 0, 0.287322537028713, 
0, 0.128636514902545, 0.409556897759118, 0.568875734848056, 0, 
0.196823828945482, 0, 0, 0, 0.0891146475576254, 0.939024390243902, 
0.690813897149201, 0, 0, 0.145459184823309, 0.802208649697296, 
0, 0.347209144528152, 0.463927826710264, 0, 0.851150854589514, 
0.934120753920821, 0.38521934799036, 0.796063847626186, 0.826013700048938, 
0.191754880547259, 0.303452747151402, 0.615473360760557, 0, 0.104047547271534, 
0.444794476077254, 0, 0.0302862194385545, 0.19527429233799, 0, 
0.106261633040538, 0, 0, 0, 0.225912616669817, 0, 0.0795228513256221, 
0.641174528690017, 0), G.pval = c(0.238662426003416, 0.470290116058559, 
0, 0.366811334912792, 0.501166714917793, 0.268619278988638, 0, 
0, 0.0295719065048259, 0.876404494381997, 0.876404494381997, 
0, 0.430573714763564, 0.876404494381997, 0.511826531255574, 0, 
0.324924842652056, 0, 0.0235480263059, 0.308297856404266, 0.305718293905817, 
0, 0.242059963971538, 0, 0.876404494381997, 0.024900389515269, 
0.0165970808251847, 0.876404494381997, 0.716392774185573, 0.428259647018202, 
0.0407774797792847, 0.374652314155541, 0, 0, 0, 0.0460082577211938, 
0.876404494381997, 0.599115248379057, 0.324421802754933, 0.797371381969689, 
0.508116272117426, 0, 0.27876952315282, 0.876404494381997, 0.128056924457155, 
0, 0.712176364132741, 0.471672303230596, 0, 0, 0.105811699215648, 
0.392435427827804, 0.657893524796426, 0, 0.527337673436537, 0.769266632714215, 
0, 0.876404494381997, 0.261710792690697, 0.589820801129642, 0, 
0.385814066234933, 0.400158678669298, 0.368499775648437, 0, 0.511721749519814, 
0.486273095048352, 0.663680386139719, 0.704266980287976, 0.67096406409601, 
0, 0.234121033686925, 0, 0.360692481887078, 1.74545459197262e-08
), A.pval = c(0, 0, 0.128933954041444, 0, 0.0238592676656727, 
0.0445984329586051, 0.270827766242553, 0.111041493639519, 0.0662238896362762, 
0.148579606494687, 0, 0.358655004030613, 0, 0.830357142857142, 
0.0176596366561254, 0.830357142857142, 0.287856364013164, 0.203017644815678, 
0.830357142857142, 0.77166642189704, 0.830357142857142, 0.451597515361447, 
0.830357142857142, 0.478377620441201, 0.709453910817532, 0, 0.384318416372805, 
0.654992577516667, 0, 0.45533562320216, 0, 0.645520969190567, 
0.286130282969254, 0.725810830755951, 0.443791402624937, 0.303936111995446, 
0, 0.520369437774674, 0.235095040250455, 0.303309728303526, 0.719382835660731, 
0.302641730179131, 0, 0.170598107253471, 0.830357142857142, 0.375081935324332, 
0, 0.830357142857142, 0.00372959604918732, 0.600339285836707, 
0.222914264837316, 0, 0, 0.389021876223035, 0, 0, 0.155949015190322, 
0.282614522965684, 0.00120831005031052, 0.250637367688937, 0.067703881510124, 
0, 0.376322626307267, 0, 0.321918945124315, 0.460639757530232, 
0.817553051437358, 0.417515080757364, 0.506597391357445, 0.275930497339468, 
0.115183592140674, 0.830357142857142, 0.0178127645340304, 0, 
0.830357142857142), guide.position = 1:75), .Names = c("A.area", 
"C.area", "G.area", "T.area", "Tot.area", "A.perc", "C.perc", 
"G.perc", "T.perc", "base.call", "index", "guide.seq", "T.pval", 
"C.pval", "G.pval", "A.pval", "guide.position"), row.names = 283:357, class = "data.frame")
```

*Report generated using EditR v1.0.8*
