# Supplementary material for: Mechanistic and genetic basis of single-strand templated repair at Cas12a-induced DNA breaks in Chlamydomonas reinhardtii
Source: Nat Commun. 2021 Nov 19;12:6751. doi: 10.1038/s41467-021-27004-1 (PMC8604939; doi:10.1038/s41467-021-27004-1)
Supplement: Supplementary file 22 — Source Data [file 41467_2021_27004_MOESM22_ESM.zip › Source Data/EditR analysis/EditR outputs/Sense/rep3_ssODN_sense_0.html]

EditR v1.0.8 report


# EditR v1.0.8 report

- Data QA
  - Filtering data
  - Percent noise peak area
  - Base information
- Predicted editing
  - Editing bar plot
  - Editing table plot
  - Table of editing results
- For use in R

## Data QA

### Filtering data

What the data looked like prefiltering:

and the post filtering signal / noise plot:

### Percent noise peak area

### Base information

Here’s information about the signal of each base, the critical percent value where any higher value would be called as significant, and Filliben’s correlation for how well the noise was modelled by the zero adjusted gamma distribution.

| Base | Average percent signal | Average peak area | Critical percent value | model mu | Fillibens correlation |
| --- | --- | --- | --- | --- | --- |
| A | 88.55610 | 160.7059 | 16.99749 | 5.489460 | 0.9971688 |
| C | 85.73635 | 168.1791 | 11.66318 | 3.778250 | 0.9923579 |
| G | 87.80127 | 154.5357 | 19.80099 | 5.097471 | 0.9807945 |
| T | 90.24396 | 186.0455 | 12.36184 | 3.972834 | 0.9910536 |

## Predicted editing

### Editing bar plot

### Editing table plot

### Table of editing results


Here’s the entire guide region

| Sanger position | Guide position | Guide sequence | Sanger base call | Focal base | Focal base peak area | p value |  |
| --- | --- | --- | --- | --- | --- | --- | --- |
| 276 | 1 | A | A | A | 98.01 | 3.330669e-16 | \* |
| 276 | 1 | A | A | C | 1.32 | 7.728753e-01 |  |
| 276 | 1 | A | A | G | 0.00 | 8.351648e-01 |  |
| 276 | 1 | A | A | T | 0.66 | 8.831418e-01 |  |
| 277 | 2 | A | A | A | 95.95 | 8.881784e-16 | \* |
| 277 | 2 | A | A | C | 0.00 | 8.928571e-01 |  |
| 277 | 2 | A | A | G | 3.47 | 4.550234e-01 |  |
| 277 | 2 | A | A | T | 0.58 | 8.905071e-01 |  |
| 278 | 3 | G | G | A | 5.74 | 3.364637e-01 |  |
| 278 | 3 | G | G | C | 6.56 | 1.161676e-01 |  |
| 278 | 3 | G | G | G | 87.70 | 3.920533e-10 | \* |
| 278 | 3 | G | G | T | 0.00 | 9.139785e-01 |  |
| 279 | 4 | A | A | A | 93.39 | 2.553513e-15 | \* |
| 279 | 4 | A | A | C | 0.86 | 8.383676e-01 |  |
| 279 | 4 | A | A | G | 1.15 | 7.132246e-01 |  |
| 279 | 4 | A | A | T | 4.60 | 2.995921e-01 |  |
| 280 | 5 | C | C | A | 7.39 | 2.151054e-01 |  |
| 280 | 5 | C | C | C | 88.64 | 0.000000e+00 | \* |
| 280 | 5 | C | C | G | 0.00 | 8.351648e-01 |  |
| 280 | 5 | C | C | T | 3.98 | 3.752329e-01 |  |
| 281 | 6 | T | T | A | 0.00 | 8.727273e-01 |  |
| 281 | 6 | T | T | C | 1.70 | 7.095187e-01 |  |
| 281 | 6 | T | T | G | 3.41 | 4.606597e-01 |  |
| 281 | 6 | T | T | T | 94.89 | 0.000000e+00 | \* |
| 282 | 7 | G | G | A | 5.00 | 4.051801e-01 |  |
| 282 | 7 | G | G | C | 0.62 | 8.639117e-01 |  |
| 282 | 7 | G | G | G | 92.50 | 1.159381e-10 | \* |
| 282 | 7 | G | G | T | 1.88 | 7.106941e-01 |  |
| 283 | 8 | G | G | A | 0.00 | 8.727273e-01 |  |
| 283 | 8 | G | G | C | 2.05 | 6.484328e-01 |  |
| 283 | 8 | G | G | G | 97.44 | 3.305356e-11 | \* |
| 283 | 8 | G | G | T | 0.51 | 8.955799e-01 |  |
| 284 | 9 | C | C | A | 10.88 | 7.531223e-02 |  |
| 284 | 9 | C | C | C | 78.91 | 0.000000e+00 | \* |
| 284 | 9 | C | C | G | 2.72 | 5.303161e-01 |  |
| 284 | 9 | C | C | T | 7.48 | 9.302008e-02 |  |
| 285 | 10 | C | C | A | 7.61 | 2.016277e-01 |  |
| 285 | 10 | C | C | C | 84.77 | 0.000000e+00 | \* |
| 285 | 10 | C | C | G | 2.03 | 6.073849e-01 |  |
| 285 | 10 | C | C | T | 5.58 | 2.048318e-01 |  |
| 286 | 11 | A | A | A | 89.63 | 1.088019e-14 | \* |
| 286 | 11 | A | A | C | 1.48 | 7.474130e-01 |  |
| 286 | 11 | A | A | G | 8.89 | 1.360532e-01 |  |
| 286 | 11 | A | A | T | 0.00 | 9.139785e-01 |  |
| 287 | 12 | G | G | A | 10.42 | 8.717266e-02 |  |
| 287 | 12 | G | G | C | 0.00 | 8.928571e-01 |  |
| 287 | 12 | G | G | G | 88.89 | 2.902228e-10 | \* |
| 287 | 12 | G | G | T | 0.69 | 8.800954e-01 |  |
| 288 | 13 | A | A | A | 92.46 | 3.552714e-15 | \* |
| 288 | 13 | A | A | C | 1.74 | 7.035127e-01 |  |
| 288 | 13 | A | A | G | 0.58 | 7.813158e-01 |  |
| 288 | 13 | A | A | T | 5.22 | 2.365850e-01 |  |
| 289 | 14 | C | C | A | 6.63 | 2.654080e-01 |  |
| 289 | 14 | C | C | C | 88.95 | 0.000000e+00 | \* |
| 289 | 14 | C | C | G | 0.00 | 8.351648e-01 |  |
| 289 | 14 | C | C | T | 4.42 | 3.199575e-01 |  |
| 290 | 15 | C | C | A | 9.87 | 1.032659e-01 |  |
| 290 | 15 | C | C | C | 82.89 | 0.000000e+00 | \* |
| 290 | 15 | C | C | G | 5.92 | 2.674874e-01 |  |
| 290 | 15 | C | C | T | 1.32 | 8.014027e-01 |  |
| 291 | 16 | G | G | A | 5.03 | 4.024164e-01 |  |
| 291 | 16 | G | G | C | 4.47 | 2.816886e-01 |  |
| 291 | 16 | G | G | G | 85.47 | 6.907145e-10 | \* |
| 291 | 16 | G | G | T | 5.03 | 2.545683e-01 |  |
| 292 | 17 | T | T | A | 0.00 | 8.727273e-01 |  |
| 292 | 17 | T | T | C | 1.47 | 7.492172e-01 |  |
| 292 | 17 | T | T | G | 5.88 | 2.698041e-01 |  |
| 292 | 17 | T | T | T | 92.65 | 0.000000e+00 | \* |
| 293 | 18 | G | G | A | 3.77 | 5.376123e-01 |  |
| 293 | 18 | G | G | C | 0.00 | 8.928571e-01 |  |
| 293 | 18 | G | G | G | 92.45 | 1.173365e-10 | \* |
| 293 | 18 | G | G | T | 3.77 | 4.028671e-01 |  |
| 294 | 19 | T | T | A | 0.00 | 8.727273e-01 |  |
| 294 | 19 | T | T | C | 1.25 | 7.844977e-01 |  |
| 294 | 19 | T | T | G | 6.88 | 2.158779e-01 |  |
| 294 | 19 | T | T | T | 91.88 | 0.000000e+00 | \* |
| 295 | 20 | T | T | A | 0.00 | 8.727273e-01 |  |
| 295 | 20 | T | T | C | 0.00 | 8.928571e-01 |  |
| 295 | 20 | T | T | G | 6.43 | 2.385311e-01 |  |
| 295 | 20 | T | T | T | 93.57 | 0.000000e+00 | \* |
| 296 | 21 | T | T | A | 3.61 | 5.568249e-01 |  |
| 296 | 21 | T | T | C | 6.70 | 1.089230e-01 |  |
| 296 | 21 | T | T | G | 6.19 | 2.521364e-01 |  |
| 296 | 21 | T | T | T | 83.51 | 0.000000e+00 | \* |
| 297 | 22 | G | G | A | 3.18 | 6.072183e-01 |  |
| 297 | 22 | G | G | C | 3.64 | 3.872722e-01 |  |
| 297 | 22 | G | G | G | 88.64 | 3.094508e-10 | \* |
| 297 | 22 | G | G | T | 4.55 | 3.054677e-01 |  |
| 298 | 23 | T | T | A | 0.72 | 8.542119e-01 |  |
| 298 | 23 | T | T | C | 2.16 | 6.293979e-01 |  |
| 298 | 23 | T | T | G | 5.76 | 2.775338e-01 |  |
| 298 | 23 | T | T | T | 91.37 | 0.000000e+00 | \* |
| 299 | 24 | G | G | A | 6.59 | 2.685352e-01 |  |
| 299 | 24 | G | G | C | 3.59 | 3.934830e-01 |  |
| 299 | 24 | G | G | G | 88.62 | 3.105225e-10 | \* |
| 299 | 24 | G | G | T | 1.20 | 8.187522e-01 |  |
| 300 | 25 | C | C | A | 5.44 | 3.629141e-01 |  |
| 300 | 25 | C | C | C | 86.39 | 0.000000e+00 | \* |
| 300 | 25 | C | C | G | 3.40 | 4.614008e-01 |  |
| 300 | 25 | C | C | T | 4.76 | 2.817072e-01 |  |
| 301 | 26 | A | A | A | 83.76 | 1.080247e-13 | \* |
| 301 | 26 | A | A | C | 2.56 | 5.578985e-01 |  |
| 301 | 26 | A | A | G | 6.84 | 2.177125e-01 |  |
| 301 | 26 | A | A | T | 6.84 | 1.224878e-01 |  |
| 302 | 27 | C | C | A | 8.52 | 1.548800e-01 |  |
| 302 | 27 | C | C | C | 84.09 | 0.000000e+00 | \* |
| 302 | 27 | C | C | G | 2.84 | 5.176527e-01 |  |
| 302 | 27 | C | C | T | 4.55 | 3.054677e-01 |  |
| 303 | 28 | T | T | A | 6.71 | 2.598486e-01 |  |
| 303 | 28 | T | T | C | 5.49 | 1.852582e-01 |  |
| 303 | 28 | T | T | G | 3.66 | 4.372540e-01 |  |
| 303 | 28 | T | T | T | 84.15 | 0.000000e+00 | \* |
| 304 | 29 | A | A | A | 92.67 | 3.330669e-15 | \* |
| 304 | 29 | A | A | C | 4.67 | 2.603125e-01 |  |
| 304 | 29 | A | A | G | 0.00 | 8.351648e-01 |  |
| 304 | 29 | A | A | T | 2.67 | 5.747841e-01 |  |
| 305 | 30 | C | C | A | 9.80 | 1.053288e-01 |  |
| 305 | 30 | C | C | C | 83.66 | 0.000000e+00 | \* |
| 305 | 30 | C | C | G | 3.92 | 4.136381e-01 |  |
| 305 | 30 | C | C | T | 2.61 | 5.836270e-01 |  |
| 306 | 31 | A | A | A | 82.91 | 1.509903e-13 | \* |
| 306 | 31 | A | A | C | 5.98 | 1.497047e-01 |  |
| 306 | 31 | A | A | G | 11.11 | 8.090237e-02 |  |
| 306 | 31 | A | A | T | 0.00 | 9.139785e-01 |  |
| 307 | 32 | C | C | A | 4.29 | 4.798793e-01 |  |
| 307 | 32 | C | C | C | 82.86 | 0.000000e+00 | \* |
| 307 | 32 | C | C | G | 9.29 | 1.240782e-01 |  |
| 307 | 32 | C | C | T | 3.57 | 4.316331e-01 |  |
| 308 | 33 | G | G | A | 6.03 | 3.113675e-01 |  |
| 308 | 33 | G | G | C | 7.76 | 6.709831e-02 |  |
| 308 | 33 | G | G | G | 86.21 | 5.735500e-10 | \* |
| 308 | 33 | G | G | T | 0.00 | 9.139785e-01 |  |
| 309 | 34 | G | G | A | 3.89 | 5.243616e-01 |  |
| 309 | 34 | G | G | C | 3.33 | 4.318652e-01 |  |
| 309 | 34 | G | G | G | 90.56 | 1.900338e-10 | \* |
| 309 | 34 | G | G | T | 2.22 | 6.509442e-01 |  |
| 310 | 35 | G | G | A | 4.83 | 4.225113e-01 |  |
| 310 | 35 | G | G | C | 4.14 | 3.206793e-01 |  |
| 310 | 35 | G | G | G | 87.59 | 4.040543e-10 | \* |
| 310 | 35 | G | G | T | 3.45 | 4.497916e-01 |  |
| 311 | 36 | C | C | A | 10.29 | 9.055483e-02 |  |
| 311 | 36 | C | C | C | 83.09 | 0.000000e+00 | \* |
| 311 | 36 | C | C | G | 3.68 | 4.356093e-01 |  |
| 311 | 36 | C | C | T | 2.94 | 5.291902e-01 |  |
| 312 | 37 | A | A | A | 90.55 | 7.549517e-15 | \* |
| 312 | 37 | A | A | C | 3.15 | 4.604136e-01 |  |
| 312 | 37 | A | A | G | 3.15 | 4.860557e-01 |  |
| 312 | 37 | A | A | T | 3.15 | 4.957094e-01 |  |
| 313 | 38 | C | C | A | 8.79 | 1.430338e-01 |  |
| 313 | 38 | C | C | C | 85.71 | 0.000000e+00 | \* |
| 313 | 38 | C | C | G | 3.30 | 4.715280e-01 |  |
| 313 | 38 | C | C | T | 2.20 | 6.551657e-01 |  |
| 314 | 39 | C | C | A | 11.76 | 5.696443e-02 |  |
| 314 | 39 | C | C | C | 80.21 | 0.000000e+00 | \* |
| 314 | 39 | C | C | G | 5.35 | 3.037490e-01 |  |
| 314 | 39 | C | C | T | 2.67 | 5.735817e-01 |  |
| 315 | 40 | C | C | A | 13.76 | 2.979729e-02 |  |
| 315 | 40 | C | C | C | 82.54 | 0.000000e+00 | \* |
| 315 | 40 | C | C | G | 0.00 | 8.351648e-01 |  |
| 315 | 40 | C | C | T | 3.70 | 4.126619e-01 |  |
| 316 | 41 | T | T | A | 0.00 | 8.727273e-01 |  |
| 316 | 41 | T | T | C | 1.38 | 7.641245e-01 |  |
| 316 | 41 | T | T | G | 7.59 | 1.836312e-01 |  |
| 316 | 41 | T | T | T | 91.03 | 0.000000e+00 | \* |
| 317 | 42 | G | G | A | 6.22 | 2.964157e-01 |  |
| 317 | 42 | G | G | C | 0.96 | 8.264043e-01 |  |
| 317 | 42 | G | G | G | 91.87 | 1.362079e-10 | \* |
| 317 | 42 | G | G | T | 0.96 | 8.509940e-01 |  |
| 318 | 43 | A | A | A | 57.80 | 2.596890e-09 | \* |
| 318 | 43 | A | A | C | 1.73 | 7.043873e-01 |  |
| 318 | 43 | A | A | G | 1.73 | 6.423201e-01 |  |
| 318 | 43 | A | A | T | 38.73 | 1.273740e-08 | \* |
| 319 | 44 | C | C | A | 8.00 | 1.804584e-01 |  |
| 319 | 44 | C | C | C | 84.67 | 0.000000e+00 | \* |
| 319 | 44 | C | C | G | 6.67 | 2.262868e-01 |  |
| 319 | 44 | C | C | T | 0.67 | 8.827314e-01 |  |
| 320 | 45 | C | C | A | 6.99 | 2.401659e-01 |  |
| 320 | 45 | C | C | C | 83.92 | 0.000000e+00 | \* |
| 320 | 45 | C | C | G | 9.09 | 1.298244e-01 |  |
| 320 | 45 | C | C | T | 0.00 | 9.139785e-01 |  |
| 321 | 46 | G | G | A | 9.09 | 1.307726e-01 |  |
| 321 | 46 | G | G | C | 5.79 | 1.631123e-01 |  |
| 321 | 46 | G | G | G | 82.64 | 1.416847e-09 | \* |
| 321 | 46 | G | G | T | 2.48 | 6.066458e-01 |  |
| 322 | 47 | A | A | A | 94.98 | 1.332268e-15 | \* |
| 322 | 47 | A | A | C | 0.72 | 8.548146e-01 |  |
| 322 | 47 | A | A | G | 1.08 | 7.222710e-01 |  |
| 322 | 47 | A | A | T | 3.23 | 4.837523e-01 |  |
| 323 | 48 | C | C | A | 8.33 | 1.637524e-01 |  |
| 323 | 48 | C | C | C | 83.33 | 0.000000e+00 | \* |
| 323 | 48 | C | C | G | 0.69 | 7.680279e-01 |  |
| 323 | 48 | C | C | T | 7.64 | 8.695789e-02 |  |
| 324 | 49 | G | G | A | 0.00 | 8.727273e-01 |  |
| 324 | 49 | G | G | C | 2.56 | 5.578985e-01 |  |
| 324 | 49 | G | G | G | 97.44 | 3.305356e-11 | \* |
| 324 | 49 | G | G | T | 0.00 | 9.139785e-01 |  |
| 325 | 50 | G | G | A | 0.00 | 8.727273e-01 |  |
| 325 | 50 | G | G | C | 0.60 | 8.659815e-01 |  |
| 325 | 50 | G | G | G | 96.99 | 3.704181e-11 | \* |
| 325 | 50 | G | G | T | 2.41 | 6.186094e-01 |  |
| 326 | 51 | C | C | A | 10.58 | 8.292614e-02 |  |
| 326 | 51 | C | C | C | 86.54 | 0.000000e+00 | \* |
| 326 | 51 | C | C | G | 2.88 | 5.130886e-01 |  |
| 326 | 51 | C | C | T | 0.00 | 9.139785e-01 |  |
| 327 | 52 | A | A | A | 85.88 | 4.707346e-14 | \* |
| 327 | 52 | A | A | C | 1.18 | 7.956237e-01 |  |
| 327 | 52 | A | A | G | 2.35 | 5.705692e-01 |  |
| 327 | 52 | A | A | T | 10.59 | 2.304850e-02 |  |
| 328 | 53 | A | A | A | 94.49 | 1.665335e-15 | \* |
| 328 | 53 | A | A | C | 0.00 | 8.928571e-01 |  |
| 328 | 53 | A | A | G | 1.57 | 6.614508e-01 |  |
| 328 | 53 | A | A | T | 3.94 | 3.805866e-01 |  |
| 329 | 54 | G | G | A | 5.88 | 3.240485e-01 |  |
| 329 | 54 | G | G | C | 1.96 | 6.645089e-01 |  |
| 329 | 54 | G | G | G | 83.33 | 1.189620e-09 | \* |
| 329 | 54 | G | G | T | 8.82 | 5.158064e-02 |  |
| 330 | 55 | A | A | A | 93.83 | 2.109424e-15 | \* |
| 330 | 55 | A | A | C | 0.00 | 8.928571e-01 |  |
| 330 | 55 | A | A | G | 1.23 | 7.028269e-01 |  |
| 330 | 55 | A | A | T | 4.94 | 2.634652e-01 |  |
| 331 | 56 | A | A | A | 92.56 | 3.441691e-15 | \* |
| 331 | 56 | A | A | C | 0.00 | 8.928571e-01 |  |
| 331 | 56 | A | A | G | 2.48 | 5.565256e-01 |  |
| 331 | 56 | A | A | T | 4.96 | 2.614182e-01 |  |
| 332 | 57 | G | G | A | 1.10 | 8.298486e-01 |  |
| 332 | 57 | G | G | C | 3.30 | 4.374688e-01 |  |
| 332 | 57 | G | G | G | 80.22 | 2.621436e-09 | \* |
| 332 | 57 | G | G | T | 15.38 | 2.308919e-03 | \* |
| 333 | 58 | T | T | A | 0.73 | 8.536596e-01 |  |
| 333 | 58 | T | T | C | 3.65 | 3.853930e-01 |  |
| 333 | 58 | T | T | G | 0.73 | 7.638530e-01 |  |
| 333 | 58 | T | T | T | 94.89 | 0.000000e+00 | \* |
| 334 | 59 | T | T | A | 0.00 | 8.727273e-01 |  |
| 334 | 59 | T | T | C | 0.00 | 8.928571e-01 |  |
| 334 | 59 | T | T | G | 6.11 | 2.566165e-01 |  |
| 334 | 59 | T | T | T | 93.89 | 0.000000e+00 | \* |
| 335 | 60 | C | C | A | 5.88 | 3.240485e-01 |  |
| 335 | 60 | C | C | C | 87.50 | 0.000000e+00 | \* |
| 335 | 60 | C | C | G | 3.68 | 4.356093e-01 |  |
| 335 | 60 | C | C | T | 2.94 | 5.291902e-01 |  |
| 336 | 61 | G | G | A | 13.00 | 3.820523e-02 |  |
| 336 | 61 | G | G | C | 6.00 | 1.485932e-01 |  |
| 336 | 61 | G | G | G | 77.00 | 5.931721e-09 | \* |
| 336 | 61 | G | G | T | 4.00 | 3.722349e-01 |  |
| 337 | 62 | A | A | A | 88.66 | 1.587619e-14 | \* |
| 337 | 62 | A | A | C | 3.09 | 4.694609e-01 |  |
| 337 | 62 | A | A | G | 3.09 | 4.917594e-01 |  |
| 337 | 62 | A | A | T | 5.15 | 2.424202e-01 |  |
| 338 | 63 | C | C | A | 5.44 | 3.629141e-01 |  |
| 338 | 63 | C | C | C | 86.39 | 0.000000e+00 | \* |
| 338 | 63 | C | C | G | 4.08 | 3.997962e-01 |  |
| 338 | 63 | C | C | T | 4.08 | 3.616089e-01 |  |
| 339 | 64 | A | A | A | 85.00 | 6.661338e-14 | \* |
| 339 | 64 | A | A | C | 7.00 | 9.515254e-02 |  |
| 339 | 64 | A | A | G | 5.00 | 3.278377e-01 |  |
| 339 | 64 | A | A | T | 3.00 | 5.196318e-01 |  |
| 340 | 65 | G | G | A | 4.23 | 4.865225e-01 |  |
| 340 | 65 | G | G | C | 2.82 | 5.147292e-01 |  |
| 340 | 65 | G | G | G | 92.96 | 1.032042e-10 | \* |
| 340 | 65 | G | G | T | 0.00 | 9.139785e-01 |  |
| 341 | 66 | C | C | A | 5.34 | 3.720678e-01 |  |
| 341 | 66 | C | C | C | 89.31 | 0.000000e+00 | \* |
| 341 | 66 | C | C | G | 3.82 | 4.229149e-01 |  |
| 341 | 66 | C | C | T | 1.53 | 7.685450e-01 |  |
| 342 | 67 | T | T | A | 1.27 | 8.158937e-01 |  |
| 342 | 67 | T | T | C | 7.64 | 7.079609e-02 |  |
| 342 | 67 | T | T | G | 3.82 | 4.224806e-01 |  |
| 342 | 67 | T | T | T | 87.26 | 0.000000e+00 | \* |
| 343 | 68 | C | C | A | 5.00 | 4.051801e-01 |  |
| 343 | 68 | C | C | C | 84.29 | 0.000000e+00 | \* |
| 343 | 68 | C | C | G | 6.43 | 2.387553e-01 |  |
| 343 | 68 | C | C | T | 4.29 | 3.360208e-01 |  |
| 344 | 69 | C | C | A | 5.36 | 3.707936e-01 |  |
| 344 | 69 | C | C | C | 87.50 | 0.000000e+00 | \* |
| 344 | 69 | C | C | G | 0.00 | 8.351648e-01 |  |
| 344 | 69 | C | C | T | 7.14 | 1.076237e-01 |  |
| 345 | 70 | C | C | A | 11.21 | 6.785455e-02 |  |
| 345 | 70 | C | C | C | 85.98 | 0.000000e+00 | \* |
| 345 | 70 | C | C | G | 2.80 | 5.215576e-01 |  |
| 345 | 70 | C | C | T | 0.00 | 9.139785e-01 |  |
| 346 | 71 | G | G | A | 0.00 | 8.727273e-01 |  |
| 346 | 71 | G | G | C | 1.01 | 8.193230e-01 |  |
| 346 | 71 | G | G | G | 95.96 | 4.811296e-11 | \* |
| 346 | 71 | G | G | T | 3.03 | 5.147404e-01 |  |
| 347 | 72 | C | C | A | 13.08 | 3.725663e-02 |  |
| 347 | 72 | C | C | C | 80.77 | 0.000000e+00 | \* |
| 347 | 72 | C | C | G | 0.00 | 8.351648e-01 |  |
| 347 | 72 | C | C | T | 6.15 | 1.627340e-01 |  |
| 348 | 73 | G | G | A | 21.15 | 2.348401e-03 | \* |
| 348 | 73 | G | G | C | 7.69 | 6.920219e-02 |  |
| 348 | 73 | G | G | G | 71.15 | 2.609386e-08 | \* |
| 348 | 73 | G | G | T | 0.00 | 9.139785e-01 |  |
| 349 | 74 | A | A | A | 92.73 | 3.219647e-15 | \* |
| 349 | 74 | A | A | C | 1.21 | 7.902745e-01 |  |
| 349 | 74 | A | A | G | 0.00 | 8.351648e-01 |  |
| 349 | 74 | A | A | T | 6.06 | 1.690503e-01 |  |
| 350 | 75 | C | C | A | 4.86 | 4.191049e-01 |  |
| 350 | 75 | C | C | C | 85.42 | 0.000000e+00 | \* |
| 350 | 75 | C | C | G | 1.39 | 6.840009e-01 |  |
| 350 | 75 | C | C | T | 8.33 | 6.415623e-02 |  |

## For use in R

If you want to work with the results in R, here is output that you can copy and paste in your terminal to get:

The base information:

```
structure(list(focal.base = c("A", "C", "G", "T"), avg.percsignal = c(88.5561008433191, 
85.7363512282149, 87.8012695863252, 90.2439552598084), avg.areasignal = c(160.705882352941, 
168.179104477612, 154.535714285714, 186.045454545455), crit.perc.area = c(16.9974941605459, 
11.6631763006079, 19.800986235304, 12.3618399485341), mu = c(5.48945957344982, 
3.77825017641277, 5.09747129843509, 3.97283433243147), fillibens = c(0.997168836389872, 
0.992357927012538, 0.980794482293667, 0.991053632385798)), .Names = c("focal.base", 
"avg.percsignal", "avg.areasignal", "crit.perc.area", "mu", "fillibens"
), row.names = c(NA, -4L), class = "data.frame")
```

the data.frame that contains information on the guide region:

```
structure(list(A.area = c(148, 166, 7, 325, 13, 0, 16, 0, 16, 
15, 121, 15, 319, 12, 15, 9, 0, 10, 0, 0, 7, 7, 1, 11, 8, 98, 
15, 11, 139, 15, 97, 6, 7, 7, 7, 14, 115, 16, 22, 26, 0, 13, 
100, 12, 10, 11, 265, 12, 0, 0, 11, 73, 120, 6, 152, 112, 1, 
1, 0, 8, 13, 172, 8, 85, 6, 7, 2, 7, 9, 12, 0, 17, 22, 153, 7
), C.area = c(2, 0, 8, 3, 156, 3, 2, 4, 116, 167, 2, 0, 6, 161, 
126, 8, 2, 0, 2, 0, 13, 8, 3, 6, 127, 3, 148, 9, 7, 128, 7, 116, 
9, 6, 6, 113, 4, 156, 150, 156, 2, 2, 3, 127, 120, 7, 2, 120, 
2, 1, 90, 1, 0, 2, 0, 0, 3, 5, 0, 119, 6, 6, 127, 7, 4, 117, 
12, 118, 147, 92, 1, 105, 8, 2, 123), G.area = c(0, 6, 107, 4, 
0, 6, 296, 190, 4, 4, 12, 128, 2, 0, 9, 153, 8, 245, 11, 11, 
12, 195, 8, 148, 5, 8, 5, 6, 0, 6, 13, 13, 100, 163, 127, 5, 
4, 6, 10, 0, 11, 192, 3, 10, 13, 100, 3, 1, 76, 161, 3, 2, 2, 
85, 2, 3, 73, 1, 8, 5, 77, 6, 6, 5, 132, 5, 6, 9, 0, 3, 95, 0, 
74, 0, 2), T.area = c(1, 1, 0, 16, 7, 167, 6, 1, 11, 11, 0, 1, 
18, 8, 2, 9, 126, 10, 147, 160, 162, 10, 127, 2, 7, 8, 8, 138, 
4, 4, 0, 5, 0, 4, 5, 4, 4, 4, 5, 7, 132, 2, 67, 1, 0, 3, 9, 11, 
0, 4, 0, 9, 5, 9, 8, 6, 14, 130, 123, 4, 4, 10, 6, 3, 0, 2, 137, 
6, 12, 0, 3, 8, 0, 10, 12), Tot.area = c(151, 173, 122, 348, 
176, 176, 320, 195, 147, 197, 135, 144, 345, 181, 152, 179, 136, 
265, 160, 171, 194, 220, 139, 167, 147, 117, 176, 164, 150, 153, 
117, 140, 116, 180, 145, 136, 127, 182, 187, 189, 145, 209, 173, 
150, 143, 121, 279, 144, 78, 166, 104, 85, 127, 102, 162, 121, 
91, 137, 131, 136, 100, 194, 147, 100, 142, 131, 157, 140, 168, 
107, 99, 130, 104, 165, 144), A.perc = c(98.0132450331126, 95.9537572254335, 
5.73770491803279, 93.3908045977011, 7.38636363636364, 0, 5, 0, 
10.8843537414966, 7.61421319796954, 89.6296296296296, 10.4166666666667, 
92.463768115942, 6.62983425414365, 9.86842105263158, 5.02793296089385, 
0, 3.77358490566038, 0, 0, 3.60824742268041, 3.18181818181818, 
0.719424460431655, 6.58682634730539, 5.4421768707483, 83.7606837606838, 
8.52272727272727, 6.70731707317073, 92.6666666666667, 9.80392156862745, 
82.9059829059829, 4.28571428571429, 6.03448275862069, 3.88888888888889, 
4.82758620689655, 10.2941176470588, 90.5511811023622, 8.79120879120879, 
11.7647058823529, 13.7566137566138, 0, 6.2200956937799, 57.8034682080925, 
8, 6.99300699300699, 9.09090909090909, 94.9820788530466, 8.33333333333333, 
0, 0, 10.5769230769231, 85.8823529411765, 94.488188976378, 5.88235294117647, 
93.8271604938272, 92.5619834710744, 1.0989010989011, 0.72992700729927, 
0, 5.88235294117647, 13, 88.659793814433, 5.4421768707483, 85, 
4.22535211267606, 5.34351145038168, 1.27388535031847, 5, 5.35714285714286, 
11.214953271028, 0, 13.0769230769231, 21.1538461538462, 92.7272727272727, 
4.86111111111111), C.perc = c(1.32450331125828, 0, 6.55737704918033, 
0.862068965517241, 88.6363636363636, 1.70454545454545, 0.625, 
2.05128205128205, 78.9115646258503, 84.7715736040609, 1.48148148148148, 
0, 1.73913043478261, 88.9502762430939, 82.8947368421053, 4.46927374301676, 
1.47058823529412, 0, 1.25, 0, 6.70103092783505, 3.63636363636364, 
2.15827338129496, 3.59281437125748, 86.3945578231292, 2.56410256410256, 
84.0909090909091, 5.48780487804878, 4.66666666666667, 83.6601307189542, 
5.98290598290598, 82.8571428571429, 7.75862068965517, 3.33333333333333, 
4.13793103448276, 83.0882352941177, 3.1496062992126, 85.7142857142857, 
80.2139037433155, 82.5396825396825, 1.37931034482759, 0.956937799043062, 
1.73410404624277, 84.6666666666667, 83.9160839160839, 5.78512396694215, 
0.716845878136201, 83.3333333333333, 2.56410256410256, 0.602409638554217, 
86.5384615384615, 1.17647058823529, 0, 1.96078431372549, 0, 0, 
3.2967032967033, 3.64963503649635, 0, 87.5, 6, 3.09278350515464, 
86.3945578231292, 7, 2.8169014084507, 89.3129770992366, 7.64331210191083, 
84.2857142857143, 87.5, 85.981308411215, 1.01010101010101, 80.7692307692308, 
7.69230769230769, 1.21212121212121, 85.4166666666667), G.perc = c(0, 
3.46820809248555, 87.7049180327869, 1.14942528735632, 0, 3.40909090909091, 
92.5, 97.4358974358974, 2.72108843537415, 2.03045685279188, 8.88888888888889, 
88.8888888888889, 0.579710144927536, 0, 5.92105263157895, 85.4748603351955, 
5.88235294117647, 92.4528301886792, 6.875, 6.4327485380117, 6.18556701030928, 
88.6363636363636, 5.75539568345324, 88.622754491018, 3.40136054421769, 
6.83760683760684, 2.84090909090909, 3.65853658536585, 0, 3.92156862745098, 
11.1111111111111, 9.28571428571429, 86.2068965517241, 90.5555555555556, 
87.5862068965517, 3.67647058823529, 3.1496062992126, 3.2967032967033, 
5.3475935828877, 0, 7.58620689655172, 91.866028708134, 1.73410404624277, 
6.66666666666667, 9.09090909090909, 82.6446280991736, 1.0752688172043, 
0.694444444444444, 97.4358974358974, 96.9879518072289, 2.88461538461538, 
2.35294117647059, 1.5748031496063, 83.3333333333333, 1.23456790123457, 
2.47933884297521, 80.2197802197802, 0.72992700729927, 6.10687022900763, 
3.67647058823529, 77, 3.09278350515464, 4.08163265306122, 5, 
92.9577464788732, 3.81679389312977, 3.82165605095541, 6.42857142857143, 
0, 2.80373831775701, 95.959595959596, 0, 71.1538461538462, 0, 
1.38888888888889), T.perc = c(0.662251655629139, 0.578034682080925, 
0, 4.59770114942529, 3.97727272727273, 94.8863636363636, 1.875, 
0.512820512820513, 7.48299319727891, 5.58375634517767, 0, 0.694444444444444, 
5.21739130434783, 4.41988950276243, 1.31578947368421, 5.02793296089385, 
92.6470588235294, 3.77358490566038, 91.875, 93.5672514619883, 
83.5051546391753, 4.54545454545455, 91.3669064748201, 1.19760479041916, 
4.76190476190476, 6.83760683760684, 4.54545454545455, 84.1463414634146, 
2.66666666666667, 2.61437908496732, 0, 3.57142857142857, 0, 2.22222222222222, 
3.44827586206897, 2.94117647058824, 3.1496062992126, 2.1978021978022, 
2.67379679144385, 3.7037037037037, 91.0344827586207, 0.956937799043062, 
38.728323699422, 0.666666666666667, 0, 2.47933884297521, 3.2258064516129, 
7.63888888888889, 0, 2.40963855421687, 0, 10.5882352941176, 3.93700787401575, 
8.82352941176471, 4.93827160493827, 4.95867768595041, 15.3846153846154, 
94.8905109489051, 93.8931297709924, 2.94117647058824, 4, 5.15463917525773, 
4.08163265306122, 3, 0, 1.52671755725191, 87.2611464968153, 4.28571428571429, 
7.14285714285714, 0, 3.03030303030303, 6.15384615384615, 0, 6.06060606060606, 
8.33333333333333), base.call = c("A", "A", "G", "A", "C", "T", 
"G", "G", "C", "C", "A", "G", "A", "C", "C", "G", "T", "G", "T", 
"T", "T", "G", "T", "G", "C", "A", "C", "T", "A", "C", "A", "C", 
"G", "G", "G", "C", "A", "C", "C", "C", "T", "G", "A", "C", "C", 
"G", "A", "C", "G", "G", "C", "A", "A", "G", "A", "A", "G", "T", 
"T", "C", "G", "A", "C", "A", "G", "C", "T", "C", "C", "C", "G", 
"C", "G", "A", "C"), index = 276:350, guide.seq = c("A", "A", 
"G", "A", "C", "T", "G", "G", "C", "C", "A", "G", "A", "C", "C", 
"G", "T", "G", "T", "T", "T", "G", "T", "G", "C", "A", "C", "T", 
"A", "C", "A", "C", "G", "G", "G", "C", "A", "C", "C", "C", "T", 
"G", "A", "C", "C", "G", "A", "C", "G", "G", "C", "A", "A", "G", 
"A", "A", "G", "T", "T", "C", "G", "A", "C", "A", "G", "C", "T", 
"C", "C", "C", "G", "C", "G", "A", "C"), T.pval = c(0.883141823806103, 
0.890507075598871, 0.913978494580569, 0.299592076248692, 0.375232874363088, 
0, 0.710694109545061, 0.895579910850286, 0.0930200834939615, 
0.204831792358706, 0.913978494580569, 0.880095355769675, 0.236584961660817, 
0.319957458389854, 0.801402678572855, 0.254568314002292, 0, 0.402867051203505, 
0, 0, 0, 0.30546765708197, 0, 0.81875219990382, 0.281707189862996, 
0.122487770966986, 0.30546765708197, 0, 0.574784106858011, 0.583627029149961, 
0.913978494580569, 0.431633081618176, 0.913978494580569, 0.650944235837832, 
0.449791628178006, 0.529190188438154, 0.495709353669008, 0.655165695581341, 
0.573581686946337, 0.412661908785659, 0, 0.850993950966107, 1.27374041314354e-08, 
0.882731396777236, 0.913978494580569, 0.606645840389397, 0.483752298910936, 
0.0869578869774025, 0.913978494580569, 0.618609360817878, 0.913978494580569, 
0.0230484965619815, 0.380586559071897, 0.0515806364907694, 0.263465198419835, 
0.261418231500859, 0.00230891881918094, 0, 0, 0.529190188438154, 
0.37223487671215, 0.242420166075936, 0.361608899749203, 0.519631791046484, 
0.913978494580569, 0.768545011456799, 0, 0.336020784356697, 0.107623662011262, 
0.913978494580569, 0.514740367064105, 0.162733966941127, 0.913978494580569, 
0.16905025045764, 0.0641562262713143), C.pval = c(0.772875333945616, 
0.892857142856464, 0.116167577788391, 0.838367565182632, 0, 0.709518713268357, 
0.863911736532459, 0.648432825076009, 0, 0, 0.747412963695452, 
0.892857142856464, 0.703512682324829, 0, 0, 0.281688568117288, 
0.749217242426398, 0.892857142856464, 0.784497655519784, 0.892857142856464, 
0.108922977246028, 0.387272248642686, 0.629397916277024, 0.393482962478802, 
0, 0.557898532258784, 0, 0.185258160749292, 0.260312501519974, 
0, 0.149704653017329, 0, 0.0670983063693489, 0.431865160984631, 
0.320679316664362, 0, 0.460413601565254, 0, 0, 0, 0.7641244768353, 
0.826404282132842, 0.704387281243001, 0, 0, 0.163112332191781, 
0.854814606436371, 0, 0.557898532258784, 0.865981463880025, 0, 
0.795623716791476, 0.892857142856464, 0.664508866575805, 0.892857142856464, 
0.892857142856464, 0.437468760748091, 0.385393006277533, 0.892857142856464, 
0, 0.148593152611848, 0.469460899465612, 0, 0.0951525442203119, 
0.514729231756038, 0, 0.0707960942221892, 0, 0, 0, 0.819323007054627, 
0, 0.0692021879240022, 0.790274480072616, 0), G.pval = c(0.835164835164835, 
0.45502341348007, 3.92053278730486e-10, 0.713224606164179, 0.835164835164835, 
0.460659693351371, 1.15938147970951e-10, 3.30535598891402e-11, 
0.530316106571054, 0.607384872851963, 0.13605320365188, 2.9022284575575e-10, 
0.781315755748774, 0.835164835164835, 0.267487433407886, 6.90714485607202e-10, 
0.269804094152951, 1.17336473870466e-10, 0.215877863128718, 0.238531109468778, 
0.25213644947323, 3.09450798319233e-10, 0.277533827445443, 3.10522496604904e-10, 
0.461400817814007, 0.217712521926464, 0.517652697303956, 0.437254040990562, 
0.835164835164835, 0.413638137780691, 0.080902374450749, 0.124078209500861, 
5.7354998528325e-10, 1.90033766500619e-10, 4.04054345537475e-10, 
0.435609266487503, 0.486055701817313, 0.47152795325671, 0.303748978807405, 
0.835164835164835, 0.183631152017501, 1.36207933820742e-10, 0.642320133424943, 
0.226286765525201, 0.12982444764159, 1.41684675103448e-09, 0.722270975368957, 
0.768027908824786, 3.30535598891402e-11, 3.70418140605011e-11, 
0.513088599583673, 0.570569164030776, 0.661450820151393, 1.18962018014201e-09, 
0.702826866126634, 0.556525646522683, 2.62143640128443e-09, 0.763853024726764, 
0.256616504294669, 0.435609266487503, 5.93172078122706e-09, 0.491759434200824, 
0.399796168446409, 0.327837746852997, 1.03204222945408e-10, 0.42291494477069, 
0.422480642597431, 0.238755256521164, 0.835164835164835, 0.521557551836252, 
4.81129580620632e-11, 0.835164835164835, 2.60938594065507e-08, 
0.835164835164835, 0.684000891268212), A.pval = c(3.33066907387547e-16, 
8.88178419700125e-16, 0.336463715481323, 2.55351295663786e-15, 
0.215105421903569, 0.87272727272726, 0.405180085482336, 0.87272727272726, 
0.0753122276651857, 0.201627698521647, 1.08801856413265e-14, 
0.087172662060933, 3.5527136788005e-15, 0.265408025568763, 0.103265924146888, 
0.402416381940841, 0.87272727272726, 0.537612305816602, 0.87272727272726, 
0.87272727272726, 0.556824884403553, 0.607218286644328, 0.854211860953548, 
0.26853520265251, 0.362914060436676, 1.08024700296028e-13, 0.154880011172481, 
0.259848553716735, 3.33066907387547e-15, 0.105328828328431, 1.50990331349021e-13, 
0.479879302694764, 0.311367472842056, 0.524361626126915, 0.422511280606641, 
0.0905548317008683, 7.54951656745106e-15, 0.14303382030777, 0.0569644274410751, 
0.0297972855118303, 0.87272727272726, 0.296415720658709, 2.59688992532148e-09, 
0.180458395792272, 0.240165910919236, 0.130772565698745, 1.33226762955019e-15, 
0.163752425164954, 0.87272727272726, 0.87272727272726, 0.0829261355942542, 
4.70734562441066e-14, 1.66533453693773e-15, 0.32404848131232, 
2.1094237467878e-15, 3.44169137633799e-15, 0.829848639772748, 
0.853659641907022, 0.87272727272726, 0.32404848131232, 0.0382052271132208, 
1.58761892521397e-14, 0.362914060436676, 6.66133814775094e-14, 
0.486522490889, 0.372067848949562, 0.815893745258454, 0.405180085482336, 
0.370793608010473, 0.0678545505594538, 0.87272727272726, 0.037256625811187, 
0.00234840119769963, 3.21964677141295e-15, 0.419104933663298), 
    guide.position = 1:75), .Names = c("A.area", "C.area", "G.area", 
"T.area", "Tot.area", "A.perc", "C.perc", "G.perc", "T.perc", 
"base.call", "index", "guide.seq", "T.pval", "C.pval", "G.pval", 
"A.pval", "guide.position"), row.names = 276:350, class = "data.frame")
```

*Report generated using EditR v1.0.8*
