# Supplementary material for: Mechanistic and genetic basis of single-strand templated repair at Cas12a-induced DNA breaks in Chlamydomonas reinhardtii
Source: Nat Commun. 2021 Nov 19;12:6751. doi: 10.1038/s41467-021-27004-1 (PMC8604939; doi:10.1038/s41467-021-27004-1)
Supplement: Supplementary file 22 — Source Data [file 41467_2021_27004_MOESM22_ESM.zip › Source Data/EditR analysis/EditR outputs/Sense/rep3_ssODN_sense_0_16_32.html]

EditR v1.0.8 report


# EditR v1.0.8 report

- Data QA
  - Filtering data
  - Percent noise peak area
  - Base information
- Predicted editing
  - Editing bar plot
  - Editing table plot
  - Table of editing results
- For use in R

## Data QA

### Filtering data

What the data looked like prefiltering:

and the post filtering signal / noise plot:

### Percent noise peak area

### Base information

Here’s information about the signal of each base, the critical percent value where any higher value would be called as significant, and Filliben’s correlation for how well the noise was modelled by the zero adjusted gamma distribution.

| Base | Average percent signal | Average peak area | Critical percent value | model mu | Fillibens correlation |
| --- | --- | --- | --- | --- | --- |
| A | 94.18533 | 165.0294 | 9.962842 | 3.772412 | 0.9913788 |
| C | 90.14185 | 170.1970 | 5.650542 | 2.435771 | 0.9966842 |
| G | 91.91033 | 169.7544 | 9.529496 | 3.337139 | 0.9936083 |
| T | 93.39009 | 190.9545 | 7.611980 | 2.636588 | 0.9948576 |

## Predicted editing

### Editing bar plot

### Editing table plot

### Table of editing results


Here’s the entire guide region

| Sanger position | Guide position | Guide sequence | Sanger base call | Focal base | Focal base peak area | p value |  |
| --- | --- | --- | --- | --- | --- | --- | --- |
| 275 | 1 | A | A | A | 95.07 | 0.000000000 | \* |
| 275 | 1 | A | A | C | 2.11 | 0.486433921 |  |
| 275 | 1 | A | A | G | 2.11 | 0.603917139 |  |
| 275 | 1 | A | A | T | 0.70 | 0.816887301 |  |
| 276 | 2 | A | A | A | 96.41 | 0.000000000 | \* |
| 276 | 2 | A | A | C | 1.80 | 0.594161679 |  |
| 276 | 2 | A | A | G | 1.20 | 0.781423242 |  |
| 276 | 2 | A | A | T | 0.60 | 0.833021610 |  |
| 277 | 3 | G | G | A | 4.51 | 0.261358461 |  |
| 277 | 3 | G | G | C | 5.26 | 0.016784216 |  |
| 277 | 3 | G | G | G | 89.47 | 0.000000000 | \* |
| 277 | 3 | G | G | T | 0.75 | 0.808779634 |  |
| 278 | 4 | A | A | A | 97.26 | 0.000000000 | \* |
| 278 | 4 | A | A | C | 1.22 | 0.769913363 |  |
| 278 | 4 | A | A | G | 1.52 | 0.723772204 |  |
| 278 | 4 | A | A | T | 0.00 | 0.870967742 |  |
| 279 | 5 | C | C | A | 6.84 | 0.072953383 |  |
| 279 | 5 | C | C | C | 82.63 | 0.000000000 | \* |
| 279 | 5 | C | C | G | 5.79 | 0.102062744 |  |
| 279 | 5 | C | C | T | 4.74 | 0.091978845 |  |
| 280 | 6 | T | T | A | 0.00 | 0.863636364 |  |
| 280 | 6 | T | T | C | 2.25 | 0.441821267 |  |
| 280 | 6 | T | T | G | 2.81 | 0.463332053 |  |
| 280 | 6 | T | T | T | 94.94 | 0.000000000 | \* |
| 281 | 7 | G | G | A | 4.30 | 0.289713838 |  |
| 281 | 7 | G | G | C | 1.43 | 0.711212637 |  |
| 281 | 7 | G | G | G | 93.41 | 0.000000000 | \* |
| 281 | 7 | G | G | T | 0.86 | 0.788772600 |  |
| 282 | 8 | G | G | A | 4.95 | 0.209125006 |  |
| 282 | 8 | G | G | C | 1.80 | 0.592330776 |  |
| 282 | 8 | G | G | G | 92.34 | 0.000000000 | \* |
| 282 | 8 | G | G | T | 0.90 | 0.780537996 |  |
| 283 | 9 | C | C | A | 7.09 | 0.062806126 |  |
| 283 | 9 | C | C | C | 84.40 | 0.000000000 | \* |
| 283 | 9 | C | C | G | 4.96 | 0.162023521 |  |
| 283 | 9 | C | C | T | 3.55 | 0.208593722 |  |
| 284 | 10 | C | C | A | 4.12 | 0.314465242 |  |
| 284 | 10 | C | C | C | 90.21 | 0.000000000 | \* |
| 284 | 10 | C | C | G | 3.09 | 0.410550189 |  |
| 284 | 10 | C | C | T | 2.58 | 0.376570568 |  |
| 285 | 11 | A | A | A | 94.93 | 0.000000000 | \* |
| 285 | 11 | A | A | C | 2.17 | 0.465954762 |  |
| 285 | 11 | A | A | G | 2.90 | 0.446303749 |  |
| 285 | 11 | A | A | T | 0.00 | 0.870967742 |  |
| 286 | 12 | G | G | A | 5.41 | 0.164967334 |  |
| 286 | 12 | G | G | C | 2.03 | 0.515431906 |  |
| 286 | 12 | G | G | G | 90.54 | 0.000000000 | \* |
| 286 | 12 | G | G | T | 2.03 | 0.503906386 |  |
| 287 | 13 | A | A | A | 97.82 | 0.000000000 | \* |
| 287 | 13 | A | A | C | 0.93 | 0.826524116 |  |
| 287 | 13 | A | A | G | 0.62 | 0.855168151 |  |
| 287 | 13 | A | A | T | 0.62 | 0.829540365 |  |
| 288 | 14 | C | C | A | 5.03 | 0.201676810 |  |
| 288 | 14 | C | C | C | 84.92 | 0.000000000 | \* |
| 288 | 14 | C | C | G | 7.04 | 0.048745102 |  |
| 288 | 14 | C | C | T | 3.02 | 0.291392253 |  |
| 289 | 15 | C | C | A | 6.90 | 0.070625300 |  |
| 289 | 15 | C | C | C | 88.28 | 0.000000000 | \* |
| 289 | 15 | C | C | G | 2.07 | 0.612982496 |  |
| 289 | 15 | C | C | T | 2.76 | 0.339425001 |  |
| 290 | 16 | G | G | A | 5.38 | 0.167561950 |  |
| 290 | 16 | G | G | C | 2.69 | 0.309371049 |  |
| 290 | 16 | G | G | G | 89.25 | 0.000000000 | \* |
| 290 | 16 | G | G | T | 2.69 | 0.353549233 |  |
| 291 | 17 | T | T | A | 0.00 | 0.863636364 |  |
| 291 | 17 | T | T | C | 0.72 | 0.852440002 |  |
| 291 | 17 | T | T | G | 3.62 | 0.322158243 |  |
| 291 | 17 | T | T | T | 95.65 | 0.000000000 | \* |
| 292 | 18 | G | G | A | 4.30 | 0.288798704 |  |
| 292 | 18 | G | G | C | 0.33 | 0.869776161 |  |
| 292 | 18 | G | G | G | 94.04 | 0.000000000 | \* |
| 292 | 18 | G | G | T | 1.32 | 0.683097997 |  |
| 293 | 19 | T | T | A | 0.00 | 0.863636364 |  |
| 293 | 19 | T | T | C | 1.78 | 0.601363116 |  |
| 293 | 19 | T | T | G | 4.73 | 0.183511906 |  |
| 293 | 19 | T | T | T | 93.49 | 0.000000000 | \* |
| 294 | 20 | T | T | A | 0.00 | 0.863636364 |  |
| 294 | 20 | T | T | C | 1.69 | 0.628285129 |  |
| 294 | 20 | T | T | G | 5.08 | 0.151714336 |  |
| 294 | 20 | T | T | T | 93.22 | 0.000000000 | \* |
| 295 | 21 | T | T | A | 0.00 | 0.863636364 |  |
| 295 | 21 | T | T | C | 1.14 | 0.786912403 |  |
| 295 | 21 | T | T | G | 2.29 | 0.568096080 |  |
| 295 | 21 | T | T | T | 96.57 | 0.000000000 | \* |
| 296 | 22 | G | G | A | 2.97 | 0.511113972 |  |
| 296 | 22 | G | G | C | 1.27 | 0.756020496 |  |
| 296 | 22 | G | G | G | 94.92 | 0.000000000 | \* |
| 296 | 22 | G | G | T | 0.85 | 0.791136679 |  |
| 297 | 23 | T | T | A | 0.00 | 0.863636364 |  |
| 297 | 23 | T | T | C | 1.38 | 0.726668149 |  |
| 297 | 23 | T | T | G | 1.38 | 0.749926972 |  |
| 297 | 23 | T | T | T | 97.24 | 0.000000000 | \* |
| 298 | 24 | G | G | A | 3.17 | 0.472295315 |  |
| 298 | 24 | G | G | C | 1.06 | 0.804658810 |  |
| 298 | 24 | G | G | G | 94.18 | 0.000000000 | \* |
| 298 | 24 | G | G | T | 1.59 | 0.616095821 |  |
| 299 | 25 | C | C | A | 1.32 | 0.796965662 |  |
| 299 | 25 | C | C | C | 91.39 | 0.000000000 | \* |
| 299 | 25 | C | C | G | 3.31 | 0.372432740 |  |
| 299 | 25 | C | C | T | 3.97 | 0.156987629 |  |
| 300 | 26 | A | A | A | 92.98 | 0.000000000 | \* |
| 300 | 26 | A | A | C | 3.51 | 0.138963460 |  |
| 300 | 26 | A | A | G | 0.00 | 0.877777778 |  |
| 300 | 26 | A | A | T | 3.51 | 0.213708142 |  |
| 301 | 27 | C | C | A | 4.73 | 0.234044747 |  |
| 301 | 27 | C | C | C | 91.12 | 0.000000000 | \* |
| 301 | 27 | C | C | G | 1.18 | 0.783748359 |  |
| 301 | 27 | C | C | T | 2.96 | 0.301515744 |  |
| 302 | 28 | T | T | A | 1.25 | 0.805897538 |  |
| 302 | 28 | T | T | C | 5.62 | 0.010351956 |  |
| 302 | 28 | T | T | G | 3.12 | 0.404785235 |  |
| 302 | 28 | T | T | T | 90.00 | 0.000000000 | \* |
| 303 | 29 | A | A | A | 94.97 | 0.000000000 | \* |
| 303 | 29 | A | A | C | 1.26 | 0.759437715 |  |
| 303 | 29 | A | A | G | 1.89 | 0.650613389 |  |
| 303 | 29 | A | A | T | 1.89 | 0.539124497 |  |
| 304 | 30 | C | C | A | 2.72 | 0.557725076 |  |
| 304 | 30 | C | C | C | 93.20 | 0.000000000 | \* |
| 304 | 30 | C | C | G | 2.72 | 0.480347902 |  |
| 304 | 30 | C | C | T | 1.36 | 0.674065765 |  |
| 305 | 31 | A | A | A | 92.52 | 0.000000000 | \* |
| 305 | 31 | A | A | C | 1.87 | 0.569379949 |  |
| 305 | 31 | A | A | G | 5.61 | 0.113256334 |  |
| 305 | 31 | A | A | T | 0.00 | 0.870967742 |  |
| 306 | 32 | C | C | A | 0.74 | 0.849688938 |  |
| 306 | 32 | C | C | C | 93.38 | 0.000000000 | \* |
| 306 | 32 | C | C | G | 2.94 | 0.438315634 |  |
| 306 | 32 | C | C | T | 2.94 | 0.304686402 |  |
| 307 | 33 | G | G | A | 3.48 | 0.417976865 |  |
| 307 | 33 | G | G | C | 4.35 | 0.053408125 |  |
| 307 | 33 | G | G | G | 92.17 | 0.000000000 | \* |
| 307 | 33 | G | G | T | 0.00 | 0.870967742 |  |
| 308 | 34 | G | G | A | 0.00 | 0.863636364 |  |
| 308 | 34 | G | G | C | 2.73 | 0.297607225 |  |
| 308 | 34 | G | G | G | 95.63 | 0.000000000 | \* |
| 308 | 34 | G | G | T | 1.64 | 0.602657070 |  |
| 309 | 35 | G | G | A | 2.76 | 0.550542486 |  |
| 309 | 35 | G | G | C | 1.38 | 0.726668149 |  |
| 309 | 35 | G | G | G | 95.86 | 0.000000000 | \* |
| 309 | 35 | G | G | T | 0.00 | 0.870967742 |  |
| 310 | 36 | C | C | A | 4.51 | 0.261358461 |  |
| 310 | 36 | C | C | C | 91.73 | 0.000000000 | \* |
| 310 | 36 | C | C | G | 2.26 | 0.574304422 |  |
| 310 | 36 | C | C | T | 1.50 | 0.637612522 |  |
| 311 | 37 | A | A | A | 94.57 | 0.000000000 | \* |
| 311 | 37 | A | A | C | 2.33 | 0.416550375 |  |
| 311 | 37 | A | A | G | 0.78 | 0.840259864 |  |
| 311 | 37 | A | A | T | 2.33 | 0.432313880 |  |
| 312 | 38 | C | C | A | 4.37 | 0.279689289 |  |
| 312 | 38 | C | C | C | 91.80 | 0.000000000 | \* |
| 312 | 38 | C | C | G | 0.55 | 0.861233412 |  |
| 312 | 38 | C | C | T | 3.28 | 0.247543929 |  |
| 313 | 39 | C | C | A | 5.85 | 0.129454328 |  |
| 313 | 39 | C | C | C | 88.89 | 0.000000000 | \* |
| 313 | 39 | C | C | G | 2.92 | 0.441529637 |  |
| 313 | 39 | C | C | T | 2.34 | 0.429185743 |  |
| 314 | 40 | C | C | A | 6.51 | 0.088776515 |  |
| 314 | 40 | C | C | C | 88.76 | 0.000000000 | \* |
| 314 | 40 | C | C | G | 0.59 | 0.857768503 |  |
| 314 | 40 | C | C | T | 4.14 | 0.139898505 |  |
| 315 | 41 | T | T | A | 2.82 | 0.539412103 |  |
| 315 | 41 | T | T | C | 1.41 | 0.718300155 |  |
| 315 | 41 | T | T | G | 2.11 | 0.603917139 |  |
| 315 | 41 | T | T | T | 93.66 | 0.000000000 | \* |
| 316 | 42 | G | G | A | 1.95 | 0.701604103 |  |
| 316 | 42 | G | G | C | 1.46 | 0.702046128 |  |
| 316 | 42 | G | G | G | 95.12 | 0.000000000 | \* |
| 316 | 42 | G | G | T | 1.46 | 0.647953129 |  |
| 317 | 43 | A | A | A | 90.72 | 0.000000000 | \* |
| 317 | 43 | A | A | C | 1.03 | 0.809902769 |  |
| 317 | 43 | A | A | G | 1.55 | 0.718667242 |  |
| 317 | 43 | A | A | T | 6.70 | 0.020746261 |  |
| 318 | 44 | C | C | A | 3.38 | 0.435505468 |  |
| 318 | 44 | C | C | C | 92.57 | 0.000000000 | \* |
| 318 | 44 | C | C | G | 4.05 | 0.261019698 |  |
| 318 | 44 | C | C | T | 0.00 | 0.870967742 |  |
| 319 | 45 | C | C | A | 6.06 | 0.114873974 |  |
| 319 | 45 | C | C | C | 87.12 | 0.000000000 | \* |
| 319 | 45 | C | C | G | 4.55 | 0.202780195 |  |
| 319 | 45 | C | C | T | 2.27 | 0.444589686 |  |
| 320 | 46 | G | G | A | 3.17 | 0.472295315 |  |
| 320 | 46 | G | G | C | 3.97 | 0.083513366 |  |
| 320 | 46 | G | G | G | 90.48 | 0.000000000 | \* |
| 320 | 46 | G | G | T | 2.38 | 0.419658126 |  |
| 321 | 47 | A | A | A | 97.77 | 0.000000000 | \* |
| 321 | 47 | A | A | C | 0.89 | 0.832790838 |  |
| 321 | 47 | A | A | G | 1.34 | 0.757113855 |  |
| 321 | 47 | A | A | T | 0.00 | 0.870967742 |  |
| 322 | 48 | C | C | A | 3.05 | 0.494732086 |  |
| 322 | 48 | C | C | C | 93.13 | 0.000000000 | \* |
| 322 | 48 | C | C | G | 0.76 | 0.841551804 |  |
| 322 | 48 | C | C | T | 3.05 | 0.284665720 |  |
| 323 | 49 | G | G | A | 7.95 | 0.036936924 |  |
| 323 | 49 | G | G | C | 2.27 | 0.433522316 |  |
| 323 | 49 | G | G | G | 88.64 | 0.000000000 | \* |
| 323 | 49 | G | G | T | 1.14 | 0.728762586 |  |
| 324 | 50 | G | G | A | 2.12 | 0.672087356 |  |
| 324 | 50 | G | G | C | 0.53 | 0.865070874 |  |
| 324 | 50 | G | G | G | 96.30 | 0.000000000 | \* |
| 324 | 50 | G | G | T | 1.06 | 0.746751549 |  |
| 325 | 51 | C | C | A | 2.86 | 0.531748114 |  |
| 325 | 51 | C | C | C | 86.67 | 0.000000000 | \* |
| 325 | 51 | C | C | G | 3.81 | 0.294532975 |  |
| 325 | 51 | C | C | T | 6.67 | 0.021316697 |  |
| 326 | 52 | A | A | A | 98.36 | 0.000000000 | \* |
| 326 | 52 | A | A | C | 1.64 | 0.646614422 |  |
| 326 | 52 | A | A | G | 0.00 | 0.877777778 |  |
| 326 | 52 | A | A | T | 0.00 | 0.870967742 |  |
| 327 | 53 | A | A | A | 96.99 | 0.000000000 | \* |
| 327 | 53 | A | A | C | 0.00 | 0.870588235 |  |
| 327 | 53 | A | A | G | 0.75 | 0.842784080 |  |
| 327 | 53 | A | A | T | 2.26 | 0.448598079 |  |
| 328 | 54 | G | G | A | 4.21 | 0.301958757 |  |
| 328 | 54 | G | G | C | 2.11 | 0.488929161 |  |
| 328 | 54 | G | G | G | 89.47 | 0.000000000 | \* |
| 328 | 54 | G | G | T | 4.21 | 0.133430378 |  |
| 329 | 55 | A | A | A | 98.05 | 0.000000000 | \* |
| 329 | 55 | A | A | C | 0.00 | 0.870588235 |  |
| 329 | 55 | A | A | G | 0.65 | 0.852859821 |  |
| 329 | 55 | A | A | T | 1.30 | 0.689517877 |  |
| 330 | 56 | A | A | A | 95.31 | 0.000000000 | \* |
| 330 | 56 | A | A | C | 0.00 | 0.870588235 |  |
| 330 | 56 | A | A | G | 1.56 | 0.715560061 |  |
| 330 | 56 | A | A | T | 3.12 | 0.272433417 |  |
| 331 | 57 | G | G | A | 4.65 | 0.243915381 |  |
| 331 | 57 | G | G | C | 1.16 | 0.782419695 |  |
| 331 | 57 | G | G | G | 88.37 | 0.000000000 | \* |
| 331 | 57 | G | G | T | 5.81 | 0.041329657 |  |
| 332 | 58 | T | T | A | 0.00 | 0.863636364 |  |
| 332 | 58 | T | T | C | 2.10 | 0.491410040 |  |
| 332 | 58 | T | T | G | 0.70 | 0.848162603 |  |
| 332 | 58 | T | T | T | 97.20 | 0.000000000 | \* |
| 333 | 59 | T | T | A | 8.97 | 0.019342497 |  |
| 333 | 59 | T | T | C | 1.38 | 0.726668149 |  |
| 333 | 59 | T | T | G | 5.52 | 0.119203338 |  |
| 333 | 59 | T | T | T | 84.14 | 0.000000000 | \* |
| 334 | 60 | C | C | A | 5.43 | 0.163117698 |  |
| 334 | 60 | C | C | C | 89.15 | 0.000000000 | \* |
| 334 | 60 | C | C | G | 3.10 | 0.409115593 |  |
| 334 | 60 | C | C | T | 2.33 | 0.432313880 |  |
| 335 | 61 | G | G | A | 7.77 | 0.041531223 |  |
| 335 | 61 | G | G | C | 4.85 | 0.028499523 |  |
| 335 | 61 | G | G | G | 82.52 | 0.000000000 | \* |
| 335 | 61 | G | G | T | 4.85 | 0.084488718 |  |
| 336 | 62 | A | A | A | 97.84 | 0.000000000 | \* |
| 336 | 62 | A | A | C | 1.62 | 0.652391602 |  |
| 336 | 62 | A | A | G | 0.54 | 0.861660029 |  |
| 336 | 62 | A | A | T | 0.00 | 0.870967742 |  |
| 337 | 63 | C | C | A | 4.67 | 0.242037963 |  |
| 337 | 63 | C | C | C | 91.33 | 0.000000000 | \* |
| 337 | 63 | C | C | G | 1.33 | 0.758171468 |  |
| 337 | 63 | C | C | T | 2.67 | 0.357939694 |  |
| 338 | 64 | A | A | A | 94.19 | 0.000000000 | \* |
| 338 | 64 | A | A | C | 2.33 | 0.416550375 |  |
| 338 | 64 | A | A | G | 2.33 | 0.559884755 |  |
| 338 | 64 | A | A | T | 1.16 | 0.722531663 |  |
| 339 | 65 | G | G | A | 4.20 | 0.304054909 |  |
| 339 | 65 | G | G | C | 2.80 | 0.280806058 |  |
| 339 | 65 | G | G | G | 93.01 | 0.000000000 | \* |
| 339 | 65 | G | G | T | 0.00 | 0.870967742 |  |
| 340 | 66 | C | C | A | 3.10 | 0.485922401 |  |
| 340 | 66 | C | C | C | 94.57 | 0.000000000 | \* |
| 340 | 66 | C | C | G | 0.78 | 0.840259864 |  |
| 340 | 66 | C | C | T | 1.55 | 0.625616074 |  |
| 341 | 67 | T | T | A | 1.94 | 0.704346481 |  |
| 341 | 67 | T | T | C | 3.23 | 0.186350786 |  |
| 341 | 67 | T | T | G | 2.58 | 0.508095991 |  |
| 341 | 67 | T | T | T | 92.26 | 0.000000000 | \* |
| 342 | 68 | C | C | A | 2.96 | 0.511705570 |  |
| 342 | 68 | C | C | C | 94.07 | 0.000000000 | \* |
| 342 | 68 | C | C | G | 2.96 | 0.434262822 |  |
| 342 | 68 | C | C | T | 0.00 | 0.870967742 |  |
| 343 | 69 | C | C | A | 2.94 | 0.515816909 |  |
| 343 | 69 | C | C | C | 91.76 | 0.000000000 | \* |
| 343 | 69 | C | C | G | 0.00 | 0.877777778 |  |
| 343 | 69 | C | C | T | 5.29 | 0.061155883 |  |
| 344 | 70 | C | C | A | 0.00 | 0.863636364 |  |
| 344 | 70 | C | C | C | 91.40 | 0.000000000 | \* |
| 344 | 70 | C | C | G | 3.23 | 0.387065457 |  |
| 344 | 70 | C | C | T | 5.38 | 0.057518943 |  |
| 345 | 71 | G | G | A | 9.57 | 0.013038757 |  |
| 345 | 71 | G | G | C | 0.87 | 0.836044541 |  |
| 345 | 71 | G | G | G | 86.96 | 0.000000000 | \* |
| 345 | 71 | G | G | T | 2.61 | 0.369956775 |  |
| 346 | 72 | C | C | A | 0.00 | 0.863636364 |  |
| 346 | 72 | C | C | C | 93.22 | 0.000000000 | \* |
| 346 | 72 | C | C | G | 0.85 | 0.831908513 |  |
| 346 | 72 | C | C | T | 5.93 | 0.037754865 |  |
| 347 | 73 | G | G | A | 9.52 | 0.013401781 |  |
| 347 | 73 | G | G | C | 1.90 | 0.557206098 |  |
| 347 | 73 | G | G | G | 84.76 | 0.000000000 | \* |
| 347 | 73 | G | G | T | 3.81 | 0.175326474 |  |
| 348 | 74 | A | A | A | 96.91 | 0.000000000 | \* |
| 348 | 74 | A | A | C | 2.47 | 0.372039988 |  |
| 348 | 74 | A | A | G | 0.62 | 0.855659184 |  |
| 348 | 74 | A | A | T | 0.00 | 0.870967742 |  |
| 349 | 75 | C | C | A | 3.33 | 0.443524366 |  |
| 349 | 75 | C | C | C | 81.33 | 0.000000000 | \* |
| 349 | 75 | C | C | G | 11.33 | 0.002996402 | \* |
| 349 | 75 | C | C | T | 4.00 | 0.154186318 |  |

## For use in R

If you want to work with the results in R, here is output that you can copy and paste in your terminal to get:

The base information:

```
structure(list(focal.base = c("A", "C", "G", "T"), avg.percsignal = c(94.1853334856248, 
90.1418514592903, 91.9103338312984, 93.3900909423437), avg.areasignal = c(165.029411764706, 
170.19696969697, 169.754385964912, 190.954545454545), crit.perc.area = c(9.96284194522295, 
5.65054239287557, 9.52949631184053, 7.61197945174494), mu = c(3.77241223509981, 
2.4357713550673, 3.33713858750085, 2.63658790330202), fillibens = c(0.991378800221168, 
0.996684196158305, 0.993608337189313, 0.994857580588095)), .Names = c("focal.base", 
"avg.percsignal", "avg.areasignal", "crit.perc.area", "mu", "fillibens"
), row.names = c(NA, -4L), class = "data.frame")
```

the data.frame that contains information on the guide region:

```
structure(list(A.area = c(135, 161, 6, 320, 13, 0, 15, 11, 10, 
8, 131, 8, 314, 10, 10, 10, 0, 13, 0, 0, 0, 7, 0, 6, 2, 106, 
8, 2, 151, 4, 99, 1, 4, 0, 4, 6, 122, 8, 10, 11, 4, 4, 176, 5, 
8, 4, 219, 4, 7, 4, 3, 60, 129, 4, 151, 122, 4, 0, 13, 7, 8, 
181, 7, 81, 6, 4, 3, 4, 5, 0, 11, 0, 10, 157, 5), C.area = c(3, 
3, 7, 4, 157, 4, 5, 4, 119, 175, 3, 3, 3, 169, 128, 5, 1, 1, 
3, 3, 2, 3, 2, 2, 138, 4, 154, 9, 2, 137, 2, 127, 5, 5, 2, 122, 
3, 168, 152, 150, 2, 3, 2, 137, 115, 5, 2, 122, 2, 1, 91, 1, 
0, 2, 0, 0, 1, 3, 2, 115, 5, 3, 137, 2, 4, 122, 5, 127, 156, 
85, 1, 110, 2, 4, 122), G.area = c(3, 2, 119, 5, 11, 5, 326, 
205, 7, 6, 4, 134, 2, 14, 3, 166, 5, 284, 8, 9, 4, 224, 2, 178, 
5, 0, 2, 5, 3, 4, 6, 4, 106, 175, 139, 3, 1, 1, 5, 1, 3, 195, 
3, 6, 6, 114, 3, 1, 78, 182, 4, 0, 1, 85, 1, 2, 76, 1, 8, 4, 
85, 1, 2, 2, 133, 1, 4, 4, 0, 3, 100, 1, 89, 1, 17), T.area = c(1, 
1, 1, 0, 9, 169, 3, 2, 5, 5, 0, 3, 2, 6, 4, 5, 132, 4, 158, 165, 
169, 2, 141, 3, 6, 4, 5, 144, 3, 2, 0, 4, 0, 3, 0, 2, 3, 6, 4, 
7, 133, 3, 13, 0, 3, 3, 0, 4, 1, 2, 7, 0, 3, 4, 2, 4, 5, 139, 
122, 3, 5, 0, 4, 1, 0, 2, 143, 0, 9, 5, 3, 7, 4, 0, 6), Tot.area = c(142, 
167, 133, 329, 190, 178, 349, 222, 141, 194, 138, 148, 321, 199, 
145, 186, 138, 302, 169, 177, 175, 236, 145, 189, 151, 114, 169, 
160, 159, 147, 107, 136, 115, 183, 145, 133, 129, 183, 171, 169, 
142, 205, 194, 148, 132, 126, 224, 131, 88, 189, 105, 61, 133, 
95, 154, 128, 86, 143, 145, 129, 103, 185, 150, 86, 143, 129, 
155, 135, 170, 93, 115, 118, 105, 162, 150), A.perc = c(95.0704225352113, 
96.4071856287425, 4.51127819548872, 97.2644376899696, 6.84210526315789, 
0, 4.29799426934097, 4.95495495495495, 7.09219858156028, 4.12371134020619, 
94.9275362318841, 5.40540540540541, 97.8193146417446, 5.0251256281407, 
6.89655172413793, 5.37634408602151, 0, 4.3046357615894, 0, 0, 
0, 2.96610169491525, 0, 3.17460317460317, 1.32450331125828, 92.9824561403509, 
4.73372781065089, 1.25, 94.9685534591195, 2.72108843537415, 92.5233644859813, 
0.735294117647059, 3.47826086956522, 0, 2.75862068965517, 4.51127819548872, 
94.5736434108527, 4.37158469945355, 5.84795321637427, 6.50887573964497, 
2.8169014084507, 1.95121951219512, 90.7216494845361, 3.37837837837838, 
6.06060606060606, 3.17460317460317, 97.7678571428571, 3.05343511450382, 
7.95454545454545, 2.11640211640212, 2.85714285714286, 98.3606557377049, 
96.9924812030075, 4.21052631578947, 98.051948051948, 95.3125, 
4.65116279069767, 0, 8.96551724137931, 5.42635658914729, 7.76699029126214, 
97.8378378378378, 4.66666666666667, 94.1860465116279, 4.1958041958042, 
3.10077519379845, 1.93548387096774, 2.96296296296296, 2.94117647058824, 
0, 9.56521739130435, 0, 9.52380952380952, 96.9135802469136, 3.33333333333333
), C.perc = c(2.11267605633803, 1.79640718562874, 5.26315789473684, 
1.21580547112462, 82.6315789473684, 2.24719101123596, 1.43266475644699, 
1.8018018018018, 84.3971631205674, 90.2061855670103, 2.17391304347826, 
2.02702702702703, 0.934579439252336, 84.9246231155779, 88.2758620689655, 
2.68817204301075, 0.72463768115942, 0.33112582781457, 1.77514792899408, 
1.69491525423729, 1.14285714285714, 1.27118644067797, 1.37931034482759, 
1.05820105820106, 91.3907284768212, 3.50877192982456, 91.1242603550296, 
5.625, 1.25786163522013, 93.1972789115646, 1.86915887850467, 
93.3823529411765, 4.34782608695652, 2.73224043715847, 1.37931034482759, 
91.7293233082707, 2.32558139534884, 91.8032786885246, 88.8888888888889, 
88.7573964497041, 1.40845070422535, 1.46341463414634, 1.03092783505155, 
92.5675675675676, 87.1212121212121, 3.96825396825397, 0.892857142857143, 
93.1297709923664, 2.27272727272727, 0.529100529100529, 86.6666666666667, 
1.63934426229508, 0, 2.10526315789474, 0, 0, 1.16279069767442, 
2.0979020979021, 1.37931034482759, 89.1472868217054, 4.85436893203883, 
1.62162162162162, 91.3333333333333, 2.32558139534884, 2.7972027972028, 
94.5736434108527, 3.2258064516129, 94.0740740740741, 91.7647058823529, 
91.3978494623656, 0.869565217391304, 93.2203389830509, 1.9047619047619, 
2.46913580246914, 81.3333333333333), G.perc = c(2.11267605633803, 
1.19760479041916, 89.4736842105263, 1.51975683890578, 5.78947368421053, 
2.80898876404494, 93.4097421203438, 92.3423423423423, 4.9645390070922, 
3.09278350515464, 2.89855072463768, 90.5405405405405, 0.623052959501558, 
7.03517587939699, 2.06896551724138, 89.247311827957, 3.6231884057971, 
94.0397350993377, 4.73372781065089, 5.08474576271186, 2.28571428571429, 
94.9152542372881, 1.37931034482759, 94.1798941798942, 3.3112582781457, 
0, 1.18343195266272, 3.125, 1.88679245283019, 2.72108843537415, 
5.60747663551402, 2.94117647058824, 92.1739130434783, 95.6284153005465, 
95.8620689655172, 2.25563909774436, 0.775193798449612, 0.546448087431694, 
2.92397660818713, 0.591715976331361, 2.11267605633803, 95.1219512195122, 
1.54639175257732, 4.05405405405405, 4.54545454545455, 90.4761904761905, 
1.33928571428571, 0.763358778625954, 88.6363636363636, 96.2962962962963, 
3.80952380952381, 0, 0.75187969924812, 89.4736842105263, 0.649350649350649, 
1.5625, 88.3720930232558, 0.699300699300699, 5.51724137931035, 
3.10077519379845, 82.5242718446602, 0.540540540540541, 1.33333333333333, 
2.32558139534884, 93.006993006993, 0.775193798449612, 2.58064516129032, 
2.96296296296296, 0, 3.2258064516129, 86.9565217391304, 0.847457627118644, 
84.7619047619048, 0.617283950617284, 11.3333333333333), T.perc = c(0.704225352112676, 
0.598802395209581, 0.75187969924812, 0, 4.73684210526316, 94.9438202247191, 
0.859598853868195, 0.900900900900901, 3.54609929078014, 2.57731958762887, 
0, 2.02702702702703, 0.623052959501558, 3.01507537688442, 2.75862068965517, 
2.68817204301075, 95.6521739130435, 1.32450331125828, 93.491124260355, 
93.2203389830509, 96.5714285714286, 0.847457627118644, 97.2413793103448, 
1.58730158730159, 3.97350993377483, 3.50877192982456, 2.9585798816568, 
90, 1.88679245283019, 1.36054421768707, 0, 2.94117647058824, 
0, 1.63934426229508, 0, 1.50375939849624, 2.32558139534884, 3.27868852459016, 
2.33918128654971, 4.14201183431953, 93.6619718309859, 1.46341463414634, 
6.70103092783505, 0, 2.27272727272727, 2.38095238095238, 0, 3.05343511450382, 
1.13636363636364, 1.05820105820106, 6.66666666666667, 0, 2.25563909774436, 
4.21052631578947, 1.2987012987013, 3.125, 5.81395348837209, 97.2027972027972, 
84.1379310344828, 2.32558139534884, 4.85436893203883, 0, 2.66666666666667, 
1.16279069767442, 0, 1.55038759689922, 92.258064516129, 0, 5.29411764705882, 
5.37634408602151, 2.60869565217391, 5.93220338983051, 3.80952380952381, 
0, 4), base.call = c("A", "A", "G", "A", "C", "T", "G", "G", 
"C", "C", "A", "G", "A", "C", "C", "G", "T", "G", "T", "T", "T", 
"G", "T", "G", "C", "A", "C", "T", "A", "C", "A", "C", "G", "G", 
"G", "C", "A", "C", "C", "C", "T", "G", "A", "C", "C", "G", "A", 
"C", "G", "G", "C", "A", "A", "G", "A", "A", "G", "T", "T", "C", 
"G", "A", "C", "A", "G", "C", "T", "C", "C", "C", "G", "C", "G", 
"A", "C"), index = 275:349, guide.seq = c("A", "A", "G", "A", 
"C", "T", "G", "G", "C", "C", "A", "G", "A", "C", "C", "G", "T", 
"G", "T", "T", "T", "G", "T", "G", "C", "A", "C", "T", "A", "C", 
"A", "C", "G", "G", "G", "C", "A", "C", "C", "C", "T", "G", "A", 
"C", "C", "G", "A", "C", "G", "G", "C", "A", "A", "G", "A", "A", 
"G", "T", "T", "C", "G", "A", "C", "A", "G", "C", "T", "C", "C", 
"C", "G", "C", "G", "A", "C"), T.pval = c(0.81688730090678, 0.833021609790912, 
0.808779633560913, 0.870967741935475, 0.0919788454285563, 0, 
0.788772600242738, 0.780537995960102, 0.208593722379096, 0.376570568131012, 
0.870967741935475, 0.503906385525944, 0.829540364758162, 0.291392252522332, 
0.339425001055522, 0.353549233333127, 0, 0.683097997175956, 0, 
0, 0, 0.791136679116765, 0, 0.616095820723714, 0.156987628777856, 
0.213708141907064, 0.301515743759315, 0, 0.539124496775184, 0.674065764992308, 
0.870967741935475, 0.304686402471401, 0.870967741935475, 0.602657069660226, 
0.870967741935475, 0.637612522098636, 0.432313879589334, 0.247543929257067, 
0.429185742800895, 0.139898504657706, 0, 0.647953129373324, 0.0207462611544884, 
0.870967741935475, 0.444589686209276, 0.419658125951008, 0.870967741935475, 
0.284665720357087, 0.728762586404015, 0.746751549387912, 0.02131669738796, 
0.870967741935475, 0.448598079117775, 0.133430377948488, 0.689517876831065, 
0.272433417311031, 0.0413296567320873, 0, 0, 0.432313879589334, 
0.0844887179785314, 0.870967741935475, 0.357939694102861, 0.722531662540482, 
0.870967741935475, 0.625616073636873, 0, 0.870967741935475, 0.0611558827380574, 
0.0575189429847416, 0.369956775097854, 0.0377548647743592, 0.175326473628904, 
0.870967741935475, 0.154186317589382), C.pval = c(0.486433920901352, 
0.594161679344061, 0.0167842155643758, 0.769913362784016, 0, 
0.441821267403997, 0.711212637004839, 0.592330775825098, 0, 0, 
0.465954762228564, 0.515431906300281, 0.826524116218999, 0, 0, 
0.309371049029996, 0.852440001806342, 0.869776160626071, 0.601363116234418, 
0.62828512940459, 0.786912403324145, 0.756020495514554, 0.726668149477946, 
0.804658809947411, 0, 0.138963459843171, 0, 0.0103519555975722, 
0.759437714919842, 0, 0.569379949150497, 0, 0.0534081247313349, 
0.297607224702845, 0.726668149477946, 0, 0.416550375210954, 0, 
0, 0, 0.718300155028401, 0.702046128130758, 0.809902769450548, 
0, 0, 0.0835133662676737, 0.832790838332334, 0, 0.433522315934789, 
0.865070873853188, 0, 0.646614422378374, 0.870588235294109, 0.488929160880059, 
0.870588235294109, 0.870588235294109, 0.78241969546987, 0.491410040195803, 
0.726668149477946, 0, 0.0284995231654079, 0.652391601910071, 
0, 0.416550375210954, 0.280806057991165, 0, 0.186350785701401, 
0, 0, 0, 0.836044540809543, 0, 0.557206098200899, 0.372039988108835, 
0), G.pval = c(0.603917138904883, 0.781423242438955, 0, 0.723772203811343, 
0.102062743998017, 0.463332053285954, 0, 0, 0.162023521182076, 
0.410550188962174, 0.446303748898086, 0, 0.855168151338075, 0.0487451021652801, 
0.612982495586515, 0, 0.322158243243385, 0, 0.183511905785307, 
0.151714335968916, 0.568096079842308, 0, 0.749926971796711, 0, 
0.372432740306558, 0.877777777777744, 0.783748358748371, 0.404785235426056, 
0.650613388824303, 0.480347901849575, 0.113256333797251, 0.438315633912711, 
0, 0, 0, 0.574304422087998, 0.840259864388267, 0.861233411619704, 
0.441529637020813, 0.857768503420922, 0.603917138904883, 0, 0.718667242374333, 
0.261019698152445, 0.202780195247419, 0, 0.757113855079635, 0.841551804420289, 
0, 0, 0.294532974971256, 0.877777777777744, 0.842784079784555, 
0, 0.852859821164286, 0.715560060619839, 0, 0.848162603008938, 
0.119203338493923, 0.40911559259079, 0, 0.861660029471336, 0.75817146811402, 
0.559884754849704, 0, 0.840259864388267, 0.508095991250175, 0.434262822240796, 
0.877777777777744, 0.387065456785736, 0, 0.831908513404465, 0, 
0.855659184148325, 0.00299640181777605), A.pval = c(0, 0, 0.261358461288475, 
0, 0.0729533827956783, 0.863636363636362, 0.289713837503268, 
0.209125005977279, 0.0628061257295766, 0.31446524243565, 0, 0.164967333659672, 
0, 0.201676809567026, 0.0706252999561264, 0.167561949890731, 
0.863636363636362, 0.288798704223157, 0.863636363636362, 0.863636363636362, 
0.863636363636362, 0.511113972356513, 0.863636363636362, 0.472295314674943, 
0.796965661971802, 0, 0.234044747116619, 0.805897538331606, 0, 
0.557725075641912, 0, 0.84968893774748, 0.417976864797866, 0.863636363636362, 
0.550542485569314, 0.261358461288475, 0, 0.27968928892741, 0.129454327831527, 
0.0887765150795629, 0.539412103062135, 0.70160410319134, 0, 0.435505468333524, 
0.114873974112251, 0.472295314674943, 0, 0.494732085748365, 0.0369369241485376, 
0.672087356062476, 0.53174811444432, 0, 0, 0.301958756802997, 
0, 0, 0.243915381490456, 0.863636363636362, 0.0193424968200561, 
0.16311769834035, 0.0415312231491799, 0, 0.242037963011332, 0, 
0.304054909011797, 0.485922400656081, 0.704346480793096, 0.511705570236145, 
0.515816908938821, 0.863636363636362, 0.013038756663033, 0.863636363636362, 
0.0134017805750543, 0, 0.44352436640354), guide.position = 1:75), .Names = c("A.area", 
"C.area", "G.area", "T.area", "Tot.area", "A.perc", "C.perc", 
"G.perc", "T.perc", "base.call", "index", "guide.seq", "T.pval", 
"C.pval", "G.pval", "A.pval", "guide.position"), row.names = 275:349, class = "data.frame")
```

*Report generated using EditR v1.0.8*
