# Supplementary material for: Mechanistic and genetic basis of single-strand templated repair at Cas12a-induced DNA breaks in Chlamydomonas reinhardtii
Source: Nat Commun. 2021 Nov 19;12:6751. doi: 10.1038/s41467-021-27004-1 (PMC8604939; doi:10.1038/s41467-021-27004-1)
Supplement: Supplementary file 22 — Source Data [file 41467_2021_27004_MOESM22_ESM.zip › Source Data/EditR analysis/EditR outputs/Sense/rep3_ssODN_sense_16.html]

EditR v1.0.8 report


# EditR v1.0.8 report

- Data QA
  - Filtering data
  - Percent noise peak area
  - Base information
- Predicted editing
  - Editing bar plot
  - Editing table plot
  - Table of editing results
- For use in R

## Data QA

### Filtering data

What the data looked like prefiltering:

and the post filtering signal / noise plot:

### Percent noise peak area

### Base information

Here’s information about the signal of each base, the critical percent value where any higher value would be called as significant, and Filliben’s correlation for how well the noise was modelled by the zero adjusted gamma distribution.

| Base | Average percent signal | Average peak area | Critical percent value | model mu | Fillibens correlation |
| --- | --- | --- | --- | --- | --- |
| A | 87.93344 | 95.05882 | 16.696388 | 5.121055 | 0.9927094 |
| C | 85.26810 | 99.46970 | 9.844447 | 3.539617 | 0.9952417 |
| G | 88.44052 | 90.22807 | 18.080093 | 5.176043 | 0.9902133 |
| T | 90.10664 | 108.61364 | 12.966364 | 4.161415 | 0.9883265 |

## Predicted editing

### Editing bar plot

### Editing table plot

### Table of editing results


Here’s the entire guide region

| Sanger position | Guide position | Guide sequence | Sanger base call | Focal base | Focal base peak area | p value |  |
| --- | --- | --- | --- | --- | --- | --- | --- |
| 275 | 1 | A | A | A | 87.50 | 9.847678e-14 | \* |
| 275 | 1 | A | A | C | 3.41 | 3.943093e-01 |  |
| 275 | 1 | A | A | G | 2.27 | 6.568491e-01 |  |
| 275 | 1 | A | A | T | 6.82 | 1.393889e-01 |  |
| 276 | 2 | A | A | A | 85.84 | 1.811884e-13 | \* |
| 276 | 2 | A | A | C | 7.08 | 5.655171e-02 |  |
| 276 | 2 | A | A | G | 1.77 | 7.184027e-01 |  |
| 276 | 2 | A | A | T | 5.31 | 2.478058e-01 |  |
| 277 | 3 | G | G | A | 4.00 | 4.462687e-01 |  |
| 277 | 3 | G | G | C | 12.00 | 2.378752e-03 | \* |
| 277 | 3 | G | G | G | 84.00 | 1.191081e-11 | \* |
| 277 | 3 | G | G | T | 0.00 | 9.032258e-01 |  |
| 278 | 4 | A | A | A | 94.65 | 7.105427e-15 | \* |
| 278 | 4 | A | A | C | 0.53 | 8.714884e-01 |  |
| 278 | 4 | A | A | G | 1.07 | 7.997830e-01 |  |
| 278 | 4 | A | A | T | 3.74 | 4.256771e-01 |  |
| 279 | 5 | C | C | A | 11.11 | 6.008433e-02 |  |
| 279 | 5 | C | C | C | 82.41 | 0.000000e+00 | \* |
| 279 | 5 | C | C | G | 0.00 | 8.777778e-01 |  |
| 279 | 5 | C | C | T | 6.48 | 1.590636e-01 |  |
| 280 | 6 | T | T | A | 6.45 | 2.373755e-01 |  |
| 280 | 6 | T | T | C | 1.08 | 8.219748e-01 |  |
| 280 | 6 | T | T | G | 2.15 | 6.718322e-01 |  |
| 280 | 6 | T | T | T | 90.32 | 0.000000e+00 | \* |
| 281 | 7 | G | G | A | 4.40 | 4.058129e-01 |  |
| 281 | 7 | G | G | C | 2.75 | 5.161125e-01 |  |
| 281 | 7 | G | G | G | 92.31 | 8.414380e-13 | \* |
| 281 | 7 | G | G | T | 0.55 | 8.837176e-01 |  |
| 282 | 8 | G | G | A | 5.98 | 2.696537e-01 |  |
| 282 | 8 | G | G | C | 2.56 | 5.521871e-01 |  |
| 282 | 8 | G | G | G | 90.60 | 1.452283e-12 | \* |
| 282 | 8 | G | G | T | 0.85 | 8.565076e-01 |  |
| 283 | 9 | C | C | A | 11.49 | 5.333536e-02 |  |
| 283 | 9 | C | C | C | 78.16 | 0.000000e+00 | \* |
| 283 | 9 | C | C | G | 4.60 | 4.003886e-01 |  |
| 283 | 9 | C | C | T | 5.75 | 2.106826e-01 |  |
| 284 | 10 | C | C | A | 5.65 | 2.950916e-01 |  |
| 284 | 10 | C | C | C | 79.03 | 0.000000e+00 | \* |
| 284 | 10 | C | C | G | 6.45 | 2.550525e-01 |  |
| 284 | 10 | C | C | T | 8.87 | 6.020750e-02 |  |
| 285 | 11 | A | A | A | 83.53 | 4.237721e-13 | \* |
| 285 | 11 | A | A | C | 1.18 | 8.083727e-01 |  |
| 285 | 11 | A | A | G | 10.59 | 8.515639e-02 |  |
| 285 | 11 | A | A | T | 4.71 | 3.077847e-01 |  |
| 286 | 12 | G | G | A | 7.78 | 1.633685e-01 |  |
| 286 | 12 | G | G | C | 1.11 | 8.172958e-01 |  |
| 286 | 12 | G | G | G | 91.11 | 1.233014e-12 | \* |
| 286 | 12 | G | G | T | 0.00 | 9.032258e-01 |  |
| 287 | 13 | A | A | A | 94.74 | 6.883383e-15 | \* |
| 287 | 13 | A | A | C | 1.05 | 8.248490e-01 |  |
| 287 | 13 | A | A | G | 0.53 | 8.515563e-01 |  |
| 287 | 13 | A | A | T | 3.68 | 4.338034e-01 |  |
| 288 | 14 | C | C | A | 8.47 | 1.333871e-01 |  |
| 288 | 14 | C | C | C | 82.20 | 0.000000e+00 | \* |
| 288 | 14 | C | C | G | 5.08 | 3.569249e-01 |  |
| 288 | 14 | C | C | T | 4.24 | 3.617250e-01 |  |
| 289 | 15 | C | C | A | 11.36 | 5.555083e-02 |  |
| 289 | 15 | C | C | C | 85.23 | 0.000000e+00 | \* |
| 289 | 15 | C | C | G | 1.14 | 7.925004e-01 |  |
| 289 | 15 | C | C | T | 2.27 | 6.509686e-01 |  |
| 290 | 16 | G | G | A | 4.35 | 4.105661e-01 |  |
| 290 | 16 | G | G | C | 2.17 | 6.302528e-01 |  |
| 290 | 16 | G | G | G | 91.30 | 1.159184e-12 | \* |
| 290 | 16 | G | G | T | 2.17 | 6.670474e-01 |  |
| 291 | 17 | T | T | A | 8.33 | 1.390273e-01 |  |
| 291 | 17 | T | T | C | 0.00 | 8.823529e-01 |  |
| 291 | 17 | T | T | G | 2.38 | 6.436120e-01 |  |
| 291 | 17 | T | T | T | 89.29 | 0.000000e+00 | \* |
| 292 | 18 | G | G | A | 3.18 | 5.367307e-01 |  |
| 292 | 18 | G | G | C | 1.27 | 7.941898e-01 |  |
| 292 | 18 | G | G | G | 91.72 | 1.015188e-12 | \* |
| 292 | 18 | G | G | T | 3.82 | 4.150590e-01 |  |
| 293 | 19 | T | T | A | 0.00 | 8.363636e-01 |  |
| 293 | 19 | T | T | C | 3.33 | 4.073836e-01 |  |
| 293 | 19 | T | T | G | 4.44 | 4.148713e-01 |  |
| 293 | 19 | T | T | T | 92.22 | 0.000000e+00 | \* |
| 294 | 20 | T | T | A | 1.00 | 7.836839e-01 |  |
| 294 | 20 | T | T | C | 0.00 | 8.823529e-01 |  |
| 294 | 20 | T | T | G | 5.00 | 3.642083e-01 |  |
| 294 | 20 | T | T | T | 94.00 | 0.000000e+00 | \* |
| 295 | 21 | T | T | A | 0.00 | 8.363636e-01 |  |
| 295 | 21 | T | T | C | 4.12 | 2.842594e-01 |  |
| 295 | 21 | T | T | G | 2.06 | 6.827146e-01 |  |
| 295 | 21 | T | T | T | 93.81 | 0.000000e+00 | \* |
| 296 | 22 | G | G | A | 3.97 | 4.496209e-01 |  |
| 296 | 22 | G | G | C | 3.97 | 3.060884e-01 |  |
| 296 | 22 | G | G | G | 89.68 | 1.945111e-12 | \* |
| 296 | 22 | G | G | T | 2.38 | 6.333519e-01 |  |
| 297 | 23 | T | T | A | 2.38 | 6.319322e-01 |  |
| 297 | 23 | T | T | C | 2.38 | 5.887694e-01 |  |
| 297 | 23 | T | T | G | 4.76 | 3.853000e-01 |  |
| 297 | 23 | T | T | T | 90.48 | 0.000000e+00 | \* |
| 298 | 24 | G | G | A | 5.61 | 2.980462e-01 |  |
| 298 | 24 | G | G | C | 1.87 | 6.901977e-01 |  |
| 298 | 24 | G | G | G | 83.18 | 1.547873e-11 | \* |
| 298 | 24 | G | G | T | 9.35 | 4.923779e-02 |  |
| 299 | 25 | C | C | A | 4.40 | 4.058129e-01 |  |
| 299 | 25 | C | C | C | 83.52 | 0.000000e+00 | \* |
| 299 | 25 | C | C | G | 1.10 | 7.965998e-01 |  |
| 299 | 25 | C | C | T | 10.99 | 2.416639e-02 |  |
| 300 | 26 | A | A | A | 77.50 | 3.875344e-12 | \* |
| 300 | 26 | A | A | C | 2.50 | 5.649512e-01 |  |
| 300 | 26 | A | A | G | 10.00 | 1.000596e-01 |  |
| 300 | 26 | A | A | T | 10.00 | 3.719002e-02 |  |
| 301 | 27 | C | C | A | 13.33 | 2.983391e-02 |  |
| 301 | 27 | C | C | C | 76.19 | 0.000000e+00 | \* |
| 301 | 27 | C | C | G | 1.90 | 7.019633e-01 |  |
| 301 | 27 | C | C | T | 8.57 | 6.827099e-02 |  |
| 302 | 28 | T | T | A | 3.53 | 4.974454e-01 |  |
| 302 | 28 | T | T | C | 3.53 | 3.740745e-01 |  |
| 302 | 28 | T | T | G | 4.71 | 3.903980e-01 |  |
| 302 | 28 | T | T | T | 88.24 | 0.000000e+00 | \* |
| 303 | 29 | A | A | A | 88.78 | 6.161738e-14 | \* |
| 303 | 29 | A | A | C | 5.10 | 1.732027e-01 |  |
| 303 | 29 | A | A | G | 3.06 | 5.622459e-01 |  |
| 303 | 29 | A | A | T | 3.06 | 5.250356e-01 |  |
| 304 | 30 | C | C | A | 9.89 | 8.738812e-02 |  |
| 304 | 30 | C | C | C | 82.42 | 0.000000e+00 | \* |
| 304 | 30 | C | C | G | 2.20 | 6.660335e-01 |  |
| 304 | 30 | C | C | T | 5.49 | 2.315027e-01 |  |
| 305 | 31 | A | A | A | 85.71 | 1.898481e-13 | \* |
| 305 | 31 | A | A | C | 4.29 | 2.627714e-01 |  |
| 305 | 31 | A | A | G | 10.00 | 1.000596e-01 |  |
| 305 | 31 | A | A | T | 0.00 | 9.032258e-01 |  |
| 306 | 32 | C | C | A | 4.88 | 3.599397e-01 |  |
| 306 | 32 | C | C | C | 84.15 | 0.000000e+00 | \* |
| 306 | 32 | C | C | G | 2.44 | 6.365296e-01 |  |
| 306 | 32 | C | C | T | 8.54 | 6.927210e-02 |  |
| 307 | 33 | G | G | A | 8.62 | 1.277726e-01 |  |
| 307 | 33 | G | G | C | 3.45 | 3.876470e-01 |  |
| 307 | 33 | G | G | G | 86.21 | 5.894507e-12 | \* |
| 307 | 33 | G | G | T | 1.72 | 7.389914e-01 |  |
| 308 | 34 | G | G | A | 6.12 | 2.596828e-01 |  |
| 308 | 34 | G | G | C | 3.06 | 4.563301e-01 |  |
| 308 | 34 | G | G | G | 89.80 | 1.876055e-12 | \* |
| 308 | 34 | G | G | T | 1.02 | 8.378937e-01 |  |
| 309 | 35 | G | G | A | 7.41 | 1.816512e-01 |  |
| 309 | 35 | G | G | C | 1.23 | 8.000365e-01 |  |
| 309 | 35 | G | G | G | 91.36 | 1.139533e-12 | \* |
| 309 | 35 | G | G | T | 0.00 | 9.032258e-01 |  |
| 310 | 36 | C | C | A | 12.35 | 4.082911e-02 |  |
| 310 | 36 | C | C | C | 81.48 | 0.000000e+00 | \* |
| 310 | 36 | C | C | G | 1.23 | 7.815548e-01 |  |
| 310 | 36 | C | C | T | 4.94 | 2.834573e-01 |  |
| 311 | 37 | A | A | A | 97.14 | 2.775558e-15 | \* |
| 311 | 37 | A | A | C | 1.43 | 7.697164e-01 |  |
| 311 | 37 | A | A | G | 1.43 | 7.592254e-01 |  |
| 311 | 37 | A | A | T | 0.00 | 9.032258e-01 |  |
| 312 | 38 | C | C | A | 10.78 | 6.647327e-02 |  |
| 312 | 38 | C | C | C | 85.29 | 0.000000e+00 | \* |
| 312 | 38 | C | C | G | 0.00 | 8.777778e-01 |  |
| 312 | 38 | C | C | T | 3.92 | 4.017736e-01 |  |
| 313 | 39 | C | C | A | 15.74 | 1.368888e-02 |  |
| 313 | 39 | C | C | C | 76.85 | 0.000000e+00 | \* |
| 313 | 39 | C | C | G | 3.70 | 4.902266e-01 |  |
| 313 | 39 | C | C | T | 3.70 | 4.311125e-01 |  |
| 314 | 40 | C | C | A | 13.89 | 2.496686e-02 |  |
| 314 | 40 | C | C | C | 79.63 | 0.000000e+00 | \* |
| 314 | 40 | C | C | G | 0.00 | 8.777778e-01 |  |
| 314 | 40 | C | C | T | 6.48 | 1.590636e-01 |  |
| 315 | 41 | T | T | A | 5.95 | 2.718765e-01 |  |
| 315 | 41 | T | T | C | 2.38 | 5.887694e-01 |  |
| 315 | 41 | T | T | G | 4.76 | 3.853000e-01 |  |
| 315 | 41 | T | T | T | 86.90 | 0.000000e+00 | \* |
| 316 | 42 | G | G | A | 5.56 | 3.021557e-01 |  |
| 316 | 42 | G | G | C | 0.93 | 8.397271e-01 |  |
| 316 | 42 | G | G | G | 91.67 | 1.032507e-12 | \* |
| 316 | 42 | G | G | T | 1.85 | 7.189090e-01 |  |
| 317 | 43 | A | A | A | 92.31 | 1.676437e-14 | \* |
| 317 | 43 | A | A | C | 0.85 | 8.471465e-01 |  |
| 317 | 43 | A | A | G | 0.85 | 8.220595e-01 |  |
| 317 | 43 | A | A | T | 5.98 | 1.927169e-01 |  |
| 318 | 44 | C | C | A | 6.19 | 2.552744e-01 |  |
| 318 | 44 | C | C | C | 82.47 | 0.000000e+00 | \* |
| 318 | 44 | C | C | G | 7.22 | 2.098423e-01 |  |
| 318 | 44 | C | C | T | 4.12 | 3.757906e-01 |  |
| 319 | 45 | C | C | A | 7.59 | 1.721786e-01 |  |
| 319 | 45 | C | C | C | 86.08 | 0.000000e+00 | \* |
| 319 | 45 | C | C | G | 6.33 | 2.630362e-01 |  |
| 319 | 45 | C | C | T | 0.00 | 9.032258e-01 |  |
| 320 | 46 | G | G | A | 7.35 | 1.844876e-01 |  |
| 320 | 46 | G | G | C | 1.47 | 7.626994e-01 |  |
| 320 | 46 | G | G | G | 88.24 | 3.086864e-12 | \* |
| 320 | 46 | G | G | T | 2.94 | 5.436451e-01 |  |
| 321 | 47 | A | A | A | 96.99 | 2.997602e-15 | \* |
| 321 | 47 | A | A | C | 0.75 | 8.565970e-01 |  |
| 321 | 47 | A | A | G | 0.00 | 8.777778e-01 |  |
| 321 | 47 | A | A | T | 2.26 | 6.537507e-01 |  |
| 322 | 48 | C | C | A | 8.05 | 1.511753e-01 |  |
| 322 | 48 | C | C | C | 85.06 | 0.000000e+00 | \* |
| 322 | 48 | C | C | G | 0.00 | 8.777778e-01 |  |
| 322 | 48 | C | C | T | 6.90 | 1.351349e-01 |  |
| 323 | 49 | G | G | A | 16.00 | 1.257410e-02 |  |
| 323 | 49 | G | G | C | 0.00 | 8.823529e-01 |  |
| 323 | 49 | G | G | G | 80.00 | 4.257372e-11 | \* |
| 323 | 49 | G | G | T | 4.00 | 3.915488e-01 |  |
| 324 | 50 | G | G | A | 1.06 | 7.778806e-01 |  |
| 324 | 50 | G | G | C | 3.19 | 4.325258e-01 |  |
| 324 | 50 | G | G | G | 95.74 | 2.806644e-13 | \* |
| 324 | 50 | G | G | T | 0.00 | 9.032258e-01 |  |
| 325 | 51 | C | C | A | 10.29 | 7.727105e-02 |  |
| 325 | 51 | C | C | C | 75.00 | 0.000000e+00 | \* |
| 325 | 51 | C | C | G | 5.88 | 2.940064e-01 |  |
| 325 | 51 | C | C | T | 8.82 | 6.142187e-02 |  |
| 326 | 52 | A | A | A | 86.36 | 1.495470e-13 | \* |
| 326 | 52 | A | A | C | 2.27 | 6.104729e-01 |  |
| 326 | 52 | A | A | G | 0.00 | 8.777778e-01 |  |
| 326 | 52 | A | A | T | 11.36 | 2.048610e-02 |  |
| 327 | 53 | A | A | A | 93.75 | 9.880985e-15 | \* |
| 327 | 53 | A | A | C | 0.00 | 8.823529e-01 |  |
| 327 | 53 | A | A | G | 1.25 | 7.798106e-01 |  |
| 327 | 53 | A | A | T | 5.00 | 2.772603e-01 |  |
| 328 | 54 | G | G | A | 2.00 | 6.771347e-01 |  |
| 328 | 54 | G | G | C | 6.00 | 1.059103e-01 |  |
| 328 | 54 | G | G | G | 92.00 | 9.283685e-13 | \* |
| 328 | 54 | G | G | T | 0.00 | 9.032258e-01 |  |
| 329 | 55 | A | A | A | 89.69 | 4.396483e-14 | \* |
| 329 | 55 | A | A | C | 1.03 | 8.275447e-01 |  |
| 329 | 55 | A | A | G | 6.19 | 2.726662e-01 |  |
| 329 | 55 | A | A | T | 3.09 | 5.201916e-01 |  |
| 330 | 56 | A | A | A | 90.79 | 2.930989e-14 | \* |
| 330 | 56 | A | A | C | 0.00 | 8.823529e-01 |  |
| 330 | 56 | A | A | G | 1.32 | 7.723087e-01 |  |
| 330 | 56 | A | A | T | 7.89 | 9.035067e-02 |  |
| 331 | 57 | G | G | A | 11.32 | 5.629705e-02 |  |
| 331 | 57 | G | G | C | 3.77 | 3.350966e-01 |  |
| 331 | 57 | G | G | G | 77.36 | 9.864742e-11 | \* |
| 331 | 57 | G | G | T | 7.55 | 1.041085e-01 |  |
| 332 | 58 | T | T | A | 0.00 | 8.363636e-01 |  |
| 332 | 58 | T | T | C | 2.63 | 5.388192e-01 |  |
| 332 | 58 | T | T | G | 3.95 | 4.644776e-01 |  |
| 332 | 58 | T | T | T | 93.42 | 0.000000e+00 | \* |
| 333 | 59 | T | T | A | 23.53 | 1.001247e-03 | \* |
| 333 | 59 | T | T | C | 1.18 | 8.083727e-01 |  |
| 333 | 59 | T | T | G | 3.53 | 5.091959e-01 |  |
| 333 | 59 | T | T | T | 71.76 | 1.554312e-15 | \* |
| 334 | 60 | C | C | A | 7.59 | 1.721786e-01 |  |
| 334 | 60 | C | C | C | 82.28 | 0.000000e+00 | \* |
| 334 | 60 | C | C | G | 1.27 | 7.780160e-01 |  |
| 334 | 60 | C | C | T | 8.86 | 6.046690e-02 |  |
| 335 | 61 | G | G | A | 11.76 | 4.901246e-02 |  |
| 335 | 61 | G | G | C | 3.92 | 3.128758e-01 |  |
| 335 | 61 | G | G | G | 80.39 | 3.757816e-11 | \* |
| 335 | 61 | G | G | T | 3.92 | 4.017736e-01 |  |
| 336 | 62 | A | A | A | 92.17 | 1.765255e-14 | \* |
| 336 | 62 | A | A | C | 1.74 | 7.148375e-01 |  |
| 336 | 62 | A | A | G | 0.87 | 8.205815e-01 |  |
| 336 | 62 | A | A | T | 5.22 | 2.563048e-01 |  |
| 337 | 63 | C | C | A | 6.33 | 2.454810e-01 |  |
| 337 | 63 | C | C | C | 87.34 | 0.000000e+00 | \* |
| 337 | 63 | C | C | G | 1.27 | 7.780160e-01 |  |
| 337 | 63 | C | C | T | 5.06 | 2.710205e-01 |  |
| 338 | 64 | A | A | A | 80.00 | 1.548872e-12 | \* |
| 338 | 64 | A | A | C | 5.45 | 1.433219e-01 |  |
| 338 | 64 | A | A | G | 1.82 | 7.125310e-01 |  |
| 338 | 64 | A | A | T | 12.73 | 1.114006e-02 |  |
| 339 | 65 | G | G | A | 0.00 | 8.363636e-01 |  |
| 339 | 65 | G | G | C | 4.05 | 2.938936e-01 |  |
| 339 | 65 | G | G | G | 89.19 | 2.276845e-12 | \* |
| 339 | 65 | G | G | T | 6.76 | 1.428069e-01 |  |
| 340 | 66 | C | C | A | 6.58 | 2.291918e-01 |  |
| 340 | 66 | C | C | C | 88.16 | 0.000000e+00 | \* |
| 340 | 66 | C | C | G | 0.00 | 8.777778e-01 |  |
| 340 | 66 | C | C | T | 5.26 | 2.520630e-01 |  |
| 341 | 67 | T | T | A | 0.00 | 8.363636e-01 |  |
| 341 | 67 | T | T | C | 3.33 | 4.073836e-01 |  |
| 341 | 67 | T | T | G | 5.56 | 3.185719e-01 |  |
| 341 | 67 | T | T | T | 91.11 | 0.000000e+00 | \* |
| 342 | 68 | C | C | A | 9.76 | 9.100906e-02 |  |
| 342 | 68 | C | C | C | 85.37 | 0.000000e+00 | \* |
| 342 | 68 | C | C | G | 3.66 | 4.950992e-01 |  |
| 342 | 68 | C | C | T | 1.22 | 8.126785e-01 |  |
| 343 | 69 | C | C | A | 4.04 | 4.420244e-01 |  |
| 343 | 69 | C | C | C | 88.89 | 0.000000e+00 | \* |
| 343 | 69 | C | C | G | 0.00 | 8.777778e-01 |  |
| 343 | 69 | C | C | T | 7.07 | 1.260979e-01 |  |
| 344 | 70 | C | C | A | 7.02 | 2.028281e-01 |  |
| 344 | 70 | C | C | C | 89.47 | 0.000000e+00 | \* |
| 344 | 70 | C | C | G | 3.51 | 5.114716e-01 |  |
| 344 | 70 | C | C | T | 0.00 | 9.032258e-01 |  |
| 345 | 71 | G | G | A | 0.00 | 8.363636e-01 |  |
| 345 | 71 | G | G | C | 5.56 | 1.356269e-01 |  |
| 345 | 71 | G | G | G | 94.44 | 4.252154e-13 | \* |
| 345 | 71 | G | G | T | 0.00 | 9.032258e-01 |  |
| 346 | 72 | C | C | A | 2.82 | 5.799015e-01 |  |
| 346 | 72 | C | C | C | 94.37 | 0.000000e+00 | \* |
| 346 | 72 | C | C | G | 0.00 | 8.777778e-01 |  |
| 346 | 72 | C | C | T | 2.82 | 5.631895e-01 |  |
| 347 | 73 | G | G | A | 9.38 | 1.020847e-01 |  |
| 347 | 73 | G | G | C | 7.81 | 3.628032e-02 |  |
| 347 | 73 | G | G | G | 79.69 | 4.702527e-11 | \* |
| 347 | 73 | G | G | T | 3.12 | 5.152686e-01 |  |
| 348 | 74 | A | A | A | 83.65 | 4.048983e-13 | \* |
| 348 | 74 | A | A | C | 5.77 | 1.205297e-01 |  |
| 348 | 74 | A | A | G | 0.00 | 8.777778e-01 |  |
| 348 | 74 | A | A | T | 10.58 | 2.894836e-02 |  |
| 349 | 75 | C | C | A | 3.30 | 5.238225e-01 |  |
| 349 | 75 | C | C | C | 84.62 | 0.000000e+00 | \* |
| 349 | 75 | C | C | G | 0.00 | 8.777778e-01 |  |
| 349 | 75 | C | C | T | 12.09 | 1.484444e-02 |  |

## For use in R

If you want to work with the results in R, here is output that you can copy and paste in your terminal to get:

The base information:

```
structure(list(focal.base = c("A", "C", "G", "T"), avg.percsignal = c(87.9334421355688, 
85.2680962617039, 88.4405171897635, 90.106636389754), avg.areasignal = c(95.0588235294118, 
99.469696969697, 90.2280701754386, 108.613636363636), crit.perc.area = c(16.6963875326785, 
9.84444687834951, 18.0800928200576, 12.9663639575222), mu = c(5.12105544359753, 
3.53961654252729, 5.17604263476502, 4.16141488454648), fillibens = c(0.992709391240568, 
0.995241665651113, 0.99021329613688, 0.988326518687476)), .Names = c("focal.base", 
"avg.percsignal", "avg.areasignal", "crit.perc.area", "mu", "fillibens"
), row.names = c(NA, -4L), class = "data.frame")
```

the data.frame that contains information on the guide region:

```
structure(list(A.area = c(77, 97, 3, 177, 12, 6, 8, 7, 10, 7, 
71, 7, 180, 10, 10, 4, 7, 5, 0, 1, 0, 5, 2, 6, 4, 62, 14, 3, 
87, 9, 60, 4, 5, 6, 6, 10, 68, 11, 17, 15, 5, 6, 108, 6, 6, 5, 
129, 7, 8, 1, 7, 38, 75, 1, 87, 69, 6, 0, 20, 6, 6, 106, 5, 44, 
0, 5, 0, 8, 4, 4, 0, 2, 6, 87, 3), C.area = c(3, 8, 9, 1, 89, 
1, 5, 3, 68, 98, 1, 1, 2, 97, 75, 2, 0, 2, 3, 0, 4, 5, 2, 2, 
76, 2, 80, 3, 5, 75, 3, 69, 2, 3, 1, 66, 1, 87, 83, 86, 2, 1, 
1, 80, 68, 1, 1, 74, 0, 3, 51, 1, 0, 3, 1, 0, 2, 2, 1, 65, 2, 
2, 69, 3, 3, 67, 3, 70, 88, 51, 3, 67, 5, 6, 77), G.area = c(2, 
2, 63, 2, 0, 2, 168, 106, 4, 8, 9, 82, 1, 6, 1, 84, 2, 144, 4, 
5, 2, 113, 4, 89, 1, 8, 2, 4, 3, 2, 7, 2, 50, 88, 74, 1, 1, 0, 
4, 0, 4, 99, 1, 7, 5, 60, 0, 0, 40, 90, 4, 0, 1, 46, 6, 1, 41, 
3, 3, 1, 41, 1, 1, 1, 66, 0, 5, 3, 0, 2, 51, 0, 51, 0, 0), T.area = c(6, 
6, 0, 7, 7, 84, 1, 1, 5, 11, 4, 0, 7, 5, 2, 2, 75, 6, 83, 94, 
91, 3, 76, 10, 10, 8, 9, 75, 3, 5, 0, 7, 1, 1, 0, 4, 0, 4, 4, 
7, 73, 2, 7, 4, 0, 2, 3, 6, 2, 0, 6, 5, 4, 0, 3, 6, 4, 71, 61, 
7, 2, 6, 4, 7, 5, 4, 82, 1, 7, 0, 0, 2, 2, 11, 11), Tot.area = c(88, 
113, 75, 187, 108, 93, 182, 117, 87, 124, 85, 90, 190, 118, 88, 
92, 84, 157, 90, 100, 97, 126, 84, 107, 91, 80, 105, 85, 98, 
91, 70, 82, 58, 98, 81, 81, 70, 102, 108, 108, 84, 108, 117, 
97, 79, 68, 133, 87, 50, 94, 68, 44, 80, 50, 97, 76, 53, 76, 
85, 79, 51, 115, 79, 55, 74, 76, 90, 82, 99, 57, 54, 71, 64, 
104, 91), A.perc = c(87.5, 85.8407079646018, 4, 94.6524064171123, 
11.1111111111111, 6.45161290322581, 4.3956043956044, 5.98290598290598, 
11.4942528735632, 5.64516129032258, 83.5294117647059, 7.77777777777778, 
94.7368421052632, 8.47457627118644, 11.3636363636364, 4.34782608695652, 
8.33333333333333, 3.18471337579618, 0, 1, 0, 3.96825396825397, 
2.38095238095238, 5.60747663551402, 4.3956043956044, 77.5, 13.3333333333333, 
3.52941176470588, 88.7755102040816, 9.89010989010989, 85.7142857142857, 
4.8780487804878, 8.62068965517241, 6.12244897959184, 7.40740740740741, 
12.3456790123457, 97.1428571428571, 10.7843137254902, 15.7407407407407, 
13.8888888888889, 5.95238095238095, 5.55555555555556, 92.3076923076923, 
6.18556701030928, 7.59493670886076, 7.35294117647059, 96.9924812030075, 
8.04597701149425, 16, 1.06382978723404, 10.2941176470588, 86.3636363636364, 
93.75, 2, 89.6907216494845, 90.7894736842105, 11.3207547169811, 
0, 23.5294117647059, 7.59493670886076, 11.7647058823529, 92.1739130434783, 
6.32911392405063, 80, 0, 6.57894736842105, 0, 9.75609756097561, 
4.04040404040404, 7.01754385964912, 0, 2.8169014084507, 9.375, 
83.6538461538462, 3.2967032967033), C.perc = c(3.40909090909091, 
7.07964601769912, 12, 0.53475935828877, 82.4074074074074, 1.0752688172043, 
2.74725274725275, 2.56410256410256, 78.1609195402299, 79.0322580645161, 
1.17647058823529, 1.11111111111111, 1.05263157894737, 82.2033898305085, 
85.2272727272727, 2.17391304347826, 0, 1.27388535031847, 3.33333333333333, 
0, 4.12371134020619, 3.96825396825397, 2.38095238095238, 1.86915887850467, 
83.5164835164835, 2.5, 76.1904761904762, 3.52941176470588, 5.10204081632653, 
82.4175824175824, 4.28571428571429, 84.1463414634146, 3.44827586206897, 
3.06122448979592, 1.23456790123457, 81.4814814814815, 1.42857142857143, 
85.2941176470588, 76.8518518518518, 79.6296296296296, 2.38095238095238, 
0.925925925925926, 0.854700854700855, 82.4742268041237, 86.0759493670886, 
1.47058823529412, 0.75187969924812, 85.0574712643678, 0, 3.19148936170213, 
75, 2.27272727272727, 0, 6, 1.03092783505155, 0, 3.77358490566038, 
2.63157894736842, 1.17647058823529, 82.2784810126582, 3.92156862745098, 
1.73913043478261, 87.3417721518987, 5.45454545454545, 4.05405405405405, 
88.1578947368421, 3.33333333333333, 85.3658536585366, 88.8888888888889, 
89.4736842105263, 5.55555555555556, 94.3661971830986, 7.8125, 
5.76923076923077, 84.6153846153846), G.perc = c(2.27272727272727, 
1.76991150442478, 84, 1.06951871657754, 0, 2.1505376344086, 92.3076923076923, 
90.5982905982906, 4.59770114942529, 6.45161290322581, 10.5882352941176, 
91.1111111111111, 0.526315789473684, 5.08474576271186, 1.13636363636364, 
91.304347826087, 2.38095238095238, 91.7197452229299, 4.44444444444444, 
5, 2.06185567010309, 89.6825396825397, 4.76190476190476, 83.1775700934579, 
1.0989010989011, 10, 1.9047619047619, 4.70588235294118, 3.06122448979592, 
2.1978021978022, 10, 2.4390243902439, 86.2068965517241, 89.7959183673469, 
91.358024691358, 1.23456790123457, 1.42857142857143, 0, 3.7037037037037, 
0, 4.76190476190476, 91.6666666666667, 0.854700854700855, 7.21649484536082, 
6.32911392405063, 88.2352941176471, 0, 0, 80, 95.7446808510638, 
5.88235294117647, 0, 1.25, 92, 6.18556701030928, 1.31578947368421, 
77.3584905660377, 3.94736842105263, 3.52941176470588, 1.26582278481013, 
80.3921568627451, 0.869565217391304, 1.26582278481013, 1.81818181818182, 
89.1891891891892, 0, 5.55555555555556, 3.65853658536585, 0, 3.50877192982456, 
94.4444444444444, 0, 79.6875, 0, 0), T.perc = c(6.81818181818182, 
5.30973451327434, 0, 3.74331550802139, 6.48148148148148, 90.3225806451613, 
0.549450549450549, 0.854700854700855, 5.74712643678161, 8.87096774193548, 
4.70588235294118, 0, 3.68421052631579, 4.23728813559322, 2.27272727272727, 
2.17391304347826, 89.2857142857143, 3.82165605095541, 92.2222222222222, 
94, 93.8144329896907, 2.38095238095238, 90.4761904761905, 9.34579439252336, 
10.989010989011, 10, 8.57142857142857, 88.2352941176471, 3.06122448979592, 
5.49450549450549, 0, 8.53658536585366, 1.72413793103448, 1.02040816326531, 
0, 4.93827160493827, 0, 3.92156862745098, 3.7037037037037, 6.48148148148148, 
86.9047619047619, 1.85185185185185, 5.98290598290598, 4.12371134020619, 
0, 2.94117647058824, 2.25563909774436, 6.89655172413793, 4, 0, 
8.82352941176471, 11.3636363636364, 5, 0, 3.09278350515464, 7.89473684210526, 
7.54716981132075, 93.4210526315789, 71.7647058823529, 8.86075949367089, 
3.92156862745098, 5.21739130434783, 5.06329113924051, 12.7272727272727, 
6.75675675675676, 5.26315789473684, 91.1111111111111, 1.21951219512195, 
7.07070707070707, 0, 0, 2.8169014084507, 3.125, 10.5769230769231, 
12.0879120879121), base.call = c("A", "A", "G", "A", "C", "T", 
"G", "G", "C", "C", "A", "G", "A", "C", "C", "G", "T", "G", "T", 
"T", "T", "G", "T", "G", "C", "A", "C", "T", "A", "C", "A", "C", 
"G", "G", "G", "C", "A", "C", "C", "C", "T", "G", "A", "C", "C", 
"G", "A", "C", "G", "G", "C", "A", "A", "G", "A", "A", "G", "T", 
"T", "C", "G", "A", "C", "A", "G", "C", "T", "C", "C", "C", "G", 
"C", "G", "A", "C"), index = 275:349, guide.seq = c("A", "A", 
"G", "A", "C", "T", "G", "G", "C", "C", "A", "G", "A", "C", "C", 
"G", "T", "G", "T", "T", "T", "G", "T", "G", "C", "A", "C", "T", 
"A", "C", "A", "C", "G", "G", "G", "C", "A", "C", "C", "C", "T", 
"G", "A", "C", "C", "G", "A", "C", "G", "G", "C", "A", "A", "G", 
"A", "A", "G", "T", "T", "C", "G", "A", "C", "A", "G", "C", "T", 
"C", "C", "C", "G", "C", "G", "A", "C"), T.pval = c(0.139388899437763, 
0.247805833731919, 0.903225806446376, 0.425677148521841, 0.159063593647121, 
0, 0.883717595215759, 0.856507559235713, 0.210682643372537, 0.0602075009811534, 
0.30778468921493, 0.903225806446376, 0.433803415771503, 0.361725039977273, 
0.650968578769155, 0.667047437669509, 0, 0.415059014389785, 0, 
0, 0, 0.633351878854183, 0, 0.0492377928480918, 0.0241663912394137, 
0.0371900200782536, 0.0682709886233683, 0, 0.525035550344532, 
0.231502680917041, 0.903225806446376, 0.069272095588599, 0.738991397057936, 
0.837893673740619, 0.903225806446376, 0.283457271878428, 0.903225806446376, 
0.401773632344007, 0.431112459112588, 0.159063593647121, 0, 0.718909025578949, 
0.19271687709887, 0.375790611726387, 0.903225806446376, 0.543645124102212, 
0.653750667193968, 0.1351349056361, 0.391548841710402, 0.903225806446376, 
0.0614218674593536, 0.020486095854878, 0.277260317324496, 0.903225806446376, 
0.5201915987292, 0.0903506742542668, 0.104108511061795, 0, 1.55431223447522e-15, 
0.0604668966879347, 0.401773632344007, 0.256304773180899, 0.271020480553137, 
0.0111400569097257, 0.142806851124588, 0.252062965694733, 0, 
0.812678540761511, 0.126097933184007, 0.903225806446376, 0.903225806446376, 
0.563189523381799, 0.515268551743232, 0.0289483642226112, 0.0148444387037754
), C.pval = c(0.394309275941231, 0.0565517117993695, 0.00237875206607308, 
0.871488384162423, 0, 0.821974784711508, 0.516112452902685, 0.552187077109467, 
0, 0, 0.808372715062701, 0.817295822435423, 0.824849017387415, 
0, 0, 0.630252846265355, 0.882352941176386, 0.794189843883828, 
0.407383609956445, 0.882352941176386, 0.284259412904863, 0.306088365855569, 
0.588769418663406, 0.690197677986044, 0, 0.564951247095725, 0, 
0.374074505101867, 0.173202718456733, 0, 0.262771398353106, 0, 
0.387646983422035, 0.456330080929778, 0.80003652198676, 0, 0.769716400369933, 
0, 0, 0, 0.588769418663406, 0.839727085339026, 0.847146469248985, 
0, 0, 0.762699437148795, 0.856597017931389, 0, 0.882352941176386, 
0.432525757789463, 0, 0.610472921514542, 0.882352941176386, 0.105910263717202, 
0.827544700530215, 0.882352941176386, 0.335096610633678, 0.538819212535454, 
0.808372715062701, 0, 0.312875778495781, 0.714837526411331, 0, 
0.143321931219451, 0.29389364502817, 0, 0.407383609956445, 0, 
0, 0, 0.135626905674112, 0, 0.0362803187113134, 0.120529654839816, 
0), G.pval = c(0.656849061162515, 0.718402741839559, 1.19108056750861e-11, 
0.799782982920781, 0.877777777777744, 0.67183216028342, 8.41438030363406e-13, 
1.45228273851217e-12, 0.400388556906601, 0.255052487369742, 0.0851563928719147, 
1.2330136911487e-12, 0.851556293896307, 0.356924893733832, 0.792500392780935, 
1.15918386001113e-12, 0.643612007647826, 1.01518793371724e-12, 
0.414871293537965, 0.364208302243965, 0.682714628133619, 1.94511073914327e-12, 
0.385299962352558, 1.54787294093239e-11, 0.796599836147888, 0.100059607778597, 
0.701963273750427, 0.390397983115944, 0.562245906897624, 0.666033516985034, 
0.100059607778597, 0.636529555536903, 5.89450710464234e-12, 1.87605486701159e-12, 
1.13953291247526e-12, 0.781554765339795, 0.759225369652085, 0.877777777777744, 
0.49022659589076, 0.877777777777744, 0.385299962352558, 1.0325074129014e-12, 
0.822059545861224, 0.209842265242928, 0.263036179376121, 3.08686409766779e-12, 
0.877777777777744, 0.877777777777744, 4.25737223253009e-11, 2.8066438062524e-13, 
0.294006372295669, 0.877777777777744, 0.779810609252902, 9.28368493191556e-13, 
0.272666198774542, 0.772308671208171, 9.86474235631363e-11, 0.464477601692029, 
0.509195863402053, 0.778015979873185, 3.75781628036975e-11, 0.820581451576496, 
0.778015979873185, 0.712531032624256, 2.27684537890127e-12, 0.877777777777744, 
0.318571909616777, 0.495099205469812, 0.877777777777744, 0.51147164564186, 
4.25215418431435e-13, 0.877777777777744, 4.70252725648379e-11, 
0.877777777777744, 0.877777777777744), A.pval = c(9.84767822842514e-14, 
1.81188397618826e-13, 0.446268675448494, 7.105427357601e-15, 
0.0600843332735771, 0.23737548417206, 0.405812871964253, 0.269653745993373, 
0.0533353585938987, 0.295091638981452, 4.23772128499422e-13, 
0.163368513676432, 6.88338275267597e-15, 0.13338711530514, 0.0555508277747109, 
0.41056605587737, 0.13902729727731, 0.536730683534851, 0.836363636363636, 
0.783683935021022, 0.836363636363636, 0.449620873508051, 0.63193221507733, 
0.298046239553658, 0.405812871964253, 3.87534448975657e-12, 0.0298339119606094, 
0.497445367032197, 6.16173778666962e-14, 0.0873881219030345, 
1.89848137210902e-13, 0.359939678278541, 0.127772597234428, 0.259682819033577, 
0.181651228147183, 0.0408291100488214, 2.77555756156289e-15, 
0.0664732700164694, 0.0136888788046846, 0.0249668555122319, 0.271876476497947, 
0.302155699020882, 1.67643676718399e-14, 0.255274384533865, 0.17217856144709, 
0.184487597933975, 2.99760216648792e-15, 0.151175281991107, 0.0125740986130127, 
0.7778806084963, 0.0772710502302753, 1.49547041417009e-13, 9.88098491916389e-15, 
0.67713470609711, 4.39648317751562e-14, 2.93098878501041e-14, 
0.0562970506198474, 0.836363636363636, 0.0010012469657682, 0.17217856144709, 
0.0490124646642569, 1.765254609154e-14, 0.2454809611056, 1.54887214165456e-12, 
0.836363636363636, 0.229191773899393, 0.836363636363636, 0.0910090560960443, 
0.442024364899661, 0.202828114401002, 0.836363636363636, 0.579901523521784, 
0.102084733069154, 4.04898337080795e-13, 0.523822485383696), 
    guide.position = 1:75), .Names = c("A.area", "C.area", "G.area", 
"T.area", "Tot.area", "A.perc", "C.perc", "G.perc", "T.perc", 
"base.call", "index", "guide.seq", "T.pval", "C.pval", "G.pval", 
"A.pval", "guide.position"), row.names = 275:349, class = "data.frame")
```

*Report generated using EditR v1.0.8*
