# Supplementary material for: Mechanistic and genetic basis of single-strand templated repair at Cas12a-induced DNA breaks in Chlamydomonas reinhardtii
Source: Nat Commun. 2021 Nov 19;12:6751. doi: 10.1038/s41467-021-27004-1 (PMC8604939; doi:10.1038/s41467-021-27004-1)
Supplement: Supplementary file 22 — Source Data [file 41467_2021_27004_MOESM22_ESM.zip › Source Data/EditR analysis/EditR outputs/Sense/rep3_ssODN_sense_-16.html]

EditR v1.0.8 report


# EditR v1.0.8 report

- Data QA
  - Filtering data
  - Percent noise peak area
  - Base information
- Predicted editing
  - Editing bar plot
  - Editing table plot
  - Table of editing results
- For use in R

## Data QA

### Filtering data

What the data looked like prefiltering:

and the post filtering signal / noise plot:

### Percent noise peak area

### Base information

Here’s information about the signal of each base, the critical percent value where any higher value would be called as significant, and Filliben’s correlation for how well the noise was modelled by the zero adjusted gamma distribution.

| Base | Average percent signal | Average peak area | Critical percent value | model mu | Fillibens correlation |
| --- | --- | --- | --- | --- | --- |
| A | 95.05614 | 318.4167 | 7.069299 | 2.527636 | 0.9901889 |
| C | 94.12083 | 340.2308 | 5.539381 | 2.005111 | 0.9835050 |
| G | 93.26943 | 299.1053 | 5.556187 | 1.609704 | 0.9923642 |
| T | 94.71902 | 354.6512 | 5.447917 | 2.129419 | 0.9951375 |

## Predicted editing

### Editing bar plot

### Editing table plot

### Table of editing results


Here’s the entire guide region

| Sanger position | Guide position | Guide sequence | Sanger base call | Focal base | Focal base peak area | p value |  |
| --- | --- | --- | --- | --- | --- | --- | --- |
| 272 | 1 | A | A | A | 94.92 | 0.000000e+00 | \* |
| 272 | 1 | A | A | C | 2.86 | 1.696937e-01 |  |
| 272 | 1 | A | A | G | 1.27 | 4.719435e-01 |  |
| 272 | 1 | A | A | T | 0.95 | 8.354362e-01 |  |
| 273 | 2 | A | A | A | 96.78 | 0.000000e+00 | \* |
| 273 | 2 | A | A | C | 1.17 | 6.180718e-01 |  |
| 273 | 2 | A | A | G | 2.05 | 2.557156e-01 |  |
| 273 | 2 | A | A | T | 0.00 | 9.468085e-01 |  |
| 274 | 3 | G | G | A | 5.74 | 3.294206e-02 |  |
| 274 | 3 | G | G | C | 1.64 | 4.625006e-01 |  |
| 274 | 3 | G | G | G | 91.39 | 0.000000e+00 | \* |
| 274 | 3 | G | G | T | 1.23 | 7.437088e-01 |  |
| 275 | 4 | A | A | A | 96.83 | 0.000000e+00 | \* |
| 275 | 4 | A | A | C | 1.27 | 5.852956e-01 |  |
| 275 | 4 | A | A | G | 0.95 | 5.904973e-01 |  |
| 275 | 4 | A | A | T | 0.95 | 8.358779e-01 |  |
| 276 | 5 | C | C | A | 4.03 | 1.350280e-01 |  |
| 276 | 5 | C | C | C | 91.94 | 0.000000e+00 | \* |
| 276 | 5 | C | C | G | 1.61 | 3.634788e-01 |  |
| 276 | 5 | C | C | T | 2.42 | 3.180651e-01 |  |
| 277 | 6 | T | T | A | 4.58 | 8.724671e-02 |  |
| 277 | 6 | T | T | C | 2.16 | 3.131198e-01 |  |
| 277 | 6 | T | T | G | 3.77 | 5.502040e-02 |  |
| 277 | 6 | T | T | T | 89.49 | 0.000000e+00 | \* |
| 278 | 7 | G | G | A | 2.27 | 4.568727e-01 |  |
| 278 | 7 | G | G | C | 0.65 | 7.707810e-01 |  |
| 278 | 7 | G | G | G | 95.95 | 0.000000e+00 | \* |
| 278 | 7 | G | G | T | 1.13 | 7.772297e-01 |  |
| 279 | 8 | G | G | A | 3.59 | 1.884018e-01 |  |
| 279 | 8 | G | G | C | 1.80 | 4.136240e-01 |  |
| 279 | 8 | G | G | G | 93.71 | 0.000000e+00 | \* |
| 279 | 8 | G | G | T | 0.90 | 8.508132e-01 |  |
| 280 | 9 | C | C | A | 3.57 | 1.914025e-01 |  |
| 280 | 9 | C | C | C | 92.86 | 0.000000e+00 | \* |
| 280 | 9 | C | C | G | 2.78 | 1.362336e-01 |  |
| 280 | 9 | C | C | T | 0.79 | 8.775012e-01 |  |
| 281 | 10 | C | C | A | 2.81 | 3.271057e-01 |  |
| 281 | 10 | C | C | C | 96.17 | 0.000000e+00 | \* |
| 281 | 10 | C | C | G | 0.00 | 9.111111e-01 |  |
| 281 | 10 | C | C | T | 1.02 | 8.147850e-01 |  |
| 282 | 11 | A | A | A | 95.55 | 0.000000e+00 | \* |
| 282 | 11 | A | A | C | 2.40 | 2.560356e-01 |  |
| 282 | 11 | A | A | G | 2.05 | 2.540147e-01 |  |
| 282 | 11 | A | A | T | 0.00 | 9.468085e-01 |  |
| 283 | 12 | G | G | A | 5.83 | 3.040460e-02 |  |
| 283 | 12 | G | G | C | 2.24 | 2.918230e-01 |  |
| 283 | 12 | G | G | G | 91.93 | 0.000000e+00 | \* |
| 283 | 12 | G | G | T | 0.00 | 9.468085e-01 |  |
| 284 | 13 | A | A | A | 96.67 | 0.000000e+00 | \* |
| 284 | 13 | A | A | C | 1.40 | 5.398155e-01 |  |
| 284 | 13 | A | A | G | 1.05 | 5.510504e-01 |  |
| 284 | 13 | A | A | T | 0.88 | 8.565023e-01 |  |
| 285 | 14 | C | C | A | 4.01 | 1.373015e-01 |  |
| 285 | 14 | C | C | C | 93.85 | 0.000000e+00 | \* |
| 285 | 14 | C | C | G | 0.53 | 7.593131e-01 |  |
| 285 | 14 | C | C | T | 1.60 | 6.010231e-01 |  |
| 286 | 15 | C | C | A | 4.02 | 1.358140e-01 |  |
| 286 | 15 | C | C | C | 91.95 | 0.000000e+00 | \* |
| 286 | 15 | C | C | G | 2.48 | 1.773782e-01 |  |
| 286 | 15 | C | C | T | 1.55 | 6.228725e-01 |  |
| 287 | 16 | G | G | A | 4.09 | 1.287433e-01 |  |
| 287 | 16 | G | G | C | 1.75 | 4.264404e-01 |  |
| 287 | 16 | G | G | G | 91.23 | 0.000000e+00 | \* |
| 287 | 16 | G | G | T | 2.92 | 1.964598e-01 |  |
| 288 | 17 | T | T | A | 0.00 | 9.259259e-01 |  |
| 288 | 17 | T | T | C | 0.80 | 7.314742e-01 |  |
| 288 | 17 | T | T | G | 3.21 | 9.218467e-02 |  |
| 288 | 17 | T | T | T | 95.98 | 0.000000e+00 | \* |
| 289 | 18 | G | G | A | 1.62 | 6.423662e-01 |  |
| 289 | 18 | G | G | C | 0.00 | 8.372093e-01 |  |
| 289 | 18 | G | G | G | 96.76 | 0.000000e+00 | \* |
| 289 | 18 | G | G | T | 1.62 | 5.951481e-01 |  |
| 290 | 19 | T | T | A | 1.70 | 6.183885e-01 |  |
| 290 | 19 | T | T | C | 0.00 | 8.372093e-01 |  |
| 290 | 19 | T | T | G | 3.74 | 5.668827e-02 |  |
| 290 | 19 | T | T | T | 94.56 | 0.000000e+00 | \* |
| 291 | 20 | T | T | A | 1.78 | 5.964513e-01 |  |
| 291 | 20 | T | T | C | 0.00 | 8.372093e-01 |  |
| 291 | 20 | T | T | G | 2.66 | 1.507953e-01 |  |
| 291 | 20 | T | T | T | 95.56 | 0.000000e+00 | \* |
| 292 | 21 | T | T | A | 0.29 | 9.205586e-01 |  |
| 292 | 21 | T | T | C | 0.58 | 7.860374e-01 |  |
| 292 | 21 | T | T | G | 3.47 | 7.299189e-02 |  |
| 292 | 21 | T | T | T | 95.66 | 0.000000e+00 | \* |
| 293 | 22 | G | G | A | 1.01 | 8.120894e-01 |  |
| 293 | 22 | G | G | C | 0.00 | 8.372093e-01 |  |
| 293 | 22 | G | G | G | 97.24 | 0.000000e+00 | \* |
| 293 | 22 | G | G | T | 1.76 | 5.415712e-01 |  |
| 294 | 23 | T | T | A | 1.36 | 7.177467e-01 |  |
| 294 | 23 | T | T | C | 1.36 | 5.541961e-01 |  |
| 294 | 23 | T | T | G | 1.36 | 4.412336e-01 |  |
| 294 | 23 | T | T | T | 95.92 | 0.000000e+00 | \* |
| 295 | 24 | G | G | A | 1.12 | 7.824808e-01 |  |
| 295 | 24 | G | G | C | 1.69 | 4.479050e-01 |  |
| 295 | 24 | G | G | G | 95.51 | 0.000000e+00 | \* |
| 295 | 24 | G | G | T | 1.69 | 5.696649e-01 |  |
| 296 | 25 | C | C | A | 1.57 | 6.576973e-01 |  |
| 296 | 25 | C | C | C | 94.04 | 0.000000e+00 | \* |
| 296 | 25 | C | C | G | 2.19 | 2.259269e-01 |  |
| 296 | 25 | C | C | T | 2.19 | 3.867288e-01 |  |
| 297 | 26 | A | A | A | 91.80 | 0.000000e+00 | \* |
| 297 | 26 | A | A | C | 4.10 | 4.912696e-02 |  |
| 297 | 26 | A | A | G | 2.05 | 2.552057e-01 |  |
| 297 | 26 | A | A | T | 2.05 | 4.354939e-01 |  |
| 298 | 27 | C | C | A | 2.14 | 4.917028e-01 |  |
| 298 | 27 | C | C | C | 85.02 | 0.000000e+00 | \* |
| 298 | 27 | C | C | G | 10.40 | 7.631818e-05 | \* |
| 298 | 27 | C | C | T | 2.45 | 3.103855e-01 |  |
| 299 | 28 | T | T | A | 3.50 | 2.012468e-01 |  |
| 299 | 28 | T | T | C | 2.23 | 2.949448e-01 |  |
| 299 | 28 | T | T | G | 0.00 | 9.111111e-01 |  |
| 299 | 28 | T | T | T | 94.27 | 0.000000e+00 | \* |
| 300 | 29 | A | A | A | 95.04 | 0.000000e+00 | \* |
| 300 | 29 | A | A | C | 2.33 | 2.705862e-01 |  |
| 300 | 29 | A | A | G | 0.87 | 6.208452e-01 |  |
| 300 | 29 | A | A | T | 1.75 | 5.451957e-01 |  |
| 301 | 30 | C | C | A | 2.19 | 4.788514e-01 |  |
| 301 | 30 | C | C | C | 95.62 | 0.000000e+00 | \* |
| 301 | 30 | C | C | G | 0.62 | 7.227430e-01 |  |
| 301 | 30 | C | C | T | 1.56 | 6.172377e-01 |  |
| 302 | 31 | A | A | A | 93.23 | 0.000000e+00 | \* |
| 302 | 31 | A | A | C | 3.19 | 1.240211e-01 |  |
| 302 | 31 | A | A | G | 3.59 | 6.550091e-02 |  |
| 302 | 31 | A | A | T | 0.00 | 9.468085e-01 |  |
| 303 | 32 | C | C | A | 1.63 | 6.380723e-01 |  |
| 303 | 32 | C | C | C | 94.44 | 0.000000e+00 | \* |
| 303 | 32 | C | C | G | 2.94 | 1.177826e-01 |  |
| 303 | 32 | C | C | T | 0.98 | 8.271058e-01 |  |
| 304 | 33 | G | G | A | 2.78 | 3.332485e-01 |  |
| 304 | 33 | G | G | C | 3.17 | 1.255509e-01 |  |
| 304 | 33 | G | G | G | 92.86 | 0.000000e+00 | \* |
| 304 | 33 | G | G | T | 1.19 | 7.576960e-01 |  |
| 305 | 34 | G | G | A | 0.00 | 9.259259e-01 |  |
| 305 | 34 | G | G | C | 1.87 | 3.919310e-01 |  |
| 305 | 34 | G | G | G | 98.13 | 0.000000e+00 | \* |
| 305 | 34 | G | G | T | 0.00 | 9.468085e-01 |  |
| 306 | 35 | G | G | A | 0.42 | 9.112504e-01 |  |
| 306 | 35 | G | G | C | 3.39 | 1.016596e-01 |  |
| 306 | 35 | G | G | G | 95.34 | 0.000000e+00 | \* |
| 306 | 35 | G | G | T | 0.85 | 8.642794e-01 |  |
| 307 | 36 | C | C | A | 1.83 | 5.799245e-01 |  |
| 307 | 36 | C | C | C | 94.87 | 0.000000e+00 | \* |
| 307 | 36 | C | C | G | 1.83 | 3.052385e-01 |  |
| 307 | 36 | C | C | T | 1.47 | 6.549575e-01 |  |
| 308 | 37 | A | A | A | 92.25 | 0.000000e+00 | \* |
| 308 | 37 | A | A | C | 5.04 | 1.762792e-02 |  |
| 308 | 37 | A | A | G | 1.16 | 5.099924e-01 |  |
| 308 | 37 | A | A | T | 1.55 | 6.219406e-01 |  |
| 309 | 38 | C | C | A | 1.43 | 6.970474e-01 |  |
| 309 | 38 | C | C | C | 96.56 | 0.000000e+00 | \* |
| 309 | 38 | C | C | G | 0.29 | 8.514783e-01 |  |
| 309 | 38 | C | C | T | 1.72 | 5.566840e-01 |  |
| 310 | 39 | C | C | A | 1.50 | 6.770267e-01 |  |
| 310 | 39 | C | C | C | 96.10 | 0.000000e+00 | \* |
| 310 | 39 | C | C | G | 0.00 | 9.111111e-01 |  |
| 310 | 39 | C | C | T | 2.40 | 3.229306e-01 |  |
| 311 | 40 | C | C | A | 2.11 | 5.006420e-01 |  |
| 311 | 40 | C | C | C | 94.28 | 0.000000e+00 | \* |
| 311 | 40 | C | C | G | 0.90 | 6.092395e-01 |  |
| 311 | 40 | C | C | T | 2.71 | 2.424139e-01 |  |
| 312 | 41 | T | T | A | 1.55 | 6.619910e-01 |  |
| 312 | 41 | T | T | C | 2.80 | 1.797196e-01 |  |
| 312 | 41 | T | T | G | 3.42 | 7.656706e-02 |  |
| 312 | 41 | T | T | T | 92.24 | 0.000000e+00 | \* |
| 313 | 42 | G | G | A | 1.76 | 6.014239e-01 |  |
| 313 | 42 | G | G | C | 1.54 | 4.951263e-01 |  |
| 313 | 42 | G | G | G | 95.16 | 0.000000e+00 | \* |
| 313 | 42 | G | G | T | 1.54 | 6.265708e-01 |  |
| 314 | 43 | A | A | A | 94.97 | 0.000000e+00 | \* |
| 314 | 43 | A | A | C | 2.23 | 2.936455e-01 |  |
| 314 | 43 | A | A | G | 2.79 | 1.343723e-01 |  |
| 314 | 43 | A | A | T | 0.00 | 9.468085e-01 |  |
| 315 | 44 | C | C | A | 2.48 | 4.015281e-01 |  |
| 315 | 44 | C | C | C | 94.10 | 0.000000e+00 | \* |
| 315 | 44 | C | C | G | 1.86 | 2.974353e-01 |  |
| 315 | 44 | C | C | T | 1.55 | 6.210059e-01 |  |
| 316 | 45 | C | C | A | 1.99 | 5.349863e-01 |  |
| 316 | 45 | C | C | C | 93.71 | 0.000000e+00 | \* |
| 316 | 45 | C | C | G | 2.32 | 2.033940e-01 |  |
| 316 | 45 | C | C | T | 1.99 | 4.574311e-01 |  |
| 317 | 46 | G | G | A | 4.49 | 9.367343e-02 |  |
| 317 | 46 | G | G | C | 3.00 | 1.489399e-01 |  |
| 317 | 46 | G | G | G | 92.51 | 0.000000e+00 | \* |
| 317 | 46 | G | G | T | 0.00 | 9.468085e-01 |  |
| 318 | 47 | A | A | A | 95.71 | 0.000000e+00 | \* |
| 318 | 47 | A | A | C | 1.77 | 4.223649e-01 |  |
| 318 | 47 | A | A | G | 1.77 | 3.213893e-01 |  |
| 318 | 47 | A | A | T | 0.76 | 8.857272e-01 |  |
| 319 | 48 | C | C | A | 3.55 | 1.950083e-01 |  |
| 319 | 48 | C | C | C | 93.26 | 0.000000e+00 | \* |
| 319 | 48 | C | C | G | 2.13 | 2.389905e-01 |  |
| 319 | 48 | C | C | T | 1.06 | 8.008930e-01 |  |
| 320 | 49 | G | G | A | 5.08 | 5.801475e-02 |  |
| 320 | 49 | G | G | C | 3.55 | 8.631529e-02 |  |
| 320 | 49 | G | G | G | 90.36 | 0.000000e+00 | \* |
| 320 | 49 | G | G | T | 1.02 | 8.164066e-01 |  |
| 321 | 50 | G | G | A | 2.37 | 4.296805e-01 |  |
| 321 | 50 | G | G | C | 1.69 | 4.449129e-01 |  |
| 321 | 50 | G | G | G | 95.25 | 0.000000e+00 | \* |
| 321 | 50 | G | G | T | 0.68 | 9.019852e-01 |  |
| 322 | 51 | C | C | A | 2.53 | 3.915301e-01 |  |
| 322 | 51 | C | C | C | 92.42 | 0.000000e+00 | \* |
| 322 | 51 | C | C | G | 3.54 | 6.861488e-02 |  |
| 322 | 51 | C | C | T | 1.52 | 6.356165e-01 |  |
| 323 | 52 | A | A | A | 93.51 | 0.000000e+00 | \* |
| 323 | 52 | A | A | C | 2.60 | 2.149354e-01 |  |
| 323 | 52 | A | A | G | 1.95 | 2.775008e-01 |  |
| 323 | 52 | A | A | T | 1.95 | 4.712977e-01 |  |
| 324 | 53 | A | A | A | 96.34 | 0.000000e+00 | \* |
| 324 | 53 | A | A | C | 0.37 | 8.202446e-01 |  |
| 324 | 53 | A | A | G | 1.47 | 4.076044e-01 |  |
| 324 | 53 | A | A | T | 1.83 | 5.141446e-01 |  |
| 325 | 54 | G | G | A | 6.35 | 1.920303e-02 |  |
| 325 | 54 | G | G | C | 1.59 | 4.792287e-01 |  |
| 325 | 54 | G | G | G | 89.42 | 0.000000e+00 | \* |
| 325 | 54 | G | G | T | 2.65 | 2.580616e-01 |  |
| 326 | 55 | A | A | A | 97.22 | 0.000000e+00 | \* |
| 326 | 55 | A | A | C | 0.00 | 8.372093e-01 |  |
| 326 | 55 | A | A | G | 1.23 | 4.842695e-01 |  |
| 326 | 55 | A | A | T | 1.54 | 6.247274e-01 |  |
| 327 | 56 | A | A | A | 96.64 | 0.000000e+00 | \* |
| 327 | 56 | A | A | C | 0.00 | 8.372093e-01 |  |
| 327 | 56 | A | A | G | 1.12 | 5.259435e-01 |  |
| 327 | 56 | A | A | T | 2.24 | 3.724756e-01 |  |
| 328 | 57 | G | G | A | 4.27 | 1.122269e-01 |  |
| 328 | 57 | G | G | C | 0.61 | 7.793695e-01 |  |
| 328 | 57 | G | G | G | 90.24 | 0.000000e+00 | \* |
| 328 | 57 | G | G | T | 4.88 | 2.076734e-02 |  |
| 329 | 58 | T | T | A | 2.26 | 4.581764e-01 |  |
| 329 | 58 | T | T | C | 0.38 | 8.189284e-01 |  |
| 329 | 58 | T | T | G | 0.75 | 6.695566e-01 |  |
| 329 | 58 | T | T | T | 96.60 | 0.000000e+00 | \* |
| 330 | 59 | T | T | A | 1.05 | 8.004788e-01 |  |
| 330 | 59 | T | T | C | 2.46 | 2.433575e-01 |  |
| 330 | 59 | T | T | G | 1.05 | 5.510504e-01 |  |
| 330 | 59 | T | T | T | 95.44 | 0.000000e+00 | \* |
| 331 | 60 | C | C | A | 2.11 | 4.994616e-01 |  |
| 331 | 60 | C | C | C | 93.31 | 0.000000e+00 | \* |
| 331 | 60 | C | C | G | 1.06 | 5.496397e-01 |  |
| 331 | 60 | C | C | T | 3.52 | 1.043581e-01 |  |
| 332 | 61 | G | G | A | 8.67 | 2.225329e-03 | \* |
| 332 | 61 | G | G | C | 3.47 | 9.402102e-02 |  |
| 332 | 61 | G | G | G | 84.97 | 0.000000e+00 | \* |
| 332 | 61 | G | G | T | 2.89 | 2.032420e-01 |  |
| 333 | 62 | A | A | A | 96.53 | 0.000000e+00 | \* |
| 333 | 62 | A | A | C | 2.31 | 2.752445e-01 |  |
| 333 | 62 | A | A | G | 1.16 | 5.124440e-01 |  |
| 333 | 62 | A | A | T | 0.00 | 9.468085e-01 |  |
| 334 | 63 | C | C | A | 3.67 | 1.783419e-01 |  |
| 334 | 63 | C | C | C | 93.33 | 0.000000e+00 | \* |
| 334 | 63 | C | C | G | 0.33 | 8.356716e-01 |  |
| 334 | 63 | C | C | T | 2.67 | 2.529113e-01 |  |
| 335 | 64 | A | A | A | 93.38 | 0.000000e+00 | \* |
| 335 | 64 | A | A | C | 2.44 | 2.469919e-01 |  |
| 335 | 64 | A | A | G | 2.79 | 1.350699e-01 |  |
| 335 | 64 | A | A | T | 1.39 | 6.824312e-01 |  |
| 336 | 65 | G | G | A | 3.21 | 2.479001e-01 |  |
| 336 | 65 | G | G | C | 2.81 | 1.770558e-01 |  |
| 336 | 65 | G | G | G | 91.97 | 0.000000e+00 | \* |
| 336 | 65 | G | G | T | 2.01 | 4.498929e-01 |  |
| 337 | 66 | C | C | A | 2.38 | 4.276062e-01 |  |
| 337 | 66 | C | C | C | 91.67 | 0.000000e+00 | \* |
| 337 | 66 | C | C | G | 1.59 | 3.708479e-01 |  |
| 337 | 66 | C | C | T | 4.37 | 3.914615e-02 |  |
| 338 | 67 | T | T | A | 0.96 | 8.237499e-01 |  |
| 338 | 67 | T | T | C | 2.87 | 1.682651e-01 |  |
| 338 | 67 | T | T | G | 0.32 | 8.407995e-01 |  |
| 338 | 67 | T | T | T | 95.86 | 0.000000e+00 | \* |
| 339 | 68 | C | C | A | 1.69 | 6.217793e-01 |  |
| 339 | 68 | C | C | C | 94.59 | 0.000000e+00 | \* |
| 339 | 68 | C | C | G | 0.68 | 7.019816e-01 |  |
| 339 | 68 | C | C | T | 3.04 | 1.744728e-01 |  |
| 340 | 69 | C | C | A | 1.53 | 6.689607e-01 |  |
| 340 | 69 | C | C | C | 95.41 | 0.000000e+00 | \* |
| 340 | 69 | C | C | G | 0.92 | 6.037351e-01 |  |
| 340 | 69 | C | C | T | 2.14 | 4.043763e-01 |  |
| 341 | 70 | C | C | A | 2.50 | 3.977025e-01 |  |
| 341 | 70 | C | C | C | 93.33 | 0.000000e+00 | \* |
| 341 | 70 | C | C | G | 1.67 | 3.483823e-01 |  |
| 341 | 70 | C | C | T | 2.50 | 2.956207e-01 |  |
| 342 | 71 | G | G | A | 5.09 | 5.721863e-02 |  |
| 342 | 71 | G | G | C | 3.70 | 7.407916e-02 |  |
| 342 | 71 | G | G | G | 89.81 | 0.000000e+00 | \* |
| 342 | 71 | G | G | T | 1.39 | 6.842790e-01 |  |
| 343 | 72 | C | C | A | 0.00 | 9.259259e-01 |  |
| 343 | 72 | C | C | C | 95.82 | 0.000000e+00 | \* |
| 343 | 72 | C | C | G | 1.67 | 3.464615e-01 |  |
| 343 | 72 | C | C | T | 2.51 | 2.927946e-01 |  |
| 344 | 73 | G | G | A | 8.33 | 3.069224e-03 | \* |
| 344 | 73 | G | G | C | 2.45 | 2.444488e-01 |  |
| 344 | 73 | G | G | G | 89.22 | 0.000000e+00 | \* |
| 344 | 73 | G | G | T | 0.00 | 9.468085e-01 |  |
| 345 | 74 | A | A | A | 95.82 | 0.000000e+00 | \* |
| 345 | 74 | A | A | C | 2.57 | 2.197741e-01 |  |
| 345 | 74 | A | A | G | 1.61 | 3.649622e-01 |  |
| 345 | 74 | A | A | T | 0.00 | 9.468085e-01 |  |
| 346 | 75 | C | C | A | 2.83 | 3.226642e-01 |  |
| 346 | 75 | C | C | C | 95.41 | 0.000000e+00 | \* |
| 346 | 75 | C | C | G | 0.00 | 9.111111e-01 |  |
| 346 | 75 | C | C | T | 1.77 | 5.385353e-01 |  |

## For use in R

If you want to work with the results in R, here is output that you can copy and paste in your terminal to get:

The base information:

```
structure(list(focal.base = c("A", "C", "G", "T"), avg.percsignal = c(95.0561375114258, 
94.1208298159129, 93.2694251637497, 94.7190151491431), avg.areasignal = c(318.416666666667, 
340.230769230769, 299.105263157895, 354.651162790698), crit.perc.area = c(7.06929868603268, 
5.53938067021095, 5.55618723323177, 5.4479166442578), mu = c(2.52763559132414, 
2.00511130367266, 1.60970437557467, 2.12941863103471), fillibens = c(0.990188916950413, 
0.983505029312576, 0.99236419100345, 0.995137484137734)), .Names = c("focal.base", 
"avg.percsignal", "avg.areasignal", "crit.perc.area", "mu", "fillibens"
), row.names = c(NA, -4L), class = "data.frame")
```

the data.frame that contains information on the guide region:

```
structure(list(A.area = c(299, 331, 14, 611, 15, 17, 14, 12, 
9, 11, 279, 13, 551, 15, 13, 14, 0, 8, 5, 6, 1, 4, 4, 4, 5, 224, 
7, 11, 326, 7, 234, 5, 7, 0, 1, 5, 238, 5, 5, 7, 5, 8, 340, 8, 
6, 12, 379, 10, 10, 7, 5, 144, 263, 12, 315, 259, 7, 6, 3, 6, 
15, 334, 11, 268, 8, 6, 3, 5, 5, 6, 11, 0, 17, 298, 8), C.area = c(9, 
4, 4, 8, 342, 8, 4, 6, 234, 377, 7, 5, 8, 351, 297, 6, 2, 0, 
0, 0, 2, 0, 4, 6, 300, 10, 278, 7, 8, 306, 8, 289, 8, 6, 8, 259, 
13, 337, 320, 313, 9, 7, 8, 303, 283, 8, 7, 263, 7, 5, 183, 4, 
1, 3, 0, 0, 1, 1, 7, 265, 6, 8, 280, 7, 7, 231, 9, 280, 312, 
224, 8, 229, 5, 8, 270), G.area = c(4, 7, 223, 6, 6, 14, 592, 
313, 7, 0, 6, 205, 6, 2, 8, 312, 8, 478, 11, 9, 12, 387, 4, 340, 
7, 5, 34, 0, 3, 2, 9, 9, 234, 315, 225, 5, 3, 1, 0, 3, 11, 433, 
10, 6, 7, 247, 7, 6, 178, 281, 7, 3, 4, 169, 4, 3, 148, 2, 3, 
3, 147, 4, 1, 8, 229, 4, 1, 2, 3, 4, 194, 4, 182, 5, 0), T.area = c(3, 
0, 3, 6, 9, 332, 7, 3, 2, 4, 0, 0, 5, 6, 5, 10, 239, 8, 278, 
323, 331, 7, 282, 6, 7, 5, 8, 296, 6, 5, 0, 3, 3, 0, 2, 4, 4, 
6, 8, 9, 297, 7, 0, 5, 6, 0, 3, 3, 2, 2, 3, 3, 5, 5, 5, 6, 8, 
256, 272, 10, 5, 0, 8, 4, 5, 11, 301, 9, 7, 6, 3, 6, 0, 0, 5), 
    Tot.area = c(315, 342, 244, 631, 372, 371, 617, 334, 252, 
    392, 292, 223, 570, 374, 323, 342, 249, 494, 294, 338, 346, 
    398, 294, 356, 319, 244, 327, 314, 343, 320, 251, 306, 252, 
    321, 236, 273, 258, 349, 333, 332, 322, 455, 358, 322, 302, 
    267, 396, 282, 197, 295, 198, 154, 273, 189, 324, 268, 164, 
    265, 285, 284, 173, 346, 300, 287, 249, 252, 314, 296, 327, 
    240, 216, 239, 204, 311, 283), A.perc = c(94.9206349206349, 
    96.7836257309941, 5.73770491803279, 96.8304278922346, 4.03225806451613, 
    4.5822102425876, 2.26904376012966, 3.59281437125748, 3.57142857142857, 
    2.80612244897959, 95.5479452054795, 5.82959641255605, 96.6666666666667, 
    4.01069518716578, 4.02476780185759, 4.09356725146199, 0, 
    1.61943319838057, 1.70068027210884, 1.77514792899408, 0.289017341040462, 
    1.00502512562814, 1.36054421768707, 1.12359550561798, 1.56739811912226, 
    91.8032786885246, 2.14067278287462, 3.5031847133758, 95.0437317784257, 
    2.1875, 93.2270916334661, 1.63398692810458, 2.77777777777778, 
    0, 0.423728813559322, 1.83150183150183, 92.2480620155039, 
    1.43266475644699, 1.5015015015015, 2.10843373493976, 1.5527950310559, 
    1.75824175824176, 94.9720670391062, 2.48447204968944, 1.98675496688742, 
    4.49438202247191, 95.7070707070707, 3.54609929078014, 5.0761421319797, 
    2.3728813559322, 2.52525252525253, 93.5064935064935, 96.3369963369963, 
    6.34920634920635, 97.2222222222222, 96.6417910447761, 4.26829268292683, 
    2.26415094339623, 1.05263157894737, 2.11267605633803, 8.67052023121387, 
    96.5317919075144, 3.66666666666667, 93.3797909407666, 3.21285140562249, 
    2.38095238095238, 0.955414012738854, 1.68918918918919, 1.52905198776758, 
    2.5, 5.09259259259259, 0, 8.33333333333333, 95.8199356913183, 
    2.82685512367491), C.perc = c(2.85714285714286, 1.16959064327485, 
    1.63934426229508, 1.26782884310618, 91.9354838709677, 2.15633423180593, 
    0.648298217179903, 1.79640718562874, 92.8571428571429, 96.1734693877551, 
    2.3972602739726, 2.24215246636771, 1.40350877192982, 93.8502673796791, 
    91.9504643962848, 1.75438596491228, 0.803212851405622, 0, 
    0, 0, 0.578034682080925, 0, 1.36054421768707, 1.68539325842697, 
    94.0438871473354, 4.0983606557377, 85.0152905198777, 2.22929936305732, 
    2.33236151603499, 95.625, 3.18725099601594, 94.4444444444444, 
    3.17460317460317, 1.86915887850467, 3.38983050847458, 94.8717948717949, 
    5.03875968992248, 96.5616045845272, 96.0960960960961, 94.2771084337349, 
    2.79503105590062, 1.53846153846154, 2.23463687150838, 94.0993788819876, 
    93.7086092715232, 2.99625468164794, 1.76767676767677, 93.2624113475177, 
    3.55329949238579, 1.69491525423729, 92.4242424242424, 2.5974025974026, 
    0.366300366300366, 1.58730158730159, 0, 0, 0.609756097560976, 
    0.377358490566038, 2.45614035087719, 93.3098591549296, 3.46820809248555, 
    2.3121387283237, 93.3333333333333, 2.4390243902439, 2.81124497991968, 
    91.6666666666667, 2.86624203821656, 94.5945945945946, 95.4128440366973, 
    93.3333333333333, 3.7037037037037, 95.81589958159, 2.45098039215686, 
    2.57234726688103, 95.4063604240283), G.perc = c(1.26984126984127, 
    2.04678362573099, 91.3934426229508, 0.950871632329635, 1.61290322580645, 
    3.77358490566038, 95.9481361426256, 93.7125748502994, 2.77777777777778, 
    0, 2.05479452054795, 91.9282511210762, 1.05263157894737, 
    0.53475935828877, 2.47678018575851, 91.2280701754386, 3.21285140562249, 
    96.7611336032389, 3.74149659863946, 2.66272189349112, 3.46820809248555, 
    97.2361809045226, 1.36054421768707, 95.5056179775281, 2.19435736677116, 
    2.04918032786885, 10.3975535168196, 0, 0.87463556851312, 
    0.625, 3.58565737051793, 2.94117647058824, 92.8571428571429, 
    98.1308411214953, 95.3389830508475, 1.83150183150183, 1.16279069767442, 
    0.286532951289398, 0, 0.903614457831325, 3.41614906832298, 
    95.1648351648352, 2.79329608938547, 1.86335403726708, 2.31788079470199, 
    92.5093632958801, 1.76767676767677, 2.12765957446809, 90.3553299492386, 
    95.2542372881356, 3.53535353535354, 1.94805194805195, 1.46520146520147, 
    89.4179894179894, 1.23456790123457, 1.11940298507463, 90.2439024390244, 
    0.754716981132076, 1.05263157894737, 1.05633802816901, 84.971098265896, 
    1.15606936416185, 0.333333333333333, 2.78745644599303, 91.9678714859438, 
    1.58730158730159, 0.318471337579618, 0.675675675675676, 0.917431192660551, 
    1.66666666666667, 89.8148148148148, 1.67364016736402, 89.2156862745098, 
    1.60771704180064, 0), T.perc = c(0.952380952380952, 0, 1.22950819672131, 
    0.950871632329635, 2.41935483870968, 89.4878706199461, 1.13452188006483, 
    0.898203592814371, 0.793650793650794, 1.02040816326531, 0, 
    0, 0.87719298245614, 1.60427807486631, 1.54798761609907, 
    2.92397660818713, 95.9839357429719, 1.61943319838057, 94.5578231292517, 
    95.5621301775148, 95.6647398843931, 1.75879396984925, 95.9183673469388, 
    1.68539325842697, 2.19435736677116, 2.04918032786885, 2.44648318042813, 
    94.2675159235669, 1.74927113702624, 1.5625, 0, 0.980392156862745, 
    1.19047619047619, 0, 0.847457627118644, 1.46520146520147, 
    1.55038759689922, 1.71919770773639, 2.4024024024024, 2.71084337349398, 
    92.2360248447205, 1.53846153846154, 0, 1.5527950310559, 1.98675496688742, 
    0, 0.757575757575758, 1.06382978723404, 1.01522842639594, 
    0.677966101694915, 1.51515151515152, 1.94805194805195, 1.83150183150183, 
    2.64550264550265, 1.54320987654321, 2.23880597014925, 4.8780487804878, 
    96.6037735849057, 95.4385964912281, 3.52112676056338, 2.89017341040462, 
    0, 2.66666666666667, 1.39372822299652, 2.00803212851406, 
    4.36507936507936, 95.859872611465, 3.04054054054054, 2.14067278287462, 
    2.5, 1.38888888888889, 2.51046025104602, 0, 0, 1.76678445229682
    ), base.call = c("A", "A", "G", "A", "C", "T", "G", "G", 
    "C", "C", "A", "G", "A", "C", "C", "G", "T", "G", "T", "T", 
    "T", "G", "T", "G", "C", "A", "C", "T", "A", "C", "A", "C", 
    "G", "G", "G", "C", "A", "C", "C", "C", "T", "G", "A", "C", 
    "C", "G", "A", "C", "G", "G", "C", "A", "A", "G", "A", "A", 
    "G", "T", "T", "C", "G", "A", "C", "A", "G", "C", "T", "C", 
    "C", "C", "G", "C", "G", "A", "C"), index = 272:346, guide.seq = c("A", 
    "A", "G", "A", "C", "T", "G", "G", "C", "C", "A", "G", "A", 
    "C", "C", "G", "T", "G", "T", "T", "T", "G", "T", "G", "C", 
    "A", "C", "T", "A", "C", "A", "C", "G", "G", "G", "C", "A", 
    "C", "C", "C", "T", "G", "A", "C", "C", "G", "A", "C", "G", 
    "G", "C", "A", "A", "G", "A", "A", "G", "T", "T", "C", "G", 
    "A", "C", "A", "G", "C", "T", "C", "C", "C", "G", "C", "G", 
    "A", "C"), T.pval = c(0.835436187701219, 0.946808510638291, 
    0.743708810823606, 0.835877896719297, 0.31806513585472, 0, 
    0.777229661681375, 0.850813211404736, 0.877501243252904, 
    0.814784962925617, 0.946808510638291, 0.946808510638291, 
    0.856502289824075, 0.601023121222658, 0.622872478335692, 
    0.196459793795817, 0, 0.595148131017574, 0, 0, 0, 0.541571192872433, 
    0, 0.569664941040605, 0.386728827181726, 0.435493862954168, 
    0.310385498143835, 0, 0.54519566857538, 0.617237650038394, 
    0.946808510638291, 0.827105809800517, 0.757696001549986, 
    0.946808510638291, 0.864279378740382, 0.6549575194991, 0.621940647166076, 
    0.556683968310662, 0.322930603400904, 0.242413868928457, 
    0, 0.62657082627577, 0.946808510638291, 0.621005903255626, 
    0.45743110953499, 0.946808510638291, 0.885727230632922, 0.800892959277505, 
    0.816406630533372, 0.901985243613442, 0.63561650711358, 0.471297670500653, 
    0.514144630284943, 0.258061573958908, 0.624727432567853, 
    0.37247562525571, 0.0207673356338303, 0, 0, 0.10435806665909, 
    0.20324203121163, 0.946808510638291, 0.252911255350205, 0.682431204516033, 
    0.449892888248233, 0.039146147227698, 0, 0.174472757732208, 
    0.404376266908281, 0.29562072726571, 0.684278969972546, 0.292794638148032, 
    0.946808510638291, 0.946808510638291, 0.538535250515747), 
    C.pval = c(0.169693680735275, 0.618071774084036, 0.462500602273462, 
    0.585295641030649, 0, 0.313119823666578, 0.770781019080473, 
    0.413624036707602, 0, 0, 0.25603563068399, 0.29182299149818, 
    0.539815506591295, 0, 0, 0.426440408065276, 0.731474173892941, 
    0.837209302325581, 0.837209302325581, 0.837209302325581, 
    0.786037396714608, 0.837209302325581, 0.554196094295863, 
    0.447905010222828, 0, 0.049126960757597, 0, 0.2949448309126, 
    0.270586168127396, 0, 0.124021089440676, 0, 0.125550909619056, 
    0.391930958635026, 0.101659552531688, 0, 0.0176279198681649, 
    0, 0, 0, 0.179719570650235, 0.49512631664552, 0.293645518715575, 
    0, 0, 0.148939900228015, 0.422364917170912, 0, 0.0863152876176689, 
    0.444912899541285, 0, 0.214935387531157, 0.820244629543957, 
    0.47922865494288, 0.837209302325581, 0.837209302325581, 0.779369524851677, 
    0.818928371001127, 0.24335751528071, 0, 0.0940210199783518, 
    0.275244458265248, 0, 0.246991927280766, 0.17705577376575, 
    0, 0.168265143784853, 0, 0, 0, 0.074079155754177, 0, 0.244448795599188, 
    0.219774085592282, 0), G.pval = c(0.471943481627697, 0.255715584066088, 
    0, 0.59049732959161, 0.363478837918941, 0.0550203961339819, 
    0, 0, 0.136233577173292, 0.91111111108653, 0.254014672643866, 
    0, 0.551050389328099, 0.759313067608944, 0.177378224589459, 
    0, 0.0921846743947751, 0, 0.0566882741777509, 0.150795343861546, 
    0.0729918922982881, 0, 0.44123358912883, 0, 0.225926936049506, 
    0.255205671495999, 7.63181771662458e-05, 0.91111111108653, 
    0.620845248210333, 0.722743028752276, 0.0655009088824736, 
    0.117782585340343, 0, 0, 0, 0.305238494462057, 0.50999236148814, 
    0.851478263118961, 0.91111111108653, 0.609239508826964, 0.0765670614719102, 
    0, 0.134372254051545, 0.297435337042927, 0.203393995553148, 
    0, 0.321389317075556, 0.238990533353856, 0, 0, 0.0686148792573712, 
    0.277500781575824, 0.407604408063446, 0, 0.484269465957443, 
    0.525943485570827, 0, 0.669556606217084, 0.551050389328099, 
    0.549639689712338, 0, 0.512443992054675, 0.835671596099155, 
    0.13506989944735, 0, 0.370847875761379, 0.840799521183446, 
    0.701981649251374, 0.603735103603006, 0.348382295429703, 
    0, 0.346461543372635, 0, 0.364962181878601, 0.91111111108653
    ), A.pval = c(0, 0, 0.0329420577764852, 0, 0.1350279822077, 
    0.0872467101651395, 0.456872659524233, 0.188401804529505, 
    0.19140253587243, 0.327105690196773, 0, 0.0304045980786647, 
    0, 0.13730150824797, 0.135813964117744, 0.128743310362779, 
    0.925925925925926, 0.642366173618814, 0.618388507184139, 
    0.596451253323954, 0.920558581759597, 0.812089441139322, 
    0.717746721011894, 0.782480849930967, 0.657697302043168, 
    0, 0.491702837841459, 0.201246762290346, 0, 0.478851355534063, 
    0, 0.638072336817445, 0.333248483265274, 0.925925925925926, 
    0.911250381623294, 0.579924482462305, 0, 0.697047389306221, 
    0.677026693035306, 0.500642008284933, 0.661990999536546, 
    0.601423924594036, 0, 0.401528074394061, 0.534986327045799, 
    0.0936734294502113, 0, 0.195008288681209, 0.0580147463163325, 
    0.429680478382914, 0.39153008215388, 0, 0, 0.0192030315885481, 
    0, 0, 0.112226917621625, 0.458176435283647, 0.800478824727755, 
    0.499461596981896, 0.00222532859513813, 0, 0.178341893340112, 
    0, 0.247900135943812, 0.427606222772908, 0.823749906258951, 
    0.621779284737825, 0.66896069279403, 0.397702475778511, 0.0572186262870604, 
    0.925925925925926, 0.0030692240735759, 0, 0.322664156205741
    ), guide.position = 1:75), .Names = c("A.area", "C.area", 
"G.area", "T.area", "Tot.area", "A.perc", "C.perc", "G.perc", 
"T.perc", "base.call", "index", "guide.seq", "T.pval", "C.pval", 
"G.pval", "A.pval", "guide.position"), row.names = 272:346, class = "data.frame")
```

*Report generated using EditR v1.0.8*
