# Supplementary material for: Mechanistic and genetic basis of single-strand templated repair at Cas12a-induced DNA breaks in Chlamydomonas reinhardtii
Source: Nat Commun. 2021 Nov 19;12:6751. doi: 10.1038/s41467-021-27004-1 (PMC8604939; doi:10.1038/s41467-021-27004-1)
Supplement: Supplementary file 22 — Source Data [file 41467_2021_27004_MOESM22_ESM.zip › Source Data/EditR analysis/EditR outputs/Sense/rep3_ssODN_sense_32.html]

EditR v1.0.8 report


# EditR v1.0.8 report

- Data QA
  - Filtering data
  - Percent noise peak area
  - Base information
- Predicted editing
  - Editing bar plot
  - Editing table plot
  - Table of editing results
- For use in R

## Data QA

### Filtering data

What the data looked like prefiltering:

and the post filtering signal / noise plot:

### Percent noise peak area

### Base information

Here’s information about the signal of each base, the critical percent value where any higher value would be called as significant, and Filliben’s correlation for how well the noise was modelled by the zero adjusted gamma distribution.

| Base | Average percent signal | Average peak area | Critical percent value | model mu | Fillibens correlation |
| --- | --- | --- | --- | --- | --- |
| A | 89.07623 | 75.76471 | 15.217815 | 5.465169 | 0.9947634 |
| C | 86.76939 | 83.67647 | 9.609877 | 3.739458 | 0.9903256 |
| G | 89.79785 | 75.66071 | 15.016266 | 4.974620 | 0.9971955 |
| T | 90.57268 | 90.76744 | 10.696162 | 3.842231 | 0.9870057 |

## Predicted editing

### Editing bar plot

### Editing table plot

### Table of editing results


Here’s the entire guide region

| Sanger position | Guide position | Guide sequence | Sanger base call | Focal base | Focal base peak area | p value |  |
| --- | --- | --- | --- | --- | --- | --- | --- |
| 278 | 1 | A | A | A | 91.0 | 0.000000e+00 | \* |
| 278 | 1 | A | A | C | 9.0 | 1.565149e-02 |  |
| 278 | 1 | A | A | G | 0.0 | 8.241758e-01 |  |
| 278 | 1 | A | A | T | 0.0 | 7.340426e-01 |  |
| 279 | 2 | A | A | A | 93.7 | 0.000000e+00 | \* |
| 279 | 2 | A | A | C | 3.8 | 3.265133e-01 |  |
| 279 | 2 | A | A | G | 0.0 | 8.241758e-01 |  |
| 279 | 2 | A | A | T | 2.5 | 4.890217e-01 |  |
| 280 | 3 | G | G | A | 5.2 | 3.691090e-01 |  |
| 280 | 3 | G | G | C | 12.1 | 1.732859e-03 | \* |
| 280 | 3 | G | G | G | 82.8 | 1.776357e-15 | \* |
| 280 | 3 | G | G | T | 0.0 | 7.340426e-01 |  |
| 281 | 4 | A | A | A | 92.7 | 0.000000e+00 | \* |
| 281 | 4 | A | A | C | 1.3 | 7.248167e-01 |  |
| 281 | 4 | A | A | G | 2.0 | 6.888039e-01 |  |
| 281 | 4 | A | A | T | 4.0 | 2.918753e-01 |  |
| 282 | 5 | C | C | A | 14.1 | 1.562349e-02 |  |
| 282 | 5 | C | C | C | 83.5 | 0.000000e+00 | \* |
| 282 | 5 | C | C | G | 0.0 | 8.241758e-01 |  |
| 282 | 5 | C | C | T | 2.4 | 5.158596e-01 |  |
| 283 | 6 | T | T | A | 0.0 | 8.090909e-01 |  |
| 283 | 6 | T | T | C | 3.8 | 3.193405e-01 |  |
| 283 | 6 | T | T | G | 3.8 | 4.611636e-01 |  |
| 283 | 6 | T | T | T | 92.3 | 0.000000e+00 | \* |
| 284 | 7 | G | G | A | 6.3 | 2.640540e-01 |  |
| 284 | 7 | G | G | C | 1.3 | 7.311820e-01 |  |
| 284 | 7 | G | G | G | 91.1 | 0.000000e+00 | \* |
| 284 | 7 | G | G | T | 1.3 | 6.655616e-01 |  |
| 285 | 8 | G | G | A | 7.3 | 1.950681e-01 |  |
| 285 | 8 | G | G | C | 3.1 | 4.345964e-01 |  |
| 285 | 8 | G | G | G | 89.6 | 2.220446e-16 | \* |
| 285 | 8 | G | G | T | 0.0 | 7.340426e-01 |  |
| 286 | 9 | C | C | A | 8.6 | 1.269539e-01 |  |
| 286 | 9 | C | C | C | 80.0 | 0.000000e+00 | \* |
| 286 | 9 | C | C | G | 7.1 | 1.747080e-01 |  |
| 286 | 9 | C | C | T | 4.3 | 2.568982e-01 |  |
| 287 | 10 | C | C | A | 7.1 | 2.046712e-01 |  |
| 287 | 10 | C | C | C | 82.7 | 0.000000e+00 | \* |
| 287 | 10 | C | C | G | 7.1 | 1.747080e-01 |  |
| 287 | 10 | C | C | T | 3.1 | 4.111429e-01 |  |
| 288 | 11 | A | A | A | 85.5 | 0.000000e+00 | \* |
| 288 | 11 | A | A | C | 1.4 | 7.101370e-01 |  |
| 288 | 11 | A | A | G | 13.0 | 2.132618e-02 |  |
| 288 | 11 | A | A | T | 0.0 | 7.340426e-01 |  |
| 289 | 12 | G | G | A | 9.4 | 9.569047e-02 |  |
| 289 | 12 | G | G | C | 0.0 | 7.831325e-01 |  |
| 289 | 12 | G | G | G | 90.6 | 2.220446e-16 | \* |
| 289 | 12 | G | G | T | 0.0 | 7.340426e-01 |  |
| 290 | 13 | A | A | A | 91.1 | 0.000000e+00 | \* |
| 290 | 13 | A | A | C | 1.9 | 6.444268e-01 |  |
| 290 | 13 | A | A | G | 2.5 | 6.207382e-01 |  |
| 290 | 13 | A | A | T | 4.5 | 2.388985e-01 |  |
| 291 | 14 | C | C | A | 14.3 | 1.460138e-02 |  |
| 291 | 14 | C | C | C | 84.6 | 0.000000e+00 | \* |
| 291 | 14 | C | C | G | 0.0 | 8.241758e-01 |  |
| 291 | 14 | C | C | T | 1.1 | 6.833049e-01 |  |
| 292 | 15 | C | C | A | 11.3 | 4.763279e-02 |  |
| 292 | 15 | C | C | C | 81.7 | 0.000000e+00 | \* |
| 292 | 15 | C | C | G | 7.0 | 1.805128e-01 |  |
| 292 | 15 | C | C | T | 0.0 | 7.340426e-01 |  |
| 293 | 16 | G | G | A | 8.3 | 1.377970e-01 |  |
| 293 | 16 | G | G | C | 6.0 | 1.053525e-01 |  |
| 293 | 16 | G | G | G | 84.5 | 8.881784e-16 | \* |
| 293 | 16 | G | G | T | 1.2 | 6.738383e-01 |  |
| 294 | 17 | T | T | A | 0.0 | 8.090909e-01 |  |
| 294 | 17 | T | T | C | 1.6 | 6.955582e-01 |  |
| 294 | 17 | T | T | G | 7.8 | 1.400421e-01 |  |
| 294 | 17 | T | T | T | 90.6 | 0.000000e+00 | \* |
| 295 | 18 | G | G | A | 4.5 | 4.392779e-01 |  |
| 295 | 18 | G | G | C | 1.5 | 7.032640e-01 |  |
| 295 | 18 | G | G | G | 91.7 | 0.000000e+00 | \* |
| 295 | 18 | G | G | T | 2.3 | 5.304506e-01 |  |
| 296 | 19 | T | T | A | 0.0 | 8.090909e-01 |  |
| 296 | 19 | T | T | C | 2.7 | 5.019310e-01 |  |
| 296 | 19 | T | T | G | 9.6 | 7.588843e-02 |  |
| 296 | 19 | T | T | T | 87.7 | 0.000000e+00 | \* |
| 297 | 20 | T | T | A | 0.0 | 8.090909e-01 |  |
| 297 | 20 | T | T | C | 0.0 | 7.831325e-01 |  |
| 297 | 20 | T | T | G | 5.2 | 3.191233e-01 |  |
| 297 | 20 | T | T | T | 94.8 | 0.000000e+00 | \* |
| 298 | 21 | T | T | A | 0.0 | 8.090909e-01 |  |
| 298 | 21 | T | T | C | 3.8 | 3.265133e-01 |  |
| 298 | 21 | T | T | G | 7.6 | 1.505753e-01 |  |
| 298 | 21 | T | T | T | 88.6 | 0.000000e+00 | \* |
| 299 | 22 | G | G | A | 2.2 | 7.062098e-01 |  |
| 299 | 22 | G | G | C | 2.2 | 6.051113e-01 |  |
| 299 | 22 | G | G | G | 91.4 | 0.000000e+00 | \* |
| 299 | 22 | G | G | T | 4.3 | 2.552594e-01 |  |
| 300 | 23 | T | T | A | 0.0 | 8.090909e-01 |  |
| 300 | 23 | T | T | C | 1.6 | 6.955582e-01 |  |
| 300 | 23 | T | T | G | 6.2 | 2.322214e-01 |  |
| 300 | 23 | T | T | T | 92.2 | 0.000000e+00 | \* |
| 301 | 24 | G | G | A | 3.8 | 5.152762e-01 |  |
| 301 | 24 | G | G | C | 1.3 | 7.294572e-01 |  |
| 301 | 24 | G | G | G | 89.7 | 2.220446e-16 | \* |
| 301 | 24 | G | G | T | 5.1 | 1.782098e-01 |  |
| 302 | 25 | C | C | A | 3.0 | 6.109174e-01 |  |
| 302 | 25 | C | C | C | 87.9 | 0.000000e+00 | \* |
| 302 | 25 | C | C | G | 4.5 | 3.833734e-01 |  |
| 302 | 25 | C | C | T | 4.5 | 2.302230e-01 |  |
| 303 | 26 | A | A | A | 87.5 | 0.000000e+00 | \* |
| 303 | 26 | A | A | C | 3.6 | 3.610645e-01 |  |
| 303 | 26 | A | A | G | 5.4 | 3.043446e-01 |  |
| 303 | 26 | A | A | T | 3.6 | 3.415034e-01 |  |
| 304 | 27 | C | C | A | 8.2 | 1.432746e-01 |  |
| 304 | 27 | C | C | C | 89.0 | 0.000000e+00 | \* |
| 304 | 27 | C | C | G | 1.4 | 7.549602e-01 |  |
| 304 | 27 | C | C | T | 1.4 | 6.534703e-01 |  |
| 305 | 28 | T | T | A | 2.8 | 6.353271e-01 |  |
| 305 | 28 | T | T | C | 9.9 | 8.412166e-03 | \* |
| 305 | 28 | T | T | G | 1.4 | 7.512450e-01 |  |
| 305 | 28 | T | T | T | 85.9 | 0.000000e+00 | \* |
| 306 | 29 | A | A | A | 93.0 | 0.000000e+00 | \* |
| 306 | 29 | A | A | C | 2.8 | 4.882807e-01 |  |
| 306 | 29 | A | A | G | 2.8 | 5.870836e-01 |  |
| 306 | 29 | A | A | T | 1.4 | 6.488050e-01 |  |
| 307 | 30 | C | C | A | 7.7 | 1.710411e-01 |  |
| 307 | 30 | C | C | C | 90.8 | 0.000000e+00 | \* |
| 307 | 30 | C | C | G | 1.5 | 7.382453e-01 |  |
| 307 | 30 | C | C | T | 0.0 | 7.340426e-01 |  |
| 308 | 31 | A | A | A | 75.9 | 4.662937e-15 | \* |
| 308 | 31 | A | A | C | 10.3 | 5.985918e-03 | \* |
| 308 | 31 | A | A | G | 10.3 | 5.792709e-02 |  |
| 308 | 31 | A | A | T | 3.4 | 3.576798e-01 |  |
| 309 | 32 | C | C | A | 3.2 | 5.881688e-01 |  |
| 309 | 32 | C | C | C | 87.1 | 0.000000e+00 | \* |
| 309 | 32 | C | C | G | 6.5 | 2.180153e-01 |  |
| 309 | 32 | C | C | T | 3.2 | 3.879565e-01 |  |
| 310 | 33 | G | G | A | 5.7 | 3.218592e-01 |  |
| 310 | 33 | G | G | C | 13.2 | 7.470139e-04 | \* |
| 310 | 33 | G | G | G | 81.1 | 3.552714e-15 | \* |
| 310 | 33 | G | G | T | 0.0 | 7.340426e-01 |  |
| 311 | 34 | G | G | A | 0.0 | 8.090909e-01 |  |
| 311 | 34 | G | G | C | 4.0 | 2.973307e-01 |  |
| 311 | 34 | G | G | G | 94.7 | 0.000000e+00 | \* |
| 311 | 34 | G | G | T | 1.3 | 6.577989e-01 |  |
| 312 | 35 | G | G | A | 3.3 | 5.755593e-01 |  |
| 312 | 35 | G | G | C | 1.7 | 6.811858e-01 |  |
| 312 | 35 | G | G | G | 95.0 | 0.000000e+00 | \* |
| 312 | 35 | G | G | T | 0.0 | 7.340426e-01 |  |
| 313 | 36 | C | C | A | 10.9 | 5.452663e-02 |  |
| 313 | 36 | C | C | C | 89.1 | 0.000000e+00 | \* |
| 313 | 36 | C | C | G | 0.0 | 8.241758e-01 |  |
| 313 | 36 | C | C | T | 0.0 | 7.340426e-01 |  |
| 314 | 37 | A | A | A | 85.9 | 0.000000e+00 | \* |
| 314 | 37 | A | A | C | 4.7 | 2.116650e-01 |  |
| 314 | 37 | A | A | G | 4.7 | 3.686277e-01 |  |
| 314 | 37 | A | A | T | 4.7 | 2.165598e-01 |  |
| 315 | 38 | C | C | A | 11.0 | 5.318139e-02 |  |
| 315 | 38 | C | C | C | 86.6 | 0.000000e+00 | \* |
| 315 | 38 | C | C | G | 1.2 | 7.687452e-01 |  |
| 315 | 38 | C | C | T | 1.2 | 6.706990e-01 |  |
| 316 | 39 | C | C | A | 10.8 | 5.588657e-02 |  |
| 316 | 39 | C | C | C | 83.1 | 0.000000e+00 | \* |
| 316 | 39 | C | C | G | 4.8 | 3.552895e-01 |  |
| 316 | 39 | C | C | T | 1.2 | 6.722956e-01 |  |
| 317 | 40 | C | C | A | 13.3 | 2.205972e-02 |  |
| 317 | 40 | C | C | C | 80.7 | 0.000000e+00 | \* |
| 317 | 40 | C | C | G | 0.0 | 8.241758e-01 |  |
| 317 | 40 | C | C | T | 6.0 | 1.174527e-01 |  |
| 318 | 41 | T | T | A | 4.8 | 4.119217e-01 |  |
| 318 | 41 | T | T | C | 6.3 | 8.333215e-02 |  |
| 318 | 41 | T | T | G | 4.8 | 3.610558e-01 |  |
| 318 | 41 | T | T | T | 84.1 | 0.000000e+00 | \* |
| 319 | 42 | G | G | A | 2.5 | 6.735918e-01 |  |
| 319 | 42 | G | G | C | 1.2 | 7.344256e-01 |  |
| 319 | 42 | G | G | G | 96.3 | 0.000000e+00 | \* |
| 319 | 42 | G | G | T | 0.0 | 7.340426e-01 |  |
| 320 | 43 | A | A | A | 92.9 | 0.000000e+00 | \* |
| 320 | 43 | A | A | C | 1.2 | 7.388242e-01 |  |
| 320 | 43 | A | A | G | 2.4 | 6.414003e-01 |  |
| 320 | 43 | A | A | T | 3.6 | 3.415034e-01 |  |
| 321 | 44 | C | C | A | 9.1 | 1.058576e-01 |  |
| 321 | 44 | C | C | C | 81.8 | 0.000000e+00 | \* |
| 321 | 44 | C | C | G | 9.1 | 9.041584e-02 |  |
| 321 | 44 | C | C | T | 0.0 | 7.340426e-01 |  |
| 322 | 45 | C | C | A | 7.7 | 1.710411e-01 |  |
| 322 | 45 | C | C | C | 78.5 | 0.000000e+00 | \* |
| 322 | 45 | C | C | G | 13.8 | 1.570733e-02 |  |
| 322 | 45 | C | C | T | 0.0 | 7.340426e-01 |  |
| 323 | 46 | G | G | A | 7.7 | 1.710411e-01 |  |
| 323 | 46 | G | G | C | 5.8 | 1.171265e-01 |  |
| 323 | 46 | G | G | G | 86.5 | 2.220446e-16 | \* |
| 323 | 46 | G | G | T | 0.0 | 7.340426e-01 |  |
| 324 | 47 | A | A | A | 92.6 | 0.000000e+00 | \* |
| 324 | 47 | A | A | C | 3.2 | 4.289777e-01 |  |
| 324 | 47 | A | A | G | 1.1 | 7.826194e-01 |  |
| 324 | 47 | A | A | T | 3.2 | 3.974486e-01 |  |
| 325 | 48 | C | C | A | 8.1 | 1.509945e-01 |  |
| 325 | 48 | C | C | C | 85.5 | 0.000000e+00 | \* |
| 325 | 48 | C | C | G | 0.0 | 8.241758e-01 |  |
| 325 | 48 | C | C | T | 6.5 | 9.545930e-02 |  |
| 326 | 49 | G | G | A | 4.9 | 3.995446e-01 |  |
| 326 | 49 | G | G | C | 7.3 | 4.587373e-02 |  |
| 326 | 49 | G | G | G | 87.8 | 2.220446e-16 | \* |
| 326 | 49 | G | G | T | 0.0 | 7.340426e-01 |  |
| 327 | 50 | G | G | A | 1.3 | 7.761448e-01 |  |
| 327 | 50 | G | G | C | 1.3 | 7.294572e-01 |  |
| 327 | 50 | G | G | G | 96.2 | 0.000000e+00 | \* |
| 327 | 50 | G | G | T | 1.3 | 6.637245e-01 |  |
| 328 | 51 | C | C | A | 2.0 | 7.205519e-01 |  |
| 328 | 51 | C | C | C | 88.0 | 0.000000e+00 | \* |
| 328 | 51 | C | C | G | 4.0 | 4.433590e-01 |  |
| 328 | 51 | C | C | T | 6.0 | 1.188156e-01 |  |
| 329 | 52 | A | A | A | 85.7 | 0.000000e+00 | \* |
| 329 | 52 | A | A | C | 3.6 | 3.610645e-01 |  |
| 329 | 52 | A | A | G | 0.0 | 8.241758e-01 |  |
| 329 | 52 | A | A | T | 10.7 | 9.898536e-03 | \* |
| 330 | 53 | A | A | A | 89.3 | 0.000000e+00 | \* |
| 330 | 53 | A | A | C | 0.0 | 7.831325e-01 |  |
| 330 | 53 | A | A | G | 7.1 | 1.747080e-01 |  |
| 330 | 53 | A | A | T | 3.6 | 3.415034e-01 |  |
| 331 | 54 | G | G | A | 2.5 | 6.702931e-01 |  |
| 331 | 54 | G | G | C | 5.0 | 1.795673e-01 |  |
| 331 | 54 | G | G | G | 90.0 | 2.220446e-16 | \* |
| 331 | 54 | G | G | T | 2.5 | 4.937698e-01 |  |
| 332 | 55 | A | A | A | 95.5 | 0.000000e+00 | \* |
| 332 | 55 | A | A | C | 0.0 | 7.831325e-01 |  |
| 332 | 55 | A | A | G | 0.0 | 8.241758e-01 |  |
| 332 | 55 | A | A | T | 4.5 | 2.369785e-01 |  |
| 333 | 56 | A | A | A | 91.1 | 0.000000e+00 | \* |
| 333 | 56 | A | A | C | 0.0 | 7.831325e-01 |  |
| 333 | 56 | A | A | G | 1.8 | 7.117386e-01 |  |
| 333 | 56 | A | A | T | 7.1 | 6.761004e-02 |  |
| 334 | 57 | G | G | A | 0.0 | 8.090909e-01 |  |
| 334 | 57 | G | G | C | 5.9 | 1.097271e-01 |  |
| 334 | 57 | G | G | G | 88.2 | 2.220446e-16 | \* |
| 334 | 57 | G | G | T | 5.9 | 1.256700e-01 |  |
| 335 | 58 | T | T | A | 4.7 | 4.199535e-01 |  |
| 335 | 58 | T | T | C | 1.6 | 6.955582e-01 |  |
| 335 | 58 | T | T | G | 0.0 | 8.241758e-01 |  |
| 335 | 58 | T | T | T | 93.8 | 0.000000e+00 | \* |
| 336 | 59 | T | T | A | 1.7 | 7.446477e-01 |  |
| 336 | 59 | T | T | C | 1.7 | 6.728966e-01 |  |
| 336 | 59 | T | T | G | 1.7 | 7.185343e-01 |  |
| 336 | 59 | T | T | T | 94.8 | 0.000000e+00 | \* |
| 337 | 60 | C | C | A | 5.2 | 3.691090e-01 |  |
| 337 | 60 | C | C | C | 87.9 | 0.000000e+00 | \* |
| 337 | 60 | C | C | G | 5.2 | 3.212025e-01 |  |
| 337 | 60 | C | C | T | 1.7 | 6.075907e-01 |  |
| 338 | 61 | G | G | A | 9.5 | 9.072206e-02 |  |
| 338 | 61 | G | G | C | 4.8 | 2.036440e-01 |  |
| 338 | 61 | G | G | G | 81.0 | 3.996803e-15 | \* |
| 338 | 61 | G | G | T | 4.8 | 2.096604e-01 |  |
| 339 | 62 | A | A | A | 87.4 | 0.000000e+00 | \* |
| 339 | 62 | A | A | C | 4.6 | 2.216667e-01 |  |
| 339 | 62 | A | A | G | 3.4 | 5.087096e-01 |  |
| 339 | 62 | A | A | T | 4.6 | 2.251220e-01 |  |
| 340 | 63 | C | C | A | 7.6 | 1.777618e-01 |  |
| 340 | 63 | C | C | C | 89.4 | 0.000000e+00 | \* |
| 340 | 63 | C | C | G | 1.5 | 7.406282e-01 |  |
| 340 | 63 | C | C | T | 1.5 | 6.354434e-01 |  |
| 341 | 64 | A | A | A | 87.5 | 0.000000e+00 | \* |
| 341 | 64 | A | A | C | 5.0 | 1.795673e-01 |  |
| 341 | 64 | A | A | G | 2.5 | 6.266780e-01 |  |
| 341 | 64 | A | A | T | 5.0 | 1.887495e-01 |  |
| 342 | 65 | G | G | A | 6.5 | 2.543532e-01 |  |
| 342 | 65 | G | G | C | 3.2 | 4.174657e-01 |  |
| 342 | 65 | G | G | G | 90.3 | 2.220446e-16 | \* |
| 342 | 65 | G | G | T | 0.0 | 7.340426e-01 |  |
| 343 | 66 | C | C | A | 3.3 | 5.755593e-01 |  |
| 343 | 66 | C | C | C | 91.7 | 0.000000e+00 | \* |
| 343 | 66 | C | C | G | 3.3 | 5.227797e-01 |  |
| 343 | 66 | C | C | T | 1.7 | 6.154456e-01 |  |
| 344 | 67 | T | T | A | 3.0 | 6.109174e-01 |  |
| 344 | 67 | T | T | C | 3.0 | 4.509118e-01 |  |
| 344 | 67 | T | T | G | 3.0 | 5.603684e-01 |  |
| 344 | 67 | T | T | T | 90.9 | 0.000000e+00 | \* |
| 345 | 68 | C | C | A | 6.2 | 2.704651e-01 |  |
| 345 | 68 | C | C | C | 85.9 | 0.000000e+00 | \* |
| 345 | 68 | C | C | G | 6.2 | 2.322214e-01 |  |
| 345 | 68 | C | C | T | 1.6 | 6.293141e-01 |  |
| 346 | 69 | C | C | A | 5.4 | 3.460314e-01 |  |
| 346 | 69 | C | C | C | 90.5 | 0.000000e+00 | \* |
| 346 | 69 | C | C | G | 0.0 | 8.241758e-01 |  |
| 346 | 69 | C | C | T | 4.1 | 2.825483e-01 |  |
| 347 | 70 | C | C | A | 7.7 | 1.710411e-01 |  |
| 347 | 70 | C | C | C | 92.3 | 0.000000e+00 | \* |
| 347 | 70 | C | C | G | 0.0 | 8.241758e-01 |  |
| 347 | 70 | C | C | T | 0.0 | 7.340426e-01 |  |
| 348 | 71 | G | G | A | 8.5 | 1.296492e-01 |  |
| 348 | 71 | G | G | C | 4.3 | 2.630716e-01 |  |
| 348 | 71 | G | G | G | 87.2 | 2.220446e-16 | \* |
| 348 | 71 | G | G | T | 0.0 | 7.340426e-01 |  |
| 349 | 72 | C | C | A | 0.0 | 8.090909e-01 |  |
| 349 | 72 | C | C | C | 90.7 | 0.000000e+00 | \* |
| 349 | 72 | C | C | G | 1.9 | 7.043130e-01 |  |
| 349 | 72 | C | C | T | 7.4 | 5.907976e-02 |  |
| 350 | 73 | G | G | A | 12.5 | 2.965521e-02 |  |
| 350 | 73 | G | G | C | 2.1 | 6.163882e-01 |  |
| 350 | 73 | G | G | G | 83.3 | 1.332268e-15 | \* |
| 350 | 73 | G | G | T | 2.1 | 5.560855e-01 |  |
| 351 | 74 | A | A | A | 93.1 | 0.000000e+00 | \* |
| 351 | 74 | A | A | C | 2.8 | 4.951948e-01 |  |
| 351 | 74 | A | A | G | 0.0 | 8.241758e-01 |  |
| 351 | 74 | A | A | T | 4.2 | 2.698607e-01 |  |
| 352 | 75 | C | C | A | 3.0 | 6.161342e-01 |  |
| 352 | 75 | C | C | C | 74.6 | 0.000000e+00 | \* |
| 352 | 75 | C | C | G | 14.9 | 1.035932e-02 |  |
| 352 | 75 | C | C | T | 7.5 | 5.742743e-02 |  |

## For use in R

If you want to work with the results in R, here is output that you can copy and paste in your terminal to get:

The base information:

```
structure(list(focal.base = c("A", "C", "G", "T"), avg.percsignal = c(89.0762331770573, 
86.7693947923298, 89.7978487804459, 90.5726806749583), avg.areasignal = c(75.7647058823529, 
83.6764705882353, 75.6607142857143, 90.7674418604651), crit.perc.area = c(15.21781457208, 
9.60987742004392, 15.0162657077128, 10.6961616998712), mu = c(5.46516940730652, 
3.73945771134026, 4.97461993502976, 3.84223075764827), fillibens = c(0.99476339180002, 
0.990325600574783, 0.997195540600062, 0.987005682709969)), .Names = c("focal.base", 
"avg.percsignal", "avg.areasignal", "crit.perc.area", "mu", "fillibens"
), row.names = c(NA, -4L), class = "data.frame")
```

the data.frame that contains information on the guide region:

```
structure(list(A.area = c(61, 74, 3, 140, 12, 0, 10, 7, 6, 7, 
59, 6, 143, 13, 8, 7, 0, 6, 0, 0, 0, 2, 0, 3, 2, 49, 6, 2, 66, 
5, 44, 2, 3, 0, 2, 6, 55, 9, 9, 11, 3, 2, 78, 7, 5, 4, 88, 5, 
2, 1, 1, 24, 50, 1, 64, 51, 0, 3, 1, 3, 4, 76, 5, 35, 4, 2, 2, 
4, 4, 3, 4, 0, 6, 67, 2), C.area = c(6, 3, 7, 2, 71, 3, 2, 3, 
56, 81, 1, 0, 3, 77, 58, 5, 1, 2, 2, 0, 3, 2, 1, 1, 58, 2, 65, 
7, 2, 59, 6, 54, 7, 3, 1, 49, 3, 71, 69, 67, 4, 1, 1, 63, 51, 
3, 3, 53, 3, 1, 44, 1, 0, 2, 0, 0, 2, 1, 1, 51, 2, 4, 59, 2, 
2, 55, 2, 55, 67, 36, 2, 49, 1, 2, 50), G.area = c(0, 0, 48, 
3, 0, 3, 144, 86, 5, 7, 9, 58, 4, 0, 5, 71, 5, 122, 7, 4, 6, 
85, 4, 70, 3, 3, 1, 1, 2, 1, 6, 4, 43, 71, 57, 0, 3, 1, 4, 0, 
3, 78, 2, 7, 9, 45, 1, 0, 36, 75, 2, 0, 4, 36, 0, 1, 30, 0, 1, 
3, 34, 3, 1, 1, 56, 2, 2, 4, 0, 0, 41, 1, 40, 0, 10), T.area = c(0, 
2, 0, 6, 2, 72, 2, 0, 3, 3, 0, 0, 7, 1, 0, 1, 58, 3, 64, 73, 
70, 4, 59, 4, 3, 2, 1, 61, 1, 0, 2, 2, 0, 1, 0, 0, 3, 1, 1, 5, 
53, 0, 3, 0, 0, 0, 3, 4, 0, 1, 3, 3, 2, 1, 3, 4, 2, 60, 55, 1, 
2, 4, 1, 2, 0, 1, 60, 1, 3, 0, 0, 4, 1, 3, 5), Tot.area = c(67, 
79, 58, 151, 85, 78, 158, 96, 70, 98, 69, 64, 157, 91, 71, 84, 
64, 133, 73, 77, 79, 93, 64, 78, 66, 56, 73, 71, 71, 65, 58, 
62, 53, 75, 60, 55, 64, 82, 83, 83, 63, 81, 84, 77, 65, 52, 95, 
62, 41, 78, 50, 28, 56, 40, 67, 56, 34, 64, 58, 58, 42, 87, 66, 
40, 62, 60, 66, 64, 74, 39, 47, 54, 48, 72, 67), A.perc = c(91.044776119403, 
93.6708860759494, 5.17241379310345, 92.7152317880795, 14.1176470588235, 
0, 6.32911392405063, 7.29166666666667, 8.57142857142857, 7.14285714285714, 
85.5072463768116, 9.375, 91.0828025477707, 14.2857142857143, 
11.2676056338028, 8.33333333333333, 0, 4.51127819548872, 0, 0, 
0, 2.1505376344086, 0, 3.84615384615385, 3.03030303030303, 87.5, 
8.21917808219178, 2.8169014084507, 92.9577464788732, 7.69230769230769, 
75.8620689655172, 3.2258064516129, 5.66037735849057, 0, 3.33333333333333, 
10.9090909090909, 85.9375, 10.9756097560976, 10.8433734939759, 
13.2530120481928, 4.76190476190476, 2.46913580246914, 92.8571428571429, 
9.09090909090909, 7.69230769230769, 7.69230769230769, 92.6315789473684, 
8.06451612903226, 4.8780487804878, 1.28205128205128, 2, 85.7142857142857, 
89.2857142857143, 2.5, 95.5223880597015, 91.0714285714286, 0, 
4.6875, 1.72413793103448, 5.17241379310345, 9.52380952380952, 
87.3563218390805, 7.57575757575758, 87.5, 6.45161290322581, 3.33333333333333, 
3.03030303030303, 6.25, 5.40540540540541, 7.69230769230769, 8.51063829787234, 
0, 12.5, 93.0555555555556, 2.98507462686567), C.perc = c(8.95522388059701, 
3.79746835443038, 12.0689655172414, 1.32450331125828, 83.5294117647059, 
3.84615384615385, 1.26582278481013, 3.125, 80, 82.6530612244898, 
1.44927536231884, 0, 1.91082802547771, 84.6153846153846, 81.6901408450704, 
5.95238095238095, 1.5625, 1.50375939849624, 2.73972602739726, 
0, 3.79746835443038, 2.1505376344086, 1.5625, 1.28205128205128, 
87.8787878787879, 3.57142857142857, 89.041095890411, 9.85915492957746, 
2.8169014084507, 90.7692307692308, 10.3448275862069, 87.0967741935484, 
13.2075471698113, 4, 1.66666666666667, 89.0909090909091, 4.6875, 
86.5853658536585, 83.1325301204819, 80.7228915662651, 6.34920634920635, 
1.23456790123457, 1.19047619047619, 81.8181818181818, 78.4615384615385, 
5.76923076923077, 3.15789473684211, 85.4838709677419, 7.31707317073171, 
1.28205128205128, 88, 3.57142857142857, 0, 5, 0, 0, 5.88235294117647, 
1.5625, 1.72413793103448, 87.9310344827586, 4.76190476190476, 
4.59770114942529, 89.3939393939394, 5, 3.2258064516129, 91.6666666666667, 
3.03030303030303, 85.9375, 90.5405405405405, 92.3076923076923, 
4.25531914893617, 90.7407407407407, 2.08333333333333, 2.77777777777778, 
74.6268656716418), G.perc = c(0, 0, 82.7586206896552, 1.98675496688742, 
0, 3.84615384615385, 91.1392405063291, 89.5833333333333, 7.14285714285714, 
7.14285714285714, 13.0434782608696, 90.625, 2.54777070063694, 
0, 7.04225352112676, 84.5238095238095, 7.8125, 91.7293233082707, 
9.58904109589041, 5.19480519480519, 7.59493670886076, 91.3978494623656, 
6.25, 89.7435897435897, 4.54545454545455, 5.35714285714286, 1.36986301369863, 
1.40845070422535, 2.8169014084507, 1.53846153846154, 10.3448275862069, 
6.45161290322581, 81.1320754716981, 94.6666666666667, 95, 0, 
4.6875, 1.21951219512195, 4.81927710843374, 0, 4.76190476190476, 
96.2962962962963, 2.38095238095238, 9.09090909090909, 13.8461538461538, 
86.5384615384615, 1.05263157894737, 0, 87.8048780487805, 96.1538461538462, 
4, 0, 7.14285714285714, 90, 0, 1.78571428571429, 88.2352941176471, 
0, 1.72413793103448, 5.17241379310345, 80.9523809523809, 3.44827586206897, 
1.51515151515152, 2.5, 90.3225806451613, 3.33333333333333, 3.03030303030303, 
6.25, 0, 0, 87.2340425531915, 1.85185185185185, 83.3333333333333, 
0, 14.9253731343284), T.perc = c(0, 2.53164556962025, 0, 3.97350993377483, 
2.35294117647059, 92.3076923076923, 1.26582278481013, 0, 4.28571428571429, 
3.06122448979592, 0, 0, 4.45859872611465, 1.0989010989011, 0, 
1.19047619047619, 90.625, 2.25563909774436, 87.6712328767123, 
94.8051948051948, 88.6075949367089, 4.3010752688172, 92.1875, 
5.12820512820513, 4.54545454545455, 3.57142857142857, 1.36986301369863, 
85.9154929577465, 1.40845070422535, 0, 3.44827586206897, 3.2258064516129, 
0, 1.33333333333333, 0, 0, 4.6875, 1.21951219512195, 1.20481927710843, 
6.02409638554217, 84.1269841269841, 0, 3.57142857142857, 0, 0, 
0, 3.15789473684211, 6.45161290322581, 0, 1.28205128205128, 6, 
10.7142857142857, 3.57142857142857, 2.5, 4.47761194029851, 7.14285714285714, 
5.88235294117647, 93.75, 94.8275862068966, 1.72413793103448, 
4.76190476190476, 4.59770114942529, 1.51515151515152, 5, 0, 1.66666666666667, 
90.9090909090909, 1.5625, 4.05405405405405, 0, 0, 7.40740740740741, 
2.08333333333333, 4.16666666666667, 7.46268656716418), base.call = c("A", 
"A", "G", "A", "C", "T", "G", "G", "C", "C", "A", "G", "A", "C", 
"C", "G", "T", "G", "T", "T", "T", "G", "T", "G", "C", "A", "C", 
"T", "A", "C", "A", "C", "G", "G", "G", "C", "A", "C", "C", "C", 
"T", "G", "A", "C", "C", "G", "A", "C", "G", "G", "C", "A", "A", 
"G", "A", "A", "G", "T", "T", "C", "G", "A", "C", "A", "G", "C", 
"T", "C", "C", "C", "G", "C", "G", "A", "C"), index = 278:352, 
    guide.seq = c("A", "A", "G", "A", "C", "T", "G", "G", "C", 
    "C", "A", "G", "A", "C", "C", "G", "T", "G", "T", "T", "T", 
    "G", "T", "G", "C", "A", "C", "T", "A", "C", "A", "C", "G", 
    "G", "G", "C", "A", "C", "C", "C", "T", "G", "A", "C", "C", 
    "G", "A", "C", "G", "G", "C", "A", "A", "G", "A", "A", "G", 
    "T", "T", "C", "G", "A", "C", "A", "G", "C", "T", "C", "C", 
    "C", "G", "C", "G", "A", "C"), T.pval = c(0.73404255319148, 
    0.489021726042208, 0.73404255319148, 0.291875313773579, 0.515859620234616, 
    0, 0.665561570662444, 0.73404255319148, 0.256898217263801, 
    0.411142877399371, 0.73404255319148, 0.73404255319148, 0.238898511241271, 
    0.683304895760426, 0.73404255319148, 0.673838337800438, 0, 
    0.530450551104239, 0, 0, 0, 0.255259393721143, 0, 0.178209832254756, 
    0.230222986728187, 0.341503419654277, 0.653470255931151, 
    0, 0.64880500809297, 0.73404255319148, 0.357679799257646, 
    0.387956512713095, 0.73404255319148, 0.657798885826009, 0.73404255319148, 
    0.73404255319148, 0.216559791970409, 0.670698959833726, 0.67229558492421, 
    0.117452720735717, 0, 0.73404255319148, 0.341503419654277, 
    0.73404255319148, 0.73404255319148, 0.73404255319148, 0.39744859787905, 
    0.0954593024906618, 0.73404255319148, 0.663724526622574, 
    0.118815592824437, 0.00989853633175386, 0.341503419654277, 
    0.493769766806324, 0.236978453881517, 0.0676100430211708, 
    0.125669988637834, 0, 0, 0.607590656604131, 0.209660379130664, 
    0.225122002695197, 0.635443367365527, 0.188749464252963, 
    0.73404255319148, 0.615445565962587, 0, 0.629314078505245, 
    0.282548313098769, 0.73404255319148, 0.73404255319148, 0.059079763252144, 
    0.556085480280932, 0.269860740598832, 0.0574274329406355), 
    C.pval = c(0.0156514861531457, 0.326513274737954, 0.0017328593542616, 
    0.724816650121553, 0, 0.319340472343068, 0.731181970279841, 
    0.434596430327451, 0, 0, 0.710136965314028, 0.78313253011575, 
    0.644426803828768, 0, 0, 0.105352529960949, 0.695558198583069, 
    0.703264044879066, 0.501930975125264, 0.78313253011575, 0.326513274737954, 
    0.605111292922963, 0.695558198583069, 0.729457246597382, 
    0, 0.361064532775619, 0, 0.00841216572557357, 0.488280664460126, 
    0, 0.00598591831679207, 0, 0.000747013884970338, 0.29733070712748, 
    0.681185829941762, 0, 0.211664977456906, 0, 0, 0, 0.0833321453130691, 
    0.734425624337817, 0.738824183308581, 0, 0, 0.117126481680391, 
    0.428977676406161, 0, 0.0458737312470079, 0.729457246597382, 
    0, 0.361064532775619, 0.78313253011575, 0.179567259897694, 
    0.78313253011575, 0.78313253011575, 0.10972706978299, 0.695558198583069, 
    0.672896627886775, 0, 0.203644008703111, 0.22166672430008, 
    0, 0.179567259897694, 0.417465712536847, 0, 0.450911844660688, 
    0, 0, 0, 0.263071602446747, 0, 0.616388203839786, 0.495194839732246, 
    0), G.pval = c(0.824175824175824, 0.824175824175824, 1.77635683940025e-15, 
    0.688803937232517, 0.824175824175824, 0.461163591040686, 
    0, 2.22044604925031e-16, 0.174707963632879, 0.174707963632879, 
    0.0213261827524118, 2.22044604925031e-16, 0.620738232176034, 
    0.824175824175824, 0.180512822138059, 8.88178419700125e-16, 
    0.140042117780499, 0, 0.0758884341078434, 0.319123345323487, 
    0.15057533602643, 0, 0.232221386593287, 2.22044604925031e-16, 
    0.383373356696229, 0.304344624525445, 0.754960193367769, 
    0.751245015883273, 0.587083568477596, 0.738245310090566, 
    0.0579270906711238, 0.218015299876975, 3.5527136788005e-15, 
    0, 0, 0.824175824175824, 0.368627712202162, 0.768745179056715, 
    0.355289500695952, 0.824175824175824, 0.361055825070017, 
    0, 0.64140029328074, 0.0904158426941122, 0.0157073289323326, 
    2.22044604925031e-16, 0.782619421478335, 0.824175824175824, 
    2.22044604925031e-16, 0, 0.443359001113389, 0.824175824175824, 
    0.174707963632879, 2.22044604925031e-16, 0.824175824175824, 
    0.711738608257491, 2.22044604925031e-16, 0.824175824175824, 
    0.718534296043536, 0.32120245915267, 3.99680288865056e-15, 
    0.508709631575635, 0.740628245054216, 0.626678015467012, 
    2.22044604925031e-16, 0.522779676562378, 0.56036841185384, 
    0.232221386593287, 0.824175824175824, 0.824175824175824, 
    2.22044604925031e-16, 0.70431296653948, 1.33226762955019e-15, 
    0.824175824175824, 0.0103593186497231), A.pval = c(0, 0, 
    0.369108965134789, 0, 0.015623486354958, 0.809090909090909, 
    0.264054026711545, 0.195068103920442, 0.126953949205955, 
    0.20467117088597, 0, 0.0956904670141581, 0, 0.014601379734567, 
    0.047632785253408, 0.137797030549039, 0.809090909090909, 
    0.439277947744783, 0.809090909090909, 0.809090909090909, 
    0.809090909090909, 0.70620978605919, 0.809090909090909, 0.515276212158627, 
    0.610917382615864, 0, 0.143274628405755, 0.635327066852662, 
    0, 0.17104108464249, 4.66293670342566e-15, 0.588168836571973, 
    0.321859186529303, 0.809090909090909, 0.575559293478424, 
    0.0545266334509373, 0, 0.0531813902938364, 0.0558865712460309, 
    0.0220597186262724, 0.411921657106645, 0.673591751133363, 
    0, 0.105857583369206, 0.17104108464249, 0.17104108464249, 
    0, 0.150994454514939, 0.399544599517996, 0.77614483312441, 
    0.720551887608353, 0, 0, 0.670293053588017, 0, 0, 0.809090909090909, 
    0.419953542038625, 0.744647703260277, 0.369108965134789, 
    0.0907220591608734, 0, 0.177761755039492, 0, 0.254353197798932, 
    0.575559293478424, 0.610917382615864, 0.270465107693901, 
    0.346031406784496, 0.17104108464249, 0.12964918382502, 0.809090909090909, 
    0.0296552132572075, 0, 0.616134248824483), guide.position = 1:75), .Names = c("A.area", 
"C.area", "G.area", "T.area", "Tot.area", "A.perc", "C.perc", 
"G.perc", "T.perc", "base.call", "index", "guide.seq", "T.pval", 
"C.pval", "G.pval", "A.pval", "guide.position"), row.names = 278:352, class = "data.frame")
```

*Report generated using EditR v1.0.8*
