# Supplementary material for: Mechanistic and genetic basis of single-strand templated repair at Cas12a-induced DNA breaks in Chlamydomonas reinhardtii
Source: Nat Commun. 2021 Nov 19;12:6751. doi: 10.1038/s41467-021-27004-1 (PMC8604939; doi:10.1038/s41467-021-27004-1)
Supplement: Supplementary file 22 — Source Data [file 41467_2021_27004_MOESM22_ESM.zip › Source Data/EditR analysis/EditR outputs/Sense/rep3_ssODN_sense_-32.html]

EditR v1.0.8 report


# EditR v1.0.8 report

- Data QA
  - Filtering data
  - Percent noise peak area
  - Base information
- Predicted editing
  - Editing bar plot
  - Editing table plot
  - Table of editing results
- For use in R

## Data QA

### Filtering data

What the data looked like prefiltering:

and the post filtering signal / noise plot:

### Percent noise peak area

### Base information

Here’s information about the signal of each base, the critical percent value where any higher value would be called as significant, and Filliben’s correlation for how well the noise was modelled by the zero adjusted gamma distribution.

| Base | Average percent signal | Average peak area | Critical percent value | model mu | Fillibens correlation |
| --- | --- | --- | --- | --- | --- |
| A | 98.39558 | 381.1176 | 0.5032601 | 0.3498991 | 0.9948543 |
| C | 99.85373 | 380.2090 | 0.7783528 | 0.4726940 | 0.9946243 |
| G | 99.78928 | 392.1607 | 0.8509341 | 0.4864646 | 0.9943969 |
| T | 99.72008 | 361.7273 | 0.5896033 | 0.3534568 | 0.9963614 |

## Predicted editing

### Editing bar plot

### Editing table plot

### Table of editing results


Here’s the entire guide region

| Sanger position | Guide position | Guide sequence | Sanger base call | Focal base | Focal base peak area | p value |  |
| --- | --- | --- | --- | --- | --- | --- | --- |
| 276 | 1 | A | A | A | 100.00 | 0.000000e+00 | \* |
| 276 | 1 | A | A | C | 0.00 | 1.666667e-01 |  |
| 276 | 1 | A | A | G | 0.00 | 2.197802e-01 |  |
| 276 | 1 | A | A | T | 0.00 | 1.075269e-01 |  |
| 277 | 2 | A | A | A | 100.00 | 0.000000e+00 | \* |
| 277 | 2 | A | A | C | 0.00 | 1.666667e-01 |  |
| 277 | 2 | A | A | G | 0.00 | 2.197802e-01 |  |
| 277 | 2 | A | A | T | 0.00 | 1.075269e-01 |  |
| 278 | 3 | G | G | A | 1.00 | 3.996858e-06 | \* |
| 278 | 3 | G | G | C | 0.00 | 1.666667e-01 |  |
| 278 | 3 | G | G | G | 99.00 | 0.000000e+00 | \* |
| 278 | 3 | G | G | T | 0.00 | 1.075269e-01 |  |
| 279 | 4 | A | A | A | 100.00 | 0.000000e+00 | \* |
| 279 | 4 | A | A | C | 0.00 | 1.666667e-01 |  |
| 279 | 4 | A | A | G | 0.00 | 2.197802e-01 |  |
| 279 | 4 | A | A | T | 0.00 | 1.075269e-01 |  |
| 280 | 5 | C | C | A | 0.00 | 1.000000e-01 |  |
| 280 | 5 | C | C | C | 100.00 | 0.000000e+00 | \* |
| 280 | 5 | C | C | G | 0.00 | 2.197802e-01 |  |
| 280 | 5 | C | C | T | 0.00 | 1.075269e-01 |  |
| 281 | 6 | T | T | A | 0.00 | 1.000000e-01 |  |
| 281 | 6 | T | T | C | 0.00 | 1.666667e-01 |  |
| 281 | 6 | T | T | G | 0.00 | 2.197802e-01 |  |
| 281 | 6 | T | T | T | 100.00 | 0.000000e+00 | \* |
| 282 | 7 | G | G | A | 0.00 | 1.000000e-01 |  |
| 282 | 7 | G | G | C | 0.00 | 1.666667e-01 |  |
| 282 | 7 | G | G | G | 100.00 | 0.000000e+00 | \* |
| 282 | 7 | G | G | T | 0.00 | 1.075269e-01 |  |
| 283 | 8 | G | G | A | 0.00 | 1.000000e-01 |  |
| 283 | 8 | G | G | C | 0.00 | 1.666667e-01 |  |
| 283 | 8 | G | G | G | 100.00 | 0.000000e+00 | \* |
| 283 | 8 | G | G | T | 0.00 | 1.075269e-01 |  |
| 284 | 9 | C | C | A | 0.00 | 1.000000e-01 |  |
| 284 | 9 | C | C | C | 99.65 | 0.000000e+00 | \* |
| 284 | 9 | C | C | G | 0.35 | 1.626516e-01 |  |
| 284 | 9 | C | C | T | 0.00 | 1.075269e-01 |  |
| 285 | 10 | C | C | A | 0.00 | 1.000000e-01 |  |
| 285 | 10 | C | C | C | 100.00 | 0.000000e+00 | \* |
| 285 | 10 | C | C | G | 0.00 | 2.197802e-01 |  |
| 285 | 10 | C | C | T | 0.00 | 1.075269e-01 |  |
| 286 | 11 | A | A | A | 51.40 | 0.000000e+00 | \* |
| 286 | 11 | A | A | C | 0.28 | 1.451543e-01 |  |
| 286 | 11 | A | A | G | 0.28 | 1.918512e-01 |  |
| 286 | 11 | A | A | T | 48.03 | 0.000000e+00 | \* |
| 287 | 12 | G | G | A | 0.72 | 4.422538e-04 | \* |
| 287 | 12 | G | G | C | 0.00 | 1.666667e-01 |  |
| 287 | 12 | G | G | G | 99.28 | 0.000000e+00 | \* |
| 287 | 12 | G | G | T | 0.00 | 1.075269e-01 |  |
| 288 | 13 | A | A | A | 99.79 | 0.000000e+00 | \* |
| 288 | 13 | A | A | C | 0.00 | 1.666667e-01 |  |
| 288 | 13 | A | A | G | 0.21 | 2.104544e-01 |  |
| 288 | 13 | A | A | T | 0.00 | 1.075269e-01 |  |
| 289 | 14 | C | C | A | 0.00 | 1.000000e-01 |  |
| 289 | 14 | C | C | C | 100.00 | 0.000000e+00 | \* |
| 289 | 14 | C | C | G | 0.00 | 2.197802e-01 |  |
| 289 | 14 | C | C | T | 0.00 | 1.075269e-01 |  |
| 290 | 15 | C | C | A | 0.00 | 1.000000e-01 |  |
| 290 | 15 | C | C | C | 100.00 | 0.000000e+00 | \* |
| 290 | 15 | C | C | G | 0.00 | 2.197802e-01 |  |
| 290 | 15 | C | C | T | 0.00 | 1.075269e-01 |  |
| 291 | 16 | G | G | A | 0.00 | 1.000000e-01 |  |
| 291 | 16 | G | G | C | 0.00 | 1.666667e-01 |  |
| 291 | 16 | G | G | G | 99.76 | 0.000000e+00 | \* |
| 291 | 16 | G | G | T | 0.24 | 7.761843e-02 |  |
| 292 | 17 | T | T | A | 0.00 | 1.000000e-01 |  |
| 292 | 17 | T | T | C | 0.00 | 1.666667e-01 |  |
| 292 | 17 | T | T | G | 0.71 | 2.709343e-02 |  |
| 292 | 17 | T | T | T | 99.29 | 0.000000e+00 | \* |
| 293 | 18 | G | G | A | 0.00 | 1.000000e-01 |  |
| 293 | 18 | G | G | C | 0.00 | 1.666667e-01 |  |
| 293 | 18 | G | G | G | 99.57 | 0.000000e+00 | \* |
| 293 | 18 | G | G | T | 0.43 | 2.997007e-02 |  |
| 294 | 19 | T | T | A | 0.00 | 1.000000e-01 |  |
| 294 | 19 | T | T | C | 0.00 | 1.666667e-01 |  |
| 294 | 19 | T | T | G | 0.00 | 2.197802e-01 |  |
| 294 | 19 | T | T | T | 100.00 | 0.000000e+00 | \* |
| 295 | 20 | T | T | A | 0.00 | 1.000000e-01 |  |
| 295 | 20 | T | T | C | 0.00 | 1.666667e-01 |  |
| 295 | 20 | T | T | G | 0.00 | 2.197802e-01 |  |
| 295 | 20 | T | T | T | 100.00 | 0.000000e+00 | \* |
| 296 | 21 | T | T | A | 0.00 | 1.000000e-01 |  |
| 296 | 21 | T | T | C | 0.00 | 1.666667e-01 |  |
| 296 | 21 | T | T | G | 0.53 | 7.948480e-02 |  |
| 296 | 21 | T | T | T | 99.47 | 0.000000e+00 | \* |
| 297 | 22 | G | G | A | 0.00 | 1.000000e-01 |  |
| 297 | 22 | G | G | C | 0.00 | 1.666667e-01 |  |
| 297 | 22 | G | G | G | 99.59 | 0.000000e+00 | \* |
| 297 | 22 | G | G | T | 0.41 | 3.376523e-02 |  |
| 298 | 23 | T | T | A | 0.00 | 1.000000e-01 |  |
| 298 | 23 | T | T | C | 0.00 | 1.666667e-01 |  |
| 298 | 23 | T | T | G | 0.99 | 3.309179e-03 | \* |
| 298 | 23 | T | T | T | 99.01 | 0.000000e+00 | \* |
| 299 | 24 | G | G | A | 0.00 | 1.000000e-01 |  |
| 299 | 24 | G | G | C | 0.00 | 1.666667e-01 |  |
| 299 | 24 | G | G | G | 100.00 | 0.000000e+00 | \* |
| 299 | 24 | G | G | T | 0.00 | 1.075269e-01 |  |
| 300 | 25 | C | C | A | 0.27 | 7.460612e-02 |  |
| 300 | 25 | C | C | C | 99.46 | 0.000000e+00 | \* |
| 300 | 25 | C | C | G | 0.27 | 1.961027e-01 |  |
| 300 | 25 | C | C | T | 0.00 | 1.075269e-01 |  |
| 301 | 26 | A | A | A | 100.00 | 0.000000e+00 | \* |
| 301 | 26 | A | A | C | 0.00 | 1.666667e-01 |  |
| 301 | 26 | A | A | G | 0.00 | 2.197802e-01 |  |
| 301 | 26 | A | A | T | 0.00 | 1.075269e-01 |  |
| 302 | 27 | C | C | A | 0.00 | 1.000000e-01 |  |
| 302 | 27 | C | C | C | 100.00 | 0.000000e+00 | \* |
| 302 | 27 | C | C | G | 0.00 | 2.197802e-01 |  |
| 302 | 27 | C | C | T | 0.00 | 1.075269e-01 |  |
| 303 | 28 | T | T | A | 0.00 | 1.000000e-01 |  |
| 303 | 28 | T | T | C | 0.00 | 1.666667e-01 |  |
| 303 | 28 | T | T | G | 0.00 | 2.197802e-01 |  |
| 303 | 28 | T | T | T | 100.00 | 0.000000e+00 | \* |
| 304 | 29 | A | A | A | 100.00 | 0.000000e+00 | \* |
| 304 | 29 | A | A | C | 0.00 | 1.666667e-01 |  |
| 304 | 29 | A | A | G | 0.00 | 2.197802e-01 |  |
| 304 | 29 | A | A | T | 0.00 | 1.075269e-01 |  |
| 305 | 30 | C | C | A | 0.00 | 1.000000e-01 |  |
| 305 | 30 | C | C | C | 100.00 | 0.000000e+00 | \* |
| 305 | 30 | C | C | G | 0.00 | 2.197802e-01 |  |
| 305 | 30 | C | C | T | 0.00 | 1.075269e-01 |  |
| 306 | 31 | A | A | A | 100.00 | 0.000000e+00 | \* |
| 306 | 31 | A | A | C | 0.00 | 1.666667e-01 |  |
| 306 | 31 | A | A | G | 0.00 | 2.197802e-01 |  |
| 306 | 31 | A | A | T | 0.00 | 1.075269e-01 |  |
| 307 | 32 | C | C | A | 0.00 | 1.000000e-01 |  |
| 307 | 32 | C | C | C | 100.00 | 0.000000e+00 | \* |
| 307 | 32 | C | C | G | 0.00 | 2.197802e-01 |  |
| 307 | 32 | C | C | T | 0.00 | 1.075269e-01 |  |
| 308 | 33 | G | G | A | 0.00 | 1.000000e-01 |  |
| 308 | 33 | G | G | C | 0.00 | 1.666667e-01 |  |
| 308 | 33 | G | G | G | 100.00 | 0.000000e+00 | \* |
| 308 | 33 | G | G | T | 0.00 | 1.075269e-01 |  |
| 309 | 34 | G | G | A | 0.00 | 1.000000e-01 |  |
| 309 | 34 | G | G | C | 0.00 | 1.666667e-01 |  |
| 309 | 34 | G | G | G | 100.00 | 0.000000e+00 | \* |
| 309 | 34 | G | G | T | 0.00 | 1.075269e-01 |  |
| 310 | 35 | G | G | A | 0.00 | 1.000000e-01 |  |
| 310 | 35 | G | G | C | 0.55 | 4.868013e-02 |  |
| 310 | 35 | G | G | G | 99.45 | 0.000000e+00 | \* |
| 310 | 35 | G | G | T | 0.00 | 1.075269e-01 |  |
| 311 | 36 | C | C | A | 0.00 | 1.000000e-01 |  |
| 311 | 36 | C | C | C | 99.70 | 0.000000e+00 | \* |
| 311 | 36 | C | C | G | 0.30 | 1.833365e-01 |  |
| 311 | 36 | C | C | T | 0.00 | 1.075269e-01 |  |
| 312 | 37 | A | A | A | 99.36 | 0.000000e+00 | \* |
| 312 | 37 | A | A | C | 0.64 | 2.769618e-02 |  |
| 312 | 37 | A | A | G | 0.00 | 2.197802e-01 |  |
| 312 | 37 | A | A | T | 0.00 | 1.075269e-01 |  |
| 313 | 38 | C | C | A | 0.23 | 8.623755e-02 |  |
| 313 | 38 | C | C | C | 99.77 | 0.000000e+00 | \* |
| 313 | 38 | C | C | G | 0.00 | 2.197802e-01 |  |
| 313 | 38 | C | C | T | 0.00 | 1.075269e-01 |  |
| 314 | 39 | C | C | A | 0.00 | 1.000000e-01 |  |
| 314 | 39 | C | C | C | 100.00 | 0.000000e+00 | \* |
| 314 | 39 | C | C | G | 0.00 | 2.197802e-01 |  |
| 314 | 39 | C | C | T | 0.00 | 1.075269e-01 |  |
| 315 | 40 | C | C | A | 0.00 | 1.000000e-01 |  |
| 315 | 40 | C | C | C | 99.73 | 0.000000e+00 | \* |
| 315 | 40 | C | C | G | 0.00 | 2.197802e-01 |  |
| 315 | 40 | C | C | T | 0.27 | 7.024181e-02 |  |
| 316 | 41 | T | T | A | 0.00 | 1.000000e-01 |  |
| 316 | 41 | T | T | C | 0.34 | 1.250905e-01 |  |
| 316 | 41 | T | T | G | 0.34 | 1.670472e-01 |  |
| 316 | 41 | T | T | T | 99.32 | 0.000000e+00 | \* |
| 317 | 42 | G | G | A | 0.44 | 1.957039e-02 |  |
| 317 | 42 | G | G | C | 0.00 | 1.666667e-01 |  |
| 317 | 42 | G | G | G | 99.56 | 0.000000e+00 | \* |
| 317 | 42 | G | G | T | 0.00 | 1.075269e-01 |  |
| 318 | 43 | A | A | A | 100.00 | 0.000000e+00 | \* |
| 318 | 43 | A | A | C | 0.00 | 1.666667e-01 |  |
| 318 | 43 | A | A | G | 0.00 | 2.197802e-01 |  |
| 318 | 43 | A | A | T | 0.00 | 1.075269e-01 |  |
| 319 | 44 | C | C | A | 0.31 | 5.858279e-02 |  |
| 319 | 44 | C | C | C | 99.69 | 0.000000e+00 | \* |
| 319 | 44 | C | C | G | 0.00 | 2.197802e-01 |  |
| 319 | 44 | C | C | T | 0.00 | 1.075269e-01 |  |
| 320 | 45 | C | C | A | 0.00 | 1.000000e-01 |  |
| 320 | 45 | C | C | C | 100.00 | 0.000000e+00 | \* |
| 320 | 45 | C | C | G | 0.00 | 2.197802e-01 |  |
| 320 | 45 | C | C | T | 0.00 | 1.075269e-01 |  |
| 321 | 46 | G | G | A | 0.00 | 1.000000e-01 |  |
| 321 | 46 | G | G | C | 0.00 | 1.666667e-01 |  |
| 321 | 46 | G | G | G | 100.00 | 0.000000e+00 | \* |
| 321 | 46 | G | G | T | 0.00 | 1.075269e-01 |  |
| 322 | 47 | A | A | A | 100.00 | 0.000000e+00 | \* |
| 322 | 47 | A | A | C | 0.00 | 1.666667e-01 |  |
| 322 | 47 | A | A | G | 0.00 | 2.197802e-01 |  |
| 322 | 47 | A | A | T | 0.00 | 1.075269e-01 |  |
| 323 | 48 | C | C | A | 0.00 | 1.000000e-01 |  |
| 323 | 48 | C | C | C | 100.00 | 0.000000e+00 | \* |
| 323 | 48 | C | C | G | 0.00 | 2.197802e-01 |  |
| 323 | 48 | C | C | T | 0.00 | 1.075269e-01 |  |
| 324 | 49 | G | G | A | 0.00 | 1.000000e-01 |  |
| 324 | 49 | G | G | C | 0.00 | 1.666667e-01 |  |
| 324 | 49 | G | G | G | 100.00 | 0.000000e+00 | \* |
| 324 | 49 | G | G | T | 0.00 | 1.075269e-01 |  |
| 325 | 50 | G | G | A | 0.00 | 1.000000e-01 |  |
| 325 | 50 | G | G | C | 0.00 | 1.666667e-01 |  |
| 325 | 50 | G | G | G | 100.00 | 0.000000e+00 | \* |
| 325 | 50 | G | G | T | 0.00 | 1.075269e-01 |  |
| 326 | 51 | C | C | A | 0.34 | 5.022645e-02 |  |
| 326 | 51 | C | C | C | 99.66 | 0.000000e+00 | \* |
| 326 | 51 | C | C | G | 0.00 | 2.197802e-01 |  |
| 326 | 51 | C | C | T | 0.00 | 1.075269e-01 |  |
| 327 | 52 | A | A | A | 100.00 | 0.000000e+00 | \* |
| 327 | 52 | A | A | C | 0.00 | 1.666667e-01 |  |
| 327 | 52 | A | A | G | 0.00 | 2.197802e-01 |  |
| 327 | 52 | A | A | T | 0.00 | 1.075269e-01 |  |
| 328 | 53 | A | A | A | 100.00 | 0.000000e+00 | \* |
| 328 | 53 | A | A | C | 0.00 | 1.666667e-01 |  |
| 328 | 53 | A | A | G | 0.00 | 2.197802e-01 |  |
| 328 | 53 | A | A | T | 0.00 | 1.075269e-01 |  |
| 329 | 54 | G | G | A | 1.03 | 2.111230e-06 | \* |
| 329 | 54 | G | G | C | 0.00 | 1.666667e-01 |  |
| 329 | 54 | G | G | G | 98.97 | 0.000000e+00 | \* |
| 329 | 54 | G | G | T | 0.00 | 1.075269e-01 |  |
| 330 | 55 | A | A | A | 100.00 | 0.000000e+00 | \* |
| 330 | 55 | A | A | C | 0.00 | 1.666667e-01 |  |
| 330 | 55 | A | A | G | 0.00 | 2.197802e-01 |  |
| 330 | 55 | A | A | T | 0.00 | 1.075269e-01 |  |
| 331 | 56 | A | A | A | 100.00 | 0.000000e+00 | \* |
| 331 | 56 | A | A | C | 0.00 | 1.666667e-01 |  |
| 331 | 56 | A | A | G | 0.00 | 2.197802e-01 |  |
| 331 | 56 | A | A | T | 0.00 | 1.075269e-01 |  |
| 332 | 57 | G | G | A | 0.29 | 6.680301e-02 |  |
| 332 | 57 | G | G | C | 0.00 | 1.666667e-01 |  |
| 332 | 57 | G | G | G | 99.71 | 0.000000e+00 | \* |
| 332 | 57 | G | G | T | 0.00 | 1.075269e-01 |  |
| 333 | 58 | T | T | A | 0.00 | 1.000000e-01 |  |
| 333 | 58 | T | T | C | 0.00 | 1.666667e-01 |  |
| 333 | 58 | T | T | G | 0.28 | 1.910193e-01 |  |
| 333 | 58 | T | T | T | 99.72 | 0.000000e+00 | \* |
| 334 | 59 | T | T | A | 0.00 | 1.000000e-01 |  |
| 334 | 59 | T | T | C | 0.00 | 1.666667e-01 |  |
| 334 | 59 | T | T | G | 0.00 | 2.197802e-01 |  |
| 334 | 59 | T | T | T | 100.00 | 0.000000e+00 | \* |
| 335 | 60 | C | C | A | 0.00 | 1.000000e-01 |  |
| 335 | 60 | C | C | C | 100.00 | 0.000000e+00 | \* |
| 335 | 60 | C | C | G | 0.00 | 2.197802e-01 |  |
| 335 | 60 | C | C | T | 0.00 | 1.075269e-01 |  |
| 336 | 61 | G | G | A | 0.84 | 6.147569e-05 | \* |
| 336 | 61 | G | G | C | 0.00 | 1.666667e-01 |  |
| 336 | 61 | G | G | G | 99.16 | 0.000000e+00 | \* |
| 336 | 61 | G | G | T | 0.00 | 1.075269e-01 |  |
| 337 | 62 | A | A | A | 99.63 | 0.000000e+00 | \* |
| 337 | 62 | A | A | C | 0.37 | 1.145738e-01 |  |
| 337 | 62 | A | A | G | 0.00 | 2.197802e-01 |  |
| 337 | 62 | A | A | T | 0.00 | 1.075269e-01 |  |
| 338 | 63 | C | C | A | 0.46 | 1.701552e-02 |  |
| 338 | 63 | C | C | C | 99.54 | 0.000000e+00 | \* |
| 338 | 63 | C | C | G | 0.00 | 2.197802e-01 |  |
| 338 | 63 | C | C | T | 0.00 | 1.075269e-01 |  |
| 339 | 64 | A | A | A | 99.66 | 0.000000e+00 | \* |
| 339 | 64 | A | A | C | 0.00 | 1.666667e-01 |  |
| 339 | 64 | A | A | G | 0.34 | 1.691174e-01 |  |
| 339 | 64 | A | A | T | 0.00 | 1.075269e-01 |  |
| 340 | 65 | G | G | A | 0.34 | 4.819628e-02 |  |
| 340 | 65 | G | G | C | 0.00 | 1.666667e-01 |  |
| 340 | 65 | G | G | G | 99.66 | 0.000000e+00 | \* |
| 340 | 65 | G | G | T | 0.00 | 1.075269e-01 |  |
| 341 | 66 | C | C | A | 0.00 | 1.000000e-01 |  |
| 341 | 66 | C | C | C | 100.00 | 0.000000e+00 | \* |
| 341 | 66 | C | C | G | 0.00 | 2.197802e-01 |  |
| 341 | 66 | C | C | T | 0.00 | 1.075269e-01 |  |
| 342 | 67 | T | T | A | 0.00 | 1.000000e-01 |  |
| 342 | 67 | T | T | C | 0.67 | 2.318082e-02 |  |
| 342 | 67 | T | T | G | 0.00 | 2.197802e-01 |  |
| 342 | 67 | T | T | T | 99.33 | 0.000000e+00 | \* |
| 343 | 68 | C | C | A | 0.00 | 1.000000e-01 |  |
| 343 | 68 | C | C | C | 100.00 | 0.000000e+00 | \* |
| 343 | 68 | C | C | G | 0.00 | 2.197802e-01 |  |
| 343 | 68 | C | C | T | 0.00 | 1.075269e-01 |  |
| 344 | 69 | C | C | A | 0.00 | 1.000000e-01 |  |
| 344 | 69 | C | C | C | 100.00 | 0.000000e+00 | \* |
| 344 | 69 | C | C | G | 0.00 | 2.197802e-01 |  |
| 344 | 69 | C | C | T | 0.00 | 1.075269e-01 |  |
| 345 | 70 | C | C | A | 0.00 | 1.000000e-01 |  |
| 345 | 70 | C | C | C | 100.00 | 0.000000e+00 | \* |
| 345 | 70 | C | C | G | 0.00 | 2.197802e-01 |  |
| 345 | 70 | C | C | T | 0.00 | 1.075269e-01 |  |
| 346 | 71 | G | G | A | 0.00 | 1.000000e-01 |  |
| 346 | 71 | G | G | C | 0.58 | 4.215573e-02 |  |
| 346 | 71 | G | G | G | 99.42 | 0.000000e+00 | \* |
| 346 | 71 | G | G | T | 0.00 | 1.075269e-01 |  |
| 347 | 72 | C | C | A | 0.00 | 1.000000e-01 |  |
| 347 | 72 | C | C | C | 99.45 | 0.000000e+00 | \* |
| 347 | 72 | C | C | G | 0.55 | 7.147713e-02 |  |
| 347 | 72 | C | C | T | 0.00 | 1.075269e-01 |  |
| 348 | 73 | G | G | A | 0.00 | 1.000000e-01 |  |
| 348 | 73 | G | G | C | 0.00 | 1.666667e-01 |  |
| 348 | 73 | G | G | G | 100.00 | 0.000000e+00 | \* |
| 348 | 73 | G | G | T | 0.00 | 1.075269e-01 |  |
| 349 | 74 | A | A | A | 99.79 | 0.000000e+00 | \* |
| 349 | 74 | A | A | C | 0.00 | 1.666667e-01 |  |
| 349 | 74 | A | A | G | 0.21 | 2.100350e-01 |  |
| 349 | 74 | A | A | T | 0.00 | 1.075269e-01 |  |
| 350 | 75 | C | C | A | 0.27 | 7.436660e-02 |  |
| 350 | 75 | C | C | C | 99.73 | 0.000000e+00 | \* |
| 350 | 75 | C | C | G | 0.00 | 2.197802e-01 |  |
| 350 | 75 | C | C | T | 0.00 | 1.075269e-01 |  |

## For use in R

If you want to work with the results in R, here is output that you can copy and paste in your terminal to get:

The base information:

```
structure(list(focal.base = c("A", "C", "G", "T"), avg.percsignal = c(98.3955811426134, 
99.8537336014037, 99.7892837039598, 99.7200772625454), avg.areasignal = c(381.117647058824, 
380.208955223881, 392.160714285714, 361.727272727273), crit.perc.area = c(0.50326013213382, 
0.778352808407571, 0.850934100685729, 0.589603310602601), mu = c(0.349899059632134, 
0.472694004750469, 0.486464643791465, 0.353456806585167), fillibens = c(0.994854265210515, 
0.994624271971976, 0.994396856950252, 0.996361371073565)), .Names = c("focal.base", 
"avg.percsignal", "avg.areasignal", "crit.perc.area", "mu", "fillibens"
), row.names = c(NA, -4L), class = "data.frame")
```

the data.frame that contains information on the guide region:

```
structure(list(A.area = c(317, 366, 3, 468, 0, 0, 0, 0, 0, 0, 
183, 2, 473, 0, 0, 0, 0, 0, 0, 0, 0, 0, 0, 0, 1, 251, 0, 0, 423, 
0, 286, 0, 0, 0, 0, 0, 310, 1, 0, 0, 0, 2, 544, 1, 0, 0, 505, 
0, 0, 0, 1, 240, 476, 3, 433, 441, 1, 0, 0, 0, 2, 538, 2, 295, 
1, 0, 0, 0, 0, 0, 0, 0, 0, 468, 1), C.area = c(0, 0, 0, 0, 361, 
0, 0, 0, 283, 567, 1, 0, 0, 423, 389, 0, 0, 0, 0, 0, 0, 0, 0, 
0, 371, 0, 426, 0, 0, 400, 0, 392, 0, 0, 2, 328, 2, 436, 426, 
373, 1, 0, 0, 318, 322, 0, 0, 339, 0, 0, 296, 0, 0, 0, 0, 0, 
0, 0, 0, 403, 0, 2, 436, 0, 0, 372, 3, 391, 453, 363, 2, 361, 
0, 0, 371), G.area = c(0, 0, 298, 0, 0, 0, 503, 401, 1, 0, 1, 
274, 1, 0, 0, 414, 2, 462, 0, 0, 2, 484, 3, 447, 1, 0, 0, 0, 
0, 0, 0, 0, 364, 507, 360, 1, 0, 0, 0, 0, 1, 449, 0, 0, 0, 346, 
0, 0, 302, 553, 0, 0, 0, 288, 0, 0, 343, 1, 0, 0, 235, 0, 0, 
1, 291, 0, 0, 0, 0, 0, 345, 2, 322, 1, 0), T.area = c(0, 0, 0, 
0, 0, 346, 0, 0, 0, 0, 171, 0, 0, 0, 0, 1, 279, 2, 351, 363, 
375, 2, 300, 0, 0, 0, 0, 396, 0, 0, 0, 0, 0, 0, 0, 0, 0, 0, 0, 
1, 290, 0, 0, 0, 0, 0, 0, 0, 0, 0, 0, 0, 0, 0, 0, 0, 0, 352, 
433, 0, 0, 0, 0, 0, 0, 0, 447, 0, 0, 0, 0, 0, 0, 0, 0), Tot.area = c(317, 
366, 301, 468, 361, 346, 503, 401, 284, 567, 356, 276, 474, 423, 
389, 415, 281, 464, 351, 363, 377, 486, 303, 447, 373, 251, 426, 
396, 423, 400, 286, 392, 364, 507, 362, 329, 312, 437, 426, 374, 
292, 451, 544, 319, 322, 346, 505, 339, 302, 553, 297, 240, 476, 
291, 433, 441, 344, 353, 433, 403, 237, 540, 438, 296, 292, 372, 
450, 391, 453, 363, 347, 363, 322, 469, 372), A.perc = c(100, 
100, 0.996677740863787, 100, 0, 0, 0, 0, 0, 0, 51.4044943820225, 
0.72463768115942, 99.789029535865, 0, 0, 0, 0, 0, 0, 0, 0, 0, 
0, 0, 0.268096514745308, 100, 0, 0, 100, 0, 100, 0, 0, 0, 0, 
0, 99.3589743589744, 0.22883295194508, 0, 0, 0, 0.443458980044346, 
100, 0.313479623824451, 0, 0, 100, 0, 0, 0, 0.336700336700337, 
100, 100, 1.03092783505155, 100, 100, 0.290697674418605, 0, 0, 
0, 0.843881856540084, 99.6296296296296, 0.45662100456621, 99.6621621621622, 
0.342465753424658, 0, 0, 0, 0, 0, 0, 0, 0, 99.7867803837953, 
0.268817204301075), C.perc = c(0, 0, 0, 0, 100, 0, 0, 0, 99.6478873239437, 
100, 0.280898876404494, 0, 0, 100, 100, 0, 0, 0, 0, 0, 0, 0, 
0, 0, 99.4638069705094, 0, 100, 0, 0, 100, 0, 100, 0, 0, 0.552486187845304, 
99.6960486322188, 0.641025641025641, 99.7711670480549, 100, 99.7326203208556, 
0.342465753424658, 0, 0, 99.6865203761756, 100, 0, 0, 100, 0, 
0, 99.6632996632997, 0, 0, 0, 0, 0, 0, 0, 0, 100, 0, 0.37037037037037, 
99.5433789954338, 0, 0, 100, 0.666666666666667, 100, 100, 100, 
0.576368876080692, 99.4490358126722, 0, 0, 99.7311827956989), 
    G.perc = c(0, 0, 99.0033222591362, 0, 0, 0, 100, 100, 0.352112676056338, 
    0, 0.280898876404494, 99.2753623188406, 0.210970464135021, 
    0, 0, 99.7590361445783, 0.711743772241993, 99.5689655172414, 
    0, 0, 0.530503978779841, 99.5884773662551, 0.99009900990099, 
    100, 0.268096514745308, 0, 0, 0, 0, 0, 0, 0, 100, 100, 99.4475138121547, 
    0.303951367781155, 0, 0, 0, 0, 0.342465753424658, 99.5565410199557, 
    0, 0, 0, 100, 0, 0, 100, 100, 0, 0, 0, 98.9690721649485, 
    0, 0, 99.7093023255814, 0.28328611898017, 0, 0, 99.1561181434599, 
    0, 0, 0.337837837837838, 99.6575342465753, 0, 0, 0, 0, 0, 
    99.4236311239193, 0.550964187327824, 100, 0.213219616204691, 
    0), T.perc = c(0, 0, 0, 0, 0, 100, 0, 0, 0, 0, 48.0337078651685, 
    0, 0, 0, 0, 0.240963855421687, 99.288256227758, 0.431034482758621, 
    100, 100, 99.4694960212202, 0.411522633744856, 99.009900990099, 
    0, 0, 0, 0, 100, 0, 0, 0, 0, 0, 0, 0, 0, 0, 0, 0, 0.267379679144385, 
    99.3150684931507, 0, 0, 0, 0, 0, 0, 0, 0, 0, 0, 0, 0, 0, 
    0, 0, 0, 99.7167138810198, 100, 0, 0, 0, 0, 0, 0, 0, 99.3333333333333, 
    0, 0, 0, 0, 0, 0, 0, 0), base.call = c("A", "A", "G", "A", 
    "C", "T", "G", "G", "C", "C", "A", "G", "A", "C", "C", "G", 
    "T", "G", "T", "T", "T", "G", "T", "G", "C", "A", "C", "T", 
    "A", "C", "A", "C", "G", "G", "G", "C", "A", "C", "C", "C", 
    "T", "G", "A", "C", "C", "G", "A", "C", "G", "G", "C", "A", 
    "A", "G", "A", "A", "G", "T", "T", "C", "G", "A", "C", "A", 
    "G", "C", "T", "C", "C", "C", "G", "C", "G", "A", "C"), index = 276:350, 
    guide.seq = c("A", "A", "G", "A", "C", "T", "G", "G", "C", 
    "C", "A", "G", "A", "C", "C", "G", "T", "G", "T", "T", "T", 
    "G", "T", "G", "C", "A", "C", "T", "A", "C", "A", "C", "G", 
    "G", "G", "C", "A", "C", "C", "C", "T", "G", "A", "C", "C", 
    "G", "A", "C", "G", "G", "C", "A", "A", "G", "A", "A", "G", 
    "T", "T", "C", "G", "A", "C", "A", "G", "C", "T", "C", "C", 
    "C", "G", "C", "G", "A", "C"), T.pval = c(0.10752688172106, 
    0.10752688172106, 0.10752688172106, 0.10752688172106, 0.10752688172106, 
    0, 0.10752688172106, 0.10752688172106, 0.10752688172106, 
    0.10752688172106, 0, 0.10752688172106, 0.10752688172106, 
    0.10752688172106, 0.10752688172106, 0.0776184279311939, 0, 
    0.0299700705308708, 0, 0, 0, 0.0337652298254634, 0, 0.10752688172106, 
    0.10752688172106, 0.10752688172106, 0.10752688172106, 0, 
    0.10752688172106, 0.10752688172106, 0.10752688172106, 0.10752688172106, 
    0.10752688172106, 0.10752688172106, 0.10752688172106, 0.10752688172106, 
    0.10752688172106, 0.10752688172106, 0.10752688172106, 0.0702418139565999, 
    0, 0.10752688172106, 0.10752688172106, 0.10752688172106, 
    0.10752688172106, 0.10752688172106, 0.10752688172106, 0.10752688172106, 
    0.10752688172106, 0.10752688172106, 0.10752688172106, 0.10752688172106, 
    0.10752688172106, 0.10752688172106, 0.10752688172106, 0.10752688172106, 
    0.10752688172106, 0, 0, 0.10752688172106, 0.10752688172106, 
    0.10752688172106, 0.10752688172106, 0.10752688172106, 0.10752688172106, 
    0.10752688172106, 0, 0.10752688172106, 0.10752688172106, 
    0.10752688172106, 0.10752688172106, 0.10752688172106, 0.10752688172106, 
    0.10752688172106, 0.10752688172106), C.pval = c(0.166666666666667, 
    0.166666666666667, 0.166666666666667, 0.166666666666667, 
    0, 0.166666666666667, 0.166666666666667, 0.166666666666667, 
    0, 0, 0.145154305485346, 0.166666666666667, 0.166666666666667, 
    0, 0, 0.166666666666667, 0.166666666666667, 0.166666666666667, 
    0.166666666666667, 0.166666666666667, 0.166666666666667, 
    0.166666666666667, 0.166666666666667, 0.166666666666667, 
    0, 0.166666666666667, 0, 0.166666666666667, 0.166666666666667, 
    0, 0.166666666666667, 0, 0.166666666666667, 0.166666666666667, 
    0.0486801329676545, 0, 0.0276961831036362, 0, 0, 0, 0.125090511448809, 
    0.166666666666667, 0.166666666666667, 0, 0, 0.166666666666667, 
    0.166666666666667, 0, 0.166666666666667, 0.166666666666667, 
    0, 0.166666666666667, 0.166666666666667, 0.166666666666667, 
    0.166666666666667, 0.166666666666667, 0.166666666666667, 
    0.166666666666667, 0.166666666666667, 0, 0.166666666666667, 
    0.114573764961854, 0, 0.166666666666667, 0.166666666666667, 
    0, 0.0231808245212399, 0, 0, 0, 0.0421557336128058, 0, 0.166666666666667, 
    0.166666666666667, 0), G.pval = c(0.219780219783574, 0.219780219783574, 
    0, 0.219780219783574, 0.219780219783574, 0.219780219783574, 
    0, 0, 0.162651557445394, 0.219780219783574, 0.191851170775196, 
    0, 0.210454369676277, 0.219780219783574, 0.219780219783574, 
    0, 0.027093432487501, 0, 0.219780219783574, 0.219780219783574, 
    0.0794847985178313, 0, 0.00330917882763226, 0, 0.196102705092246, 
    0.219780219783574, 0.219780219783574, 0.219780219783574, 
    0.219780219783574, 0.219780219783574, 0.219780219783574, 
    0.219780219783574, 0, 0, 0, 0.183336492436003, 0.219780219783574, 
    0.219780219783574, 0.219780219783574, 0.219780219783574, 
    0.16704715038484, 0, 0.219780219783574, 0.219780219783574, 
    0.219780219783574, 0, 0.219780219783574, 0.219780219783574, 
    0, 0, 0.219780219783574, 0.219780219783574, 0.219780219783574, 
    0, 0.219780219783574, 0.219780219783574, 0, 0.191019275168572, 
    0.219780219783574, 0.219780219783574, 0, 0.219780219783574, 
    0.219780219783574, 0.169117430556467, 0, 0.219780219783574, 
    0.219780219783574, 0.219780219783574, 0.219780219783574, 
    0.219780219783574, 0, 0.0714771263379099, 0, 0.210035006846183, 
    0.219780219783574), A.pval = c(0, 0, 3.9968577945082e-06, 
    0, 0.100000000002778, 0.100000000002778, 0.100000000002778, 
    0.100000000002778, 0.100000000002778, 0.100000000002778, 
    0, 0.000442253750362331, 0, 0.100000000002778, 0.100000000002778, 
    0.100000000002778, 0.100000000002778, 0.100000000002778, 
    0.100000000002778, 0.100000000002778, 0.100000000002778, 
    0.100000000002778, 0.100000000002778, 0.100000000002778, 
    0.074606123077675, 0, 0.100000000002778, 0.100000000002778, 
    0, 0.100000000002778, 0, 0.100000000002778, 0.100000000002778, 
    0.100000000002778, 0.100000000002778, 0.100000000002778, 
    0, 0.0862375476877258, 0.100000000002778, 0.100000000002778, 
    0.100000000002778, 0.0195703883376432, 0, 0.0585827862090303, 
    0.100000000002778, 0.100000000002778, 0, 0.100000000002778, 
    0.100000000002778, 0.100000000002778, 0.0502264539480344, 
    0, 0, 2.11123033511118e-06, 0, 0, 0.066803013065347, 0.100000000002778, 
    0.100000000002778, 0.100000000002778, 6.14756893076995e-05, 
    0, 0.0170155245246102, 0, 0.0481962788212883, 0.100000000002778, 
    0.100000000002778, 0.100000000002778, 0.100000000002778, 
    0.100000000002778, 0.100000000002778, 0.100000000002778, 
    0.100000000002778, 0, 0.0743665955999175), guide.position = 1:75), .Names = c("A.area", 
"C.area", "G.area", "T.area", "Tot.area", "A.perc", "C.perc", 
"G.perc", "T.perc", "base.call", "index", "guide.seq", "T.pval", 
"C.pval", "G.pval", "A.pval", "guide.position"), row.names = 276:350, class = "data.frame")
```

*Report generated using EditR v1.0.8*
