# Supplementary material for: Mechanistic and genetic basis of single-strand templated repair at Cas12a-induced DNA breaks in Chlamydomonas reinhardtii
Source: Nat Commun. 2021 Nov 19;12:6751. doi: 10.1038/s41467-021-27004-1 (PMC8604939; doi:10.1038/s41467-021-27004-1)
Supplement: Supplementary file 22 — Source Data [file 41467_2021_27004_MOESM22_ESM.zip › Source Data/EditR analysis/EditR outputs/Sense/rep3_ssODN_sense_-32_-16_0.html]

EditR v1.0.8 report


# EditR v1.0.8 report

- Data QA
  - Filtering data
  - Percent noise peak area
  - Base information
- Predicted editing
  - Editing bar plot
  - Editing table plot
  - Table of editing results
- For use in R

## Data QA

### Filtering data

What the data looked like prefiltering:

and the post filtering signal / noise plot:

### Percent noise peak area

### Base information

Here’s information about the signal of each base, the critical percent value where any higher value would be called as significant, and Filliben’s correlation for how well the noise was modelled by the zero adjusted gamma distribution.

| Base | Average percent signal | Average peak area | Critical percent value | model mu | Fillibens correlation |
| --- | --- | --- | --- | --- | --- |
| A | 89.84888 | 91.82353 | 11.653302 | 3.968094 | 0.9946797 |
| C | 89.86851 | 99.29851 | 8.636476 | 3.370787 | 0.9921733 |
| G | 90.96461 | 91.03509 | 11.713197 | 4.119420 | 0.9940832 |
| T | 92.23705 | 108.72093 | 10.768540 | 3.676828 | 0.9884182 |

## Predicted editing

### Editing bar plot

### Editing table plot

### Table of editing results


Here’s the entire guide region

| Sanger position | Guide position | Guide sequence | Sanger base call | Focal base | Focal base peak area | p value |  |
| --- | --- | --- | --- | --- | --- | --- | --- |
| 277 | 1 | A | A | A | 92.86 | 0.000000000 | \* |
| 277 | 1 | A | A | C | 5.95 | 0.075152769 |  |
| 277 | 1 | A | A | G | 1.19 | 0.719804172 |  |
| 277 | 1 | A | A | T | 0.00 | 0.872340426 |  |
| 278 | 2 | A | A | A | 86.27 | 0.000000000 | \* |
| 278 | 2 | A | A | C | 8.82 | 0.008613974 | \* |
| 278 | 2 | A | A | G | 1.96 | 0.624214758 |  |
| 278 | 2 | A | A | T | 2.94 | 0.481115308 |  |
| 279 | 3 | G | G | A | 2.70 | 0.532713863 |  |
| 279 | 3 | G | G | C | 10.81 | 0.001679338 | \* |
| 279 | 3 | G | G | G | 86.49 | 0.000000000 | \* |
| 279 | 3 | G | G | T | 0.00 | 0.872340426 |  |
| 280 | 4 | A | A | A | 94.27 | 0.000000000 | \* |
| 280 | 4 | A | A | C | 1.56 | 0.741158257 |  |
| 280 | 4 | A | A | G | 0.52 | 0.768797392 |  |
| 280 | 4 | A | A | T | 3.65 | 0.368014715 |  |
| 281 | 5 | C | C | A | 5.94 | 0.156654719 |  |
| 281 | 5 | C | C | C | 86.14 | 0.000000000 | \* |
| 281 | 5 | C | C | G | 5.94 | 0.157723998 |  |
| 281 | 5 | C | C | T | 1.98 | 0.654695654 |  |
| 282 | 6 | T | T | A | 5.61 | 0.180726907 |  |
| 282 | 6 | T | T | C | 1.87 | 0.682882176 |  |
| 282 | 6 | T | T | G | 5.61 | 0.181455881 |  |
| 282 | 6 | T | T | T | 86.92 | 0.000000000 | \* |
| 283 | 7 | G | G | A | 5.79 | 0.167208953 |  |
| 283 | 7 | G | G | C | 1.05 | 0.815075575 |  |
| 283 | 7 | G | G | G | 92.11 | 0.000000000 | \* |
| 283 | 7 | G | G | T | 1.05 | 0.805495627 |  |
| 284 | 8 | G | G | A | 6.56 | 0.119379742 |  |
| 284 | 8 | G | G | C | 2.46 | 0.556907577 |  |
| 284 | 8 | G | G | G | 90.98 | 0.000000000 | \* |
| 284 | 8 | G | G | T | 0.00 | 0.872340426 |  |
| 285 | 9 | C | C | A | 4.55 | 0.279052284 |  |
| 285 | 9 | C | C | C | 77.27 | 0.000000000 | \* |
| 285 | 9 | C | C | G | 12.50 | 0.006654244 | \* |
| 285 | 9 | C | C | T | 5.68 | 0.148904599 |  |
| 286 | 10 | C | C | A | 1.65 | 0.699742915 |  |
| 286 | 10 | C | C | C | 83.47 | 0.000000000 | \* |
| 286 | 10 | C | C | G | 10.74 | 0.016391496 |  |
| 286 | 10 | C | C | T | 4.13 | 0.301063007 |  |
| 287 | 11 | A | A | A | 77.65 | 0.000000000 | \* |
| 287 | 11 | A | A | C | 2.35 | 0.580104102 |  |
| 287 | 11 | A | A | G | 9.41 | 0.031803565 |  |
| 287 | 11 | A | A | T | 10.59 | 0.011074412 |  |
| 288 | 12 | G | G | A | 3.37 | 0.429794102 |  |
| 288 | 12 | G | G | C | 2.25 | 0.603147599 |  |
| 288 | 12 | G | G | G | 94.38 | 0.000000000 | \* |
| 288 | 12 | G | G | T | 0.00 | 0.872340426 |  |
| 289 | 13 | A | A | A | 92.93 | 0.000000000 | \* |
| 289 | 13 | A | A | C | 2.17 | 0.619002109 |  |
| 289 | 13 | A | A | G | 1.63 | 0.668703056 |  |
| 289 | 13 | A | A | T | 3.26 | 0.427624003 |  |
| 290 | 14 | C | C | A | 4.81 | 0.251507323 |  |
| 290 | 14 | C | C | C | 93.27 | 0.000000000 | \* |
| 290 | 14 | C | C | G | 0.00 | 0.777777778 |  |
| 290 | 14 | C | C | T | 1.92 | 0.665052772 |  |
| 291 | 15 | C | C | A | 6.90 | 0.102447842 |  |
| 291 | 15 | C | C | C | 82.76 | 0.000000000 | \* |
| 291 | 15 | C | C | G | 10.34 | 0.020033520 |  |
| 291 | 15 | C | C | T | 0.00 | 0.872340426 |  |
| 292 | 16 | G | G | A | 6.32 | 0.132923867 |  |
| 292 | 16 | G | G | C | 1.05 | 0.815075575 |  |
| 292 | 16 | G | G | G | 91.58 | 0.000000000 | \* |
| 292 | 16 | G | G | T | 1.05 | 0.805495627 |  |
| 293 | 17 | T | T | A | 0.00 | 0.836363636 |  |
| 293 | 17 | T | T | C | 0.00 | 0.857142857 |  |
| 293 | 17 | T | T | G | 1.39 | 0.698196896 |  |
| 293 | 17 | T | T | T | 98.61 | 0.000000000 | \* |
| 294 | 18 | G | G | A | 4.05 | 0.336736340 |  |
| 294 | 18 | G | G | C | 1.35 | 0.775872504 |  |
| 294 | 18 | G | G | G | 93.92 | 0.000000000 | \* |
| 294 | 18 | G | G | T | 0.68 | 0.846459909 |  |
| 295 | 19 | T | T | A | 1.10 | 0.773865808 |  |
| 295 | 19 | T | T | C | 0.00 | 0.857142857 |  |
| 295 | 19 | T | T | G | 9.89 | 0.025126842 |  |
| 295 | 19 | T | T | T | 89.01 | 0.000000000 | \* |
| 296 | 20 | T | T | A | 1.06 | 0.777839819 |  |
| 296 | 20 | T | T | C | 0.00 | 0.857142857 |  |
| 296 | 20 | T | T | G | 6.38 | 0.130345852 |  |
| 296 | 20 | T | T | T | 92.55 | 0.000000000 | \* |
| 297 | 21 | T | T | A | 0.00 | 0.836363636 |  |
| 297 | 21 | T | T | C | 2.22 | 0.608563138 |  |
| 297 | 21 | T | T | G | 3.33 | 0.425971315 |  |
| 297 | 21 | T | T | T | 94.44 | 0.000000000 | \* |
| 298 | 22 | G | G | A | 1.75 | 0.684439759 |  |
| 298 | 22 | G | G | C | 1.75 | 0.705595391 |  |
| 298 | 22 | G | G | G | 96.49 | 0.000000000 | \* |
| 298 | 22 | G | G | T | 0.00 | 0.872340426 |  |
| 299 | 23 | T | T | A | 0.00 | 0.836363636 |  |
| 299 | 23 | T | T | C | 1.30 | 0.783681562 |  |
| 299 | 23 | T | T | G | 2.60 | 0.531805554 |  |
| 299 | 23 | T | T | T | 96.10 | 0.000000000 | \* |
| 300 | 24 | G | G | A | 2.17 | 0.618219924 |  |
| 300 | 24 | G | G | C | 1.09 | 0.811226228 |  |
| 300 | 24 | G | G | G | 94.57 | 0.000000000 | \* |
| 300 | 24 | G | G | T | 2.17 | 0.619255269 |  |
| 301 | 25 | C | C | A | 2.44 | 0.575261890 |  |
| 301 | 25 | C | C | C | 89.02 | 0.000000000 | \* |
| 301 | 25 | C | C | G | 0.00 | 0.777777778 |  |
| 301 | 25 | C | C | T | 8.54 | 0.034438250 |  |
| 302 | 26 | A | A | A | 82.19 | 0.000000000 | \* |
| 302 | 26 | A | A | C | 2.74 | 0.495958963 |  |
| 302 | 26 | A | A | G | 9.59 | 0.029153660 |  |
| 302 | 26 | A | A | T | 5.48 | 0.163998530 |  |
| 303 | 27 | C | C | A | 4.55 | 0.279052284 |  |
| 303 | 27 | C | C | C | 84.09 | 0.000000000 | \* |
| 303 | 27 | C | C | G | 10.23 | 0.021246570 |  |
| 303 | 27 | C | C | T | 1.14 | 0.794260626 |  |
| 304 | 28 | T | T | A | 1.28 | 0.751526360 |  |
| 304 | 28 | T | T | C | 5.13 | 0.131192565 |  |
| 304 | 28 | T | T | G | 0.00 | 0.777777778 |  |
| 304 | 28 | T | T | T | 93.59 | 0.000000000 | \* |
| 305 | 29 | A | A | A | 89.89 | 0.000000000 | \* |
| 305 | 29 | A | A | C | 4.49 | 0.195786575 |  |
| 305 | 29 | A | A | G | 3.37 | 0.420814603 |  |
| 305 | 29 | A | A | T | 2.25 | 0.605790278 |  |
| 306 | 30 | C | C | A | 2.50 | 0.565380024 |  |
| 306 | 30 | C | C | C | 93.75 | 0.000000000 | \* |
| 306 | 30 | C | C | G | 1.25 | 0.713603767 |  |
| 306 | 30 | C | C | T | 2.50 | 0.559499044 |  |
| 307 | 31 | A | A | A | 86.15 | 0.000000000 | \* |
| 307 | 31 | A | A | C | 1.54 | 0.745363916 |  |
| 307 | 31 | A | A | G | 10.77 | 0.016182290 |  |
| 307 | 31 | A | A | T | 1.54 | 0.732346985 |  |
| 308 | 32 | C | C | A | 3.70 | 0.382664284 |  |
| 308 | 32 | C | C | C | 83.95 | 0.000000000 | \* |
| 308 | 32 | C | C | G | 4.94 | 0.238083160 |  |
| 308 | 32 | C | C | T | 7.41 | 0.062667005 |  |
| 309 | 33 | G | G | A | 10.34 | 0.019475202 |  |
| 309 | 33 | G | G | C | 3.45 | 0.353872303 |  |
| 309 | 33 | G | G | G | 86.21 | 0.000000000 | \* |
| 309 | 33 | G | G | T | 0.00 | 0.872340426 |  |
| 310 | 34 | G | G | A | 0.00 | 0.836363636 |  |
| 310 | 34 | G | G | C | 1.04 | 0.816267706 |  |
| 310 | 34 | G | G | G | 96.88 | 0.000000000 | \* |
| 310 | 34 | G | G | T | 2.08 | 0.635870988 |  |
| 311 | 35 | G | G | A | 2.82 | 0.514513916 |  |
| 311 | 35 | G | G | C | 1.41 | 0.767003372 |  |
| 311 | 35 | G | G | G | 95.77 | 0.000000000 | \* |
| 311 | 35 | G | G | T | 0.00 | 0.872340426 |  |
| 312 | 36 | C | C | A | 4.29 | 0.308551833 |  |
| 312 | 36 | C | C | C | 90.00 | 0.000000000 | \* |
| 312 | 36 | C | C | G | 0.00 | 0.777777778 |  |
| 312 | 36 | C | C | T | 5.71 | 0.146599491 |  |
| 313 | 37 | A | A | A | 88.73 | 0.000000000 | \* |
| 313 | 37 | A | A | C | 1.41 | 0.767003372 |  |
| 313 | 37 | A | A | G | 7.04 | 0.097274089 |  |
| 313 | 37 | A | A | T | 2.82 | 0.502742405 |  |
| 314 | 38 | C | C | A | 2.97 | 0.490395471 |  |
| 314 | 38 | C | C | C | 89.11 | 0.000000000 | \* |
| 314 | 38 | C | C | G | 4.95 | 0.236935781 |  |
| 314 | 38 | C | C | T | 2.97 | 0.476109819 |  |
| 315 | 39 | C | C | A | 7.69 | 0.070944339 |  |
| 315 | 39 | C | C | C | 80.77 | 0.000000000 | \* |
| 315 | 39 | C | C | G | 7.69 | 0.072255019 |  |
| 315 | 39 | C | C | T | 3.85 | 0.339280833 |  |
| 316 | 40 | C | C | A | 10.42 | 0.018783270 |  |
| 316 | 40 | C | C | C | 87.50 | 0.000000000 | \* |
| 316 | 40 | C | C | G | 1.04 | 0.734134875 |  |
| 316 | 40 | C | C | T | 1.04 | 0.806915724 |  |
| 317 | 41 | T | T | A | 0.00 | 0.836363636 |  |
| 317 | 41 | T | T | C | 2.53 | 0.541037676 |  |
| 317 | 41 | T | T | G | 2.53 | 0.541490164 |  |
| 317 | 41 | T | T | T | 94.94 | 0.000000000 | \* |
| 318 | 42 | G | G | A | 2.78 | 0.520728243 |  |
| 318 | 42 | G | G | C | 2.78 | 0.487817132 |  |
| 318 | 42 | G | G | G | 93.52 | 0.000000000 | \* |
| 318 | 42 | G | G | T | 0.93 | 0.821130347 |  |
| 319 | 43 | A | A | A | 88.12 | 0.000000000 | \* |
| 319 | 43 | A | A | C | 0.99 | 0.821628442 |  |
| 319 | 43 | A | A | G | 0.99 | 0.738683487 |  |
| 319 | 43 | A | A | T | 9.90 | 0.016287733 |  |
| 320 | 44 | C | C | A | 3.85 | 0.363519124 |  |
| 320 | 44 | C | C | C | 96.15 | 0.000000000 | \* |
| 320 | 44 | C | C | G | 0.00 | 0.777777778 |  |
| 320 | 44 | C | C | T | 0.00 | 0.872340426 |  |
| 321 | 45 | C | C | A | 0.00 | 0.836363636 |  |
| 321 | 45 | C | C | C | 87.50 | 0.000000000 | \* |
| 321 | 45 | C | C | G | 8.33 | 0.053494434 |  |
| 321 | 45 | C | C | T | 4.17 | 0.296687355 |  |
| 322 | 46 | G | G | A | 6.15 | 0.142748475 |  |
| 322 | 46 | G | G | C | 3.08 | 0.425520200 |  |
| 322 | 46 | G | G | G | 90.77 | 0.000000000 | \* |
| 322 | 46 | G | G | T | 0.00 | 0.872340426 |  |
| 323 | 47 | A | A | A | 96.75 | 0.000000000 | \* |
| 323 | 47 | A | A | C | 0.81 | 0.836918072 |  |
| 323 | 47 | A | A | G | 0.00 | 0.777777778 |  |
| 323 | 47 | A | A | T | 2.44 | 0.570614305 |  |
| 324 | 48 | C | C | A | 3.85 | 0.363519124 |  |
| 324 | 48 | C | C | C | 92.31 | 0.000000000 | \* |
| 324 | 48 | C | C | G | 0.00 | 0.777777778 |  |
| 324 | 48 | C | C | T | 3.85 | 0.339280833 |  |
| 325 | 49 | G | G | A | 0.00 | 0.836363636 |  |
| 325 | 49 | G | G | C | 2.50 | 0.547948206 |  |
| 325 | 49 | G | G | G | 97.50 | 0.000000000 | \* |
| 325 | 49 | G | G | T | 0.00 | 0.872340426 |  |
| 326 | 50 | G | G | A | 3.03 | 0.481081553 |  |
| 326 | 50 | G | G | C | 1.01 | 0.819597419 |  |
| 326 | 50 | G | G | G | 93.94 | 0.000000000 | \* |
| 326 | 50 | G | G | T | 2.02 | 0.647410133 |  |
| 327 | 51 | C | C | A | 1.69 | 0.693453360 |  |
| 327 | 51 | C | C | C | 91.53 | 0.000000000 | \* |
| 327 | 51 | C | C | G | 1.69 | 0.660342980 |  |
| 327 | 51 | C | C | T | 5.08 | 0.197265536 |  |
| 328 | 52 | A | A | A | 86.84 | 0.000000000 | \* |
| 328 | 52 | A | A | C | 0.00 | 0.857142857 |  |
| 328 | 52 | A | A | G | 2.63 | 0.526774721 |  |
| 328 | 52 | A | A | T | 10.53 | 0.011468517 |  |
| 329 | 53 | A | A | A | 95.65 | 0.000000000 | \* |
| 329 | 53 | A | A | C | 1.45 | 0.760417809 |  |
| 329 | 53 | A | A | G | 1.45 | 0.691121491 |  |
| 329 | 53 | A | A | T | 1.45 | 0.747030286 |  |
| 330 | 54 | G | G | A | 2.00 | 0.646099622 |  |
| 330 | 54 | G | G | C | 0.00 | 0.857142857 |  |
| 330 | 54 | G | G | G | 94.00 | 0.000000000 | \* |
| 330 | 54 | G | G | T | 4.00 | 0.318314028 |  |
| 331 | 55 | A | A | A | 92.31 | 0.000000000 | \* |
| 331 | 55 | A | A | C | 1.10 | 0.809845215 |  |
| 331 | 55 | A | A | G | 1.10 | 0.728829110 |  |
| 331 | 55 | A | A | T | 5.49 | 0.162831683 |  |
| 332 | 56 | A | A | A | 90.67 | 0.000000000 | \* |
| 332 | 56 | A | A | C | 1.33 | 0.778585605 |  |
| 332 | 56 | A | A | G | 0.00 | 0.777777778 |  |
| 332 | 56 | A | A | T | 8.00 | 0.045893774 |  |
| 333 | 57 | G | G | A | 2.27 | 0.602236827 |  |
| 333 | 57 | G | G | C | 2.27 | 0.597597142 |  |
| 333 | 57 | G | G | G | 95.45 | 0.000000000 | \* |
| 333 | 57 | G | G | T | 0.00 | 0.872340426 |  |
| 334 | 58 | T | T | A | 0.00 | 0.836363636 |  |
| 334 | 58 | T | T | C | 0.00 | 0.857142857 |  |
| 334 | 58 | T | T | G | 1.37 | 0.700380772 |  |
| 334 | 58 | T | T | T | 98.63 | 0.000000000 | \* |
| 335 | 59 | T | T | A | 0.00 | 0.836363636 |  |
| 335 | 59 | T | T | C | 1.35 | 0.775872504 |  |
| 335 | 59 | T | T | G | 1.35 | 0.702484032 |  |
| 335 | 59 | T | T | T | 97.30 | 0.000000000 | \* |
| 336 | 60 | C | C | A | 2.86 | 0.508147083 |  |
| 336 | 60 | C | C | C | 91.43 | 0.000000000 | \* |
| 336 | 60 | C | C | G | 1.43 | 0.693571493 |  |
| 336 | 60 | C | C | T | 4.29 | 0.281930628 |  |
| 337 | 61 | G | G | A | 4.00 | 0.343565550 |  |
| 337 | 61 | G | G | C | 4.00 | 0.262108996 |  |
| 337 | 61 | G | G | G | 86.00 | 0.000000000 | \* |
| 337 | 61 | G | G | T | 6.00 | 0.127631672 |  |
| 338 | 62 | A | A | A | 90.65 | 0.000000000 | \* |
| 338 | 62 | A | A | C | 0.93 | 0.826943208 |  |
| 338 | 62 | A | A | G | 1.87 | 0.636929781 |  |
| 338 | 62 | A | A | T | 6.54 | 0.097561585 |  |
| 339 | 63 | C | C | A | 1.20 | 0.761255743 |  |
| 339 | 63 | C | C | C | 90.36 | 0.000000000 | \* |
| 339 | 63 | C | C | G | 1.20 | 0.718333458 |  |
| 339 | 63 | C | C | T | 7.23 | 0.068744354 |  |
| 340 | 64 | A | A | A | 91.11 | 0.000000000 | \* |
| 340 | 64 | A | A | C | 4.44 | 0.201818519 |  |
| 340 | 64 | A | A | G | 2.22 | 0.586848189 |  |
| 340 | 64 | A | A | T | 2.22 | 0.610379000 |  |
| 341 | 65 | G | G | A | 1.32 | 0.747145564 |  |
| 341 | 65 | G | G | C | 2.63 | 0.519290629 |  |
| 341 | 65 | G | G | G | 94.74 | 0.000000000 | \* |
| 341 | 65 | G | G | T | 1.32 | 0.768082822 |  |
| 342 | 66 | C | C | A | 1.45 | 0.729106342 |  |
| 342 | 66 | C | C | C | 94.20 | 0.000000000 | \* |
| 342 | 66 | C | C | G | 1.45 | 0.691121491 |  |
| 342 | 66 | C | C | T | 2.90 | 0.488485935 |  |
| 343 | 67 | T | T | A | 0.00 | 0.836363636 |  |
| 343 | 67 | T | T | C | 2.50 | 0.547948206 |  |
| 343 | 67 | T | T | G | 2.50 | 0.546150801 |  |
| 343 | 67 | T | T | T | 95.00 | 0.000000000 | \* |
| 344 | 68 | C | C | A | 0.00 | 0.836363636 |  |
| 344 | 68 | C | C | C | 95.95 | 0.000000000 | \* |
| 344 | 68 | C | C | G | 2.70 | 0.516318300 |  |
| 344 | 68 | C | C | T | 1.35 | 0.762593128 |  |
| 345 | 69 | C | C | A | 0.00 | 0.836363636 |  |
| 345 | 69 | C | C | C | 94.51 | 0.000000000 | \* |
| 345 | 69 | C | C | G | 2.20 | 0.590390443 |  |
| 345 | 69 | C | C | T | 3.30 | 0.421842529 |  |
| 346 | 70 | C | C | A | 0.00 | 0.836363636 |  |
| 346 | 70 | C | C | C | 96.08 | 0.000000000 | \* |
| 346 | 70 | C | C | G | 3.92 | 0.348818946 |  |
| 346 | 70 | C | C | T | 0.00 | 0.872340426 |  |
| 347 | 71 | G | G | A | 3.51 | 0.409868959 |  |
| 347 | 71 | G | G | C | 5.26 | 0.120068360 |  |
| 347 | 71 | G | G | G | 89.47 | 0.000000000 | \* |
| 347 | 71 | G | G | T | 1.75 | 0.695201239 |  |
| 348 | 72 | C | C | A | 5.56 | 0.184750219 |  |
| 348 | 72 | C | C | C | 88.89 | 0.000000000 | \* |
| 348 | 72 | C | C | G | 1.39 | 0.698196896 |  |
| 348 | 72 | C | C | T | 4.17 | 0.296687355 |  |
| 349 | 73 | G | G | A | 9.26 | 0.033415510 |  |
| 349 | 73 | G | G | C | 5.56 | 0.098734242 |  |
| 349 | 73 | G | G | G | 83.33 | 0.000000000 | \* |
| 349 | 73 | G | G | T | 1.85 | 0.677873264 |  |
| 350 | 74 | A | A | A | 92.63 | 0.000000000 | \* |
| 350 | 74 | A | A | C | 1.05 | 0.815075575 |  |
| 350 | 74 | A | A | G | 1.05 | 0.733139054 |  |
| 350 | 74 | A | A | T | 5.26 | 0.181577732 |  |
| 351 | 75 | C | C | A | 1.20 | 0.761255743 |  |
| 351 | 75 | C | C | C | 93.98 | 0.000000000 | \* |
| 351 | 75 | C | C | G | 1.20 | 0.718333458 |  |
| 351 | 75 | C | C | T | 3.61 | 0.372660172 |  |

## For use in R

If you want to work with the results in R, here is output that you can copy and paste in your terminal to get:

The base information:

```
structure(list(focal.base = c("A", "C", "G", "T"), avg.percsignal = c(89.8488753697809, 
89.8685116839085, 90.9646097144038, 92.2370454626689), avg.areasignal = c(91.8235294117647, 
99.2985074626866, 91.0350877192982, 108.720930232558), crit.perc.area = c(11.6533020151859, 
8.63647597947936, 11.7131969162733, 10.7685399599599), mu = c(3.96809419371011, 
3.37078719977564, 4.11941979473029, 3.67682775631693), fillibens = c(0.994679739581888, 
0.992173321534197, 0.994083199451064, 0.988418202816357)), .Names = c("focal.base", 
"avg.percsignal", "avg.areasignal", "crit.perc.area", "mu", "fillibens"
), row.names = c(NA, -4L), class = "data.frame")
```

the data.frame that contains information on the guide region:

```
structure(list(A.area = c(78, 88, 2, 181, 6, 6, 11, 8, 4, 2, 
66, 3, 171, 5, 6, 6, 0, 6, 1, 1, 0, 2, 0, 2, 2, 60, 4, 1, 80, 
2, 56, 3, 6, 0, 2, 3, 63, 3, 8, 10, 0, 3, 89, 3, 0, 4, 119, 3, 
0, 3, 1, 33, 66, 1, 84, 68, 1, 0, 0, 2, 2, 97, 1, 41, 1, 1, 0, 
0, 0, 0, 2, 4, 5, 88, 1), C.area = c(5, 9, 8, 3, 87, 2, 2, 3, 
68, 101, 2, 2, 4, 97, 72, 1, 0, 2, 0, 0, 2, 2, 1, 1, 73, 2, 74, 
4, 4, 75, 1, 68, 2, 1, 1, 63, 1, 90, 84, 84, 2, 3, 1, 75, 63, 
2, 1, 72, 1, 1, 54, 0, 1, 0, 1, 1, 1, 0, 1, 64, 2, 1, 75, 2, 
2, 65, 2, 71, 86, 49, 3, 64, 3, 1, 78), G.area = c(1, 2, 64, 
1, 6, 6, 175, 111, 11, 13, 8, 84, 3, 0, 9, 87, 1, 139, 9, 6, 
3, 110, 2, 87, 0, 7, 9, 0, 3, 1, 7, 4, 50, 93, 68, 0, 5, 5, 8, 
1, 2, 101, 1, 0, 6, 59, 0, 0, 39, 93, 1, 1, 1, 47, 1, 0, 42, 
1, 1, 1, 43, 2, 1, 1, 72, 1, 2, 2, 2, 2, 51, 1, 45, 1, 1), T.area = c(0, 
3, 0, 7, 2, 93, 2, 0, 5, 5, 9, 0, 6, 2, 0, 1, 71, 1, 81, 87, 
85, 0, 74, 2, 7, 4, 1, 73, 2, 2, 1, 6, 0, 2, 0, 4, 2, 3, 4, 1, 
75, 1, 10, 0, 3, 0, 3, 3, 0, 2, 3, 4, 1, 2, 5, 6, 0, 72, 72, 
3, 3, 7, 6, 1, 1, 2, 76, 1, 3, 0, 1, 3, 1, 5, 3), Tot.area = c(84, 
102, 74, 192, 101, 107, 190, 122, 88, 121, 85, 89, 184, 104, 
87, 95, 72, 148, 91, 94, 90, 114, 77, 92, 82, 73, 88, 78, 89, 
80, 65, 81, 58, 96, 71, 70, 71, 101, 104, 96, 79, 108, 101, 78, 
72, 65, 123, 78, 40, 99, 59, 38, 69, 50, 91, 75, 44, 73, 74, 
70, 50, 107, 83, 45, 76, 69, 80, 74, 91, 51, 57, 72, 54, 95, 
83), A.perc = c(92.8571428571429, 86.2745098039216, 2.7027027027027, 
94.2708333333333, 5.94059405940594, 5.60747663551402, 5.78947368421053, 
6.55737704918033, 4.54545454545455, 1.65289256198347, 77.6470588235294, 
3.37078651685393, 92.9347826086957, 4.80769230769231, 6.89655172413793, 
6.31578947368421, 0, 4.05405405405405, 1.0989010989011, 1.06382978723404, 
0, 1.75438596491228, 0, 2.17391304347826, 2.4390243902439, 82.1917808219178, 
4.54545454545455, 1.28205128205128, 89.8876404494382, 2.5, 86.1538461538462, 
3.7037037037037, 10.3448275862069, 0, 2.8169014084507, 4.28571428571429, 
88.7323943661972, 2.97029702970297, 7.69230769230769, 10.4166666666667, 
0, 2.77777777777778, 88.1188118811881, 3.84615384615385, 0, 6.15384615384615, 
96.7479674796748, 3.84615384615385, 0, 3.03030303030303, 1.69491525423729, 
86.8421052631579, 95.6521739130435, 2, 92.3076923076923, 90.6666666666667, 
2.27272727272727, 0, 0, 2.85714285714286, 4, 90.6542056074766, 
1.20481927710843, 91.1111111111111, 1.31578947368421, 1.44927536231884, 
0, 0, 0, 0, 3.50877192982456, 5.55555555555556, 9.25925925925926, 
92.6315789473684, 1.20481927710843), C.perc = c(5.95238095238095, 
8.82352941176471, 10.8108108108108, 1.5625, 86.1386138613861, 
1.86915887850467, 1.05263157894737, 2.45901639344262, 77.2727272727273, 
83.4710743801653, 2.35294117647059, 2.24719101123596, 2.17391304347826, 
93.2692307692308, 82.7586206896552, 1.05263157894737, 0, 1.35135135135135, 
0, 0, 2.22222222222222, 1.75438596491228, 1.2987012987013, 1.08695652173913, 
89.0243902439024, 2.73972602739726, 84.0909090909091, 5.12820512820513, 
4.49438202247191, 93.75, 1.53846153846154, 83.9506172839506, 
3.44827586206897, 1.04166666666667, 1.40845070422535, 90, 1.40845070422535, 
89.1089108910891, 80.7692307692308, 87.5, 2.53164556962025, 2.77777777777778, 
0.99009900990099, 96.1538461538462, 87.5, 3.07692307692308, 0.813008130081301, 
92.3076923076923, 2.5, 1.01010101010101, 91.5254237288136, 0, 
1.44927536231884, 0, 1.0989010989011, 1.33333333333333, 2.27272727272727, 
0, 1.35135135135135, 91.4285714285714, 4, 0.934579439252336, 
90.3614457831325, 4.44444444444444, 2.63157894736842, 94.2028985507246, 
2.5, 95.945945945946, 94.5054945054945, 96.078431372549, 5.26315789473684, 
88.8888888888889, 5.55555555555556, 1.05263157894737, 93.9759036144578
), G.perc = c(1.19047619047619, 1.96078431372549, 86.4864864864865, 
0.520833333333333, 5.94059405940594, 5.60747663551402, 92.1052631578947, 
90.983606557377, 12.5, 10.7438016528926, 9.41176470588235, 94.3820224719101, 
1.6304347826087, 0, 10.3448275862069, 91.5789473684211, 1.38888888888889, 
93.9189189189189, 9.89010989010989, 6.38297872340426, 3.33333333333333, 
96.4912280701754, 2.5974025974026, 94.5652173913043, 0, 9.58904109589041, 
10.2272727272727, 0, 3.37078651685393, 1.25, 10.7692307692308, 
4.93827160493827, 86.2068965517241, 96.875, 95.7746478873239, 
0, 7.04225352112676, 4.95049504950495, 7.69230769230769, 1.04166666666667, 
2.53164556962025, 93.5185185185185, 0.99009900990099, 0, 8.33333333333333, 
90.7692307692308, 0, 0, 97.5, 93.9393939393939, 1.69491525423729, 
2.63157894736842, 1.44927536231884, 94, 1.0989010989011, 0, 95.4545454545455, 
1.36986301369863, 1.35135135135135, 1.42857142857143, 86, 1.86915887850467, 
1.20481927710843, 2.22222222222222, 94.7368421052632, 1.44927536231884, 
2.5, 2.7027027027027, 2.1978021978022, 3.92156862745098, 89.4736842105263, 
1.38888888888889, 83.3333333333333, 1.05263157894737, 1.20481927710843
), T.perc = c(0, 2.94117647058824, 0, 3.64583333333333, 1.98019801980198, 
86.9158878504673, 1.05263157894737, 0, 5.68181818181818, 4.13223140495868, 
10.5882352941176, 0, 3.26086956521739, 1.92307692307692, 0, 1.05263157894737, 
98.6111111111111, 0.675675675675676, 89.010989010989, 92.5531914893617, 
94.4444444444444, 0, 96.1038961038961, 2.17391304347826, 8.53658536585366, 
5.47945205479452, 1.13636363636364, 93.5897435897436, 2.24719101123596, 
2.5, 1.53846153846154, 7.40740740740741, 0, 2.08333333333333, 
0, 5.71428571428571, 2.8169014084507, 2.97029702970297, 3.84615384615385, 
1.04166666666667, 94.9367088607595, 0.925925925925926, 9.9009900990099, 
0, 4.16666666666667, 0, 2.4390243902439, 3.84615384615385, 0, 
2.02020202020202, 5.08474576271186, 10.5263157894737, 1.44927536231884, 
4, 5.49450549450549, 8, 0, 98.6301369863014, 97.2972972972973, 
4.28571428571429, 6, 6.54205607476636, 7.2289156626506, 2.22222222222222, 
1.31578947368421, 2.89855072463768, 95, 1.35135135135135, 3.2967032967033, 
0, 1.75438596491228, 4.16666666666667, 1.85185185185185, 5.26315789473684, 
3.6144578313253), base.call = c("A", "A", "G", "A", "C", "T", 
"G", "G", "C", "C", "A", "G", "A", "C", "C", "G", "T", "G", "T", 
"T", "T", "G", "T", "G", "C", "A", "C", "T", "A", "C", "A", "C", 
"G", "G", "G", "C", "A", "C", "C", "C", "T", "G", "A", "C", "C", 
"G", "A", "C", "G", "G", "C", "A", "A", "G", "A", "A", "G", "T", 
"T", "C", "G", "A", "C", "A", "G", "C", "T", "C", "C", "C", "G", 
"C", "G", "A", "C"), index = 277:351, guide.seq = c("A", "A", 
"G", "A", "C", "T", "G", "G", "C", "C", "A", "G", "A", "C", "C", 
"G", "T", "G", "T", "T", "T", "G", "T", "G", "C", "A", "C", "T", 
"A", "C", "A", "C", "G", "G", "G", "C", "A", "C", "C", "C", "T", 
"G", "A", "C", "C", "G", "A", "C", "G", "G", "C", "A", "A", "G", 
"A", "A", "G", "T", "T", "C", "G", "A", "C", "A", "G", "C", "T", 
"C", "C", "C", "G", "C", "G", "A", "C"), T.pval = c(0.872340425531903, 
0.48111530849728, 0.872340425531903, 0.368014715342595, 0.654695653622981, 
0, 0.805495626884271, 0.872340425531903, 0.14890459890737, 0.301063006546832, 
0.0110744118023112, 0.872340425531903, 0.427624002865947, 0.665052771601961, 
0.872340425531903, 0.805495626884271, 0, 0.846459908888796, 0, 
0, 0, 0.872340425531903, 0, 0.619255269333826, 0.0344382498830976, 
0.163998529616814, 0.794260625642973, 0, 0.605790278026685, 0.559499044345495, 
0.732346985263875, 0.0626670046697925, 0.872340425531903, 0.635870987716181, 
0.872340425531903, 0.146599490586567, 0.502742405282042, 0.4761098188972, 
0.339280832686453, 0.806915723510373, 0, 0.821130347128945, 0.0162877329457997, 
0.872340425531903, 0.29668735505418, 0.872340425531903, 0.570614304663827, 
0.339280832686453, 0.872340425531903, 0.647410133362816, 0.197265535957781, 
0.0114685174763901, 0.747030285869628, 0.318314027534644, 0.162831683006865, 
0.0458937737287738, 0.872340425531903, 0, 0, 0.281930628215496, 
0.127631672460856, 0.0975615845974899, 0.0687443538923692, 0.610379000193844, 
0.768082821563898, 0.48848593507349, 0, 0.762593128145213, 0.421842528937163, 
0.872340425531903, 0.695201238951375, 0.29668735505418, 0.677873264433125, 
0.181577732445069, 0.372660171529375), C.pval = c(0.0751527689110548, 
0.00861397413415554, 0.00167933828677436, 0.74115825723352, 0, 
0.682882176063282, 0.815075574718218, 0.556907577125348, 0, 0, 
0.580104102468286, 0.603147598999385, 0.619002108927828, 0, 0, 
0.815075574718218, 0.857142857142857, 0.775872503798389, 0.857142857142857, 
0.857142857142857, 0.608563137793262, 0.705595391007869, 0.783681561837645, 
0.811226228320831, 0, 0.495958962674919, 0, 0.131192565416078, 
0.195786575007997, 0, 0.745363915760131, 0, 0.353872302577444, 
0.816267705847341, 0.767003371517578, 0, 0.767003371517578, 0, 
0, 0, 0.54103767599999, 0.487817131968106, 0.821628441934076, 
0, 0, 0.425520200169707, 0.836918071737218, 0, 0.547948206235813, 
0.819597419269029, 0, 0.857142857142857, 0.760417808671286, 0.857142857142857, 
0.809845215378872, 0.778585604678447, 0.597597141880351, 0.857142857142857, 
0.775872503798389, 0, 0.262108996275087, 0.826943208299301, 0, 
0.201818518945638, 0.51929062886488, 0, 0.547948206235813, 0, 
0, 0, 0.120068360030148, 0, 0.0987342417161341, 0.815075574718218, 
0), G.pval = c(0.719804172377832, 0.624214758400596, 0, 0.768797392227034, 
0.157723997720215, 0.181455881203167, 0, 0, 0.0066542444777471, 
0.0163914964377757, 0.0318035653629043, 0, 0.668703055848208, 
0.777777777775269, 0.0200335197059212, 0, 0.698196895553502, 
0, 0.0251268417806196, 0.130345851977349, 0.425971315443762, 
0, 0.531805553711521, 0, 0.777777777775269, 0.0291536603953039, 
0.0212465696263344, 0.777777777775269, 0.420814603093502, 0.713603766847878, 
0.0161822901885682, 0.238083160472283, 0, 0, 0, 0.777777777775269, 
0.0972740885054646, 0.236935780619669, 0.0722550192889703, 0.734134875017934, 
0.541490164319869, 0, 0.738683486812477, 0.777777777775269, 0.0534944335313544, 
0, 0.777777777775269, 0.777777777775269, 0, 0, 0.660342980349632, 
0.526774721345025, 0.691121491166631, 0, 0.728829109516305, 0.777777777775269, 
0, 0.700380771908857, 0.702484032007476, 0.693571492883567, 0, 
0.636929780661079, 0.718333457772176, 0.586848188856864, 0, 0.691121491166631, 
0.546150800731221, 0.516318299782234, 0.590390442627003, 0.348818946209842, 
0, 0.698196895553502, 0, 0.733139053854406, 0.718333457772176
), A.pval = c(0, 0, 0.532713863326883, 0, 0.156654718914168, 
0.180726907035223, 0.167208953123349, 0.119379741718796, 0.279052284173656, 
0.69974291545472, 0, 0.429794102383004, 0, 0.251507323347491, 
0.102447842470665, 0.132923867019411, 0.836363636363636, 0.336736339867383, 
0.7738658080342, 0.777839819046569, 0.836363636363636, 0.684439758933769, 
0.836363636363636, 0.618219923922453, 0.575261890180357, 0, 0.279052284173656, 
0.751526360136354, 0, 0.565380024447779, 0, 0.382664283713357, 
0.0194752018253076, 0.836363636363636, 0.514513916353225, 0.308551832807647, 
0, 0.490395471404999, 0.0709443392385807, 0.0187832700077238, 
0.836363636363636, 0.520728242997254, 0, 0.363519124187528, 0.836363636363636, 
0.142748474952988, 0, 0.363519124187528, 0.836363636363636, 0.481081552842013, 
0.693453359577228, 0, 0, 0.646099621988182, 0, 0, 0.602236826981356, 
0.836363636363636, 0.836363636363636, 0.508147082909215, 0.343565549815911, 
0, 0.761255742966345, 0, 0.747145563715734, 0.729106341900675, 
0.836363636363636, 0.836363636363636, 0.836363636363636, 0.836363636363636, 
0.409868959145453, 0.184750218657871, 0.0334155099547235, 0, 
0.761255742966345), guide.position = 1:75), .Names = c("A.area", 
"C.area", "G.area", "T.area", "Tot.area", "A.perc", "C.perc", 
"G.perc", "T.perc", "base.call", "index", "guide.seq", "T.pval", 
"C.pval", "G.pval", "A.pval", "guide.position"), row.names = 277:351, class = "data.frame")
```

*Report generated using EditR v1.0.8*
