# Supplementary material for: Mechanistic and genetic basis of single-strand templated repair at Cas12a-induced DNA breaks in Chlamydomonas reinhardtii
Source: Nat Commun. 2021 Nov 19;12:6751. doi: 10.1038/s41467-021-27004-1 (PMC8604939; doi:10.1038/s41467-021-27004-1)
Supplement: Supplementary file 22 — Source Data [file 41467_2021_27004_MOESM22_ESM.zip › Source Data/EditR analysis/EditR outputs/Sense/rep3_ssODN_sense_-32_-16_0_16_32.html]

EditR v1.0.8 report


# EditR v1.0.8 report

- Data QA
  - Filtering data
  - Percent noise peak area
  - Base information
- Predicted editing
  - Editing bar plot
  - Editing table plot
  - Table of editing results
- For use in R

## Data QA

### Filtering data

What the data looked like prefiltering:

and the post filtering signal / noise plot:

### Percent noise peak area

### Base information

Here’s information about the signal of each base, the critical percent value where any higher value would be called as significant, and Filliben’s correlation for how well the noise was modelled by the zero adjusted gamma distribution.

| Base | Average percent signal | Average peak area | Critical percent value | model mu | Fillibens correlation |
| --- | --- | --- | --- | --- | --- |
| A | 92.69508 | 170.4857 | 7.513324 | 2.453488 | 0.9958477 |
| C | 93.60644 | 183.5303 | 5.083071 | 2.051533 | 0.9949627 |
| G | 93.73823 | 179.6667 | 7.893184 | 2.540859 | 0.9904063 |
| T | 94.22210 | 205.1395 | 5.276846 | 2.093124 | 0.9896897 |

## Predicted editing

### Editing bar plot

### Editing table plot

### Table of editing results


Here’s the entire guide region

| Sanger position | Guide position | Guide sequence | Sanger base call | Focal base | Focal base peak area | p value |  |
| --- | --- | --- | --- | --- | --- | --- | --- |
| 274 | 1 | A | A | A | 95.86 | 0.0000000000 | \* |
| 274 | 1 | A | A | C | 2.07 | 0.4073086269 |  |
| 274 | 1 | A | A | G | 1.38 | 0.6517751229 |  |
| 274 | 1 | A | A | T | 0.69 | 0.8171010663 |  |
| 275 | 2 | A | A | A | 94.94 | 0.0000000000 | \* |
| 275 | 2 | A | A | C | 0.56 | 0.9299572378 |  |
| 275 | 2 | A | A | G | 1.69 | 0.5709021632 |  |
| 275 | 2 | A | A | T | 2.81 | 0.1908634645 |  |
| 276 | 3 | G | G | A | 5.63 | 0.0423875843 |  |
| 276 | 3 | G | G | C | 4.23 | 0.0332874408 |  |
| 276 | 3 | G | G | G | 90.14 | 0.0000000000 | \* |
| 276 | 3 | G | G | T | 0.00 | 0.8617021277 |  |
| 277 | 4 | A | A | A | 94.48 | 0.0000000000 | \* |
| 277 | 4 | A | A | C | 0.87 | 0.8666016284 |  |
| 277 | 4 | A | A | G | 2.03 | 0.4829199664 |  |
| 277 | 4 | A | A | T | 2.62 | 0.2311395636 |  |
| 278 | 5 | C | C | A | 4.28 | 0.1130327372 |  |
| 278 | 5 | C | C | C | 93.05 | 0.0000000000 | \* |
| 278 | 5 | C | C | G | 0.00 | 0.9000000000 |  |
| 278 | 5 | C | C | T | 2.67 | 0.2184856612 |  |
| 279 | 6 | T | T | A | 0.00 | 0.8899082569 |  |
| 279 | 6 | T | T | C | 3.00 | 0.1559300204 |  |
| 279 | 6 | T | T | G | 3.50 | 0.2108807838 |  |
| 279 | 6 | T | T | T | 93.50 | 0.0000000000 | \* |
| 280 | 7 | G | G | A | 3.04 | 0.2578161498 |  |
| 280 | 7 | G | G | C | 0.83 | 0.8781134438 |  |
| 280 | 7 | G | G | G | 95.03 | 0.0000000000 | \* |
| 280 | 7 | G | G | T | 1.10 | 0.7111289786 |  |
| 281 | 8 | G | G | A | 3.48 | 0.1944126550 |  |
| 281 | 8 | G | G | C | 1.30 | 0.7160363810 |  |
| 281 | 8 | G | G | G | 94.78 | 0.0000000000 | \* |
| 281 | 8 | G | G | T | 0.43 | 0.8504843327 |  |
| 282 | 9 | C | C | A | 2.82 | 0.2957244479 |  |
| 282 | 9 | C | C | C | 92.25 | 0.0000000000 | \* |
| 282 | 9 | C | C | G | 2.82 | 0.3168274590 |  |
| 282 | 9 | C | C | T | 2.11 | 0.3655125553 |  |
| 283 | 10 | C | C | A | 3.02 | 0.2616538575 |  |
| 283 | 10 | C | C | C | 95.48 | 0.0000000000 | \* |
| 283 | 10 | C | C | G | 0.00 | 0.9000000000 |  |
| 283 | 10 | C | C | T | 1.51 | 0.5723897777 |  |
| 284 | 11 | A | A | A | 85.71 | 0.0000000000 | \* |
| 284 | 11 | A | A | C | 2.04 | 0.4176112950 |  |
| 284 | 11 | A | A | G | 4.76 | 0.0932421120 |  |
| 284 | 11 | A | A | T | 7.48 | 0.0004625399 | \* |
| 285 | 12 | G | G | A | 4.40 | 0.1035986701 |  |
| 285 | 12 | G | G | C | 0.63 | 0.9200763413 |  |
| 285 | 12 | G | G | G | 94.97 | 0.0000000000 | \* |
| 285 | 12 | G | G | T | 0.00 | 0.8617021277 |  |
| 286 | 13 | A | A | A | 95.61 | 0.0000000000 | \* |
| 286 | 13 | A | A | C | 1.17 | 0.7685328845 |  |
| 286 | 13 | A | A | G | 0.88 | 0.7804442953 |  |
| 286 | 13 | A | A | T | 2.34 | 0.2998680708 |  |
| 287 | 14 | C | C | A | 4.74 | 0.0817126939 |  |
| 287 | 14 | C | C | C | 94.74 | 0.0000000000 | \* |
| 287 | 14 | C | C | G | 0.00 | 0.9000000000 |  |
| 287 | 14 | C | C | T | 0.53 | 0.8414704697 |  |
| 288 | 15 | C | C | A | 4.43 | 0.1015873418 |  |
| 288 | 15 | C | C | C | 89.24 | 0.0000000000 | \* |
| 288 | 15 | C | C | G | 6.33 | 0.0313485478 |  |
| 288 | 15 | C | C | T | 0.00 | 0.8617021277 |  |
| 289 | 16 | G | G | A | 3.30 | 0.2187901691 |  |
| 289 | 16 | G | G | C | 1.65 | 0.5733055202 |  |
| 289 | 16 | G | G | G | 93.41 | 0.0000000000 | \* |
| 289 | 16 | G | G | T | 1.65 | 0.5218028913 |  |
| 290 | 17 | T | T | A | 0.00 | 0.8899082569 |  |
| 290 | 17 | T | T | C | 2.65 | 0.2301616889 |  |
| 290 | 17 | T | T | G | 2.65 | 0.3484247291 |  |
| 290 | 17 | T | T | T | 94.70 | 0.0000000000 | \* |
| 291 | 18 | G | G | A | 2.73 | 0.3116514752 |  |
| 291 | 18 | G | G | C | 1.37 | 0.6913468313 |  |
| 291 | 18 | G | G | G | 95.22 | 0.0000000000 | \* |
| 291 | 18 | G | G | T | 0.68 | 0.8183770660 |  |
| 292 | 19 | T | T | A | 0.00 | 0.8899082569 |  |
| 292 | 19 | T | T | C | 1.11 | 0.7900485413 |  |
| 292 | 19 | T | T | G | 3.89 | 0.1652440283 |  |
| 292 | 19 | T | T | T | 95.00 | 0.0000000000 | \* |
| 293 | 20 | T | T | A | 0.00 | 0.8899082569 |  |
| 293 | 20 | T | T | C | 0.00 | 0.9529411765 |  |
| 293 | 20 | T | T | G | 2.62 | 0.3545477395 |  |
| 293 | 20 | T | T | T | 97.38 | 0.0000000000 | \* |
| 294 | 21 | T | T | A | 0.00 | 0.8899082569 |  |
| 294 | 21 | T | T | C | 0.00 | 0.9529411765 |  |
| 294 | 21 | T | T | G | 3.72 | 0.1834866035 |  |
| 294 | 21 | T | T | T | 96.28 | 0.0000000000 | \* |
| 295 | 22 | G | G | A | 2.02 | 0.4656122214 |  |
| 295 | 22 | G | G | C | 2.02 | 0.4237249092 |  |
| 295 | 22 | G | G | G | 95.55 | 0.0000000000 | \* |
| 295 | 22 | G | G | T | 0.40 | 0.8527507666 |  |
| 296 | 23 | T | T | A | 0.00 | 0.8899082569 |  |
| 296 | 23 | T | T | C | 0.00 | 0.9529411765 |  |
| 296 | 23 | T | T | G | 1.92 | 0.5103287517 |  |
| 296 | 23 | T | T | T | 98.08 | 0.0000000000 | \* |
| 297 | 24 | G | G | A | 2.51 | 0.3546222891 |  |
| 297 | 24 | G | G | C | 3.52 | 0.0837391973 |  |
| 297 | 24 | G | G | G | 92.46 | 0.0000000000 | \* |
| 297 | 24 | G | G | T | 1.51 | 0.5723897777 |  |
| 298 | 25 | C | C | A | 1.83 | 0.5150358904 |  |
| 298 | 25 | C | C | C | 92.07 | 0.0000000000 | \* |
| 298 | 25 | C | C | G | 3.66 | 0.1911037262 |  |
| 298 | 25 | C | C | T | 2.44 | 0.2736128700 |  |
| 299 | 26 | A | A | A | 89.31 | 0.0000000000 | \* |
| 299 | 26 | A | A | C | 3.05 | 0.1465954015 |  |
| 299 | 26 | A | A | G | 4.58 | 0.1052984476 |  |
| 299 | 26 | A | A | T | 3.05 | 0.1480852198 |  |
| 300 | 27 | C | C | A | 3.45 | 0.1982708996 |  |
| 300 | 27 | C | C | C | 89.66 | 0.0000000000 | \* |
| 300 | 27 | C | C | G | 5.17 | 0.0705576376 |  |
| 300 | 27 | C | C | T | 1.72 | 0.4948736543 |  |
| 301 | 28 | T | T | A | 3.43 | 0.2008419032 |  |
| 301 | 28 | T | T | C | 5.14 | 0.0091700861 | \* |
| 301 | 28 | T | T | G | 0.00 | 0.9000000000 |  |
| 301 | 28 | T | T | T | 91.43 | 0.0000000000 | \* |
| 302 | 29 | A | A | A | 91.67 | 0.0000000000 | \* |
| 302 | 29 | A | A | C | 5.36 | 0.0067045033 | \* |
| 302 | 29 | A | A | G | 1.79 | 0.5449981060 |  |
| 302 | 29 | A | A | T | 1.19 | 0.6834199428 |  |
| 303 | 30 | C | C | A | 1.28 | 0.6627786659 |  |
| 303 | 30 | C | C | C | 95.51 | 0.0000000000 | \* |
| 303 | 30 | C | C | G | 2.56 | 0.3652677384 |  |
| 303 | 30 | C | C | T | 0.64 | 0.8254834245 |  |
| 304 | 31 | A | A | A | 92.17 | 0.0000000000 | \* |
| 304 | 31 | A | A | C | 2.61 | 0.2402245739 |  |
| 304 | 31 | A | A | G | 5.22 | 0.0684129528 |  |
| 304 | 31 | A | A | T | 0.00 | 0.8617021277 |  |
| 305 | 32 | C | C | A | 1.34 | 0.6463003774 |  |
| 305 | 32 | C | C | C | 93.96 | 0.0000000000 | \* |
| 305 | 32 | C | C | G | 4.03 | 0.1512792279 |  |
| 305 | 32 | C | C | T | 0.67 | 0.8204042944 |  |
| 306 | 33 | G | G | A | 4.00 | 0.1369896522 |  |
| 306 | 33 | G | G | C | 4.00 | 0.0450040715 |  |
| 306 | 33 | G | G | G | 91.20 | 0.0000000000 | \* |
| 306 | 33 | G | G | T | 0.80 | 0.7946346795 |  |
| 307 | 34 | G | G | A | 0.96 | 0.7490625700 |  |
| 307 | 34 | G | G | C | 2.39 | 0.2999826686 |  |
| 307 | 34 | G | G | G | 95.69 | 0.0000000000 | \* |
| 307 | 34 | G | G | T | 0.96 | 0.7551297334 |  |
| 308 | 35 | G | G | A | 1.89 | 0.5001992069 |  |
| 308 | 35 | G | G | C | 1.89 | 0.4763383616 |  |
| 308 | 35 | G | G | G | 95.60 | 0.0000000000 | \* |
| 308 | 35 | G | G | T | 0.63 | 0.8274202442 |  |
| 309 | 36 | C | C | A | 3.55 | 0.1859214264 |  |
| 309 | 36 | C | C | C | 95.04 | 0.0000000000 | \* |
| 309 | 36 | C | C | G | 1.42 | 0.6413659427 |  |
| 309 | 36 | C | C | T | 0.00 | 0.8617021277 |  |
| 310 | 37 | A | A | A | 90.65 | 0.0000000000 | \* |
| 310 | 37 | A | A | C | 2.88 | 0.1791725090 |  |
| 310 | 37 | A | A | G | 2.88 | 0.3059435732 |  |
| 310 | 37 | A | A | T | 3.60 | 0.0811161728 |  |
| 311 | 38 | C | C | A | 1.07 | 0.7199653069 |  |
| 311 | 38 | C | C | C | 96.26 | 0.0000000000 | \* |
| 311 | 38 | C | C | G | 1.60 | 0.5921318129 |  |
| 311 | 38 | C | C | T | 1.07 | 0.7221699625 |  |
| 312 | 39 | C | C | A | 5.49 | 0.0470145572 |  |
| 312 | 39 | C | C | C | 90.66 | 0.0000000000 | \* |
| 312 | 39 | C | C | G | 3.30 | 0.2387743131 |  |
| 312 | 39 | C | C | T | 0.55 | 0.8386735043 |  |
| 313 | 40 | C | C | A | 3.98 | 0.1391362828 |  |
| 313 | 40 | C | C | C | 92.61 | 0.0000000000 | \* |
| 313 | 40 | C | C | G | 0.00 | 0.9000000000 |  |
| 313 | 40 | C | C | T | 3.41 | 0.1004213145 |  |
| 314 | 41 | T | T | A | 0.00 | 0.8899082569 |  |
| 314 | 41 | T | T | C | 1.88 | 0.4809838621 |  |
| 314 | 41 | T | T | G | 5.00 | 0.0793745661 |  |
| 314 | 41 | T | T | T | 93.12 | 0.0000000000 | \* |
| 315 | 42 | G | G | A | 3.59 | 0.1809052362 |  |
| 315 | 42 | G | G | C | 1.35 | 0.6994725985 |  |
| 315 | 42 | G | G | G | 94.17 | 0.0000000000 | \* |
| 315 | 42 | G | G | T | 0.90 | 0.7712201343 |  |
| 316 | 43 | A | A | A | 89.39 | 0.0000000000 | \* |
| 316 | 43 | A | A | C | 1.52 | 0.6290565068 |  |
| 316 | 43 | A | A | G | 1.52 | 0.6156731275 |  |
| 316 | 43 | A | A | T | 7.58 | 0.0004043014 | \* |
| 317 | 44 | C | C | A | 1.94 | 0.4878042113 |  |
| 317 | 44 | C | C | C | 96.77 | 0.0000000000 | \* |
| 317 | 44 | C | C | G | 0.00 | 0.9000000000 |  |
| 317 | 44 | C | C | T | 1.29 | 0.6495057249 |  |
| 318 | 45 | C | C | A | 2.11 | 0.4440949414 |  |
| 318 | 45 | C | C | C | 92.25 | 0.0000000000 | \* |
| 318 | 45 | C | C | G | 5.63 | 0.0512647003 |  |
| 318 | 45 | C | C | T | 0.00 | 0.8617021277 |  |
| 319 | 46 | G | G | A | 5.93 | 0.0340497387 |  |
| 319 | 46 | G | G | C | 2.96 | 0.1626878552 |  |
| 319 | 46 | G | G | G | 90.37 | 0.0000000000 | \* |
| 319 | 46 | G | G | T | 0.74 | 0.8072831168 |  |
| 320 | 47 | A | A | A | 95.85 | 0.0000000000 | \* |
| 320 | 47 | A | A | C | 1.66 | 0.5685593012 |  |
| 320 | 47 | A | A | G | 0.83 | 0.7914860678 |  |
| 320 | 47 | A | A | T | 1.66 | 0.5177323198 |  |
| 321 | 48 | C | C | A | 2.78 | 0.3028460013 |  |
| 321 | 48 | C | C | C | 96.53 | 0.0000000000 | \* |
| 321 | 48 | C | C | G | 0.69 | 0.8212291152 |  |
| 321 | 48 | C | C | T | 0.00 | 0.8617021277 |  |
| 322 | 49 | G | G | A | 7.78 | 0.0081146909 | \* |
| 322 | 49 | G | G | C | 2.22 | 0.3538283520 |  |
| 322 | 49 | G | G | G | 87.78 | 0.0000000000 | \* |
| 322 | 49 | G | G | T | 2.22 | 0.3327350328 |  |
| 323 | 50 | G | G | A | 1.47 | 0.6111411168 |  |
| 323 | 50 | G | G | C | 1.47 | 0.6476987795 |  |
| 323 | 50 | G | G | G | 95.59 | 0.0000000000 | \* |
| 323 | 50 | G | G | T | 1.47 | 0.5856744883 |  |
| 324 | 51 | C | C | A | 2.68 | 0.3214973133 |  |
| 324 | 51 | C | C | C | 91.07 | 0.0000000000 | \* |
| 324 | 51 | C | C | G | 2.68 | 0.3426960669 |  |
| 324 | 51 | C | C | T | 3.57 | 0.0835430862 |  |
| 325 | 52 | A | A | A | 92.75 | 0.0000000000 | \* |
| 325 | 52 | A | A | C | 1.45 | 0.6565870094 |  |
| 325 | 52 | A | A | G | 0.00 | 0.9000000000 |  |
| 325 | 52 | A | A | T | 5.80 | 0.0049703964 | \* |
| 326 | 53 | A | A | A | 95.04 | 0.0000000000 | \* |
| 326 | 53 | A | A | C | 0.00 | 0.9529411765 |  |
| 326 | 53 | A | A | G | 0.71 | 0.8181352780 |  |
| 326 | 53 | A | A | T | 4.26 | 0.0370098273 |  |
| 327 | 54 | G | G | A | 1.80 | 0.5221896219 |  |
| 327 | 54 | G | G | C | 2.70 | 0.2172683759 |  |
| 327 | 54 | G | G | G | 93.69 | 0.0000000000 | \* |
| 327 | 54 | G | G | T | 1.80 | 0.4676925864 |  |
| 328 | 55 | A | A | A | 95.38 | 0.0000000000 | \* |
| 328 | 55 | A | A | C | 1.16 | 0.7735860332 |  |
| 328 | 55 | A | A | G | 0.58 | 0.8440776957 |  |
| 328 | 55 | A | A | T | 2.89 | 0.1756633241 |  |
| 329 | 56 | A | A | A | 95.04 | 0.0000000000 | \* |
| 329 | 56 | A | A | C | 0.00 | 0.9529411765 |  |
| 329 | 56 | A | A | G | 0.71 | 0.8181352780 |  |
| 329 | 56 | A | A | T | 4.26 | 0.0370098273 |  |
| 330 | 57 | G | G | A | 2.17 | 0.4295385931 |  |
| 330 | 57 | G | G | C | 1.09 | 0.7986622338 |  |
| 330 | 57 | G | G | G | 92.39 | 0.0000000000 | \* |
| 330 | 57 | G | G | T | 4.35 | 0.0330095349 |  |
| 331 | 58 | T | T | A | 1.89 | 0.5001992069 |  |
| 331 | 58 | T | T | C | 3.14 | 0.1317511290 |  |
| 331 | 58 | T | T | G | 0.63 | 0.8344379654 |  |
| 331 | 58 | T | T | T | 94.34 | 0.0000000000 | \* |
| 332 | 59 | T | T | A | 7.24 | 0.0124247005 |  |
| 332 | 59 | T | T | C | 1.32 | 0.7114308884 |  |
| 332 | 59 | T | T | G | 3.29 | 0.2398207486 |  |
| 332 | 59 | T | T | T | 88.16 | 0.0000000000 | \* |
| 333 | 60 | C | C | A | 0.00 | 0.8899082569 |  |
| 333 | 60 | C | C | C | 95.56 | 0.0000000000 | \* |
| 333 | 60 | C | C | G | 2.96 | 0.2911741119 |  |
| 333 | 60 | C | C | T | 1.48 | 0.5817608303 |  |
| 334 | 61 | G | G | A | 7.62 | 0.0092000609 | \* |
| 334 | 61 | G | G | C | 3.81 | 0.0577528513 |  |
| 334 | 61 | G | G | G | 85.71 | 0.0000000000 | \* |
| 334 | 61 | G | G | T | 2.86 | 0.1817248973 |  |
| 335 | 62 | A | A | A | 96.59 | 0.0000000000 | \* |
| 335 | 62 | A | A | C | 1.46 | 0.6506929377 |  |
| 335 | 62 | A | A | G | 0.49 | 0.8596617309 |  |
| 335 | 62 | A | A | T | 1.46 | 0.5882500376 |  |
| 336 | 63 | C | C | A | 2.56 | 0.3440812787 |  |
| 336 | 63 | C | C | C | 93.59 | 0.0000000000 | \* |
| 336 | 63 | C | C | G | 1.92 | 0.5103287517 |  |
| 336 | 63 | C | C | T | 1.92 | 0.4264022131 |  |
| 337 | 64 | A | A | A | 90.00 | 0.0000000000 | \* |
| 337 | 64 | A | A | C | 3.00 | 0.1559300204 |  |
| 337 | 64 | A | A | G | 4.00 | 0.1539115089 |  |
| 337 | 64 | A | A | T | 3.00 | 0.1566852268 |  |
| 338 | 65 | G | G | A | 1.90 | 0.4971450217 |  |
| 338 | 65 | G | G | C | 3.16 | 0.1286885497 |  |
| 338 | 65 | G | G | G | 94.94 | 0.0000000000 | \* |
| 338 | 65 | G | G | T | 0.00 | 0.8617021277 |  |
| 339 | 66 | C | C | A | 1.41 | 0.6281616210 |  |
| 339 | 66 | C | C | C | 95.07 | 0.0000000000 | \* |
| 339 | 66 | C | C | G | 1.41 | 0.6440232854 |  |
| 339 | 66 | C | C | T | 2.11 | 0.3655125553 |  |
| 340 | 67 | T | T | A | 1.15 | 0.6987204555 |  |
| 340 | 67 | T | T | C | 0.57 | 0.9282195894 |  |
| 340 | 67 | T | T | G | 2.87 | 0.3066744184 |  |
| 340 | 67 | T | T | T | 95.40 | 0.0000000000 | \* |
| 341 | 68 | C | C | A | 1.32 | 0.6511696912 |  |
| 341 | 68 | C | C | C | 94.04 | 0.0000000000 | \* |
| 341 | 68 | C | C | G | 3.31 | 0.2366791448 |  |
| 341 | 68 | C | C | T | 1.32 | 0.6376018046 |  |
| 342 | 69 | C | C | A | 0.54 | 0.8409862933 |  |
| 342 | 69 | C | C | C | 95.14 | 0.0000000000 | \* |
| 342 | 69 | C | C | G | 2.70 | 0.3380724639 |  |
| 342 | 69 | C | C | T | 1.62 | 0.5313695942 |  |
| 343 | 70 | C | C | A | 0.99 | 0.7406134497 |  |
| 343 | 70 | C | C | C | 93.07 | 0.0000000000 | \* |
| 343 | 70 | C | C | G | 4.95 | 0.0820896962 |  |
| 343 | 70 | C | C | T | 0.99 | 0.7457804145 |  |
| 344 | 71 | G | G | A | 5.31 | 0.0538923067 |  |
| 344 | 71 | G | G | C | 0.88 | 0.8630322125 |  |
| 344 | 71 | G | G | G | 92.92 | 0.0000000000 | \* |
| 344 | 71 | G | G | T | 0.88 | 0.7742717025 |  |
| 345 | 72 | C | C | A | 0.00 | 0.8899082569 |  |
| 345 | 72 | C | C | C | 96.80 | 0.0000000000 | \* |
| 345 | 72 | C | C | G | 0.00 | 0.9000000000 |  |
| 345 | 72 | C | C | T | 3.20 | 0.1265089566 |  |
| 346 | 73 | G | G | A | 9.57 | 0.0019240363 | \* |
| 346 | 73 | G | G | C | 1.74 | 0.5357451919 |  |
| 346 | 73 | G | G | G | 87.83 | 0.0000000000 | \* |
| 346 | 73 | G | G | T | 0.87 | 0.7781465958 |  |
| 347 | 74 | A | A | A | 95.91 | 0.0000000000 | \* |
| 347 | 74 | A | A | C | 1.17 | 0.7685328845 |  |
| 347 | 74 | A | A | G | 0.58 | 0.8428306292 |  |
| 347 | 74 | A | A | T | 2.34 | 0.2998680708 |  |
| 348 | 75 | C | C | A | 2.53 | 0.3506925106 |  |
| 348 | 75 | C | C | C | 82.91 | 0.0000000000 | \* |
| 348 | 75 | C | C | G | 12.03 | 0.0004189309 | \* |
| 348 | 75 | C | C | T | 2.53 | 0.2507599424 |  |

## For use in R

If you want to work with the results in R, here is output that you can copy and paste in your terminal to get:

The base information:

```
structure(list(focal.base = c("A", "C", "G", "T"), avg.percsignal = c(92.6950798885496, 
93.6064430303707, 93.7382308928947, 94.2221028816412), avg.areasignal = c(170.485714285714, 
183.530303030303, 179.666666666667, 205.139534883721), crit.perc.area = c(7.5133243351416, 
5.08307092144495, 7.89318352828117, 5.27684635261132), mu = c(2.45348797358303, 
2.05153331176272, 2.54085902298207, 2.09312367461885), fillibens = c(0.995847709327196, 
0.994962698239947, 0.990406338657915, 0.989689697984398)), .Names = c("focal.base", 
"avg.percsignal", "avg.areasignal", "crit.perc.area", "mu", "fillibens"
), row.names = c(NA, -4L), class = "data.frame")
```

the data.frame that contains information on the guide region:

```
structure(list(A.area = c(139, 169, 8, 325, 8, 0, 11, 8, 4, 6, 
126, 7, 327, 9, 7, 6, 0, 8, 0, 0, 0, 5, 0, 5, 3, 117, 6, 6, 154, 
2, 106, 2, 5, 2, 3, 5, 126, 2, 10, 7, 0, 8, 177, 3, 3, 8, 231, 
4, 7, 3, 3, 64, 134, 2, 165, 134, 2, 3, 11, 0, 8, 198, 4, 90, 
3, 2, 2, 2, 1, 1, 6, 0, 11, 164, 4), C.area = c(3, 1, 6, 3, 174, 
6, 3, 3, 131, 190, 3, 1, 4, 180, 141, 3, 4, 4, 2, 0, 0, 5, 0, 
7, 151, 4, 156, 9, 9, 149, 3, 140, 5, 5, 3, 134, 4, 180, 165, 
163, 3, 3, 3, 150, 131, 4, 4, 139, 2, 3, 102, 1, 0, 3, 2, 0, 
1, 5, 2, 129, 4, 3, 146, 3, 5, 135, 1, 142, 176, 94, 1, 121, 
2, 2, 131), G.area = c(2, 3, 128, 7, 0, 7, 344, 218, 4, 0, 7, 
151, 3, 0, 10, 170, 4, 279, 7, 5, 7, 236, 3, 184, 6, 6, 9, 0, 
3, 4, 6, 6, 114, 200, 152, 2, 4, 3, 6, 0, 8, 210, 3, 0, 8, 122, 
2, 1, 79, 195, 3, 0, 1, 104, 1, 1, 85, 1, 5, 4, 90, 1, 3, 4, 
150, 2, 5, 5, 5, 5, 105, 0, 101, 1, 19), T.area = c(1, 5, 0, 
9, 5, 187, 4, 1, 3, 3, 11, 0, 8, 1, 0, 3, 143, 2, 171, 186, 181, 
1, 153, 3, 4, 4, 3, 160, 2, 1, 0, 1, 1, 2, 1, 0, 5, 2, 1, 6, 
149, 2, 15, 2, 0, 1, 4, 0, 2, 3, 4, 4, 6, 2, 5, 6, 4, 150, 134, 
2, 3, 3, 3, 3, 0, 3, 166, 2, 3, 1, 1, 4, 1, 4, 4), Tot.area = c(145, 
178, 142, 344, 187, 200, 362, 230, 142, 199, 147, 159, 342, 190, 
158, 182, 151, 293, 180, 191, 188, 247, 156, 199, 164, 131, 174, 
175, 168, 156, 115, 149, 125, 209, 159, 141, 139, 187, 182, 176, 
160, 223, 198, 155, 142, 135, 241, 144, 90, 204, 112, 69, 141, 
111, 173, 141, 92, 159, 152, 135, 105, 205, 156, 100, 158, 142, 
174, 151, 185, 101, 113, 125, 115, 171, 158), A.perc = c(95.8620689655172, 
94.9438202247191, 5.63380281690141, 94.4767441860465, 4.27807486631016, 
0, 3.03867403314917, 3.47826086956522, 2.8169014084507, 3.01507537688442, 
85.7142857142857, 4.40251572327044, 95.6140350877193, 4.73684210526316, 
4.43037974683544, 3.2967032967033, 0, 2.73037542662116, 0, 0, 
0, 2.02429149797571, 0, 2.51256281407035, 1.82926829268293, 89.3129770992366, 
3.44827586206897, 3.42857142857143, 91.6666666666667, 1.28205128205128, 
92.1739130434783, 1.34228187919463, 4, 0.956937799043062, 1.88679245283019, 
3.54609929078014, 90.6474820143885, 1.06951871657754, 5.49450549450549, 
3.97727272727273, 0, 3.58744394618834, 89.3939393939394, 1.93548387096774, 
2.11267605633803, 5.92592592592593, 95.850622406639, 2.77777777777778, 
7.77777777777778, 1.47058823529412, 2.67857142857143, 92.7536231884058, 
95.0354609929078, 1.8018018018018, 95.3757225433526, 95.0354609929078, 
2.17391304347826, 1.88679245283019, 7.23684210526316, 0, 7.61904761904762, 
96.5853658536585, 2.56410256410256, 90, 1.89873417721519, 1.40845070422535, 
1.14942528735632, 1.32450331125828, 0.540540540540541, 0.99009900990099, 
5.30973451327434, 0, 9.56521739130435, 95.906432748538, 2.53164556962025
), C.perc = c(2.06896551724138, 0.561797752808989, 4.22535211267606, 
0.872093023255814, 93.048128342246, 3, 0.828729281767956, 1.30434782608696, 
92.2535211267606, 95.4773869346734, 2.04081632653061, 0.628930817610063, 
1.16959064327485, 94.7368421052632, 89.2405063291139, 1.64835164835165, 
2.64900662251656, 1.36518771331058, 1.11111111111111, 0, 0, 2.02429149797571, 
0, 3.51758793969849, 92.0731707317073, 3.05343511450382, 89.6551724137931, 
5.14285714285714, 5.35714285714286, 95.5128205128205, 2.60869565217391, 
93.9597315436242, 4, 2.39234449760766, 1.88679245283019, 95.0354609929078, 
2.87769784172662, 96.2566844919786, 90.6593406593407, 92.6136363636364, 
1.875, 1.34529147982063, 1.51515151515152, 96.7741935483871, 
92.2535211267606, 2.96296296296296, 1.6597510373444, 96.5277777777778, 
2.22222222222222, 1.47058823529412, 91.0714285714286, 1.44927536231884, 
0, 2.7027027027027, 1.15606936416185, 0, 1.08695652173913, 3.14465408805031, 
1.31578947368421, 95.5555555555556, 3.80952380952381, 1.46341463414634, 
93.5897435897436, 3, 3.16455696202532, 95.0704225352113, 0.574712643678161, 
94.0397350993377, 95.1351351351351, 93.0693069306931, 0.884955752212389, 
96.8, 1.73913043478261, 1.16959064327485, 82.9113924050633), 
    G.perc = c(1.37931034482759, 1.68539325842697, 90.1408450704225, 
    2.03488372093023, 0, 3.5, 95.0276243093923, 94.7826086956522, 
    2.8169014084507, 0, 4.76190476190476, 94.9685534591195, 0.87719298245614, 
    0, 6.32911392405063, 93.4065934065934, 2.64900662251656, 
    95.221843003413, 3.88888888888889, 2.61780104712042, 3.72340425531915, 
    95.5465587044534, 1.92307692307692, 92.462311557789, 3.65853658536585, 
    4.58015267175572, 5.17241379310345, 0, 1.78571428571429, 
    2.56410256410256, 5.21739130434783, 4.02684563758389, 91.2, 
    95.6937799043062, 95.5974842767296, 1.41843971631206, 2.87769784172662, 
    1.60427807486631, 3.2967032967033, 0, 5, 94.1704035874439, 
    1.51515151515152, 0, 5.63380281690141, 90.3703703703704, 
    0.829875518672199, 0.694444444444444, 87.7777777777778, 95.5882352941177, 
    2.67857142857143, 0, 0.709219858156028, 93.6936936936937, 
    0.578034682080925, 0.709219858156028, 92.3913043478261, 0.628930817610063, 
    3.28947368421053, 2.96296296296296, 85.7142857142857, 0.48780487804878, 
    1.92307692307692, 4, 94.9367088607595, 1.40845070422535, 
    2.8735632183908, 3.3112582781457, 2.7027027027027, 4.95049504950495, 
    92.9203539823009, 0, 87.8260869565217, 0.584795321637427, 
    12.0253164556962), T.perc = c(0.689655172413793, 2.80898876404494, 
    0, 2.61627906976744, 2.67379679144385, 93.5, 1.10497237569061, 
    0.434782608695652, 2.11267605633803, 1.50753768844221, 7.48299319727891, 
    0, 2.33918128654971, 0.526315789473684, 0, 1.64835164835165, 
    94.7019867549669, 0.68259385665529, 95, 97.3821989528796, 
    96.2765957446808, 0.404858299595142, 98.0769230769231, 1.50753768844221, 
    2.4390243902439, 3.05343511450382, 1.72413793103448, 91.4285714285714, 
    1.19047619047619, 0.641025641025641, 0, 0.671140939597315, 
    0.8, 0.956937799043062, 0.628930817610063, 0, 3.59712230215827, 
    1.06951871657754, 0.549450549450549, 3.40909090909091, 93.125, 
    0.896860986547085, 7.57575757575758, 1.29032258064516, 0, 
    0.740740740740741, 1.6597510373444, 0, 2.22222222222222, 
    1.47058823529412, 3.57142857142857, 5.79710144927536, 4.25531914893617, 
    1.8018018018018, 2.89017341040462, 4.25531914893617, 4.34782608695652, 
    94.3396226415094, 88.1578947368421, 1.48148148148148, 2.85714285714286, 
    1.46341463414634, 1.92307692307692, 3, 0, 2.11267605633803, 
    95.4022988505747, 1.32450331125828, 1.62162162162162, 0.99009900990099, 
    0.884955752212389, 3.2, 0.869565217391304, 2.33918128654971, 
    2.53164556962025), base.call = c("A", "A", "G", "A", "C", 
    "T", "G", "G", "C", "C", "A", "G", "A", "C", "C", "G", "T", 
    "G", "T", "T", "T", "G", "T", "G", "C", "A", "C", "T", "A", 
    "C", "A", "C", "G", "G", "G", "C", "A", "C", "C", "C", "T", 
    "G", "A", "C", "C", "G", "A", "C", "G", "G", "C", "A", "A", 
    "G", "A", "A", "G", "T", "T", "C", "G", "A", "C", "A", "G", 
    "C", "T", "C", "C", "C", "G", "C", "G", "A", "C"), index = 274:348, 
    guide.seq = c("A", "A", "G", "A", "C", "T", "G", "G", "C", 
    "C", "A", "G", "A", "C", "C", "G", "T", "G", "T", "T", "T", 
    "G", "T", "G", "C", "A", "C", "T", "A", "C", "A", "C", "G", 
    "G", "G", "C", "A", "C", "C", "C", "T", "G", "A", "C", "C", 
    "G", "A", "C", "G", "G", "C", "A", "A", "G", "A", "A", "G", 
    "T", "T", "C", "G", "A", "C", "A", "G", "C", "T", "C", "C", 
    "C", "G", "C", "G", "A", "C"), T.pval = c(0.817101066254929, 
    0.190863464478458, 0.861702127659573, 0.231139563633212, 
    0.218485661161506, 0, 0.711128978583502, 0.850484332682799, 
    0.365512555282617, 0.572389777731567, 0.000462539939124484, 
    0.861702127659573, 0.299868070791834, 0.841470469731516, 
    0.861702127659573, 0.521802891329162, 0, 0.818377065988625, 
    0, 0, 0, 0.85275076659046, 0, 0.572389777731567, 0.27361287004428, 
    0.148085219838521, 0.494873654294391, 0, 0.683419942811378, 
    0.825483424541771, 0.861702127659573, 0.82040429442747, 0.79463467949844, 
    0.755129733390983, 0.827420244222304, 0.861702127659573, 
    0.0811161727713481, 0.722169962458612, 0.838673504271421, 
    0.100421314495684, 0, 0.771220134253386, 0.000404301394517859, 
    0.649505724898411, 0.861702127659573, 0.807283116792378, 
    0.517732319751015, 0.861702127659573, 0.332735032815652, 
    0.585674488332235, 0.0835430861899551, 0.00497039637791796, 
    0.0370098273479198, 0.467692586378122, 0.175663324089739, 
    0.0370098273479198, 0.0330095348782172, 0, 0, 0.581760830346763, 
    0.181724897261129, 0.588250037614776, 0.426402213141993, 
    0.156685226843108, 0.861702127659573, 0.365512555282617, 
    0, 0.637601804588025, 0.531369594214584, 0.745780414516909, 
    0.774271702525327, 0.126508956563864, 0.778146595826764, 
    0.299868070791834, 0.250759942367963), C.pval = c(0.407308626900608, 
    0.929957237782907, 0.0332874407664799, 0.86660162835116, 
    0, 0.155930020395208, 0.878113443755808, 0.716036381044361, 
    0, 0, 0.417611295042506, 0.920076341305118, 0.768532884470385, 
    0, 0, 0.573305520196868, 0.230161688935297, 0.691346831271808, 
    0.790048541314086, 0.952941176470502, 0.952941176470502, 
    0.423724909160965, 0.952941176470502, 0.0837391972552192, 
    0, 0.14659540152098, 0, 0.00917008612207226, 0.00670450326161909, 
    0, 0.240224573934786, 0, 0.0450040715290743, 0.299982668587241, 
    0.476338361564513, 0, 0.179172508971452, 0, 0, 0, 0.480983862098857, 
    0.699472598535424, 0.629056506815528, 0, 0, 0.162687855173019, 
    0.568559301166405, 0, 0.353828352028594, 0.647698779500054, 
    0, 0.65658700942569, 0.952941176470502, 0.217268375944483, 
    0.773586033208949, 0.952941176470502, 0.798662233796456, 
    0.131751128961542, 0.711430888397821, 0, 0.0577528512926706, 
    0.650692937678992, 0, 0.155930020395208, 0.128688549734958, 
    0, 0.928219589440105, 0, 0, 0, 0.863032212547871, 0, 0.535745191883285, 
    0.768532884470385, 0), G.pval = c(0.651775122898769, 0.570902163181278, 
    0, 0.482919966404282, 0.899999999997222, 0.210880783780507, 
    0, 0, 0.316827459047761, 0.899999999997222, 0.0932421119642693, 
    0, 0.780444295260273, 0.899999999997222, 0.0313485477697827, 
    0, 0.348424729143952, 0, 0.165244028272821, 0.35454773949369, 
    0.183486603468381, 0, 0.510328751739423, 0, 0.191103726210397, 
    0.10529844760989, 0.0705576376358575, 0.899999999997222, 
    0.544998105970831, 0.365267738370674, 0.0684129528312457, 
    0.151279227874097, 0, 0, 0, 0.641365942662729, 0.305943573233214, 
    0.592131812946591, 0.238774313053273, 0.899999999997222, 
    0.079374566097707, 0, 0.615673127471799, 0.899999999997222, 
    0.0512647002937627, 0, 0.791486067786546, 0.82122911519548, 
    0, 0, 0.342696066892285, 0.899999999997222, 0.818135277968796, 
    0, 0.844077695678788, 0.818135277968796, 0, 0.834437965401538, 
    0.239820748608475, 0.291174111907245, 0, 0.859661730921974, 
    0.510328751739423, 0.153911508909766, 0, 0.644023285362241, 
    0.306674418367105, 0.236679144799661, 0.338072463902227, 
    0.0820896961791077, 0, 0.899999999997222, 0, 0.842830629180009, 
    0.000418930895667802), A.pval = c(0, 0, 0.0423875843350923, 
    0, 0.113032737207377, 0.889908256880355, 0.257816149775447, 
    0.194412655038554, 0.295724447865264, 0.261653857479403, 
    0, 0.103598670088413, 0, 0.0817126938946331, 0.101587341843026, 
    0.218790169070436, 0.889908256880355, 0.31165147524166, 0.889908256880355, 
    0.889908256880355, 0.889908256880355, 0.465612221441222, 
    0.889908256880355, 0.354622289106268, 0.515035890395221, 
    0, 0.198270899554206, 0.200841903217958, 0, 0.662778665902119, 
    0, 0.64630037735748, 0.136989652203173, 0.74906257004576, 
    0.500199206942042, 0.185921426412874, 0, 0.719965306868204, 
    0.0470145572356974, 0.139136282782847, 0.889908256880355, 
    0.180905236151292, 0, 0.4878042112557, 0.444094941370039, 
    0.0340497387346791, 0, 0.302846001339552, 0.00811469092525896, 
    0.611141116848142, 0.321497313318611, 0, 0, 0.522189621924684, 
    0, 0, 0.429538593060367, 0.500199206942042, 0.012424700492893, 
    0.889908256880355, 0.00920006088017489, 0, 0.344081278696606, 
    0, 0.49714502170206, 0.628161620992843, 0.698720455523793, 
    0.651169691248517, 0.840986293280661, 0.740613449656057, 
    0.053892306712102, 0.889908256880355, 0.00192403634749216, 
    0, 0.35069251056346), guide.position = 1:75), .Names = c("A.area", 
"C.area", "G.area", "T.area", "Tot.area", "A.perc", "C.perc", 
"G.perc", "T.perc", "base.call", "index", "guide.seq", "T.pval", 
"C.pval", "G.pval", "A.pval", "guide.position"), row.names = 274:348, class = "data.frame")
```

*Report generated using EditR v1.0.8*
