# Supplementary material for: Mechanistic and genetic basis of single-strand templated repair at Cas12a-induced DNA breaks in Chlamydomonas reinhardtii
Source: Nat Commun. 2021 Nov 19;12:6751. doi: 10.1038/s41467-021-27004-1 (PMC8604939; doi:10.1038/s41467-021-27004-1)
Supplement: Supplementary file 22 — Source Data [file 41467_2021_27004_MOESM22_ESM.zip › Source Data/EditR analysis/EditR outputs/wt/rep1_wt.html]

EditR v1.0.10 report


# EditR v1.0.10 report

- Data QA
  - Filtering data
  - Percent noise peak area
  - Base information
- Predicted editing
  - Editing bar plot
  - Editing table plot
  - Table of editing results
- For use in R

## Data QA

### Filtering data

What the data looked like prefiltering:

and the post filtering signal / noise plot:

### Percent noise peak area

### Base information

Here’s information about the signal of each base, the critical percent value where any higher value would be called as significant, and Filliben’s correlation for how well the noise was modelled by the zero adjusted gamma distribution.

| Base | Average percent signal | Average peak area | Critical percent value | model mu | Fillibens correlation |
| --- | --- | --- | --- | --- | --- |
| A | 91.26786 | 367.5000 | 6.537220 | 2.443624 | 0.9924938 |
| C | 92.07874 | 428.1449 | 7.145838 | 2.462743 | 0.9896805 |
| G | 93.17229 | 451.7368 | 9.023865 | 2.614256 | 0.9954098 |
| T | 93.40458 | 468.0233 | 7.926881 | 2.842770 | 0.9954269 |

## Predicted editing

### Editing bar plot

### Editing table plot

### Table of editing results


Here’s the entire guide region

| Sanger position | Guide position | Guide sequence | Sanger base call | Focal base | Focal base peak area | p value |  |
| --- | --- | --- | --- | --- | --- | --- | --- |
| 282 | 1 | A | A | A | 93.88 | 0.000000000 | \* |
| 282 | 1 | A | A | C | 3.36 | 0.221218166 |  |
| 282 | 1 | A | A | G | 0.92 | 0.787157000 |  |
| 282 | 1 | A | A | T | 1.83 | 0.679767616 |  |
| 283 | 2 | A | A | A | 92.59 | 0.000000000 | \* |
| 283 | 2 | A | A | C | 3.17 | 0.252750921 |  |
| 283 | 2 | A | A | G | 1.85 | 0.546051988 |  |
| 283 | 2 | A | A | T | 2.38 | 0.529464002 |  |
| 284 | 3 | G | G | A | 3.90 | 0.128180108 |  |
| 284 | 3 | G | G | C | 0.90 | 0.852979698 |  |
| 284 | 3 | G | G | G | 92.49 | 0.000000000 | \* |
| 284 | 3 | G | G | T | 2.70 | 0.447604534 |  |
| 285 | 4 | A | A | A | 93.08 | 0.000000000 | \* |
| 285 | 4 | A | A | C | 1.33 | 0.732071573 |  |
| 285 | 4 | A | A | G | 5.06 | 0.104287346 |  |
| 285 | 4 | A | A | T | 0.53 | 0.959836616 |  |
| 286 | 5 | C | C | A | 3.12 | 0.246269840 |  |
| 286 | 5 | C | C | C | 90.65 | 0.000000000 | \* |
| 286 | 5 | C | C | G | 4.01 | 0.186439296 |  |
| 286 | 5 | C | C | T | 2.23 | 0.570801627 |  |
| 287 | 6 | T | T | A | 3.55 | 0.174056182 |  |
| 287 | 6 | T | T | C | 4.02 | 0.136435524 |  |
| 287 | 6 | T | T | G | 3.78 | 0.210526358 |  |
| 287 | 6 | T | T | T | 88.65 | 0.000000000 | \* |
| 288 | 7 | G | G | A | 1.83 | 0.588742280 |  |
| 288 | 7 | G | G | C | 1.26 | 0.753535945 |  |
| 288 | 7 | G | G | G | 95.99 | 0.000000000 | \* |
| 288 | 7 | G | G | T | 0.92 | 0.906478381 |  |
| 289 | 8 | G | G | A | 4.92 | 0.050423501 |  |
| 289 | 8 | G | G | C | 1.12 | 0.794717823 |  |
| 289 | 8 | G | G | G | 93.96 | 0.000000000 | \* |
| 289 | 8 | G | G | T | 0.00 | 0.978723404 |  |
| 290 | 9 | C | C | A | 8.41 | 0.001330226 | \* |
| 290 | 9 | C | C | C | 86.73 | 0.000000000 | \* |
| 290 | 9 | C | C | G | 2.91 | 0.330512186 |  |
| 290 | 9 | C | C | T | 1.94 | 0.649857650 |  |
| 291 | 10 | C | C | A | 5.22 | 0.037907971 |  |
| 291 | 10 | C | C | C | 91.74 | 0.000000000 | \* |
| 291 | 10 | C | C | G | 1.09 | 0.742556848 |  |
| 291 | 10 | C | C | T | 1.96 | 0.645724980 |  |
| 292 | 11 | A | A | A | 89.58 | 0.000000000 | \* |
| 292 | 11 | A | A | C | 5.21 | 0.052824134 |  |
| 292 | 11 | A | A | G | 2.28 | 0.449482459 |  |
| 292 | 11 | A | A | T | 2.93 | 0.394064222 |  |
| 293 | 12 | G | G | A | 3.29 | 0.214891639 |  |
| 293 | 12 | G | G | C | 2.30 | 0.445036546 |  |
| 293 | 12 | G | G | G | 92.76 | 0.000000000 | \* |
| 293 | 12 | G | G | T | 1.64 | 0.732537500 |  |
| 294 | 13 | A | A | A | 93.19 | 0.000000000 | \* |
| 294 | 13 | A | A | C | 1.21 | 0.768235057 |  |
| 294 | 13 | A | A | G | 3.18 | 0.289033063 |  |
| 294 | 13 | A | A | T | 2.42 | 0.519017950 |  |
| 295 | 14 | C | C | A | 2.51 | 0.387190568 |  |
| 295 | 14 | C | C | C | 89.29 | 0.000000000 | \* |
| 295 | 14 | C | C | G | 3.64 | 0.226523114 |  |
| 295 | 14 | C | C | T | 4.56 | 0.138474687 |  |
| 296 | 15 | C | C | A | 3.12 | 0.244929156 |  |
| 296 | 15 | C | C | C | 83.75 | 0.000000000 | \* |
| 296 | 15 | C | C | G | 5.31 | 0.090383027 |  |
| 296 | 15 | C | C | T | 7.81 | 0.011000801 |  |
| 297 | 16 | G | G | A | 2.48 | 0.392882947 |  |
| 297 | 16 | G | G | C | 0.83 | 0.870536658 |  |
| 297 | 16 | G | G | G | 91.30 | 0.000000000 | \* |
| 297 | 16 | G | G | T | 5.38 | 0.075866494 |  |
| 298 | 17 | T | T | A | 1.44 | 0.716295070 |  |
| 298 | 17 | T | T | C | 1.72 | 0.611671593 |  |
| 298 | 17 | T | T | G | 2.30 | 0.445550694 |  |
| 298 | 17 | T | T | T | 94.54 | 0.000000000 | \* |
| 299 | 18 | G | G | A | 0.98 | 0.846284869 |  |
| 299 | 18 | G | G | C | 0.00 | 0.963414634 |  |
| 299 | 18 | G | G | G | 96.78 | 0.000000000 | \* |
| 299 | 18 | G | G | T | 2.24 | 0.567918409 |  |
| 300 | 19 | T | T | A | 1.27 | 0.767645840 |  |
| 300 | 19 | T | T | C | 1.52 | 0.673543689 |  |
| 300 | 19 | T | T | G | 5.58 | 0.077410100 |  |
| 300 | 19 | T | T | T | 91.62 | 0.000000000 | \* |
| 301 | 20 | T | T | A | 1.53 | 0.686133826 |  |
| 301 | 20 | T | T | C | 1.31 | 0.737698544 |  |
| 301 | 20 | T | T | G | 2.84 | 0.341942655 |  |
| 301 | 20 | T | T | T | 94.31 | 0.000000000 | \* |
| 302 | 21 | T | T | A | 0.45 | 0.933787114 |  |
| 302 | 21 | T | T | C | 2.23 | 0.463943189 |  |
| 302 | 21 | T | T | G | 3.35 | 0.264601046 |  |
| 302 | 21 | T | T | T | 93.97 | 0.000000000 | \* |
| 303 | 22 | G | G | A | 1.55 | 0.681429699 |  |
| 303 | 22 | G | G | C | 1.72 | 0.613480961 |  |
| 303 | 22 | G | G | G | 95.88 | 0.000000000 | \* |
| 303 | 22 | G | G | T | 0.86 | 0.916612304 |  |
| 304 | 23 | T | T | A | 1.78 | 0.606884631 |  |
| 304 | 23 | T | T | C | 3.05 | 0.276237205 |  |
| 304 | 23 | T | T | G | 1.27 | 0.694260398 |  |
| 304 | 23 | T | T | T | 93.91 | 0.000000000 | \* |
| 305 | 24 | G | G | A | 0.79 | 0.886765781 |  |
| 305 | 24 | G | G | C | 1.98 | 0.535021918 |  |
| 305 | 24 | G | G | G | 94.85 | 0.000000000 | \* |
| 305 | 24 | G | G | T | 2.38 | 0.530713032 |  |
| 306 | 25 | C | C | A | 1.99 | 0.540070356 |  |
| 306 | 25 | C | C | C | 92.06 | 0.000000000 | \* |
| 306 | 25 | C | C | G | 2.23 | 0.459437764 |  |
| 306 | 25 | C | C | T | 3.72 | 0.243370140 |  |
| 307 | 26 | A | A | A | 91.26 | 0.000000000 | \* |
| 307 | 26 | A | A | C | 4.55 | 0.090596635 |  |
| 307 | 26 | A | A | G | 0.70 | 0.842323041 |  |
| 307 | 26 | A | A | T | 3.50 | 0.280917298 |  |
| 308 | 27 | C | C | A | 1.93 | 0.558279399 |  |
| 308 | 27 | C | C | C | 95.18 | 0.000000000 | \* |
| 308 | 27 | C | C | G | 0.48 | 0.891964924 |  |
| 308 | 27 | C | C | T | 2.41 | 0.521892003 |  |
| 309 | 28 | T | T | A | 2.62 | 0.357678021 |  |
| 309 | 28 | T | T | C | 3.81 | 0.159778757 |  |
| 309 | 28 | T | T | G | 0.71 | 0.838663739 |  |
| 309 | 28 | T | T | T | 92.86 | 0.000000000 | \* |
| 310 | 29 | A | A | A | 91.40 | 0.000000000 | \* |
| 310 | 29 | A | A | C | 2.71 | 0.344297361 |  |
| 310 | 29 | A | A | G | 4.07 | 0.180146861 |  |
| 310 | 29 | A | A | T | 1.81 | 0.686727853 |  |
| 311 | 30 | C | C | A | 2.04 | 0.522588760 |  |
| 311 | 30 | C | C | C | 90.56 | 0.000000000 | \* |
| 311 | 30 | C | C | G | 5.61 | 0.076155166 |  |
| 311 | 30 | C | C | T | 1.79 | 0.693492999 |  |
| 312 | 31 | A | A | A | 86.74 | 0.000000000 | \* |
| 312 | 31 | A | A | C | 2.87 | 0.311511559 |  |
| 312 | 31 | A | A | G | 8.96 | 0.010394312 |  |
| 312 | 31 | A | A | T | 1.43 | 0.789131540 |  |
| 313 | 32 | C | C | A | 1.14 | 0.805593417 |  |
| 313 | 32 | C | C | C | 93.47 | 0.000000000 | \* |
| 313 | 32 | C | C | G | 3.41 | 0.256354386 |  |
| 313 | 32 | C | C | T | 1.99 | 0.636748977 |  |
| 314 | 33 | G | G | A | 4.46 | 0.078011767 |  |
| 314 | 33 | G | G | C | 3.06 | 0.272786211 |  |
| 314 | 33 | G | G | G | 92.48 | 0.000000000 | \* |
| 314 | 33 | G | G | T | 0.00 | 0.978723404 |  |
| 315 | 34 | G | G | A | 1.36 | 0.739056151 |  |
| 315 | 34 | G | G | C | 3.64 | 0.181644241 |  |
| 315 | 34 | G | G | G | 94.77 | 0.000000000 | \* |
| 315 | 34 | G | G | T | 0.23 | 0.976881190 |  |
| 316 | 35 | G | G | A | 0.32 | 0.941851291 |  |
| 316 | 35 | G | G | C | 6.62 | 0.015845416 |  |
| 316 | 35 | G | G | G | 91.80 | 0.000000000 | \* |
| 316 | 35 | G | G | T | 1.26 | 0.832357646 |  |
| 317 | 36 | C | C | A | 1.75 | 0.614100894 |  |
| 317 | 36 | C | C | C | 94.44 | 0.000000000 | \* |
| 317 | 36 | C | C | G | 1.46 | 0.643637778 |  |
| 317 | 36 | C | C | T | 2.34 | 0.540572917 |  |
| 318 | 37 | A | A | A | 88.47 | 0.000000000 | \* |
| 318 | 37 | A | A | C | 5.08 | 0.058639420 |  |
| 318 | 37 | A | A | G | 2.71 | 0.365195492 |  |
| 318 | 37 | A | A | T | 3.73 | 0.242314839 |  |
| 319 | 38 | C | C | A | 1.20 | 0.788841089 |  |
| 319 | 38 | C | C | C | 92.11 | 0.000000000 | \* |
| 319 | 38 | C | C | G | 3.35 | 0.264454409 |  |
| 319 | 38 | C | C | T | 3.35 | 0.307746897 |  |
| 320 | 39 | C | C | A | 2.98 | 0.274545649 |  |
| 320 | 39 | C | C | C | 89.33 | 0.000000000 | \* |
| 320 | 39 | C | C | G | 5.71 | 0.072110734 |  |
| 320 | 39 | C | C | T | 1.99 | 0.637733541 |  |
| 321 | 40 | C | C | A | 2.38 | 0.421377879 |  |
| 321 | 40 | C | C | C | 94.97 | 0.000000000 | \* |
| 321 | 40 | C | C | G | 0.53 | 0.881841219 |  |
| 321 | 40 | C | C | T | 2.12 | 0.601219061 |  |
| 322 | 41 | T | T | A | 0.85 | 0.875776282 |  |
| 322 | 41 | T | T | C | 5.65 | 0.036652939 |  |
| 322 | 41 | T | T | G | 3.11 | 0.299511481 |  |
| 322 | 41 | T | T | T | 90.40 | 0.000000000 | \* |
| 323 | 42 | G | G | A | 1.24 | 0.774968848 |  |
| 323 | 42 | G | G | C | 1.71 | 0.615767491 |  |
| 323 | 42 | G | G | G | 96.11 | 0.000000000 | \* |
| 323 | 42 | G | G | T | 0.93 | 0.903384913 |  |
| 324 | 43 | A | A | A | 92.52 | 0.000000000 | \* |
| 324 | 43 | A | A | C | 2.04 | 0.517460626 |  |
| 324 | 43 | A | A | G | 4.54 | 0.139825635 |  |
| 324 | 43 | A | A | T | 0.91 | 0.908180473 |  |
| 325 | 44 | C | C | A | 3.20 | 0.230843397 |  |
| 325 | 44 | C | C | C | 95.20 | 0.000000000 | \* |
| 325 | 44 | C | C | G | 0.00 | 0.955555556 |  |
| 325 | 44 | C | C | T | 1.60 | 0.744766279 |  |
| 326 | 45 | C | C | A | 2.24 | 0.460956988 |  |
| 326 | 45 | C | C | C | 93.59 | 0.000000000 | \* |
| 326 | 45 | C | C | G | 2.88 | 0.335182446 |  |
| 326 | 45 | C | C | T | 1.28 | 0.827446771 |  |
| 327 | 46 | G | G | A | 2.35 | 0.430163092 |  |
| 327 | 46 | G | G | C | 1.83 | 0.580270770 |  |
| 327 | 46 | G | G | G | 94.78 | 0.000000000 | \* |
| 327 | 46 | G | G | T | 1.04 | 0.881423010 |  |
| 328 | 47 | A | A | A | 93.79 | 0.000000000 | \* |
| 328 | 47 | A | A | C | 1.71 | 0.615054904 |  |
| 328 | 47 | A | A | G | 3.64 | 0.227049788 |  |
| 328 | 47 | A | A | T | 0.86 | 0.917051391 |  |
| 329 | 48 | C | C | A | 2.76 | 0.322954273 |  |
| 329 | 48 | C | C | C | 94.79 | 0.000000000 | \* |
| 329 | 48 | C | C | G | 0.92 | 0.786424725 |  |
| 329 | 48 | C | C | T | 1.53 | 0.762674040 |  |
| 330 | 49 | G | G | A | 8.60 | 0.001086382 | \* |
| 330 | 49 | G | G | C | 2.26 | 0.455759424 |  |
| 330 | 49 | G | G | G | 89.14 | 0.000000000 | \* |
| 330 | 49 | G | G | T | 0.00 | 0.978723404 |  |
| 331 | 50 | G | G | A | 3.03 | 0.263665435 |  |
| 331 | 50 | G | G | C | 1.26 | 0.752761001 |  |
| 331 | 50 | G | G | G | 94.95 | 0.000000000 | \* |
| 331 | 50 | G | G | T | 0.76 | 0.932791218 |  |
| 332 | 51 | C | C | A | 5.41 | 0.031530139 |  |
| 332 | 51 | C | C | C | 88.42 | 0.000000000 | \* |
| 332 | 51 | C | C | G | 3.86 | 0.201875517 |  |
| 332 | 51 | C | C | T | 2.32 | 0.546616819 |  |
| 333 | 52 | A | A | A | 90.76 | 0.000000000 | \* |
| 333 | 52 | A | A | C | 3.36 | 0.221623365 |  |
| 333 | 52 | A | A | G | 0.84 | 0.807053168 |  |
| 333 | 52 | A | A | T | 5.04 | 0.097652348 |  |
| 334 | 53 | A | A | A | 96.30 | 0.000000000 | \* |
| 334 | 53 | A | A | C | 0.34 | 0.951572294 |  |
| 334 | 53 | A | A | G | 1.68 | 0.587224911 |  |
| 334 | 53 | A | A | T | 1.68 | 0.721867737 |  |
| 335 | 54 | G | G | A | 2.47 | 0.397028563 |  |
| 335 | 54 | G | G | C | 0.41 | 0.944176361 |  |
| 335 | 54 | G | G | G | 92.59 | 0.000000000 | \* |
| 335 | 54 | G | G | T | 4.53 | 0.141334452 |  |
| 336 | 55 | A | A | A | 93.70 | 0.000000000 | \* |
| 336 | 55 | A | A | C | 0.00 | 0.963414634 |  |
| 336 | 55 | A | A | G | 1.21 | 0.709730865 |  |
| 336 | 55 | A | A | T | 5.08 | 0.094642038 |  |
| 337 | 56 | A | A | A | 92.28 | 0.000000000 | \* |
| 337 | 56 | A | A | C | 1.85 | 0.573009152 |  |
| 337 | 56 | A | A | G | 1.23 | 0.703389178 |  |
| 337 | 56 | A | A | T | 4.63 | 0.131437241 |  |
| 338 | 57 | G | G | A | 2.83 | 0.306828349 |  |
| 338 | 57 | G | G | C | 2.36 | 0.430387308 |  |
| 338 | 57 | G | G | G | 91.51 | 0.000000000 | \* |
| 338 | 57 | G | G | T | 3.30 | 0.316782098 |  |
| 339 | 58 | T | T | A | 2.80 | 0.313505628 |  |
| 339 | 58 | T | T | C | 1.12 | 0.794184406 |  |
| 339 | 58 | T | T | G | 1.12 | 0.733674200 |  |
| 339 | 58 | T | T | T | 94.96 | 0.000000000 | \* |
| 340 | 59 | T | T | A | 2.70 | 0.338658959 |  |
| 340 | 59 | T | T | C | 2.16 | 0.484778965 |  |
| 340 | 59 | T | T | G | 1.62 | 0.603855840 |  |
| 340 | 59 | T | T | T | 93.53 | 0.000000000 | \* |
| 341 | 60 | C | C | A | 2.10 | 0.503605585 |  |
| 341 | 60 | C | C | C | 94.89 | 0.000000000 | \* |
| 341 | 60 | C | C | G | 0.90 | 0.791450555 |  |
| 341 | 60 | C | C | T | 2.10 | 0.605176129 |  |
| 342 | 61 | G | G | A | 4.46 | 0.077477130 |  |
| 342 | 61 | G | G | C | 2.23 | 0.463943189 |  |
| 342 | 61 | G | G | G | 93.30 | 0.000000000 | \* |
| 342 | 61 | G | G | T | 0.00 | 0.978723404 |  |
| 343 | 62 | A | A | A | 93.40 | 0.000000000 | \* |
| 343 | 62 | A | A | C | 2.20 | 0.472581401 |  |
| 343 | 62 | A | A | G | 3.18 | 0.288813926 |  |
| 343 | 62 | A | A | T | 1.22 | 0.841757274 |  |
| 344 | 63 | C | C | A | 2.23 | 0.463602124 |  |
| 344 | 63 | C | C | C | 93.30 | 0.000000000 | \* |
| 344 | 63 | C | C | G | 0.56 | 0.875290645 |  |
| 344 | 63 | C | C | T | 3.91 | 0.215178251 |  |
| 345 | 64 | A | A | A | 84.66 | 0.000000000 | \* |
| 345 | 64 | A | A | C | 6.88 | 0.012676748 |  |
| 345 | 64 | A | A | G | 3.17 | 0.289389027 |  |
| 345 | 64 | A | A | T | 5.29 | 0.081260141 |  |
| 346 | 65 | G | G | A | 0.84 | 0.877239041 |  |
| 346 | 65 | G | G | C | 4.48 | 0.095276129 |  |
| 346 | 65 | G | G | G | 93.00 | 0.000000000 | \* |
| 346 | 65 | G | G | T | 1.68 | 0.722648554 |  |
| 347 | 66 | C | C | A | 0.00 | 0.946428571 |  |
| 347 | 66 | C | C | C | 95.76 | 0.000000000 | \* |
| 347 | 66 | C | C | G | 1.82 | 0.554153805 |  |
| 347 | 66 | C | C | T | 2.42 | 0.518055726 |  |
| 348 | 67 | T | T | A | 0.25 | 0.944108584 |  |
| 348 | 67 | T | T | C | 2.02 | 0.524874307 |  |
| 348 | 67 | T | T | G | 2.02 | 0.507781704 |  |
| 348 | 67 | T | T | T | 95.72 | 0.000000000 | \* |
| 349 | 68 | C | C | A | 1.91 | 0.563111829 |  |
| 349 | 68 | C | C | C | 92.62 | 0.000000000 | \* |
| 349 | 68 | C | C | G | 3.28 | 0.274299368 |  |
| 349 | 68 | C | C | T | 2.19 | 0.582111230 |  |
| 350 | 69 | C | C | A | 1.80 | 0.597991969 |  |
| 350 | 69 | C | C | C | 94.33 | 0.000000000 | \* |
| 350 | 69 | C | C | G | 2.06 | 0.497147292 |  |
| 350 | 69 | C | C | T | 1.80 | 0.688356081 |  |
| 351 | 70 | C | C | A | 7.24 | 0.004765986 | \* |
| 351 | 70 | C | C | C | 89.14 | 0.000000000 | \* |
| 351 | 70 | C | C | G | 2.71 | 0.364644248 |  |
| 351 | 70 | C | C | T | 0.90 | 0.908551560 |  |
| 352 | 71 | G | G | A | 5.94 | 0.018521072 |  |
| 352 | 71 | G | G | C | 2.19 | 0.476151878 |  |
| 352 | 71 | G | G | G | 91.25 | 0.000000000 | \* |
| 352 | 71 | G | G | T | 0.62 | 0.950246485 |  |
| 353 | 72 | C | C | A | 0.00 | 0.946428571 |  |
| 353 | 72 | C | C | C | 97.07 | 0.000000000 | \* |
| 353 | 72 | C | C | G | 1.30 | 0.685301049 |  |
| 353 | 72 | C | C | T | 1.63 | 0.736942145 |  |
| 354 | 73 | G | G | A | 4.08 | 0.109591388 |  |
| 354 | 73 | G | G | C | 3.06 | 0.273317601 |  |
| 354 | 73 | G | G | G | 92.86 | 0.000000000 | \* |
| 354 | 73 | G | G | T | 0.00 | 0.978723404 |  |
| 355 | 74 | A | A | A | 91.88 | 0.000000000 | \* |
| 355 | 74 | A | A | C | 1.57 | 0.658800580 |  |
| 355 | 74 | A | A | G | 4.19 | 0.169138543 |  |
| 355 | 74 | A | A | T | 2.36 | 0.536082923 |  |
| 356 | 75 | C | C | A | 1.97 | 0.544266897 |  |
| 356 | 75 | C | C | C | 91.27 | 0.000000000 | \* |
| 356 | 75 | C | C | G | 3.94 | 0.193112523 |  |
| 356 | 75 | C | C | T | 2.82 | 0.420363913 |  |

## For use in R

If you want to work with the results in R, here is output that you can copy and paste in your terminal to get:

The base information:

```
structure(list(focal.base = c("A", "C", "G", "T"), avg.percsignal = c(91.2678621679189, 
92.078744112771, 93.1722915576115, 93.4045783425676), avg.areasignal = c(367.5, 
428.144927536232, 451.736842105263, 468.023255813953), crit.perc.area = c(6.53722039426401, 
7.14583781490893, 9.02386549679342, 7.92688111113379), mu = c(2.44362442916747, 
2.46274284356437, 2.61425581629546, 2.84276966419014), fillibens = c(0.992493848355824, 
0.989680543083102, 0.995409750274427, 0.995426856365325)), class = "data.frame", row.names = c(NA, 
-4L))
```

the data.frame that contains information on the guide region:

```
structure(list(A.area = c(307, 350, 13, 699, 14, 15, 16, 22, 
26, 24, 275, 10, 616, 11, 10, 12, 5, 7, 5, 7, 2, 9, 7, 4, 8, 
261, 8, 11, 404, 8, 242, 4, 16, 6, 1, 6, 261, 5, 12, 9, 3, 8, 
408, 12, 7, 9, 438, 9, 19, 12, 14, 108, 286, 6, 387, 299, 6, 
10, 10, 7, 10, 382, 8, 160, 3, 0, 1, 7, 7, 16, 19, 0, 12, 351, 
7), C.area = c(11, 12, 3, 10, 407, 17, 11, 5, 268, 422, 16, 7, 
8, 392, 268, 4, 6, 0, 6, 6, 10, 10, 12, 10, 371, 13, 395, 16, 
12, 355, 8, 329, 11, 16, 21, 323, 15, 385, 360, 359, 20, 11, 
9, 357, 292, 7, 8, 309, 5, 5, 229, 4, 1, 1, 0, 6, 5, 4, 8, 316, 
5, 9, 334, 13, 16, 316, 8, 339, 366, 197, 7, 298, 9, 6, 324), 
    G.area = c(3, 7, 308, 38, 18, 16, 838, 420, 9, 5, 7, 282, 
    21, 16, 17, 441, 8, 692, 22, 13, 15, 558, 5, 479, 9, 2, 2, 
    3, 18, 22, 25, 12, 332, 417, 291, 5, 8, 14, 23, 2, 11, 618, 
    20, 0, 9, 363, 17, 3, 197, 376, 10, 1, 5, 225, 5, 4, 194, 
    4, 6, 3, 209, 13, 2, 6, 332, 6, 8, 12, 8, 6, 292, 4, 273, 
    16, 14), T.area = c(6, 9, 9, 4, 10, 375, 8, 0, 6, 9, 9, 5, 
    16, 20, 25, 26, 329, 16, 361, 431, 421, 5, 370, 12, 15, 10, 
    10, 390, 8, 7, 4, 7, 0, 1, 4, 8, 11, 14, 8, 8, 320, 6, 4, 
    6, 4, 4, 4, 5, 0, 3, 6, 6, 5, 11, 21, 15, 7, 339, 347, 7, 
    0, 5, 14, 10, 6, 8, 380, 8, 7, 2, 2, 5, 0, 9, 10), Tot.area = c(327, 
    378, 333, 751, 449, 423, 873, 447, 309, 460, 307, 304, 661, 
    439, 320, 483, 348, 715, 394, 457, 448, 582, 394, 505, 403, 
    286, 415, 420, 442, 392, 279, 352, 359, 440, 317, 342, 295, 
    418, 403, 378, 354, 643, 441, 375, 312, 383, 467, 326, 221, 
    396, 259, 119, 297, 243, 413, 324, 212, 357, 371, 333, 224, 
    409, 358, 189, 357, 330, 397, 366, 388, 221, 320, 307, 294, 
    382, 355), A.perc = c(93.8837920489297, 92.5925925925926, 
    3.9039039039039, 93.0758988015979, 3.11804008908686, 3.54609929078014, 
    1.83276059564719, 4.92170022371365, 8.41423948220065, 5.21739130434783, 
    89.5765472312704, 3.28947368421053, 93.1921331316188, 2.50569476082005, 
    3.125, 2.48447204968944, 1.4367816091954, 0.979020979020979, 
    1.26903553299492, 1.53172866520788, 0.446428571428571, 1.54639175257732, 
    1.77664974619289, 0.792079207920792, 1.98511166253102, 91.2587412587413, 
    1.92771084337349, 2.61904761904762, 91.4027149321267, 2.04081632653061, 
    86.7383512544803, 1.13636363636364, 4.45682451253482, 1.36363636363636, 
    0.315457413249211, 1.75438596491228, 88.4745762711864, 1.19617224880383, 
    2.97766749379653, 2.38095238095238, 0.847457627118644, 1.24416796267496, 
    92.5170068027211, 3.2, 2.24358974358974, 2.34986945169713, 
    93.7901498929336, 2.76073619631902, 8.5972850678733, 3.03030303030303, 
    5.40540540540541, 90.7563025210084, 96.2962962962963, 2.46913580246914, 
    93.7046004842615, 92.2839506172839, 2.83018867924528, 2.80112044817927, 
    2.69541778975741, 2.1021021021021, 4.46428571428571, 93.398533007335, 
    2.23463687150838, 84.6560846560847, 0.840336134453782, 0, 
    0.251889168765743, 1.91256830601093, 1.80412371134021, 7.23981900452489, 
    5.9375, 0, 4.08163265306122, 91.8848167539267, 1.97183098591549
    ), C.perc = c(3.36391437308869, 3.17460317460317, 0.900900900900901, 
    1.33155792276964, 90.6458797327394, 4.01891252955083, 1.26002290950745, 
    1.11856823266219, 86.7313915857605, 91.7391304347826, 5.21172638436482, 
    2.30263157894737, 1.21028744326778, 89.2938496583144, 83.75, 
    0.82815734989648, 1.72413793103448, 0, 1.52284263959391, 
    1.31291028446389, 2.23214285714286, 1.71821305841924, 3.04568527918782, 
    1.98019801980198, 92.0595533498759, 4.54545454545455, 95.1807228915663, 
    3.80952380952381, 2.71493212669683, 90.5612244897959, 2.8673835125448, 
    93.4659090909091, 3.06406685236769, 3.63636363636364, 6.62460567823344, 
    94.4444444444444, 5.08474576271186, 92.1052631578947, 89.3300248138958, 
    94.973544973545, 5.64971751412429, 1.71073094867807, 2.04081632653061, 
    95.2, 93.5897435897436, 1.82767624020888, 1.71306209850107, 
    94.7852760736196, 2.26244343891403, 1.26262626262626, 88.4169884169884, 
    3.36134453781513, 0.336700336700337, 0.411522633744856, 0, 
    1.85185185185185, 2.35849056603774, 1.12044817927171, 2.15633423180593, 
    94.8948948948949, 2.23214285714286, 2.20048899755501, 93.2960893854749, 
    6.87830687830688, 4.48179271708683, 95.7575757575758, 2.01511335012594, 
    92.6229508196721, 94.3298969072165, 89.1402714932127, 2.1875, 
    97.0684039087948, 3.06122448979592, 1.57068062827225, 91.2676056338028
    ), G.perc = c(0.917431192660551, 1.85185185185185, 92.4924924924925, 
    5.05992010652463, 4.00890868596882, 3.78250591016548, 95.9908361970218, 
    93.9597315436242, 2.9126213592233, 1.08695652173913, 2.28013029315961, 
    92.7631578947368, 3.17700453857791, 3.64464692482916, 5.3125, 
    91.304347826087, 2.29885057471264, 96.7832167832168, 5.58375634517767, 
    2.84463894967177, 3.34821428571429, 95.8762886597938, 1.26903553299492, 
    94.8514851485149, 2.23325062034739, 0.699300699300699, 0.481927710843373, 
    0.714285714285714, 4.07239819004525, 5.61224489795918, 8.96057347670251, 
    3.40909090909091, 92.4791086350975, 94.7727272727273, 91.7981072555205, 
    1.46198830409357, 2.71186440677966, 3.34928229665072, 5.70719602977668, 
    0.529100529100529, 3.10734463276836, 96.1119751166407, 4.53514739229025, 
    0, 2.88461538461538, 94.7780678851175, 3.64025695931478, 
    0.920245398773006, 89.1402714932127, 94.9494949494949, 3.86100386100386, 
    0.840336134453782, 1.68350168350168, 92.5925925925926, 1.21065375302663, 
    1.23456790123457, 91.5094339622642, 1.12044817927171, 1.61725067385445, 
    0.900900900900901, 93.3035714285714, 3.17848410757946, 0.558659217877095, 
    3.17460317460317, 92.9971988795518, 1.81818181818182, 2.01511335012594, 
    3.27868852459016, 2.06185567010309, 2.71493212669683, 91.25, 
    1.30293159609121, 92.8571428571429, 4.18848167539267, 3.94366197183099
    ), T.perc = c(1.8348623853211, 2.38095238095238, 2.7027027027027, 
    0.532623169107856, 2.2271714922049, 88.6524822695036, 0.916380297823597, 
    0, 1.94174757281553, 1.95652173913043, 2.93159609120521, 
    1.64473684210526, 2.42057488653555, 4.55580865603645, 7.8125, 
    5.38302277432712, 94.5402298850575, 2.23776223776224, 91.6243654822335, 
    94.3107221006564, 93.9732142857143, 0.859106529209622, 93.9086294416244, 
    2.37623762376238, 3.72208436724566, 3.4965034965035, 2.40963855421687, 
    92.8571428571429, 1.80995475113122, 1.78571428571429, 1.4336917562724, 
    1.98863636363636, 0, 0.227272727272727, 1.26182965299685, 
    2.33918128654971, 3.72881355932203, 3.34928229665072, 1.98511166253102, 
    2.11640211640212, 90.3954802259887, 0.933125972006221, 0.90702947845805, 
    1.6, 1.28205128205128, 1.0443864229765, 0.856531049250535, 
    1.53374233128834, 0, 0.757575757575758, 2.31660231660232, 
    5.04201680672269, 1.68350168350168, 4.52674897119342, 5.08474576271186, 
    4.62962962962963, 3.30188679245283, 94.9579831932773, 93.5309973045822, 
    2.1021021021021, 0, 1.22249388753056, 3.91061452513966, 5.29100529100529, 
    1.68067226890756, 2.42424242424242, 95.7178841309824, 2.18579234972678, 
    1.80412371134021, 0.904977375565611, 0.625, 1.62866449511401, 
    0, 2.35602094240838, 2.8169014084507), base.call = c("A", 
    "A", "G", "A", "C", "T", "G", "G", "C", "C", "A", "G", "A", 
    "C", "C", "G", "T", "G", "T", "T", "T", "G", "T", "G", "C", 
    "A", "C", "T", "A", "C", "A", "C", "G", "G", "G", "C", "A", 
    "C", "C", "C", "T", "G", "A", "C", "C", "G", "A", "C", "G", 
    "G", "C", "A", "A", "G", "A", "A", "G", "T", "T", "C", "G", 
    "A", "C", "A", "G", "C", "T", "C", "C", "C", "G", "C", "G", 
    "A", "C"), index = 282:356, guide.seq = c("A", "A", "G", 
    "A", "C", "T", "G", "G", "C", "C", "A", "G", "A", "C", "C", 
    "G", "T", "G", "T", "T", "T", "G", "T", "G", "C", "A", "C", 
    "T", "A", "C", "A", "C", "G", "G", "G", "C", "A", "C", "C", 
    "C", "T", "G", "A", "C", "C", "G", "A", "C", "G", "G", "C", 
    "A", "A", "G", "A", "A", "G", "T", "T", "C", "G", "A", "C", 
    "A", "G", "C", "T", "C", "C", "C", "G", "C", "G", "A", "C"
    ), T.pval = c(0.679767616247777, 0.529464002403029, 0.447604533736593, 
    0.959836615668139, 0.57080162658611, 0, 0.906478381413787, 
    0.978723404255317, 0.64985765010087, 0.645724979731531, 0.394064222178659, 
    0.732537500212429, 0.519017949777885, 0.13847468693421, 0.0110008010502044, 
    0.0758664940024126, 0, 0.567918409153735, 0, 0, 0, 0.916612304367587, 
    0, 0.530713031603232, 0.243370140357146, 0.280917298381483, 
    0.521892003479584, 0, 0.686727852825915, 0.693492998700025, 
    0.789131539647483, 0.636748977419824, 0.978723404255317, 
    0.976881190020909, 0.832357645788318, 0.54057291699892, 0.242314838500545, 
    0.307746897172594, 0.637733540941746, 0.601219060683418, 
    0, 0.903384912634711, 0.908180472844386, 0.744766279156462, 
    0.827446770724022, 0.881423010284464, 0.917051391096988, 
    0.762674039852175, 0.978723404255317, 0.932791218230828, 
    0.546616818915476, 0.0976523475406443, 0.721867737498322, 
    0.14133445236343, 0.0946420384838752, 0.131437240930493, 
    0.316782098494403, 0, 0, 0.605176128570148, 0.978723404255317, 
    0.841757273691501, 0.215178251159565, 0.0812601407811846, 
    0.722648553848525, 0.518055725531022, 0, 0.582111230405364, 
    0.688356081451328, 0.908551559735371, 0.950246485101104, 
    0.736942145372785, 0.978723404255317, 0.536082922678167, 
    0.420363913426741), C.pval = c(0.221218166406496, 0.252750921040262, 
    0.852979697687655, 0.732071572709625, 0, 0.136435524320019, 
    0.753535945268663, 0.794717822962978, 0, 0, 0.0528241336268683, 
    0.445036546120219, 0.768235056741701, 0, 0, 0.870536657914819, 
    0.61167159300484, 0.963414634138581, 0.673543688593425, 0.737698544322309, 
    0.463943189063396, 0.613480960679882, 0.27623720482319, 0.535021917533006, 
    0, 0.0905966346794745, 0, 0.159778757083545, 0.344297361456022, 
    0, 0.311511559110526, 0, 0.272786210584952, 0.181644241417836, 
    0.0158454158291162, 0, 0.0586394197073029, 0, 0, 0, 0.0366529386252277, 
    0.61576749103532, 0.517460625673987, 0, 0, 0.580270770111221, 
    0.615054904042864, 0, 0.455759424424677, 0.752761001209311, 
    0, 0.221623364957823, 0.951572294038474, 0.944176360935805, 
    0.963414634138581, 0.573009151545558, 0.43038730760078, 0.794184406149055, 
    0.484778964651086, 0, 0.463943189063396, 0.472581401420972, 
    0, 0.0126767475026944, 0.0952761286262318, 0, 0.524874307250487, 
    0, 0, 0, 0.476151877844675, 0, 0.273317600839646, 0.658800580222495, 
    0), G.pval = c(0.787157000478922, 0.546051987666455, 0, 0.104287346480477, 
    0.186439296049996, 0.210526357798216, 0, 0, 0.33051218631464, 
    0.742556847581604, 0.449482458527511, 0, 0.289033062621121, 
    0.226523113554428, 0.0903830265675077, 0, 0.445550693573128, 
    0, 0.0774100996988361, 0.341942655422626, 0.264601046330787, 
    0, 0.694260398254249, 0, 0.459437764492926, 0.842323041125963, 
    0.89196492385745, 0.838663738591173, 0.18014686110006, 0.0761551659392032, 
    0.0103943124068782, 0.256354385603866, 0, 0, 0, 0.643637778229201, 
    0.365195491688294, 0.264454408809174, 0.0721107340820617, 
    0.881841219351689, 0.299511480901183, 0, 0.139825634604831, 
    0.955555555555295, 0.335182446457458, 0, 0.227049788299748, 
    0.786424724934035, 0, 0, 0.201875516632338, 0.807053168119982, 
    0.587224910756815, 0, 0.709730864934774, 0.703389177936349, 
    0, 0.733674200099385, 0.60385584002561, 0.791450555282885, 
    0, 0.288813925675852, 0.875290644917037, 0.289389026781657, 
    0, 0.554153804957051, 0.507781703940361, 0.274299367807502, 
    0.497147292031469, 0.364644247674552, 0, 0.685301048577573, 
    0, 0.169138543218905, 0.193112523251962), A.pval = c(0, 0, 
    0.128180108393516, 0, 0.246269840084139, 0.174056182165272, 
    0.588742280204597, 0.0504235008320878, 0.00133022621104661, 
    0.0379079706751172, 0, 0.214891639351355, 0, 0.387190567917186, 
    0.244929155910761, 0.392882946938693, 0.716295070293127, 
    0.846284869058159, 0.767645840496861, 0.686133825691106, 
    0.933787114359289, 0.68142969876828, 0.60688463088933, 0.886765780749564, 
    0.540070356033184, 0, 0.558279398626141, 0.357678021144964, 
    0, 0.522588760404844, 0, 0.805593416698437, 0.0780117668681823, 
    0.739056151001826, 0.941851290884556, 0.614100893559599, 
    0, 0.788841089407342, 0.274545649189378, 0.421377879084252, 
    0.87577628169426, 0.774968848139853, 0, 0.230843396990432, 
    0.460956987753344, 0.430163091939773, 0, 0.3229542731108, 
    0.00108638232702263, 0.263665434533, 0.0315301390774458, 
    0, 0, 0.397028563058228, 0, 0, 0.306828348624269, 0.313505627658344, 
    0.338658959479164, 0.503605585398265, 0.0774771298681883, 
    0, 0.463602124006152, 0, 0.877239040779615, 0.946428571428566, 
    0.944108583573523, 0.563111828783251, 0.59799196904414, 0.00476598612840784, 
    0.01852107187853, 0.946428571428566, 0.10959138757718, 0, 
    0.544266897136379), guide.position = 1:75), row.names = 282:356, class = "data.frame")
```

*Report generated using EditR v1.0.10*
