# Supplementary material for: Mechanistic and genetic basis of single-strand templated repair at Cas12a-induced DNA breaks in Chlamydomonas reinhardtii
Source: Nat Commun. 2021 Nov 19;12:6751. doi: 10.1038/s41467-021-27004-1 (PMC8604939; doi:10.1038/s41467-021-27004-1)
Supplement: Supplementary file 22 — Source Data [file 41467_2021_27004_MOESM22_ESM.zip › Source Data/EditR analysis/EditR outputs/wt/rep2_wt.html]

EditR v1.0.10 report


# EditR v1.0.10 report

- Data QA
  - Filtering data
  - Percent noise peak area
  - Base information
- Predicted editing
  - Editing bar plot
  - Editing table plot
  - Table of editing results
- For use in R

## Data QA

### Filtering data

What the data looked like prefiltering:

and the post filtering signal / noise plot:

### Percent noise peak area

### Base information

Here’s information about the signal of each base, the critical percent value where any higher value would be called as significant, and Filliben’s correlation for how well the noise was modelled by the zero adjusted gamma distribution.

| Base | Average percent signal | Average peak area | Critical percent value | model mu | Fillibens correlation |
| --- | --- | --- | --- | --- | --- |
| A | 94.06296 | 347.6944 | 5.280043 | 1.859575 | 0.9964770 |
| C | 93.54910 | 373.0462 | 5.813217 | 2.161466 | 0.9858137 |
| G | 94.74748 | 350.6379 | 6.891684 | 2.001068 | 0.9928523 |
| T | 94.55203 | 379.2143 | 5.888008 | 2.255122 | 0.9963555 |

## Predicted editing

### Editing bar plot

### Editing table plot

### Table of editing results


Here’s the entire guide region

| Sanger position | Guide position | Guide sequence | Sanger base call | Focal base | Focal base peak area | p value |  |
| --- | --- | --- | --- | --- | --- | --- | --- |
| 270 | 1 | A | A | A | 94.27 | 0.0000000000 | \* |
| 270 | 1 | A | A | C | 3.18 | 0.1561533067 |  |
| 270 | 1 | A | A | G | 1.59 | 0.4932429183 |  |
| 270 | 1 | A | A | T | 0.96 | 0.8525562050 |  |
| 271 | 2 | A | A | A | 94.69 | 0.0000000000 | \* |
| 271 | 2 | A | A | C | 2.23 | 0.3507341304 |  |
| 271 | 2 | A | A | G | 1.68 | 0.4690632043 |  |
| 271 | 2 | A | A | T | 1.40 | 0.7112259145 |  |
| 272 | 3 | G | G | A | 4.01 | 0.0440281033 |  |
| 272 | 3 | G | G | C | 0.73 | 0.8060384853 |  |
| 272 | 3 | G | G | G | 93.43 | 0.0000000000 | \* |
| 272 | 3 | G | G | T | 1.82 | 0.5563587385 |  |
| 273 | 4 | A | A | A | 95.04 | 0.0000000000 | \* |
| 273 | 4 | A | A | C | 1.10 | 0.7068060172 |  |
| 273 | 4 | A | A | G | 3.31 | 0.1571526212 |  |
| 273 | 4 | A | A | T | 0.55 | 0.9339331565 |  |
| 274 | 5 | C | C | A | 3.46 | 0.0815464955 |  |
| 274 | 5 | C | C | C | 91.36 | 0.0000000000 | \* |
| 274 | 5 | C | C | G | 2.96 | 0.2004911390 |  |
| 274 | 5 | C | C | T | 2.22 | 0.4215664856 |  |
| 275 | 6 | T | T | A | 1.87 | 0.3832672360 |  |
| 275 | 6 | T | T | C | 3.47 | 0.1197603857 |  |
| 275 | 6 | T | T | G | 3.73 | 0.1151822311 |  |
| 275 | 6 | T | T | T | 90.93 | 0.0000000000 | \* |
| 276 | 7 | G | G | A | 1.23 | 0.6135175429 |  |
| 276 | 7 | G | G | C | 1.10 | 0.7086514786 |  |
| 276 | 7 | G | G | G | 96.58 | 0.0000000000 | \* |
| 276 | 7 | G | G | T | 1.10 | 0.8118821286 |  |
| 277 | 8 | G | G | A | 2.18 | 0.2921832007 |  |
| 277 | 8 | G | G | C | 1.63 | 0.5327578425 |  |
| 277 | 8 | G | G | G | 95.64 | 0.0000000000 | \* |
| 277 | 8 | G | G | T | 0.54 | 0.9346818643 |  |
| 278 | 9 | C | C | A | 4.95 | 0.0149095224 |  |
| 278 | 9 | C | C | C | 90.81 | 0.0000000000 | \* |
| 278 | 9 | C | C | G | 2.83 | 0.2204955142 |  |
| 278 | 9 | C | C | T | 1.41 | 0.7052579704 |  |
| 279 | 10 | C | C | A | 3.55 | 0.0740246849 |  |
| 279 | 10 | C | C | C | 93.62 | 0.0000000000 | \* |
| 279 | 10 | C | C | G | 0.71 | 0.7852505979 |  |
| 279 | 10 | C | C | T | 2.13 | 0.4520435255 |  |
| 280 | 11 | A | A | A | 94.18 | 0.0000000000 | \* |
| 280 | 11 | A | A | C | 4.11 | 0.0633159672 |  |
| 280 | 11 | A | A | G | 1.71 | 0.4588119633 |  |
| 280 | 11 | A | A | T | 0.00 | 0.9578947368 |  |
| 281 | 12 | G | G | A | 3.69 | 0.0633384473 |  |
| 281 | 12 | G | G | C | 2.46 | 0.2937755771 |  |
| 281 | 12 | G | G | G | 93.44 | 0.0000000000 | \* |
| 281 | 12 | G | G | T | 0.41 | 0.9479672669 |  |
| 282 | 13 | A | A | A | 95.49 | 0.0000000000 | \* |
| 282 | 13 | A | A | C | 1.09 | 0.7108579030 |  |
| 282 | 13 | A | A | G | 2.18 | 0.3419093242 |  |
| 282 | 13 | A | A | T | 1.24 | 0.7640501190 |  |
| 283 | 14 | C | C | A | 3.16 | 0.1123616946 |  |
| 283 | 14 | C | C | C | 90.78 | 0.0000000000 | \* |
| 283 | 14 | C | C | G | 2.91 | 0.2076926211 |  |
| 283 | 14 | C | C | T | 3.16 | 0.1912732094 |  |
| 284 | 15 | C | C | A | 1.62 | 0.4675227279 |  |
| 284 | 15 | C | C | C | 90.29 | 0.0000000000 | \* |
| 284 | 15 | C | C | G | 3.56 | 0.1307674333 |  |
| 284 | 15 | C | C | T | 4.53 | 0.0471574845 |  |
| 285 | 16 | G | G | A | 2.01 | 0.3408705071 |  |
| 285 | 16 | G | G | C | 1.25 | 0.6590419847 |  |
| 285 | 16 | G | G | G | 93.23 | 0.0000000000 | \* |
| 285 | 16 | G | G | T | 3.51 | 0.1364246631 |  |
| 286 | 17 | T | T | A | 1.30 | 0.5877773619 |  |
| 286 | 17 | T | T | C | 1.30 | 0.6441979051 |  |
| 286 | 17 | T | T | G | 2.27 | 0.3211447217 |  |
| 286 | 17 | T | T | T | 95.13 | 0.0000000000 | \* |
| 287 | 18 | G | G | A | 0.00 | 0.9166666666 |  |
| 287 | 18 | G | G | C | 0.00 | 0.8720930233 |  |
| 287 | 18 | G | G | G | 98.82 | 0.0000000000 | \* |
| 287 | 18 | G | G | T | 1.18 | 0.7844796870 |  |
| 288 | 19 | T | T | A | 0.59 | 0.8437885301 |  |
| 288 | 19 | T | T | C | 0.59 | 0.8335909459 |  |
| 288 | 19 | T | T | G | 4.69 | 0.0561363476 |  |
| 288 | 19 | T | T | T | 94.13 | 0.0000000000 | \* |
| 289 | 20 | T | T | A | 0.76 | 0.7917093467 |  |
| 289 | 20 | T | T | C | 0.00 | 0.8720930233 |  |
| 289 | 20 | T | T | G | 2.78 | 0.2281330444 |  |
| 289 | 20 | T | T | T | 96.46 | 0.0000000000 | \* |
| 290 | 21 | T | T | A | 0.00 | 0.9166666666 |  |
| 290 | 21 | T | T | C | 0.00 | 0.8720930233 |  |
| 290 | 21 | T | T | G | 3.13 | 0.1777782375 |  |
| 290 | 21 | T | T | T | 96.87 | 0.0000000000 | \* |
| 291 | 22 | G | G | A | 1.02 | 0.6963699634 |  |
| 291 | 22 | G | G | C | 0.82 | 0.7861572735 |  |
| 291 | 22 | G | G | G | 97.55 | 0.0000000000 | \* |
| 291 | 22 | G | G | T | 0.61 | 0.9254997604 |  |
| 292 | 23 | T | T | A | 1.42 | 0.5392306172 |  |
| 292 | 23 | T | T | C | 1.71 | 0.5083633302 |  |
| 292 | 23 | T | T | G | 1.42 | 0.5442030845 |  |
| 292 | 23 | T | T | T | 95.44 | 0.0000000000 | \* |
| 293 | 24 | G | G | A | 1.14 | 0.6513527652 |  |
| 293 | 24 | G | G | C | 1.59 | 0.5472774627 |  |
| 293 | 24 | G | G | G | 95.45 | 0.0000000000 | \* |
| 293 | 24 | G | G | T | 1.82 | 0.5587328246 |  |
| 294 | 25 | C | C | A | 1.85 | 0.3895768215 |  |
| 294 | 25 | C | C | C | 92.35 | 0.0000000000 | \* |
| 294 | 25 | C | C | G | 2.11 | 0.3570112883 |  |
| 294 | 25 | C | C | T | 3.69 | 0.1135226231 |  |
| 295 | 26 | A | A | A | 93.29 | 0.0000000000 | \* |
| 295 | 26 | A | A | C | 4.24 | 0.0553585362 |  |
| 295 | 26 | A | A | G | 0.71 | 0.7861023198 |  |
| 295 | 26 | A | A | T | 1.77 | 0.5772075176 |  |
| 296 | 27 | C | C | A | 2.03 | 0.3334430664 |  |
| 296 | 27 | C | C | C | 95.43 | 0.0000000000 | \* |
| 296 | 27 | C | C | G | 0.25 | 0.9208661956 |  |
| 296 | 27 | C | C | T | 2.28 | 0.4022195562 |  |
| 297 | 28 | T | T | A | 2.85 | 0.1538442716 |  |
| 297 | 28 | T | T | C | 2.85 | 0.2111545887 |  |
| 297 | 28 | T | T | G | 0.52 | 0.8480751945 |  |
| 297 | 28 | T | T | T | 93.78 | 0.0000000000 | \* |
| 298 | 29 | A | A | A | 93.88 | 0.0000000000 | \* |
| 298 | 29 | A | A | C | 2.12 | 0.3831154218 |  |
| 298 | 29 | A | A | G | 2.35 | 0.3045107238 |  |
| 298 | 29 | A | A | T | 1.65 | 0.6206581245 |  |
| 299 | 30 | C | C | A | 1.89 | 0.3768907230 |  |
| 299 | 30 | C | C | C | 92.18 | 0.0000000000 | \* |
| 299 | 30 | C | C | G | 4.04 | 0.0915859682 |  |
| 299 | 30 | C | C | T | 1.89 | 0.5343305850 |  |
| 300 | 31 | A | A | A | 91.21 | 0.0000000000 | \* |
| 300 | 31 | A | A | C | 2.56 | 0.2694949019 |  |
| 300 | 31 | A | A | G | 6.23 | 0.0169850721 |  |
| 300 | 31 | A | A | T | 0.00 | 0.9578947368 |  |
| 301 | 32 | C | C | A | 1.49 | 0.5151990707 |  |
| 301 | 32 | C | C | C | 93.75 | 0.0000000000 | \* |
| 301 | 32 | C | C | G | 2.38 | 0.2988750244 |  |
| 301 | 32 | C | C | T | 2.38 | 0.3731550811 |  |
| 302 | 33 | G | G | A | 3.97 | 0.0461150180 |  |
| 302 | 33 | G | G | C | 1.99 | 0.4213709806 |  |
| 302 | 33 | G | G | G | 93.38 | 0.0000000000 | \* |
| 302 | 33 | G | G | T | 0.66 | 0.9175450733 |  |
| 303 | 34 | G | G | A | 0.78 | 0.7857934730 |  |
| 303 | 34 | G | G | C | 2.33 | 0.3268160283 |  |
| 303 | 34 | G | G | G | 96.38 | 0.0000000000 | \* |
| 303 | 34 | G | G | T | 0.52 | 0.9380097390 |  |
| 304 | 35 | G | G | A | 0.37 | 0.8918458122 |  |
| 304 | 35 | G | G | C | 3.68 | 0.0977196286 |  |
| 304 | 35 | G | G | G | 95.22 | 0.0000000000 | \* |
| 304 | 35 | G | G | T | 0.74 | 0.9042122113 |  |
| 305 | 36 | C | C | A | 1.53 | 0.4999539648 |  |
| 305 | 36 | C | C | C | 95.11 | 0.0000000000 | \* |
| 305 | 36 | C | C | G | 1.53 | 0.5120910258 |  |
| 305 | 36 | C | C | T | 1.83 | 0.5527702795 |  |
| 306 | 37 | A | A | A | 92.91 | 0.0000000000 | \* |
| 306 | 37 | A | A | C | 3.38 | 0.1302699994 |  |
| 306 | 37 | A | A | G | 1.35 | 0.5673597341 |  |
| 306 | 37 | A | A | T | 2.36 | 0.3778968042 |  |
| 307 | 38 | C | C | A | 1.26 | 0.6031116359 |  |
| 307 | 38 | C | C | C | 93.95 | 0.0000000000 | \* |
| 307 | 38 | C | C | G | 1.76 | 0.4447275847 |  |
| 307 | 38 | C | C | T | 3.02 | 0.2161026345 |  |
| 308 | 39 | C | C | A | 2.86 | 0.1526993820 |  |
| 308 | 39 | C | C | C | 91.43 | 0.0000000000 | \* |
| 308 | 39 | C | C | G | 3.12 | 0.1798448948 |  |
| 308 | 39 | C | C | T | 2.60 | 0.3131468234 |  |
| 309 | 40 | C | C | A | 2.36 | 0.2471987378 |  |
| 309 | 40 | C | C | C | 93.18 | 0.0000000000 | \* |
| 309 | 40 | C | C | G | 1.31 | 0.5799275211 |  |
| 309 | 40 | C | C | T | 3.15 | 0.1922957410 |  |
| 310 | 41 | T | T | A | 1.76 | 0.4166313371 |  |
| 310 | 41 | T | T | C | 3.24 | 0.1490014496 |  |
| 310 | 41 | T | T | G | 2.94 | 0.2035798079 |  |
| 310 | 41 | T | T | T | 92.06 | 0.0000000000 | \* |
| 311 | 42 | G | G | A | 1.46 | 0.5258203084 |  |
| 311 | 42 | G | G | C | 1.09 | 0.7089565471 |  |
| 311 | 42 | G | G | G | 96.35 | 0.0000000000 | \* |
| 311 | 42 | G | G | T | 1.09 | 0.8121892526 |  |
| 312 | 43 | A | A | A | 94.54 | 0.0000000000 | \* |
| 312 | 43 | A | A | C | 1.66 | 0.5236122586 |  |
| 312 | 43 | A | A | G | 3.09 | 0.1835808683 |  |
| 312 | 43 | A | A | T | 0.71 | 0.9085719629 |  |
| 313 | 44 | C | C | A | 3.00 | 0.1323830899 |  |
| 313 | 44 | C | C | C | 94.28 | 0.0000000000 | \* |
| 313 | 44 | C | C | G | 0.82 | 0.7480985304 |  |
| 313 | 44 | C | C | T | 1.91 | 0.5270852841 |  |
| 314 | 45 | C | C | A | 2.22 | 0.2812044289 |  |
| 314 | 45 | C | C | C | 93.02 | 0.0000000000 | \* |
| 314 | 45 | C | C | G | 1.90 | 0.4072236226 |  |
| 314 | 45 | C | C | T | 2.86 | 0.2506306193 |  |
| 315 | 46 | G | G | A | 2.80 | 0.1611278493 |  |
| 315 | 46 | G | G | C | 1.25 | 0.6613163462 |  |
| 315 | 46 | G | G | G | 95.95 | 0.0000000000 | \* |
| 315 | 46 | G | G | T | 0.00 | 0.9578947368 |  |
| 316 | 47 | A | A | A | 95.90 | 0.0000000000 | \* |
| 316 | 47 | A | A | C | 1.30 | 0.6451159837 |  |
| 316 | 47 | A | A | G | 2.38 | 0.2999030131 |  |
| 316 | 47 | A | A | T | 0.43 | 0.9462449604 |  |
| 317 | 48 | C | C | A | 2.84 | 0.1555005797 |  |
| 317 | 48 | C | C | C | 94.32 | 0.0000000000 | \* |
| 317 | 48 | C | C | G | 0.95 | 0.7034191759 |  |
| 317 | 48 | C | C | T | 1.89 | 0.5322300438 |  |
| 318 | 49 | G | G | A | 8.00 | 0.0003245511 | \* |
| 318 | 49 | G | G | C | 2.50 | 0.2841234395 |  |
| 318 | 49 | G | G | G | 89.50 | 0.0000000000 | \* |
| 318 | 49 | G | G | T | 0.00 | 0.9578947368 |  |
| 319 | 50 | G | G | A | 1.68 | 0.4454058522 |  |
| 319 | 50 | G | G | C | 1.40 | 0.6105773534 |  |
| 319 | 50 | G | G | G | 96.92 | 0.0000000000 | \* |
| 319 | 50 | G | G | T | 0.00 | 0.9578947368 |  |
| 320 | 51 | C | C | A | 3.70 | 0.0622878646 |  |
| 320 | 51 | C | C | C | 90.95 | 0.0000000000 | \* |
| 320 | 51 | C | C | G | 3.29 | 0.1586948724 |  |
| 320 | 51 | C | C | T | 2.06 | 0.4753358140 |  |
| 321 | 52 | A | A | A | 95.35 | 0.0000000000 | \* |
| 321 | 52 | A | A | C | 3.10 | 0.1686635105 |  |
| 321 | 52 | A | A | G | 1.55 | 0.5056872745 |  |
| 321 | 52 | A | A | T | 0.00 | 0.9578947368 |  |
| 322 | 53 | A | A | A | 97.67 | 0.0000000000 | \* |
| 322 | 53 | A | A | C | 0.33 | 0.8636202291 |  |
| 322 | 53 | A | A | G | 1.00 | 0.6848792928 |  |
| 322 | 53 | A | A | T | 1.00 | 0.8402178098 |  |
| 323 | 54 | G | G | A | 1.94 | 0.3598391768 |  |
| 323 | 54 | G | G | C | 0.49 | 0.8485022309 |  |
| 323 | 54 | G | G | G | 94.66 | 0.0000000000 | \* |
| 323 | 54 | G | G | T | 2.91 | 0.2386075935 |  |
| 324 | 55 | A | A | A | 96.21 | 0.0000000000 | \* |
| 324 | 55 | A | A | C | 0.00 | 0.8720930233 |  |
| 324 | 55 | A | A | G | 0.71 | 0.7846791386 |  |
| 324 | 55 | A | A | T | 3.08 | 0.2049635771 |  |
| 325 | 56 | A | A | A | 95.30 | 0.0000000000 | \* |
| 325 | 56 | A | A | C | 0.63 | 0.8265549709 |  |
| 325 | 56 | A | A | G | 0.94 | 0.7054743689 |  |
| 325 | 56 | A | A | T | 3.13 | 0.1949578069 |  |
| 326 | 57 | G | G | A | 2.69 | 0.1807472595 |  |
| 326 | 57 | G | G | C | 2.15 | 0.3738329156 |  |
| 326 | 57 | G | G | G | 91.40 | 0.0000000000 | \* |
| 326 | 57 | G | G | T | 3.76 | 0.1058389211 |  |
| 327 | 58 | T | T | A | 2.72 | 0.1753182557 |  |
| 327 | 58 | T | T | C | 1.81 | 0.4751632024 |  |
| 327 | 58 | T | T | G | 0.91 | 0.7172925952 |  |
| 327 | 58 | T | T | T | 94.56 | 0.0000000000 | \* |
| 328 | 59 | T | T | A | 2.88 | 0.1489320880 |  |
| 328 | 59 | T | T | C | 2.02 | 0.4122648781 |  |
| 328 | 59 | T | T | G | 0.86 | 0.7317843072 |  |
| 328 | 59 | T | T | T | 94.24 | 0.0000000000 | \* |
| 329 | 60 | C | C | A | 1.85 | 0.3880057320 |  |
| 329 | 60 | C | C | C | 95.06 | 0.0000000000 | \* |
| 329 | 60 | C | C | G | 0.93 | 0.7105035344 |  |
| 329 | 60 | C | C | T | 2.16 | 0.4413306138 |  |
| 330 | 61 | G | G | A | 4.59 | 0.0226717810 |  |
| 330 | 61 | G | G | C | 3.06 | 0.1748438335 |  |
| 330 | 61 | G | G | G | 90.31 | 0.0000000000 | \* |
| 330 | 61 | G | G | T | 2.04 | 0.4810055300 |  |
| 331 | 62 | A | A | A | 95.57 | 0.0000000000 | \* |
| 331 | 62 | A | A | C | 1.97 | 0.4262783936 |  |
| 331 | 62 | A | A | G | 1.97 | 0.3906550367 |  |
| 331 | 62 | A | A | T | 0.49 | 0.9406281861 |  |
| 332 | 63 | C | C | A | 2.36 | 0.2477351671 |  |
| 332 | 63 | C | C | C | 94.10 | 0.0000000000 | \* |
| 332 | 63 | C | C | G | 0.00 | 0.9550561798 |  |
| 332 | 63 | C | C | T | 3.54 | 0.1323248559 |  |
| 333 | 64 | A | A | A | 89.64 | 0.0000000000 | \* |
| 333 | 64 | A | A | C | 5.18 | 0.0202585520 |  |
| 333 | 64 | A | A | G | 2.59 | 0.2594313888 |  |
| 333 | 64 | A | A | T | 2.59 | 0.3149044241 |  |
| 334 | 65 | G | G | A | 1.00 | 0.7054482516 |  |
| 334 | 65 | G | G | C | 3.65 | 0.0998469063 |  |
| 334 | 65 | G | G | G | 94.68 | 0.0000000000 | \* |
| 334 | 65 | G | G | T | 0.66 | 0.9171730222 |  |
| 335 | 66 | C | C | A | 0.00 | 0.9166666666 |  |
| 335 | 66 | C | C | C | 95.92 | 0.0000000000 | \* |
| 335 | 66 | C | C | G | 1.57 | 0.5006189415 |  |
| 335 | 66 | C | C | T | 2.51 | 0.3371122724 |  |
| 336 | 67 | T | T | A | 0.81 | 0.7750882850 |  |
| 336 | 67 | T | T | C | 1.61 | 0.5400045651 |  |
| 336 | 67 | T | T | G | 1.34 | 0.5696889730 |  |
| 336 | 67 | T | T | T | 96.24 | 0.0000000000 | \* |
| 337 | 68 | C | C | A | 0.28 | 0.9036723707 |  |
| 337 | 68 | C | C | C | 94.63 | 0.0000000000 | \* |
| 337 | 68 | C | C | G | 2.82 | 0.2208017111 |  |
| 337 | 68 | C | C | T | 2.26 | 0.4097577664 |  |
| 338 | 69 | C | C | A | 1.32 | 0.5811229675 |  |
| 338 | 69 | C | C | C | 93.68 | 0.0000000000 | \* |
| 338 | 69 | C | C | G | 1.84 | 0.4235229642 |  |
| 338 | 69 | C | C | T | 3.16 | 0.1908189975 |  |
| 339 | 70 | C | C | A | 3.75 | 0.0591824758 |  |
| 339 | 70 | C | C | C | 91.25 | 0.0000000000 | \* |
| 339 | 70 | C | C | G | 2.50 | 0.2758992361 |  |
| 339 | 70 | C | C | T | 2.50 | 0.3392678210 |  |
| 340 | 71 | G | G | A | 4.41 | 0.0279564344 |  |
| 340 | 71 | G | G | C | 1.84 | 0.4670844641 |  |
| 340 | 71 | G | G | G | 93.38 | 0.0000000000 | \* |
| 340 | 71 | G | G | T | 0.37 | 0.9507901418 |  |
| 341 | 72 | C | C | A | 0.99 | 0.7067058429 |  |
| 341 | 72 | C | C | C | 96.03 | 0.0000000000 | \* |
| 341 | 72 | C | C | G | 0.66 | 0.8011208366 |  |
| 341 | 72 | C | C | T | 2.32 | 0.3919615274 |  |
| 342 | 73 | G | G | A | 3.44 | 0.0834745390 |  |
| 342 | 73 | G | G | C | 2.67 | 0.2462070071 |  |
| 342 | 73 | G | G | G | 93.13 | 0.0000000000 | \* |
| 342 | 73 | G | G | T | 0.76 | 0.8985597293 |  |
| 343 | 74 | A | A | A | 94.50 | 0.0000000000 | \* |
| 343 | 74 | A | A | C | 1.57 | 0.5539812938 |  |
| 343 | 74 | A | A | G | 2.88 | 0.2125440414 |  |
| 343 | 74 | A | A | T | 1.05 | 0.8265845460 |  |
| 344 | 75 | C | C | A | 2.05 | 0.3270206762 |  |
| 344 | 75 | C | C | C | 92.67 | 0.0000000000 | \* |
| 344 | 75 | C | C | G | 2.64 | 0.2509567606 |  |
| 344 | 75 | C | C | T | 2.64 | 0.3023615910 |  |

## For use in R

If you want to work with the results in R, here is output that you can copy and paste in your terminal to get:

The base information:

```
structure(list(focal.base = c("A", "C", "G", "T"), avg.percsignal = c(94.0629608157041, 
93.5491000303773, 94.7474850240664, 94.5520330654297), avg.areasignal = c(347.694444444444, 
373.046153846154, 350.637931034483, 379.214285714286), crit.perc.area = c(5.28004289591776, 
5.81321700565192, 6.89168428631747, 5.88800839539204), mu = c(1.8595747883735, 
2.16146618620238, 2.00106828908629, 2.25512209309324), fillibens = c(0.996477000717795, 
0.985813732164172, 0.992852309275716, 0.996355526004256)), class = "data.frame", row.names = c(NA, 
-4L))
```

the data.frame that contains information on the guide region:

```
structure(list(A.area = c(296, 339, 11, 690, 14, 7, 9, 8, 14, 
15, 275, 9, 614, 13, 5, 8, 4, 0, 2, 3, 0, 5, 5, 5, 7, 264, 8, 
11, 399, 7, 249, 5, 12, 3, 1, 5, 275, 5, 11, 9, 6, 8, 398, 11, 
7, 9, 444, 9, 16, 6, 9, 123, 293, 4, 406, 304, 5, 9, 10, 6, 9, 
388, 8, 173, 3, 0, 3, 1, 5, 9, 12, 3, 9, 361, 7), C.area = c(10, 
8, 2, 8, 370, 13, 8, 6, 257, 396, 12, 6, 7, 374, 279, 5, 4, 0, 
2, 0, 0, 4, 6, 7, 350, 12, 376, 11, 9, 342, 7, 315, 6, 9, 10, 
311, 10, 373, 352, 355, 11, 6, 7, 346, 293, 4, 6, 299, 5, 5, 
221, 4, 1, 1, 0, 2, 4, 6, 7, 308, 6, 8, 319, 10, 11, 306, 6, 
335, 356, 219, 5, 290, 7, 6, 316), G.area = c(5, 6, 256, 24, 
12, 14, 705, 351, 8, 3, 5, 228, 14, 12, 11, 372, 7, 585, 16, 
11, 12, 478, 5, 420, 8, 2, 1, 2, 10, 15, 17, 8, 282, 373, 259, 
5, 4, 7, 12, 5, 10, 528, 13, 3, 6, 308, 11, 3, 179, 346, 8, 2, 
3, 195, 3, 3, 170, 3, 3, 3, 177, 8, 0, 5, 285, 5, 5, 10, 7, 6, 
254, 2, 244, 11, 9), T.area = c(3, 5, 5, 4, 9, 341, 8, 2, 4, 
9, 0, 1, 8, 13, 14, 14, 293, 7, 321, 382, 371, 3, 335, 8, 14, 
5, 9, 362, 7, 7, 0, 8, 2, 2, 2, 6, 7, 12, 10, 12, 313, 6, 3, 
7, 9, 0, 2, 6, 0, 0, 5, 0, 3, 6, 13, 10, 7, 313, 327, 7, 4, 2, 
12, 5, 2, 8, 358, 8, 12, 6, 1, 7, 2, 4, 9), Tot.area = c(314, 
358, 274, 726, 405, 375, 730, 367, 283, 423, 292, 244, 643, 412, 
309, 399, 308, 592, 341, 396, 383, 490, 351, 440, 379, 283, 394, 
386, 425, 371, 273, 336, 302, 387, 272, 327, 296, 397, 385, 381, 
340, 548, 421, 367, 315, 321, 463, 317, 200, 357, 243, 129, 300, 
206, 422, 319, 186, 331, 347, 324, 196, 406, 339, 193, 301, 319, 
372, 354, 380, 240, 272, 302, 262, 382, 341), A.perc = c(94.2675159235669, 
94.6927374301676, 4.01459854014599, 95.0413223140496, 3.45679012345679, 
1.86666666666667, 1.23287671232877, 2.17983651226158, 4.9469964664311, 
3.54609929078014, 94.1780821917808, 3.68852459016393, 95.4898911353033, 
3.15533980582524, 1.61812297734628, 2.00501253132832, 1.2987012987013, 
0, 0.586510263929619, 0.757575757575758, 0, 1.02040816326531, 
1.42450142450142, 1.13636363636364, 1.84696569920844, 93.2862190812721, 
2.03045685279188, 2.84974093264249, 93.8823529411765, 1.88679245283019, 
91.2087912087912, 1.48809523809524, 3.97350993377483, 0.775193798449612, 
0.367647058823529, 1.52905198776758, 92.9054054054054, 1.25944584382872, 
2.85714285714286, 2.36220472440945, 1.76470588235294, 1.45985401459854, 
94.5368171021378, 2.99727520435967, 2.22222222222222, 2.80373831775701, 
95.8963282937365, 2.8391167192429, 8, 1.68067226890756, 3.7037037037037, 
95.3488372093023, 97.6666666666667, 1.94174757281553, 96.2085308056872, 
95.2978056426332, 2.68817204301075, 2.7190332326284, 2.88184438040346, 
1.85185185185185, 4.59183673469388, 95.5665024630542, 2.3598820058997, 
89.6373056994819, 0.996677740863787, 0, 0.806451612903226, 0.282485875706215, 
1.31578947368421, 3.75, 4.41176470588235, 0.993377483443709, 
3.43511450381679, 94.5026178010471, 2.05278592375367), C.perc = c(3.18471337579618, 
2.23463687150838, 0.72992700729927, 1.10192837465565, 91.358024691358, 
3.46666666666667, 1.0958904109589, 1.63487738419619, 90.8127208480565, 
93.6170212765958, 4.10958904109589, 2.45901639344262, 1.08864696734059, 
90.7766990291262, 90.2912621359223, 1.2531328320802, 1.2987012987013, 
0, 0.586510263929619, 0, 0, 0.816326530612245, 1.70940170940171, 
1.59090909090909, 92.3482849604222, 4.24028268551237, 95.4314720812183, 
2.84974093264249, 2.11764705882353, 92.1832884097035, 2.56410256410256, 
93.75, 1.98675496688742, 2.32558139534884, 3.67647058823529, 
95.1070336391437, 3.37837837837838, 93.9546599496222, 91.4285714285714, 
93.1758530183727, 3.23529411764706, 1.09489051094891, 1.66270783847981, 
94.2779291553133, 93.015873015873, 1.24610591900312, 1.29589632829374, 
94.3217665615142, 2.5, 1.40056022408964, 90.9465020576132, 3.10077519379845, 
0.333333333333333, 0.485436893203884, 0, 0.626959247648903, 2.1505376344086, 
1.81268882175227, 2.01729106628242, 95.0617283950617, 3.06122448979592, 
1.97044334975369, 94.1002949852507, 5.18134715025907, 3.65448504983389, 
95.9247648902821, 1.61290322580645, 94.6327683615819, 93.6842105263158, 
91.25, 1.83823529411765, 96.0264900662252, 2.67175572519084, 
1.57068062827225, 92.6686217008798), G.perc = c(1.59235668789809, 
1.67597765363128, 93.4306569343066, 3.30578512396694, 2.96296296296296, 
3.73333333333333, 96.5753424657534, 95.6403269754768, 2.82685512367491, 
0.709219858156028, 1.71232876712329, 93.4426229508197, 2.17729393468118, 
2.9126213592233, 3.55987055016181, 93.2330827067669, 2.27272727272727, 
98.8175675675676, 4.69208211143695, 2.77777777777778, 3.1331592689295, 
97.5510204081633, 1.42450142450142, 95.4545454545455, 2.11081794195251, 
0.706713780918728, 0.253807106598985, 0.518134715025907, 2.35294117647059, 
4.04312668463612, 6.22710622710623, 2.38095238095238, 93.3774834437086, 
96.3824289405685, 95.2205882352941, 1.52905198776758, 1.35135135135135, 
1.7632241813602, 3.11688311688312, 1.31233595800525, 2.94117647058824, 
96.3503649635037, 3.08788598574822, 0.817438692098093, 1.9047619047619, 
95.9501557632399, 2.37580993520518, 0.946372239747634, 89.5, 
96.9187675070028, 3.29218106995885, 1.55038759689922, 1, 94.6601941747573, 
0.710900473933649, 0.940438871473354, 91.3978494623656, 0.906344410876133, 
0.864553314121037, 0.925925925925926, 90.3061224489796, 1.97044334975369, 
0, 2.59067357512953, 94.6843853820598, 1.56739811912226, 1.34408602150538, 
2.82485875706215, 1.84210526315789, 2.5, 93.3823529411765, 0.662251655629139, 
93.1297709923664, 2.87958115183246, 2.63929618768328), T.perc = c(0.955414012738854, 
1.39664804469274, 1.82481751824818, 0.550964187327824, 2.22222222222222, 
90.9333333333333, 1.0958904109589, 0.544959128065395, 1.41342756183746, 
2.12765957446809, 0, 0.409836065573771, 1.24416796267496, 3.15533980582524, 
4.53074433656958, 3.50877192982456, 95.1298701298701, 1.18243243243243, 
94.1348973607038, 96.4646464646465, 96.8668407310705, 0.612244897959184, 
95.4415954415954, 1.81818181818182, 3.69393139841689, 1.76678445229682, 
2.28426395939086, 93.7823834196891, 1.64705882352941, 1.88679245283019, 
0, 2.38095238095238, 0.662251655629139, 0.516795865633075, 0.735294117647059, 
1.8348623853211, 2.36486486486486, 3.02267002518892, 2.5974025974026, 
3.1496062992126, 92.0588235294118, 1.09489051094891, 0.712589073634204, 
1.90735694822888, 2.85714285714286, 0, 0.431965442764579, 1.89274447949527, 
0, 0, 2.05761316872428, 0, 1, 2.9126213592233, 3.08056872037915, 
3.13479623824451, 3.76344086021505, 94.5619335347432, 94.2363112391931, 
2.16049382716049, 2.04081632653061, 0.492610837438424, 3.53982300884956, 
2.59067357512953, 0.664451827242525, 2.50783699059561, 96.2365591397849, 
2.25988700564972, 3.15789473684211, 2.5, 0.367647058823529, 2.31788079470199, 
0.763358778625954, 1.04712041884817, 2.63929618768328), base.call = c("A", 
"A", "G", "A", "C", "T", "G", "G", "C", "C", "A", "G", "A", "C", 
"C", "G", "T", "G", "T", "T", "T", "G", "T", "G", "C", "A", "C", 
"T", "A", "C", "A", "C", "G", "G", "G", "C", "A", "C", "C", "C", 
"T", "G", "A", "C", "C", "G", "A", "C", "G", "G", "C", "A", "A", 
"G", "A", "A", "G", "T", "T", "C", "G", "A", "C", "A", "G", "C", 
"T", "C", "C", "C", "G", "C", "G", "A", "C"), index = 270:344, 
    guide.seq = c("A", "A", "G", "A", "C", "T", "G", "G", "C", 
    "C", "A", "G", "A", "C", "C", "G", "T", "G", "T", "T", "T", 
    "G", "T", "G", "C", "A", "C", "T", "A", "C", "A", "C", "G", 
    "G", "G", "C", "A", "C", "C", "C", "T", "G", "A", "C", "C", 
    "G", "A", "C", "G", "G", "C", "A", "A", "G", "A", "A", "G", 
    "T", "T", "C", "G", "A", "C", "A", "G", "C", "T", "C", "C", 
    "C", "G", "C", "G", "A", "C"), T.pval = c(0.852556204992975, 
    0.711225914540323, 0.556358738477355, 0.933933156512963, 
    0.421566485617405, 0, 0.811882128560088, 0.934681864297263, 
    0.705257970381017, 0.452043525484543, 0.957894736841395, 
    0.947967266900368, 0.764050119047257, 0.191273209434673, 
    0.0471574845430484, 0.136424663134828, 0, 0.784479687003684, 
    0, 0, 0, 0.925499760394533, 0, 0.558732824570487, 0.113522623090717, 
    0.577207517559052, 0.402219556211548, 0, 0.620658124527374, 
    0.534330584981636, 0.957894736841395, 0.373155081105999, 
    0.917545073297215, 0.938009738951182, 0.904212211326773, 
    0.552770279455712, 0.377896804185681, 0.216102634452097, 
    0.313146823444192, 0.192295740971973, 0, 0.81218925259743, 
    0.908571962880139, 0.527085284051414, 0.25063061933472, 0.957894736841395, 
    0.946244960379503, 0.532230043845399, 0.957894736841395, 
    0.957894736841395, 0.4753358139553, 0.957894736841395, 0.840217809767703, 
    0.238607593458107, 0.204963577073015, 0.194957806902338, 
    0.105838921148423, 0, 0, 0.441330613831257, 0.481005530003222, 
    0.940628186124801, 0.132324855862159, 0.314904424106433, 
    0.917173022249231, 0.337112272409677, 0, 0.409757766448233, 
    0.19081899751064, 0.339267820955862, 0.950790141767953, 0.3919615274329, 
    0.898559729262832, 0.826584545984485, 0.302361590957782), 
    C.pval = c(0.156153306746299, 0.350734130359875, 0.806038485340876, 
    0.706806017161028, 0, 0.119760385685402, 0.70865147857203, 
    0.53275784253066, 0, 0, 0.0633159671629497, 0.293775577123213, 
    0.710857903029267, 0, 0, 0.659041984705032, 0.644197905064869, 
    0.872093023255804, 0.833590945857321, 0.872093023255804, 
    0.872093023255804, 0.786157273465789, 0.508363330195024, 
    0.547277462703152, 0, 0.0553585362347417, 0, 0.211154588736151, 
    0.383115421772189, 0, 0.269494901881188, 0, 0.421370980608175, 
    0.326816028286111, 0.097719628568446, 0, 0.130269999407155, 
    0, 0, 0, 0.149001449590974, 0.708956547056127, 0.523612258615625, 
    0, 0, 0.661316346177926, 0.645115983692121, 0, 0.284123439506489, 
    0.610577353401788, 0, 0.168663510522761, 0.863620229116629, 
    0.848502230890182, 0.872093023255804, 0.826554970927241, 
    0.373832915610974, 0.475163202371624, 0.412264878118036, 
    0, 0.174843833519092, 0.426278393593546, 0, 0.02025855202174, 
    0.0998469062598555, 0, 0.540004565078907, 0, 0, 0, 0.467084464131018, 
    0, 0.246207007099985, 0.553981293774406, 0), G.pval = c(0.493242918284574, 
    0.469063204326367, 0, 0.157152621230744, 0.200491139044519, 
    0.115182231146805, 0, 0, 0.220495514239316, 0.785250597858201, 
    0.458811963317454, 0, 0.341909324238826, 0.207692621074601, 
    0.130767433335844, 0, 0.321144721727156, 0, 0.0561363475753208, 
    0.228133044385347, 0.177778237488368, 0, 0.54420308454919, 
    0, 0.357011288321262, 0.786102319796019, 0.920866195589212, 
    0.84807519448602, 0.304510723780567, 0.0915859681855192, 
    0.0169850721409399, 0.298875024358289, 0, 0, 0, 0.51209102583959, 
    0.567359734124093, 0.44472758465821, 0.17984489476815, 0.579927521082284, 
    0.203579807850262, 0, 0.183580868335577, 0.748098530402003, 
    0.407223622601506, 0, 0.299903013068542, 0.703419175880823, 
    0, 0, 0.158694872381832, 0.505687274468799, 0.684879292847535, 
    0, 0.784679138586381, 0.705474368946274, 0, 0.717292595230358, 
    0.731784307198506, 0.710503534426618, 0, 0.390655036701591, 
    0.95505617977507, 0.259431388767728, 0, 0.500618941451268, 
    0.569688972963598, 0.220801711144443, 0.423522964244181, 
    0.275899236090759, 0, 0.801120836562354, 0, 0.212544041371742, 
    0.250956760569282), A.pval = c(0, 0, 0.0440281033030869, 
    0, 0.0815464954966481, 0.383267236026307, 0.613517542853601, 
    0.292183200714763, 0.0149095224170195, 0.0740246848770942, 
    0, 0.0633384473228351, 0, 0.112361694575969, 0.467522727860475, 
    0.340870507109022, 0.587777361922557, 0.916666666593764, 
    0.843788530075554, 0.791709346708492, 0.916666666593764, 
    0.696369963412123, 0.539230617229371, 0.651352765179251, 
    0.38957682153656, 0, 0.333443066379944, 0.153844271619909, 
    0, 0.376890722990766, 0, 0.515199070655663, 0.046115017976766, 
    0.785793473028986, 0.891845812173721, 0.499953964754827, 
    0, 0.60311163592332, 0.152699382044004, 0.24719873781865, 
    0.416631337081157, 0.525820308430138, 0, 0.132383089912374, 
    0.281204428867072, 0.161127849276983, 0, 0.155500579701387, 
    0.000324551092817149, 0.44540585223443, 0.0622878645616709, 
    0, 0, 0.359839176806161, 0, 0, 0.180747259512954, 0.175318255728873, 
    0.148932087958926, 0.388005732037559, 0.0226717809863324, 
    0, 0.247735167140128, 0, 0.705448251559031, 0.916666666593764, 
    0.775088284963125, 0.90367237067812, 0.581122967463524, 0.0591824757793131, 
    0.0279564344371126, 0.706705842927691, 0.0834745389989291, 
    0, 0.327020676188942), guide.position = 1:75), row.names = 270:344, class = "data.frame")
```

*Report generated using EditR v1.0.10*
