# Supplementary material for: Mechanistic and genetic basis of single-strand templated repair at Cas12a-induced DNA breaks in Chlamydomonas reinhardtii
Source: Nat Commun. 2021 Nov 19;12:6751. doi: 10.1038/s41467-021-27004-1 (PMC8604939; doi:10.1038/s41467-021-27004-1)
Supplement: Supplementary file 22 — Source Data [file 41467_2021_27004_MOESM22_ESM.zip › Source Data/EditR analysis/EditR outputs/wt/rep3_wt.html]

EditR v1.0.10 report


# EditR v1.0.10 report

- Data QA
  - Filtering data
  - Percent noise peak area
  - Base information
- Predicted editing
  - Editing bar plot
  - Editing table plot
  - Table of editing results
- For use in R

## Data QA

### Filtering data

What the data looked like prefiltering:

and the post filtering signal / noise plot:

### Percent noise peak area

### Base information

Here’s information about the signal of each base, the critical percent value where any higher value would be called as significant, and Filliben’s correlation for how well the noise was modelled by the zero adjusted gamma distribution.

| Base | Average percent signal | Average peak area | Critical percent value | model mu | Fillibens correlation |
| --- | --- | --- | --- | --- | --- |
| A | 93.48678 | 295.1667 | 5.532756 | 1.780406 | 0.9972349 |
| C | 93.35039 | 343.9077 | 5.764425 | 2.143189 | 0.9926205 |
| G | 94.77679 | 327.4828 | 7.861618 | 2.294365 | 0.9935810 |
| T | 94.58786 | 361.6905 | 6.528992 | 2.441322 | 0.9969933 |

## Predicted editing

### Editing bar plot

### Editing table plot

### Table of editing results


Here’s the entire guide region

| Sanger position | Guide position | Guide sequence | Sanger base call | Focal base | Focal base peak area | p value |  |
| --- | --- | --- | --- | --- | --- | --- | --- |
| 268 | 1 | A | A | A | 94.40 | 0.000000000 | \* |
| 268 | 1 | A | A | C | 2.61 | 0.250900064 |  |
| 268 | 1 | A | A | G | 1.49 | 0.592620040 |  |
| 268 | 1 | A | A | T | 1.49 | 0.665373353 |  |
| 269 | 2 | A | A | A | 95.45 | 0.000000000 | \* |
| 269 | 2 | A | A | C | 1.82 | 0.461823720 |  |
| 269 | 2 | A | A | G | 1.52 | 0.586111437 |  |
| 269 | 2 | A | A | T | 1.21 | 0.747318717 |  |
| 270 | 3 | G | G | A | 3.31 | 0.098488459 |  |
| 270 | 3 | G | G | C | 0.37 | 0.849015642 |  |
| 270 | 3 | G | G | G | 94.12 | 0.000000000 | \* |
| 270 | 3 | G | G | T | 2.21 | 0.449927180 |  |
| 271 | 4 | A | A | A | 93.65 | 0.000000000 | \* |
| 271 | 4 | A | A | C | 1.06 | 0.706717239 |  |
| 271 | 4 | A | A | G | 4.99 | 0.072314465 |  |
| 271 | 4 | A | A | T | 0.30 | 0.901096414 |  |
| 272 | 5 | C | C | A | 3.47 | 0.084466404 |  |
| 272 | 5 | C | C | C | 91.20 | 0.000000000 | \* |
| 272 | 5 | C | C | G | 3.73 | 0.163838786 |  |
| 272 | 5 | C | C | T | 1.60 | 0.632464111 |  |
| 273 | 6 | T | T | A | 1.47 | 0.485760097 |  |
| 273 | 6 | T | T | C | 3.54 | 0.107345666 |  |
| 273 | 6 | T | T | G | 4.13 | 0.127250139 |  |
| 273 | 6 | T | T | T | 90.86 | 0.000000000 | \* |
| 274 | 7 | G | G | A | 0.70 | 0.782145953 |  |
| 274 | 7 | G | G | C | 1.26 | 0.643076425 |  |
| 274 | 7 | G | G | G | 97.05 | 0.000000000 | \* |
| 274 | 7 | G | G | T | 0.98 | 0.806384997 |  |
| 275 | 8 | G | G | A | 1.96 | 0.332936545 |  |
| 275 | 8 | G | G | C | 1.40 | 0.597775379 |  |
| 275 | 8 | G | G | G | 95.80 | 0.000000000 | \* |
| 275 | 8 | G | G | T | 0.84 | 0.837010847 |  |
| 276 | 9 | C | C | A | 4.69 | 0.024496074 |  |
| 276 | 9 | C | C | C | 90.62 | 0.000000000 | \* |
| 276 | 9 | C | C | G | 2.34 | 0.377746636 |  |
| 276 | 9 | C | C | T | 2.34 | 0.411749754 |  |
| 277 | 10 | C | C | A | 3.63 | 0.072143175 |  |
| 277 | 10 | C | C | C | 94.04 | 0.000000000 | \* |
| 277 | 10 | C | C | G | 0.52 | 0.880945769 |  |
| 277 | 10 | C | C | T | 1.81 | 0.566587793 |  |
| 278 | 11 | A | A | A | 90.27 | 0.000000000 | \* |
| 278 | 11 | A | A | C | 4.67 | 0.033642904 |  |
| 278 | 11 | A | A | G | 1.95 | 0.470081731 |  |
| 278 | 11 | A | A | T | 3.11 | 0.236639569 |  |
| 279 | 12 | G | G | A | 2.80 | 0.159452188 |  |
| 279 | 12 | G | G | C | 1.60 | 0.531942035 |  |
| 279 | 12 | G | G | G | 94.40 | 0.000000000 | \* |
| 279 | 12 | G | G | T | 1.20 | 0.750656530 |  |
| 280 | 13 | A | A | A | 94.54 | 0.000000000 | \* |
| 280 | 13 | A | A | C | 1.23 | 0.652712471 |  |
| 280 | 13 | A | A | G | 2.46 | 0.352657830 |  |
| 280 | 13 | A | A | T | 1.76 | 0.582882301 |  |
| 281 | 14 | C | C | A | 2.37 | 0.234077171 |  |
| 281 | 14 | C | C | C | 91.03 | 0.000000000 | \* |
| 281 | 14 | C | C | G | 3.17 | 0.232918302 |  |
| 281 | 14 | C | C | T | 3.43 | 0.183801357 |  |
| 282 | 15 | C | C | A | 1.16 | 0.604109510 |  |
| 282 | 15 | C | C | C | 88.42 | 0.000000000 | \* |
| 282 | 15 | C | C | G | 5.02 | 0.071032297 |  |
| 282 | 15 | C | C | T | 5.41 | 0.030829897 |  |
| 283 | 16 | G | G | A | 2.04 | 0.311605214 |  |
| 283 | 16 | G | G | C | 1.02 | 0.718141451 |  |
| 283 | 16 | G | G | G | 93.37 | 0.000000000 | \* |
| 283 | 16 | G | G | T | 3.57 | 0.163609916 |  |
| 284 | 17 | T | T | A | 0.00 | 0.935185185 |  |
| 284 | 17 | T | T | C | 1.74 | 0.485874716 |  |
| 284 | 17 | T | T | G | 2.44 | 0.357883327 |  |
| 284 | 17 | T | T | T | 95.82 | 0.000000000 | \* |
| 285 | 18 | G | G | A | 0.84 | 0.730358836 |  |
| 285 | 18 | G | G | C | 0.00 | 0.860465116 |  |
| 285 | 18 | G | G | G | 97.82 | 0.000000000 | \* |
| 285 | 18 | G | G | T | 1.34 | 0.710910895 |  |
| 286 | 19 | T | T | A | 0.31 | 0.900851498 |  |
| 286 | 19 | T | T | C | 1.57 | 0.541030122 |  |
| 286 | 19 | T | T | G | 4.40 | 0.106651156 |  |
| 286 | 19 | T | T | T | 93.71 | 0.000000000 | \* |
| 287 | 20 | T | T | A | 0.52 | 0.843768595 |  |
| 287 | 20 | T | T | C | 1.57 | 0.541571702 |  |
| 287 | 20 | T | T | G | 2.88 | 0.276801291 |  |
| 287 | 20 | T | T | T | 95.03 | 0.000000000 | \* |
| 288 | 21 | T | T | A | 0.00 | 0.935185185 |  |
| 288 | 21 | T | T | C | 0.00 | 0.860465116 |  |
| 288 | 21 | T | T | G | 3.01 | 0.255448888 |  |
| 288 | 21 | T | T | T | 96.99 | 0.000000000 | \* |
| 289 | 22 | G | G | A | 0.62 | 0.809654058 |  |
| 289 | 22 | G | G | C | 1.46 | 0.578673222 |  |
| 289 | 22 | G | G | G | 97.29 | 0.000000000 | \* |
| 289 | 22 | G | G | T | 0.62 | 0.873162055 |  |
| 290 | 23 | T | T | A | 1.22 | 0.578961144 |  |
| 290 | 23 | T | T | C | 2.14 | 0.366354989 |  |
| 290 | 23 | T | T | G | 1.22 | 0.672501415 |  |
| 290 | 23 | T | T | T | 95.41 | 0.000000000 | \* |
| 291 | 24 | G | G | A | 0.95 | 0.687043951 |  |
| 291 | 24 | G | G | C | 2.37 | 0.306439664 |  |
| 291 | 24 | G | G | G | 95.02 | 0.000000000 | \* |
| 291 | 24 | G | G | T | 1.66 | 0.614330477 |  |
| 292 | 25 | C | C | A | 1.77 | 0.388248256 |  |
| 292 | 25 | C | C | C | 92.92 | 0.000000000 | \* |
| 292 | 25 | C | C | G | 2.06 | 0.440825780 |  |
| 292 | 25 | C | C | T | 3.24 | 0.213341910 |  |
| 293 | 26 | A | A | A | 95.10 | 0.000000000 | \* |
| 293 | 26 | A | A | C | 4.08 | 0.062393396 |  |
| 293 | 26 | A | A | G | 0.82 | 0.796492217 |  |
| 293 | 26 | A | A | T | 0.00 | 0.905263158 |  |
| 294 | 27 | C | C | A | 1.98 | 0.328417593 |  |
| 294 | 27 | C | C | C | 95.48 | 0.000000000 | \* |
| 294 | 27 | C | C | G | 0.56 | 0.868550775 |  |
| 294 | 27 | C | C | T | 1.98 | 0.516720185 |  |
| 295 | 28 | T | T | A | 2.56 | 0.199066922 |  |
| 295 | 28 | T | T | C | 3.12 | 0.159171076 |  |
| 295 | 28 | T | T | G | 0.57 | 0.867685878 |  |
| 295 | 28 | T | T | T | 93.75 | 0.000000000 | \* |
| 296 | 29 | A | A | A | 92.67 | 0.000000000 | \* |
| 296 | 29 | A | A | C | 2.88 | 0.198854062 |  |
| 296 | 29 | A | A | G | 2.62 | 0.322885767 |  |
| 296 | 29 | A | A | T | 1.83 | 0.560756754 |  |
| 297 | 30 | C | C | A | 2.13 | 0.289685524 |  |
| 297 | 30 | C | C | C | 91.79 | 0.000000000 | \* |
| 297 | 30 | C | C | G | 3.95 | 0.142666967 |  |
| 297 | 30 | C | C | T | 2.13 | 0.472348000 |  |
| 298 | 31 | A | A | A | 88.84 | 0.000000000 | \* |
| 298 | 31 | A | A | C | 2.58 | 0.258819710 |  |
| 298 | 31 | A | A | G | 6.44 | 0.027096659 |  |
| 298 | 31 | A | A | T | 2.15 | 0.467067796 |  |
| 299 | 32 | C | C | A | 1.37 | 0.525430781 |  |
| 299 | 32 | C | C | C | 92.83 | 0.000000000 | \* |
| 299 | 32 | C | C | G | 3.41 | 0.200170669 |  |
| 299 | 32 | C | C | T | 2.39 | 0.399603651 |  |
| 300 | 33 | G | G | A | 4.78 | 0.022280360 |  |
| 300 | 33 | G | G | C | 2.05 | 0.392620297 |  |
| 300 | 33 | G | G | G | 92.83 | 0.000000000 | \* |
| 300 | 33 | G | G | T | 0.34 | 0.899330988 |  |
| 301 | 34 | G | G | A | 0.57 | 0.828622247 |  |
| 301 | 34 | G | G | C | 2.85 | 0.204322386 |  |
| 301 | 34 | G | G | G | 96.30 | 0.000000000 | \* |
| 301 | 34 | G | G | T | 0.28 | 0.901776403 |  |
| 302 | 35 | G | G | A | 0.41 | 0.878353558 |  |
| 302 | 35 | G | G | C | 4.07 | 0.063465011 |  |
| 302 | 35 | G | G | G | 94.72 | 0.000000000 | \* |
| 302 | 35 | G | G | T | 0.81 | 0.842336001 |  |
| 303 | 36 | C | C | A | 1.77 | 0.389206494 |  |
| 303 | 36 | C | C | C | 95.41 | 0.000000000 | \* |
| 303 | 36 | C | C | G | 1.41 | 0.615660181 |  |
| 303 | 36 | C | C | T | 1.41 | 0.689221203 |  |
| 304 | 37 | A | A | A | 92.59 | 0.000000000 | \* |
| 304 | 37 | A | A | C | 4.12 | 0.060274335 |  |
| 304 | 37 | A | A | G | 0.82 | 0.794479208 |  |
| 304 | 37 | A | A | T | 2.47 | 0.378674264 |  |
| 305 | 38 | C | C | A | 1.15 | 0.608857631 |  |
| 305 | 38 | C | C | C | 94.27 | 0.000000000 | \* |
| 305 | 38 | C | C | G | 1.72 | 0.529141028 |  |
| 305 | 38 | C | C | T | 2.87 | 0.285614768 |  |
| 306 | 39 | C | C | A | 3.04 | 0.127441568 |  |
| 306 | 39 | C | C | C | 93.01 | 0.000000000 | \* |
| 306 | 39 | C | C | G | 3.95 | 0.142666967 |  |
| 306 | 39 | C | C | T | 0.00 | 0.905263158 |  |
| 307 | 40 | C | C | A | 2.47 | 0.215316035 |  |
| 307 | 40 | C | C | C | 94.14 | 0.000000000 | \* |
| 307 | 40 | C | C | G | 0.93 | 0.763352209 |  |
| 307 | 40 | C | C | T | 2.47 | 0.378674264 |  |
| 308 | 41 | T | T | A | 1.39 | 0.514955802 |  |
| 308 | 41 | T | T | C | 3.83 | 0.080339898 |  |
| 308 | 41 | T | T | G | 3.48 | 0.191501312 |  |
| 308 | 41 | T | T | T | 91.29 | 0.000000000 | \* |
| 309 | 42 | G | G | A | 1.35 | 0.531511981 |  |
| 309 | 42 | G | G | C | 0.96 | 0.734505900 |  |
| 309 | 42 | G | G | G | 96.72 | 0.000000000 | \* |
| 309 | 42 | G | G | T | 0.96 | 0.810637693 |  |
| 310 | 43 | A | A | A | 94.81 | 0.000000000 | \* |
| 310 | 43 | A | A | C | 1.64 | 0.519077242 |  |
| 310 | 43 | A | A | G | 3.01 | 0.256717488 |  |
| 310 | 43 | A | A | T | 0.55 | 0.882867146 |  |
| 311 | 44 | C | C | A | 3.50 | 0.081497547 |  |
| 311 | 44 | C | C | C | 95.22 | 0.000000000 | \* |
| 311 | 44 | C | C | G | 0.00 | 0.966292135 |  |
| 311 | 44 | C | C | T | 1.27 | 0.729991681 |  |
| 312 | 45 | C | C | A | 2.71 | 0.172717197 |  |
| 312 | 45 | C | C | C | 92.25 | 0.000000000 | \* |
| 312 | 45 | C | C | G | 2.33 | 0.381631582 |  |
| 312 | 45 | C | C | T | 2.71 | 0.319227597 |  |
| 313 | 46 | G | G | A | 2.66 | 0.181676466 |  |
| 313 | 46 | G | G | C | 1.33 | 0.621362129 |  |
| 313 | 46 | G | G | G | 96.01 | 0.000000000 | \* |
| 313 | 46 | G | G | T | 0.00 | 0.905263158 |  |
| 314 | 47 | A | A | A | 95.20 | 0.000000000 | \* |
| 314 | 47 | A | A | C | 1.26 | 0.642961776 |  |
| 314 | 47 | A | A | G | 2.27 | 0.393111004 |  |
| 314 | 47 | A | A | T | 1.26 | 0.733187965 |  |
| 315 | 48 | C | C | A | 2.57 | 0.196093123 |  |
| 315 | 48 | C | C | C | 94.85 | 0.000000000 | \* |
| 315 | 48 | C | C | G | 0.74 | 0.820499232 |  |
| 315 | 48 | C | C | T | 1.84 | 0.558985745 |  |
| 316 | 49 | G | G | A | 7.27 | 0.001480673 | \* |
| 316 | 49 | G | G | C | 2.42 | 0.293217903 |  |
| 316 | 49 | G | G | G | 90.30 | 0.000000000 | \* |
| 316 | 49 | G | G | T | 0.00 | 0.905263158 |  |
| 317 | 50 | G | G | A | 1.92 | 0.345089481 |  |
| 317 | 50 | G | G | C | 1.28 | 0.637990892 |  |
| 317 | 50 | G | G | G | 96.81 | 0.000000000 | \* |
| 317 | 50 | G | G | T | 0.00 | 0.905263158 |  |
| 318 | 51 | C | C | A | 3.81 | 0.060160421 |  |
| 318 | 51 | C | C | C | 92.38 | 0.000000000 | \* |
| 318 | 51 | C | C | G | 2.38 | 0.369888969 |  |
| 318 | 51 | C | C | T | 1.43 | 0.684689196 |  |
| 319 | 52 | A | A | A | 93.62 | 0.000000000 | \* |
| 319 | 52 | A | A | C | 3.19 | 0.149644690 |  |
| 319 | 52 | A | A | G | 1.06 | 0.721155284 |  |
| 319 | 52 | A | A | T | 2.13 | 0.472348000 |  |
| 320 | 53 | A | A | A | 96.84 | 0.000000000 | \* |
| 320 | 53 | A | A | C | 0.40 | 0.846512532 |  |
| 320 | 53 | A | A | G | 1.58 | 0.567362941 |  |
| 320 | 53 | A | A | T | 1.19 | 0.754546707 |  |
| 321 | 54 | G | G | A | 1.50 | 0.476935733 |  |
| 321 | 54 | G | G | C | 0.00 | 0.860465116 |  |
| 321 | 54 | G | G | G | 95.50 | 0.000000000 | \* |
| 321 | 54 | G | G | T | 3.00 | 0.258098566 |  |
| 322 | 55 | A | A | A | 95.59 | 0.000000000 | \* |
| 322 | 55 | A | A | C | 0.00 | 0.860465116 |  |
| 322 | 55 | A | A | G | 0.88 | 0.776595381 |  |
| 322 | 55 | A | A | T | 3.53 | 0.169407404 |  |
| 323 | 56 | A | A | A | 95.49 | 0.000000000 | \* |
| 323 | 56 | A | A | C | 0.38 | 0.848293923 |  |
| 323 | 56 | A | A | G | 0.75 | 0.815631162 |  |
| 323 | 56 | A | A | T | 3.38 | 0.190899142 |  |
| 324 | 57 | G | G | A | 2.42 | 0.224066213 |  |
| 324 | 57 | G | G | C | 1.82 | 0.461823720 |  |
| 324 | 57 | G | G | G | 92.12 | 0.000000000 | \* |
| 324 | 57 | G | G | T | 3.64 | 0.154980363 |  |
| 325 | 58 | T | T | A | 2.08 | 0.300715404 |  |
| 325 | 58 | T | T | C | 2.08 | 0.382440528 |  |
| 325 | 58 | T | T | G | 0.69 | 0.832369435 |  |
| 325 | 58 | T | T | T | 95.14 | 0.000000000 | \* |
| 326 | 59 | T | T | A | 2.96 | 0.137293647 |  |
| 326 | 59 | T | T | C | 1.64 | 0.517319889 |  |
| 326 | 59 | T | T | G | 0.66 | 0.842826347 |  |
| 326 | 59 | T | T | T | 94.74 | 0.000000000 | \* |
| 327 | 60 | C | C | A | 1.47 | 0.487296210 |  |
| 327 | 60 | C | C | C | 95.22 | 0.000000000 | \* |
| 327 | 60 | C | C | G | 1.47 | 0.598971113 |  |
| 327 | 60 | C | C | T | 1.84 | 0.558985745 |  |
| 328 | 61 | G | G | A | 4.44 | 0.031530942 |  |
| 328 | 61 | G | G | C | 2.78 | 0.217529901 |  |
| 328 | 61 | G | G | G | 92.22 | 0.000000000 | \* |
| 328 | 61 | G | G | T | 0.56 | 0.881840232 |  |
| 329 | 62 | A | A | A | 95.83 | 0.000000000 | \* |
| 329 | 62 | A | A | C | 1.79 | 0.472036515 |  |
| 329 | 62 | A | A | G | 1.79 | 0.511310235 |  |
| 329 | 62 | A | A | T | 0.60 | 0.877064646 |  |
| 330 | 63 | C | C | A | 2.39 | 0.231129254 |  |
| 330 | 63 | C | C | C | 93.86 | 0.000000000 | \* |
| 330 | 63 | C | C | G | 0.34 | 0.922959011 |  |
| 330 | 63 | C | C | T | 3.41 | 0.186380485 |  |
| 331 | 64 | A | A | A | 87.33 | 0.000000000 | \* |
| 331 | 64 | A | A | C | 5.33 | 0.016256442 |  |
| 331 | 64 | A | A | G | 3.33 | 0.210260838 |  |
| 331 | 64 | A | A | T | 4.00 | 0.113524245 |  |
| 332 | 65 | G | G | A | 0.72 | 0.775410773 |  |
| 332 | 65 | G | G | C | 3.24 | 0.143352685 |  |
| 332 | 65 | G | G | G | 95.32 | 0.000000000 | \* |
| 332 | 65 | G | G | T | 0.72 | 0.858984945 |  |
| 333 | 66 | C | C | A | 0.00 | 0.935185185 |  |
| 333 | 66 | C | C | C | 96.30 | 0.000000000 | \* |
| 333 | 66 | C | C | G | 1.48 | 0.595814988 |  |
| 333 | 66 | C | C | T | 2.22 | 0.445310063 |  |
| 334 | 67 | T | T | A | 0.62 | 0.813021001 |  |
| 334 | 67 | T | T | C | 1.54 | 0.552186756 |  |
| 334 | 67 | T | T | G | 1.85 | 0.495445822 |  |
| 334 | 67 | T | T | T | 96.00 | 0.000000000 | \* |
| 335 | 68 | C | C | A | 0.68 | 0.791522514 |  |
| 335 | 68 | C | C | C | 93.24 | 0.000000000 | \* |
| 335 | 68 | C | C | G | 4.05 | 0.133595549 |  |
| 335 | 68 | C | C | T | 2.03 | 0.501895597 |  |
| 336 | 69 | C | C | A | 0.95 | 0.685260193 |  |
| 336 | 69 | C | C | C | 94.29 | 0.000000000 | \* |
| 336 | 69 | C | C | G | 1.90 | 0.480376783 |  |
| 336 | 69 | C | C | T | 2.86 | 0.287355033 |  |
| 337 | 70 | C | C | A | 3.39 | 0.091043618 |  |
| 337 | 70 | C | C | C | 90.40 | 0.000000000 | \* |
| 337 | 70 | C | C | G | 3.39 | 0.203056801 |  |
| 337 | 70 | C | C | T | 2.82 | 0.294293506 |  |
| 338 | 71 | G | G | A | 3.73 | 0.064842266 |  |
| 338 | 71 | G | G | C | 1.66 | 0.512435295 |  |
| 338 | 71 | G | G | G | 94.61 | 0.000000000 | \* |
| 338 | 71 | G | G | T | 0.00 | 0.905263158 |  |
| 339 | 72 | C | C | A | 0.80 | 0.746095951 |  |
| 339 | 72 | C | C | C | 96.02 | 0.000000000 | \* |
| 339 | 72 | C | C | G | 0.80 | 0.802321219 |  |
| 339 | 72 | C | C | T | 2.39 | 0.399242548 |  |
| 340 | 73 | G | G | A | 3.04 | 0.126964357 |  |
| 340 | 73 | G | G | C | 3.04 | 0.171545565 |  |
| 340 | 73 | G | G | G | 93.04 | 0.000000000 | \* |
| 340 | 73 | G | G | T | 0.87 | 0.831093893 |  |
| 341 | 74 | A | A | A | 94.43 | 0.000000000 | \* |
| 341 | 74 | A | A | C | 1.64 | 0.519077242 |  |
| 341 | 74 | A | A | G | 2.95 | 0.265275588 |  |
| 341 | 74 | A | A | T | 0.98 | 0.805955101 |  |
| 342 | 75 | C | C | A | 1.72 | 0.404303988 |  |
| 342 | 75 | C | C | C | 92.78 | 0.000000000 | \* |
| 342 | 75 | C | C | G | 2.75 | 0.299016868 |  |
| 342 | 75 | C | C | T | 2.75 | 0.311040849 |  |

## For use in R

If you want to work with the results in R, here is output that you can copy and paste in your terminal to get:

The base information:

```
structure(list(focal.base = c("A", "C", "G", "T"), avg.percsignal = c(93.4867756953118, 
93.3503898080757, 94.7767944825344, 94.5878626549292), avg.areasignal = c(295.166666666667, 
343.907692307692, 327.48275862069, 361.690476190476), crit.perc.area = c(5.53275552483414, 
5.76442498688482, 7.86161778543871, 6.52899189990442), mu = c(1.78040605532666, 
2.14318902876097, 2.29436520991396, 2.44132208080103), fillibens = c(0.997234892322934, 
0.992620486458242, 0.993580960598204, 0.996993335724692)), class = "data.frame", row.names = c(NA, 
-4L))
```

the data.frame that contains information on the guide region:

```
structure(list(A.area = c(253, 315, 9, 619, 13, 5, 5, 7, 12, 
14, 232, 7, 537, 9, 3, 8, 0, 5, 1, 2, 0, 3, 4, 4, 6, 233, 7, 
9, 354, 7, 207, 4, 14, 2, 1, 5, 225, 4, 10, 8, 4, 7, 347, 11, 
7, 8, 377, 7, 12, 6, 8, 88, 245, 3, 325, 254, 4, 6, 9, 4, 8, 
322, 7, 131, 2, 0, 2, 2, 3, 6, 9, 2, 7, 288, 5), C.area = c(7, 
6, 1, 7, 342, 12, 9, 5, 232, 363, 12, 4, 7, 345, 229, 4, 5, 0, 
5, 6, 0, 7, 7, 10, 315, 10, 338, 11, 11, 302, 6, 272, 6, 10, 
10, 270, 10, 329, 306, 305, 11, 5, 6, 299, 238, 4, 5, 258, 4, 
4, 194, 3, 1, 0, 0, 1, 3, 6, 5, 259, 5, 6, 275, 8, 9, 260, 5, 
276, 297, 160, 4, 241, 7, 5, 270), G.area = c(4, 5, 256, 33, 
14, 14, 692, 342, 6, 2, 5, 236, 14, 12, 13, 366, 7, 584, 14, 
11, 11, 467, 4, 401, 7, 2, 2, 2, 10, 13, 15, 10, 272, 338, 233, 
4, 2, 6, 13, 3, 10, 502, 11, 0, 6, 289, 9, 2, 149, 303, 5, 1, 
4, 191, 3, 2, 152, 2, 2, 4, 166, 6, 1, 5, 265, 4, 6, 12, 6, 6, 
228, 2, 214, 9, 8), T.area = c(4, 4, 6, 2, 6, 308, 7, 3, 6, 7, 
8, 3, 10, 13, 14, 14, 275, 8, 298, 363, 354, 3, 312, 7, 11, 0, 
7, 330, 7, 7, 5, 7, 1, 1, 2, 4, 6, 10, 0, 8, 262, 5, 2, 4, 7, 
0, 5, 5, 0, 0, 3, 2, 3, 6, 12, 9, 6, 274, 288, 5, 1, 2, 10, 6, 
2, 6, 312, 6, 9, 5, 0, 6, 2, 3, 8), Tot.area = c(268, 330, 272, 
661, 375, 339, 713, 357, 256, 386, 257, 250, 568, 379, 259, 392, 
287, 597, 318, 382, 365, 480, 327, 422, 339, 245, 354, 352, 382, 
329, 233, 293, 293, 351, 246, 283, 243, 349, 329, 324, 287, 519, 
366, 314, 258, 301, 396, 272, 165, 313, 210, 94, 253, 200, 340, 
266, 165, 288, 304, 272, 180, 336, 293, 150, 278, 270, 325, 296, 
315, 177, 241, 251, 230, 305, 291), A.perc = c(94.4029850746269, 
95.4545454545455, 3.30882352941176, 93.6459909228442, 3.46666666666667, 
1.47492625368732, 0.701262272089762, 1.96078431372549, 4.6875, 
3.62694300518135, 90.272373540856, 2.8, 94.5422535211268, 2.37467018469657, 
1.15830115830116, 2.04081632653061, 0, 0.837520938023451, 0.314465408805031, 
0.523560209424084, 0, 0.625, 1.22324159021407, 0.947867298578199, 
1.76991150442478, 95.1020408163265, 1.9774011299435, 2.55681818181818, 
92.6701570680628, 2.12765957446809, 88.8412017167382, 1.36518771331058, 
4.77815699658703, 0.56980056980057, 0.40650406504065, 1.76678445229682, 
92.5925925925926, 1.14613180515759, 3.03951367781155, 2.46913580246914, 
1.39372822299652, 1.34874759152216, 94.8087431693989, 3.5031847133758, 
2.71317829457364, 2.6578073089701, 95.2020202020202, 2.57352941176471, 
7.27272727272727, 1.91693290734824, 3.80952380952381, 93.6170212765958, 
96.8379446640316, 1.5, 95.5882352941177, 95.4887218045113, 2.42424242424242, 
2.08333333333333, 2.96052631578947, 1.47058823529412, 4.44444444444444, 
95.8333333333333, 2.38907849829352, 87.3333333333333, 0.719424460431655, 
0, 0.615384615384615, 0.675675675675676, 0.952380952380952, 3.38983050847458, 
3.7344398340249, 0.796812749003984, 3.04347826086957, 94.4262295081967, 
1.71821305841924), C.perc = c(2.61194029850746, 1.81818181818182, 
0.367647058823529, 1.0590015128593, 91.2, 3.53982300884956, 1.26227208976157, 
1.40056022408964, 90.625, 94.0414507772021, 4.66926070038911, 
1.6, 1.23239436619718, 91.0290237467019, 88.4169884169884, 1.02040816326531, 
1.74216027874564, 0, 1.57232704402516, 1.57068062827225, 0, 1.45833333333333, 
2.14067278287462, 2.3696682464455, 92.9203539823009, 4.08163265306122, 
95.4802259887006, 3.125, 2.87958115183246, 91.7933130699088, 
2.57510729613734, 92.8327645051195, 2.04778156996587, 2.84900284900285, 
4.0650406504065, 95.4063604240283, 4.11522633744856, 94.269340974212, 
93.0091185410334, 94.1358024691358, 3.83275261324042, 0.963391136801541, 
1.63934426229508, 95.2229299363057, 92.2480620155039, 1.32890365448505, 
1.26262626262626, 94.8529411764706, 2.42424242424242, 1.2779552715655, 
92.3809523809524, 3.19148936170213, 0.395256916996047, 0, 0, 
0.37593984962406, 1.81818181818182, 2.08333333333333, 1.64473684210526, 
95.2205882352941, 2.77777777777778, 1.78571428571429, 93.8566552901024, 
5.33333333333333, 3.23741007194245, 96.2962962962963, 1.53846153846154, 
93.2432432432432, 94.2857142857143, 90.3954802259887, 1.6597510373444, 
96.0159362549801, 3.04347826086957, 1.63934426229508, 92.7835051546392
), G.perc = c(1.49253731343284, 1.51515151515152, 94.1176470588235, 
4.99243570347958, 3.73333333333333, 4.12979351032448, 97.054698457223, 
95.7983193277311, 2.34375, 0.518134715025907, 1.94552529182879, 
94.4, 2.46478873239437, 3.16622691292876, 5.01930501930502, 93.3673469387755, 
2.4390243902439, 97.822445561139, 4.40251572327044, 2.87958115183246, 
3.01369863013699, 97.2916666666667, 1.22324159021407, 95.0236966824645, 
2.06489675516224, 0.816326530612245, 0.564971751412429, 0.568181818181818, 
2.61780104712042, 3.95136778115502, 6.43776824034335, 3.41296928327645, 
92.8327645051195, 96.2962962962963, 94.7154471544715, 1.41342756183746, 
0.823045267489712, 1.71919770773639, 3.95136778115502, 0.925925925925926, 
3.48432055749129, 96.7244701348748, 3.00546448087432, 0, 2.32558139534884, 
96.0132890365448, 2.27272727272727, 0.735294117647059, 90.3030303030303, 
96.8051118210863, 2.38095238095238, 1.06382978723404, 1.58102766798419, 
95.5, 0.882352941176471, 0.75187969924812, 92.1212121212121, 
0.694444444444444, 0.657894736842105, 1.47058823529412, 92.2222222222222, 
1.78571428571429, 0.341296928327645, 3.33333333333333, 95.3237410071942, 
1.48148148148148, 1.84615384615385, 4.05405405405405, 1.9047619047619, 
3.38983050847458, 94.6058091286307, 0.796812749003984, 93.0434782608696, 
2.95081967213115, 2.74914089347079), T.perc = c(1.49253731343284, 
1.21212121212121, 2.20588235294118, 0.302571860816944, 1.6, 90.8554572271386, 
0.981767180925666, 0.840336134453782, 2.34375, 1.81347150259067, 
3.11284046692607, 1.2, 1.76056338028169, 3.43007915567282, 5.40540540540541, 
3.57142857142857, 95.8188153310105, 1.34003350083752, 93.7106918238994, 
95.0261780104712, 96.986301369863, 0.625, 95.4128440366973, 1.65876777251185, 
3.24483775811209, 0, 1.9774011299435, 93.75, 1.83246073298429, 
2.12765957446809, 2.14592274678112, 2.38907849829352, 0.341296928327645, 
0.284900284900285, 0.813008130081301, 1.41342756183746, 2.46913580246914, 
2.86532951289398, 0, 2.46913580246914, 91.2891986062718, 0.963391136801541, 
0.546448087431694, 1.27388535031847, 2.71317829457364, 0, 1.26262626262626, 
1.83823529411765, 0, 0, 1.42857142857143, 2.12765957446809, 1.18577075098814, 
3, 3.52941176470588, 3.38345864661654, 3.63636363636364, 95.1388888888889, 
94.7368421052632, 1.83823529411765, 0.555555555555556, 0.595238095238095, 
3.41296928327645, 4, 0.719424460431655, 2.22222222222222, 96, 
2.02702702702703, 2.85714285714286, 2.82485875706215, 0, 2.39043824701195, 
0.869565217391304, 0.983606557377049, 2.74914089347079), base.call = c("A", 
"A", "G", "A", "C", "T", "G", "G", "C", "C", "A", "G", "A", "C", 
"C", "G", "T", "G", "T", "T", "T", "G", "T", "G", "C", "A", "C", 
"T", "A", "C", "A", "C", "G", "G", "G", "C", "A", "C", "C", "C", 
"T", "G", "A", "C", "C", "G", "A", "C", "G", "G", "C", "A", "A", 
"G", "A", "A", "G", "T", "T", "C", "G", "A", "C", "A", "G", "C", 
"T", "C", "C", "C", "G", "C", "G", "A", "C"), index = 268:342, 
    guide.seq = c("A", "A", "G", "A", "C", "T", "G", "G", "C", 
    "C", "A", "G", "A", "C", "C", "G", "T", "G", "T", "T", "T", 
    "G", "T", "G", "C", "A", "C", "T", "A", "C", "A", "C", "G", 
    "G", "G", "C", "A", "C", "C", "C", "T", "G", "A", "C", "C", 
    "G", "A", "C", "G", "G", "C", "A", "A", "G", "A", "A", "G", 
    "T", "T", "C", "G", "A", "C", "A", "G", "C", "T", "C", "C", 
    "C", "G", "C", "G", "A", "C"), T.pval = c(0.665373352761804, 
    0.747318716928807, 0.449927179734108, 0.901096414054914, 
    0.632464111121225, 0, 0.806384996981537, 0.83701084727603, 
    0.411749753703038, 0.566587793241365, 0.236639569021981, 
    0.750656529956987, 0.58288230110515, 0.183801357442874, 0.0308298967239741, 
    0.163609916067117, 0, 0.710910894555447, 0, 0, 0, 0.873162055256293, 
    0, 0.614330477132621, 0.213341910037655, 0.905263157886924, 
    0.516720185024897, 0, 0.560756754478705, 0.472347999827626, 
    0.467067795863051, 0.399603650663952, 0.899330987715304, 
    0.901776402792991, 0.84233600115211, 0.689221203108796, 0.378674263554672, 
    0.285614768274343, 0.905263157886924, 0.378674263554672, 
    0, 0.810637693061794, 0.882867146284067, 0.729991680521902, 
    0.319227596692789, 0.905263157886924, 0.733187965389252, 
    0.558985745062181, 0.905263157886924, 0.905263157886924, 
    0.684689196368251, 0.472347999827626, 0.75454670731675, 0.258098565604833, 
    0.169407404402901, 0.190899141721067, 0.154980362728397, 
    0, 0, 0.558985745062181, 0.881840231963516, 0.877064646471161, 
    0.186380484526374, 0.113524245302802, 0.858984945281908, 
    0.445310063476359, 0, 0.501895597438628, 0.287355033075685, 
    0.294293505986474, 0.905263157886924, 0.399242548450644, 
    0.831093893078312, 0.805955101465492, 0.311040848860687), 
    C.pval = c(0.250900063512167, 0.461823720252529, 0.849015641572193, 
    0.706717238508165, 0, 0.107345666271355, 0.643076425225303, 
    0.597775379362464, 0, 0, 0.0336429039815815, 0.531942034593633, 
    0.652712471268989, 0, 0, 0.718141450890647, 0.48587471618375, 
    0.860465116279069, 0.541030121705724, 0.541571702213814, 
    0.860465116279069, 0.578673221532984, 0.366354988971086, 
    0.306439663753795, 0, 0.0623933960959655, 0, 0.159171075596917, 
    0.198854062204727, 0, 0.258819710222624, 0, 0.3926202970265, 
    0.204322386425284, 0.0634650109653186, 0, 0.0602743345589587, 
    0, 0, 0, 0.0803398975682316, 0.734505899776861, 0.519077241591654, 
    0, 0, 0.621362128569684, 0.642961775895076, 0, 0.293217902752091, 
    0.637990891656931, 0, 0.149644689819188, 0.846512531883422, 
    0.860465116279069, 0.860465116279069, 0.84829392300845, 0.461823720252529, 
    0.382440528487991, 0.517319889030065, 0, 0.217529900657009, 
    0.472036514747902, 0, 0.0162564419268291, 0.143352684518498, 
    0, 0.552186755518067, 0, 0, 0, 0.512435294730668, 0, 0.171545564628337, 
    0.519077241591654, 0), G.pval = c(0.592620040397499, 0.58611143653393, 
    0, 0.0723144651913669, 0.163838785646464, 0.12725013938449, 
    0, 0, 0.377746636316288, 0.880945768548842, 0.47008173076767, 
    0, 0.352657830121373, 0.232918302391951, 0.0710322969066678, 
    0, 0.357883327428498, 0, 0.106651155759323, 0.276801290819431, 
    0.255448887824077, 0, 0.672501415269408, 0, 0.440825780214879, 
    0.796492217260904, 0.868550774618519, 0.867685878448385, 
    0.32288576712651, 0.142666967267016, 0.0270966589087155, 
    0.200170669301024, 0, 0, 0, 0.61566018117806, 0.794479208241582, 
    0.529141028392095, 0.142666967267016, 0.763352209336124, 
    0.191501312311635, 0, 0.256717487612426, 0.966292134803909, 
    0.381631581770771, 0, 0.393111003939204, 0.820499232226277, 
    0, 0, 0.369888969356568, 0.721155284198081, 0.567362941217175, 
    0, 0.776595380713591, 0.815631162476929, 0, 0.832369435021596, 
    0.842826347191561, 0.598971112846214, 0, 0.511310234913428, 
    0.922959011458578, 0.210260837949434, 0, 0.595814987653045, 
    0.495445821579625, 0.133595549443105, 0.480376783036308, 
    0.203056800811355, 0, 0.802321219033623, 0, 0.265275588215094, 
    0.299016868378961), A.pval = c(0, 0, 0.0984884589216281, 
    0, 0.084466403795925, 0.485760096800875, 0.782145952792471, 
    0.332936544549437, 0.0244960735237014, 0.0721431754165588, 
    0, 0.159452188402485, 0, 0.23407717088021, 0.604109509886401, 
    0.311605213947952, 0.935185185185185, 0.730358835872377, 
    0.900851498388249, 0.843768595154043, 0.935185185185185, 
    0.809654057908812, 0.578961144194315, 0.687043950851126, 
    0.388248255940486, 0, 0.328417592726928, 0.199066922132487, 
    0, 0.289685523661362, 0, 0.525430781225022, 0.0222803600730971, 
    0.828622246795303, 0.878353558339712, 0.38920649354076, 0, 
    0.60885763068648, 0.127441568352134, 0.215316035337933, 0.514955802476024, 
    0.531511980679552, 0, 0.0814975470424015, 0.172717197283383, 
    0.18167646614333, 0, 0.196093122987327, 0.00148067285623865, 
    0.345089481005027, 0.0601604210057178, 0, 0, 0.476935733285472, 
    0, 0, 0.224066213387475, 0.300715403918939, 0.137293647013393, 
    0.487296209603121, 0.0315309415239345, 0, 0.231129254468933, 
    0, 0.775410772740634, 0.935185185185185, 0.813021001043899, 
    0.79152251357907, 0.685260193428423, 0.0910436182800592, 
    0.0648422658565124, 0.74609595097721, 0.126964356824721, 
    0, 0.404303988133876), guide.position = 1:75), row.names = 268:342, class = "data.frame")
```

*Report generated using EditR v1.0.10*
